# Natural antisense transcripts are significantly involved in regulation of drought stress in maize

**DOI:** 10.1093/nar/gkx085

**Published:** 2017-02-08

**Authors:** Jie Xu, Qi Wang, Micheal Freeling, Xuecai Zhang, Yunbi Xu, Yan Mao, Xin Tang, Fengkai Wu, Hai Lan, Moju Cao, Tingzhao Rong, Damon Lisch, Yanli Lu

**Affiliations:** 1 Maize Research Institute, Sichuan Agricultural University, Wenjiang 611130, Sichuan, China; 2 Key Laboratory of Biology and Genetic Improvement of Maize in Southwest Region, Ministry of Agriculture, China; 3 Department of Plant and Microbial Biology, University of California, Berkeley, CA 94703, USA; 4 International Maize and Wheat Improvement Center (CIMMYT), El Batan 56130, Texcoco, Mexico; 5 Institute of Crop Science, Chinese Academy of Agricultural Sciences, Haidian, Beijing 100081, China; 6 Department of Botany and Plant Pathology, Purdue University, West Lafayette, IN 47907, USA

## Abstract

Natural antisense transcripts (NATs) are a prominent and complex class of regulatory RNAs. Using strand-specific RNA sequencing, we identified 1769 sense and antisense transcript pairs (NAT pairs) in two maize inbreds with different sensitivity to drought, as well as in two derivative recombination inbred lines (RILs). A significantly higher proportion of NATs relative to non-NATs are specifically expressed under water stress (WS). Surprisingly, expression of sense and antisense transcripts produced by NAT pairs is significantly correlated, particularly under WS. We found an unexpected large proportion of NATs with protein coding potential, as estimated by ribosome release scores. Small RNAs significantly accumulate within NAT pairs, with 21 nt smRNA particularly enriched in overlapping regions of these pairs of genes. The abundance of these smRNAs is significantly altered in the *leafbladeless1* mutant, suggesting that these genes may be regulated by the tasiRNA pathway. Further, NATs are significantly hypomethylated and include fewer transposable element sequences relative to non-NAT genes. NAT gene regions also exhibit higher levels of H3K36me3, H3K9ac, and H3K4me3, but lower levels of H3K27me3, indicating that NAT gene pairs generally exhibit an open chromatin configuration. Finally, NAT pairs in 368 diverse maize inbreds and 19 segregating populations were specifically enriched for polymorphisms associated with drought tolerance. Taken together, the data highlight the potential impact of that small RNAs and histone modifications have in regulation of NAT expression, and the significance of NATs in response to WS.

## INTRODUCTION

Natural antisense transcripts (NATs) are a prominent and complex class of regulatory RNAs, the function of which is only beginning to be understood (1). By definition, NATs are transcribed from strands opposite to the sense transcripts of coding or noncoding genes (2). NATs were initially discovered in bacteria (3), and have since been characterized in animals and plants (4–6). NAT pairs are pairs of adjacent genes that express transcripts that are overlapping and complementary. Genome-wide NATs have been surveyed in Arabidopsis (6), *Brassica rapa* (7), rice (8), wheat (9), sugarcane (10) and legumes (11). Such surveys indicate that NAT pairs are prevalent. Indeed, many NAT pairs are conserved, and play various biological roles via both transcriptional and post-transcriptional gene regulation (12,13).

NAT expression and function have been investigated either by strand-specific RNA quantification or by techniques such as global run-on sequencing (14,15). In Arabidopsis, NATs are transcribed from ∼30% of all annotated genes (16,17). Antisense transcription was found to be even more prevalent in mammalian cells, with 50–70% of sense transcripts having antisense partners (3). However, NATs are generally 10-fold less abundant than NAT pair sense transcripts (18). In 16 human tissues, genes in NAT pairs can be either co-expressed or expressed in inverse correlation to each other, and exhibit a wider range of expression than genes without NATs (15,19,20). These observations suggest that NATs may contribute to regulatory complexity, but to an extent that remains poorly characterized.

For obvious reasons, stress response has been a long-standing focus of plant breeding and biotechnology. Recently, our understanding of metabolic and genetic determinants that underlie drought resistance has been complemented by accumulating evidence of stress-induced changes in chromatin, DNA methylation, and regulation by small RNA (smRNA) (21). These epigenetic changes are likely to play an important role in transcriptional and post-transcriptional control of genes critical for stress response (22,23). At least some NATs have been proposed to respond to developmental or abiotic stimuli (24,25) via similar mechanisms, which can be associated with transcriptional interference, chromatin modification, or silencing by methylation of cytosines (26,27). For instance, cold-assisted intronic non-coding RNA (*COOLAIR*) in Arabidopsis represses the FLOWERING LOCUS C (*FLC*) sense transcript via changes in histone marks (28). On the other hand, NATs may also enhance expression of cognate genes. For example, a *cis* natural antisense RNA in rice enhances translation of its cognate sense mRNA to regulate phosphate homeostasis and plant fitness (24).

A growing body of evidence suggests that NATs may regulate stress response. In line with this hypothesis, NATs are much more environmentally sensitive than the average plant gene (2,15), and *cis* NAT pairs have been shown to generate siRNAs associated with salt and heat tolerance (7,29). Although the mechanism of NAT action remains unclear in higher eukaryotes, it has been suggested that sense and antisense expression may be in a homeostatic balance under normal conditions, and that balance is altered in response to developmental, physiological, or environmental cues (20).

We sought to determine whether and how NAT genes might specifically mediate drought tolerance. To do this, we identified genes with expressed NATs in two maize inbred lines that carry multiple loci responsible for drought tolerance, as well as in two recombinant inbred lines (RILs) generated from these two parental lines that are fixed for combinations of loci that confer either high or low drought tolerance (30,31). Expression of NAT pairs in each maize line with each of these genetic backgrounds was measured under water stress (WS) and well water (WW) using strand-specific transcriptome sequencing. In addition, translational efficiency was measured by ribosome profiling, and potential epigenetic regulation of NATs was examined by whole-genome analysis of DNA methylation and smRNA accumulation under drought conditions, as well as by histone marks and transposon coverage. To test whether accumulated smRNA in NAT pairs are regulated by the LEAFBLADELESS1 protein (*LBL1*), a homolog of SUPPRESSOR OF GENE SILENCING3 (*SGS3*) (32), the ratio of smRNA content in *lbl1*-deficient maize mutant relative to that in wild type was compared among sense transcripts from NAT pairs, NATs, overlapping regions between sense and NATs, housekeeping genes, long noncoding RNAs (lncRNAs), and non-NATs (transcripts that are not in a NAT pair) (32). Finally, potential NAT functions were investigated by association analysis in a diverse panel of 368 maize inbreds, as well as in 19 bi-parental populations derived from 23 elite maize inbreds. Overall, we found strong evidence that NATs are important elements of drought response in maize.

## MATERIALS AND METHODS

### Plant materials

Two parental maize lines, AC7643 and AC7729/TZSRW, and two derivative RILs, RIL208 and RIL64, were selected for analysis based on drought tolerance or sensitivity, as previously characterized (30,31). The four lines used in this study were provided by the International Maize and Wheat Improvement Center (CIMMYT). Seedlings at the three-leaf stage were treated with 10% (w/v) polyethylene glycol PEG 8000 (Sigma-Aldrich) for 24 h and harvested to evaluate root morphology. For each sample, at least three plants were pooled and two independent biological replicates were carried out. Detailed methods are available in Supplemental File 1.

### Library construction and sequencing

Total RNA was extracted from roots using TRIzol^®^ (Invitrogen, USA), and treated with RNase-free DNase I. Libraries for strand-specific RNA sequencing and smRNA profiling were constructed as described by Hirsch *et al*. (33) and Wang *et al*. (34), respectively. DNA was extracted using CTAB from the same samples used for RNA isolation. Methylated fragments were enriched by Magnetic Methylated DNA Immunoprecipitation Kit (Diagenod, Liège, Belgium). RNA was isolated from two independent replicates of each line under two water conditions for library construction and sequencing. Libraries were sequenced on HiSeq 2000 and HiSeq X10 system (Illumina, San Diego, CA, USA) for replicates I and II, respectively. Detailed methods can be found in Supplemental File 1. Raw sequencing data have been deposited in the NCBI Sequence Read Archive under accession number PRJNA294848 (SRP063383).

### Detection and abundance of NATs

Strand-specific RNA sequencing data were collected under WW and WS conditions from roots of the drought-tolerant maize line AC7643, and the drought-sensitive line AC7729/TZSRW, as well as from the two derivative RILs. In addition, strand-specific RNA sequencing reads with extremely high sequencing depth were retrieved from the Sequence Read Archive (SRR765211, http://www.ncbi.nlm.nih.gov/sra/) (33) for the maize reference line B73. These reads were obtained from whole seedlings at the V1 stage, at which the seedling has one leaf with a visible collar. The Sequence Read Archive toolkit was used to move data between formats (35). Raw reads were then processed with FASTX Toolkit (version 0.0.14) (http://hannonlab.cshl.edu/fastx_toolkit/). Specifically, fastx_clipper and fastx_artifacts_filter were used to remove Illumina adapter sequences and artifactual sequences. Low-quality reads were then discarded using fastq_quality_trimmer. The non-contiguous alignment tool, spliced transcripts alignment to a reference (STAR, version 2.5.2) (36), was used to map the remaining, high-quality reads to the maize reference genome B73 RefGen_V3 (ftp://ftp.ensemblgenomes.org/pub/release-31/plants/fasta/zea_mays/dna/Zea_mays.AGPv3.31.dna.genome.fa.gz) according to known transcripts and annotations (ftp://ftp.ensemblgenomes.org/pub/release-31/plants/gff3/zea_mays/Zea_mays.AGPv3.31.gff3.gz) with the parameters –outSAMattrIHstart 0 –alignIntronMax 2000 –outFilterMismatchNmax 4. The alignment of reads by STAR is accurate and allows the mismatches, insertions and deletions caused by genomic variation and sequencing errors. The unique mapping reads were used in all subsequent analysis. The mapped reads saturation for the expressed genes in all samples were estimated using RSeQC (version: 2.6.4) (37). Percent relative error is defined as:

\begin{equation*}100\% \times \frac{{\left| {RPK{M_{obs}} - RPK{M_{real}}} \right|}}{{RPK{M_{real}}}},\end{equation*}

where *RPKM_obs_* is measured from resampling subsets of reads, and *RPKM_real_* is estimated from total reads. The strand specificity of eight libraries was calculated followed by the method proposed by Yassour *et al*. (38). A set of transcripts that did not overlap any other transcripts within 3 kb were chosen to calculate the sum of all opposite strand reads divided by the total read count. SAMtools (version 1.3.1) (39) and BEDTools (version 2.26.0) (40) were then applied to split the bam file into minus- and plus-strands. The transcriptome in each strand was reconstructed using StringTie (version 1.2.3), with a required minimum junction coverage of 5 (-j) and 5 minimum reads per bp coverage to be considered for transcript assembly (-c) (41). Transcripts from each strand were merged using Cuffmerge in Cufflinks (version 2.2.1) with default setting (42). Transcriptomes for each sample of the two replicates were reconstructed independently and the common transcripts were identified using Cuffcompare in Cufflinks for further analysis. The merged common transcriptome annotation file was compared with the maize B73 RefGen_V3 annotation using Cuffcompare in Cufflinks to obtain the known annotations. The characteristics of the assembled transcripts were calculated in Biostrings 2.38.4 in Bioconductor. Cufflinks was applied to quantitate each gene's expression level against the final merged genome annotation (–G) strand (–library-type fr-firststrand). The longest transcript of each gene obtained in this manner was quantified as a fragment per kilobase of exon model per million mapped reads (FPKM). Only transcripts with expression values of > 0.1 were counted as being detected and used for subsequent analysis. The read number of each gene was calculated using Rsubread (version 1.20.3) featureCounts (43). Spearman's correlation coefficients between two replicates for each sample was calculated using mapped read numbers for each expressed genes in R version 3.2.3. Reads were normalized using the upper-quartile model in edgeR (version 3.8.6) (44). Sense and antisense transcripts with exactly the same structure were filtered out, along with transcripts with <8 reads. The ratio of reads between sense and antisense transcripts was assumed to be between 0.01 and 100. By comparing transcript structures and read distribution on opposite DNA strands, potential NAT pairs were identified based on an overlap >50 bp and a FKPM value >0.1, which has been used as a standard to define NATs in Arabidopsis (5). Potential protein sequences of NAT pairs were blasted against sorghum and rice protein sequences downloaded from (ftp://ftp.ensemblgenomes.org/pub/release-32/plants/fasta/sorghum_bicolor/pep/Sorghum_bicolor.Sorbi1.pep.all.fa.gz, ftp://ftp.ensemblgenomes.org/pub/release-32/plants/fasta/oryza_sativa/pep/Oryza_sativa.IRGSP-1.0.pep.all.fa.gz). Protein blast (blastp) was conducted using NCBI BLAST version 2.3.0 (with alignment identity ≥30%, alignment length ≥30 residues, *e* value ≤0.01). Protein sequences of the newly assembled genes were predicted using TransDecoder version 2.0.1 with default parameters (https://transdecoder.github.io/). For each pair, the transcript that showed the greatest conservation with sorghum or rice and that was detected in a majority of samples was annotated as sense, and the corresponding transcript on the opposite strand was designated as antisense. GlmQLFit model in edgeR was used to estimate the dispersion and to correct for the batch effects of two replicates, and the glmLRT test was used to analyze differential expression under WS and WW conditions (44). Using a false discovery rate of 0.001 after Benjamini-Hochberg correction for multiple tests, genes with a log_2_ fold change in expression > 1 or < –1 were considered differentially expressed (DE). The response of both sense and antisense transcripts to WS was visualized as a heat map using the R package ggplot2 (version 1.0.1).

The overlaps of NAT pairs were used to categorize NAT pairs into three different types: convergent (tail-to-tail overlap), divergent (head-to-head overlap), or enclosed (one transcript is encompassed by the other) (45). In addition, the TSSP program in Softberry (http://www.softberry.com) (46) was used to predict potential promoters of genes encoding sense and antisense transcripts. The position of potential antisense gene promotors was compared with transcripts and transposable elements (TEs) using BEDTools. TE annotation in the maize genome B73 is included in the RefGen_V3 genome annotations. NATs were characterized in comparison to non-NATs (transcripts not paired with NATs), housekeeping genes identified in the maize transcriptome (47), and high-quality lncRNAs in the maize reference genome (48,49). NATs only expressed in WW or WS in at least two samples were categorized as either WW or WS specifically expressed NATs. Housekeeping genes and lncRNAs expressed in the same orientation as in our study (FPKM>0.1) are listed in Supplemental File 2 and Supplemental File 3, respectively. All of the NATs identified in any of the eight samples were integrated into a dataset for the further analysis. The number of NATs in different subsets (parental lines vs. offspring inbred lines; WW vs. WS for each line) was then calculated and a χ2 test was made for each comparison. A Venn diagram of detectable NATs among different materials and water conditions was then made in VennDiagram 1.6.16. Strand-specific reverse transcription and SYBR Green-based quantitative real-time PCR (qPCR) were performed to validate and quantify sense and antisense transcripts in maize lines AC7643 and AC7729/TZSRW. Detailed methods are available in Supplemental File 1.

### Expression and inheritance of NAT pairs

FPKM values for NAT pairs were analyzed by non-parametric Spearman correlation to test whether NATs may modulate expression of the corresponding sense transcripts. This analysis was carried out because FPKM values were not normally distributed based on the Shapiro-Wilk test (*P* value < 0.001), even in log space (*P* value < 0.001). Correlations between sense and antisense expression were calculated in R. Permutation tests were performed in R to confirm statistical significance of differences between WW and WS conditions. Expression specificity was measured by Shannon entropy (50,51). Inheritance of patterns of NAT pair expression was investigated by comparing expression of specific genes in parental lines (AC7643 and AC7729/TZSRW) and in the RILs (RIL208 and RIL64). For this analysis, a statistical model developed by Li *et al*. (48) for maize lncRNA was applied. Detailed formulas can be found in Supplemental File 1.

### Translational efficiency by ribosome profiling

Ribosome profiling data for B73 seedlings were obtained from NCBI (SRP052520) (52). This dataset is suitable for investigation of transcriptional, translational, and post-translational gene expression before and after drought stress. Data were analyzed as described by Ingolia *et al.* (53). Briefly, low-quality reads were filtered out, linker sequences were trimmed, and the first nucleotide was removed from the 5΄ end of each read. Reads matching known structural RNAs in Rfam 12.0 (54), including rRNAs, tRNAs, snRNAs and sno-RNAs, were excluded from further analysis. TransDecoder was used to predict UTR and open reading frame (ORF) regions of each transcript (55). For transcripts for which a putative ORF could not be defined, the 3΄-UTR was defined as the region between the last putative stop codon (UAA/UAG/UGA) and the next possible start codon (ATG) in any frame. Ribosome release scores (RRS) were calculated according to Guttman *et al*. (56) in order to assess translational efficiency:

\begin{equation*}RRS = \frac{{\ {{\left( {\frac{{Read{s_{CDS}}}}{{Read{s_{3{^/prime}UTR}}}}} \right)}_{Ribosome}}}}{{{{\left( {\frac{{Read{s_{CDS}}}}{{Read{s_{3{^/prime}UTR}}}}} \right)}_{mRNA}}}}\end{equation*}

To verify that NATs identified by strand-specific RNA-sequencing are also expressed in samples used for ribosome profiling, transcripts were assembled *de novo* based on the mRNA sequencing transcriptome using Trinity 2.1.1 (57). *De novo* assembled transcripts were then mapped back to the maize reference genome to search for complementary regions adjacent to transcripts (58) (adjacent region >50 bp). The NATs identified in four drought tolerant/sensitive lines under WS and in WW as well as those previously identified in B73 seedlings (52) were used for subsequent analysis. Finally, translational efficiency was compared among maize NATs, housekeeping genes, lncRNAs, and non-NAT genes randomly sampled in 1000 bootstraps into pools with the same sample size as NATs.

### SmRNA coverage and abundance

SmRNA sequencing reads from roots of AC7643, AC7729/TZSRW, RIL208 and RIL64 plants under WS and WW were processed with Cutadapt version 1.8 (59) and FASTX Toolkit to remove adapters and low-quality bases. The remaining reads were used to identify and annotate smRNA (in nostitch mode) using ShortStack version 3.4 (60,61). For each sample, the number of reads per million (RPM) was calculated using the number of mapped reads in gene regions (including 1 kb flanking sequences), which was then normalized to the total count of genome-mapped reads. To assess the correlation of two biological replicates, the normalized RPM values were used to calculate correlation coefficients. The overlapped regions of identified smRNA (in ShortStack) in NAT pairs was characterized using BEDTools (40). The number of NATs that overlapped with smRNAs (with more than five smRNA reads) was calculated in R. The counts of 20–24 nt smRNAs were calculated in ShortStack separately. A sliding window was applied to calculate smRNA coverage and abundance along genes, which was defined as the average number of smRNAs within 1 kb of the transcription start and termination sites. In order to obtain normally distributed data, we discarded both highly abundant and extremely rare smRNAs (mean +2.5 SD). Coverage of more than 2.5 standard deviations from the mean (mean ± 2.5 SD) was discarded. To eliminate the possible confounding signals from TEs, transcripts that overlapped with TEs were excluded from group analysis of smRNA, DNA methylation and chromatin modifications. For each sample, the read number of smRNAs in all expressed genes (FPKM > 0.1) was calculated using ShortStack in count mode (-nostitch, -loci). Transcripts with DE small RNAs were explored using edgeR with GlmQLFit modes to estimate the dispersion, and analyze differential expression using glmLRT test. Genes with log_2_ fold change in smRNA expression > 1 or < –1 (false discovery rate of 0.05 after Benjamini–Hochberg correction for multiple tests) were considered to be DE smRNA under drought stress.

### Examination of the affect of the *lbl1* mutant on the abundance of 21 nt NAT smRNAs

As observed in Arabidopsis, NATs may be processed by Dicer-like proteins into 21 nt smRNAs. Previous research has also suggested that NAT pairs are regulated by components of the *trans*-acting siRNA (tasiRNA) pathway, including *SGS3* (29,32,62). To determine whether NAT smRNA abundance depends on *LBL1*, the *SGS3* homolog in maize, smRNA profiles for wild type and *lbl1* maize mutants were downloaded from the Sequence Read Archive SRP029451 (32), and compared with sense and antisense transcripts, overlapping regions, housekeeping genes, lncRNAs and 1000 bootstrap samples of non-NATs with the same sample size as the NATs. The ratio of smRNA reads between wild type and the mutant maize was considered to be a measure of the effect of *LBL1* on smRNA abundance.

### MeDIP and bisulfite sequencing

Methylated DNA Immunoprecipitation (MeDIP) sequencing reads from four maize lines under WW and WS were aligned to the B73 reference genome RefGen_V3 in Bowtie2 (63) with default settings. Best-matched reads (with exactly 49 bp alignment length) were analyzed using Rsubread featureCounts. Average coverage within 1 kb of the transcription start and termination sites was calculated in 100 bp windows with a 10 bp increment. The script in R used to scan genome-wide DNA methylation along genes in sliding windows is provided in Supplemental File 4.

Bisulfite sequencing reads of B73 coleoptiles were obtained from the Sequence Read Archive SRP014211 (64), and processed by Trim Galore (http://www.bioinformatics.babraham.ac.uk/projects/trim_galore/) and cutadapt to exclude low-quality bases and overrepresented sequences (59). Reads were mapped and methylation marks were called in Bismark Version 0.12.2 (65). Methylation of CpG, CHG and CHH in NAT pairs was estimated using BEDTools (40) and custom R scripts. Average coverage for methylation calls >60% was calculated in 100 bp windows with a 10 bp step size for 1 kb regions flanking the transcription start and termination sites.

### NAT expression and chromatin modifications

To investigate if and how antisense transcription is associated with specific chromatin structure, we analyzed sequencing data of chromatin immunoprecipitated from roots of 14-day-old maize B73. This data set was downloaded from the Sequence Read Archive (SRP001359), and was collected by Illumina/Solexa 1G parallel sequencing of chromatin immunoprecipitated with antibodies against H3K4me3, H3K9ac, H3K27me3 and H3K36me3 (34). High-quality reads were mapped to the maize reference genome, and best-matched reads were analyzed for histone marks. Average coverage was calculated in 100 bp windows with 10 bp step size over 1 kb upstream and downstream of the transcription start and termination sites.

### Transposable elements near target genes

To assess the impact of transposable elements on antisense transcription, the relative positions of TEs and transcripts were analyzed using BEDTools (40). Average coverage of transposable elements within 1 kb of the transcription start and termination sites was calculated using 100 bp windows in 10 bp steps. Differences in epigenetic marks along genes, including smRNA abundance, DNA methylation, chromatin modifications, and TE content, were compared among NAT pair sense transcripts, NATs, and non-NATs randomly sampled with 1000 bootstraps with the same sample size as NATs.

### Potential function of drought-induced maize NATs in the association mapping and biparental populations

Drought-induced NAT pairs in a panel of 368 maize inbred lines (66) were investigated for association with drought survival (67), kernel oil content (66) and days to tassel (68). For this analysis, we used the mixed linear model and general linear model in Tassel v3.0 (69). Kernel oil concentration (66) and days to tassel (68) were measured under normal conditions, and used as reference for experiments under drought (67). The number of SNPs associated with different traits (Bonferroni-corrected *P* value < 0.01) was calculated using the general linear model and mixed linear model incorporated population structure and kinship. ANNOVAR (2016Feb01) was used to distinguish nonsynonymous and synonymous SNPs (70). Nonsynonymous-to-synonymous substitution ratios for NAT pairs genes and non-NAT genes randomly sampled in 1000 bootstraps were calculated in R.

The association between NAT SNPs and grain yield, anthesis, and plant height was investigated in 19 populations of biparental tropical maize using *t*-test with a Bonferroni correction. These populations comprised 3273 lines derived from crosses or backcrosses among 23 elite inbred lines from the CIMMYT. These lines were evaluated in 3–4 WW and WS environments in Kenya and Zimbabwe in 2010 and 2011, and genotyped by genotyping-by-sequencing SNPs, with 955 690 informative SNPs evenly distributed on maize chromosomes for all the lines in each population (71).

## RESULTS

### NATs in maize under WW and WS

Using analysis of variance (ANOVA), we first compared the root systems in maize inbred lines, AC7643 and AC7729/TZSRW, as well as in RIL208 and RIL64, which are recombinant inbred lines derived from these two inbred lines (Supplemental Table S1). AC7643 and RIL208 are drought-tolerant, while AC7729/TZSRW and RIL64 are drought-sensitive. As expected, the root system under both WW and WS was significantly larger in drought-tolerant lines.

Strand-specific RNA sequencing was then used to survey the root transcriptome under WW and WS. A total of 979 760 886 reads were obtained, of which 83.78% (820 848 834) were successfully mapped by STAR (36) to the B73 maize reference genome B73 RefGen_V3 (Supplemental Table S2). The mapped read saturation analysis for the expressed genes (Supplemental Figure S1) and the number of the unique mapped reads (Supplemental Table S2) suggest that saturation was indeed reached in this experiment. The Spearman correlations for each sample between two replicates ranged from 0.80 to 0.89, with average of 0.85 (*P* value < 0.001) (Supplemental Table S2). To reconstruct genes as completely and accurately as possible, transcripts were then assembled *de novo* using StringTie (41). The longest transcripts obtained in this manner with FPKM expression value >0.1 corresponded to 33 149 genes in the eight samples, of which 25.84% (8566) were newly assembled. The N50 transcript length in eight samples was 2.03 kb, which is significantly longer than the maize reference genome annotations RefGen_V2 (N50:1.61 kb) and RefGen_V3 (N50:1.83 kb, Kolmogorov-Smirnov test *P* value < 0.001). To estimate the library specificity, the directions of reads mapped to 3691 genes, without any other transcript annotated within 3 kb, were analyzed (Supplemental Table S2). The strand specificity in our eight samples varied from 99.09% to 99.84% (SD = 0.24), indicating high strandedness of the sequencing libraries.

Transcripts (defined as the for the purposes of this analysis as the longest transcript expressed from each gene) from opposite DNA strands and overlapping more than 50 bp were selected as candidate *cis* NAT pairs, based on the criterion established in Arabidopsis (5). For each pair, the transcript that showed stronger conservation with homologs in sorghum or rice was designated as sense. If neither showed higher conservation, the transcript that was detected in a majority of samples was annotated as sense, and the corresponding transcript on the opposite strand was designated as antisense. The pool of candidate NAT pairs was further filtered and verified based on the orientation and distribution of mapped reads, as described in Materials and Methods. The final pool from all samples contained 3460 transcripts and consisted of 1711 sense and 1759 NATs (58 sense transcripts with more than one NAT and 10 NATs with more than one sense transcript), of which 46.18% (1598 out of 3460) were novel (Supplemental Table S3). The gene structure annotation file (in GTF format) of the NATs is provided in Supplemental File 5. There were 1769 NAT pairs detectable in at least one sample. Of the candidate NAT pairs, 65.29% (1155 out of 1769) were detectable in at least two of the four lines (Supplemental Figure S2A).

Notably, a significantly higher proportion of the total number of expressed NAT pairs were detected in the RILs (83.32% of the total) than were expressed in the parental lines (64.44% of the total, χ^2^ test *P* value < 2.20E–16, Supplemental Figure S2A). This suggests that the hybridization and subsequent inbreeding that gave rise to the RILs may have activated otherwise quiescent NATs. The RILs shared a larger set of NAT pairs with each other (44.77%) than did the parental lines with each other (31.54%) (Supplemental Figure S2A), which is not surprising given their recombinant nature. Among all samples, a significantly higher percentage of NATs were expressed under WS (84.40%) than in WW (73.49%, χ^2^ test *P* value 2.43E–15, Supplemental Figure S2B–D). Indeed, 26.51% of NATs were only expressed under WS, while only 15.60% were only expressed in WW (Supplemental Figure S2B–D). Overall, only 21.93% of NATs were expressed in all four lines. In contrast, 87.09% of all NAT pair sense transcripts were expressed in all of these lines (Supplemental Figure S2A), suggesting that NATs transcription, but not cognate sense transcription, is far more variable than transcription of the average maize gene.

NAT pairs could be categorized into three different types: convergent, divergent, or enclosed (45). Notably, 61.05% (1080 out of 1769) of maize NAT pairs were enclosed (Supplemental Table S3). This result is similar to observations in rice (8), but not to those in the dicots Arabidopsis or *Brassica rapa*, in which most NAT pairs are convergent (7). 21.82% (386 out of 1769) of maize NAT pairs were divergent, and 17.13% (303 out of 1769) were convergent. Surprisingly, 65.69% of NATs are transcribed from within exons of sense genes, and only 12.72% are transcribed from within introns. For NAT pairs in the enclosed category, the putative promoters of a large portion of NATs (24.48%) were located in 3΄ end of the nearby genes. In B73, there were 1492 NATs pairs genes identified, with a small fraction (26 out of 1492, 1.74%) initiated from within transposable elements. Only 7.37% and 4.42% of the enclosed NATs’ putative promoters were in exons or introns, respectively. In contrast, the putative promoters of 19.03% enclosed NATs in B73 are located in transposons.

Four NAT pairs (eight genes) were randomly selected for experimental validation. Primers with sequences and annealing temperatures are listed in Supplemental Table S4. Read distributions of these genes were visualized in Supplemental Figure S3. The presence of sense and antisense transcripts in maize was validated by strand-specific reverse transcriptase PCR and SYBR Green-based qPCR. All four NATs were successfully detected and changes in expression were consistent with strand-specific transcriptome sequencing (Supplemental Figure S4).

### NAT pairs in response to drought stress

**Figure 1.**
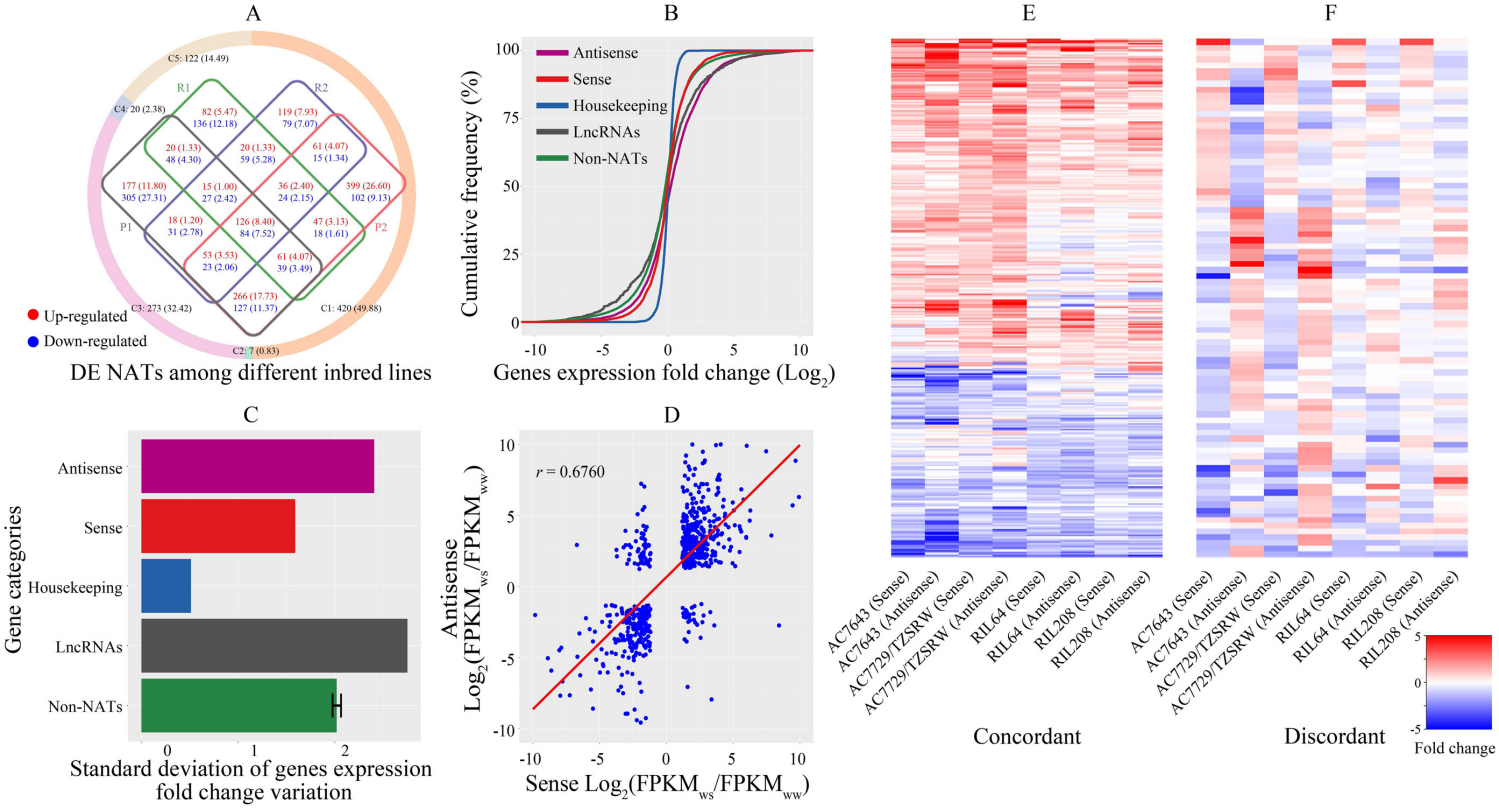
Response of natural antisense transcript (NAT) pairs to drought stress. (**A**) Differentially expressed (DE) NATs among the tested lines with different levels of drought sensitivity. The colored rings represent the numbers and percentages (%) of DE NATs between different samples. C1: Up- or down-regulated DE NATs in both the two drought tolerant lines and the two drought sensitive lines. C2: Up-regulated DE NATs in the two drought tolerant lines, but down-regulated in the two drought sensitive lines, or vice versa. C3: Up- or down-regulated DE NATs in one of the drought tolerant lines and also in one of the drought sensitive lines. C4: Up- or down-regulated DE NATs in one of the drought tolerant lines, but down- or up-regulated in one of the drought sensitive lines. C5: No significant expression changes. The Venn diagram of the DE NATs is in the center of the circle. P1: AC7643. P2: AC7729/TZSRW. R1: RIL208. R2: RIL64. The up- and down-regulated NATs are presented by red and blue colors, respectively. (**B**) Cumulative frequency distribution of gene expression fold change under water stress. Different colors indicate different groups of genes. Antisense transcript (*n* = 3948), sense transcript (*n* = 3884), housekeeping genes (*n* = 2328), lncRNAs of maize (*n* = 1550) and non-NATs in the maize transcriptome (*n* = 112 636) are depicted in dark pink, vivid red, dark blue, dark grey and dark cyan, respectively. The color code is displayed in left corner. (**C**) Standard deviation of fold change variation in gene expression. (**D**) Expression correlation of sense and antisense transcription in NAT pairs in response to water stress. Portrayed are the 516 NAT pair genes with at least a twofold expression increase or decrease under water stress. For each NAT pair, expression fold change under water stress (WS) and well water (WW) for the sense transcript was plotted against the antisense transcript. (**E** and **F**) Concordant and discordant NAT pairs with respect to response to drought stress, respectively.

A large body of research suggests that drought induces tissue-specific and developmental stage-specific changes in gene expression (72–75) and there is evidence that NAT expression can be sensitive to some forms of stress. However, the effect of drought on NAT expression in maize is unknown. We found that 28.56% of all transcripts were sensitive to drought, with log_2_ fold change >1 or < –1 and FDR <0.001, on average. However, NAT pairs responded to drought more robustly than the average gene, and a larger fraction of NATs than expected was up regulated in response to drought (Table 1). To validate this observation, we analyzed 1000 bootstrap re-samples of random transcripts with the same sample size as the NAT pairs (Table 1), and found that the fraction of up-regulated NAT pairs was, indeed, significantly higher than randomly selected gene sets (*t*-test, *P* value < 0.001). In addition, a Venn diagram of the DE NATs among different lines is portrayed in Figure 1A, and 8.02% were consistent in both drought tolerant and sensitive lines. Of the up-regulated NATs, 14.80% and 18.40% were expressed in the drought tolerant lines (AC7643 and RIL208) and the drought sensitive lines (AC7729/TZSRW and RIL64), respectively. Concordantly, 17.73% and 13.07% of the down-regulated NATs were expressed in the drought tolerant lines and sensitive lines, respectively. As a result, the drought sensitive lines showed significantly more up-regulated NATs and fewer down-regulated NATs than did drought tolerant lines (χ^2^ test *P* value < 0.001). Moreover, 222 common up-regulated genes were shared in both of the two drought tolerant lines, of which 126 (56.76%) genes also showed up-regulated expression in drought sensitive lines. The expression change tendency of all the DE NATs was further compared. The ratios of concordant and discordant DE NATs between drought tolerant lines and sensitive lines were shown in Figure 1A. The majority of the DE NATs (49.88 + 32.42%) showed concordant expression changes in all lines examined.

**Table 1.** The number and ratio of drought responsive transcripts and NAT pairs transcripts in four maize lines

Category	AC7643		AC7729/TZSRW		RIL208		RIL64	
	Up-regulated	Down-regulated	Up-regulated	Down-regulated	Up-regulated	Down-regulated	Up-regulated	Down-regulated
Sig.transcripts	4,649 (14.86)	7443 (23.79)	4960 (15.81)	6167 (19.66)	2805 (8.86)	3592 (11.35)	3196 (10.13)	3073 (9.74)
Sig. Sense	163 (20.66)	178 (22.56)	274 (30.14)	141 (15.51)	87 (7.80)	127 (11.39)	121 (10.51)	110 (9.56)
Sig. NATs	237 (30.04)	183 (23.19)	391 (43.01)	101 (11.11)	202 (18.12)	168 (15.07)	206 (17.90)	144 (12.51)
Sig. Sense or NATs	400 (25.35)	361 (22.88)	665 (36.58)	242 (13.31)	289 (12.96)	295 (13.23)	327 (14.21)	254 (11.03)
*P* value (χ^2^ test)	2.65E–29	4.23E–01	3.68E–116	3.19E–11	1.12E–10	7.96E–03	7.91E–10	4.80E–02
Sig. bootstrap	233.58 (14.80)	370.15 (23.46)	287.21 (15.80)	351.99 (19.36)	196.84 (8.83)	248.3 (11.13)	231.89 (10.07)	221.05 (9.60)
*P* value (*t*-test)	0.00E+00	2.82E–01	0.00E+00	4.72E–12	2.91E–12	5.43E–04	3.88E–12	8.21E–03

The ratio of drought responsive transcripts and NAT pairs is in parentheses (%). Sig.: Significantly responded to drought stress. Sense: sense transcripts from NAT pairs; NATs: natural antisense transcripts from NAT pairs; *P* value (χ^2^ test): *P* value of χ^2^: test for assessing differences between significantly up- or down-regulated genes of overall transcripts and NAT pairs identified in each line. *P* value (*t*-test): *P* value of *t*-test for assessing differences between significantly up- or down-regulated genes of bootstrap samples in overall transcripts compared with NAT pairs identified in each line.

We also compared drought-induced fold change in expression of sense and antisense transcripts within NAT pairs, housekeeping genes (47), lncRNA (48), and non-NAT transcripts in 1000 bootstrap re-samples. As expected, the fold change in expression of housekeeping genes under WS was closest to zero. In contrast, the fold change in expression of NATs, especially those up-regulated by WS, was higher than all other transcripts with the exception of lncRNAs (Figure 1B).

We also calculated expression variability using a standard deviation (SD) value, which was calculated from the log_2_ fold change under WS relative to WW (Figure 1C). The SD value for NATs was 2.41, which was significantly higher than that of randomly selected non-NAT transcripts in 1000 bootstrap re-samples with the same sample size (SD = 2.02, *t*-test *P* value < 0.001). These results suggest that NATs exhibit quantitatively greater variability under drought response than other genes in the genome. Interestingly, lncRNAs exhibit even higher fold change variability than NATs (Figure 1C).

Notably, we found that in 29.17% of NAT pairs (516 out of 1769), both the sense and antisense transcripts were drought-responsive in at least one of the four test lines, with a log_2_ fold change >1 or < –1 and FDR < 0.001. Of these, the sense and antisense transcripts in 431 pairs were concordantly regulated (both up or down) under WS. The correlation of fold change of sense and antisense expression was clear in dynamic transcriptome comparisons: the majority of up-regulated genes with at least 2-fold expression change in WS showed greatly increased antisense transcription as well, and repressed genes showed a substantial decrease (Figure 1D). In contrast, 80 NAT pairs were discordantly expressed under WS, with one transcript up-regulated and the other down-regulated. Only five NAT pairs were regulated concordantly in some maize lines and discordantly regulated in others.

A heat map of fold change in gene expression revealed consistently concordant (Figure 1E) or discordant response to drought (Figure 1F). To verify the specific NAT response to drought, sense transcripts from NAT pairs were paired with random, drought-responsive non-NAT transcripts. The fold-change profile in this artificial set was very different from that of NAT pairs, and contained significantly more discordant pairs (χ^2^ test *P* value < 0.001), and significantly fewer concordant pairs (Table 2).

**Table 2.** The number of NAT pairs genes showing concordant and discordant expression patterns in different maize lines

Material\categories		Discordant			Concordant			Total number
		UD	DU	Total	UU	DD	Total	
AC7643	Ob. (%)	18 (2.28)	20 (2.53)	38 (4.82)	79 (10.01)	80 (10.14)	159 (20.15)	197 (24.97)
	Ra. (%)	40.33 (5.11)	21.79 (2.76)	62.12 (7.87)	23.32 (2.96)	37.55 (4.76)	60.87 (7.72)	122.99 (15.59)
	Sig.	***	.	***	***	***	***	***
AC7729/TZSRW	Ob. (%)	9 (0.99)	30 (3.3)	39 (4.29)	181 (19.91)	37 (4.07)	218 (23.98)	257 (28.27)
	Ra. (%)	56.25 (6.19)	15.86 (1.74)	72.12 (7.93)	38.44 (4.23)	23.11 (2.54)	61.55 (6.77)	133.66 (14.70)
	Sig.	***	***	***	***	***	***	***
RIL64	Ob. (%)	4 (0.36)	3 (0.27)	7 (0.63)	49 (4.39)	60 (5.38)	109 (9.78)	116 (10.40)
	Ra. (%)	14.78 (1.33)	11.97 (1.07)	26.75 (2.40)	11.21 (1.01)	15.42 (1.38)	26.63 (2.39)	53.38 (4.79)
	Sig.	**	**	***	***	***	***	***
RIL208	Ob. (%)	3 (0.26)	6 (0.52)	9 (0.78)	58 (5.04)	54 (4.69)	112 (9.73)	121 (10.51)
	Ra. (%)	14.72 (1.28)	10.99 (0.95)	25.71 (2.23)	14.50 (1.26)	11.10 (0.96)	25.60 (2.22)	51.31 (4.46)
	Sig.	***	.	***	***	***	***	***

UD represents up-regulated sense and down-regulated antisense; DU represents down-regulated sense and up-regulated antisense; UU represents up-regulated sense and up-regulated antisense; DD represents down-regulated sense and down-regulated antisense; Ob. means the number of NAT pairs observed in the samples; Ra. means the average number of gene pairs detected in 1000 resamples; Sig. means *P* value in a significant test; *P value < 0.05; **P value < 0.01; ***P value < 0.001.

### Effect of drought stress on NAT expression

**Figure 2.**
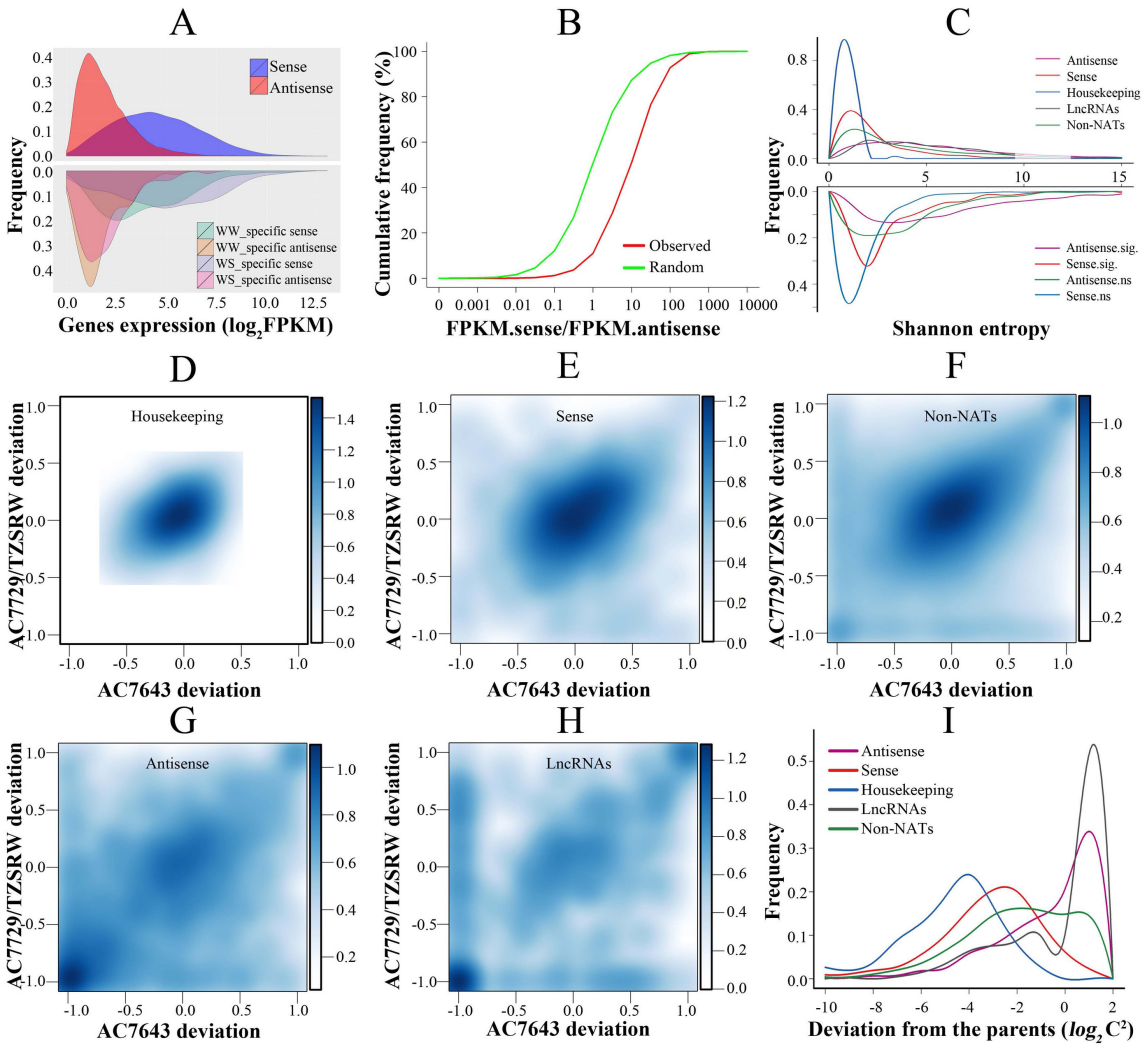
Expression and inheritance patterns of NAT pairs. (**A**) Density distribution for expression levels of sense and antisense transcripts, as well as WW-specific and WS-specific sense and antisense transcripts. Kinds of transcripts are as indicated. WW: well water; WS: water stress. (**B**) Cumulative frequency distribution of sense and antisense transcripts expression ratios. (**C**) Density distribution of Shannon entropy estimates of NATs, sense transcripts, housekeeping genes, lncRNAs and non-NATs, as well as antisense and sense transcripts whose response to drought was significant (sig.) or nonsignificant (ns). A color code is displayed in the top right corner and bottom right corner, respectively. (**D–H**) Two-dimensional density estimation of gene expression patterns in two recombinant inbred lines compared with two parental lines for housekeeping genes, NAT pair sense transcripts, non-NATs (non-housekeeping genes), NATs and lncRNAs, respectively. The x-axis and y-axis represent the gene expression-level deviation in recombination inbred lines relative to their parental lines, AC7643 and AC7729/TZSRW, respectively. (**I**) Distribution of expression-level deviations in recombination inbred lines relative to their parental lines, AC7643 and AC7729/TZSRW.

As shown in Figure 2A, there was, on balance, a significant difference in the distribution of FPKM values for sense and antisense transcripts. Expression of sense transcripts in NAT pairs was generally less variable than NATs, and was higher in 89.04% of the NAT pairs. In addition, NAT pairs only detected under WS showed higher expression levels than those detected only in the WW condition (Figure 2A). To test for consistency in the difference between sense and antisense transcripts, the ratio of FPKM_sense_ to FPKM_antisense_ was calculated for each NAT pair, and was found to be ∼10 on average (Figure 2B). This ratio was significantly higher in NAT pairs than in 1000 similarly sized bootstrap samples of adjacent non-NAT pairs (Figure 2B).

FPKM values for NAT pairs were then analyzed by non-parametric Spearman correlation to determine whether NATs can modulate expression of the corresponding sense transcripts. Intriguingly, expression was positively correlated between sense and antisense transcripts, with a correlation coefficient 0.23 (*P* value < 0.001). In convergent, divergent and enclosed categories, the correlation coefficients were 0.14, 0.18 and 0.30 (*P* value < 0.001), respectively. For NAT pairs expressed under WS, the coefficient was 0.19, 0.28 and 0.35, indicating significantly stronger correlation under stressed conditions (*P* value < 0.001 from 1000 permutation tests).

The specificity of NAT expression was investigated by calculating the Shannon entropy for FPKM values, which is a measure of the specificity of transcription under different conditions or in different samples (50). Using housekeeping genes as reference, NATs were more specifically expressed than sense transcripts from NAT pairs. In fact, expression of these NAT pair sense transcripts was relatively uniform across all four maize lines under WW and WS (Figure 2C). In addition, expression specificity was higher for drought-responsive NATs than for drought-insensitive NATs (Figure 2C). These results indicate that expression of NATs is specifically associated with the response to WS.

Expression of NAT genes in root tissues was compared between parental lines and offspring RILs using statistics developed for the analysis of maize lncRNAs (48), which were themselves used as reference, along with housekeeping genes (47). As expected, expression of the majority of housekeeping genes was centrally distributed, and did not vary significantly between parents and RILs (Figure 2D), in contrast to both sense (Figure 2E) and antisense transcripts in NAT pairs (Figure 2G). In particular, expression of NATs was uniformly scattered, indicating a wide difference in expression between parents and derivative RILs, as is also seen for lncRNAs (Figure 2H). Notably, expression of NATs was also significantly more variable between parental lines (Kolmogorov–Smirnov test *P* value < 0.001) than sense, non-NAT pair genes or housekeeping genes (Figure 2I).

### Translational efficiency of NAT pairs under drought stress

**Figure 3.**
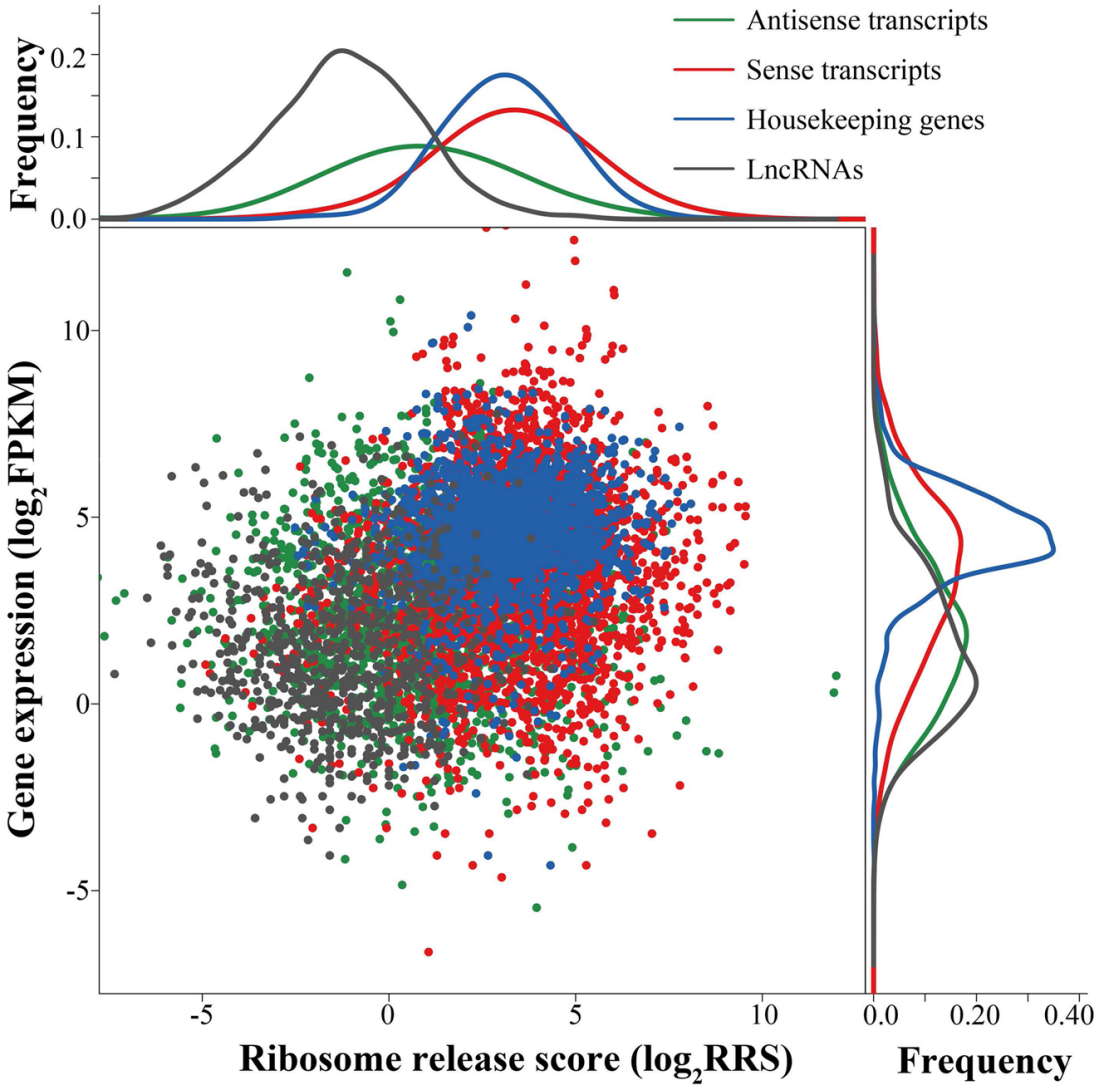
Correlation of gene expression and translational efficiency. A scatterplot of gene translational efficiency (log scale, y-axis) and expression level (log scale, x-axis) for sense and antisense transcripts, housekeeping genes and lncRNAs. Along each axis, all points are summarized using an overlaid density plot. The color code is displayed in top right corner.

Because they are often seen as a class of lncRNAs, the protein-coding potential of NATs has not been investigated (2). In our study, translation efficiency was compared between sense and antisense transcripts using RRS generated by ribosome profiling from B73 seedlings before and after drought stress. There were 944 NAT pairs that were present in both B73 seedlings and in the drought tolerant/sensitive lines. Housekeeping genes, lncRNAs, and non-NAT genes in 1000 randomized, bootstrapped samples were used for comparison. The average length of ORFs was 1237.42, 714.10 and 1011.67 bp in NAT pair sense transcripts, NATs and non-NAT transcripts, respectively. For NATs, the Spearman correlation coefficient for the length of ORF and the RRS was 0.32 (*P* value < 0.001), indicating that the longer ORF, the higher RRS value. No ORF was detected in 37.68% of the NATs. Not surprisingly, NATs are expressed less abundantly and have lower RRS values than sense transcripts from NAT pairs, indicating lower (but also more variable) translational efficiency (Figure 3). However, an unexpectedly high ratio of NATs (30.51%, or 288 out of 944 NATs) in B73 had RRS value greater than 3.36, which corresponds to the 95% quartile of lncRNA RRS values. The FPKM values of these NATs were also significantly higher than the other NATs, with Wilcoxon rank sum *P* value < 0.001. In contrast to that of NATs expression and translational efficiency of housekeeping genes were stable and consistent (Figure 3).

### smRNA enrichment in NATs and regulation by *Leafbladeless 1*

Given that NAT pairs have been associated with small RNAs in the past, and are sometimes directly regulated by small RNAs (29), we decided to examine our NAT pairs for evidence of enriched association with these RNAs. Identification and annotation of smRNA were performed in ShortStack (60). Spearman correlations for each sample between two replicates were all above 0.83, with an average of 0.85 (The Spearman correlation *P* value <0.001, Supplemental Table S2). The smRNAs identified in ShortStack with more than five smRNA mapped reads had sequence identity with 1595 NAT pairs (90.16%), and 80.63% (1286) of the NAT pairs overlapping regions matched more than five smRNA reads. 21 nt smRNAs were particularly enriched in these overlapping regions, although the most abundant unique smRNA overall were 24 nt (Table 3).

**Table 3.** SmRNA reads number per kb in NAT pairs genes

smRNA length	20nt		21nt		22nt		23nt		24nt		Total smRNA	
Categories	Num.	Cov.	Num.	Cov.	Num.	Cov.	Num.	Cov.	Num.	Cov.	Num.	Cov.
**Sense transcripts**	0.58	8.43	0.94	16.14	0.76	12.83	0.83	14.33	6.14	45.71	18.58	142.13
**Antisense transcripts**	0.50	8.74	0.89	17.66	0.67	13.24	0.63	13.52	4.57	36.36	17.07	144.47
**Non-NATs**	0.25	2.24	0.74	5.11	0.40	4.95	0.53	7.88	4.93	38.35	13.14	65.46
**Overlapping regions**	0.86	15.13	1.41	28.62	0.97	20.94	0.46	15.46	0.83	16.04	18.72	193.31

Num. means smRNA reads number per kb; Cov. means smRNA read coverage per kb.

**Figure 4.**
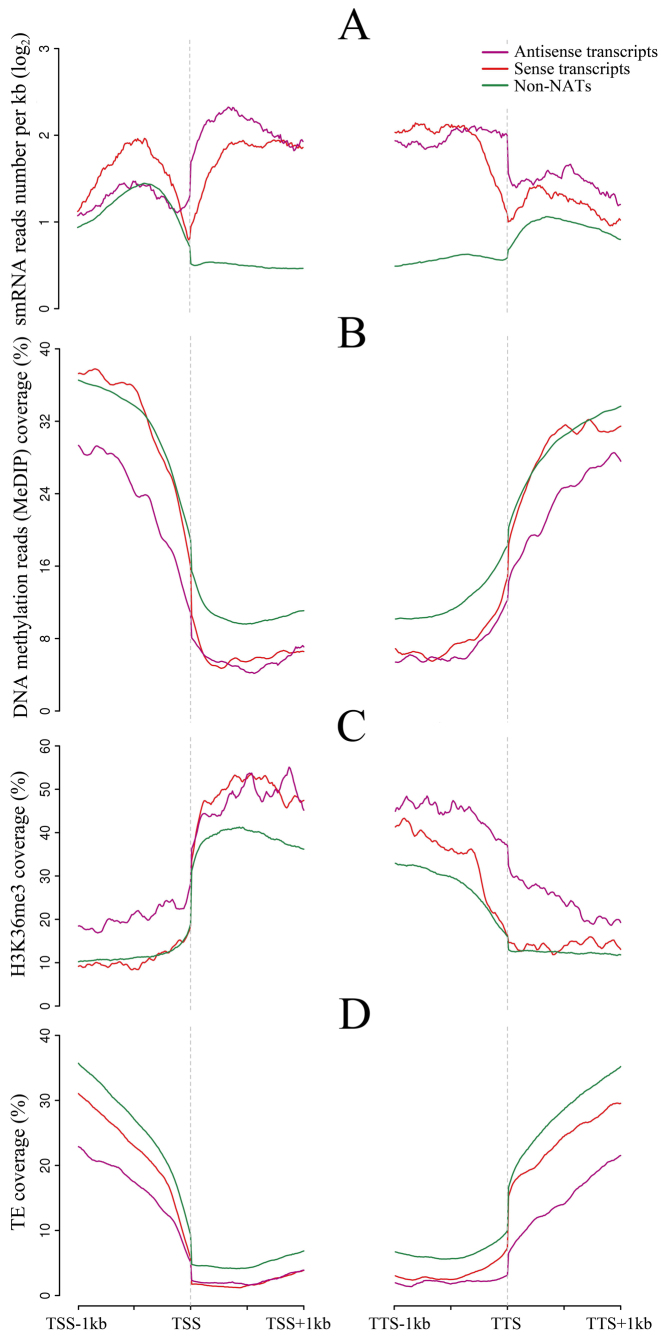
(A-D) The degree of enrichment of smRNAs, DNA methylation, H3K36me3 and TEs. The average enrichment levels are plotted with respect to transcription start sites (TSS) and transcription termination sites (TTS). The figure legend is in the top right corner. On the x-axis, kilobytes from the start and end of transcription are shown. In the y-axis, the number of reads was averaged in a 100 bp sliding window moving in 10 bp increments.

Figure 4A depicts smRNA enrichment 1 kb upstream and downstream of the transcription start and termination sites, as well as within the genes. The number of smRNA reads per kb was 13.14 in 1000 bootstrap samples of non-NAT genes, but 17.07 and 18.58 in NAT genes and the corresponding sense genes. In addition, average coverage (defined as the length of sequences covered by smRNA reads per kb was 65.46 (bp per kb) in non-NAT pools, but 142.13 and 144.47 in sense and antisense transcripts, respectively. Coverage was highest, at 193.31, in overlapping regions of NAT pairs (Wilcoxon rank sum, *P* value < 0.001, Table 3). 21nt smRNAs were enriched in gene body regions and 24 nt smRNAs were abundant in upstream and downstream flanking sequences of genes (Supplemental Figure S5B and E). These results indicate that smRNAs were significantly enriched both in and around NATs and 21nt smRNAs were more abundant at sense and antisense transcripts compared with non-NAT transcripts.

Transcripts with significant smRNA abundance changes under drought stress were also explored. A total of 5.41% of all transcripts showed a change (smRNA log_2_ fold change >1 or < –1 and FDR < 0.05) in the number of small RNAs associated with them under water stress. Notably, a significantly higher ratio of NAT pairs showed changes in smRNA abundance than did randomly selected gene sets (*t*-test, *P* value < 0.001, Supplemental Table S5). To further explore whether the changes of small RNA abundance under WS were associated with the differential expression of NATs, the correlation of transcripts expression fold changes and smRNA abundance changes under WS was investigated (Supplemental Figure S6). Generally, fold changes of smRNA and transcript abundance were positively correlated (Pearson correlations *P* value < 0.001), especially in gene body region. The smRNA fold changes in the flanking regions of NATs also correlated with expression levels, but this was not observed in sense transcripts (Supplemental Figure S6). These results suggest that changes in small RNA populations in NAT pairs are likely a consequence rather than a cause of changes in gene expression of these genes.

Dicer-like proteins have been shown to process at least some NATs into 21 nt smRNA (29,32,62). Components of the tasiRNA pathway, including RNA-dependent RNA polymerase 6 (*RDR6*) and *SGS3*, can also regulate NAT pairs (76,77). Thus, we surveyed smRNAs in mutants lacking *LBL1*, the maize homolog of *SGS3* (32). We found 21 nt smRNAs to be more strongly diminished than 24 nt smRNA in the mutants, as has been reported. The decrease was most pronounced in 21 nt smRNA matching NATs, particularly in regions that overlap with sense transcripts from NAT pairs (Supplemental Figure S5F).

### Hypomethylation and chromatin modification in NAT pairs

DNA methylation in NAT pairs was investigated by next-generation sequencing of immunoprecipitated methylated DNA (Supplemental Table S2). As before, 1000 bootstrapped samples of randomly selected non-NAT genes (without overlapped TEs) were used for comparison. The bodies of sense and antisense genes were found to be significantly less methylated than average genes (Figure 4B). However, sequences 1 kb upstream of the transcription start site were consistently hypomethylated only in antisense genes (Wilcoxon rank sum, *P* value < 0.001), but not in sense genes. The divergent type of NAT pair (head to head) showed a much higher level of DNA methylation than the convergent (tail to tail) type in the 1kb flanking sequences downstream of the 3΄ TTS (Wilcoxon rank sum, *P* value < 0.001, Supplemental Figure S7A).

As in the genome as a whole, in NAT pairs in the B73 genome the most methylated cytosines were found by bisulfite sequencing to be in the CG context, followed by CHG and then CHH (64) (Supplemental Figure S8). Methylated CG and CHG, but not CHH, were significantly hypomethylated relative to non-NAT genes in the main body of antisense genes, (Wilcoxon rank sum, *P* value < 0.001). Interestingly, CHH nucleotides were relatively hypermethylated 1 kb upstream of the transcription start site in sense genes and hypomethylated in antisense genes (Wilcoxon rank sum, *P* value < 0.001, Supplemental Figure S8).

Histone H3K36me3 trimethylation is often associated with active transcription and with alternative splicing (78,79). H3K4me3 promotes transcription (80), and H3 acetylation, especially H3K9ac, has been shown to be involved in transcription of light-responsive NATs (5). As shown in Figure 4C, H3K36me3 was significantly (Wilcoxon rank sum, *P* value < 0.001) enriched near the transcription termination site in NAT antisense genes, as well as in the 5΄ portion of the sense genes. Similar patterns were observed for H3K9ac and H3K4me3 (Supplemental Figure S9). In contrast, repressive H3K27me3 marks were consistently reduced in both sense and antisense NAT gene pairs relative to non-NAT genes (Supplemental Figure S9). Thus, the chromatin at NAT genes appears configured for active transcription. Intriguingly, NATs in the convergent category exhibited more enriched H3K4me3 and H3K36me3 in 1kb flanking sequences downstream of the 3΄TTS (Supplemental Figure S7B and C) than in the divergent category.

### Transposable element coverage in NATs

To determine whether transposable elements have an impact on antisense transcription, transposon coverage was compared between NAT pairs and 1000 bootstrapped samples of random non-NAT pairs. As can be seen in Figure 4D, transposon coverage 1 kb upstream of the transcription start site was significantly higher in non-NAT genes than upstream of genes with expressed NATs (Wilcoxon rank sum, *P* value < 0.001). However, transposon coverage of NATs in convergent (tail to tail) category was much lower 1 kb downstream of the 3΄TTS than the divergent type (Wilcoxon rank sum, *P* value < 0.001, Supplemental Figure S7D).

### Potential functions of NAT pairs responsive to drought stress in the association and bi-parental populations

**Figure 5.**
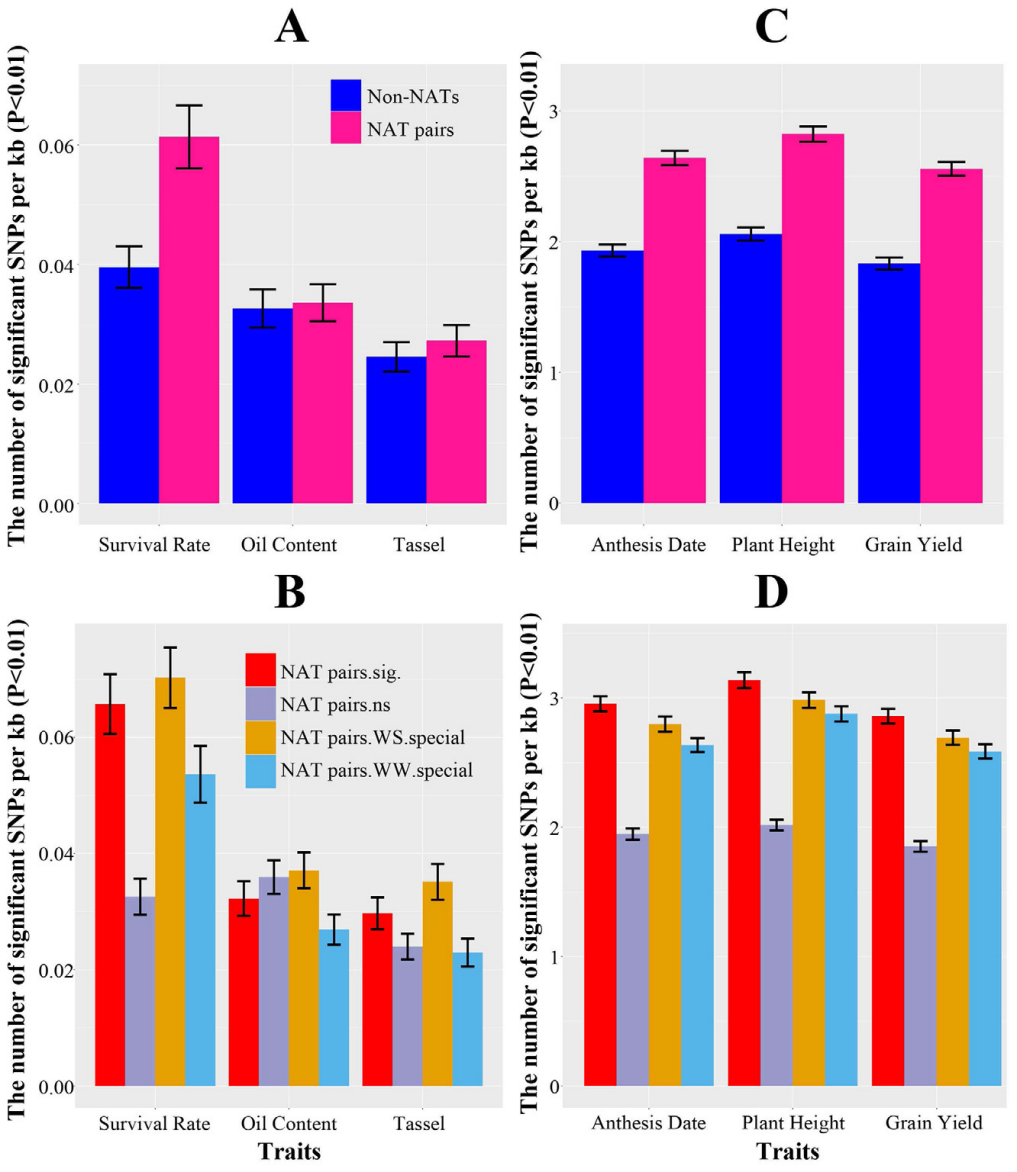
NAT pairs associated with drought-related traits in different populations. The average number of SNPs (per kb) associated with survival rate under water stress (WS) condition, kernel oil content and days to tassel under water well (WW) condition in maize association population for (**A**) NAT pair genes and non-NAT genes, (**B**) NAT pair genes that significantly responded to drought stress (NAT.sig), showed no response to drought stress (NAT.ns), only expressed under WS condition (NAT.WS.special) and only expressed under WW condition (NAT.WW.special). The average number of SNPs (per kb) associated with anthesis date, plant height and grain yield under WW in 19 bi-parental populations for (**C**) NAT pairs genes and non-NAT genes, (**D**) NAT pairs genes that significantly responded to drought stress (NAT.sig), showed no response to drought stress (NAT.ns), only expressed under WS condition (NAT.WS.special) and only expressed under WW condition (NAT.WW.special).

The association between drought tolerance and single nucleotide polymorphisms (SNPs) in NAT genes was evaluated in 368 maize inbreds representing tropical and temperate germplasms (66–68), as well as in 19 tropical maize biparental populations (71). Within the 368 maize inbreds, SNPs within 86.42% (2990 of 3460) of NAT pairs were informative, of which 13.28% (397 of 2990) were significantly associated (*P* value < 0.01) with drought survival rate in 368 maize inbreds (67) (Supplemental Table S6). The difference between the drought-associated NATs (13.28%) and bootstrapped samples of non-NAT genes (11.61%) is statistically significant (χ^2^ test *P* value 8.05E–3). In contrast, NAT pairs were not specifically enriched for SNPs associated with kernel oil content (66) or days to tassel (68) under normal conditions (Figure 5A). For transcripts with protein-coding potential (RRS > 3.36), nonsynonymous-to-synonymous substitution ratios of sense genes, antisense genes and non-NAT genes in the panel of 368 maize inbred lines were 0.73, 1.33 and 0.80, respectively. The ratio for antisense genes was significantly higher than both sense and non-NAT genes (Wilcoxon rank sum, *P* value <0.001).

The 19 biparental populations consisting of 3273 lines derived from crosses among 23 tropical maize inbreds were used to test associations between NAT polymorphisms and flowering, plant height, and grain yield under WS (71) (Supplemental Table S7). We found that 94.84% (2813 out of 2966) of NAT pair genes contained SNPs in genic regions with significantly association with drought tolerance (*t*-test with Bonferroni correction, *P* value < 0.01). On average, there were ∼2.68 SNPs per kb in each NAT pair gene linked to drought response, but only 1.94 in non-NAT genes. The difference is statistically significant by the Wilcoxon rank sum test (*P* value < 0.001) (Figure 5C). These SNPs were more heavily concentrated in drought-responsive NATs than in drought-insensitive NATs (Figure 5D).

SNPs linked to drought responsiveness were detected in 259 NAT pairs of genes common to association mapping and biparental populations (Supplemental File 6). Of these, 50.19% (130 out of 259) were significantly responsive to drought stress in either of the two parental lines or the offspring RILs. The difference between the drought-responsive NAT pairs with SNPs significantly associated with drought tolerance (50.19%) and corresponding bootstrapped samples of non-NAT genes (30.38%) is statistically significant (χ^2^ test, *P* value 3.42E–06). Moreover, many of these NAT pair genes, including transcription factors, oxidoreductases, and signal transduction factors such as serine/threonine protein kinase, have been previously demonstrated to control plant defenses against abiotic stress (30) (Supplemental File 6). Notably, overexpression of candidate gene, *ARGOS* (auxin-regulated gene involved in organ size) homolog3/auxin-inducible protein in maize, which is involved in regulation of cell expansion, resulted in a greater grain yield than non-transgenic controls under both WS and WW conditions (81).

## DISCUSSION

While ubiquitous in plants, the function of NATs is not well understood, particularly under environmental stress. Thus, we sought to identify *cis*NATs in maize and to investigate their potential contribution to drought tolerance. We identified 1769 NAT pairs in two maize inbred lines exhibiting different sensitivity to drought, as well as in two derivative RILs. We found that although NATs were expressed less abundantly than the corresponding sense transcripts, the expression is positively correlated between both in divergent and enclosed categories. Notably, this correlation is sensitive to WS conditions, such that both sense and antisense transcripts in these NAT pairs tend to be coordinately up or down regulated under water stress.

In both yeast and humans, the available data suggest a complex relationship between sense and antisense expression. In general, highly expressed NAT pair sense transcripts are negatively correlated with their cognate antisense transcripts, while sense-antisense gene pairs that are expressed at lower levels are positively correlated, with the positive effect generally predominating (4,15,18). Ultimately, we do not yet know enough about the causes or consequences of antisense transcription. One attractive model involves processing of double stranded RNA derived from pairing of sense and antisense transcript. In at least one documented case, both salt stress and the tasiRNA pathway have been implicated (29). In this case, enhanced expression of a non-coding antisense transcript results in a reduction of the NAT pair sense transcript. However, this may be only one of many effects of antisense transcription, as is suggested by the predominance of coordinate regulation of sense antisense pairs observed in yeast, mammals and plants (18,24). For instance, overall coordinate expression in NAT pairs that express at a low level may be due to processing of both transcripts due to interaction between them, and may be a mechanism for stable but rapidly alterable regulation of transcript levels (4). In addition, NATs may play a role in the deposition of chromatin modification. For instance, expression of *COOLAIR*, an antisense transcript that associated with heritable down-regulation of *FLC*, is required for deposition of the repressive mark H3K27me3 by the polycomb complex (28,82). Not all antisense transcription is correlated with down-regulation, however. Expression of *cis-*NAT_PHO1;2_ in Arabidopsis, for instance, is associated with increased levels of translation of the cognate *PHOSPHATE1;2* gene (24). In addition, NATs can activate expression of other genes such as observed for *HOTTIP*, whose expression is associated with activation of several *HOX* genes in drosophila via the deposition of marks associated with active chromatin (83). Finally, it should be noted that the presence of both sense and antisense transcripts in a complex organ like a root does not necessarily mean that both sense and antisense transcripts is actually produced within the same cell at the same time.

Based on all of these observations, the specific role of any of the NATs we have detected cannot be determined at this time, and indeed may vary depending on the particular NAT pair examined. We can, however, draw some general conclusions based on generic differences observed between NAT pairs and non-NAT genes in the tested maize lines.

As seen in Arabidopsis, NAT transcripts can be associated with the generation of smRNAs (29). Indeed, Arabidopsis siRNAs are enriched 6-fold in overlapping regions of *cis* NATs relative to non-overlapping regions (84). Presumably, this enrichment is due to the formation of double-stranded RNAs in overlapping regions, which are then processed by DICER-LIKE 1 and/or 3 (85), and amplified by RNA-directed RNA polymerase (85). In maize, we also found that smRNAs are enriched in NAT pairs, particularly in regions of transcript overlap. Further, we observed that those smRNAs were reduced in *lbl1* mutants, consistent with a role for *lbl1* in regulation of these genes.

In rice, a number of transposons appear to regulate nearby genes by antisense transcription (86). Similarly, miniature inverted-repeat transposable elements transcribed with coding genes form 1130 pairs of possible trans-acting sense/antisense transcripts (87). However, we find that transposable elements are significantly less frequently detected within maize NAT pairs than average genes, perhaps because these insertions are removed by purifying selection to prevent interference with interactions between sense and antisense transcripts. It should also be noted that 65.69% of NATs are transcribed from exons of sense genes, and only 12.72% from introns. Since exons of sense genes are rarely composed of transposable elements, our finding that antisense transcripts in maize rarely contain transposon sequences is unsurprising. Similarly, as maize transposons are highly methylated (64), the underrepresentation of transposons may also explain the observed relative hypomethylation in NATs, particularly those that are enclosed or read into a NAT pair sense transcript. It is, however, interesting to note that 19.03% of putative promoters for enclosed NATs are located in transposons, raising the possibility that TEs may in fact contribute to the formation of NAT pairs by enhancing expression of antisense transcripts. An analysis of polymorphic TE insertions in various backgrounds may help to determine if this is the case.

Chromatin marks such as histone methylation and acetylation greatly influence chromatin structure and gene function, as do the binding of an array of transcription factors (88). Of these, H3K36me3 and H3K4me3 are euchromatic, and are often enriched in highly expressed genes (89,90). In contrast, genes expressed at very low levels tend to be densely marked with H3K27me3 (34). Indeed, H3K27me3 marks are suppressive (78) and highly tissue-specific, implying that these marks are relieved only when gene expression is critically required (50,91). In at least some cases, antisense transcript has been found to trigger histone modifications (5,6). In maize and other plants, regions rich in transposable elements are depleted for the histone modifications H3K4me3, and H3K9ac, which typically mark active regions of chromatin (27). We found that these marks were enriched within the transcriptional start sites of sense and antisense gene pairs, consistent with the paucity of transposable elements in these regions and with their transcriptional activity. Similarly, the repressive mark H3K27me3 and repressive DNA methylation often occur in opposition to each other (92,93), presumably reflecting their distinct roles in gene regulation and transposon control and, again, consistent with the low frequency of transposons in NAT pairs. Overall, the relatively low level of H3K27me3, H3K4me3 and H3K9ac marks in maize NATs are consistent with active chromatin, and do not support the hypothesis that sense antisense gene pairs in maize generically trigger a repressed chromatin state.

Generally, RNAs can be classified into protein coding RNAs or regulatory RNAs. Recent findings in zebrafish and mammals suggest that many previously annotated lncRNAs may be translated (94). Indeed, lncRNAs have been shown to produce short peptides in six different species (95). Bi-functional RNAs with both protein coding and non-coding functions have emerged as new players in cellular systems (96). Similarly, a number of bacterial antisense RNAs have been found to encode functional proteins (97). In our study, NATs showed relative low RSS scores, but they were still higher than lncRNAs, indicating that some maize NATs may indeed be translated. Further, NAT genes with protein-coding potential based on their RSS score also showed significantly higher nonsynonymous-to-synonymous substitution ratios than non-NAT genes (Wilcoxon rank sum, *P* value < 0.001).

Functional characterization of some NAT pairs highlights the potential for NATs to regulate gene expression positively (24). The strong response of NAT genes to WS is an indication of physiological relevance in maize. The drought-induced fold change in NAT expression was highly variable, suggesting extensive regulation and on-demand adaptation to environmental changes. Gene families that function in essential metabolic process (e.g. the ubiquitin family) tended to have substantially lower nonsynonymous-to-synonymous substitution ratios, whereas gene families that participate in regulatory processes or signal transduction, have higher ratios (98). Since the expression of NATs was responsive and adaptive to abiotic stress, and many may not encode proteins, it is reasonable that they have higher average mutation rates.

It is important to acknowledge the null hypothesis, which is that under abiotic stress, many antisense messages are simply misregulated to varying degrees. The fact that in a few examples antisense transcripts are physiologically relevant does not in itself demonstrate that they are so in the majority of cases. However, we found that NAT pairs in over 360 maize inbred lines are specifically and significantly enriched for polymorphisms associated with survival rate, grain yield, plant height and anthesis under WS, but not for other traits such as oil content. Importantly, the association was highest for NAT pairs whose expression was specifically altered only under WS. Most of these candidate genes were previously demonstrated to involved in plant abiotic stress regulation and overexpression one of these candidate genes have proved to improve grain yield under both WW and WS conditions (81). These NATs are excellent candidates for further investigation.

## ACCESSION NUMBER

Raw sequencing data have been deposited in the NCBI Sequence Read Archive under accession number PRJNA294848 (SRP063383).

## Supplementary Material

Supplementary Data
