# Supplementary material for: Natural antisense transcripts are significantly involved in regulation of drought stress in maize
Source: Nucleic Acids Res. 2017 Feb 8;45(9):5126–41. doi: 10.1093/nar/gkx085 (PMC5435991; doi:10.1093/nar/gkx085)
Supplement: Supplementary Data [file gkx085_Supp.pdf]

Supplementary Material

Supplemental Figure 1. Saturation analysis for the samples with the highest and lowest reads mapping rate. The resampling in the x-axis resamples a series of subsets of data the from total mapped RNA reads and then calculates the RPKM value using each subset. Y-axis is “Percent Relative Error”, which is used to measures how the RPKM estimated from subset of reads deviates from real expression level. The highest reads mapping rate sample (RIL208-WS.repI) is in red and the lowest reads mapping rate sample (RIL64-WW.repI) is in blue. The yellow dashed line is 5%.

Supplemental Figure 2. Venn diagram depicting detectable NATs among different material and water conditions. The percentage of NATs is in red and the corresponding percentage of all detected transcripts is in black. (A) Percentages of NATs detected in different maize inbred lines. AC7729/TZSRW, RIL64, RIL208 and AC7643 were represented in green, purple, yellow and blue colors, respectively. (B) Percentages of NATs detected under well water (WW) and water stress (WS) conditions in parents and RILs. The parental lines (AC7643 and AC7729/TZSRW) and the RILs (RIL208 and RIL64) under WW were represented in blue and yellow, and the parental lines and RILs under WS were in purple and green. (C-D) Percentages of NATs detected under WW and WS conditions.

Supplemental Figure 3. Distributions of strand-specific RNA-seq reads of four NAT pairs as well as MeDIP-seq reads, smRNA-seq reads in WW and WS merged synthesized by IGV. Red and blue bars indicate ssRNA-seq reads mapped onto the minus strand and plus strand on chromosomes. WW: well water. WS: water stress.

Supplemental Figure 4. Relative expression of four NAT pairs under well water (WW) and water stress (WS) conditions detected by qPCR and separation of strand-specific reverse transcriptase PCR products on 1% agarose gel. Figure legend is in top left corner. The molecular weight marker (DNA ladder) is shown on the first lane. (A) The

NAT pairs genes GRMZM2G033219:GRMZM2G033430. (B) The NAT pairs genes GRMZM2G132212:GRMZM2G432801. (C) The NAT pairs genes GRMZM2G442277:GRMZM2G141704. (D) The NAT pairs genes GRMZM2G018595:GRMZM2G019500.

Supplemental Figure 5. The degree of enrichment of smRNAs in each class and relative smRNA expression level in *lbl1* and wild type (WT). The average enrichment levels of 20 nt (A), 21 nt (B), 22 nt (C), 23 nt (D) and 24 nt (E) smRNA are plotted with respect to transcription start sites (TSS) and transcription termination sites (TTS). (F) The ratio of smRNA reads number in *lbl1* and WT plants was investigated with respect to different categories of genes. The figure legend is in top right corner. 21nt and 24nt smRNA re represented in red and blue. The error bars represent 1.5 standard deviations from the mean.

Supplemental Figure 6. The correlation of transcript expression fold changes and smRNA abundance changes under water stress. Expression fold changes of sense transcripts, NATs and non-NATs were plotted on the x-axis. Small RNA abundance changes 1 kb upstream of TSS's, gene bodies and 1 kb downstream of the TTSs were plotted on the y-axis.

Supplemental Figure 7. The degree of enrichment of DNA methylation, histone marks and TE coverage plotted along the transcription start site (TSS) and transcription termination site (TTS) in different NAT pair categories. The figure legend is at the bottom. In the x-axis, kilobytes from the start and end of transcription are shown. (A-D) The average enrichment of DNA methylation, H3K4me3, H3K36me3 and TE coverage, respectively.

Supplemental Figure 8. DNA methylation levels in NAT pairs from genome bisulfite sequencing. The average DNA methylation levels in each context are plotted along transcription start site (TSS) and transcription termination site (TTS). The figure

legend is in top right corner. In the x-axis, kilobytes from the start and end of transcription are shown. In the y-axis, average coverage of DNA methylated bases was calculated in a 100 bp sliding window, moving in 10 bp increments. (A-C) DNA methylation was calculated for CpG, CHG and CHH context, respectively.

Supplemental Figure 9. The degree of enrichment of histone marks plotted along transcription start site (TSS) and transcription termination site (TTS). The figure legend is in top right corner. In the x-axis, kilobytes from the start and end of transcription are shown. (A-C) The average enrichment of H3K27me3, H3K9ac and H3K4me3, respectively.

Supplemental Table 1. ANOVA for root phenotypes among two maize parental lines and two recombinant inbred lines (RILs) under different water regimes.

Supplemental Table 2. The tested maize lines and read number from strand-specific RNA-seq, MeDIP-seq, small RNA-seq, ribosome profiling and ChIP-seq.

Supplemental Table 3. Detailed information for maize NAT pairs in four maize lines under two water regimes.

Supplemental Table 4. Information on primers used for experimental validation of natural antisense transcript pairs.

Supplemental Table 5. The number and ratio of transcripts and NATs pairs with significant smRNA abundance changes under drought stress in four maize lines.

Supplemental Table 6. Association analysis of natural variation in NAT pair genes with survival rate under drought stress in 368 maize inbreds.

Supplemental Table 7. Natural variation in NAT pairs genes associated with grain yield, plant height and anthesis date under drought stress in 19 maize bi-parental populations.

Supplemental File 1. Detailed methods and any associated references.

Supplemental File 2. The information of housekeeping genes in maize.

Supplemental File 3. The information of lncRNAs in maize.

Supplemental File 4. R script to scan genome-wide DNA methylation along genes in sliding windows.

Supplemental File 5. GTF File of NATs identified in eight samples.

Supplemental File 6. Functional annotations of NAT pairs genes with SNPs significantly associated with drought tolerance in both association and bi-parental populations.

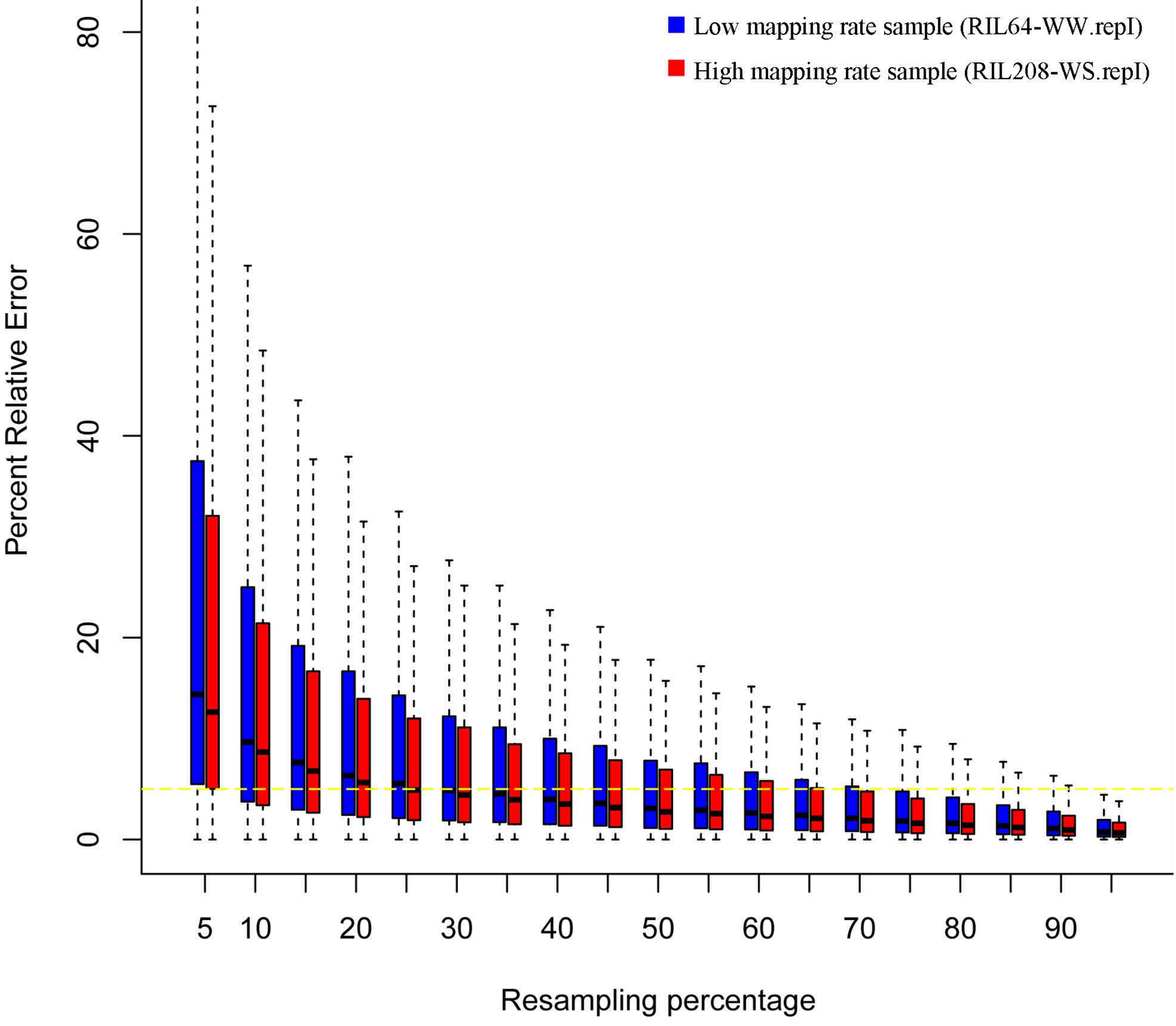

Supplemental Figure 1

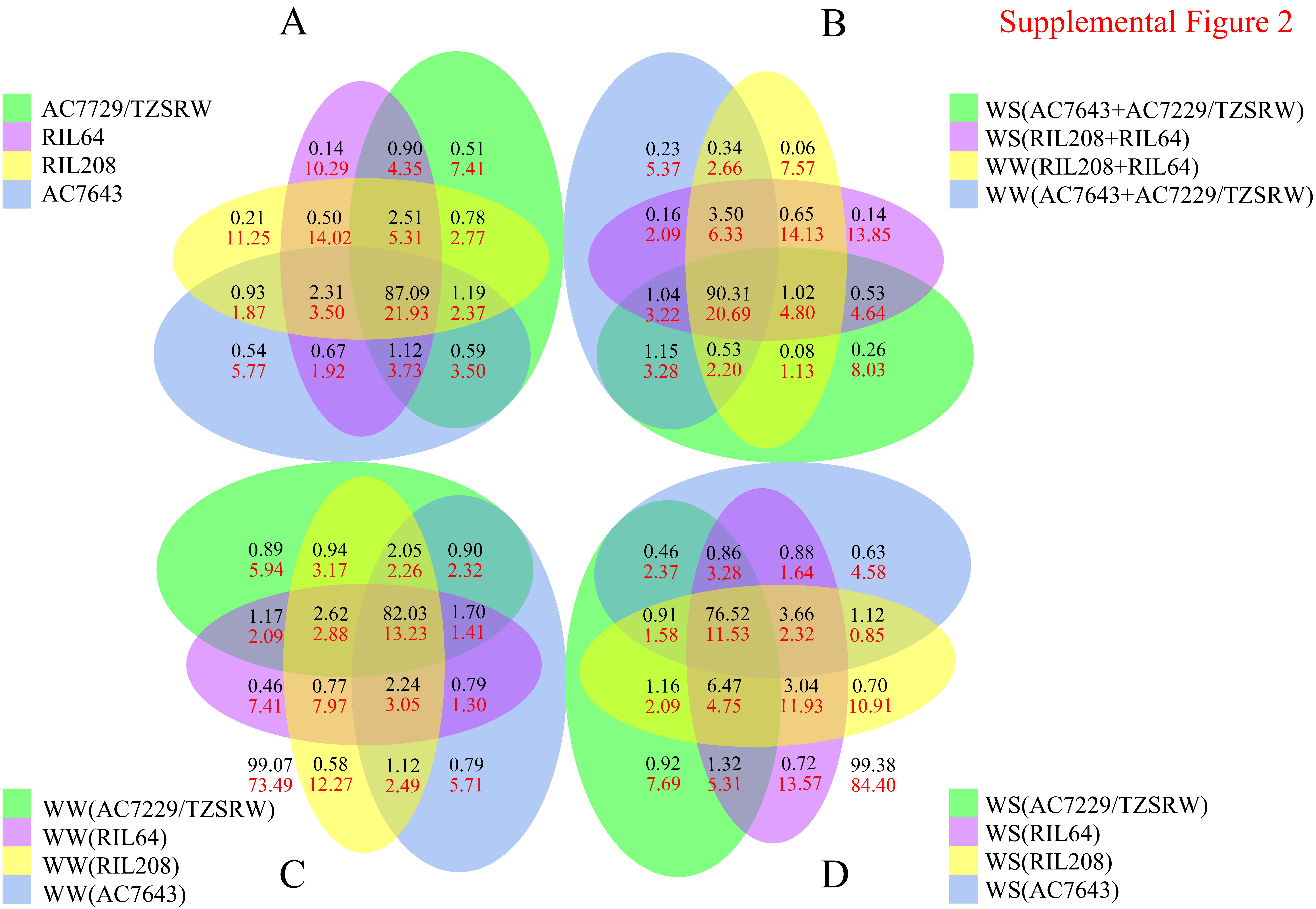
Supplemental Figure 2
A
AC7729/TZSRW
RIL64
RIL208
AC7643
0.14 10.29
0.90 4.35
0.51 7.41
0.21 11.25
0.50 14.02
2.51 5.31
0.78 2.77
0.93 1.87
2.31 3.50
87.09 21.93
1.19 2.37
0.54 5.77
0.67 1.92
1.12 3.73
0.59 3.50
B
WS(AC7643+AC7229/TZSRW)
WS(RIL208+RIL64)
WW(RIL208+RIL64)
WW(AC7643+AC7229/TZSRW)
0.23 5.37
0.34 2.66
0.06 7.57
0.16 2.09
3.50 6.33
0.65 14.13
0.14 13.85
1.04 3.22
90.31 20.69
1.02 4.80
0.53 4.64
1.15 3.28
0.53 2.20
0.08 1.13
0.26 8.03
C
0.89 5.94
0.94 3.17
2.05 2.26
0.90 2.32
1.17 2.09
2.62 2.88
82.03 13.23
1.70 1.41
0.46 7.41
0.77 7.97
2.24 3.05
0.79 1.30
99.07 73.49
0.58 12.27
1.12 2.49
0.79 5.71
WW(AC7229/TZSRW)
WW(RIL64)
WW(RIL208)
WW(AC7643)
D
0.46 2.37
0.86 3.28
0.88 1.64
0.63 4.58
0.91 1.58
76.52 11.53
3.66 2.32
1.12 0.85
1.16 2.09
6.47 4.75
3.04 11.93
0.70 10.91
0.92 7.69
1.32 5.31
0.72 13.57
99.38 84.40
WS(AC7229/TZSRW)
WS(RIL64)
WS(RIL208)
WS(AC7643)

Supplemental Figure 3

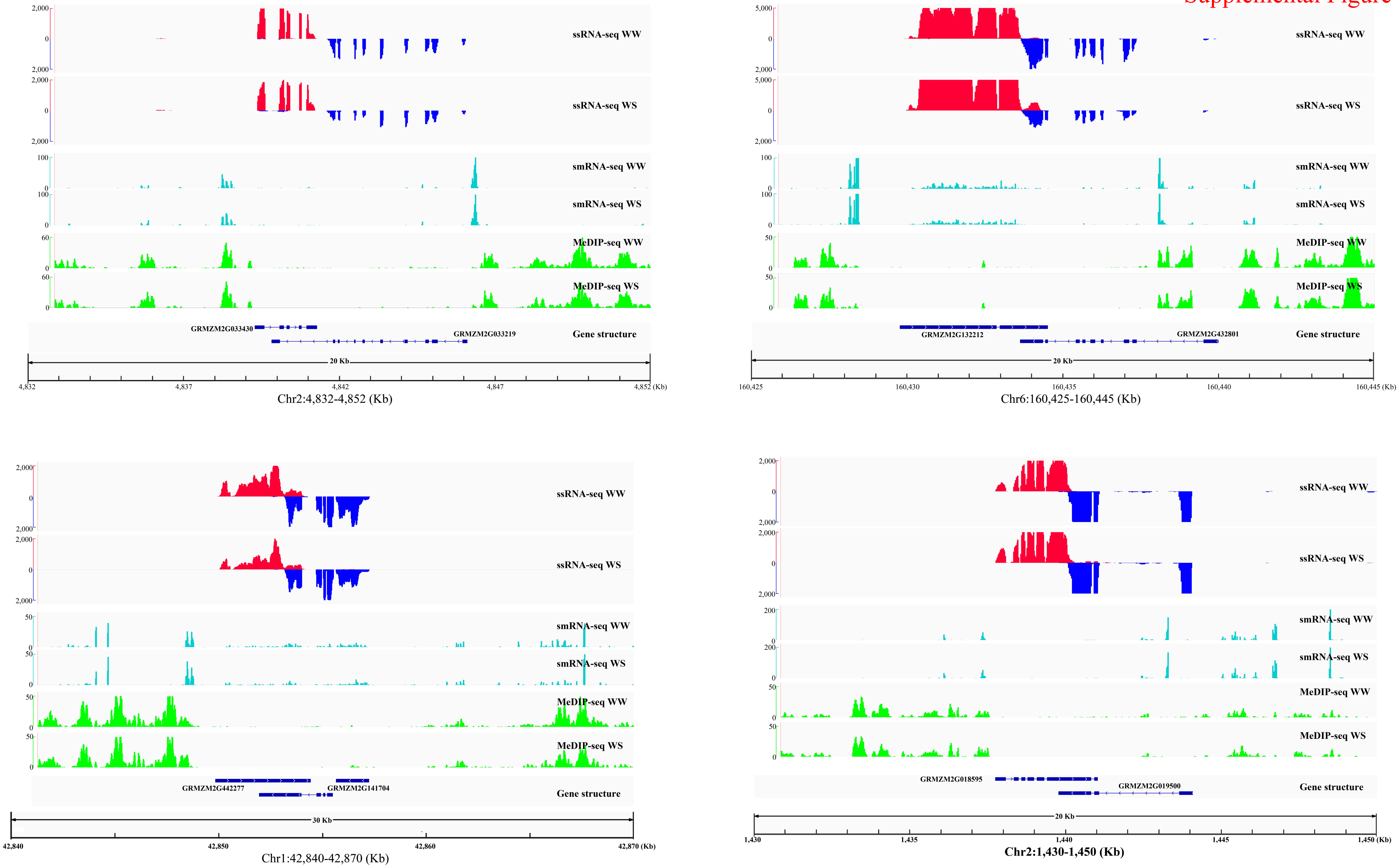

Supplemental Figure 4

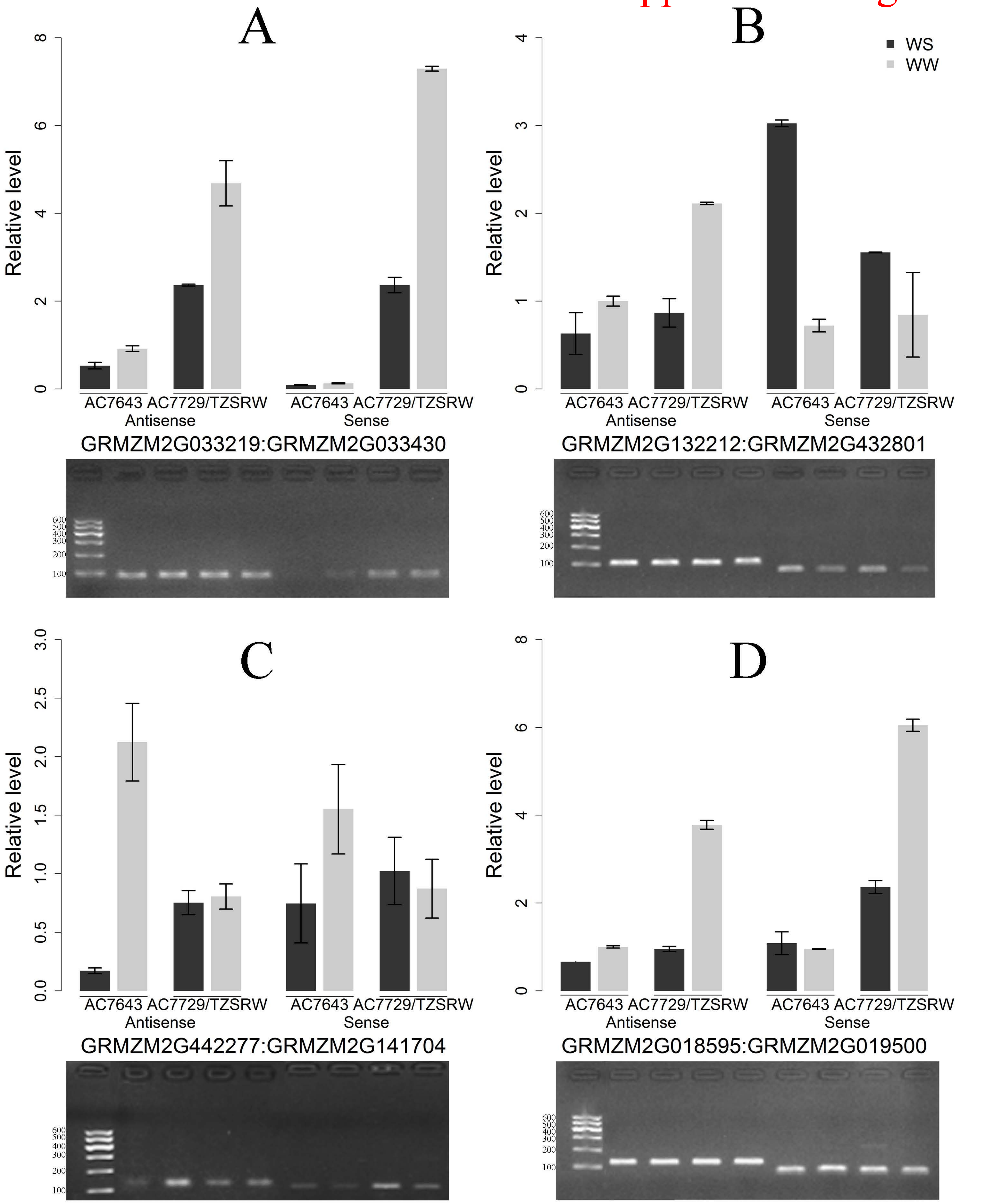

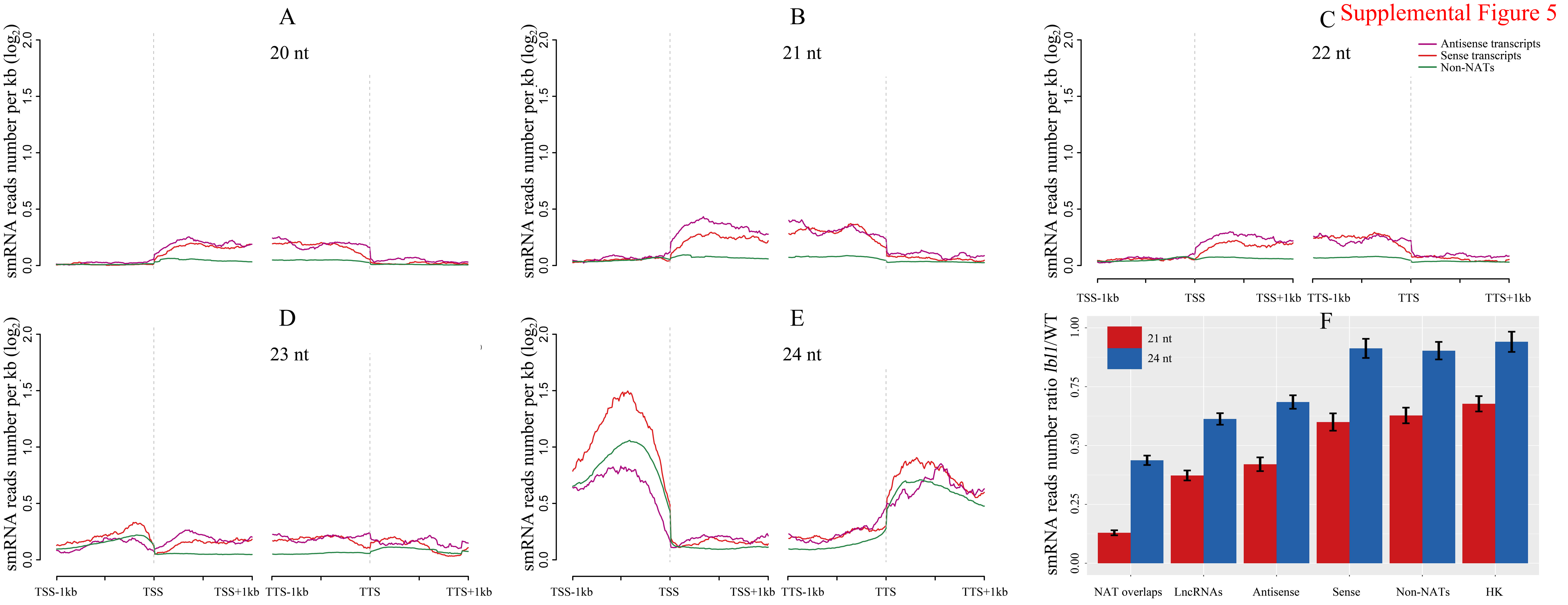
Supplemental Figure 5
A
20 nt
B
21 nt
C
22 nt
Antisense transcripts
Sense transcripts
Non-NATs
D
23 nt
E
24 nt
F
smRNA reads number per kb (log2)
TSS-1kb
TSS
TSS+1kb
TTS-1kb
TTS
TTS+1kb
21 nt
24 nt
smRNA reads number ratio lbl1/WT
NAT overlaps
LncRNAs
Antisense
Sense
Non-NATs
HK

Supplemental Figure 6

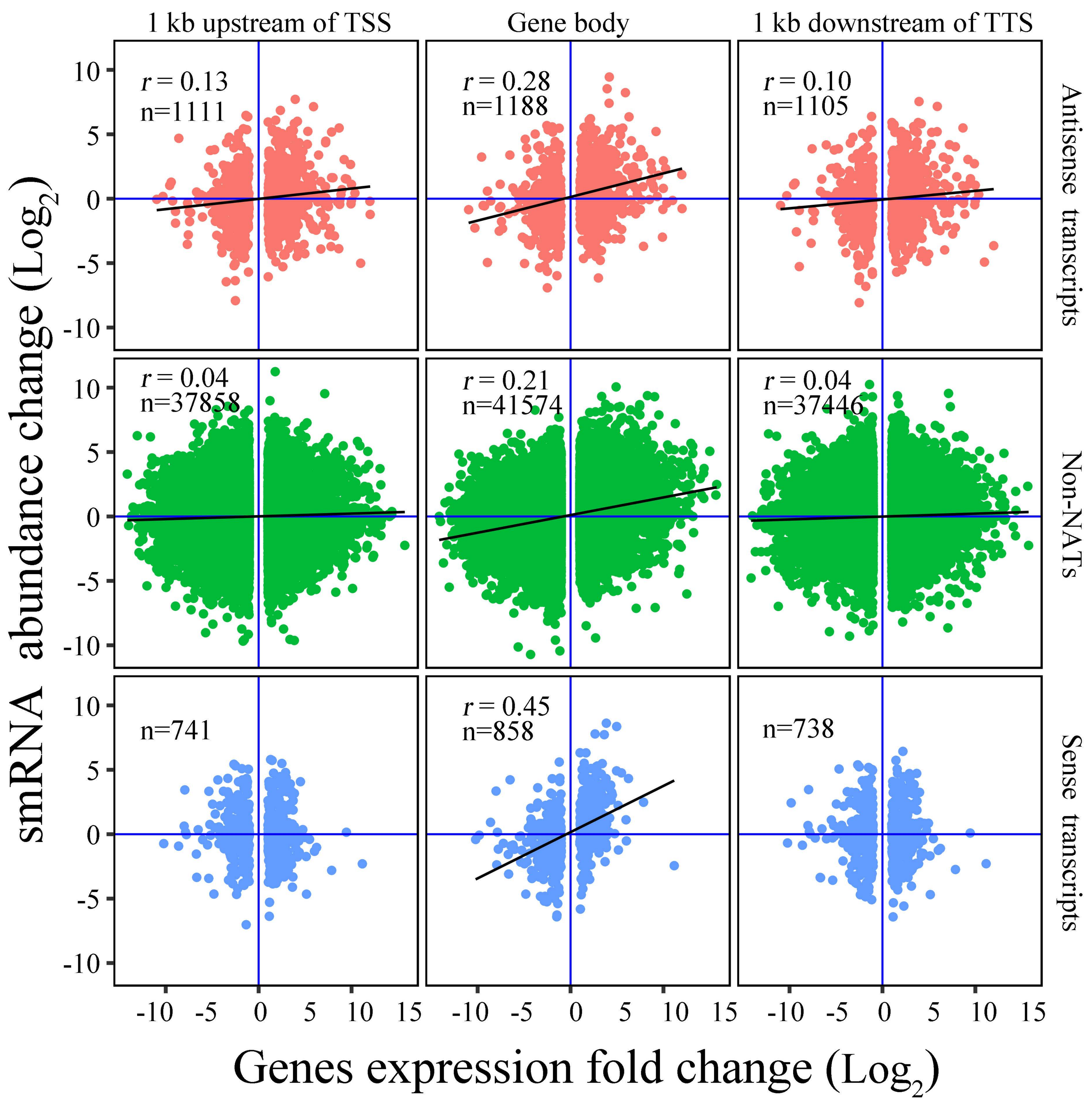

Supplemental Figure 7

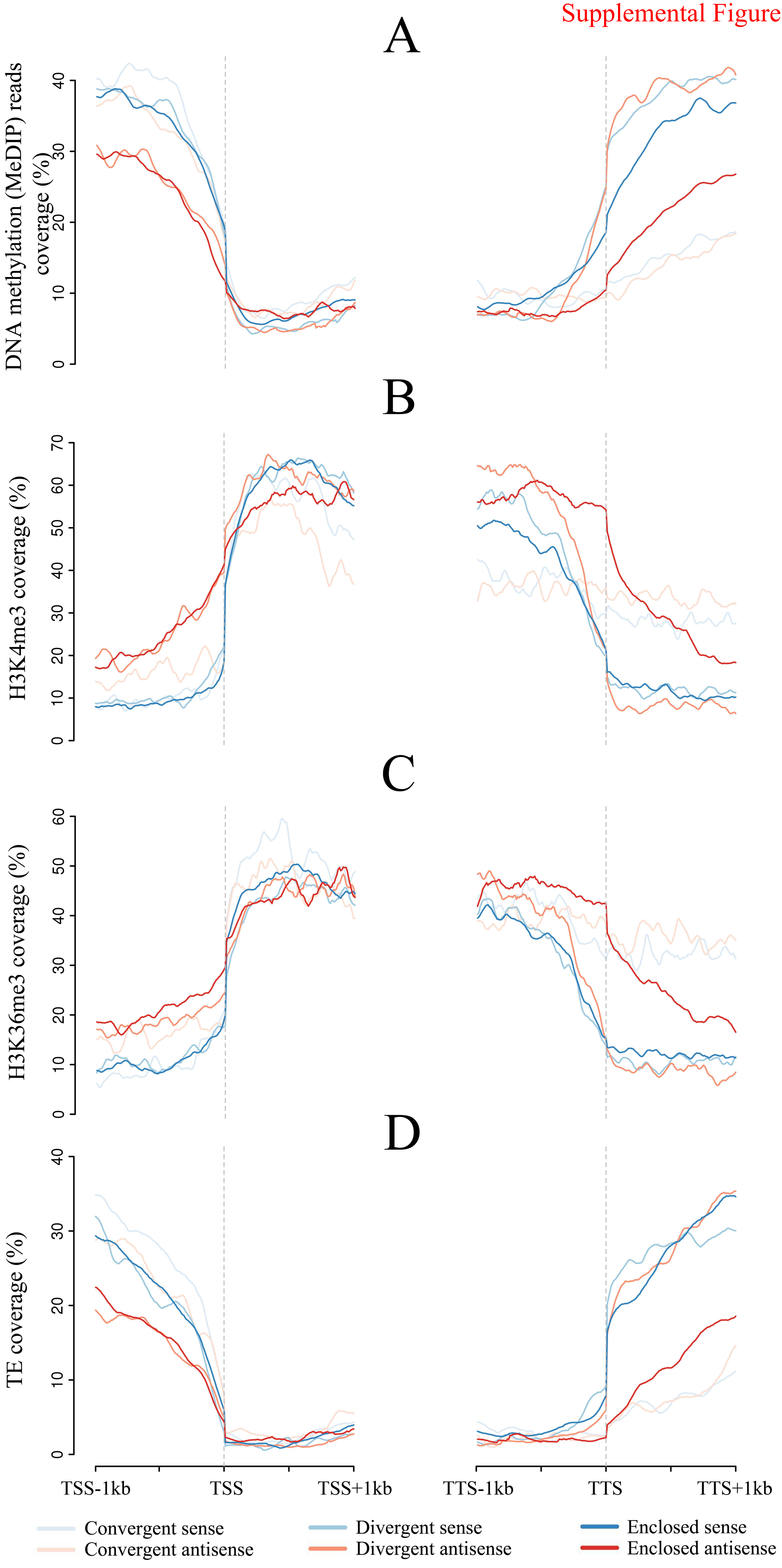

Supplemental Figure 8

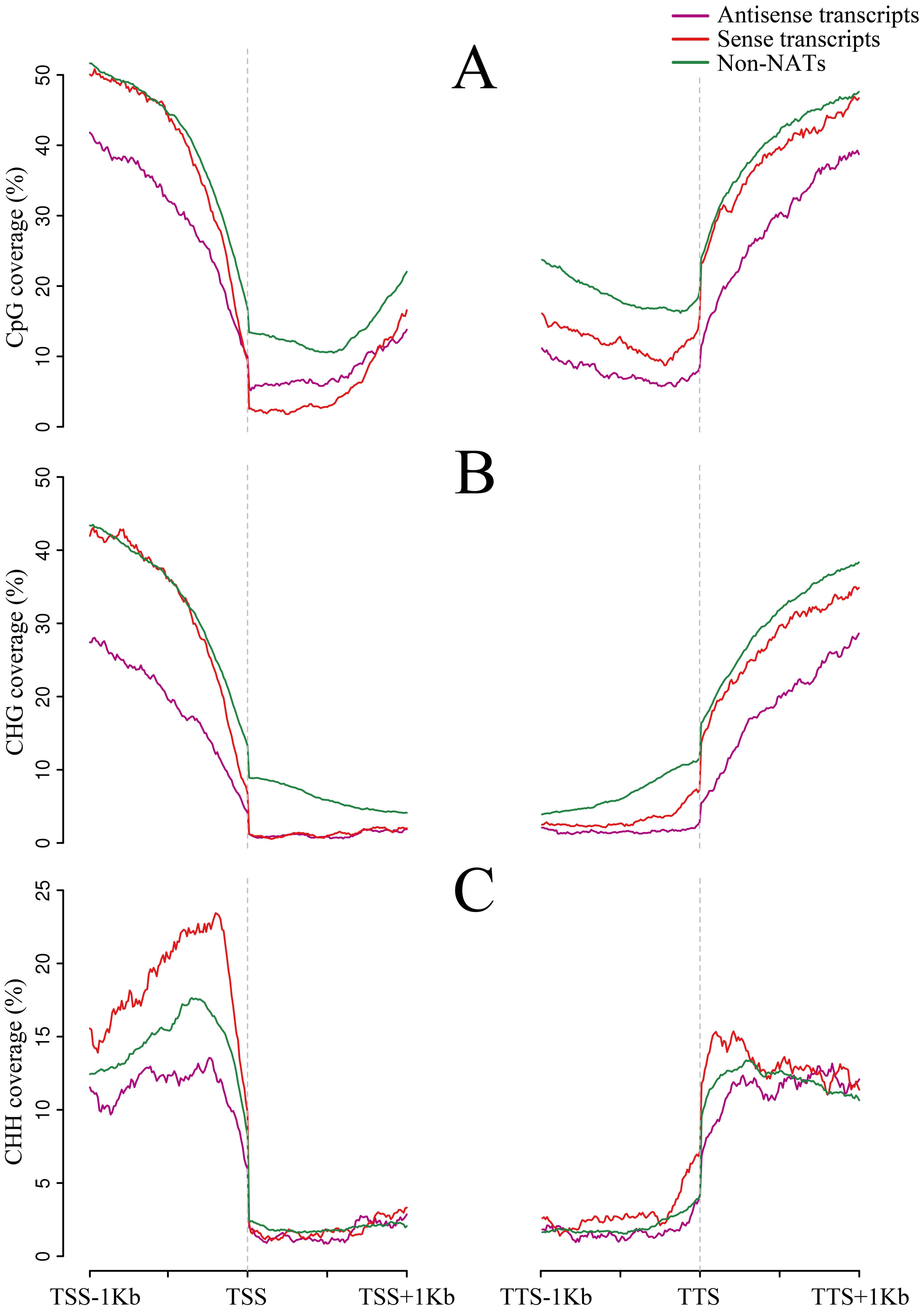

Supplemental Figure 9

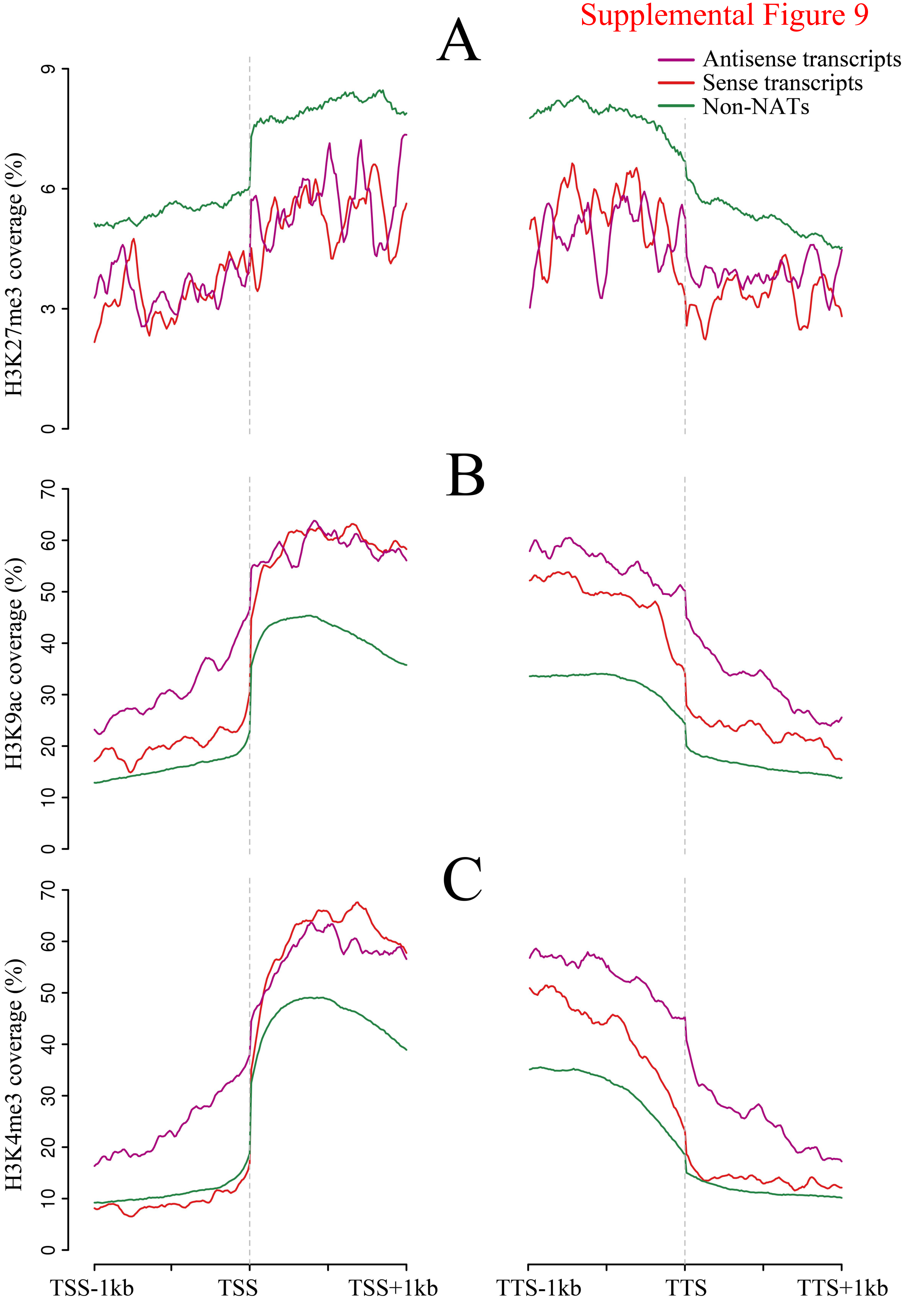

Supplemental Table 1. ANOVA for root phenotype among two maize parental lines and two recombinant inbred lines (RILs) under different water regimes

Root Traits	Condition	Length (cm)	Surf Area (cm2)	Total volume (cm3)	Forks	Tips
AC7643	WW	705.01	133.65	2.02	5813.30	990.67
	WS	416.56	74.94	1.07	4356.50	631.00
AC7729/TZSRW	WW	451.81	83.75	1.24	2666.00	745.00
	WS	195.21	39.99	0.65	1714.00	339.00
RIL208	WW	1066.29	217.76	3.40	6086.00	990.00
	WS	671.76	129.21	1.90	4996.00	694.00
RIL64	WW	282.41	54.30	0.74	1548.00	372.00
	WS	124.00	26.30	0.40	1012.30	172.60
ANOVA-Genotype		***	***	***	***	**
ANOVA-Water Stress		***	***	***	*	*

*P<0.05; **P<0.01; ***P<0.001. WW: well water; WS: water stress condition.

Supplemental Table 2. The tested maize lines and read number from ssRNA-seq, MeDIP-seq, smRNA-seq, ribosome profiling and ChIP-seq.

Library type	Sample	Reads number (Paired-end/Single-End)	Mapping parameter	Mapping reads number	Mapping rate (%)	The number of expressed genes in each sample	The number of expressed genes in each inbred line	The spearman correlation effeciency of two replicates (p value $\leqslant 0.001$)	ssRNA-seq protocol's strand specificity (%)
ssRNA-seq	AC7643-WW.rep1	53,985,634	max mismatch: 4 , max intron length: 2000 (unique match/best match)	45,246,934/47,368,070	83.81/87.74	30,374	31,308	0.82	99.81
ssRNA-seq	AC7643-WW.rep2	76,340,148	max mismatch: 4 , max intron length: 2000 (unique match/best match)	66,279,042/70,502,908	86.82/92.35				99.65
ssRNA-seq	AC7643-WS.rep1	52,626,438	max mismatch: 4 , max intron length: 2000 (unique match/best match)	44,949,974/46,808,124	85.41/88.94	28,193		0.86	99.82
ssRNA-seq	AC7643-WS.rep2	75,231,642	max mismatch: 4 , max intron length: 2000 (unique match/best match)	64,928,740/68,964,150	86.31/91.67				99.69
ssRNA-seq	AC7729/TZSRW-WW	54,509,240	max mismatch: 4 , max intron length: 2000 (unique match/best match)	46,438,370/48,563,802	85.19/89.09	30,599	31,388	0.87	99.84
ssRNA-seq	AC7729/TZSRW-WW	68,130,064	max mismatch: 4 , max intron length: 2000 (unique match/best match)	58,362,022/62,048,800	85.66/91.07				99.67
ssRNA-seq	AC7729/TZSRW-WS.	51,638,216	max mismatch: 4 , max intron length: 2000 (unique match/best match)	44,275,012/46,213,638	85.74/89.50	29,374		0.87	99.76
ssRNA-seq	AC7729/TZSRW-WS.	76,167,802	max mismatch: 4 , max intron length: 2000 (unique match/best match)	63,795,122/67,758,762	83.76/88.96				99.64
ssRNA-seq	RIL208-WW.rep1	44,832,440	max mismatch: 4 , max intron length: 2000 (unique match/best match)	34,227,508/35,861,196	76.35/79.99	30,614	31,664	0.82	99.60
ssRNA-seq	RIL208-WW.rep2	71,756,076	max mismatch: 4 , max intron length: 2000 (unique match/best match)	59,251,468/67,952,602	82.57/94.70				99.10
ssRNA-seq	RIL208-WS.rep1	46,251,296	max mismatch: 4 , max intron length: 2000 (unique match/best match)	40,804,194/42,635,014	88.22/92.18	31,019		0.80	99.50
ssRNA-seq	RIL208-WS.rep2	66,474,994	max mismatch: 4 , max intron length: 2000 (unique match/best match)	50,872,174/62,939,328	76.53/94.68				99.09
ssRNA-seq	RIL64-WW.rep1	46,557,362	max mismatch: 4 , max intron length: 2000 (unique match/best match)	33,467,052/35,067,296	71.88/75.32	30,430	31,574	0.88	99.71
ssRNA-seq	RIL64-WW.rep2	66,390,904	max mismatch: 4 , max intron length: 2000 (unique match/best match)	56,862,536/61,587,058	85.65/92.76				99.25
ssRNA-seq	RIL64-WS.rep1	46,108,128	max mismatch: 4 , max intron length: 2000 (unique match/best match)	39,967,514/41,664,712	86.68/90.36	30,984		0.89	99.52
ssRNA-seq	RIL64-WS.rep2	82,760,502	max mismatch: 4 , max intron length: 2000 (unique match/best match)	71,121,172/75,216,390	85.94/90.88				99.46
**ssRNA-seq**	**Total**	**979,760,886**	max mismatch: 4 , max intron length: 2000 (unique match/best match)	**820,848,834/881,151,850**	**83.78/89.94**	**33,149**	**33,149**	**0.85**	**99.57**
MeDIP-seq	AC7643-WW	97,959,184	Best match	91,111,382	93.01				
MeDIP-seq	AC7643-WS	97,959,184	Best match	91,241,445	93.14				
MeDIP-seq	AC7729/TZSRW-WW	97,959,184	Best match	90,976,635	92.87				
MeDIP-seq	AC7729/TZSRW-WS	97,959,184	Best match	90,948,091	92.84				
MeDIP-seq	RIL208-WW	97,959,184	Best match	91,199,050	93.10				
MeDIP-seq	RIL208-WS	97,959,184	Best match	91,340,718	93.24				
MeDIP-seq	RIL64-WW	97,959,184	Best match	90,892,449	92.79				
MeDIP-seq	RIL64-WS	97,959,184	Best match	90,977,969	92.87				
**MeDIP-seq**	**Total**	**783,673,472**	Best match	**728,687,739**	**92.98**				
small RNA-seq	AC7643-WW.rep1	8,320,262	Shortstacks default setting	4,831,918	58.07			0.83	
small RNA-seq	AC7643-WW.rep2	6,478,166	Shortstacks default setting	3,661,933	56.53				
small RNA-seq	AC7643-WS.rep1	8,920,598	Shortstacks default setting	4,962,508	55.63			0.88	
small RNA-seq	AC7643-WS.rep2	10,190,217	Shortstacks default setting	5,795,494	56.87				
small RNA-seq	AC7729/TZSRW-WW	7,463,160	Shortstacks default setting	4,386,433	58.77			0.87	
small RNA-seq	AC7729/TZSRW-WW	8,362,925	Shortstacks default setting	4,841,701	57.89				
small RNA-seq	AC7729/TZSRW-WS.	8,493,029	Shortstacks default setting	5,180,084	60.99			0.85	
small RNA-seq	AC7729/TZSRW-WS.	8,208,456	Shortstacks default setting	3,884,295	47.32				
small RNA-seq	RIL208-WW.rep1	8,424,134	Shortstacks default setting	5,433,457	64.50			0.83	
small RNA-seq	RIL208-WW.rep2	7,795,495	Shortstacks default setting	4,715,530	60.49				
small RNA-seq	RIL208-WS.rep1	8,613,297	Shortstacks default setting	5,759,263	66.86			0.85	
small RNA-seq	RIL208-WS.rep2	7,628,366	Shortstacks default setting	3,880,931	50.87				
small RNA-seq	RIL64-WW.rep1	7,850,335	Shortstacks default setting	5,127,773	65.32			0.83	
small RNA-seq	RIL64-WW.rep2	7,796,479	Shortstacks default setting	4,153,208	53.27				
small RNA-seq	RIL64-WS.rep1	8,069,513	Shortstacks default setting	5,297,396	65.65			0.83	
small RNA-seq	RIL64-WS.rep2	6,904,344	Shortstacks default setting	4,203,460	60.88				
**small RNA-seq**	**Total**	**129,518,776**	Shortstacks default setting	**76,115,384**	**58.77**			**0.85**	
RNA-seq	B73-WW.rep1	16,031,138	max mismatch: 4 , max intron length: 2000	14,362,914	89.59	26,319			
RNA-seq	B73-WW.rep2	15,805,238	max mismatch: 4 , max intron length: 2000	14,229,098	90.03	26,275	27,302		
RNA-seq	B73-WS.rep1	29,612,848	max mismatch: 4 , max intron length: 2000	26,293,646	88.79	26,224			
RNA-seq	B73-WS.rep1	18,275,884	max mismatch: 4 , max intron length: 2000	16,393,184	89.70	26,068	27,250		
**RNA-seq**	**Total**	**79,725,108**	max mismatch: 4 , max intron length: 2000	**71,278,842**	**89.41**	**28,466**	**28,466**		
ribosome profilir	B73-WW.rep1	33,515,204	Unique match	12,596,891	37.59				
ribosome profilir	B73-WW.rep2	36,651,421	Unique match	13,124,755	35.81				
ribosome profilir	B73-WS.rep1	19,535,941	Unique match	6,333,768	32.42				
ribosome profilir	B73-WS.rep1	21,276,437	Unique match	6,912,643	32.49				
**ribosome profili**	**Total**	**110,979,003**	Unique match	**38,968,057**	**35.11**				
small RNA-seq	*lbl* .rep2	8,464,869	Shortstacks default setting	3,595,321	42.47				
small RNA-seq	*lbl* .rep3	8,723,044	Shortstacks default setting	3,832,948	43.94				
small RNA-seq	WT.rep2	9,485,842	Shortstacks default setting	4,492,388	47.36				
small RNA-seq	WT.rep3	8,818,192	Shortstacks default setting	4,178,180	47.38				
**small RNA-seq**	**Total**	**35,491,947**	Shortstacks default setting	**16,098,837**	**45.36**				
ChIP-seq	H3K27me3_root	6,731,390	Unique match	981,907	14.59				
ChIP-seq	H3K36me3_root	11,607,120	Unique match	3,191,331	27.49				
ChIP-seq	H3K4me3_root	8,427,836	Unique match	2,495,694	29.61				
ChIP-seq	H3K9ac_root	11,220,867	Unique match	2,580,686	23.00				
**ChIP-seq**	**Total**	**37,987,213**	Unique match	**9,249,618**	**24.35**				

ssRNA-seq: strand specific RNA sequencing; MeDIP-seq: methylated DNA immunoprecipitation sequencing; smRNA-seq: small RNA sequencing; Chip-seq:Chromatin Immunoprecipitation sequencing.

Supplemental Table 3. Detailed information for maize NAT pairs in four maize lines under two water regimes.

Sense.name	Antisense.name	Transcription direction	Chromosome	Sense.start (bp)	Sense.end (bp)	Sense.strand	Antiense.start (bp)	Antiense.end (bp)	Antiense.strand
GRMZM2G032104	XLOC_000012	convergent	1	645,004	652,666	-	644,614	645,991	+
GRMZM2G172183	XLOC_003145	enclosed	1	979,720	991,166	+	988,460	990,951	-
GRMZM2G374779	GRMZM2G374812	enclosed	1	999,326	1,005,012	+	1,002,079	1,004,790	-
GRMZM5G802392	XLOC_003174	enclosed	1	2,839,306	2,840,779	+	2,839,365	2,840,264	-
GRMZM2G015384	GRMZM2G007025	convergent	1	2,873,956	2,894,310	-	2,871,385	2,875,166	+
XLOC_003179	XLOC_000050	convergent	1	3,127,903	3,129,982	-	3,125,558	3,129,266	+
XLOC_000052	XLOC_003179	divergent	1	3,128,229	3,130,830	+	3,127,903	3,129,982	-
XLOC_000052	XLOC_003180	enclosed	1	3,128,229	3,130,830	+	3,130,242	3,130,740	-
GRMZM2G055698	XLOC_003189	enclosed	1	3,462,290	3,464,383	+	3,462,444	3,462,724	-
GRMZM2G176840	XLOC_000098	divergent	1	4,758,424	4,766,151	-	4,765,059	4,766,393	+
GRMZM2G026223	XLOC_000099	convergent	1	4,855,668	4,871,367	-	4,855,614	4,857,299	+
GRMZM2G159477	XLOC_003216	divergent	1	5,039,291	5,042,641	+	5,038,799	5,040,058	-
GRMZM2G327059	XLOC_003218	enclosed	1	5,080,787	5,086,610	+	5,084,935	5,086,394	-
XLOC_000106	XLOC_003219	divergent	1	5,238,161	5,242,193	+	5,236,971	5,241,181	-
GRMZM2G176548	XLOC_003222	enclosed	1	5,435,030	5,436,495	+	5,435,129	5,436,156	-
GRMZM2G055704	XLOC_003226	enclosed	1	5,524,111	5,526,066	+	5,525,086	5,525,881	-
GRMZM2G032409	XLOC_000123	enclosed	1	6,213,707	6,218,417	-	6,217,891	6,218,279	+
GRMZM2G409976	GRMZM2G109509	convergent	1	7,078,142	7,082,865	+	7,079,988	7,085,969	-
GRMZM2G095968	XLOC_003263	enclosed	1	7,353,118	7,354,749	+	7,353,850	7,354,627	-
XLOC_003266	GRMZM2G110345	convergent	1	7,560,304	7,561,013	-	7,555,836	7,560,949	+
GRMZM2G110616	GRMZM2G410991	convergent	1	7,563,041	7,565,951	-	7,561,410	7,564,192	+
GRMZM2G075701	XLOC_003278	enclosed	1	8,025,073	8,027,145	+	8,025,460	8,025,864	-
GRMZM2G475168	GRMZM2G176523	enclosed	1	8,093,438	8,094,458	+	8,093,433	8,097,320	-
GRMZM2G041899	XLOC_003296	enclosed	1	9,117,659	9,120,601	+	9,117,974	9,120,417	-
GRMZM5G823004	XLOC_003298	enclosed	1	9,293,832	9,296,953	+	9,296,313	9,296,741	-
GRMZM2G415390	GRMZM2G415343	convergent	1	9,575,028	9,576,757	+	9,575,785	9,581,046	-
XLOC_000189	XLOC_003308	enclosed	1	10,069,416	10,069,763	+	10,069,416	10,069,760	-
XLOC_000190	XLOC_003309	enclosed	1	10,069,879	10,071,167	+	10,069,879	10,070,768	-
GRMZM2G086163	XLOC_000210	enclosed	1	11,473,558	11,475,611	-	11,473,930	11,474,971	+
GRMZM2G000749	XLOC_003336	enclosed	1	12,096,409	12,104,425	+	12,100,693	12,101,201	-
GRMZM2G377780	GRMZM2G377787	enclosed	1	12,172,623	12,177,552	-	12,173,174	12,174,065	+
GRMZM2G472266	XLOC_000228	divergent	1	12,251,757	12,253,695	-	12,252,948	12,254,065	+
GRMZM2G162749	XLOC_003373	enclosed	1	14,850,090	14,853,017	+	14,851,376	14,852,775	-
GRMZM2G139963	XLOC_000274	divergent	1	14,958,183	14,960,872	-	14,960,091	14,960,882	+
GRMZM2G035243	XLOC_003383	enclosed	1	15,168,071	15,171,112	+	15,168,080	15,168,574	-
GRMZM2G156486	XLOC_003390	enclosed	1	15,989,656	15,994,613	+	15,991,596	15,992,246	-
GRMZM2G012216	XLOC_000289	enclosed	1	16,199,110	16,209,371	-	16,202,286	16,202,881	+
GRMZM2G175164	XLOC_003399	enclosed	1	16,467,715	16,473,343	+	16,472,162	16,473,053	-
GRMZM2G129674	XLOC_000309	enclosed	1	17,459,873	17,462,949	-	17,462,417	17,462,811	+
AC206951.3_FG016	XLOC_000312	divergent	1	17,518,667	17,520,030	-	17,519,366	17,520,530	+
GRMZM2G109589	XLOC_003415	convergent	1	17,713,154	17,715,952	+	17,715,428	17,716,202	-
GRMZM2G148706	XLOC_000340	divergent	1	18,983,737	18,985,312	-	18,984,003	18,985,420	+
GRMZM2G006631	XLOC_003427	enclosed	1	19,098,935	19,101,431	+	19,099,356	19,099,730	-
GRMZM2G154007	XLOC_000345	enclosed	1	19,326,432	19,329,023	-	19,327,983	19,328,287	+
GRMZM2G464510	GRMZM2G464515	divergent	1	21,247,887	21,250,509	-	21,248,981	21,251,201	+
GRMZM2G006977	XLOC_000380	enclosed	1	22,378,485	22,384,451	-	22,383,382	22,384,394	+
GRMZM2G077127	XLOC_000381	enclosed	1	22,435,812	22,439,519	-	22,437,196	22,438,908	+
GRMZM2G055101	XLOC_003467	enclosed	1	22,638,292	22,642,889	+	22,642,061	22,642,416	-
GRMZM2G055217	GRMZM2G055172	divergent	1	22,645,597	22,650,341	+	22,643,460	22,647,118	-
GRMZM2G159632	XLOC_000403	enclosed	1	24,403,021	24,407,908	-	24,403,483	24,407,584	+
GRMZM2G163154	GRMZM2G163129	convergent	1	27,099,652	27,108,024	-	27,095,059	27,100,615	+
AC208897.3_FG004	XLOC_000429	enclosed	1	27,125,856	27,128,302	-	27,125,995	27,126,318	+
GRMZM2G091456	XLOC_003535	divergent	1	29,400,571	29,405,558	+	29,400,429	29,401,250	-
GRMZM2G153536	XLOC_000463	enclosed	1	29,512,941	29,516,697	-	29,513,532	29,516,007	+
GRMZM2G126808	XLOC_000465	enclosed	1	29,540,914	29,544,577	-	29,543,125	29,543,575	+
GRMZM2G012501	GRMZM2G012758	convergent	1	30,758,187	30,767,144	-	30,748,051	30,758,493	+
GRMZM2G116902	XLOC_000483	enclosed	1	31,253,767	31,255,665	-	31,254,138	31,254,520	+
GRMZM2G152663	XLOC_003560	enclosed	1	32,095,243	32,098,991	+	32,097,153	32,097,436	-
GRMZM2G099528	GRMZM5G884325	divergent	1	32,322,594	32,326,736	-	32,326,220	32,333,213	+
GRMZM2G077942	XLOC_000510	enclosed	1	32,873,395	32,876,087	-	32,875,353	32,875,768	+
GRMZM2G138103	XLOC_000539	enclosed	1	34,974,187	34,975,255	-	34,974,445	34,975,174	+
GRMZM2G338702	AC214040.3_FG009	enclosed	1	36,160,110	36,165,881	-	36,160,144	36,162,528	+
GRMZM2G060148	XLOC_000550	divergent	1	36,224,583	36,227,464	-	36,225,101	36,227,564	+
GRMZM2G128206	XLOC_003643	divergent	1	38,621,544	38,623,079	+	38,621,448	38,622,702	-
GRMZM2G160506	XLOC_000584	enclosed	1	39,115,144	39,118,333	-	39,117,344	39,118,213	+
XLOC_003652	XLOC_000585	enclosed	1	39,144,013	39,146,199	-	39,144,595	39,146,126	+
GRMZM2G179432	XLOC_003663	enclosed	1	40,062,759	40,065,030	+	40,064,443	40,065,015	-
GRMZM2G048482	XLOC_003681	enclosed	1	41,279,818	41,283,349	+	41,279,940	41,283,039	-
GRMZM2G043878	XLOC_000621	enclosed	1	41,440,932	41,442,284	-	41,441,234	41,441,685	+
GRMZM2G119886	XLOC_000625	enclosed	1	41,763,869	41,767,021	-	41,766,299	41,766,912	+
GRMZM2G442277	GRMZM2G141704	convergent	1	42,849,835	42,854,421	+	42,851,939	42,855,499	-
GRMZM2G017792	XLOC_000648	enclosed	1	43,690,068	43,692,986	-	43,692,630	43,692,886	+
AC212219.3_FG005	XLOC_000662	enclosed	1	44,959,567	44,965,488	-	44,959,729	44,965,076	+
GRMZM2G053669	XLOC_000663	enclosed	1	45,119,006	45,122,764	-	45,119,161	45,120,699	+
AC208201.3_FG002	XLOC_003727	enclosed	1	45,370,514	45,373,009	+	45,371,092	45,372,046	-
GRMZM2G087186	XLOC_000672	enclosed	1	45,517,326	45,520,489	-	45,519,821	45,520,211	+
GRMZM5G850640	XLOC_003731	convergent	1	45,637,463	45,640,713	+	45,640,504	45,641,402	-
GRMZM2G007206	XLOC_000682	enclosed	1	45,953,216	45,954,617	-	45,954,234	45,954,542	+
GRMZM2G144224	XLOC_000689	convergent	1	46,236,692	46,238,160	-	46,236,163	46,237,825	+
GRMZM2G010545	GRMZM2G313934	enclosed	1	46,844,940	46,848,551	-	46,845,162	46,845,951	+
GRMZM2G016734	XLOC_000708	enclosed	1	47,329,448	47,331,142	-	47,330,719	47,330,940	+
GRMZM2G377215	XLOC_003761	enclosed	1	47,508,955	47,511,718	+	47,510,283	47,511,240	-
XLOC_000719	XLOC_003766	enclosed	1	48,358,219	48,359,262	+	48,358,409	48,358,808	-
GRMZM2G318652	XLOC_003796	divergent	1	51,892,116	51,893,672	+	51,891,410	51,893,253	-
GRMZM2G009060	XLOC_000765	convergent	1	52,392,189	52,395,169	-	52,390,956	52,393,884	+
GRMZM2G033570	XLOC_000769	enclosed	1	52,561,965	52,565,938	-	52,562,240	52,564,454	+
GRMZM2G312661	XLOC_000790	enclosed	1	54,602,876	54,605,395	-	54,604,498	54,604,949	+
GRMZM2G064814	XLOC_003874	enclosed	1	57,893,672	57,900,344	+	57,893,719	57,895,045	-
GRMZM2G467072	XLOC_003898	divergent	1	60,155,734	60,157,039	+	60,155,654	60,156,604	-
AC204530.4_FG002	AC204530.4_FG003	convergent	1	61,615,166	61,623,614	+	61,623,111	61,624,072	-
GRMZM2G081322	XLOC_003919	convergent	1	61,934,146	61,944,291	+	61,943,533	61,944,858	-
GRMZM5G822100	XLOC_003931	divergent	1	62,561,406	62,563,927	+	62,561,354	62,562,760	-
GRMZM2G168665	XLOC_003932	divergent	1	62,678,621	62,679,843	+	62,678,619	62,679,687	-
GRMZM2G140989	XLOC_003934	enclosed	1	62,719,076	62,721,689	+	62,719,153	62,720,477	-
GRMZM2G026417	XLOC_000900	enclosed	1	64,021,077	64,022,794	-	64,021,299	64,021,898	+
GRMZM2G179792	XLOC_003969	enclosed	1	67,777,631	67,781,637	+	67,780,993	67,781,504	-
GRMZM2G382717	XLOC_000949	enclosed	1	68,121,169	68,122,907	-	68,121,352	68,121,966	+
GRMZM2G077897	XLOC_000952	convergent	1	68,568,918	68,572,988	-	68,568,365	68,569,907	+
GRMZM2G022061	XLOC_003977	enclosed	1	68,883,902	68,889,980	+	68,887,110	68,887,325	-
GRMZM2G049070	XLOC_003979	divergent	1	69,295,636	69,297,279	+	69,295,619	69,296,188	-
GRMZM2G071688	XLOC_003987	enclosed	1	70,582,852	70,585,097	+	70,583,867	70,585,080	-
GRMZM2G142390	XLOC_000969	divergent	1	70,775,839	70,780,013	-	70,779,206	70,780,258	+
XLOC_003999	XLOC_000973	enclosed	1	71,221,941	71,223,924	-	71,222,469	71,223,408	+
GRMZM2G304442	XLOC_001011	enclosed	1	74,718,500	74,719,924	-	74,719,104	74,719,886	+
GRMZM2G084587	GRMZM2G385050	convergent	1	76,878,489	76,889,924	+	76,889,570	76,904,210	-
GRMZM2G157164	XLOC_004055	enclosed	1	77,040,208	77,041,589	+	77,040,876	77,041,414	-
GRMZM2G044902	XLOC_001033	enclosed	1	77,152,281	77,154,119	-	77,153,673	77,153,979	+
GRMZM2G024175	XLOC_001037	enclosed	1	78,064,391	78,065,838	-	78,064,712	78,065,532	+
GRMZM5G826389	XLOC_004082	divergent	1	79,336,931	79,338,465	+	79,336,553	79,338,285	-
GRMZM2G143703	GRMZM6G399977	convergent	1	80,173,019	80,176,153	+	80,175,428	80,193,276	-
GRMZM2G025190	XLOC_004102	enclosed	1	81,450,983	81,452,132	+	81,451,571	81,452,080	-
GRMZM2G302373	XLOC_004104	enclosed	1	81,559,325	81,560,291	+	81,559,953	81,560,235	-
GRMZM2G178079	XLOC_004105	enclosed	1	81,588,714	81,589,906	+	81,589,129	81,589,704	-
GRMZM2G028821	XLOC_001068	enclosed	1	81,612,706	81,613,830	-	81,612,836	81,613,690	+
GRMZM2G026147	XLOC_004131	enclosed	1	85,027,826	85,029,741	+	85,029,194	85,029,701	-
GRMZM2G453832	GRMZM2G154549	divergent	1	85,998,283	86,001,830	-	86,001,434	86,004,907	+
GRMZM2G079470	XLOC_001134	enclosed	1	88,487,882	88,491,179	-	88,490,036	88,491,131	+
GRMZM2G089248	XLOC_004165	enclosed	1	89,004,748	89,006,489	+	89,004,822	89,006,240	-
GRMZM2G317386	XLOC_004174	convergent	1	90,797,641	90,799,416	+	90,798,196	90,800,178	-
GRMZM2G114772	GRMZM2G114707	convergent	1	92,248,217	92,252,805	+	92,250,389	92,253,227	-
GRMZM2G094639	XLOC_004184	enclosed	1	92,473,694	92,474,849	+	92,473,871	92,474,678	-
GRMZM2G161459	XLOC_001161	enclosed	1	92,793,547	92,797,895	-	92,794,480	92,794,761	+
GRMZM2G088524	XLOC_004191	enclosed	1	94,375,915	94,377,975	+	94,376,624	94,377,755	-
GRMZM2G178576	XLOC_001172	enclosed	1	94,691,673	94,693,914	-	94,692,189	94,692,906	+
GRMZM2G007939	XLOC_001182	enclosed	1	95,197,725	95,201,525	-	95,199,285	95,201,045	+
AC194914.3_FG002	XLOC_001211	enclosed	1	99,546,686	99,548,327	-	99,547,343	99,547,922	+
GRMZM5G866843	GRMZM2G089147	convergent	1	103,834,561	103,838,408	-	103,831,944	103,835,466	+
GRMZM2G012306	XLOC_004251	enclosed	1	106,902,765	106,905,799	+	106,903,378	106,904,918	-
XLOC_004266	XLOC_001267	divergent	1	111,555,606	111,556,464	-	111,555,834	111,558,264	+
AC211467.4_FG002	XLOC_004341	divergent	1	126,123,363	126,124,398	+	126,122,680	126,124,282	-
XLOC_004364	XLOC_001339	enclosed	1	137,254,058	137,256,652	-	137,254,903	137,255,792	+
GRMZM2G167584	GRMZM2G167649	enclosed	1	139,799,347	139,801,075	+	139,800,143	139,801,008	-
GRMZM2G159587	XLOC_001351	divergent	1	140,725,406	140,729,780	-	140,729,075	140,729,880	+
GRMZM2G479885	XLOC_004417	enclosed	1	147,170,692	147,172,458	+	147,170,825	147,172,007	-
GRMZM2G006704	XLOC_001374	enclosed	1	147,208,726	147,209,953	-	147,208,824	147,209,541	+
GRMZM6G924006	XLOC_004441	enclosed	1	151,734,008	151,736,151	+	151,735,404	151,736,135	-
GRMZM2G138419	XLOC_004442	enclosed	1	151,744,602	151,751,395	+	151,749,886	151,751,089	-
GRMZM2G056039	XLOC_004448	convergent	1	154,092,672	154,096,549	+	154,092,677	154,096,775	-
GRMZM5G878732	XLOC_001442	enclosed	1	161,322,162	161,324,184	-	161,322,448	161,323,383	+
GRMZM2G546081	GRMZM2G039112	convergent	1	162,442,194	162,449,008	-	162,442,172	162,444,014	+
GRMZM2G476637	XLOC_001451	enclosed	1	162,478,590	162,481,146	-	162,480,410	162,480,689	+
GRMZM2G428216	XLOC_004506	enclosed	1	165,420,861	165,422,748	+	165,420,894	165,421,789	-
GRMZM2G428216	GRMZM2G127739	convergent	1	165,420,861	165,422,748	+	165,422,123	165,427,760	-
GRMZM2G017486	XLOC_004512	divergent	1	166,943,082	166,945,351	+	166,942,218	166,945,246	-
GRMZM2G115615	XLOC_001519	divergent	1	170,979,650	170,986,759	-	170,986,484	170,986,768	+
GRMZM2G029262	GRMZM2G332821	convergent	1	172,141,002	172,147,261	-	172,138,864	172,141,926	+
GRMZM2G047835	XLOC_004575	enclosed	1	174,235,528	174,238,895	+	174,236,215	174,238,681	-
GRMZM2G022365	AC212976.3_FG003	convergent	1	175,322,870	175,330,028	-	175,321,975	175,323,121	+
GRMZM2G149751	XLOC_004593	convergent	1	176,865,344	176,871,673	+	176,870,093	176,873,163	-
XLOC_004593	XLOC_001559	divergent	1	176,870,093	176,873,163	-	176,872,260	176,873,419	+
GRMZM2G080603	XLOC_001566	enclosed	1	178,503,499	178,504,739	-	178,503,773	178,504,714	+
GRMZM2G093755	XLOC_001584	divergent	1	180,018,445	180,021,636	-	180,020,968	180,021,646	+
GRMZM5G893912	XLOC_001589	enclosed	1	180,635,433	180,637,762	-	180,635,595	180,637,502	+
GRMZM2G310947	XLOC_001590	enclosed	1	180,651,899	180,653,885	-	180,651,917	180,653,172	+
GRMZM2G423472	XLOC_004639	enclosed	1	180,715,451	180,720,707	+	180,717,585	180,718,436	-
GRMZM2G023791	XLOC_001611	divergent	1	182,125,031	182,126,803	-	182,125,586	182,126,923	+
GRMZM2G179351	XLOC_004665	enclosed	1	183,321,740	183,334,968	+	183,333,777	183,334,701	-
GRMZM2G042488	GRMZM2G042371	convergent	1	183,477,478	183,479,191	-	183,474,570	183,477,893	+
GRMZM2G083755	AC213857.4_FG003	enclosed	1	187,375,298	187,379,353	+	187,378,034	187,378,819	-
GRMZM2G441903	XLOC_004709	enclosed	1	187,432,586	187,435,072	+	187,433,111	187,434,213	-
GRMZM2G121312	XLOC_001677	divergent	1	187,917,195	187,919,314	-	187,917,466	187,919,447	+
GRMZM5G822593	XLOC_004719	enclosed	1	188,147,934	188,153,498	+	188,151,948	188,153,420	-
GRMZM2G022499	XLOC_001686	enclosed	1	188,491,054	188,494,924	-	188,493,609	188,494,765	+
GRMZM2G124557	GRMZM2G124638	convergent	1	189,385,201	189,390,321	+	189,389,542	189,393,722	-
GRMZM2G337191	XLOC_001725	enclosed	1	191,613,121	191,614,821	-	191,614,180	191,614,649	+
GRMZM5G828396	XLOC_004751	enclosed	1	192,065,376	192,068,429	+	192,065,391	192,067,850	-
GRMZM2G054252	XLOC_004779	enclosed	1	195,218,148	195,221,731	+	195,220,012	195,220,946	-
GRMZM2G054252	XLOC_004778	enclosed	1	195,218,148	195,221,731	+	195,218,610	195,219,235	-
GRMZM2G048129	XLOC_004788	enclosed	1	196,084,489	196,086,931	+	196,085,024	196,086,807	-
GRMZM2G069476	XLOC_001785	enclosed	1	196,458,606	196,463,550	-	196,458,881	196,460,106	+
GRMZM2G477872	XLOC_004800	convergent	1	197,104,152	197,113,073	+	197,110,037	197,113,267	-
GRMZM2G477872	XLOC_004799	enclosed	1	197,104,152	197,113,073	+	197,105,380	197,106,330	-
GRMZM2G175423	XLOC_004803	divergent	1	197,301,278	197,304,421	+	197,301,191	197,303,333	-
XLOC_004808	XLOC_001799	enclosed	1	197,743,008	197,745,407	-	197,743,016	197,743,467	+
GRMZM5G881887	XLOC_004833	enclosed	1	199,788,867	199,791,107	+	199,789,794	199,790,838	-
GRMZM2G104938	XLOC_004834	divergent	1	199,871,050	199,872,477	+	199,871,006	199,872,115	-
GRMZM2G457309	XLOC_004841	enclosed	1	200,504,395	200,505,328	+	200,504,383	200,506,023	-
GRMZM5G899800	XLOC_004849	enclosed	1	201,502,172	201,509,140	+	201,502,572	201,502,846	-
GRMZM2G318794	XLOC_004853	enclosed	1	201,668,843	201,669,743	+	201,668,928	201,669,258	-
GRMZM2G315375	XLOC_004857	enclosed	1	202,334,807	202,342,249	+	202,340,036	202,340,725	-
GRMZM2G179703	XLOC_004868	enclosed	1	202,955,781	202,958,669	+	202,955,781	202,956,669	-
GRMZM2G320689	GRMZM2G320708	convergent	1	204,903,793	204,910,161	+	204,909,826	204,911,008	-
GRMZM2G319357	AC212244.4_FG004	convergent	1	205,287,282	205,292,061	-	205,285,975	205,287,839	+
GRMZM2G401147	GRMZM2G101268	enclosed	1	208,340,735	208,341,691	+	208,340,744	208,341,268	-
GRMZM2G448185	XLOC_001946	enclosed	1	208,868,009	208,873,195	-	208,870,529	208,870,976	+
XLOC_004952	XLOC_001947	divergent	1	208,873,296	208,874,008	-	208,873,436	208,874,144	+
GRMZM2G428554	XLOC_001964	enclosed	1	210,641,599	210,646,145	-	210,641,726	210,643,665	+
GRMZM2G131205	XLOC_004979	convergent	1	211,567,120	211,572,922	+	211,570,193	211,573,240	-
GRMZM2G556078	XLOC_004983	enclosed	1	212,088,923	212,089,444	+	212,089,021	212,089,389	-
GRMZM2G167694	GRMZM2G167669	enclosed	1	216,747,715	216,750,376	-	216,747,936	216,748,356	+
GRMZM2G142249	XLOC_005022	enclosed	1	216,924,039	216,931,011	+	216,924,085	216,924,463	-
AC217910.3_FG004	XLOC_005026	enclosed	1	217,331,974	217,333,074	+	217,332,510	217,332,972	-
GRMZM2G114093	XLOC_002039	divergent	1	218,469,216	218,473,811	-	218,473,265	218,474,165	+
GRMZM2G003426	XLOC_002042	divergent	1	218,852,692	218,853,654	-	218,852,913	218,853,736	+
GRMZM2G321010	GRMZM2G015925	convergent	1	219,952,350	219,953,886	-	219,944,821	219,953,265	+
GRMZM2G136439	XLOC_005054	enclosed	1	220,147,969	220,150,934	+	220,148,133	220,148,722	-
GRMZM2G179063	XLOC_002070	divergent	1	221,239,135	221,240,968	-	221,239,260	221,241,115	+
GRMZM2G181236	XLOC_002108	enclosed	1	223,849,323	223,851,220	-	223,849,490	223,850,364	+
XLOC_002116	XLOC_005098	divergent	1	224,091,063	224,091,982	+	224,090,231	224,091,493	-
GRMZM2G414727	XLOC_005105	divergent	1	224,437,062	224,438,028	+	224,436,921	224,438,023	-
GRMZM2G172451	GRMZM2G475956	convergent	1	224,482,341	224,485,355	+	224,484,800	224,485,876	-
GRMZM5G882228	XLOC_005127	divergent	1	226,230,810	226,234,051	+	226,230,644	226,231,367	-
XLOC_005148	XLOC_002148	divergent	1	226,822,640	226,823,502	-	226,823,143	226,823,557	+
GRMZM2G082529	XLOC_005176	enclosed	1	227,894,328	227,899,992	+	227,894,417	227,895,044	-
GRMZM2G459291	GRMZM2G162758	convergent	1	229,666,605	229,670,274	-	229,664,358	229,667,449	+
GRMZM2G404443	XLOC_002182	enclosed	1	230,073,669	230,077,848	-	230,075,799	230,077,837	+
GRMZM2G010362	XLOC_002196	divergent	1	231,541,834	231,575,479	-	231,574,753	231,575,534	+
GRMZM2G134747	XLOC_002206	enclosed	1	233,290,453	233,293,630	-	233,293,225	233,293,591	+
GRMZM2G398198	XLOC_002210	divergent	1	233,875,216	233,877,958	-	233,875,715	233,878,017	+
GRMZM2G445905	XLOC_005257	enclosed	1	234,322,570	234,326,740	+	234,325,510	234,325,858	-
XLOC_002220	XLOC_005260	enclosed	1	234,403,006	234,404,583	+	234,403,212	234,403,769	-
XLOC_005265	XLOC_002224	convergent	1	234,658,115	234,661,066	-	234,657,581	234,660,961	+
GRMZM2G450825	GRMZM2G450762	enclosed	1	234,706,337	234,718,755	-	234,715,321	234,717,256	+
GRMZM2G020295	XLOC_005283	divergent	1	235,420,397	235,425,098	+	235,420,172	235,420,870	-
XLOC_002237	XLOC_005293	convergent	1	235,769,118	235,771,246	+	235,769,492	235,776,073	-
GRMZM2G029518	XLOC_002238	divergent	1	235,922,074	235,923,485	-	235,922,447	235,923,497	+
GRMZM2G152901	XLOC_005311	enclosed	1	237,009,950	237,014,064	+	237,009,966	237,013,206	-
GRMZM2G303965	GRMZM2G003724	convergent	1	237,545,346	237,548,821	-	237,544,777	237,546,713	+
GRMZM2G008290	XLOC_002254	convergent	1	237,715,089	237,717,335	-	237,715,070	237,716,604	+
GRMZM2G000540	XLOC_002264	enclosed	1	238,877,136	238,886,130	-	238,877,518	238,878,461	+

GRMZM2G016241	XLOC_002293	enclosed	1	241,430,778	241,431,741	-	241,430,167	241,431,867	+
GRMZM2G138455	XLOC_005351	enclosed	1	242,766,570	242,768,953	+	242,767,148	242,768,825	-
GRMZM2G146589	GRMZM2G146486	divergent	1	246,761,616	246,769,046	-	246,768,933	246,782,639	+
GRMZM2G153887	GRMZM2G153928	enclosed	1	248,481,668	248,482,632	+	248,480,724	248,509,681	-
GRMZM2G364069	XLOC_002389	enclosed	1	250,153,484	250,156,801	-	250,153,600	250,156,741	+
GRMZM5G805732	XLOC_005428	enclosed	1	251,500,979	251,505,855	+	251,503,379	251,503,728	-
GRMZM5G805732	XLOC_005429	enclosed	1	251,500,979	251,505,855	+	251,503,953	251,504,683	-
GRMZM2G066067	XLOC_002420	enclosed	1	253,370,904	253,375,697	-	253,373,863	253,375,695	+
GRMZM2G021055	XLOC_005451	enclosed	1	253,775,521	253,780,778	+	253,775,607	253,776,040	-
GRMZM2G017319	XLOC_005472	enclosed	1	255,383,820	255,386,002	+	255,383,820	255,384,076	-
GRMZM2G082686	XLOC_005475	convergent	1	255,803,441	255,804,699	+	255,803,514	255,804,904	-
GRMZM2G150772	XLOC_005479	enclosed	1	256,131,686	256,145,103	+	256,142,498	256,143,727	-
GRMZM2G409093	GRMZM2G106552	convergent	1	257,374,641	257,384,724	+	257,384,553	257,386,539	-
GRMZM2G055037	XLOC_005500	enclosed	1	258,031,281	258,033,115	+	258,031,736	258,032,238	-
GRMZM2G467893	XLOC_002505	enclosed	1	260,803,289	260,806,517	-	260,804,712	260,806,407	+
GRMZM2G068575	XLOC_002507	enclosed	1	261,178,421	261,192,543	-	261,191,923	261,192,453	+
GRMZM2G133684	GRMZM2G133633	convergent	1	261,723,724	261,726,331	-	261,720,652	261,725,055	+
GRMZM5G858417	XLOC_002528	convergent	1	263,064,702	263,068,643	-	263,063,270	263,066,541	+
GRMZM2G109056	XLOC_005585	enclosed	1	264,275,003	264,290,616	+	264,275,079	264,275,767	-
GRMZM2G033787	XLOC_005587	enclosed	1	264,577,362	264,580,333	+	264,579,109	264,580,330	-
XLOC_005589	XLOC_002553	enclosed	1	264,593,352	264,595,682	-	264,594,695	264,595,635	+
GRMZM2G704032	XLOC_002572	enclosed	1	265,971,512	265,979,530	-	265,979,009	265,979,330	+
GRMZM5G800853	XLOC_002587	divergent	1	267,614,418	267,624,839	-	267,624,371	267,624,858	+
GRMZM2G026780	XLOC_002634	enclosed	1	270,898,020	270,899,100	-	270,898,329	270,898,894	+
GRMZM2G153292	XLOC_005675	enclosed	1	271,178,054	271,180,939	+	271,179,186	271,180,879	-
GRMZM2G442658	XLOC_002674	enclosed	1	274,050,247	274,053,994	-	274,052,049	274,053,751	+
GRMZM2G001869	XLOC_005725	enclosed	1	274,785,474	274,790,748	+	274,789,368	274,790,183	-
GRMZM2G159295	XLOC_002694	enclosed	1	275,052,364	275,059,001	-	275,058,388	275,058,797	+
GRMZM2G159285	XLOC_002696	convergent	1	275,098,368	275,101,121	-	275,094,121	275,099,076	+
GRMZM2G169671	XLOC_002701	enclosed	1	275,290,498	275,294,240	-	275,293,889	275,294,213	+
GRMZM2G128564	XLOC_002702	enclosed	1	275,694,757	275,696,881	-	275,694,984	275,696,172	+
GRMZM2G377131	XLOC_002707	enclosed	1	275,759,689	275,761,549	-	275,759,900	275,761,423	+
GRMZM2G147687	XLOC_005755	divergent	1	276,371,897	276,377,338	+	276,371,474	276,372,465	-
GRMZM2G147687	XLOC_005756	enclosed	1	276,371,897	276,377,338	+	276,375,849	276,377,038	-
GRMZM2G017186	XLOC_005758	enclosed	1	276,389,960	276,395,246	+	276,393,398	276,393,847	-
GRMZM2G421491	GRMZM2G120484	convergent	1	276,882,941	276,887,539	-	276,876,174	276,883,112	+
GRMZM2G058032	XLOC_005776	enclosed	1	277,055,488	277,057,838	+	277,056,474	277,057,254	-
GRMZM2G057935	XLOC_002732	enclosed	1	277,059,425	277,064,749	-	277,063,140	277,064,199	+
GRMZM5G826714	XLOC_002739	enclosed	1	277,446,604	277,450,109	-	277,446,460	277,450,131	+
GRMZM5G826714	XLOC_002740	divergent	1	277,446,604	277,450,109	-	277,449,320	277,450,131	+
GRMZM2G090422	XLOC_005786	enclosed	1	277,805,999	277,807,838	+	277,806,426	277,807,762	-
GRMZM2G328500	XLOC_005789	enclosed	1	278,196,221	278,199,359	+	278,197,373	278,199,188	-
GRMZM2G091578	XLOC_002767	divergent	1	279,251,625	279,254,873	-	279,251,712	279,254,882	+
GRMZM2G047759	XLOC_002770	divergent	1	279,541,752	279,543,297	-	279,542,062	279,545,350	+
GRMZM2G047456	XLOC_005809	enclosed	1	279,632,338	279,634,477	+	279,633,156	279,634,477	-
GRMZM2G156800	XLOC_002778	divergent	1	280,154,367	280,163,933	-	280,163,123	280,164,102	+
GRMZM2G138861	XLOC_005819	enclosed	1	280,356,771	280,364,944	+	280,356,887	280,357,232	-
XLOC_002789	XLOC_005828	divergent	1	280,846,114	280,846,636	+	280,845,925	280,846,624	-
XLOC_005831	GRMZM2G072814	enclosed	1	280,894,123	280,894,576	-	280,894,065	280,895,067	+
GRMZM2G028286	XLOC_002799	enclosed	1	281,711,998	281,716,153	-	281,714,919	281,715,898	+
GRMZM2G427697	XLOC_002804	divergent	1	281,976,636	281,978,946	-	281,977,000	281,979,054	+
GRMZM2G165709	XLOC_002805	divergent	1	282,015,181	282,016,843	-	282,015,389	282,016,975	+
GRMZM2G040145	XLOC_002839	enclosed	1	285,280,649	285,281,991	-	285,281,158	285,281,565	+
GRMZM2G308687	XLOC_002841	divergent	1	285,410,446	285,411,691	-	285,410,613	285,412,021	+
GRMZM2G414540	XLOC_005910	enclosed	1	285,855,288	285,857,095	+	285,855,548	285,856,928	-
GRMZM2G329181	GRMZM2G028926	convergent	1	286,049,844	286,053,001	-	286,045,297	286,050,143	+
GRMZM2G019799	XLOC_002860	enclosed	1	286,429,050	286,437,267	-	286,432,765	286,437,028	+
GRMZM2G019799	XLOC_002859	convergent	1	286,429,050	286,437,267	-	286,428,657	286,429,831	+
GRMZM2G060765	GRMZM2G060924	convergent	1	286,736,778	286,741,262	-	286,730,567	286,737,443	+
GRMZM2G120674	GRMZM2G120657	convergent	1	286,916,349	286,921,522	+	286,920,950	286,923,692	-
GRMZM2G005991	XLOC_002873	enclosed	1	287,216,930	287,218,310	-	287,217,889	287,218,154	+
GRMZM2G178415	XLOC_002877	convergent	1	287,361,393	287,367,874	-	287,360,747	287,361,598	+
GRMZM2G178415	XLOC_002878	enclosed	1	287,361,393	287,367,874	-	287,361,426	287,363,549	+
XLOC_005938	GRMZM2G170851	divergent	1	287,541,417	287,542,365	-	287,541,494	287,544,013	+
GRMZM2G070322	XLOC_002882	enclosed	1	287,652,900	287,655,520	-	287,653,653	287,655,436	+
GRMZM2G453320	XLOC_005960	enclosed	1	288,992,277	288,993,039	+	288,992,430	288,992,879	-
GRMZM2G137321	XLOC_005965	divergent	1	289,403,622	289,405,770	+	289,402,962	289,405,468	-
GRMZM2G009901	XLOC_005986	enclosed	1	290,273,078	290,275,448	+	290,273,163	290,275,009	-
GRMZM2G082664	GRMZM2G432560	enclosed	1	290,337,450	290,359,023	+	290,344,248	290,346,241	-
GRMZM2G004511	XLOC_002940	enclosed	1	290,878,028	290,879,761	-	290,877,819	290,880,168	+
GRMZM2G347043	XLOC_006017	divergent	1	292,162,383	292,164,080	+	292,162,313	292,164,056	-
GRMZM2G151997	XLOC_002964	enclosed	1	292,412,495	292,413,903	-	292,412,705	292,413,447	+
GRMZM2G139250	XLOC_002967	enclosed	1	292,594,128	292,598,884	-	292,594,168	292,595,450	+
GRMZM2G155580	XLOC_006037	enclosed	1	293,065,790	293,074,538	+	293,066,717	293,066,966	-
GRMZM2G068244	GRMZM2G068443	divergent	1	293,721,769	293,724,068	-	293,722,839	293,726,484	+
GRMZM2G139041	XLOC_006075	enclosed	1	295,097,680	295,100,439	+	295,097,968	295,098,274	-
GRMZM2G139123	XLOC_006076	enclosed	1	295,100,532	295,101,411	+	295,100,872	295,101,411	-
XLOC_006078	XLOC_003015	enclosed	1	295,340,295	295,343,097	-	295,340,807	295,342,977	+
GRMZM2G156516	XLOC_006088	divergent	1	295,613,912	295,617,079	+	295,613,880	295,614,520	-
GRMZM2G366681	GRMZM2G448523	enclosed	1	296,067,223	296,069,043	-	296,062,422	296,069,083	+
GRMZM2G053916	GRMZM2G053803	convergent	1	296,076,874	296,085,483	+	296,082,206	296,088,792	-
GRMZM2G053803	GRMZM2G351160	divergent	1	296,082,206	296,088,792	-	296,088,128	296,096,856	+
GRMZM2G113158	XLOC_003038	enclosed	1	296,739,858	296,743,544	-	296,740,624	296,742,835	+
GRMZM2G338809	GRMZM5G883969	enclosed	1	296,788,163	296,790,581	+	296,789,724	296,790,547	-
GRMZM2G170137	GRMZM2G470422	convergent	1	296,849,361	296,855,071	-	296,847,943	296,850,494	+
GRMZM2G352415	XLOC_003050	enclosed	1	297,095,291	297,098,628	-	297,096,040	297,098,364	+
GRMZM2G016084	XLOC_003061	enclosed	1	297,827,097	297,832,384	-	297,830,785	297,832,332	+
GRMZM2G123901	XLOC_003067	enclosed	1	298,210,469	298,213,577	-	298,212,453	298,212,781	+
GRMZM5G876146	XLOC_006147	enclosed	1	298,813,813	298,817,252	+	298,814,031	298,814,417	-
GRMZM2G011923	XLOC_003086	enclosed	1	298,817,393	298,820,925	-	298,819,416	298,820,340	+
GRMZM2G100020	GRMZM2G099987	convergent	1	299,060,665	299,064,887	+	299,064,805	299,069,896	-
GRMZM2G115456	GRMZM2G417843	convergent	1	299,272,391	299,278,057	+	299,276,941	299,281,868	-
GRMZM2G363229	XLOC_003103	enclosed	1	299,980,376	299,982,620	-	299,980,674	299,981,223	+
GRMZM2G363229	XLOC_003104	divergent	1	299,980,376	299,982,620	-	299,981,775	299,982,624	+
GRMZM2G077183	GRMZM2G077222	convergent	1	300,547,988	300,550,532	-	300,543,777	300,548,423	+
GRMZM2G115162	GRMZM2G415077	enclosed	1	300,821,132	300,824,570	+	300,823,694	300,824,035	-
GRMZM2G131939	GRMZM2G132084	convergent	1	300,951,291	300,963,853	-	300,938,076	300,951,517	+
XLOC_011451	XLOC_008977	enclosed	2	177,675	179,791	-	177,859	179,378	+
XLOC_008979	XLOC_011457	enclosed	2	529,436	529,968	+	529,618	529,968	-
GRMZM2G064212	XLOC_011464	enclosed	2	1,038,129	1,042,294	+	1,038,189	1,038,486	-
GRMZM2G018595	GRMZM2G019500	convergent	2	1,437,737	1,441,025	+	1,439,756	1,444,066	-
GRMZM2G149958	XLOC_011485	enclosed	2	1,678,757	1,682,890	+	1,679,179	1,679,524	-
GRMZM2G078033	XLOC_011500	enclosed	2	2,366,176	2,369,867	+	2,366,238	2,366,609	-
GRMZM2G010257	GRMZM2G009994	divergent	2	2,549,740	2,552,578	+	2,544,036	2,550,544	-
GRMZM2G102349	XLOC_011507	enclosed	2	2,787,752	2,791,301	+	2,789,648	2,791,180	-
GRMZM2G043932	XLOC_009047	enclosed	2	2,821,204	2,822,336	-	2,821,513	2,821,874	+
GRMZM2G025536	XLOC_011511	divergent	2	3,019,428	3,021,698	+	3,018,548	3,020,854	-
GRMZM2G035325	XLOC_009065	enclosed	2	3,115,670	3,119,831	-	3,118,994	3,119,809	+
GRMZM2G119941	XLOC_011518	enclosed	2	3,191,332	3,193,885	+	3,192,839	3,193,216	-
GRMZM2G018716	XLOC_011519	enclosed	2	3,229,402	3,232,016	+	3,230,346	3,230,889	-
GRMZM2G044011	XLOC_009091	enclosed	2	3,928,213	3,938,819	-	3,938,479	3,938,709	+
GRMZM2G056513	XLOC_009093	enclosed	2	3,978,709	3,979,948	-	3,978,982	3,979,406	+
GRMZM2G040095	XLOC_011539	enclosed	2	4,192,323	4,196,291	+	4,194,864	4,195,911	-
XLOC_011543	GRMZM2G314171	enclosed	2	4,333,730	4,334,080	-	4,329,121	4,335,029	+
GRMZM2G033219	GRMZM2G033430	convergent	2	4,839,823	4,846,096	-	4,839,281	4,841,272	+
GRMZM2G032977	XLOC_009122	enclosed	2	4,852,765	4,855,560	-	4,853,539	4,854,344	+
GRMZM2G160585	XLOC_011565	enclosed	2	5,235,067	5,239,077	+	5,236,510	5,236,895	-
XLOC_011568	XLOC_009137	enclosed	2	5,306,289	5,309,292	-	5,306,604	5,307,559	+
GRMZM2G174917	XLOC_011572	enclosed	2	5,568,332	5,570,261	+	5,569,296	5,569,921	-
GRMZM2G174926	XLOC_009145	enclosed	2	5,572,045	5,578,697	-	5,572,048	5,572,757	+
GRMZM2G021635	XLOC_009154	enclosed	2	6,055,581	6,059,653	-	6,058,782	6,059,605	+
GRMZM5G829840	XLOC_009165	enclosed	2	6,642,341	6,646,369	-	6,642,462	6,643,178	+
GRMZM2G106245	XLOC_009175	enclosed	2	6,988,897	6,991,891	-	6,991,269	6,991,847	+
XLOC_011624	XLOC_009182	enclosed	2	7,047,927	7,051,492	-	7,049,348	7,050,124	+
GRMZM2G164400	GRMZM2G164277	convergent	2	7,519,507	7,527,867	+	7,527,470	7,531,925	-
GRMZM2G084181	XLOC_011646	enclosed	2	9,117,236	9,130,542	+	9,126,849	9,127,558	-
GRMZM2G094328	XLOC_011660	enclosed	2	9,551,436	9,552,864	+	9,551,732	9,552,554	-
GRMZM2G005310	XLOC_011677	enclosed	2	10,343,672	10,353,395	+	10,348,422	10,348,804	-
GRMZM2G005040	XLOC_011679	enclosed	2	10,387,109	10,389,573	+	10,387,374	10,389,429	-
GRMZM2G071832	XLOC_011714	divergent	2	11,769,293	11,771,169	+	11,769,111	11,771,067	-
GRMZM2G131280	XLOC_011739	enclosed	2	13,552,922	13,555,183	+	13,554,335	13,554,617	-
GRMZM2G131280	XLOC_011740	enclosed	2	13,552,922	13,555,183	+	13,554,719	13,555,152	-
GRMZM2G300111	XLOC_011741	enclosed	2	13,715,062	13,717,432	+	13,715,840	13,716,398	-
GRMZM2G314396	GRMZM2G017197	convergent	2	14,548,496	14,554,289	-	14,545,767	14,549,044	+
GRMZM2G314396	XLOC_009351	enclosed	2	14,548,496	14,554,289	-	14,552,836	14,553,772	+
XLOC_009355	XLOC_011760	enclosed	2	14,732,437	14,733,097	+	14,732,505	14,732,883	-
GRMZM2G080992	XLOC_009380	divergent	2	16,654,034	16,656,556	-	16,654,335	16,656,671	+
GRMZM2G379540	GRMZM2G076841	enclosed	2	17,085,454	17,092,794	-	17,085,870	17,090,770	+
GRMZM2G145968	XLOC_009400	enclosed	2	17,972,445	17,980,306	-	17,979,433	17,979,702	+
GRMZM2G174807	GRMZM2G175504	convergent	2	18,757,659	18,759,526	-	18,735,506	18,758,909	+
GRMZM2G320827	XLOC_011854	enclosed	2	21,891,133	21,896,631	+	21,891,777	21,892,731	-
GRMZM2G351330	XLOC_011863	divergent	2	22,221,051	22,222,694	+	22,220,989	22,221,632	-
GRMZM5G810061	XLOC_011876	enclosed	2	23,123,860	23,128,108	+	23,125,153	23,127,375	-
GRMZM2G062554	GRMZM2G062471	convergent	2	23,193,426	23,199,044	+	23,198,729	23,201,285	-
GRMZM2G108219	XLOC_009503	convergent	2	25,437,808	25,439,460	-	25,437,797	25,439,378	+
XLOC_011916	XLOC_009505	enclosed	2	25,458,560	25,461,491	-	25,458,589	25,459,321	+
GRMZM2G108123	XLOC_009506	divergent	2	25,497,178	25,498,828	-	25,497,200	25,498,897	+
XLOC_009507	GRMZM2G081957	enclosed	2	25,628,639	25,629,596	+	25,628,559	25,631,894	-
GRMZM2G123122	XLOC_011932	divergent	2	26,759,318	26,763,396	+	26,759,213	26,762,530	-
GRMZM2G127591	GRMZM2G428386	convergent	2	26,915,506	26,918,624	+	26,917,555	26,922,771	-
GRMZM2G138125	GRMZM2G138190	convergent	2	27,115,035	27,125,249	+	27,124,232	27,125,695	-
GRMZM2G362413	XLOC_011944	enclosed	2	27,500,744	27,502,059	+	27,501,203	27,501,645	-
GRMZM2G178693	XLOC_009548	enclosed	2	28,493,228	28,495,467	-	28,494,828	28,495,455	+
GRMZM2G441347	XLOC_011959	enclosed	2	28,745,695	28,748,913	+	28,746,597	28,748,681	-
GRMZM2G048295	XLOC_009576	enclosed	2	30,170,017	30,172,758	-	30,170,685	30,171,385	+
GRMZM2G347361	GRMZM2G171620	convergent	2	30,305,903	30,309,532	+	30,308,490	30,311,407	-
GRMZM2G082191	XLOC_011994	enclosed	2	31,214,984	31,222,672	+	31,220,291	31,221,563	-
GRMZM2G419342	XLOC_009602	enclosed	2	32,571,806	32,573,931	-	32,571,968	32,573,911	+
GRMZM2G112530	XLOC_009621	divergent	2	33,976,171	33,979,292	-	33,977,709	33,979,429	+
GRMZM2G062673	XLOC_009629	enclosed	2	34,359,171	34,360,419	-	34,359,265	34,360,412	+
GRMZM2G080176	XLOC_009631	enclosed	2	34,636,307	34,657,655	-	34,655,098	34,655,300	+
GRMZM2G102183	XLOC_012069	enclosed	2	36,945,636	36,948,162	+	36,946,248	36,947,937	-
GRMZM2G063262	GRMZM2G063163	convergent	2	37,392,043	37,396,674	+	37,396,078	37,402,459	-
GRMZM2G147884	GRMZM2G447691	convergent	2	38,760,903	38,768,270	+	38,767,291	38,770,475	-
GRMZM2G121700	XLOC_012098	divergent	2	38,821,032	38,822,692	+	38,820,651	38,822,523	-
XLOC_009676	XLOC_012103	divergent	2	39,466,301	39,468,407	+	39,466,052	39,467,802	-
XLOC_009682	GRMZM2G330430	convergent	2	40,051,916	40,054,726	+	40,054,195	40,056,907	-
XLOC_009682	XLOC_012105	enclosed	2	40,051,916	40,054,726	+	40,051,927	40,052,520	-
GRMZM2G102499	XLOC_009707	enclosed	2	41,931,997	41,935,666	-	41,934,436	41,934,852	+
GRMZM2G012328	GRMZM2G315401	convergent	2	42,992,969	42,994,079	+	42,993,870	42,996,389	-
GRMZM2G065073	XLOC_012141	enclosed	2	43,413,441	43,421,006	+	43,419,169	43,419,719	-
GRMZM2G360374	XLOC_012149	enclosed	2	44,106,201	44,109,990	+	44,108,211	44,109,509	-
GRMZM2G171254	XLOC_009745	enclosed	2	44,816,328	44,824,949	-	44,823,979	44,824,372	+
GRMZM2G171277	XLOC_012161	enclosed	2	44,829,337	44,831,648	+	44,830,075	44,831,590	-
GRMZM2G127139	XLOC_009751	enclosed	2	45,069,108	45,078,053	-	45,073,255	45,073,509	+
GRMZM2G028258	GRMZM2G028139	convergent	2	46,616,631	46,620,668	+	46,620,242	46,632,084	-
GRMZM2G053111	XLOC_012207	enclosed	2	48,775,553	48,778,515	+	48,776,032	48,776,812	-
GRMZM2G047681	XLOC_009786	enclosed	2	49,031,132	49,036,412	-	49,036,076	49,036,390	+
GRMZM2G131155	XLOC_009826	divergent	2	51,867,500	51,869,605	-	51,868,677	51,870,074	+
GRMZM2G069093	XLOC_009840	enclosed	2	54,244,859	54,246,513	-	54,245,127	54,245,771	+
GRMZM2G044132	XLOC_012256	enclosed	2	54,473,290	54,474,810	+	54,473,371	54,474,810	-
GRMZM2G048793	XLOC_009857	divergent	2	56,456,421	56,457,513	-	56,456,998	56,457,735	+
GRMZM2G134668	XLOC_009891	enclosed	2	62,121,451	62,125,108	-	62,122,873	62,123,618	+
GRMZM2G032225	XLOC_012320	enclosed	2	62,920,594	62,922,074	+	62,921,103	62,921,705	-
GRMZM2G087059	XLOC_012328	enclosed	2	64,001,685	64,004,274	+	64,002,611	64,003,234	-
GRMZM2G436533	GRMZM2G136412	convergent	2	64,294,902	64,298,527	+	64,298,381	64,301,370	-
GRMZM2G086267	XLOC_012351	enclosed	2	68,168,970	68,174,713	+	68,169,226	68,169,426	-
GRMZM2G155281	XLOC_009935	enclosed	2	69,807,585	69,810,746	-	69,807,982	69,808,578	+
XLOC_009939	XLOC_012369	divergent	2	71,189,910	71,190,894	+	71,189,907	71,190,586	-
GRMZM2G027272	XLOC_009967	enclosed	2	74,719,597	74,724,317	-	74,721,867	74,722,089	+
GRMZM2G027307	XLOC_012446	enclosed	2	86,977,308	86,991,387	+	86,977,473	86,977,751	-
GRMZM5G803404	XLOC_010062	enclosed	2	101,895,519	101,901,259	-	101,899,220	101,901,186	+
GRMZM5G803404	XLOC_010061	enclosed	2	101,895,519	101,901,259	-	101,897,618	101,897,932	+
GRMZM2G172695	XLOC_012500	enclosed	2	104,803,852	104,806,393	+	104,805,122	104,806,195	-
GRMZM2G076343	XLOC_012567	divergent	2	113,942,575	113,944,157	+	113,942,348	113,943,804	-
GRMZM2G164074	XLOC_010163	divergent	2	124,226,002	124,228,806	-	124,226,792	124,229,015	+
GRMZM2G085924	XLOC_010173	enclosed	2	127,723,784	127,725,392	-	127,724,600	127,725,352	+
GRMZM2G301355	XLOC_012641	divergent	2	128,454,614	128,458,045	+	128,454,357	128,457,728	-
GRMZM2G143397	XLOC_010183	divergent	2	130,328,542	130,329,737	-	130,329,090	130,329,738	+
GRMZM2G164064	XLOC_010187	enclosed	2	130,778,406	130,779,394	-	130,778,699	130,779,350	+
GRMZM2G111143	XLOC_012675	divergent	2	134,470,091	134,472,840	+	134,469,981	134,471,436	-
GRMZM2G133716	XLOC_012684	enclosed	2	136,740,072	136,745,887	+	136,743,919	136,745,759	-
GRMZM2G146472	XLOC_010222	enclosed	2	138,609,364	138,620,146	-	138,610,202	138,611,163	+
GRMZM2G099502	XLOC_012696	divergent	2	140,149,834	140,152,470	+	140,149,765	140,151,440	-
GRMZM2G032478	XLOC_012711	divergent	2	142,057,153	142,058,876	+	142,057,146	142,058,616	-
GRMZM2G348866	GRMZM2G348855	convergent	2	143,679,351	143,684,440	-	143,678,133	143,679,858	+
GRMZM2G028179	XLOC_012718	enclosed	2	143,893,289	143,895,477	+	143,894,058	143,895,355	-
GRMZM2G450044	XLOC_012719	enclosed	2	144,155,245	144,156,375	+	144,155,308	144,156,067	-
GRMZM2G127418	XLOC_012726	divergent	2	144,827,260	144,828,751	+	144,827,195	144,828,011	-
GRMZM2G132000	XLOC_010271	enclosed	2	145,653,249	145,656,755	-	145,656,350	145,656,679	+
GRMZM2G460138	XLOC_010306	divergent	2	151,389,889	151,390,712	-	151,390,008	151,390,734	+
GRMZM2G365035	XLOC_010349	enclosed	2	157,924,539	157,932,534	-	157,931,493	157,932,381	+
GRMZM5G876616	XLOC_012822	enclosed	2	159,084,980	159,087,515	+	159,085,004	159,085,643	-
GRMZM2G068212	XLOC_010373	enclosed	2	162,036,804	162,097,539	-	162,045,456	162,047,512	+
AC210204.3_FG002	XLOC_010390	enclosed	2	165,300,551	165,302,574	-	165,300,781	165,301,621	+
GRMZM2G045706	XLOC_010418	divergent	2	167,840,064	167,841,733	-	167,841,444	167,841,892	+
GRMZM2G092125	XLOC_012901	enclosed	2	169,186,883	169,190,374	+	169,186,924	169,187,674	-
GRMZM2G092125	XLOC_012902	enclosed	2	169,186,883	169,190,374	+	169,189,856	169,190,340	-
GRMZM2G092648	XLOC_010448	divergent	2	171,021,988	171,027,132	-	171,026,303	171,027,370	+
GRMZM2G019119	XLOC_012923	enclosed	2	172,384,572	172,387,831	+	172,384,740	172,385,013	-
XLOC_010473	GRMZM2G104546	enclosed	2	173,494,956	173,496,273	+	173,490,360	173,497,277	-
AC203957.3_FG004	XLOC_010487	enclosed	2	175,112,138	175,113,156	-	175,112,354	175,112,845	+

GRMZM2G037150	GRMZM2G037131	convergent	2	176,669,016	176,673,281	-	176,668,559	176,669,692	+
GRMZM5G868354	XLOC_012973	enclosed	2	177,200,642	177,204,531	+	177,200,717	177,201,068	-
GRMZM2G373554	XLOC_012976	enclosed	2	177,562,284	177,563,575	+	177,562,929	177,563,476	-
GRMZM2G340933	XLOC_012981	divergent	2	178,377,065	178,378,920	+	178,376,981	178,378,697	-
XLOC_010530	XLOC_012986	enclosed	2	178,670,829	178,671,877	+	178,670,884	178,671,554	-
GRMZM2G125775	XLOC_010532	enclosed	2	179,011,169	179,012,353	-	179,011,357	179,012,135	+
GRMZM2G040840	XLOC_013013	divergent	2	180,452,226	180,457,230	+	180,452,055	180,453,525	-
GRMZM2G359521	XLOC_013019	enclosed	2	181,475,801	181,477,592	+	181,476,797	181,477,422	-
GRMZM2G057852	XLOC_013031	enclosed	2	183,846,887	183,847,834	+	183,846,951	183,847,432	-
XLOC_010571	XLOC_013033	enclosed	2	183,946,317	183,947,096	+	183,946,385	183,946,791	-
GRMZM2G002131	XLOC_013076	enclosed	2	186,829,385	186,833,823	+	186,829,842	186,830,387	-
GRMZM2G381071	XLOC_013079	enclosed	2	187,110,003	187,110,972	+	187,110,216	187,110,887	-
GRMZM2G018070	XLOC_010643	enclosed	2	189,550,226	189,551,687	-	189,550,546	189,551,309	+
GRMZM2G004138	XLOC_010651	divergent	2	189,923,260	189,928,367	-	189,927,099	189,928,613	+
GRMZM2G134334	XLOC_013120	enclosed	2	190,220,678	190,223,077	+	190,221,109	190,222,380	-
GRMZM2G003947	XLOC_010664	divergent	2	190,587,060	190,589,608	-	190,588,589	190,589,744	+
GRMZM2G014341	GRMZM2G013892	divergent	2	192,312,471	192,316,197	+	192,310,632	192,313,184	-
GRMZM2G150503	XLOC_013165	enclosed	2	194,151,567	194,157,270	+	194,155,565	194,156,945	-
GRMZM5G808017	XLOC_010707	enclosed	2	194,298,662	194,299,623	-	194,299,099	194,299,374	+
GRMZM2G170253	XLOC_010713	divergent	2	194,491,687	194,493,980	-	194,492,414	194,494,026	+
GRMZM2G164956	XLOC_013172	enclosed	2	194,808,387	194,812,823	+	194,812,435	194,812,814	-
GRMZM2G063533	XLOC_010731	enclosed	2	196,480,040	196,484,796	-	196,480,477	196,480,984	+
GRMZM2G002828	XLOC_013192	enclosed	2	196,564,846	196,569,244	+	196,564,900	196,565,266	-
GRMZM2G058105	GRMZM2G050714	enclosed	2	196,700,585	196,747,523	+	196,730,795	196,734,631	-
GRMZM2G312501	XLOC_010762	divergent	2	198,178,361	198,183,070	-	198,182,722	198,183,289	+
AC233910.1_FG008	GRMZM5G887647	convergent	2	198,426,229	198,427,884	-	198,422,869	198,427,142	+
GRMZM2G081571	GRMZM2G081790	convergent	2	199,224,685	199,231,534	+	199,230,719	199,233,993	-
GRMZM2G004619	XLOC_010788	enclosed	2	199,378,450	199,380,793	-	199,380,359	199,380,622	+
GRMZM2G169694	XLOC_013269	enclosed	2	202,063,279	202,073,506	+	202,063,445	202,063,721	-
GRMZM2G060271	XLOC_010912	divergent	2	206,709,530	206,711,963	-	206,709,714	206,712,301	+
GRMZM2G062156	XLOC_013368	divergent	2	208,298,516	208,300,768	+	208,298,218	208,300,410	-
GRMZM2G431309	GRMZM2G130854	convergent	2	208,500,848	208,505,599	+	208,504,875	208,511,227	-
GRMZM2G376432	GRMZM2G077845	convergent	2	209,675,368	209,678,600	-	209,674,829	209,675,515	+
GRMZM2G079082	XLOC_010967	enclosed	2	209,789,137	209,793,396	-	209,791,906	209,792,788	+
GRMZM2G102138	XLOC_010974	enclosed	2	210,207,281	210,208,102	-	210,207,604	210,208,075	+
GRMZM2G412888	XLOC_013412	enclosed	2	211,499,169	211,505,370	+	211,499,333	211,499,610	-
GRMZM2G101769	XLOC_013421	divergent	2	211,875,412	211,877,983	+	211,875,392	211,876,119	-
GRMZM2G075002	XLOC_011039	enclosed	2	213,830,523	213,835,155	-	213,832,870	213,834,143	+
GRMZM2G123212	XLOC_011054	enclosed	2	214,245,816	214,246,895	-	214,245,985	214,246,562	+
XLOC_013465	XLOC_011057	enclosed	2	214,483,221	214,484,563	-	214,483,242	214,483,828	+
GRMZM2G060160	XLOC_011061	enclosed	2	214,616,867	214,622,270	-	214,620,687	214,620,972	+
GRMZM2G327595	XLOC_013479	enclosed	2	215,329,117	215,334,037	+	215,329,676	215,333,000	-
GRMZM2G327595	GRMZM2G327686	convergent	2	215,329,117	215,334,037	+	215,333,156	215,334,980	-
GRMZM2G459363	XLOC_013483	divergent	2	215,589,169	215,591,897	+	215,589,066	215,591,467	-
GRMZM2G166971	XLOC_013484	enclosed	2	215,615,958	215,617,442	+	215,616,519	215,616,786	-
GRMZM2G141432	XLOC_013486	enclosed	2	215,752,666	215,753,667	+	215,752,840	215,753,423	-
XLOC_013491	XLOC_011092	divergent	2	215,925,940	215,927,828	-	215,926,301	215,927,938	+
XLOC_011093	XLOC_013492	divergent	2	215,950,474	215,954,466	+	215,949,546	215,952,234	-
XLOC_011093	XLOC_013493	convergent	2	215,950,474	215,954,466	+	215,953,968	215,955,328	-
GRMZM2G001247	XLOC_013519	enclosed	2	217,334,825	217,335,843	+	217,335,074	217,335,603	-
XLOC_013523	GRMZM2G308752	divergent	2	217,520,133	217,522,001	-	217,520,351	217,522,200	+
GRMZM2G128109	XLOC_013540	enclosed	2	218,889,998	218,892,152	+	218,891,286	218,891,937	-
GRMZM2G140667	GRMZM2G140908	convergent	2	219,256,203	219,261,002	-	219,255,789	219,258,018	+
GRMZM2G068220	XLOC_013550	enclosed	2	219,606,880	219,608,943	+	219,606,971	219,607,483	-
XLOC_013559	XLOC_011164	enclosed	2	220,397,639	220,399,809	-	220,398,191	220,398,448	+
GRMZM2G137546	XLOC_013562	enclosed	2	220,671,360	220,672,972	+	220,672,012	220,672,735	-
GRMZM2G129268	XLOC_013567	enclosed	2	221,286,810	221,291,728	+	221,286,814	221,287,471	-
GRMZM5G843434	XLOC_013568	enclosed	2	221,314,181	221,316,190	+	221,314,265	221,316,032	-
GRMZM2G171818	XLOC_011184	enclosed	2	221,338,800	221,341,247	-	221,338,943	221,341,232	+
GRMZM2G106560	XLOC_013579	enclosed	2	222,422,457	222,427,553	+	222,423,023	222,423,280	-
XLOC_013593	XLOC_011235	enclosed	2	224,404,275	224,405,053	-	224,404,157	224,405,815	+
GRMZM2G169095	XLOC_013606	enclosed	2	225,001,746	225,013,108	+	225,012,370	225,012,954	-
GRMZM2G158872	XLOC_013682	enclosed	2	231,189,722	231,195,711	+	231,191,529	231,191,923	-
GRMZM2G053206	XLOC_011352	enclosed	2	232,655,940	232,657,533	-	232,655,972	232,657,479	+
GRMZM2G395128	XLOC_011372	divergent	2	233,507,197	233,508,793	-	233,507,725	233,509,411	+
GRMZM2G135013	XLOC_011373	enclosed	2	233,546,257	233,548,974	-	233,546,506	233,548,765	+
GRMZM2G082886	GRMZM2G082853	convergent	2	233,641,444	233,644,826	+	233,643,319	233,646,053	-
GRMZM5G878490	XLOC_013747	enclosed	2	234,414,167	234,415,092	+	234,413,745	234,415,092	-
GRMZM2G324544	GRMZM2G324507	enclosed	2	235,377,544	235,378,343	+	235,377,387	235,389,082	-
AC206764.4_FG003	XLOC_013797	divergent	3	654,707	655,811	-	655,360	657,996	+
GRMZM2G091047	GRMZM5G816891	convergent	3	1,349,029	1,351,202	+	1,349,760	1,354,995	-
GRMZM2G013201	GRMZM2G148723	convergent	3	1,612,503	1,613,952	-	1,609,911	1,613,862	+
GRMZM2G309152	GRMZM2G309063	convergent	3	1,615,839	1,631,263	+	1,630,778	1,633,534	-
GRMZM2G011636	GRMZM2G011553	convergent	3	1,672,575	1,677,158	+	1,676,514	1,678,951	-
GRMZM2G123540	GRMZM2G123519	convergent	3	1,721,076	1,725,886	+	1,725,071	1,729,621	-
GRMZM2G099678	XLOC_016051	enclosed	3	2,001,563	2,003,991	+	2,003,027	2,003,644	-
GRMZM2G085266	XLOC_016075	convergent	3	3,200,730	3,210,711	+	3,209,985	3,210,733	-
GRMZM2G102572	GRMZM2G400907	divergent	3	3,457,016	3,462,868	+	3,452,011	3,457,360	-
AC233853.1_FG003	XLOC_016089	enclosed	3	3,830,012	3,832,754	+	3,831,343	3,832,351	-
GRMZM2G346457	GRMZM2G045330	enclosed	3	4,344,185	4,345,443	-	4,344,330	4,345,391	+
GRMZM2G035131	XLOC_013872	enclosed	3	4,550,708	4,554,681	-	4,553,962	4,554,480	+
GRMZM2G055089	XLOC_016106	enclosed	3	4,674,607	4,680,711	+	4,678,895	4,680,599	-
GRMZM2G055313	XLOC_016107	divergent	3	4,707,293	4,710,578	+	4,707,024	4,709,135	-
GRMZM2G055313	XLOC_016108	enclosed	3	4,707,293	4,710,578	+	4,709,572	4,709,965	-
GRMZM2G355572	XLOC_013910	enclosed	3	7,938,344	7,940,976	-	7,939,013	7,940,694	+
XLOC_013911	GRMZM2G367898	enclosed	3	7,966,853	7,968,441	+	7,966,861	7,967,903	-
GRMZM2G176489	XLOC_013917	enclosed	3	8,219,031	8,221,674	-	8,220,468	8,221,022	+
XLOC_013921	GRMZM2G015788	enclosed	3	8,340,294	8,340,812	+	8,337,845	8,340,989	-
GRMZM2G158194	XLOC_016189	divergent	3	9,045,046	9,047,445	+	9,044,823	9,046,830	-
GRMZM2G355381	XLOC_016191	divergent	3	9,114,106	9,118,344	+	9,113,934	9,115,143	-
GRMZM2G083418	XLOC_013956	enclosed	3	9,808,882	9,811,705	-	9,811,021	9,811,476	+
GRMZM2G305900	XLOC_016200	enclosed	3	9,855,605	9,858,253	+	9,856,999	9,857,500	-
GRMZM2G021410	XLOC_016230	enclosed	3	13,278,182	13,280,956	+	13,278,194	13,278,623	-
GRMZM2G452564	XLOC_016255	divergent	3	15,137,688	15,139,240	+	15,136,784	15,139,075	-
GRMZM2G034534	XLOC_016291	enclosed	3	18,085,249	18,086,875	+	18,085,278	18,085,649	-
GRMZM2G320521	XLOC_016312	divergent	3	19,762,347	19,764,749	+	19,762,167	19,764,374	-
GRMZM2G467671	XLOC_014117	enclosed	3	19,973,177	19,977,322	-	19,973,505	19,974,270	+
GRMZM2G156950	XLOC_014132	convergent	3	22,005,530	22,007,136	-	22,005,326	22,006,215	+
GRMZM2G090584	XLOC_016363	enclosed	3	26,258,962	26,261,458	+	26,259,115	26,259,614	-
GRMZM2G104829	XLOC_014167	divergent	3	26,309,355	26,312,448	-	26,310,637	26,312,874	+
GRMZM2G472248	XLOC_014230	enclosed	3	36,311,520	36,313,337	-	36,311,860	36,313,187	+
GRMZM2G132913	XLOC_014236	enclosed	3	37,359,987	37,361,918	-	37,360,058	37,361,385	+
GRMZM2G368388	XLOC_014244	divergent	3	38,438,067	38,440,389	-	38,438,506	38,440,607	+
GRMZM2G132465	XLOC_016483	enclosed	3	39,477,546	39,482,124	+	39,477,668	39,478,338	-
GRMZM2G089982	XLOC_014260	divergent	3	40,529,064	40,531,245	-	40,530,819	40,532,893	+
GRMZM2G144648	XLOC_014266	divergent	3	41,742,447	41,744,589	-	41,742,767	41,744,902	+
GRMZM2G003883	XLOC_014275	enclosed	3	44,224,052	44,229,674	-	44,229,170	44,229,601	+
GRMZM2G017520	XLOC_014298	divergent	3	48,494,950	48,497,362	-	48,496,982	48,497,546	+
GRMZM2G115960	XLOC_014300	divergent	3	48,806,998	48,810,388	-	48,809,033	48,810,511	+
GRMZM2G363908	GRMZM2G363893	convergent	3	52,448,552	52,452,420	+	52,452,031	52,454,284	-
GRMZM2G381473	GRMZM2G080320	convergent	3	57,097,944	57,101,742	-	57,091,548	57,098,312	+
GRMZM2G381473	XLOC_014367	enclosed	3	57,097,944	57,101,742	-	57,099,106	57,101,623	+
GRMZM2G107228	XLOC_016598	enclosed	3	59,327,568	59,329,698	+	59,328,610	59,328,924	-
GRMZM2G174975	XLOC_016608	divergent	3	60,645,763	60,648,379	+	60,645,606	60,646,302	-
GRMZM2G095039	XLOC_014417	enclosed	3	66,707,678	66,709,478	-	66,708,373	66,708,927	+
GRMZM2G044724	XLOC_016701	enclosed	3	85,672,795	85,676,822	+	85,674,690	85,674,920	-
GRMZM2G391794	XLOC_016738	enclosed	3	91,614,897	91,618,962	+	91,617,008	91,618,186	-
GRMZM2G391794	XLOC_016737	enclosed	3	91,614,897	91,618,962	+	91,615,051	91,616,155	-
GRMZM2G133793	XLOC_014507	divergent	3	93,102,434	93,104,203	-	93,102,813	93,104,274	+
GRMZM2G017835	XLOC_016757	enclosed	3	95,281,733	95,287,244	+	95,281,770	95,282,337	-
XLOC_016818	XLOC_014550	divergent	3	109,716,966	109,717,972	-	109,717,705	109,718,299	+
GRMZM2G022629	XLOC_014595	divergent	3	117,275,976	117,278,216	-	117,277,486	117,278,440	+
XLOC_014607	AC203865.3_FG001	enclosed	3	119,994,992	119,997,548	+	119,994,715	119,998,479	-
GRMZM2G351387	XLOC_016921	enclosed	3	126,175,759	126,191,667	+	126,190,434	126,191,618	-
GRMZM2G060680	XLOC_016934	enclosed	3	127,119,556	127,121,213	+	127,120,122	127,120,988	-
XLOC_016942	XLOC_014644	divergent	3	127,874,065	127,877,164	-	127,876,049	127,877,357	+
GRMZM2G160902	XLOC_014656	divergent	3	128,957,613	128,959,445	-	128,958,274	128,959,485	+
GRMZM2G046061	GRMZM2G046088	convergent	3	129,109,128	129,111,686	+	129,110,506	129,113,876	-
GRMZM2G155998	XLOC_016987	convergent	3	133,809,662	133,812,883	+	133,811,695	133,813,619	-
GRMZM2G155974	XLOC_016987	divergent	3	133,812,942	133,826,472	+	133,811,695	133,813,619	-
GRMZM2G323936	XLOC_016989	convergent	3	134,042,812	134,045,336	+	134,044,263	134,045,881	-
XLOC_016990	GRMZM2G323936	convergent	3	134,044,263	134,051,169	-	134,042,812	134,045,336	+
AC209377.3_FG003	XLOC_014721	divergent	3	137,557,525	137,558,581	-	137,557,685	137,558,726	+
GRMZM2G138003	XLOC_017014	enclosed	3	138,121,498	138,123,238	+	138,121,201	138,123,238	-
GRMZM2G152889	GRMZM2G152952	convergent	3	142,724,521	142,734,864	-	142,722,457	142,726,219	+
GRMZM2G122431	XLOC_017074	enclosed	3	145,209,774	145,213,115	+	145,209,893	145,210,556	-
GRMZM2G309512	GRMZM2G309479	enclosed	3	146,284,010	146,287,961	+	146,286,679	146,287,500	-
GRMZM2G103342	XLOC_014816	divergent	3	146,556,764	146,558,820	-	146,557,193	146,558,978	+
GRMZM2G102056	XLOC_017124	divergent	3	147,559,407	147,561,340	+	147,559,395	147,561,086	-
GRMZM2G428168	GRMZM2G127789	convergent	3	149,351,183	149,353,705	-	149,351,145	149,352,030	+
XLOC_014841	XLOC_017149	enclosed	3	149,437,918	149,438,345	+	149,437,930	149,438,304	-
GRMZM2G369652	GRMZM2G068665	convergent	3	150,308,311	150,311,184	-	150,304,861	150,310,060	+
GRMZM2G050997	XLOC_014868	enclosed	3	152,240,411	152,243,105	-	152,240,443	152,241,766	+
GRMZM2G145840	XLOC_017183	convergent	3	153,898,814	153,900,122	+	153,899,269	153,900,406	-
GRMZM2G428987	XLOC_017193	enclosed	3	154,652,380	154,669,702	+	154,666,729	154,667,335	-
GRMZM2G425004	XLOC_017197	enclosed	3	154,780,689	154,782,525	+	154,781,735	154,782,216	-
XLOC_017207	XLOC_014904	divergent	3	155,839,330	155,842,013	-	155,840,176	155,842,742	+
GRMZM2G448834	XLOC_017223	enclosed	3	157,126,999	157,130,351	+	157,128,739	157,130,173	-
GRMZM2G129114	XLOC_014944	divergent	3	160,370,641	160,372,491	-	160,370,919	160,372,685	+
GRMZM2G146697	XLOC_017267	enclosed	3	161,889,750	161,898,826	+	161,889,843	161,890,571	-
GRMZM2G139822	XLOC_017296	enclosed	3	164,927,183	164,941,378	+	164,927,342	164,927,696	-
GRMZM2G177227	XLOC_015002	divergent	3	165,900,055	165,902,629	-	165,900,808	165,902,750	+
GRMZM2G035092	XLOC_017336	divergent	3	168,968,535	168,969,630	+	168,968,523	168,969,254	-
GRMZM2G110277	GRMZM2G110398	convergent	3	171,482,926	171,486,892	+	171,485,569	171,487,165	-
GRMZM2G138176	GRMZM2G138161	convergent	3	171,752,389	171,756,517	+	171,756,041	171,760,110	-
GRMZM2G135743	XLOC_015094	enclosed	3	172,092,917	172,094,704	-	172,093,718	172,094,315	+
GRMZM2G078500	XLOC_017375	enclosed	3	172,376,539	172,379,695	+	172,376,634	172,377,105	-
GRMZM2G431006	XLOC_015099	divergent	3	172,487,141	172,489,677	-	172,489,396	172,490,339	+
GRMZM2G049373	XLOC_015103	enclosed	3	173,048,156	173,050,637	-	173,048,324	173,049,084	+
XLOC_017404	XLOC_015116	enclosed	3	174,739,115	174,743,856	-	174,742,240	174,743,099	+
GRMZM5G858197	GRMZM5G893764	convergent	3	175,673,165	175,677,076	+	175,675,750	175,680,444	-
AC155610.2_FG007	XLOC_017411	enclosed	3	175,729,103	175,732,391	+	175,731,179	175,732,139	-
GRMZM2G061321	XLOC_017421	enclosed	3	176,088,170	176,090,219	+	176,088,318	176,089,631	-
GRMZM2G074248	XLOC_017423	enclosed	3	176,346,351	176,349,021	+	176,347,957	176,348,895	-
GRMZM2G074248	XLOC_017422	enclosed	3	176,346,351	176,349,021	+	176,346,802	176,347,318	-
GRMZM2G127117	XLOC_015163	enclosed	3	176,607,781	176,610,780	-	176,607,974	176,609,165	+
GRMZM2G003682	XLOC_017449	enclosed	3	178,149,296	178,152,013	+	178,150,170	178,151,372	-
GRMZM2G177518	XLOC_015190	divergent	3	178,185,823	178,188,427	-	178,186,779	178,188,651	+
XLOC_015198	XLOC_017459	enclosed	3	178,920,343	178,923,945	+	178,923,328	178,923,663	-
GRMZM2G065557	XLOC_017468	convergent	3	179,467,655	179,468,810	+	179,468,333	179,472,231	-
XLOC_017482	XLOC_015216	divergent	3	180,092,014	180,092,401	-	180,092,030	180,092,931	+
GRMZM2G054807	XLOC_017485	divergent	3	180,252,370	180,255,087	+	180,252,317	180,254,703	-
GRMZM2G126077	XLOC_015243	enclosed	3	181,805,519	181,807,107	-	181,805,644	181,805,986	+
AC214448.3_FG007	XLOC_017510	enclosed	3	181,844,506	181,847,013	+	181,844,862	181,845,535	-
GRMZM2G426953	GRMZM2G127034	convergent	3	182,096,591	182,101,272	-	182,091,206	182,097,283	+
GRMZM2G132169	XLOC_015269	enclosed	3	183,701,067	183,703,595	-	183,702,635	183,702,843	+
GRMZM2G350410	GRMZM2G350399	enclosed	3	185,806,850	185,811,071	+	185,808,548	185,810,097	-
GRMZM2G010447	XLOC_017571	divergent	3	186,273,513	186,275,044	+	186,273,476	186,274,094	-
GRMZM2G138074	XLOC_017607	enclosed	3	187,769,141	187,771,874	+	187,770,273	187,771,591	-
GRMZM2G702059	XLOC_017614	enclosed	3	188,039,626	188,043,090	+	188,042,731	188,043,031	-
GRMZM2G047274	XLOC_015342	enclosed	3	188,387,390	188,390,373	-	188,387,394	188,388,307	+
GRMZM2G310368	XLOC_015345	enclosed	3	188,728,061	188,729,514	-	188,728,781	188,729,273	+
AC233851.1_FG017	XLOC_017636	enclosed	3	189,593,197	189,595,375	+	189,594,096	189,594,783	-
GRMZM2G173647	XLOC_017639	enclosed	3	189,832,965	189,837,084	+	189,832,965	189,833,531	-
GRMZM2G322723	XLOC_017645	divergent	3	190,499,330	190,507,302	+	190,499,018	190,501,286	-
GRMZM2G322728	XLOC_015373	divergent	3	190,513,119	190,518,692	-	190,516,912	190,519,079	+
GRMZM2G054905	XLOC_015379	convergent	3	191,183,824	191,244,226	-	191,183,128	191,184,336	+
GRMZM2G446220	XLOC_015438	enclosed	3	195,671,907	195,673,492	-	195,671,890	195,674,277	+
XLOC_017755	XLOC_015471	enclosed	3	197,959,705	197,974,998	-	197,962,850	197,963,438	+
XLOC_017756	XLOC_015471	divergent	3	197,962,668	197,963,235	-	197,962,850	197,963,438	+
GRMZM2G141299	XLOC_015479	enclosed	3	198,284,004	198,286,691	-	198,284,180	198,286,643	+
GRMZM2G055898	XLOC_015481	divergent	3	198,454,743	198,459,545	-	198,458,900	198,460,981	+
GRMZM2G358830	XLOC_015482	enclosed	3	198,462,555	198,466,556	-	198,462,757	198,463,330	+
GRMZM2G176699	GRMZM2G474326	convergent	3	198,668,535	198,673,264	-	198,667,643	198,668,930	+
GRMZM2G177929	XLOC_015499	enclosed	3	199,322,717	199,326,605	-	199,323,630	199,324,147	+
GRMZM2G057067	XLOC_015503	enclosed	3	199,377,243	199,380,242	-	199,378,034	199,380,119	+
GRMZM5G836674	GRMZM2G051151	convergent	3	199,723,335	199,732,187	-	199,722,345	199,723,907	+
GRMZM2G381025	XLOC_015518	enclosed	3	200,263,486	200,265,308	-	200,263,737	200,265,175	+
XLOC_017795	XLOC_015519	enclosed	3	200,290,750	200,292,783	-	200,290,750	200,293,283	+
XLOC_017798	XLOC_015521	enclosed	3	200,668,046	200,668,710	-	200,668,046	200,668,721	+
GRMZM2G040298	XLOC_017804	enclosed	3	201,085,122	201,088,035	+	201,085,643	201,087,293	-
GRMZM2G025860	XLOC_017816	enclosed	3	201,694,605	201,701,003	+	201,696,573	201,700,349	-
GRMZM2G159724	XLOC_015538	enclosed	3	201,756,915	201,761,523	-	201,757,013	201,758,683	+
GRMZM2G055276	XLOC_017833	enclosed	3	202,504,869	202,507,948	+	202,505,176	202,506,715	-
GRMZM2G111611	XLOC_015587	enclosed	3	204,542,143	204,548,156	-	204,542,398	204,542,665	+
GRMZM2G165099	XLOC_015603	enclosed	3	205,448,996	205,451,333	-	205,449,433	205,449,797	+
GRMZM2G157317	XLOC_017881	enclosed	3	205,695,411	205,702,868	+	205,701,185	205,702,703	-
GRMZM2G051683	XLOC_015609	enclosed	3	205,769,467	205,770,820	-	205,769,378	205,770,850	+
AC233882.1_FG003	XLOC_015611	enclosed	3	205,915,252	205,922,597	-	205,915,987	205,917,102	+
GRMZM2G146913	XLOC_017899	enclosed	3	206,848,092	206,849,182	+	206,848,752	206,849,130	-
GRMZM2G056388	XLOC_017900	enclosed	3	206,923,316	206,924,388	+	206,923,316	206,924,346	-
GRMZM2G447632	XLOC_017903	enclosed	3	206,949,878	206,950,721	+	206,950,153	206,950,714	-
GRMZM2G028568	GRMZM2G028467	convergent	3	207,028,261	207,037,329	+	207,036,751	207,039,713	-
GRMZM2G452807	XLOC_017927	divergent	3	208,488,885	208,490,355	+	208,488,417	208,489,596	-
GRMZM2G423202	XLOC_015662	enclosed	3	209,050,298	209,053,592	-	209,050,410	209,053,433	+
GRMZM2G022931	XLOC_017959	enclosed	3	210,588,285	210,589,844	+	210,588,766	210,589,321	-
GRMZM2G023436	XLOC_015686	enclosed	3	210,617,138	210,619,025	-	210,617,343	210,618,206	+
GRMZM2G122362	XLOC_017967	enclosed	3	211,203,728	211,209,219	+	211,203,731	211,204,235	-
GRMZM2G125044	XLOC_017970	enclosed	3	211,402,697	211,410,523	+	211,402,750	211,403,115	-
GRMZM2G344848	XLOC_017976	enclosed	3	211,845,106	211,846,918	+	211,845,283	211,846,698	-
GRMZM5G814314	XLOC_017987	enclosed	3	212,517,687	212,521,608	+	212,517,867	212,521,224	-
GRMZM2G330302	XLOC_015723	enclosed	3	213,469,179	213,475,662	-	213,472,943	213,473,385	+

GRMZM2G022311	XLOC_015728	divergent	3	213,639,419	213,641,365	-	213,639,551	213,641,474	+
GRMZM2G105140	XLOC_018046	enclosed	3	217,542,866	217,545,519	+	217,543,819	217,544,737	-
GRMZM2G079089	XLOC_015795	enclosed	3	219,419,497	219,420,765	-	219,420,009	219,420,721	+
GRMZM2G116584	XLOC_015815	enclosed	3	220,604,720	220,608,431	-	220,604,720	220,608,764	+
GRMZM2G087675	GRMZM2G087628	divergent	3	221,653,089	221,654,953	+	221,649,424	221,654,718	-
GRMZM2G019807	XLOC_018120	divergent	3	221,718,648	221,720,095	+	221,718,415	221,719,225	-
GRMZM2G071021	XLOC_018125	enclosed	3	221,771,306	221,775,473	+	221,774,725	221,775,130	-
GRMZM2G058021	XLOC_015881	divergent	3	223,930,441	223,931,772	-	223,930,610	223,931,905	+
XLOC_015910	XLOC_018198	divergent	3	226,500,341	226,500,972	+	226,500,327	226,500,904	-
GRMZM2G071154	GRMZM2G071113	convergent	3	226,856,807	226,859,571	-	226,851,477	226,859,535	+
GRMZM2G368491	XLOC_015929	enclosed	3	227,565,909	227,567,701	-	227,566,067	227,567,279	+
GRMZM2G344924	XLOC_015957	enclosed	3	229,070,754	229,076,299	-	229,070,973	229,071,445	+
GRMZM2G001457	XLOC_018225	divergent	3	229,218,369	229,241,730	+	229,218,227	229,218,934	-
GRMZM2G467123	XLOC_018233	divergent	3	229,640,542	229,644,275	+	229,640,519	229,641,139	-
GRMZM2G005749	GRMZM2G005834	enclosed	3	230,083,969	230,085,238	+	230,083,958	230,086,240	-
GRMZM2G054703	XLOC_018251	enclosed	3	230,522,661	230,526,086	+	230,523,385	230,523,682	-
GRMZM2G177912	GRMZM2G177895	convergent	3	230,593,917	230,599,898	+	230,599,509	230,600,829	-
GRMZM2G152781	XLOC_018266	enclosed	3	231,024,532	231,025,858	+	231,024,891	231,025,397	-
GRMZM2G092008	GRMZM2G092021	enclosed	3	231,198,433	231,200,286	-	231,196,835	231,205,771	+
GRMZM2G313320	XLOC_016006	divergent	3	231,562,253	231,563,381	-	231,562,330	231,563,531	+
GRMZM2G397661	GRMZM2G000510	convergent	4	32,363	33,792	+	32,440	63,818	-
GRMZM2G018254	XLOC_018284	divergent	4	512,986	515,273	-	513,590	515,381	+
GRMZM2G073823	XLOC_018292	divergent	4	829,560	832,103	-	831,188	832,279	+
GRMZM2G073805	XLOC_018293	enclosed	4	832,506	835,056	-	832,823	835,056	+
GRMZM2G164224	XLOC_018304	enclosed	4	1,070,157	1,075,181	-	1,070,183	1,074,395	+
XLOC_020442	XLOC_018305	convergent	4	1,078,823	1,079,883	-	1,078,335	1,079,772	+
GRMZM2G171036	XLOC_020472	divergent	4	2,386,082	2,387,319	+	2,385,958	2,386,696	-
GRMZM2G448213	XLOC_018321	enclosed	4	2,431,594	2,433,746	-	2,432,796	2,433,635	+
XLOC_020483	XLOC_018327	enclosed	4	2,611,736	2,612,297	-	2,611,736	2,612,238	+
GRMZM2G126453	XLOC_020489	enclosed	4	2,755,561	2,766,689	+	2,765,969	2,766,352	-
GRMZM2G070429	XLOC_018349	divergent	4	3,483,206	3,488,614	-	3,487,958	3,489,663	+
GRMZM2G165325	XLOC_020510	enclosed	4	4,790,674	4,792,253	+	4,791,414	4,792,218	-
GRMZM5G877647	XLOC_018393	enclosed	4	5,006,400	5,007,839	-	5,006,764	5,007,111	+
AC212755.4_FG001	XLOC_018421	enclosed	4	6,247,911	6,249,250	-	6,248,121	6,248,792	+
GRMZM2G027021	XLOC_020556	divergent	4	9,490,215	9,499,465	+	9,490,202	9,491,088	-
GRMZM2G117971	XLOC_018447	enclosed	4	9,685,582	9,686,560	-	9,685,645	9,685,961	+
GRMZM2G113470	XLOC_018453	enclosed	4	9,896,505	9,902,617	-	9,902,135	9,902,425	+
GRMZM2G581155	XLOC_020600	enclosed	4	14,054,454	14,059,644	+	14,054,715	14,055,963	-
GRMZM2G091481	XLOC_018498	enclosed	4	14,877,039	14,881,740	-	14,877,134	14,877,930	+
GRMZM2G025109	XLOC_020611	divergent	4	15,037,603	15,039,172	+	15,037,278	15,038,658	-
XLOC_018508	XLOC_020616	enclosed	4	15,842,899	15,846,425	+	15,843,477	15,844,369	-
GRMZM2G080168	XLOC_020630	enclosed	4	17,257,026	17,259,706	+	17,257,105	17,258,600	-
GRMZM2G139894	XLOC_018536	enclosed	4	18,610,025	18,616,532	-	18,610,785	18,611,676	+
GRMZM2G574782	XLOC_020646	divergent	4	18,698,719	18,705,550	+	18,698,629	18,703,364	-
GRMZM2G177668	XLOC_018538	enclosed	4	19,133,772	19,136,181	-	19,134,262	19,135,158	+
GRMZM2G089995	XLOC_020702	convergent	4	26,450,748	26,452,606	+	26,452,045	26,454,670	-
GRMZM2G089995	XLOC_020701	convergent	4	26,450,748	26,452,606	+	26,452,045	26,454,285	-
GRMZM2G146862	XLOC_020709	enclosed	4	26,847,982	26,889,010	+	26,852,157	26,852,616	-
GRMZM2G382323	XLOC_018633	enclosed	4	29,179,242	29,181,600	-	29,179,242	29,181,499	+
GRMZM2G122277	XLOC_020746	enclosed	4	31,271,410	31,278,209	+	31,276,128	31,277,846	-
AC196475.3_FG004	XLOC_018658	convergent	4	32,278,497	32,280,957	-	32,278,467	32,280,852	+
GRMZM2G074280	GRMZM2G074087	enclosed	4	36,017,140	36,020,195	+	36,019,078	36,019,888	-
GRMZM2G046743	XLOC_020787	enclosed	4	36,911,334	36,914,601	+	36,912,206	36,913,001	-
GRMZM2G046804	GRMZM2G046743	convergent	4	36,913,994	36,917,730	-	36,911,334	36,914,601	+
GRMZM2G363540	XLOC_018714	convergent	4	37,262,877	37,263,974	-	37,262,864	37,263,878	+
GRMZM2G324643	XLOC_020801	enclosed	4	37,603,564	37,604,830	+	37,603,712	37,604,136	-
GRMZM2G105438	GRMZM2G105387	convergent	4	38,788,778	38,791,422	+	38,791,036	38,792,802	-
GRMZM2G135568	GRMZM2G135547	convergent	4	39,633,113	39,637,478	-	39,629,147	39,633,705	+
GRMZM2G056573	XLOC_020835	enclosed	4	40,005,741	40,012,885	+	40,012,198	40,012,616	-
GRMZM2G075496	XLOC_018755	enclosed	4	42,127,982	42,131,478	-	42,128,033	42,129,663	+
GRMZM2G006953	XLOC_018775	enclosed	4	44,885,720	44,888,367	-	44,887,934	44,888,291	+
GRMZM2G100583	XLOC_020878	enclosed	4	46,961,515	46,966,149	+	46,961,562	46,962,159	-
GRMZM2G107289	XLOC_018785	enclosed	4	47,746,128	47,751,481	-	47,750,743	47,751,284	+
GRMZM2G121928	XLOC_018846	enclosed	4	60,384,110	60,385,046	-	60,384,206	60,385,041	+
GRMZM2G011806	XLOC_018851	enclosed	4	61,742,444	61,748,096	-	61,742,780	61,743,475	+
GRMZM5G884985	XLOC_020967	divergent	4	63,028,898	63,030,916	+	63,028,730	63,030,827	-
GRMZM2G119071	XLOC_018861	enclosed	4	63,118,826	63,119,812	-	63,119,243	63,119,725	+
GRMZM2G082087	XLOC_018862	enclosed	4	63,562,329	63,566,123	-	63,562,639	63,563,315	+
GRMZM2G352132	XLOC_020984	enclosed	4	67,045,579	67,052,872	+	67,051,659	67,052,660	-
GRMZM2G359986	XLOC_018894	enclosed	4	67,675,287	67,679,206	-	67,677,536	67,679,076	+
GRMZM2G174732	XLOC_021018	divergent	4	71,935,082	71,945,964	+	71,934,793	71,936,351	-
GRMZM2G091478	XLOC_021034	divergent	4	74,338,529	74,349,897	+	74,338,305	74,339,402	-
GRMZM2G066599	XLOC_021046	enclosed	4	76,410,462	76,412,170	+	76,410,602	76,410,931	-
XLOC_018952	XLOC_021048	enclosed	4	77,234,530	77,237,022	+	77,234,574	77,236,038	-
GRMZM2G091155	XLOC_018979	enclosed	4	81,729,034	81,732,613	-	81,729,329	81,729,643	+
GRMZM2G089698	XLOC_018991	enclosed	4	86,308,802	86,310,939	-	86,309,261	86,310,675	+
XLOC_021125	XLOC_019017	divergent	4	92,859,072	92,860,700	-	92,860,154	92,860,755	+
GRMZM2G147279	GRMZM2G147288	convergent	4	116,891,237	116,893,515	-	116,886,271	116,892,167	+
GRMZM5G898290	XLOC_021244	enclosed	4	125,797,420	125,799,414	+	125,798,578	125,799,254	-
GRMZM2G133568	GRMZM2G133563	enclosed	4	126,033,031	126,036,615	-	126,025,307	126,037,302	+
GRMZM2G407996	XLOC_021276	enclosed	4	131,204,417	131,207,606	+	131,205,067	131,205,607	-
GRMZM2G107896	XLOC_019221	enclosed	4	135,677,987	135,707,206	-	135,678,353	135,678,697	+
XLOC_021322	GRMZM2G159700	divergent	4	141,306,653	141,307,645	-	141,306,798	141,312,900	+
GRMZM2G127857	XLOC_019246	enclosed	4	142,662,836	142,664,368	-	142,663,441	142,663,645	+
GRMZM2G081582	XLOC_019252	enclosed	4	143,491,590	143,497,192	-	143,491,887	143,494,313	+
GRMZM2G081192	XLOC_021338	enclosed	4	143,891,282	143,894,290	+	143,891,905	143,892,153	-
GRMZM2G129569	XLOC_019297	enclosed	4	149,310,880	149,317,247	-	149,311,160	149,312,065	+
GRMZM2G145226	GRMZM2G145221	convergent	4	151,675,886	151,682,478	+	151,682,337	151,684,641	-
GRMZM2G145221	XLOC_019309	divergent	4	151,682,337	151,684,641	-	151,683,084	151,684,683	+
GRMZM2G026922	GRMZM2G323719	convergent	4	152,425,543	152,433,694	-	152,424,058	152,425,840	+
GRMZM2G102502	XLOC_019324	enclosed	4	152,813,342	152,818,202	-	152,817,608	152,817,855	+
GRMZM2G392975	XLOC_021399	enclosed	4	153,654,778	153,659,027	+	153,654,840	153,658,135	-
GRMZM2G392975	GRMZM2G093316	convergent	4	153,654,778	153,659,027	+	153,658,281	153,668,278	-
GRMZM2G114998	XLOC_019354	divergent	4	155,035,662	155,038,571	-	155,037,503	155,038,790	+
GRMZM2G028438	XLOC_019360	divergent	4	156,049,488	156,052,142	-	156,050,218	156,052,167	+
GRMZM2G134023	XLOC_019362	divergent	4	156,217,617	156,219,742	-	156,218,446	156,219,862	+
XLOC_021429	GRMZM2G528010	convergent	4	156,443,715	156,445,719	-	156,439,025	156,444,649	+
GRMZM2G081037	GRMZM2G081172	convergent	4	156,981,541	156,986,962	+	156,986,015	156,987,610	-
GRMZM2G054354	GRMZM2G054013	convergent	4	158,166,367	158,170,289	+	158,169,952	158,174,569	-
GRMZM2G054354	XLOC_021449	enclosed	4	158,166,367	158,170,289	+	158,166,397	158,167,576	-
GRMZM2G350157	XLOC_019396	divergent	4	158,326,931	158,329,099	-	158,328,012	158,329,138	+
GRMZM2G066615	XLOC_021453	enclosed	4	158,364,982	158,372,319	+	158,370,468	158,372,295	-
GRMZM2G121075	XLOC_019399	enclosed	4	158,585,836	158,587,198	-	158,586,700	158,587,145	+
GRMZM2G119258	XLOC_019416	convergent	4	160,367,219	160,374,647	-	160,367,021	160,368,068	+
GRMZM2G342515	XLOC_021488	divergent	4	160,982,522	160,983,293	+	160,982,519	160,982,968	-
GRMZM2G022514	XLOC_019440	enclosed	4	161,579,119	161,583,314	-	161,579,943	161,581,091	+
GRMZM2G309950	GRMZM2G011085	divergent	4	163,674,452	163,675,848	+	163,672,570	163,675,007	-
GRMZM2G021339	XLOC_021540	divergent	4	165,861,729	165,864,137	+	165,861,367	165,863,784	-
GRMZM2G471733	XLOC_021547	enclosed	4	166,895,031	166,897,669	+	166,895,138	166,896,844	-
GRMZM2G014854	XLOC_021554	enclosed	4	167,105,924	167,108,481	+	167,106,377	167,106,872	-
GRMZM2G036464	XLOC_019498	convergent	4	167,111,209	167,114,536	-	167,111,036	167,113,698	+
GRMZM2G017852	GRMZM2G017365	convergent	4	168,574,502	168,577,580	+	168,576,413	168,580,294	-
GRMZM2G082007	GRMZM2G082222	convergent	4	169,989,941	169,993,608	-	169,987,963	169,990,290	+
GRMZM2G081843	XLOC_019521	enclosed	4	170,004,620	170,006,968	-	170,004,832	170,006,074	+
GRMZM2G146207	GRMZM2G146228	convergent	4	170,880,755	170,885,518	+	170,884,819	170,886,073	-
GRMZM2G466545	GRMZM2G164854	convergent	4	171,946,372	171,951,467	+	171,951,028	171,955,087	-
GRMZM2G178398	XLOC_021629	enclosed	4	172,777,612	172,783,745	+	172,782,890	172,783,635	-
GRMZM2G054210	XLOC_019579	divergent	4	173,239,888	173,253,971	-	173,253,159	173,254,280	+
GRMZM2G316787	GRMZM2G316778	convergent	4	174,072,810	174,073,935	+	174,072,841	174,074,369	-
GRMZM2G312521	XLOC_019600	enclosed	4	174,991,068	174,996,208	-	174,992,060	174,994,904	+
GRMZM2G314946	XLOC_021670	enclosed	4	175,361,055	175,361,851	+	175,361,439	175,361,691	-
GRMZM2G181359	GRMZM2G181362	convergent	4	175,765,088	175,769,387	+	175,767,391	175,780,753	-
GRMZM2G170313	GRMZM2G473016	convergent	4	178,486,128	178,499,296	+	178,498,368	178,500,694	-
GRMZM2G015983	XLOC_021715	divergent	4	178,702,590	178,706,283	+	178,702,308	178,703,524	-
GRMZM2G041699	XLOC_019669	divergent	4	180,401,112	180,403,035	-	180,401,155	180,403,087	+
GRMZM2G470524	GRMZM2G169617	convergent	4	180,463,493	180,465,143	+	180,464,258	180,475,317	-
GRMZM2G322950	XLOC_019677	divergent	4	180,679,873	180,681,240	-	180,679,954	180,681,492	+
GRMZM2G151107	XLOC_021744	divergent	4	180,924,991	180,926,213	+	180,924,869	180,925,861	-
GRMZM2G169033	XLOC_021752	enclosed	4	181,560,393	181,563,791	+	181,561,726	181,562,111	-
GRMZM2G041809	XLOC_021758	divergent	4	181,903,993	181,906,445	+	181,903,925	181,905,990	-
GRMZM2G041959	XLOC_021759	enclosed	4	181,917,370	181,923,296	+	181,921,872	181,922,751	-
GRMZM2G020996	GRMZM2G318033	convergent	4	183,213,856	183,225,003	-	183,213,169	183,220,384	+
GRMZM2G114052	XLOC_021790	enclosed	4	184,148,616	184,152,991	+	184,149,627	184,150,231	-
GRMZM2G113967	XLOC_019721	enclosed	4	184,154,926	184,157,682	-	184,155,293	184,156,738	+
GRMZM2G060732	XLOC_021804	enclosed	4	185,025,956	185,029,049	+	185,026,024	185,028,786	-
GRMZM2G060470	XLOC_021805	divergent	4	185,030,955	185,035,131	+	185,029,082	185,031,304	-
GRMZM2G151516	XLOC_019743	enclosed	4	185,209,888	185,211,510	-	185,210,476	185,210,873	+
GRMZM2G380784	XLOC_019750	divergent	4	185,642,046	185,643,948	-	185,642,093	185,644,023	+
GRMZM2G179733	XLOC_019752	divergent	4	185,702,075	185,703,839	-	185,702,432	185,704,873	+
GRMZM2G146553	GRMZM2G146720	enclosed	4	186,214,078	186,220,919	-	186,214,119	186,215,211	+
GRMZM2G171605	XLOC_021845	enclosed	4	186,764,494	186,766,965	+	186,764,601	186,764,876	-
GRMZM2G095141	GRMZM2G095326	convergent	4	186,832,765	186,835,999	+	186,835,570	186,841,247	-
GRMZM2G051787	XLOC_019790	divergent	4	188,475,242	188,477,024	-	188,475,513	188,478,292	+
GRMZM2G030877	XLOC_019795	enclosed	4	188,665,523	188,672,996	-	188,667,884	188,669,203	+
GRMZM2G030877	GRMZM2G031094	convergent	4	188,665,523	188,672,996	-	188,663,683	188,665,638	+
GRMZM2G451792	XLOC_021953	enclosed	4	195,018,290	195,032,289	+	195,030,508	195,032,201	-
GRMZM2G149843	GRMZM2G451366	convergent	4	195,797,433	195,801,279	+	195,800,111	195,805,721	-
GRMZM2G163671	XLOC_019888	enclosed	4	195,975,252	195,979,088	-	195,975,699	195,976,927	+
GRMZM2G440925	XLOC_021978	divergent	4	197,104,904	197,106,971	+	197,104,888	197,106,605	-
GRMZM2G332522	XLOC_019910	enclosed	4	198,256,542	198,257,719	-	198,256,600	198,257,361	+
GRMZM2G332809	GRMZM2G033228	enclosed	4	198,946,845	198,953,299	+	198,951,228	198,952,967	-
GRMZM2G046111	XLOC_022029	enclosed	4	201,141,912	201,143,147	+	201,141,953	201,142,723	-
GRMZM2G079491	XLOC_022031	divergent	4	201,299,690	201,302,345	+	201,299,625	201,301,921	-
AC216067.3_FG002	XLOC_019986	enclosed	4	204,522,509	204,525,059	-	204,522,703	204,522,947	+
GRMZM2G059706	XLOC_019997	divergent	4	206,672,898	206,674,203	-	206,673,299	206,674,219	+
XLOC_022084	XLOC_020010	enclosed	4	207,618,649	207,619,251	-	207,618,649	207,619,296	+
GRMZM2G089856	XLOC_020014	enclosed	4	209,050,229	209,052,176	-	209,051,277	209,051,536	+
GRMZM2G071478	XLOC_022113	enclosed	4	211,330,470	211,333,066	+	211,331,241	211,332,851	-
GRMZM2G014071	XLOC_022119	enclosed	4	212,126,276	212,130,478	+	212,126,428	212,127,100	-
GRMZM2G458728	XLOC_020036	divergent	4	212,853,674	212,859,386	-	212,857,831	212,859,418	+
GRMZM2G079365	XLOC_022130	enclosed	4	213,863,768	213,880,142	+	213,864,442	213,865,170	-
GRMZM2G590333	XLOC_020056	enclosed	4	215,441,250	215,445,850	-	215,442,637	215,445,193	+
XLOC_020073	XLOC_022156	enclosed	4	217,184,907	217,189,560	+	217,185,342	217,185,795	-
XLOC_020133	XLOC_022215	divergent	4	225,418,358	225,420,404	+	225,418,052	225,419,587	-
GRMZM2G052920	XLOC_022221	divergent	4	226,108,085	226,108,881	+	226,107,900	226,108,706	-
GRMZM2G035077	XLOC_020146	enclosed	4	226,605,254	226,607,090	-	226,606,273	226,606,895	+
GRMZM2G033855	XLOC_022237	divergent	4	226,843,654	226,844,563	+	226,843,625	226,844,335	-
GRMZM2G017022	XLOC_022248	enclosed	4	228,425,361	228,428,399	+	228,426,856	228,427,885	-
GRMZM2G069631	XLOC_022270	enclosed	4	230,038,398	230,047,846	+	230,046,909	230,047,649	-
GRMZM2G022538	XLOC_022280	enclosed	4	231,263,824	231,272,537	+	231,270,481	231,272,402	-
GRMZM2G015419	XLOC_022325	enclosed	4	234,164,884	234,168,656	+	234,165,958	234,168,605	-
GRMZM2G008859	XLOC_020246	convergent	4	235,108,555	235,111,831	-	235,108,395	235,110,759	+
GRMZM2G125308	XLOC_022347	enclosed	4	235,227,459	235,229,436	+	235,228,487	235,229,021	-
XLOC_020263	XLOC_022355	enclosed	4	235,657,814	235,659,734	+	235,658,484	235,659,038	-
XLOC_022369	XLOC_020286	divergent	4	236,262,710	236,264,243	-	236,263,993	236,264,323	+
XLOC_022369	XLOC_020285	enclosed	4	236,262,710	236,264,243	-	236,263,278	236,263,657	+
GRMZM2G303374	GRMZM2G003157	convergent	4	237,811,718	237,813,201	-	237,801,951	237,811,959	+
XLOC_020330	XLOC_022406	divergent	4	237,855,481	237,858,140	+	237,855,270	237,857,865	-
GRMZM2G166035	XLOC_020341	enclosed	4	238,052,080	238,055,542	-	238,052,108	238,052,348	+
XLOC_022421	XLOC_020354	divergent	4	238,664,013	238,666,194	-	238,665,605	238,666,775	+
GRMZM2G060451	XLOC_020356	enclosed	4	238,872,905	238,878,643	-	238,872,952	238,873,626	+
GRMZM2G060451	XLOC_020358	enclosed	4	238,872,905	238,878,643	-	238,874,856	238,875,709	+
GRMZM2G027209	GRMZM2G027640	convergent	4	239,288,712	239,300,856	-	239,288,344	239,289,063	+
GRMZM2G324956	XLOC_022453	enclosed	4	239,528,888	239,529,910	+	239,529,220	239,529,624	-
XLOC_020374	XLOC_022459	enclosed	4	239,641,012	239,641,380	+	239,641,083	239,641,380	-
GRMZM2G169998	XLOC_020387	divergent	4	240,049,949	240,053,626	-	240,053,313	240,053,631	+
GRMZM2G025127	XLOC_022492	enclosed	4	240,272,545	240,276,538	+	240,275,454	240,276,216	-
GRMZM2G014170	XLOC_022534	enclosed	5	803,571	810,274	-	803,629	804,211	+
AC190636.3_FG005	GRMZM2G019866	convergent	5	813,840	815,338	-	812,882	814,072	+
AC190636.3_FG005	XLOC_022536	divergent	5	813,840	815,338	-	814,586	815,398	+
GRMZM2G384339	GRMZM2G384338	divergent	5	888,240	893,249	-	892,919	893,892	+
GRMZM2G384070	XLOC_024909	enclosed	5	922,448	924,190	+	923,046	923,893	-
GRMZM2G448001	GRMZM2G447976	convergent	5	1,526,661	1,529,125	-	1,524,444	1,527,639	+
XLOC_022572	GRMZM2G148937	enclosed	5	1,545,003	1,545,310	+	1,543,474	1,546,975	-
GRMZM2G125784	XLOC_022577	divergent	5	1,582,563	1,583,065	-	1,582,617	1,583,214	+
GRMZM2G023081	XLOC_024939	enclosed	5	1,728,200	1,729,396	+	1,728,820	1,729,172	-
GRMZM2G138421	GRMZM2G138494	convergent	5	1,846,419	1,853,070	-	1,843,396	1,848,286	+
GRMZM2G100524	XLOC_024948	enclosed	5	1,872,661	1,875,805	+	1,873,072	1,874,163	-
GRMZM2G121715	XLOC_024956	enclosed	5	2,109,630	2,112,133	+	2,111,143	2,111,810	-
XLOC_024980	GRMZM2G110952	divergent	5	2,593,362	2,594,082	-	2,593,535	2,595,061	+
GRMZM2G410865	GRMZM2G110952	convergent	5	2,594,463	2,604,977	-	2,593,535	2,595,061	+
GRMZM2G124441	XLOC_022628	enclosed	5	2,926,017	2,928,711	-	2,927,085	2,928,199	+
GRMZM2G332976	GRMZM2G038075	enclosed	5	3,145,962	3,147,816	-	3,143,125	3,147,996	+
GRMZM2G054224	XLOC_025015	convergent	5	3,777,982	3,779,876	+	3,779,249	3,781,015	-
GRMZM5G897976	XLOC_025018	enclosed	5	3,813,478	3,818,430	+	3,813,634	3,814,093	-
GRMZM2G701297	XLOC_025021	enclosed	5	3,944,180	3,955,109	+	3,953,124	3,953,974	-
GRMZM2G009928	XLOC_025021	enclosed	5	3,953,080	3,956,606	+	3,953,124	3,953,974	-
GRMZM2G099474	GRMZM2G099449	convergent	5	4,312,135	4,317,196	-	4,309,513	4,315,155	+
GRMZM2G099615	XLOC_025031	divergent	5	4,345,261	4,349,288	+	4,345,145	4,346,123	-
GRMZM2G130544	XLOC_025033	convergent	5	4,423,409	4,425,429	+	4,423,438	4,427,276	-
GRMZM2G124229	XLOC_025038	divergent	5	4,606,404	4,608,549	+	4,605,996	4,607,845	-
GRMZM2G029101	XLOC_025044	enclosed	5	4,766,059	4,769,132	+	4,767,236	4,767,975	-
GRMZM2G029101	XLOC_025045	convergent	5	4,766,059	4,769,132	+	4,768,343	4,769,519	-
GRMZM2G171468	XLOC_022735	enclosed	5	6,146,163	6,149,014	-	6,146,270	6,148,226	+
GRMZM2G035103	XLOC_025077	enclosed	5	6,320,889	6,322,687	+	6,321,319	6,321,822	-
GRMZM2G071970	XLOC_025097	convergent	5	6,914,005	6,917,726	+	6,916,911	6,920,360	-
GRMZM2G071970	XLOC_025096	enclosed	5	6,914,005	6,917,726	+	6,914,660	6,915,335	-
GRMZM2G077389	XLOC_022769	enclosed	5	7,772,722	7,774,847	-	7,773,900	7,774,829	+
GRMZM2G077295	GRMZM2G077356	convergent	5	7,781,628	7,787,521	-	7,780,703	7,783,409	+
GRMZM2G147814	GRMZM2G147809	enclosed	5	7,866,856	7,869,861	-	7,866,128	7,870,805	+
GRMZM2G096591	GRMZM2G096596	convergent	5	9,918,543	9,921,805	-	9,911,089	9,919,240	+
GRMZM2G152548	XLOC_022819	divergent	5	10,240,536	10,243,482	-	10,242,631	10,243,514	+

GRMZM2G145827	GRMZM2G145850	convergent	5	10,796,873	10,804,954	-	10,796,396	10,797,296	+
GRMZM2G102616	XLOC_022856	enclosed	5	12,237,732	12,238,164	-	12,237,732	12,238,164	+
GRMZM2G102760	XLOC_022858	enclosed	5	12,285,642	12,290,523	-	12,289,155	12,289,430	+
GRMZM2G176723	XLOC_022911	enclosed	5	14,609,748	14,610,757	-	14,609,902	14,610,741	+
GRMZM2G176688	GRMZM5G822180	convergent	5	14,610,901	14,625,816	+	14,625,518	14,626,540	-
GRMZM2G066191	XLOC_022917	enclosed	5	14,870,256	14,874,075	-	14,870,816	14,871,240	+
XLOC_025279	XLOC_022949	enclosed	5	16,709,490	16,713,961	-	16,713,440	16,713,755	+
GRMZM2G087326	XLOC_025299	enclosed	5	18,789,107	18,791,842	+	18,789,219	18,789,560	-
GRMZM2G124863	GRMZM2G124918	convergent	5	19,696,842	19,699,376	+	19,698,812	19,702,780	-
GRMZM2G124918	XLOC_022991	divergent	5	19,698,812	19,702,780	-	19,699,624	19,702,860	+
GRMZM2G170128	XLOC_022997	enclosed	5	20,622,861	20,626,562	-	20,624,595	20,624,934	+
GRMZM2G132036	XLOC_023002	enclosed	5	20,929,213	20,934,778	-	20,929,481	20,930,339	+
GRMZM5G851862	XLOC_025335	divergent	5	21,362,862	21,366,235	+	21,362,636	21,363,839	-
GRMZM2G100158	XLOC_025355	enclosed	5	22,675,530	22,679,029	+	22,678,363	22,678,971	-
GRMZM2G113135	XLOC_025361	enclosed	5	23,017,949	23,018,871	+	23,018,093	23,018,717	-
GRMZM2G018695	XLOC_023042	enclosed	5	23,677,551	23,679,570	-	23,677,896	23,679,481	+
GRMZM2G107444	XLOC_025382	enclosed	5	24,175,048	24,178,212	+	24,175,168	24,175,544	-
GRMZM2G073034	XLOC_023058	divergent	5	24,988,574	24,989,582	-	24,989,489	24,989,908	+
GRMZM2G021973	XLOC_023106	divergent	5	30,326,757	30,327,652	-	30,327,205	30,327,906	+
GRMZM2G154954	XLOC_025452	enclosed	5	31,464,389	31,465,555	+	31,464,541	31,465,344	-
GRMZM2G326707	XLOC_025458	divergent	5	31,900,737	31,903,637	+	31,900,586	31,902,800	-
GRMZM2G337048	XLOC_025464	divergent	5	32,245,099	32,247,118	+	32,244,735	32,247,057	-
GRMZM5G865576	XLOC_025491	enclosed	5	34,419,553	34,433,877	+	34,430,419	34,430,697	-
GRMZM2G084021	XLOC_023151	enclosed	5	35,018,140	35,021,326	-	35,020,613	35,021,303	+
GRMZM2G017805	XLOC_025498	enclosed	5	35,290,711	35,295,535	+	35,291,098	35,291,752	-
GRMZM2G043182	XLOC_025508	divergent	5	35,731,107	35,732,791	+	35,731,101	35,732,701	-
GRMZM2G012209	XLOC_025549	convergent	5	39,895,036	39,906,590	+	39,904,934	39,908,229	-
GRMZM2G134597	XLOC_023196	enclosed	5	39,980,153	39,982,962	-	39,981,012	39,982,858	+
GRMZM2G366077	XLOC_025566	enclosed	5	41,225,312	41,226,535	+	41,225,477	41,225,885	-
XLOC_025579	XLOC_023220	convergent	5	43,684,627	43,686,211	-	43,683,623	43,686,190	+
AC209987.4_FG010	GRMZM5G882378	convergent	5	46,975,199	46,976,425	-	46,973,011	46,976,155	+
GRMZM2G463913	GRMZM2G163544	divergent	5	48,477,760	48,480,813	+	48,472,809	48,477,815	-
XLOC_025618	GRMZM5G848124	divergent	5	48,600,426	48,601,438	-	48,600,490	48,601,830	+
GRMZM2G140443	XLOC_025621	divergent	5	48,902,473	48,912,285	+	48,902,394	48,902,868	-
GRMZM2G141858	XLOC_025626	enclosed	5	49,575,119	49,577,760	+	49,575,144	49,575,550	-
GRMZM2G113655	XLOC_023265	enclosed	5	50,115,509	50,120,425	-	50,119,317	50,120,005	+
GRMZM2G382785	XLOC_025635	divergent	5	50,372,099	50,374,372	+	50,371,817	50,373,787	-
GRMZM2G026881	XLOC_023268	enclosed	5	50,478,793	50,481,470	-	50,479,714	50,480,691	+
GRMZM2G080183	XLOC_023299	enclosed	5	53,425,033	53,427,956	-	53,425,395	53,426,300	+
GRMZM2G026980	XLOC_025661	enclosed	5	53,935,017	53,936,879	+	53,935,638	53,936,044	-
GRMZM2G099434	XLOC_025682	enclosed	5	56,392,388	56,394,936	+	56,392,398	56,393,951	-
GRMZM2G099413	XLOC_023317	convergent	5	56,397,216	56,398,582	-	56,396,973	56,397,760	+
GRMZM2G135904	XLOC_025700	enclosed	5	59,063,729	59,069,485	+	59,063,826	59,064,502	-
GRMZM2G135904	GRMZM2G439203	convergent	5	59,063,729	59,069,485	+	59,068,780	59,072,816	-
GRMZM2G385945	XLOC_025710	enclosed	5	59,299,608	59,301,346	+	59,299,682	59,300,279	-
GRMZM2G085117	XLOC_025713	enclosed	5	59,338,041	59,339,527	+	59,337,823	59,339,561	-
GRMZM2G102447	XLOC_025722	enclosed	5	59,753,812	59,756,561	+	59,754,028	59,754,289	-
XLOC_025737	XLOC_023360	enclosed	5	60,683,906	60,685,891	-	60,684,602	60,685,656	+
XLOC_023371	GRMZM2G417454	enclosed	5	61,454,332	61,457,358	+	61,455,740	61,457,120	-
GRMZM2G399073	GRMZM2G541730	convergent	5	61,555,926	61,560,984	-	61,554,990	61,556,871	+
GRMZM2G065125	XLOC_023383	enclosed	5	62,142,491	62,147,249	-	62,142,767	62,143,464	+
GRMZM2G048073	XLOC_023388	enclosed	5	62,624,370	62,626,140	-	62,625,055	62,626,051	+
GRMZM2G132331	XLOC_023389	enclosed	5	62,711,015	62,712,701	-	62,711,807	62,712,234	+
XLOC_025771	XLOC_023396	convergent	5	63,306,785	63,308,362	-	63,305,844	63,307,092	+
GRMZM2G057000	XLOC_025800	enclosed	5	65,138,689	65,142,574	+	65,141,483	65,141,724	-
GRMZM2G055682	GRMZM2G055619	convergent	5	65,230,942	65,233,276	+	65,233,060	65,236,881	-
GRMZM2G128929	XLOC_025823	enclosed	5	67,432,374	67,434,191	+	67,433,439	67,433,878	-
GRMZM2G159047	GRMZM2G159142	convergent	5	67,701,021	67,703,449	-	67,697,937	67,701,535	+
GRMZM2G108474	XLOC_023438	enclosed	5	68,048,954	68,051,724	-	68,049,906	68,051,281	+
GRMZM2G035073	XLOC_023440	divergent	5	68,336,691	68,338,762	-	68,336,872	68,338,931	+
GRMZM2G088114	XLOC_025845	enclosed	5	69,340,886	69,351,028	+	69,341,489	69,343,791	-
GRMZM2G156824	XLOC_025853	enclosed	5	70,111,506	70,115,642	+	70,114,064	70,115,222	-
GRMZM2G466281	GRMZM2G164202	convergent	5	70,795,837	70,802,844	+	70,802,312	70,804,163	-
GRMZM2G164242	XLOC_023479	convergent	5	70,805,268	70,812,495	-	70,804,919	70,806,365	+
GRMZM2G029879	XLOC_025877	enclosed	5	73,076,939	73,079,038	+	73,078,048	73,079,005	-
XLOC_025887	XLOC_023504	divergent	5	74,436,334	74,438,340	-	74,437,381	74,438,365	+
GRMZM2G171354	GRMZM2G171349	enclosed	5	76,138,603	76,148,199	+	76,144,354	76,147,672	-
GRMZM6G869379	XLOC_025906	enclosed	5	76,240,628	76,243,376	+	76,240,730	76,241,160	-
AC192244.3_FG007	XLOC_025907	convergent	5	76,249,702	76,252,730	+	76,252,113	76,255,338	-
GRMZM2G139689	GRMZM2G139714	convergent	5	76,410,293	76,415,357	+	76,413,820	76,418,130	-
GRMZM2G042278	GRMZM2G042245	convergent	5	76,714,970	76,718,433	-	76,710,383	76,716,709	+
GRMZM2G010920	GRMZM2G010779	convergent	5	77,781,686	77,784,624	-	77,780,713	77,781,895	+
GRMZM2G097207	XLOC_025924	convergent	5	77,949,479	77,954,635	+	77,952,734	77,954,985	-
XLOC_023583	XLOC_025954	enclosed	5	81,799,333	81,801,056	+	81,799,469	81,799,920	-
GRMZM2G088396	GRMZM2G088235	convergent	5	83,895,830	83,898,120	+	83,896,631	83,902,358	-
GRMZM2G322129	XLOC_025974	enclosed	5	84,097,690	84,102,291	+	84,098,409	84,099,212	-
AC207043.3_FG002	XLOC_025989	enclosed	5	86,050,120	86,052,355	+	86,050,854	86,051,671	-
GRMZM2G180211	GRMZM2G180218	convergent	5	86,185,986	86,189,477	-	86,185,411	86,186,569	+
GRMZM2G138527	XLOC_025998	enclosed	5	87,452,593	87,457,026	+	87,452,681	87,453,022	-
GRMZM2G075333	XLOC_023638	enclosed	5	89,189,606	89,194,342	-	89,193,300	89,194,264	+
GRMZM2G057328	XLOC_026024	enclosed	5	92,390,468	92,393,828	+	92,392,744	92,393,814	-
GRMZM2G120320	XLOC_026028	enclosed	5	92,841,722	92,844,459	+	92,842,158	92,842,805	-
GRMZM2G060485	XLOC_023659	enclosed	5	93,274,116	93,276,858	-	93,275,224	93,275,553	+
GRMZM2G117544	XLOC_023660	enclosed	5	93,551,459	93,555,969	-	93,551,777	93,552,691	+
GRMZM2G344163	XLOC_026084	enclosed	5	106,298,211	106,301,969	+	106,298,927	106,299,362	-
GRMZM2G138349	XLOC_023749	enclosed	5	118,709,138	118,712,441	-	118,710,432	118,710,838	+
GRMZM2G029979	GRMZM2G331374	convergent	5	122,676,304	122,689,150	+	122,688,380	122,692,932	-
GRMZM2G009479	XLOC_026137	enclosed	5	123,239,697	123,243,181	+	123,240,208	123,243,052	-
GRMZM2G429540	XLOC_023893	divergent	5	146,922,333	146,924,650	-	146,924,241	146,924,930	+
GRMZM2G429540	GRMZM2G129713	enclosed	5	146,922,333	146,924,650	-	146,922,694	146,923,762	+
GRMZM2G026490	XLOC_023896	enclosed	5	147,664,347	147,670,122	-	147,664,563	147,665,713	+
GRMZM2G176206	XLOC_023914	divergent	5	151,922,535	151,926,261	-	151,923,737	151,926,498	+
GRMZM2G161299	XLOC_023917	enclosed	5	152,470,035	152,479,785	-	152,472,231	152,472,631	+
XLOC_026313	XLOC_023924	enclosed	5	153,416,615	153,417,927	-	153,416,709	153,417,683	+
GRMZM2G318633	XLOC_026322	divergent	5	155,564,693	155,565,998	+	155,564,633	155,565,984	-
GRMZM2G028677	XLOC_026349	enclosed	5	159,951,449	159,953,399	+	159,952,069	159,952,709	-
GRMZM2G012046	XLOC_026357	divergent	5	160,693,221	160,699,305	+	160,692,953	160,693,951	-
GRMZM2G149115	XLOC_026373	enclosed	5	162,094,523	162,098,211	+	162,097,544	162,097,925	-
GRMZM2G430526	XLOC_023994	convergent	5	162,737,531	162,743,559	-	162,737,140	162,738,369	+
XLOC_023995	GRMZM2G430526	enclosed	5	162,742,238	162,743,559	+	162,737,531	162,743,559	-
GRMZM2G004856	XLOC_024013	convergent	5	163,455,311	163,457,941	-	163,455,309	163,455,697	+
GRMZM2G004856	XLOC_024014	enclosed	5	163,455,311	163,457,941	-	163,456,033	163,456,790	+
GRMZM2G011631	XLOC_026392	convergent	5	164,380,707	164,401,819	+	164,401,022	164,401,850	-
GRMZM2G018820	XLOC_024036	divergent	5	164,767,387	164,770,778	-	164,769,816	164,771,166	+
XLOC_024053	XLOC_026411	enclosed	5	167,468,740	167,469,535	+	167,468,601	167,469,684	-
GRMZM2G020840	XLOC_024055	enclosed	5	167,625,007	167,626,599	-	167,625,144	167,625,890	+
GRMZM5G876303	XLOC_024063	enclosed	5	168,572,548	168,575,048	-	168,573,632	168,574,495	+
GRMZM2G139300	XLOC_026442	enclosed	5	169,497,565	169,502,117	+	169,499,086	169,501,429	-
GRMZM2G139300	XLOC_026443	enclosed	5	169,497,565	169,502,117	+	169,501,558	169,502,071	-
GRMZM2G451314	XLOC_024086	enclosed	5	170,458,785	170,463,276	-	170,462,806	170,463,097	+
GRMZM2G303964	XLOC_024088	enclosed	5	170,543,803	170,545,707	-	170,545,309	170,545,691	+
GRMZM2G303964	XLOC_024087	enclosed	5	170,543,803	170,545,707	-	170,544,174	170,544,600	+
GRMZM2G118825	XLOC_024096	divergent	5	171,317,073	171,318,365	-	171,317,220	171,318,479	+
XLOC_026470	GRMZM2G043193	convergent	5	171,563,296	171,567,672	-	171,561,191	171,564,389	+
GRMZM2G031581	XLOC_026481	enclosed	5	172,660,909	172,663,831	+	172,660,909	172,663,243	-
GRMZM2G119517	GRMZM2G119566	convergent	5	172,851,847	172,858,649	-	172,849,643	172,852,744	+
XLOC_024136	GRMZM2G421231	enclosed	5	174,638,160	174,638,628	+	174,638,160	174,638,454	-
GRMZM2G436328	XLOC_024137	convergent	5	174,664,930	174,666,991	-	174,664,668	174,665,015	+
GRMZM2G023988	XLOC_024153	enclosed	5	175,731,393	175,736,518	-	175,735,743	175,736,375	+
XLOC_026536	XLOC_024159	enclosed	5	176,155,022	176,156,880	-	176,155,032	176,155,421	+
GRMZM2G132706	XLOC_026539	enclosed	5	176,656,120	176,658,005	+	176,656,189	176,657,863	-
GRMZM2G146380	XLOC_024174	enclosed	5	176,985,247	176,986,711	-	176,985,833	176,986,251	+
GRMZM2G492156	XLOC_026542	enclosed	5	177,275,115	177,279,005	+	177,275,738	177,278,150	-
GRMZM2G410710	XLOC_024185	enclosed	5	177,872,339	177,873,651	-	177,873,141	177,873,462	+
GRMZM2G044359	XLOC_026582	enclosed	5	180,233,335	180,236,245	+	180,235,632	180,236,130	-
GRMZM2G095194	GRMZM2G095147	convergent	5	182,500,574	182,506,295	+	182,505,927	182,506,522	-
GRMZM2G129189	XLOC_026618	enclosed	5	182,568,960	182,570,172	+	182,569,224	182,570,100	-
GRMZM2G035584	XLOC_024267	enclosed	5	183,657,809	183,662,949	-	183,658,189	183,662,680	+
GRMZM2G028830	XLOC_026656	divergent	5	185,696,130	185,697,035	+	185,696,058	185,696,877	-
GRMZM2G141411	XLOC_024305	enclosed	5	187,620,886	187,622,518	-	187,621,682	187,622,099	+
GRMZM2G139765	XLOC_024321	enclosed	5	188,716,613	188,718,822	-	188,718,037	188,718,626	+
GRMZM2G139765	XLOC_024320	enclosed	5	188,716,613	188,718,822	-	188,717,132	188,717,767	+
XLOC_026704	GRMZM2G169967	convergent	5	188,881,231	188,884,099	-	188,877,054	188,881,772	+
GRMZM2G463291	XLOC_024338	enclosed	5	189,855,981	189,862,743	-	189,857,312	189,858,886	+
GRMZM2G117164	XLOC_024345	enclosed	5	190,526,842	190,528,626	-	190,528,090	190,528,564	+
GRMZM2G156599	XLOC_024350	divergent	5	190,726,076	190,729,337	-	190,728,306	190,729,357	+
GRMZM2G097704	XLOC_026733	enclosed	5	191,140,510	191,142,976	+	191,141,025	191,142,528	-
GRMZM2G094768	XLOC_024359	enclosed	5	191,180,794	191,183,392	-	191,181,067	191,182,830	+
GRMZM5G840002	XLOC_026737	enclosed	5	191,419,331	191,422,728	+	191,419,426	191,419,679	-
GRMZM2G170591	XLOC_026742	convergent	5	191,730,938	191,735,780	+	191,734,943	191,735,815	-
AC209208.3_FG002	AC209208.3_FG001	convergent	5	193,359,086	193,363,241	+	193,363,118	193,371,223	-
GRMZM2G074053	XLOC_026772	enclosed	5	194,144,303	194,146,501	+	194,144,551	194,146,414	-
XLOC_026779	XLOC_024414	enclosed	5	194,884,329	194,887,265	-	194,884,662	194,885,678	+
XLOC_026779	XLOC_024415	divergent	5	194,884,329	194,887,265	-	194,886,364	194,887,629	+
GRMZM2G154628	XLOC_026781	enclosed	5	195,239,858	195,242,631	+	195,240,448	195,240,744	-
GRMZM5G833032	XLOC_024432	enclosed	5	196,333,393	196,347,397	-	196,336,155	196,336,999	+
GRMZM2G413829	XLOC_026825	divergent	5	198,928,604	198,930,118	+	198,928,222	198,928,999	-
GRMZM2G413829	XLOC_026826	enclosed	5	198,928,604	198,930,118	+	198,929,056	198,929,674	-
GRMZM2G141975	XLOC_024467	convergent	5	199,217,605	199,222,280	-	199,216,642	199,219,395	+
GRMZM2G021270	XLOC_024483	enclosed	5	199,912,838	199,920,152	-	199,913,016	199,913,887	+
GRMZM2G325019	GRMZM2G325038	convergent	5	200,487,573	200,491,201	+	200,490,849	200,492,345	-
GRMZM2G179147	XLOC_024496	enclosed	5	200,704,676	200,707,817	-	200,705,200	200,707,736	+
GRMZM2G101287	GRMZM2G101350	convergent	5	200,990,577	200,993,961	+	200,993,550	200,995,952	-
GRMZM2G170595	GRMZM2G170602	convergent	5	201,723,129	201,726,317	-	201,721,167	201,723,705	+
GRMZM2G009506	XLOC_026868	enclosed	5	201,992,788	201,996,158	+	201,995,204	201,995,662	-
GRMZM2G315176	XLOC_026869	enclosed	5	202,034,268	202,050,016	+	202,049,090	202,049,570	-
GRMZM2G481604	GRMZM2G180983	divergent	5	202,139,172	202,141,517	-	202,141,025	202,150,154	+
GRMZM2G056252	XLOC_024545	enclosed	5	203,092,250	203,097,186	-	203,092,300	203,093,896	+
GRMZM5G848822	XLOC_026901	enclosed	5	203,730,336	203,732,571	+	203,731,859	203,732,426	-
GRMZM5G848822	XLOC_026900	enclosed	5	203,730,336	203,732,571	+	203,730,871	203,731,291	-
GRMZM2G128992	XLOC_026903	enclosed	5	203,754,997	203,756,242	+	203,755,612	203,756,080	-
XLOC_024603	GRMZM6G946111	enclosed	5	204,771,774	204,772,556	+	204,771,774	204,772,556	-
GRMZM2G385999	XLOC_026933	enclosed	5	205,283,735	205,284,798	+	205,284,023	205,284,450	-
GRMZM2G181507	GRMZM2G481730	convergent	5	205,389,374	205,401,139	-	205,385,840	205,390,104	+
GRMZM2G151204	XLOC_024629	divergent	5	205,591,027	205,593,334	-	205,591,579	205,593,459	+
GRMZM2G142779	XLOC_024635	enclosed	5	205,782,086	205,786,901	-	205,782,086	205,783,762	+
GRMZM2G142709	XLOC_024636	enclosed	5	205,803,187	205,805,607	-	205,804,786	205,805,352	+
GRMZM5G856201	XLOC_024639	enclosed	5	205,888,002	205,897,899	-	205,896,837	205,897,823	+
GRMZM5G856201	XLOC_024638	enclosed	5	205,888,002	205,897,899	-	205,896,279	205,896,680	+
GRMZM2G335280	XLOC_024639	enclosed	5	205,896,071	205,898,040	-	205,896,837	205,897,823	+
GRMZM2G335280	XLOC_024638	enclosed	5	205,896,071	205,898,040	-	205,896,279	205,896,680	+
GRMZM2G480364	XLOC_024641	enclosed	5	205,983,909	205,985,356	-	205,984,369	205,984,925	+
GRMZM2G179768	XLOC_024642	divergent	5	205,986,279	205,987,943	-	205,986,559	205,988,233	+
GRMZM2G140651	XLOC_026963	divergent	5	206,668,533	206,670,932	+	206,668,390	206,669,348	-
GRMZM2G028325	XLOC_026972	enclosed	5	206,972,607	206,976,286	+	206,973,115	206,975,993	-
GRMZM2G035503	XLOC_024678	enclosed	5	208,137,640	208,142,328	-	208,141,589	208,142,230	+
GRMZM2G323013	XLOC_027011	divergent	5	208,973,665	208,975,706	+	208,973,552	208,975,257	-
GRMZM2G103128	XLOC_027033	divergent	5	209,912,145	209,917,207	+	209,911,903	209,913,130	-
GRMZM2G113418	GRMZM2G414621	convergent	5	210,349,025	210,351,808	+	210,350,135	210,352,786	-
GRMZM2G149452	XLOC_024728	enclosed	5	210,458,456	210,461,256	-	210,458,777	210,459,232	+
GRMZM2G154332	XLOC_024731	enclosed	5	210,524,837	210,526,166	-	210,525,082	210,525,511	+
GRMZM2G052422	XLOC_027066	enclosed	5	210,825,897	210,828,454	+	210,827,628	210,828,045	-
GRMZM2G163749	XLOC_024762	enclosed	5	212,075,870	212,078,778	-	212,076,038	212,078,347	+
GRMZM2G124416	GRMZM2G124377	convergent	5	212,222,159	212,227,637	-	212,213,297	212,223,198	+
XLOC_024786	XLOC_027111	enclosed	5	213,088,218	213,090,006	+	213,088,484	213,089,266	-
GRMZM2G122231	XLOC_027112	enclosed	5	213,103,814	213,107,810	+	213,106,063	213,106,920	-
GRMZM2G469111	XLOC_024802	enclosed	5	213,993,280	213,996,291	-	213,993,902	213,994,790	+
GRMZM2G180384	XLOC_027142	enclosed	5	214,192,037	214,195,300	+	214,192,115	214,192,485	-
GRMZM2G163200	XLOC_027154	enclosed	5	214,574,405	214,580,589	+	214,574,785	214,576,245	-
GRMZM2G150984	XLOC_024823	enclosed	5	215,072,597	215,077,359	-	215,072,718	215,073,186	+
GRMZM2G058289	XLOC_027171	enclosed	5	215,100,492	215,104,749	+	215,101,686	215,102,542	-
GRMZM5G851655	XLOC_024826	enclosed	5	215,112,799	215,116,277	-	215,113,770	215,114,112	+
GRMZM5G862817	XLOC_027174	enclosed	5	215,314,528	215,316,506	+	215,315,528	215,316,236	-
GRMZM5G806449	XLOC_027175	enclosed	5	215,337,121	215,346,503	+	215,337,508	215,345,982	-
GRMZM2G078887	XLOC_024833	enclosed	5	215,406,418	215,407,900	-	215,406,977	215,407,577	+
GRMZM5G848768	XLOC_024851	enclosed	5	215,868,272	215,872,218	-	215,868,390	215,868,937	+
GRMZM2G013981	GRMZM5G894916	divergent	5	215,883,819	215,887,609	-	215,886,969	215,893,442	+
GRMZM2G390076	GRMZM2G002124	enclosed	5	216,124,616	216,127,494	-	216,124,627	216,126,640	+
GRMZM2G133919	AC208348.3_FG012	enclosed	5	216,746,254	216,756,209	-	216,749,139	216,751,299	+
GRMZM2G048497	GRMZM2G048470	divergent	5	217,060,938	217,063,036	+	217,060,912	217,061,698	-
GRMZM2G375002	GRMZM2G073609	convergent	5	217,656,072	217,664,485	+	217,664,134	217,665,072	-
GRMZM2G358540	XLOC_027233	enclosed	6	655,906	657,038	-	656,433	656,943	+
GRMZM2G098174	XLOC_029097	enclosed	6	1,280,395	1,288,498	+	1,280,515	1,281,668	-
GRMZM2G367941	XLOC_027260	convergent	6	2,724,260	2,725,829	-	2,724,149	2,724,788	+
GRMZM2G374827	XLOC_027263	enclosed	6	3,041,977	3,053,364	-	3,052,254	3,053,325	+
GRMZM2G157296	XLOC_029119	enclosed	6	3,195,407	3,198,606	+	3,195,478	3,195,727	-
GRMZM2G117961	GRMZM2G117984	convergent	6	3,237,181	3,239,818	+	3,239,342	3,252,177	-
GRMZM2G364977	XLOC_029150	convergent	6	6,186,552	6,190,122	+	6,186,556	6,190,745	-
GRMZM2G009661	XLOC_027290	enclosed	6	6,434,660	6,438,643	-	6,437,835	6,438,475	+
GRMZM2G180659	XLOC_027296	enclosed	6	6,892,924	6,896,969	-	6,893,878	6,895,111	+
GRMZM2G127328	XLOC_029164	enclosed	6	7,047,564	7,050,778	+	7,048,330	7,049,129	-
GRMZM2G068955	XLOC_029170	divergent	6	8,109,337	8,111,820	+	8,109,205	8,109,622	-
GRMZM2G136662	XLOC_027310	enclosed	6	8,112,897	8,136,923	-	8,116,573	8,118,254	+
GRMZM2G301884	XLOC_029184	convergent	6	9,493,165	9,496,796	+	9,496,444	9,499,331	-
GRMZM2G134020	XLOC_029297	enclosed	6	13,653,638	13,654,912	+	13,653,532	13,654,945	-
XLOC_029304	XLOC_027435	divergent	6	14,021,636	14,022,577	-	14,021,973	14,022,731	+
XLOC_027436	XLOC_029309	divergent	6	14,236,270	14,236,911	+	14,236,161	14,236,484	-
GRMZM2G172980	XLOC_029332	enclosed	6	14,969,927	14,974,172	+	14,970,311	14,970,744	-
GRMZM2G155252	XLOC_027524	enclosed	6	21,463,563	21,465,430	-	21,464,815	21,465,424	+
GRMZM2G448607	XLOC_027540	enclosed	6	24,279,652	24,282,016	-	24,281,335	24,281,874	+
GRMZM2G119583	AC203408.4_FG003	convergent	6	24,318,350	24,320,654	-	24,316,692	24,320,269	+
GRMZM2G111579	XLOC_027544	enclosed	6	24,549,334	24,554,738	-	24,553,394	24,554,144	+

GRMZM2G016637	XLOC_027546	enclosed	6	25,700,899	25,705,088	-	25,702,321	25,703,777	+
GRMZM2G531804	AC203093.3_FG001	enclosed	6	26,716,656	26,722,230	-	26,716,656	26,717,457	+
GRMZM2G381051	GRMZM2G381086	convergent	6	27,107,889	27,118,645	+	27,118,145	27,120,852	-
GRMZM2G020091	GRMZM2G313448	enclosed	6	28,129,083	28,173,808	-	28,140,500	28,146,597	+
GRMZM2G099317	XLOC_029449	enclosed	6	31,415,780	31,420,025	+	31,419,080	31,419,330	-
GRMZM2G013581	XLOC_029470	enclosed	6	34,825,142	34,827,192	+	34,825,979	34,826,788	-
GRMZM2G305856	XLOC_027591	divergent	6	34,888,892	34,890,920	-	34,889,334	34,891,116	+
GRMZM2G001205	XLOC_029472	enclosed	6	35,061,175	35,062,828	+	35,062,195	35,062,742	-
GRMZM2G035741	GRMZM2G037140	convergent	6	35,892,962	35,896,659	+	35,896,003	35,897,785	-
GRMZM2G109821	XLOC_029482	enclosed	6	36,088,959	36,091,188	+	36,090,380	36,090,741	-
GRMZM2G334181	XLOC_029485	enclosed	6	36,482,625	36,484,087	+	36,483,546	36,484,012	-
GRMZM2G066326	XLOC_027626	enclosed	6	39,529,258	39,530,806	-	39,529,339	39,530,458	+
GRMZM2G312970	XLOC_027629	enclosed	6	39,822,086	39,827,373	-	39,822,601	39,823,721	+
GRMZM2G463462	GRMZM2G164916	convergent	6	55,754,752	55,763,319	+	55,760,353	55,764,329	-
GRMZM2G107073	GRMZM2G453388	convergent	6	56,261,420	56,264,391	-	56,251,970	56,263,608	+
GRMZM2G385287	XLOC_027699	enclosed	6	56,376,617	56,378,293	-	56,377,804	56,378,194	+
GRMZM2G127798	XLOC_027707	enclosed	6	57,903,594	57,906,115	-	57,903,816	57,905,297	+
GRMZM2G155943	XLOC_029599	enclosed	6	59,237,967	59,293,671	+	59,260,577	59,261,040	-
GRMZM2G456023	XLOC_029613	divergent	6	61,126,264	61,140,722	+	61,126,233	61,127,035	-
GRMZM2G077837	XLOC_029621	divergent	6	62,703,857	62,705,991	+	62,703,742	62,705,554	-
GRMZM2G069335	XLOC_027748	divergent	6	63,696,202	63,697,776	-	63,697,116	63,697,832	+
GRMZM2G430902	XLOC_029644	enclosed	6	67,615,156	67,617,583	+	67,615,942	67,616,607	-
GRMZM2G447347	XLOC_027793	enclosed	6	70,452,079	70,454,503	-	70,453,959	70,454,244	+
GRMZM2G362298	XLOC_029681	enclosed	6	74,071,296	74,073,747	+	74,072,319	74,072,936	-
GRMZM2G362298	XLOC_029680	divergent	6	74,071,296	74,073,747	+	74,071,067	74,072,057	-
GRMZM2G407279	XLOC_029685	convergent	6	74,511,521	74,512,256	+	74,511,770	74,512,313	-
GRMZM2G419542	XLOC_029697	enclosed	6	76,846,012	76,848,163	+	76,847,017	76,847,473	-
GRMZM2G700968	GRMZM2G700969	enclosed	6	78,786,923	78,787,431	-	78,785,552	78,790,300	+
GRMZM2G121374	XLOC_027860	divergent	6	81,784,893	81,785,834	-	81,785,309	81,785,930	+
GRMZM2G020912	XLOC_029728	enclosed	6	82,128,247	82,135,184	+	82,129,100	82,129,534	-
GRMZM2G065888	XLOC_027890	enclosed	6	83,989,254	83,994,612	-	83,989,400	83,990,186	+
GRMZM2G065105	XLOC_029744	enclosed	6	84,094,029	84,098,506	+	84,094,292	84,094,834	-
GRMZM2G308689	XLOC_027905	enclosed	6	85,681,129	85,683,710	-	85,683,407	85,683,665	+
GRMZM2G365961	XLOC_029759	enclosed	6	85,959,795	85,961,400	+	85,959,727	85,961,669	-
GRMZM2G134930	XLOC_029760	enclosed	6	86,045,729	86,048,942	+	86,045,763	86,046,735	-
GRMZM2G134759	XLOC_027911	enclosed	6	86,050,769	86,052,508	-	86,051,785	86,052,435	+
XLOC_029764	XLOC_027915	divergent	6	86,345,343	86,347,299	-	86,345,625	86,347,854	+
GRMZM2G434935	XLOC_027935	divergent	6	88,008,788	88,010,435	-	88,009,296	88,010,525	+
GRMZM2G175562	GRMZM2G175543	convergent	6	88,908,389	88,909,796	+	88,909,489	88,912,116	-
GRMZM2G150209	XLOC_029797	enclosed	6	89,061,130	89,064,359	+	89,061,498	89,062,022	-
GRMZM2G150256	XLOC_027953	enclosed	6	89,066,375	89,071,273	-	89,068,336	89,068,774	+
GRMZM2G150276	XLOC_027956	enclosed	6	89,072,232	89,074,190	-	89,072,372	89,073,406	+
GRMZM2G150302	XLOC_027958	enclosed	6	89,081,149	89,085,692	-	89,084,484	89,085,224	+
GRMZM2G089400	XLOC_027964	enclosed	6	89,228,410	89,230,672	-	89,230,128	89,230,670	+
GRMZM2G119714	XLOC_029829	enclosed	6	91,723,056	91,733,893	+	91,731,581	91,733,115	-
GRMZM2G043822	XLOC_027994	enclosed	6	92,976,168	92,979,012	-	92,978,624	92,978,883	+
GRMZM2G048092	GRMZM2G047969	convergent	6	93,375,032	93,377,483	-	93,373,671	93,375,664	+
GRMZM2G420715	XLOC_028005	enclosed	6	93,749,148	93,750,906	-	93,749,599	93,750,313	+
GRMZM2G420715	XLOC_028006	enclosed	6	93,749,148	93,750,906	-	93,750,550	93,750,889	+
GRMZM2G473162	GRMZM2G172413	convergent	6	94,663,619	94,665,309	-	94,661,977	94,664,066	+
GRMZM2G131020	XLOC_029895	divergent	6	97,274,349	97,277,738	+	97,274,338	97,274,901	-
GRMZM2G082118	XLOC_029909	divergent	6	98,420,954	98,422,447	+	98,420,496	98,422,255	-
GRMZM2G041714	XLOC_028083	divergent	6	100,364,390	100,365,388	-	100,364,490	100,365,727	+
GRMZM2G043310	XLOC_028096	enclosed	6	102,760,900	102,765,449	-	102,764,632	102,765,222	+
GRMZM2G310144	XLOC_028103	enclosed	6	103,569,642	103,577,833	-	103,569,817	103,570,762	+
GRMZM2G335618	XLOC_028114	enclosed	6	104,021,742	104,022,644	-	104,021,788	104,022,587	+
GRMZM2G032028	XLOC_028115	enclosed	6	104,030,544	104,033,188	-	104,031,359	104,032,086	+
GRMZM2G449817	XLOC_028123	enclosed	6	104,838,658	104,842,995	-	104,839,450	104,840,850	+
GRMZM2G348846	XLOC_029967	divergent	6	105,706,618	105,708,350	+	105,706,541	105,708,349	-
XLOC_029975	XLOC_028149	divergent	6	106,612,044	106,612,947	-	106,612,472	106,613,000	+
XLOC_028172	XLOC_029999	enclosed	6	108,236,815	108,241,211	+	108,237,703	108,238,301	-
GRMZM2G025491	XLOC_030013	enclosed	6	109,558,799	109,560,880	+	109,558,845	109,560,431	-
GRMZM2G136058	XLOC_030017	enclosed	6	110,002,951	110,008,161	+	110,003,021	110,003,296	-
GRMZM2G135970	XLOC_030018	enclosed	6	110,052,982	110,055,216	+	110,053,721	110,055,098	-
GRMZM2G118657	XLOC_030041	divergent	6	112,149,529	112,151,755	+	112,149,472	112,151,348	-
AC202439.3_FG001	XLOC_030054	divergent	6	113,900,332	113,901,471	+	113,900,300	113,901,293	-
GRMZM2G324375	XLOC_030074	divergent	6	116,206,148	116,207,201	+	116,206,088	116,206,943	-
GRMZM2G157113	XLOC_028270	divergent	6	116,979,182	116,981,588	-	116,980,830	116,981,749	+
XLOC_028309	XLOC_030139	enclosed	6	121,567,042	121,568,491	+	121,567,118	121,567,490	-
GRMZM2G176910	XLOC_028345	enclosed	6	124,546,088	124,548,966	-	124,547,569	124,547,840	+
GRMZM2G135108	XLOC_030169	enclosed	6	125,194,477	125,196,129	+	125,194,489	125,195,880	-
GRMZM2G357296	XLOC_028357	enclosed	6	125,424,088	125,424,971	-	125,424,395	125,424,938	+
GRMZM2G040702	GRMZM2G341729	convergent	6	126,329,584	126,334,694	-	126,329,458	126,332,466	+
GRMZM2G163054	XLOC_030200	divergent	6	127,773,719	127,775,141	+	127,773,484	127,775,089	-
GRMZM2G167387	XLOC_030202	enclosed	6	127,870,096	127,871,796	+	127,870,521	127,871,444	-
GRMZM2G108714	XLOC_030209	enclosed	6	128,235,624	128,237,977	+	128,236,074	128,237,326	-
GRMZM2G352618	XLOC_030216	enclosed	6	128,637,993	128,638,947	+	128,638,235	128,638,848	-
GRMZM2G447795	XLOC_030226	divergent	6	129,268,752	129,270,611	+	129,268,701	129,270,059	-
GRMZM2G032896	XLOC_028414	divergent	6	132,244,350	132,248,959	-	132,247,916	132,248,974	+
GRMZM2G118951	XLOC_030279	enclosed	6	132,410,000	132,412,045	+	132,410,072	132,411,648	-
GRMZM2G339091	XLOC_028447	divergent	6	134,280,739	134,285,176	-	134,284,618	134,285,372	+
GRMZM2G147245	XLOC_030307	enclosed	6	135,723,001	135,725,403	+	135,724,233	135,725,239	-
GRMZM2G447480	XLOC_028494	divergent	6	138,474,315	138,476,165	-	138,474,734	138,476,438	+
GRMZM2G340444	XLOC_030347	divergent	6	138,775,095	138,776,992	+	138,774,979	138,776,272	-
GRMZM2G122479	XLOC_030357	enclosed	6	139,464,334	139,470,019	+	139,464,546	139,466,534	-
GRMZM2G423863	GRMZM2G423898	divergent	6	139,641,367	139,643,749	-	139,642,333	139,643,913	+
GRMZM2G068323	GRMZM2G068436	convergent	6	140,114,567	140,120,483	-	140,106,997	140,115,632	+
GRMZM2G093325	XLOC_028518	enclosed	6	140,914,068	140,915,871	-	140,914,832	140,915,320	+
GRMZM2G039113	XLOC_028541	enclosed	6	142,758,866	142,760,748	-	142,758,863	142,760,970	+
GRMZM2G132478	XLOC_030415	divergent	6	142,833,016	142,835,191	+	142,832,661	142,834,858	-
GRMZM2G132461	XLOC_028545	divergent	6	142,836,658	142,839,545	-	142,838,170	142,839,820	+
GRMZM5G869788	XLOC_028566	enclosed	6	144,147,804	144,153,012	-	144,148,114	144,148,914	+
GRMZM2G479608	XLOC_028587	enclosed	6	145,511,301	145,516,044	-	145,512,115	145,515,915	+
GRMZM2G173630	XLOC_030460	enclosed	6	146,310,907	146,313,590	+	146,311,139	146,313,337	-
GRMZM5G827496	XLOC_028605	enclosed	6	146,885,515	146,888,876	-	146,886,717	146,888,479	+
GRMZM2G070199	XLOC_030488	convergent	6	147,917,374	147,922,817	+	147,921,865	147,922,850	-
GRMZM2G180328	XLOC_030490	enclosed	6	148,062,224	148,064,507	+	148,063,742	148,064,148	-
XLOC_030495	XLOC_028627	enclosed	6	148,154,920	148,162,601	-	148,160,727	148,161,594	+
GRMZM2G111955	XLOC_028633	convergent	6	148,628,022	148,632,519	-	148,627,957	148,629,694	+
GRMZM2G111955	XLOC_028632	convergent	6	148,628,022	148,632,519	-	148,627,686	148,629,694	+
GRMZM2G073310	XLOC_030512	divergent	6	149,280,665	149,282,838	+	149,279,487	149,282,523	-
GRMZM2G061906	XLOC_030548	enclosed	6	151,319,337	151,321,751	+	151,319,368	151,321,310	-
GRMZM2G431350	GRMZM2G130239	convergent	6	151,980,700	151,983,784	-	151,977,873	151,981,648	+
GRMZM2G143745	XLOC_028691	enclosed	6	152,066,408	152,067,533	-	152,066,590	152,067,328	+
GRMZM2G127297	GRMZM2G127350	convergent	6	153,503,421	153,508,370	+	153,507,939	153,512,599	-
XLOC_028717	GRMZM2G107739	convergent	6	153,691,452	153,694,259	+	153,692,636	153,697,073	-
GRMZM2G125034	XLOC_030599	convergent	6	153,977,792	153,980,864	+	153,980,084	153,981,320	-
GRMZM2G125034	XLOC_030598	enclosed	6	153,977,792	153,980,864	+	153,978,666	153,979,619	-
GRMZM2G390436	XLOC_028736	divergent	6	154,430,572	154,432,792	-	154,431,415	154,432,970	+
XLOC_028750	XLOC_030619	divergent	6	155,074,658	155,076,625	+	155,074,555	155,076,308	-
GRMZM2G429714	XLOC_028755	enclosed	6	155,208,311	155,214,291	-	155,208,385	155,211,113	+
GRMZM2G116258	XLOC_028766	divergent	6	155,783,970	155,788,502	-	155,787,657	155,788,601	+
GRMZM2G440968	GRMZM2G440949	convergent	6	155,861,586	155,862,536	-	155,855,927	155,862,458	+
GRMZM2G090300	XLOC_028777	enclosed	6	155,951,341	155,953,332	-	155,952,708	155,953,265	+
GRMZM2G305159	XLOC_030654	divergent	6	156,405,012	156,413,226	+	156,404,946	156,405,356	-
GRMZM2G078781	XLOC_030671	enclosed	6	157,072,612	157,077,219	+	157,074,323	157,077,111	-
GRMZM2G375904	XLOC_030694	enclosed	6	158,380,660	158,388,837	+	158,380,664	158,381,595	-
GRMZM2G074792	XLOC_030695	enclosed	6	158,417,573	158,422,014	+	158,421,162	158,421,506	-
GRMZM2G043695	XLOC_030722	divergent	6	160,053,924	160,055,102	+	160,053,752	160,055,101	-
AC206788.3_FG015	GRMZM2G058314	convergent	6	160,084,248	160,087,779	+	160,084,716	160,087,818	-
GRMZM2G004349	XLOC_030725	enclosed	6	160,191,073	160,193,306	+	160,191,162	160,191,402	-
GRMZM2G004932	XLOC_028869	enclosed	6	160,229,718	160,235,410	-	160,233,892	160,235,240	+
GRMZM2G132212	GRMZM2G432801	convergent	6	160,429,758	160,434,506	+	160,433,610	160,439,979	-
GRMZM2G158972	XLOC_028899	divergent	6	161,413,828	161,420,521	-	161,419,537	161,420,979	+
GRMZM2G090245	XLOC_028905	divergent	6	161,647,137	161,648,173	-	161,647,173	161,648,309	+
GRMZM2G021406	XLOC_030774	enclosed	6	162,029,195	162,031,955	+	162,029,919	162,031,685	-
GRMZM2G179797	XLOC_030794	divergent	6	162,746,428	162,748,871	+	162,746,423	162,747,799	-
GRMZM2G037998	XLOC_030801	divergent	6	163,081,648	163,083,193	+	163,081,613	163,083,105	-
GRMZM2G038284	GRMZM2G038183	convergent	6	164,572,072	164,575,526	-	164,567,128	164,575,478	+
GRMZM2G038284	XLOC_028982	enclosed	6	164,572,072	164,575,526	-	164,574,502	164,575,478	+
XLOC_028986	XLOC_030842	enclosed	6	164,757,065	164,759,753	+	164,757,491	164,758,850	-
GRMZM2G021549	XLOC_028987	enclosed	6	164,760,362	164,767,127	-	164,760,526	164,762,818	+
GRMZM2G021470	GRMZM2G021549	divergent	6	164,766,609	164,768,517	+	164,760,362	164,767,127	-
GRMZM2G048892	XLOC_030858	divergent	6	165,444,350	165,445,593	+	165,443,968	165,445,433	-
GRMZM2G062738	XLOC_029025	enclosed	6	166,141,755	166,146,367	-	166,141,205	166,146,953	+
GRMZM2G163809	XLOC_029045	enclosed	6	167,024,418	167,028,053	-	167,024,418	167,025,382	+
GRMZM2G159028	XLOC_030895	enclosed	6	167,082,887	167,085,534	+	167,084,398	167,084,671	-
GRMZM2G070659	XLOC_030910	enclosed	6	167,774,318	167,777,115	+	167,776,365	167,776,826	-
GRMZM2G156905	XLOC_029066	enclosed	6	168,167,423	168,170,200	-	168,168,646	168,169,311	+
GRMZM2G141818	GRMZM2G141925	convergent	6	168,642,899	168,650,480	-	168,642,324	168,643,842	+
GRMZM2G095025	XLOC_029073	divergent	6	168,897,062	168,898,823	-	168,897,219	168,898,900	+
GRMZM5G833747	GRMZM2G127426	convergent	6	169,124,326	169,127,492	+	169,125,962	169,128,127	-
GRMZM5G894233	GRMZM2G424147	enclosed	7	272,380	276,643	-	272,954	273,522	+
GRMZM2G020281	XLOC_032540	enclosed	7	955,586	961,591	+	955,649	956,597	-
GRMZM2G135739	GRMZM2G135808	convergent	7	1,233,056	1,236,779	-	1,225,533	1,233,199	+
GRMZM2G420743	GRMZM2G420758	divergent	7	1,270,762	1,272,128	-	1,270,943	1,272,637	+
GRMZM2G420733	XLOC_030952	divergent	7	1,272,770	1,273,988	-	1,272,958	1,274,081	+
GRMZM2G555108	GRMZM2G120563	convergent	7	1,304,879	1,310,538	+	1,309,967	1,311,875	-
AC205122.4_FG003	XLOC_032566	enclosed	7	2,137,542	2,139,233	+	2,137,597	2,138,901	-
GRMZM2G458082	XLOC_030965	divergent	7	2,296,054	2,297,237	-	2,296,558	2,297,438	+
GRMZM2G456997	XLOC_032592	enclosed	7	3,252,351	3,253,259	+	3,252,792	3,253,074	-
AC205320.3_FG004	GRMZM2G026614	enclosed	7	6,073,356	6,075,719	+	6,073,362	6,074,529	-
GRMZM2G406014	XLOC_032635	enclosed	7	6,922,475	6,924,247	+	6,922,617	6,923,867	-
GRMZM2G132568	XLOC_031066	enclosed	7	9,547,566	9,551,755	-	9,551,283	9,551,687	+
GRMZM2G030138	GRMZM2G328171	convergent	7	10,578,360	10,580,594	+	10,578,984	10,581,443	-
XLOC_031079	XLOC_032682	divergent	7	10,656,525	10,658,447	+	10,656,435	10,657,883	-
GRMZM2G162382	XLOC_031080	enclosed	7	10,699,699	10,703,137	-	10,699,818	10,700,185	+
GRMZM2G401308	XLOC_031090	enclosed	7	11,446,449	11,447,690	-	11,446,548	11,447,642	+
XLOC_032699	XLOC_031109	convergent	7	13,208,213	13,213,879	-	13,208,122	13,208,779	+
GRMZM2G458494	XLOC_031116	enclosed	7	13,623,866	13,626,771	-	13,624,455	13,625,156	+
GRMZM2G028129	XLOC_031133	enclosed	7	15,013,266	15,015,247	-	15,013,395	15,014,207	+
GRMZM2G424832	XLOC_031162	enclosed	7	18,620,430	18,626,288	-	18,621,004	18,621,402	+
GRMZM2G340656	XLOC_032747	enclosed	7	19,063,721	19,067,235	+	19,063,747	19,066,238	-
XLOC_032757	XLOC_031169	enclosed	7	19,636,888	19,642,833	-	19,641,007	19,641,568	+
GRMZM5G892627	XLOC_031193	enclosed	7	23,364,784	23,366,705	-	23,365,115	23,366,572	+
XLOC_032804	XLOC_031212	divergent	7	25,552,619	25,553,236	-	25,552,651	25,553,257	+
GRMZM2G303829	XLOC_032807	enclosed	7	25,819,517	25,820,504	+	25,819,517	25,820,377	-
GRMZM2G000221	XLOC_032835	divergent	7	31,098,634	31,099,387	+	31,098,451	31,099,106	-
GRMZM2G104231	XLOC_032836	divergent	7	31,781,244	31,783,659	+	31,780,328	31,782,023	-
GRMZM2G140394	XLOC_031289	enclosed	7	38,403,781	38,408,314	-	38,407,722	38,408,007	+
GRMZM2G014914	XLOC_031307	enclosed	7	41,435,269	41,438,511	-	41,437,732	41,438,446	+
GRMZM5G893913	XLOC_032898	enclosed	7	46,202,870	46,207,496	+	46,205,325	46,207,465	-
GRMZM2G061562	XLOC_032918	enclosed	7	50,166,355	50,172,435	+	50,170,951	50,172,266	-
GRMZM2G050550	XLOC_032924	divergent	7	51,486,971	51,489,900	+	51,486,818	51,488,870	-
GRMZM2G048257	XLOC_031361	enclosed	7	51,667,538	51,669,099	-	51,667,897	51,668,499	+
GRMZM2G027198	XLOC_032933	enclosed	7	52,269,164	52,270,135	+	52,269,319	52,269,859	-
GRMZM2G318299	GRMZM2G318292	convergent	7	54,345,908	54,347,701	+	54,345,911	54,348,241	-
GRMZM2G092137	XLOC_033037	enclosed	7	83,788,520	83,791,400	+	83,790,397	83,790,735	-
GRMZM2G117878	XLOC_033047	enclosed	7	85,288,907	85,290,654	+	85,288,917	85,290,497	-
GRMZM2G047592	XLOC_031479	convergent	7	89,458,280	89,461,662	-	89,456,998	89,459,759	+
GRMZM2G162396	XLOC_033120	enclosed	7	99,233,022	99,234,662	+	99,233,077	99,234,124	-
GRMZM2G016660	GRMZM2G016551	convergent	7	101,604,486	101,613,981	-	101,603,002	101,604,542	+
GRMZM2G036916	XLOC_031561	divergent	7	103,534,410	103,539,199	-	103,538,461	103,539,271	+
GRMZM2G032160	XLOC_031565	enclosed	7	104,201,951	104,204,914	-	104,202,160	104,204,790	+
GRMZM2G019183	XLOC_033183	enclosed	7	107,954,326	107,958,837	+	107,955,577	107,958,837	-
XLOC_033185	XLOC_031588	divergent	7	108,330,462	108,332,092	-	108,330,559	108,332,566	+
XLOC_033186	XLOC_031590	enclosed	7	108,414,550	108,416,494	-	108,414,615	108,416,397	+
GRMZM2G126507	XLOC_031594	enclosed	7	108,871,160	108,874,537	-	108,873,979	108,874,199	+
GRMZM2G092525	XLOC_031596	divergent	7	109,353,827	109,357,542	-	109,356,210	109,357,565	+
GRMZM2G121200	GRMZM2G121189	convergent	7	110,085,353	110,088,614	-	110,084,378	110,085,727	+
GRMZM2G159221	GRMZM2G159160	divergent	7	111,685,454	111,688,840	-	111,688,572	111,690,233	+
GRMZM2G129018	XLOC_031629	divergent	7	115,719,658	115,722,055	-	115,719,873	115,722,227	+
XLOC_033227	GRMZM2G125853	divergent	7	115,996,532	115,997,272	-	115,996,767	116,010,834	+
GRMZM2G142918	XLOC_033238	enclosed	7	117,727,876	117,730,526	+	117,728,776	117,730,447	-
GRMZM2G071959	XLOC_033257	divergent	7	119,943,201	119,944,169	+	119,942,997	119,943,754	-
XLOC_031663	XLOC_033259	enclosed	7	119,983,090	119,984,190	+	119,983,118	119,983,535	-
GRMZM2G135763	XLOC_033268	enclosed	7	121,285,089	121,289,636	+	121,285,598	121,287,999	-
XLOC_031687	XLOC_033273	enclosed	7	121,804,250	121,804,694	+	121,804,250	121,804,951	-
GRMZM2G004207	XLOC_033274	divergent	7	121,850,282	121,852,444	+	121,850,262	121,851,370	-
GRMZM2G024865	XLOC_031723	enclosed	7	124,779,178	124,781,786	-	124,780,337	124,781,632	+
GRMZM2G416632	GRMZM2G416625	convergent	7	128,406,301	128,408,544	+	128,407,526	128,412,102	-
GRMZM2G082653	XLOC_031757	divergent	7	128,679,183	128,680,735	-	128,679,213	128,681,250	+
GRMZM2G096952	XLOC_033326	divergent	7	129,753,381	129,758,428	+	129,753,354	129,754,232	-
GRMZM2G055469	XLOC_033368	divergent	7	132,423,389	132,426,153	+	132,421,650	132,424,591	-
GRMZM2G176430	XLOC_033369	enclosed	7	132,548,737	132,551,662	+	132,548,900	132,549,402	-
GRMZM2G083932	XLOC_033371	divergent	7	132,660,472	132,661,613	+	132,660,271	132,661,577	-
GRMZM2G083894	XLOC_033373	enclosed	7	132,664,469	132,666,026	+	132,664,910	132,665,906	-
GRMZM2G085467	XLOC_033379	divergent	7	132,922,501	132,925,523	+	132,922,387	132,924,053	-
GRMZM2G168364	XLOC_033407	enclosed	7	134,168,481	134,170,279	+	134,168,847	134,169,418	-
GRMZM2G131281	XLOC_031833	divergent	7	135,625,837	135,626,999	-	135,625,966	135,627,055	+
GRMZM2G081048	GRMZM2G080871	convergent	7	139,144,164	139,155,145	+	139,152,738	139,156,303	-
GRMZM2G132486	XLOC_031883	enclosed	7	140,268,676	140,271,906	-	140,268,784	140,269,784	+
GRMZM2G071147	XLOC_033463	enclosed	7	140,455,266	140,461,976	+	140,460,238	140,461,566	-
GRMZM2G149520	XLOC_031896	enclosed	7	140,847,928	140,853,032	-	140,848,023	140,848,380	+
GRMZM2G017013	XLOC_031903	enclosed	7	141,612,374	141,615,097	-	141,612,979	141,613,942	+
GRMZM2G017013	XLOC_031904	enclosed	7	141,612,374	141,615,097	-	141,614,458	141,614,818	+
GRMZM2G001639	XLOC_033478	enclosed	7	141,630,856	141,633,508	+	141,631,174	141,632,009	-
GRMZM2G073228	XLOC_031906	enclosed	7	141,674,424	141,678,723	-	141,675,214	141,678,290	+
GRMZM2G073504	GRMZM2G073377	convergent	7	141,681,145	141,690,742	-	141,680,690	141,681,890	+
GRMZM2G052610	GRMZM2G052509	convergent	7	141,917,927	141,921,808	+	141,921,207	141,924,166	-
GRMZM2G165272	GRMZM2G465685	enclosed	7	142,270,553	142,272,208	+	142,270,740	142,271,931	-
GRMZM2G104551	XLOC_031931	enclosed	7	143,239,536	143,250,307	-	143,244,178	143,246,692	+

XLOC_031935	GRMZM2G335111	convergent	7	143,286,301	143,288,804	+	143,288,306	143,289,828	-
GRMZM2G091586	XLOC_033518	enclosed	7	143,551,214	143,557,231	+	143,551,214	143,551,708	-
GRMZM2G006117	XLOC_031968	enclosed	7	145,808,406	145,812,881	-	145,809,169	145,809,829	+
GRMZM2G075719	XLOC_033617	enclosed	7	150,455,168	150,458,991	+	150,455,264	150,455,567	-
GRMZM2G038449	XLOC_033625	divergent	7	150,906,444	150,909,453	+	150,906,366	150,906,852	-
GRMZM2G095261	XLOC_033631	divergent	7	151,123,095	151,125,335	+	151,122,730	151,124,888	-
GRMZM2G155911	XLOC_033635	enclosed	7	151,226,660	151,228,687	+	151,226,861	151,228,001	-
GRMZM2G010298	XLOC_032048	enclosed	7	151,545,591	151,555,845	-	151,554,826	151,555,375	+
GRMZM2G325783	XLOC_032049	enclosed	7	151,560,059	151,564,762	-	151,560,171	151,562,048	+
GRMZM2G325008	XLOC_033649	divergent	7	152,431,465	152,438,328	+	152,431,276	152,433,930	-
GRMZM2G108849	XLOC_033651	enclosed	7	152,808,921	152,813,134	+	152,809,230	152,810,705	-
GRMZM2G449709	XLOC_032067	enclosed	7	153,211,543	153,213,159	-	153,211,728	153,212,562	+
GRMZM2G429396	XLOC_033662	divergent	7	153,330,905	153,331,987	+	153,330,572	153,331,553	-
GRMZM2G167591	XLOC_033672	enclosed	7	153,692,958	153,695,938	+	153,695,447	153,695,725	-
GRMZM2G357631	GRMZM2G357636	enclosed	7	154,224,100	154,225,746	-	154,221,509	154,226,616	+
GRMZM2G058310	GRMZM2G358467	enclosed	7	155,396,590	155,399,705	+	155,396,211	155,401,842	-
GRMZM5G889999	XLOC_032125	enclosed	7	156,219,834	156,225,721	-	156,224,860	156,225,621	+
GRMZM2G060993	XLOC_032140	enclosed	7	157,049,353	157,051,304	-	157,049,914	157,050,398	+
GRMZM5G801949	XLOC_032147	enclosed	7	157,477,093	157,479,679	-	157,477,186	157,478,848	+
XLOC_032159	GRMZM5G897592	enclosed	7	158,405,841	158,406,153	+	158,405,601	158,408,516	-
GRMZM2G106548	GRMZM5G897592	divergent	7	158,406,545	158,409,322	+	158,405,601	158,408,516	-
XLOC_033767	XLOC_032175	enclosed	7	159,268,927	159,276,192	-	159,270,024	159,272,786	+
XLOC_033774	GRMZM2G019084	divergent	7	159,570,727	159,573,245	-	159,571,286	159,575,960	+
GRMZM5G889052	XLOC_033789	enclosed	7	160,189,515	160,193,189	+	160,191,526	160,192,363	-
GRMZM5G889052	XLOC_033788	enclosed	7	160,189,515	160,193,189	+	160,190,641	160,190,959	-
XLOC_033790	XLOC_032196	enclosed	7	160,254,346	160,255,890	-	160,254,909	160,255,804	+
GRMZM2G302604	XLOC_033801	divergent	7	160,854,635	160,856,784	+	160,854,309	160,856,519	-
XLOC_033803	XLOC_032209	enclosed	7	161,158,692	161,160,676	-	161,159,057	161,160,508	+
GRMZM2G023444	XLOC_033806	enclosed	7	161,376,313	161,379,215	+	161,376,439	161,376,899	-
GRMZM2G023346	XLOC_033807	enclosed	7	161,386,057	161,389,217	+	161,386,211	161,388,685	-
GRMZM2G172657	XLOC_033820	divergent	7	161,941,447	161,944,260	+	161,941,387	161,943,936	-
GRMZM2G057247	XLOC_033828	enclosed	7	162,108,103	162,114,970	+	162,108,890	162,109,828	-
GRMZM2G057247	XLOC_033829	enclosed	7	162,108,103	162,114,970	+	162,113,500	162,114,498	-
GRMZM2G113696	XLOC_032238	enclosed	7	163,042,057	163,045,208	-	163,042,101	163,042,441	+
GRMZM2G080549	XLOC_032287	enclosed	7	165,382,432	165,389,417	-	165,383,657	165,384,304	+
GRMZM2G089736	XLOC_033883	enclosed	7	165,496,269	165,498,636	+	165,496,321	165,497,031	-
GRMZM2G079397	GRMZM2G079226	divergent	7	166,191,360	166,197,002	+	166,186,897	166,191,764	-
GRMZM2G056732	XLOC_033901	enclosed	7	166,410,307	166,416,117	+	166,410,567	166,414,219	-
GRMZM2G117707	XLOC_032314	enclosed	7	166,502,692	166,506,595	-	166,504,385	166,505,811	+
GRMZM2G017145	AC195864.3_FG017	convergent	7	167,541,463	167,544,286	-	167,539,708	167,541,966	+
GRMZM2G141322	XLOC_033929	enclosed	7	167,674,297	167,675,623	+	167,674,835	167,675,315	-
GRMZM2G150683	GRMZM2G150758	convergent	7	167,947,896	167,955,945	-	167,946,724	167,948,218	+
GRMZM2G318843	XLOC_033938	enclosed	7	168,019,762	168,022,899	+	168,021,383	168,022,711	-
GRMZM2G477325	XLOC_033944	enclosed	7	168,441,397	168,442,596	+	168,441,795	168,442,543	-
GRMZM2G301934	XLOC_032383	enclosed	7	169,977,821	169,979,383	-	169,977,981	169,978,650	+
GRMZM2G356894	GRMZM2G055607	convergent	7	170,074,356	170,080,041	-	170,071,170	170,074,488	+
GRMZM2G433767	XLOC_032403	enclosed	7	170,696,934	170,699,642	-	170,697,273	170,697,504	+
GRMZM2G037189	XLOC_032421	enclosed	7	171,577,707	171,578,957	-	171,577,972	171,578,887	+
GRMZM2G025885	XLOC_034006	enclosed	7	171,769,812	171,771,300	+	171,769,839	171,771,033	-
GRMZM2G025992	XLOC_032429	enclosed	7	171,775,066	171,778,176	-	171,776,631	171,778,079	+
GRMZM2G414423	XLOC_034009	enclosed	7	171,863,190	171,865,343	+	171,863,280	171,865,189	-
GRMZM2G020766	XLOC_032437	enclosed	7	172,301,215	172,312,681	-	172,301,623	172,302,947	+
GRMZM2G414460	XLOC_032447	enclosed	7	172,543,068	172,547,171	-	172,543,187	172,544,780	+
GRMZM2G025441	XLOC_034039	enclosed	7	172,793,831	172,796,061	+	172,794,845	172,795,897	-
GRMZM2G427815	XLOC_034040	enclosed	7	172,809,428	172,811,102	+	172,810,282	172,810,699	-
GRMZM2G166738	XLOC_032461	enclosed	7	172,977,273	172,981,385	-	172,980,469	172,981,129	+
GRMZM2G311665	XLOC_032475	enclosed	7	173,486,169	173,490,497	-	173,490,053	173,490,328	+
GRMZM2G430849	XLOC_032477	enclosed	7	173,556,079	173,558,804	-	173,556,354	173,557,829	+
GRMZM2G469380	XLOC_032478	enclosed	7	173,595,180	173,596,955	-	173,596,426	173,596,757	+
GRMZM2G056772	XLOC_034093	divergent	7	174,614,466	174,615,537	+	174,614,465	174,615,416	-
GRMZM2G056569	XLOC_032498	enclosed	7	174,627,232	174,631,734	-	174,630,036	174,630,579	+
GRMZM2G134284	GRMZM2G134295	divergent	7	174,834,523	174,847,147	+	174,832,020	174,835,057	-
GRMZM2G032711	GRMZM2G032376	divergent	7	175,605,863	175,613,266	-	175,611,974	175,615,934	+
GRMZM2G000818	XLOC_034115	convergent	7	176,182,074	176,183,077	+	176,182,240	176,183,508	-
XLOC_032527	XLOC_034118	divergent	7	176,436,743	176,439,281	+	176,436,043	176,438,830	-
GRMZM2G085111	XLOC_035987	enclosed	8	400,460	407,184	+	401,337	404,870	-
GRMZM2G386987	XLOC_035994	enclosed	8	704,959	705,716	+	705,086	705,419	-
GRMZM2G063223	XLOC_034134	enclosed	8	1,145,660	1,147,184	-	1,146,664	1,147,130	+
GRMZM2G589579	XLOC_036018	enclosed	8	2,511,344	2,518,808	+	2,511,400	2,512,210	-
GRMZM2G019515	XLOC_034151	enclosed	8	2,901,206	2,904,218	-	2,903,301	2,903,831	+
GRMZM2G050325	XLOC_036031	enclosed	8	3,921,958	3,925,143	+	3,921,958	3,923,002	-
GRMZM2G134941	XLOC_036046	enclosed	8	4,969,642	4,970,267	+	4,969,933	4,970,262	-
GRMZM2G058404	XLOC_034207	divergent	8	6,359,079	6,360,548	-	6,359,315	6,360,734	+
GRMZM2G110504	XLOC_036063	enclosed	8	6,920,014	6,921,235	+	6,920,162	6,920,992	-
AC216871.3_FG004	XLOC_034265	enclosed	8	10,013,056	10,014,230	-	10,013,125	10,013,839	+
GRMZM2G096358	XLOC_034286	divergent	8	11,486,313	11,488,153	-	11,486,497	11,488,203	+
GRMZM2G095964	XLOC_036123	enclosed	8	11,922,956	11,924,511	+	11,923,225	11,923,580	-
GRMZM2G119361	XLOC_034316	enclosed	8	13,371,529	13,374,745	-	13,374,182	13,374,574	+
GRMZM2G079458	XLOC_034324	enclosed	8	13,801,218	13,804,482	-	13,802,079	13,802,995	+
GRMZM2G110063	XLOC_036160	enclosed	8	14,730,093	14,780,789	+	14,749,038	14,750,512	-
GRMZM2G139600	XLOC_036161	enclosed	8	14,796,888	14,799,245	+	14,797,419	14,799,093	-
AC217965.2_FG012	XLOC_034340	enclosed	8	15,108,533	15,109,664	-	15,108,641	15,109,157	+
GRMZM2G083328	XLOC_034347	convergent	8	15,987,340	15,989,474	-	15,987,336	15,988,847	+
GRMZM2G081848	XLOC_034349	enclosed	8	16,431,439	16,433,808	-	16,433,026	16,433,339	+
GRMZM2G095595	XLOC_036196	enclosed	8	17,177,806	17,182,719	+	17,178,910	17,179,303	-
GRMZM2G375159	XLOC_034371	enclosed	8	18,444,281	18,446,241	-	18,444,771	18,445,411	+
GRMZM2G032190	XLOC_036220	enclosed	8	18,612,350	18,616,112	+	18,612,463	18,613,061	-
GRMZM2G080375	GRMZM2G080588	convergent	8	20,703,355	20,706,994	-	20,696,323	20,703,544	+
GRMZM2G004182	GRMZM2G004083	convergent	8	22,175,678	22,180,874	+	22,180,368	22,182,467	-
GRMZM2G472625	GRMZM2G472654	convergent	8	23,427,704	23,432,837	-	23,427,241	23,428,339	+
GRMZM2G099483	XLOC_036278	divergent	8	23,892,754	23,895,063	+	23,892,459	23,894,829	-
GRMZM2G047139	XLOC_036289	divergent	8	24,724,776	24,728,219	+	24,724,760	24,726,896	-
GRMZM2G369182	XLOC_036300	divergent	8	25,881,226	25,882,741	+	25,880,908	25,881,985	-
GRMZM2G413717	XLOC_036307	enclosed	8	26,635,509	26,636,651	+	26,635,609	26,636,105	-
GRMZM2G127687	XLOC_034457	enclosed	8	26,863,089	26,867,549	-	26,865,699	26,866,324	+
GRMZM2G010596	XLOC_034461	convergent	8	27,218,394	27,221,078	-	27,218,363	27,218,894	+
GRMZM2G014580	XLOC_036317	divergent	8	27,670,562	27,672,733	+	27,670,252	27,672,389	-
GRMZM5G842965	XLOC_034509	enclosed	8	36,144,983	36,147,538	-	36,145,184	36,147,155	+
GRMZM2G061088	XLOC_034519	enclosed	8	37,813,042	37,816,875	-	37,813,413	37,813,825	+
GRMZM2G035395	GRMZM2G035542	convergent	8	43,074,144	43,078,508	+	43,077,873	43,086,649	-
AC205274.3_FG001	XLOC_034562	enclosed	8	51,816,339	51,817,463	-	51,816,725	51,817,436	+
XLOC_034566	XLOC_036426	divergent	8	52,818,656	52,820,059	+	52,817,772	52,819,178	-
GRMZM6G514393	XLOC_036436	divergent	8	55,520,438	55,522,327	+	55,520,183	55,522,160	-
GRMZM2G339206	XLOC_034583	enclosed	8	55,843,374	55,846,170	-	55,843,293	55,846,303	+
GRMZM2G159592	XLOC_036481	divergent	8	64,222,114	64,223,758	+	64,222,075	64,223,671	-
GRMZM2G345039	GRMZM2G043764	convergent	8	67,245,670	67,250,897	-	67,239,332	67,245,829	+
GRMZM2G001514	XLOC_036518	enclosed	8	69,107,919	69,110,163	+	69,108,973	69,109,970	-
XLOC_034669	XLOC_036521	enclosed	8	69,273,412	69,276,762	+	69,273,990	69,274,639	-
GRMZM2G031827	XLOC_036522	enclosed	8	69,651,928	69,660,583	+	69,659,485	69,660,454	-
GRMZM2G023872	XLOC_036544	enclosed	8	72,494,681	72,496,570	+	72,494,703	72,496,238	-
GRMZM2G001991	GRMZM2G002034	convergent	8	73,364,861	73,370,694	+	73,369,546	73,372,613	-
GRMZM2G097768	XLOC_034703	enclosed	8	73,418,363	73,424,793	-	73,423,869	73,424,293	+
GRMZM2G045664	XLOC_034712	enclosed	8	74,456,610	74,457,885	-	74,456,822	74,457,598	+
GRMZM2G058305	GRMZM2G058456	convergent	8	75,118,942	75,123,679	-	75,116,592	75,119,705	+
GRMZM2G128995	XLOC_036576	enclosed	8	77,264,067	77,274,209	+	77,271,479	77,272,668	-
GRMZM5G892645	XLOC_036584	enclosed	8	78,161,466	78,166,379	+	78,163,924	78,165,052	-
GRMZM2G456000	XLOC_034744	convergent	8	78,524,069	78,528,819	-	78,522,738	78,524,404	+
GRMZM2G086964	XLOC_034750	enclosed	8	78,942,781	78,951,473	-	78,951,149	78,951,442	+
GRMZM2G128682	XLOC_036620	enclosed	8	86,105,887	86,107,965	+	86,106,557	86,107,464	-
GRMZM2G306851	GRMZM2G007404	convergent	8	86,549,083	86,555,756	+	86,555,339	86,559,750	-
GRMZM2G023755	XLOC_036675	convergent	8	93,946,368	93,949,244	+	93,948,001	93,950,694	-
GRMZM5G898867	XLOC_036676	enclosed	8	94,085,437	94,089,730	+	94,086,238	94,086,551	-
GRMZM2G358606	XLOC_034847	divergent	8	94,309,687	94,310,894	-	94,310,000	94,311,063	+
GRMZM2G094017	XLOC_034863	enclosed	8	96,330,315	96,332,969	-	96,331,102	96,331,558	+
GRMZM2G094017	XLOC_034864	divergent	8	96,330,315	96,332,969	-	96,331,887	96,333,277	+
GRMZM2G107721	XLOC_034876	enclosed	8	97,451,652	97,453,287	-	97,452,226	97,452,612	+
GRMZM2G016605	XLOC_036784	divergent	8	106,153,653	106,156,495	+	106,153,026	106,155,085	-
GRMZM2G116238	XLOC_034971	enclosed	8	109,359,888	109,361,490	-	109,361,001	109,361,451	+
GRMZM2G449681	XLOC_034976	enclosed	8	109,739,873	109,742,256	-	109,740,144	109,742,205	+
GRMZM2G029756	XLOC_035009	enclosed	8	113,237,540	113,242,322	-	113,240,802	113,241,236	+
XLOC_035059	XLOC_036878	enclosed	8	118,087,730	118,088,907	+	118,087,800	118,088,310	-
XLOC_035077	GRMZM2G138817	convergent	8	118,795,173	118,797,211	+	118,795,818	118,797,252	-
GRMZM2G300589	XLOC_035078	enclosed	8	119,169,594	119,172,752	-	119,170,288	119,170,988	+
GRMZM2G073347	XLOC_035081	divergent	8	119,461,575	119,462,915	-	119,461,775	119,462,949	+
GRMZM2G062555	XLOC_035083	enclosed	8	119,542,597	119,544,810	-	119,544,238	119,544,607	+
GRMZM2G025459	XLOC_035084	enclosed	8	119,745,262	119,748,192	-	119,745,323	119,747,929	+
XLOC_035097	XLOC_036937	divergent	8	121,416,903	121,418,458	+	121,416,852	121,418,183	-
XLOC_035098	XLOC_036937	divergent	8	121,416,903	121,425,056	+	121,416,852	121,418,183	-
GRMZM2G048200	XLOC_035147	enclosed	8	125,000,004	125,004,076	-	125,003,196	125,004,056	+
GRMZM2G048200	GRMZM2G048117	convergent	8	125,000,004	125,004,076	-	124,996,332	125,001,496	+
GRMZM2G150367	GRMZM2G150434	convergent	8	126,229,305	126,232,019	+	126,231,541	126,232,916	-
XLOC_037004	GRMZM2G700655	enclosed	8	128,078,312	128,078,825	-	128,075,569	128,081,851	+
XLOC_037037	XLOC_035212	convergent	8	131,058,700	131,060,803	-	131,052,957	131,059,699	+
AC214648.3_FG003	XLOC_035215	enclosed	8	131,331,979	131,336,740	-	131,336,046	131,336,543	+
GRMZM2G700665	XLOC_037044	enclosed	8	131,576,700	131,580,312	+	131,578,426	131,579,537	-
GRMZM2G474726	GRMZM2G173763	convergent	8	131,727,186	131,733,880	+	131,733,467	131,738,126	-
GRMZM2G053991	GRMZM2G353250	enclosed	8	131,904,461	131,905,991	+	131,904,201	131,905,991	-
GRMZM2G080191	XLOC_035227	divergent	8	132,540,044	132,541,658	-	132,540,497	132,541,980	+
GRMZM2G386228	XLOC_037056	enclosed	8	132,972,943	132,974,324	+	132,973,314	132,973,657	-
XLOC_035237	XLOC_037059	enclosed	8	133,091,428	133,093,951	+	133,091,531	133,091,829	-
GRMZM2G028986	XLOC_035246	divergent	8	133,998,505	134,000,685	-	133,999,615	134,000,856	+
GRMZM2G470513	GRMZM2G168590	convergent	8	134,376,621	134,379,299	+	134,379,238	134,379,777	-
GRMZM2G162525	XLOC_037080	enclosed	8	134,443,644	134,444,960	+	134,444,295	134,444,960	-
GRMZM2G456835	XLOC_037089	enclosed	8	135,307,939	135,312,198	+	135,310,261	135,312,197	-
GRMZM2G088132	XLOC_035273	enclosed	8	135,584,864	135,586,005	-	135,585,278	135,585,576	+
AC207342.3_FG008	XLOC_037094	enclosed	8	135,947,268	135,949,742	+	135,947,857	135,949,508	-
GRMZM2G458650	XLOC_037103	divergent	8	136,652,681	136,655,650	+	136,652,554	136,652,953	-
GRMZM5G803433	GRMZM2G117742	convergent	8	142,537,765	142,543,929	-	142,535,900	142,538,931	+
GRMZM2G050270	XLOC_035355	convergent	8	142,813,533	142,817,608	-	142,811,980	142,813,848	+
GRMZM2G074735	XLOC_037179	enclosed	8	144,697,840	144,699,628	+	144,697,907	144,698,440	-
GRMZM2G149683	XLOC_035377	enclosed	8	144,765,266	144,766,964	-	144,765,497	144,765,969	+
GRMZM2G021436	XLOC_037185	enclosed	8	145,218,849	145,221,114	+	145,219,676	145,220,486	-
GRMZM2G179693	GRMZM2G179696	convergent	8	145,719,688	145,726,829	-	145,718,291	145,722,336	+
GRMZM2G063672	XLOC_037201	enclosed	8	146,150,403	146,153,728	+	146,151,084	146,151,525	-
GRMZM2G086088	XLOC_035431	enclosed	8	147,738,191	147,741,508	-	147,740,511	147,740,974	+
GRMZM2G144028	GRMZM2G144021	convergent	8	151,441,785	151,450,139	-	151,438,263	151,442,577	+
GRMZM2G144028	XLOC_035491	enclosed	8	151,441,785	151,450,139	-	151,442,987	151,444,263	+
GRMZM2G092018	GRMZM2G091973	convergent	8	151,669,118	151,680,781	-	151,667,315	151,669,713	+
GRMZM5G843389	XLOC_035495	enclosed	8	151,798,812	151,803,317	-	151,802,508	151,802,839	+
XLOC_035503	XLOC_037286	divergent	8	152,285,732	152,293,012	+	152,285,587	152,287,295	-
GRMZM2G439350	XLOC_037288	enclosed	8	152,497,093	152,498,604	+	152,497,944	152,498,597	-
XLOC_037303	XLOC_035526	enclosed	8	153,934,934	153,937,499	-	153,934,973	153,936,891	+
GRMZM2G107985	XLOC_035530	divergent	8	154,553,618	154,562,574	-	154,562,135	154,562,609	+
GRMZM2G069203	XLOC_037324	enclosed	8	155,020,506	155,021,940	+	155,020,627	155,021,908	-
GRMZM2G151252	GRMZM2G151245	convergent	8	155,184,005	155,186,489	-	155,182,657	155,184,839	+
GRMZM2G367701	XLOC_035566	enclosed	8	156,268,171	156,269,856	-	156,268,512	156,269,644	+
GRMZM2G167758	XLOC_037363	enclosed	8	156,423,920	156,425,734	+	156,425,476	156,425,725	-
GRMZM2G446960	GRMZM2G147709	convergent	8	156,777,102	156,780,465	-	156,774,170	156,777,892	+
GRMZM2G010065	GRMZM2G010176	convergent	8	159,534,577	159,539,292	-	159,531,387	159,535,110	+
GRMZM2G134162	GRMZM2G433959	convergent	8	159,952,116	159,957,434	+	159,956,854	159,957,921	-
GRMZM2G170969	XLOC_035676	enclosed	8	162,305,487	162,306,280	-	162,305,650	162,306,050	+
GRMZM2G470942	XLOC_037473	convergent	8	162,335,276	162,337,118	+	162,335,899	162,340,140	-
GRMZM2G175406	XLOC_037480	enclosed	8	162,588,168	162,589,986	+	162,588,472	162,589,576	-
GRMZM2G100318	XLOC_037489	enclosed	8	163,128,903	163,133,067	+	163,131,948	163,132,447	-
XLOC_037490	XLOC_035697	enclosed	8	163,203,210	163,204,952	-	163,203,559	163,204,199	+
GRMZM2G024799	XLOC_035716	enclosed	8	163,925,848	163,929,685	-	163,927,153	163,928,646	+
GRMZM2G171996	XLOC_035726	divergent	8	164,285,437	164,286,435	-	164,285,571	164,286,601	+
GRMZM2G068361	XLOC_037516	enclosed	8	164,450,478	164,452,474	+	164,450,999	164,451,287	-
GRMZM2G092817	XLOC_037525	enclosed	8	164,822,797	164,826,113	+	164,824,872	164,825,883	-
GRMZM2G119397	XLOC_035742	enclosed	8	164,952,357	164,956,271	-	164,948,306	164,956,406	+
GRMZM2G143640	XLOC_035766	enclosed	8	166,202,041	166,203,641	-	166,202,461	166,203,023	+
GRMZM2G143640	XLOC_035767	divergent	8	166,202,041	166,203,641	-	166,203,213	166,204,219	+
AC232238.2_FG004	GRMZM2G168087	convergent	8	166,341,329	166,349,660	+	166,345,530	166,354,968	-
AC232238.2_FG004	XLOC_037558	enclosed	8	166,341,329	166,349,660	+	166,345,444	166,349,060	-
GRMZM2G396248	XLOC_037564	enclosed	8	166,450,330	166,453,602	+	166,450,439	166,452,505	-
GRMZM2G123394	XLOC_037576	enclosed	8	166,704,257	166,706,406	+	166,705,309	166,706,025	-
GRMZM2G147544	XLOC_035780	divergent	8	166,741,560	166,743,934	-	166,742,338	166,743,981	+
GRMZM2G165177	XLOC_037608	enclosed	8	168,042,162	168,044,733	+	168,042,162	168,044,284	-
GRMZM2G112912	XLOC_037618	enclosed	8	168,604,741	168,605,617	+	168,604,901	168,605,361	-
GRMZM2G112836	XLOC_037620	divergent	8	168,615,332	168,618,760	+	168,614,934	168,616,983	-
XLOC_037628	XLOC_035823	enclosed	8	168,809,267	168,815,005	-	168,814,382	168,814,995	+
GRMZM5G840487	XLOC_037635	enclosed	8	169,068,643	169,073,066	+	169,071,358	169,072,345	-
GRMZM2G066755	XLOC_037640	enclosed	8	169,175,095	169,178,292	+	169,175,204	169,175,643	-
GRMZM2G311220	XLOC_035833	enclosed	8	169,208,422	169,215,320	-	169,209,691	169,209,923	+
GRMZM2G036448	XLOC_037654	enclosed	8	169,675,588	169,681,153	+	169,676,228	169,679,455	-
AC218972.3_FG002	XLOC_035857	divergent	8	170,179,327	170,181,277	-	170,180,913	170,181,318	+
AC218972.3_FG004	XLOC_035858	enclosed	8	170,197,942	170,201,141	-	170,198,174	170,198,543	+
XLOC_037665	XLOC_035859	enclosed	8	170,218,080	170,221,722	-	170,218,672	170,221,170	+
GRMZM2G069078	XLOC_035863	enclosed	8	170,417,531	170,420,149	-	170,418,065	170,420,083	+
GRMZM2G076152	XLOC_037673	enclosed	8	170,514,020	170,517,247	+	170,516,288	170,516,967	-
GRMZM2G476009	XLOC_037681	enclosed	8	170,885,667	170,888,437	+	170,885,781	170,886,008	-
GRMZM2G139374	XLOC_037695	divergent	8	171,307,877	171,313,576	+	171,307,811	171,308,656	-
GRMZM5G852779	XLOC_035902	convergent	8	171,685,242	171,688,996	-	171,684,881	171,685,894	+
GRMZM2G116086	XLOC_037721	enclosed	8	172,112,785	172,121,960	+	172,113,702	172,114,108	-
GRMZM2G424873	XLOC_037736	enclosed	8	172,626,960	172,628,488	+	172,627,115	172,627,558	-
GRMZM2G302639	GRMZM2G002858	divergent	8	172,961,557	172,963,511	-	172,963,108	172,967,039	+
GRMZM2G428179	XLOC_035950	enclosed	8	173,488,303	173,489,765	-	173,488,595	173,488,860	+
GRMZM2G469502	GRMZM2G469593	convergent	9	6,950,322	6,961,103	-	6,948,280	6,961,033	+
GRMZM2G106303	XLOC_039370	divergent	9	7,393,694	7,395,273	+	7,393,341	7,394,579	-
GRMZM2G033413	XLOC_037868	enclosed	9	8,229,971	8,240,288	-	8,239,976	8,240,287	+
GRMZM5G864689	XLOC_039403	enclosed	9	9,510,044	9,513,407	+	9,512,594	9,512,978	-
XLOC_039408	XLOC_037884	divergent	9	9,955,287	9,956,481	-	9,955,397	9,956,538	+

GRMZM2G135501	XLOC_037888	divergent	9	10,946,567	10,947,842	-	10,947,383	10,948,025	+
GRMZM2G177231	XLOC_037898	divergent	9	11,715,793	11,720,000	-	11,718,330	11,720,005	+
XLOC_037912	GRMZM2G067053	enclosed	9	12,797,426	12,798,671	+	12,796,516	12,799,152	-
GRMZM2G079306	GRMZM2G081573	convergent	9	12,875,085	12,883,372	-	12,874,481	12,883,237	+
GRMZM2G453349	XLOC_039474	enclosed	9	13,358,509	13,364,979	+	13,363,266	13,364,514	-
GRMZM2G008305	XLOC_039478	enclosed	9	13,663,138	13,664,819	+	13,663,717	13,664,603	-
GRMZM2G009139	XLOC_039485	enclosed	9	14,119,204	14,121,439	+	14,119,262	14,120,618	-
GRMZM2G007683	XLOC_037946	divergent	9	14,917,204	14,925,544	-	14,925,130	14,925,915	+
XLOC_039504	XLOC_037949	divergent	9	15,374,868	15,386,749	-	15,385,595	15,386,863	+
XLOC_039505	XLOC_037949	enclosed	9	15,386,103	15,386,781	-	15,385,595	15,386,863	+
GRMZM2G398736	GRMZM5G843474	divergent	9	15,590,769	15,592,948	-	15,592,791	15,598,567	+
GRMZM5G831993	GRMZM5G865595	convergent	9	15,616,216	15,621,318	+	15,620,220	15,624,084	-
GRMZM2G005080	XLOC_039514	enclosed	9	16,001,623	16,002,878	+	16,001,752	16,002,137	-
GRMZM2G529313	GRMZM2G075488	convergent	9	16,689,343	16,701,067	-	16,685,283	16,689,432	+
GRMZM2G002227	XLOC_039525	divergent	9	16,906,529	16,909,767	+	16,906,526	16,907,818	-
AC231745.1_FG003	GRMZM5G838414	convergent	9	18,335,677	18,338,579	-	18,328,876	18,337,267	+
GRMZM2G103137	GRMZM2G020216	convergent	9	18,885,308	18,888,376	-	18,879,285	18,887,000	+
XLOC_038013	XLOC_039547	enclosed	9	18,958,599	18,959,447	+	18,958,583	18,962,887	-
GRMZM2G028594	XLOC_038017	enclosed	9	19,034,946	19,040,945	-	19,035,099	19,035,555	+
XLOC_039594	XLOC_038068	divergent	9	22,170,607	22,171,789	-	22,170,770	22,173,445	+
GRMZM2G404608	XLOC_039600	enclosed	9	23,220,892	23,222,189	+	23,221,405	23,221,809	-
XLOC_038085	GRMZM2G118690	convergent	9	23,764,778	23,765,960	+	23,765,379	23,771,219	-
GRMZM2G050166	GRMZM2G350319	divergent	9	24,486,952	24,491,050	+	24,481,779	24,487,390	-
GRMZM2G304528	XLOC_039625	divergent	9	24,558,570	24,560,564	+	24,558,416	24,559,236	-
GRMZM2G071491	GRMZM2G071602	convergent	9	25,260,929	25,270,126	+	25,268,948	25,272,830	-
GRMZM2G383540	GRMZM2G083711	convergent	9	25,535,087	25,537,349	-	25,533,695	25,535,338	+
GRMZM2G475293	GRMZM2G475360	convergent	9	26,833,575	26,834,967	+	26,833,946	26,839,205	-
XLOC_038172	XLOC_039692	divergent	9	32,305,175	32,308,115	+	32,305,077	32,306,671	-
XLOC_038172	XLOC_039693	divergent	9	32,305,175	32,308,115	+	32,305,077	32,307,246	-
XLOC_039694	XLOC_038173	convergent	9	32,308,239	32,309,434	-	32,308,200	32,308,972	+
GRMZM2G058675	XLOC_039707	enclosed	9	34,129,749	34,143,684	+	34,129,816	34,130,120	-
GRMZM2G027958	XLOC_038209	divergent	9	38,545,391	38,547,608	-	38,545,694	38,547,957	+
GRMZM2G099740	XLOC_038228	divergent	9	43,625,799	43,627,733	-	43,626,080	43,627,895	+
XLOC_038252	XLOC_039757	enclosed	9	46,856,491	46,857,697	+	46,856,634	46,857,418	-
GRMZM2G027860	XLOC_039774	enclosed	9	48,763,025	48,772,161	+	48,768,128	48,768,528	-
XLOC_039870	XLOC_038357	enclosed	9	69,385,549	69,386,024	-	69,385,542	69,386,024	+
XLOC_038379	XLOC_039882	enclosed	9	73,801,048	73,812,636	+	73,804,250	73,806,195	-
XLOC_038380	XLOC_039882	enclosed	9	73,803,691	73,812,636	+	73,804,250	73,806,195	-
GRMZM2G136300	XLOC_038391	enclosed	9	75,178,361	75,189,267	-	75,188,193	75,188,547	+
GRMZM2G080839	XLOC_039902	enclosed	9	78,160,268	78,162,284	+	78,160,997	78,162,100	-
GRMZM2G161472	XLOC_038410	enclosed	9	79,629,909	79,634,177	-	79,630,453	79,632,390	+
GRMZM2G022558	XLOC_039951	convergent	9	87,869,539	87,872,095	+	87,870,644	87,873,337	-
GRMZM2G159641	XLOC_038450	enclosed	9	88,365,936	88,368,071	-	88,367,476	88,367,788	+
GRMZM2G155868	XLOC_039962	enclosed	9	89,166,309	89,169,976	+	89,166,358	89,167,073	-
GRMZM2G101004	XLOC_038460	enclosed	9	89,922,996	89,933,844	-	89,933,126	89,933,395	+
GRMZM2G141288	XLOC_040016	enclosed	9	97,192,447	97,196,646	+	97,195,479	97,195,816	-
GRMZM2G071573	XLOC_040028	enclosed	9	98,257,402	98,262,701	+	98,260,096	98,262,511	-
GRMZM2G071573	XLOC_040027	divergent	9	98,257,402	98,262,701	+	98,256,395	98,257,480	-
GRMZM2G033555	XLOC_040048	divergent	9	100,416,810	100,423,707	+	100,416,596	100,417,256	-
GRMZM2G092493	GRMZM2G092586	convergent	9	100,766,352	100,770,684	+	100,769,455	100,779,590	-
GRMZM2G122447	XLOC_040057	divergent	9	101,311,298	101,312,871	+	101,311,215	101,312,056	-
GRMZM5G836222	XLOC_038563	enclosed	9	102,482,867	102,485,230	-	102,483,375	102,484,233	+
GRMZM2G065394	XLOC_040069	enclosed	9	103,110,845	103,113,461	+	103,112,503	103,113,053	-
GRMZM2G086773	XLOC_040083	enclosed	9	104,241,227	104,244,459	+	104,241,829	104,242,613	-
GRMZM2G139082	XLOC_040115	enclosed	9	107,641,650	107,645,156	+	107,644,186	107,644,947	-
GRMZM2G055320	XLOC_040120	divergent	9	107,810,066	107,816,743	+	107,809,927	107,811,180	-
GRMZM2G405474	GRMZM2G106357	divergent	9	108,225,231	108,228,267	+	108,220,507	108,227,166	-
GRMZM2G151496	XLOC_040126	enclosed	9	108,316,263	108,318,243	+	108,317,269	108,317,880	-
GRMZM2G038783	XLOC_040129	enclosed	9	108,447,914	108,449,734	+	108,449,125	108,449,560	-
GRMZM2G103805	XLOC_038611	enclosed	9	109,146,317	109,151,812	-	109,150,958	109,151,675	+
GRMZM2G103647	XLOC_038612	enclosed	9	109,162,497	109,164,977	-	109,164,376	109,164,863	+
GRMZM2G157115	XLOC_038613	enclosed	9	109,311,410	109,317,567	-	109,316,961	109,317,310	+
GRMZM2G095302	GRMZM2G095287	convergent	9	109,332,530	109,337,572	+	109,336,954	109,340,083	-
GRMZM2G017739	XLOC_038629	enclosed	9	111,478,926	111,485,379	-	111,479,068	111,481,244	+
GRMZM2G017685	XLOC_038630	convergent	9	111,500,037	111,500,591	-	111,500,012	111,500,553	+
GRMZM2G049229	XLOC_038635	divergent	9	112,024,912	112,028,149	-	112,025,237	112,028,773	+
GRMZM2G144818	GRMZM2G144841	divergent	9	113,509,124	113,515,269	-	113,514,930	113,517,932	+
GRMZM2G428470	XLOC_038676	enclosed	9	114,574,678	114,576,847	-	114,574,997	114,575,758	+
GRMZM2G028796	XLOC_040198	enclosed	9	114,714,284	114,741,438	+	114,715,073	114,715,420	-
GRMZM2G069807	XLOC_038688	divergent	9	115,402,677	115,404,899	-	115,403,001	115,405,100	+
GRMZM2G409245	XLOC_038710	divergent	9	117,079,224	117,080,432	-	117,079,453	117,080,654	+
GRMZM2G035517	XLOC_040245	convergent	9	120,199,697	120,203,721	+	120,202,329	120,204,097	-
GRMZM2G152908	XLOC_038769	enclosed	9	122,479,087	122,485,767	-	122,479,195	122,485,674	+
GRMZM2G359033	XLOC_040284	divergent	9	124,820,597	124,822,010	+	124,820,087	124,821,692	-
GRMZM2G105092	XLOC_038795	enclosed	9	124,924,237	124,925,395	-	124,924,627	124,925,337	+
GRMZM2G145794	XLOC_038817	divergent	9	126,705,611	126,709,017	-	126,708,810	126,709,369	+
GRMZM2G030458	XLOC_040325	enclosed	9	127,611,784	127,613,690	+	127,611,832	127,613,195	-
GRMZM2G157683	XLOC_040335	enclosed	9	128,348,131	128,349,850	+	128,348,817	128,349,061	-
GRMZM2G017460	XLOC_038851	enclosed	9	129,107,641	129,110,218	-	129,109,606	129,110,192	+
GRMZM2G121360	XLOC_040362	divergent	9	130,353,591	130,355,317	+	130,352,977	130,355,313	-
GRMZM2G134107	XLOC_040373	enclosed	9	130,759,495	130,760,703	+	130,759,584	130,759,876	-
GRMZM5G889372	XLOC_038931	enclosed	9	136,728,264	136,734,142	-	136,732,038	136,733,527	+
GRMZM2G078472	GRMZM2G078469	convergent	9	138,040,992	138,044,636	-	138,039,784	138,042,756	+
GRMZM2G053987	XLOC_038956	enclosed	9	138,684,219	138,687,449	-	138,687,118	138,687,386	+
GRMZM2G049318	XLOC_038971	enclosed	9	139,137,817	139,139,408	-	139,138,144	139,138,561	+
GRMZM2G305027	XLOC_038977	enclosed	9	139,445,735	139,446,720	-	139,445,962	139,446,689	+
GRMZM2G103490	XLOC_040519	enclosed	9	139,870,591	139,873,524	+	139,871,295	139,871,630	-
GRMZM2G151992	XLOC_038989	enclosed	9	140,100,585	140,102,820	-	140,100,599	140,101,751	+
GRMZM2G031447	XLOC_039018	divergent	9	141,795,025	141,797,141	-	141,795,550	141,797,599	+
XLOC_039024	XLOC_040562	convergent	9	142,276,005	142,281,908	+	142,281,711	142,282,929	-
GRMZM2G081175	XLOC_040581	enclosed	9	143,586,299	143,590,618	+	143,586,334	143,588,750	-
GRMZM2G178279	XLOC_040591	enclosed	9	144,209,790	144,213,235	+	144,212,381	144,213,018	-
GRMZM2G177914	XLOC_040600	enclosed	9	144,855,111	144,858,225	+	144,855,254	144,855,542	-
GRMZM2G177914	XLOC_040601	enclosed	9	144,855,111	144,858,225	+	144,857,040	144,857,982	-
GRMZM2G105834	XLOC_040607	enclosed	9	145,045,717	145,047,357	+	145,046,440	145,047,353	-
GRMZM2G091742	XLOC_040615	divergent	9	145,424,529	145,426,536	+	145,424,444	145,425,939	-
GRMZM2G067426	XLOC_040619	divergent	9	145,592,092	145,595,076	+	145,591,378	145,592,855	-
GRMZM2G067225	XLOC_039083	enclosed	9	145,604,342	145,606,411	-	145,604,342	145,606,676	+
GRMZM2G046601	XLOC_040634	enclosed	9	146,319,637	146,328,720	+	146,326,185	146,327,327	-
GRMZM2G113232	GRMZM2G113205	convergent	9	147,651,828	147,666,658	+	147,665,984	147,667,439	-
GRMZM2G050159	XLOC_039142	divergent	9	149,280,835	149,282,968	-	149,281,940	149,282,971	+
GRMZM2G004466	XLOC_040707	enclosed	9	149,512,257	149,513,861	+	149,512,679	149,513,156	-
GRMZM2G004377	GRMZM2G004466	convergent	9	149,513,489	149,519,493	-	149,512,257	149,513,861	+
GRMZM2G036411	XLOC_039167	divergent	9	150,509,518	150,510,116	-	150,509,633	150,510,254	+
GRMZM2G036351	XLOC_039168	enclosed	9	150,514,279	150,515,757	-	150,514,799	150,515,518	+
AC149829.2_FG002	XLOC_039183	enclosed	9	151,381,654	151,383,564	-	151,382,817	151,383,310	+
XLOC_040760	XLOC_039184	divergent	9	151,414,935	151,418,059	-	151,417,535	151,418,083	+
GRMZM2G178787	GRMZM2G178826	convergent	9	151,538,905	151,543,176	+	151,542,239	151,546,401	-
GRMZM2G054969	XLOC_039195	enclosed	9	151,692,176	151,696,132	-	151,692,379	151,692,756	+
GRMZM2G127072	XLOC_040778	enclosed	9	151,918,650	151,921,069	+	151,919,604	151,920,602	-
GRMZM2G126682	XLOC_040785	enclosed	9	151,998,333	152,000,250	+	151,998,593	151,999,043	-
GRMZM2G126682	XLOC_040786	enclosed	9	151,998,333	152,000,250	+	151,999,349	152,000,146	-
GRMZM2G010017	XLOC_040788	enclosed	9	152,081,582	152,086,034	+	152,081,653	152,082,635	-
GRMZM2G330019	XLOC_039241	enclosed	9	153,128,005	153,133,217	-	153,129,753	153,132,086	+
GRMZM2G472696	XLOC_039267	enclosed	9	154,211,297	154,212,031	-	154,211,427	154,211,993	+
GRMZM2G393334	GRMZM2G092497	convergent	9	154,723,964	154,729,849	+	154,729,257	154,731,780	-
GRMZM2G168355	XLOC_039294	enclosed	9	155,707,392	155,709,337	-	155,707,632	155,709,055	+
GRMZM2G462243	XLOC_040883	enclosed	9	155,855,551	155,856,182	+	155,855,551	155,856,028	-
GRMZM2G161905	XLOC_040885	divergent	9	155,866,514	155,867,635	+	155,865,947	155,867,472	-
GRMZM2G066165	GRMZM2G066142	divergent	10	1,078,071	1,084,308	-	1,083,998	1,085,635	+
GRMZM2G043150	GRMZM2G043119	convergent	10	1,950,526	1,959,850	-	1,948,867	1,951,755	+
GRMZM2G151903	XLOC_007601	enclosed	10	2,435,439	2,439,171	+	2,436,111	2,437,872	-
GRMZM2G151977	XLOC_007603	enclosed	10	2,448,850	2,453,307	+	2,449,057	2,449,866	-
GRMZM2G058481	XLOC_007620	divergent	10	3,176,422	3,177,326	+	3,176,413	3,176,925	-
GRMZM2G058595	XLOC_007621	divergent	10	3,193,010	3,194,101	+	3,192,437	3,194,049	-
XLOC_006252	XLOC_007622	enclosed	10	3,194,760	3,196,017	+	3,194,813	3,195,851	-
XLOC_007649	GRMZM2G088313	divergent	10	4,056,048	4,057,136	-	4,056,253	4,065,016	+
GRMZM2G054481	XLOC_006267	enclosed	10	4,529,937	4,531,806	-	4,531,087	4,531,735	+
GRMZM2G022318	XLOC_007662	enclosed	10	4,614,582	4,617,945	+	4,616,867	4,617,850	-
GRMZM2G434170	XLOC_007686	enclosed	10	5,225,476	5,228,126	+	5,226,560	5,227,949	-
GRMZM2G464328	GRMZM2G164113	enclosed	10	5,645,825	5,646,958	+	5,645,510	5,651,577	-
GRMZM2G057753	XLOC_007707	enclosed	10	6,402,110	6,402,921	+	6,402,289	6,402,853	-
AC202107.3_FG001	XLOC_007708	divergent	10	6,533,858	6,535,307	+	6,533,850	6,534,518	-
GRMZM2G136910	XLOC_006324	enclosed	10	8,853,009	8,853,996	-	8,853,027	8,853,868	+
GRMZM5G813470	XLOC_006347	enclosed	10	10,887,778	10,892,143	-	10,890,911	10,891,138	+
GRMZM2G015869	XLOC_006355	enclosed	10	12,927,390	12,933,138	-	12,930,630	12,933,118	+
GRMZM2G009448	XLOC_007808	enclosed	10	17,611,983	17,615,854	+	17,614,876	17,615,435	-
AC198699.3_FG004	XLOC_007815	enclosed	10	18,744,368	18,745,364	+	18,742,238	18,745,462	-
GRMZM2G169240	XLOC_006394	enclosed	10	19,075,431	19,077,551	-	19,075,885	19,076,795	+
XLOC_006404	XLOC_007823	divergent	10	20,328,528	20,329,584	+	20,327,689	20,328,985	-
GRMZM2G036427	XLOC_007842	enclosed	10	23,221,552	23,223,794	+	23,221,601	23,222,141	-
XLOC_007844	XLOC_006424	enclosed	10	23,390,500	23,392,415	-	23,390,706	23,392,369	+
GRMZM2G054250	GRMZM2G054393	divergent	10	25,562,436	25,565,712	-	25,565,234	25,575,047	+
GRMZM2G420407	GRMZM2G120530	convergent	10	29,229,592	29,231,490	+	29,230,376	29,236,119	-
GRMZM2G102683	XLOC_007896	enclosed	10	29,631,122	29,634,845	+	29,633,337	29,634,690	-
GRMZM2G171518	XLOC_007910	convergent	10	33,613,510	33,625,476	+	33,623,886	33,625,576	-
GRMZM2G565911	XLOC_007917	enclosed	10	35,929,292	35,930,296	+	35,929,743	35,930,125	-
GRMZM2G094352	XLOC_006523	enclosed	10	49,989,446	49,992,027	-	49,989,787	49,992,026	+
GRMZM2G178817	XLOC_007999	enclosed	10	58,462,136	58,463,278	+	58,462,564	58,463,138	-
XLOC_006580	XLOC_008016	enclosed	10	61,960,766	61,968,571	+	61,960,807	61,961,374	-
GRMZM2G364748	XLOC_006623	enclosed	10	68,937,520	68,938,909	-	68,937,698	68,938,766	+
GRMZM2G113682	XLOC_006656	enclosed	10	75,157,271	75,167,108	-	75,157,846	75,158,315	+
GRMZM2G110145	XLOC_008118	enclosed	10	77,282,595	77,289,172	+	77,287,726	77,288,880	-
GRMZM2G078906	XLOC_008126	enclosed	10	78,074,137	78,076,764	+	78,074,671	78,075,747	-
GRMZM2G123886	GRMZM5G806309	enclosed	10	79,150,828	79,152,284	+	79,150,370	79,160,123	-
XLOC_008140	XLOC_006681	enclosed	10	79,422,406	79,423,830	-	79,422,812	79,423,830	+
XLOC_008141	XLOC_006682	enclosed	10	79,424,059	79,424,752	-	79,424,059	79,424,883	+
GRMZM2G086496	XLOC_008144	enclosed	10	80,056,275	80,062,527	+	80,057,798	80,058,334	-
GRMZM2G325907	XLOC_006709	enclosed	10	81,840,736	81,843,543	-	81,841,212	81,841,481	+
XLOC_008193	XLOC_006759	enclosed	10	86,066,475	86,067,579	-	86,066,482	86,066,878	+
GRMZM2G028104	XLOC_008201	divergent	10	86,998,288	87,000,589	+	86,998,117	87,000,393	-
GRMZM2G015605	XLOC_006771	enclosed	10	87,283,734	87,284,881	-	87,284,105	87,284,585	+
GRMZM2G138252	XLOC_008230	enclosed	10	90,257,150	90,259,318	+	90,258,469	90,259,237	-
GRMZM2G110567	XLOC_008246	enclosed	10	92,550,666	92,552,639	+	92,550,696	92,552,545	-
GRMZM2G131611	XLOC_006835	divergent	10	95,136,396	95,139,678	-	95,139,127	95,139,940	+
GRMZM2G330635	XLOC_006848	enclosed	10	96,774,485	96,775,465	-	96,774,706	96,775,443	+
GRMZM2G075315	XLOC_008296	enclosed	10	98,012,816	98,014,124	+	98,013,226	98,013,624	-
GRMZM2G019246	XLOC_006863	enclosed	10	99,220,164	99,223,049	-	99,220,564	99,221,170	+
GRMZM2G119116	GRMZM2G119127	enclosed	10	99,619,669	99,624,690	-	99,624,013	99,624,641	+
XLOC_006878	XLOC_008322	divergent	10	100,699,317	100,701,433	+	100,699,105	100,699,594	-
GRMZM2G122437	XLOC_008380	enclosed	10	109,913,509	109,916,009	+	109,915,061	109,915,553	-
GRMZM2G451716	XLOC_008393	enclosed	10	111,444,985	111,453,558	+	111,445,144	111,445,552	-
GRMZM2G138355	XLOC_008397	divergent	10	112,222,702	112,225,808	+	112,222,537	112,223,557	-
XLOC_007018	XLOC_008454	divergent	10	117,485,115	117,486,672	+	117,484,811	117,486,494	-
GRMZM2G136508	XLOC_008458	enclosed	10	117,641,432	117,645,132	+	117,643,682	117,643,935	-
GRMZM2G096695	XLOC_007032	enclosed	10	119,842,835	119,843,683	-	119,842,960	119,843,231	+
GRMZM2G359735	XLOC_008552	enclosed	10	126,160,075	126,165,445	+	126,160,350	126,160,680	-
GRMZM2G000261	XLOC_007148	enclosed	10	128,580,332	128,582,775	-	128,580,520	128,581,374	+
GRMZM5G893444	XLOC_007189	divergent	10	131,910,570	131,914,628	-	131,913,905	131,914,803	+
GRMZM2G046952	XLOC_007197	enclosed	10	132,120,374	132,122,335	-	132,121,098	132,121,375	+
GRMZM2G131969	GRMZM2G132218	convergent	10	132,197,230	132,201,623	+	132,201,404	132,204,663	-
GRMZM2G046841	XLOC_007203	enclosed	10	132,446,398	132,447,255	-	132,446,577	132,447,208	+
GRMZM2G168953	XLOC_008632	enclosed	10	132,569,729	132,572,415	+	132,569,731	132,570,548	-
XLOC_008661	XLOC_007252	enclosed	10	134,829,905	134,830,675	-	134,830,087	134,830,456	+
GRMZM2G169690	XLOC_008671	divergent	10	135,506,682	135,509,408	+	135,506,483	135,507,638	-
GRMZM2G098676	XLOC_008678	divergent	10	136,041,276	136,044,237	+	136,041,206	136,042,977	-
XLOC_007279	XLOC_008699	divergent	10	136,721,249	136,721,959	+	136,718,978	136,721,714	-
GRMZM2G142832	XLOC_008712	enclosed	10	137,853,661	137,856,863	+	137,853,826	137,854,820	-
GRMZM5G803874	XLOC_007308	convergent	10	138,512,829	138,515,377	-	138,512,572	138,514,126	+
GRMZM2G555422	XLOC_007322	enclosed	10	139,384,342	139,385,051	-	139,384,420	139,384,780	+
GRMZM2G109753	GRMZM2G129169	enclosed	10	140,355,466	140,426,941	+	140,384,896	140,389,821	-
GRMZM2G147408	XLOC_007349	convergent	10	141,312,645	141,313,335	-	141,312,471	141,313,203	+
GRMZM2G148495	XLOC_008813	enclosed	10	142,868,690	142,869,793	+	142,868,770	142,869,727	-
GRMZM2G431504	XLOC_008824	enclosed	10	143,178,859	143,181,050	+	143,178,937	143,179,364	-
GRMZM5G895313	XLOC_008827	enclosed	10	143,487,116	143,488,331	+	143,487,540	143,488,048	-
GRMZM5G818977	XLOC_008830	enclosed	10	143,740,959	143,744,585	+	143,743,661	143,744,585	-
GRMZM2G085236	XLOC_007429	enclosed	10	144,340,337	144,347,928	-	144,346,275	144,346,895	+
GRMZM2G153017	XLOC_008854	enclosed	10	145,054,951	145,056,959	+	145,055,233	145,056,650	-
GRMZM2G143480	GRMZM2G143499	divergent	10	145,330,728	145,339,330	+	145,327,308	145,330,955	-
GRMZM2G143402	XLOC_008858	divergent	10	145,369,441	145,373,354	+	145,369,416	145,371,738	-
GRMZM2G067424	XLOC_008876	enclosed	10	146,078,469	146,080,245	+	146,078,896	146,080,074	-
GRMZM2G179532	XLOC_008900	divergent	10	146,745,295	146,747,273	+	146,745,094	146,746,414	-
GRMZM2G340065	XLOC_007491	convergent	10	146,895,394	146,899,390	-	146,893,472	146,899,173	+
GRMZM2G150014	XLOC_007493	enclosed	10	147,014,559	147,017,456	-	147,015,584	147,015,930	+
GRMZM2G150286	XLOC_007494	enclosed	10	147,043,160	147,045,718	-	147,045,247	147,045,660	+
GRMZM2G142757	XLOC_008935	enclosed	10	147,855,454	147,859,264	+	147,857,896	147,859,019	-
GRMZM2G142820	XLOC_007519	divergent	10	147,868,922	147,872,174	-	147,871,601	147,872,416	+
GRMZM2G074759	XLOC_007524	enclosed	10	148,065,497	148,067,641	-	148,065,617	148,066,552	+
GRMZM2G074718	GRMZM2G074107	enclosed	10	148,075,099	148,076,695	+	148,074,813	148,081,156	-
GRMZM2G106792	XLOC_008952	enclosed	10	148,253,993	148,255,416	+	148,254,333	148,255,179	-
GRMZM2G406099	XLOC_007531	divergent	10	148,283,968	148,286,266	-	148,284,757	148,286,459	+
GRMZM2G001934	XLOC_007535	enclosed	10	148,312,549	148,316,275	-	148,313,887	148,314,859	+
GRMZM2G104613	GRMZM2G104658	convergent	10	148,407,014	148,410,710	+	148,410,223	148,412,237	-
GRMZM2G110908	GRMZM2G110869	convergent	10	148,737,011	148,739,143	+	148,737,480	148,741,164	-
GRMZM2G011590	XLOC_008968	enclosed	10	148,821,382	148,830,411	+	148,828,169	148,829,357	-
GRMZM5G824597	GRMZM5G885706	divergent	10	149,178,679	149,182,049	-	149,181,221	149,182,500	+
GRMZM5G836471	XLOC_007556	enclosed	10	149,245,361	149,251,265	-	149,246,609	149,248,076	+

Supplemental Table 4. Information of primers used in experimental validation of natural antisense transcript pairs

Primer name		Forward Primers sequences	Reverse Primers sequences	Descriptions	Annealing temperatures
GAPDH		CCATCACTGCCACACAGAAAAC	AGGAACACGGAAGGACATACCAG	Housekeeping gene for qPCR reaction	62 ^oC
18S		CTGAGAAACGGCTACCACA	CCCAAGGTCCAACTACGAG	Housekeeping gene for qPCR reaction	53 ^oC
GRMZM2G033219:GRMZM2G033430	GRMZM2G033219	TGTGAAGACATGGATCAG	AATATGCTCGGCAGTAAG	Gene specific primers*	52 ^oC
	GRMZM2G033430	AACGATTGCTGCTGATGA	CTCAATTCTTCACGAAGTTGTT	Gene specific primers	52 ^oC
GRMZM2G132212:GRMZM2G432801	GRMZM2G132212	GTACACCAACAACCTCTC	TCAACCTCTACCAAACCT	Gene specific primers	52 ^oC
	GRMZM2G432801	TCCTGATGATATTGATGTT	TTGATACTGAAGGTGAAG	Gene specific primers	52 ^oC
GRMZM2G442277:GRMZM2G141704	GRMZM2G442277	AATGACAGTGATATGCTT	TGATTGGAGATTTGGTTA	Gene specific primers	45 ^oC
	GRMZM2G141704	ACTATCTCCTGCTTATGTT	AATTCATGGCACTTTCTG	Gene specific primers	45 ^oC
GRMZM2G018595:GRMZM2G019500	GRMZM2G018595	GTCCTTGTCCTGGAGATA	CTCTAGGAGAATCTTGTCATC	Gene specific primers	52 ^oC
	GRMZM2G019500	TGTTGTTGACCATATTCG	AAGAACAGAAGGAGAAGG	Gene specific primers	52 ^oC

*Gene specific primers were used for both strand-specific reverse transcriptase PCR and qPCR. The reverse primer of each gene was also used for first-strand cDNA synthesis.

Supplemental Table 5. The number and ratio of transcripts and NAT pairs with significant smRNA abundance changes under drought stress in four maize lines.

Category	AC7643		AC7729/TZSRW		RIL208		RIL64	
	Up-regulated	Down-regulated	Up-regulated	Down-regulated	Up-regulated	Down-regulated	Up-regulated	Down-regulated
Transcripts with smRNA abundance changes Sig.	358 (2.30%)	533 (3.42%)	448 (2.87%)	351 (2.25%)	373 (2.38%)	355 (2.27%)	517 (3.30%)	448 (2.86%)
Sense with smRNA abundance changes Sig.	20 (4.90%)	37 (9.07%)	36 (7.52%)	19 (3.97%)	34 (5.64%)	40 (6.63%)	66 (10.82%)	33 (5.41%)
Antisense with smRNA abundance changes Sig.	18 (4.41%)	23 (5.64%)	36 (7.52%)	17 (3.55%)	38 (6.30%)	31 (5.14%)	73 (11.97%)	25 (4.10%)
NAT pair with smRNA abundance changes Sig.	38 (4.66%)	60 (7.35%)	72 (7.52%)	36 (3.76%)	72 (5.97%)	71 (5.89%)	139 (11.39%)	58 (4.75%)
P value (χ^2 test)	3.23E-05	8.34E-09	2.34E-15	3.73E-03	1.33E-13	2.35E-14	1.13E-44	2.56E-04
Sig. bootstrap	9.34 (2.29%)	13.84 (3.39%)	13.71 (2.86%)	10.57 (2.21%)	14.34 (2.38%)	13.62 (2.26%)	20.1 (3.29%)	17.5 (2.87%)
P value (t test)	0.00E+00	0.00E+00	0.00E+00	0.00E+00	0.00E+00	0.00E+00	0.00E+00	0.00E+00

The ratio of drought responsive transcripts and NAT pairs with significant smRNA abundance changes under drought stress is in parentheses (%).

NATs: natural antisense transcripts; *P* value (χ2 test): P value of χ2 test for assessing differences between significantly up- or down-regulated genes of overall transcripts and NAT pairs identified in each inbred.

P value (t test): *P* value of t test for assessing differences between significantly up- or down-regulated genes of bootstrap samples in overall transcripts and NAT pairs identified in each inbred.

Supplemental Table 6. Association analysis of natural variation in NAT pairs genes with survival rate under drought stress in 368 maize inbreds

Sense Transcripts								Antisense Transcripts							
Gene Name	Chromosome	Start (bp)	End (bp)	SNP number	Significant SNP number GLM (P<=0.01)	Significant SNP number MLM (P<=0.01)	Significant SNP number in total	Gene Name	Chromosome	Start (bp)	End (bp)	SNP number	Significant SNP number GLM (P<=0.01)	Significant SNP number MLM (P<=0.01)	Significant SNP number in total
GRMZM2G032104	1	645004	652666	14	1	0	1	XLOC_000012	1	644614	645991	13	1	0	1
GRMZM5G802392	1	2839306	2840779	25	0	1	1	XLOC_003174	1	2839365	2840264	25	0	1	1
GRMZM2G015384	1	2873956	2894310	16	3	4	4	GRMZM2G007025	1	2871385	2875166	22	0	0	0
XLOC_003179	1	3127903	3129982	36	10	10	10	XLOC_000050	1	3125558	3129266	38	10	10	10
XLOC_000052	1	3128229	3130830	58	16	13	16	XLOC_003179	1	3127903	3129982	36	10	10	10
XLOC_000052	1	3128229	3130830	58	16	13	16	XLOC_003180	1	3130242	3130740	15	6	3	6
GRMZM2G159477	1	5039291	5042641	43	16	7	16	XLOC_003216	1	5038799	5040058	33	17	8	17
GRMZM2G327059	1	5080787	5086610	17	0	1	1	XLOC_003218	1	5084935	5086394	6	0	0	0
GRMZM2G409976	1	7078142	7082865	-	-	-	-	GRMZM2G109509	1	7079988	7085969	36	0	1	1
GRMZM2G110616	1	7563041	7565951	44	0	0	0	GRMZM2G410991	1	7561410	7564192	55	0	1	1
GRMZM2G086163	1	11473558	11475611	94	3	4	4	XLOC_000210	1	11473930	11474971	45	0	0	0
GRMZM2G162749	1	14850090	14853017	38	2	4	4	XLOC_003373	1	14851376	14852775	18	0	0	0
GRMZM2G139963	1	14958183	14960872	26	1	2	2	XLOC_000274	1	14960091	14960882	9	0	0	0
GRMZM2G163154	1	27099652	27108024	17	0	0	0	GRMZM2G163129	1	27095059	27100615	41	1	0	1
GRMZM2G126808	1	29540914	29544577	20	1	0	1	XLOC_000465	1	29543125	29543575	1	0	0	0
GRMZM2G077942	1	32873395	32876087	64	1	1	1	XLOC_000510	1	32875353	32875768	2	1	1	1
XLOC_003652	1	39144013	39146199	34	2	2	2	XLOC_000585	1	39144595	39146126	20	2	2	2
GRMZM2G017792	1	43690068	43692986	37	1	0	1	XLOC_000648	1	43692630	43692886	9	0	0	0
GRMZM2G053669	1	45119006	45122764	14	1	0	1	XLOC_000663	1	45119161	45120699	10	1	0	1
GRMZM2G033570	1	52561965	52565938	56	2	0	2	XLOC_000769	1	52562240	52564454	51	2	0	2
GRMZM2G026417	1	64021077	64022794	36	2	2	2	XLOC_000900	1	64021299	64021898	13	0	0	0
GRMZM2G084587	1	76878489	76889924	22	1	1	1	GRMZM2G385050	1	76889570	76904210	35	0	0	0
GRMZM2G028821	1	81612706	81613830	25	3	3	3	XLOC_001068	1	81612836	81613690	18	3	3	3
GRMZM2G026147	1	85027826	85029741	52	20	2	20	XLOC_004131	1	85029194	85029701	14	3	0	3
GRMZM2G453832	1	85998283	86001830	26	1	1	1	GRMZM2G154549	1	86001434	86004907	12	0	0	0
GRMZM2G079470	1	88487882	88491179	23	1	2	2	XLOC_001134	1	88490036	88491131	5	0	0	0
GRMZM2G317386	1	90797641	90799416	-	-	-	-	XLOC_004174	1	90798196	90800178	27	5	0	5
GRMZM2G114772	1	92248217	92252805	25	1	0	1	GRMZM2G114707	1	92250389	92253227	30	0	0	0
GRMZM2G094639	1	92473694	92474849	44	3	0	3	XLOC_004184	1	92473871	92474678	35	3	0	3
GRMZM2G093755	1	180018445	180021636	20	1	1	1	XLOC_001584	1	180020968	180021646	9	0	0	0
GRMZM2G310947	1	180651899	180653885	58	1	2	2	XLOC_001590	1	180651917	180653172	47	1	2	2
GRMZM2G423472	1	180715451	180720707	1	0	0	0	XLOC_004639	1	180717585	180718436	8	2	1	2
GRMZM2G023791	1	182125031	182126803	41	2	2	2	XLOC_001611	1	182125586	182126923	40	2	2	2
GRMZM5G899800	1	201502172	201509140	4	1	0	1	XLOC_004849	1	201502572	201502846	1	0	0	0
GRMZM2G315375	1	202334807	202342249	62	3	2	3	XLOC_004857	1	202340036	202340725	5	0	0	0
GRMZM2G448185	1	208868009	208873195	41	2	1	2	XLOC_001946	1	208870529	208870976	4	0	0	0
GRMZM2G131205	1	211567120	211572922	62	0	1	1	XLOC_004979	1	211570193	211573240	19	0	1	1
GRMZM5G882228	1	226230810	226234051	41	1	1	1	XLOC_005127	1	226230644	226231367	8	0	0	0
GRMZM2G152901	1	237009950	237014064	34	1	1	1	XLOC_005311	1	237009966	237013206	25	1	1	1
GRMZM2G442658	1	274050247	274053994	86	3	2	3	XLOC_002674	1	274052049	274053751	37	3	2	3
GRMZM2G001869	1	274785474	274790748	41	2	0	2	XLOC_005725	1	274789368	274790183	15	0	0	0
GRMZM2G159285	1	275098368	275101121	25	1	0	1	XLOC_002696	1	275094121	275099076	68	1	0	1
GRMZM2G147687	1	276371897	276377338	90	6	5	6	XLOC_005755	1	276371474	276372465	25	0	0	0
GRMZM2G147687	1	276371897	276377338	90	6	5	6	XLOC_005756	1	276375849	276377038	22	4	3	4
GRMZM2G017186	1	276389960	276395246	62	1	0	1	XLOC_005758	1	276393398	276393847	5	0	0	0
GRMZM2G090422	1	277805999	277807838	14	0	1	1	XLOC_005786	1	277806426	277807762	8	0	1	1
GRMZM2G329181	1	286049844	286053001	71	1	1	1	GRMZM2G028926	1	286045297	286050143	16	1	0	1
GRMZM2G019799	1	286429050	286437267	15	1	0	1	XLOC_002860	1	286432765	286437028	12	0	0	0
GRMZM2G019799	1	286429050	286437267	15	1	0	1	XLOC_002859	1	286428657	286429831	16	4	0	4
GRMZM2G060765	1	286736778	286741262	40	0	0	0	GRMZM2G060924	1	286730567	286737443	43	1	0	1
GRMZM2G120674	1	286916349	286921522	31	0	0	0	GRMZM2G120657	1	286920950	286923692	51	1	0	1
GRMZM2G151997	1	292412495	292413903	9	0	1	1	XLOC_002964	1	292412705	292413447	4	0	1	1
GRMZM2G139250	1	292594128	292598884	30	1	1	2	XLOC_002967	1	292594168	292595450	19	1	0	1
GRMZM2G113158	1	296739858	296743544	53	1	1	1	XLOC_003038	1	296740624	296742835	31	1	1	1
GRMZM2G170137	1	296849361	296855071	73	4	2	4	GRMZM2G470422	1	296847943	296850494	16	0	0	0
GRMZM2G352415	1	297095291	297098628	50	3	0	3	XLOC_003050	1	297096040	297098364	27	0	0	0
GRMZM2G016084	1	297827097	297832384	25	0	0	0	XLOC_003061	1	297830785	297832332	26	1	1	1
GRMZM2G011923	1	298817393	298820925	58	1	0	1	XLOC_003086	1	298819416	298820340	36	0	0	0
GRMZM2G115456	1	299272391	299278057	58	4	1	4	GRMZM2G417843	1	299276941	299281868	24	0	0	0
XLOC_011451	2	177675	179791	21	9	3	9	XLOC_008977	2	177859	179378	15	6	1	6
GRMZM2G064212	2	1038129	1042294	15	1	1	1	XLOC_011464	2	1038189	1038486	2	0	0	0
GRMZM2G018595	2	1437737	1441025	51	0	0	0	GRMZM2G019500	2	1439756	1444066	47	0	1	1
GRMZM2G033219	2	4839823	4846096	91	0	0	0	GRMZM2G033430	2	4839281	4841272	65	2	2	2
GRMZM2G032977	2	4852765	4855560	18	2	0	2	XLOC_009122	2	4853539	4854344	4	0	0	0
XLOC_011568	2	5306289	5309292	49	1	0	1	XLOC_009137	2	5306604	5307559	28	1	0	1
GRMZM2G174917	2	5568332	5570261	47	1	1	1	XLOC_011572	2	5569296	5569921	24	0	0	0
XLOC_011624	2	7047927	7051492	35	3	3	4	XLOC_009182	2	7049348	7050124	1	0	0	0
GRMZM2G164400	2	7519507	7527867	33	0	0	0	GRMZM2G164277	2	7527470	7531925	61	0	3	3
GRMZM2G084181	2	9117236	9130542	35	2	2	2	XLOC_011646	2	9126849	9127558	5	0	0	0
GRMZM2G005040	2	10387109	10389573	27	1	2	2	XLOC_011679	2	10387374	10389429	15	1	2	2
GRMZM2G071832	2	11769293	11771169	2	1	1	1	XLOC_011714	2	11769111	11771067	2	1	1	1
GRMZM2G131280	2	13552922	13555183	62	4	5	5	XLOC_011739	2	13554335	13554617	30	0	0	0
GRMZM2G131280	2	13552922	13555183	62	4	5	5	XLOC_011740	2	13554719	13555152	17	2	2	2
GRMZM5G810061	2	23123860	23128108	22	2	5	5	XLOC_011876	2	23125153	23127375	14	2	4	4
GRMZM2G362413	2	27500744	27502059	19	2	1	2	XLOC_011944	2	27501203	27501645	9	1	0	1
GRMZM2G347361	2	30305903	30309532	34	1	0	1	GRMZM2G171620	2	30308490	30311407	17	0	0	0
GRMZM2G063262	2	37392043	37396674	77	0	0	0	GRMZM2G063163	2	37396078	37402459	74	0	2	2
XLOC_009682	2	40051916	40054726	44	0	2	2	GRMZM2G330430	2	40054195	40056907	57	0	3	3
XLOC_009682	2	40051916	40054726	44	0	2	2	XLOC_012105	2	40051927	40052520	18	0	0	0
GRMZM2G127139	2	45069108	45078053	17	0	1	1	XLOC_009751	2	45073255	45073509	-	-	-	-
GRMZM2G436533	2	64294902	64298527	18	0	0	0	GRMZM2G136412	2	64298381	64301370	38	1	1	1
GRMZM2G086267	2	68168970	68174713	18	1	1	1	XLOC_012351	2	68169226	68169426	1	0	0	0
GRMZM2G027272	2	74719597	74724317	2	0	1	1	XLOC_009967	2	74721867	74722089	1	0	0	0
GRMZM2G172695	2	104803852	104806393	11	0	1	1	XLOC_012500	2	104805122	104806195	9	0	1	1
GRMZM2G133716	2	136740072	136745887	57	0	1	1	XLOC_012684	2	136743919	136745759	32	0	1	1
GRMZM2G032478	2	142057153	142058876	2	1	0	1	XLOC_012711	2	142057146	142058616	2	1	0	1
GRMZM2G037150	2	176669016	176673281	12	1	1	1	GRMZM2G037131	2	176668559	176669692	2	0	0	0
GRMZM2G373554	2	177562284	177563575	34	1	1	1	XLOC_012976	2	177562929	177563476	22	0	0	0
GRMZM2G003947	2	190587060	190589608	14	1	1	1	XLOC_010664	2	190588589	190589447	12	0	0	0
GRMZM2G014341	2	192312471	192316197	50	10	0	10	GRMZM2G013892	2	192310632	192313184	31	2	0	2
GRMZM2G101769	2	211875412	211877983	31	2	0	2	XLOC_013421	2	211875392	211876119	10	0	0	0
XLOC_013465	2	214483221	214484563	9	1	0	1	XLOC_011057	2	214483242	214483828	9	1	0	1
GRMZM2G141432	2	215752666	215753667	22	3	2	3	XLOC_013486	2	215752840	215753423	13	2	1	2
XLOC_011093	2	215950474	215954466	47	2	13	13	XLOC_013492	2	215949546	215952234	35	2	10	10
XLOC_011093	2	215950474	215954466	47	2	13	13	XLOC_013493	2	215953968	215955328	18	0	0	0
GRMZM2G137546	2	220671360	220672972	34	1	1	1	XLOC_013562	2	220672012	220672735	10	1	1	1
GRMZM5G843434	2	221314181	221316190	3	1	0	1	XLOC_013568	2	221314265	221316032	15	1	0	1
GRMZM2G324544	2	235377544	235378343	20	1	1	1	GRMZM2G324507	2	235377387	235389082	39	1	1	1
GRMZM2G055313	3	4707293	4710578	21	1	1	1	XLOC_016107	3	4707024	4709135	6	0	0	0
GRMZM2G055313	3	4707293	4710578	21	1	1	1	XLOC_016108	3	4709572	4709965	3	1	1	1
GRMZM2G355572	3	7938344	7940976	41	7	0	7	XLOC_013910	3	7939013	7940694	34	7	0	7
GRMZM2G355381	3	9114106	9118344	21	1	1	1	XLOC_016191	3	9113934	9115143	7	0	0	0
GRMZM2G452564	3	15137688	15139240	47	5	2	5	XLOC_016255	3	15136784	15139075	38	5	2	5
GRMZM2G034534	3	18085249	18086875	37	0	1	1	XLOC_016291	3	18085278	18085649	6	0	0	0
GRMZM2G320521	3	19762347	19764749	59	0	0	0	XLOC_016312	3	19762167	19764374	46	0	1	1
GRMZM2G090584	3	26258962	26261458	47	3	0	3	XLOC_016363	3	26259115	26259614	15	0	0	0
GRMZM2G104829	3	26309355	26312448	20	1	0	1	XLOC_014167	3	26310637	26312874	7	1	0	1
GRMZM2G144648	3	41742447	41744589	26	3	1	3	XLOC_014266	3	41742767	41744902	20	2	1	2
GRMZM2G115960	3	48806998	48810388	19	1	0	1	XLOC_014300	3	48809033	48810511	9	0	0	0
GRMZM2G351387	3	126175759	126191667	43	1	0	1	XLOC_016921	3	126190434	126191618	21	1	0	1

GRMZM2G160902	3	128957613	128959445	42	1	0	1	XLOC_014656	3	128958274	128959485	29	1	0	1
GRMZM2G428168	3	149351183	149353705	13	1	1	1	GRMZM2G127789	3	149351145	149352030	1	0	0	0
GRMZM2G369652	3	150308311	150311184	12	0	0	0	GRMZM2G068665	3	150304861	150310060	17	1	1	1
GRMZM2G129114	3	160370641	160372491	23	1	0	1	XLOC_014944	3	160370919	160372685	20	1	0	1
GRMZM2G177227	3	165900055	165902629	31	1	0	1	XLOC_015002	3	165900808	165902750	22	1	0	1
GRMZM2G078500	3	172376539	172379695	79	0	1	1	XLOC_017375	3	172376634	172377105	28	0	1	1
GRMZM2G127117	3	176607781	176610780	29	1	0	1	XLOC_015163	3	176607974	176609165	16	0	0	0
GRMZM2G054807	3	180252370	180255087	28	1	0	1	XLOC_017485	3	180252317	180254703	27	0	0	0
GRMZM2G310368	3	188728061	188729514	30	4	0	4	XLOC_015345	3	188728781	188729273	8	1	0	1
GRMZM2G054905	3	191183824	191244226	24	0	1	1	XLOC_015379	3	191183128	191184336	13	0	0	0
GRMZM2G446220	3	195671907	195673492	26	0	1	1	XLOC_015438	3	195671890	195674277	25	0	1	1
GRMZM2G176699	3	198668535	198673264	15	0	1	1	GRMZM2G474326	3	198667643	198668930	-	-	-	-
GRMZM2G057067	3	199377243	199380242	55	2	2	2	XLOC_015503	3	199378034	199380119	41	2	2	2
GRMZM5G836674	3	199723335	199732187	26	3	3	3	GRMZM2G051151	3	199722345	199723907	30	0	1	1
XLOC_017795	3	200290750	200292783	39	3	3	3	XLOC_015519	3	200290750	200293283	39	3	3	3
XLOC_017798	3	200668046	200668710	16	1	3	3	XLOC_015521	3	200668046	200668721	16	1	3	3
GRMZM5G814314	3	212517687	212521608	22	2	2	2	XLOC_017987	3	212517867	212521224	9	0	0	0
GRMZM2G071021	3	221771306	221775473	39	1	0	1	XLOC_018125	3	221774725	221775130	8	0	0	0
GRMZM2G171036	4	2386082	2387319	8	1	0	1	XLOC_020472	4	2385958	2386696	6	1	0	1
GRMZM2G117971	4	9685582	9686560	15	1	0	1	XLOC_018447	4	9685645	9685961	12	1	0	1
GRMZM2G091481	4	14877039	14881740	87	3	0	3	XLOC_018498	4	14877134	14877930	25	2	0	2
GRMZM2G139894	4	18610025	18616532	65	1	0	1	XLOC_018536	4	18610785	18611676	8	1	0	1
GRMZM2G089995	4	26450748	26452606	21	3	1	3	XLOC_020702	4	26452045	26454670	6	0	0	0
GRMZM2G089995	4	26450748	26452606	21	3	1	3	XLOC_020701	4	26452045	26454285	6	0	0	0
GRMZM2G074280	4	36017140	36020195	23	0	1	1	GRMZM2G074087	4	36019078	36019888	20	0	0	0
GRMZM2G075496	4	42127982	42131478	31	2	3	3	XLOC_018755	4	42128033	42129663	17	0	0	0
GRMZM2G174732	4	71935082	71945964	32	1	1	1	XLOC_021018	4	71934793	71936351	22	0	0	0
XLOC_021125	4	92859072	92860700	1	0	1	1	XLOC_019017	4	92860154	92860755	1	0	1	1
GRMZM2G026922	4	152425543	152433694	56	1	1	1	GRMZM2G323719	4	152424058	152425840	39	0	0	0
GRMZM2G114998	4	155035662	155038571	23	5	3	6	XLOC_019354	4	155037503	155038790	1	0	0	0
GRMZM2G134023	4	156217617	156219742	46	11	4	11	XLOC_019362	4	156218446	156219862	47	11	4	11
GRMZM2G119258	4	160367219	160374647	12	3	0	3	XLOC_019416	4	160367021	160368068	3	0	0	0
GRMZM2G022514	4	161579119	161583314	25	1	0	1	XLOC_019440	4	161579943	161581091	12	0	0	0
GRMZM2G014854	4	167105924	167108481	55	4	6	6	XLOC_021554	4	167106377	167106872	8	0	0	0
GRMZM2G036464	4	167111209	167114536	26	1	0	1	XLOC_019498	4	167111036	167113698	22	1	0	1
GRMZM2G178398	4	172777612	172783745	24	0	1	1	XLOC_021629	4	172782890	172783635	8	0	0	0
GRMZM2G054210	4	173239888	173253971	34	1	1	1	XLOC_019579	4	173253159	173254280	3	0	0	0
GRMZM2G312521	4	174991068	174996208	68	2	1	2	XLOC_019600	4	174992060	174994904	49	2	1	2
GRMZM2G181359	4	175765088	175769387	27	0	2	2	GRMZM2G181362	4	175767391	175780753	37	0	0	0
GRMZM2G060470	4	185030955	185035131	18	2	2	2	XLOC_021805	4	185029082	185031304	3	0	0	0
GRMZM2G151516	4	185209888	185211510	27	1	2	2	XLOC_019743	4	185210476	185210873	11	0	1	1
GRMZM2G095141	4	186832765	186835999	58	1	1	1	GRMZM2G095326	4	186835570	186841247	37	0	0	0
GRMZM2G008859	4	235108555	235111831	48	4	1	4	XLOC_020246	4	235108395	235110759	34	4	1	4
XLOC_022369	4	236262710	236264243	23	1	0	1	XLOC_020286	4	236263993	236264323	-	-	-	-
XLOC_022369	4	236262710	236264243	23	1	0	1	XLOC_020285	4	236263278	236263657	16	0	0	0
GRMZM2G166035	4	238052080	238055542	30	2	1	2	XLOC_020341	4	238052108	238052348	7	0	0	0
GRMZM2G169998	4	240049949	240053626	63	1	0	1	XLOC_020387	4	240053313	240053631	7	1	0	1
GRMZM2G448001	5	1526661	1529125	41	0	0	0	GRMZM2G447976	5	1524444	1527639	44	1	1	1
GRMZM2G124229	5	4606404	4608549	11	3	0	3	XLOC_025038	5	4605996	4607845	10	3	0	3
GRMZM2G171468	5	6146163	6149014	95	1	0	1	XLOC_022735	5	6146270	6148226	88	1	0	1
GRMZM2G077295	5	7781628	7787521	21	0	0	0	GRMZM2G077356	5	7780703	7783409	48	1	0	1
GRMZM2G147814	5	7866856	7869861	36	2	0	2	GRMZM2G147809	5	7866128	7870805	52	3	0	3
GRMZM2G145827	5	10796873	10804954	40	10	9	10	GRMZM2G145850	5	10796396	10797296	15	0	0	0
GRMZM2G124863	5	19696842	19699376	91	0	1	1	GRMZM2G124918	5	19698812	19702780	28	0	0	0
GRMZM2G132036	5	20929213	20934778	25	1	0	1	XLOC_023002	5	20929481	20930339	17	0	0	0
GRMZM2G012209	5	39895036	39906590	44	1	0	1	XLOC_025549	5	39904934	39908229	32	1	0	1
GRMZM2G080183	5	53425033	53427956	49	1	0	1	XLOC_023299	5	53425395	53426300	11	0	0	0
GRMZM2G088114	5	69340886	69351028	40	10	6	10	XLOC_025845	5	69341489	69343791	20	0	0	0
GRMZM2G164242	5	70805268	70812495	16	0	0	0	XLOC_023479	5	70804919	70806365	18	1	0	1
GRMZM2G139689	5	76410293	76415357	39	0	0	0	GRMZM2G139714	5	76413820	76418130	22	0	1	1
GRMZM2G117544	5	93551459	93555969	15	1	0	1	XLOC_023660	5	93551777	93552691	1	0	0	0
GRMZM2G012046	5	160693221	160699305	42	1	0	1	XLOC_026357	5	160692953	160693951	8	0	0	0
GRMZM5G876303	5	168572548	168575048	30	1	0	1	XLOC_024063	5	168573632	168574495	14	1	0	1
GRMZM2G303964	5	170543803	170545707	38	1	0	1	XLOC_024088	5	170545309	170545691	8	1	0	1
GRMZM2G303964	5	170543803	170545707	38	1	0	1	XLOC_024087	5	170544174	170544600	17	0	0	0
GRMZM2G118825	5	171317073	171318365	38	5	2	5	XLOC_024096	5	171317220	171318479	29	5	2	5
GRMZM2G119517	5	172851847	172858649	38	0	0	0	GRMZM2G119566	5	172849643	172852744	11	0	1	1
GRMZM2G023988	5	175731393	175736518	39	0	1	1	XLOC_024153	5	175735743	175736375	15	0	0	0
XLOC_026704	5	188881231	188884099	95	1	1	1	GRMZM2G169967	5	188877054	188881772	16	0	0	0
GRMZM2G117164	5	190526842	190528626	12	2	2	2	XLOC_024345	5	190528090	190528564	3	0	0	0
GRMZM5G840002	5	191419331	191422728	20	4	0	4	XLOC_026737	5	191419426	191419679	3	0	0	0
GRMZM2G074053	5	194144303	194146501	53	2	0	2	XLOC_026772	5	194144551	194146414	47	2	0	2
GRMZM2G325019	5	200487573	200491201	54	1	1	1	GRMZM2G325038	5	200490849	200492345	25	1	2	3
GRMZM2G101287	5	200990577	200993961	27	1	0	1	GRMZM2G101350	5	200993550	200995952	13	1	0	1
GRMZM2G170595	5	201723129	201726317	39	0	0	0	GRMZM2G170602	5	201721167	201723705	49	2	2	3
GRMZM2G009506	5	201992788	201996158	9	1	0	1	XLOC_026868	5	201995204	201995662	3	1	0	1
GRMZM2G113418	5	210349025	210351808	16	0	0	0	GRMZM2G414621	5	210350135	210352786	19	0	4	4
GRMZM2G154332	5	210524837	210526166	14	1	1	1	XLOC_024731	5	210525082	210525511	4	0	0	0
GRMZM2G058289	5	215100492	215104749	34	1	2	3	XLOC_027171	5	215101686	215102542	7	0	0	0
GRMZM5G862817	5	215314528	215316506	53	2	0	2	XLOC_027174	5	215315528	215316236	20	0	0	0
GRMZM2G078887	5	215406418	215407900	36	2	0	2	XLOC_024833	5	215406977	215407577	7	0	0	0
GRMZM2G013981	5	215883819	215887609	34	1	1	1	GRMZM5G894916	5	215886969	215893442	5	0	0	0
GRMZM2G098174	6	1280395	1288498	59	0	0	0	XLOC_029097	6	1280515	1281668	34	2	0	2
GRMZM2G136662	6	8112897	8136923	44	1	0	1	XLOC_027310	6	8116573	8118254	-	-	-	-
GRMZM2G448607	6	24279652	24282016	25	2	0	2	XLOC_027540	6	24281335	24281874	16	2	0	2
GRMZM2G155943	6	59237967	59293671	23	0	1	1	XLOC_029599	6	59260577	59261040	-	-	-	-
GRMZM2G308689	6	85681129	85683710	36	2	2	2	XLOC_027905	6	85683407	85683665	7	0	0	0
GRMZM2G150209	6	89061130	89064359	55	5	0	5	XLOC_029797	6	89061498	89062022	-	-	-	-
GRMZM2G150256	6	89066375	89071273	52	5	8	8	XLOC_027953	6	89068336	89068774	9	0	1	1
GRMZM2G150302	6	89081149	89085692	49	5	5	5	XLOC_027958	6	89084484	89085224	10	0	0	0
XLOC_028172	6	108236815	108241211	27	1	0	1	XLOC_029999	6	108237703	108238301	17	1	0	1
GRMZM2G108714	6	128235624	128237977	59	1	0	1	XLOC_030209	6	128236074	128237326	13	0	0	0
GRMZM2G339091	6	134280739	134285176	41	1	1	1	XLOC_028447	6	134284618	134285372	15	1	1	1
GRMZM2G423863	6	139641367	139643749	12	0	1	1	GRMZM2G423898	6	139642333	139643913	12	0	1	1
GRMZM2G093325	6	140914068	140915871	20	1	0	1	XLOC_028518	6	140914832	140915320	6	1	0	1
GRMZM2G479608	6	145511301	145516044	40	3	3	3	XLOC_028587	6	145512115	145515915	19	0	0	0
GRMZM2G125034	6	153977792	153980864	62	1	0	1	XLOC_030599	6	153980084	153981320	22	1	0	1
GRMZM2G125034	6	153977792	153980864	62	1	0	1	XLOC_030598	6	153978666	153979619	19	0	0	0
GRMZM2G440968	6	155861586	155862536	30	0	0	0	GRMZM2G440949	6	155855927	155862458	42	1	0	1
GRMZM2G078781	6	157072612	157077219	51	1	0	1	XLOC_030671	6	157074323	157077111	17	1	0	1
AC206788.3_FG01[illegible]	6	160084248	160087779	83	1	0	1	GRMZM2G058314	6	160084716	160087818	49	0	0	0
GRMZM2G004932	6	160229718	160235410	39	1	1	2	XLOC_028869	6	160233892	160235240	7	0	0	0
GRMZM2G132212	6	160429758	160434506	73	20	23	23	GRMZM2G432801	6	160433610	160439979	41	13	14	14
GRMZM2G158972	6	161413828	161420521	12	1	1	1	XLOC_028899	6	161419537	161420979	2	0	0	0
GRMZM2G179797	6	162746428	162748871	42	2	1	2	XLOC_030794	6	162746423	162747799	24	2	1	2
XLOC_028986	6	164757065	164759753	31	1	0	1	XLOC_030842	6	164757491	164758850	30	1	0	1
GRMZM2G020281	7	955586	961591	23	5	4	6	XLOC_032540	7	955649	956597	6	0	0	0
GRMZM2G132568	7	9547566	9551755	40	1	0	1	XLOC_031066	7	9551283	9551687	13	0	0	0
GRMZM2G030138	7	10578360	10580594	43	2	0	2	GRMZM2G328171	7	10578984	10581443	19	0	0	0
GRMZM2G340656	7	19063721	19067235	78	2	5	5	XLOC_032747	7	19063747	19066238	34	2	5	5
GRMZM2G019183	7	107954326	107958837	81	2	0	2	XLOC_033183	7	107955577	107958837	65	2	0	2
GRMZM2G129018	7	115719658	115722055	26	2	0	2	XLOC_031629	7	115719873	115722227	22	0	0	0
GRMZM2G073504	7	141681145	141690742	41	1	1	1	GRMZM2G073377	7	141680690	141681890	26	1	1	1
GRMZM2G052610	7	141917927	141921808	8	0	0	0	GRMZM2G052509	7	141921207	141924166	18	0	2	2
GRMZM2G325008	7	152431465	152438328	56	3	1	3	XLOC_033649	7	152431276	152433930	44	3	1	3

GRMZM2G357631	7	154224100	154225746	31	2	3	4	GRMZM2G357636	7	154221509	154226616	-	-	-	-
GRMZM2G117707	7	166502692	166506595	41	2	2	2	XLOC_032314	7	166504385	166505811	-	-	-	-
GRMZM2G017145	7	167541463	167544286	44	1	1	1	AC195864.3_FG01	7	167539708	167541966	4	0	0	0
GRMZM2G141322	7	167674297	167675623	17	1	0	1	XLOC_033929	7	167674835	167675315	8	0	0	0
GRMZM2G356894	7	170074356	170080041	31	0	0	0	GRMZM2G055607	7	170071170	170074488	32	2	0	2
GRMZM2G025992	7	171775066	171778176	23	2	3	3	XLOC_032429	7	171776631	171778079	12	2	2	2
GRMZM2G050325	8	3921958	3925143	31	3	6	6	XLOC_036031	8	3921958	3923002	12	1	1	1
GRMZM2G375159	8	18444281	18446241	43	0	2	2	XLOC_034371	8	18444771	18445411	8	0	0	0
GRMZM2G004182	8	22175678	22180874	33	0	0	0	GRMZM2G004083	8	22180368	22182467	34	1	0	1
GRMZM2G045664	8	74456610	74457885	31	1	0	1	XLOC_034712	8	74456822	74457598	22	0	0	0
GRMZM2G116238	8	109359888	109361490	17	0	1	1	XLOC_034971	8	109361001	109361451	8	0	0	0
GRMZM2G062555	8	119542597	119544810	33	1	0	1	XLOC_035083	8	119544238	119544607	2	0	0	0
GRMZM2G456835	8	135307939	135312198	54	8	0	8	XLOC_037089	8	135310261	135312197	37	8	0	8
GRMZM5G803433	8	142537765	142543929	5	0	0	0	GRMZM2G117742	8	142535900	142538931	26	1	1	1
GRMZM2G144028	8	151441785	151450139	96	1	1	1	GRMZM2G144021	8	151438263	151442577	54	0	0	0
GRMZM2G144028	8	151441785	151450139	96	1	1	1	XLOC_035491	8	151442987	151444263	16	1	1	1
GRMZM2G010065	8	159534577	159539292	59	6	5	6	GRMZM2G010176	8	159531387	159535110	24	0	0	0
GRMZM2G470942	8	162335276	162337118	22	1	1	1	XLOC_037473	8	162335899	162340140	41	1	1	1
GRMZM2G100318	8	163128903	163133067	37	12	8	12	XLOC_037489	8	163131948	163132447	15	1	1	1
GRMZM2G396248	8	166450330	166453602	20	2	2	2	XLOC_037564	8	166450439	166452505	20	2	2	2
GRMZM2G123394	8	166704257	166706406	44	1	1	1	XLOC_037576	8	166705309	166706025	14	1	1	1
GRMZM2G147544	8	166741560	166743934	57	2	1	2	XLOC_035780	8	166742338	166743981	26	2	1	2
GRMZM5G840487	8	169068643	169073066	141	2	3	4	XLOC_037635	8	169071358	169072345	41	0	0	0
GRMZM2G066755	8	169175095	169178292	70	11	7	12	XLOC_037640	8	169175204	169175643	20	11	6	11
GRMZM2G036448	8	169675588	169681153	49	0	1	1	XLOC_037654	8	169676228	169679455	6	0	1	1
AC218972.3_FG004	8	170197942	170201141	21	1	0	1	XLOC_035858	8	170198174	170198543	3	1	0	1
GRMZM2G476009	8	170885667	170888437	44	6	6	6	XLOC_037681	8	170885781	170886008	4	0	0	0
GRMZM2G529313	9	16689343	16701067	37	0	0	0	GRMZM2G075488	9	16685283	16689432	21	0	1	1
AC231745.1_FG003	9	18335677	18338579	20	0	0	0	GRMZM5G838414	9	18328876	18337267	44	2	0	2
GRMZM2G103137	9	18885308	18888376	23	2	0	2	GRMZM2G020216	9	18879285	18887000	5	0	0	0
GRMZM2G136300	9	75178361	75189267	38	1	0	1	XLOC_038391	9	75188193	75188547	-	-	-	-
GRMZM2G141288	9	97192447	97196646	37	2	3	3	XLOC_040016	9	97195479	97195816	6	0	0	0
GRMZM2G103647	9	109162497	109164977	33	1	0	1	XLOC_038612	9	109164376	109164863	8	0	0	0
GRMZM2G095302	9	109332530	109337572	13	0	0	0	GRMZM2G095287	9	109336954	109340083	18	4	0	4
GRMZM2G144818	9	113509124	113515269	46	3	0	3	GRMZM2G144841	9	113514930	113517932	44	3	0	3
GRMZM2G152908	9	122479087	122485767	71	0	2	2	XLOC_038769	9	122479195	122485674	66	0	1	1
GRMZM2G046601	9	146319637	146328720	7	0	3	3	XLOC_040634	9	146326185	146327327	5	0	3	3
GRMZM2G004377	9	149513489	149519493	77	6	1	6	GRMZM2G004466	9	149512257	149513861	27	0	0	0
GRMZM2G178787	9	151538905	151543176	75	3	3	4	GRMZM2G178826	9	151542239	151546401	76	7	7	9
GRMZM2G127072	9	151918650	151921069	24	1	0	1	XLOC_040778	9	151919604	151920602	18	1	0	1
GRMZM2G126682	9	151998333	152000250	50	3	0	3	XLOC_040785	9	151998593	151999043	5	1	0	1
GRMZM2G126682	9	151998333	152000250	50	3	0	3	XLOC_040786	9	151999349	152000146	39	1	0	1
GRMZM2G010017	9	152081582	152086034	83	0	1	1	XLOC_040788	9	152081653	152082635	28	0	0	0
GRMZM2G393334	9	154723964	154729849	64	0	0	0	GRMZM2G092497	9	154729257	154731780	31	0	1	1
GRMZM2G151903	10	2435439	2439171	62	2	1	2	XLOC_007601	10	2436111	2437872	24	2	1	2
GRMZM2G022318	10	4614582	4617945	71	4	0	4	XLOC_007662	10	4616867	4617850	21	0	0	0
GRMZM2G434170	10	5225476	5228126	57	1	0	1	XLOC_007686	10	5226560	5227949	41	0	0	0
GRMZM2G464328	10	5645825	5646958	13	0	0	0	GRMZM2G164113	10	5645510	5651577	34	1	0	1
GRMZM2G110145	10	77282595	77289172	135	1	0	1	XLOC_008118	10	77287726	77288880	11	0	0	0
XLOC_008140	10	79422406	79423830	27	4	0	4	XLOC_006681	10	79422812	79423830	24	4	0	4
XLOC_008141	10	79424059	79424752	13	1	1	1	XLOC_006682	10	79424059	79424883	13	1	1	1
GRMZM2G019246	10	99220164	99223049	51	1	0	1	XLOC_006863	10	99220564	99221170	8	0	0	0
GRMZM2G000261	10	128580332	128582775	9	1	1	1	XLOC_007148	10	128580520	128581374	12	1	1	1
GRMZM5G893444	10	131910570	131914628	33	1	1	2	XLOC_007189	10	131913905	131914803	6	0	0	0
GRMZM2G046952	10	132120374	132122335	45	2	0	2	XLOC_007197	10	132121098	132121375	4	0	0	0
GRMZM2G431504	10	143178859	143181050	1	0	1	1	XLOC_008824	10	143178937	143179364	-	-	-	-
GRMZM5G895313	10	143487116	143488331	32	1	0	1	XLOC_008827	10	143487540	143488048	14	0	0	0
GRMZM5G818977	10	143740959	143744585	110	10	0	10	XLOC_008830	10	143743661	143744585	8	0	0	0
GRMZM2G085236	10	144340337	144347928	61	15	11	15	XLOC_007429	10	144346275	144346895	5	2	2	2
GRMZM2G150286	10	147043160	147045718	23	1	0	1	XLOC_007494	10	147045247	147045660	8	0	0	0
GRMZM2G142757	10	147855454	147859264	50	1	0	1	XLOC_008935	10	147857896	147859019	21	0	0	0
GRMZM2G142820	10	147868922	147872174	24	2	2	2	XLOC_007519	10	147871601	147872416	2	0	0	0
GRMZM2G106792	10	148253993	148255416	31	2	0	2	XLOC_008952	10	148254333	148255179	21	2	0	2
GRMZM2G104613	10	148407014	148410710	19	0	0	0	GRMZM2G104658	10	148410223	148412237	37	3	0	3

Supplemental Table 7. Natural variation in NAT gene pairs genes associated with grain yield, plant height and anthesis date under drought stress in 19 maize bi-parental populations

Sense Transcripts								Antiense Transcripts							
Gene Name	Chromosome	Start (bp)	End (bp)	SNP number	Significant SNP number (P<0.01)			Gene Name	Chromosome	Start (bp)	End (bp)	SNP number	Significant SNP number (P<0.01)		
					Anthesis Date	Plant Height	Grain Yield						Anthesis Date	Plant Height	Grain Yield
GRMZM2G032104	1	645,004	652,666	19	7	9	7	XLOC_000012	1	644,614	645,991	11	2	3	3
GRMZM2G172183	1	979,720	991,166	9	2	3	1	XLOC_003145	1	988,460	990,951	9	2	3	1
GRMZM2G374779	1	999,326	1,005,012	18	3	3	2	GRMZM2G374812	1	1,002,079	1,004,790	20	1	2	1
GRMZM5G802392	1	2,839,306	2,840,779	1	1	1	1	XLOC_003174	1	2,839,365	2,840,264	2	2	2	2
GRMZM2G015384	1	2,873,956	2,894,310	24	3	4	1	GRMZM2G007025	1	2,871,385	2,875,166	9	2	3	3
XLOC_003179	1	3,127,903	3,129,982	12	3	6	3	XLOC_000050	1	3,125,558	3,129,266	12	3	6	3
XLOC_000052	1	3,128,229	3,130,830	12	3	6	3	XLOC_003179	1	3,127,903	3,129,982	12	3	6	3
XLOC_000052	1	3,128,229	3,130,830	12	3	6	3	XLOC_003180	1	3,130,242	3,130,740	-	-	-	-
GRMZM2G055698	1	3,462,290	3,464,383	29	7	5	7	XLOC_003189	1	3,462,444	3,462,724	11	4	3	5
GRMZM2G176840	1	4,758,424	4,766,151	22	9	9	9	XLOC_000098	1	4,765,059	4,766,393	16	10	10	10
GRMZM2G026223	1	4,855,668	4,871,367	24	4	7	8	XLOC_000099	1	4,855,614	4,857,299	24	4	7	8
GRMZM2G159477	1	5,039,291	5,042,641	31	5	10	8	XLOC_003216	1	5,038,799	5,040,058	25	5	6	8
GRMZM2G327059	1	5,080,787	5,086,610	109	32	34	30	XLOC_003218	1	5,084,935	5,086,394	18	5	5	1
XLOC_000106	1	5,238,161	5,242,193	60	23	21	22	XLOC_003219	1	5,236,971	5,241,181	60	23	21	22
GRMZM2G176548	1	5,435,030	5,436,495	4	1	1	1	XLOC_003222	1	5,435,129	5,436,156	4	1	1	1
GRMZM2G032409	1	6,213,707	6,218,417	13	5	4	5	XLOC_000123	1	6,217,891	6,218,279	-	-	-	-
GRMZM2G409976	1	7,078,142	7,082,865	27	7	7	5	GRMZM2G109509	1	7,079,988	7,085,969	61	17	23	25
GRMZM2G095968	1	7,353,118	7,354,749	33	9	12	15	XLOC_003263	1	7,353,850	7,354,627	16	3	4	4
XLOC_003266	1	7,560,304	7,561,013	-	-	-	-	GRMZM2G110345	1	7,555,836	7,560,949	3	2	2	1
GRMZM2G110616	1	7,563,041	7,565,951	43	17	15	14	GRMZM2G410991	1	7,561,410	7,564,192	28	8	6	7
GRMZM2G075701	1	8,025,073	8,027,145	7	2	3	2	XLOC_003278	1	8,025,460	8,025,864	-	-	-	-
GRMZM2G475168	1	8,093,438	8,094,458	8	1	1	2	GRMZM2G176523	1	8,093,433	8,097,320	18	8	6	9
GRMZM2G041899	1	9,117,659	9,120,601	38	19	21	23	XLOC_003296	1	9,117,974	9,120,417	38	19	21	23
GRMZM5G823004	1	9,293,832	9,296,953	10	3	5	5	XLOC_003298	1	9,296,313	9,296,741	10	3	5	5
GRMZM2G415390	1	9,575,028	9,576,757	19	8	7	5	GRMZM2G415343	1	9,575,785	9,581,046	12	5	6	3
GRMZM2G086163	1	11,473,558	11,475,611	44	20	19	16	XLOC_000210	1	11,473,930	11,474,971	43	20	19	16
GRMZM2G000749	1	12,096,409	12,104,425	23	6	6	3	XLOC_003336	1	12,100,693	12,101,201	-	-	-	-
GRMZM2G377780	1	12,172,623	12,177,552	40	20	14	15	GRMZM2G377787	1	12,173,174	12,174,065	9	1	1	1
GRMZM2G472266	1	12,251,757	12,253,695	29	13	13	9	XLOC_000228	1	12,252,948	12,254,065	31	11	16	10
GRMZM2G162749	1	14,850,090	14,853,017	48	19	15	12	XLOC_003373	1	14,851,376	14,852,775	21	11	5	3
GRMZM2G139963	1	14,958,183	14,960,872	85	22	21	14	XLOC_000274	1	14,960,091	14,960,882	19	0	0	0
GRMZM2G035243	1	15,168,071	15,171,112	10	7	6	7	XLOC_003383	1	15,168,080	15,168,574	-	-	-	-
GRMZM2G156486	1	15,989,656	15,994,613	32	15	12	14	XLOC_003390	1	15,991,596	15,992,246	-	-	-	-
GRMZM2G012216	1	16,199,110	16,209,371	1	1	1	1	XLOC_000289	1	16,202,286	16,202,881	-	-	-	-
GRMZM2G175164	1	16,467,715	16,473,343	35	14	15	14	XLOC_003399	1	16,472,162	16,473,053	-	-	-	-
GRMZM2G129674	1	17,459,873	17,462,949	8	7	4	3	XLOC_000309	1	17,462,417	17,462,811	2	1	2	1
AC206951.3_FG016	1	17,518,667	17,520,030	4	0	2	1	XLOC_000312	1	17,519,366	17,520,530	-	-	-	-
GRMZM2G109589	1	17,713,154	17,715,952	24	11	11	7	XLOC_003415	1	17,715,428	17,716,202	-	-	-	-
GRMZM2G148706	1	18,983,737	18,985,312	9	6	6	2	XLOC_000340	1	18,984,003	18,985,420	6	6	3	2
GRMZM2G006631	1	19,098,935	19,101,431	29	10	12	13	XLOC_003427	1	19,099,356	19,099,730	-	-	-	-
GRMZM2G154007	1	19,326,432	19,329,023	52	19	22	22	XLOC_000345	1	19,327,983	19,328,287	6	4	4	3
GRMZM2G464510	1	21,247,887	21,250,509	19	2	4	3	GRMZM2G464515	1	21,248,981	21,251,201	2	0	0	0
GRMZM2G006977	1	22,378,485	22,384,451	26	6	8	8	XLOC_000380	1	22,383,382	22,384,394	14	3	4	4
GRMZM2G077127	1	22,435,812	22,439,519	21	9	8	8	XLOC_000381	1	22,437,196	22,438,908	21	9	8	8
GRMZM2G055101	1	22,638,292	22,642,889	37	12	11	19	XLOC_003467	1	22,642,061	22,642,416	-	-	-	-
GRMZM2G055217	1	22,645,597	22,650,341	9	2	1	6	GRMZM2G055172	1	22,643,460	22,647,118	-	-	-	-
GRMZM2G159632	1	24,403,021	24,407,908	23	2	0	3	XLOC_000403	1	24,403,483	24,407,584	20	2	0	3
GRMZM2G163154	1	27,099,652	27,108,024	33	14	9	9	GRMZM2G163129	1	27,095,059	27,100,615	-	-	-	-
AC208897.3_FG004	1	27,125,856	27,128,302	45	9	10	11	XLOC_000429	1	27,125,995	27,126,318	3	0	0	0
GRMZM2G091456	1	29,400,571	29,405,558	24	9	8	5	XLOC_003535	1	29,400,429	29,401,250	12	3	3	2
GRMZM2G153536	1	29,512,941	29,516,697	33	10	15	14	XLOC_000463	1	29,513,532	29,516,007	22	5	10	8
GRMZM2G126808	1	29,540,914	29,544,577	24	9	9	8	XLOC_000465	1	29,543,125	29,543,575	5	3	2	2
GRMZM2G012501	1	30,758,187	30,767,144	7	3	3	3	GRMZM2G012758	1	30,748,051	30,758,493	13	3	5	3
GRMZM2G116902	1	31,253,767	31,255,665	41	16	9	13	XLOC_000483	1	31,254,138	31,254,520	7	5	5	5
GRMZM2G152663	1	32,095,243	32,098,991	45	18	10	14	XLOC_003560	1	32,097,153	32,097,436	5	3	2	3
GRMZM2G099528	1	32,322,594	32,326,736	7	2	1	1	GRMZM5G884325	1	32,326,220	32,333,213	3	1	1	1
GRMZM2G077942	1	32,873,395	32,876,087	89	36	38	31	XLOC_000510	1	32,875,353	32,875,768	2	1	1	0
GRMZM2G138103	1	34,974,187	34,975,255	8	2	5	3	XLOC_000539	1	34,974,445	34,975,174	7	2	4	2
GRMZM2G338702	1	36,160,110	36,165,881	6	0	0	0	AC214040.3_FG009	1	36,160,144	36,162,528	37	12	13	14
GRMZM2G060148	1	36,224,583	36,227,464	58	23	29	27	XLOC_000550	1	36,225,101	36,227,564	52	18	25	22
GRMZM2G128206	1	38,621,544	38,623,079	20	5	8	9	XLOC_003643	1	38,621,448	38,622,702	20	5	8	9
GRMZM2G160506	1	39,115,144	39,118,333	39	6	10	8	XLOC_000584	1	39,117,344	39,118,213	11	0	2	2
XLOC_003652	1	39,144,013	39,146,199	39	11	13	10	XLOC_000585	1	39,144,595	39,146,126	39	11	13	10
GRMZM2G179432	1	40,062,759	40,065,030	13	8	8	6	XLOC_003663	1	40,064,443	40,065,015	-	-	-	-
GRMZM2G048482	1	41,279,818	41,283,349	41	17	21	19	XLOC_003681	1	41,279,940	41,283,039	45	18	22	19
GRMZM2G043878	1	41,440,932	41,442,284	16	3	2	2	XLOC_000621	1	41,441,234	41,441,685	2	0	0	0
GRMZM2G119886	1	41,763,869	41,767,021	4	2	2	1	XLOC_000625	1	41,766,299	41,766,912	2	1	1	1
GRMZM2G442277	1	42,849,835	42,854,421	43	20	23	17	GRMZM2G141704	1	42,851,939	42,855,499	50	22	26	20
GRMZM2G017792	1	43,690,068	43,692,986	28	11	11	13	XLOC_000648	1	43,692,630	43,692,886	-	-	-	-
AC212219.3_FG005	1	44,959,567	44,965,488	27	13	10	11	XLOC_000662	1	44,959,729	44,965,076	27	13	10	11
GRMZM2G053669	1	45,119,006	45,122,764	17	5	4	5	XLOC_000663	1	45,119,161	45,120,699	6	2	2	2
AC208201.3_FG002	1	45,370,514	45,373,009	6	3	3	2	XLOC_003727	1	45,371,092	45,372,046	1	1	1	0
GRMZM2G087186	1	45,517,326	45,520,489	42	17	14	12	XLOC_000672	1	45,519,821	45,520,211	2	2	2	2
GRMZM5G850640	1	45,637,463	45,640,713	10	3	3	2	XLOC_003731	1	45,640,504	45,641,402	5	0	0	0
GRMZM2G007206	1	45,953,216	45,954,617	11	6	6	5	XLOC_000682	1	45,954,234	45,954,542	1	0	0	0
GRMZM2G144224	1	46,236,692	46,238,160	14	7	6	7	XLOC_000689	1	46,236,163	46,237,825	16	8	7	8
GRMZM2G010545	1	46,844,940	46,848,551	46	21	21	21	GRMZM2G313934	1	46,845,162	46,845,951	28	13	12	11
GRMZM2G016734	1	47,329,448	47,331,142	5	2	0	1	XLOC_000708	1	47,330,719	47,330,940	-	-	-	-
GRMZM2G377215	1	47,508,955	47,511,718	15	6	8	6	XLOC_003761	1	47,510,283	47,511,240	4	2	2	1
GRMZM2G318652	1	51,892,116	51,893,672	17	9	7	8	XLOC_003796	1	51,891,410	51,893,253	13	6	5	6
GRMZM2G009060	1	52,392,189	52,395,169	2	1	0	0	XLOC_000765	1	52,390,956	52,393,884	16	5	4	5
GRMZM2G033570	1	52,561,965	52,565,938	21	7	5	5	XLOC_000769	1	52,562,240	52,564,454	5	1	1	1
GRMZM2G312661	1	54,602,876	54,605,395	4	1	3	1	XLOC_000790	1	54,604,498	54,604,949	4	1	3	1
GRMZM2G064814	1	57,893,672	57,900,344	6	4	5	4	XLOC_003874	1	57,893,719	57,895,045	10	4	8	7
GRMZM2G467072	1	60,155,734	60,157,039	17	11	11	11	XLOC_003898	1	60,155,654	60,156,604	31	14	14	14
AC204530.4_FG002	1	61,615,166	61,623,614	6	2	1	2	AC204530.4_FG003	1	61,623,111	61,624,072	27	9	11	6
GRMZM2G081322	1	61,934,146	61,944,291	20	11	9	11	XLOC_003919	1	61,943,533	61,944,858	15	8	7	9
GRMZM5G822100	1	62,561,406	62,563,927	13	6	6	5	XLOC_003931	1	62,561,354	62,562,760	-	-	-	-
GRMZM2G168665	1	62,678,621	62,679,843	34	7	6	10	XLOC_003932	1	62,678,619	62,679,687	32	6	6	9
GRMZM2G140989	1	62,719,076	62,721,689	10	3	1	3	XLOC_003934	1	62,719,153	62,720,477	10	3	1	3
GRMZM2G026417	1	64,021,077	64,022,794	17	6	7	5	XLOC_000900	1	64,021,299	64,021,898	17	6	7	5
GRMZM2G179792	1	67,777,631	67,781,637	27	13	10	8	XLOC_003969	1	67,780,993	67,781,504	-	-	-	-
GRMZM2G382717	1	68,121,169	68,122,907	13	4	4	6	XLOC_000949	1	68,121,352	68,121,966	-	-	-	-
GRMZM2G077897	1	68,568,918	68,572,988	21	11	10	11	XLOC_000952	1	68,568,365	68,569,907	9	6	4	5
GRMZM2G022061	1	68,883,902	68,889,980	5	2	2	1	XLOC_003977	1	68,887,110	68,887,325	-	-	-	-
GRMZM2G049070	1	69,295,636	69,297,279	27	12	10	7	XLOC_003979	1	69,295,619	69,296,188	7	4	2	1
GRMZM2G071688	1	70,582,852	70,585,097	16	0	2	0	XLOC_003987	1	70,583,867	70,585,080	15	0	2	0
GRMZM2G142390	1	70,775,839	70,780,013	49	15	17	21	XLOC_000969	1	70,779,206	70,780,258	26	10	9	14
XLOC_003999	1	71,221,941	71,223,924	23	9	5	8	XLOC_000973	1	71,222,469	71,223,408	12	4	4	3
GRMZM2G304442	1	74,718,500	74,719,924	7	1	1	0	XLOC_001011	1	74,719,104	74,719,886	7	1	1	0
GRMZM2G084587	1	76,878,489	76,889,924	5	1	2	0	GRMZM2G385050	1	76,889,570	76,904,210	9	1	2	0
GRMZM2G157164	1	77,040,208	77,041,589	41	19	23	21	XLOC_004055	1	77,040,876	77,041,414	22	8	9	12
GRMZM2G044902	1	77,152,281	77,154,119	31	15	13	12	XLOC_001033	1	77,153,673	77,153,979	3	1	1	1
GRMZM2G024175	1	78,064,391	78,065,838	2	2	2	0	XLOC_001037	1	78,064,712	78,065,532	2	2	2	0
GRMZM5G826389	1	79,336,931	79,338,465	34	10	11	12	XLOC_004082	1	79,336,553	79,338,285	34	10	11	12
GRMZM2G143703	1	80,173,019	80,176,153	38	18	11	9	GRMZM6G399977	1	80,175,428	80,193,276	-	-	-	-
GRMZM2G025190	1	81,450,983	81,452,132	35	12	10	8	XLOC_004102	1	81,451,571	81,452,080	9	5	6	4
GRMZM2G302373	1	81,559,325	81,560,291	24	11	10	7	XLOC_004104	1	81,559,953	81,560,235	-	-	-	-

GRMZM2G178079	1	81,588,714	81,589,906	9	5	6	5	XLOC_004105	1	81,589,129	81,589,704	9	5	6	5
GRMZM2G028821	1	81,612,706	81,613,830	8	8	1	0	XLOC_001068	1	81,612,836	81,613,690	8	8	1	0
GRMZM2G026147	1	85,027,826	85,029,741	46	15	18	14	XLOC_004131	1	85,029,194	85,029,701	-	-	-	-
GRMZM2G453832	1	85,998,283	86,001,830	25	8	9	8	GRMZM2G154549	1	86,001,434	86,004,907	12	4	4	4
GRMZM2G079470	1	88,487,882	88,491,179	27	9	7	8	XLOC_001134	1	88,490,036	88,491,131	1	0	0	0
GRMZM2G089248	1	89,004,748	89,006,489	10	2	1	1	XLOC_004165	1	89,004,822	89,006,240	10	2	1	1
GRMZM2G317386	1	90,797,641	90,799,416	31	7	12	7	XLOC_004174	1	90,798,196	90,800,178	20	3	7	2
GRMZM2G114772	1	92,248,217	92,252,805	41	16	15	19	GRMZM2G114707	1	92,250,389	92,253,227	31	11	12	10
GRMZM2G094639	1	92,473,694	92,474,849	19	7	8	7	XLOC_004184	1	92,473,871	92,474,678	19	7	8	7
GRMZM2G161459	1	92,793,547	92,797,895	55	17	22	22	XLOC_001161	1	92,794,480	92,794,761	1	0	0	0
GRMZM2G088524	1	94,375,915	94,377,975	24	7	7	8	XLOC_004191	1	94,376,624	94,377,755	10	3	0	3
GRMZM2G178576	1	94,691,673	94,693,914	12	5	8	4	XLOC_001172	1	94,692,189	94,692,906	7	2	6	2
GRMZM2G007939	1	95,197,725	95,201,525	32	10	10	7	XLOC_001182	1	95,199,285	95,201,045	10	3	3	2
AC194914.3_FG002	1	99,546,686	99,548,327	2	2	2	2	XLOC_001211	1	99,547,343	99,547,922	2	2	2	2
GRMZM5G866843	1	103,834,561	103,838,408	24	5	6	6	GRMZM2G089147	1	103,831,944	103,835,466	3	0	0	0
GRMZM2G012306	1	106,902,765	106,905,799	69	15	26	14	XLOC_004251	1	106,903,378	106,904,918	40	9	14	11
AC211467.4_FG002	1	126,123,363	126,124,398	5	1	1	1	XLOC_004341	1	126,122,680	126,124,282	5	1	1	1
GRMZM2G167584	1	139,799,347	139,801,075	4	2	3	2	GRMZM2G167649	1	139,800,143	139,801,008	4	2	3	2
GRMZM2G159587	1	140,725,406	140,729,780	14	4	1	3	XLOC_001351	1	140,729,075	140,729,880	10	3	0	2
GRMZM2G479885	1	147,170,692	147,172,458	13	2	2	2	XLOC_004417	1	147,170,825	147,172,007	13	2	2	2
GRMZM2G056039	1	154,092,672	154,096,549	15	8	7	7	XLOC_004448	1	154,092,677	154,096,775	15	8	7	7
GRMZM5G878732	1	161,322,162	161,324,184	49	25	29	24	XLOC_001442	1	161,322,448	161,323,383	39	19	22	18
GRMZM2G546081	1	162,442,194	162,449,008	35	12	12	12	GRMZM2G039112	1	162,442,172	162,444,014	27	9	10	9
GRMZM2G476637	1	162,478,590	162,481,146	25	11	11	11	XLOC_001451	1	162,480,410	162,480,689	3	2	2	2
GRMZM2G428216	1	165,420,861	165,422,748	10	5	2	6	XLOC_004506	1	165,420,894	165,421,789	10	5	2	6
GRMZM2G428216	1	165,420,861	165,422,748	10	5	2	6	GRMZM2G127739	1	165,422,123	165,427,760	33	15	13	14
GRMZM2G017486	1	166,943,082	166,945,351	21	9	11	11	XLOC_004512	1	166,942,218	166,945,246	23	12	12	13
GRMZM2G115615	1	170,979,650	170,986,759	20	2	3	5	XLOC_001519	1	170,986,484	170,986,768	1	0	0	0
GRMZM2G029262	1	172,141,002	172,147,261	3	1	1	1	GRMZM2G332821	1	172,138,864	172,141,926	20	9	9	8
GRMZM2G047835	1	174,235,528	174,238,895	30	16	20	21	XLOC_004575	1	174,236,215	174,238,681	7	2	6	5
GRMZM2G022365	1	175,322,870	175,330,028	53	14	10	13	AC212976.3_FG003	1	175,321,975	175,323,121	7	3	2	3
GRMZM2G149751	1	176,865,344	176,871,673	52	22	26	20	XLOC_004593	1	176,870,093	176,873,163	19	10	10	7
XLOC_004593	1	176,870,093	176,873,163	19	10	10	7	XLOC_001559	1	176,872,260	176,873,419	12	6	7	6
GRMZM2G080603	1	178,503,499	178,504,739	8	3	4	4	XLOC_001566	1	178,503,773	178,504,714	5	2	2	2
GRMZM5G893912	1	180,635,433	180,637,762	45	19	22	19	XLOC_001589	1	180,635,595	180,637,502	49	20	23	19
GRMZM2G310947	1	180,651,899	180,653,885	28	15	12	10	XLOC_001590	1	180,651,917	180,653,172	17	9	8	7
GRMZM2G423472	1	180,715,451	180,720,707	15	7	7	6	XLOC_004639	1	180,717,585	180,718,436	10	1	1	2
GRMZM2G023791	1	182,125,031	182,126,803	13	5	3	4	XLOC_001611	1	182,125,586	182,126,923	13	5	3	4
GRMZM2G179351	1	183,321,740	183,334,968	-	-	-	-	XLOC_004665	1	183,333,777	183,334,701	7	2	2	3
GRMZM2G042488	1	183,477,478	183,479,191	19	10	8	8	GRMZM2G042371	1	183,474,570	183,477,893	39	14	9	9
GRMZM2G083755	1	187,375,298	187,379,353	18	2	5	1	AC213857.4_FG003	1	187,378,034	187,378,819	-	-	-	-
GRMZM2G441903	1	187,432,586	187,435,072	13	4	7	6	XLOC_004709	1	187,433,111	187,434,213	12	4	7	6
GRMZM2G121312	1	187,917,195	187,919,314	19	6	8	4	XLOC_001677	1	187,917,466	187,919,447	19	6	8	4
GRMZM5G822593	1	188,147,934	188,153,498	17	10	12	7	XLOC_004719	1	188,151,948	188,153,420	6	4	4	2
GRMZM2G022499	1	188,491,054	188,494,924	23	8	7	9	XLOC_001686	1	188,493,609	188,494,765	7	4	4	4
GRMZM2G124557	1	189,385,201	189,390,321	11	4	6	6	GRMZM2G124638	1	189,389,542	189,393,722	3	2	2	2
GRMZM5G828396	1	192,065,376	192,068,429	32	23	23	10	XLOC_004751	1	192,065,391	192,067,850	30	23	23	10
GRMZM2G054252	1	195,218,148	195,221,731	63	17	23	15	XLOC_004779	1	195,220,012	195,220,946	21	9	8	7
GRMZM2G054252	1	195,218,148	195,221,731	63	17	23	15	XLOC_004778	1	195,218,610	195,219,235	28	6	10	6
GRMZM2G048129	1	196,084,489	196,086,931	42	12	17	14	XLOC_004788	1	196,085,024	196,086,807	42	12	17	14
GRMZM2G069476	1	196,458,606	196,463,550	71	28	26	27	XLOC_001785	1	196,458,881	196,460,106	12	3	3	3
GRMZM2G477872	1	197,104,152	197,113,073	42	17	24	19	XLOC_004800	1	197,110,037	197,113,267	14	4	6	4
GRMZM2G477872	1	197,104,152	197,113,073	42	17	24	19	XLOC_004799	1	197,105,380	197,106,330	-	-	-	-
GRMZM2G175423	1	197,301,278	197,304,421	20	9	12	12	XLOC_004803	1	197,301,191	197,303,333	14	6	8	10
GRMZM5G881887	1	199,788,867	199,791,107	29	12	11	9	XLOC_004833	1	199,789,794	199,790,838	17	9	7	6
GRMZM2G104938	1	199,871,050	199,872,477	8	4	5	1	XLOC_004834	1	199,871,006	199,872,115	1	1	1	0
GRMZM2G457309	1	200,504,395	200,505,328	11	2	4	2	XLOC_004841	1	200,504,383	200,506,023	13	3	5	3
GRMZM5G899800	1	201,502,172	201,509,140	12	4	5	4	XLOC_004849	1	201,502,572	201,502,846	5	2	1	1
GRMZM2G318794	1	201,668,843	201,669,743	3	0	0	1	XLOC_004853	1	201,668,928	201,669,258	3	0	0	1
GRMZM2G315375	1	202,334,807	202,342,249	64	20	23	19	XLOC_004857	1	202,340,036	202,340,725	5	1	1	1
GRMZM2G179703	1	202,955,781	202,958,669	26	8	5	4	XLOC_004868	1	202,955,781	202,956,669	14	3	0	0
GRMZM2G320689	1	204,903,793	204,910,161	38	9	10	8	GRMZM2G320708	1	204,909,826	204,911,008	16	5	5	3
GRMZM2G319357	1	205,287,282	205,292,061	33	10	10	8	AC212244.4_FG004	1	205,285,975	205,287,839	32	8	10	8
GRMZM2G448185	1	208,868,009	208,873,195	68	27	27	23	XLOC_001946	1	208,870,529	208,870,976	-	-	-	-
XLOC_004952	1	208,873,296	208,874,008	6	5	4	3	XLOC_001947	1	208,873,436	208,874,144	6	5	4	3
GRMZM2G131205	1	211,567,120	211,572,922	75	19	21	21	XLOC_004979	1	211,570,193	211,573,240	29	9	10	8
GRMZM2G167694	1	216,747,715	216,750,376	26	11	14	13	GRMZM2G167669	1	216,747,936	216,748,356	42	22	20	23
GRMZM2G142249	1	216,924,039	216,931,011	10	4	7	5	XLOC_005022	1	216,924,085	216,924,463	2	0	0	0
AC217910.3_FG004	1	217,331,974	217,333,074	24	6	7	6	XLOC_005026	1	217,332,510	217,332,972	12	1	3	2
GRMZM2G114093	1	218,469,216	218,473,811	20	4	5	5	XLOC_002039	1	218,473,265	218,474,165	3	0	0	1
GRMZM2G003426	1	218,852,692	218,853,654	20	5	5	6	XLOC_002042	1	218,852,913	218,853,736	20	5	5	6
GRMZM2G321010	1	219,952,350	219,953,886	2	0	0	0	GRMZM2G015925	1	219,944,821	219,953,265	9	4	4	4
GRMZM2G136439	1	220,147,969	220,150,934	36	9	21	21	XLOC_005054	1	220,148,133	220,148,722	33	6	19	19
GRMZM2G179063	1	221,239,135	221,240,968	15	4	4	3	XLOC_002070	1	221,239,260	221,241,115	15	4	4	3
GRMZM2G181236	1	223,849,323	223,851,220	20	11	11	9	XLOC_002108	1	223,849,490	223,850,364	19	10	10	8
XLOC_002116	1	224,091,063	224,091,982	-	-	-	-	XLOC_005098	1	224,090,231	224,091,493	21	10	8	7
GRMZM2G414727	1	224,437,062	224,438,028	7	3	3	3	XLOC_005105	1	224,436,921	224,438,023	7	3	3	3
GRMZM2G172451	1	224,482,341	224,485,355	6	2	2	2	GRMZM2G475956	1	224,484,800	224,485,876	18	8	3	8
GRMZM5G882228	1	226,230,810	226,234,051	53	24	14	14	XLOC_005127	1	226,230,644	226,231,367	19	5	4	4
GRMZM2G082529	1	227,894,328	227,899,992	25	9	10	6	XLOC_005176	1	227,894,417	227,895,044	5	3	3	2
GRMZM2G459291	1	229,666,605	229,670,274	-	-	-	-	GRMZM2G162758	1	229,664,358	229,667,449	9	4	5	2
GRMZM2G404443	1	230,073,669	230,077,848	17	6	4	6	XLOC_002182	1	230,075,799	230,077,837	11	2	5	3
GRMZM2G010362	1	231,541,834	231,575,479	40	6	9	9	XLOC_002196	1	231,574,753	231,575,534	-	-	-	-
GRMZM2G134747	1	233,290,453	233,293,630	27	11	11	6	XLOC_002206	1	233,293,225	233,293,591	15	8	7	3
GRMZM2G398198	1	233,875,216	233,877,958	23	7	6	6	XLOC_002210	1	233,875,715	233,878,017	23	7	6	6
GRMZM2G445905	1	234,322,570	234,326,740	48	19	23	17	XLOC_005257	1	234,325,510	234,325,858	2	0	0	0
XLOC_002220	1	234,403,006	234,404,583	7	4	4	4	XLOC_005260	1	234,403,212	234,403,769	7	4	4	4
GRMZM2G020295	1	235,420,397	235,425,098	7	1	1	1	XLOC_005283	1	235,420,172	235,420,870	1	0	0	0
XLOC_002237	1	235,769,118	235,771,246	-	-	-	-	XLOC_005293	1	235,769,492	235,776,073	17	8	4	2
GRMZM2G029518	1	235,922,074	235,923,485	4	0	1	1	XLOC_002238	1	235,922,447	235,923,497	4	0	1	1
GRMZM2G152901	1	237,009,950	237,014,064	40	16	14	14	XLOC_005311	1	237,009,966	237,013,206	40	16	14	14
GRMZM2G303965	1	237,545,346	237,548,821	11	6	3	4	GRMZM2G003724	1	237,544,777	237,546,713	2	1	0	0
GRMZM2G000540	1	238,877,136	238,886,130	13	4	4	4	XLOC_002264	1	238,877,518	238,878,461	11	4	2	3
GRMZM2G016241	1	241,430,778	241,431,741	26	11	11	4	XLOC_002293	1	241,430,167	241,431,867	26	11	11	4
GRMZM2G138455	1	242,766,570	242,768,953	38	9	16	7	XLOC_005351	1	242,767,148	242,768,825	27	6	13	5
GRMZM2G146589	1	246,761,616	246,769,046	14	2	7	0	GRMZM2G146486	1	246,768,933	246,782,639	20	10	8	3
GRMZM2G153887	1	248,481,668	248,482,632	8	0	0	0	GRMZM2G153928	1	248,480,724	248,509,681	17	2	1	1
GRMZM2G364069	1	250,153,484	250,156,801	43	8	10	9	XLOC_002389	1	250,153,600	250,156,741	39	6	8	6
GRMZM5G805732	1	251,500,979	251,505,855	21	7	9	6	XLOC_005428	1	251,503,379	251,503,728	-	-	-	-
GRMZM5G805732	1	251,500,979	251,505,855	21	7	9	6	XLOC_005429	1	251,503,953	251,504,683	11	4	5	3
GRMZM2G066067	1	253,370,904	253,375,697	22	6	7	7	XLOC_002420	1	253,373,863	253,375,695	22	6	7	7
GRMZM2G021055	1	253,775,521	253,780,778	45	14	16	13	XLOC_005451	1	253,775,607	253,776,040	15	5	3	3
GRMZM2G017319	1	255,383,820	255,386,002	28	10	10	7	XLOC_005472	1	255,383,820	255,384,076	4	1	1	0
GRMZM2G082686	1	255,803,441	255,804,699	26	9	10	7	XLOC_005475	1	255,803,514	255,804,904	26	9	10	7
GRMZM2G150772	1	256,131,686	256,145,103	53	11	10	10	XLOC_005479	1	256,142,498	256,143,727	13	2	3	3
GRMZM2G409093	1	257,374,641	257,384,724	4	1	1	1	GRMZM2G106552	1	257,384,553	257,386,539	-	-	-	-
GRMZM2G055037	1	258,031,281	258,033,115	70	27	27	23	XLOC_005500	1	258,031,736	258,032,238	24	10	9	9
GRMZM2G467893	1	260,803,289	260,806,517	2	1	0	0	XLOC_002505	1	260,804,712	260,806,407	2	1	0	0
GRMZM2G068575	1	261,178,421	261,192,543	2	1	1	1	XLOC_002507	1	261,191,923	261,192,453	-	-	-	-
GRMZM2G133684	1	261,723,724	261,726,331	7	1	2	1	GRMZM2G133633	1	261,720,652	261,725,055	14	3	2	3
GRMZM5G858417	1	263,064,702	263,068,643	44	17	16	13	XLOC_002528	1	263,063,270	263,066,541	22	10	10	8
GRMZM2G109056	1	264,275,003	264,290,616	16	3	4	3	XLOC_005585	1	264,275,079	264,275,767	-	-	-	-
GRMZM2G033787	1	264,577,362	264,580,333	27	9	8	4	XLOC_005587	1	264,579,109	264,580,330	-	-	-	-

XLOC_005589	1	264,593,352	264,595,682	14	9	9	6	XLOC_002553	1	264,594,695	264,595,635	8	4	4	4
GRMZM2G026780	1	270,898,020	270,899,100	4	1	0	0	XLOC_002634	1	270,898,329	270,898,894	4	1	0	0
GRMZM2G153292	1	271,178,054	271,180,939	1	1	1	1	XLOC_005675	1	271,179,186	271,180,879	1	1	1	1
GRMZM2G442658	1	274,050,247	274,053,994	4	1	1	1	XLOC_002674	1	274,052,049	274,053,751	1	1	1	1
GRMZM2G001869	1	274,785,474	274,790,748	16	3	9	1	XLOC_005725	1	274,789,368	274,790,183	3	1	2	1
GRMZM2G159295	1	275,052,364	275,059,001	46	20	16	10	XLOC_002694	1	275,058,388	275,058,797	9	1	2	2
GRMZM2G159285	1	275,098,368	275,101,121	16	7	8	7	XLOC_002696	1	275,094,121	275,099,076	27	10	14	6
GRMZM2G169671	1	275,290,498	275,294,240	18	8	6	7	XLOC_002701	1	275,293,889	275,294,213	-	-	-	-
GRMZM2G128564	1	275,694,757	275,696,881	36	11	12	13	XLOC_002702	1	275,694,984	275,696,172	22	9	11	10
GRMZM2G377131	1	275,759,689	275,761,549	19	10	7	10	XLOC_002707	1	275,759,900	275,761,423	16	9	6	9
GRMZM2G147687	1	276,371,897	276,377,338	41	18	22	13	XLOC_005755	1	276,371,474	276,372,465	19	3	4	3
GRMZM2G147687	1	276,371,897	276,377,338	41	18	22	13	XLOC_005756	1	276,375,849	276,377,038	4	2	3	1
GRMZM2G017186	1	276,389,960	276,395,246	56	33	37	31	XLOC_005758	1	276,393,398	276,393,847	-	-	-	-
GRMZM2G421491	1	276,882,941	276,887,539	24	15	12	14	GRMZM2G120484	1	276,876,174	276,883,112	14	3	5	3
GRMZM2G058032	1	277,055,488	277,057,838	4	1	1	1	XLOC_005776	1	277,056,474	277,057,254	3	0	0	0
GRMZM2G057935	1	277,059,425	277,064,749	22	8	8	6	XLOC_002732	1	277,063,140	277,064,199	12	3	3	2
GRMZM5G826714	1	277,446,604	277,450,109	14	6	6	8	XLOC_002739	1	277,446,460	277,450,131	14	6	6	8
GRMZM5G826714	1	277,446,604	277,450,109	14	6	6	8	XLOC_002740	1	277,449,320	277,450,131	8	3	3	4
GRMZM2G090422	1	277,805,999	277,807,838	10	3	3	4	XLOC_005786	1	277,806,426	277,807,762	-	-	-	-
GRMZM2G328500	1	278,196,221	278,199,359	8	5	2	2	XLOC_005789	1	278,197,373	278,199,188	2	2	2	2
GRMZM2G091578	1	279,251,625	279,254,873	14	6	7	6	XLOC_002767	1	279,251,712	279,254,882	26	8	10	9
GRMZM2G047759	1	279,541,752	279,543,297	13	11	4	9	XLOC_002770	1	279,542,062	279,545,350	20	10	9	9
GRMZM2G047456	1	279,632,338	279,634,477	56	14	12	15	XLOC_005809	1	279,633,156	279,634,477	11	4	2	2
GRMZM2G156800	1	280,154,367	280,163,933	41	9	11	10	XLOC_002778	1	280,163,123	280,164,102	10	1	2	1
GRMZM2G138861	1	280,356,771	280,364,944	4	1	1	2	XLOC_005819	1	280,356,887	280,357,232	-	-	-	-
XLOC_002789	1	280,846,114	280,846,636	-	-	-	-	XLOC_005828	1	280,845,925	280,846,624	4	1	0	1
XLOC_005831	1	280,894,123	280,894,576	15	9	10	8	GRMZM2G072814	1	280,894,065	280,895,067	15	9	10	8
GRMZM2G028286	1	281,711,998	281,716,153	33	10	9	12	XLOC_002799	1	281,714,919	281,715,898	7	1	3	2
GRMZM2G427697	1	281,976,636	281,978,946	17	10	11	7	XLOC_002804	1	281,977,000	281,979,054	4	1	1	2
GRMZM2G165709	1	282,015,181	282,016,843	21	6	7	7	XLOC_002805	1	282,015,389	282,016,975	11	0	1	0
GRMZM2G040145	1	285,280,649	285,281,991	19	8	7	10	XLOC_002839	1	285,281,158	285,281,565	-	-	-	-
GRMZM2G308687	1	285,410,446	285,411,691	38	17	13	20	XLOC_002841	1	285,410,613	285,412,021	38	17	13	20
GRMZM2G414540	1	285,855,288	285,857,095	8	1	1	1	XLOC_005910	1	285,855,548	285,856,928	8	1	1	1
GRMZM2G329181	1	286,049,844	286,053,001	11	8	7	4	GRMZM2G028926	1	286,045,297	286,050,143	24	6	7	7
GRMZM2G019799	1	286,429,050	286,437,267	43	21	16	24	XLOC_002860	1	286,432,765	286,437,028	18	7	6	8
GRMZM2G019799	1	286,429,050	286,437,267	43	21	16	24	XLOC_002859	1	286,428,657	286,429,831	10	2	2	4
GRMZM2G060765	1	286,736,778	286,741,262	4	1	1	1	GRMZM2G060924	1	286,730,567	286,737,443	1	1	1	1
GRMZM2G120674	1	286,916,349	286,921,522	16	6	7	8	GRMZM2G120657	1	286,920,950	286,923,692	38	17	20	14
GRMZM2G005991	1	287,216,930	287,218,310	37	9	13	14	XLOC_002873	1	287,217,889	287,218,154	10	3	4	4
GRMZM2G178415	1	287,361,393	287,367,874	43	16	20	20	XLOC_002877	1	287,360,747	287,361,598	3	1	2	2
GRMZM2G178415	1	287,361,393	287,367,874	43	16	20	20	XLOC_002878	1	287,361,426	287,363,549	19	3	4	4
XLOC_005938	1	287,541,417	287,542,365	9	3	3	3	GRMZM2G170851	1	287,541,494	287,544,013	9	3	3	3
GRMZM2G070322	1	287,652,900	287,655,520	25	9	10	9	XLOC_002882	1	287,653,653	287,655,436	25	9	10	9
GRMZM2G137321	1	289,403,622	289,405,770	5	3	4	1	XLOC_005965	1	289,402,962	289,405,468	6	4	4	2
GRMZM2G009901	1	290,273,078	290,275,448	42	15	19	14	XLOC_005986	1	290,273,163	290,275,009	42	15	19	14
GRMZM2G082664	1	290,337,450	290,359,023	28	13	15	9	GRMZM2G432560	1	290,344,248	290,346,241	-	-	-	-
GRMZM2G004511	1	290,878,028	290,879,761	12	7	5	6	XLOC_002940	1	290,877,819	290,880,168	12	7	5	6
GRMZM2G347043	1	292,162,383	292,164,080	5	1	1	1	XLOC_006017	1	292,162,313	292,164,056	7	2	2	2
GRMZM2G151997	1	292,412,495	292,413,903	30	18	13	17	XLOC_002964	1	292,412,705	292,413,447	17	7	1	7
GRMZM2G139250	1	292,594,128	292,598,884	29	14	14	15	XLOC_002967	1	292,594,168	292,595,450	21	11	11	12
GRMZM2G155580	1	293,065,790	293,074,538	32	14	14	14	XLOC_006037	1	293,066,717	293,066,966	4	4	3	4
GRMZM2G068244	1	293,721,769	293,724,068	14	7	4	8	GRMZM2G068443	1	293,722,839	293,726,484	14	5	9	8
GRMZM2G139041	1	295,097,680	295,100,439	11	4	2	5	XLOC_006075	1	295,097,968	295,098,274	-	-	-	-
GRMZM2G139123	1	295,100,532	295,101,411	23	4	3	4	XLOC_006076	1	295,100,872	295,101,411	11	2	1	1
XLOC_006078	1	295,340,295	295,343,097	11	1	2	3	XLOC_003015	1	295,340,807	295,342,977	11	1	2	3
GRMZM2G366681	1	296,067,223	296,069,043	3	0	0	0	GRMZM2G448523	1	296,062,422	296,069,083	17	7	9	7
GRMZM2G053916	1	296,076,874	296,085,483	49	26	20	25	GRMZM2G053803	1	296,082,206	296,088,792	5	3	3	4
GRMZM2G053803	1	296,082,206	296,088,792	5	3	3	4	GRMZM2G351160	1	296,088,128	296,096,856	25	6	4	9
GRMZM2G113158	1	296,739,858	296,743,544	68	17	17	14	XLOC_003038	1	296,740,624	296,742,835	45	15	14	12
GRMZM2G338809	1	296,788,163	296,790,581	25	10	11	11	GRMZM5G883969	1	296,789,724	296,790,547	23	9	11	11
GRMZM2G170137	1	296,849,361	296,855,071	14	5	5	5	GRMZM2G470422	1	296,847,943	296,850,494	21	11	12	12
GRMZM2G352415	1	297,095,291	297,098,628	20	7	6	7	XLOC_003050	1	297,096,040	297,098,364	10	2	1	1
GRMZM2G016084	1	297,827,097	297,832,384	25	10	9	8	XLOC_003061	1	297,830,785	297,832,332	21	8	6	5
GRMZM2G123901	1	298,210,469	298,213,577	10	3	4	4	XLOC_003067	1	298,212,453	298,212,781	-	-	-	-
GRMZM5G876146	1	298,813,813	298,817,252	5	3	3	1	XLOC_006147	1	298,814,031	298,814,417	-	-	-	-
GRMZM2G011923	1	298,817,393	298,820,925	49	2	9	5	XLOC_003086	1	298,819,416	298,820,340	36	1	8	2
GRMZM2G100020	1	299,060,665	299,064,887	59	19	16	16	GRMZM2G099987	1	299,064,805	299,069,896	38	4	2	3
GRMZM2G115456	1	299,272,391	299,278,057	17	5	4	5	GRMZM2G417843	1	299,276,941	299,281,868	30	12	9	10
GRMZM2G363229	1	299,980,376	299,982,620	14	5	6	4	XLOC_003103	1	299,980,674	299,981,223	1	0	0	0
GRMZM2G363229	1	299,980,376	299,982,620	14	5	6	4	XLOC_003104	1	299,981,775	299,982,624	11	4	5	4
GRMZM2G115162	1	300,821,132	300,824,570	7	2	1	2	GRMZM2G415077	1	300,823,694	300,824,035	7	2	1	2
GRMZM2G131939	1	300,951,291	300,963,853	28	6	9	6	GRMZM2G132084	1	300,938,076	300,951,517	11	0	0	0
XLOC_011451	2	177,675	179,791	8	5	1	5	XLOC_008977	2	177,859	179,378	3	0	1	0
GRMZM2G064212	2	1,038,129	1,042,294	15	2	2	3	XLOC_011464	2	1,038,189	1,038,486	1	1	1	1
GRMZM2G018595	2	1,437,737	1,441,025	67	25	27	27	GRMZM2G019500	2	1,439,756	1,444,066	23	4	3	3
GRMZM2G149958	2	1,678,757	1,682,890	22	4	6	5	XLOC_011485	2	1,679,179	1,679,524	2	0	0	0
GRMZM2G078033	2	2,366,176	2,369,867	11	6	4	6	XLOC_011500	2	2,366,238	2,366,609	1	0	0	0
GRMZM2G010257	2	2,549,740	2,552,578	13	3	2	4	GRMZM2G009994	2	2,544,036	2,550,544	26	8	9	9
GRMZM2G102349	2	2,787,752	2,791,301	8	2	1	1	XLOC_011507	2	2,789,648	2,791,180	8	2	1	1
GRMZM2G043932	2	2,821,204	2,822,336	10	3	3	2	XLOC_009047	2	2,821,513	2,821,874	8	2	2	1
GRMZM2G025536	2	3,019,428	3,021,698	22	7	8	9	XLOC_011511	2	3,018,548	3,020,854	16	6	7	6
GRMZM2G035325	2	3,115,670	3,119,831	21	5	5	4	XLOC_009065	2	3,118,994	3,119,809	14	4	5	3
GRMZM2G119941	2	3,191,332	3,193,885	8	2	3	2	XLOC_011518	2	3,192,839	3,193,216	6	2	3	2
GRMZM2G018716	2	3,229,402	3,232,016	6	1	0	1	XLOC_011519	2	3,230,346	3,230,889	2	0	0	0
GRMZM2G044011	2	3,928,213	3,938,819	18	7	5	6	XLOC_009091	2	3,938,479	3,938,709	-	-	-	-
GRMZM2G056513	2	3,978,709	3,979,948	57	26	23	22	XLOC_009093	2	3,978,982	3,979,406	29	12	11	11
GRMZM2G040095	2	4,192,323	4,196,291	77	25	24	20	XLOC_011539	2	4,194,864	4,195,911	7	2	2	3
GRMZM2G033219	2	4,839,823	4,846,096	46	18	18	9	GRMZM2G033430	2	4,839,281	4,841,272	38	19	17	8
GRMZM2G032977	2	4,852,765	4,855,560	24	17	17	18	XLOC_009122	2	4,853,539	4,854,344	5	1	1	1
GRMZM2G160585	2	5,235,067	5,239,077	28	15	12	6	XLOC_011565	2	5,236,510	5,236,895	5	2	1	1
GRMZM2G174917	2	5,568,332	5,570,261	24	7	6	7	XLOC_011572	2	5,569,296	5,569,921	16	6	5	6
GRMZM2G174926	2	5,572,045	5,578,697	16	3	3	1	XLOC_009145	2	5,572,048	5,572,757	5	1	2	0
GRMZM2G021635	2	6,055,581	6,059,653	19	6	3	5	XLOC_009154	2	6,058,782	6,059,605	4	2	2	2
GRMZM5G829840	2	6,642,341	6,646,369	24	6	4	5	XLOC_009165	2	6,642,462	6,643,178	4	1	1	1
GRMZM2G106245	2	6,988,897	6,991,891	15	7	3	6	XLOC_009175	2	6,991,269	6,991,847	2	1	0	1
XLOC_011624	2	7,047,927	7,051,492	18	9	7	10	XLOC_009182	2	7,049,348	7,050,124	-	-	-	-
GRMZM2G164400	2	7,519,507	7,527,867	12	3	3	3	GRMZM2G164277	2	7,527,470	7,531,925	28	10	11	8
GRMZM2G084181	2	9,117,236	9,130,542	17	8	9	7	XLOC_011646	2	9,126,849	9,127,558	-	-	-	-
GRMZM2G094328	2	9,551,436	9,552,864	10	2	4	3	XLOC_011660	2	9,551,732	9,552,554	10	2	4	3
GRMZM2G005310	2	10,343,672	10,353,395	34	7	11	10	XLOC_011677	2	10,348,422	10,348,804	5	3	2	1
GRMZM2G005040	2	10,387,109	10,389,573	16	4	8	7	XLOC_011679	2	10,387,374	10,389,429	16	4	8	7
GRMZM2G071832	2	11,769,293	11,771,169	21	6	8	7	XLOC_011714	2	11,769,111	11,771,067	21	6	8	7
GRMZM2G131280	2	13,552,922	13,555,183	20	7	8	5	XLOC_011739	2	13,554,335	13,554,617	7	6	6	3
GRMZM2G131280	2	13,552,922	13,555,183	20	7	8	5	XLOC_011740	2	13,554,719	13,555,152	-	-	-	-
GRMZM2G300111	2	13,715,062	13,717,432	13	1	1	2	XLOC_011741	2	13,715,840	13,716,398	2	0	0	1
GRMZM2G314396	2	14,548,496	14,554,289	55	24	30	20	GRMZM2G017197	2	14,545,767	14,549,044	18	6	8	7
GRMZM2G314396	2	14,548,496	14,554,289	55	24	30	20	XLOC_009351	2	14,552,836	14,553,772	2	0	1	1
GRMZM2G080992	2	16,654,034	16,656,556	4	1	2	1	XLOC_009380	2	16,654,335	16,656,671	4	1	2	1
GRMZM2G379540	2	17,085,454	17,092,794	12	4	5	4	GRMZM2G076841	2	17,085,870	17,090,770	15	2	4	4
GRMZM2G174807	2	18,757,659	18,759,526	20	11	6	8	GRMZM2G175504	2	18,735,506	18,758,909	61	30	25	19
GRMZM2G351330	2	22,221,051	22,222,694	4	2	2	2	XLOC_011863	2	22,220,989	22,221,632	4	2	2	2
GRMZM5G810061	2	23,123,860	23,128,108	6	3	1	0	XLOC_011876	2	23,125,153	23,127,375	-	-	-	-
GRMZM2G062554	2	23,193,426	23,199,044	15	7	4	3	GRMZM2G062471	2	23,198,729	23,201,285	30	12	11	8

GRMZM2G108219	2	25,437,808	25,439,460	14	3	3	6	XLOC_009503	2	25,437,797	25,439,378	14	3	3	6
XLOC_011916	2	25,458,560	25,461,491	10	1	5	3	XLOC_009505	2	25,458,589	25,459,321	6	1	2	1
GRMZM2G108123	2	25,497,178	25,498,828	10	5	1	2	XLOC_009506	2	25,497,200	25,498,897	10	5	1	2
XLOC_009507	2	25,628,639	25,629,596	5	1	2	2	GRMZM2G081957	2	25,628,559	25,631,894	5	1	2	2
GRMZM2G123122	2	26,759,318	26,763,396	26	7	10	10	XLOC_011932	2	26,759,213	26,762,530	23	6	8	8
GRMZM2G127591	2	26,915,506	26,918,624	4	3	4	0	GRMZM2G428386	2	26,917,555	26,922,771	22	14	14	10
GRMZM2G138125	2	27,115,035	27,125,249	17	3	1	0	GRMZM2G138190	2	27,124,232	27,125,695	21	1	0	1
GRMZM2G362413	2	27,500,744	27,502,059	2	1	1	0	XLOC_011944	2	27,501,203	27,501,645	2	1	1	0
GRMZM2G441347	2	28,745,695	28,748,913	40	21	20	7	XLOC_011959	2	28,746,597	28,748,681	35	17	17	4
GRMZM2G048295	2	30,170,017	30,172,758	15	5	7	6	XLOC_009576	2	30,170,685	30,171,385	3	0	1	1
GRMZM2G347361	2	30,305,903	30,309,532	16	4	5	5	GRMZM2G171620	2	30,308,490	30,311,407	22	6	6	8
GRMZM2G082191	2	31,214,984	31,222,672	10	4	3	4	XLOC_011994	2	31,220,291	31,221,563	5	2	2	2
GRMZM2G419342	2	32,571,806	32,573,931	49	12	12	10	XLOC_009602	2	32,571,968	32,573,911	56	13	14	10
GRMZM2G112530	2	33,976,171	33,979,292	20	7	8	5	XLOC_009621	2	33,977,709	33,979,429	13	3	5	3
GRMZM2G062673	2	34,359,171	34,360,419	27	9	10	8	XLOC_009629	2	34,359,265	34,360,412	29	9	10	8
GRMZM2G080176	2	34,636,307	34,657,655	12	3	4	2	XLOC_009631	2	34,655,098	34,655,300	-	-	-	-
GRMZM2G102183	2	36,945,636	36,948,162	21	10	10	9	XLOC_012069	2	36,946,248	36,947,937	16	8	9	7
GRMZM2G063262	2	37,392,043	37,396,674	16	6	3	5	GRMZM2G063163	2	37,396,078	37,402,459	34	16	18	18
GRMZM2G147884	2	38,760,903	38,768,270	67	11	11	10	GRMZM2G447691	2	38,767,291	38,770,475	55	7	9	7
GRMZM2G121700	2	38,821,032	38,822,692	21	7	3	4	XLOC_012098	2	38,820,651	38,822,523	27	9	5	5
XLOC_009682	2	40,051,916	40,054,726	-	-	-	-	GRMZM2G330430	2	40,054,195	40,056,907	36	11	13	12
GRMZM2G102499	2	41,931,997	41,935,666	17	12	4	13	XLOC_009707	2	41,934,436	41,934,852	-	-	-	-
GRMZM2G012328	2	42,992,969	42,994,079	9	3	3	2	GRMZM2G315401	2	42,993,870	42,996,389	45	19	25	15
GRMZM2G065073	2	43,413,441	43,421,006	44	6	12	9	XLOC_012141	2	43,419,169	43,419,719	-	-	-	-
GRMZM2G171254	2	44,816,328	44,824,949	10	5	4	5	XLOC_009745	2	44,823,979	44,824,372	-	-	-	-
GRMZM2G171277	2	44,829,337	44,831,648	19	5	10	2	XLOC_012161	2	44,830,075	44,831,590	17	4	9	2
GRMZM2G127139	2	45,069,108	45,078,053	24	11	12	5	XLOC_009751	2	45,073,255	45,073,509	3	2	2	1
GRMZM2G028258	2	46,616,631	46,620,668	92	38	36	34	GRMZM2G028139	2	46,620,242	46,632,084	3	0	0	0
GRMZM2G053111	2	48,775,553	48,778,515	30	13	14	11	XLOC_012207	2	48,776,032	48,776,812	11	4	5	4
GRMZM2G047681	2	49,031,132	49,036,412	5	2	3	3	XLOC_009786	2	49,036,076	49,036,390	1	0	0	1
GRMZM2G131155	2	51,867,500	51,869,605	4	1	1	1	XLOC_009826	2	51,868,677	51,870,074	2	2	0	0
GRMZM2G069093	2	54,244,859	54,246,513	21	4	7	1	XLOC_009840	2	54,245,127	54,245,771	7	1	4	1
GRMZM2G044132	2	54,473,290	54,474,810	14	3	3	3	XLOC_012256	2	54,473,371	54,474,810	14	3	3	3
GRMZM2G048793	2	56,456,421	56,457,513	5	2	3	2	XLOC_009857	2	56,456,998	56,457,735	4	2	3	2
GRMZM2G134668	2	62,121,451	62,125,108	32	13	13	12	XLOC_009891	2	62,122,873	62,123,618	2	0	0	0
GRMZM2G032225	2	62,920,594	62,922,074	55	16	22	14	XLOC_012320	2	62,921,103	62,921,705	42	13	17	12
GRMZM2G087059	2	64,001,685	64,004,274	46	16	16	14	XLOC_012328	2	64,002,611	64,003,234	8	1	3	2
GRMZM2G436533	2	64,294,902	64,298,527	10	3	6	3	GRMZM2G136412	2	64,298,381	64,301,370	26	3	6	2
GRMZM2G086267	2	68,168,970	68,174,713	12	4	3	7	XLOC_012351	2	68,169,226	68,169,426	-	-	-	-
GRMZM2G155281	2	69,807,585	69,810,746	29	1	3	1	XLOC_009935	2	69,807,982	69,808,578	-	-	-	-
XLOC_009939	2	71,189,910	71,190,894	10	2	1	3	XLOC_012369	2	71,189,907	71,190,586	10	2	1	3
GRMZM2G027307	2	86,977,308	86,991,387	10	2	1	1	XLOC_012446	2	86,977,473	86,977,751	4	1	1	0
GRMZM5G803404	2	101,895,519	101,901,259	19	1	5	2	XLOC_010062	2	101,899,220	101,901,186	28	7	16	4
GRMZM5G803404	2	101,895,519	101,901,259	19	1	5	2	XLOC_010061	2	101,897,618	101,897,932	-	-	-	-
GRMZM2G172695	2	104,803,852	104,806,393	25	9	11	5	XLOC_012500	2	104,805,122	104,806,195	18	7	9	3
GRMZM2G076343	2	113,942,575	113,944,157	5	2	2	2	XLOC_012567	2	113,942,348	113,943,804	5	2	2	2
GRMZM2G164074	2	124,226,002	124,228,806	13	2	2	2	XLOC_010163	2	124,226,792	124,229,015	13	2	2	2
GRMZM2G085924	2	127,723,784	127,725,392	7	2	4	4	XLOC_010173	2	127,724,600	127,725,352	7	2	4	4
GRMZM2G301355	2	128,454,614	128,458,045	66	28	25	21	XLOC_012641	2	128,454,357	128,457,728	80	32	31	29
GRMZM2G111143	2	134,470,091	134,472,840	32	12	15	12	XLOC_012675	2	134,469,981	134,471,436	15	8	8	5
GRMZM2G133716	2	136,740,072	136,745,887	27	7	11	11	XLOC_012684	2	136,743,919	136,745,759	10	4	5	5
GRMZM2G146472	2	138,609,364	138,620,146	19	2	2	2	XLOC_010222	2	138,610,202	138,611,163	4	0	0	0
GRMZM2G099502	2	140,149,834	140,152,470	30	10	11	12	XLOC_012696	2	140,149,765	140,151,440	13	3	3	3
GRMZM2G032478	2	142,057,153	142,058,876	21	5	7	13	XLOC_012711	2	142,057,146	142,058,616	21	5	7	13
GRMZM2G348866	2	143,679,351	143,684,440	19	7	7	7	GRMZM2G348855	2	143,678,133	143,679,858	7	2	2	3
GRMZM2G028179	2	143,893,289	143,895,477	16	4	5	4	XLOC_012718	2	143,894,058	143,895,355	14	2	4	3
GRMZM2G450044	2	144,155,245	144,156,375	31	18	14	10	XLOC_012719	2	144,155,308	144,156,067	26	17	13	8
GRMZM2G127418	2	144,827,260	144,828,751	13	3	4	5	XLOC_012726	2	144,827,195	144,828,011	6	2	2	2
GRMZM2G132000	2	145,653,249	145,656,755	6	4	3	3	XLOC_010271	2	145,656,350	145,656,679	-	-	-	-
GRMZM2G460138	2	151,389,889	151,390,712	3	1	1	1	XLOC_010306	2	151,390,008	151,390,734	7	1	4	4
GRMZM2G365035	2	157,924,539	157,932,534	2	2	2	1	XLOC_010349	2	157,931,493	157,932,381	2	2	2	1
GRMZM5G876616	2	159,084,980	159,087,515	15	4	5	4	XLOC_012822	2	159,085,004	159,085,643	7	1	1	2
GRMZM2G068212	2	162,036,804	162,097,539	23	4	2	3	XLOC_010373	2	162,045,456	162,047,512	-	-	-	-
AC210204.3_FG002	2	165,300,551	165,302,574	13	6	5	4	XLOC_010390	2	165,300,781	165,301,621	6	3	3	3
GRMZM2G092125	2	169,186,883	169,190,374	16	4	10	8	XLOC_012901	2	169,186,924	169,187,674	16	4	10	8
GRMZM2G092125	2	169,186,883	169,190,374	16	4	10	8	XLOC_012902	2	169,189,856	169,190,340	-	-	-	-
GRMZM2G092648	2	171,021,988	171,027,132	27	8	11	5	XLOC_010448	2	171,026,303	171,027,370	17	6	7	3
XLOC_010473	2	173,494,956	173,496,273	-	-	-	-	GRMZM2G104546	2	173,490,360	173,497,277	8	2	1	1
AC203957.3_FG004	2	175,112,138	175,113,156	32	15	16	15	XLOC_010487	2	175,112,354	175,112,845	26	10	11	10
GRMZM2G037150	2	176,669,016	176,673,281	-	-	-	-	GRMZM2G037131	2	176,668,559	176,669,692	5	2	3	2
GRMZM5G868354	2	177,200,642	177,204,531	11	6	3	3	XLOC_012973	2	177,200,717	177,201,068	-	-	-	-
GRMZM2G373554	2	177,562,284	177,563,575	6	2	4	2	XLOC_012976	2	177,562,929	177,563,476	-	-	-	-
GRMZM2G340933	2	178,377,065	178,378,920	3	1	1	0	XLOC_012981	2	178,376,981	178,378,697	3	1	1	0
XLOC_010530	2	178,670,829	178,671,877	31	23	17	25	XLOC_012986	2	178,670,884	178,671,554	31	23	17	25
GRMZM2G125775	2	179,011,169	179,012,353	14	6	6	5	XLOC_010532	2	179,011,357	179,012,135	12	6	6	5
GRMZM2G040840	2	180,452,226	180,457,230	32	16	5	15	XLOC_013013	2	180,452,055	180,453,525	55	28	19	30
GRMZM2G359521	2	181,475,801	181,477,592	9	4	4	0	XLOC_013019	2	181,476,797	181,477,422	9	4	4	0
GRMZM2G057852	2	183,846,887	183,847,834	15	4	1	3	XLOC_013031	2	183,846,951	183,847,432	-	-	-	-
XLOC_010571	2	183,946,317	183,947,096	23	13	12	11	XLOC_013033	2	183,946,385	183,946,791	-	-	-	-
GRMZM2G002131	2	186,829,385	186,833,823	35	15	15	15	XLOC_013076	2	186,829,842	186,830,387	8	7	7	7
GRMZM2G381071	2	187,110,003	187,110,972	12	5	8	9	XLOC_013079	2	187,110,216	187,110,887	7	5	5	6
GRMZM2G018070	2	189,550,226	189,551,687	16	9	10	10	XLOC_010643	2	189,550,546	189,551,309	10	6	7	7
GRMZM2G004138	2	189,923,260	189,928,367	22	8	6	7	XLOC_010651	2	189,927,099	189,928,613	19	6	5	7
GRMZM2G134334	2	190,220,678	190,223,077	7	4	4	6	XLOC_013120	2	190,221,109	190,222,380	7	4	4	6
GRMZM2G003947	2	190,587,060	190,589,608	9	2	4	4	XLOC_010664	2	190,588,589	190,589,744	4	2	2	2
GRMZM2G014341	2	192,312,471	192,316,197	25	16	16	16	GRMZM2G013892	2	192,310,632	192,313,184	25	12	15	11
GRMZM2G150503	2	194,151,567	194,157,270	55	21	28	21	XLOC_013165	2	194,155,565	194,156,945	23	7	12	9
GRMZM2G170253	2	194,491,687	194,493,980	24	6	11	10	XLOC_010713	2	194,492,414	194,494,026	32	11	16	12
GRMZM2G164956	2	194,808,387	194,812,823	68	13	18	14	XLOC_013172	2	194,812,435	194,812,814	7	1	1	0
GRMZM2G063533	2	196,480,040	196,484,796	12	4	4	2	XLOC_010731	2	196,480,477	196,480,984	3	1	1	0
GRMZM2G058105	2	196,700,585	196,747,523	68	31	28	29	GRMZM2G050714	2	196,730,795	196,734,631	7	1	2	2
GRMZM2G312501	2	198,178,361	198,183,070	30	7	5	3	XLOC_010762	2	198,182,722	198,183,289	3	2	1	2
AC233910.1_FG008	2	198,426,229	198,427,884	5	1	2	1	GRMZM5G887647	2	198,422,869	198,427,142	5	2	2	2
GRMZM2G081571	2	199,224,685	199,231,534	33	15	5	10	GRMZM2G081790	2	199,230,719	199,233,993	4	0	0	1
GRMZM2G004619	2	199,378,450	199,380,793	13	6	5	5	XLOC_010788	2	199,380,359	199,380,622	2	1	1	1
GRMZM2G169694	2	202,063,279	202,073,506	6	1	0	1	XLOC_013269	2	202,063,445	202,063,721	2	1	0	1
GRMZM2G060271	2	206,709,530	206,711,963	13	7	8	6	XLOC_010912	2	206,709,714	206,712,301	13	7	8	6
GRMZM2G062156	2	208,298,516	208,300,768	30	11	9	10	XLOC_013368	2	208,298,218	208,300,410	30	11	9	10
GRMZM2G431309	2	208,500,848	208,505,599	82	26	35	27	GRMZM2G130854	2	208,504,875	208,511,227	11	1	1	0
GRMZM2G376432	2	209,675,368	209,678,600	19	6	12	4	GRMZM2G077845	2	209,674,829	209,675,515	16	5	9	4
GRMZM2G079082	2	209,789,137	209,793,396	17	3	4	5	XLOC_010967	2	209,791,906	209,792,788	7	1	1	3
GRMZM2G102138	2	210,207,281	210,208,102	67	25	24	33	XLOC_010974	2	210,207,604	210,208,075	49	15	17	23
GRMZM2G412888	2	211,499,169	211,505,370	13	2	3	3	XLOC_013412	2	211,499,333	211,499,610	1	0	0	1
GRMZM2G101769	2	211,875,412	211,877,983	12	5	4	3	XLOC_013421	2	211,875,392	211,876,119	10	5	4	3
GRMZM2G075002	2	213,830,523	213,835,155	44	10	8	12	XLOC_011039	2	213,832,870	213,834,143	39	10	8	12
GRMZM2G123212	2	214,245,816	214,246,895	26	4	10	8	XLOC_011054	2	214,245,985	214,246,562	21	2	9	8
XLOC_013465	2	214,483,221	214,484,563	10	3	3	2	XLOC_011057	2	214,483,242	214,483,828	-	-	-	-
GRMZM2G060160	2	214,616,867	214,622,270	27	7	9	7	XLOC_011061	2	214,620,687	214,620,972	1	0	0	0
GRMZM2G327595	2	215,329,117	215,334,037	35	9	17	15	XLOC_013479	2	215,329,676	215,333,000	30	7	16	13
GRMZM2G327595	2	215,329,117	215,334,037	35	9	17	15	GRMZM2G327686	2	215,333,156	215,334,980	12	3	6	4
GRMZM2G459363	2	215,589,169	215,591,897	19	2	4	4	XLOC_013483	2	215,589,066	215,591,467	19	2	4	4
GRMZM2G166971	2	215,615,958	215,617,442	11	5	6	6	XLOC_013484	2	215,616,519	215,616,786	3	2	2	2
XLOC_013491	2	215,925,940	215,927,828	1	1	1	0	XLOC_011092	2	215,926,301	215,927,938	1	1	1	0

XLOC_011093	2	215,950,474	215,954,466	43	11	21	9	XLOC_013492	2	215,949,546	215,952,234	27	5	14	6
XLOC_011093	2	215,950,474	215,954,466	43	11	21	9	XLOC_013493	2	215,953,968	215,955,328	4	1	1	0
GRMZM2G001247	2	217,334,825	217,335,843	24	8	9	8	XLOC_013519	2	217,335,074	217,335,603	8	2	2	2
XLOC_013523	2	217,520,133	217,522,001	5	3	1	3	GRMZM2G308752	2	217,520,351	217,522,200	9	5	2	4
GRMZM2G128109	2	218,889,998	218,892,152	10	0	1	2	XLOC_013540	2	218,891,286	218,891,937	10	0	1	2
GRMZM2G068220	2	219,606,880	219,608,943	11	1	2	2	XLOC_013550	2	219,606,971	219,607,483	-	-	-	-
XLOC_013559	2	220,397,639	220,399,809	9	4	4	1	XLOC_011164	2	220,398,191	220,398,448	-	-	-	-
GRMZM2G137546	2	220,671,360	220,672,972	19	7	8	8	XLOC_013562	2	220,672,012	220,672,735	3	1	1	0
GRMZM2G129268	2	221,286,810	221,291,728	12	2	2	1	XLOC_013567	2	221,286,814	221,287,471	-	-	-	-
GRMZM5G843434	2	221,314,181	221,316,190	-	-	-	-	XLOC_013568	2	221,314,265	221,316,032	17	8	7	6
GRMZM2G171818	2	221,338,800	221,341,247	28	10	8	8	XLOC_011184	2	221,338,943	221,341,232	28	10	8	8
GRMZM2G106560	2	222,422,457	222,427,553	37	18	20	15	XLOC_013579	2	222,423,023	222,423,280	16	7	8	4
GRMZM2G169095	2	225,001,746	225,013,108	69	17	8	14	XLOC_013606	2	225,012,370	225,012,954	1	1	0	0
GRMZM2G158872	2	231,189,722	231,195,711	31	14	9	12	XLOC_013682	2	231,191,529	231,191,923	-	-	-	-
GRMZM2G053206	2	232,655,940	232,657,533	17	7	6	2	XLOC_011352	2	232,655,972	232,657,479	17	7	6	2
GRMZM2G395128	2	233,507,197	233,508,793	73	32	31	22	XLOC_011372	2	233,507,725	233,509,411	46	18	20	19
GRMZM2G135013	2	233,546,257	233,548,974	43	19	20	17	XLOC_011373	2	233,546,506	233,548,765	38	17	18	15
GRMZM2G082886	2	233,641,444	233,644,826	3	1	1	1	GRMZM2G082853	2	233,643,319	233,646,053	37	15	12	14
GRMZM5G878490	2	234,414,167	234,415,092	6	1	2	3	XLOC_013747	2	234,413,745	234,415,092	8	1	2	3
GRMZM2G324544	2	235,377,544	235,378,343	1	0	0	0	GRMZM2G324507	2	235,377,387	235,389,082	45	14	15	14
AC206764.4_FG003	3	654,707	655,811	1	0	1	0	XLOC_013797	3	655,360	657,996	3	0	1	1
GRMZM2G091047	3	1,349,029	1,351,202	24	8	10	9	GRMZM5G816891	3	1,349,760	1,354,995	18	12	5	10
GRMZM2G013201	3	1,612,503	1,613,952	30	6	8	6	GRMZM2G148723	3	1,609,911	1,613,862	30	6	8	6
GRMZM2G309152	3	1,615,839	1,631,263	50	17	18	19	GRMZM2G309063	3	1,630,778	1,633,534	51	18	20	19
GRMZM2G011636	3	1,672,575	1,677,158	15	4	5	2	GRMZM2G011553	3	1,676,514	1,678,951	13	2	4	4
GRMZM2G123540	3	1,721,076	1,725,886	41	9	6	8	GRMZM2G123519	3	1,725,071	1,729,621	14	5	2	3
GRMZM2G099678	3	2,001,563	2,003,991	31	2	7	12	XLOC_016051	3	2,003,027	2,003,644	19	2	3	8
GRMZM2G085266	3	3,200,730	3,210,711	11	6	7	3	XLOC_016075	3	3,209,985	3,210,733	1	0	0	0
GRMZM2G102572	3	3,457,016	3,462,868	8	0	1	0	GRMZM2G400907	3	3,452,011	3,457,360	27	5	8	4
AC233853.1_FG003	3	3,830,012	3,832,754	5	2	4	1	XLOC_016089	3	3,831,343	3,832,351	4	3	3	2
GRMZM2G035131	3	4,550,708	4,554,681	8	2	2	2	XLOC_013872	3	4,553,962	4,554,480	-	-	-	-
GRMZM2G055089	3	4,674,607	4,680,711	35	10	12	13	XLOC_016106	3	4,678,895	4,680,599	22	6	7	7
GRMZM2G055313	3	4,707,293	4,710,578	13	2	2	1	XLOC_016107	3	4,707,024	4,709,135	11	2	2	1
GRMZM2G055313	3	4,707,293	4,710,578	13	2	2	1	XLOC_016108	3	4,709,572	4,709,965	1	0	0	0
GRMZM2G355572	3	7,938,344	7,940,976	26	10	14	9	XLOC_013910	3	7,939,013	7,940,694	26	10	14	9
XLOC_013911	3	7,966,853	7,968,441	2	1	1	1	GRMZM2G367898	3	7,966,861	7,967,903	-	-	-	-
GRMZM2G176489	3	8,219,031	8,221,674	41	22	30	22	XLOC_013917	3	8,220,468	8,221,022	12	3	7	4
GRMZM2G158194	3	9,045,046	9,047,445	37	10	12	11	XLOC_016189	3	9,044,823	9,046,830	37	10	12	11
GRMZM2G355381	3	9,114,106	9,118,344	41	11	11	12	XLOC_016191	3	9,113,934	9,115,143	11	6	7	5
GRMZM2G083418	3	9,808,882	9,811,705	16	4	4	4	XLOC_013956	3	9,811,021	9,811,476	4	1	1	2
GRMZM2G305900	3	9,855,605	9,858,253	15	8	8	3	XLOC_016200	3	9,856,999	9,857,500	6	2	2	3
GRMZM2G021410	3	13,278,182	13,280,956	25	8	9	6	XLOC_016230	3	13,278,194	13,278,623	2	0	0	0
GRMZM2G452564	3	15,137,688	15,139,240	1	0	0	0	XLOC_016255	3	15,136,784	15,139,075	4	2	2	0
GRMZM2G034534	3	18,085,249	18,086,875	6	4	3	2	XLOC_016291	3	18,085,278	18,085,649	3	2	2	1
GRMZM2G320521	3	19,762,347	19,764,749	41	19	20	12	XLOC_016312	3	19,762,167	19,764,374	43	19	21	12
GRMZM2G467671	3	19,973,177	19,977,322	43	11	10	10	XLOC_014117	3	19,973,505	19,974,270	5	2	2	1
GRMZM2G156950	3	22,005,530	22,007,136	14	6	6	7	XLOC_014132	3	22,005,326	22,006,215	7	5	4	4
GRMZM2G090584	3	26,258,962	26,261,458	12	5	8	8	XLOC_016363	3	26,259,115	26,259,614	9	4	7	6
GRMZM2G104829	3	26,309,355	26,312,448	54	20	27	19	XLOC_014167	3	26,310,637	26,312,874	36	11	16	8
GRMZM2G132913	3	37,359,987	37,361,918	55	14	24	13	XLOC_014236	3	37,360,058	37,361,385	36	8	19	10
GRMZM2G368388	3	38,438,067	38,440,389	13	3	5	3	XLOC_014244	3	38,438,506	38,440,607	11	3	5	3
GRMZM2G132465	3	39,477,546	39,482,124	17	7	6	7	XLOC_016483	3	39,477,668	39,478,338	1	0	0	0
GRMZM2G089982	3	40,529,064	40,531,245	6	1	1	1	XLOC_014260	3	40,530,819	40,532,893	18	6	6	6
GRMZM2G144648	3	41,742,447	41,744,589	15	5	6	7	XLOC_014266	3	41,742,767	41,744,902	15	5	6	7
GRMZM2G003883	3	44,224,052	44,229,674	8	1	3	2	XLOC_014275	3	44,229,170	44,229,601	1	0	0	0
GRMZM2G017520	3	48,494,950	48,497,362	53	31	25	28	XLOC_014298	3	48,496,982	48,497,546	12	6	3	6
GRMZM2G115960	3	48,806,998	48,810,388	40	7	6	6	XLOC_014300	3	48,809,033	48,810,511	31	6	6	4
GRMZM2G363908	3	52,448,552	52,452,420	4	0	0	1	GRMZM2G363893	3	52,452,031	52,454,284	19	5	6	6
GRMZM2G381473	3	57,097,944	57,101,742	8	1	2	2	GRMZM2G080320	3	57,091,548	57,098,312	18	6	9	8
GRMZM2G381473	3	57,097,944	57,101,742	8	1	2	2	XLOC_014367	3	57,099,106	57,101,623	8	1	2	2
GRMZM2G107228	3	59,327,568	59,329,698	9	6	5	5	XLOC_016598	3	59,328,610	59,328,924	-	-	-	-
GRMZM2G174975	3	60,645,763	60,648,379	59	19	29	24	XLOC_016608	3	60,645,606	60,646,302	5	2	2	2
GRMZM2G095039	3	66,707,678	66,709,478	21	2	3	2	XLOC_014417	3	66,708,373	66,708,927	5	1	1	0
GRMZM2G044724	3	85,672,795	85,676,822	25	8	7	7	XLOC_016701	3	85,674,690	85,674,920	-	-	-	-
GRMZM2G391794	3	91,614,897	91,618,962	27	6	8	7	XLOC_016738	3	91,617,008	91,618,186	9	2	2	3
GRMZM2G391794	3	91,614,897	91,618,962	27	6	8	7	XLOC_016737	3	91,615,051	91,616,155	3	0	0	0
GRMZM2G133793	3	93,102,434	93,104,203	20	3	4	3	XLOC_014507	3	93,102,813	93,104,274	20	3	4	3
XLOC_016818	3	109,716,966	109,717,972	6	1	2	1	XLOC_014550	3	109,717,705	109,718,299	1	0	1	0
GRMZM2G022629	3	117,275,976	117,278,216	3	2	1	1	XLOC_014595	3	117,277,486	117,278,440	-	-	-	-
XLOC_014607	3	119,994,992	119,997,548	24	7	9	8	AC203865.3_FG001	3	119,994,715	119,998,479	36	10	11	10
GRMZM2G351387	3	126,175,759	126,191,667	30	8	9	8	XLOC_016921	3	126,190,434	126,191,618	8	1	2	2
GRMZM2G060680	3	127,119,556	127,121,213	25	3	12	5	XLOC_016934	3	127,120,122	127,120,988	17	3	8	5
XLOC_016942	3	127,874,065	127,877,164	33	10	12	9	XLOC_014644	3	127,876,049	127,877,357	-	-	-	-
GRMZM2G160902	3	128,957,613	128,959,445	32	9	15	12	XLOC_014656	3	128,958,274	128,959,485	16	6	9	6
GRMZM2G046061	3	129,109,128	129,111,686	14	6	6	5	GRMZM2G046088	3	129,110,506	129,113,876	-	-	-	-
GRMZM2G155998	3	133,809,662	133,812,883	74	27	26	29	XLOC_016987	3	133,811,695	133,813,619	17	6	7	6
GRMZM2G155974	3	133,812,942	133,826,472	10	3	3	3	XLOC_016987	3	133,811,695	133,813,619	17	6	7	6
GRMZM2G323936	3	134,042,812	134,045,336	11	9	6	6	XLOC_016989	3	134,044,263	134,045,881	-	-	-	-
XLOC_016990	3	134,044,263	134,051,169	13	5	6	6	GRMZM2G323936	3	134,042,812	134,045,336	11	9	6	6
AC209377.3_FG003	3	137,557,525	137,558,581	16	11	11	9	XLOC_014721	3	137,557,685	137,558,726	17	11	11	9
GRMZM2G138003	3	138,121,498	138,123,238	9	3	2	1	XLOC_017014	3	138,121,201	138,123,238	9	3	2	1
GRMZM2G152889	3	142,724,521	142,734,864	2	0	1	1	GRMZM2G152952	3	142,722,457	142,726,219	-	-	-	-
GRMZM2G122431	3	145,209,774	145,213,115	44	11	12	15	XLOC_017074	3	145,209,893	145,210,556	10	2	3	4
GRMZM2G309512	3	146,284,010	146,287,961	32	5	7	11	GRMZM2G309479	3	146,286,679	146,287,500	15	2	3	3
GRMZM2G103342	3	146,556,764	146,558,820	35	11	13	13	XLOC_014816	3	146,557,193	146,558,978	45	15	16	16
GRMZM2G102056	3	147,559,407	147,561,340	4	1	1	1	XLOC_017124	3	147,559,395	147,561,086	4	1	1	1
GRMZM2G428168	3	149,351,183	149,353,705	19	7	5	8	GRMZM2G127789	3	149,351,145	149,352,030	4	2	2	3
GRMZM2G369652	3	150,308,311	150,311,184	-	-	-	-	GRMZM2G068665	3	150,304,861	150,310,060	16	0	1	1
GRMZM2G050997	3	152,240,411	152,243,105	2	1	1	1	XLOC_014868	3	152,240,443	152,241,766	-	-	-	-
GRMZM2G145840	3	153,898,814	153,900,122	26	12	15	10	XLOC_017183	3	153,899,269	153,900,406	4	2	2	1
GRMZM2G428987	3	154,652,380	154,669,702	50	18	22	20	XLOC_017193	3	154,666,729	154,667,335	5	2	4	2
GRMZM2G425004	3	154,780,689	154,782,525	16	2	6	5	XLOC_017197	3	154,781,735	154,782,216	9	2	2	1
XLOC_017207	3	155,839,330	155,842,013	-	-	-	-	XLOC_014904	3	155,840,176	155,842,742	24	1	5	6
GRMZM2G448834	3	157,126,999	157,130,351	47	21	20	16	XLOC_017223	3	157,128,739	157,130,173	10	2	2	2
GRMZM2G129114	3	160,370,641	160,372,491	26	11	9	12	XLOC_014944	3	160,370,919	160,372,685	26	11	9	12
GRMZM2G146697	3	161,889,750	161,898,826	23	7	10	7	XLOC_017267	3	161,889,843	161,890,571	3	1	1	0
GRMZM2G139822	3	164,927,183	164,941,378	17	6	6	3	XLOC_017296	3	164,927,342	164,927,696	5	3	3	1
GRMZM2G177227	3	165,900,055	165,902,629	11	5	1	3	XLOC_015002	3	165,900,808	165,902,750	6	2	0	1
GRMZM2G110277	3	171,482,926	171,486,892	8	5	4	3	GRMZM2G110398	3	171,485,569	171,487,165	8	5	4	3
GRMZM2G138176	3	171,752,389	171,756,517	21	7	5	7	GRMZM2G138161	3	171,756,041	171,760,110	2	2	2	1
GRMZM2G135743	3	172,092,917	172,094,704	36	16	20	13	XLOC_015094	3	172,093,718	172,094,315	8	6	4	4
GRMZM2G078500	3	172,376,539	172,379,695	82	22	21	22	XLOC_017375	3	172,376,634	172,377,105	26	6	7	5
GRMZM2G431006	3	172,487,141	172,489,677	9	6	6	1	XLOC_015099	3	172,489,396	172,490,339	8	6	6	1
GRMZM2G049373	3	173,048,156	173,050,637	18	5	8	4	XLOC_015103	3	173,048,324	173,049,084	11	4	5	3
GRMZM5G858197	3	175,673,165	175,677,076	10	4	5	5	GRMZM5G893764	3	175,675,750	175,680,444	18	4	6	6
AC155610.2_FG007	3	175,729,103	175,732,391	25	10	7	7	XLOC_017411	3	175,731,179	175,732,139	28	13	8	9
GRMZM2G061321	3	176,088,170	176,090,219	19	9	6	7	XLOC_017421	3	176,088,318	176,089,631	19	9	6	7
GRMZM2G074248	3	176,346,351	176,349,021	10	5	3	3	XLOC_017423	3	176,347,957	176,348,895	-	-	-	-
GRMZM2G074248	3	176,346,351	176,349,021	10	5	3	3	XLOC_017422	3	176,346,802	176,347,318	5	2	2	2
GRMZM2G127117	3	176,607,781	176,610,780	17	7	5	5	XLOC_015163	3	176,607,974	176,609,165	4	2	2	1
GRMZM2G003682	3	178,149,296	178,152,013	33	16	15	13	XLOC_017449	3	178,150,170	178,151,372	24	10	9	10
GRMZM2G177518	3	178,185,823	178,188,427	48	23	16	20	XLOC_015190	3	178,186,779	178,188,651	37	17	10	15
XLOC_015198	3	178,920,343	178,923,945	23	10	8	9	XLOC_017459	3	178,923,328	178,923,663	9	4	4	5

GRMZM2G065557	3	179,467,655	179,468,810	45	9	9	12	XLOC_017468	3	179,468,333	179,472,231	26	2	3	3
GRMZM2G054807	3	180,252,370	180,255,087	42	21	22	21	XLOC_017485	3	180,252,317	180,254,703	48	23	23	22
GRMZM2G126077	3	181,805,519	181,807,107	30	10	12	10	XLOC_015243	3	181,805,644	181,805,986	13	8	6	4
AC214448.3_FG007	3	181,844,506	181,847,013	6	3	3	3	XLOC_017510	3	181,844,862	181,845,535	-	-	-	-
GRMZM2G426953	3	182,096,591	182,101,272	24	8	10	9	GRMZM2G127034	3	182,091,206	182,097,283	-	-	-	-
GRMZM2G132169	3	183,701,067	183,703,595	30	10	8	11	XLOC_015269	3	183,702,635	183,702,843	-	-	-	-
GRMZM2G350410	3	185,806,850	185,811,071	21	7	4	8	GRMZM2G350399	3	185,808,548	185,810,097	10	3	4	5
GRMZM2G010447	3	186,273,513	186,275,044	28	11	14	11	XLOC_017571	3	186,273,476	186,274,094	27	11	14	11
GRMZM2G138074	3	187,769,141	187,771,874	54	30	34	20	XLOC_017607	3	187,770,273	187,771,591	22	10	13	9
GRMZM2G702059	3	188,039,626	188,043,090	10	2	2	1	XLOC_017614	3	188,042,731	188,043,031	2	1	1	1
GRMZM2G047274	3	188,387,390	188,390,373	24	5	7	9	XLOC_015342	3	188,387,394	188,388,307	-	-	-	-
GRMZM2G310368	3	188,728,061	188,729,514	4	0	0	1	XLOC_015345	3	188,728,781	188,729,273	4	0	0	1
AC233851.1_FG017	3	189,593,197	189,595,375	9	6	6	7	XLOC_017636	3	189,594,096	189,594,783	3	2	2	3
GRMZM2G173647	3	189,832,965	189,837,084	24	8	10	6	XLOC_017639	3	189,832,965	189,833,531	6	2	3	3
GRMZM2G322723	3	190,499,330	190,507,302	57	24	29	33	XLOC_017645	3	190,499,018	190,501,286	41	19	21	26
GRMZM2G322728	3	190,513,119	190,518,692	69	24	29	27	XLOC_015373	3	190,516,912	190,519,079	31	12	15	12
GRMZM2G054905	3	191,183,824	191,244,226	48	23	17	20	XLOC_015379	3	191,183,128	191,184,336	8	3	2	3
GRMZM2G446220	3	195,671,907	195,673,492	33	11	11	8	XLOC_015438	3	195,671,890	195,674,277	23	10	10	8
XLOC_017755	3	197,959,705	197,974,998	26	4	2	5	XLOC_015471	3	197,962,850	197,963,438	-	-	-	-
GRMZM2G141299	3	198,284,004	198,286,691	29	9	10	6	XLOC_015479	3	198,284,180	198,286,643	30	10	11	7
GRMZM2G055898	3	198,454,743	198,459,545	54	22	24	18	XLOC_015481	3	198,458,900	198,460,981	31	15	14	14
GRMZM2G358830	3	198,462,555	198,466,556	61	22	21	22	XLOC_015482	3	198,462,757	198,463,330	3	2	2	2
GRMZM2G176699	3	198,668,535	198,673,264	4	2	1	1	GRMZM2G474326	3	198,667,643	198,668,930	9	2	2	3
GRMZM2G177929	3	199,322,717	199,326,605	58	21	22	20	XLOC_015499	3	199,323,630	199,324,147	1	0	0	0
GRMZM2G057067	3	199,377,243	199,380,242	58	22	23	20	XLOC_015503	3	199,378,034	199,380,119	56	22	22	19
GRMZM5G836674	3	199,723,335	199,732,187	29	7	9	8	GRMZM2G051151	3	199,722,345	199,723,907	-	-	-	-
GRMZM2G381025	3	200,263,486	200,265,308	15	7	9	6	XLOC_015518	3	200,263,737	200,265,175	15	7	9	6
XLOC_017795	3	200,290,750	200,292,783	20	7	13	10	XLOC_015519	3	200,290,750	200,293,283	20	7	13	10
XLOC_017798	3	200,668,046	200,668,710	16	9	10	7	XLOC_015521	3	200,668,046	200,668,721	16	9	10	7
GRMZM2G040298	3	201,085,122	201,088,035	9	7	7	5	XLOC_017804	3	201,085,643	201,087,293	9	7	7	5
GRMZM2G025860	3	201,694,605	201,701,003	34	8	8	7	XLOC_017816	3	201,696,573	201,700,349	32	7	8	7
GRMZM2G159724	3	201,756,915	201,761,523	14	4	4	3	XLOC_015538	3	201,757,013	201,758,683	8	3	2	2
GRMZM2G055276	3	202,504,869	202,507,948	29	13	14	14	XLOC_017833	3	202,505,176	202,506,715	-	-	-	-
GRMZM2G111611	3	204,542,143	204,548,156	27	10	11	10	XLOC_015587	3	204,542,398	204,542,665	6	1	3	2
GRMZM2G165099	3	205,448,996	205,451,333	17	6	4	6	XLOC_015603	3	205,449,433	205,449,797	-	-	-	-
GRMZM2G157317	3	205,695,411	205,702,868	24	15	11	14	XLOC_017881	3	205,701,185	205,702,703	6	2	2	2
GRMZM2G051683	3	205,769,467	205,770,820	11	7	6	6	XLOC_015609	3	205,769,378	205,770,850	11	7	6	6
AC233882.1_FG003	3	205,915,252	205,922,597	19	5	5	10	XLOC_015611	3	205,915,987	205,917,102	14	4	4	9
GRMZM2G146913	3	206,848,092	206,849,182	8	2	3	3	XLOC_017899	3	206,848,752	206,849,130	-	-	-	-
GRMZM2G028568	3	207,028,261	207,037,329	117	48	35	32	GRMZM2G028467	3	207,036,751	207,039,713	20	3	3	2
GRMZM2G423202	3	209,050,298	209,053,592	29	10	6	13	XLOC_015662	3	209,050,410	209,053,433	29	10	6	13
GRMZM2G022931	3	210,588,285	210,589,844	3	2	2	1	XLOC_017959	3	210,588,766	210,589,321	3	2	2	1
GRMZM2G023436	3	210,617,138	210,619,025	11	8	4	6	XLOC_015686	3	210,617,343	210,618,206	1	0	0	0
GRMZM2G122362	3	211,203,728	211,209,219	7	2	1	2	XLOC_017967	3	211,203,731	211,204,235	4	1	0	1
GRMZM2G125044	3	211,402,697	211,410,523	58	20	14	21	XLOC_017970	3	211,402,750	211,403,115	17	2	1	0
GRMZM2G344848	3	211,845,106	211,846,918	14	6	6	5	XLOC_017976	3	211,845,283	211,846,698	14	6	6	5
GRMZM5G814314	3	212,517,687	212,521,608	16	5	2	4	XLOC_017987	3	212,517,867	212,521,224	1	1	1	0
GRMZM2G330302	3	213,469,179	213,475,662	8	5	3	4	XLOC_015723	3	213,472,943	213,473,385	-	-	-	-
GRMZM2G022311	3	213,639,419	213,641,365	38	12	11	14	XLOC_015728	3	213,639,551	213,641,474	37	11	10	13
GRMZM2G105140	3	217,542,866	217,545,519	43	24	22	20	XLOC_018046	3	217,543,819	217,544,737	28	15	15	15
GRMZM2G116584	3	220,604,720	220,608,431	29	8	8	8	XLOC_015815	3	220,604,720	220,608,764	29	8	8	8
GRMZM2G087675	3	221,653,089	221,654,953	18	1	4	3	GRMZM2G087628	3	221,649,424	221,654,718	13	4	5	6
GRMZM2G019807	3	221,718,648	221,720,095	15	1	4	2	XLOC_018120	3	221,718,415	221,719,225	15	1	4	2
GRMZM2G071021	3	221,771,306	221,775,473	26	15	15	11	XLOC_018125	3	221,774,725	221,775,130	-	-	-	-
GRMZM2G058021	3	223,930,441	223,931,772	5	3	2	4	XLOC_015881	3	223,930,610	223,931,905	5	3	2	4
XLOC_015910	3	226,500,341	226,500,972	3	3	3	3	XLOC_018198	3	226,500,327	226,500,904	3	3	3	3
GRMZM2G071154	3	226,856,807	226,859,571	21	16	15	14	GRMZM2G071113	3	226,851,477	226,859,535	47	13	8	7
GRMZM2G368491	3	227,565,909	227,567,701	29	8	11	9	XLOC_015929	3	227,566,067	227,567,279	29	8	11	9
GRMZM2G344924	3	229,070,754	229,076,299	23	11	7	12	XLOC_015957	3	229,070,973	229,071,445	14	8	5	8
GRMZM2G001457	3	229,218,369	229,241,730	24	8	7	8	XLOC_018225	3	229,218,227	229,218,934	30	15	16	14
GRMZM2G467123	3	229,640,542	229,644,275	12	6	6	5	XLOC_018233	3	229,640,519	229,641,139	-	-	-	-
GRMZM2G005749	3	230,083,969	230,085,238	3	1	1	1	GRMZM2G005834	3	230,083,958	230,086,240	3	1	1	1
GRMZM2G054703	3	230,522,661	230,526,086	23	7	7	7	XLOC_018251	3	230,523,385	230,523,682	3	0	1	1
GRMZM2G177912	3	230,593,917	230,599,898	10	4	5	5	GRMZM2G177895	3	230,599,509	230,600,829	21	10	8	10
GRMZM2G092008	3	231,198,433	231,200,286	-	-	-	-	GRMZM2G092021	3	231,196,835	231,205,771	8	1	1	2
GRMZM2G397661	4	32,363	33,792	14	2	2	1	GRMZM2G000510	4	32,440	63,818	36	3	4	3
GRMZM2G018254	4	512,986	515,273	21	7	11	4	XLOC_018284	4	513,590	515,381	14	5	7	1
GRMZM2G073823	4	829,560	832,103	56	17	12	19	XLOC_018292	4	831,188	832,279	18	6	4	7
GRMZM2G073805	4	832,506	835,056	40	11	9	10	XLOC_018293	4	832,823	835,056	39	11	9	10
GRMZM2G164224	4	1,070,157	1,075,181	42	18	20	13	XLOC_018304	4	1,070,183	1,074,395	42	18	20	13
XLOC_020442	4	1,078,823	1,079,883	7	1	1	2	XLOC_018305	4	1,078,335	1,079,772	7	1	1	2
GRMZM2G171036	4	2,386,082	2,387,319	25	5	7	7	XLOC_020472	4	2,385,958	2,386,696	11	0	0	0
GRMZM2G126453	4	2,755,561	2,766,689	6	2	2	2	XLOC_020489	4	2,765,969	2,766,352	-	-	-	-
GRMZM2G070429	4	3,483,206	3,488,614	37	8	8	7	XLOC_018349	4	3,487,958	3,489,663	1	0	0	0
GRMZM2G165325	4	4,790,674	4,792,253	16	7	10	8	XLOC_020510	4	4,791,414	4,792,218	14	6	9	8
GRMZM5G877647	4	5,006,400	5,007,839	11	7	4	3	XLOC_018393	4	5,006,764	5,007,111	-	-	-	-
AC212755.4_FG001	4	6,247,911	6,249,250	7	2	1	1	XLOC_018421	4	6,248,121	6,248,792	7	2	1	1
GRMZM2G027021	4	9,490,215	9,499,465	13	4	10	11	XLOC_020556	4	9,490,202	9,491,088	11	2	8	9
GRMZM2G113470	4	9,896,505	9,902,617	20	8	10	12	XLOC_018453	4	9,902,135	9,902,425	-	-	-	-
GRMZM2G581155	4	14,054,454	14,059,644	58	20	19	18	XLOC_020600	4	14,054,715	14,055,963	23	9	7	8
GRMZM2G091481	4	14,877,039	14,881,740	14	4	3	4	XLOC_018498	4	14,877,134	14,877,930	1	1	0	0
GRMZM2G025109	4	15,037,603	15,039,172	11	1	2	2	XLOC_020611	4	15,037,278	15,038,658	8	0	1	1
XLOC_018508	4	15,842,899	15,846,425	4	0	1	2	XLOC_020616	4	15,843,477	15,844,369	1	0	0	0
GRMZM2G080168	4	17,257,026	17,259,706	47	19	24	17	XLOC_020630	4	17,257,105	17,258,600	38	19	23	17
GRMZM2G139894	4	18,610,025	18,616,532	15	4	2	2	XLOC_018536	4	18,610,785	18,611,676	-	-	-	-
GRMZM2G574782	4	18,698,719	18,705,550	26	12	10	11	XLOC_020646	4	18,698,629	18,703,364	16	6	6	6
GRMZM2G177668	4	19,133,772	19,136,181	21	6	5	5	XLOC_018538	4	19,134,262	19,135,158	16	5	4	4
GRMZM2G089995	4	26,450,748	26,452,606	39	15	18	10	XLOC_020702	4	26,452,045	26,454,670	38	18	20	21
GRMZM2G089995	4	26,450,748	26,452,606	39	15	18	10	XLOC_020701	4	26,452,045	26,454,285	38	18	20	21
GRMZM2G146862	4	26,847,982	26,889,010	44	10	15	8	XLOC_020709	4	26,852,157	26,852,616	-	-	-	-
GRMZM2G382323	4	29,179,242	29,181,600	10	4	4	3	XLOC_018633	4	29,179,242	29,181,499	10	4	4	3
GRMZM2G122277	4	31,271,410	31,278,209	16	6	6	6	XLOC_020746	4	31,276,128	31,277,846	9	4	3	4
AC196475.3_FG004	4	32,278,497	32,280,957	9	2	1	1	XLOC_018658	4	32,278,467	32,280,852	17	5	2	3
GRMZM2G074280	4	36,017,140	36,020,195	18	6	1	2	GRMZM2G074087	4	36,019,078	36,019,888	16	5	2	5
GRMZM2G046743	4	36,911,334	36,914,601	11	5	6	1	XLOC_020787	4	36,912,206	36,913,001	-	-	-	-
GRMZM2G046804	4	36,913,994	36,917,730	27	8	11	5	GRMZM2G046743	4	36,911,334	36,914,601	11	5	6	1
GRMZM2G363540	4	37,262,877	37,263,974	1	0	1	1	XLOC_018714	4	37,262,864	37,263,878	1	0	1	1
GRMZM2G324643	4	37,603,564	37,604,830	2	1	1	1	XLOC_020801	4	37,603,712	37,604,136	-	-	-	-
GRMZM2G105438	4	38,788,778	38,791,422	4	1	0	0	GRMZM2G105387	4	38,791,036	38,792,802	49	12	16	13
GRMZM2G135568	4	39,633,113	39,637,478	41	16	7	4	GRMZM2G135547	4	39,629,147	39,633,705	31	6	5	5
GRMZM2G056573	4	40,005,741	40,012,885	53	20	23	22	XLOC_020835	4	40,012,198	40,012,616	-	-	-	-
GRMZM2G075496	4	42,127,982	42,131,478	40	16	17	20	XLOC_018755	4	42,128,033	42,129,663	19	8	5	7
GRMZM2G006953	4	44,885,720	44,888,367	2	2	1	2	XLOC_018775	4	44,887,934	44,888,291	-	-	-	-
GRMZM2G100583	4	46,961,515	46,966,149	17	8	10	9	XLOC_020878	4	46,961,562	46,962,159	7	4	4	4
GRMZM2G107289	4	47,746,128	47,751,481	9	1	0	1	XLOC_018785	4	47,750,743	47,751,284	3	1	0	1
GRMZM2G121928	4	60,384,110	60,385,046	5	2	0	0	XLOC_018846	4	60,384,206	60,385,041	5	2	0	0
GRMZM2G011806	4	61,742,444	61,748,096	16	6	4	5	XLOC_018851	4	61,742,780	61,743,475	14	4	4	3
GRMZM5G884985	4	63,028,898	63,030,916	21	8	8	7	XLOC_020967	4	63,028,730	63,030,827	21	8	8	7
GRMZM2G119071	4	63,118,826	63,119,812	6	5	6	5	XLOC_018861	4	63,119,243	63,119,725	5	4	5	4
GRMZM2G082087	4	63,562,329	63,566,123	21	1	6	5	XLOC_018862	4	63,562,639	63,563,315	17	1	6	4
GRMZM2G352132	4	67,045,579	67,052,872	63	28	30	27	XLOC_020984	4	67,051,659	67,052,660	1	1	0	1
GRMZM2G359986	4	67,675,287	67,679,206	50	16	21	15	XLOC_018894	4	67,677,536	67,679,076	42	15	18	11
GRMZM2G174732	4	71,935,082	71,945,964	26	10	15	11	XLOC_021018	4	71,934,793	71,936,351	8	3	4	4

GRMZM2G091478	4	74,338,529	74,349,897	-	-	-	-	XLOC_021034	4	74,338,305	74,339,402	4	2	2	2
GRMZM2G066599	4	76,410,462	76,412,170	64	22	24	26	XLOC_021046	4	76,410,602	76,410,931	3	2	2	0
XLOC_018952	4	77,234,530	77,237,022	16	5	3	4	XLOC_021048	4	77,234,574	77,236,038	16	5	3	4
GRMZM2G091155	4	81,729,034	81,732,613	5	2	3	2	XLOC_018979	4	81,729,329	81,729,643	-	-	-	-
GRMZM2G089698	4	86,308,802	86,310,939	10	3	5	3	XLOC_018991	4	86,309,261	86,310,675	10	3	5	3
XLOC_021125	4	92,859,072	92,860,700	17	6	4	2	XLOC_019017	4	92,860,154	92,860,755	-	-	-	-
GRMZM2G147279	4	116,891,237	116,893,515	1	1	1	1	GRMZM2G147288	4	116,886,271	116,892,167	3	0	0	0
GRMZM5G898290	4	125,797,420	125,799,414	15	0	1	2	XLOC_021244	4	125,798,578	125,799,254	7	0	1	1
GRMZM2G133568	4	126,033,031	126,036,615	-	-	-	-	GRMZM2G133563	4	126,025,307	126,037,302	10	5	3	3
GRMZM2G407996	4	131,204,417	131,207,606	12	2	4	3	XLOC_021276	4	131,205,067	131,205,607	3	0	1	1
GRMZM2G107896	4	135,677,987	135,707,206	13	4	4	5	XLOC_019221	4	135,678,353	135,678,697	1	0	1	0
XLOC_021322	4	141,306,653	141,307,645	22	12	11	12	GRMZM2G159700	4	141,306,798	141,312,900	22	12	11	12
GRMZM2G127857	4	142,662,836	142,664,368	9	4	4	4	XLOC_019246	4	142,663,441	142,663,645	6	3	3	3
GRMZM2G081582	4	143,491,590	143,497,192	29	5	7	7	XLOC_019252	4	143,491,887	143,494,313	21	5	5	7
GRMZM2G081192	4	143,891,282	143,894,290	29	10	14	13	XLOC_021338	4	143,891,905	143,892,153	-	-	-	-
GRMZM2G129569	4	149,310,880	149,317,247	10	1	1	1	XLOC_019297	4	149,311,160	149,312,065	9	1	1	1
GRMZM2G145226	4	151,675,886	151,682,478	11	7	8	7	GRMZM2G145221	4	151,682,337	151,684,641	18	5	2	4
GRMZM2G145221	4	151,682,337	151,684,641	18	5	2	4	XLOC_019309	4	151,683,084	151,684,683	9	4	1	2
GRMZM2G026922	4	152,425,543	152,433,694	19	4	11	4	GRMZM2G323719	4	152,424,058	152,425,840	19	4	11	4
GRMZM2G102502	4	152,813,342	152,818,202	13	0	1	5	XLOC_019324	4	152,817,608	152,817,855	-	-	-	-
GRMZM2G392975	4	153,654,778	153,659,027	15	4	4	4	XLOC_021399	4	153,654,840	153,658,135	10	1	1	2
GRMZM2G392975	4	153,654,778	153,659,027	15	4	4	4	GRMZM2G093316	4	153,658,281	153,668,278	22	9	11	7
GRMZM2G114998	4	155,035,662	155,038,571	35	14	15	13	XLOC_019354	4	155,037,503	155,038,790	-	-	-	-
GRMZM2G028438	4	156,049,488	156,052,142	63	26	27	33	XLOC_019360	4	156,050,218	156,052,167	62	25	25	31
GRMZM2G134023	4	156,217,617	156,219,742	13	4	4	4	XLOC_019362	4	156,218,446	156,219,862	4	0	0	0
XLOC_021429	4	156,443,715	156,445,719	6	2	3	2	GRMZM2G528010	4	156,439,025	156,444,649	12	5	5	4
GRMZM2G081037	4	156,981,541	156,986,962	7	3	2	2	GRMZM2G081172	4	156,986,015	156,987,610	2	0	0	1
GRMZM2G054354	4	158,166,367	158,170,289	21	5	7	7	GRMZM2G054013	4	158,169,952	158,174,569	24	9	13	11
GRMZM2G054354	4	158,166,367	158,170,289	21	5	7	7	XLOC_021449	4	158,166,397	158,167,576	-	-	-	-
GRMZM2G350157	4	158,326,931	158,329,099	12	5	4	1	XLOC_019396	4	158,328,012	158,329,138	11	4	3	1
GRMZM2G066615	4	158,364,982	158,372,319	26	13	12	11	XLOC_021453	4	158,370,468	158,372,295	-	-	-	-
GRMZM2G121075	4	158,585,836	158,587,198	6	1	1	1	XLOC_019399	4	158,586,700	158,587,145	6	1	1	1
GRMZM2G119258	4	160,367,219	160,374,647	20	2	4	4	XLOC_019416	4	160,367,021	160,368,068	10	1	1	0
GRMZM2G022514	4	161,579,119	161,583,314	83	32	40	35	XLOC_019440	4	161,579,943	161,581,091	28	10	13	11
GRMZM2G309950	4	163,674,452	163,675,848	3	2	3	1	GRMZM2G011085	4	163,672,570	163,675,007	3	2	3	1
GRMZM2G021339	4	165,861,729	165,864,137	11	5	4	5	XLOC_021540	4	165,861,367	165,863,784	13	7	6	6
GRMZM2G471733	4	166,895,031	166,897,669	30	13	15	12	XLOC_021547	4	166,895,138	166,896,844	21	9	11	8
GRMZM2G036464	4	167,111,209	167,114,536	23	9	6	8	XLOC_019498	4	167,111,036	167,113,698	30	11	8	12
GRMZM2G017852	4	168,574,502	168,577,580	31	10	10	10	GRMZM2G017365	4	168,576,413	168,580,294	38	10	12	11
GRMZM2G082007	4	169,989,941	169,993,608	24	9	7	10	GRMZM2G082222	4	169,987,963	169,990,290	16	7	6	8
GRMZM2G081843	4	170,004,620	170,006,968	7	3	2	2	XLOC_019521	4	170,004,832	170,006,074	7	3	2	2
GRMZM2G146207	4	170,880,755	170,885,518	34	4	9	4	GRMZM2G146228	4	170,884,819	170,886,073	18	3	6	2
GRMZM2G466545	4	171,946,372	171,951,467	59	24	23	26	GRMZM2G164854	4	171,951,028	171,955,087	10	4	5	4
GRMZM2G178398	4	172,777,612	172,783,745	35	11	10	9	XLOC_021629	4	172,782,890	172,783,635	-	-	-	-
GRMZM2G054210	4	173,239,888	173,253,971	28	5	8	6	XLOC_019579	4	173,253,159	173,254,280	-	-	-	-
GRMZM2G316787	4	174,072,810	174,073,935	5	0	1	1	GRMZM2G316778	4	174,072,841	174,074,369	7	0	1	1
GRMZM2G312521	4	174,991,068	174,996,208	39	15	15	16	XLOC_019600	4	174,992,060	174,994,904	21	7	6	5
GRMZM2G314946	4	175,361,055	175,361,851	35	16	10	12	XLOC_021670	4	175,361,439	175,361,691	4	2	0	1
GRMZM2G181359	4	175,765,088	175,769,387	12	4	10	6	GRMZM2G181362	4	175,767,391	175,780,753	21	8	7	7
GRMZM2G170313	4	178,486,128	178,499,296	3	1	1	2	GRMZM2G473016	4	178,498,368	178,500,694	23	4	6	2
GRMZM2G015983	4	178,702,590	178,706,283	20	11	8	11	XLOC_021715	4	178,702,308	178,703,524	4	1	4	3
GRMZM2G041699	4	180,401,112	180,403,035	19	9	9	9	XLOC_019669	4	180,401,155	180,403,087	20	9	9	9
GRMZM2G470524	4	180,463,493	180,465,143	12	2	4	2	GRMZM2G169617	4	180,464,258	180,475,317	41	13	13	10
GRMZM2G322950	4	180,679,873	180,681,240	27	6	8	5	XLOC_019677	4	180,679,954	180,681,492	27	6	8	5
GRMZM2G151107	4	180,924,991	180,926,213	8	1	1	1	XLOC_021744	4	180,924,869	180,925,861	5	0	0	0
GRMZM2G169033	4	181,560,393	181,563,791	9	2	3	3	XLOC_021752	4	181,561,726	181,562,111	-	-	-	-
GRMZM2G041809	4	181,903,993	181,906,445	17	5	4	5	XLOC_021758	4	181,903,925	181,905,990	17	5	4	5
GRMZM2G041959	4	181,917,370	181,923,296	24	9	8	9	XLOC_021759	4	181,921,872	181,922,751	4	3	3	4
GRMZM2G020996	4	183,213,856	183,225,003	38	9	9	9	GRMZM2G318033	4	183,213,169	183,220,384	18	5	5	5
GRMZM2G114052	4	184,148,616	184,152,991	9	2	4	3	XLOC_021790	4	184,149,627	184,150,231	-	-	-	-
GRMZM2G060732	4	185,025,956	185,029,049	10	5	6	6	XLOC_021804	4	185,026,024	185,028,786	24	9	10	10
GRMZM2G060470	4	185,030,955	185,035,131	24	8	12	12	XLOC_021805	4	185,029,082	185,031,304	13	6	7	7
GRMZM2G151516	4	185,209,888	185,211,510	34	17	24	14	XLOC_019743	4	185,210,476	185,210,873	21	11	14	11
GRMZM2G380784	4	185,642,046	185,643,948	35	8	7	11	XLOC_019750	4	185,642,093	185,644,023	6	4	3	3
GRMZM2G179733	4	185,702,075	185,703,839	7	1	2	2	XLOC_019752	4	185,702,432	185,704,873	8	1	2	2
GRMZM2G146553	4	186,214,078	186,220,919	36	13	11	10	GRMZM2G146720	4	186,214,119	186,215,211	3	2	2	2
GRMZM2G171605	4	186,764,494	186,766,965	48	7	8	7	XLOC_021845	4	186,764,601	186,764,876	22	2	3	3
GRMZM2G095141	4	186,832,765	186,835,999	12	3	2	5	GRMZM2G095326	4	186,835,570	186,841,247	-	-	-	-
GRMZM2G051787	4	188,475,242	188,477,024	13	2	2	2	XLOC_019790	4	188,475,513	188,478,292	9	1	1	1
GRMZM2G030877	4	188,665,523	188,672,996	52	24	20	24	XLOC_019795	4	188,667,884	188,669,203	6	3	1	3
GRMZM2G030877	4	188,665,523	188,672,996	52	24	20	24	GRMZM2G031094	4	188,663,683	188,665,638	4	1	2	2
GRMZM2G451792	4	195,018,290	195,032,289	60	17	18	19	XLOC_021953	4	195,030,508	195,032,201	5	2	2	1
GRMZM2G149843	4	195,797,433	195,801,279	20	7	7	3	GRMZM2G451366	4	195,800,111	195,805,721	29	8	17	7
GRMZM2G163671	4	195,975,252	195,979,088	31	11	14	7	XLOC_019888	4	195,975,699	195,976,927	7	3	5	3
GRMZM2G440925	4	197,104,904	197,106,971	11	2	3	3	XLOC_021978	4	197,104,888	197,106,605	11	2	3	3
GRMZM2G332522	4	198,256,542	198,257,719	33	14	9	12	XLOC_019910	4	198,256,600	198,257,361	21	10	7	8
GRMZM2G332809	4	198,946,845	198,953,299	34	7	10	11	GRMZM2G033228	4	198,951,228	198,952,967	22	6	10	8
GRMZM2G046111	4	201,141,912	201,143,147	14	3	3	4	XLOC_022029	4	201,141,953	201,142,723	14	3	3	4
GRMZM2G079491	4	201,299,690	201,302,345	30	7	12	5	XLOC_022031	4	201,299,625	201,301,921	30	7	12	5
AC216067.3_FG002	4	204,522,509	204,525,059	8	3	1	1	XLOC_019986	4	204,522,703	204,522,947	-	-	-	-
GRMZM2G059706	4	206,672,898	206,674,203	5	3	3	2	XLOC_019997	4	206,673,299	206,674,219	5	3	3	2
GRMZM2G089856	4	209,050,229	209,052,176	4	1	2	1	XLOC_020014	4	209,051,277	209,051,536	-	-	-	-
GRMZM2G071478	4	211,330,470	211,333,066	29	13	13	14	XLOC_022113	4	211,331,241	211,332,851	18	9	10	10
GRMZM2G014071	4	212,126,276	212,130,478	15	6	5	7	XLOC_022119	4	212,126,428	212,127,100	6	1	2	3
GRMZM2G458728	4	212,853,674	212,859,386	28	5	6	6	XLOC_020036	4	212,857,831	212,859,418	-	-	-	-
GRMZM2G079365	4	213,863,768	213,880,142	29	4	5	8	XLOC_022130	4	213,864,442	213,865,170	6	1	2	2
GRMZM2G590333	4	215,441,250	215,445,850	25	3	8	4	XLOC_020056	4	215,442,637	215,445,193	-	-	-	-
XLOC_020133	4	225,418,358	225,420,404	6	2	4	5	XLOC_022215	4	225,418,052	225,419,587	6	2	4	5
GRMZM2G052920	4	226,108,085	226,108,881	27	14	14	11	XLOC_022221	4	226,107,900	226,108,706	27	14	14	11
GRMZM2G035077	4	226,605,254	226,607,090	24	11	12	13	XLOC_020146	4	226,606,273	226,606,895	-	-	-	-
GRMZM2G033855	4	226,843,654	226,844,563	30	8	14	9	XLOC_022237	4	226,843,625	226,844,335	32	8	16	9
GRMZM2G017022	4	228,425,361	228,428,399	20	5	6	5	XLOC_022248	4	228,426,856	228,427,885	16	8	7	7
GRMZM2G069631	4	230,038,398	230,047,846	19	5	5	6	XLOC_022270	4	230,046,909	230,047,649	2	0	1	0
GRMZM2G022538	4	231,263,824	231,272,537	13	3	3	3	XLOC_022280	4	231,270,481	231,272,402	8	2	1	2
GRMZM2G015419	4	234,164,884	234,168,656	63	27	25	21	XLOC_022325	4	234,165,958	234,168,605	33	17	14	11
GRMZM2G008859	4	235,108,555	235,111,831	21	8	11	6	XLOC_020246	4	235,108,395	235,110,759	15	6	7	5
GRMZM2G125308	4	235,227,459	235,229,436	40	17	14	13	XLOC_022347	4	235,228,487	235,229,021	9	4	3	3
XLOC_020263	4	235,657,814	235,659,734	40	16	23	22	XLOC_022355	4	235,658,484	235,659,038	27	9	16	15
XLOC_022369	4	236,262,710	236,264,243	34	10	14	13	XLOC_020286	4	236,263,993	236,264,323	4	1	1	1
XLOC_022369	4	236,262,710	236,264,243	34	10	14	13	XLOC_020285	4	236,263,278	236,263,657	6	2	1	3
GRMZM2G303374	4	237,811,718	237,813,201	20	10	10	8	GRMZM2G003157	4	237,801,951	237,811,959	36	6	7	5
XLOC_020330	4	237,855,481	237,858,140	22	6	8	4	XLOC_022406	4	237,855,270	237,857,865	22	6	8	4
GRMZM2G166035	4	238,052,080	238,055,542	29	13	14	10	XLOC_020341	4	238,052,108	238,052,348	11	6	6	5
XLOC_022421	4	238,664,013	238,666,194	42	14	15	13	XLOC_020354	4	238,665,605	238,666,775	25	8	9	9
GRMZM2G060451	4	238,872,905	238,878,643	38	12	16	13	XLOC_020356	4	238,872,952	238,873,626	20	3	5	6
GRMZM2G060451	4	238,872,905	238,878,643	38	12	16	13	XLOC_020358	4	238,874,856	238,875,709	-	-	-	-
GRMZM2G027209	4	239,288,712	239,300,856	24	7	6	7	GRMZM2G027640	4	239,288,344	239,289,063	3	1	2	1
GRMZM2G324956	4	239,528,888	239,529,910	5	0	1	0	XLOC_022453	4	239,529,220	239,529,624	1	0	0	0
GRMZM2G169998	4	240,049,949	240,053,626	7	5	5	1	XLOC_020387	4	240,053,313	240,053,631	5	3	3	1
GRMZM2G025127	4	240,272,545	240,276,538	24	7	10	9	XLOC_022492	4	240,275,454	240,276,216	15	2	3	3
GRMZM2G014170	5	803,571	810,274	17	3	5	2	XLOC_022534	5	803,629	804,211	1	0	1	0
AC190636.3_FG005	5	813,840	815,338	4	1	1	0	GRMZM2G019866	5	812,882	814,072	13	3	3	0
AC190636.3_FG005	5	813,840	815,338	4	1	1	0	XLOC_022536	5	814,586	815,398	7	1	1	0

GRMZM2G384339	5	888,240	893,249	10	4	4	4	GRMZM2G384338	5	892,919	893,892	4	0	0	0
GRMZM2G384070	5	922,448	924,190	25	3	6	4	XLOC_024909	5	923,046	923,893	2	0	0	0
GRMZM2G448001	5	1,526,661	1,529,125	20	7	6	7	GRMZM2G447976	5	1,524,444	1,527,639	12	3	3	3
XLOC_022572	5	1,545,003	1,545,310	10	0	0	0	GRMZM2G148937	5	1,543,474	1,546,975	34	10	10	3
GRMZM2G125784	5	1,582,563	1,583,065	18	3	8	5	XLOC_022577	5	1,582,617	1,583,214	18	3	8	5
GRMZM2G023081	5	1,728,200	1,729,396	30	7	12	6	XLOC_024939	5	1,728,820	1,729,172	-	-	-	-
GRMZM2G138421	5	1,846,419	1,853,070	84	17	23	14	GRMZM2G138494	5	1,843,396	1,848,286	44	5	11	4
GRMZM2G100524	5	1,872,661	1,875,805	22	6	8	7	XLOC_024948	5	1,873,072	1,874,163	11	4	4	2
GRMZM2G121715	5	2,109,630	2,112,133	51	7	11	6	XLOC_024956	5	2,111,143	2,111,810	25	4	7	3
XLOC_024980	5	2,593,362	2,594,082	1	0	1	0	GRMZM2G110952	5	2,593,535	2,595,061	24	5	12	1
GRMZM2G410865	5	2,594,463	2,604,977	17	6	6	6	GRMZM2G110952	5	2,593,535	2,595,061	24	5	12	1
GRMZM2G124441	5	2,926,017	2,928,711	32	11	11	11	XLOC_022628	5	2,927,085	2,928,199	6	3	3	2
GRMZM2G332976	5	3,145,962	3,147,816	4	1	2	1	GRMZM2G038075	5	3,143,125	3,147,996	53	16	19	16
GRMZM2G054224	5	3,777,982	3,779,876	67	18	22	18	XLOC_025015	5	3,779,249	3,781,015	41	13	10	18
GRMZM5G897976	5	3,813,478	3,818,430	17	12	12	4	XLOC_025018	5	3,813,634	3,814,093	-	-	-	-
GRMZM2G009928	5	3,953,080	3,956,606	28	13	10	10	XLOC_025021	5	3,953,124	3,953,974	-	-	-	-
GRMZM2G099474	5	4,312,135	4,317,196	17	5	5	5	GRMZM2G099449	5	4,309,513	4,315,155	8	3	5	2
GRMZM2G099615	5	4,345,261	4,349,288	20	5	9	6	XLOC_025031	5	4,345,145	4,346,123	13	4	7	5
GRMZM2G130544	5	4,423,409	4,425,429	2	0	0	0	XLOC_025033	5	4,423,438	4,427,276	10	0	1	1
GRMZM2G124229	5	4,606,404	4,608,549	22	5	6	4	XLOC_025038	5	4,605,996	4,607,845	29	6	7	6
GRMZM2G029101	5	4,766,059	4,769,132	13	5	5	4	XLOC_025044	5	4,767,236	4,767,975	11	3	3	3
GRMZM2G029101	5	4,766,059	4,769,132	13	5	5	4	XLOC_025045	5	4,768,343	4,769,519	22	8	9	4
GRMZM2G171468	5	6,146,163	6,149,014	39	10	14	12	XLOC_022735	5	6,146,270	6,148,226	39	10	14	12
GRMZM2G035103	5	6,320,889	6,322,687	25	6	6	6	XLOC_025077	5	6,321,319	6,321,822	18	5	4	4
GRMZM2G071970	5	6,914,005	6,917,726	44	10	12	12	XLOC_025097	5	6,916,911	6,920,360	15	2	0	3
GRMZM2G071970	5	6,914,005	6,917,726	44	10	12	12	XLOC_025096	5	6,914,660	6,915,335	9	2	3	3
GRMZM2G077389	5	7,772,722	7,774,847	19	5	3	5	XLOC_022769	5	7,773,900	7,774,829	1	0	0	0
GRMZM2G077295	5	7,781,628	7,787,521	21	4	4	3	GRMZM2G077356	5	7,780,703	7,783,409	27	11	10	8
GRMZM2G147814	5	7,866,856	7,869,861	-	-	-	-	GRMZM2G147809	5	7,866,128	7,870,805	13	2	6	2
GRMZM2G096591	5	9,918,543	9,921,805	43	14	13	7	GRMZM2G096596	5	9,911,089	9,919,240	55	16	14	16
GRMZM2G152548	5	10,240,536	10,243,482	40	19	19	17	XLOC_022819	5	10,242,631	10,243,514	33	16	17	14
GRMZM2G145827	5	10,796,873	10,804,954	26	8	4	13	GRMZM2G145850	5	10,796,396	10,797,296	-	-	-	-
GRMZM2G102616	5	12,237,732	12,238,164	1	1	1	1	XLOC_022856	5	12,237,732	12,238,164	1	1	1	1
GRMZM2G102760	5	12,285,642	12,290,523	39	21	20	11	XLOC_022858	5	12,289,155	12,289,430	1	0	1	1
GRMZM2G176723	5	14,609,748	14,610,757	1	1	1	0	XLOC_022911	5	14,609,902	14,610,741	1	1	1	0
GRMZM2G176688	5	14,610,901	14,625,816	12	4	3	4	GRMZM5G822180	5	14,625,518	14,626,540	32	10	8	6
GRMZM2G066191	5	14,870,256	14,874,075	43	7	10	9	XLOC_022917	5	14,870,816	14,871,240	9	2	2	3
XLOC_025279	5	16,709,490	16,713,961	10	0	3	2	XLOC_022949	5	16,713,440	16,713,755	5	0	2	1
GRMZM2G087326	5	18,789,107	18,791,842	19	6	5	3	XLOC_025299	5	18,789,219	18,789,560	-	-	-	-
GRMZM2G124863	5	19,696,842	19,699,376	32	6	10	6	GRMZM2G124918	5	19,698,812	19,702,780	44	14	19	17
GRMZM2G124918	5	19,698,812	19,702,780	44	14	19	17	XLOC_022991	5	19,699,624	19,702,860	33	11	15	12
GRMZM2G170128	5	20,622,861	20,626,562	27	14	16	12	XLOC_022997	5	20,624,595	20,624,934	1	1	1	0
GRMZM2G132036	5	20,929,213	20,934,778	25	11	12	9	XLOC_023002	5	20,929,481	20,930,339	-	-	-	-
GRMZM5G851862	5	21,362,862	21,366,235	31	6	10	6	XLOC_025335	5	21,362,636	21,363,839	19	2	6	3
GRMZM2G100158	5	22,675,530	22,679,029	23	8	8	6	XLOC_025355	5	22,678,363	22,678,971	-	-	-	-
GRMZM2G113135	5	23,017,949	23,018,871	13	6	6	7	XLOC_025361	5	23,018,093	23,018,717	8	3	4	5
GRMZM2G018695	5	23,677,551	23,679,570	4	1	1	3	XLOC_023042	5	23,677,896	23,679,481	4	1	1	3
GRMZM2G107444	5	24,175,048	24,178,212	24	9	2	10	XLOC_025382	5	24,175,168	24,175,544	12	4	1	3
GRMZM2G073034	5	24,988,574	24,989,582	-	-	-	-	XLOC_023058	5	24,989,489	24,989,908	6	5	5	5
GRMZM2G021973	5	30,326,757	30,327,652	5	0	1	1	XLOC_023106	5	30,327,205	30,327,906	12	1	3	2
GRMZM2G154954	5	31,464,389	31,465,555	21	4	4	3	XLOC_025452	5	31,464,541	31,465,344	17	3	3	3
GRMZM2G326707	5	31,900,737	31,903,637	13	7	5	6	XLOC_025458	5	31,900,586	31,902,800	13	7	5	6
GRMZM2G337048	5	32,245,099	32,247,118	33	9	10	9	XLOC_025464	5	32,244,735	32,247,057	33	9	10	9
GRMZM2G017805	5	35,290,711	35,295,535	7	1	2	1	XLOC_025498	5	35,291,098	35,291,752	2	0	0	1
GRMZM2G043182	5	35,731,107	35,732,791	12	4	7	2	XLOC_025508	5	35,731,101	35,732,701	14	6	9	4
GRMZM2G012209	5	39,895,036	39,906,590	35	9	9	8	XLOC_025549	5	39,904,934	39,908,229	19	8	7	6
GRMZM2G134597	5	39,980,153	39,982,962	28	2	8	8	XLOC_023196	5	39,981,012	39,982,858	28	2	8	8
GRMZM2G366077	5	41,225,312	41,226,535	5	4	1	4	XLOC_025566	5	41,225,477	41,225,885	4	3	0	3
AC209987.4_FG010	5	46,975,199	46,976,425	14	3	3	2	GRMZM5G882378	5	46,973,011	46,976,155	31	6	5	10
GRMZM2G463913	5	48,477,760	48,480,813	9	2	1	1	GRMZM2G163544	5	48,472,809	48,477,815	20	5	5	5
XLOC_025618	5	48,600,426	48,601,438	8	4	1	4	GRMZM5G848124	5	48,600,490	48,601,830	8	4	1	4
GRMZM2G140443	5	48,902,473	48,912,285	2	1	1	0	XLOC_025621	5	48,902,394	48,902,868	-	-	-	-
GRMZM2G141858	5	49,575,119	49,577,760	4	2	0	0	XLOC_025626	5	49,575,144	49,575,550	4	2	0	0
GRMZM2G113655	5	50,115,509	50,120,425	10	4	7	6	XLOC_023265	5	50,119,317	50,120,005	-	-	-	-
GRMZM2G382785	5	50,372,099	50,374,372	15	4	4	7	XLOC_025635	5	50,371,817	50,373,787	24	5	5	8
GRMZM2G026881	5	50,478,793	50,481,470	18	5	4	3	XLOC_023268	5	50,479,714	50,480,691	-	-	-	-
GRMZM2G080183	5	53,425,033	53,427,956	49	22	21	21	XLOC_023299	5	53,425,395	53,426,300	-	-	-	-
GRMZM2G026980	5	53,935,017	53,936,879	43	14	22	9	XLOC_025661	5	53,935,638	53,936,044	6	2	2	1
GRMZM2G099434	5	56,392,388	56,394,936	6	1	0	1	XLOC_025682	5	56,392,398	56,393,951	1	1	0	1
GRMZM2G099413	5	56,397,216	56,398,582	59	26	29	28	XLOC_023317	5	56,396,973	56,397,760	20	7	5	6
GRMZM2G135904	5	59,063,729	59,069,485	3	1	1	1	XLOC_025700	5	59,063,826	59,064,502	3	1	1	1
GRMZM2G135904	5	59,063,729	59,069,485	3	1	1	1	GRMZM2G439203	5	59,068,780	59,072,816	17	8	11	10
GRMZM2G385945	5	59,299,608	59,301,346	4	4	4	4	XLOC_025710	5	59,299,682	59,300,279	4	4	4	4
GRMZM2G085117	5	59,338,041	59,339,527	18	4	1	4	XLOC_025713	5	59,337,823	59,339,561	18	4	1	4
GRMZM2G102447	5	59,753,812	59,756,561	19	14	15	14	XLOC_025722	5	59,754,028	59,754,289	1	1	1	1
XLOC_025737	5	60,683,906	60,685,891	20	6	4	2	XLOC_023360	5	60,684,602	60,685,656	4	2	2	1
XLOC_023371	5	61,454,332	61,457,358	13	4	7	6	GRMZM2G417454	5	61,455,740	61,457,120	4	1	2	2
GRMZM2G399073	5	61,555,926	61,560,984	18	6	9	5	GRMZM2G541730	5	61,554,990	61,556,871	20	8	5	4
GRMZM2G065125	5	62,142,491	62,147,249	10	5	6	2	XLOC_023383	5	62,142,767	62,143,464	-	-	-	-
GRMZM2G048073	5	62,624,370	62,626,140	17	6	4	2	XLOC_023388	5	62,625,055	62,626,051	9	3	1	2
GRMZM2G132331	5	62,711,015	62,712,701	3	1	2	2	XLOC_023389	5	62,711,807	62,712,234	-	-	-	-
XLOC_025771	5	63,306,785	63,308,362	26	9	7	9	XLOC_023396	5	63,305,844	63,307,092	5	1	0	1
GRMZM2G057000	5	65,138,689	65,142,574	19	4	7	4	XLOC_025800	5	65,141,483	65,141,724	-	-	-	-
GRMZM2G055682	5	65,230,942	65,233,276	10	3	5	6	GRMZM2G055619	5	65,233,060	65,236,881	34	8	15	13
GRMZM2G128929	5	67,432,374	67,434,191	9	3	3	1	XLOC_025823	5	67,433,439	67,433,878	1	1	1	0
GRMZM2G159047	5	67,701,021	67,703,449	19	8	6	7	GRMZM2G159142	5	67,697,937	67,701,535	26	6	9	7
GRMZM2G035073	5	68,336,691	68,338,762	23	4	5	10	XLOC_023440	5	68,336,872	68,338,931	23	4	5	10
GRMZM2G088114	5	69,340,886	69,351,028	20	1	2	2	XLOC_025845	5	69,341,489	69,343,791	-	-	-	-
GRMZM2G156824	5	70,111,506	70,115,642	21	3	11	9	XLOC_025853	5	70,114,064	70,115,222	15	2	7	6
GRMZM2G466281	5	70,795,837	70,802,844	53	19	13	15	GRMZM2G164202	5	70,802,312	70,804,163	35	14	9	11
GRMZM2G164242	5	70,805,268	70,812,495	41	8	16	15	XLOC_023479	5	70,804,919	70,806,365	5	1	1	1
GRMZM2G029879	5	73,076,939	73,079,038	32	9	9	11	XLOC_025877	5	73,078,048	73,079,005	5	2	3	3
XLOC_025887	5	74,436,334	74,438,340	16	5	4	3	XLOC_023504	5	74,437,381	74,438,365	16	5	4	3
GRMZM2G171354	5	76,138,603	76,148,199	1	0	0	0	GRMZM2G171349	5	76,144,354	76,147,672	18	10	7	3
AC192244.3_FG007	5	76,249,702	76,252,730	33	12	21	11	XLOC_025907	5	76,252,113	76,255,338	3	2	2	1
GRMZM2G139689	5	76,410,293	76,415,357	40	14	19	19	GRMZM2G139714	5	76,413,820	76,418,130	38	6	9	9
GRMZM2G042278	5	76,714,970	76,718,433	37	20	15	15	GRMZM2G042245	5	76,710,383	76,716,709	41	24	20	19
GRMZM2G010920	5	77,781,686	77,784,624	38	26	28	18	GRMZM2G010779	5	77,780,713	77,781,895	21	9	12	6
GRMZM2G097207	5	77,949,479	77,954,635	7	1	1	1	XLOC_025924	5	77,952,734	77,954,985	14	3	2	9
XLOC_023583	5	81,799,333	81,801,056	9	1	4	2	XLOC_025954	5	81,799,469	81,799,920	-	-	-	-
GRMZM2G088396	5	83,895,830	83,898,120	22	7	7	8	GRMZM2G088235	5	83,896,631	83,902,358	17	7	7	7
GRMZM2G322129	5	84,097,690	84,102,291	22	7	8	7	XLOC_025974	5	84,098,409	84,099,212	4	0	2	2
AC207043.3_FG002	5	86,050,120	86,052,355	30	18	18	15	XLOC_025989	5	86,050,854	86,051,671	18	10	9	7
GRMZM2G180211	5	86,185,986	86,189,477	11	4	4	5	GRMZM2G180218	5	86,185,411	86,186,569	-	-	-	-
GRMZM2G075333	5	89,189,606	89,194,342	88	31	39	28	XLOC_023638	5	89,193,300	89,194,264	17	7	6	5
GRMZM2G057328	5	92,390,468	92,393,828	23	6	9	10	XLOC_026024	5	92,392,744	92,393,814	5	1	5	5
GRMZM2G120320	5	92,841,722	92,844,459	25	9	5	7	XLOC_026028	5	92,842,158	92,842,805	8	2	2	1
GRMZM2G060485	5	93,274,116	93,276,858	18	5	6	3	XLOC_023659	5	93,275,224	93,275,553	2	0	0	0
GRMZM2G117544	5	93,551,459	93,555,969	26	7	10	6	XLOC_023660	5	93,551,777	93,552,691	22	8	7	8
GRMZM2G344163	5	106,298,211	106,301,969	20	7	5	3	XLOC_026084	5	106,298,927	106,299,362	4	0	0	0
GRMZM2G138349	5	118,709,138	118,712,441	4	1	1	1	XLOC_023749	5	118,710,432	118,710,838	1	0	0	0
GRMZM2G029979	5	122,676,304	122,689,150	6	3	1	3	GRMZM2G331374	5	122,688,380	122,692,932	1	0	0	0
GRMZM2G009479	5	123,239,697	123,243,181	15	3	5	4	XLOC_026137	5	123,240,208	123,243,052	10	2	4	3

GRMZM2G429540	5	146,922,333	146,924,650	31	11	13	13	XLOC_023893	5	146,924,241	146,924,930	20	6	7	5
GRMZM2G429540	5	146,922,333	146,924,650	31	11	13	13	GRMZM2G129713	5	146,922,694	146,923,762	36	13	15	15
GRMZM2G026490	5	147,664,347	147,670,122	15	1	1	1	XLOC_023896	5	147,664,563	147,665,713	1	1	1	1
GRMZM2G176206	5	151,922,535	151,926,261	31	7	9	8	XLOC_023914	5	151,923,737	151,926,498	22	6	7	6
XLOC_026313	5	153,416,615	153,417,927	16	4	5	3	XLOC_023924	5	153,416,709	153,417,683	16	4	5	3
GRMZM2G318633	5	155,564,693	155,565,998	9	4	4	2	XLOC_026322	5	155,564,633	155,565,984	9	4	4	2
GRMZM2G028677	5	159,951,449	159,953,399	45	16	18	12	XLOC_026349	5	159,952,069	159,952,709	8	1	1	0
GRMZM2G012046	5	160,693,221	160,699,305	20	8	8	4	XLOC_026357	5	160,692,953	160,693,951	10	3	3	1
GRMZM2G149115	5	162,094,523	162,098,211	36	1	2	2	XLOC_026373	5	162,097,544	162,097,925	2	0	0	0
GRMZM2G430526	5	162,737,531	162,743,559	23	2	3	2	XLOC_023994	5	162,737,140	162,738,369	-	-	-	-
XLOC_023995	5	162,742,238	162,743,559	14	0	0	1	GRMZM2G430526	5	162,737,531	162,743,559	23	2	3	2
GRMZM2G004856	5	163,455,311	163,457,941	43	19	18	17	XLOC_024013	5	163,455,309	163,455,697	-	-	-	-
GRMZM2G004856	5	163,455,311	163,457,941	43	19	18	17	XLOC_024014	5	163,456,033	163,456,790	15	4	5	5
GRMZM2G011631	5	164,380,707	164,401,819	55	35	31	18	XLOC_026392	5	164,401,022	164,401,850	11	3	4	2
GRMZM2G018820	5	164,767,387	164,770,778	6	5	5	5	XLOC_024036	5	164,769,816	164,771,166	-	-	-	-
XLOC_024053	5	167,468,740	167,469,535	12	5	4	4	XLOC_026411	5	167,468,601	167,469,684	12	5	4	4
GRMZM2G020840	5	167,625,007	167,626,599	18	6	5	4	XLOC_024055	5	167,625,144	167,625,890	10	3	3	1
GRMZM5G876303	5	168,572,548	168,575,048	22	8	12	9	XLOC_024063	5	168,573,632	168,574,495	16	7	11	9
GRMZM2G139300	5	169,497,565	169,502,117	59	19	27	20	XLOC_026442	5	169,499,086	169,501,429	39	9	16	11
GRMZM2G139300	5	169,497,565	169,502,117	59	19	27	20	XLOC_026443	5	169,501,558	169,502,071	8	2	3	2
GRMZM2G451314	5	170,458,785	170,463,276	35	12	10	14	XLOC_024086	5	170,462,806	170,463,097	13	3	1	4
GRMZM2G303964	5	170,543,803	170,545,707	8	5	4	6	XLOC_024088	5	170,545,309	170,545,691	-	-	-	-
GRMZM2G303964	5	170,543,803	170,545,707	8	5	4	6	XLOC_024087	5	170,544,174	170,544,600	1	1	1	1
GRMZM2G118825	5	171,317,073	171,318,365	11	5	5	6	XLOC_024096	5	171,317,220	171,318,479	11	5	5	6
XLOC_026470	5	171,563,296	171,567,672	12	0	2	1	GRMZM2G043193	5	171,561,191	171,564,389	9	1	5	3
GRMZM2G031581	5	172,660,909	172,663,831	21	4	5	3	XLOC_026481	5	172,660,909	172,663,243	21	4	5	3
GRMZM2G119517	5	172,851,847	172,858,649	43	16	11	16	GRMZM2G119566	5	172,849,643	172,852,744	4	2	1	2
XLOC_024136	5	174,638,160	174,638,628	1	1	1	0	GRMZM2G421231	5	174,638,160	174,638,454	1	1	1	0
GRMZM2G436328	5	174,664,930	174,666,991	56	20	18	18	XLOC_024137	5	174,664,668	174,665,015	-	-	-	-
GRMZM2G023988	5	175,731,393	175,736,518	9	4	4	5	XLOC_024153	5	175,735,743	175,736,375	6	2	2	3
XLOC_026536	5	176,155,022	176,156,880	22	9	7	8	XLOC_024159	5	176,155,032	176,155,421	2	2	2	2
GRMZM2G132706	5	176,656,120	176,658,005	49	17	6	16	XLOC_026539	5	176,656,189	176,657,863	49	17	6	16
GRMZM2G146380	5	176,985,247	176,986,711	32	13	14	11	XLOC_024174	5	176,985,833	176,986,251	24	11	12	10
GRMZM2G492156	5	177,275,115	177,279,005	8	3	1	4	XLOC_026542	5	177,275,738	177,278,150	8	3	1	4
GRMZM2G044359	5	180,233,335	180,236,245	21	6	6	9	XLOC_026582	5	180,235,632	180,236,130	1	0	0	0
GRMZM2G095194	5	182,500,574	182,506,295	6	5	6	5	GRMZM2G095147	5	182,505,927	182,506,522	-	-	-	-
GRMZM2G129189	5	182,568,960	182,570,172	15	4	4	5	XLOC_026618	5	182,569,224	182,570,100	9	2	2	3
GRMZM2G035584	5	183,657,809	183,662,949	13	2	2	1	XLOC_024267	5	183,658,189	183,662,680	13	2	2	1
GRMZM2G028830	5	185,696,130	185,697,035	12	3	8	3	XLOC_026656	5	185,696,058	185,696,877	12	3	8	3
GRMZM2G141411	5	187,620,886	187,622,518	4	2	2	0	XLOC_024305	5	187,621,682	187,622,099	1	1	1	0
GRMZM2G139765	5	188,716,613	188,718,822	61	20	30	25	XLOC_024321	5	188,718,037	188,718,626	15	7	6	6
GRMZM2G139765	5	188,716,613	188,718,822	61	20	30	25	XLOC_024320	5	188,717,132	188,717,767	29	10	15	10
XLOC_026704	5	188,881,231	188,884,099	38	18	20	18	GRMZM2G169967	5	188,877,054	188,881,772	27	7	4	8
GRMZM2G463291	5	189,855,981	189,862,743	8	2	5	3	XLOC_024338	5	189,857,312	189,858,886	1	1	1	1
GRMZM2G117164	5	190,526,842	190,528,626	16	7	5	6	XLOC_024345	5	190,528,090	190,528,564	8	5	4	4
GRMZM2G156599	5	190,726,076	190,729,337	23	7	6	9	XLOC_024350	5	190,728,306	190,729,357	17	5	5	6
GRMZM2G097704	5	191,140,510	191,142,976	16	6	6	7	XLOC_026733	5	191,141,025	191,142,528	16	6	6	7
GRMZM2G094768	5	191,180,794	191,183,392	32	9	15	14	XLOC_024359	5	191,181,067	191,182,830	30	7	13	12
GRMZM5G840002	5	191,419,331	191,422,728	35	11	10	10	XLOC_026737	5	191,419,426	191,419,679	4	2	2	3
GRMZM2G170591	5	191,730,938	191,735,780	19	2	7	5	XLOC_026742	5	191,734,943	191,735,815	-	-	-	-
AC209208.3_FG002	5	193,359,086	193,363,241	10	2	1	1	AC209208.3_FG001	5	193,363,118	193,371,223	7	6	5	6
GRMZM2G074053	5	194,144,303	194,146,501	44	23	20	18	XLOC_026772	5	194,144,551	194,146,414	41	21	18	17
XLOC_026779	5	194,884,329	194,887,265	29	16	17	12	XLOC_024414	5	194,884,662	194,885,678	19	11	12	10
XLOC_026779	5	194,884,329	194,887,265	29	16	17	12	XLOC_024415	5	194,886,364	194,887,629	-	-	-	-
GRMZM2G154628	5	195,239,858	195,242,631	24	11	11	12	XLOC_026781	5	195,240,448	195,240,744	1	1	1	1
GRMZM5G833032	5	196,333,393	196,347,397	3	3	2	3	XLOC_024432	5	196,336,155	196,336,999	-	-	-	-
GRMZM2G413829	5	198,928,604	198,930,118	-	-	-	-	XLOC_026825	5	198,928,222	198,928,999	8	3	4	4
GRMZM2G141975	5	199,217,605	199,222,280	64	21	21	22	XLOC_024467	5	199,216,642	199,219,395	31	16	15	17
GRMZM2G021270	5	199,912,838	199,920,152	18	4	4	4	XLOC_024483	5	199,913,016	199,913,887	4	1	1	1
GRMZM2G325019	5	200,487,573	200,491,201	17	6	7	7	GRMZM2G325038	5	200,490,849	200,492,345	16	6	7	7
GRMZM2G179147	5	200,704,676	200,707,817	29	13	14	7	XLOC_024496	5	200,705,200	200,707,736	26	12	13	7
GRMZM2G101287	5	200,990,577	200,993,961	9	7	7	1	GRMZM2G101350	5	200,993,550	200,995,952	91	36	44	37
GRMZM2G170595	5	201,723,129	201,726,317	5	1	1	0	GRMZM2G170602	5	201,721,167	201,723,705	49	20	22	22
GRMZM2G009506	5	201,992,788	201,996,158	44	14	16	15	XLOC_026868	5	201,995,204	201,995,662	6	2	3	2
GRMZM2G315176	5	202,034,268	202,050,016	13	3	3	1	XLOC_026869	5	202,049,090	202,049,570	2	0	0	1
GRMZM2G481604	5	202,139,172	202,141,517	11	6	6	7	GRMZM2G180983	5	202,141,025	202,150,154	35	15	11	16
GRMZM2G056252	5	203,092,250	203,097,186	42	13	12	11	XLOC_024545	5	203,092,300	203,093,896	11	4	4	4
GRMZM5G848822	5	203,730,336	203,732,571	8	5	5	3	XLOC_026901	5	203,731,859	203,732,426	-	-	-	-
GRMZM5G848822	5	203,730,336	203,732,571	8	5	5	3	XLOC_026900	5	203,730,871	203,731,291	-	-	-	-
GRMZM2G385999	5	205,283,735	205,284,798	3	1	2	1	XLOC_026933	5	205,284,023	205,284,450	2	0	2	0
GRMZM2G181507	5	205,389,374	205,401,139	65	17	24	27	GRMZM2G481730	5	205,385,840	205,390,104	14	4	5	5
GRMZM2G151204	5	205,591,027	205,593,334	81	36	31	28	XLOC_024629	5	205,591,579	205,593,459	66	31	28	23
GRMZM2G142779	5	205,782,086	205,786,901	57	25	30	29	XLOC_024635	5	205,782,086	205,783,762	16	4	9	10
GRMZM2G142709	5	205,803,187	205,805,607	11	2	2	0	XLOC_024636	5	205,804,786	205,805,352	6	2	1	0
GRMZM5G856201	5	205,888,002	205,897,899	9	2	2	1	XLOC_024639	5	205,896,837	205,897,823	20	6	7	6
GRMZM5G856201	5	205,888,002	205,897,899	9	2	2	1	XLOC_024638	5	205,896,279	205,896,680	9	1	1	2
GRMZM2G335280	5	205,896,071	205,898,040	34	8	9	8	XLOC_024639	5	205,896,837	205,897,823	20	6	7	6
GRMZM2G335280	5	205,896,071	205,898,040	34	8	9	8	XLOC_024638	5	205,896,279	205,896,680	9	1	1	2
GRMZM2G480364	5	205,983,909	205,985,356	8	1	3	2	XLOC_024641	5	205,984,369	205,984,925	8	1	3	2
GRMZM2G179768	5	205,986,279	205,987,943	16	5	6	4	XLOC_024642	5	205,986,559	205,988,233	26	9	10	8
GRMZM2G140651	5	206,668,533	206,670,932	7	6	6	5	XLOC_026963	5	206,668,390	206,669,348	7	6	6	5
GRMZM2G028325	5	206,972,607	206,976,286	22	7	12	9	XLOC_026972	5	206,973,115	206,975,993	13	3	8	5
GRMZM2G035503	5	208,137,640	208,142,328	86	35	41	26	XLOC_024678	5	208,141,589	208,142,230	42	14	14	13
GRMZM2G323013	5	208,973,665	208,975,706	50	17	17	16	XLOC_027011	5	208,973,552	208,975,257	47	12	16	12
GRMZM2G103128	5	209,912,145	209,917,207	24	9	6	3	XLOC_027033	5	209,911,903	209,913,130	2	0	1	1
GRMZM2G113418	5	210,349,025	210,351,808	-	-	-	-	GRMZM2G414621	5	210,350,135	210,352,786	5	2	4	3
GRMZM2G149452	5	210,458,456	210,461,256	35	13	13	15	XLOC_024728	5	210,458,777	210,459,232	7	1	4	4
GRMZM2G154332	5	210,524,837	210,526,166	15	3	5	3	XLOC_024731	5	210,525,082	210,525,511	7	3	4	2
GRMZM2G052422	5	210,825,897	210,828,454	19	7	9	6	XLOC_027066	5	210,827,628	210,828,045	5	1	1	1
GRMZM2G163749	5	212,075,870	212,078,778	51	21	29	18	XLOC_024762	5	212,076,038	212,078,347	37	16	23	12
GRMZM2G124416	5	212,222,159	212,227,637	44	8	11	9	GRMZM2G124377	5	212,213,297	212,223,198	29	4	11	5
XLOC_024786	5	213,088,218	213,090,006	4	0	0	1	XLOC_027111	5	213,088,484	213,089,266	1	0	0	0
GRMZM2G122231	5	213,103,814	213,107,810	29	5	6	6	XLOC_027112	5	213,106,063	213,106,920	14	1	0	1
GRMZM2G469111	5	213,993,280	213,996,291	15	8	10	7	XLOC_024802	5	213,993,902	213,994,790	-	-	-	-
GRMZM2G163200	5	214,574,405	214,580,589	51	21	17	16	XLOC_027154	5	214,574,785	214,576,245	18	5	3	5
GRMZM2G150984	5	215,072,597	215,077,359	29	11	11	8	XLOC_024823	5	215,072,718	215,073,186	8	2	2	3
GRMZM2G058289	5	215,100,492	215,104,749	14	5	5	6	XLOC_027171	5	215,101,686	215,102,542	-	-	-	-
GRMZM5G851655	5	215,112,799	215,116,277	16	9	11	11	XLOC_024826	5	215,113,770	215,114,112	2	1	1	1
GRMZM5G806449	5	215,337,121	215,346,503	20	9	12	7	XLOC_027175	5	215,337,508	215,345,982	13	7	9	6
GRMZM2G078887	5	215,406,418	215,407,900	16	9	12	10	XLOC_024833	5	215,406,977	215,407,577	6	3	4	3
GRMZM5G848768	5	215,868,272	215,872,218	28	13	13	11	XLOC_024851	5	215,868,390	215,868,937	9	4	5	4
GRMZM2G013981	5	215,883,819	215,887,609	12	5	5	4	GRMZM5G894916	5	215,886,969	215,893,442	38	13	9	11
GRMZM2G390076	5	216,124,616	216,127,494	26	6	10	7	GRMZM2G002124	5	216,124,627	216,126,640	18	5	9	6
GRMZM2G133919	5	216,746,254	216,756,209	22	9	10	10	AC208348.3_FG012	5	216,749,139	216,751,299	19	6	8	8
GRMZM2G048497	5	217,060,938	217,063,036	7	0	2	3	GRMZM2G048470	5	217,060,912	217,061,698	6	0	2	3
GRMZM2G375002	5	217,656,072	217,664,485	15	4	5	4	GRMZM2G073609	5	217,664,134	217,665,072	12	4	5	4
GRMZM2G358540	6	655,906	657,038	14	1	3	3	XLOC_027233	6	656,433	656,943	7	0	2	2
GRMZM2G098174	6	1,280,395	1,288,498	24	14	11	9	XLOC_029097	6	1,280,515	1,281,668	23	7	6	7
GRMZM2G367941	6	2,724,260	2,725,829	15	9	11	5	XLOC_027260	6	2,724,149	2,724,788	2	1	1	0
GRMZM2G374827	6	3,041,977	3,053,364	70	30	20	35	XLOC_027263	6	3,052,254	3,053,325	4	1	1	2
GRMZM2G157296	6	3,195,407	3,198,606	20	5	3	3	XLOC_029119	6	3,195,478	3,195,727	4	0	0	1
GRMZM2G117961	6	3,237,181	3,239,818	16	6	11	9	GRMZM2G117984	6	3,239,342	3,252,177	37	9	10	12
GRMZM2G364977	6	6,186,552	6,190,122	57	23	17	17	XLOC_029150	6	6,186,556	6,190,745	63	23	17	19

GRMZM2G009661	6	6,434,660	6,438,643	13	3	3	2	XLOC_027290	6	6,437,835	6,438,475	5	0	0	0
GRMZM2G180659	6	6,892,924	6,896,969	51	31	30	29	XLOC_027296	6	6,893,878	6,895,111	7	2	1	2
GRMZM2G127328	6	7,047,564	7,050,778	32	18	22	17	XLOC_029164	6	7,048,330	7,049,129	6	4	5	4
GRMZM2G068955	6	8,109,337	8,111,820	29	10	11	11	XLOC_029170	6	8,109,205	8,109,622	3	0	0	0
GRMZM2G136662	6	8,112,897	8,136,923	1	1	1	0	XLOC_027310	6	8,116,573	8,118,254	-	-	-	-
GRMZM2G301884	6	9,493,165	9,496,796	3	1	1	0	XLOC_029184	6	9,496,444	9,499,331	19	6	5	5
GRMZM2G134020	6	13,653,638	13,654,912	6	2	1	5	XLOC_029297	6	13,653,532	13,654,945	6	2	1	5
GRMZM2G172980	6	14,969,927	14,974,172	10	3	1	3	XLOC_029332	6	14,970,311	14,970,744	-	-	-	-
GRMZM2G155252	6	21,463,563	21,465,430	11	3	5	4	XLOC_027524	6	21,464,815	21,465,424	3	2	3	2
GRMZM2G448607	6	24,279,652	24,282,016	19	10	11	10	XLOC_027540	6	24,281,335	24,281,874	6	5	5	5
GRMZM2G119583	6	24,318,350	24,320,654	6	0	0	0	AC203408.4_FG003	6	24,316,692	24,320,269	13	0	2	1
GRMZM2G111579	6	24,549,334	24,554,738	6	3	4	3	XLOC_027544	6	24,553,394	24,554,144	-	-	-	-
GRMZM2G016637	6	25,700,899	25,705,088	7	3	1	4	XLOC_027546	6	25,702,321	25,703,777	-	-	-	-
GRMZM2G531804	6	26,716,656	26,722,230	16	6	5	8	AC203093.3_FG001	6	26,716,656	26,717,457	-	-	-	-
GRMZM2G381051	6	27,107,889	27,118,645	21	6	5	4	GRMZM2G381086	6	27,118,145	27,120,852	24	10	9	7
GRMZM2G020091	6	28,129,083	28,173,808	-	-	-	-	GRMZM2G313448	6	28,140,500	28,146,597	118	58	63	70
GRMZM2G099317	6	31,415,780	31,420,025	13	4	4	4	XLOC_029449	6	31,419,080	31,419,330	5	2	2	2
GRMZM2G013581	6	34,825,142	34,827,192	28	2	9	3	XLOC_029470	6	34,825,979	34,826,788	7	1	2	2
GRMZM2G305856	6	34,888,892	34,890,920	47	14	16	15	XLOC_027591	6	34,889,334	34,891,116	47	14	17	15
GRMZM2G035741	6	35,892,962	35,896,659	30	12	16	13	GRMZM2G037140	6	35,896,003	35,897,785	3	3	3	3
GRMZM2G109821	6	36,088,959	36,091,188	25	12	12	12	XLOC_029482	6	36,090,380	36,090,741	1	0	0	0
GRMZM2G066326	6	39,529,258	39,530,806	39	14	12	14	XLOC_027626	6	39,529,339	39,530,458	14	7	5	6
GRMZM2G312970	6	39,822,086	39,827,373	18	7	6	6	XLOC_027629	6	39,822,601	39,823,721	3	0	0	0
GRMZM2G463462	6	55,754,752	55,763,319	36	16	13	11	GRMZM2G164916	6	55,760,353	55,764,329	18	3	3	4
GRMZM2G107073	6	56,261,420	56,264,391	10	5	4	6	GRMZM2G453388	6	56,251,970	56,263,608	9	4	4	4
GRMZM2G385287	6	56,376,617	56,378,293	10	0	0	2	XLOC_027699	6	56,377,804	56,378,194	-	-	-	-
GRMZM2G127798	6	57,903,594	57,906,115	54	16	13	17	XLOC_027707	6	57,903,816	57,905,297	30	9	7	8
GRMZM2G155943	6	59,237,967	59,293,671	55	13	15	9	XLOC_029599	6	59,260,577	59,261,040	-	-	-	-
GRMZM2G456023	6	61,126,264	61,140,722	13	4	4	4	XLOC_029613	6	61,126,233	61,127,035	5	2	3	2
GRMZM2G077837	6	62,703,857	62,705,991	24	15	15	15	XLOC_029621	6	62,703,742	62,705,554	18	13	13	13
GRMZM2G069335	6	63,696,202	63,697,776	21	9	8	9	XLOC_027748	6	63,697,116	63,697,832	15	7	5	6
GRMZM2G430902	6	67,615,156	67,617,583	24	6	7	6	XLOC_029644	6	67,615,942	67,616,607	7	1	1	1
GRMZM2G447347	6	70,452,079	70,454,503	16	5	5	4	XLOC_027793	6	70,453,959	70,454,244	6	2	2	2
GRMZM2G362298	6	74,071,296	74,073,747	8	2	3	2	XLOC_029681	6	74,072,319	74,072,936	-	-	-	-
GRMZM2G362298	6	74,071,296	74,073,747	8	2	3	2	XLOC_029680	6	74,071,067	74,072,057	8	2	3	2
GRMZM2G419542	6	76,846,012	76,848,163	12	5	5	2	XLOC_029697	6	76,847,017	76,847,473	-	-	-	-
GRMZM2G700968	6	78,786,923	78,787,431	9	2	3	4	GRMZM2G700969	6	78,785,552	78,790,300	3	1	1	1
GRMZM2G020912	6	82,128,247	82,135,184	20	7	8	8	XLOC_029728	6	82,129,100	82,129,534	-	-	-	-
GRMZM2G065888	6	83,989,254	83,994,612	27	8	9	8	XLOC_027890	6	83,989,400	83,990,186	11	2	2	2
GRMZM2G065105	6	84,094,029	84,098,506	8	0	2	1	XLOC_029744	6	84,094,292	84,094,834	7	0	2	1
GRMZM2G308689	6	85,681,129	85,683,710	33	20	17	19	XLOC_027905	6	85,683,407	85,683,665	4	1	1	1
GRMZM2G365961	6	85,959,795	85,961,400	14	5	8	4	XLOC_029759	6	85,959,727	85,961,669	15	5	9	4
GRMZM2G134930	6	86,045,729	86,048,942	2	1	1	0	XLOC_029760	6	86,045,763	86,046,735	2	1	1	0
GRMZM2G134759	6	86,050,769	86,052,508	10	3	3	4	XLOC_027911	6	86,051,785	86,052,435	10	3	3	4
XLOC_029764	6	86,345,343	86,347,299	31	14	9	13	XLOC_027915	6	86,345,625	86,347,854	33	15	10	14
GRMZM2G434935	6	88,008,788	88,010,435	17	3	2	3	XLOC_027935	6	88,009,296	88,010,525	30	14	13	14
GRMZM2G175562	6	88,908,389	88,909,796	1	0	0	0	GRMZM2G175543	6	88,909,489	88,912,116	9	2	4	1
GRMZM2G150209	6	89,061,130	89,064,359	31	16	16	17	XLOC_027797	6	89,061,498	89,062,022	-	-	-	-
GRMZM2G150256	6	89,066,375	89,071,273	43	25	25	25	XLOC_027953	6	89,068,336	89,068,774	8	6	6	5
GRMZM2G150276	6	89,072,232	89,074,190	53	23	23	19	XLOC_027956	6	89,072,372	89,073,406	26	10	10	8
GRMZM2G089400	6	89,228,410	89,230,672	7	3	2	3	XLOC_027964	6	89,230,128	89,230,670	2	0	1	1
GRMZM2G119714	6	91,723,056	91,733,893	2	0	2	0	XLOC_029829	6	91,731,581	91,733,115	2	0	2	0
GRMZM2G043822	6	92,976,168	92,979,012	15	7	7	7	XLOC_027994	6	92,978,624	92,978,883	-	-	-	-
GRMZM2G048092	6	93,375,032	93,377,483	4	1	1	1	GRMZM2G047969	6	93,373,671	93,375,664	13	2	3	3
GRMZM2G420715	6	93,749,148	93,750,906	26	5	5	5	XLOC_028005	6	93,749,599	93,750,313	19	2	1	2
GRMZM2G420715	6	93,749,148	93,750,906	26	5	5	5	XLOC_028006	6	93,750,550	93,750,889	27	15	16	13
GRMZM2G473162	6	94,663,619	94,665,309	13	5	5	6	GRMZM2G172413	6	94,661,977	94,664,066	3	2	2	2
GRMZM2G082118	6	98,420,954	98,422,447	11	3	5	3	XLOC_029909	6	98,420,496	98,422,255	30	7	9	5
GRMZM2G041714	6	100,364,390	100,365,388	32	12	16	15	XLOC_028083	6	100,364,490	100,365,727	32	12	16	15
GRMZM2G043310	6	102,760,900	102,765,449	23	4	4	3	XLOC_028096	6	102,764,632	102,765,222	17	2	2	1
GRMZM2G310144	6	103,569,642	103,577,833	12	10	8	0	XLOC_028103	6	103,569,817	103,570,762	6	6	6	0
GRMZM2G335618	6	104,021,742	104,022,644	13	8	8	8	XLOC_028114	6	104,021,788	104,022,587	13	8	8	8
GRMZM2G032028	6	104,030,544	104,033,188	11	2	2	0	XLOC_028115	6	104,031,359	104,032,086	11	2	2	0
GRMZM2G449817	6	104,838,658	104,842,995	51	11	12	8	XLOC_028123	6	104,839,450	104,840,850	18	2	3	2
GRMZM2G348846	6	105,706,618	105,708,350	40	9	7	8	XLOC_029967	6	105,706,541	105,708,349	40	9	7	8
XLOC_029975	6	106,612,044	106,612,947	17	8	9	8	XLOC_028149	6	106,612,472	106,613,000	10	4	5	4
XLOC_028172	6	108,236,815	108,241,211	27	11	10	9	XLOC_029999	6	108,237,703	108,238,301	8	1	2	2
GRMZM2G025491	6	109,558,799	109,560,880	20	4	5	3	XLOC_030013	6	109,558,845	109,560,431	15	4	4	2
GRMZM2G136058	6	110,002,951	110,008,161	15	3	8	2	XLOC_030017	6	110,003,021	110,003,296	-	-	-	-
GRMZM2G135970	6	110,052,982	110,055,216	1	1	0	0	XLOC_030018	6	110,053,721	110,055,098	1	1	0	0
GRMZM2G118657	6	112,149,529	112,151,755	25	9	10	6	XLOC_030041	6	112,149,472	112,151,348	29	11	12	8
AC202439.3_FG001	6	113,900,332	113,901,471	9	5	3	3	XLOC_030054	6	113,900,300	113,901,293	11	6	4	3
GRMZM2G324375	6	116,206,148	116,207,201	28	9	8	7	XLOC_030074	6	116,206,088	116,206,943	31	11	10	8
GRMZM2G157113	6	116,979,182	116,981,588	15	6	6	4	XLOC_028270	6	116,980,830	116,981,749	11	4	3	2
XLOC_028309	6	121,567,042	121,568,491	25	3	2	3	XLOC_030139	6	121,567,118	121,567,490	20	3	2	3
GRMZM2G176910	6	124,546,088	124,548,966	10	3	1	4	XLOC_028345	6	124,547,569	124,547,840	1	0	0	1
GRMZM2G135108	6	125,194,477	125,196,129	5	0	1	0	XLOC_030169	6	125,194,489	125,195,880	5	0	1	0
GRMZM2G357296	6	125,424,088	125,424,971	5	1	0	0	XLOC_028357	6	125,424,395	125,424,938	5	1	0	0
GRMZM2G040702	6	126,329,584	126,334,694	1	1	1	0	GRMZM2G341729	6	126,329,458	126,332,466	2	2	0	2
GRMZM2G167387	6	127,870,096	127,871,796	15	5	4	4	XLOC_030202	6	127,870,521	127,871,444	9	3	2	2
GRMZM2G108714	6	128,235,624	128,237,977	37	18	16	20	XLOC_030209	6	128,236,074	128,237,326	34	17	15	19
GRMZM2G352618	6	128,637,993	128,638,947	17	8	8	4	XLOC_030216	6	128,638,235	128,638,848	17	8	8	4
GRMZM2G447795	6	129,268,752	129,270,611	11	4	3	4	XLOC_030226	6	129,268,701	129,270,059	11	4	3	4
GRMZM2G032896	6	132,244,350	132,248,959	25	5	6	2	XLOC_028414	6	132,247,916	132,248,974	9	2	3	2
GRMZM2G118951	6	132,410,000	132,412,045	15	3	3	1	XLOC_030279	6	132,410,072	132,411,648	15	3	3	1
GRMZM2G339091	6	134,280,739	134,285,176	45	21	20	9	XLOC_028447	6	134,284,618	134,285,372	-	-	-	-
GRMZM2G147245	6	135,723,001	135,725,403	37	12	14	9	XLOC_030307	6	135,724,233	135,725,239	5	2	2	1
GRMZM2G447480	6	138,474,315	138,476,165	13	4	4	4	XLOC_028494	6	138,474,734	138,476,438	13	4	4	4
GRMZM2G340444	6	138,775,095	138,776,992	18	6	5	6	XLOC_030347	6	138,774,979	138,776,272	20	6	5	6
GRMZM2G122479	6	139,464,334	139,470,019	13	8	7	1	XLOC_030357	6	139,464,546	139,466,534	13	8	7	1
GRMZM2G423863	6	139,641,367	139,643,749	7	1	2	1	GRMZM2G423898	6	139,642,333	139,643,913	-	-	-	-
GRMZM2G068323	6	140,114,567	140,120,483	9	2	1	1	GRMZM2G068436	6	140,106,997	140,115,632	11	2	0	1
GRMZM2G093325	6	140,914,068	140,915,871	13	3	3	6	XLOC_028518	6	140,914,832	140,915,320	2	2	2	2
GRMZM2G039113	6	142,758,866	142,760,748	42	12	14	3	XLOC_028541	6	142,758,863	142,760,970	42	14	16	4
GRMZM2G132478	6	142,833,016	142,835,191	11	6	7	8	XLOC_030415	6	142,832,661	142,834,858	11	6	7	8
GRMZM2G132461	6	142,836,658	142,839,545	30	14	12	13	XLOC_028545	6	142,838,170	142,839,820	29	14	12	12
GRMZM5G869788	6	144,147,804	144,153,012	61	22	24	17	XLOC_028566	6	144,148,114	144,148,914	15	4	5	4
GRMZM2G479608	6	145,511,301	145,516,044	31	14	14	12	XLOC_028587	6	145,512,115	145,515,915	16	2	2	2
GRMZM2G173630	6	146,310,907	146,313,590	17	3	3	4	XLOC_030460	6	146,311,139	146,313,337	17	3	3	4
GRMZM5G827496	6	146,885,515	146,888,876	20	10	7	9	XLOC_028605	6	146,886,717	146,888,479	17	9	6	8
GRMZM2G070199	6	147,917,374	147,922,817	77	30	21	32	XLOC_030488	6	147,921,865	147,922,850	1	0	0	0
GRMZM2G180328	6	148,062,224	148,064,507	14	9	7	10	XLOC_030490	6	148,063,742	148,064,148	2	0	1	1
XLOC_030495	6	148,154,920	148,162,601	44	11	13	11	XLOC_028627	6	148,160,727	148,161,594	-	-	-	-
GRMZM2G111955	6	148,628,022	148,632,519	19	7	6	3	XLOC_028633	6	148,627,957	148,629,694	5	4	3	2
GRMZM2G111955	6	148,628,022	148,632,519	19	7	6	3	XLOC_028632	6	148,627,686	148,629,694	5	4	3	2
GRMZM2G073310	6	149,280,665	149,282,838	25	12	10	12	XLOC_030512	6	149,279,487	149,282,523	25	12	10	12
GRMZM2G061906	6	151,319,337	151,321,751	9	3	4	3	XLOC_030548	6	151,319,368	151,321,310	9	3	4	3
GRMZM2G431350	6	151,980,700	151,983,784	5	3	1	2	GRMZM2G130239	6	151,977,873	151,981,648	10	1	3	1
GRMZM2G127297	6	153,503,421	153,508,370	2	1	1	0	GRMZM2G127350	6	153,507,939	153,512,599	2	0	0	0
GRMZM2G125034	6	153,977,792	153,980,864	48	14	20	13	XLOC_030599	6	153,980,084	153,981,320	18	3	3	4
GRMZM2G125034	6	153,977,792	153,980,864	48	14	20	13	XLOC_030598	6	153,978,666	153,979,619	14	5	7	6
GRMZM2G390436	6	154,430,572	154,432,792	45	30	19	27	XLOC_028736	6	154,431,415	154,432,970	18	12	12	12
XLOC_028750	6	155,074,658	155,076,625	37	5	20	20	XLOC_030619	6	155,074,555	155,076,308	37	5	20	20

GRMZM2G429714	6	155,208,311	155,214,291	69	24	26	32	XLOC_028755	6	155,208,385	155,211,113	26	11	11	13
GRMZM2G116258	6	155,783,970	155,788,502	10	4	4	4	XLOC_028766	6	155,787,657	155,788,601	-	-	-	-
GRMZM2G440968	6	155,861,586	155,862,536	5	2	2	3	GRMZM2G440949	6	155,855,927	155,862,458	15	3	3	5
GRMZM2G090300	6	155,951,341	155,953,332	3	1	2	1	XLOC_028777	6	155,952,708	155,953,265	-	-	-	-
GRMZM2G305159	6	156,405,012	156,413,226	27	4	6	6	XLOC_030654	6	156,404,946	156,405,356	3	1	1	1
GRMZM2G078781	6	157,072,612	157,077,219	104	40	48	39	XLOC_030671	6	157,074,323	157,077,111	34	11	11	11
GRMZM2G375904	6	158,380,660	158,388,837	15	5	4	5	XLOC_030694	6	158,380,664	158,381,595	1	0	1	1
GRMZM2G074792	6	158,417,573	158,422,014	25	7	11	10	XLOC_030695	6	158,421,162	158,421,506	14	3	6	6
GRMZM2G043695	6	160,053,924	160,055,102	35	16	17	16	XLOC_030722	6	160,053,752	160,055,101	27	14	15	14
AC206788.3_FG015	6	160,084,248	160,087,779	50	14	19	21	GRMZM2G058314	6	160,084,716	160,087,818	14	7	6	7
GRMZM2G004349	6	160,191,073	160,193,306	20	12	14	13	XLOC_030725	6	160,191,162	160,191,402	4	3	3	3
GRMZM2G004932	6	160,229,718	160,235,410	19	10	7	10	XLOC_028869	6	160,233,892	160,235,240	-	-	-	-
GRMZM2G132212	6	160,429,758	160,434,506	30	9	8	8	GRMZM2G432801	6	160,433,610	160,439,979	35	16	15	18
GRMZM2G158972	6	161,413,828	161,420,521	6	3	3	3	XLOC_028899	6	161,419,537	161,420,979	5	2	2	2
GRMZM2G090245	6	161,647,137	161,648,173	9	4	4	4	XLOC_028905	6	161,647,173	161,648,309	9	4	4	4
GRMZM2G021406	6	162,029,195	162,031,955	30	10	10	10	XLOC_030774	6	162,029,919	162,031,685	10	5	4	6
GRMZM2G179797	6	162,746,428	162,748,871	6	0	2	2	XLOC_030794	6	162,746,423	162,747,799	6	0	2	2
GRMZM2G037998	6	163,081,648	163,083,193	18	7	9	5	XLOC_030801	6	163,081,613	163,083,105	18	7	9	5
GRMZM2G038284	6	164,572,072	164,575,526	45	8	10	7	GRMZM2G038183	6	164,567,128	164,575,478	33	9	11	11
GRMZM2G038284	6	164,572,072	164,575,526	45	8	10	7	XLOC_028982	6	164,574,502	164,575,478	6	0	0	0
XLOC_028986	6	164,757,065	164,759,753	25	11	8	9	XLOC_030842	6	164,757,491	164,758,850	25	11	8	9
GRMZM2G021549	6	164,760,362	164,767,127	25	11	7	6	XLOC_028987	6	164,760,526	164,762,818	17	6	3	2
GRMZM2G021470	6	164,766,609	164,768,517	-	-	-	-	GRMZM2G021549	6	164,760,362	164,767,127	25	11	7	6
GRMZM2G048892	6	165,444,350	165,445,593	13	3	3	3	XLOC_030858	6	165,443,968	165,445,433	13	3	3	3
GRMZM2G062738	6	166,141,755	166,146,367	30	11	9	12	XLOC_029025	6	166,141,205	166,146,953	54	22	20	25
GRMZM2G163809	6	167,024,418	167,028,053	46	15	15	11	XLOC_029045	6	167,024,418	167,025,382	15	4	3	5
GRMZM2G159028	6	167,082,887	167,085,534	11	1	1	1	XLOC_030895	6	167,084,398	167,084,671	2	0	0	0
GRMZM2G070659	6	167,774,318	167,777,115	16	8	8	5	XLOC_030910	6	167,776,365	167,776,826	-	-	-	-
GRMZM2G156905	6	168,167,423	168,170,200	34	3	8	7	XLOC_029066	6	168,168,646	168,169,311	4	0	0	0
GRMZM2G141818	6	168,642,899	168,650,480	56	18	15	14	GRMZM2G141925	6	168,642,324	168,643,842	10	3	6	4
GRMZM2G095025	6	168,897,062	168,898,823	21	6	5	6	XLOC_029073	6	168,897,219	168,898,900	24	6	5	6
GRMZM5G833747	6	169,124,326	169,127,492	1	0	0	0	GRMZM2G127426	6	169,125,962	169,128,127	16	2	3	3
GRMZM5G894233	7	272,380	276,643	12	2	3	2	GRMZM2G424147	7	272,954	273,522	4	1	1	0
GRMZM2G020281	7	955,586	961,591	18	5	6	8	XLOC_032540	7	955,649	956,597	6	2	2	3
GRMZM2G135739	7	1,233,056	1,236,779	16	11	9	9	GRMZM2G135808	7	1,225,533	1,233,199	9	6	5	4
GRMZM2G420743	7	1,270,762	1,272,128	27	14	13	9	GRMZM2G420758	7	1,270,943	1,272,637	9	8	6	4
GRMZM2G555108	7	1,304,879	1,310,538	20	5	7	4	GRMZM2G120563	7	1,309,967	1,311,875	20	2	2	3
AC205122.4_FG003	7	2,137,542	2,139,233	8	5	2	6	XLOC_032566	7	2,137,597	2,138,901	24	1	3	4
GRMZM2G458082	7	2,296,054	2,297,237	7	4	4	5	XLOC_030965	7	2,296,558	2,297,438	-	-	-	-
GRMZM2G456997	7	3,252,351	3,253,259	6	0	0	1	XLOC_032592	7	3,252,792	3,253,074	5	0	0	1
AC205320.3_FG004	7	6,073,356	6,075,719	10	6	5	5	GRMZM2G026614	7	6,073,362	6,074,529	18	3	6	2
GRMZM2G406014	7	6,922,475	6,924,247	2	0	0	1	XLOC_032635	7	6,922,617	6,923,867	2	0	0	1
GRMZM2G132568	7	9,547,566	9,551,755	41	24	24	24	XLOC_031066	7	9,551,283	9,551,687	5	2	3	3
GRMZM2G030138	7	10,578,360	10,580,594	47	12	12	17	GRMZM2G328171	7	10,578,984	10,581,443	14	3	2	8
XLOC_031079	7	10,656,525	10,658,447	27	10	6	6	XLOC_032682	7	10,656,435	10,657,883	27	10	6	6
GRMZM2G162382	7	10,699,699	10,703,137	37	14	17	18	XLOC_031080	7	10,699,818	10,700,185	-	-	-	-
GRMZM2G401308	7	11,446,449	11,447,690	33	13	12	11	XLOC_031090	7	11,446,548	11,447,642	32	13	12	11
GRMZM2G458494	7	13,623,866	13,626,771	25	9	15	14	XLOC_031116	7	13,624,455	13,625,156	21	9	14	14
GRMZM2G028129	7	15,013,266	15,015,247	30	12	5	6	XLOC_031133	7	15,013,395	15,014,207	9	4	2	3
GRMZM2G424832	7	18,620,430	18,626,288	11	3	5	3	XLOC_031162	7	18,621,004	18,621,402	-	-	-	-
GRMZM2G340656	7	19,063,721	19,067,235	36	10	9	10	XLOC_032747	7	19,063,747	19,066,238	23	5	3	5
GRMZM5G892627	7	23,364,784	23,366,705	35	14	18	18	XLOC_031193	7	23,365,115	23,366,572	35	14	18	18
GRMZM2G000221	7	31,098,634	31,099,387	22	5	7	5	XLOC_032835	7	31,098,451	31,099,106	23	10	11	9
GRMZM2G104231	7	31,781,244	31,783,659	39	14	10	15	XLOC_032836	7	31,780,328	31,782,023	21	7	6	11
GRMZM2G140394	7	38,403,781	38,408,314	13	0	5	1	XLOC_031289	7	38,407,722	38,408,007	-	-	-	-
GRMZM2G014914	7	41,435,269	41,438,511	13	4	4	1	XLOC_031307	7	41,437,732	41,438,446	4	2	2	1
GRMZM5G893913	7	46,202,870	46,207,496	18	6	3	5	XLOC_032898	7	46,205,325	46,207,465	9	2	0	1
GRMZM2G061562	7	50,166,355	50,172,435	36	26	23	20	XLOC_032918	7	50,170,951	50,172,266	3	0	0	1
GRMZM2G050550	7	51,486,971	51,489,900	17	8	7	9	XLOC_032924	7	51,486,818	51,488,870	13	7	6	7
GRMZM2G048257	7	51,667,538	51,669,099	11	2	1	3	XLOC_031361	7	51,667,897	51,668,499	8	1	1	2
GRMZM2G318299	7	54,345,908	54,347,701	13	2	5	3	GRMZM2G318292	7	54,345,911	54,348,241	15	2	5	3
GRMZM2G117878	7	85,288,907	85,290,654	8	3	4	6	XLOC_033047	7	85,288,917	85,290,497	8	3	4	6
GRMZM2G047592	7	89,458,280	89,461,662	8	1	2	2	XLOC_031479	7	89,456,998	89,459,759	4	0	0	1
GRMZM2G162396	7	99,233,022	99,234,662	16	8	8	8	XLOC_033120	7	99,233,077	99,234,124	16	8	8	8
GRMZM2G032160	7	104,201,951	104,204,914	32	9	7	14	XLOC_031565	7	104,202,160	104,204,790	32	9	7	14
GRMZM2G019183	7	107,954,326	107,958,837	73	32	28	30	XLOC_033183	7	107,955,577	107,958,837	37	20	17	19
XLOC_033185	7	108,330,462	108,332,092	24	9	5	5	XLOC_031588	7	108,330,559	108,332,566	24	9	5	5
XLOC_033186	7	108,414,550	108,416,494	19	4	6	6	XLOC_031590	7	108,414,615	108,416,397	19	4	6	6
GRMZM2G126507	7	108,871,160	108,874,537	85	27	32	28	XLOC_031594	7	108,873,979	108,874,199	4	1	0	0
GRMZM2G092525	7	109,353,827	109,357,542	33	16	18	19	XLOC_031596	7	109,356,210	109,357,565	35	16	18	19
GRMZM2G121200	7	110,085,353	110,088,614	8	0	2	1	GRMZM2G121189	7	110,084,378	110,085,727	-	-	-	-
GRMZM2G159221	7	111,685,454	111,688,840	17	5	5	5	GRMZM2G159160	7	111,688,572	111,690,233	2	1	1	1
GRMZM2G129018	7	115,719,658	115,722,055	6	2	0	0	XLOC_031629	7	115,719,873	115,722,227	6	2	0	0
XLOC_033227	7	115,996,532	115,997,272	10	4	5	2	GRMZM2G125853	7	115,996,767	116,010,834	19	6	8	3
GRMZM2G142918	7	117,727,876	117,730,526	43	17	17	16	XLOC_033238	7	117,728,776	117,730,447	21	6	7	7
GRMZM2G135763	7	121,285,089	121,289,636	6	3	3	3	XLOC_033268	7	121,285,598	121,287,999	6	3	3	3
XLOC_031687	7	121,804,250	121,804,694	-	-	-	-	XLOC_033273	7	121,804,250	121,804,951	2	1	1	1
GRMZM2G004207	7	121,850,282	121,852,444	6	0	3	4	XLOC_033274	7	121,850,262	121,851,370	2	0	1	1
GRMZM2G024865	7	124,779,178	124,781,786	29	14	10	7	XLOC_031723	7	124,780,337	124,781,632	16	11	6	6
GRMZM2G416632	7	128,406,301	128,408,544	23	11	8	11	GRMZM2G416625	7	128,407,526	128,412,102	33	9	10	12
GRMZM2G082653	7	128,679,185	128,680,735	50	20	12	11	XLOC_031757	7	128,679,213	128,681,250	50	20	12	11
GRMZM2G096952	7	129,753,381	129,758,428	31	12	13	11	XLOC_033326	7	129,753,354	129,754,232	19	7	10	7
GRMZM2G055469	7	132,423,389	132,426,153	41	14	17	17	XLOC_033368	7	132,421,650	132,424,591	25	13	12	11
GRMZM2G176430	7	132,548,737	132,551,662	45	9	7	5	XLOC_033369	7	132,548,900	132,549,402	1	1	0	0
GRMZM2G083932	7	132,660,472	132,661,613	8	2	2	2	XLOC_033371	7	132,660,271	132,661,577	8	2	2	2
GRMZM2G083894	7	132,664,469	132,666,026	14	7	7	7	XLOC_033373	7	132,664,910	132,665,906	4	3	2	3
GRMZM2G085467	7	132,922,501	132,925,523	27	2	1	3	XLOC_033379	7	132,922,387	132,924,053	5	1	0	1
GRMZM2G168364	7	134,168,481	134,170,279	5	2	2	3	XLOC_033407	7	134,168,847	134,169,418	1	0	0	0
GRMZM2G131281	7	135,625,837	135,626,999	22	7	8	7	XLOC_031833	7	135,625,966	135,627,055	22	7	8	7
GRMZM2G081048	7	139,144,164	139,155,145	18	4	0	3	GRMZM2G080871	7	139,152,738	139,156,303	9	5	3	4
GRMZM2G132486	7	140,268,676	140,271,906	46	13	12	6	XLOC_031883	7	140,268,784	140,269,784	15	4	3	3
GRMZM2G071147	7	140,455,266	140,461,976	52	12	10	7	XLOC_033463	7	140,460,238	140,461,566	23	4	5	4
GRMZM2G149520	7	140,847,928	140,853,032	44	7	11	9	XLOC_031896	7	140,848,023	140,848,380	11	2	2	2
GRMZM2G017013	7	141,612,374	141,615,097	32	11	12	15	XLOC_031903	7	141,612,979	141,613,942	3	2	0	2
GRMZM2G017013	7	141,612,374	141,615,097	32	11	12	15	XLOC_031904	7	141,614,458	141,614,818	3	3	3	2
GRMZM2G001639	7	141,630,856	141,633,508	18	7	5	5	XLOC_033478	7	141,631,174	141,632,009	17	7	4	4
GRMZM2G073228	7	141,674,424	141,678,723	7	3	2	3	XLOC_031906	7	141,675,214	141,678,290	7	3	2	3
GRMZM2G073504	7	141,681,145	141,690,742	47	6	7	7	GRMZM2G073377	7	141,680,690	141,681,890	14	3	3	3
GRMZM2G052610	7	141,917,927	141,921,808	7	0	2	1	GRMZM2G052509	7	141,921,207	141,924,166	7	0	2	2
GRMZM2G165272	7	142,270,553	142,272,208	13	2	1	2	GRMZM2G465685	7	142,270,740	142,271,931	13	2	1	2
GRMZM2G104551	7	143,239,536	143,250,307	72	4	13	10	XLOC_031931	7	143,244,178	143,246,692	46	2	8	7
XLOC_031935	7	143,286,301	143,288,804	9	3	1	2	GRMZM2G335111	7	143,288,306	143,289,828	8	3	0	1
GRMZM2G091586	7	143,551,214	143,557,231	5	2	3	3	XLOC_033518	7	143,551,214	143,551,708	-	-	-	-
GRMZM2G006117	7	145,808,406	145,812,881	3	0	3	2	XLOC_031968	7	145,809,169	145,809,829	-	-	-	-
GRMZM2G075719	7	150,455,168	150,458,991	7	0	1	0	XLOC_033617	7	150,455,264	150,455,567	-	-	-	-
GRMZM2G095261	7	151,123,095	151,125,335	5	2	1	2	XLOC_033631	7	151,122,730	151,124,888	5	2	1	2
GRMZM2G155911	7	151,226,660	151,228,687	15	2	3	4	XLOC_033635	7	151,226,861	151,228,001	15	2	3	4
GRMZM2G010298	7	151,545,591	151,555,845	2	1	1	0	XLOC_032048	7	151,554,826	151,555,375	-	-	-	-
GRMZM2G325783	7	151,560,059	151,564,762	19	4	6	3	XLOC_032049	7	151,560,171	151,562,048	9	3	3	2
GRMZM2G325008	7	152,431,465	152,438,328	67	32	27	25	XLOC_033649	7	152,431,276	152,433,930	26	9	10	4
GRMZM2G108849	7	152,808,921	152,813,134	27	9	7	9	XLOC_033651	7	152,809,230	152,810,705	20	5	3	5
GRMZM2G429396	7	153,330,905	153,331,987	12	2	2	2	XLOC_033662	7	153,330,572	153,331,553	12	2	2	2
GRMZM2G167591	7	153,692,958	153,695,938	38	17	16	15	XLOC_033672	7	153,695,447	153,695,725	2	1	0	0

GRMZM2G357631	7	154,224,100	154,225,746	42	10	9	11	GRMZM2G357636	7	154,221,509	154,226,616	5	1	3	4
GRMZM2G058310	7	155,396,590	155,399,705	5	1	1	1	GRMZM2G358467	7	155,396,211	155,401,842	8	4	4	4
GRMZM5G889999	7	156,219,834	156,225,721	13	2	3	3	XLOC_032125	7	156,224,860	156,225,621	8	2	2	2
GRMZM2G060993	7	157,049,353	157,051,304	6	0	0	2	XLOC_032140	7	157,049,914	157,050,398	1	0	0	1
GRMZM5G801949	7	157,477,093	157,479,679	17	8	10	8	XLOC_032147	7	157,477,186	157,478,848	6	3	2	1
XLOC_032159	7	158,405,841	158,406,153	9	3	4	3	GRMZM5G897592	7	158,405,601	158,408,516	52	16	17	11
GRMZM2G106548	7	158,406,545	158,409,322	41	9	10	6	GRMZM5G897592	7	158,405,601	158,408,516	52	16	17	11
XLOC_033767	7	159,268,927	159,276,192	70	20	19	18	XLOC_032175	7	159,270,024	159,272,786	36	11	11	9
GRMZM5G889052	7	160,189,515	160,193,189	45	14	15	7	XLOC_033789	7	160,191,526	160,192,363	13	5	7	7
GRMZM5G889052	7	160,189,515	160,193,189	45	14	15	7	XLOC_033788	7	160,190,641	160,190,959	5	1	1	0
XLOC_033790	7	160,254,346	160,255,890	6	2	3	2	XLOC_032196	7	160,254,909	160,255,804	6	2	3	2
GRMZM2G302604	7	160,854,635	160,856,784	13	5	7	8	XLOC_033801	7	160,854,309	160,856,519	13	5	7	8
GRMZM2G023444	7	161,376,313	161,379,215	31	13	11	8	XLOC_033806	7	161,376,439	161,376,899	1	0	0	0
GRMZM2G023346	7	161,386,057	161,389,217	91	22	25	14	XLOC_033807	7	161,386,211	161,388,685	76	20	22	12
GRMZM2G172657	7	161,941,447	161,944,260	35	11	13	11	XLOC_033820	7	161,941,387	161,943,936	35	11	13	11
GRMZM2G057247	7	162,108,103	162,114,970	57	10	15	18	XLOC_033828	7	162,108,890	162,109,828	29	5	9	10
GRMZM2G057247	7	162,108,103	162,114,970	57	10	15	18	XLOC_033829	7	162,113,500	162,114,498	16	4	5	6
GRMZM2G089736	7	165,496,269	165,498,636	11	4	7	5	XLOC_033883	7	165,496,321	165,497,031	11	4	7	5
GRMZM2G079397	7	166,191,360	166,197,002	5	1	2	2	GRMZM2G079226	7	166,186,897	166,191,764	1	0	0	0
GRMZM2G056732	7	166,410,307	166,416,117	14	3	3	1	XLOC_033901	7	166,410,567	166,414,219	3	1	0	0
GRMZM2G017145	7	167,541,463	167,544,286	40	16	9	17	AC195864.3_FG017	7	167,539,708	167,541,966	20	8	4	9
GRMZM2G141322	7	167,674,297	167,675,623	37	15	18	13	XLOC_033929	7	167,674,835	167,675,315	8	4	4	3
GRMZM2G150683	7	167,947,896	167,955,945	38	6	5	7	GRMZM2G150758	7	167,946,724	167,948,218	28	11	18	14
GRMZM2G318843	7	168,019,762	168,022,899	43	17	14	13	XLOC_033938	7	168,021,383	168,022,711	28	10	9	7
GRMZM2G477325	7	168,441,397	168,442,596	51	21	28	26	XLOC_033944	7	168,441,795	168,442,543	50	21	27	26
GRMZM2G301934	7	169,977,821	169,979,383	32	10	9	10	XLOC_032383	7	169,977,981	169,978,650	10	3	4	3
GRMZM2G356894	7	170,074,356	170,080,041	26	8	7	5	GRMZM2G055607	7	170,071,170	170,074,488	33	12	9	10
GRMZM2G433767	7	170,696,934	170,699,642	28	9	6	6	XLOC_032403	7	170,697,273	170,697,504	6	1	0	1
GRMZM2G037189	7	171,577,707	171,578,957	18	4	3	4	XLOC_032421	7	171,577,972	171,578,887	12	3	2	3
GRMZM2G025885	7	171,769,812	171,771,300	11	3	3	2	XLOC_034006	7	171,769,839	171,771,033	11	3	3	2
GRMZM2G025992	7	171,775,066	171,778,176	6	3	2	1	XLOC_032429	7	171,776,631	171,778,079	6	3	2	1
GRMZM2G414423	7	171,863,190	171,865,343	36	13	13	11	XLOC_034009	7	171,863,280	171,865,189	36	13	13	11
GRMZM2G020766	7	172,301,215	172,312,681	4	3	3	1	XLOC_032437	7	172,301,623	172,302,947	1	0	0	0
GRMZM2G414460	7	172,543,068	172,547,171	77	25	21	30	XLOC_032447	7	172,543,187	172,544,780	28	10	4	10
GRMZM2G025441	7	172,793,831	172,796,061	12	6	6	5	XLOC_034039	7	172,794,845	172,795,897	9	3	3	2
GRMZM2G427815	7	172,809,428	172,811,102	45	19	18	13	XLOC_034040	7	172,810,282	172,810,699	-	-	-	-
GRMZM2G166738	7	172,977,273	172,981,385	30	14	13	11	XLOC_032461	7	172,980,469	172,981,129	2	1	1	1
GRMZM2G311665	7	173,486,169	173,490,497	147	55	55	50	XLOC_032475	7	173,490,053	173,490,328	9	2	2	2
GRMZM2G430849	7	173,556,079	173,558,804	34	13	10	9	XLOC_032477	7	173,556,354	173,557,829	24	11	8	7
GRMZM2G469380	7	173,595,180	173,596,955	8	5	4	5	XLOC_032478	7	173,596,426	173,596,757	-	-	-	-
GRMZM2G056772	7	174,614,466	174,615,537	7	4	6	6	XLOC_034093	7	174,614,465	174,615,416	7	4	6	6
GRMZM2G056569	7	174,627,232	174,631,734	19	4	10	12	XLOC_032498	7	174,630,036	174,630,579	13	3	3	6
GRMZM2G134284	7	174,834,523	174,847,147	16	5	5	7	GRMZM2G134295	7	174,832,020	174,835,057	15	5	6	6
GRMZM2G032711	7	175,605,863	175,613,266	48	17	19	13	GRMZM2G032376	7	175,611,974	175,615,934	11	2	2	3
GRMZM2G000818	7	176,182,074	176,183,077	47	15	15	7	XLOC_034115	7	176,182,240	176,183,508	31	6	6	4
XLOC_032527	7	176,436,743	176,439,281	3	0	1	0	XLOC_034118	7	176,436,043	176,438,830	14	1	2	1
GRMZM2G085111	8	400,460	407,184	52	14	18	15	XLOC_035987	8	401,337	404,870	18	3	5	4
GRMZM2G386987	8	704,959	705,716	2	0	0	0	XLOC_035994	8	705,086	705,419	7	4	4	3
GRMZM2G063223	8	1,145,660	1,147,184	2	1	1	1	XLOC_034134	8	1,146,664	1,147,130	-	-	-	-
GRMZM2G589579	8	2,511,344	2,518,808	32	17	16	17	XLOC_036018	8	2,511,400	2,512,210	6	4	3	4
GRMZM2G019515	8	2,901,206	2,904,218	20	9	9	9	XLOC_034151	8	2,903,301	2,903,831	4	3	2	2
GRMZM2G050325	8	3,921,958	3,925,143	8	1	1	1	XLOC_036031	8	3,921,958	3,923,002	3	1	1	1
GRMZM2G058404	8	6,359,079	6,360,548	14	4	5	4	XLOC_034207	8	6,359,315	6,360,734	14	4	5	4
GRMZM2G110504	8	6,920,014	6,921,235	21	4	5	6	XLOC_036063	8	6,920,162	6,920,992	21	4	5	6
AC216871.3_FG004	8	10,013,056	10,014,230	20	6	7	7	XLOC_034265	8	10,013,125	10,013,839	10	1	1	2
GRMZM2G096358	8	11,486,313	11,488,153	45	12	15	13	XLOC_034286	8	11,486,497	11,488,203	45	12	15	13
GRMZM2G095964	8	11,922,956	11,924,511	32	11	11	11	XLOC_036123	8	11,923,225	11,923,580	16	9	9	9
GRMZM2G119361	8	13,371,529	13,374,745	60	25	29	24	XLOC_034316	8	13,374,182	13,374,574	13	7	7	4
GRMZM2G079458	8	13,801,218	13,804,482	21	9	10	11	XLOC_034324	8	13,802,079	13,802,995	12	4	5	5
GRMZM2G110063	8	14,730,093	14,780,789	32	18	18	16	XLOC_036160	8	14,749,038	14,750,512	-	-	-	-
GRMZM2G139600	8	14,796,888	14,799,245	28	16	12	12	XLOC_036161	8	14,797,419	14,799,093	16	8	4	6
AC217965.2_FG012	8	15,108,533	15,109,664	2	1	1	1	XLOC_034340	8	15,108,641	15,109,157	-	-	-	-
GRMZM2G083328	8	15,987,340	15,989,474	17	8	7	7	XLOC_034347	8	15,987,336	15,988,847	8	3	5	3
GRMZM2G081848	8	16,431,439	16,433,808	13	7	5	6	XLOC_034349	8	16,433,026	16,433,339	5	4	4	4
GRMZM2G095595	8	17,177,806	17,182,719	18	8	9	9	XLOC_036196	8	17,178,910	17,179,303	-	-	-	-
GRMZM2G375159	8	18,444,281	18,446,241	24	7	9	7	XLOC_034371	8	18,444,771	18,445,411	3	2	2	1
GRMZM2G032190	8	18,612,350	18,616,112	27	2	11	4	XLOC_036220	8	18,612,463	18,613,061	-	-	-	-
GRMZM2G080375	8	20,703,355	20,706,994	4	0	3	0	GRMZM2G080588	8	20,696,323	20,703,544	33	20	18	20
GRMZM2G004182	8	22,175,678	22,180,874	59	11	18	13	GRMZM2G004083	8	22,180,368	22,182,467	12	5	8	6
GRMZM2G472625	8	23,427,704	23,432,837	54	28	33	32	GRMZM2G472654	8	23,427,241	23,428,339	12	4	3	3
GRMZM2G099483	8	23,892,754	23,895,063	37	20	22	19	XLOC_036278	8	23,892,459	23,894,829	35	20	21	19
GRMZM2G047139	8	24,724,776	24,728,219	25	13	16	15	XLOC_036289	8	24,724,760	24,726,896	25	13	16	15
GRMZM2G369182	8	25,881,226	25,882,741	18	5	4	8	XLOC_036300	8	25,880,908	25,881,985	12	3	3	5
GRMZM2G127687	8	26,863,089	26,867,549	61	19	23	25	XLOC_034457	8	26,865,699	26,866,324	3	2	2	2
GRMZM2G010596	8	27,218,394	27,221,078	21	11	9	8	XLOC_034461	8	27,218,363	27,218,894	6	3	3	2
GRMZM2G014580	8	27,670,562	27,672,733	6	4	1	0	XLOC_036317	8	27,670,252	27,672,389	6	4	1	0
GRMZM5G842965	8	36,144,983	36,147,538	21	6	6	6	XLOC_034509	8	36,145,184	36,147,155	21	6	6	6
GRMZM2G061088	8	37,813,042	37,816,875	46	16	18	15	XLOC_034519	8	37,813,413	37,813,825	11	4	4	4
GRMZM2G035395	8	43,074,144	43,078,508	1	1	1	1	GRMZM2G035542	8	43,077,873	43,086,649	14	2	4	3
AC205274.3_FG001	8	51,816,339	51,817,463	11	7	7	7	XLOC_034562	8	51,816,725	51,817,436	11	7	7	7
XLOC_034566	8	52,818,656	52,820,059	9	2	2	0	XLOC_036426	8	52,817,772	52,819,178	18	5	4	3
GRMZM2G159592	8	64,222,114	64,223,758	23	7	9	9	XLOC_036481	8	64,222,075	64,223,671	23	7	9	9
GRMZM2G345039	8	67,245,670	67,250,897	4	2	2	3	GRMZM2G043764	8	67,239,332	67,245,829	15	7	8	8
GRMZM2G001514	8	69,107,919	69,110,163	15	3	8	4	XLOC_036518	8	69,108,973	69,109,970	5	1	4	2
XLOC_034669	8	69,273,412	69,276,762	4	2	3	3	XLOC_036521	8	69,273,990	69,274,639	1	0	1	1
GRMZM2G031827	8	69,651,928	69,660,583	10	5	5	2	XLOC_036522	8	69,659,485	69,660,454	-	-	-	-
GRMZM2G023872	8	72,494,681	72,496,570	1	1	1	1	XLOC_036544	8	72,494,703	72,496,238	1	1	1	1
GRMZM2G001991	8	73,364,861	73,370,694	21	7	9	10	GRMZM2G002034	8	73,369,546	73,372,613	24	9	11	10
GRMZM2G097768	8	73,418,363	73,424,793	27	5	7	4	XLOC_034703	8	73,423,869	73,424,293	-	-	-	-
GRMZM2G058305	8	75,118,942	75,123,679	3	2	2	1	GRMZM2G058456	8	75,116,592	75,119,705	36	20	8	7
GRMZM2G128995	8	77,264,067	77,274,209	12	2	2	2	XLOC_036576	8	77,271,479	77,272,668	12	5	6	5
GRMZM5G892645	8	78,161,466	78,166,379	15	2	4	3	XLOC_036584	8	78,163,924	78,165,052	1	0	0	0
GRMZM2G456000	8	78,524,069	78,528,819	16	2	5	3	XLOC_034744	8	78,522,738	78,524,404	9	0	2	1
GRMZM2G086964	8	78,942,781	78,951,473	13	2	1	0	XLOC_034750	8	78,951,149	78,951,442	-	-	-	-
GRMZM2G128682	8	86,105,887	86,107,965	11	1	4	1	XLOC_036620	8	86,106,557	86,107,464	4	1	2	0
GRMZM2G306851	8	86,549,083	86,555,756	53	18	16	13	GRMZM2G007404	8	86,555,339	86,559,750	11	3	3	5
GRMZM2G023755	8	93,946,368	93,949,244	14	2	2	2	XLOC_036675	8	93,948,001	93,950,694	24	8	4	7
GRMZM5G898867	8	94,085,437	94,089,730	38	10	17	10	XLOC_036676	8	94,086,238	94,086,551	7	2	2	2
GRMZM2G358606	8	94,309,687	94,310,894	20	11	9	12	XLOC_034847	8	94,310,000	94,311,063	20	11	9	12
GRMZM2G107721	8	97,451,652	97,453,287	8	1	0	1	XLOC_034876	8	97,452,226	97,452,612	1	0	0	0
GRMZM2G016605	8	106,153,653	106,156,495	18	5	6	4	XLOC_036784	8	106,153,026	106,155,085	8	0	2	1
GRMZM2G116238	8	109,359,888	109,361,490	12	6	6	6	XLOC_034971	8	109,361,001	109,361,451	-	-	-	-
GRMZM2G449681	8	109,739,873	109,742,256	59	24	29	29	XLOC_034976	8	109,740,144	109,742,205	59	24	29	29
GRMZM2G029756	8	113,237,540	113,242,322	23	5	5	3	XLOC_035009	8	113,240,802	113,241,236	8	2	2	2
XLOC_035059	8	118,087,730	118,088,907	7	1	2	1	XLOC_036878	8	118,087,800	118,088,310	-	-	-	-
XLOC_035077	8	118,795,173	118,797,211	8	2	4	3	GRMZM2G138817	8	118,795,818	118,797,252	13	4	5	3
GRMZM2G300589	8	119,169,594	119,172,752	10	1	7	0	XLOC_035078	8	119,170,288	119,170,988	-	-	-	-
GRMZM2G073347	8	119,461,575	119,462,915	11	2	1	2	XLOC_035081	8	119,461,775	119,462,949	16	4	3	4
GRMZM2G062555	8	119,542,597	119,544,810	5	4	3	4	XLOC_035083	8	119,544,238	119,544,607	-	-	-	-
GRMZM2G025459	8	119,745,262	119,748,192	13	5	7	7	XLOC_035084	8	119,745,323	119,747,929	13	5	7	7
XLOC_035097	8	121,416,903	121,418,458	6	4	4	1	XLOC_036937	8	121,416,852	121,418,183	6	4	4	1
XLOC_035098	8	121,416,903	121,425,056	25	12	16	13	XLOC_036937	8	121,416,852	121,418,183	6	4	4	1
GRMZM2G048200	8	125,000,004	125,004,076	9	3	4	4	XLOC_035147	8	125,003,196	125,004,056	4	0	1	1

GRMZM2G048200	8	125,000,004	125,004,076	9	3	4	4	GRMZM2G048117	8	124,996,332	125,001,496	-	-	-	-
GRMZM2G150367	8	126,229,305	126,232,019	2	1	0	0	GRMZM2G150434	8	126,231,541	126,232,916	1	1	0	0
XLOC_037004	8	128,078,312	128,078,825	-	-	-	-	GRMZM2G700655	8	128,075,569	128,081,851	2	1	1	1
XLOC_037037	8	131,058,700	131,060,803	25	7	7	5	XLOC_035212	8	131,052,957	131,059,699	39	11	12	11
AC214648.3_FG003	8	131,331,979	131,336,740	33	7	5	7	XLOC_035215	8	131,336,046	131,336,543	1	0	0	0
GRMZM2G700665	8	131,576,700	131,580,312	42	14	12	17	XLOC_037044	8	131,578,426	131,579,537	19	7	6	11
GRMZM2G474726	8	131,727,186	131,733,880	24	15	15	14	GRMZM2G173763	8	131,733,467	131,738,126	11	4	2	7
GRMZM2G053991	8	131,904,461	131,905,991	21	5	8	5	GRMZM2G353250	8	131,904,201	131,905,991	11	5	6	4
GRMZM2G080191	8	132,540,044	132,541,658	1	1	1	1	XLOC_035227	8	132,540,497	132,541,980	4	1	1	1
GRMZM2G028986	8	133,998,505	134,000,685	8	2	2	1	XLOC_035246	8	133,999,615	134,000,856	-	-	-	-
GRMZM2G470513	8	134,376,621	134,379,299	4	2	2	1	GRMZM2G168590	8	134,379,238	134,379,777	9	5	4	2
GRMZM2G162525	8	134,443,644	134,444,960	10	4	4	2	XLOC_037080	8	134,444,295	134,444,960	6	3	4	2
GRMZM2G456835	8	135,307,939	135,312,198	30	8	5	6	XLOC_037089	8	135,310,261	135,312,197	24	6	4	5
GRMZM2G088132	8	135,584,864	135,586,005	3	1	1	1	XLOC_035273	8	135,585,278	135,585,576	3	1	1	1
AC207342.3_FG008	8	135,947,268	135,949,742	29	10	12	13	XLOC_037094	8	135,947,857	135,949,508	14	5	8	8
GRMZM5G803433	8	142,537,765	142,543,929	1	1	1	0	GRMZM2G117742	8	142,535,900	142,538,931	1	0	0	0
GRMZM2G050270	8	142,813,533	142,817,608	3	1	2	1	XLOC_035355	8	142,811,980	142,813,848	11	2	2	2
GRMZM2G074735	8	144,697,840	144,699,628	5	1	2	1	XLOC_037179	8	144,697,907	144,698,440	2	1	1	1
GRMZM2G149683	8	144,765,266	144,766,964	26	7	6	8	XLOC_035377	8	144,765,497	144,765,969	4	2	2	2
GRMZM2G021436	8	145,218,849	145,221,114	35	18	18	18	XLOC_037185	8	145,219,676	145,220,486	-	-	-	-
GRMZM2G179693	8	145,719,688	145,726,829	13	2	3	2	GRMZM2G179696	8	145,718,291	145,722,336	2	0	0	0
GRMZM2G063672	8	146,150,403	146,153,728	37	15	13	16	XLOC_037201	8	146,151,084	146,151,525	-	-	-	-
GRMZM2G086088	8	147,738,191	147,741,508	16	4	3	3	XLOC_035431	8	147,740,511	147,740,974	-	-	-	-
GRMZM2G144028	8	151,441,785	151,450,139	154	53	63	68	GRMZM2G144021	8	151,438,263	151,442,577	21	7	9	8
GRMZM2G144028	8	151,441,785	151,450,139	154	53	63	68	XLOC_035491	8	151,442,987	151,444,263	9	2	3	3
GRMZM2G092018	8	151,669,118	151,680,781	44	12	18	13	GRMZM2G091973	8	151,667,315	151,669,713	78	37	33	29
GRMZM5G843389	8	151,798,812	151,803,317	11	1	2	2	XLOC_035495	8	151,802,508	151,802,839	-	-	-	-
XLOC_035503	8	152,285,732	152,293,012	50	19	21	18	XLOC_037286	8	152,285,587	152,287,295	25	7	6	6
GRMZM2G439350	8	152,497,093	152,498,604	16	6	6	5	XLOC_037288	8	152,497,944	152,498,597	6	2	1	2
XLOC_037303	8	153,934,934	153,937,499	18	2	3	4	XLOC_035526	8	153,934,973	153,936,891	-	-	-	-
GRMZM2G107985	8	154,553,618	154,562,574	15	4	6	1	XLOC_035530	8	154,562,135	154,562,609	-	-	-	-
GRMZM2G069203	8	155,020,506	155,021,940	21	12	12	5	XLOC_037324	8	155,020,627	155,021,908	11	7	6	3
GRMZM2G151252	8	155,184,005	155,186,489	4	0	0	0	GRMZM2G151245	8	155,182,657	155,184,839	19	5	7	5
GRMZM2G367701	8	156,268,171	156,269,856	17	2	5	6	XLOC_035566	8	156,268,512	156,269,644	17	2	5	6
GRMZM2G167758	8	156,423,920	156,425,734	16	3	4	3	XLOC_037363	8	156,425,476	156,425,725	1	0	0	0
GRMZM2G446960	8	156,777,102	156,780,465	-	-	-	-	GRMZM2G147709	8	156,774,170	156,777,892	16	4	7	6
GRMZM2G010065	8	159,534,577	159,539,292	12	1	1	2	GRMZM2G010176	8	159,531,387	159,535,110	42	8	11	6
GRMZM2G134162	8	159,952,116	159,957,434	28	6	7	5	GRMZM2G433959	8	159,956,854	159,957,921	3	2	2	2
GRMZM2G170969	8	162,305,487	162,306,280	28	19	7	4	XLOC_035676	8	162,305,650	162,306,050	15	6	6	3
GRMZM2G470942	8	162,335,276	162,337,118	19	9	5	1	XLOC_037473	8	162,335,899	162,340,140	29	10	4	2
GRMZM2G175406	8	162,588,168	162,589,986	18	1	3	2	XLOC_037480	8	162,588,472	162,589,576	9	1	2	1
GRMZM2G100318	8	163,128,903	163,133,067	5	5	5	4	XLOC_037489	8	163,131,948	163,132,447	3	3	3	2
XLOC_037490	8	163,203,210	163,204,952	22	4	6	3	XLOC_035697	8	163,203,559	163,204,199	7	2	3	2
GRMZM2G024799	8	163,925,848	163,929,685	56	15	19	15	XLOC_035716	8	163,927,153	163,928,646	16	3	6	2
GRMZM2G171996	8	164,285,437	164,286,435	16	4	4	4	XLOC_035726	8	164,285,571	164,286,601	35	7	10	6
GRMZM2G092817	8	164,822,797	164,826,113	70	18	21	18	XLOC_037525	8	164,824,872	164,825,883	20	4	2	3
GRMZM2G119397	8	164,952,357	164,956,271	6	0	1	0	XLOC_035742	8	164,948,306	164,956,406	27	8	11	8
GRMZM2G143640	8	166,202,041	166,203,641	16	2	4	0	XLOC_035766	8	166,202,461	166,203,023	8	0	1	0
GRMZM2G143640	8	166,202,041	166,203,641	16	2	4	0	XLOC_035767	8	166,203,213	166,204,219	6	1	2	0
AC232238.2_FG004	8	166,341,329	166,349,660	139	52	63	40	GRMZM2G168087	8	166,345,530	166,354,968	-	-	-	-
AC232238.2_FG004	8	166,341,329	166,349,660	139	52	63	40	XLOC_037558	8	166,345,444	166,349,060	23	8	10	4
GRMZM2G396248	8	166,450,330	166,453,602	30	11	14	10	XLOC_037564	8	166,450,439	166,452,505	22	8	11	6
GRMZM2G123394	8	166,704,257	166,706,406	31	14	15	12	XLOC_037576	8	166,705,309	166,706,025	7	4	5	2
GRMZM2G147544	8	166,741,560	166,743,934	53	18	19	18	XLOC_035780	8	166,742,338	166,743,981	20	8	9	9
GRMZM2G165177	8	168,042,162	168,044,733	20	8	9	7	XLOC_037608	8	168,042,162	168,044,284	20	8	9	7
GRMZM2G112836	8	168,615,332	168,618,760	20	4	7	10	XLOC_037620	8	168,614,934	168,616,983	44	17	21	24
XLOC_037628	8	168,809,267	168,815,005	18	7	9	8	XLOC_035823	8	168,814,382	168,814,995	-	-	-	-
GRMZM5G840487	8	169,068,643	169,073,066	42	15	21	23	XLOC_037635	8	169,071,358	169,072,345	7	4	2	5
GRMZM2G036448	8	169,675,588	169,681,153	28	14	13	10	XLOC_037654	8	169,676,228	169,679,455	17	9	7	6
AC218972.3_FG002	8	170,179,327	170,181,277	61	34	32	31	XLOC_035857	8	170,180,913	170,181,318	8	1	2	3
AC218972.3_FG004	8	170,197,942	170,201,141	7	2	2	3	XLOC_035858	8	170,198,174	170,198,543	-	-	-	-
XLOC_037665	8	170,218,080	170,221,722	3	0	2	2	XLOC_035859	8	170,218,672	170,221,170	3	0	2	2
GRMZM2G069078	8	170,417,531	170,420,149	10	2	2	2	XLOC_035863	8	170,418,065	170,420,083	10	2	2	2
GRMZM2G476009	8	170,885,667	170,888,437	23	4	5	5	XLOC_037681	8	170,885,781	170,886,008	-	-	-	-
GRMZM2G139374	8	171,307,877	171,313,576	34	18	17	12	XLOC_037695	8	171,307,811	171,308,656	29	14	13	8
GRMZM5G852779	8	171,685,242	171,688,996	-	-	-	-	XLOC_035902	8	171,684,881	171,685,894	6	5	2	5
GRMZM2G116086	8	172,112,785	172,121,960	8	2	1	3	XLOC_037721	8	172,113,702	172,114,108	-	-	-	-
GRMZM2G302639	8	172,961,557	172,963,511	53	15	8	13	GRMZM2G002858	8	172,963,108	172,967,039	22	3	4	4
GRMZM2G428179	8	173,488,303	173,489,765	37	18	14	18	XLOC_035950	8	173,488,595	173,488,860	13	6	0	6
GRMZM2G106303	9	7,393,694	7,395,273	-	-	-	-	XLOC_039370	9	7,393,341	7,394,579	6	3	3	3
GRMZM2G033413	9	8,229,971	8,240,288	36	13	13	14	XLOC_037868	9	8,239,976	8,240,287	15	5	5	4
GRMZM5G864689	9	9,510,044	9,513,407	11	3	3	2	XLOC_039403	9	9,512,594	9,512,978	2	0	0	1
XLOC_039408	9	9,955,287	9,956,481	10	2	1	3	XLOC_037884	9	9,955,397	9,956,538	10	2	1	3
GRMZM2G135501	9	10,946,567	10,947,842	18	9	8	8	XLOC_037888	9	10,947,383	10,948,025	17	9	7	8
GRMZM2G177231	9	11,715,793	11,720,000	8	2	3	1	XLOC_037898	9	11,718,330	11,720,005	5	0	1	0
XLOC_037912	9	12,797,426	12,798,671	13	4	3	3	GRMZM2G067053	9	12,796,516	12,799,152	97	29	32	26
GRMZM2G079306	9	12,875,085	12,883,372	29	17	18	11	GRMZM2G081573	9	12,874,481	12,883,237	39	17	22	11
GRMZM2G453349	9	13,358,509	13,364,979	6	3	3	3	XLOC_039474	9	13,363,266	13,364,514	-	-	-	-
GRMZM2G008305	9	13,663,138	13,664,819	18	5	6	5	XLOC_039478	9	13,663,717	13,664,603	18	5	6	5
GRMZM2G009139	9	14,119,204	14,121,439	31	9	11	7	XLOC_039485	9	14,119,262	14,120,618	30	9	11	7
GRMZM2G007683	9	14,917,204	14,925,544	18	7	8	7	XLOC_037946	9	14,925,130	14,925,915	-	-	-	-
XLOC_039504	9	15,374,868	15,386,749	28	9	8	7	XLOC_037949	9	15,385,595	15,386,863	6	4	3	2
XLOC_039505	9	15,386,103	15,386,781	6	4	3	2	XLOC_037949	9	15,385,595	15,386,863	6	4	3	2
GRMZM5G831993	9	15,616,216	15,621,318	5	1	1	1	GRMZM5G865595	9	15,620,220	15,624,084	18	4	5	4
GRMZM2G005080	9	16,001,623	16,002,878	2	1	0	1	XLOC_039514	9	16,001,752	16,002,137	-	-	-	-
GRMZM2G529313	9	16,689,343	16,701,067	11	7	6	6	GRMZM2G075488	9	16,685,283	16,689,432	6	2	3	3
GRMZM2G002227	9	16,906,529	16,909,767	6	3	4	4	XLOC_039525	9	16,906,526	16,907,818	3	1	2	2
AC231745.1_FG003	9	18,335,677	18,338,579	29	9	11	5	GRMZM5G838414	9	18,328,876	18,337,267	35	12	11	8
GRMZM2G103137	9	18,885,308	18,888,376	-	-	-	-	GRMZM2G020216	9	18,879,285	18,887,000	10	2	1	3
GRMZM2G028594	9	19,034,946	19,040,945	3	2	2	1	XLOC_038017	9	19,035,099	19,035,555	-	-	-	-
XLOC_039594	9	22,170,607	22,171,789	17	6	6	8	XLOC_038068	9	22,170,770	22,173,445	40	14	16	16
XLOC_038085	9	23,764,778	23,765,960	5	2	3	1	GRMZM2G118690	9	23,765,379	23,771,219	34	4	6	8
GRMZM2G050166	9	24,486,952	24,491,050	11	1	1	1	GRMZM2G350319	9	24,481,779	24,487,390	14	4	6	3
GRMZM2G304528	9	24,558,570	24,560,564	9	4	3	4	XLOC_039625	9	24,558,416	24,559,236	15	4	6	7
GRMZM2G071491	9	25,260,929	25,270,126	33	6	9	7	GRMZM2G071602	9	25,268,948	25,272,830	48	18	20	14
GRMZM2G383540	9	25,535,087	25,537,349	9	3	2	2	GRMZM2G083711	9	25,533,695	25,535,338	3	3	3	3
GRMZM2G475293	9	26,833,575	26,834,967	6	5	3	5	GRMZM2G475360	9	26,833,946	26,839,205	61	27	29	24
XLOC_038172	9	32,305,175	32,308,115	41	10	13	13	XLOC_039692	9	32,305,077	32,306,671	29	6	10	6
XLOC_038172	9	32,305,175	32,308,115	41	10	13	13	XLOC_039693	9	32,305,077	32,307,246	29	6	10	6
GRMZM2G058675	9	34,129,749	34,143,684	32	7	11	8	XLOC_039707	9	34,129,816	34,130,120	2	0	1	1
GRMZM2G027958	9	38,545,391	38,547,608	25	6	6	5	XLOC_038209	9	38,545,694	38,547,957	25	6	6	5
XLOC_038252	9	46,856,491	46,857,697	10	4	5	4	XLOC_039757	9	46,856,634	46,857,418	10	4	5	4
GRMZM2G027860	9	48,763,025	48,772,161	7	3	2	1	XLOC_039774	9	48,768,128	48,768,528	1	0	0	0
GRMZM2G136300	9	75,178,361	75,189,267	43	11	12	6	XLOC_038391	9	75,188,193	75,188,547	1	0	0	0
GRMZM2G080839	9	78,160,268	78,162,284	20	4	6	7	XLOC_039902	9	78,160,997	78,162,100	7	1	1	0
GRMZM2G161472	9	79,629,909	79,634,177	10	1	1	0	XLOC_038410	9	79,630,453	79,632,390	2	0	0	0
GRMZM2G022558	9	87,869,539	87,872,095	20	8	4	11	XLOC_039951	9	87,870,644	87,873,337	16	5	3	8
GRMZM2G159641	9	88,365,936	88,368,071	18	7	6	7	XLOC_038450	9	88,367,476	88,367,788	5	1	2	2
GRMZM2G155868	9	89,166,309	89,169,976	3	2	1	0	XLOC_039962	9	89,166,358	89,167,073	3	2	1	0
GRMZM2G101004	9	89,922,996	89,933,844	27	12	7	9	XLOC_038460	9	89,933,126	89,933,395	6	4	4	4
GRMZM2G141288	9	97,192,447	97,196,646	104	38	41	41	XLOC_040016	9	97,195,479	97,195,816	1	0	0	0
GRMZM2G071573	9	98,257,402	98,262,701	58	25	27	23	XLOC_040028	9	98,260,096	98,262,511	49	21	22	23
GRMZM2G071573	9	98,257,402	98,262,701	58	25	27	23	XLOC_040027	9	98,256,395	98,257,480	2	0	1	0

GRMZM2G033555	9	100,416,810	100,423,707	45	16	16	14	XLOC_040048	9	100,416,596	100,417,256	12	3	2	3
GRMZM2G092493	9	100,766,352	100,770,684	11	6	6	7	GRMZM2G092586	9	100,769,455	100,779,590	17	7	9	4
GRMZM2G122447	9	101,311,298	101,312,871	15	10	9	6	XLOC_040057	9	101,311,215	101,312,056	4	3	3	2
GRMZM5G836222	9	102,482,867	102,485,230	15	10	11	11	XLOC_038563	9	102,483,375	102,484,233	14	10	11	11
GRMZM2G065394	9	103,110,845	103,113,461	37	15	15	12	XLOC_040069	9	103,112,503	103,113,053	-	-	-	-
GRMZM2G086773	9	104,241,227	104,244,459	21	9	7	9	XLOC_040083	9	104,241,829	104,242,613	20	9	7	9
GRMZM2G139082	9	107,641,650	107,645,156	67	40	41	31	XLOC_040115	9	107,644,186	107,644,947	35	18	21	16
GRMZM2G055320	9	107,810,066	107,816,743	11	4	5	4	XLOC_040120	9	107,809,927	107,811,180	9	3	4	3
GRMZM2G405474	9	108,225,231	108,228,267	8	4	6	1	GRMZM2G106357	9	108,220,507	108,227,166	21	8	11	5
GRMZM2G038783	9	108,447,914	108,449,734	21	8	9	10	XLOC_040129	9	108,449,125	108,449,560	5	3	2	2
GRMZM2G103805	9	109,146,317	109,151,812	8	2	5	5	XLOC_038611	9	109,150,958	109,151,675	2	2	2	2
GRMZM2G157115	9	109,311,410	109,317,567	16	6	1	7	XLOC_038613	9	109,316,961	109,317,310	-	-	-	-
GRMZM2G095302	9	109,332,530	109,337,572	38	10	11	9	GRMZM2G095287	9	109,336,954	109,340,083	27	15	15	15
GRMZM2G017739	9	111,478,926	111,485,379	12	7	7	6	XLOC_038629	9	111,479,068	111,481,244	-	-	-	-
GRMZM2G017685	9	111,500,037	111,500,591	2	1	1	0	XLOC_038630	9	111,500,012	111,500,553	-	-	-	-
GRMZM2G049229	9	112,024,912	112,028,149	12	2	4	4	XLOC_038635	9	112,025,237	112,028,773	21	5	8	8
GRMZM2G144818	9	113,509,124	113,515,269	10	6	6	5	GRMZM2G144841	9	113,514,930	113,517,932	20	9	12	9
GRMZM2G428470	9	114,574,678	114,576,847	34	11	12	7	XLOC_038676	9	114,574,997	114,575,758	10	4	5	3
GRMZM2G028796	9	114,714,284	114,741,438	29	6	8	4	XLOC_040198	9	114,715,073	114,715,420	2	0	1	1
GRMZM2G069807	9	115,402,677	115,404,899	23	6	8	9	XLOC_038688	9	115,403,001	115,405,100	23	6	8	9
GRMZM2G409245	9	117,079,224	117,080,432	23	5	8	5	XLOC_038710	9	117,079,453	117,080,654	23	5	8	5
GRMZM2G035517	9	120,199,697	120,203,721	10	5	5	5	XLOC_040245	9	120,202,329	120,204,097	9	2	2	2
GRMZM2G152908	9	122,479,087	122,485,767	8	4	1	4	XLOC_038769	9	122,479,195	122,485,674	8	4	1	4
GRMZM2G359033	9	124,820,597	124,822,010	11	3	4	4	XLOC_040284	9	124,820,087	124,821,692	17	6	7	7
GRMZM2G105092	9	124,924,237	124,925,395	6	4	3	5	XLOC_038795	9	124,924,627	124,925,337	6	4	3	5
GRMZM2G145794	9	126,705,611	126,709,017	20	2	9	4	XLOC_038817	9	126,708,810	126,709,369	1	0	0	0
GRMZM2G030458	9	127,611,784	127,613,690	14	7	7	7	XLOC_040325	9	127,611,832	127,613,195	14	7	7	7
GRMZM2G157683	9	128348131	128349850	4	1	0	1	XLOC_040335	9	128348817	128349061	-	-	-	-
GRMZM2G017460	9	129107641	129110218	21	4	1	1	XLOC_038851	9	129109606	129110192	7	0	0	0
GRMZM2G121360	9	130353591	130355317	62	15	22	21	XLOC_040362	9	130352977	130355313	62	15	22	21
GRMZM2G134107	9	130759495	130760703	14	0	9	9	XLOC_040373	9	130759584	130759876	-	-	-	-
GRMZM5G889372	9	136728264	136734142	20	10	8	6	XLOC_038931	9	136732038	136733527	17	9	8	5
GRMZM2G078472	9	138040992	138044636	33	12	11	11	GRMZM2G07846	9	138039784	138042756	71	14	15	10
GRMZM2G053987	9	138684219	138687449	89	14	20	17	XLOC_038956	9	138687118	138687386	3	0	0	0
GRMZM2G049318	9	139137817	139139408	20	1	7	7	XLOC_038971	9	139138144	139138561	1	0	0	0
GRMZM2G103490	9	139870591	139873524	33	6	7	6	XLOC_040519	9	139871295	139871630	-	-	-	-
GRMZM2G151992	9	140100585	140102820	37	11	12	15	XLOC_038989	9	140100599	140101751	10	2	2	3
GRMZM2G031447	9	141795025	141797141	25	10	12	11	XLOC_039018	9	141795550	141797599	25	10	12	11
XLOC_039024	9	142276005	142281908	11	7	8	7	XLOC_040562	9	142281711	142282929	12	8	6	8
GRMZM2G081175	9	143586299	143590618	21	7	7	4	XLOC_040581	9	143586334	143588750	12	3	3	1
GRMZM2G178279	9	144209790	144213235	44	20	21	14	XLOC_040591	9	144212381	144213018	16	4	4	5
GRMZM2G177914	9	144855111	144858225	10	2	4	3	XLOC_040600	9	144855254	144855542	4	0	1	1
GRMZM2G177914	9	144855111	144858225	10	2	4	3	XLOC_040601	9	144857040	144857982	6	2	3	2
GRMZM2G105834	9	145045717	145047357	45	15	14	11	XLOC_040607	9	145046440	145047353	6	0	0	0
GRMZM2G091742	9	145424529	145426536	18	3	3	2	XLOC_040615	9	145424444	145425939	2	0	0	1
GRMZM2G067426	9	145592092	145595076	90	30	38	31	XLOC_040619	9	145591378	145592855	20	10	10	6
GRMZM2G067225	9	145604342	145606411	8	4	5	3	XLOC_039083	9	145604342	145606676	8	4	5	3
GRMZM2G050159	9	149280835	149282968	4	2	2	2	XLOC_039142	9	149281940	149282971	3	1	1	2
GRMZM2G004466	9	149512257	149513861	58	34	34	30	XLOC_040707	9	149512679	149513156	5	1	1	1
GRMZM2G004377	9	149513489	149519493	56	31	33	33	GRMZM2G00446	9	149512257	149513861	58	34	34	30
GRMZM2G036411	9	150509518	150510116	15	13	10	5	XLOC_039167	9	150509633	150510254	15	13	10	5
GRMZM2G036351	9	150514279	150515757	11	5	5	4	XLOC_039168	9	150514799	150515518	3	1	1	0
AC149829.2_FG00	9	151381654	151383564	13	3	3	2	XLOC_039183	9	151382817	151383310	13	3	3	2
XLOC_040760	9	151414935	151418059	10	4	5	3	XLOC_039184	9	151417535	151418083	5	2	2	2
GRMZM2G178787	9	151538905	151543176	31	21	21	17	GRMZM2G17882	9	151542239	151546401	40	25	26	21
GRMZM2G054969	9	151692176	151696132	34	12	11	11	XLOC_039195	9	151692379	151692756	1	1	1	1
GRMZM2G127072	9	151918650	151921069	48	33	29	28	XLOC_040778	9	151919604	151920602	4	0	0	0
GRMZM2G126682	9	151998333	152000250	21	7	6	5	XLOC_040785	9	151998593	151999043	-	-	-	-
GRMZM2G126682	9	151998333	152000250	21	7	6	5	XLOC_040786	9	151999349	152000146	13	3	3	3
GRMZM2G010017	9	152081582	152086034	13	6	3	4	XLOC_040788	9	152081653	152082635	-	-	-	-
GRMZM2G330019	9	153128005	153133217	48	20	22	20	XLOC_039241	9	153129753	153132086	9	0	0	0
GRMZM2G393334	9	154723964	154729849	26	7	8	7	GRMZM2G09249	9	154729257	154731780	23	3	6	3
GRMZM2G168355	9	155707392	155709337	18	4	5	4	XLOC_039294	9	155707632	155709055	18	4	5	4
GRMZM2G462243	9	155855551	155856182	12	7	7	7	XLOC_040883	9	155855551	155856028	8	6	7	6
GRMZM2G161905	9	155866514	155867635	8	2	2	3	XLOC_040885	9	155865947	155867472	8	2	2	3
GRMZM2G066165	10	1078071	1084308	6	0	1	1	GRMZM2G06614	10	1083998	1085635	7	2	1	2
GRMZM2G043150	10	1950526	1959850	28	9	10	5	GRMZM2G04311	10	1948867	1951755	11	5	7	3
GRMZM2G151903	10	2435439	2439171	22	6	7	4	XLOC_007601	10	2436111	2437872	21	5	6	4
GRMZM2G151977	10	2448850	2453307	29	14	14	13	XLOC_007603	10	2449057	2449866	25	11	11	11
GRMZM2G058481	10	3176422	3177326	12	3	5	2	XLOC_007620	10	3176413	3176925	6	2	4	1
GRMZM2G058595	10	3193010	3194101	66	21	33	25	XLOC_007621	10	3192437	3194049	27	10	15	13
XLOC_006252	10	3194760	3196017	26	7	11	9	XLOC_007622	10	3194813	3195851	26	7	11	9
XLOC_007649	10	4056048	4057136	4	1	3	2	GRMZM2G08831	10	4056253	4065016	4	1	3	2
GRMZM2G054481	10	4529937	4531806	1	1	1	1	XLOC_006267	10	4531087	4531735	-	-	-	-
GRMZM2G022318	10	4614582	4617945	71	32	33	22	XLOC_007662	10	4616867	4617850	20	15	15	10
GRMZM2G434170	10	5225476	5228126	3	1	0	1	XLOC_007686	10	5226560	5227949	1	1	0	1
GRMZM2G464328	10	5645825	5646958	17	5	3	7	GRMZM2G16411	10	5645510	5651577	43	9	16	14
GRMZM2G057753	10	6402110	6402921	35	9	14	12	XLOC_007707	10	6402289	6402853	16	6	6	6
AC202107.3_FG00	10	6533858	6535307	4	1	1	1	XLOC_007708	10	6533850	6534518	-	-	-	-
GRMZM2G136910	10	8853009	8853996	7	2	2	2	XLOC_006324	10	8853027	8853868	7	2	2	2
GRMZM5G813470	10	10887778	10892143	36	9	8	7	XLOC_006347	10	10890911	10891138	-	-	-	-
GRMZM2G015869	10	12927390	12933138	47	18	19	12	XLOC_006355	10	12930630	12933118	12	2	1	1
GRMZM2G009448	10	17611983	17615854	8	4	4	3	XLOC_007808	10	17614876	17615435	5	2	2	2
GRMZM2G169240	10	19075431	19077551	18	6	5	7	XLOC_006394	10	19075885	19076795	16	4	4	5
XLOC_006404	10	20328528	20329584	2	1	1	1	XLOC_007823	10	20327689	20328985	2	1	1	1
GRMZM2G036427	10	23221552	23223794	40	14	17	16	XLOC_007842	10	23221601	23222141	11	7	8	7
XLOC_007844	10	23390500	23392415	5	1	1	1	XLOC_006424	10	23390706	23392369	5	1	1	1
GRMZM2G054250	10	25562436	25565712	12	3	4	2	GRMZM2G05439	10	25565234	25575047	59	18	26	25
GRMZM2G420407	10	29229592	29231490	10	3	2	3	GRMZM2G12053	10	29230376	29236119	-	-	-	-
GRMZM2G102683	10	29631122	29634845	22	7	7	5	XLOC_007896	10	29633337	29634690	15	5	4	2
GRMZM2G171518	10	33613510	33625476	41	9	11	6	XLOC_007910	10	33623886	33625576	6	1	0	0
GRMZM2G565911	10	35929292	35930296	4	1	2	1	XLOC_007917	10	35929743	35930125	-	-	-	-
GRMZM2G094352	10	49989446	49992027	21	6	6	9	XLOC_006523	10	49989787	49992026	21	6	6	9
GRMZM2G178817	10	58462136	58463278	27	7	7	7	XLOC_007999	10	58462564	58463138	14	3	2	3
XLOC_006580	10	61960766	61968571	18	8	6	7	XLOC_008016	10	61960807	61961374	3	1	1	1
GRMZM2G364748	10	68937520	68938909	3	2	2	2	XLOC_006623	10	68937698	68938766	2	1	1	1
GRMZM2G113682	10	75157271	75167108	23	6	5	4	XLOC_006656	10	75157846	75158315	1	1	0	1
GRMZM2G110145	10	77282595	77289172	45	17	13	21	XLOC_008118	10	77287726	77288880	4	0	0	0
GRMZM2G078906	10	78074137	78076764	24	9	11	9	XLOC_008126	10	78074671	78075747	12	3	6	5
GRMZM2G123886	10	79150828	79152284	14	8	8	9	GRMZM5G80630	10	79150370	79160123	-	-	-	-
XLOC_008140	10	79422406	79423830	28	4	5	4	XLOC_006681	10	79422812	79423830	28	4	5	4
GRMZM2G086496	10	80056275	80062527	93	25	24	17	XLOC_008144	10	80057798	80058334	6	2	2	2
GRMZM2G325907	10	81840736	81843543	20	5	6	8	XLOC_006709	10	81841212	81841481	5	2	3	3
GRMZM2G028104	10	86998288	87000589	15	6	4	4	XLOC_008201	10	86998117	87000393	16	6	5	5

GRMZM2G015605	10	87283734	87284881	4	3	3	4	XLOC_006771	10	87284105	87284585	1	1	0	1
GRMZM2G138252	10	90257150	90259318	24	3	7	4	XLOC_008230	10	90258469	90259237	17	5	8	4
GRMZM2G110567	10	92550666	92552639	10	4	5	2	XLOC_008246	10	92550696	92552545	8	2	3	1
GRMZM2G131611	10	95136396	95139678	41	16	11	20	XLOC_006835	10	95139127	95139940	5	3	2	1
GRMZM2G019246	10	99220164	99223049	89	35	35	29	XLOC_006863	10	99220564	99221170	10	5	5	4
GRMZM2G119116	10	99619669	99624690	15	6	6	3	GRMZM2G119127	10	99624013	99624641	15	6	6	3
XLOC_006878	10	100699317	100701433	7	0	1	1	XLOC_008322	10	100699105	100699594	3	0	1	1
GRMZM2G122437	10	109913509	109916009	6	4	4	4	XLOC_008380	10	109915061	109915553	-	-	-	-
GRMZM2G451716	10	111444985	111453558	20	5	2	4	XLOC_008393	10	111445144	111445552	7	2	1	1
GRMZM2G138355	10	112222702	112225808	40	11	9	11	XLOC_008397	10	112222537	112223557	9	4	2	5
XLOC_007018	10	117485115	117486672	12	3	4	1	XLOC_008454	10	117484811	117486494	5	0	1	0
GRMZM2G136508	10	117641432	117645132	30	13	12	11	XLOC_008458	10	117643682	117643935	3	1	2	2
GRMZM2G359735	10	126160075	126165445	22	5	10	8	XLOC_008552	10	126160350	126160680	3	1	1	0
GRMZM2G000261	10	128580332	128582775	15	9	9	10	XLOC_007148	10	128580520	128581374	-	-	-	-
GRMZM5G893444	10	131910570	131914628	40	25	26	23	XLOC_007189	10	131913905	131914803	26	15	18	15
GRMZM2G046952	10	132120374	132122335	24	4	10	7	XLOC_007197	10	132121098	132121375	2	1	2	1
GRMZM2G131969	10	132197230	132201623	20	3	3	4	GRMZM2G132218	10	132201404	132204663	4	1	1	1
GRMZM2G046841	10	132446398	132447255	21	10	7	11	XLOC_007203	10	132446577	132447208	21	10	7	11
GRMZM2G169690	10	135506682	135509408	14	5	8	8	XLOC_008671	10	135506483	135507638	12	5	7	7
GRMZM2G098676	10	136041276	136044237	47	14	18	15	XLOC_008678	10	136041206	136042977	35	10	13	10
XLOC_007279	10	136721249	136721959	4	1	0	2	XLOC_008699	10	136718978	136721714	-	-	-	-
GRMZM2G142832	10	137853661	137856863	85	21	20	25	XLOC_008712	10	137853826	137854820	34	4	4	7
GRMZM5G803874	10	138512829	138515377	19	4	5	2	XLOC_007308	10	138512572	138514126	8	3	4	2
GRMZM2G555422	10	139384342	139385051	17	3	4	2	XLOC_007322	10	139384420	139384780	3	1	1	1
GRMZM2G148495	10	142868690	142869793	49	21	21	25	XLOC_008813	10	142868770	142869727	41	18	20	21
GRMZM2G431504	10	143178859	143181050	11	4	4	4	XLOC_008824	10	143178937	143179364	9	2	2	2
GRMZM5G895313	10	143487116	143488331	2	0	1	1	XLOC_008827	10	143487540	143488048	2	0	1	1
GRMZM5G818977	10	143740959	143744585	19	12	12	10	XLOC_008830	10	143743661	143744585	6	6	6	3
GRMZM2G085236	10	144340337	144347928	40	9	20	17	XLOC_007429	10	144346275	144346895	8	2	5	5
GRMZM2G143480	10	145330728	145339330	21	9	8	4	GRMZM2G143499	10	145327308	145330955	10	4	4	4
GRMZM2G143402	10	145369441	145373354	3	0	1	0	XLOC_008858	10	145369416	145371738	3	0	1	0
GRMZM2G067424	10	146078469	146080245	20	8	11	8	XLOC_008876	10	146078896	146080074	11	4	5	4
GRMZM2G179532	10	146745295	146747273	27	4	5	4	XLOC_008900	10	146745094	146746414	32	5	8	5
GRMZM2G340065	10	146895394	146899390	48	18	20	17	XLOC_007491	10	146893472	146899173	64	22	23	20
GRMZM2G150014	10	147014559	147017456	12	4	7	2	XLOC_007493	10	147015584	147015930	-	-	-	-
GRMZM2G150286	10	147043160	147045718	30	10	4	10	XLOC_007494	10	147045247	147045660	9	2	2	1
GRMZM2G142757	10	147855454	147859264	19	13	11	13	XLOC_008935	10	147857896	147859019	12	8	7	8
GRMZM2G142820	10	147868922	147872174	35	13	13	12	XLOC_007519	10	147871601	147872416	7	6	6	6
GRMZM2G074759	10	148065497	148067641	22	12	12	12	XLOC_007524	10	148065617	148066552	9	6	6	5
GRMZM2G074718	10	148075099	148076695	8	4	6	3	GRMZM2G074107	10	148074813	148081156	29	5	7	6
GRMZM2G406099	10	148283968	148286266	19	4	4	8	XLOC_007531	10	148284757	148286459	19	4	4	8
GRMZM2G001934	10	148312549	148316275	22	6	5	3	XLOC_007535	10	148313887	148314859	16	4	4	1
GRMZM2G104613	10	148407014	148410710	7	3	3	0	GRMZM2G104658	10	148410223	148412237	22	4	4	4
GRMZM2G110908	10	148737011	148739143	9	1	3	2	GRMZM2G110869	10	148737480	148741164	14	4	4	3
GRMZM2G011590	10	148821382	148830411	7	1	1	1	XLOC_008968	10	148828169	148829357	5	1	1	1
GRMZM5G824597	10	149178679	149182049	7	0	1	2	GRMZM5G885706	10	149181221	149182500	2	0	0	1
GRMZM5G836471	10	149245361	149251265	18	5	5	3	XLOC_007556	10	149246609	149248076	13	4	4	3

Supplemental File 1. Detailed methods and any associated references.

METHODS

Plant materials

Two maize parental lines AC7643 and AC7729/TZSRW, and two offspring recombination inbred lines (RILs), RIL208 and RIL64, were selected for analysis based on drought tolerance or sensitivity, as previously characterized (1,2). Seeds were surface-sterilized, and grown under well water (WW) and water stress (WS) as previously described (3). Seedlings at the three-leaf stage were then treated with 10% (w/v) polyethylene glycol PEG 8000 (Sigma-Aldrich) for 24 h, and roots from three plants per inbred line were harvested, pooled, flash frozen in liquid nitrogen, and stored at -80 ℃. In addition, root morphology was examined with a professional-grade Epson XL1000 scanner (Japan) equipped with a WinRhizo Pro 2007a (Regent Instrument Inc., Quebec, Canada) image analysis system. Root length (cm), surface area (cm^2) and volume (cm^3) were measured, along with number of root forks, and total root tips.

Libraries constructions and sequencing

Library construction and strand-specific RNA sequencing

Total RNA was extracted from roots using TRIzol® (Invitrogen, USA), and treated with RNase-free DNase I. RNA integrity number was measured to be > 8 on a 2100 Bioanalyzer (Agilent Technologies). Libraries for strand-specific RNA sequencing were constructed as described (4), with minor modifications. Briefly, mRNA was purified by OligoTex mRNA Midi Kit (Qiagen), and processed with RNA fragmentation reagents (Ambion). First-strand cDNA was synthesized using random hexamer primers and second-strand cDNA was synthesized with dUTP instead of

dTTP. Double-stranded cDNA fragments were purified and ligated with adaptors. Finally, second-strand cDNA was removed by digesting dUTP with AmpErase UNG (Applied Biosystems). Libraries were sequenced at Beijing Genomics Institute (Shenzhen, China) using Illumina deep sequencing, following the manufacturer's instructions. RNAs were isolated from two independent replicates of each line under two water conditions for library construction and sequencing. For replicate I, libraries were sequenced on HiSeq 2000 (Illumina, San Diego, CA, USA) at Beijing Genomics Institute. For replicate II, libraries were sequenced on HiSeq X10 system (Illumina, San Diego, CA, USA). We calculated Spearman's correlation coefficients between two replicates for each sample using mapped reads number of each expressed genes in R.

Library construction and smRNA sequencing

SmRNA libraries were constructed from root total RNA using commercially available reagent sets, following the manufacturer’s protocol (Solexa) and Wang et al. (5). Briefly, 16~35 nt smRNAs were separated from total RNA by 15% denaturing PAGE, and ligated to adaptors both at 5' and 3'. Ligation products were gel-purified by 10% denaturing PAGE, and reverse-transcribed. For replicate I and replicate II, libraries were sequenced on HiSeq 2000 and HiSeq 2500 SE50 system, respectively. (Illumina, California, USA).

Methylated DNA immunoprecipitation sequencing (MeDIP sequencing)

DNA was extracted using CTAB from the same samples used for RNA isolation. Purified DNA was analyzed by MeDIP sequencing at Beijing Genomics Institute, following published methods (6). Briefly, DNA was fragmented by sonication into 100-500 bp fragments, purified with a Gel Extraction Kit (Qiagen) and processed with a Paired-End DNA Sample Prep Kit (Illumina, San Diego, CA, USA) to end-repair, phosphorylate, and ligate with Illumina sequencing primer adapters. Methylated fragments were then enriched by Magnetic Methylated DNA Immunoprecipitation Kit (Diagenod, Liège, Belgium) following the manufacturer’s recommendations, and amplified by PCR. Amplification products between 220 and 320 bp were gel-purified with QIAquick Gel Extraction Kit (Qiagen, Valencia, CA, USA), and sequenced on

Hiseq 2000 (Illumina, San Diego, CA, USA) to generate paired-end 50-bp reads.

Expression and inheritance of NAT pairs

Fragment per kilobase of exon model per million mapped reads (FPKM) values for Natural antisense transcript (NAT) pairs were analyzed by non-parametric Spearman correlation to test whether antisense transcripts modulate expression of the corresponding sense transcripts. This analysis was carried out because FPKM values were not normally distributed based on the Shapiro-Wilk test (*P* value < 0.001), even in log space (*P* value < 0.001). Correlations between sense and antisense expression were calculated in R version 3.2.3.

Expression specificity was measured by Shannon entropy (7,8), which was calculated according to

$$H_p = \sum_{i=1}^{n} p_i \log p_i$$

This formula is as reported (7), except that p_i was replaced with relative abundance of the transcript in sample i:

$$p_i = \frac{FPKM_i}{\sum_{i=1}^{n} FPKM_i}$$

Inheritance of NAT pairs was investigated by comparing expression of specific genes in parental lines (AC7643 and AC7729/TZSRW) and offspring RILs (RIL208 and RIL64). For this analysis, a statistical model developed by Li et al. (9) for maize lncRNA was applied. The difference in expression of the same gene between AC7643 and derivative lines was calculated as $(\mathrm{FPKM}_{\mathrm{AC7643}} - \mu_{\mathrm{RIL208+RIL64}}) / (\mathrm{FPKM}_{\mathrm{AC7643}} + \mu_{\mathrm{RIL208+RIL64}})$, where $\mu_{\mathrm{RIL208+RIL64}}$ is the average expression in derivative lines. Similarly, the difference in expression between AC7729/TZSRW and RILs was calculated according to $(\mathrm{FPKM}_{\mathrm{AC7729/TZSRW}} - \mu_{\mathrm{RIL208+RIL64}}) / (\mathrm{FPKM}_{\mathrm{AC7729/TZSRW}} + \mu_{\mathrm{RIL208+RIL64}})$. If gene

expression was similar between parental and RILs, a scatter plot of differences from AC7643 against those from AC7729/TZSRW would have data points centered together (9). The difference in expression from both parental lines was then calculated as the vector $C^2 = A^2 + B^2$.

Experimental validation of maize NAT pairs

Strand-specific reverse transcriptase and SYBR Green-based quantitative real-time PCR (qPCR) were performed to validate and quantify sense and antisense transcripts in maize lines AC7643 and AC7729/TZSRW. The total RNA used in qPCR are generated from the same RNAs used for library construction. To avoid genomic DNA contamination, total RNA was treated with 2 U DNase I (Takara) for 30 minutes at 37 ℃ and then was inactivated by incubating at 85 ℃ for 10 minutes before reverse transcription (RT). First-strand cDNA synthesis was conducted using a specific primer (reverse primer) for each gene and random primers in the Prime-Script RT reagent Kit (TaKaRa, Dalian, China) according to the manufacturer's instructions. Primers with sequences and annealing temperatures listed in Supplemental Table 4 were designed within transcript specific sequences using Beacon Designer version 8.0 (Palo Alto, CA, USA), and synthesized by Invitrogen (Shanghai, China). The protocol for strand-specific amplification has been described in detail (10). Specific primers of each NAT pair sense transcript and NAT were used for qPCR analysis and 18s rRNA and glyceraldehyde-3-phosphate dehydrogenase (*GAPDH*) were amplified as endogenous housekeeping controls. Relative gene expression in different samples was determined based on the cycle threshold (CT) value of target gene and two housekeeping controls using the following formula: $2^{-\Delta CT}$ ($\Delta CT = CT_{target} - CT_{housekeeping}$). For each sample, qPCR was conducted in technical triplicates with at least two biological replicates. Strand specific PCR reactions were initially denatured at 94 ℃ for 5 minutes, and then amplified over 35 cycles of denaturation at 94 ℃ for 1.5 minutes, annealing at primer-specific temperatures for 1

minutes, and extension at 72 ℃ for 2 minutes. Reactions were held at 4 ℃ and PCR products were visualized on 1% agarose. qPCR was conducted in 96-well plates and performed on the Bio-Rad CFX96 real-time PCR System (Bio-Rad, CA) under the following cycling conditions (95 ℃ for 1 min, 40 cycles of 95 ℃ for 5 s, and primer-specific temperatures for 30 s). The melting curves were generated at 60 ℃–95 ℃ after 40 cycles to check for primer specificity.

References

1. Lu, Y., Hao, Z., Xie, C., Crossa, J., Araus, J.-L., Gao, S., Vivek, B.S., Magorokosho, C., Mugo, S. and Makumbi, D. (2011) Large-scale screening for maize drought resistance using multiple selection criteria evaluated under water-stressed and well-watered environments. *Field Crops Research*, **124**, 37-45.
2. Lu, Y., Zhang, S., Shah, T., Xie, C., Hao, Z., Li, X., Farkhari, M., Ribaut, J.M., Cao, M., Rong, T. *et al.* (2010) Joint linkage-linkage disequilibrium mapping is a powerful approach to detecting quantitative trait loci underlying drought tolerance in maize. *Proceedings of the National Academy of Sciences of the United States of America*, **107**, 19585-19590.
3. Li, Y., Sun, C., Huang, Z., Pan, J., Wang, L. and Fan, X. (2009) Mechanisms of progressive water deficit tolerance and growth recovery of Chinese maize foundation genotypes Huangzao 4 and Chang 7-2, which are proposed on the basis of comparison of physiological and transcriptomic responses. *Plant Cell Physiol*, **50**, 2092-2111.
4. Hirsch, C.N., Foerster, J.M., Johnson, J.M., Sekhon, R.S., Muttoni, G., Vaillancourt, B., Penagaricano, F., Lindquist, E., Pedraza, M.A., Barry, K. *et al.* (2014) Insights into the maize pan-genome and pan-transcriptome. *Plant Cell*, **26**, 121-135.
5. Wang, X., Elling, A.A., Li, X., Li, N., Peng, Z., He, G., Sun, H., Qi, Y., Liu, X.S. and Deng, X.W. (2009) Genome-wide and organ-specific landscapes of epigenetic modifications and their relationships to mRNA and small RNA transcriptomes in maize. *Plant Cell*, **21**, 1053-1069.
6. Taiwo, O., Wilson, G.A., Morris, T., Seisenberger, S., Reik, W., Pearce, D., Beck, S. and Butcher, L.M. (2012) Methylome analysis using MeDIP-seq with low DNA concentrations. *Nat. Protocols*, **7**, 617-636.
7. Makarevitch, I., Eichten, S.R., Briskine, R., Waters, A.J., Danilevskaya, O.N., Meeley, R.B., Myers, C.L., Vaughn, M.W. and Springer, N.M. (2013) Genomic distribution of maize facultative heterochromatin marked by trimethylation of H3K27. *Plant Cell*, **25**, 780-793.
8. Schug, J., Schuller, W.P., Kappen, C., Salbaum, J.M., Bucan, M. and Stoeckert, C.J., Jr. (2005) Promoter features related to tissue specificity as measured by Shannon entropy. *Genome Biol*, **6**, R33.
9. Li, L., Eichten, S.R., Shimizu, R., Petsch, K., Yeh, C.T., Wu, W., Chettoor, A.M., Givan, S.A., Cole, R.A., Fowler, J.E. *et al.* (2014) Genome-wide discovery and characterization of maize long non-coding RNAs. *Genome Biol*, **15**, R40.
10. Ho, E.C., Donaldson, M.E. and Saville, B.J. (2010) Detection of antisense RNA transcripts by

strand-specific RT-PCR. *Methods in molecular biology*, **630**, 125-138.

Supplemental File 2. The information of housekeeping genes in maize (genome B73_RefGen_v3)

Gene_ID	Chr:Start-End	Strand	Gene_ID	Chr:Start-End	Strand
GRMZM2G137236	1:3002518-3020918	+	GRMZM2G421857	4:235983602-235992984	-
GRMZM2G014382	1:4055334-4063464	-	GRMZM2G165917	4:238064651-238068549	-
GRMZM2G169320	1:13535097-13538715	+	GRMZM2G155384	4:238153955-238162605	-
GRMZM2G142557	1:15726595-15735402	+	GRMZM2G174568	4:238972142-238976952	+
GRMZM2G016511	1:16602780-16605847	-	GRMZM2G169998	4:240050066-240053668	-
GRMZM5G808775	1:20634063-20639087	-	GRMZM2G018251	4:240584583-240588730	-
GRMZM5G880508	1:22246085-22252609	+	GRMZM2G163277	4:241004643-241011898	-
GRMZM2G457201	1:22508554-22512149	+	GRMZM2G089498	5:108149-110727	-
GRMZM2G007486	1:22735201-22738828	+	GRMZM2G069916	5:1315093-1322855	+
GRMZM2G032423	1:24930249-24940943	+	GRMZM2G004140	5:1682058-1686853	-
GRMZM2G376074	1:25149188-25170018	-	GRMZM2G165209	5:2745757-2750912	+
GRMZM2G472693	1:26428391-26433582	-	GRMZM2G108355	5:3660470-3664915	-
GRMZM2G128613	1:28070072-28078940	-	GRMZM5G844195	5:3841759-3850283	+
GRMZM2G180458	1:29255283-29257147	+	GRMZM2G099630	5:4351432-4366692	-
GRMZM2G096877	1:31378835-31383251	+	GRMZM2G166049	5:4571239-4574942	+
GRMZM2G165926	1:32997147-33004423	-	AC195340.3_FG002	5:5425210-5428042	+
GRMZM2G058910	1:34658309-34665859	+	GRMZM2G347645	5:10684673-10692253	+
GRMZM2G164562	1:35459890-35470305	-	GRMZM2G145854	5:10792003-10795994	+
GRMZM2G048703	1:37545427-37556438	-	GRMZM2G300944	5:12398192-12403243	-
GRMZM2G100620	1:37875667-37879128	-	GRMZM2G026180	5:12536047-12543477	+
GRMZM2G096424	1:42294432-42312413	-	GRMZM5G895064	5:16701221-16703382	-
GRMZM2G024159	1:44535920-44547250	+	GRMZM2G403218	5:19269959-19277452	-
GRMZM2G069618	1:56894719-56897083	+	GRMZM2G034276	5:24734055-24743740	-
GRMZM5G885628	1:61021664-61024477	-	GRMZM2G120973	5:25818354-25825972	+
GRMZM2G030805	1:74783737-74792959	-	GRMZM2G058345	5:30967094-30972320	+
GRMZM2G098208	1:95094430-95100116	+	GRMZM2G046021	5:33838765-33844703	+
GRMZM2G033876	1:99855221-99859703	+	GRMZM2G111238	5:39201981-39215991	+
GRMZM2G009655	1:123760528-123780037	-	GRMZM2G014180	5:40103819-40128576	+
GRMZM2G152526	1:143634168-143638506	+	GRMZM2G151195	5:40362229-40381752	-
GRMZM2G118743	1:145298496-145324380	+	GRMZM2G113592	5:40789466-40794667	-
GRMZM2G135045	1:148459825-148467847	+	GRMZM2G350626	5:40855918-40879122	-
GRMZM2G065066	1:155206232-155209985	-	GRMZM2G091151	5:49545068-49551568	+
GRMZM2G086887	1:157239313-157254106	+	GRMZM2G415846	5:52415675-52424885	-
GRMZM2G069410	1:170688753-170691656	-	GRMZM2G162184	5:55243566-55261337	+
GRMZM2G028700	1:174660753-174671403	-	GRMZM5G817439	5:55814659-55822811	-
GRMZM2G028900	1:174695630-174699469	-	GRMZM2G084942	5:59318789-59331769	-
GRMZM2G113911	1:183876411-183883053	+	GRMZM2G075257	5:61250039-61255769	-
GRMZM2G166355	1:190728885-190734308	+	GRMZM2G152111	5:62225546-62230385	+
GRMZM2G074114	1:191480086-191483144	+	GRMZM2G145112	5:64131621-64133526	+
GRMZM2G045503	1:196671756-196675994	+	GRMZM2G132060	5:65249498-65254103	-
GRMZM2G019050	1:196729334-196736717	-	GRMZM2G466270	5:65470804-65484230	+
GRMZM2G115750	1:201522268-201525873	-	GRMZM2G377887	5:65977716-65983043	+
GRMZM5G833477	1:202028504-202031821	+	GRMZM2G429241	5:67423849-67430870	+
GRMZM2G389233	1:202789101-202801071	+	GRMZM2G163849	5:68902393-68913355	-
GRMZM2G167836	1:204845614-204848541	+	GRMZM2G120578	5:70714197-70720379	-
GRMZM2G122064	1:207642133-207648514	-	GRMZM2G171354	5:76138702-76143443	+
GRMZM2G167651	1:216734844-216741515	+	GRMZM2G109268	5:76970869-76974497	+
GRMZM2G108277	1:218719115-218722046	+	GRMZM2G139977	5:77231501-77243624	+
GRMZM2G146111	1:219421847-219428321	+	GRMZM2G410393	5:78853855-78860664	+
GRMZM5G868683	1:226189256-226196151	+	GRMZM2G138505	5:87439113-87448768	+

GRMZM2G449083	1:227311148-227316282	-
GRMZM2G170699	1:235504527-235508830	-
GRMZM2G036765	1:237146881-237152159	-
GRMZM2G000540	1:238877406-238886155	-
GRMZM2G118467	1:241962723-241976179	+
GRMZM2G382077	1:242209875-242228024	-
GRMZM2G147402	1:242450765-242454259	+
GRMZM2G070061	1:253553474-253556659	+
GRMZM2G420121	1:261237302-261240035	-
GRMZM2G097164	1:263265598-263269102	-
GRMZM2G397836	1:263270032-263275500	-
GRMZM5G810275	1:264804400-264808840	-
GRMZM2G002687	1:264809026-264817001	+
GRMZM2G126038	1:268235564-268239476	-
GRMZM2G044569	1:268942960-268947663	+
GRMZM2G133652	1:274128154-274131626	-
GRMZM2G156800	1:280154384-280164446	-
GRMZM2G138861	1:280356771-280364881	+
GRMZM2G060765	1:286737004-286741315	-
GRMZM5G825524	1:289263282-289288561	+
GRMZM2G134227	1:291327116-291332488	-
GRMZM2G116908	1:291596762-291605143	+
GRMZM2G479744	1:294782663-294790423	+
GRMZM2G369912	1:297451770-297457026	+
GRMZM2G016892	1:297877793-297883989	-
GRMZM2G115456	1:299272434-299277717	+
GRMZM2G036169	1:300351265-300364771	+
GRMZM2G084252	2:1547887-1552664	+
GRMZM2G078541	2:2370730-2375919	+
GRMZM2G009994	2:2544057-2550523	-
GRMZM2G061745	2:3610614-3614343	-
GRMZM2G056572	2:3973404-3978693	+
GRMZM2G001334	2:8295857-8302610	-
GRMZM2G094255	2:9552778-9557118	-
GRMZM2G102088	2:11204909-11210740	+
GRMZM2G130889	2:11392998-11404255	-
GRMZM2G156638	2:11999763-12008624	+
GRMZM2G125728	2:12359037-12364897	-
GRMZM2G032260	2:13877191-13883313	-
GRMZM2G114220	2:15995986-16001887	-
GRMZM2G161913	2:16461518-16484071	+
GRMZM2G019876	2:18627525-18629118	-
GRMZM2G099130	2:19134109-19138634	-
GRMZM5G827171	2:25924091-25934715	+
GRMZM2G177273	2:27017463-27020999	-
GRMZM2G176881	2:27064850-27068690	+
GRMZM2G160801	2:33391502-33414606	+
GRMZM2G139038	2:35820756-35825527	-
GRMZM2G124079	2:39322132-39324061	+
GRMZM2G029001	2:41635038-41637113	+
GRMZM2G049568	2:44178108-44186133	+
GRMZM2G147619	2:52449235-52456233	-
GRMZM2G109818	5:88695793-88701952	-
GRMZM2G462639	5:96430593-96440162	+
GRMZM2G041878	5:105681405-105818171	-
GRMZM2G090480	5:123874743-123877316	-
GRMZM2G038126	5:125838970-125854508	+
GRMZM2G118098	5:130532791-130538994	+
GRMZM2G174572	5:130986092-130995528	+
GRMZM2G102021	5:135622335-135627731	+
GRMZM2G011777	5:136996393-136999749	+
GRMZM2G021110	5:152955772-152971505	-
GRMZM2G177781	5:153582672-153591101	+
GRMZM2G137528	5:158644303-158649135	+
GRMZM2G099334	5:167471479-167473890	-
GRMZM2G128189	5:173733240-173739944	+
GRMZM2G113596	5:174403414-174412387	-
GRMZM2G115420	5:176261637-176267226	-
GRMZM2G092447	5:179981344-179986272	-
GRMZM2G006707	5:181690998-181698715	-
GRMZM2G095194	5:182500583-182506476	+
GRMZM2G139858	5:188657088-188662186	+
GRMZM2G588223	5:188801740-188807563	+
AC209208.3_FG005	5:193289286-193293211	-
GRMZM2G037200	5:196068947-196075524	+
GRMZM2G331032	5:198570127-198576608	+
GRMZM2G027741	5:199427281-199430954	+
GRMZM2G092959	5:200522047-200533509	-
GRMZM2G038375	5:203271347-203275228	+
GRMZM2G145594	5:204479884-204482990	+
GRMZM2G114371	5:207078523-207083411	+
GRMZM2G110185	5:212423808-212427681	-
GRMZM2G089466	5:212727809-212732740	+
GRMZM2G005791	5:213047055-213053537	+
GRMZM2G018103	5:213330139-213332966	-
GRMZM2G027282	5:214382241-214386531	-
GRMZM2G150912	5:215060200-215065390	-
GRMZM2G013890	5:216547625-216554815	-
GRMZM2G014793	5:216591218-216598666	+
GRMZM2G145107	5:216637674-216642140	+
GRMZM2G133919	5:216749557-216756161	-
GRMZM2G552322	6:2427497-2432408	+
GRMZM2G061186	6:8382834-8403391	+
GRMZM2G164916	6:55760239-55764314	-
GRMZM2G164352	6:64676766-64683561	+
GRMZM2G110983	6:66362127-66366301	-
GRMZM2G047204	6:74232836-74235724	+
GRMZM2G017421	6:80796527-80806200	+
GRMZM2G055413	6:83866787-83869911	+
GRMZM2G044398	6:89844833-89855828	+
GRMZM2G082322	6:89986353-89991104	+
GRMZM2G087850	6:93163137-93169430	-
GRMZM2G131020	6:97274324-97277766	+
GRMZM2G105398	6:102986994-102994277	+

GRMZM2G422938	2:61396278-61400527	-	GRMZM2G181151	6:104274311-104281719	+
GRMZM2G023020	2:63554418-63558011	-	GRMZM2G109009	6:104883752-104890034	-
GRMZM2G007871	2:82436798-82448704	-	GRMZM2G142443	6:107222259-107227499	-
GRMZM2G084863	2:84080134-84089339	+	GRMZM2G171488	6:109264696-109274470	-
GRMZM2G162353	2:113485701-113494162	+	GRMZM2G174170	6:121033833-121040632	-
GRMZM2G003692	2:118693753-118711201	+	GRMZM2G558539	6:127957401-127970074	-
GRMZM2G056695	2:121282812-121297510	+	GRMZM2G113156	6:140068111-140076698	+
GRMZM2G451357	2:123554583-123575961	-	GRMZM2G427337	6:144308806-144318825	+
GRMZM2G087105	2:131581090-131587451	+	GRMZM2G100946	6:145086063-145097609	-
GRMZM2G095392	2:147669328-147674252	-	GRMZM2G106140	6:149661860-149667791	-
GRMZM2G101867	2:148341982-148350516	-	GRMZM2G053610	6:151395055-151402799	+
GRMZM2G017941	2:150652796-150657061	+	GRMZM2G459063	6:155429983-155433325	+
GRMZM2G146267	2:154150352-154158967	-	GRMZM2G368448	6:157939551-157953826	-
GRMZM2G180691	2:154590456-154602373	-	GRMZM2G049091	6:158082951-158087562	-
GRMZM2G158575	2:162230925-162242868	+	GRMZM2G120173	6:158598542-158604741	+
GRMZM5G872997	2:168859120-168862276	-	GRMZM2G114513	6:162531782-162538263	-
GRMZM2G138178	2:174739894-174753703	-	GRMZM2G085849	6:164035491-164040532	-
GRMZM2G111204	2:175320204-175326578	-	GRMZM2G163888	6:166995641-167004294	+
GRMZM2G140156	2:184041779-184053401	-	AC233870.1_FG006	6:167207086-167219697	+
GRMZM2G013892	2:192310577-192313813	-	GRMZM2G019200	7:1949666-1957643	-
GRMZM5G866100	2:192615850-192623898	-	GRMZM5G845755	7:2138862-2144213	-
GRMZM2G703303	2:193965710-193969586	+	GRMZM2G480002	7:4543316-4550902	+
GRMZM2G022921	2:194058715-194063798	+	GRMZM2G018508	7:7153306-7174594	-
GRMZM2G023080	2:194066330-194069720	-	GRMZM2G074479	7:8056369-8065662	-
GRMZM2G155337	2:195554938-195560968	+	GRMZM2G074323	7:8066461-8071872	-
GRMZM2G058105	2:196700576-196747432	+	GRMZM2G164265	7:8573470-8579079	+
GRMZM2G114107	2:197056811-197062437	-	GRMZM2G458665	7:15342731-15350796	-
GRMZM2G081812	2:199236109-199241879	-	GRMZM2G144618	7:26243246-26250203	-
GRMZM2G004619	2:199378455-199380799	-	GRMZM2G141941	7:35622159-35625677	-
GRMZM5G836167	2:201830499-201832320	+	GRMZM2G034288	7:38217966-38231106	+
GRMZM2G163444	2:202271026-202273953	+	GRMZM2G173777	7:41183896-41191521	+
GRMZM2G179505	2:202929976-202935840	-	GRMZM2G048257	7:51666557-51669114	-
GRMZM2G480809	2:202939269-202954181	-	GRMZM2G009575	7:69803681-69808004	-
GRMZM2G099487	2:204970958-204979197	+	GRMZM2G470740	7:76599842-76604526	+
GRMZM5G821988	2:205354963-205357746	+	GRMZM2G056501	7:82030845-82034276	+
GRMZM2G039251	2:207695919-207702116	+	GRMZM2G173943	7:83291537-83295947	+
GRMZM2G130854	2:208504961-208511327	-	GRMZM2G115670	7:93403615-93412635	-
GRMZM2G113696	2:209954275-209957586	-	GRMZM2G085630	7:96195462-96202971	+
GRMZM2G464000	2:210487633-210500912	-	GRMZM2G108546	7:101287600-101294529	-
GRMZM2G412888	2:211499139-211504940	+	GRMZM2G061620	7:109749098-109756329	+
GRMZM2G077823	2:213013815-213018729	+	GRMZM2G071059	7:115159138-115160870	+
GRMZM2G060160	2:214616932-214622028	-	GRMZM5G824944	7:123509910-123514398	+
GRMZM2G040209	2:214723508-214726970	+	GRMZM2G140614	7:127894067-127898930	-
GRMZM5G871572	2:214903809-214909197	+	GRMZM2G103902	7:133392694-133400249	+
GRMZM2G059015	2:215168964-215174603	+	AC234154.1_FG007	7:138762094-138766933	-
GRMZM2G035985	2:215498779-215518573	+	GRMZM2G323912	7:141038459-141045540	+
GRMZM2G151905	2:219017501-219029746	+	GRMZM2G162167	7:144693522-144698077	-
GRMZM2G072339	2:220570584-220580827	+	GRMZM2G168913	7:145668595-145677919	-
GRMZM2G026311	2:221562565-221565517	-	GRMZM2G316191	7:150195961-150215431	+
GRMZM2G064366	2:225503180-225511481	+	GRMZM2G138230	7:150538439-150548918	+
GRMZM2G037104	2:230276620-230282304	-	GRMZM2G138220	7:150579410-150587614	+
GRMZM2G134027	2:230484457-230492707	+	GRMZM2G165681	7:154495292-154507383	-

GRMZM2G158872	2:231189746-231195706	+
GRMZM2G052403	2:231279315-231287945	-
GRMZM5G864266	2:234678507-234700316	+
GRMZM5G881605	2:236262545-236266748	+
GRMZM2G132468	2:236302854-236310465	-
GRMZM2G048494	2:236620009-236626349	+
GRMZM2G148723	3:1600639-1613076	+
GRMZM2G121452	3:6304666-6313009	-
GRMZM2G166646	3:6927923-6932618	-
GRMZM5G820781	3:7672454-7675155	-
GRMZM2G051458	3:8536278-8540142	+
GRMZM2G083763	3:8877397-8881709	+
GRMZM2G083518	3:9812439-9817566	-
GRMZM2G066440	3:12288829-12295190	+
GRMZM2G150838	3:12576931-12581127	+
GRMZM2G541193	3:17616180-17622799	-
GRMZM2G088834	3:19461658-19470533	+
GRMZM2G373175	3:26787620-26811161	-
GRMZM2G051622	3:29473573-29480102	+
GRMZM2G057150	3:30234360-30239339	-
GRMZM2G049672	3:32025488-32033351	-
GRMZM2G064580	3:38047486-38053581	+
GRMZM2G132465	3:39477481-39480438	+
GRMZM2G157316	3:42543384-42551746	-
GRMZM2G096548	3:45696959-45712949	-
GRMZM2G046924	3:47959820-47966264	+
GRMZM2G322186	3:49620840-49626586	-
GRMZM2G087749	3:62599157-62606372	-
GRMZM2G156251	3:68051300-68060406	+
GRMZM2G009593	3:87809965-87820099	-
GRMZM2G164418	3:89049209-89058000	+
GRMZM2G135599	3:90035264-90038781	+
GRMZM2G018447	3:93201404-93219385	-
GRMZM2G017835	3:95281802-95287312	+
GRMZM2G014805	3:106351200-106368030	+
GRMZM5G826456	3:108436933-108439265	+
GRMZM2G034083	3:128543602-128547650	+
GRMZM2G138077	3:129074219-129076477	-
GRMZM2G122999	3:129245933-129252692	+
GRMZM2G096070	3:140753563-140759990	-
GRMZM2G006806	3:141514940-141517893	+
GRMZM2G061446	3:146407583-146470886	-
GRMZM2G030902	3:151333041-151338416	+
GRMZM2G017404	3:155656712-155661965	-
GRMZM2G092112	3:158481307-158487300	-
GRMZM2G146697	3:161889399-161898813	+
GRMZM2G127756	3:165676096-165685551	+
GRMZM2G357399	3:186634201-186637449	-
GRMZM5G851026	3:191968920-191988742	+
GRMZM2G324886	3:196083502-196088773	+
GRMZM2G027023	3:204370321-204374240	-
GRMZM2G150631	3:210902222-210932732	-
GRMZM2G158062	7:155653647-155658404	+
GRMZM2G019999	7:156059521-156065449	+
GRMZM2G060579	7:158336653-158342207	+
GRMZM2G019084	7:159515313-159576155	+
GRMZM2G384327	7:160062430-160068019	+
GRMZM2G037444	7:164864122-164868947	+
GRMZM2G009538	7:164988124-164993149	+
GRMZM2G157598	7:165069224-165075065	-
GRMZM2G079226	7:166186888-166192016	-
GRMZM2G025281	7:166719378-166725729	+
GRMZM2G037177	7:169878080-169881096	-
GRMZM2G111672	7:170460497-170462364	-
GRMZM2G148213	7:170591410-170601470	-
GRMZM2G106790	7:173448889-173462476	+
GRMZM2G342386	7:174487835-174495553	-
GRMZM2G052875	8:2052722-2060836	+
GRMZM2G056145	8:6804092-6812742	+
GRMZM2G334457	8:8119643-8130134	-
GRMZM2G126860	8:13598283-13603883	-
GRMZM2G080722	8:17875265-17879910	+
GRMZM2G032190	8:18612393-18615853	+
GRMZM2G412234	8:19554612-19560661	-
GRMZM2G069816	8:23965656-23970068	-
GRMZM2G035395	8:43074194-43078030	+
GRMZM2G048611	8:61516605-61524895	-
GRMZM2G109651	8:62492747-62520417	+
GRMZM2G054247	8:64164837-64167645	+
GRMZM2G045818	8:64759364-64762065	-
GRMZM2G021331	8:65484575-65488309	+
GRMZM2G101042	8:65656733-65662011	-
GRMZM2G043764	8:67233891-67245255	+
GRMZM2G150484	8:74040042-74050277	+
GRMZM2G350628	8:77707097-77711218	-
GRMZM2G053261	8:77927134-77932299	-
GRMZM2G131249	8:82917060-82921065	+
GRMZM2G132633	8:84665739-84671114	-
GRMZM2G002130	8:89870869-89874340	-
GRMZM2G102745	8:93304359-93311331	+
GRMZM2G158918	8:102976534-102982864	-
GRMZM2G098227	8:107560854-107581415	-
GRMZM2G117388	8:108530756-108538547	-
GRMZM2G149286	8:113926527-113943334	+
GRMZM2G038401	8:115783521-115792131	+
GRMZM2G062555	8:119542661-119544845	-
GRMZM2G060213	8:121936384-121946840	-
GRMZM2G089406	8:130868798-130875685	+
GRMZM2G474658	8:132053632-132060698	+
GRMZM2G067985	8:134054893-134065785	+
GRMZM2G363460	8:134991206-134995085	-
GRMZM2G077125	8:136502550-136507547	-
GRMZM2G003734	8:137305634-137329952	-
GRMZM2G096764	8:138677873-138685412	-

GRMZM2G422651	3:211227556-211229856	-
GRMZM2G088397	3:215288396-215292950	-
GRMZM2G105019	3:217545398-217552377	-
GRMZM2G058039	3:218107555-218113303	+
GRMZM2G463032	3:219772224-219780093	+
GRMZM2G036720	3:220829360-220837678	-
GRMZM2G052088	3:221368481-221372574	-
GRMZM2G019386	3:221696366-221699588	-
GRMZM2G366935	3:221761935-221766913	+
GRMZM2G034943	3:223392262-223395012	+
GRMZM2G022383	3:224095432-224109221	-
GRMZM2G174335	3:226238439-226242286	-
GRMZM2G078826	3:226564458-226569877	+
GRMZM2G064031	3:227133973-227141739	+
GRMZM2G035785	3:227665638-227674344	+
GRMZM2G400238	3:229284068-229298779	+
GRMZM2G011980	3:230774085-230778420	-
GRMZM5G899349	3:232159860-232163704	+
GRMZM2G126453	4:2758911-2766621	+
GRMZM2G104833	4:10771404-10781735	+
GRMZM2G176698	4:14130242-14144638	+
GRMZM2G173354	4:14401180-14408179	-
GRMZM2G151050	4:14953389-14973375	-
GRMZM2G165137	4:25686127-25690698	-
GRMZM2G449355	4:29222249-29240912	-
GRMZM2G055520	4:32811849-32818488	+
GRMZM2G104907	4:33143077-33148731	+
GRMZM5G870572	4:33727744-33735419	-
GRMZM2G103430	4:38214151-38217822	-
GRMZM2G162079	4:38324526-38330896	+
GRMZM2G064807	4:38968162-38971996	+
GRMZM2G005737	4:39360976-39365209	+
GRMZM2G078283	4:48570462-48572773	-
GRMZM2G032367	4:53700603-53704302	-
GRMZM2G098496	4:54627784-54636639	-
GRMZM5G838910	4:64520331-64544078	+
GRMZM2G146118	4:72971101-72978764	+
GRMZM2G023008	4:74229118-74238078	+
GRMZM2G132857	4:77899326-77905901	+
GRMZM2G045944	4:80193031-80200225	+
GRMZM2G095552	4:82802193-82807142	-
GRMZM2G125668	4:103129613-103157428	-
GRMZM2G082642	4:116725155-116732172	-
GRMZM2G149138	4:119723908-119727226	-
GRMZM2G022694	4:121863937-121865666	-
GRMZM2G026043	4:128873877-128884598	+
GRMZM2G020653	4:129765820-129768530	-
GRMZM2G063792	4:131279153-131282696	-
GRMZM2G072690	4:133480125-133485253	+
GRMZM2G030125	4:135339429-135342026	-
GRMZM2G041842	4:137595241-137619272	-
GRMZM2G168337	4:167219964-167225717	-

GRMZM2G086236	8:139699018-139705863	+
GRMZM2G028190	8:140743143-140758475	+
AC194341.4_FG002	8:140927427-140929545	-
GRMZM2G164185	8:141051358-141058594	+
GRMZM2G425377	8:141706193-141711698	-
GRMZM2G157589	8:142201711-142206667	-
GRMZM2G064336	8:148049588-148054134	-
GRMZM2G076827	8:150721874-150727007	+
GRMZM2G436835	8:153128150-153133910	+
GRMZM2G067601	8:156263468-156265092	-
GRMZM2G060977	8:158622298-158629215	+
GRMZM2G180335	8:161293943-161303399	+
GRMZM5G812921	8:162011366-162016883	+
GRMZM2G166855	8:162399396-162409530	-
GRMZM2G421829	8:163632810-163641809	+
GRMZM2G114172	8:165228258-165231972	+
GRMZM2G047214	8:165922184-165927385	+
GRMZM2G046025	8:165960286-165965830	-
GRMZM2G173868	8:171956808-171977242	-
GRMZM2G116086	8:172112725-172121903	+
GRMZM2G153615	8:173705487-173710861	+
GRMZM2G124288	9:4832871-4841388	+
GRMZM2G082198	9:6307757-6313260	-
GRMZM2G080120	9:7661272-7664714	-
GRMZM2G308083	9:8608312-8615327	+
GRMZM2G142072	9:8949490-8962539	+
GRMZM2G467169	9:12108547-12115783	-
GRMZM2G151923	9:12328207-12332219	-
GRMZM2G409865	9:13441353-13452917	+
GRMZM2G029370	9:15375115-15386731	-
GRMZM2G030144	9:16040794-16050905	-
GRMZM2G114098	9:21591970-21597213	-
GRMZM2G122135	9:24155549-24162364	-
GRMZM2G153792	9:28406066-28413513	+
GRMZM2G165418	9:58834504-58858255	+
GRMZM5G814159	9:62411284-62423437	-
GRMZM2G119546	9:82647825-82661556	+
GRMZM5G839512	9:91972448-91976378	+
GRMZM2G087806	9:95033444-95044390	-
GRMZM2G117935	9:95580781-95583611	-
GRMZM2G130905	9:99285720-99289868	+
GRMZM2G108309	9:126595252-126599715	+
GRMZM2G108384	9:128116054-128129443	-
GRMZM2G068479	9:134558710-134563155	-
GRMZM2G090869	9:135333239-135337881	-
GRMZM2G141596	9:141100796-141107494	-
GRMZM2G119627	9:141227222-141235818	-
GRMZM2G550865	9:142243496-142250986	+
GRMZM5G838403	9:147868932-147874956	+
GRMZM2G175463	9:153176980-153187993	+
GRMZM2G060564	9:154003810-154007380	+
GRMZM2G116681	9:154186862-154189335	+

GRMZM2G041799	4:168859416-168862974	-		GRMZM2G092741	9:154674136-154679245	-
GRMZM2G113950	4:170273726-170278146	-		GRMZM2G168257	9:155724469-155725855	-
GRMZM2G143854	4:170493126-170497485	-		GRMZM2G048067	10:1411349-1423184	-
GRMZM2G142806	4:171392302-171396812	+		GRMZM2G036685	10:4561180-4568012	-
GRMZM2G178919	4:173344237-173347777	-		GRMZM2G118316	10:9366054-9382922	+
GRMZM2G181359	4:175765125-175769372	+		GRMZM2G478370	10:63825283-63828614	+
GRMZM2G170313	4:178486058-178499312	+		GRMZM2G103287	10:70234927-70240656	-
GRMZM2G108919	4:180219231-180226844	+		GRMZM2G102231	10:70586575-70590956	-
GRMZM2G111720	4:183571349-183581535	-		GRMZM2G022359	10:73140439-73143884	+
GRMZM2G044862	4:184714280-184721026	+		GRMZM5G806309	10:79153483-79157093	-
AC217271.3_FG002	4:187885985-187888435	-		GRMZM2G097289	10:89218737-89223927	-
GRMZM2G100913	4:194173149-194189344	+		GRMZM2G083459	10:90776034-90781199	+
GRMZM2G084406	4:195222279-195238872	+		GRMZM2G000710	10:95619012-95634688	+
GRMZM2G079471	4:195504059-195509361	−		GRMZM2G159330	10:101047761-10106551	-
GRMZM2G125596	4:195559254-195562953	+		GRMZM2G052166	10:101748275-10175153	+
GRMZM2G363038	4:195710028-195718292	+		GRMZM2G035899	10:102301982-10230936	-
GRMZM2G163726	4:195960621-195964652	+		GRMZM2G009346	10:105809033-10581096	+
GRMZM2G014004	4:196691429-196723656	−		GRMZM2G028945	10:107779958-10778470	+
GRMZM2G119930	4:201019775-201024443	−		GRMZM2G159720	10:109729128-10973306	+
GRMZM2G012416	4:204229309-204239513	+		GRMZM2G164663	10:114508292-11451472	+
GRMZM5G873194	4:205862659-205863398	+		GRMZM2G050647	10:115945143-11595540	+
GRMZM2G139760	4:208030163-208035544	−		GRMZM2G010323	10:117919166-11792369	+
GRMZM2G148913	4:218445184-218457848	−		GRMZM2G139419	10:118167728-11817925	-
GRMZM2G071010	4:221022433-221025423	+		GRMZM2G057437	10:123696238-12370448	+
GRMZM2G027378	4:221041836-221045285	+		GRMZM2G065292	10:125146341-12515173	+
GRMZM2G412899	4:222770046-222777371	−		GRMZM2G126665	10:134056984-13405925	+
GRMZM2G132653	4:224304276-224313237	+		AC233979.1_FG009	10:135188729-13519385	+
GRMZM2G007854	4:228239151-228289180	−		GRMZM2G159389	10:139769106-13977192	-
GRMZM2G121790	4:229199657-229204882	−		GRMZM2G109753	10:140355566-14042671	+
GRMZM2G107362	4:232013668-232019150	+		GRMZM2G148633	10:142931727-14293558	+
GRMZM2G064328	4:234391361-234395800	−		GRMZM2G150262	10:147037098-14704163	-
GRMZM2G040320	4:234867575-234878958	+		GRMZM2G173636	10:147950488-14795312	-
GRMZM2G433591	4:235745133-235750283	+		GRMZM2G110869	10:148738549-14874116	-

Supplemental File 3. The information of lncRNAs in maize (genome B73_RefGen_v3)

Gene_ID	Chr:Start-End	Strand	Gene_ID	Chr:Start-End	Strand
GRMZM2G374777	1:997921-998660	+	GRMZM2G159364	5:48000850-48003699	-
GRMZM5G855560	1:2101609-2102312	+	GRMZM2G017730	5:50755927-50759804	+
GRMZM2G025552	1:5625359-5626038	-	XLOC_033309	5:51209927-51210470	-
XLOC_003242	1:6186211-6186625	-	XLOC_033310	5:52116185-52116476	+
GRMZM2G574383	1:12421891-12422543	-	XLOC_033311	5:52117034-52117317	+
XLOC_033164	1:12593975-12594658	+	XLOC_033312	5:59751457-59753093	-
XLOC_033165	1:12594803-12595782	+	GRMZM2G152111	5:62225546-62226567	+
GRMZM2G541687	1:13209114-13211766	+	XLOC_025775	5:63568837-63569443	-
XLOC_033166	1:17320573-17322042	+	XLOC_033313	5:66697394-66697761	+
XLOC_033167	1:22491983-22492495	+	XLOC_033314	5:74361549-74363401	-
GRMZM5G812298	1:24792851-24793115	+	XLOC_033315	5:74449242-74449531	+
XLOC_033168	1:30817691-30818026	-	GRMZM2G318418	5:74693809-74694314	-
XLOC_033169	1:33164295-33165464	+	GRMZM5G824062	5:76463679-76464347	+
XLOC_003592	1:34815724-34816026	-	XLOC_033316	5:79677941-79679769	+
XLOC_003625	1:36995393-36997756	-	GRMZM2G005562	5:82591152-82592214	-
XLOC_033170	1:40097491-40098182	+	XLOC_033317	5:84381345-84381680	-
XLOC_033171	1:40098342-40099121	+	XLOC_023614	5:84542290-84542665	+
XLOC_033172	1:40759387-40760233	+	XLOC_033318	5:87400376-87400728	+
XLOC_000646	1:43673381-43674239	+	XLOC_033319	5:87408042-87408307	+
GRMZM2G010545	1:46847568-46848543	-	XLOC_033320	5:90463733-90464157	+
XLOC_033173	1:55469718-55470638	-	XLOC_033321	5:91010640-91012672	+
XLOC_000881	1:63049108-63050206	+	XLOC_033322	5:92393623-92406122	-
GRMZM2G703598	1:63543792-63544679	-	XLOC_033323	5:94295470-94295870	+
GRMZM2G505545	1:63611800-63612031	+	XLOC_033324	5:98418799-98419197	-
XLOC_033174	1:75583491-75584051	-	XLOC_033325	5:111465048-111466044	+
GRMZM2G147562	1:88025591-88025852	+	XLOC_033326	5:112333479-112336965	-
XLOC_033175	1:88167980-88168437	+	XLOC_033327	5:114283479-114286761	-
XLOC_001125	1:88169155-88170705	+	XLOC_033328	5:116752107-116752765	+
GRMZM2G034383	1:97676755-97677184	-	XLOC_026143	5:124308258-124309756	-
GRMZM2G047781	1:102095624-102097032	-	XLOC_033329	5:128286923-128287334	-
GRMZM2G025303	1:104813360-104816858	-	XLOC_033330	5:128361566-128362078	-
GRMZM2G166979	1:107016280-107018776	+	XLOC_033331	5:142050067-142050525	-
XLOC_033176	1:119149274-119149749	-	XLOC_023874	5:144105862-144107142	+
XLOC_033177	1:121627147-121627885	+	XLOC_033332	5:150113495-150114022	-
GRMZM2G022804	1:123262187-123263712	+	XLOC_033333	5:152878772-152879082	+
XLOC_033178	1:132154492-132154765	-	XLOC_023963	5:158965217-158965793	+
XLOC_004364	1:137254060-137256498	-	XLOC_033334	5:161785512-161786208	+
GRMZM2G157127	1:140969721-140971208	+	GRMZM2G701985	5:168007900-168011168	+
GRMZM5G819500	1:148373492-148374331	-	XLOC_033335	5:169805524-169805765	-
XLOC_033179	1:148871474-148873131	-	XLOC_033336	5:172147906-172148214	-
XLOC_033180	1:149605142-149606012	-	XLOC_033337	5:180475364-180477767	-
XLOC_033181	1:154775351-154779576	-	GRMZM2G117108	5:182816312-182816922	+
XLOC_033182	1:159400149-159400466	+	XLOC_026652	5:184873514-184873862	-
XLOC_033183	1:165584881-165585520	+	XLOC_033338	5:190428131-190428659	-
GRMZM2G142660	1:166735296-166736510	-	XLOC_024450	5:198330528-198331282	+
GRMZM2G059064	1:179747289-179747944	+	GRMZM2G399921	5:203454140-203454857	-
XLOC_033184	1:180330568-180331612	+	XLOC_033339	5:206660463-206660769	+
XLOC_033185	1:182390956-182391443	-	XLOC_033340	5:210045472-210045736	-
XLOC_033186	1:203501196-203502594	+	XLOC_033341	5:210045931-210046395	-
XLOC_033187	1:206377262-206387123	-	XLOC_024784	5:213019629-213021259	+

XLOC_033188	1:206481361-206481747	-	GRMZM2G018573	5:213304572-213305336	-
GRMZM2G576564	1:210087789-210088271	-	XLOC_027139	5:214129148-214130566	-
XLOC_005057	1:220257292-220257496	-	GRMZM2G180435	5:214130208-214130510	-
GRMZM2G063679	1:224584708-224588276	-	XLOC_029083	6:353327-355496	-
XLOC_033189	1:226991599-226993999	+	GRMZM2G073495	6:3055040-3055778	-
XLOC_033190	1:246275414-246276893	-	XLOC_033342	6:6619772-6620031	-
XLOC_033191	1:246470220-246470655	+	XLOC_033343	6:13448272-13451444	+
GRMZM5G840740	1:248480929-248481202	-	GRMZM2G046416	6:13707874-13708107	-
XLOC_005447	1:253629882-253630810	-	XLOC_033344	6:14407321-14407906	-
GRMZM2G068024	1:258429349-258430453	-	XLOC_033345	6:15136809-15139006	+
GRMZM2G044569	1:268943446-268944049	+	GRMZM2G056038	6:16155614-16157482	-
GRMZM2G000197	1:280925191-280925458	-	GRMZM5G856609	6:19986970-19987199	+
XLOC_033192	1:288802506-288803080	+	XLOC_033346	6:20230305-20230885	-
XLOC_033193	1:290383251-290388171	-	XLOC_033347	6:20962814-20963340	+
GRMZM2G148405	1:293362558-293362820	+	GRMZM2G060373	6:22305061-22306508	-
GRMZM2G366681	1:296067675-296069043	-	GRMZM2G123923	6:26577780-26578192	+
XLOC_033194	1:296301600-296302055	+	XLOC_033348	6:35705142-35705505	+
XLOC_033195	1:297266826-297267328	+	GRMZM5G844094	6:38115138-38115838	-
XLOC_033196	1:299665408-299666717	+	GRMZM2G110378	6:41397425-41398185	-
XLOC_006164	1:299700425-299700803	-	XLOC_033349	6:49557682-49559179	-
XLOC_003122	1:301223563-301225573	+	XLOC_033350	6:51862159-51862959	+
GRMZM2G009653	2:3702442-3703089	-	XLOC_033351	6:52741295-52742281	+
XLOC_033197	2:4208882-4209565	+	XLOC_033352	6:54053802-54054887	-
XLOC_033198	2:4621529-4621899	+	XLOC_027717	6:59947232-59948050	+
XLOC_033199	2:5086794-5087076	-	XLOC_033353	6:64125317-64125902	-
GRMZM5G866678	2:6364802-6365236	-	XLOC_029647	6:68072935-68074443	-
XLOC_011655	2:9430139-9430580	-	GRMZM5G816887	6:68424131-68424364	-
XLOC_033200	2:13549878-13550237	+	XLOC_027778	6:68651087-68651305	+
XLOC_033201	2:15038521-15039371	+	XLOC_033354	6:71890343-71891669	-
GRMZM2G175528	2:18735771-18735972	+	XLOC_033355	6:72694891-72695555	+
XLOC_033202	2:19867412-19867760	+	GRMZM2G700969	6:78787861-78790727	+
XLOC_033203	2:25493841-25495674	-	XLOC_029713	6:79845269-79848227	-
GRMZM2G176677	2:27059443-27059977	+	GRMZM2G356338	6:79847300-79848153	-
XLOC_009552	2:28672250-28672735	+	XLOC_033356	6:84447729-84448280	+
GRMZM2G110279	2:41514834-41516623	+	GRMZM2G543070	6:90207578-90208583	-
XLOC_033204	2:41858435-41859024	-	GRMZM2G094074	6:90475244-90475658	-
XLOC_033205	2:45846402-45847056	-	XLOC_033357	6:91522678-91522986	-
XLOC_033206	2:46724265-46725082	+	GRMZM2G061465	6:94582711-94585301	+
GRMZM2G578489	2:48281582-48281963	+	XLOC_033358	6:98082148-98083420	+
XLOC_033207	2:54908481-54908770	+	XLOC_033359	6:105533763-105535206	-
GRMZM2G148198	2:55107843-55110270	-	XLOC_033360	6:111313741-111314026	-
XLOC_012265	2:55376948-55377359	-	XLOC_033361	6:113968377-113968789	-
XLOC_033208	2:55382076-55382322	-	GRMZM2G443881	6:119036750-119037231	-
GRMZM2G138786	2:56446660-56446907	+	XLOC_033362	6:124985312-124985726	+
XLOC_009921	2:66528172-66529419	+	XLOC_030190	6:127444852-127446677	-
XLOC_033209	2:70743804-70745517	-	GRMZM5G837137	6:137587872-137588158	+
XLOC_033210	2:75891085-75891684	+	XLOC_028522	6:141138293-141139251	+
XLOC_033211	2:84577439-84578539	+	XLOC_033363	6:142946538-142947014	+
XLOC_033212	2:86666859-86668522	+	XLOC_033364	6:143290735-143291009	-
XLOC_012448	2:88036682-88036960	-	GRMZM2G381318	6:146413281-146414549	-
XLOC_012453	2:88980184-88981043	-	XLOC_033365	6:147737952-147738788	-
XLOC_033213	2:93848090-93848514	+	GRMZM2G000816	6:149867816-149868428	+

GRMZM2G455290	2:98702976-98704359	+
XLOC_033214	2:98904616-98906898	-
GRMZM2G065979	2:100526136-100526457	+
XLOC_033215	2:102646330-102646767	-
XLOC_033216	2:104284865-104285116	-
XLOC_033217	2:109214433-109215117	-
GRMZM2G021223	2:111766444-111766785	+
GRMZM2G119081	2:112456009-112460044	+
XLOC_010127	2:113760488-113760897	+
XLOC_033218	2:116949560-116949929	-
XLOC_033219	2:120540977-120541228	+
GRMZM2G118817	2:127211753-127212121	+
XLOC_012651	2:129008866-129009538	-
XLOC_033220	2:132331371-132331800	+
GRMZM2G565940	2:132746641-132747026	-
XLOC_033221	2:135135154-135135508	+
XLOC_010328	2:154071730-154072606	+
XLOC_033222	2:171427185-171427432	+
XLOC_033223	2:171523665-171524505	-
GRMZM5G892032	2:174284306-174284917	-
XLOC_012974	2:177286710-177287568	-
GRMZM2G397297	2:178139888-178145144	-
XLOC_010585	2:185129762-185130100	+
XLOC_033224	2:188511784-188512918	-
GRMZM2G047777	2:189632630-189633471	-
XLOC_033225	2:190424355-190425158	-
XLOC_033226	2:203006203-203006740	+
GRMZM2G026672	2:204166886-204168162	+
GRMZM5G808394	2:209157030-209157254	+
XLOC_033227	2:211554325-211555237	-
XLOC_033228	2:215520781-215521382	-
GRMZM2G010551	2:217269770-217270535	+
XLOC_013560	2:220470324-220470895	-
GRMZM5G843434	2:221314239-221315150	+
XLOC_011222	2:223726754-223727023	+
XLOC_033229	2:223727207-223727586	+
XLOC_033230	2:224308826-224309452	-
GRMZM2G003115	2:224641455-224644387	-
GRMZM5G862594	2:224808077-224809444	+
XLOC_033231	2:227580129-227581315	+
GRMZM2G078523	2:229440532-229440971	+
XLOC_033232	2:232594210-232595585	-
XLOC_033233	2:233049567-233049815	+
XLOC_033234	2:236853735-236854453	+
XLOC_033235	3:219862-220632	+
XLOC_033236	3:297552-297898	-
GRMZM2G383240	3:372723-373328	-
XLOC_033237	3:1882830-1883826	-
XLOC_016091	3:3840232-3843762	-
XLOC_033238	3:10465807-10466551	-
XLOC_016264	3:15721444-15723939	-
GRMZM2G380195	3:18016367-18018478	-
GRMZM2G088349	6:152679335-152680521	-
GRMZM2G140719	6:155802308-155802611	+
XLOC_033366	6:155931739-155932318	-
GRMZM2G389903	6:157238411-157238975	+
XLOC_028861	6:159968118-159969476	+
GRMZM2G060726	6:160813441-160814870	-
GRMZM2G170400	6:162503096-162503725	-
XLOC_028963	6:163600929-163601534	+
GRMZM2G531385	6:164054107-164054646	-
XLOC_029011	6:165520305-165520546	+
XLOC_033367	6:166829539-166830229	-
XLOC_029079	6:169313458-169313771	+
GRMZM2G101545	7:2576253-2577844	+
XLOC_033368	7:2725062-2725850	-
GRMZM2G117281	7:2744692-2746114	+
XLOC_030982	7:3086101-3087278	+
GRMZM2G002910	7:6422635-6425638	+
GRMZM2G144645	7:8269424-8270323	+
XLOC_033369	7:8998090-8998922	+
GRMZM2G499381	7:10558396-10559801	-
XLOC_033370	7:15427352-15427665	+
GRMZM2G122810	7:18150408-18150977	-
GRMZM2G141941	7:35624286-35625677	-
XLOC_033371	7:40616090-40616322	-
XLOC_033372	7:56085640-56085883	-
XLOC_033373	7:57967878-57969228	-
XLOC_031394	7:63865890-63866424	+
GRMZM2G312838	7:64600625-64601593	+
XLOC_031403	7:68159312-68159784	+
XLOC_031425	7:74908575-74909111	+
XLOC_033000	7:75678733-75679967	-
XLOC_033374	7:85371064-85372608	+
XLOC_033375	7:87758026-87759926	+
XLOC_033376	7:96004436-96004913	+
XLOC_033377	7:97808833-97809377	+
XLOC_033378	7:109977610-109977863	+
XLOC_033379	7:111655328-111655709	-
XLOC_033380	7:112441571-112442342	-
XLOC_033381	7:112447459-112447751	-
GRMZM2G125853	7:116001617-116002590	+
GRMZM2G146731	7:117818204-117819076	+
XLOC_031681	7:121487140-121488153	+
GRMZM2G039036	7:122013290-122014126	+
XLOC_031692	7:122098912-122099313	+
XLOC_033382	7:130937689-130938060	+
XLOC_031796	7:132429789-132430208	+
XLOC_033383	7:137122240-137123173	+
GRMZM2G104504	7:143253123-143253359	+
GRMZM5G858838	7:144343264-144343743	+
GRMZM2G568454	7:145339299-145339531	-
XLOC_033384	7:145794592-145796184	-
XLOC_033385	7:146617192-146619058	+

XLOC_014106	3:19491106-19491434	+	GRMZM2G133590	7:147545126-147545344	-
XLOC_016369	3:27808902-27809542	-	GRMZM2G088590	7:149799029-149800188	-
XLOC_033239	3:30386504-30386926	+	GRMZM2G148268	7:153221907-153222419	+
XLOC_014319	3:51988113-51988493	+	XLOC_033386	7:155638907-155639184	+
XLOC_033240	3:56291855-56297367	+	XLOC_033387	7:157472816-157473181	-
XLOC_016654	3:71491871-71492178	-	GRMZM5G886969	7:159034533-159038578	-
GRMZM2G154056	3:76125082-76126029	+	XLOC_033799	7:160665238-160666180	-
XLOC_033241	3:78007786-78008683	+	XLOC_033388	7:161360816-161361094	-
XLOC_033242	3:78650794-78651419	+	XLOC_033389	7:169209991-169210625	+
XLOC_033243	3:109375531-109376943	+	GRMZM2G414460	7:172549347-172550332	-
GRMZM2G044503	3:110266800-110269197	-	GRMZM2G042636	8:1374320-1374620	-
XLOC_033244	3:114041996-114042482	-	GRMZM2G157007	8:1830837-1831377	+
XLOC_033245	3:121558564-121560043	+	XLOC_036071	8:7770912-7771591	-
GRMZM2G114924	3:122085032-122085710	+	XLOC_033390	8:21215017-21215612	-
XLOC_033246	3:124071208-124072679	+	XLOC_036247	8:21710340-21712463	-
XLOC_033247	3:131077118-131078796	-	XLOC_033391	8:24894481-24895305	-
GRMZM2G034868	3:142985289-142986336	-	XLOC_033392	8:26459563-26459986	+
GRMZM2G136635	3:146756647-146757488	-	XLOC_033393	8:32885788-32886493	+
XLOC_033248	3:153417694-153418653	+	XLOC_033394	8:34191520-34195837	-
XLOC_033249	3:157085984-157087287	-	GRMZM5G857986	8:34668887-34670371	+
GRMZM5G858444	3:160833299-160834058	-	XLOC_033395	8:44743253-44744257	+
XLOC_017276	3:163236879-163237146	-	XLOC_033396	8:52424493-52424941	+
GRMZM2G004320	3:165014850-165016905	+	XLOC_033397	8:56459399-56460838	-
GRMZM2G346312	3:169360362-169360676	-	XLOC_033398	8:62728268-62728677	+
GRMZM2G498765	3:171079687-171079981	-	GRMZM2G492296	8:65971718-65973228	-
XLOC_017376	3:172422064-172422782	-	XLOC_033399	8:74751577-74752861	+
XLOC_033250	3:176838272-176839146	+	XLOC_033400	8:78697753-78698003	+
GRMZM2G004949	3:179279578-179280027	-	XLOC_033401	8:87272444-87273017	+
XLOC_033251	3:179501037-179501773	+	XLOC_033402	8:90039951-90044800	+
XLOC_033252	3:179849390-179849679	-	GRMZM2G532340	8:90688695-90688923	+
GRMZM2G064853	3:180559786-180560836	+	GRMZM2G074725	8:95238340-95238754	-
GRMZM2G518003	3:181756177-181756437	+	XLOC_033403	8:104212265-104212966	-
XLOC_015306	3:186407878-186409404	+	XLOC_036782	8:105801479-105801771	-
XLOC_033253	3:188723470-188724256	+	GRMZM2G035568	8:106822775-106823065	-
XLOC_033254	3:192388107-192388805	+	XLOC_033404	8:110703023-110703599	+
GRMZM2G016640	3:193374429-193375528	+	XLOC_035017	8:114173178-114173544	+
GRMZM2G111637	3:194884570-194885066	-	XLOC_033405	8:116961406-116961975	-
GRMZM2G021698	3:197395914-197396235	+	GRMZM2G064426	8:117332542-117333893	+
GRMZM2G126853	3:205336019-205336569	-	XLOC_035060	8:118216224-118216557	+
GRMZM2G054715	3:208123839-208124410	-	XLOC_033406	8:119517992-119518410	-
GRMZM5G807953	3:209921037-209921247	-	XLOC_033407	8:123066033-123066565	-
XLOC_033255	3:212580781-212581267	-	XLOC_033408	8:125044315-125044536	+
GRMZM2G047324	3:213855806-213856341	+	XLOC_035178	8:128279985-128280732	+
GRMZM2G061439	3:214453881-214454616	+	GRMZM2G399541	8:128344260-128344929	-
GRMZM2G464976	3:216723740-216724129	-	XLOC_033409	8:130702797-130703629	+
GRMZM2G125392	3:219798107-219798390	-	GRMZM2G474658	8:132053632-132055719	+
XLOC_033256	3:221332372-221333007	-	XLOC_033410	8:145553173-145553644	+
XLOC_033257	3:221778323-221779125	-	GRMZM2G063431	8:146158331-146159609	-
XLOC_033258	3:221916112-221916668	+	XLOC_033411	8:148145999-148146317	+
XLOC_033259	3:222680172-222681070	-	GRMZM2G369485	8:149796612-149797048	+
GRMZM2G001296	3:225283647-225284349	-	GRMZM2G488001	8:150809229-150809881	-
XLOC_033260	3:228166609-228167033	+	XLOC_033412	8:151087202-151087776	-

XLOC_033261	4:2078000-2078786	-
GRMZM2G039630	4:4481664-4482509	+
GRMZM2G133675	4:6601618-6602508	+
XLOC_018485	4:13722834-13723548	+
GRMZM2G089436	4:24936939-24937306	+
XLOC_033262	4:29899360-29899717	+
XLOC_033263	4:34085268-34086340	+
XLOC_033264	4:36868174-36869484	+
XLOC_033265	4:39414137-39415760	+
XLOC_033266	4:39677176-39679837	-
XLOC_018746	4:40704031-40705039	+
XLOC_033267	4:47250446-47251753	-
XLOC_033268	4:47481498-47482252	+
XLOC_033269	4:47482307-47482639	+
XLOC_033270	4:49701570-49702047	-
XLOC_033271	4:57120189-57120546	+
XLOC_033272	4:62243210-62248749	-
XLOC_033273	4:65739577-65741153	+
XLOC_033274	4:67257919-67259268	-
XLOC_033275	4:87906686-87906991	-
XLOC_021118	4:89884182-89884658	-
XLOC_033276	4:90250631-90253219	+
XLOC_033277	4:90880941-90881506	+
XLOC_033278	4:94125037-94126170	+
XLOC_033279	4:120690245-120690542	+
GRMZM2G105579	4:121631603-121635474	-
XLOC_033280	4:122773791-122774483	-
XLOC_033281	4:123727888-123728467	-
XLOC_033282	4:131381485-131381823	+
XLOC_019213	4:134944312-134944871	+
XLOC_033283	4:135829901-135831554	+
GRMZM2G094083	4:139504119-139505316	+
XLOC_033284	4:149188730-149189159	+
XLOC_033285	4:149576668-149577308	+
GRMZM2G146409	4:157139798-157140313	+
XLOC_033286	4:157795711-157796046	+
GRMZM2G085547	4:170257867-170258431	+
GRMZM2G075489	4:170836007-170836759	+
GRMZM2G420571	4:171155233-171156775	+
XLOC_033287	4:172250041-172250629	+
GRMZM5G841309	4:173326034-173327029	+
XLOC_021689	4:176471866-176473114	-
GRMZM2G166005	4:177474467-177476458	+
XLOC_033288	4:181550312-181550926	-
GRMZM2G077299	4:186455306-186455826	-
XLOC_033289	4:191042008-191042628	+
XLOC_033290	4:194790087-194790521	+
XLOC_033291	4:196542456-196546375	-
XLOC_022002	4:198739447-198739673	-
XLOC_033292	4:199405123-199407469	-
GRMZM2G041039	4:207347095-207347776	+
XLOC_033293	4:209997502-209997984	-
XLOC_035541	8:155165425-155165950	+
XLOC_033413	8:159093076-159093544	+
XLOC_033414	8:164098901-164100075	-
XLOC_033415	8:166983298-166983923	+
XLOC_033416	8:168219311-168219575	+
GRMZM2G028054	8:170727993-170728865	-
XLOC_033417	8:173410479-173410865	-
XLOC_033418	9:3575858-3577153	+
GRMZM2G167594	9:11459478-11461317	+
XLOC_033419	9:12816359-12818498	+
XLOC_039528	9:17026350-17026755	-
GRMZM5G869917	9:25605905-25606659	+
XLOC_033420	9:29074395-29074768	+
GRMZM2G156376	9:29880471-29882039	-
XLOC_033421	9:39395949-39396884	+
XLOC_033422	9:42830638-42830839	+
XLOC_033423	9:51109399-51110311	+
XLOC_033424	9:51590057-51590541	+
GRMZM5G894376	9:68986933-68988251	+
XLOC_033425	9:72612859-72613658	-
GRMZM2G497928	9:76062075-76062726	-
XLOC_033426	9:85176147-85178308	-
XLOC_033427	9:88445989-88446893	+
XLOC_038480	9:92911162-92911626	+
XLOC_033428	9:93115351-93115842	-
GRMZM2G302083	9:94821699-94822069	+
XLOC_033429	9:95642011-95642583	-
XLOC_033430	9:100023478-100023923	-
GRMZM2G409101	9:104358303-104358871	+
GRMZM5G836827	9:106901369-106902901	-
XLOC_038600	9:108314021-108314491	+
XLOC_033431	9:108873674-108874370	+
XLOC_033432	9:109319754-109320053	+
XLOC_033433	9:109508115-109509460	-
XLOC_033434	9:109509600-109510032	-
GRMZM2G556522	9:113191569-113192342	+
XLOC_033435	9:113242590-113242979	-
XLOC_033436	9:117920702-117921054	+
XLOC_033437	9:118439673-118440226	-
XLOC_033438	9:119778421-119779136	-
XLOC_033439	9:124308574-124309165	+
XLOC_033440	9:129262568-129267819	-
XLOC_040390	9:131635930-131636261	-
XLOC_033441	9:136815155-136816002	+
GRMZM2G095868	9:138870971-138871608	-
XLOC_033442	9:140012571-140013021	+
XLOC_033443	9:151213231-151213450	-
XLOC_033444	9:152748163-152749797	+
XLOC_006204	10:1426360-1426724	+
GRMZM2G404802	10:1912158-1912456	-
GRMZM2G156134	10:4480822-4481156	+
XLOC_033445	10:6079266-6080416	-

XLOC_033294	4:213749978-213751007	+	XLOC_033446	10:8938428-8938803	-
XLOC_033295	4:213900165-213904576	+	GRMZM2G093950	10:10288365-10288813	+
XLOC_033296	4:227041761-227042712	+	XLOC_033447	10:11183927-11184357	+
XLOC_033297	4:228772969-228775118	-	XLOC_033448	10:17300053-17300258	-
XLOC_033298	4:234988087-234988762	+	XLOC_007874	10:26906386-26907679	-
GRMZM2G485274	4:235112018-235112595	+	XLOC_033449	10:45214113-45214649	-
GRMZM2G018837	4:235806854-235807502	+	XLOC_007972	10:51792935-51793371	-
XLOC_020302	4:237186859-237187906	+	XLOC_033450	10:61514870-61515341	+
GRMZM2G178100	4:239202184-239202811	+	GRMZM2G074489	10:65496723-65501619	-
GRMZM2G163247	4:241012486-241013033	-	XLOC_033451	10:65932989-65934374	+
XLOC_033299	4:241900973-241902583	-	GRMZM2G141136	10:72621787-72623231	+
XLOC_024900	5:345298-346269	-	GRMZM2G101836	10:85207958-85208533	+
GRMZM2G524840	5:1326554-1326883	+	XLOC_033452	10:89120674-89121925	-
XLOC_033300	5:1955343-1955600	-	XLOC_033453	10:91281324-91282017	+
GRMZM5G883250	5:3835979-3836756	+	GRMZM2G347623	10:91794667-91794968	-
XLOC_033301	5:3995063-3996241	+	XLOC_033454	10:94585169-94585647	+
GRMZM2G077088	5:6448091-6448630	+	GRMZM2G084547	10:101729984-10173193	-
GRMZM2G023858	5:8007214-8007618	-	GRMZM5G885862	10:104752909-10475334	+
XLOC_033302	5:9117182-9118738	+	GRMZM2G043737	10:116881112-11688174	+
GRMZM2G177942	5:11495276-11496097	+	GRMZM2G010323	10:117921977-11792369	+
XLOC_022860	5:12392868-12393218	+	GRMZM2G420713	10:120964292-12097202	+
XLOC_025209	5:12557584-12560652	-	XLOC_033455	10:130747641-13074824	-
GRMZM2G026157	5:12557664-12558570	-	XLOC_033456	10:132792494-13279852	+
GRMZM2G010604	5:14199464-14202029	+	XLOC_033457	10:134075962-13407697	-
GRMZM2G073934	5:21594619-21594930	-	XLOC_033458	10:134537756-13453802	-
GRMZM2G026892	5:23284770-23286306	-	XLOC_033459	10:136729702-13673022	-
XLOC_033303	5:23679867-23680140	-	XLOC_033460	10:138211364-13821483	-
XLOC_023075	5:27367961-27368221	+	GRMZM2G555422	10:139382816-13938610	-
XLOC_033304	5:28234052-28234475	-	XLOC_007410	10:143657877-14366005	+
GRMZM2G390804	5:30517313-30517558	-	GRMZM2G427054	10:144168157-14416899	-
XLOC_033305	5:45749971-45750273	-	GRMZM2G008730	10:146161891-14616335	+
XLOC_033306	5:46483019-46484171	+	GRMZM2G150286	10:147043504-14704488	-
XLOC_033307	5:47171252-47174203	+	XLOC_033461	10:148268481-14826888	+
XLOC_033308	5:47396974-47397294	-			

Supplemental File 4. R script to scan genome-wide DNA methylation along genes in sliding windows.

```
library("plyr")

# read regions infor. and split
split.win<-function(x){
    split.list<-list()
    by.len=10;win.len=100
    pindex=(x$strand=="+")
    mindex=(x$strand=="-")
    for(i in 1:100){
        tmp<-x
        tmp[pindex,"end"]<-x[pindex,"start"]-((100-1)*by.len)+by.len*(i-1)-1
        tmp[pindex,"start"]<-tmp[pindex,"end"]-(win.len-1)
        tmp[mindex,"end"]<-x[mindex,"end"]+(win.len+(100-1)*by.len)-by.len*(i-1)
        tmp[mindex,"start"]<-tmp[mindex,"end"]-(win.len-1)
        tmp[tmp$start<=0,"start"]=1
        tmp[tmp$end<=0,"end"]=1
        tmp[tmp$end-tmp$start==99,]->tmp
        split.list[[i]]<-tmp
    }
    split.list
}
#ob1k
split.ob1k<-function(x){
    split.list<-list()
    by.len=10;win.len=100
    pindex=(x$strand=="+")
    mindex=(x$strand=="-")
    for(i in 1:100){
        tmp<-x
        tmp[pindex,"start"]<-tmp[pindex,"end"]+1+by.len*(i-1)
        tmp[pindex,"end"]<-tmp[pindex,"start"]+(win.len-1)

        tmp[mindex,"end"]<-x[mindex,"start"]-1-by.len*(i-1)
        tmp[mindex,"start"]<-tmp[mindex,"end"]-(win.len-1)
        tmp[tmp$start<=0,"start"]=1
        tmp[tmp$end<=0,"end"]=1
        tmp[tmp$end-tmp$start==99,]->tmp
        split.list[[i]]<-tmp
    }
    split.list
}
```

```
#gene
split.gene<-function(x){
    split.list<-list()
    by.len=10;win.len=100
    pindex=(x$strand=="+")
    mindex=(x$strand=="-")
    for(i in 1:100){
        tmp<-x
        tmp[pindex,"start"]<-tmp[pindex,"start"]+by.len*(i-1)
        tmp[pindex,"end"]<-tmp[pindex,"start"]+(win.len-1)
        tmp[pindex & tmp$start>x$end,"start"]<-x[pindex & tmp$start>x$end,"end"]
        tmp[pindex & tmp$end>x$end,"end"]<-x[pindex & tmp$end>x$end,"end"]

        tmp[mindex,"end"]<-x[mindex,"end"]-by.len*(i-1)
        tmp[mindex,"start"]<-tmp[mindex,"end"]-(win.len-1)
        tmp[mindex & tmp$end<x$start,"end"]<-x[mindex    & tmp$end<x$start,"start"]
        tmp[mindex & tmp$start<x$start,"start"]<-x[mindex & tmp$start<x$start,"start"]
        tmp[tmp$end-tmp$start==99,]->tmp
        split.list[[i]]<-tmp
    }
    for(i in 101:200){
        j=i-100
        tmp<-x
        tmp[pindex,"end"]<-tmp[pindex,"end"]-by.len*(j-1)
        tmp[pindex,"start"]<-tmp[pindex,"end"]-(win.len-1)
        tmp[pindex & tmp$start<x$start,"start"]<-x[pindex & tmp$start<x$start,"start"]
        tmp[pindex & tmp$end<x$start,"end"]<-x[pindex & tmp$end<x$start,"start"]

        tmp[mindex,"start"]<-x[mindex,"start"]+by.len*(j-1)
        tmp[mindex,"end"]<-tmp[mindex,"start"]+(win.len-1)
        tmp[mindex & tmp$start>x$end,"start"]<-x[mindex & tmp$start>x$end,"end"]
        tmp[mindex & tmp$end>x$end,"end"]<-x[mindex & tmp$end>x$end,"end"]
        tmp[tmp$end-tmp$start==99,]->tmp
        split.list[[301-i]]<-tmp
    }
    split.list
}

#multi-core
library(doMC)
registerDoMC(15)
## Check how many cores we are using
getDoParWorkers()

#read genes infor.
read.table("~/NAT.v3/STAR/rep.compare/gene.bed",header=F,stringsAsFactors=F)->gene.bed
```

```
colnames(gene.bed)<-c("chr","start","end","id","score","strand")

of1k.break=split.win(gene.bed)
any(sapply(of1k.break,function(x){any(x$start>x$end)}))
names(of1k.break)<-paste("of1k",1:100,sep=".")
ldply(of1k.break,function(x){x})->of1k.bed

win.scan<-function(x,name){
  ldply(x,.parallel = T,function(x){
material=c("P1_WW","P1_WS","P2_WW","P2_WS","H1_WW","H1_WS","H2_WW","H2_WS")
    options(scipen=10)
    filename=tempfile(tmpdir=".")
    write.table(x = x[order(as.character(x[,1]),x[,2]),],file = filename,row.names = F,col.names =
F,sep = "\t",quote = F)
    data<-data.frame()
    for (mat in material){
      system(paste("bedtools coverage -sorted -b ", mat, "/prefect_sorted.bed   -a   ",filename," >
",filename,".out",sep = ""))
      read.table(paste(filename,".out",sep=""),header=F,stringsAsFactors = F)->tmp
      rbind(data,data.frame(mat=mat,id=tmp[,4],num=tmp[,7],len=tmp[,8],stringsAsFactors        =
F))->data
    }
    unlink(filename)
    unlink(paste(filename,".out",sep = ""))
    data
  })->y
  assign(name,y,envir=globalenv())
}

win.scan(of1k.break,"of1k.out")


ob1k.break=split.ob1k(gene.bed)
any(sapply(ob1k.break,function(x){any(x$start>x$end)}))
names(ob1k.break)<-paste("ob1k",1:100,sep=".")
win.scan(ob1k.break,"ob1k.out")


gene.break=split.gene(gene.bed)
any(sapply(gene.break,function(x){any(x$start>x$end)}))
names(gene.break)<-paste("gene",1:200,sep=".")
win.scan(gene.break,"gene.out")

save(of1k.out,ob1k.out, gene.out, file="MACS.win.RData")
```

Supplemental File 5. GTF File of NATs identified in eight samples.

1	Cufflinks	exon	999326	999549	.	+	.	gene_id GRMZM2G374779; transcript_id TCONS_00000019; exon_number 1; oId CUFF.61.2; tss_id TSS16;
1	Cufflinks	exon	999659	999743	.	+	.	gene_id GRMZM2G374779; transcript_id TCONS_00000019; exon_number 2; oId CUFF.61.2; tss_id TSS16;
1	Cufflinks	exon	999845	999896	.	+	.	gene_id GRMZM2G374779; transcript_id TCONS_00000019; exon_number 3; oId CUFF.61.2; tss_id TSS16;
1	Cufflinks	exon	999989	1000009	.	+	.	gene_id GRMZM2G374779; transcript_id TCONS_00000019; exon_number 4; oId CUFF.61.2; tss_id TSS16;
1	Cufflinks	exon	1000087	1000140	.	+	.	gene_id GRMZM2G374779; transcript_id TCONS_00000019; exon_number 5; oId CUFF.61.2; tss_id TSS16;
1	Cufflinks	exon	1000494	1000544	.	+	.	gene_id GRMZM2G374779; transcript_id TCONS_00000019; exon_number 6; oId CUFF.61.2; tss_id TSS16;
1	Cufflinks	exon	1000704	1000766	.	+	.	gene_id GRMZM2G374779; transcript_id TCONS_00000019; exon_number 7; oId CUFF.61.2; tss_id TSS16;
1	Cufflinks	exon	1001050	1001115	.	+	.	gene_id GRMZM2G374779; transcript_id TCONS_00000019; exon_number 8; oId CUFF.61.2; tss_id TSS16;
1	Cufflinks	exon	1001226	1001264	.	+	.	gene_id GRMZM2G374779; transcript_id TCONS_00000019; exon_number 9; oId CUFF.61.2; tss_id TSS16;
1	Cufflinks	exon	1001867	1001959	.	+	.	gene_id GRMZM2G374779; transcript_id TCONS_00000019; exon_number 10; oId CUFF.61.2; tss_id TSS16;
1	Cufflinks	exon	1002038	1003017	.	+	.	gene_id GRMZM2G374779; transcript_id TCONS_00000019; exon_number 11; oId CUFF.61.2; tss_id TSS16;
1	Cufflinks	exon	3462290	3462723	.	+	.	gene_id GRMZM2G055698; transcript_id TCONS_00000071; exon_number 1; oId CUFF.126.1; tss_id TSS65;
1	Cufflinks	exon	3462819	3462982	.	+	.	gene_id GRMZM2G055698; transcript_id TCONS_00000071; exon_number 2; oId CUFF.126.1; tss_id TSS65;
1	Cufflinks	exon	3463071	3463488	.	+	.	gene_id GRMZM2G055698; transcript_id TCONS_00000071; exon_number 3; oId CUFF.126.1; tss_id TSS65;
1	Cufflinks	exon	3463763	3464383	.	+	.	gene_id GRMZM2G055698; transcript_id TCONS_00000071; exon_number 4; oId CUFF.126.1; tss_id TSS65;
1	Cufflinks	exon	5524111	5524748	.	+	.	gene_id GRMZM2G055704; transcript_id TCONS_00000129; exon_number 1; oId CUFF.209.1; tss_id TSS118;
1	Cufflinks	exon	5524910	5524985	.	+	.	gene_id GRMZM2G055704; transcript_id TCONS_00000129; exon_number 2; oId CUFF.209.1; tss_id TSS118;
1	Cufflinks	exon	5525075	5526066	.	+	.	gene_id GRMZM2G055704; transcript_id TCONS_00000129; exon_number 3; oId CUFF.209.1; tss_id TSS118;
1	Cufflinks	exon	8025073	8027145	.	+	.	gene_id GRMZM2G075701; transcript_id TCONS_00000176; exon_number 1; oId CUFF.305.1; tss_id TSS158;
1	Cufflinks	exon	8093438	8094458	.	+	.	gene_id GRMZM2G475168; transcript_id TCONS_00000178; exon_number 1; oId CUFF.313.1; tss_id TSS160;
1	Cufflinks	exon	9117659	9117808	.	+	.	gene_id GRMZM2G041899; transcript_id TCONS_00000205; exon_number 1; oId CUFF.345.1; tss_id TSS182;
1	Cufflinks	exon	9118062	9120601	.	+	.	gene_id GRMZM2G041899; transcript_id TCONS_00000205; exon_number 2; oId CUFF.345.1; tss_id TSS182;
1	Cufflinks	exon	9293832	9294265	.	+	.	gene_id GRMZM5G823004; transcript_id TCONS_00000208; exon_number 1; oId CUFF.349.1; tss_id TSS184;
1	Cufflinks	exon	9295212	9296953	.	+	.	gene_id GRMZM5G823004; transcript_id TCONS_00000208; exon_number 2; oId CUFF.349.1; tss_id TSS184;
1	Cufflinks	exon	10069416	10069763	.	+	.	gene_id XLOC_000189; transcript_id TCONS_00000218; exon_number 1; oId CUFF.366.1; tss_id TSS194;
1	Cufflinks	exon	12096409	12097186	.	+	.	gene_id GRMZM2G000749; transcript_id TCONS_00000259; exon_number 1; oId CUFF.462.2; tss_id TSS227;
1	Cufflinks	exon	12097816	12097971	.	+	.	gene_id GRMZM2G000749; transcript_id TCONS_00000259; exon_number 2; oId CUFF.462.2; tss_id TSS227;
1	Cufflinks	exon	12098509	12098667	.	+	.	gene_id GRMZM2G000749; transcript_id TCONS_00000259; exon_number 3; oId CUFF.462.2; tss_id TSS227;
1	Cufflinks	exon	12098740	12098823	.	+	.	gene_id GRMZM2G000749; transcript_id TCONS_00000259; exon_number 4; oId CUFF.462.2; tss_id TSS227;
1	Cufflinks	exon	12098960	12099039	.	+	.	gene_id GRMZM2G000749; transcript_id TCONS_00000259; exon_number 5; oId CUFF.462.2; tss_id TSS227;
1	Cufflinks	exon	12099145	12099195	.	+	.	gene_id GRMZM2G000749; transcript_id TCONS_00000259; exon_number 6; oId CUFF.462.2; tss_id TSS227;
1	Cufflinks	exon	12099651	12099750	.	+	.	gene_id GRMZM2G000749; transcript_id TCONS_00000259; exon_number 7; oId CUFF.462.2; tss_id TSS227;
1	Cufflinks	exon	12099975	12100057	.	+	.	gene_id GRMZM2G000749; transcript_id TCONS_00000259; exon_number 8; oId CUFF.462.2; tss_id TSS227;

1	Cufflinks	exon	12100190	12100235	.	+	.	gene_id GRMZM2G000749; transcript_id TCONS_00000259; exon_number 9; oId CUFF.462.2; tss_id TSS227;
1	Cufflinks	exon	12100500	12100587	.	+	.	gene_id GRMZM2G000749; transcript_id TCONS_00000259; exon_number 10; oId CUFF.462.2; tss_id TSS227;
1	Cufflinks	exon	12100677	12100846	.	+	.	gene_id GRMZM2G000749; transcript_id TCONS_00000259; exon_number 11; oId CUFF.462.2; tss_id TSS227;
1	Cufflinks	exon	12100956	12101225	.	+	.	gene_id GRMZM2G000749; transcript_id TCONS_00000259; exon_number 12; oId CUFF.462.2; tss_id TSS227;
1	Cufflinks	exon	12101301	12101375	.	+	.	gene_id GRMZM2G000749; transcript_id TCONS_00000259; exon_number 13; oId CUFF.462.2; tss_id TSS227;
1	Cufflinks	exon	12101672	12104425	.	+	.	gene_id GRMZM2G000749; transcript_id TCONS_00000259; exon_number 14; oId CUFF.462.2; tss_id TSS227;
1	Cufflinks	exon	12096409	12097186	.	+	.	gene_id GRMZM2G000749; transcript_id TCONS_00000258; exon_number 1; oId CUFF.462.1; tss_id TSS227;
1	Cufflinks	exon	12097816	12097971	.	+	.	gene_id GRMZM2G000749; transcript_id TCONS_00000258; exon_number 2; oId CUFF.462.1; tss_id TSS227;
1	Cufflinks	exon	12098509	12098667	.	+	.	gene_id GRMZM2G000749; transcript_id TCONS_00000258; exon_number 3; oId CUFF.462.1; tss_id TSS227;
1	Cufflinks	exon	12098740	12098823	.	+	.	gene_id GRMZM2G000749; transcript_id TCONS_00000258; exon_number 4; oId CUFF.462.1; tss_id TSS227;
1	Cufflinks	exon	12098960	12099039	.	+	.	gene_id GRMZM2G000749; transcript_id TCONS_00000258; exon_number 5; oId CUFF.462.1; tss_id TSS227;
1	Cufflinks	exon	12099145	12099195	.	+	.	gene_id GRMZM2G000749; transcript_id TCONS_00000258; exon_number 6; oId CUFF.462.1; tss_id TSS227;
1	Cufflinks	exon	12099651	12099750	.	+	.	gene_id GRMZM2G000749; transcript_id TCONS_00000258; exon_number 7; oId CUFF.462.1; tss_id TSS227;
1	Cufflinks	exon	12099975	12100057	.	+	.	gene_id GRMZM2G000749; transcript_id TCONS_00000258; exon_number 8; oId CUFF.462.1; tss_id TSS227;
1	Cufflinks	exon	12100190	12100235	.	+	.	gene_id GRMZM2G000749; transcript_id TCONS_00000258; exon_number 9; oId CUFF.462.1; tss_id TSS227;
1	Cufflinks	exon	12100500	12100587	.	+	.	gene_id GRMZM2G000749; transcript_id TCONS_00000258; exon_number 10; oId CUFF.462.1; tss_id TSS227;
1	Cufflinks	exon	12100677	12100846	.	+	.	gene_id GRMZM2G000749; transcript_id TCONS_00000258; exon_number 11; oId CUFF.462.1; tss_id TSS227;
1	Cufflinks	exon	12100956	12101225	.	+	.	gene_id GRMZM2G000749; transcript_id TCONS_00000258; exon_number 12; oId CUFF.462.1; tss_id TSS227;
1	Cufflinks	exon	12101301	12101375	.	+	.	gene_id GRMZM2G000749; transcript_id TCONS_00000258; exon_number 13; oId CUFF.462.1; tss_id TSS227;
1	Cufflinks	exon	12103752	12104425	.	+	.	gene_id GRMZM2G000749; transcript_id TCONS_00000258; exon_number 14; oId CUFF.462.1; tss_id TSS227;
1	Cufflinks	exon	14850090	14850603	.	+	.	gene_id GRMZM2G162749; transcript_id TCONS_00000318; exon_number 1; oId CUFF.521.1; tss_id TSS282;
1	Cufflinks	exon	14851486	14853017	.	+	.	gene_id GRMZM2G162749; transcript_id TCONS_00000318; exon_number 2; oId CUFF.521.1; tss_id TSS282;
1	Cufflinks	exon	16467715	16468334	.	+	.	gene_id GRMZM2G175164; transcript_id TCONS_00000344; exon_number 1; oId CUFF.578.1; tss_id TSS307;
1	Cufflinks	exon	16469522	16469657	.	+	.	gene_id GRMZM2G175164; transcript_id TCONS_00000344; exon_number 2; oId CUFF.578.1; tss_id TSS307;
1	Cufflinks	exon	16471600	16471736	.	+	.	gene_id GRMZM2G175164; transcript_id TCONS_00000344; exon_number 3; oId CUFF.578.1; tss_id TSS307;
1	Cufflinks	exon	16472360	16473343	.	+	.	gene_id GRMZM2G175164; transcript_id TCONS_00000344; exon_number 4; oId CUFF.578.1; tss_id TSS307;
1	Cufflinks	exon	17713154	17713691	.	+	.	gene_id GRMZM2G109589; transcript_id TCONS_00000374; exon_number 1; oId CUFF.612.1; tss_id TSS333;
1	Cufflinks	exon	17713856	17713984	.	+	.	gene_id GRMZM2G109589; transcript_id TCONS_00000374; exon_number 2; oId CUFF.612.1; tss_id TSS333;
1	Cufflinks	exon	17714656	17714820	.	+	.	gene_id GRMZM2G109589; transcript_id TCONS_00000374; exon_number 3; oId CUFF.612.1; tss_id TSS333;
1	Cufflinks	exon	17714967	17715272	.	+	.	gene_id GRMZM2G109589; transcript_id TCONS_00000374; exon_number 4; oId CUFF.612.1; tss_id TSS333;
1	Cufflinks	exon	17715369	17715952	.	+	.	gene_id GRMZM2G109589; transcript_id TCONS_00000374; exon_number 5; oId CUFF.612.1; tss_id TSS333;
1	Cufflinks	exon	19098935	19099802	.	+	.	gene_id GRMZM2G006631; transcript_id TCONS_00000396; exon_number 1; oId CUFF.638.1; tss_id TSS354;
1	Cufflinks	exon	19099940	19100356	.	+	.	gene_id GRMZM2G006631; transcript_id TCONS_00000396; exon_number 2; oId CUFF.638.1; tss_id TSS354;
1	Cufflinks	exon	19100611	19101431	.	+	.	gene_id GRMZM2G006631; transcript_id TCONS_00000396; exon_number 3; oId CUFF.638.1; tss_id TSS354;
1	Cufflinks	exon	19327983	19328287	.	+	.	gene_id XLOC_000345; transcript_id TCONS_00000401; exon_number 1; oId CUFF.652.1; tss_id TSS358;
1	Cufflinks	exon	21248981	21250167	.	+	.	gene_id GRMZM2G464515; transcript_id TCONS_00000418; exon_number 1; oId CUFF.686.1; tss_id TSS374;

1	Cufflinks	exon	21250302	21250879	.	+	.	gene_id GRMZM2G464515; transcript_id TCONS_00000418; exon_number 2; oId CUFF.686.1; tss_id TSS374;
1	Cufflinks	exon	22638292	22638569	.	+	.	gene_id GRMZM2G055101; transcript_id TCONS_00000453; exon_number 1; oId CUFF.727.1; tss_id TSS401;
1	Cufflinks	exon	22638724	22638773	.	+	.	gene_id GRMZM2G055101; transcript_id TCONS_00000453; exon_number 2; oId CUFF.727.1; tss_id TSS401;
1	Cufflinks	exon	22640706	22640928	.	+	.	gene_id GRMZM2G055101; transcript_id TCONS_00000453; exon_number 3; oId CUFF.727.1; tss_id TSS401;
1	Cufflinks	exon	22641033	22641196	.	+	.	gene_id GRMZM2G055101; transcript_id TCONS_00000453; exon_number 4; oId CUFF.727.1; tss_id TSS401;
1	Cufflinks	exon	22641342	22642889	.	+	.	gene_id GRMZM2G055101; transcript_id TCONS_00000453; exon_number 5; oId CUFF.727.1; tss_id TSS401;
1	Cufflinks	exon	24403483	24403857	.	+	.	gene_id XLOC_000403; transcript_id TCONS_00000472; exon_number 1; oId CUFF.785.2; tss_id TSS418;
1	Cufflinks	exon	24405310	24407584	.	+	.	gene_id XLOC_000403; transcript_id TCONS_00000472; exon_number 2; oId CUFF.785.2; tss_id TSS418;
1	Cufflinks	exon	27095059	27096692	.	+	.	gene_id GRMZM2G163129; transcript_id TCONS_00000506; exon_number 1; oId CUFF.830.2; tss_id TSS446;
1	Cufflinks	exon	27096807	27097080	.	+	.	gene_id GRMZM2G163129; transcript_id TCONS_00000506; exon_number 2; oId CUFF.830.2; tss_id TSS446;
1	Cufflinks	exon	27097158	27097293	.	+	.	gene_id GRMZM2G163129; transcript_id TCONS_00000506; exon_number 3; oId CUFF.830.2; tss_id TSS446;
1	Cufflinks	exon	27097491	27097701	.	+	.	gene_id GRMZM2G163129; transcript_id TCONS_00000506; exon_number 4; oId CUFF.830.2; tss_id TSS446;
1	Cufflinks	exon	27097789	27097995	.	+	.	gene_id GRMZM2G163129; transcript_id TCONS_00000506; exon_number 5; oId CUFF.830.2; tss_id TSS446;
1	Cufflinks	exon	27098642	27100615	.	+	.	gene_id GRMZM2G163129; transcript_id TCONS_00000506; exon_number 6; oId CUFF.830.2; tss_id TSS446;
1	Cufflinks	exon	27095059	27096692	.	+	.	gene_id GRMZM2G163129; transcript_id TCONS_00000505; exon_number 1; oId CUFF.830.1; tss_id TSS446;
1	Cufflinks	exon	27096807	27097080	.	+	.	gene_id GRMZM2G163129; transcript_id TCONS_00000505; exon_number 2; oId CUFF.830.1; tss_id TSS446;
1	Cufflinks	exon	27097158	27097293	.	+	.	gene_id GRMZM2G163129; transcript_id TCONS_00000505; exon_number 3; oId CUFF.830.1; tss_id TSS446;
1	Cufflinks	exon	27097491	27097701	.	+	.	gene_id GRMZM2G163129; transcript_id TCONS_00000505; exon_number 4; oId CUFF.830.1; tss_id TSS446;
1	Cufflinks	exon	27097789	27097995	.	+	.	gene_id GRMZM2G163129; transcript_id TCONS_00000505; exon_number 5; oId CUFF.830.1; tss_id TSS446;
1	Cufflinks	exon	27098642	27099010	.	+	.	gene_id GRMZM2G163129; transcript_id TCONS_00000505; exon_number 6; oId CUFF.830.1; tss_id TSS446;
1	Cufflinks	exon	27099361	27100615	.	+	.	gene_id GRMZM2G163129; transcript_id TCONS_00000505; exon_number 7; oId CUFF.830.1; tss_id TSS446;
1	Cufflinks	exon	29400571	29401185	.	+	.	gene_id GRMZM2G091456; transcript_id TCONS_00000545; exon_number 1; oId CUFF.882.1; tss_id TSS480;
1	Cufflinks	exon	29402837	29402948	.	+	.	gene_id GRMZM2G091456; transcript_id TCONS_00000545; exon_number 2; oId CUFF.882.1; tss_id TSS480;
1	Cufflinks	exon	29403027	29403201	.	+	.	gene_id GRMZM2G091456; transcript_id TCONS_00000545; exon_number 3; oId CUFF.882.1; tss_id TSS480;
1	Cufflinks	exon	29403530	29403698	.	+	.	gene_id GRMZM2G091456; transcript_id TCONS_00000545; exon_number 4; oId CUFF.882.1; tss_id TSS480;
1	Cufflinks	exon	29403780	29403998	.	+	.	gene_id GRMZM2G091456; transcript_id TCONS_00000545; exon_number 5; oId CUFF.882.1; tss_id TSS480;
1	Cufflinks	exon	29404102	29404377	.	+	.	gene_id GRMZM2G091456; transcript_id TCONS_00000545; exon_number 6; oId CUFF.882.1; tss_id TSS480;
1	Cufflinks	exon	29404456	29404734	.	+	.	gene_id GRMZM2G091456; transcript_id TCONS_00000545; exon_number 7; oId CUFF.882.1; tss_id TSS480;
1	Cufflinks	exon	29405079	29405558	.	+	.	gene_id GRMZM2G091456; transcript_id TCONS_00000545; exon_number 8; oId CUFF.882.1; tss_id TSS480;
1	Cufflinks	exon	36160144	36162528	.	+	.	gene_id AC214040.3_FG009; transcript_id TCONS_00000638; exon_number 1; oId CUFF.1054.1; tss_id TSS568;
1	Cufflinks	exon	40062759	40062923	.	+	.	gene_id GRMZM2G179432; transcript_id TCONS_00000696; exon_number 1; oId CUFF.1139.1; tss_id TSS624;
1	Cufflinks	exon	40063183	40063269	.	+	.	gene_id GRMZM2G179432; transcript_id TCONS_00000696; exon_number 2; oId CUFF.1139.1; tss_id TSS624;
1	Cufflinks	exon	40063409	40063474	.	+	.	gene_id GRMZM2G179432; transcript_id TCONS_00000696; exon_number 3; oId CUFF.1139.1; tss_id TSS624;
1	Cufflinks	exon	40063586	40063683	.	+	.	gene_id GRMZM2G179432; transcript_id TCONS_00000696; exon_number 4; oId CUFF.1139.1; tss_id TSS624;
1	Cufflinks	exon	40063795	40063920	.	+	.	gene_id GRMZM2G179432; transcript_id TCONS_00000696; exon_number 5; oId CUFF.1139.1; tss_id TSS624;
1	Cufflinks	exon	40064002	40064271	.	+	.	gene_id GRMZM2G179432; transcript_id TCONS_00000696; exon_number 6; oId CUFF.1139.1; tss_id TSS624;

1	Cufflinks	exon	40064420	40065030	.	+	.	gene_id GRMZM2G179432; transcript_id TCONS_00000696; exon_number 7; oId CUFF.1139.1; tss_id TSS624;
1	Cufflinks	exon	45370514	45371148	.	+	.	gene_id AC208201.3_FG002; transcript_id TCONS_00000774; exon_number 1; oId CUFF.1271.1; tss_id TSS694;
1	Cufflinks	exon	45371302	45371640	.	+	.	gene_id AC208201.3_FG002; transcript_id TCONS_00000774; exon_number 2; oId CUFF.1271.1; tss_id TSS694;
1	Cufflinks	exon	45371760	45373009	.	+	.	gene_id AC208201.3_FG002; transcript_id TCONS_00000774; exon_number 3; oId CUFF.1271.1; tss_id TSS694;
1	Cufflinks	exon	47508955	47511718	.	+	.	gene_id GRMZM2G377215; transcript_id TCONS_00000821; exon_number 1; oId CUFF.1348.1; tss_id TSS739;
1	Cufflinks	exon	60155734	60157039	.	+	.	gene_id GRMZM2G467072; transcript_id TCONS_00000978; exon_number 1; oId CUFF.1620.1; tss_id TSS881;
1	Cufflinks	exon	62719076	62721689	.	+	.	gene_id GRMZM2G140989; transcript_id TCONS_00001013; exon_number 1; oId CUFF.1683.1; tss_id TSS911;
1	Cufflinks	exon	67777644	67778057	.	+	.	gene_id GRMZM2G179792; transcript_id TCONS_00001099; exon_number 1; oId CUFF.1827.4; tss_id TSS985;
1	Cufflinks	exon	67778152	67778411	.	+	.	gene_id GRMZM2G179792; transcript_id TCONS_00001099; exon_number 2; oId CUFF.1827.4; tss_id TSS985;
1	Cufflinks	exon	67778887	67778946	.	+	.	gene_id GRMZM2G179792; transcript_id TCONS_00001099; exon_number 3; oId CUFF.1827.4; tss_id TSS985;
1	Cufflinks	exon	67779053	67780949	.	+	.	gene_id GRMZM2G179792; transcript_id TCONS_00001099; exon_number 4; oId CUFF.1827.4; tss_id TSS985;
1	Cufflinks	exon	67781024	67781541	.	+	.	gene_id GRMZM2G179792; transcript_id TCONS_00001099; exon_number 5; oId CUFF.1827.4; tss_id TSS985;
1	Cufflinks	exon	67777669	67778057	.	+	.	gene_id GRMZM2G179792; transcript_id TCONS_00001100; exon_number 1; oId CUFF.1827.5; tss_id TSS985;
1	Cufflinks	exon	67778152	67778411	.	+	.	gene_id GRMZM2G179792; transcript_id TCONS_00001100; exon_number 2; oId CUFF.1827.5; tss_id TSS985;
1	Cufflinks	exon	67778887	67778946	.	+	.	gene_id GRMZM2G179792; transcript_id TCONS_00001100; exon_number 3; oId CUFF.1827.5; tss_id TSS985;
1	Cufflinks	exon	67779047	67780949	.	+	.	gene_id GRMZM2G179792; transcript_id TCONS_00001100; exon_number 4; oId CUFF.1827.5; tss_id TSS985;
1	Cufflinks	exon	67781024	67781541	.	+	.	gene_id GRMZM2G179792; transcript_id TCONS_00001100; exon_number 5; oId CUFF.1827.5; tss_id TSS985;
1	Cufflinks	exon	70583236	70583727	.	+	.	gene_id GRMZM2G071688; transcript_id TCONS_00001126; exon_number 1; oId CUFF.1826.2; tss_id TSS1008;
1	Cufflinks	exon	70583823	70583911	.	+	.	gene_id GRMZM2G071688; transcript_id TCONS_00001126; exon_number 2; oId CUFF.1826.2; tss_id TSS1008;
1	Cufflinks	exon	70584009	70584258	.	+	.	gene_id GRMZM2G071688; transcript_id TCONS_00001126; exon_number 3; oId CUFF.1826.2; tss_id TSS1008;
1	Cufflinks	exon	70584356	70584604	.	+	.	gene_id GRMZM2G071688; transcript_id TCONS_00001126; exon_number 4; oId CUFF.1826.2; tss_id TSS1008;
1	Cufflinks	exon	70584679	70585097	.	+	.	gene_id GRMZM2G071688; transcript_id TCONS_00001126; exon_number 5; oId CUFF.1826.2; tss_id TSS1008;
1	Cufflinks	exon	77040208	77040581	.	+	.	gene_id GRMZM2G157164; transcript_id TCONS_00001202; exon_number 1; oId CUFF.1969.1; tss_id TSS1075;
1	Cufflinks	exon	77040732	77041589	.	+	.	gene_id GRMZM2G157164; transcript_id TCONS_00001202; exon_number 2; oId CUFF.1969.1; tss_id TSS1075;
1	Cufflinks	exon	79336931	79337283	.	+	.	gene_id GRMZM5G826389; transcript_id TCONS_00001214; exon_number 1; oId CUFF.2005.1; tss_id TSS1086;
1	Cufflinks	exon	79337401	79337648	.	+	.	gene_id GRMZM5G826389; transcript_id TCONS_00001214; exon_number 2; oId CUFF.2005.1; tss_id TSS1086;
1	Cufflinks	exon	79337759	79338465	.	+	.	gene_id GRMZM5G826389; transcript_id TCONS_00001214; exon_number 3; oId CUFF.2005.1; tss_id TSS1086;
1	Cufflinks	exon	81588714	81589172	.	+	.	gene_id GRMZM2G178079; transcript_id TCONS_00001244; exon_number 1; oId CUFF.2050.1; tss_id TSS1111;
1	Cufflinks	exon	81589287	81589906	.	+	.	gene_id GRMZM2G178079; transcript_id TCONS_00001244; exon_number 2; oId CUFF.2050.1; tss_id TSS1111;
1	Cufflinks	exon	85027826	85028366	.	+	.	gene_id GRMZM2G026147; transcript_id TCONS_00001275; exon_number 1; oId CUFF.2115.1; tss_id TSS1141;
1	Cufflinks	exon	85028456	85028565	.	+	.	gene_id GRMZM2G026147; transcript_id TCONS_00001275; exon_number 2; oId CUFF.2115.1; tss_id TSS1141;
1	Cufflinks	exon	85028691	85028872	.	+	.	gene_id GRMZM2G026147; transcript_id TCONS_00001275; exon_number 3; oId CUFF.2115.1; tss_id TSS1141;
1	Cufflinks	exon	85028989	85029124	.	+	.	gene_id GRMZM2G026147; transcript_id TCONS_00001275; exon_number 4; oId CUFF.2115.1; tss_id TSS1141;
1	Cufflinks	exon	85029320	85029741	.	+	.	gene_id GRMZM2G026147; transcript_id TCONS_00001275; exon_number 5; oId CUFF.2115.1; tss_id TSS1141;
1	Cufflinks	exon	86001434	86001765	.	+	.	gene_id GRMZM2G154549; transcript_id TCONS_00001285; exon_number 1; oId CUFF.2140.1; tss_id TSS1150;
1	Cufflinks	exon	86001863	86001939	.	+	.	gene_id GRMZM2G154549; transcript_id TCONS_00001285; exon_number 2; oId CUFF.2140.1; tss_id TSS1150;

1	Cufflinks	exon	86002050	86002505	.	+	.	gene_id GRMZM2G154549; transcript_id TCONS_00001285; exon_number 3; oId CUFF.2140.1; tss_id TSS1150;
1	Cufflinks	exon	89004748	89006489	.	+	.	gene_id GRMZM2G089248; transcript_id TCONS_00001327; exon_number 1; oId CUFF.2176.1; tss_id TSS1188;
1	Cufflinks	exon	90797641	90799416	.	+	.	gene_id GRMZM2G317386; transcript_id TCONS_00001336; exon_number 1; oId CUFF.2202.1; tss_id TSS1197;
1	Cufflinks	exon	94375915	94376346	.	+	.	gene_id GRMZM2G088524; transcript_id TCONS_00001362; exon_number 1; oId CUFF.2242.1; tss_id TSS1221;
1	Cufflinks	exon	94376614	94376837	.	+	.	gene_id GRMZM2G088524; transcript_id TCONS_00001362; exon_number 2; oId CUFF.2242.1; tss_id TSS1221;
1	Cufflinks	exon	94376956	94377036	.	+	.	gene_id GRMZM2G088524; transcript_id TCONS_00001362; exon_number 3; oId CUFF.2242.1; tss_id TSS1221;
1	Cufflinks	exon	94377136	94377183	.	+	.	gene_id GRMZM2G088524; transcript_id TCONS_00001362; exon_number 4; oId CUFF.2242.1; tss_id TSS1221;
1	Cufflinks	exon	94377299	94377975	.	+	.	gene_id GRMZM2G088524; transcript_id TCONS_00001362; exon_number 5; oId CUFF.2242.1; tss_id TSS1221;
1	Cufflinks	exon	147170692	147172458	.	+	.	gene_id GRMZM2G479885; transcript_id TCONS_00001585; exon_number 1; oId CUFF.2658.1; tss_id TSS1429;
1	Cufflinks	exon	162442172	162444014	.	+	.	gene_id GRMZM2G039112; transcript_id TCONS_00001675; exon_number 1; oId CUFF.2835.1; tss_id TSS1508;
1	Cufflinks	exon	174235528	174235862	.	+	.	gene_id GRMZM2G047835; transcript_id TCONS_00001788; exon_number 1; oId CUFF.2996.1; tss_id TSS1606;
1	Cufflinks	exon	174236417	174238895	.	+	.	gene_id GRMZM2G047835; transcript_id TCONS_00001788; exon_number 2; oId CUFF.2996.1; tss_id TSS1606;
1	Cufflinks	exon	176872260	176873419	.	+	.	gene_id XLOC_001559; transcript_id TCONS_00001812; exon_number 1; oId CUFF.3069.1; tss_id TSS1627;
1	Cufflinks	exon	178503773	178504401	.	+	.	gene_id XLOC_001566; transcript_id TCONS_00001819; exon_number 1; oId CUFF.3065.1; tss_id TSS1634;
1	Cufflinks	exon	178504502	178504714	.	+	.	gene_id XLOC_001566; transcript_id TCONS_00001819; exon_number 2; oId CUFF.3065.1; tss_id TSS1634;
1	Cufflinks	exon	180020968	180021646	.	+	.	gene_id XLOC_001584; transcript_id TCONS_00001838; exon_number 1; oId CUFF.3094.1; tss_id TSS1653;
1	Cufflinks	exon	180715451	180716127	.	+	.	gene_id GRMZM2G423472; transcript_id TCONS_00001846; exon_number 1; oId CUFF.3157.2; tss_id TSS1660;
1	Cufflinks	exon	180716208	180716283	.	+	.	gene_id GRMZM2G423472; transcript_id TCONS_00001846; exon_number 2; oId CUFF.3157.2; tss_id TSS1660;
1	Cufflinks	exon	180716397	180716512	.	+	.	gene_id GRMZM2G423472; transcript_id TCONS_00001846; exon_number 3; oId CUFF.3157.2; tss_id TSS1660;
1	Cufflinks	exon	180717811	180718850	.	+	.	gene_id GRMZM2G423472; transcript_id TCONS_00001846; exon_number 4; oId CUFF.3157.2; tss_id TSS1660;
1	Cufflinks	exon	180718976	180719208	.	+	.	gene_id GRMZM2G423472; transcript_id TCONS_00001846; exon_number 5; oId CUFF.3157.2; tss_id TSS1660;
1	Cufflinks	exon	180719360	180719501	.	+	.	gene_id GRMZM2G423472; transcript_id TCONS_00001846; exon_number 6; oId CUFF.3157.2; tss_id TSS1660;
1	Cufflinks	exon	180719642	180719762	.	+	.	gene_id GRMZM2G423472; transcript_id TCONS_00001846; exon_number 7; oId CUFF.3157.2; tss_id TSS1660;
1	Cufflinks	exon	180719860	180719922	.	+	.	gene_id GRMZM2G423472; transcript_id TCONS_00001846; exon_number 8; oId CUFF.3157.2; tss_id TSS1660;
1	Cufflinks	exon	180720076	180720707	.	+	.	gene_id GRMZM2G423472; transcript_id TCONS_00001846; exon_number 9; oId CUFF.3157.2; tss_id TSS1660;
1	Cufflinks	exon	182125586	182126923	.	+	.	gene_id XLOC_001611; transcript_id TCONS_00001870; exon_number 1; oId CUFF.3139.1; tss_id TSS1682;
1	Cufflinks	exon	183321740	183322864	.	+	.	gene_id GRMZM2G179351; transcript_id TCONS_00001885; exon_number 1; oId CUFF.3193.1; tss_id TSS1696;
1	Cufflinks	exon	183327944	183329317	.	+	.	gene_id GRMZM2G179351; transcript_id TCONS_00001885; exon_number 2; oId CUFF.3193.1; tss_id TSS1696;
1	Cufflinks	exon	183330439	183330621	.	+	.	gene_id GRMZM2G179351; transcript_id TCONS_00001885; exon_number 3; oId CUFF.3193.1; tss_id TSS1696;
1	Cufflinks	exon	183330782	183331065	.	+	.	gene_id GRMZM2G179351; transcript_id TCONS_00001885; exon_number 4; oId CUFF.3193.1; tss_id TSS1696;
1	Cufflinks	exon	183331160	183331276	.	+	.	gene_id GRMZM2G179351; transcript_id TCONS_00001885; exon_number 5; oId CUFF.3193.1; tss_id TSS1696;
1	Cufflinks	exon	183331739	183331995	.	+	.	gene_id GRMZM2G179351; transcript_id TCONS_00001885; exon_number 6; oId CUFF.3193.1; tss_id TSS1696;
1	Cufflinks	exon	183332158	183332335	.	+	.	gene_id GRMZM2G179351; transcript_id TCONS_00001885; exon_number 7; oId CUFF.3193.1; tss_id TSS1696;
1	Cufflinks	exon	183332467	183332739	.	+	.	gene_id GRMZM2G179351; transcript_id TCONS_00001885; exon_number 8; oId CUFF.3193.1; tss_id TSS1696;
1	Cufflinks	exon	183333454	183334968	.	+	.	gene_id GRMZM2G179351; transcript_id TCONS_00001885; exon_number 9; oId CUFF.3193.1; tss_id TSS1696;
1	Cufflinks	exon	183474570	183474725	.	+	.	gene_id GRMZM2G042371; transcript_id TCONS_00001889; exon_number 1; oId CUFF.3189.1; tss_id TSS1700;

1	Cufflinks	exon	183474892	183475052	.	+	.	gene_id GRMZM2G042371; transcript_id TCONS_00001889; exon_number 2; oId CUFF.3189.1; tss_id TSS1700;
1	Cufflinks	exon	183475141	183475812	.	+	.	gene_id GRMZM2G042371; transcript_id TCONS_00001889; exon_number 3; oId CUFF.3189.1; tss_id TSS1700;
1	Cufflinks	exon	183475919	183476237	.	+	.	gene_id GRMZM2G042371; transcript_id TCONS_00001889; exon_number 4; oId CUFF.3189.1; tss_id TSS1700;
1	Cufflinks	exon	183476380	183476597	.	+	.	gene_id GRMZM2G042371; transcript_id TCONS_00001889; exon_number 5; oId CUFF.3189.1; tss_id TSS1700;
1	Cufflinks	exon	183476850	183477027	.	+	.	gene_id GRMZM2G042371; transcript_id TCONS_00001889; exon_number 6; oId CUFF.3189.1; tss_id TSS1700;
1	Cufflinks	exon	183477146	183477893	.	+	.	gene_id GRMZM2G042371; transcript_id TCONS_00001889; exon_number 7; oId CUFF.3189.1; tss_id TSS1700;
1	Cufflinks	exon	187375298	187375581	.	+	.	gene_id GRMZM2G083755; transcript_id TCONS_00001935; exon_number 1; oId CUFF.3287.4; tss_id TSS1740;
1	Cufflinks	exon	187376033	187376429	.	+	.	gene_id GRMZM2G083755; transcript_id TCONS_00001935; exon_number 2; oId CUFF.3287.4; tss_id TSS1740;
1	Cufflinks	exon	187377130	187377202	.	+	.	gene_id GRMZM2G083755; transcript_id TCONS_00001935; exon_number 3; oId CUFF.3287.4; tss_id TSS1740;
1	Cufflinks	exon	187377299	187377346	.	+	.	gene_id GRMZM2G083755; transcript_id TCONS_00001935; exon_number 4; oId CUFF.3287.4; tss_id TSS1740;
1	Cufflinks	exon	187377455	187377549	.	+	.	gene_id GRMZM2G083755; transcript_id TCONS_00001935; exon_number 5; oId CUFF.3287.4; tss_id TSS1740;
1	Cufflinks	exon	187377978	187379353	.	+	.	gene_id GRMZM2G083755; transcript_id TCONS_00001935; exon_number 6; oId CUFF.3287.4; tss_id TSS1740;
1	Cufflinks	exon	188147934	188148673	.	+	.	gene_id GRMZM5G822593; transcript_id TCONS_00001954; exon_number 1; oId CUFF.3300.1; tss_id TSS1755;
1	Cufflinks	exon	188150137	188150468	.	+	.	gene_id GRMZM5G822593; transcript_id TCONS_00001954; exon_number 2; oId CUFF.3300.1; tss_id TSS1755;
1	Cufflinks	exon	188150988	188151552	.	+	.	gene_id GRMZM5G822593; transcript_id TCONS_00001954; exon_number 3; oId CUFF.3300.1; tss_id TSS1755;
1	Cufflinks	exon	188151699	188151784	.	+	.	gene_id GRMZM5G822593; transcript_id TCONS_00001954; exon_number 4; oId CUFF.3300.1; tss_id TSS1755;
1	Cufflinks	exon	188151896	188153498	.	+	.	gene_id GRMZM5G822593; transcript_id TCONS_00001954; exon_number 5; oId CUFF.3300.1; tss_id TSS1755;
1	Cufflinks	exon	189385201	189385760	.	+	.	gene_id GRMZM2G124557; transcript_id TCONS_00001969; exon_number 1; oId CUFF.3317.1; tss_id TSS1770;
1	Cufflinks	exon	189387745	189387917	.	+	.	gene_id GRMZM2G124557; transcript_id TCONS_00001969; exon_number 2; oId CUFF.3317.1; tss_id TSS1770;
1	Cufflinks	exon	189388005	189388158	.	+	.	gene_id GRMZM2G124557; transcript_id TCONS_00001969; exon_number 3; oId CUFF.3317.1; tss_id TSS1770;
1	Cufflinks	exon	189388276	189388392	.	+	.	gene_id GRMZM2G124557; transcript_id TCONS_00001969; exon_number 4; oId CUFF.3317.1; tss_id TSS1770;
1	Cufflinks	exon	189388558	189388624	.	+	.	gene_id GRMZM2G124557; transcript_id TCONS_00001969; exon_number 5; oId CUFF.3317.1; tss_id TSS1770;
1	Cufflinks	exon	189388730	189388878	.	+	.	gene_id GRMZM2G124557; transcript_id TCONS_00001969; exon_number 6; oId CUFF.3317.1; tss_id TSS1770;
1	Cufflinks	exon	189389242	189389375	.	+	.	gene_id GRMZM2G124557; transcript_id TCONS_00001969; exon_number 7; oId CUFF.3317.1; tss_id TSS1770;
1	Cufflinks	exon	189389477	189389575	.	+	.	gene_id GRMZM2G124557; transcript_id TCONS_00001969; exon_number 8; oId CUFF.3317.1; tss_id TSS1770;
1	Cufflinks	exon	189389680	189389755	.	+	.	gene_id GRMZM2G124557; transcript_id TCONS_00001969; exon_number 9; oId CUFF.3317.1; tss_id TSS1770;
1	Cufflinks	exon	189389852	189390321	.	+	.	gene_id GRMZM2G124557; transcript_id TCONS_00001969; exon_number 10; oId CUFF.3317.1; tss_id TSS1770;
1	Cufflinks	exon	192065376	192065991	.	+	.	gene_id GRMZM5G828396; transcript_id TCONS_00002012; exon_number 1; oId CUFF.3372.1; tss_id TSS1809;
1	Cufflinks	exon	192066115	192066381	.	+	.	gene_id GRMZM5G828396; transcript_id TCONS_00002012; exon_number 2; oId CUFF.3372.1; tss_id TSS1809;
1	Cufflinks	exon	192066493	192066558	.	+	.	gene_id GRMZM5G828396; transcript_id TCONS_00002012; exon_number 3; oId CUFF.3372.1; tss_id TSS1809;
1	Cufflinks	exon	192066659	192066727	.	+	.	gene_id GRMZM5G828396; transcript_id TCONS_00002012; exon_number 4; oId CUFF.3372.1; tss_id TSS1809;
1	Cufflinks	exon	192066864	192066935	.	+	.	gene_id GRMZM5G828396; transcript_id TCONS_00002012; exon_number 5; oId CUFF.3372.1; tss_id TSS1809;
1	Cufflinks	exon	192067065	192067283	.	+	.	gene_id GRMZM5G828396; transcript_id TCONS_00002012; exon_number 6; oId CUFF.3372.1; tss_id TSS1809;
1	Cufflinks	exon	192067377	192067488	.	+	.	gene_id GRMZM5G828396; transcript_id TCONS_00002012; exon_number 7; oId CUFF.3372.1; tss_id TSS1809;
1	Cufflinks	exon	192067605	192068429	.	+	.	gene_id GRMZM5G828396; transcript_id TCONS_00002012; exon_number 8; oId CUFF.3372.1; tss_id TSS1809;
1	Cufflinks	exon	192065381	192065991	.	+	.	gene_id GRMZM5G828396; transcript_id TCONS_00002013; exon_number 1; oId CUFF.3372.2; tss_id TSS1809;

1	Cufflinks	exon	192066115	192066381	.	+	.	gene_id GRMZM5G828396; transcript_id TCONS_00002013; exon_number 2; oId CUFF.3372.2; tss_id TSS1809;
1	Cufflinks	exon	192066493	192066558	.	+	.	gene_id GRMZM5G828396; transcript_id TCONS_00002013; exon_number 3; oId CUFF.3372.2; tss_id TSS1809;
1	Cufflinks	exon	192066659	192066727	.	+	.	gene_id GRMZM5G828396; transcript_id TCONS_00002013; exon_number 4; oId CUFF.3372.2; tss_id TSS1809;
1	Cufflinks	exon	192066864	192066935	.	+	.	gene_id GRMZM5G828396; transcript_id TCONS_00002013; exon_number 5; oId CUFF.3372.2; tss_id TSS1809;
1	Cufflinks	exon	192067074	192067283	.	+	.	gene_id GRMZM5G828396; transcript_id TCONS_00002013; exon_number 6; oId CUFF.3372.2; tss_id TSS1809;
1	Cufflinks	exon	192067377	192067488	.	+	.	gene_id GRMZM5G828396; transcript_id TCONS_00002013; exon_number 7; oId CUFF.3372.2; tss_id TSS1809;
1	Cufflinks	exon	192067605	192068429	.	+	.	gene_id GRMZM5G828396; transcript_id TCONS_00002013; exon_number 8; oId CUFF.3372.2; tss_id TSS1809;
1	Cufflinks	exon	197104152	197104355	.	+	.	gene_id GRMZM2G477872; transcript_id TCONS_00002079; exon_number 1; oId CUFF.3485.1; tss_id TSS1871;
1	Cufflinks	exon	197104493	197104613	.	+	.	gene_id GRMZM2G477872; transcript_id TCONS_00002079; exon_number 2; oId CUFF.3485.1; tss_id TSS1871;
1	Cufflinks	exon	197106198	197106286	.	+	.	gene_id GRMZM2G477872; transcript_id TCONS_00002079; exon_number 3; oId CUFF.3485.1; tss_id TSS1871;
1	Cufflinks	exon	197106517	197106601	.	+	.	gene_id GRMZM2G477872; transcript_id TCONS_00002079; exon_number 4; oId CUFF.3485.1; tss_id TSS1871;
1	Cufflinks	exon	197106724	197106883	.	+	.	gene_id GRMZM2G477872; transcript_id TCONS_00002079; exon_number 5; oId CUFF.3485.1; tss_id TSS1871;
1	Cufflinks	exon	197107072	197107148	.	+	.	gene_id GRMZM2G477872; transcript_id TCONS_00002079; exon_number 6; oId CUFF.3485.1; tss_id TSS1871;
1	Cufflinks	exon	197107224	197107274	.	+	.	gene_id GRMZM2G477872; transcript_id TCONS_00002079; exon_number 7; oId CUFF.3485.1; tss_id TSS1871;
1	Cufflinks	exon	197107344	197107434	.	+	.	gene_id GRMZM2G477872; transcript_id TCONS_00002079; exon_number 8; oId CUFF.3485.1; tss_id TSS1871;
1	Cufflinks	exon	197107728	197108029	.	+	.	gene_id GRMZM2G477872; transcript_id TCONS_00002079; exon_number 9; oId CUFF.3485.1; tss_id TSS1871;
1	Cufflinks	exon	197108387	197108982	.	+	.	gene_id GRMZM2G477872; transcript_id TCONS_00002079; exon_number 10; oId CUFF.3485.1; tss_id TSS1871;
1	Cufflinks	exon	197109071	197109231	.	+	.	gene_id GRMZM2G477872; transcript_id TCONS_00002079; exon_number 11; oId CUFF.3485.1; tss_id TSS1871;
1	Cufflinks	exon	197109340	197109443	.	+	.	gene_id GRMZM2G477872; transcript_id TCONS_00002079; exon_number 12; oId CUFF.3485.1; tss_id TSS1871;
1	Cufflinks	exon	197109543	197109702	.	+	.	gene_id GRMZM2G477872; transcript_id TCONS_00002079; exon_number 13; oId CUFF.3485.1; tss_id TSS1871;
1	Cufflinks	exon	197109786	197109949	.	+	.	gene_id GRMZM2G477872; transcript_id TCONS_00002079; exon_number 14; oId CUFF.3485.1; tss_id TSS1871;
1	Cufflinks	exon	197110040	197110372	.	+	.	gene_id GRMZM2G477872; transcript_id TCONS_00002079; exon_number 15; oId CUFF.3485.1; tss_id TSS1871;
1	Cufflinks	exon	197110456	197110746	.	+	.	gene_id GRMZM2G477872; transcript_id TCONS_00002079; exon_number 16; oId CUFF.3485.1; tss_id TSS1871;
1	Cufflinks	exon	197111206	197111289	.	+	.	gene_id GRMZM2G477872; transcript_id TCONS_00002079; exon_number 17; oId CUFF.3485.1; tss_id TSS1871;
1	Cufflinks	exon	197111363	197111496	.	+	.	gene_id GRMZM2G477872; transcript_id TCONS_00002079; exon_number 18; oId CUFF.3485.1; tss_id TSS1871;
1	Cufflinks	exon	197111600	197112088	.	+	.	gene_id GRMZM2G477872; transcript_id TCONS_00002079; exon_number 19; oId CUFF.3485.1; tss_id TSS1871;
1	Cufflinks	exon	197112194	197112448	.	+	.	gene_id GRMZM2G477872; transcript_id TCONS_00002079; exon_number 20; oId CUFF.3485.1; tss_id TSS1871;
1	Cufflinks	exon	197112570	197113073	.	+	.	gene_id GRMZM2G477872; transcript_id TCONS_00002079; exon_number 21; oId CUFF.3485.1; tss_id TSS1871;
1	Cufflinks	exon	197301278	197301634	.	+	.	gene_id GRMZM2G175423; transcript_id TCONS_00002080; exon_number 1; oId CUFF.3491.1; tss_id TSS1872;
1	Cufflinks	exon	197303015	197303082	.	+	.	gene_id GRMZM2G175423; transcript_id TCONS_00002080; exon_number 2; oId CUFF.3491.1; tss_id TSS1872;
1	Cufflinks	exon	197303180	197304421	.	+	.	gene_id GRMZM2G175423; transcript_id TCONS_00002080; exon_number 3; oId CUFF.3491.1; tss_id TSS1872;
1	Cufflinks	exon	200504395	200505328	.	+	.	gene_id GRMZM2G457309; transcript_id TCONS_00002135; exon_number 1; oId CUFF.3555.1; tss_id TSS1920;
1	Cufflinks	exon	201668843	201669743	.	+	.	gene_id GRMZM2G318794; transcript_id TCONS_00002146; exon_number 1; oId CUFF.3583.1; tss_id TSS1931;
1	Cufflinks	exon	208870529	208870976	.	+	.	gene_id XLOC_001946; transcript_id TCONS_00002261; exon_number 1; oId CUFF.3789.1; tss_id TSS2033;
1	Cufflinks	exon	216924039	216924541	.	+	.	gene_id GRMZM2G142249; transcript_id TCONS_00002345; exon_number 1; oId CUFF.3937.2; tss_id TSS2112;
1	Cufflinks	exon	216925849	216926063	.	+	.	gene_id GRMZM2G142249; transcript_id TCONS_00002345; exon_number 2; oId CUFF.3937.2; tss_id TSS2112;

1	Cufflinks	exon	216924039	216924541	.	+	.	gene_id GRMZM2G142249; transcript_id TCONS_00002346; exon_number 1; oId CUFF.3937.1; tss_id TSS2112;
1	Cufflinks	exon	216926623	216926854	.	+	.	gene_id GRMZM2G142249; transcript_id TCONS_00002346; exon_number 2; oId CUFF.3937.1; tss_id TSS2112;
1	Cufflinks	exon	216927175	216927344	.	+	.	gene_id GRMZM2G142249; transcript_id TCONS_00002346; exon_number 3; oId CUFF.3937.1; tss_id TSS2112;
1	Cufflinks	exon	216927421	216927606	.	+	.	gene_id GRMZM2G142249; transcript_id TCONS_00002346; exon_number 4; oId CUFF.3937.1; tss_id TSS2112;
1	Cufflinks	exon	216927785	216927880	.	+	.	gene_id GRMZM2G142249; transcript_id TCONS_00002346; exon_number 5; oId CUFF.3937.1; tss_id TSS2112;
1	Cufflinks	exon	216927964	216928164	.	+	.	gene_id GRMZM2G142249; transcript_id TCONS_00002346; exon_number 6; oId CUFF.3937.1; tss_id TSS2112;
1	Cufflinks	exon	216928249	216928405	.	+	.	gene_id GRMZM2G142249; transcript_id TCONS_00002346; exon_number 7; oId CUFF.3937.1; tss_id TSS2112;
1	Cufflinks	exon	216928486	216928715	.	+	.	gene_id GRMZM2G142249; transcript_id TCONS_00002346; exon_number 8; oId CUFF.3937.1; tss_id TSS2112;
1	Cufflinks	exon	216928802	216928919	.	+	.	gene_id GRMZM2G142249; transcript_id TCONS_00002346; exon_number 9; oId CUFF.3937.1; tss_id TSS2112;
1	Cufflinks	exon	216929009	216929085	.	+	.	gene_id GRMZM2G142249; transcript_id TCONS_00002346; exon_number 10; oId CUFF.3937.1; tss_id TSS2112;
1	Cufflinks	exon	216929177	216929266	.	+	.	gene_id GRMZM2G142249; transcript_id TCONS_00002346; exon_number 11; oId CUFF.3937.1; tss_id TSS2112;
1	Cufflinks	exon	216929357	216929500	.	+	.	gene_id GRMZM2G142249; transcript_id TCONS_00002346; exon_number 12; oId CUFF.3937.1; tss_id TSS2112;
1	Cufflinks	exon	216929594	216929908	.	+	.	gene_id GRMZM2G142249; transcript_id TCONS_00002346; exon_number 13; oId CUFF.3937.1; tss_id TSS2112;
1	Cufflinks	exon	216930000	216930080	.	+	.	gene_id GRMZM2G142249; transcript_id TCONS_00002346; exon_number 14; oId CUFF.3937.1; tss_id TSS2112;
1	Cufflinks	exon	216930156	216930332	.	+	.	gene_id GRMZM2G142249; transcript_id TCONS_00002346; exon_number 15; oId CUFF.3937.1; tss_id TSS2112;
1	Cufflinks	exon	216930522	216931011	.	+	.	gene_id GRMZM2G142249; transcript_id TCONS_00002346; exon_number 16; oId CUFF.3937.1; tss_id TSS2112;
1	Cufflinks	exon	217331974	217333074	.	+	.	gene_id AC217910.3_FG004; transcript_id TCONS_00002350; exon_number 1; oId CUFF.3925.1; tss_id TSS2115;
1	Cufflinks	exon	218852913	218853736	.	+	.	gene_id XLOC_002042; transcript_id TCONS_00002368; exon_number 1; oId CUFF.3955.1; tss_id TSS2131;
1	Cufflinks	exon	219944821	219945181	.	+	.	gene_id GRMZM2G015925; transcript_id TCONS_00002384; exon_number 1; oId CUFF.3997.1; tss_id TSS2146;
1	Cufflinks	exon	219945275	219945359	.	+	.	gene_id GRMZM2G015925; transcript_id TCONS_00002384; exon_number 2; oId CUFF.3997.1; tss_id TSS2146;
1	Cufflinks	exon	219951823	219951931	.	+	.	gene_id GRMZM2G015925; transcript_id TCONS_00002384; exon_number 3; oId CUFF.3997.1; tss_id TSS2146;
1	Cufflinks	exon	219952038	219952126	.	+	.	gene_id GRMZM2G015925; transcript_id TCONS_00002384; exon_number 4; oId CUFF.3997.1; tss_id TSS2146;
1	Cufflinks	exon	219952198	219952262	.	+	.	gene_id GRMZM2G015925; transcript_id TCONS_00002384; exon_number 5; oId CUFF.3997.1; tss_id TSS2146;
1	Cufflinks	exon	219952348	219952426	.	+	.	gene_id GRMZM2G015925; transcript_id TCONS_00002384; exon_number 6; oId CUFF.3997.1; tss_id TSS2146;
1	Cufflinks	exon	219952492	219953265	.	+	.	gene_id GRMZM2G015925; transcript_id TCONS_00002384; exon_number 7; oId CUFF.3997.1; tss_id TSS2146;
1	Cufflinks	exon	221239260	221241115	.	+	.	gene_id XLOC_002070; transcript_id TCONS_00002401; exon_number 1; oId CUFF.4009.1; tss_id TSS2161;
1	Cufflinks	exon	224437062	224438028	.	+	.	gene_id GRMZM2G414727; transcript_id TCONS_00002457; exon_number 1; oId CUFF.4102.1; tss_id TSS2211;
1	Cufflinks	exon	226230810	226231286	.	+	.	gene_id GRMZM5G882228; transcript_id TCONS_00002481; exon_number 1; oId CUFF.4150.1; tss_id TSS2232;
1	Cufflinks	exon	226233234	226234051	.	+	.	gene_id GRMZM5G882228; transcript_id TCONS_00002481; exon_number 2; oId CUFF.4150.1; tss_id TSS2232;
1	Cufflinks	exon	227894328	227895186	.	+	.	gene_id GRMZM2G082529; transcript_id TCONS_00002501; exon_number 1; oId CUFF.4218.1; tss_id TSS2248;
1	Cufflinks	exon	227895718	227896668	.	+	.	gene_id GRMZM2G082529; transcript_id TCONS_00002501; exon_number 2; oId CUFF.4218.1; tss_id TSS2248;
1	Cufflinks	exon	227896834	227896923	.	+	.	gene_id GRMZM2G082529; transcript_id TCONS_00002501; exon_number 3; oId CUFF.4218.1; tss_id TSS2248;
1	Cufflinks	exon	227897011	227897079	.	+	.	gene_id GRMZM2G082529; transcript_id TCONS_00002501; exon_number 4; oId CUFF.4218.1; tss_id TSS2248;
1	Cufflinks	exon	227897542	227897682	.	+	.	gene_id GRMZM2G082529; transcript_id TCONS_00002501; exon_number 5; oId CUFF.4218.1; tss_id TSS2248;
1	Cufflinks	exon	227897945	227897993	.	+	.	gene_id GRMZM2G082529; transcript_id TCONS_00002501; exon_number 6; oId CUFF.4218.1; tss_id TSS2248;
1	Cufflinks	exon	227898942	227899045	.	+	.	gene_id GRMZM2G082529; transcript_id TCONS_00002501; exon_number 7; oId CUFF.4218.1; tss_id TSS2248;

1	Cufflinks	exon	227899153	227899992	.	+	.	gene_id GRMZM2G082529; transcript_id TCONS_00002501; exon_number 8; oId CUFF.4218.1; tss_id TSS2248;
1	Cufflinks	exon	229664358	229665687	.	+	.	gene_id GRMZM2G162758; transcript_id TCONS_00002523; exon_number 1; oId CUFF.4258.1; tss_id TSS2268;
1	Cufflinks	exon	229666756	229667449	.	+	.	gene_id GRMZM2G162758; transcript_id TCONS_00002523; exon_number 2; oId CUFF.4258.1; tss_id TSS2268;
1	Cufflinks	exon	234403145	234403853	.	+	.	gene_id XLOC_002220; transcript_id TCONS_00002577; exon_number 1; oId CUFF.4366.2; tss_id TSS2316;
1	Cufflinks	exon	237009950	237010237	.	+	.	gene_id GRMZM2G152901; transcript_id TCONS_00002607; exon_number 1; oId CUFF.4444.1; tss_id TSS2344;
1	Cufflinks	exon	237010866	237011571	.	+	.	gene_id GRMZM2G152901; transcript_id TCONS_00002607; exon_number 2; oId CUFF.4444.1; tss_id TSS2344;
1	Cufflinks	exon	237011731	237011850	.	+	.	gene_id GRMZM2G152901; transcript_id TCONS_00002607; exon_number 3; oId CUFF.4444.1; tss_id TSS2344;
1	Cufflinks	exon	237012543	237014064	.	+	.	gene_id GRMZM2G152901; transcript_id TCONS_00002607; exon_number 4; oId CUFF.4444.1; tss_id TSS2344;
1	Cufflinks	exon	237009952	237010237	.	+	.	gene_id GRMZM2G152901; transcript_id TCONS_00002608; exon_number 1; oId CUFF.4444.2; tss_id TSS2344;
1	Cufflinks	exon	237010871	237011571	.	+	.	gene_id GRMZM2G152901; transcript_id TCONS_00002608; exon_number 2; oId CUFF.4444.2; tss_id TSS2344;
1	Cufflinks	exon	237011731	237011850	.	+	.	gene_id GRMZM2G152901; transcript_id TCONS_00002608; exon_number 3; oId CUFF.4444.2; tss_id TSS2344;
1	Cufflinks	exon	237012543	237014064	.	+	.	gene_id GRMZM2G152901; transcript_id TCONS_00002608; exon_number 4; oId CUFF.4444.2; tss_id TSS2344;
1	Cufflinks	exon	242766570	242767111	.	+	.	gene_id GRMZM2G138455; transcript_id TCONS_00002675; exon_number 1; oId CUFF.4543.1; tss_id TSS2406;
1	Cufflinks	exon	242767342	242768953	.	+	.	gene_id GRMZM2G138455; transcript_id TCONS_00002675; exon_number 2; oId CUFF.4543.1; tss_id TSS2406;
1	Cufflinks	exon	253775521	253776063	.	+	.	gene_id GRMZM2G021055; transcript_id TCONS_00002813; exon_number 1; oId CUFF.4762.1; tss_id TSS2527;
1	Cufflinks	exon	253778881	253779422	.	+	.	gene_id GRMZM2G021055; transcript_id TCONS_00002813; exon_number 2; oId CUFF.4762.1; tss_id TSS2527;
1	Cufflinks	exon	253779506	253779592	.	+	.	gene_id GRMZM2G021055; transcript_id TCONS_00002813; exon_number 3; oId CUFF.4762.1; tss_id TSS2527;
1	Cufflinks	exon	253779684	253779740	.	+	.	gene_id GRMZM2G021055; transcript_id TCONS_00002813; exon_number 4; oId CUFF.4762.1; tss_id TSS2527;
1	Cufflinks	exon	253779851	253780208	.	+	.	gene_id GRMZM2G021055; transcript_id TCONS_00002813; exon_number 5; oId CUFF.4762.1; tss_id TSS2527;
1	Cufflinks	exon	253780302	253780778	.	+	.	gene_id GRMZM2G021055; transcript_id TCONS_00002813; exon_number 6; oId CUFF.4762.1; tss_id TSS2527;
1	Cufflinks	exon	255383820	255384041	.	+	.	gene_id GRMZM2G017319; transcript_id TCONS_00002833; exon_number 1; oId CUFF.4808.2; tss_id TSS2546;
1	Cufflinks	exon	255384789	255386002	.	+	.	gene_id GRMZM2G017319; transcript_id TCONS_00002833; exon_number 2; oId CUFF.4808.2; tss_id TSS2546;
1	Cufflinks	exon	255383820	255384155	.	+	.	gene_id GRMZM2G017319; transcript_id TCONS_00002832; exon_number 1; oId CUFF.4808.1; tss_id TSS2546;
1	Cufflinks	exon	255384360	255386002	.	+	.	gene_id GRMZM2G017319; transcript_id TCONS_00002832; exon_number 2; oId CUFF.4808.1; tss_id TSS2546;
1	Cufflinks	exon	256131686	256132059	.	+	.	gene_id GRMZM2G150772; transcript_id TCONS_00002838; exon_number 1; oId CUFF.4831.1; tss_id TSS2551;
1	Cufflinks	exon	256132925	256133034	.	+	.	gene_id GRMZM2G150772; transcript_id TCONS_00002838; exon_number 2; oId CUFF.4831.1; tss_id TSS2551;
1	Cufflinks	exon	256133358	256133523	.	+	.	gene_id GRMZM2G150772; transcript_id TCONS_00002838; exon_number 3; oId CUFF.4831.1; tss_id TSS2551;
1	Cufflinks	exon	256133645	256133745	.	+	.	gene_id GRMZM2G150772; transcript_id TCONS_00002838; exon_number 4; oId CUFF.4831.1; tss_id TSS2551;
1	Cufflinks	exon	256133838	256133910	.	+	.	gene_id GRMZM2G150772; transcript_id TCONS_00002838; exon_number 5; oId CUFF.4831.1; tss_id TSS2551;
1	Cufflinks	exon	256133994	256134135	.	+	.	gene_id GRMZM2G150772; transcript_id TCONS_00002838; exon_number 6; oId CUFF.4831.1; tss_id TSS2551;
1	Cufflinks	exon	256134660	256134820	.	+	.	gene_id GRMZM2G150772; transcript_id TCONS_00002838; exon_number 7; oId CUFF.4831.1; tss_id TSS2551;
1	Cufflinks	exon	256134997	256135121	.	+	.	gene_id GRMZM2G150772; transcript_id TCONS_00002838; exon_number 8; oId CUFF.4831.1; tss_id TSS2551;
1	Cufflinks	exon	256135207	256135306	.	+	.	gene_id GRMZM2G150772; transcript_id TCONS_00002838; exon_number 9; oId CUFF.4831.1; tss_id TSS2551;
1	Cufflinks	exon	256135548	256135644	.	+	.	gene_id GRMZM2G150772; transcript_id TCONS_00002838; exon_number 10; oId CUFF.4831.1; tss_id TSS2551;
1	Cufflinks	exon	256135803	256135854	.	+	.	gene_id GRMZM2G150772; transcript_id TCONS_00002838; exon_number 11; oId CUFF.4831.1; tss_id TSS2551;
1	Cufflinks	exon	256136245	256136346	.	+	.	gene_id GRMZM2G150772; transcript_id TCONS_00002838; exon_number 12; oId CUFF.4831.1; tss_id TSS2551;

1	Cufflinks	exon	256136428	256136574	.	+	.	gene_id GRMZM2G150772; transcript_id TCONS_00002838; exon_number 13; oId CUFF.4831.1; tss_id TSS2551;
1	Cufflinks	exon	256136654	256136825	.	+	.	gene_id GRMZM2G150772; transcript_id TCONS_00002838; exon_number 14; oId CUFF.4831.1; tss_id TSS2551;
1	Cufflinks	exon	256137061	256137306	.	+	.	gene_id GRMZM2G150772; transcript_id TCONS_00002838; exon_number 15; oId CUFF.4831.1; tss_id TSS2551;
1	Cufflinks	exon	256137470	256137624	.	+	.	gene_id GRMZM2G150772; transcript_id TCONS_00002838; exon_number 16; oId CUFF.4831.1; tss_id TSS2551;
1	Cufflinks	exon	256138193	256138321	.	+	.	gene_id GRMZM2G150772; transcript_id TCONS_00002838; exon_number 17; oId CUFF.4831.1; tss_id TSS2551;
1	Cufflinks	exon	256138435	256138552	.	+	.	gene_id GRMZM2G150772; transcript_id TCONS_00002838; exon_number 18; oId CUFF.4831.1; tss_id TSS2551;
1	Cufflinks	exon	256138686	256138881	.	+	.	gene_id GRMZM2G150772; transcript_id TCONS_00002838; exon_number 19; oId CUFF.4831.1; tss_id TSS2551;
1	Cufflinks	exon	256139078	256139189	.	+	.	gene_id GRMZM2G150772; transcript_id TCONS_00002838; exon_number 20; oId CUFF.4831.1; tss_id TSS2551;
1	Cufflinks	exon	256139263	256139417	.	+	.	gene_id GRMZM2G150772; transcript_id TCONS_00002838; exon_number 21; oId CUFF.4831.1; tss_id TSS2551;
1	Cufflinks	exon	256142454	256143897	.	+	.	gene_id GRMZM2G150772; transcript_id TCONS_00002838; exon_number 22; oId CUFF.4831.1; tss_id TSS2551;
1	Cufflinks	exon	256143978	256144172	.	+	.	gene_id GRMZM2G150772; transcript_id TCONS_00002838; exon_number 23; oId CUFF.4831.1; tss_id TSS2551;
1	Cufflinks	exon	256144298	256145103	.	+	.	gene_id GRMZM2G150772; transcript_id TCONS_00002838; exon_number 24; oId CUFF.4831.1; tss_id TSS2551;
1	Cufflinks	exon	258031281	258032086	.	+	.	gene_id GRMZM2G055037; transcript_id TCONS_00002861; exon_number 1; oId CUFF.4852.1; tss_id TSS2572;
1	Cufflinks	exon	258032234	258033115	.	+	.	gene_id GRMZM2G055037; transcript_id TCONS_00002861; exon_number 2; oId CUFF.4852.1; tss_id TSS2572;
1	Cufflinks	exon	264275003	264275455	.	+	.	gene_id GRMZM2G109056; transcript_id TCONS_00002954; exon_number 1; oId CUFF.5046.1; tss_id TSS2654;
1	Cufflinks	exon	264276096	264276385	.	+	.	gene_id GRMZM2G109056; transcript_id TCONS_00002954; exon_number 2; oId CUFF.5046.1; tss_id TSS2654;
1	Cufflinks	exon	264287576	264287819	.	+	.	gene_id GRMZM2G109056; transcript_id TCONS_00002954; exon_number 3; oId CUFF.5046.1; tss_id TSS2654;
1	Cufflinks	exon	264287912	264288256	.	+	.	gene_id GRMZM2G109056; transcript_id TCONS_00002954; exon_number 4; oId CUFF.5046.1; tss_id TSS2654;
1	Cufflinks	exon	264288355	264288461	.	+	.	gene_id GRMZM2G109056; transcript_id TCONS_00002954; exon_number 5; oId CUFF.5046.1; tss_id TSS2654;
1	Cufflinks	exon	264288564	264288671	.	+	.	gene_id GRMZM2G109056; transcript_id TCONS_00002954; exon_number 6; oId CUFF.5046.1; tss_id TSS2654;
1	Cufflinks	exon	264288771	264289078	.	+	.	gene_id GRMZM2G109056; transcript_id TCONS_00002954; exon_number 7; oId CUFF.5046.1; tss_id TSS2654;
1	Cufflinks	exon	264289189	264289452	.	+	.	gene_id GRMZM2G109056; transcript_id TCONS_00002954; exon_number 8; oId CUFF.5046.1; tss_id TSS2654;
1	Cufflinks	exon	264289555	264290616	.	+	.	gene_id GRMZM2G109056; transcript_id TCONS_00002954; exon_number 9; oId CUFF.5046.1; tss_id TSS2654;
1	Cufflinks	exon	271178054	271178326	.	+	.	gene_id GRMZM2G153292; transcript_id TCONS_00003053; exon_number 1; oId CUFF.5199.1; tss_id TSS2745;
1	Cufflinks	exon	271179191	271179425	.	+	.	gene_id GRMZM2G153292; transcript_id TCONS_00003053; exon_number 2; oId CUFF.5199.1; tss_id TSS2745;
1	Cufflinks	exon	271179519	271179889	.	+	.	gene_id GRMZM2G153292; transcript_id TCONS_00003053; exon_number 3; oId CUFF.5199.1; tss_id TSS2745;
1	Cufflinks	exon	271179995	271180939	.	+	.	gene_id GRMZM2G153292; transcript_id TCONS_00003053; exon_number 4; oId CUFF.5199.1; tss_id TSS2745;
1	Cufflinks	exon	274785474	274785912	.	+	.	gene_id GRMZM2G001869; transcript_id TCONS_00003103; exon_number 1; oId CUFF.5298.1; tss_id TSS2793;
1	Cufflinks	exon	274789098	274789226	.	+	.	gene_id GRMZM2G001869; transcript_id TCONS_00003103; exon_number 2; oId CUFF.5298.1; tss_id TSS2793;
1	Cufflinks	exon	274789463	274789585	.	+	.	gene_id GRMZM2G001869; transcript_id TCONS_00003103; exon_number 3; oId CUFF.5298.1; tss_id TSS2793;
1	Cufflinks	exon	274789862	274790748	.	+	.	gene_id GRMZM2G001869; transcript_id TCONS_00003103; exon_number 4; oId CUFF.5298.1; tss_id TSS2793;
1	Cufflinks	exon	275759900	275760808	.	+	.	gene_id XLOC_002707; transcript_id TCONS_00003128; exon_number 1; oId CUFF.5334.1; tss_id TSS2817;
1	Cufflinks	exon	275760924	275761423	.	+	.	gene_id XLOC_002707; transcript_id TCONS_00003128; exon_number 2; oId CUFF.5334.1; tss_id TSS2817;
1	Cufflinks	exon	276372686	276373546	.	+	.	gene_id GRMZM2G147687; transcript_id TCONS_00003142; exon_number 1; oId CUFF.5394.3; tss_id TSS2828;
1	Cufflinks	exon	276373971	276374240	.	+	.	gene_id GRMZM2G147687; transcript_id TCONS_00003142; exon_number 2; oId CUFF.5394.3; tss_id TSS2828;
1	Cufflinks	exon	276374342	276374546	.	+	.	gene_id GRMZM2G147687; transcript_id TCONS_00003142; exon_number 3; oId CUFF.5394.3; tss_id TSS2828;

1	Cufflinks	exon	276374674	276374764	.	+	.	gene_id GRMZM2G147687; transcript_id TCONS_00003142; exon_number 4; oId CUFF.5394.3; tss_id TSS2828;
1	Cufflinks	exon	276374875	276375017	.	+	.	gene_id GRMZM2G147687; transcript_id TCONS_00003142; exon_number 5; oId CUFF.5394.3; tss_id TSS2828;
1	Cufflinks	exon	276375160	276375403	.	+	.	gene_id GRMZM2G147687; transcript_id TCONS_00003142; exon_number 6; oId CUFF.5394.3; tss_id TSS2828;
1	Cufflinks	exon	276375498	276375602	.	+	.	gene_id GRMZM2G147687; transcript_id TCONS_00003142; exon_number 7; oId CUFF.5394.3; tss_id TSS2828;
1	Cufflinks	exon	276375910	276376092	.	+	.	gene_id GRMZM2G147687; transcript_id TCONS_00003142; exon_number 8; oId CUFF.5394.3; tss_id TSS2828;
1	Cufflinks	exon	276376179	276376380	.	+	.	gene_id GRMZM2G147687; transcript_id TCONS_00003142; exon_number 9; oId CUFF.5394.3; tss_id TSS2828;
1	Cufflinks	exon	276376490	276377276	.	+	.	gene_id GRMZM2G147687; transcript_id TCONS_00003142; exon_number 10; oId CUFF.5394.3; tss_id TSS2828;
1	Cufflinks	exon	276390522	276393121	.	+	.	gene_id GRMZM2G017186; transcript_id TCONS_00003143; exon_number 1; oId CUFF.5382.1; tss_id TSS2829;
1	Cufflinks	exon	276393220	276393310	.	+	.	gene_id GRMZM2G017186; transcript_id TCONS_00003143; exon_number 2; oId CUFF.5382.1; tss_id TSS2829;
1	Cufflinks	exon	276393412	276393557	.	+	.	gene_id GRMZM2G017186; transcript_id TCONS_00003143; exon_number 3; oId CUFF.5382.1; tss_id TSS2829;
1	Cufflinks	exon	276393644	276393887	.	+	.	gene_id GRMZM2G017186; transcript_id TCONS_00003143; exon_number 4; oId CUFF.5382.1; tss_id TSS2829;
1	Cufflinks	exon	276393965	276394069	.	+	.	gene_id GRMZM2G017186; transcript_id TCONS_00003143; exon_number 5; oId CUFF.5382.1; tss_id TSS2829;
1	Cufflinks	exon	276394149	276395246	.	+	.	gene_id GRMZM2G017186; transcript_id TCONS_00003143; exon_number 6; oId CUFF.5382.1; tss_id TSS2829;
1	Cufflinks	exon	276876174	276876392	.	+	.	gene_id GRMZM2G120484; transcript_id TCONS_00003156; exon_number 1; oId CUFF.5408.1; tss_id TSS2840;
1	Cufflinks	exon	276876518	276876588	.	+	.	gene_id GRMZM2G120484; transcript_id TCONS_00003156; exon_number 2; oId CUFF.5408.1; tss_id TSS2840;
1	Cufflinks	exon	276876693	276876775	.	+	.	gene_id GRMZM2G120484; transcript_id TCONS_00003156; exon_number 3; oId CUFF.5408.1; tss_id TSS2840;
1	Cufflinks	exon	276876869	276876912	.	+	.	gene_id GRMZM2G120484; transcript_id TCONS_00003156; exon_number 4; oId CUFF.5408.1; tss_id TSS2840;
1	Cufflinks	exon	276877058	276877553	.	+	.	gene_id GRMZM2G120484; transcript_id TCONS_00003156; exon_number 5; oId CUFF.5408.1; tss_id TSS2840;
1	Cufflinks	exon	276877927	276879722	.	+	.	gene_id GRMZM2G120484; transcript_id TCONS_00003156; exon_number 6; oId CUFF.5408.1; tss_id TSS2840;
1	Cufflinks	exon	276880015	276881240	.	+	.	gene_id GRMZM2G120484; transcript_id TCONS_00003156; exon_number 7; oId CUFF.5408.1; tss_id TSS2840;
1	Cufflinks	exon	276881383	276883112	.	+	.	gene_id GRMZM2G120484; transcript_id TCONS_00003156; exon_number 8; oId CUFF.5408.1; tss_id TSS2840;
1	Cufflinks	exon	277805999	277806330	.	+	.	gene_id GRMZM2G090422; transcript_id TCONS_00003179; exon_number 1; oId CUFF.5404.1; tss_id TSS2858;
1	Cufflinks	exon	277806430	277806546	.	+	.	gene_id GRMZM2G090422; transcript_id TCONS_00003179; exon_number 2; oId CUFF.5404.1; tss_id TSS2858;
1	Cufflinks	exon	277806643	277806730	.	+	.	gene_id GRMZM2G090422; transcript_id TCONS_00003179; exon_number 3; oId CUFF.5404.1; tss_id TSS2858;
1	Cufflinks	exon	277807464	277807838	.	+	.	gene_id GRMZM2G090422; transcript_id TCONS_00003179; exon_number 4; oId CUFF.5404.1; tss_id TSS2858;
1	Cufflinks	exon	278196221	278196428	.	+	.	gene_id GRMZM2G328500; transcript_id TCONS_00003189; exon_number 1; oId CUFF.5427.1; tss_id TSS2867;
1	Cufflinks	exon	278197431	278199359	.	+	.	gene_id GRMZM2G328500; transcript_id TCONS_00003189; exon_number 2; oId CUFF.5427.1; tss_id TSS2867;
1	Cufflinks	exon	280356771	280357243	.	+	.	gene_id GRMZM2G138861; transcript_id TCONS_00003223; exon_number 1; oId CUFF.5504.1; tss_id TSS2896;
1	Cufflinks	exon	280359861	280359935	.	+	.	gene_id GRMZM2G138861; transcript_id TCONS_00003223; exon_number 2; oId CUFF.5504.1; tss_id TSS2896;
1	Cufflinks	exon	280360013	280360114	.	+	.	gene_id GRMZM2G138861; transcript_id TCONS_00003223; exon_number 3; oId CUFF.5504.1; tss_id TSS2896;
1	Cufflinks	exon	280360198	280360251	.	+	.	gene_id GRMZM2G138861; transcript_id TCONS_00003223; exon_number 4; oId CUFF.5504.1; tss_id TSS2896;
1	Cufflinks	exon	280360350	280360442	.	+	.	gene_id GRMZM2G138861; transcript_id TCONS_00003223; exon_number 5; oId CUFF.5504.1; tss_id TSS2896;
1	Cufflinks	exon	280363334	280363426	.	+	.	gene_id GRMZM2G138861; transcript_id TCONS_00003223; exon_number 6; oId CUFF.5504.1; tss_id TSS2896;
1	Cufflinks	exon	280363642	280363746	.	+	.	gene_id GRMZM2G138861; transcript_id TCONS_00003223; exon_number 7; oId CUFF.5504.1; tss_id TSS2896;
1	Cufflinks	exon	280363951	280364049	.	+	.	gene_id GRMZM2G138861; transcript_id TCONS_00003223; exon_number 8; oId CUFF.5504.1; tss_id TSS2896;
1	Cufflinks	exon	280364172	280364944	.	+	.	gene_id GRMZM2G138861; transcript_id TCONS_00003223; exon_number 9; oId CUFF.5504.1; tss_id TSS2896;

1	Cufflinks	exon	285281158	285281565	.	+	.	gene_id XLOC_002839; transcript_id TCONS_00003284; exon_number 1; oId CUFF.5621.1; tss_id TSS2954;
1	Cufflinks	exon	287217889	287218154	.	+	.	gene_id XLOC_002873; transcript_id TCONS_00003322; exon_number 1; oId CUFF.5683.1; tss_id TSS2988;
1	Cufflinks	exon	287541494	287542191	.	+	.	gene_id GRMZM2G170851; transcript_id TCONS_00003328; exon_number 1; oId CUFF.5695.1; tss_id TSS2994;
1	Cufflinks	exon	287543593	287544013	.	+	.	gene_id GRMZM2G170851; transcript_id TCONS_00003328; exon_number 2; oId CUFF.5695.1; tss_id TSS2994;
1	Cufflinks	exon	287653653	287655436	.	+	.	gene_id XLOC_002882; transcript_id TCONS_00003331; exon_number 1; oId CUFF.5699.1; tss_id TSS2997;
1	Cufflinks	exon	289403622	289405770	.	+	.	gene_id GRMZM2G137321; transcript_id TCONS_00003365; exon_number 1; oId CUFF.5760.1; tss_id TSS3028;
1	Cufflinks	exon	290337450	290337742	.	+	.	gene_id GRMZM2G082664; transcript_id TCONS_00003379; exon_number 1; oId CUFF.5816.1; tss_id TSS3041;
1	Cufflinks	exon	290338047	290338108	.	+	.	gene_id GRMZM2G082664; transcript_id TCONS_00003379; exon_number 2; oId CUFF.5816.1; tss_id TSS3041;
1	Cufflinks	exon	290338207	290338260	.	+	.	gene_id GRMZM2G082664; transcript_id TCONS_00003379; exon_number 3; oId CUFF.5816.1; tss_id TSS3041;
1	Cufflinks	exon	290338755	290338791	.	+	.	gene_id GRMZM2G082664; transcript_id TCONS_00003379; exon_number 4; oId CUFF.5816.1; tss_id TSS3041;
1	Cufflinks	exon	290338866	290338899	.	+	.	gene_id GRMZM2G082664; transcript_id TCONS_00003379; exon_number 5; oId CUFF.5816.1; tss_id TSS3041;
1	Cufflinks	exon	290339356	290339405	.	+	.	gene_id GRMZM2G082664; transcript_id TCONS_00003379; exon_number 6; oId CUFF.5816.1; tss_id TSS3041;
1	Cufflinks	exon	290339507	290339551	.	+	.	gene_id GRMZM2G082664; transcript_id TCONS_00003379; exon_number 7; oId CUFF.5816.1; tss_id TSS3041;
1	Cufflinks	exon	290339688	290339761	.	+	.	gene_id GRMZM2G082664; transcript_id TCONS_00003379; exon_number 8; oId CUFF.5816.1; tss_id TSS3041;
1	Cufflinks	exon	290340528	290340597	.	+	.	gene_id GRMZM2G082664; transcript_id TCONS_00003379; exon_number 9; oId CUFF.5816.1; tss_id TSS3041;
1	Cufflinks	exon	290340676	290341074	.	+	.	gene_id GRMZM2G082664; transcript_id TCONS_00003379; exon_number 10; oId CUFF.5816.1; tss_id TSS3041;
1	Cufflinks	exon	290356082	290356142	.	+	.	gene_id GRMZM2G082664; transcript_id TCONS_00003379; exon_number 11; oId CUFF.5816.1; tss_id TSS3041;
1	Cufflinks	exon	290356224	290356268	.	+	.	gene_id GRMZM2G082664; transcript_id TCONS_00003379; exon_number 12; oId CUFF.5816.1; tss_id TSS3041;
1	Cufflinks	exon	290356384	290356467	.	+	.	gene_id GRMZM2G082664; transcript_id TCONS_00003379; exon_number 13; oId CUFF.5816.1; tss_id TSS3041;
1	Cufflinks	exon	290357464	290357540	.	+	.	gene_id GRMZM2G082664; transcript_id TCONS_00003379; exon_number 14; oId CUFF.5816.1; tss_id TSS3041;
1	Cufflinks	exon	290357622	290357684	.	+	.	gene_id GRMZM2G082664; transcript_id TCONS_00003379; exon_number 15; oId CUFF.5816.1; tss_id TSS3041;
1	Cufflinks	exon	290357941	290358011	.	+	.	gene_id GRMZM2G082664; transcript_id TCONS_00003379; exon_number 16; oId CUFF.5816.1; tss_id TSS3041;
1	Cufflinks	exon	290358109	290358169	.	+	.	gene_id GRMZM2G082664; transcript_id TCONS_00003379; exon_number 17; oId CUFF.5816.1; tss_id TSS3041;
1	Cufflinks	exon	290358259	290358319	.	+	.	gene_id GRMZM2G082664; transcript_id TCONS_00003379; exon_number 18; oId CUFF.5816.1; tss_id TSS3041;
1	Cufflinks	exon	290358398	290358478	.	+	.	gene_id GRMZM2G082664; transcript_id TCONS_00003379; exon_number 19; oId CUFF.5816.1; tss_id TSS3041;
1	Cufflinks	exon	290358592	290359023	.	+	.	gene_id GRMZM2G082664; transcript_id TCONS_00003379; exon_number 20; oId CUFF.5816.1; tss_id TSS3041;
1	Cufflinks	exon	290877819	290878895	.	+	.	gene_id XLOC_002940; transcript_id TCONS_00003396; exon_number 1; oId CUFF.5822.1; tss_id TSS3055;
1	Cufflinks	exon	290879005	290880168	.	+	.	gene_id XLOC_002940; transcript_id TCONS_00003396; exon_number 2; oId CUFF.5822.1; tss_id TSS3055;
1	Cufflinks	exon	292594168	292594772	.	+	.	gene_id XLOC_002967; transcript_id TCONS_00003427; exon_number 1; oId CUFF.5885.1; tss_id TSS3085;
1	Cufflinks	exon	292594933	292595450	.	+	.	gene_id XLOC_002967; transcript_id TCONS_00003427; exon_number 2; oId CUFF.5885.1; tss_id TSS3085;
1	Cufflinks	exon	293065790	293066965	.	+	.	gene_id GRMZM2G155580; transcript_id TCONS_00003431; exon_number 1; oId CUFF.5912.1; tss_id TSS3089;
1	Cufflinks	exon	293071182	293071415	.	+	.	gene_id GRMZM2G155580; transcript_id TCONS_00003431; exon_number 2; oId CUFF.5912.1; tss_id TSS3089;
1	Cufflinks	exon	293071537	293071863	.	+	.	gene_id GRMZM2G155580; transcript_id TCONS_00003431; exon_number 3; oId CUFF.5912.1; tss_id TSS3089;
1	Cufflinks	exon	293073950	293074538	.	+	.	gene_id GRMZM2G155580; transcript_id TCONS_00003431; exon_number 4; oId CUFF.5912.1; tss_id TSS3089;
1	Cufflinks	exon	295097680	295098932	.	+	.	gene_id GRMZM2G139041; transcript_id TCONS_00003473; exon_number 1; oId CUFF.5969.1; tss_id TSS3127;
1	Cufflinks	exon	295099424	295099600	.	+	.	gene_id GRMZM2G139041; transcript_id TCONS_00003473; exon_number 2; oId CUFF.5969.1; tss_id TSS3127;

1	Cufflinks	exon	295099734	295100439	.	+	.	gene_id GRMZM2G139041; transcript_id TCONS_00003473; exon_number 3; oId CUFF.5969.1; tss_id TSS3127;
1	Cufflinks	exon	296847943	296850494	.	+	.	gene_id GRMZM2G470422; transcript_id TCONS_00003513; exon_number 1; oId CUFF.6028.1; tss_id TSS3162;
1	Cufflinks	exon	298813813	298814661	.	+	.	gene_id GRMZM5G876146; transcript_id TCONS_00003574; exon_number 1; oId CUFF.6116.1; tss_id TSS3211;
1	Cufflinks	exon	298815063	298815176	.	+	.	gene_id GRMZM5G876146; transcript_id TCONS_00003574; exon_number 2; oId CUFF.6116.1; tss_id TSS3211;
1	Cufflinks	exon	298816144	298816275	.	+	.	gene_id GRMZM5G876146; transcript_id TCONS_00003574; exon_number 3; oId CUFF.6116.1; tss_id TSS3211;
1	Cufflinks	exon	298816484	298817252	.	+	.	gene_id GRMZM5G876146; transcript_id TCONS_00003574; exon_number 4; oId CUFF.6116.1; tss_id TSS3211;
1	Cufflinks	exon	299272391	299272985	.	+	.	gene_id GRMZM2G115456; transcript_id TCONS_00003584; exon_number 1; oId CUFF.6161.1; tss_id TSS3221;
1	Cufflinks	exon	299275812	299278057	.	+	.	gene_id GRMZM2G115456; transcript_id TCONS_00003584; exon_number 2; oId CUFF.6161.1; tss_id TSS3221;
1	Cufflinks	exon	299981775	299982624	.	+	.	gene_id XLOC_003104; transcript_id TCONS_00003594; exon_number 1; oId CUFF.6156.1; tss_id TSS3230;
1	Cufflinks	exon	300821132	300823017	.	+	.	gene_id GRMZM2G115162; transcript_id TCONS_00003610; exon_number 1; oId CUFF.6194.1; tss_id TSS3244;
1	Cufflinks	exon	300823091	300824570	.	+	.	gene_id GRMZM2G115162; transcript_id TCONS_00003610; exon_number 2; oId CUFF.6194.1; tss_id TSS3244;
1	Cufflinks	exon	6213707	6214140	.	-	.	gene_id GRMZM2G032409; transcript_id TCONS_00003759; exon_number 1; oId CUFF.253.1; tss_id TSS3378;
1	Cufflinks	exon	6214285	6214509	.	-	.	gene_id GRMZM2G032409; transcript_id TCONS_00003759; exon_number 2; oId CUFF.253.1; tss_id TSS3378;
1	Cufflinks	exon	6215166	6215324	.	-	.	gene_id GRMZM2G032409; transcript_id TCONS_00003759; exon_number 3; oId CUFF.253.1; tss_id TSS3378;
1	Cufflinks	exon	6215637	6215726	.	-	.	gene_id GRMZM2G032409; transcript_id TCONS_00003759; exon_number 4; oId CUFF.253.1; tss_id TSS3378;
1	Cufflinks	exon	6215806	6215874	.	-	.	gene_id GRMZM2G032409; transcript_id TCONS_00003759; exon_number 5; oId CUFF.253.1; tss_id TSS3378;
1	Cufflinks	exon	6216124	6216300	.	-	.	gene_id GRMZM2G032409; transcript_id TCONS_00003759; exon_number 6; oId CUFF.253.1; tss_id TSS3378;
1	Cufflinks	exon	6216379	6216526	.	-	.	gene_id GRMZM2G032409; transcript_id TCONS_00003759; exon_number 7; oId CUFF.253.1; tss_id TSS3378;
1	Cufflinks	exon	6216630	6217258	.	-	.	gene_id GRMZM2G032409; transcript_id TCONS_00003759; exon_number 8; oId CUFF.253.1; tss_id TSS3378;
1	Cufflinks	exon	6218010	6218054	.	-	.	gene_id GRMZM2G032409; transcript_id TCONS_00003759; exon_number 9; oId CUFF.253.1; tss_id TSS3378;
1	Cufflinks	exon	6218164	6218417	.	-	.	gene_id GRMZM2G032409; transcript_id TCONS_00003759; exon_number 10; oId CUFF.253.1; tss_id TSS3378;
1	Cufflinks	exon	7560304	7561013	.	-	.	gene_id XLOC_003266; transcript_id TCONS_00003783; exon_number 1; oId CUFF.277.1; tss_id TSS3400;
1	Cufflinks	exon	10069416	10069760	.	-	.	gene_id XLOC_003308; transcript_id TCONS_00003829; exon_number 1; oId CUFF.367.1; tss_id TSS3444;
1	Cufflinks	exon	12251757	12253695	.	-	.	gene_id GRMZM2G472266; transcript_id TCONS_00003865; exon_number 1; oId CUFF.434.1; tss_id TSS3478;
1	Cufflinks	exon	14958183	14958918	.	-	.	gene_id GRMZM2G139963; transcript_id TCONS_00003908; exon_number 1; oId CUFF.524.1; tss_id TSS3517;
1	Cufflinks	exon	14960067	14960470	.	-	.	gene_id GRMZM2G139963; transcript_id TCONS_00003908; exon_number 2; oId CUFF.524.1; tss_id TSS3517;
1	Cufflinks	exon	14960575	14960872	.	-	.	gene_id GRMZM2G139963; transcript_id TCONS_00003908; exon_number 3; oId CUFF.524.1; tss_id TSS3517;
1	Cufflinks	exon	19326432	19326981	.	-	.	gene_id GRMZM2G154007; transcript_id TCONS_00003970; exon_number 1; oId CUFF.651.1; tss_id TSS3567;
1	Cufflinks	exon	19327135	19327230	.	-	.	gene_id GRMZM2G154007; transcript_id TCONS_00003970; exon_number 2; oId CUFF.651.1; tss_id TSS3567;
1	Cufflinks	exon	19327326	19327390	.	-	.	gene_id GRMZM2G154007; transcript_id TCONS_00003970; exon_number 3; oId CUFF.651.1; tss_id TSS3567;
1	Cufflinks	exon	19327563	19327638	.	-	.	gene_id GRMZM2G154007; transcript_id TCONS_00003970; exon_number 4; oId CUFF.651.1; tss_id TSS3567;
1	Cufflinks	exon	19327774	19327856	.	-	.	gene_id GRMZM2G154007; transcript_id TCONS_00003970; exon_number 5; oId CUFF.651.1; tss_id TSS3567;
1	Cufflinks	exon	19327973	19328298	.	-	.	gene_id GRMZM2G154007; transcript_id TCONS_00003970; exon_number 6; oId CUFF.651.1; tss_id TSS3567;
1	Cufflinks	exon	19328430	19328473	.	-	.	gene_id GRMZM2G154007; transcript_id TCONS_00003970; exon_number 7; oId CUFF.651.1; tss_id TSS3567;
1	Cufflinks	exon	19328630	19328766	.	-	.	gene_id GRMZM2G154007; transcript_id TCONS_00003970; exon_number 8; oId CUFF.651.1; tss_id TSS3567;
1	Cufflinks	exon	19328909	19329023	.	-	.	gene_id GRMZM2G154007; transcript_id TCONS_00003970; exon_number 9; oId CUFF.651.1; tss_id TSS3567;

1	Cufflinks	exon	22378485	22379132	.	-	.	gene_id GRMZM2G006977; transcript_id TCONS_00004010; exon_number 1; oId CUFF.737.1; tss_id TSS3603;
1	Cufflinks	exon	22379254	22379552	.	-	.	gene_id GRMZM2G006977; transcript_id TCONS_00004010; exon_number 2; oId CUFF.737.1; tss_id TSS3603;
1	Cufflinks	exon	22379686	22379857	.	-	.	gene_id GRMZM2G006977; transcript_id TCONS_00004010; exon_number 3; oId CUFF.737.1; tss_id TSS3603;
1	Cufflinks	exon	22380144	22380302	.	-	.	gene_id GRMZM2G006977; transcript_id TCONS_00004010; exon_number 4; oId CUFF.737.1; tss_id TSS3603;
1	Cufflinks	exon	22380401	22382352	.	-	.	gene_id GRMZM2G006977; transcript_id TCONS_00004010; exon_number 5; oId CUFF.737.1; tss_id TSS3603;
1	Cufflinks	exon	22382451	22382505	.	-	.	gene_id GRMZM2G006977; transcript_id TCONS_00004010; exon_number 6; oId CUFF.737.1; tss_id TSS3603;
1	Cufflinks	exon	22383909	22384451	.	-	.	gene_id GRMZM2G006977; transcript_id TCONS_00004010; exon_number 7; oId CUFF.737.1; tss_id TSS3603;
1	Cufflinks	exon	22643460	22643937	.	-	.	gene_id GRMZM2G055172; transcript_id TCONS_00004014; exon_number 1; oId CUFF.739.1; tss_id TSS3607;
1	Cufflinks	exon	22644038	22644113	.	-	.	gene_id GRMZM2G055172; transcript_id TCONS_00004014; exon_number 2; oId CUFF.739.1; tss_id TSS3607;
1	Cufflinks	exon	22644229	22644318	.	-	.	gene_id GRMZM2G055172; transcript_id TCONS_00004014; exon_number 3; oId CUFF.739.1; tss_id TSS3607;
1	Cufflinks	exon	22644417	22644640	.	-	.	gene_id GRMZM2G055172; transcript_id TCONS_00004014; exon_number 4; oId CUFF.739.1; tss_id TSS3607;
1	Cufflinks	exon	22644871	22644940	.	-	.	gene_id GRMZM2G055172; transcript_id TCONS_00004014; exon_number 5; oId CUFF.739.1; tss_id TSS3607;
1	Cufflinks	exon	22645348	22645394	.	-	.	gene_id GRMZM2G055172; transcript_id TCONS_00004014; exon_number 6; oId CUFF.739.1; tss_id TSS3607;
1	Cufflinks	exon	22645481	22645656	.	-	.	gene_id GRMZM2G055172; transcript_id TCONS_00004014; exon_number 7; oId CUFF.739.1; tss_id TSS3607;
1	Cufflinks	exon	22646337	22646429	.	-	.	gene_id GRMZM2G055172; transcript_id TCONS_00004014; exon_number 8; oId CUFF.739.1; tss_id TSS3607;
1	Cufflinks	exon	22646524	22647118	.	-	.	gene_id GRMZM2G055172; transcript_id TCONS_00004014; exon_number 9; oId CUFF.739.1; tss_id TSS3607;
1	Cufflinks	exon	24403021	24403868	.	-	.	gene_id GRMZM2G159632; transcript_id TCONS_00004030; exon_number 1; oId CUFF.783.1; tss_id TSS3620;
1	Cufflinks	exon	24404001	24404077	.	-	.	gene_id GRMZM2G159632; transcript_id TCONS_00004030; exon_number 2; oId CUFF.783.1; tss_id TSS3620;
1	Cufflinks	exon	24404167	24404252	.	-	.	gene_id GRMZM2G159632; transcript_id TCONS_00004030; exon_number 3; oId CUFF.783.1; tss_id TSS3620;
1	Cufflinks	exon	24404515	24404705	.	-	.	gene_id GRMZM2G159632; transcript_id TCONS_00004030; exon_number 4; oId CUFF.783.1; tss_id TSS3620;
1	Cufflinks	exon	24405060	24405346	.	-	.	gene_id GRMZM2G159632; transcript_id TCONS_00004030; exon_number 5; oId CUFF.783.1; tss_id TSS3620;
1	Cufflinks	exon	24405430	24405556	.	-	.	gene_id GRMZM2G159632; transcript_id TCONS_00004030; exon_number 6; oId CUFF.783.1; tss_id TSS3620;
1	Cufflinks	exon	24405682	24405798	.	-	.	gene_id GRMZM2G159632; transcript_id TCONS_00004030; exon_number 7; oId CUFF.783.1; tss_id TSS3620;
1	Cufflinks	exon	24405909	24406022	.	-	.	gene_id GRMZM2G159632; transcript_id TCONS_00004030; exon_number 8; oId CUFF.783.1; tss_id TSS3620;
1	Cufflinks	exon	24406117	24406182	.	-	.	gene_id GRMZM2G159632; transcript_id TCONS_00004030; exon_number 9; oId CUFF.783.1; tss_id TSS3620;
1	Cufflinks	exon	24406287	24406461	.	-	.	gene_id GRMZM2G159632; transcript_id TCONS_00004030; exon_number 10; oId CUFF.783.1; tss_id TSS3620;
1	Cufflinks	exon	24406558	24406765	.	-	.	gene_id GRMZM2G159632; transcript_id TCONS_00004030; exon_number 11; oId CUFF.783.1; tss_id TSS3620;
1	Cufflinks	exon	24406860	24406908	.	-	.	gene_id GRMZM2G159632; transcript_id TCONS_00004030; exon_number 12; oId CUFF.783.1; tss_id TSS3620;
1	Cufflinks	exon	24406978	24407601	.	-	.	gene_id GRMZM2G159632; transcript_id TCONS_00004030; exon_number 13; oId CUFF.783.1; tss_id TSS3620;
1	Cufflinks	exon	24403021	24403868	.	-	.	gene_id GRMZM2G159632; transcript_id TCONS_00004032; exon_number 1; oId CUFF.783.3; tss_id TSS3621;
1	Cufflinks	exon	24404001	24404077	.	-	.	gene_id GRMZM2G159632; transcript_id TCONS_00004032; exon_number 2; oId CUFF.783.3; tss_id TSS3621;
1	Cufflinks	exon	24404167	24404252	.	-	.	gene_id GRMZM2G159632; transcript_id TCONS_00004032; exon_number 3; oId CUFF.783.3; tss_id TSS3621;
1	Cufflinks	exon	24404515	24404705	.	-	.	gene_id GRMZM2G159632; transcript_id TCONS_00004032; exon_number 4; oId CUFF.783.3; tss_id TSS3621;
1	Cufflinks	exon	24405060	24405346	.	-	.	gene_id GRMZM2G159632; transcript_id TCONS_00004032; exon_number 5; oId CUFF.783.3; tss_id TSS3621;
1	Cufflinks	exon	24405430	24405556	.	-	.	gene_id GRMZM2G159632; transcript_id TCONS_00004032; exon_number 6; oId CUFF.783.3; tss_id TSS3621;
1	Cufflinks	exon	24405682	24405798	.	-	.	gene_id GRMZM2G159632; transcript_id TCONS_00004032; exon_number 7; oId CUFF.783.3; tss_id TSS3621;

1	Cufflinks	exon	24405909	24406022	.	-	.	gene_id GRMZM2G159632; transcript_id TCONS_00004032; exon_number 8; oId CUFF.783.3; tss_id TSS3621;
1	Cufflinks	exon	24406117	24406182	.	-	.	gene_id GRMZM2G159632; transcript_id TCONS_00004032; exon_number 9; oId CUFF.783.3; tss_id TSS3621;
1	Cufflinks	exon	24406287	24406461	.	-	.	gene_id GRMZM2G159632; transcript_id TCONS_00004032; exon_number 10; oId CUFF.783.3; tss_id TSS3621;
1	Cufflinks	exon	24406558	24406765	.	-	.	gene_id GRMZM2G159632; transcript_id TCONS_00004032; exon_number 11; oId CUFF.783.3; tss_id TSS3621;
1	Cufflinks	exon	24406860	24406908	.	-	.	gene_id GRMZM2G159632; transcript_id TCONS_00004032; exon_number 12; oId CUFF.783.3; tss_id TSS3621;
1	Cufflinks	exon	24406978	24407366	.	-	.	gene_id GRMZM2G159632; transcript_id TCONS_00004032; exon_number 13; oId CUFF.783.3; tss_id TSS3621;
1	Cufflinks	exon	24407723	24407908	.	-	.	gene_id GRMZM2G159632; transcript_id TCONS_00004032; exon_number 14; oId CUFF.783.3; tss_id TSS3621;
1	Cufflinks	exon	24403021	24403868	.	-	.	gene_id GRMZM2G159632; transcript_id TCONS_00004031; exon_number 1; oId CUFF.783.2; tss_id TSS3621;
1	Cufflinks	exon	24404001	24404077	.	-	.	gene_id GRMZM2G159632; transcript_id TCONS_00004031; exon_number 2; oId CUFF.783.2; tss_id TSS3621;
1	Cufflinks	exon	24404167	24404252	.	-	.	gene_id GRMZM2G159632; transcript_id TCONS_00004031; exon_number 3; oId CUFF.783.2; tss_id TSS3621;
1	Cufflinks	exon	24404515	24404705	.	-	.	gene_id GRMZM2G159632; transcript_id TCONS_00004031; exon_number 4; oId CUFF.783.2; tss_id TSS3621;
1	Cufflinks	exon	24405060	24405346	.	-	.	gene_id GRMZM2G159632; transcript_id TCONS_00004031; exon_number 5; oId CUFF.783.2; tss_id TSS3621;
1	Cufflinks	exon	24405430	24405556	.	-	.	gene_id GRMZM2G159632; transcript_id TCONS_00004031; exon_number 6; oId CUFF.783.2; tss_id TSS3621;
1	Cufflinks	exon	24405682	24405798	.	-	.	gene_id GRMZM2G159632; transcript_id TCONS_00004031; exon_number 7; oId CUFF.783.2; tss_id TSS3621;
1	Cufflinks	exon	24405909	24406022	.	-	.	gene_id GRMZM2G159632; transcript_id TCONS_00004031; exon_number 8; oId CUFF.783.2; tss_id TSS3621;
1	Cufflinks	exon	24406117	24406182	.	-	.	gene_id GRMZM2G159632; transcript_id TCONS_00004031; exon_number 9; oId CUFF.783.2; tss_id TSS3621;
1	Cufflinks	exon	24406287	24406461	.	-	.	gene_id GRMZM2G159632; transcript_id TCONS_00004031; exon_number 10; oId CUFF.783.2; tss_id TSS3621;
1	Cufflinks	exon	24406558	24406765	.	-	.	gene_id GRMZM2G159632; transcript_id TCONS_00004031; exon_number 11; oId CUFF.783.2; tss_id TSS3621;
1	Cufflinks	exon	24406860	24406908	.	-	.	gene_id GRMZM2G159632; transcript_id TCONS_00004031; exon_number 12; oId CUFF.783.2; tss_id TSS3621;
1	Cufflinks	exon	24406978	24407366	.	-	.	gene_id GRMZM2G159632; transcript_id TCONS_00004031; exon_number 13; oId CUFF.783.2; tss_id TSS3621;
1	Cufflinks	exon	24407717	24407908	.	-	.	gene_id GRMZM2G159632; transcript_id TCONS_00004031; exon_number 14; oId CUFF.783.2; tss_id TSS3621;
1	Cufflinks	exon	32873395	32873984	.	-	.	gene_id GRMZM2G077942; transcript_id TCONS_00004140; exon_number 1; oId CUFF.957.1; tss_id TSS3716;
1	Cufflinks	exon	32875510	32875769	.	-	.	gene_id GRMZM2G077942; transcript_id TCONS_00004140; exon_number 2; oId CUFF.957.1; tss_id TSS3716;
1	Cufflinks	exon	32875878	32876087	.	-	.	gene_id GRMZM2G077942; transcript_id TCONS_00004140; exon_number 3; oId CUFF.957.1; tss_id TSS3716;
1	Cufflinks	exon	34974187	34974696	.	-	.	gene_id GRMZM2G138103; transcript_id TCONS_00004166; exon_number 1; oId CUFF.1008.1; tss_id TSS3741;
1	Cufflinks	exon	34974806	34975255	.	-	.	gene_id GRMZM2G138103; transcript_id TCONS_00004166; exon_number 2; oId CUFF.1008.1; tss_id TSS3741;
1	Cufflinks	exon	39115144	39116341	.	-	.	gene_id GRMZM2G160506; transcript_id TCONS_00004228; exon_number 1; oId CUFF.1144.2; tss_id TSS3799;
1	Cufflinks	exon	39116470	39116532	.	-	.	gene_id GRMZM2G160506; transcript_id TCONS_00004228; exon_number 2; oId CUFF.1144.2; tss_id TSS3799;
1	Cufflinks	exon	39116731	39116882	.	-	.	gene_id GRMZM2G160506; transcript_id TCONS_00004228; exon_number 3; oId CUFF.1144.2; tss_id TSS3799;
1	Cufflinks	exon	39117902	39118311	.	-	.	gene_id GRMZM2G160506; transcript_id TCONS_00004228; exon_number 4; oId CUFF.1144.2; tss_id TSS3799;
1	Cufflinks	exon	39115144	39116341	.	-	.	gene_id GRMZM2G160506; transcript_id TCONS_00004227; exon_number 1; oId CUFF.1144.1; tss_id TSS3799;
1	Cufflinks	exon	39116470	39116532	.	-	.	gene_id GRMZM2G160506; transcript_id TCONS_00004227; exon_number 2; oId CUFF.1144.1; tss_id TSS3799;
1	Cufflinks	exon	39116731	39116882	.	-	.	gene_id GRMZM2G160506; transcript_id TCONS_00004227; exon_number 3; oId CUFF.1144.1; tss_id TSS3799;
1	Cufflinks	exon	39117933	39118311	.	-	.	gene_id GRMZM2G160506; transcript_id TCONS_00004227; exon_number 4; oId CUFF.1144.1; tss_id TSS3799;
1	Cufflinks	exon	39144013	39145057	.	-	.	gene_id XLOC_003652; transcript_id TCONS_00004230; exon_number 1; oId CUFF.1116.1; tss_id TSS3800;
1	Cufflinks	exon	39145209	39145544	.	-	.	gene_id XLOC_003652; transcript_id TCONS_00004230; exon_number 2; oId CUFF.1116.1; tss_id TSS3800;

1	Cufflinks	exon	39145644	39146199	.	-	.	gene_id XLOC_003652; transcript_id TCONS_00004230; exon_number 3; oId CUFF.1116.1; tss_id TSS3800;
1	Cufflinks	exon	41440932	41441699	.	-	.	gene_id GRMZM2G043878; transcript_id TCONS_00004270; exon_number 1; oId CUFF.1181.1; tss_id TSS3835;
1	Cufflinks	exon	41441816	41442284	.	-	.	gene_id GRMZM2G043878; transcript_id TCONS_00004270; exon_number 2; oId CUFF.1181.1; tss_id TSS3835;
1	Cufflinks	exon	41763869	41764641	.	-	.	gene_id GRMZM2G119886; transcript_id TCONS_00004274; exon_number 1; oId CUFF.1200.1; tss_id TSS3838;
1	Cufflinks	exon	41765089	41765178	.	-	.	gene_id GRMZM2G119886; transcript_id TCONS_00004274; exon_number 2; oId CUFF.1200.1; tss_id TSS3838;
1	Cufflinks	exon	41766083	41767021	.	-	.	gene_id GRMZM2G119886; transcript_id TCONS_00004274; exon_number 3; oId CUFF.1200.1; tss_id TSS3838;
1	Cufflinks	exon	43690068	43690590	.	-	.	gene_id GRMZM2G017792; transcript_id TCONS_00004299; exon_number 1; oId CUFF.1238.1; tss_id TSS3862;
1	Cufflinks	exon	43691281	43691464	.	-	.	gene_id GRMZM2G017792; transcript_id TCONS_00004299; exon_number 2; oId CUFF.1238.1; tss_id TSS3862;
1	Cufflinks	exon	43691547	43692017	.	-	.	gene_id GRMZM2G017792; transcript_id TCONS_00004299; exon_number 3; oId CUFF.1238.1; tss_id TSS3862;
1	Cufflinks	exon	43692110	43692239	.	-	.	gene_id GRMZM2G017792; transcript_id TCONS_00004299; exon_number 4; oId CUFF.1238.1; tss_id TSS3862;
1	Cufflinks	exon	43692587	43692986	.	-	.	gene_id GRMZM2G017792; transcript_id TCONS_00004299; exon_number 5; oId CUFF.1238.1; tss_id TSS3862;
1	Cufflinks	exon	45119006	45119779	.	-	.	gene_id GRMZM2G053669; transcript_id TCONS_00004315; exon_number 1; oId CUFF.1283.1; tss_id TSS3875;
1	Cufflinks	exon	45119892	45119972	.	-	.	gene_id GRMZM2G053669; transcript_id TCONS_00004315; exon_number 2; oId CUFF.1283.1; tss_id TSS3875;
1	Cufflinks	exon	45120091	45120447	.	-	.	gene_id GRMZM2G053669; transcript_id TCONS_00004315; exon_number 3; oId CUFF.1283.1; tss_id TSS3875;
1	Cufflinks	exon	45120546	45120626	.	-	.	gene_id GRMZM2G053669; transcript_id TCONS_00004315; exon_number 4; oId CUFF.1283.1; tss_id TSS3875;
1	Cufflinks	exon	45120746	45120907	.	-	.	gene_id GRMZM2G053669; transcript_id TCONS_00004315; exon_number 5; oId CUFF.1283.1; tss_id TSS3875;
1	Cufflinks	exon	45121042	45121232	.	-	.	gene_id GRMZM2G053669; transcript_id TCONS_00004315; exon_number 6; oId CUFF.1283.1; tss_id TSS3875;
1	Cufflinks	exon	45121435	45121576	.	-	.	gene_id GRMZM2G053669; transcript_id TCONS_00004315; exon_number 7; oId CUFF.1283.1; tss_id TSS3875;
1	Cufflinks	exon	45121755	45121853	.	-	.	gene_id GRMZM2G053669; transcript_id TCONS_00004315; exon_number 8; oId CUFF.1283.1; tss_id TSS3875;
1	Cufflinks	exon	45121966	45122104	.	-	.	gene_id GRMZM2G053669; transcript_id TCONS_00004315; exon_number 9; oId CUFF.1283.1; tss_id TSS3875;
1	Cufflinks	exon	45122574	45122764	.	-	.	gene_id GRMZM2G053669; transcript_id TCONS_00004315; exon_number 10; oId CUFF.1283.1; tss_id TSS3875;
1	Cufflinks	exon	45517326	45517933	.	-	.	gene_id GRMZM2G087186; transcript_id TCONS_00004319; exon_number 1; oId CUFF.1280.1; tss_id TSS3879;
1	Cufflinks	exon	45518034	45518222	.	-	.	gene_id GRMZM2G087186; transcript_id TCONS_00004319; exon_number 2; oId CUFF.1280.1; tss_id TSS3879;
1	Cufflinks	exon	45518326	45518443	.	-	.	gene_id GRMZM2G087186; transcript_id TCONS_00004319; exon_number 3; oId CUFF.1280.1; tss_id TSS3879;
1	Cufflinks	exon	45518534	45519184	.	-	.	gene_id GRMZM2G087186; transcript_id TCONS_00004319; exon_number 4; oId CUFF.1280.1; tss_id TSS3879;
1	Cufflinks	exon	45519576	45519739	.	-	.	gene_id GRMZM2G087186; transcript_id TCONS_00004319; exon_number 5; oId CUFF.1280.1; tss_id TSS3879;
1	Cufflinks	exon	45519817	45520489	.	-	.	gene_id GRMZM2G087186; transcript_id TCONS_00004319; exon_number 6; oId CUFF.1280.1; tss_id TSS3879;
1	Cufflinks	exon	45953216	45953755	.	-	.	gene_id GRMZM2G007206; transcript_id TCONS_00004337; exon_number 1; oId CUFF.1312.1; tss_id TSS3893;
1	Cufflinks	exon	45953837	45953941	.	-	.	gene_id GRMZM2G007206; transcript_id TCONS_00004337; exon_number 2; oId CUFF.1312.1; tss_id TSS3893;
1	Cufflinks	exon	45954025	45954105	.	-	.	gene_id GRMZM2G007206; transcript_id TCONS_00004337; exon_number 3; oId CUFF.1312.1; tss_id TSS3893;
1	Cufflinks	exon	45954180	45954617	.	-	.	gene_id GRMZM2G007206; transcript_id TCONS_00004337; exon_number 4; oId CUFF.1312.1; tss_id TSS3893;
1	Cufflinks	exon	46844940	46846925	.	-	.	gene_id GRMZM2G010545; transcript_id TCONS_00004348; exon_number 1; oId CUFF.1334.2; tss_id TSS3903;
1	Cufflinks	exon	46847882	46847963	.	-	.	gene_id GRMZM2G010545; transcript_id TCONS_00004348; exon_number 2; oId CUFF.1334.2; tss_id TSS3903;
1	Cufflinks	exon	46848186	46848533	.	-	.	gene_id GRMZM2G010545; transcript_id TCONS_00004348; exon_number 3; oId CUFF.1334.2; tss_id TSS3903;
1	Cufflinks	exon	46844940	46846925	.	-	.	gene_id GRMZM2G010545; transcript_id TCONS_00004349; exon_number 1; oId CUFF.1334.1; tss_id TSS3903;
1	Cufflinks	exon	46848186	46848551	.	-	.	gene_id GRMZM2G010545; transcript_id TCONS_00004349; exon_number 2; oId CUFF.1334.1; tss_id TSS3903;

1	Cufflinks	exon	52561965	52564360	.	-	.	gene_id GRMZM2G033570; transcript_id TCONS_00004415; exon_number 1; oId CUFF.1459.1; tss_id TSS3963;
1	Cufflinks	exon	52565699	52565938	.	-	.	gene_id GRMZM2G033570; transcript_id TCONS_00004415; exon_number 2; oId CUFF.1459.1; tss_id TSS3963;
1	Cufflinks	exon	60155654	60156604	.	-	.	gene_id XLOC_003898; transcript_id TCONS_00004518; exon_number 1; oId CUFF.1619.1; tss_id TSS4055;
1	Cufflinks	exon	64021077	64022794	.	-	.	gene_id GRMZM2G026417; transcript_id TCONS_00004575; exon_number 1; oId CUFF.1726.1; tss_id TSS4104;
1	Cufflinks	exon	68121169	68122907	.	-	.	gene_id GRMZM2G382717; transcript_id TCONS_00004607; exon_number 1; oId CUFF.1794.1; tss_id TSS4130;
1	Cufflinks	exon	68568918	68570950	.	-	.	gene_id GRMZM2G077897; transcript_id TCONS_00004612; exon_number 1; oId CUFF.1805.1; tss_id TSS4135;
1	Cufflinks	exon	68571129	68571751	.	-	.	gene_id GRMZM2G077897; transcript_id TCONS_00004612; exon_number 2; oId CUFF.1805.1; tss_id TSS4135;
1	Cufflinks	exon	68572716	68572988	.	-	.	gene_id GRMZM2G077897; transcript_id TCONS_00004612; exon_number 3; oId CUFF.1805.1; tss_id TSS4135;
1	Cufflinks	exon	70775839	70776758	.	-	.	gene_id GRMZM2G142390; transcript_id TCONS_00004627; exon_number 1; oId CUFF.1834.1; tss_id TSS4149;
1	Cufflinks	exon	70777927	70778063	.	-	.	gene_id GRMZM2G142390; transcript_id TCONS_00004627; exon_number 2; oId CUFF.1834.1; tss_id TSS4149;
1	Cufflinks	exon	70779194	70780013	.	-	.	gene_id GRMZM2G142390; transcript_id TCONS_00004627; exon_number 3; oId CUFF.1834.1; tss_id TSS4149;
1	Cufflinks	exon	71221941	71222773	.	-	.	gene_id XLOC_003999; transcript_id TCONS_00004638; exon_number 1; oId CUFF.1859.1; tss_id TSS4160;
1	Cufflinks	exon	71223525	71223924	.	-	.	gene_id XLOC_003999; transcript_id TCONS_00004638; exon_number 2; oId CUFF.1859.1; tss_id TSS4160;
1	Cufflinks	exon	77040876	77041414	.	-	.	gene_id XLOC_004055; transcript_id TCONS_00004702; exon_number 1; oId CUFF.1970.1; tss_id TSS4216;
1	Cufflinks	exon	77152281	77153650	.	-	.	gene_id GRMZM2G044902; transcript_id TCONS_00004703; exon_number 1; oId CUFF.1973.1; tss_id TSS4217;
1	Cufflinks	exon	77153766	77154119	.	-	.	gene_id GRMZM2G044902; transcript_id TCONS_00004703; exon_number 2; oId CUFF.1973.1; tss_id TSS4217;
1	Cufflinks	exon	78064391	78065838	.	-	.	gene_id GRMZM2G024175; transcript_id TCONS_00004715; exon_number 1; oId CUFF.1981.1; tss_id TSS4228;
1	Cufflinks	exon	80175428	80176363	.	-	.	gene_id GRMZM6G399977; transcript_id TCONS_00004741; exon_number 1; oId CUFF.2039.1; tss_id TSS4252;
1	Cufflinks	exon	80176863	80176954	.	-	.	gene_id GRMZM6G399977; transcript_id TCONS_00004741; exon_number 2; oId CUFF.2039.1; tss_id TSS4252;
1	Cufflinks	exon	80177725	80177868	.	-	.	gene_id GRMZM6G399977; transcript_id TCONS_00004741; exon_number 3; oId CUFF.2039.1; tss_id TSS4252;
1	Cufflinks	exon	80178143	80178189	.	-	.	gene_id GRMZM6G399977; transcript_id TCONS_00004741; exon_number 4; oId CUFF.2039.1; tss_id TSS4252;
1	Cufflinks	exon	80191317	80191375	.	-	.	gene_id GRMZM6G399977; transcript_id TCONS_00004741; exon_number 5; oId CUFF.2039.1; tss_id TSS4252;
1	Cufflinks	exon	80191468	80191628	.	-	.	gene_id GRMZM6G399977; transcript_id TCONS_00004741; exon_number 6; oId CUFF.2039.1; tss_id TSS4252;
1	Cufflinks	exon	80191698	80191906	.	-	.	gene_id GRMZM6G399977; transcript_id TCONS_00004741; exon_number 7; oId CUFF.2039.1; tss_id TSS4252;
1	Cufflinks	exon	80192001	80192072	.	-	.	gene_id GRMZM6G399977; transcript_id TCONS_00004741; exon_number 8; oId CUFF.2039.1; tss_id TSS4252;
1	Cufflinks	exon	80192626	80192685	.	-	.	gene_id GRMZM6G399977; transcript_id TCONS_00004741; exon_number 9; oId CUFF.2039.1; tss_id TSS4252;
1	Cufflinks	exon	80192762	80193276	.	-	.	gene_id GRMZM6G399977; transcript_id TCONS_00004741; exon_number 10; oId CUFF.2039.1; tss_id TSS4252;
1	Cufflinks	exon	81612706	81613274	.	-	.	gene_id GRMZM2G028821; transcript_id TCONS_00004759; exon_number 1; oId CUFF.2056.1; tss_id TSS4268;
1	Cufflinks	exon	81613354	81613830	.	-	.	gene_id GRMZM2G028821; transcript_id TCONS_00004759; exon_number 2; oId CUFF.2056.1; tss_id TSS4268;
1	Cufflinks	exon	92473871	92474678	.	-	.	gene_id XLOC_004184; transcript_id TCONS_00004846; exon_number 1; oId CUFF.2219.1; tss_id TSS4348;
1	Cufflinks	exon	94691673	94692217	.	-	.	gene_id GRMZM2G178576; transcript_id TCONS_00004856; exon_number 1; oId CUFF.2247.2; tss_id TSS4357;
1	Cufflinks	exon	94692304	94692490	.	-	.	gene_id GRMZM2G178576; transcript_id TCONS_00004856; exon_number 2; oId CUFF.2247.2; tss_id TSS4357;
1	Cufflinks	exon	94693013	94693914	.	-	.	gene_id GRMZM2G178576; transcript_id TCONS_00004856; exon_number 3; oId CUFF.2247.2; tss_id TSS4357;
1	Cufflinks	exon	94691673	94692217	.	-	.	gene_id GRMZM2G178576; transcript_id TCONS_00004855; exon_number 1; oId CUFF.2247.1; tss_id TSS4357;
1	Cufflinks	exon	94692304	94693914	.	-	.	gene_id GRMZM2G178576; transcript_id TCONS_00004855; exon_number 2; oId CUFF.2247.1; tss_id TSS4357;
1	Cufflinks	exon	95197725	95198942	.	-	.	gene_id GRMZM2G007939; transcript_id TCONS_00004860; exon_number 1; oId CUFF.2268.2; tss_id TSS4361;

1	Cufflinks	exon	95199052	95199162	.	-	.	gene_id GRMZM2G007939; transcript_id TCONS_00004860; exon_number 2; oId CUFF.2268.2; tss_id TSS4361;
1	Cufflinks	exon	95199286	95199496	.	-	.	gene_id GRMZM2G007939; transcript_id TCONS_00004860; exon_number 3; oId CUFF.2268.2; tss_id TSS4361;
1	Cufflinks	exon	95200813	95201523	.	-	.	gene_id GRMZM2G007939; transcript_id TCONS_00004860; exon_number 4; oId CUFF.2268.2; tss_id TSS4361;
1	Cufflinks	exon	95197725	95198942	.	-	.	gene_id GRMZM2G007939; transcript_id TCONS_00004861; exon_number 1; oId CUFF.2268.1; tss_id TSS4361;
1	Cufflinks	exon	95199052	95199162	.	-	.	gene_id GRMZM2G007939; transcript_id TCONS_00004861; exon_number 2; oId CUFF.2268.1; tss_id TSS4361;
1	Cufflinks	exon	95199286	95201525	.	-	.	gene_id GRMZM2G007939; transcript_id TCONS_00004861; exon_number 3; oId CUFF.2268.1; tss_id TSS4361;
1	Cufflinks	exon	99546686	99548327	.	-	.	gene_id AC194914.3_FG002; transcript_id TCONS_00004877; exon_number 1; oId CUFF.2299.1; tss_id TSS4377;
1	Cufflinks	exon	137254058	137254403	.	-	.	gene_id XLOC_004364; transcript_id TCONS_00005041; exon_number 1; oId CUFF.2578.1; tss_id TSS4534;
1	Cufflinks	exon	137256279	137256652	.	-	.	gene_id XLOC_004364; transcript_id TCONS_00005041; exon_number 2; oId CUFF.2578.1; tss_id TSS4534;
1	Cufflinks	exon	161322162	161322881	.	-	.	gene_id GRMZM5G878732; transcript_id TCONS_00005174; exon_number 1; oId CUFF.2801.1; tss_id TSS4654;
1	Cufflinks	exon	161322992	161323218	.	-	.	gene_id GRMZM5G878732; transcript_id TCONS_00005174; exon_number 2; oId CUFF.2801.1; tss_id TSS4654;
1	Cufflinks	exon	161323929	161324184	.	-	.	gene_id GRMZM5G878732; transcript_id TCONS_00005174; exon_number 3; oId CUFF.2801.1; tss_id TSS4654;
1	Cufflinks	exon	162442194	162444501	.	-	.	gene_id GRMZM2G546081; transcript_id TCONS_00005182; exon_number 1; oId CUFF.2836.1; tss_id TSS4662;
1	Cufflinks	exon	162444701	162444810	.	-	.	gene_id GRMZM2G546081; transcript_id TCONS_00005182; exon_number 2; oId CUFF.2836.1; tss_id TSS4662;
1	Cufflinks	exon	162444897	162445090	.	-	.	gene_id GRMZM2G546081; transcript_id TCONS_00005182; exon_number 3; oId CUFF.2836.1; tss_id TSS4662;
1	Cufflinks	exon	162445984	162446052	.	-	.	gene_id GRMZM2G546081; transcript_id TCONS_00005182; exon_number 4; oId CUFF.2836.1; tss_id TSS4662;
1	Cufflinks	exon	162446251	162446437	.	-	.	gene_id GRMZM2G546081; transcript_id TCONS_00005182; exon_number 5; oId CUFF.2836.1; tss_id TSS4662;
1	Cufflinks	exon	162446857	162447143	.	-	.	gene_id GRMZM2G546081; transcript_id TCONS_00005182; exon_number 6; oId CUFF.2836.1; tss_id TSS4662;
1	Cufflinks	exon	162448651	162449008	.	-	.	gene_id GRMZM2G546081; transcript_id TCONS_00005182; exon_number 7; oId CUFF.2836.1; tss_id TSS4662;
1	Cufflinks	exon	165422123	165423173	.	-	.	gene_id GRMZM2G127739; transcript_id TCONS_00005198; exon_number 1; oId CUFF.2871.1; tss_id TSS4678;
1	Cufflinks	exon	165424054	165424220	.	-	.	gene_id GRMZM2G127739; transcript_id TCONS_00005198; exon_number 2; oId CUFF.2871.1; tss_id TSS4678;
1	Cufflinks	exon	165424335	165424579	.	-	.	gene_id GRMZM2G127739; transcript_id TCONS_00005198; exon_number 3; oId CUFF.2871.1; tss_id TSS4678;
1	Cufflinks	exon	165424777	165424957	.	-	.	gene_id GRMZM2G127739; transcript_id TCONS_00005198; exon_number 4; oId CUFF.2871.1; tss_id TSS4678;
1	Cufflinks	exon	165425148	165425324	.	-	.	gene_id GRMZM2G127739; transcript_id TCONS_00005198; exon_number 5; oId CUFF.2871.1; tss_id TSS4678;
1	Cufflinks	exon	165425415	165425519	.	-	.	gene_id GRMZM2G127739; transcript_id TCONS_00005198; exon_number 6; oId CUFF.2871.1; tss_id TSS4678;
1	Cufflinks	exon	165425652	165425789	.	-	.	gene_id GRMZM2G127739; transcript_id TCONS_00005198; exon_number 7; oId CUFF.2871.1; tss_id TSS4678;
1	Cufflinks	exon	165426510	165427760	.	-	.	gene_id GRMZM2G127739; transcript_id TCONS_00005198; exon_number 8; oId CUFF.2871.1; tss_id TSS4678;
1	Cufflinks	exon	170979650	170980054	.	-	.	gene_id GRMZM2G115615; transcript_id TCONS_00005234; exon_number 1; oId CUFF.2951.1; tss_id TSS4713;
1	Cufflinks	exon	170980416	170980549	.	-	.	gene_id GRMZM2G115615; transcript_id TCONS_00005234; exon_number 2; oId CUFF.2951.1; tss_id TSS4713;
1	Cufflinks	exon	170980727	170980846	.	-	.	gene_id GRMZM2G115615; transcript_id TCONS_00005234; exon_number 3; oId CUFF.2951.1; tss_id TSS4713;
1	Cufflinks	exon	170981073	170981252	.	-	.	gene_id GRMZM2G115615; transcript_id TCONS_00005234; exon_number 4; oId CUFF.2951.1; tss_id TSS4713;
1	Cufflinks	exon	170981617	170981790	.	-	.	gene_id GRMZM2G115615; transcript_id TCONS_00005234; exon_number 5; oId CUFF.2951.1; tss_id TSS4713;
1	Cufflinks	exon	170982613	170982663	.	-	.	gene_id GRMZM2G115615; transcript_id TCONS_00005234; exon_number 6; oId CUFF.2951.1; tss_id TSS4713;
1	Cufflinks	exon	170982753	170982846	.	-	.	gene_id GRMZM2G115615; transcript_id TCONS_00005234; exon_number 7; oId CUFF.2951.1; tss_id TSS4713;
1	Cufflinks	exon	170986470	170986759	.	-	.	gene_id GRMZM2G115615; transcript_id TCONS_00005234; exon_number 8; oId CUFF.2951.1; tss_id TSS4713;
1	Cufflinks	exon	180018445	180018827	.	-	.	gene_id GRMZM2G093755; transcript_id TCONS_00005333; exon_number 1; oId CUFF.3093.2; tss_id TSS4801;

1	Cufflinks	exon	180018916	180019154	.	-	.	gene_id GRMZM2G093755; transcript_id TCONS_00005333; exon_number 2; oId CUFF.3093.2; tss_id TSS4801;
1	Cufflinks	exon	180020903	180021079	.	-	.	gene_id GRMZM2G093755; transcript_id TCONS_00005333; exon_number 3; oId CUFF.3093.2; tss_id TSS4801;
1	Cufflinks	exon	180021201	180021636	.	-	.	gene_id GRMZM2G093755; transcript_id TCONS_00005333; exon_number 4; oId CUFF.3093.2; tss_id TSS4801;
1	Cufflinks	exon	180635433	180637762	.	-	.	gene_id GRMZM5G893912; transcript_id TCONS_00005344; exon_number 1; oId CUFF.3101.1; tss_id TSS4811;
1	Cufflinks	exon	183333777	183334701	.	-	.	gene_id XLOC_004665; transcript_id TCONS_00005375; exon_number 1; oId CUFF.3194.1; tss_id TSS4840;
1	Cufflinks	exon	191613121	191613787	.	-	.	gene_id GRMZM2G337191; transcript_id TCONS_00005464; exon_number 1; oId CUFF.3348.1; tss_id TSS4921;
1	Cufflinks	exon	191614200	191614821	.	-	.	gene_id GRMZM2G337191; transcript_id TCONS_00005464; exon_number 2; oId CUFF.3348.1; tss_id TSS4921;
1	Cufflinks	exon	192065391	192066923	.	-	.	gene_id XLOC_004751; transcript_id TCONS_00005471; exon_number 1; oId CUFF.3373.1; tss_id TSS4928;
1	Cufflinks	exon	192067033	192067277	.	-	.	gene_id XLOC_004751; transcript_id TCONS_00005471; exon_number 2; oId CUFF.3373.1; tss_id TSS4928;
1	Cufflinks	exon	192067410	192067850	.	-	.	gene_id XLOC_004751; transcript_id TCONS_00005471; exon_number 3; oId CUFF.3373.1; tss_id TSS4928;
1	Cufflinks	exon	196458606	196459740	.	-	.	gene_id GRMZM2G069476; transcript_id TCONS_00005518; exon_number 1; oId CUFF.3462.1; tss_id TSS4973;
1	Cufflinks	exon	196460767	196460858	.	-	.	gene_id GRMZM2G069476; transcript_id TCONS_00005518; exon_number 2; oId CUFF.3462.1; tss_id TSS4973;
1	Cufflinks	exon	196461051	196461326	.	-	.	gene_id GRMZM2G069476; transcript_id TCONS_00005518; exon_number 3; oId CUFF.3462.1; tss_id TSS4973;
1	Cufflinks	exon	196461577	196461610	.	-	.	gene_id GRMZM2G069476; transcript_id TCONS_00005518; exon_number 4; oId CUFF.3462.1; tss_id TSS4973;
1	Cufflinks	exon	196461632	196462569	.	-	.	gene_id GRMZM2G069476; transcript_id TCONS_00005518; exon_number 5; oId CUFF.3462.1; tss_id TSS4973;
1	Cufflinks	exon	196463105	196463550	.	-	.	gene_id GRMZM2G069476; transcript_id TCONS_00005518; exon_number 6; oId CUFF.3462.1; tss_id TSS4973;
1	Cufflinks	exon	201502572	201502846	.	-	.	gene_id XLOC_004849; transcript_id TCONS_00005578; exon_number 1; oId CUFF.3575.1; tss_id TSS5030;
1	Cufflinks	exon	205287282	205287620	.	-	.	gene_id GRMZM2G319357; transcript_id TCONS_00005635; exon_number 1; oId CUFF.3695.1; tss_id TSS5082;
1	Cufflinks	exon	205288374	205288460	.	-	.	gene_id GRMZM2G319357; transcript_id TCONS_00005635; exon_number 2; oId CUFF.3695.1; tss_id TSS5082;
1	Cufflinks	exon	205289154	205289221	.	-	.	gene_id GRMZM2G319357; transcript_id TCONS_00005635; exon_number 3; oId CUFF.3695.1; tss_id TSS5082;
1	Cufflinks	exon	205289324	205289459	.	-	.	gene_id GRMZM2G319357; transcript_id TCONS_00005635; exon_number 4; oId CUFF.3695.1; tss_id TSS5082;
1	Cufflinks	exon	205289549	205289661	.	-	.	gene_id GRMZM2G319357; transcript_id TCONS_00005635; exon_number 5; oId CUFF.3695.1; tss_id TSS5082;
1	Cufflinks	exon	205291582	205292061	.	-	.	gene_id GRMZM2G319357; transcript_id TCONS_00005635; exon_number 6; oId CUFF.3695.1; tss_id TSS5082;
1	Cufflinks	exon	208873296	208874008	.	-	.	gene_id XLOC_004952; transcript_id TCONS_00005691; exon_number 1; oId CUFF.3771.1; tss_id TSS5135;
1	Cufflinks	exon	210641599	210642195	.	-	.	gene_id GRMZM2G428554; transcript_id TCONS_00005713; exon_number 1; oId CUFF.3822.2; tss_id TSS5155;
1	Cufflinks	exon	210642636	210646145	.	-	.	gene_id GRMZM2G428554; transcript_id TCONS_00005713; exon_number 2; oId CUFF.3822.2; tss_id TSS5155;
1	Cufflinks	exon	212089021	212089389	.	-	.	gene_id XLOC_004983; transcript_id TCONS_00005728; exon_number 1; oId CUFF.3835.1; tss_id TSS5169;
1	Cufflinks	exon	216747715	216748361	.	-	.	gene_id GRMZM2G167694; transcript_id TCONS_00005781; exon_number 1; oId CUFF.3920.1; tss_id TSS5211;
1	Cufflinks	exon	216748437	216748720	.	-	.	gene_id GRMZM2G167694; transcript_id TCONS_00005781; exon_number 2; oId CUFF.3920.1; tss_id TSS5211;
1	Cufflinks	exon	216748944	216749211	.	-	.	gene_id GRMZM2G167694; transcript_id TCONS_00005781; exon_number 3; oId CUFF.3920.1; tss_id TSS5211;
1	Cufflinks	exon	216749398	216749435	.	-	.	gene_id GRMZM2G167694; transcript_id TCONS_00005781; exon_number 4; oId CUFF.3920.1; tss_id TSS5211;
1	Cufflinks	exon	216749892	216750376	.	-	.	gene_id GRMZM2G167694; transcript_id TCONS_00005781; exon_number 5; oId CUFF.3920.1; tss_id TSS5211;
1	Cufflinks	exon	218469216	218469942	.	-	.	gene_id GRMZM2G114093; transcript_id TCONS_00005795; exon_number 1; oId CUFF.3972.2; tss_id TSS5224;
1	Cufflinks	exon	218470177	218470632	.	-	.	gene_id GRMZM2G114093; transcript_id TCONS_00005795; exon_number 2; oId CUFF.3972.2; tss_id TSS5224;
1	Cufflinks	exon	218471436	218472813	.	-	.	gene_id GRMZM2G114093; transcript_id TCONS_00005795; exon_number 3; oId CUFF.3972.2; tss_id TSS5224;
1	Cufflinks	exon	218473012	218473337	.	-	.	gene_id GRMZM2G114093; transcript_id TCONS_00005795; exon_number 4; oId CUFF.3972.2; tss_id TSS5224;

1	Cufflinks	exon	218852692	218853654	.	-	.	gene_id GRMZM2G003426; transcript_id TCONS_00005804; exon_number 1; oId CUFF.3954.1; tss_id TSS5233;
1	Cufflinks	exon	223849323	223851220	.	-	.	gene_id GRMZM2G181236; transcript_id TCONS_00005874; exon_number 1; oId CUFF.4082.1; tss_id TSS5292;
1	Cufflinks	exon	250153484	250154150	.	-	.	gene_id GRMZM2G364069; transcript_id TCONS_00006248; exon_number 1; oId CUFF.4683.1; tss_id TSS5620;
1	Cufflinks	exon	250154233	250154431	.	-	.	gene_id GRMZM2G364069; transcript_id TCONS_00006248; exon_number 2; oId CUFF.4683.1; tss_id TSS5620;
1	Cufflinks	exon	250154521	250154797	.	-	.	gene_id GRMZM2G364069; transcript_id TCONS_00006248; exon_number 3; oId CUFF.4683.1; tss_id TSS5620;
1	Cufflinks	exon	250154883	250155027	.	-	.	gene_id GRMZM2G364069; transcript_id TCONS_00006248; exon_number 4; oId CUFF.4683.1; tss_id TSS5620;
1	Cufflinks	exon	250155119	250155281	.	-	.	gene_id GRMZM2G364069; transcript_id TCONS_00006248; exon_number 5; oId CUFF.4683.1; tss_id TSS5620;
1	Cufflinks	exon	250156439	250156801	.	-	.	gene_id GRMZM2G364069; transcript_id TCONS_00006248; exon_number 6; oId CUFF.4683.1; tss_id TSS5620;
1	Cufflinks	exon	253373027	253374759	.	-	.	gene_id GRMZM2G066067; transcript_id TCONS_00006276; exon_number 1; oId CUFF.4747.2; tss_id TSS5646;
1	Cufflinks	exon	253375066	253375697	.	-	.	gene_id GRMZM2G066067; transcript_id TCONS_00006276; exon_number 2; oId CUFF.4747.2; tss_id TSS5646;
1	Cufflinks	exon	258031736	258032238	.	-	.	gene_id XLOC_005500; transcript_id TCONS_00006347; exon_number 1; oId CUFF.4853.1; tss_id TSS5708;
1	Cufflinks	exon	261178421	261178520	.	-	.	gene_id GRMZM2G068575; transcript_id TCONS_00006393; exon_number 1; oId CUFF.4951.1; tss_id TSS5751;
1	Cufflinks	exon	261192100	261192543	.	-	.	gene_id GRMZM2G068575; transcript_id TCONS_00006393; exon_number 2; oId CUFF.4951.1; tss_id TSS5751;
1	Cufflinks	exon	261723724	261724186	.	-	.	gene_id GRMZM2G133684; transcript_id TCONS_00006401; exon_number 1; oId CUFF.4950.1; tss_id TSS5759;
1	Cufflinks	exon	261724285	261724391	.	-	.	gene_id GRMZM2G133684; transcript_id TCONS_00006401; exon_number 2; oId CUFF.4950.1; tss_id TSS5759;
1	Cufflinks	exon	261724510	261724657	.	-	.	gene_id GRMZM2G133684; transcript_id TCONS_00006401; exon_number 3; oId CUFF.4950.1; tss_id TSS5759;
1	Cufflinks	exon	261725251	261725384	.	-	.	gene_id GRMZM2G133684; transcript_id TCONS_00006401; exon_number 4; oId CUFF.4950.1; tss_id TSS5759;
1	Cufflinks	exon	261725776	261726331	.	-	.	gene_id GRMZM2G133684; transcript_id TCONS_00006401; exon_number 5; oId CUFF.4950.1; tss_id TSS5759;
1	Cufflinks	exon	265971512	265972239	.	-	.	gene_id GRMZM2G704032; transcript_id TCONS_00006466; exon_number 1; oId CUFF.5072.1; tss_id TSS5816;
1	Cufflinks	exon	265972347	265972426	.	-	.	gene_id GRMZM2G704032; transcript_id TCONS_00006466; exon_number 2; oId CUFF.5072.1; tss_id TSS5816;
1	Cufflinks	exon	265972742	265972822	.	-	.	gene_id GRMZM2G704032; transcript_id TCONS_00006466; exon_number 3; oId CUFF.5072.1; tss_id TSS5816;
1	Cufflinks	exon	265972924	265972993	.	-	.	gene_id GRMZM2G704032; transcript_id TCONS_00006466; exon_number 4; oId CUFF.5072.1; tss_id TSS5816;
1	Cufflinks	exon	265973099	265973173	.	-	.	gene_id GRMZM2G704032; transcript_id TCONS_00006466; exon_number 5; oId CUFF.5072.1; tss_id TSS5816;
1	Cufflinks	exon	265973284	265973489	.	-	.	gene_id GRMZM2G704032; transcript_id TCONS_00006466; exon_number 6; oId CUFF.5072.1; tss_id TSS5816;
1	Cufflinks	exon	265973842	265974091	.	-	.	gene_id GRMZM2G704032; transcript_id TCONS_00006466; exon_number 7; oId CUFF.5072.1; tss_id TSS5816;
1	Cufflinks	exon	265974167	265974212	.	-	.	gene_id GRMZM2G704032; transcript_id TCONS_00006466; exon_number 8; oId CUFF.5072.1; tss_id TSS5816;
1	Cufflinks	exon	265974523	265974592	.	-	.	gene_id GRMZM2G704032; transcript_id TCONS_00006466; exon_number 9; oId CUFF.5072.1; tss_id TSS5816;
1	Cufflinks	exon	265977250	265977319	.	-	.	gene_id GRMZM2G704032; transcript_id TCONS_00006466; exon_number 10; oId CUFF.5072.1; tss_id TSS5816;
1	Cufflinks	exon	265977887	265977971	.	-	.	gene_id GRMZM2G704032; transcript_id TCONS_00006466; exon_number 11; oId CUFF.5072.1; tss_id TSS5816;
1	Cufflinks	exon	265978122	265978206	.	-	.	gene_id GRMZM2G704032; transcript_id TCONS_00006466; exon_number 12; oId CUFF.5072.1; tss_id TSS5816;
1	Cufflinks	exon	265978313	265978403	.	-	.	gene_id GRMZM2G704032; transcript_id TCONS_00006466; exon_number 13; oId CUFF.5072.1; tss_id TSS5816;
1	Cufflinks	exon	265979010	265979530	.	-	.	gene_id GRMZM2G704032; transcript_id TCONS_00006466; exon_number 14; oId CUFF.5072.1; tss_id TSS5816;
1	Cufflinks	exon	267614418	267614784	.	-	.	gene_id GRMZM5G800853; transcript_id TCONS_00006487; exon_number 1; oId CUFF.5114.1; tss_id TSS5836;
1	Cufflinks	exon	267614871	267614927	.	-	.	gene_id GRMZM5G800853; transcript_id TCONS_00006487; exon_number 2; oId CUFF.5114.1; tss_id TSS5836;
1	Cufflinks	exon	267615014	267615070	.	-	.	gene_id GRMZM5G800853; transcript_id TCONS_00006487; exon_number 3; oId CUFF.5114.1; tss_id TSS5836;
1	Cufflinks	exon	267624435	267624475	.	-	.	gene_id GRMZM5G800853; transcript_id TCONS_00006487; exon_number 4; oId CUFF.5114.1; tss_id TSS5836;

1	Cufflinks	exon	267624581	267624839	.	-	.	gene_id GRMZM5G800853; transcript_id TCONS_00006487; exon_number 5; oId CUFF.5114.1; tss_id TSS5836;
1	Cufflinks	exon	275052364	275052738	.	-	.	gene_id GRMZM2G159295; transcript_id TCONS_00006598; exon_number 1; oId CUFF.5316.1; tss_id TSS5940;
1	Cufflinks	exon	275052810	275052929	.	-	.	gene_id GRMZM2G159295; transcript_id TCONS_00006598; exon_number 2; oId CUFF.5316.1; tss_id TSS5940;
1	Cufflinks	exon	275053146	275053175	.	-	.	gene_id GRMZM2G159295; transcript_id TCONS_00006598; exon_number 3; oId CUFF.5316.1; tss_id TSS5940;
1	Cufflinks	exon	275053268	275053312	.	-	.	gene_id GRMZM2G159295; transcript_id TCONS_00006598; exon_number 4; oId CUFF.5316.1; tss_id TSS5940;
1	Cufflinks	exon	275053479	275053523	.	-	.	gene_id GRMZM2G159295; transcript_id TCONS_00006598; exon_number 5; oId CUFF.5316.1; tss_id TSS5940;
1	Cufflinks	exon	275053611	275053838	.	-	.	gene_id GRMZM2G159295; transcript_id TCONS_00006598; exon_number 6; oId CUFF.5316.1; tss_id TSS5940;
1	Cufflinks	exon	275055409	275055483	.	-	.	gene_id GRMZM2G159295; transcript_id TCONS_00006598; exon_number 7; oId CUFF.5316.1; tss_id TSS5940;
1	Cufflinks	exon	275056417	275056538	.	-	.	gene_id GRMZM2G159295; transcript_id TCONS_00006598; exon_number 8; oId CUFF.5316.1; tss_id TSS5940;
1	Cufflinks	exon	275057406	275057550	.	-	.	gene_id GRMZM2G159295; transcript_id TCONS_00006598; exon_number 9; oId CUFF.5316.1; tss_id TSS5940;
1	Cufflinks	exon	275057915	275057944	.	-	.	gene_id GRMZM2G159295; transcript_id TCONS_00006598; exon_number 10; oId CUFF.5316.1; tss_id TSS5940;
1	Cufflinks	exon	275058388	275059001	.	-	.	gene_id GRMZM2G159295; transcript_id TCONS_00006598; exon_number 11; oId CUFF.5316.1; tss_id TSS5940;
1	Cufflinks	exon	275098368	275098755	.	-	.	gene_id GRMZM2G159285; transcript_id TCONS_00006599; exon_number 1; oId CUFF.5307.1; tss_id TSS5941;
1	Cufflinks	exon	275098855	275098916	.	-	.	gene_id GRMZM2G159285; transcript_id TCONS_00006599; exon_number 2; oId CUFF.5307.1; tss_id TSS5941;
1	Cufflinks	exon	275099018	275099129	.	-	.	gene_id GRMZM2G159285; transcript_id TCONS_00006599; exon_number 3; oId CUFF.5307.1; tss_id TSS5941;
1	Cufflinks	exon	275099269	275099525	.	-	.	gene_id GRMZM2G159285; transcript_id TCONS_00006599; exon_number 4; oId CUFF.5307.1; tss_id TSS5941;
1	Cufflinks	exon	275100452	275101121	.	-	.	gene_id GRMZM2G159285; transcript_id TCONS_00006599; exon_number 5; oId CUFF.5307.1; tss_id TSS5941;
1	Cufflinks	exon	275694757	275695982	.	-	.	gene_id GRMZM2G128564; transcript_id TCONS_00006611; exon_number 1; oId CUFF.5322.1; tss_id TSS5952;
1	Cufflinks	exon	275696313	275696881	.	-	.	gene_id GRMZM2G128564; transcript_id TCONS_00006611; exon_number 2; oId CUFF.5322.1; tss_id TSS5952;
1	Cufflinks	exon	275759689	275760793	.	-	.	gene_id GRMZM2G377131; transcript_id TCONS_00006614; exon_number 1; oId CUFF.5333.1; tss_id TSS5955;
1	Cufflinks	exon	275760913	275761549	.	-	.	gene_id GRMZM2G377131; transcript_id TCONS_00006614; exon_number 2; oId CUFF.5333.1; tss_id TSS5955;
1	Cufflinks	exon	276882941	276884024	.	-	.	gene_id GRMZM2G421491; transcript_id TCONS_00006646; exon_number 1; oId CUFF.5409.1; tss_id TSS5986;
1	Cufflinks	exon	276884126	276884308	.	-	.	gene_id GRMZM2G421491; transcript_id TCONS_00006646; exon_number 2; oId CUFF.5409.1; tss_id TSS5986;
1	Cufflinks	exon	276884396	276884651	.	-	.	gene_id GRMZM2G421491; transcript_id TCONS_00006646; exon_number 3; oId CUFF.5409.1; tss_id TSS5986;
1	Cufflinks	exon	276884753	276885331	.	-	.	gene_id GRMZM2G421491; transcript_id TCONS_00006646; exon_number 4; oId CUFF.5409.1; tss_id TSS5986;
1	Cufflinks	exon	276885467	276885564	.	-	.	gene_id GRMZM2G421491; transcript_id TCONS_00006646; exon_number 5; oId CUFF.5409.1; tss_id TSS5986;
1	Cufflinks	exon	276886549	276887539	.	-	.	gene_id GRMZM2G421491; transcript_id TCONS_00006646; exon_number 6; oId CUFF.5409.1; tss_id TSS5986;
1	Cufflinks	exon	279541752	279542665	.	-	.	gene_id GRMZM2G047759; transcript_id TCONS_00006685; exon_number 1; oId CUFF.5458.1; tss_id TSS6020;
1	Cufflinks	exon	279542769	279542891	.	-	.	gene_id GRMZM2G047759; transcript_id TCONS_00006685; exon_number 2; oId CUFF.5458.1; tss_id TSS6020;
1	Cufflinks	exon	279543190	279543297	.	-	.	gene_id GRMZM2G047759; transcript_id TCONS_00006685; exon_number 3; oId CUFF.5458.1; tss_id TSS6020;
1	Cufflinks	exon	281711998	281713296	.	-	.	gene_id GRMZM2G028286; transcript_id TCONS_00006723; exon_number 1; oId CUFF.5526.1; tss_id TSS6055;
1	Cufflinks	exon	281713944	281714057	.	-	.	gene_id GRMZM2G028286; transcript_id TCONS_00006723; exon_number 2; oId CUFF.5526.1; tss_id TSS6055;
1	Cufflinks	exon	281714143	281714433	.	-	.	gene_id GRMZM2G028286; transcript_id TCONS_00006723; exon_number 3; oId CUFF.5526.1; tss_id TSS6055;
1	Cufflinks	exon	281714519	281714827	.	-	.	gene_id GRMZM2G028286; transcript_id TCONS_00006723; exon_number 4; oId CUFF.5526.1; tss_id TSS6055;
1	Cufflinks	exon	281714919	281716153	.	-	.	gene_id GRMZM2G028286; transcript_id TCONS_00006723; exon_number 5; oId CUFF.5526.1; tss_id TSS6055;
1	Cufflinks	exon	281976636	281978946	.	-	.	gene_id GRMZM2G427697; transcript_id TCONS_00006729; exon_number 1; oId CUFF.5540.1; tss_id TSS6061;

1	Cufflinks	exon	286049844	286050305	.	-	.	gene_id GRMZM2G329181; transcript_id TCONS_00006800; exon_number 1; oId CUFF.5648.1; tss_id TSS6126;
1	Cufflinks	exon	286051365	286053001	.	-	.	gene_id GRMZM2G329181; transcript_id TCONS_00006800; exon_number 2; oId CUFF.5648.1; tss_id TSS6126;
1	Cufflinks	exon	286429050	286429439	.	-	.	gene_id GRMZM2G019799; transcript_id TCONS_00006806; exon_number 1; oId CUFF.5676.1; tss_id TSS6132;
1	Cufflinks	exon	286429801	286429990	.	-	.	gene_id GRMZM2G019799; transcript_id TCONS_00006806; exon_number 2; oId CUFF.5676.1; tss_id TSS6132;
1	Cufflinks	exon	286430075	286430173	.	-	.	gene_id GRMZM2G019799; transcript_id TCONS_00006806; exon_number 3; oId CUFF.5676.1; tss_id TSS6132;
1	Cufflinks	exon	286430292	286430381	.	-	.	gene_id GRMZM2G019799; transcript_id TCONS_00006806; exon_number 4; oId CUFF.5676.1; tss_id TSS6132;
1	Cufflinks	exon	286430589	286431554	.	-	.	gene_id GRMZM2G019799; transcript_id TCONS_00006806; exon_number 5; oId CUFF.5676.1; tss_id TSS6132;
1	Cufflinks	exon	286431657	286431875	.	-	.	gene_id GRMZM2G019799; transcript_id TCONS_00006806; exon_number 6; oId CUFF.5676.1; tss_id TSS6132;
1	Cufflinks	exon	286431993	286432199	.	-	.	gene_id GRMZM2G019799; transcript_id TCONS_00006806; exon_number 7; oId CUFF.5676.1; tss_id TSS6132;
1	Cufflinks	exon	286432319	286432581	.	-	.	gene_id GRMZM2G019799; transcript_id TCONS_00006806; exon_number 8; oId CUFF.5676.1; tss_id TSS6132;
1	Cufflinks	exon	286432936	286434594	.	-	.	gene_id GRMZM2G019799; transcript_id TCONS_00006806; exon_number 9; oId CUFF.5676.1; tss_id TSS6132;
1	Cufflinks	exon	286436990	286437267	.	-	.	gene_id GRMZM2G019799; transcript_id TCONS_00006806; exon_number 10; oId CUFF.5676.1; tss_id TSS6132;
1	Cufflinks	exon	287216930	287217491	.	-	.	gene_id GRMZM2G005991; transcript_id TCONS_00006819; exon_number 1; oId CUFF.5682.1; tss_id TSS6145;
1	Cufflinks	exon	287217582	287217617	.	-	.	gene_id GRMZM2G005991; transcript_id TCONS_00006819; exon_number 2; oId CUFF.5682.1; tss_id TSS6145;
1	Cufflinks	exon	287217737	287217763	.	-	.	gene_id GRMZM2G005991; transcript_id TCONS_00006819; exon_number 3; oId CUFF.5682.1; tss_id TSS6145;
1	Cufflinks	exon	287217850	287218310	.	-	.	gene_id GRMZM2G005991; transcript_id TCONS_00006819; exon_number 4; oId CUFF.5682.1; tss_id TSS6145;
1	Cufflinks	exon	287361393	287361864	.	-	.	gene_id GRMZM2G178415; transcript_id TCONS_00006825; exon_number 1; oId CUFF.5717.2; tss_id TSS6150;
1	Cufflinks	exon	287361967	287362053	.	-	.	gene_id GRMZM2G178415; transcript_id TCONS_00006825; exon_number 2; oId CUFF.5717.2; tss_id TSS6150;
1	Cufflinks	exon	287362132	287362207	.	-	.	gene_id GRMZM2G178415; transcript_id TCONS_00006825; exon_number 3; oId CUFF.5717.2; tss_id TSS6150;
1	Cufflinks	exon	287362346	287362596	.	-	.	gene_id GRMZM2G178415; transcript_id TCONS_00006825; exon_number 4; oId CUFF.5717.2; tss_id TSS6150;
1	Cufflinks	exon	287362826	287362942	.	-	.	gene_id GRMZM2G178415; transcript_id TCONS_00006825; exon_number 5; oId CUFF.5717.2; tss_id TSS6150;
1	Cufflinks	exon	287363208	287363282	.	-	.	gene_id GRMZM2G178415; transcript_id TCONS_00006825; exon_number 6; oId CUFF.5717.2; tss_id TSS6150;
1	Cufflinks	exon	287363391	287363612	.	-	.	gene_id GRMZM2G178415; transcript_id TCONS_00006825; exon_number 7; oId CUFF.5717.2; tss_id TSS6150;
1	Cufflinks	exon	287364295	287364408	.	-	.	gene_id GRMZM2G178415; transcript_id TCONS_00006825; exon_number 8; oId CUFF.5717.2; tss_id TSS6150;
1	Cufflinks	exon	287364651	287364803	.	-	.	gene_id GRMZM2G178415; transcript_id TCONS_00006825; exon_number 9; oId CUFF.5717.2; tss_id TSS6150;
1	Cufflinks	exon	287367443	287367874	.	-	.	gene_id GRMZM2G178415; transcript_id TCONS_00006825; exon_number 10; oId CUFF.5717.2; tss_id TSS6150;
1	Cufflinks	exon	287541417	287542365	.	-	.	gene_id XLOC_005938; transcript_id TCONS_00006828; exon_number 1; oId CUFF.5694.1; tss_id TSS6153;
1	Cufflinks	exon	287652900	287655520	.	-	.	gene_id GRMZM2G070322; transcript_id TCONS_00006830; exon_number 1; oId CUFF.5698.1; tss_id TSS6155;
1	Cufflinks	exon	289402962	289405468	.	-	.	gene_id XLOC_005965; transcript_id TCONS_00006857; exon_number 1; oId CUFF.5759.1; tss_id TSS6180;
1	Cufflinks	exon	292412495	292412997	.	-	.	gene_id GRMZM2G151997; transcript_id TCONS_00006926; exon_number 1; oId CUFF.5867.1; tss_id TSS6243;
1	Cufflinks	exon	292413083	292413316	.	-	.	gene_id GRMZM2G151997; transcript_id TCONS_00006926; exon_number 2; oId CUFF.5867.1; tss_id TSS6243;
1	Cufflinks	exon	292413432	292413903	.	-	.	gene_id GRMZM2G151997; transcript_id TCONS_00006926; exon_number 3; oId CUFF.5867.1; tss_id TSS6243;
1	Cufflinks	exon	293721769	293722203	.	-	.	gene_id GRMZM2G068244; transcript_id TCONS_00006957; exon_number 1; oId CUFF.5921.1; tss_id TSS6274;
1	Cufflinks	exon	293722351	293722474	.	-	.	gene_id GRMZM2G068244; transcript_id TCONS_00006957; exon_number 2; oId CUFF.5921.1; tss_id TSS6274;
1	Cufflinks	exon	293722794	293722855	.	-	.	gene_id GRMZM2G068244; transcript_id TCONS_00006957; exon_number 3; oId CUFF.5921.1; tss_id TSS6274;
1	Cufflinks	exon	293722943	293723040	.	-	.	gene_id GRMZM2G068244; transcript_id TCONS_00006957; exon_number 4; oId CUFF.5921.1; tss_id TSS6274;

1	Cufflinks	exon	293723147	293723183	.	-	.	gene_id GRMZM2G068244; transcript_id TCONS_00006957; exon_number 5; oId CUFF.5921.1; tss_id TSS6274;
1	Cufflinks	exon	293723279	293723550	.	-	.	gene_id GRMZM2G068244; transcript_id TCONS_00006957; exon_number 6; oId CUFF.5921.1; tss_id TSS6274;
1	Cufflinks	exon	293723845	293724068	.	-	.	gene_id GRMZM2G068244; transcript_id TCONS_00006957; exon_number 7; oId CUFF.5921.1; tss_id TSS6274;
1	Cufflinks	exon	295340295	295341653	.	-	.	gene_id XLOC_006078; transcript_id TCONS_00006982; exon_number 1; oId CUFF.5980.2; tss_id TSS6296;
1	Cufflinks	exon	295341996	295342118	.	-	.	gene_id XLOC_006078; transcript_id TCONS_00006982; exon_number 2; oId CUFF.5980.2; tss_id TSS6296;
1	Cufflinks	exon	295342214	295342408	.	-	.	gene_id XLOC_006078; transcript_id TCONS_00006982; exon_number 3; oId CUFF.5980.2; tss_id TSS6296;
1	Cufflinks	exon	295342526	295343097	.	-	.	gene_id XLOC_006078; transcript_id TCONS_00006982; exon_number 4; oId CUFF.5980.2; tss_id TSS6296;
1	Cufflinks	exon	295613880	295614520	.	-	.	gene_id XLOC_006088; transcript_id TCONS_00006994; exon_number 1; oId CUFF.5994.1; tss_id TSS6306;
1	Cufflinks	exon	296082206	296082635	.	-	.	gene_id GRMZM2G053803; transcript_id TCONS_00007004; exon_number 1; oId CUFF.6040.1; tss_id TSS6314;
1	Cufflinks	exon	296082729	296082788	.	-	.	gene_id GRMZM2G053803; transcript_id TCONS_00007004; exon_number 2; oId CUFF.6040.1; tss_id TSS6314;
1	Cufflinks	exon	296083144	296083299	.	-	.	gene_id GRMZM2G053803; transcript_id TCONS_00007004; exon_number 3; oId CUFF.6040.1; tss_id TSS6314;
1	Cufflinks	exon	296083488	296083646	.	-	.	gene_id GRMZM2G053803; transcript_id TCONS_00007004; exon_number 4; oId CUFF.6040.1; tss_id TSS6314;
1	Cufflinks	exon	296083720	296083803	.	-	.	gene_id GRMZM2G053803; transcript_id TCONS_00007004; exon_number 5; oId CUFF.6040.1; tss_id TSS6314;
1	Cufflinks	exon	296083889	296083977	.	-	.	gene_id GRMZM2G053803; transcript_id TCONS_00007004; exon_number 6; oId CUFF.6040.1; tss_id TSS6314;
1	Cufflinks	exon	296084214	296084328	.	-	.	gene_id GRMZM2G053803; transcript_id TCONS_00007004; exon_number 7; oId CUFF.6040.1; tss_id TSS6314;
1	Cufflinks	exon	296084409	296084535	.	-	.	gene_id GRMZM2G053803; transcript_id TCONS_00007004; exon_number 8; oId CUFF.6040.1; tss_id TSS6314;
1	Cufflinks	exon	296085187	296085326	.	-	.	gene_id GRMZM2G053803; transcript_id TCONS_00007004; exon_number 9; oId CUFF.6040.1; tss_id TSS6314;
1	Cufflinks	exon	296085967	296086028	.	-	.	gene_id GRMZM2G053803; transcript_id TCONS_00007004; exon_number 10; oId CUFF.6040.1; tss_id TSS6314;
1	Cufflinks	exon	296086123	296086189	.	-	.	gene_id GRMZM2G053803; transcript_id TCONS_00007004; exon_number 11; oId CUFF.6040.1; tss_id TSS6314;
1	Cufflinks	exon	296086277	296086387	.	-	.	gene_id GRMZM2G053803; transcript_id TCONS_00007004; exon_number 12; oId CUFF.6040.1; tss_id TSS6314;
1	Cufflinks	exon	296086929	296087006	.	-	.	gene_id GRMZM2G053803; transcript_id TCONS_00007004; exon_number 13; oId CUFF.6040.1; tss_id TSS6314;
1	Cufflinks	exon	296087088	296087288	.	-	.	gene_id GRMZM2G053803; transcript_id TCONS_00007004; exon_number 14; oId CUFF.6040.1; tss_id TSS6314;
1	Cufflinks	exon	296087457	296087528	.	-	.	gene_id GRMZM2G053803; transcript_id TCONS_00007004; exon_number 15; oId CUFF.6040.1; tss_id TSS6314;
1	Cufflinks	exon	296087640	296087703	.	-	.	gene_id GRMZM2G053803; transcript_id TCONS_00007004; exon_number 16; oId CUFF.6040.1; tss_id TSS6314;
1	Cufflinks	exon	296088104	296088174	.	-	.	gene_id GRMZM2G053803; transcript_id TCONS_00007004; exon_number 17; oId CUFF.6040.1; tss_id TSS6314;
1	Cufflinks	exon	296088393	296088792	.	-	.	gene_id GRMZM2G053803; transcript_id TCONS_00007004; exon_number 18; oId CUFF.6040.1; tss_id TSS6314;
1	Cufflinks	exon	296739858	296743544	.	-	.	gene_id GRMZM2G113158; transcript_id TCONS_00007014; exon_number 1; oId CUFF.6018.1; tss_id TSS6323;
1	Cufflinks	exon	296849361	296850350	.	-	.	gene_id GRMZM2G170137; transcript_id TCONS_00007017; exon_number 1; oId CUFF.6029.1; tss_id TSS6325;
1	Cufflinks	exon	296850439	296850511	.	-	.	gene_id GRMZM2G170137; transcript_id TCONS_00007017; exon_number 2; oId CUFF.6029.1; tss_id TSS6325;
1	Cufflinks	exon	296850870	296851676	.	-	.	gene_id GRMZM2G170137; transcript_id TCONS_00007017; exon_number 3; oId CUFF.6029.1; tss_id TSS6325;
1	Cufflinks	exon	296851962	296852013	.	-	.	gene_id GRMZM2G170137; transcript_id TCONS_00007017; exon_number 4; oId CUFF.6029.1; tss_id TSS6325;
1	Cufflinks	exon	296852214	296852315	.	-	.	gene_id GRMZM2G170137; transcript_id TCONS_00007017; exon_number 5; oId CUFF.6029.1; tss_id TSS6325;
1	Cufflinks	exon	296853581	296853777	.	-	.	gene_id GRMZM2G170137; transcript_id TCONS_00007017; exon_number 6; oId CUFF.6029.1; tss_id TSS6325;
1	Cufflinks	exon	296854732	296855071	.	-	.	gene_id GRMZM2G170137; transcript_id TCONS_00007017; exon_number 7; oId CUFF.6029.1; tss_id TSS6325;
1	Cufflinks	exon	297095291	297096113	.	-	.	gene_id GRMZM2G352415; transcript_id TCONS_00007029; exon_number 1; oId CUFF.6058.1; tss_id TSS6337;
1	Cufflinks	exon	297097285	297098273	.	-	.	gene_id GRMZM2G352415; transcript_id TCONS_00007029; exon_number 2; oId CUFF.6058.1; tss_id TSS6337;

1	Cufflinks	exon	297098424	297098628	.	-	.	gene_id GRMZM2G352415; transcript_id TCONS_00007029; exon_number 3; oId CUFF.6058.1; tss_id TSS6337;
1	Cufflinks	exon	298817393	298818384	.	-	.	gene_id GRMZM2G011923; transcript_id TCONS_00007067; exon_number 1; oId CUFF.6119.1; tss_id TSS6370;
1	Cufflinks	exon	298818631	298818741	.	-	.	gene_id GRMZM2G011923; transcript_id TCONS_00007067; exon_number 2; oId CUFF.6119.1; tss_id TSS6370;
1	Cufflinks	exon	298818817	298818900	.	-	.	gene_id GRMZM2G011923; transcript_id TCONS_00007067; exon_number 3; oId CUFF.6119.1; tss_id TSS6370;
1	Cufflinks	exon	298819476	298819576	.	-	.	gene_id GRMZM2G011923; transcript_id TCONS_00007067; exon_number 4; oId CUFF.6119.1; tss_id TSS6370;
1	Cufflinks	exon	298819709	298820351	.	-	.	gene_id GRMZM2G011923; transcript_id TCONS_00007067; exon_number 5; oId CUFF.6119.1; tss_id TSS6370;
1	Cufflinks	exon	298820733	298820925	.	-	.	gene_id GRMZM2G011923; transcript_id TCONS_00007067; exon_number 6; oId CUFF.6119.1; tss_id TSS6370;
1	Cufflinks	exon	299064805	299066036	.	-	.	gene_id GRMZM2G099987; transcript_id TCONS_00007073; exon_number 1; oId CUFF.6146.1; tss_id TSS6376;
1	Cufflinks	exon	299066511	299066703	.	-	.	gene_id GRMZM2G099987; transcript_id TCONS_00007073; exon_number 2; oId CUFF.6146.1; tss_id TSS6376;
1	Cufflinks	exon	299066797	299066852	.	-	.	gene_id GRMZM2G099987; transcript_id TCONS_00007073; exon_number 3; oId CUFF.6146.1; tss_id TSS6376;
1	Cufflinks	exon	299069543	299069896	.	-	.	gene_id GRMZM2G099987; transcript_id TCONS_00007073; exon_number 4; oId CUFF.6146.1; tss_id TSS6376;
10	Cufflinks	exon	3194760	3196017	.	+	.	gene_id XLOC_006252; transcript_id TCONS_00007176; exon_number 1; oId CUFF.6334.1; tss_id TSS6475;
10	Cufflinks	exon	4614582	4614749	.	+	.	gene_id GRMZM2G022318; transcript_id TCONS_00007194; exon_number 1; oId CUFF.6387.1; tss_id TSS6492;
10	Cufflinks	exon	4615249	4615359	.	+	.	gene_id GRMZM2G022318; transcript_id TCONS_00007194; exon_number 2; oId CUFF.6387.1; tss_id TSS6492;
10	Cufflinks	exon	4615497	4615572	.	+	.	gene_id GRMZM2G022318; transcript_id TCONS_00007194; exon_number 3; oId CUFF.6387.1; tss_id TSS6492;
10	Cufflinks	exon	4615830	4617945	.	+	.	gene_id GRMZM2G022318; transcript_id TCONS_00007194; exon_number 4; oId CUFF.6387.1; tss_id TSS6492;
10	Cufflinks	exon	17611983	17612173	.	+	.	gene_id GRMZM2G009448; transcript_id TCONS_00007321; exon_number 1; oId CUFF.6646.1; tss_id TSS6612;
10	Cufflinks	exon	17613591	17613708	.	+	.	gene_id GRMZM2G009448; transcript_id TCONS_00007321; exon_number 2; oId CUFF.6646.1; tss_id TSS6612;
10	Cufflinks	exon	17613794	17613862	.	+	.	gene_id GRMZM2G009448; transcript_id TCONS_00007321; exon_number 3; oId CUFF.6646.1; tss_id TSS6612;
10	Cufflinks	exon	17613933	17614039	.	+	.	gene_id GRMZM2G009448; transcript_id TCONS_00007321; exon_number 4; oId CUFF.6646.1; tss_id TSS6612;
10	Cufflinks	exon	17614876	17615854	.	+	.	gene_id GRMZM2G009448; transcript_id TCONS_00007321; exon_number 5; oId CUFF.6646.1; tss_id TSS6612;
10	Cufflinks	exon	18744368	18744700	.	+	.	gene_id AC198699.3_FG004; transcript_id TCONS_00007328; exon_number 1; oId CUFF.6658.1; tss_id TSS6619;
10	Cufflinks	exon	18744779	18745364	.	+	.	gene_id AC198699.3_FG004; transcript_id TCONS_00007328; exon_number 2; oId CUFF.6658.1; tss_id TSS6619;
10	Cufflinks	exon	20328528	20329030	.	+	.	gene_id XLOC_006404; transcript_id TCONS_00007344; exon_number 1; oId CUFF.6674.1; tss_id TSS6633;
10	Cufflinks	exon	20329305	20329584	.	+	.	gene_id XLOC_006404; transcript_id TCONS_00007344; exon_number 2; oId CUFF.6674.1; tss_id TSS6633;
10	Cufflinks	exon	23221552	23221949	.	+	.	gene_id GRMZM2G036427; transcript_id TCONS_00007363; exon_number 1; oId CUFF.6724.1; tss_id TSS6652;
10	Cufflinks	exon	23222052	23222140	.	+	.	gene_id GRMZM2G036427; transcript_id TCONS_00007363; exon_number 2; oId CUFF.6724.1; tss_id TSS6652;
10	Cufflinks	exon	23222367	23222488	.	+	.	gene_id GRMZM2G036427; transcript_id TCONS_00007363; exon_number 3; oId CUFF.6724.1; tss_id TSS6652;
10	Cufflinks	exon	23223350	23223794	.	+	.	gene_id GRMZM2G036427; transcript_id TCONS_00007363; exon_number 4; oId CUFF.6724.1; tss_id TSS6652;
10	Cufflinks	exon	29229592	29229865	.	+	.	gene_id GRMZM2G420407; transcript_id TCONS_00007389; exon_number 1; oId CUFF.6810.1; tss_id TSS6677;
10	Cufflinks	exon	29229942	29231490	.	+	.	gene_id GRMZM2G420407; transcript_id TCONS_00007389; exon_number 2; oId CUFF.6810.1; tss_id TSS6677;
10	Cufflinks	exon	29631122	29631541	.	+	.	gene_id GRMZM2G102683; transcript_id TCONS_00007393; exon_number 1; oId CUFF.6801.1; tss_id TSS6681;
10	Cufflinks	exon	29631741	29632087	.	+	.	gene_id GRMZM2G102683; transcript_id TCONS_00007393; exon_number 2; oId CUFF.6801.1; tss_id TSS6681;
10	Cufflinks	exon	29633627	29634845	.	+	.	gene_id GRMZM2G102683; transcript_id TCONS_00007393; exon_number 3; oId CUFF.6801.1; tss_id TSS6681;
10	Cufflinks	exon	58462136	58462386	.	+	.	gene_id GRMZM2G178817; transcript_id TCONS_00007515; exon_number 1; oId CUFF.7009.1; tss_id TSS6791;
10	Cufflinks	exon	58462548	58463278	.	+	.	gene_id GRMZM2G178817; transcript_id TCONS_00007515; exon_number 2; oId CUFF.7009.1; tss_id TSS6791;

10	Cufflinks	exon	77282595	77283483	.	+	.	gene_id GRMZM2G110145; transcript_id TCONS_00007635; exon_number 1; oId CUFF.7255.2; tss_id TSS6898;
10	Cufflinks	exon	77285486	77286245	.	+	.	gene_id GRMZM2G110145; transcript_id TCONS_00007635; exon_number 2; oId CUFF.7255.2; tss_id TSS6898;
10	Cufflinks	exon	77286911	77289172	.	+	.	gene_id GRMZM2G110145; transcript_id TCONS_00007635; exon_number 3; oId CUFF.7255.2; tss_id TSS6898;
10	Cufflinks	exon	80056275	80056729	.	+	.	gene_id GRMZM2G086496; transcript_id TCONS_00007660; exon_number 1; oId CUFF.7291.1; tss_id TSS6920;
10	Cufflinks	exon	80056853	80057070	.	+	.	gene_id GRMZM2G086496; transcript_id TCONS_00007660; exon_number 2; oId CUFF.7291.1; tss_id TSS6920;
10	Cufflinks	exon	80057710	80058314	.	+	.	gene_id GRMZM2G086496; transcript_id TCONS_00007660; exon_number 3; oId CUFF.7291.1; tss_id TSS6920;
10	Cufflinks	exon	80061269	80062527	.	+	.	gene_id GRMZM2G086496; transcript_id TCONS_00007660; exon_number 4; oId CUFF.7291.1; tss_id TSS6920;
10	Cufflinks	exon	81841212	81841481	.	+	.	gene_id XLOC_006709; transcript_id TCONS_00007686; exon_number 1; oId CUFF.7313.1; tss_id TSS6944;
10	Cufflinks	exon	90257150	90259318	.	+	.	gene_id GRMZM2G138252; transcript_id TCONS_00007788; exon_number 1; oId CUFF.7473.1; tss_id TSS7037;
10	Cufflinks	exon	92550666	92550769	.	+	.	gene_id GRMZM2G110567; transcript_id TCONS_00007804; exon_number 1; oId CUFF.7526.1; tss_id TSS7052;
10	Cufflinks	exon	92550940	92552639	.	+	.	gene_id GRMZM2G110567; transcript_id TCONS_00007804; exon_number 2; oId CUFF.7526.1; tss_id TSS7052;
10	Cufflinks	exon	109913509	109913628	.	+	.	gene_id GRMZM2G122437; transcript_id TCONS_00007965; exon_number 1; oId CUFF.7796.1; tss_id TSS7196;
10	Cufflinks	exon	109913770	109914019	.	+	.	gene_id GRMZM2G122437; transcript_id TCONS_00007965; exon_number 2; oId CUFF.7796.1; tss_id TSS7196;
10	Cufflinks	exon	109914358	109914514	.	+	.	gene_id GRMZM2G122437; transcript_id TCONS_00007965; exon_number 3; oId CUFF.7796.1; tss_id TSS7196;
10	Cufflinks	exon	109914536	109914602	.	+	.	gene_id GRMZM2G122437; transcript_id TCONS_00007965; exon_number 4; oId CUFF.7796.1; tss_id TSS7196;
10	Cufflinks	exon	109914730	109914817	.	+	.	gene_id GRMZM2G122437; transcript_id TCONS_00007965; exon_number 5; oId CUFF.7796.1; tss_id TSS7196;
10	Cufflinks	exon	109914939	109916009	.	+	.	gene_id GRMZM2G122437; transcript_id TCONS_00007965; exon_number 6; oId CUFF.7796.1; tss_id TSS7196;
10	Cufflinks	exon	112222702	112222861	.	+	.	gene_id GRMZM2G138355; transcript_id TCONS_00007983; exon_number 1; oId CUFF.7832.2; tss_id TSS7211;
10	Cufflinks	exon	112223013	112223219	.	+	.	gene_id GRMZM2G138355; transcript_id TCONS_00007983; exon_number 2; oId CUFF.7832.2; tss_id TSS7211;
10	Cufflinks	exon	112223339	112223422	.	+	.	gene_id GRMZM2G138355; transcript_id TCONS_00007983; exon_number 3; oId CUFF.7832.2; tss_id TSS7211;
10	Cufflinks	exon	112224957	112225109	.	+	.	gene_id GRMZM2G138355; transcript_id TCONS_00007983; exon_number 4; oId CUFF.7832.2; tss_id TSS7211;
10	Cufflinks	exon	112225226	112225808	.	+	.	gene_id GRMZM2G138355; transcript_id TCONS_00007983; exon_number 5; oId CUFF.7832.2; tss_id TSS7211;
10	Cufflinks	exon	119842960	119843231	.	+	.	gene_id XLOC_007032; transcript_id TCONS_00008047; exon_number 1; oId CUFF.7961.1; tss_id TSS7272;
10	Cufflinks	exon	126160075	126160753	.	+	.	gene_id GRMZM2G359735; transcript_id TCONS_00008137; exon_number 1; oId CUFF.8122.1; tss_id TSS7354;
10	Cufflinks	exon	126161559	126161628	.	+	.	gene_id GRMZM2G359735; transcript_id TCONS_00008137; exon_number 2; oId CUFF.8122.1; tss_id TSS7354;
10	Cufflinks	exon	126161739	126161911	.	+	.	gene_id GRMZM2G359735; transcript_id TCONS_00008137; exon_number 3; oId CUFF.8122.1; tss_id TSS7354;
10	Cufflinks	exon	126162033	126162207	.	+	.	gene_id GRMZM2G359735; transcript_id TCONS_00008137; exon_number 4; oId CUFF.8122.1; tss_id TSS7354;
10	Cufflinks	exon	126162294	126162460	.	+	.	gene_id GRMZM2G359735; transcript_id TCONS_00008137; exon_number 5; oId CUFF.8122.1; tss_id TSS7354;
10	Cufflinks	exon	126163299	126163518	.	+	.	gene_id GRMZM2G359735; transcript_id TCONS_00008137; exon_number 6; oId CUFF.8122.1; tss_id TSS7354;
10	Cufflinks	exon	126163600	126163704	.	+	.	gene_id GRMZM2G359735; transcript_id TCONS_00008137; exon_number 7; oId CUFF.8122.1; tss_id TSS7354;
10	Cufflinks	exon	126163951	126164103	.	+	.	gene_id GRMZM2G359735; transcript_id TCONS_00008137; exon_number 8; oId CUFF.8122.1; tss_id TSS7354;
10	Cufflinks	exon	126165004	126165445	.	+	.	gene_id GRMZM2G359735; transcript_id TCONS_00008137; exon_number 9; oId CUFF.8122.1; tss_id TSS7354;
10	Cufflinks	exon	132197230	132197495	.	+	.	gene_id GRMZM2G131969; transcript_id TCONS_00008240; exon_number 1; oId CUFF.8334.2; tss_id TSS7443;
10	Cufflinks	exon	132198409	132198688	.	+	.	gene_id GRMZM2G131969; transcript_id TCONS_00008240; exon_number 2; oId CUFF.8334.2; tss_id TSS7443;
10	Cufflinks	exon	132198817	132198888	.	+	.	gene_id GRMZM2G131969; transcript_id TCONS_00008240; exon_number 3; oId CUFF.8334.2; tss_id TSS7443;
10	Cufflinks	exon	132198984	132199042	.	+	.	gene_id GRMZM2G131969; transcript_id TCONS_00008240; exon_number 4; oId CUFF.8334.2; tss_id TSS7443;

10	Cufflinks	exon	132199137	132199272	.	+	.	gene_id GRMZM2G131969; transcript_id TCONS_00008240; exon_number 5; oId CUFF.8334.2; tss_id TSS7443;
10	Cufflinks	exon	132199456	132199552	.	+	.	gene_id GRMZM2G131969; transcript_id TCONS_00008240; exon_number 6; oId CUFF.8334.2; tss_id TSS7443;
10	Cufflinks	exon	132199647	132199713	.	+	.	gene_id GRMZM2G131969; transcript_id TCONS_00008240; exon_number 7; oId CUFF.8334.2; tss_id TSS7443;
10	Cufflinks	exon	132200165	132200353	.	+	.	gene_id GRMZM2G131969; transcript_id TCONS_00008240; exon_number 8; oId CUFF.8334.2; tss_id TSS7443;
10	Cufflinks	exon	132200509	132200663	.	+	.	gene_id GRMZM2G131969; transcript_id TCONS_00008240; exon_number 9; oId CUFF.8334.2; tss_id TSS7443;
10	Cufflinks	exon	132200800	132200901	.	+	.	gene_id GRMZM2G131969; transcript_id TCONS_00008240; exon_number 10; oId CUFF.8334.2; tss_id TSS7443;
10	Cufflinks	exon	132200987	132201623	.	+	.	gene_id GRMZM2G131969; transcript_id TCONS_00008240; exon_number 11; oId CUFF.8334.2; tss_id TSS7443;
10	Cufflinks	exon	132569729	132572415	.	+	.	gene_id GRMZM2G168953; transcript_id TCONS_00008254; exon_number 1; oId CUFF.8296.1; tss_id TSS7454;
10	Cufflinks	exon	136721249	136721959	.	+	.	gene_id XLOC_007279; transcript_id TCONS_00008334; exon_number 1; oId CUFF.8436.1; tss_id TSS7526;
10	Cufflinks	exon	137853661	137854077	.	+	.	gene_id GRMZM2G142832; transcript_id TCONS_00008354; exon_number 1; oId CUFF.8513.2; tss_id TSS7544;
10	Cufflinks	exon	137854285	137854742	.	+	.	gene_id GRMZM2G142832; transcript_id TCONS_00008354; exon_number 2; oId CUFF.8513.2; tss_id TSS7544;
10	Cufflinks	exon	137855164	137855299	.	+	.	gene_id GRMZM2G142832; transcript_id TCONS_00008354; exon_number 3; oId CUFF.8513.2; tss_id TSS7544;
10	Cufflinks	exon	137855605	137855747	.	+	.	gene_id GRMZM2G142832; transcript_id TCONS_00008354; exon_number 4; oId CUFF.8513.2; tss_id TSS7544;
10	Cufflinks	exon	137855856	137855979	.	+	.	gene_id GRMZM2G142832; transcript_id TCONS_00008354; exon_number 5; oId CUFF.8513.2; tss_id TSS7544;
10	Cufflinks	exon	137856096	137856863	.	+	.	gene_id GRMZM2G142832; transcript_id TCONS_00008354; exon_number 6; oId CUFF.8513.2; tss_id TSS7544;
10	Cufflinks	exon	137853661	137854077	.	+	.	gene_id GRMZM2G142832; transcript_id TCONS_00008353; exon_number 1; oId CUFF.8513.1; tss_id TSS7544;
10	Cufflinks	exon	137854282	137854742	.	+	.	gene_id GRMZM2G142832; transcript_id TCONS_00008353; exon_number 2; oId CUFF.8513.1; tss_id TSS7544;
10	Cufflinks	exon	137855164	137855299	.	+	.	gene_id GRMZM2G142832; transcript_id TCONS_00008353; exon_number 3; oId CUFF.8513.1; tss_id TSS7544;
10	Cufflinks	exon	137855605	137855747	.	+	.	gene_id GRMZM2G142832; transcript_id TCONS_00008353; exon_number 4; oId CUFF.8513.1; tss_id TSS7544;
10	Cufflinks	exon	137855856	137855979	.	+	.	gene_id GRMZM2G142832; transcript_id TCONS_00008353; exon_number 5; oId CUFF.8513.1; tss_id TSS7544;
10	Cufflinks	exon	137856096	137856863	.	+	.	gene_id GRMZM2G142832; transcript_id TCONS_00008353; exon_number 6; oId CUFF.8513.1; tss_id TSS7544;
10	Cufflinks	exon	137853709	137854077	.	+	.	gene_id GRMZM2G142832; transcript_id TCONS_00008357; exon_number 1; oId CUFF.8513.5; tss_id TSS7544;
10	Cufflinks	exon	137854285	137854742	.	+	.	gene_id GRMZM2G142832; transcript_id TCONS_00008357; exon_number 2; oId CUFF.8513.5; tss_id TSS7544;
10	Cufflinks	exon	137855164	137855747	.	+	.	gene_id GRMZM2G142832; transcript_id TCONS_00008357; exon_number 3; oId CUFF.8513.5; tss_id TSS7544;
10	Cufflinks	exon	137855856	137855979	.	+	.	gene_id GRMZM2G142832; transcript_id TCONS_00008357; exon_number 4; oId CUFF.8513.5; tss_id TSS7544;
10	Cufflinks	exon	137856096	137856863	.	+	.	gene_id GRMZM2G142832; transcript_id TCONS_00008357; exon_number 5; oId CUFF.8513.5; tss_id TSS7544;
10	Cufflinks	exon	138512572	138514126	.	+	.	gene_id XLOC_007308; transcript_id TCONS_00008371; exon_number 1; oId CUFF.8479.1; tss_id TSS7557;
10	Cufflinks	exon	142868690	142869159	.	+	.	gene_id GRMZM2G148495; transcript_id TCONS_00008463; exon_number 1; oId CUFF.8647.1; tss_id TSS7643;
10	Cufflinks	exon	142869274	142869363	.	+	.	gene_id GRMZM2G148495; transcript_id TCONS_00008463; exon_number 2; oId CUFF.8647.1; tss_id TSS7643;
10	Cufflinks	exon	142869490	142869793	.	+	.	gene_id GRMZM2G148495; transcript_id TCONS_00008463; exon_number 3; oId CUFF.8647.1; tss_id TSS7643;
10	Cufflinks	exon	143178859	143179364	.	+	.	gene_id GRMZM2G431504; transcript_id TCONS_00008469; exon_number 1; oId CUFF.8673.1; tss_id TSS7648;
10	Cufflinks	exon	143179479	143179685	.	+	.	gene_id GRMZM2G431504; transcript_id TCONS_00008469; exon_number 2; oId CUFF.8673.1; tss_id TSS7648;
10	Cufflinks	exon	143179815	143180188	.	+	.	gene_id GRMZM2G431504; transcript_id TCONS_00008469; exon_number 3; oId CUFF.8673.1; tss_id TSS7648;
10	Cufflinks	exon	143180652	143181050	.	+	.	gene_id GRMZM2G431504; transcript_id TCONS_00008469; exon_number 4; oId CUFF.8673.1; tss_id TSS7648;
10	Cufflinks	exon	143487116	143488331	.	+	.	gene_id GRMZM5G895313; transcript_id TCONS_00008476; exon_number 1; oId CUFF.8671.1; tss_id TSS7655;
10	Cufflinks	exon	143740959	143741706	.	+	.	gene_id GRMZM5G818977; transcript_id TCONS_00008489; exon_number 1; oId CUFF.8698.1; tss_id TSS7668;

10	Cufflinks	exon	143742335	143744585	.	+	.	gene_id GRMZM5G818977; transcript_id TCONS_00008489; exon_number 2; oId CUFF.8698.1; tss_id TSS7668;
10	Cufflinks	exon	146745295	146747273	.	+	.	gene_id GRMZM2G179532; transcript_id TCONS_00008570; exon_number 1; oId CUFF.8834.1; tss_id TSS7742;
10	Cufflinks	exon	147855454	147855891	.	+	.	gene_id GRMZM2G142757; transcript_id TCONS_00008602; exon_number 1; oId CUFF.8909.1; tss_id TSS7774;
10	Cufflinks	exon	147858250	147858403	.	+	.	gene_id GRMZM2G142757; transcript_id TCONS_00008602; exon_number 2; oId CUFF.8909.1; tss_id TSS7774;
10	Cufflinks	exon	147858503	147859264	.	+	.	gene_id GRMZM2G142757; transcript_id TCONS_00008602; exon_number 3; oId CUFF.8909.1; tss_id TSS7774;
10	Cufflinks	exon	148253993	148255416	.	+	.	gene_id GRMZM2G106792; transcript_id TCONS_00008618; exon_number 1; oId CUFF.8936.1; tss_id TSS7788;
10	Cufflinks	exon	148284757	148286459	.	+	.	gene_id XLOC_007531; transcript_id TCONS_00008619; exon_number 1; oId CUFF.8940.1; tss_id TSS7789;
10	Cufflinks	exon	148407014	148407321	.	+	.	gene_id GRMZM2G104613; transcript_id TCONS_00008627; exon_number 1; oId CUFF.8975.1; tss_id TSS7797;
10	Cufflinks	exon	148407434	148407563	.	+	.	gene_id GRMZM2G104613; transcript_id TCONS_00008627; exon_number 2; oId CUFF.8975.1; tss_id TSS7797;
10	Cufflinks	exon	148407704	148407815	.	+	.	gene_id GRMZM2G104613; transcript_id TCONS_00008627; exon_number 3; oId CUFF.8975.1; tss_id TSS7797;
10	Cufflinks	exon	148408095	148408170	.	+	.	gene_id GRMZM2G104613; transcript_id TCONS_00008627; exon_number 4; oId CUFF.8975.1; tss_id TSS7797;
10	Cufflinks	exon	148408294	148408382	.	+	.	gene_id GRMZM2G104613; transcript_id TCONS_00008627; exon_number 5; oId CUFF.8975.1; tss_id TSS7797;
10	Cufflinks	exon	148408626	148408712	.	+	.	gene_id GRMZM2G104613; transcript_id TCONS_00008627; exon_number 6; oId CUFF.8975.1; tss_id TSS7797;
10	Cufflinks	exon	148408991	148409107	.	+	.	gene_id GRMZM2G104613; transcript_id TCONS_00008627; exon_number 7; oId CUFF.8975.1; tss_id TSS7797;
10	Cufflinks	exon	148409208	148409315	.	+	.	gene_id GRMZM2G104613; transcript_id TCONS_00008627; exon_number 8; oId CUFF.8975.1; tss_id TSS7797;
10	Cufflinks	exon	148409425	148409478	.	+	.	gene_id GRMZM2G104613; transcript_id TCONS_00008627; exon_number 9; oId CUFF.8975.1; tss_id TSS7797;
10	Cufflinks	exon	148409589	148409741	.	+	.	gene_id GRMZM2G104613; transcript_id TCONS_00008627; exon_number 10; oId CUFF.8975.1; tss_id TSS7797;
10	Cufflinks	exon	148410205	148410710	.	+	.	gene_id GRMZM2G104613; transcript_id TCONS_00008627; exon_number 11; oId CUFF.8975.1; tss_id TSS7797;
10	Cufflinks	exon	5645510	5647289	.	-	.	gene_id GRMZM2G164113; transcript_id TCONS_00008796; exon_number 1; oId CUFF.6454.2; tss_id TSS7952;
10	Cufflinks	exon	5647374	5647506	.	-	.	gene_id GRMZM2G164113; transcript_id TCONS_00008796; exon_number 2; oId CUFF.6454.2; tss_id TSS7952;
10	Cufflinks	exon	5648069	5648162	.	-	.	gene_id GRMZM2G164113; transcript_id TCONS_00008796; exon_number 3; oId CUFF.6454.2; tss_id TSS7952;
10	Cufflinks	exon	5648832	5648918	.	-	.	gene_id GRMZM2G164113; transcript_id TCONS_00008796; exon_number 4; oId CUFF.6454.2; tss_id TSS7952;
10	Cufflinks	exon	5649006	5649063	.	-	.	gene_id GRMZM2G164113; transcript_id TCONS_00008796; exon_number 5; oId CUFF.6454.2; tss_id TSS7952;
10	Cufflinks	exon	5649683	5649746	.	-	.	gene_id GRMZM2G164113; transcript_id TCONS_00008796; exon_number 6; oId CUFF.6454.2; tss_id TSS7952;
10	Cufflinks	exon	5649928	5649989	.	-	.	gene_id GRMZM2G164113; transcript_id TCONS_00008796; exon_number 7; oId CUFF.6454.2; tss_id TSS7952;
10	Cufflinks	exon	5650069	5650120	.	-	.	gene_id GRMZM2G164113; transcript_id TCONS_00008796; exon_number 8; oId CUFF.6454.2; tss_id TSS7952;
10	Cufflinks	exon	5650202	5650274	.	-	.	gene_id GRMZM2G164113; transcript_id TCONS_00008796; exon_number 9; oId CUFF.6454.2; tss_id TSS7952;
10	Cufflinks	exon	5650373	5650448	.	-	.	gene_id GRMZM2G164113; transcript_id TCONS_00008796; exon_number 10; oId CUFF.6454.2; tss_id TSS7952;
10	Cufflinks	exon	5650553	5650629	.	-	.	gene_id GRMZM2G164113; transcript_id TCONS_00008796; exon_number 11; oId CUFF.6454.2; tss_id TSS7952;
10	Cufflinks	exon	5651400	5651577	.	-	.	gene_id GRMZM2G164113; transcript_id TCONS_00008796; exon_number 12; oId CUFF.6454.2; tss_id TSS7952;
10	Cufflinks	exon	5645510	5647273	.	-	.	gene_id GRMZM2G164113; transcript_id TCONS_00008795; exon_number 1; oId CUFF.6454.1; tss_id TSS7952;
10	Cufflinks	exon	5647374	5647506	.	-	.	gene_id GRMZM2G164113; transcript_id TCONS_00008795; exon_number 2; oId CUFF.6454.1; tss_id TSS7952;
10	Cufflinks	exon	5648069	5648162	.	-	.	gene_id GRMZM2G164113; transcript_id TCONS_00008795; exon_number 3; oId CUFF.6454.1; tss_id TSS7952;
10	Cufflinks	exon	5648832	5648918	.	-	.	gene_id GRMZM2G164113; transcript_id TCONS_00008795; exon_number 4; oId CUFF.6454.1; tss_id TSS7952;
10	Cufflinks	exon	5649006	5649063	.	-	.	gene_id GRMZM2G164113; transcript_id TCONS_00008795; exon_number 5; oId CUFF.6454.1; tss_id TSS7952;
10	Cufflinks	exon	5649683	5649746	.	-	.	gene_id GRMZM2G164113; transcript_id TCONS_00008795; exon_number 6; oId CUFF.6454.1; tss_id TSS7952;

10	Cufflinks	exon	5649928	5649989	.	-	.	gene_id GRMZM2G164113; transcript_id TCONS_00008795; exon_number 7; oId CUFF.6454.1; tss_id TSS7952;
10	Cufflinks	exon	5650069	5650120	.	-	.	gene_id GRMZM2G164113; transcript_id TCONS_00008795; exon_number 8; oId CUFF.6454.1; tss_id TSS7952;
10	Cufflinks	exon	5650202	5650274	.	-	.	gene_id GRMZM2G164113; transcript_id TCONS_00008795; exon_number 9; oId CUFF.6454.1; tss_id TSS7952;
10	Cufflinks	exon	5650373	5650448	.	-	.	gene_id GRMZM2G164113; transcript_id TCONS_00008795; exon_number 10; oId CUFF.6454.1; tss_id TSS7952;
10	Cufflinks	exon	5650553	5650629	.	-	.	gene_id GRMZM2G164113; transcript_id TCONS_00008795; exon_number 11; oId CUFF.6454.1; tss_id TSS7952;
10	Cufflinks	exon	5651400	5651577	.	-	.	gene_id GRMZM2G164113; transcript_id TCONS_00008795; exon_number 12; oId CUFF.6454.1; tss_id TSS7952;
10	Cufflinks	exon	6533850	6534518	.	-	.	gene_id XLOC_007708; transcript_id TCONS_00008811; exon_number 1; oId CUFF.6467.1; tss_id TSS7967;
10	Cufflinks	exon	8853009	8853474	.	-	.	gene_id GRMZM2G136910; transcript_id TCONS_00008835; exon_number 1; oId CUFF.6509.1; tss_id TSS7990;
10	Cufflinks	exon	8853606	8853996	.	-	.	gene_id GRMZM2G136910; transcript_id TCONS_00008835; exon_number 2; oId CUFF.6509.1; tss_id TSS7990;
10	Cufflinks	exon	10887778	10888819	.	-	.	gene_id GRMZM5G813470; transcript_id TCONS_00008864; exon_number 1; oId CUFF.6573.2; tss_id TSS8017;
10	Cufflinks	exon	10888893	10888990	.	-	.	gene_id GRMZM5G813470; transcript_id TCONS_00008864; exon_number 2; oId CUFF.6573.2; tss_id TSS8017;
10	Cufflinks	exon	10889076	10889204	.	-	.	gene_id GRMZM5G813470; transcript_id TCONS_00008864; exon_number 3; oId CUFF.6573.2; tss_id TSS8017;
10	Cufflinks	exon	10889872	10890156	.	-	.	gene_id GRMZM5G813470; transcript_id TCONS_00008864; exon_number 4; oId CUFF.6573.2; tss_id TSS8017;
10	Cufflinks	exon	10890887	10892009	.	-	.	gene_id GRMZM5G813470; transcript_id TCONS_00008864; exon_number 5; oId CUFF.6573.2; tss_id TSS8017;
10	Cufflinks	exon	10887778	10888819	.	-	.	gene_id GRMZM5G813470; transcript_id TCONS_00008865; exon_number 1; oId CUFF.6573.1; tss_id TSS8018;
10	Cufflinks	exon	10888893	10888990	.	-	.	gene_id GRMZM5G813470; transcript_id TCONS_00008865; exon_number 2; oId CUFF.6573.1; tss_id TSS8018;
10	Cufflinks	exon	10889076	10889204	.	-	.	gene_id GRMZM5G813470; transcript_id TCONS_00008865; exon_number 3; oId CUFF.6573.1; tss_id TSS8018;
10	Cufflinks	exon	10889872	10890156	.	-	.	gene_id GRMZM5G813470; transcript_id TCONS_00008865; exon_number 4; oId CUFF.6573.1; tss_id TSS8018;
10	Cufflinks	exon	10890887	10891473	.	-	.	gene_id GRMZM5G813470; transcript_id TCONS_00008865; exon_number 5; oId CUFF.6573.1; tss_id TSS8018;
10	Cufflinks	exon	10891759	10892143	.	-	.	gene_id GRMZM5G813470; transcript_id TCONS_00008865; exon_number 6; oId CUFF.6573.1; tss_id TSS8018;
10	Cufflinks	exon	19075431	19076884	.	-	.	gene_id GRMZM2G169240; transcript_id TCONS_00008934; exon_number 1; oId CUFF.6663.1; tss_id TSS8081;
10	Cufflinks	exon	19077357	19077551	.	-	.	gene_id GRMZM2G169240; transcript_id TCONS_00008934; exon_number 2; oId CUFF.6663.1; tss_id TSS8081;
10	Cufflinks	exon	23390500	23391073	.	-	.	gene_id XLOC_007844; transcript_id TCONS_00008967; exon_number 1; oId CUFF.6715.1; tss_id TSS8109;
10	Cufflinks	exon	23392234	23392415	.	-	.	gene_id XLOC_007844; transcript_id TCONS_00008967; exon_number 2; oId CUFF.6715.1; tss_id TSS8109;
10	Cufflinks	exon	25562436	25563192	.	-	.	gene_id GRMZM2G054250; transcript_id TCONS_00008990; exon_number 1; oId CUFF.6761.1; tss_id TSS8131;
10	Cufflinks	exon	25563265	25563537	.	-	.	gene_id GRMZM2G054250; transcript_id TCONS_00008990; exon_number 2; oId CUFF.6761.1; tss_id TSS8131;
10	Cufflinks	exon	25563736	25564012	.	-	.	gene_id GRMZM2G054250; transcript_id TCONS_00008990; exon_number 3; oId CUFF.6761.1; tss_id TSS8131;
10	Cufflinks	exon	25564099	25564244	.	-	.	gene_id GRMZM2G054250; transcript_id TCONS_00008990; exon_number 4; oId CUFF.6761.1; tss_id TSS8131;
10	Cufflinks	exon	25564737	25565712	.	-	.	gene_id GRMZM2G054250; transcript_id TCONS_00008990; exon_number 5; oId CUFF.6761.1; tss_id TSS8131;
10	Cufflinks	exon	68937520	68938909	.	-	.	gene_id GRMZM2G364748; transcript_id TCONS_00009196; exon_number 1; oId CUFF.7129.1; tss_id TSS8321;
10	Cufflinks	exon	79422406	79423830	.	-	.	gene_id XLOC_008140; transcript_id TCONS_00009293; exon_number 1; oId CUFF.7271.1; tss_id TSS8412;
10	Cufflinks	exon	79424059	79424752	.	-	.	gene_id XLOC_008141; transcript_id TCONS_00009294; exon_number 1; oId CUFF.7269.1; tss_id TSS8413;
10	Cufflinks	exon	81840736	81841920	.	-	.	gene_id GRMZM2G325907; transcript_id TCONS_00009306; exon_number 1; oId CUFF.7312.1; tss_id TSS8425;
10	Cufflinks	exon	81842791	81842920	.	-	.	gene_id GRMZM2G325907; transcript_id TCONS_00009306; exon_number 2; oId CUFF.7312.1; tss_id TSS8425;
10	Cufflinks	exon	81843123	81843543	.	-	.	gene_id GRMZM2G325907; transcript_id TCONS_00009306; exon_number 3; oId CUFF.7312.1; tss_id TSS8425;
10	Cufflinks	exon	86066475	86067579	.	-	.	gene_id XLOC_008193; transcript_id TCONS_00009349; exon_number 1; oId CUFF.7406.1; tss_id TSS8466;

10	Cufflinks	exon	96774485	96775014	.	-	.	gene_id GRMZM2G330635; transcript_id TCONS_00009453; exon_number 1; oId CUFF.7584.1; tss_id TSS8563;
10	Cufflinks	exon	96775090	96775465	.	-	.	gene_id GRMZM2G330635; transcript_id TCONS_00009453; exon_number 2; oId CUFF.7584.1; tss_id TSS8563;
10	Cufflinks	exon	99220164	99220972	.	-	.	gene_id GRMZM2G019246; transcript_id TCONS_00009473; exon_number 1; oId CUFF.7633.1; tss_id TSS8581;
10	Cufflinks	exon	99221074	99221155	.	-	.	gene_id GRMZM2G019246; transcript_id TCONS_00009473; exon_number 2; oId CUFF.7633.1; tss_id TSS8581;
10	Cufflinks	exon	99221268	99221351	.	-	.	gene_id GRMZM2G019246; transcript_id TCONS_00009473; exon_number 3; oId CUFF.7633.1; tss_id TSS8581;
10	Cufflinks	exon	99221454	99221636	.	-	.	gene_id GRMZM2G019246; transcript_id TCONS_00009473; exon_number 4; oId CUFF.7633.1; tss_id TSS8581;
10	Cufflinks	exon	99221741	99221918	.	-	.	gene_id GRMZM2G019246; transcript_id TCONS_00009473; exon_number 5; oId CUFF.7633.1; tss_id TSS8581;
10	Cufflinks	exon	99222025	99223049	.	-	.	gene_id GRMZM2G019246; transcript_id TCONS_00009473; exon_number 6; oId CUFF.7633.1; tss_id TSS8581;
10	Cufflinks	exon	131910570	131911984	.	-	.	gene_id GRMZM5G893444; transcript_id TCONS_00009851; exon_number 1; oId CUFF.8266.1; tss_id TSS8908;
10	Cufflinks	exon	131912070	131912259	.	-	.	gene_id GRMZM5G893444; transcript_id TCONS_00009851; exon_number 2; oId CUFF.8266.1; tss_id TSS8908;
10	Cufflinks	exon	131912346	131912480	.	-	.	gene_id GRMZM5G893444; transcript_id TCONS_00009851; exon_number 3; oId CUFF.8266.1; tss_id TSS8908;
10	Cufflinks	exon	131912570	131912667	.	-	.	gene_id GRMZM5G893444; transcript_id TCONS_00009851; exon_number 4; oId CUFF.8266.1; tss_id TSS8908;
10	Cufflinks	exon	131912764	131912927	.	-	.	gene_id GRMZM5G893444; transcript_id TCONS_00009851; exon_number 5; oId CUFF.8266.1; tss_id TSS8908;
10	Cufflinks	exon	131913997	131914628	.	-	.	gene_id GRMZM5G893444; transcript_id TCONS_00009851; exon_number 6; oId CUFF.8266.1; tss_id TSS8908;
10	Cufflinks	exon	132446398	132447255	.	-	.	gene_id GRMZM2G046841; transcript_id TCONS_00009859; exon_number 1; oId CUFF.8280.1; tss_id TSS8915;
10	Cufflinks	exon	136041206	136042977	.	-	.	gene_id XLOC_008678; transcript_id TCONS_00009915; exon_number 1; oId CUFF.8416.1; tss_id TSS8965;
10	Cufflinks	exon	136718978	136720291	.	-	.	gene_id XLOC_008699; transcript_id TCONS_00009940; exon_number 1; oId CUFF.8435.1; tss_id TSS8986;
10	Cufflinks	exon	136721526	136721714	.	-	.	gene_id XLOC_008699; transcript_id TCONS_00009940; exon_number 2; oId CUFF.8435.1; tss_id TSS8986;
10	Cufflinks	exon	138512829	138514242	.	-	.	gene_id GRMZM5G803874; transcript_id TCONS_00009963; exon_number 1; oId CUFF.8481.1; tss_id TSS9006;
10	Cufflinks	exon	138515199	138515377	.	-	.	gene_id GRMZM5G803874; transcript_id TCONS_00009963; exon_number 2; oId CUFF.8481.1; tss_id TSS9006;
10	Cufflinks	exon	140384896	140385416	.	-	.	gene_id GRMZM2G129169; transcript_id TCONS_00010002; exon_number 1; oId CUFF.8701.1; tss_id TSS9045;
10	Cufflinks	exon	140385492	140385699	.	-	.	gene_id GRMZM2G129169; transcript_id TCONS_00010002; exon_number 2; oId CUFF.8701.1; tss_id TSS9045;
10	Cufflinks	exon	140386049	140386179	.	-	.	gene_id GRMZM2G129169; transcript_id TCONS_00010002; exon_number 3; oId CUFF.8701.1; tss_id TSS9045;
10	Cufflinks	exon	140386330	140386401	.	-	.	gene_id GRMZM2G129169; transcript_id TCONS_00010002; exon_number 4; oId CUFF.8701.1; tss_id TSS9045;
10	Cufflinks	exon	140386578	140386664	.	-	.	gene_id GRMZM2G129169; transcript_id TCONS_00010002; exon_number 5; oId CUFF.8701.1; tss_id TSS9045;
10	Cufflinks	exon	140386764	140386790	.	-	.	gene_id GRMZM2G129169; transcript_id TCONS_00010002; exon_number 6; oId CUFF.8701.1; tss_id TSS9045;
10	Cufflinks	exon	140387079	140387171	.	-	.	gene_id GRMZM2G129169; transcript_id TCONS_00010002; exon_number 7; oId CUFF.8701.1; tss_id TSS9045;
10	Cufflinks	exon	140388053	140388141	.	-	.	gene_id GRMZM2G129169; transcript_id TCONS_00010002; exon_number 8; oId CUFF.8701.1; tss_id TSS9045;
10	Cufflinks	exon	140388226	140388304	.	-	.	gene_id GRMZM2G129169; transcript_id TCONS_00010002; exon_number 9; oId CUFF.8701.1; tss_id TSS9045;
10	Cufflinks	exon	140388609	140388738	.	-	.	gene_id GRMZM2G129169; transcript_id TCONS_00010002; exon_number 10; oId CUFF.8701.1; tss_id TSS9045;
10	Cufflinks	exon	140388845	140388965	.	-	.	gene_id GRMZM2G129169; transcript_id TCONS_00010002; exon_number 11; oId CUFF.8701.1; tss_id TSS9045;
10	Cufflinks	exon	140389123	140389230	.	-	.	gene_id GRMZM2G129169; transcript_id TCONS_00010002; exon_number 12; oId CUFF.8701.1; tss_id TSS9045;
10	Cufflinks	exon	140389331	140389821	.	-	.	gene_id GRMZM2G129169; transcript_id TCONS_00010002; exon_number 13; oId CUFF.8701.1; tss_id TSS9045;
10	Cufflinks	exon	141312645	141312975	.	-	.	gene_id GRMZM2G147408; transcript_id TCONS_00010028; exon_number 1; oId CUFF.8572.1; tss_id TSS9067;
10	Cufflinks	exon	141313057	141313335	.	-	.	gene_id GRMZM2G147408; transcript_id TCONS_00010028; exon_number 2; oId CUFF.8572.1; tss_id TSS9067;
10	Cufflinks	exon	143487540	143488048	.	-	.	gene_id XLOC_008827; transcript_id TCONS_00010089; exon_number 1; oId CUFF.8672.1; tss_id TSS9122;

10	Cufflinks	exon	147014559	147015018	.	-	.	gene_id GRMZM2G150014; transcript_id TCONS_00010194; exon_number 1; oId CUFF.8866.2; tss_id TSS9207;
10	Cufflinks	exon	147015269	147015971	.	-	.	gene_id GRMZM2G150014; transcript_id TCONS_00010194; exon_number 2; oId CUFF.8866.2; tss_id TSS9207;
10	Cufflinks	exon	147016910	147017338	.	-	.	gene_id GRMZM2G150014; transcript_id TCONS_00010194; exon_number 3; oId CUFF.8866.2; tss_id TSS9207;
10	Cufflinks	exon	147014559	147015018	.	-	.	gene_id GRMZM2G150014; transcript_id TCONS_00010195; exon_number 1; oId CUFF.8866.1; tss_id TSS9208;
10	Cufflinks	exon	147015269	147015541	.	-	.	gene_id GRMZM2G150014; transcript_id TCONS_00010195; exon_number 2; oId CUFF.8866.1; tss_id TSS9208;
10	Cufflinks	exon	147015831	147015971	.	-	.	gene_id GRMZM2G150014; transcript_id TCONS_00010195; exon_number 3; oId CUFF.8866.1; tss_id TSS9208;
10	Cufflinks	exon	147016910	147017456	.	-	.	gene_id GRMZM2G150014; transcript_id TCONS_00010195; exon_number 4; oId CUFF.8866.1; tss_id TSS9208;
10	Cufflinks	exon	147043160	147043404	.	-	.	gene_id GRMZM2G150286; transcript_id TCONS_00010202; exon_number 1; oId CUFF.8876.2; tss_id TSS9212;
10	Cufflinks	exon	147045249	147045370	.	-	.	gene_id GRMZM2G150286; transcript_id TCONS_00010202; exon_number 2; oId CUFF.8876.2; tss_id TSS9212;
10	Cufflinks	exon	147045451	147045718	.	-	.	gene_id GRMZM2G150286; transcript_id TCONS_00010202; exon_number 3; oId CUFF.8876.2; tss_id TSS9212;
10	Cufflinks	exon	147043160	147043404	.	-	.	gene_id GRMZM2G150286; transcript_id TCONS_00010201; exon_number 1; oId CUFF.8876.1; tss_id TSS9212;
10	Cufflinks	exon	147043714	147043761	.	-	.	gene_id GRMZM2G150286; transcript_id TCONS_00010201; exon_number 2; oId CUFF.8876.1; tss_id TSS9212;
10	Cufflinks	exon	147045249	147045370	.	-	.	gene_id GRMZM2G150286; transcript_id TCONS_00010201; exon_number 3; oId CUFF.8876.1; tss_id TSS9212;
10	Cufflinks	exon	147045451	147045718	.	-	.	gene_id GRMZM2G150286; transcript_id TCONS_00010201; exon_number 4; oId CUFF.8876.1; tss_id TSS9212;
10	Cufflinks	exon	147868922	147869350	.	-	.	gene_id GRMZM2G142820; transcript_id TCONS_00010227; exon_number 1; oId CUFF.8915.1; tss_id TSS9234;
10	Cufflinks	exon	147869440	147869634	.	-	.	gene_id GRMZM2G142820; transcript_id TCONS_00010227; exon_number 2; oId CUFF.8915.1; tss_id TSS9234;
10	Cufflinks	exon	147870029	147870133	.	-	.	gene_id GRMZM2G142820; transcript_id TCONS_00010227; exon_number 3; oId CUFF.8915.1; tss_id TSS9234;
10	Cufflinks	exon	147870233	147870313	.	-	.	gene_id GRMZM2G142820; transcript_id TCONS_00010227; exon_number 4; oId CUFF.8915.1; tss_id TSS9234;
10	Cufflinks	exon	147870725	147870824	.	-	.	gene_id GRMZM2G142820; transcript_id TCONS_00010227; exon_number 5; oId CUFF.8915.1; tss_id TSS9234;
10	Cufflinks	exon	147871791	147872174	.	-	.	gene_id GRMZM2G142820; transcript_id TCONS_00010227; exon_number 6; oId CUFF.8915.1; tss_id TSS9234;
10	Cufflinks	exon	148065497	148067197	.	-	.	gene_id GRMZM2G074759; transcript_id TCONS_00010241; exon_number 1; oId CUFF.8934.1; tss_id TSS9247;
10	Cufflinks	exon	148067314	148067641	.	-	.	gene_id GRMZM2G074759; transcript_id TCONS_00010241; exon_number 2; oId CUFF.8934.1; tss_id TSS9247;
10	Cufflinks	exon	148254333	148255179	.	-	.	gene_id XLOC_008952; transcript_id TCONS_00010244; exon_number 1; oId CUFF.8938.1; tss_id TSS9250;
10	Cufflinks	exon	148312549	148313668	.	-	.	gene_id GRMZM2G001934; transcript_id TCONS_00010249; exon_number 1; oId CUFF.8951.1; tss_id TSS9255;
10	Cufflinks	exon	148313738	148316275	.	-	.	gene_id GRMZM2G001934; transcript_id TCONS_00010249; exon_number 2; oId CUFF.8951.1; tss_id TSS9255;
10	Cufflinks	exon	149245361	149248323	.	-	.	gene_id GRMZM5G836471; transcript_id TCONS_00010269; exon_number 1; oId CUFF.9003.1; tss_id TSS9272;
10	Cufflinks	exon	149248550	149248678	.	-	.	gene_id GRMZM5G836471; transcript_id TCONS_00010269; exon_number 2; oId CUFF.9003.1; tss_id TSS9272;
10	Cufflinks	exon	149248771	149248892	.	-	.	gene_id GRMZM5G836471; transcript_id TCONS_00010269; exon_number 3; oId CUFF.9003.1; tss_id TSS9272;
10	Cufflinks	exon	149249350	149249415	.	-	.	gene_id GRMZM5G836471; transcript_id TCONS_00010269; exon_number 4; oId CUFF.9003.1; tss_id TSS9272;
10	Cufflinks	exon	149250738	149250806	.	-	.	gene_id GRMZM5G836471; transcript_id TCONS_00010269; exon_number 5; oId CUFF.9003.1; tss_id TSS9272;
10	Cufflinks	exon	149250899	149251026	.	-	.	gene_id GRMZM5G836471; transcript_id TCONS_00010269; exon_number 6; oId CUFF.9003.1; tss_id TSS9272;
10	Cufflinks	exon	149251155	149251265	.	-	.	gene_id GRMZM5G836471; transcript_id TCONS_00010269; exon_number 7; oId CUFF.9003.1; tss_id TSS9272;
2	Cufflinks	exon	1038129	1038395	.	+	.	gene_id GRMZM2G064212; transcript_id TCONS_00010285; exon_number 1; oId CUFF.9029.1; tss_id TSS9287;
2	Cufflinks	exon	1038895	1039421	.	+	.	gene_id GRMZM2G064212; transcript_id TCONS_00010285; exon_number 2; oId CUFF.9029.1; tss_id TSS9287;
2	Cufflinks	exon	1039507	1039592	.	+	.	gene_id GRMZM2G064212; transcript_id TCONS_00010285; exon_number 3; oId CUFF.9029.1; tss_id TSS9287;
2	Cufflinks	exon	1039701	1039751	.	+	.	gene_id GRMZM2G064212; transcript_id TCONS_00010285; exon_number 4; oId CUFF.9029.1; tss_id TSS9287;

2	Cufflinks	exon	1039881	1040266	.	+	.	gene_id GRMZM2G064212; transcript_id TCONS_00010285; exon_number 5; oId CUFF.9029.1; tss_id TSS9287;
2	Cufflinks	exon	1040610	1040748	.	+	.	gene_id GRMZM2G064212; transcript_id TCONS_00010285; exon_number 6; oId CUFF.9029.1; tss_id TSS9287;
2	Cufflinks	exon	1041451	1041506	.	+	.	gene_id GRMZM2G064212; transcript_id TCONS_00010285; exon_number 7; oId CUFF.9029.1; tss_id TSS9287;
2	Cufflinks	exon	1041612	1041682	.	+	.	gene_id GRMZM2G064212; transcript_id TCONS_00010285; exon_number 8; oId CUFF.9029.1; tss_id TSS9287;
2	Cufflinks	exon	1041772	1042294	.	+	.	gene_id GRMZM2G064212; transcript_id TCONS_00010285; exon_number 9; oId CUFF.9029.1; tss_id TSS9287;
2	Cufflinks	exon	1038233	1038794	.	+	.	gene_id GRMZM2G064212; transcript_id TCONS_00010286; exon_number 1; oId CUFF.9029.2; tss_id TSS9288;
2	Cufflinks	exon	1038895	1039421	.	+	.	gene_id GRMZM2G064212; transcript_id TCONS_00010286; exon_number 2; oId CUFF.9029.2; tss_id TSS9288;
2	Cufflinks	exon	1039507	1039592	.	+	.	gene_id GRMZM2G064212; transcript_id TCONS_00010286; exon_number 3; oId CUFF.9029.2; tss_id TSS9288;
2	Cufflinks	exon	1039701	1039751	.	+	.	gene_id GRMZM2G064212; transcript_id TCONS_00010286; exon_number 4; oId CUFF.9029.2; tss_id TSS9288;
2	Cufflinks	exon	1039881	1040266	.	+	.	gene_id GRMZM2G064212; transcript_id TCONS_00010286; exon_number 5; oId CUFF.9029.2; tss_id TSS9288;
2	Cufflinks	exon	1040610	1040748	.	+	.	gene_id GRMZM2G064212; transcript_id TCONS_00010286; exon_number 6; oId CUFF.9029.2; tss_id TSS9288;
2	Cufflinks	exon	1041451	1041506	.	+	.	gene_id GRMZM2G064212; transcript_id TCONS_00010286; exon_number 7; oId CUFF.9029.2; tss_id TSS9288;
2	Cufflinks	exon	1041612	1041682	.	+	.	gene_id GRMZM2G064212; transcript_id TCONS_00010286; exon_number 8; oId CUFF.9029.2; tss_id TSS9288;
2	Cufflinks	exon	1041772	1042294	.	+	.	gene_id GRMZM2G064212; transcript_id TCONS_00010286; exon_number 9; oId CUFF.9029.2; tss_id TSS9288;
2	Cufflinks	exon	1678757	1679543	.	+	.	gene_id GRMZM2G149958; transcript_id TCONS_00010312; exon_number 1; oId CUFF.9060.1; tss_id TSS9311;
2	Cufflinks	exon	1681930	1682890	.	+	.	gene_id GRMZM2G149958; transcript_id TCONS_00010312; exon_number 2; oId CUFF.9060.1; tss_id TSS9311;
2	Cufflinks	exon	2366176	2366675	.	+	.	gene_id GRMZM2G078033; transcript_id TCONS_00010340; exon_number 1; oId CUFF.9104.1; tss_id TSS9336;
2	Cufflinks	exon	2366876	2366991	.	+	.	gene_id GRMZM2G078033; transcript_id TCONS_00010340; exon_number 2; oId CUFF.9104.1; tss_id TSS9336;
2	Cufflinks	exon	2367111	2367199	.	+	.	gene_id GRMZM2G078033; transcript_id TCONS_00010340; exon_number 3; oId CUFF.9104.1; tss_id TSS9336;
2	Cufflinks	exon	2367304	2367417	.	+	.	gene_id GRMZM2G078033; transcript_id TCONS_00010340; exon_number 4; oId CUFF.9104.1; tss_id TSS9336;
2	Cufflinks	exon	2367508	2367595	.	+	.	gene_id GRMZM2G078033; transcript_id TCONS_00010340; exon_number 5; oId CUFF.9104.1; tss_id TSS9336;
2	Cufflinks	exon	2367687	2367772	.	+	.	gene_id GRMZM2G078033; transcript_id TCONS_00010340; exon_number 6; oId CUFF.9104.1; tss_id TSS9336;
2	Cufflinks	exon	2367858	2367960	.	+	.	gene_id GRMZM2G078033; transcript_id TCONS_00010340; exon_number 7; oId CUFF.9104.1; tss_id TSS9336;
2	Cufflinks	exon	2368182	2368279	.	+	.	gene_id GRMZM2G078033; transcript_id TCONS_00010340; exon_number 8; oId CUFF.9104.1; tss_id TSS9336;
2	Cufflinks	exon	2368664	2369093	.	+	.	gene_id GRMZM2G078033; transcript_id TCONS_00010340; exon_number 9; oId CUFF.9104.1; tss_id TSS9336;
2	Cufflinks	exon	2369177	2369867	.	+	.	gene_id GRMZM2G078033; transcript_id TCONS_00010340; exon_number 10; oId CUFF.9104.1; tss_id TSS9336;
2	Cufflinks	exon	3191332	3192257	.	+	.	gene_id GRMZM2G119941; transcript_id TCONS_00010374; exon_number 1; oId CUFF.9153.1; tss_id TSS9369;
2	Cufflinks	exon	3192709	3192734	.	+	.	gene_id GRMZM2G119941; transcript_id TCONS_00010374; exon_number 2; oId CUFF.9153.1; tss_id TSS9369;
2	Cufflinks	exon	3192832	3193329	.	+	.	gene_id GRMZM2G119941; transcript_id TCONS_00010374; exon_number 3; oId CUFF.9153.1; tss_id TSS9369;
2	Cufflinks	exon	3193407	3193885	.	+	.	gene_id GRMZM2G119941; transcript_id TCONS_00010374; exon_number 4; oId CUFF.9153.1; tss_id TSS9369;
2	Cufflinks	exon	3229402	3229711	.	+	.	gene_id GRMZM2G018716; transcript_id TCONS_00010375; exon_number 1; oId CUFF.9157.1; tss_id TSS9370;
2	Cufflinks	exon	3229836	3229844	.	+	.	gene_id GRMZM2G018716; transcript_id TCONS_00010375; exon_number 2; oId CUFF.9157.1; tss_id TSS9370;
2	Cufflinks	exon	3229957	3231226	.	+	.	gene_id GRMZM2G018716; transcript_id TCONS_00010375; exon_number 3; oId CUFF.9157.1; tss_id TSS9370;
2	Cufflinks	exon	3231356	3231449	.	+	.	gene_id GRMZM2G018716; transcript_id TCONS_00010375; exon_number 4; oId CUFF.9157.1; tss_id TSS9370;
2	Cufflinks	exon	3231538	3232016	.	+	.	gene_id GRMZM2G018716; transcript_id TCONS_00010375; exon_number 5; oId CUFF.9157.1; tss_id TSS9370;
2	Cufflinks	exon	4192323	4192742	.	+	.	gene_id GRMZM2G040095; transcript_id TCONS_00010409; exon_number 1; oId CUFF.9205.1; tss_id TSS9399;

2	Cufflinks	exon	4192866	4193149	.	+	.	gene_id GRMZM2G040095; transcript_id TCONS_00010409; exon_number 2; oId CUFF.9205.1; tss_id TSS9399;
2	Cufflinks	exon	4193258	4193507	.	+	.	gene_id GRMZM2G040095; transcript_id TCONS_00010409; exon_number 3; oId CUFF.9205.1; tss_id TSS9399;
2	Cufflinks	exon	4193634	4193867	.	+	.	gene_id GRMZM2G040095; transcript_id TCONS_00010409; exon_number 4; oId CUFF.9205.1; tss_id TSS9399;
2	Cufflinks	exon	4193965	4194158	.	+	.	gene_id GRMZM2G040095; transcript_id TCONS_00010409; exon_number 5; oId CUFF.9205.1; tss_id TSS9399;
2	Cufflinks	exon	4194299	4194657	.	+	.	gene_id GRMZM2G040095; transcript_id TCONS_00010409; exon_number 6; oId CUFF.9205.1; tss_id TSS9399;
2	Cufflinks	exon	4194724	4195017	.	+	.	gene_id GRMZM2G040095; transcript_id TCONS_00010409; exon_number 7; oId CUFF.9205.1; tss_id TSS9399;
2	Cufflinks	exon	4195101	4196291	.	+	.	gene_id GRMZM2G040095; transcript_id TCONS_00010409; exon_number 8; oId CUFF.9205.1; tss_id TSS9399;
2	Cufflinks	exon	4839281	4839592	.	+	.	gene_id GRMZM2G033430; transcript_id TCONS_00010435; exon_number 1; oId CUFF.9258.1; tss_id TSS9423;
2	Cufflinks	exon	4840069	4840210	.	+	.	gene_id GRMZM2G033430; transcript_id TCONS_00010435; exon_number 2; oId CUFF.9258.1; tss_id TSS9423;
2	Cufflinks	exon	4840296	4840397	.	+	.	gene_id GRMZM2G033430; transcript_id TCONS_00010435; exon_number 3; oId CUFF.9258.1; tss_id TSS9423;
2	Cufflinks	exon	4840701	4840774	.	+	.	gene_id GRMZM2G033430; transcript_id TCONS_00010435; exon_number 4; oId CUFF.9258.1; tss_id TSS9423;
2	Cufflinks	exon	4840941	4841272	.	+	.	gene_id GRMZM2G033430; transcript_id TCONS_00010435; exon_number 5; oId CUFF.9258.1; tss_id TSS9423;
2	Cufflinks	exon	5235067	5236283	.	+	.	gene_id GRMZM2G160585; transcript_id TCONS_00010451; exon_number 1; oId CUFF.9277.1; tss_id TSS9437;
2	Cufflinks	exon	5236443	5236937	.	+	.	gene_id GRMZM2G160585; transcript_id TCONS_00010451; exon_number 2; oId CUFF.9277.1; tss_id TSS9437;
2	Cufflinks	exon	5238471	5239077	.	+	.	gene_id GRMZM2G160585; transcript_id TCONS_00010451; exon_number 3; oId CUFF.9277.1; tss_id TSS9437;
2	Cufflinks	exon	5568332	5570261	.	+	.	gene_id GRMZM2G174917; transcript_id TCONS_00010460; exon_number 1; oId CUFF.9279.1; tss_id TSS9446;
2	Cufflinks	exon	10343672	10344452	.	+	.	gene_id GRMZM2G005310; transcript_id TCONS_00010598; exon_number 1; oId CUFF.9518.1; tss_id TSS9562;
2	Cufflinks	exon	10344569	10344665	.	+	.	gene_id GRMZM2G005310; transcript_id TCONS_00010598; exon_number 2; oId CUFF.9518.1; tss_id TSS9562;
2	Cufflinks	exon	10346335	10346442	.	+	.	gene_id GRMZM2G005310; transcript_id TCONS_00010598; exon_number 3; oId CUFF.9518.1; tss_id TSS9562;
2	Cufflinks	exon	10346514	10346651	.	+	.	gene_id GRMZM2G005310; transcript_id TCONS_00010598; exon_number 4; oId CUFF.9518.1; tss_id TSS9562;
2	Cufflinks	exon	10346728	10346827	.	+	.	gene_id GRMZM2G005310; transcript_id TCONS_00010598; exon_number 5; oId CUFF.9518.1; tss_id TSS9562;
2	Cufflinks	exon	10349822	10350000	.	+	.	gene_id GRMZM2G005310; transcript_id TCONS_00010598; exon_number 6; oId CUFF.9518.1; tss_id TSS9562;
2	Cufflinks	exon	10350081	10350146	.	+	.	gene_id GRMZM2G005310; transcript_id TCONS_00010598; exon_number 7; oId CUFF.9518.1; tss_id TSS9562;
2	Cufflinks	exon	10350229	10350441	.	+	.	gene_id GRMZM2G005310; transcript_id TCONS_00010598; exon_number 8; oId CUFF.9518.1; tss_id TSS9562;
2	Cufflinks	exon	10350571	10350629	.	+	.	gene_id GRMZM2G005310; transcript_id TCONS_00010598; exon_number 9; oId CUFF.9518.1; tss_id TSS9562;
2	Cufflinks	exon	10350706	10350772	.	+	.	gene_id GRMZM2G005310; transcript_id TCONS_00010598; exon_number 10; oId CUFF.9518.1; tss_id TSS9562;
2	Cufflinks	exon	10350860	10351189	.	+	.	gene_id GRMZM2G005310; transcript_id TCONS_00010598; exon_number 11; oId CUFF.9518.1; tss_id TSS9562;
2	Cufflinks	exon	10351280	10351348	.	+	.	gene_id GRMZM2G005310; transcript_id TCONS_00010598; exon_number 12; oId CUFF.9518.1; tss_id TSS9562;
2	Cufflinks	exon	10351420	10351506	.	+	.	gene_id GRMZM2G005310; transcript_id TCONS_00010598; exon_number 13; oId CUFF.9518.1; tss_id TSS9562;
2	Cufflinks	exon	10351575	10351662	.	+	.	gene_id GRMZM2G005310; transcript_id TCONS_00010598; exon_number 14; oId CUFF.9518.1; tss_id TSS9562;
2	Cufflinks	exon	10351779	10351900	.	+	.	gene_id GRMZM2G005310; transcript_id TCONS_00010598; exon_number 15; oId CUFF.9518.1; tss_id TSS9562;
2	Cufflinks	exon	10351977	10352081	.	+	.	gene_id GRMZM2G005310; transcript_id TCONS_00010598; exon_number 16; oId CUFF.9518.1; tss_id TSS9562;
2	Cufflinks	exon	10352226	10352269	.	+	.	gene_id GRMZM2G005310; transcript_id TCONS_00010598; exon_number 17; oId CUFF.9518.1; tss_id TSS9562;
2	Cufflinks	exon	10352360	10352429	.	+	.	gene_id GRMZM2G005310; transcript_id TCONS_00010598; exon_number 18; oId CUFF.9518.1; tss_id TSS9562;
2	Cufflinks	exon	10352525	10352628	.	+	.	gene_id GRMZM2G005310; transcript_id TCONS_00010598; exon_number 19; oId CUFF.9518.1; tss_id TSS9562;
2	Cufflinks	exon	10352863	10353395	.	+	.	gene_id GRMZM2G005310; transcript_id TCONS_00010598; exon_number 20; oId CUFF.9518.1; tss_id TSS9562;

2	Cufflinks	exon	11769293	11771169	.	+	.	gene_id GRMZM2G071832; transcript_id TCONS_00010630; exon_number 1; oId CUFF.9560.1; tss_id TSS9591;
2	Cufflinks	exon	13552922	13553314	.	+	.	gene_id GRMZM2G131280; transcript_id TCONS_00010679; exon_number 1; oId CUFF.9637.1; tss_id TSS9634;
2	Cufflinks	exon	13553444	13554160	.	+	.	gene_id GRMZM2G131280; transcript_id TCONS_00010679; exon_number 2; oId CUFF.9637.1; tss_id TSS9634;
2	Cufflinks	exon	13554256	13555183	.	+	.	gene_id GRMZM2G131280; transcript_id TCONS_00010679; exon_number 3; oId CUFF.9637.1; tss_id TSS9634;
2	Cufflinks	exon	13553144	13553314	.	+	.	gene_id GRMZM2G131280; transcript_id TCONS_00010680; exon_number 1; oId CUFF.9637.2; tss_id TSS9635;
2	Cufflinks	exon	13553444	13553644	.	+	.	gene_id GRMZM2G131280; transcript_id TCONS_00010680; exon_number 2; oId CUFF.9637.2; tss_id TSS9635;
2	Cufflinks	exon	13553945	13554160	.	+	.	gene_id GRMZM2G131280; transcript_id TCONS_00010680; exon_number 3; oId CUFF.9637.2; tss_id TSS9635;
2	Cufflinks	exon	13554256	13555183	.	+	.	gene_id GRMZM2G131280; transcript_id TCONS_00010680; exon_number 4; oId CUFF.9637.2; tss_id TSS9635;
2	Cufflinks	exon	14732437	14733097	.	+	.	gene_id XLOC_009355; transcript_id TCONS_00010711; exon_number 1; oId CUFF.9675.1; tss_id TSS9665;
2	Cufflinks	exon	23123884	23124296	.	+	.	gene_id GRMZM5G810061; transcript_id TCONS_00010846; exon_number 1; oId CUFF.9930.2; tss_id TSS9787;
2	Cufflinks	exon	23125390	23126798	.	+	.	gene_id GRMZM5G810061; transcript_id TCONS_00010846; exon_number 2; oId CUFF.9930.2; tss_id TSS9787;
2	Cufflinks	exon	23126961	23127473	.	+	.	gene_id GRMZM5G810061; transcript_id TCONS_00010846; exon_number 3; oId CUFF.9930.2; tss_id TSS9787;
2	Cufflinks	exon	23127583	23128108	.	+	.	gene_id GRMZM5G810061; transcript_id TCONS_00010846; exon_number 4; oId CUFF.9930.2; tss_id TSS9787;
2	Cufflinks	exon	27115035	27115497	.	+	.	gene_id GRMZM2G138125; transcript_id TCONS_00010919; exon_number 1; oId CUFF.10053.1; tss_id TSS9849;
2	Cufflinks	exon	27115584	27116494	.	+	.	gene_id GRMZM2G138125; transcript_id TCONS_00010919; exon_number 2; oId CUFF.10053.1; tss_id TSS9849;
2	Cufflinks	exon	27116968	27117098	.	+	.	gene_id GRMZM2G138125; transcript_id TCONS_00010919; exon_number 3; oId CUFF.10053.1; tss_id TSS9849;
2	Cufflinks	exon	27117188	27117242	.	+	.	gene_id GRMZM2G138125; transcript_id TCONS_00010919; exon_number 4; oId CUFF.10053.1; tss_id TSS9849;
2	Cufflinks	exon	27117444	27117686	.	+	.	gene_id GRMZM2G138125; transcript_id TCONS_00010919; exon_number 5; oId CUFF.10053.1; tss_id TSS9849;
2	Cufflinks	exon	27117782	27117837	.	+	.	gene_id GRMZM2G138125; transcript_id TCONS_00010919; exon_number 6; oId CUFF.10053.1; tss_id TSS9849;
2	Cufflinks	exon	27117935	27118025	.	+	.	gene_id GRMZM2G138125; transcript_id TCONS_00010919; exon_number 7; oId CUFF.10053.1; tss_id TSS9849;
2	Cufflinks	exon	27120591	27120728	.	+	.	gene_id GRMZM2G138125; transcript_id TCONS_00010919; exon_number 8; oId CUFF.10053.1; tss_id TSS9849;
2	Cufflinks	exon	27120815	27120898	.	+	.	gene_id GRMZM2G138125; transcript_id TCONS_00010919; exon_number 9; oId CUFF.10053.1; tss_id TSS9849;
2	Cufflinks	exon	27121103	27121312	.	+	.	gene_id GRMZM2G138125; transcript_id TCONS_00010919; exon_number 10; oId CUFF.10053.1; tss_id TSS9849;
2	Cufflinks	exon	27121398	27121469	.	+	.	gene_id GRMZM2G138125; transcript_id TCONS_00010919; exon_number 11; oId CUFF.10053.1; tss_id TSS9849;
2	Cufflinks	exon	27122071	27122151	.	+	.	gene_id GRMZM2G138125; transcript_id TCONS_00010919; exon_number 12; oId CUFF.10053.1; tss_id TSS9849;
2	Cufflinks	exon	27122235	27122353	.	+	.	gene_id GRMZM2G138125; transcript_id TCONS_00010919; exon_number 13; oId CUFF.10053.1; tss_id TSS9849;
2	Cufflinks	exon	27122449	27122560	.	+	.	gene_id GRMZM2G138125; transcript_id TCONS_00010919; exon_number 14; oId CUFF.10053.1; tss_id TSS9849;
2	Cufflinks	exon	27122918	27122986	.	+	.	gene_id GRMZM2G138125; transcript_id TCONS_00010919; exon_number 15; oId CUFF.10053.1; tss_id TSS9849;
2	Cufflinks	exon	27123068	27123162	.	+	.	gene_id GRMZM2G138125; transcript_id TCONS_00010919; exon_number 16; oId CUFF.10053.1; tss_id TSS9849;
2	Cufflinks	exon	27123517	27123645	.	+	.	gene_id GRMZM2G138125; transcript_id TCONS_00010919; exon_number 17; oId CUFF.10053.1; tss_id TSS9849;
2	Cufflinks	exon	27123785	27123856	.	+	.	gene_id GRMZM2G138125; transcript_id TCONS_00010919; exon_number 18; oId CUFF.10053.1; tss_id TSS9849;
2	Cufflinks	exon	27123934	27124066	.	+	.	gene_id GRMZM2G138125; transcript_id TCONS_00010919; exon_number 19; oId CUFF.10053.1; tss_id TSS9849;
2	Cufflinks	exon	27124141	27125249	.	+	.	gene_id GRMZM2G138125; transcript_id TCONS_00010919; exon_number 20; oId CUFF.10053.1; tss_id TSS9849;
2	Cufflinks	exon	28745695	28746203	.	+	.	gene_id GRMZM2G441347; transcript_id TCONS_00010948; exon_number 1; oId CUFF.10072.1; tss_id TSS9876;
2	Cufflinks	exon	28746831	28748913	.	+	.	gene_id GRMZM2G441347; transcript_id TCONS_00010948; exon_number 2; oId CUFF.10072.1; tss_id TSS9876;
2	Cufflinks	exon	31214984	31215355	.	+	.	gene_id GRMZM2G082191; transcript_id TCONS_00010985; exon_number 1; oId CUFF.10164.2; tss_id TSS9910;

2	Cufflinks	exon	31216046	31219041	.	+	.	gene_id GRMZM2G082191; transcript_id TCONS_00010985; exon_number 2; oId CUFF.10164.2; tss_id TSS9910;
2	Cufflinks	exon	31219609	31222672	.	+	.	gene_id GRMZM2G082191; transcript_id TCONS_00010985; exon_number 3; oId CUFF.10164.2; tss_id TSS9910;
2	Cufflinks	exon	31214991	31215797	.	+	.	gene_id GRMZM2G082191; transcript_id TCONS_00010986; exon_number 1; oId CUFF.10164.3; tss_id TSS9910;
2	Cufflinks	exon	31216046	31219041	.	+	.	gene_id GRMZM2G082191; transcript_id TCONS_00010986; exon_number 2; oId CUFF.10164.3; tss_id TSS9910;
2	Cufflinks	exon	31219609	31222672	.	+	.	gene_id GRMZM2G082191; transcript_id TCONS_00010986; exon_number 3; oId CUFF.10164.3; tss_id TSS9910;
2	Cufflinks	exon	34359265	34360412	.	+	.	gene_id XLOC_009629; transcript_id TCONS_00011033; exon_number 1; oId CUFF.10227.1; tss_id TSS9953;
2	Cufflinks	exon	34655098	34655300	.	+	.	gene_id XLOC_009631; transcript_id TCONS_00011035; exon_number 1; oId CUFF.10264.1; tss_id TSS9955;
2	Cufflinks	exon	36945636	36946149	.	+	.	gene_id GRMZM2G102183; transcript_id TCONS_00011054; exon_number 1; oId CUFF.10283.1; tss_id TSS9970;
2	Cufflinks	exon	36946271	36946596	.	+	.	gene_id GRMZM2G102183; transcript_id TCONS_00011054; exon_number 2; oId CUFF.10283.1; tss_id TSS9970;
2	Cufflinks	exon	36946754	36947084	.	+	.	gene_id GRMZM2G102183; transcript_id TCONS_00011054; exon_number 3; oId CUFF.10283.1; tss_id TSS9970;
2	Cufflinks	exon	36947162	36947798	.	+	.	gene_id GRMZM2G102183; transcript_id TCONS_00011054; exon_number 4; oId CUFF.10283.1; tss_id TSS9970;
2	Cufflinks	exon	36947888	36948162	.	+	.	gene_id GRMZM2G102183; transcript_id TCONS_00011054; exon_number 5; oId CUFF.10283.1; tss_id TSS9970;
2	Cufflinks	exon	38821032	38821727	.	+	.	gene_id GRMZM2G121700; transcript_id TCONS_00011085; exon_number 1; oId CUFF.10336.1; tss_id TSS9997;
2	Cufflinks	exon	38821854	38822178	.	+	.	gene_id GRMZM2G121700; transcript_id TCONS_00011085; exon_number 2; oId CUFF.10336.1; tss_id TSS9997;
2	Cufflinks	exon	38822268	38822692	.	+	.	gene_id GRMZM2G121700; transcript_id TCONS_00011085; exon_number 3; oId CUFF.10336.1; tss_id TSS9997;
2	Cufflinks	exon	44106201	44106798	.	+	.	gene_id GRMZM2G360374; transcript_id TCONS_00011160; exon_number 1; oId CUFF.10456.1; tss_id TSS10065;
2	Cufflinks	exon	44107169	44109990	.	+	.	gene_id GRMZM2G360374; transcript_id TCONS_00011160; exon_number 2; oId CUFF.10456.1; tss_id TSS10065;
2	Cufflinks	exon	44829337	44831648	.	+	.	gene_id GRMZM2G171277; transcript_id TCONS_00011172; exon_number 1; oId CUFF.10471.1; tss_id TSS10074;
2	Cufflinks	exon	48775553	48778515	.	+	.	gene_id GRMZM2G053111; transcript_id TCONS_00011212; exon_number 1; oId CUFF.10558.1; tss_id TSS10113;
2	Cufflinks	exon	54473290	54474043	.	+	.	gene_id GRMZM2G044132; transcript_id TCONS_00011270; exon_number 1; oId CUFF.10670.1; tss_id TSS10170;
2	Cufflinks	exon	54474217	54474810	.	+	.	gene_id GRMZM2G044132; transcript_id TCONS_00011270; exon_number 2; oId CUFF.10670.1; tss_id TSS10170;
2	Cufflinks	exon	62920594	62920794	.	+	.	gene_id GRMZM2G032225; transcript_id TCONS_00011326; exon_number 1; oId CUFF.10789.1; tss_id TSS10224;
2	Cufflinks	exon	62920891	62920986	.	+	.	gene_id GRMZM2G032225; transcript_id TCONS_00011326; exon_number 2; oId CUFF.10789.1; tss_id TSS10224;
2	Cufflinks	exon	62921092	62922074	.	+	.	gene_id GRMZM2G032225; transcript_id TCONS_00011326; exon_number 3; oId CUFF.10789.1; tss_id TSS10224;
2	Cufflinks	exon	64294921	64296104	.	+	.	gene_id GRMZM2G436533; transcript_id TCONS_00011337; exon_number 1; oId CUFF.10822.2; tss_id TSS10233;
2	Cufflinks	exon	64296926	64296976	.	+	.	gene_id GRMZM2G436533; transcript_id TCONS_00011337; exon_number 2; oId CUFF.10822.2; tss_id TSS10233;
2	Cufflinks	exon	64297994	64298527	.	+	.	gene_id GRMZM2G436533; transcript_id TCONS_00011337; exon_number 3; oId CUFF.10822.2; tss_id TSS10233;
2	Cufflinks	exon	68168970	68169431	.	+	.	gene_id GRMZM2G086267; transcript_id TCONS_00011366; exon_number 1; oId CUFF.10845.1; tss_id TSS10255;
2	Cufflinks	exon	68171435	68171605	.	+	.	gene_id GRMZM2G086267; transcript_id TCONS_00011366; exon_number 2; oId CUFF.10845.1; tss_id TSS10255;
2	Cufflinks	exon	68174243	68174713	.	+	.	gene_id GRMZM2G086267; transcript_id TCONS_00011366; exon_number 3; oId CUFF.10845.1; tss_id TSS10255;
2	Cufflinks	exon	124226792	124229015	.	+	.	gene_id XLOC_010163; transcript_id TCONS_00011631; exon_number 1; oId CUFF.11359.1; tss_id TSS10497;
2	Cufflinks	exon	128454614	128458045	.	+	.	gene_id GRMZM2G301355; transcript_id TCONS_00011648; exon_number 1; oId CUFF.11396.1; tss_id TSS10512;
2	Cufflinks	exon	134470091	134471300	.	+	.	gene_id GRMZM2G111143; transcript_id TCONS_00011674; exon_number 1; oId CUFF.11449.2; tss_id TSS10536;
2	Cufflinks	exon	134472049	134472331	.	+	.	gene_id GRMZM2G111143; transcript_id TCONS_00011674; exon_number 2; oId CUFF.11449.2; tss_id TSS10536;
2	Cufflinks	exon	134472432	134472840	.	+	.	gene_id GRMZM2G111143; transcript_id TCONS_00011674; exon_number 3; oId CUFF.11449.2; tss_id TSS10536;
2	Cufflinks	exon	134470091	134471300	.	+	.	gene_id GRMZM2G111143; transcript_id TCONS_00011673; exon_number 1; oId CUFF.11449.1; tss_id TSS10536;

2	Cufflinks	exon	134472046	134472331	.	+	.	gene_id GRMZM2G111143; transcript_id TCONS_00011673; exon_number 2; oId CUFF.11449.1; tss_id TSS10536;
2	Cufflinks	exon	134472432	134472840	.	+	.	gene_id GRMZM2G111143; transcript_id TCONS_00011673; exon_number 3; oId CUFF.11449.1; tss_id TSS10536;
2	Cufflinks	exon	134470129	134471300	.	+	.	gene_id GRMZM2G111143; transcript_id TCONS_00011675; exon_number 1; oId CUFF.11449.3; tss_id TSS10536;
2	Cufflinks	exon	134472183	134472331	.	+	.	gene_id GRMZM2G111143; transcript_id TCONS_00011675; exon_number 2; oId CUFF.11449.3; tss_id TSS10536;
2	Cufflinks	exon	134472432	134472840	.	+	.	gene_id GRMZM2G111143; transcript_id TCONS_00011675; exon_number 3; oId CUFF.11449.3; tss_id TSS10536;
2	Cufflinks	exon	134470130	134472331	.	+	.	gene_id GRMZM2G111143; transcript_id TCONS_00011676; exon_number 1; oId CUFF.11449.4; tss_id TSS10536;
2	Cufflinks	exon	134472432	134472840	.	+	.	gene_id GRMZM2G111143; transcript_id TCONS_00011676; exon_number 2; oId CUFF.11449.4; tss_id TSS10536;
2	Cufflinks	exon	140149834	140151278	.	+	.	gene_id GRMZM2G099502; transcript_id TCONS_00011706; exon_number 1; oId CUFF.11500.2; tss_id TSS10562;
2	Cufflinks	exon	140151400	140152470	.	+	.	gene_id GRMZM2G099502; transcript_id TCONS_00011706; exon_number 2; oId CUFF.11500.2; tss_id TSS10562;
2	Cufflinks	exon	140149834	140151278	.	+	.	gene_id GRMZM2G099502; transcript_id TCONS_00011705; exon_number 1; oId CUFF.11500.1; tss_id TSS10562;
2	Cufflinks	exon	140151415	140152470	.	+	.	gene_id GRMZM2G099502; transcript_id TCONS_00011705; exon_number 2; oId CUFF.11500.1; tss_id TSS10562;
2	Cufflinks	exon	142057153	142058876	.	+	.	gene_id GRMZM2G032478; transcript_id TCONS_00011718; exon_number 1; oId CUFF.11520.1; tss_id TSS10574;
2	Cufflinks	exon	143893289	143893355	.	+	.	gene_id GRMZM2G028179; transcript_id TCONS_00011727; exon_number 1; oId CUFF.11532.1; tss_id TSS10583;
2	Cufflinks	exon	143893475	143893965	.	+	.	gene_id GRMZM2G028179; transcript_id TCONS_00011727; exon_number 2; oId CUFF.11532.1; tss_id TSS10583;
2	Cufflinks	exon	143894041	143895477	.	+	.	gene_id GRMZM2G028179; transcript_id TCONS_00011727; exon_number 3; oId CUFF.11532.1; tss_id TSS10583;
2	Cufflinks	exon	144827260	144828166	.	+	.	gene_id GRMZM2G127418; transcript_id TCONS_00011741; exon_number 1; oId CUFF.11552.1; tss_id TSS10594;
2	Cufflinks	exon	144828263	144828751	.	+	.	gene_id GRMZM2G127418; transcript_id TCONS_00011741; exon_number 2; oId CUFF.11552.1; tss_id TSS10594;
2	Cufflinks	exon	169186883	169187672	.	+	.	gene_id GRMZM2G092125; transcript_id TCONS_00011937; exon_number 1; oId CUFF.11905.1; tss_id TSS10770;
2	Cufflinks	exon	169189618	169189758	.	+	.	gene_id GRMZM2G092125; transcript_id TCONS_00011937; exon_number 2; oId CUFF.11905.1; tss_id TSS10770;
2	Cufflinks	exon	169189856	169190374	.	+	.	gene_id GRMZM2G092125; transcript_id TCONS_00011937; exon_number 3; oId CUFF.11905.1; tss_id TSS10770;
2	Cufflinks	exon	172384572	172385019	.	+	.	gene_id GRMZM2G019119; transcript_id TCONS_00011979; exon_number 1; oId CUFF.11955.1; tss_id TSS10807;
2	Cufflinks	exon	172386879	172387831	.	+	.	gene_id GRMZM2G019119; transcript_id TCONS_00011979; exon_number 2; oId CUFF.11955.1; tss_id TSS10807;
2	Cufflinks	exon	176668559	176668745	.	+	.	gene_id GRMZM2G037131; transcript_id TCONS_00012024; exon_number 1; oId CUFF.12050.1; tss_id TSS10848;
2	Cufflinks	exon	176668863	176668927	.	+	.	gene_id GRMZM2G037131; transcript_id TCONS_00012024; exon_number 2; oId CUFF.12050.1; tss_id TSS10848;
2	Cufflinks	exon	176669049	176669692	.	+	.	gene_id GRMZM2G037131; transcript_id TCONS_00012024; exon_number 3; oId CUFF.12050.1; tss_id TSS10848;
2	Cufflinks	exon	177200642	177201067	.	+	.	gene_id GRMZM5G868354; transcript_id TCONS_00012031; exon_number 1; oId CUFF.12057.1; tss_id TSS10854;
2	Cufflinks	exon	177203249	177203329	.	+	.	gene_id GRMZM5G868354; transcript_id TCONS_00012031; exon_number 2; oId CUFF.12057.1; tss_id TSS10854;
2	Cufflinks	exon	177203670	177204531	.	+	.	gene_id GRMZM5G868354; transcript_id TCONS_00012031; exon_number 3; oId CUFF.12057.1; tss_id TSS10854;
2	Cufflinks	exon	177562284	177563575	.	+	.	gene_id GRMZM2G373554; transcript_id TCONS_00012040; exon_number 1; oId CUFF.12053.1; tss_id TSS10861;
2	Cufflinks	exon	178670829	178671877	.	+	.	gene_id XLOC_010530; transcript_id TCONS_00012051; exon_number 1; oId CUFF.12079.1; tss_id TSS10872;
2	Cufflinks	exon	180452226	180453300	.	+	.	gene_id GRMZM2G040840; transcript_id TCONS_00012067; exon_number 1; oId CUFF.12134.1; tss_id TSS10886;
2	Cufflinks	exon	180455485	180455900	.	+	.	gene_id GRMZM2G040840; transcript_id TCONS_00012067; exon_number 2; oId CUFF.12134.1; tss_id TSS10886;
2	Cufflinks	exon	180455989	180457230	.	+	.	gene_id GRMZM2G040840; transcript_id TCONS_00012067; exon_number 3; oId CUFF.12134.1; tss_id TSS10886;
2	Cufflinks	exon	183846887	183847834	.	+	.	gene_id GRMZM2G057852; transcript_id TCONS_00012095; exon_number 1; oId CUFF.12160.1; tss_id TSS10913;
2	Cufflinks	exon	187110003	187110972	.	+	.	gene_id GRMZM2G381071; transcript_id TCONS_00012136; exon_number 1; oId CUFF.12244.1; tss_id TSS10950;
2	Cufflinks	exon	190220678	190223077	.	+	.	gene_id GRMZM2G134334; transcript_id TCONS_00012195; exon_number 1; oId CUFF.12344.1; tss_id TSS11007;

2	Cufflinks	exon	194151567	194151726	.	+	.	gene_id GRMZM2G150503; transcript_id TCONS_00012248; exon_number 1; oId CUFF.12431.1; tss_id TSS11052;
2	Cufflinks	exon	194152578	194153605	.	+	.	gene_id GRMZM2G150503; transcript_id TCONS_00012248; exon_number 2; oId CUFF.12431.1; tss_id TSS11052;
2	Cufflinks	exon	194153690	194153971	.	+	.	gene_id GRMZM2G150503; transcript_id TCONS_00012248; exon_number 3; oId CUFF.12431.1; tss_id TSS11052;
2	Cufflinks	exon	194154096	194154187	.	+	.	gene_id GRMZM2G150503; transcript_id TCONS_00012248; exon_number 4; oId CUFF.12431.1; tss_id TSS11052;
2	Cufflinks	exon	194156057	194157270	.	+	.	gene_id GRMZM2G150503; transcript_id TCONS_00012248; exon_number 5; oId CUFF.12431.1; tss_id TSS11052;
2	Cufflinks	exon	194299099	194299374	.	+	.	gene_id XLOC_010707; transcript_id TCONS_00012252; exon_number 1; oId CUFF.12428.1; tss_id TSS11056;
2	Cufflinks	exon	194808387	194809019	.	+	.	gene_id GRMZM2G164956; transcript_id TCONS_00012261; exon_number 1; oId CUFF.12478.1; tss_id TSS11065;
2	Cufflinks	exon	194809461	194809595	.	+	.	gene_id GRMZM2G164956; transcript_id TCONS_00012261; exon_number 2; oId CUFF.12478.1; tss_id TSS11065;
2	Cufflinks	exon	194809689	194809829	.	+	.	gene_id GRMZM2G164956; transcript_id TCONS_00012261; exon_number 3; oId CUFF.12478.1; tss_id TSS11065;
2	Cufflinks	exon	194809920	194810031	.	+	.	gene_id GRMZM2G164956; transcript_id TCONS_00012261; exon_number 4; oId CUFF.12478.1; tss_id TSS11065;
2	Cufflinks	exon	194810259	194810329	.	+	.	gene_id GRMZM2G164956; transcript_id TCONS_00012261; exon_number 5; oId CUFF.12478.1; tss_id TSS11065;
2	Cufflinks	exon	194810414	194810568	.	+	.	gene_id GRMZM2G164956; transcript_id TCONS_00012261; exon_number 6; oId CUFF.12478.1; tss_id TSS11065;
2	Cufflinks	exon	194810652	194810763	.	+	.	gene_id GRMZM2G164956; transcript_id TCONS_00012261; exon_number 7; oId CUFF.12478.1; tss_id TSS11065;
2	Cufflinks	exon	194811077	194811178	.	+	.	gene_id GRMZM2G164956; transcript_id TCONS_00012261; exon_number 8; oId CUFF.12478.1; tss_id TSS11065;
2	Cufflinks	exon	194811253	194811313	.	+	.	gene_id GRMZM2G164956; transcript_id TCONS_00012261; exon_number 9; oId CUFF.12478.1; tss_id TSS11065;
2	Cufflinks	exon	194811395	194811566	.	+	.	gene_id GRMZM2G164956; transcript_id TCONS_00012261; exon_number 10; oId CUFF.12478.1; tss_id TSS11065;
2	Cufflinks	exon	194811657	194811753	.	+	.	gene_id GRMZM2G164956; transcript_id TCONS_00012261; exon_number 11; oId CUFF.12478.1; tss_id TSS11065;
2	Cufflinks	exon	194811842	194811936	.	+	.	gene_id GRMZM2G164956; transcript_id TCONS_00012261; exon_number 12; oId CUFF.12478.1; tss_id TSS11065;
2	Cufflinks	exon	194812019	194812152	.	+	.	gene_id GRMZM2G164956; transcript_id TCONS_00012261; exon_number 13; oId CUFF.12478.1; tss_id TSS11065;
2	Cufflinks	exon	194812352	194812823	.	+	.	gene_id GRMZM2G164956; transcript_id TCONS_00012261; exon_number 14; oId CUFF.12478.1; tss_id TSS11065;
2	Cufflinks	exon	196480477	196480984	.	+	.	gene_id XLOC_010731; transcript_id TCONS_00012279; exon_number 1; oId CUFF.12487.1; tss_id TSS11081;
2	Cufflinks	exon	208298516	208298767	.	+	.	gene_id GRMZM2G062156; transcript_id TCONS_00012509; exon_number 1; oId CUFF.12876.1; tss_id TSS11296;
2	Cufflinks	exon	208298878	208300768	.	+	.	gene_id GRMZM2G062156; transcript_id TCONS_00012509; exon_number 2; oId CUFF.12876.1; tss_id TSS11296;
2	Cufflinks	exon	209674829	209675032	.	+	.	gene_id GRMZM2G077845; transcript_id TCONS_00012535; exon_number 1; oId CUFF.12908.1; tss_id TSS11320;
2	Cufflinks	exon	209675129	209675515	.	+	.	gene_id GRMZM2G077845; transcript_id TCONS_00012535; exon_number 2; oId CUFF.12908.1; tss_id TSS11320;
2	Cufflinks	exon	215615958	215616844	.	+	.	gene_id GRMZM2G166971; transcript_id TCONS_00012677; exon_number 1; oId CUFF.13130.1; tss_id TSS11446;
2	Cufflinks	exon	215616943	215617442	.	+	.	gene_id GRMZM2G166971; transcript_id TCONS_00012677; exon_number 2; oId CUFF.13130.1; tss_id TSS11446;
2	Cufflinks	exon	215752666	215753667	.	+	.	gene_id GRMZM2G141432; transcript_id TCONS_00012682; exon_number 1; oId CUFF.13135.1; tss_id TSS11450;
2	Cufflinks	exon	217334825	217335194	.	+	.	gene_id GRMZM2G001247; transcript_id TCONS_00012717; exon_number 1; oId CUFF.13206.1; tss_id TSS11483;
2	Cufflinks	exon	217335284	217335843	.	+	.	gene_id GRMZM2G001247; transcript_id TCONS_00012717; exon_number 2; oId CUFF.13206.1; tss_id TSS11483;
2	Cufflinks	exon	219255789	219256085	.	+	.	gene_id GRMZM2G140908; transcript_id TCONS_00012752; exon_number 1; oId CUFF.13264.1; tss_id TSS11516;
2	Cufflinks	exon	219256563	219257474	.	+	.	gene_id GRMZM2G140908; transcript_id TCONS_00012752; exon_number 2; oId CUFF.13264.1; tss_id TSS11516;
2	Cufflinks	exon	219257562	219258018	.	+	.	gene_id GRMZM2G140908; transcript_id TCONS_00012752; exon_number 3; oId CUFF.13264.1; tss_id TSS11516;
2	Cufflinks	exon	219606880	219608943	.	+	.	gene_id GRMZM2G068220; transcript_id TCONS_00012754; exon_number 1; oId CUFF.13270.1; tss_id TSS11518;
2	Cufflinks	exon	220671360	220671898	.	+	.	gene_id GRMZM2G137546; transcript_id TCONS_00012768; exon_number 1; oId CUFF.13299.1; tss_id TSS11532;
2	Cufflinks	exon	220672098	220672972	.	+	.	gene_id GRMZM2G137546; transcript_id TCONS_00012768; exon_number 2; oId CUFF.13299.1; tss_id TSS11532;

2	Cufflinks	exon	221286810	221287227	.	+	.	gene_id GRMZM2G129268; transcript_id TCONS_00012779; exon_number 1; oId CUFF.13323.1; tss_id TSS11541;
2	Cufflinks	exon	221288114	221288170	.	+	.	gene_id GRMZM2G129268; transcript_id TCONS_00012779; exon_number 2; oId CUFF.13323.1; tss_id TSS11541;
2	Cufflinks	exon	221288819	221288909	.	+	.	gene_id GRMZM2G129268; transcript_id TCONS_00012779; exon_number 3; oId CUFF.13323.1; tss_id TSS11541;
2	Cufflinks	exon	221288996	221289156	.	+	.	gene_id GRMZM2G129268; transcript_id TCONS_00012779; exon_number 4; oId CUFF.13323.1; tss_id TSS11541;
2	Cufflinks	exon	221289302	221289429	.	+	.	gene_id GRMZM2G129268; transcript_id TCONS_00012779; exon_number 5; oId CUFF.13323.1; tss_id TSS11541;
2	Cufflinks	exon	221289538	221289675	.	+	.	gene_id GRMZM2G129268; transcript_id TCONS_00012779; exon_number 6; oId CUFF.13323.1; tss_id TSS11541;
2	Cufflinks	exon	221290320	221290412	.	+	.	gene_id GRMZM2G129268; transcript_id TCONS_00012779; exon_number 7; oId CUFF.13323.1; tss_id TSS11541;
2	Cufflinks	exon	221290817	221290922	.	+	.	gene_id GRMZM2G129268; transcript_id TCONS_00012779; exon_number 8; oId CUFF.13323.1; tss_id TSS11541;
2	Cufflinks	exon	221291001	221291728	.	+	.	gene_id GRMZM2G129268; transcript_id TCONS_00012779; exon_number 9; oId CUFF.13323.1; tss_id TSS11541;
2	Cufflinks	exon	224404157	224404657	.	+	.	gene_id XLOC_011235; transcript_id TCONS_00012843; exon_number 1; oId CUFF.13390.1; tss_id TSS11599;
2	Cufflinks	exon	224405361	224405815	.	+	.	gene_id XLOC_011235; transcript_id TCONS_00012843; exon_number 2; oId CUFF.13390.1; tss_id TSS11599;
2	Cufflinks	exon	225001746	225002012	.	+	.	gene_id GRMZM2G169095; transcript_id TCONS_00012854; exon_number 1; oId CUFF.13434.1; tss_id TSS11610;
2	Cufflinks	exon	225002148	225002211	.	+	.	gene_id GRMZM2G169095; transcript_id TCONS_00012854; exon_number 2; oId CUFF.13434.1; tss_id TSS11610;
2	Cufflinks	exon	225003836	225003975	.	+	.	gene_id GRMZM2G169095; transcript_id TCONS_00012854; exon_number 3; oId CUFF.13434.1; tss_id TSS11610;
2	Cufflinks	exon	225005358	225005642	.	+	.	gene_id GRMZM2G169095; transcript_id TCONS_00012854; exon_number 4; oId CUFF.13434.1; tss_id TSS11610;
2	Cufflinks	exon	225005759	225005881	.	+	.	gene_id GRMZM2G169095; transcript_id TCONS_00012854; exon_number 5; oId CUFF.13434.1; tss_id TSS11610;
2	Cufflinks	exon	225006001	225006246	.	+	.	gene_id GRMZM2G169095; transcript_id TCONS_00012854; exon_number 6; oId CUFF.13434.1; tss_id TSS11610;
2	Cufflinks	exon	225007337	225007423	.	+	.	gene_id GRMZM2G169095; transcript_id TCONS_00012854; exon_number 7; oId CUFF.13434.1; tss_id TSS11610;
2	Cufflinks	exon	225007657	225007724	.	+	.	gene_id GRMZM2G169095; transcript_id TCONS_00012854; exon_number 8; oId CUFF.13434.1; tss_id TSS11610;
2	Cufflinks	exon	225007940	225008003	.	+	.	gene_id GRMZM2G169095; transcript_id TCONS_00012854; exon_number 9; oId CUFF.13434.1; tss_id TSS11610;
2	Cufflinks	exon	225008405	225008539	.	+	.	gene_id GRMZM2G169095; transcript_id TCONS_00012854; exon_number 10; oId CUFF.13434.1; tss_id TSS11610;
2	Cufflinks	exon	225008618	225008698	.	+	.	gene_id GRMZM2G169095; transcript_id TCONS_00012854; exon_number 11; oId CUFF.13434.1; tss_id TSS11610;
2	Cufflinks	exon	225009103	225009157	.	+	.	gene_id GRMZM2G169095; transcript_id TCONS_00012854; exon_number 12; oId CUFF.13434.1; tss_id TSS11610;
2	Cufflinks	exon	225011234	225011338	.	+	.	gene_id GRMZM2G169095; transcript_id TCONS_00012854; exon_number 13; oId CUFF.13434.1; tss_id TSS11610;
2	Cufflinks	exon	225011432	225011503	.	+	.	gene_id GRMZM2G169095; transcript_id TCONS_00012854; exon_number 14; oId CUFF.13434.1; tss_id TSS11610;
2	Cufflinks	exon	225011639	225011763	.	+	.	gene_id GRMZM2G169095; transcript_id TCONS_00012854; exon_number 15; oId CUFF.13434.1; tss_id TSS11610;
2	Cufflinks	exon	225012610	225013108	.	+	.	gene_id GRMZM2G169095; transcript_id TCONS_00012854; exon_number 16; oId CUFF.13434.1; tss_id TSS11610;
2	Cufflinks	exon	231189722	231190531	.	+	.	gene_id GRMZM2G158872; transcript_id TCONS_00012950; exon_number 1; oId CUFF.13598.1; tss_id TSS11703;
2	Cufflinks	exon	231190760	231192194	.	+	.	gene_id GRMZM2G158872; transcript_id TCONS_00012950; exon_number 2; oId CUFF.13598.1; tss_id TSS11703;
2	Cufflinks	exon	231192280	231192415	.	+	.	gene_id GRMZM2G158872; transcript_id TCONS_00012950; exon_number 3; oId CUFF.13598.1; tss_id TSS11703;
2	Cufflinks	exon	231192548	231192645	.	+	.	gene_id GRMZM2G158872; transcript_id TCONS_00012950; exon_number 4; oId CUFF.13598.1; tss_id TSS11703;
2	Cufflinks	exon	231192825	231192890	.	+	.	gene_id GRMZM2G158872; transcript_id TCONS_00012950; exon_number 5; oId CUFF.13598.1; tss_id TSS11703;
2	Cufflinks	exon	231193330	231193419	.	+	.	gene_id GRMZM2G158872; transcript_id TCONS_00012950; exon_number 6; oId CUFF.13598.1; tss_id TSS11703;
2	Cufflinks	exon	231194208	231194384	.	+	.	gene_id GRMZM2G158872; transcript_id TCONS_00012950; exon_number 7; oId CUFF.13598.1; tss_id TSS11703;
2	Cufflinks	exon	231195198	231195711	.	+	.	gene_id GRMZM2G158872; transcript_id TCONS_00012950; exon_number 8; oId CUFF.13598.1; tss_id TSS11703;
2	Cufflinks	exon	234414167	234415092	.	+	.	gene_id GRMZM5G878490; transcript_id TCONS_00013017; exon_number 1; oId CUFF.13714.1; tss_id TSS11764;

2	Cufflinks	exon	529618	529968	.	-	.	gene_id XLOC_011457; transcript_id TCONS_00013077; exon_number 1; oId CUFF.9001.1; tss_id TSS11822;
2	Cufflinks	exon	2821204	2821873	.	-	.	gene_id GRMZM2G043932; transcript_id TCONS_00013142; exon_number 1; oId CUFF.9124.1; tss_id TSS11875;
2	Cufflinks	exon	2821963	2822038	.	-	.	gene_id GRMZM2G043932; transcript_id TCONS_00013142; exon_number 2; oId CUFF.9124.1; tss_id TSS11875;
2	Cufflinks	exon	2822138	2822336	.	-	.	gene_id GRMZM2G043932; transcript_id TCONS_00013142; exon_number 3; oId CUFF.9124.1; tss_id TSS11875;
2	Cufflinks	exon	3115670	3116145	.	-	.	gene_id GRMZM2G035325; transcript_id TCONS_00013151; exon_number 1; oId CUFF.9143.1; tss_id TSS11882;
2	Cufflinks	exon	3116352	3116488	.	-	.	gene_id GRMZM2G035325; transcript_id TCONS_00013151; exon_number 2; oId CUFF.9143.1; tss_id TSS11882;
2	Cufflinks	exon	3117127	3117311	.	-	.	gene_id GRMZM2G035325; transcript_id TCONS_00013151; exon_number 3; oId CUFF.9143.1; tss_id TSS11882;
2	Cufflinks	exon	3117415	3117583	.	-	.	gene_id GRMZM2G035325; transcript_id TCONS_00013151; exon_number 4; oId CUFF.9143.1; tss_id TSS11882;
2	Cufflinks	exon	3117685	3117790	.	-	.	gene_id GRMZM2G035325; transcript_id TCONS_00013151; exon_number 5; oId CUFF.9143.1; tss_id TSS11882;
2	Cufflinks	exon	3119073	3119831	.	-	.	gene_id GRMZM2G035325; transcript_id TCONS_00013151; exon_number 6; oId CUFF.9143.1; tss_id TSS11882;
2	Cufflinks	exon	3928213	3928488	.	-	.	gene_id GRMZM2G044011; transcript_id TCONS_00013170; exon_number 1; oId CUFF.9201.1; tss_id TSS11899;
2	Cufflinks	exon	3928879	3928985	.	-	.	gene_id GRMZM2G044011; transcript_id TCONS_00013170; exon_number 2; oId CUFF.9201.1; tss_id TSS11899;
2	Cufflinks	exon	3931316	3931410	.	-	.	gene_id GRMZM2G044011; transcript_id TCONS_00013170; exon_number 3; oId CUFF.9201.1; tss_id TSS11899;
2	Cufflinks	exon	3931623	3931661	.	-	.	gene_id GRMZM2G044011; transcript_id TCONS_00013170; exon_number 4; oId CUFF.9201.1; tss_id TSS11899;
2	Cufflinks	exon	3932157	3932229	.	-	.	gene_id GRMZM2G044011; transcript_id TCONS_00013170; exon_number 5; oId CUFF.9201.1; tss_id TSS11899;
2	Cufflinks	exon	3932545	3932651	.	-	.	gene_id GRMZM2G044011; transcript_id TCONS_00013170; exon_number 6; oId CUFF.9201.1; tss_id TSS11899;
2	Cufflinks	exon	3933131	3933199	.	-	.	gene_id GRMZM2G044011; transcript_id TCONS_00013170; exon_number 7; oId CUFF.9201.1; tss_id TSS11899;
2	Cufflinks	exon	3933281	3933313	.	-	.	gene_id GRMZM2G044011; transcript_id TCONS_00013170; exon_number 8; oId CUFF.9201.1; tss_id TSS11899;
2	Cufflinks	exon	3933400	3933475	.	-	.	gene_id GRMZM2G044011; transcript_id TCONS_00013170; exon_number 9; oId CUFF.9201.1; tss_id TSS11899;
2	Cufflinks	exon	3933931	3934006	.	-	.	gene_id GRMZM2G044011; transcript_id TCONS_00013170; exon_number 10; oId CUFF.9201.1; tss_id TSS11899;
2	Cufflinks	exon	3934293	3934338	.	-	.	gene_id GRMZM2G044011; transcript_id TCONS_00013170; exon_number 11; oId CUFF.9201.1; tss_id TSS11899;
2	Cufflinks	exon	3937140	3937196	.	-	.	gene_id GRMZM2G044011; transcript_id TCONS_00013170; exon_number 12; oId CUFF.9201.1; tss_id TSS11899;
2	Cufflinks	exon	3937277	3937351	.	-	.	gene_id GRMZM2G044011; transcript_id TCONS_00013170; exon_number 13; oId CUFF.9201.1; tss_id TSS11899;
2	Cufflinks	exon	3937715	3937790	.	-	.	gene_id GRMZM2G044011; transcript_id TCONS_00013170; exon_number 14; oId CUFF.9201.1; tss_id TSS11899;
2	Cufflinks	exon	3938318	3938400	.	-	.	gene_id GRMZM2G044011; transcript_id TCONS_00013170; exon_number 15; oId CUFF.9201.1; tss_id TSS11899;
2	Cufflinks	exon	3938565	3938819	.	-	.	gene_id GRMZM2G044011; transcript_id TCONS_00013170; exon_number 16; oId CUFF.9201.1; tss_id TSS11899;
2	Cufflinks	exon	14732505	14732883	.	-	.	gene_id XLOC_011760; transcript_id TCONS_00013431; exon_number 1; oId CUFF.9676.1; tss_id TSS12135;
2	Cufflinks	exon	18757659	18758028	.	-	.	gene_id GRMZM2G174807; transcript_id TCONS_00013503; exon_number 1; oId CUFF.9831.1; tss_id TSS12196;
2	Cufflinks	exon	18758377	18758517	.	-	.	gene_id GRMZM2G174807; transcript_id TCONS_00013503; exon_number 2; oId CUFF.9831.1; tss_id TSS12196;
2	Cufflinks	exon	18758613	18758908	.	-	.	gene_id GRMZM2G174807; transcript_id TCONS_00013503; exon_number 3; oId CUFF.9831.1; tss_id TSS12196;
2	Cufflinks	exon	18759085	18759526	.	-	.	gene_id GRMZM2G174807; transcript_id TCONS_00013503; exon_number 4; oId CUFF.9831.1; tss_id TSS12196;
2	Cufflinks	exon	25497178	25498828	.	-	.	gene_id GRMZM2G108123; transcript_id TCONS_00013618; exon_number 1; oId CUFF.9990.1; tss_id TSS12301;
2	Cufflinks	exon	27124232	27124805	.	-	.	gene_id GRMZM2G138190; transcript_id TCONS_00013639; exon_number 1; oId CUFF.10054.1; tss_id TSS12322;
2	Cufflinks	exon	27124885	27125205	.	-	.	gene_id GRMZM2G138190; transcript_id TCONS_00013639; exon_number 2; oId CUFF.10054.1; tss_id TSS12322;
2	Cufflinks	exon	27125297	27125695	.	-	.	gene_id GRMZM2G138190; transcript_id TCONS_00013639; exon_number 3; oId CUFF.10054.1; tss_id TSS12322;
2	Cufflinks	exon	28493228	28493625	.	-	.	gene_id GRMZM2G178693; transcript_id TCONS_00013660; exon_number 1; oId CUFF.10059.1; tss_id TSS12342;

2	Cufflinks	exon	28494571	28494711	.	-	.	gene_id GRMZM2G178693; transcript_id TCONS_00013660; exon_number 2; oId CUFF.10059.1; tss_id TSS12342;
2	Cufflinks	exon	28494793	28495467	.	-	.	gene_id GRMZM2G178693; transcript_id TCONS_00013660; exon_number 3; oId CUFF.10059.1; tss_id TSS12342;
2	Cufflinks	exon	28746597	28748681	.	-	.	gene_id XLOC_011959; transcript_id TCONS_00013667; exon_number 1; oId CUFF.10073.1; tss_id TSS12347;
2	Cufflinks	exon	32571806	32572565	.	-	.	gene_id GRMZM2G419342; transcript_id TCONS_00013731; exon_number 1; oId CUFF.10177.1; tss_id TSS12400;
2	Cufflinks	exon	32572724	32573152	.	-	.	gene_id GRMZM2G419342; transcript_id TCONS_00013731; exon_number 2; oId CUFF.10177.1; tss_id TSS12400;
2	Cufflinks	exon	32573251	32573931	.	-	.	gene_id GRMZM2G419342; transcript_id TCONS_00013731; exon_number 3; oId CUFF.10177.1; tss_id TSS12400;
2	Cufflinks	exon	33976171	33977109	.	-	.	gene_id GRMZM2G112530; transcript_id TCONS_00013745; exon_number 1; oId CUFF.10218.1; tss_id TSS12414;
2	Cufflinks	exon	33977806	33978101	.	-	.	gene_id GRMZM2G112530; transcript_id TCONS_00013745; exon_number 2; oId CUFF.10218.1; tss_id TSS12414;
2	Cufflinks	exon	33978213	33978612	.	-	.	gene_id GRMZM2G112530; transcript_id TCONS_00013745; exon_number 3; oId CUFF.10218.1; tss_id TSS12414;
2	Cufflinks	exon	33978778	33979292	.	-	.	gene_id GRMZM2G112530; transcript_id TCONS_00013745; exon_number 4; oId CUFF.10218.1; tss_id TSS12414;
2	Cufflinks	exon	34359171	34360419	.	-	.	gene_id GRMZM2G062673; transcript_id TCONS_00013756; exon_number 1; oId CUFF.10226.1; tss_id TSS12425;
2	Cufflinks	exon	39466052	39467802	.	-	.	gene_id XLOC_012103; transcript_id TCONS_00013834; exon_number 1; oId CUFF.10343.1; tss_id TSS12497;
2	Cufflinks	exon	40054195	40054536	.	-	.	gene_id GRMZM2G330430; transcript_id TCONS_00013837; exon_number 1; oId CUFF.10369.1; tss_id TSS12500;
2	Cufflinks	exon	40055194	40055262	.	-	.	gene_id GRMZM2G330430; transcript_id TCONS_00013837; exon_number 2; oId CUFF.10369.1; tss_id TSS12500;
2	Cufflinks	exon	40055345	40055549	.	-	.	gene_id GRMZM2G330430; transcript_id TCONS_00013837; exon_number 3; oId CUFF.10369.1; tss_id TSS12500;
2	Cufflinks	exon	40055659	40055741	.	-	.	gene_id GRMZM2G330430; transcript_id TCONS_00013837; exon_number 4; oId CUFF.10369.1; tss_id TSS12500;
2	Cufflinks	exon	40056335	40056474	.	-	.	gene_id GRMZM2G330430; transcript_id TCONS_00013837; exon_number 5; oId CUFF.10369.1; tss_id TSS12500;
2	Cufflinks	exon	40056642	40056907	.	-	.	gene_id GRMZM2G330430; transcript_id TCONS_00013837; exon_number 6; oId CUFF.10369.1; tss_id TSS12500;
2	Cufflinks	exon	41931997	41932382	.	-	.	gene_id GRMZM2G102499; transcript_id TCONS_00013856; exon_number 1; oId CUFF.10400.1; tss_id TSS12518;
2	Cufflinks	exon	41932489	41932605	.	-	.	gene_id GRMZM2G102499; transcript_id TCONS_00013856; exon_number 2; oId CUFF.10400.1; tss_id TSS12518;
2	Cufflinks	exon	41933996	41934118	.	-	.	gene_id GRMZM2G102499; transcript_id TCONS_00013856; exon_number 3; oId CUFF.10400.1; tss_id TSS12518;
2	Cufflinks	exon	41934245	41934323	.	-	.	gene_id GRMZM2G102499; transcript_id TCONS_00013856; exon_number 4; oId CUFF.10400.1; tss_id TSS12518;
2	Cufflinks	exon	41934411	41934855	.	-	.	gene_id GRMZM2G102499; transcript_id TCONS_00013856; exon_number 5; oId CUFF.10400.1; tss_id TSS12518;
2	Cufflinks	exon	41935488	41935666	.	-	.	gene_id GRMZM2G102499; transcript_id TCONS_00013856; exon_number 6; oId CUFF.10400.1; tss_id TSS12518;
2	Cufflinks	exon	42993870	42994657	.	-	.	gene_id GRMZM2G315401; transcript_id TCONS_00013869; exon_number 1; oId CUFF.10430.1; tss_id TSS12529;
2	Cufflinks	exon	42994753	42994860	.	-	.	gene_id GRMZM2G315401; transcript_id TCONS_00013869; exon_number 2; oId CUFF.10430.1; tss_id TSS12529;
2	Cufflinks	exon	42995017	42995140	.	-	.	gene_id GRMZM2G315401; transcript_id TCONS_00013869; exon_number 3; oId CUFF.10430.1; tss_id TSS12529;
2	Cufflinks	exon	42995829	42995878	.	-	.	gene_id GRMZM2G315401; transcript_id TCONS_00013869; exon_number 4; oId CUFF.10430.1; tss_id TSS12529;
2	Cufflinks	exon	42995979	42996151	.	-	.	gene_id GRMZM2G315401; transcript_id TCONS_00013869; exon_number 5; oId CUFF.10430.1; tss_id TSS12529;
2	Cufflinks	exon	42996258	42996389	.	-	.	gene_id GRMZM2G315401; transcript_id TCONS_00013869; exon_number 6; oId CUFF.10430.1; tss_id TSS12529;
2	Cufflinks	exon	44816328	44816744	.	-	.	gene_id GRMZM2G171254; transcript_id TCONS_00013892; exon_number 1; oId CUFF.10486.1; tss_id TSS12552;
2	Cufflinks	exon	44817003	44817196	.	-	.	gene_id GRMZM2G171254; transcript_id TCONS_00013892; exon_number 2; oId CUFF.10486.1; tss_id TSS12552;
2	Cufflinks	exon	44817317	44817384	.	-	.	gene_id GRMZM2G171254; transcript_id TCONS_00013892; exon_number 3; oId CUFF.10486.1; tss_id TSS12552;
2	Cufflinks	exon	44817474	44817560	.	-	.	gene_id GRMZM2G171254; transcript_id TCONS_00013892; exon_number 4; oId CUFF.10486.1; tss_id TSS12552;
2	Cufflinks	exon	44817707	44818022	.	-	.	gene_id GRMZM2G171254; transcript_id TCONS_00013892; exon_number 5; oId CUFF.10486.1; tss_id TSS12552;
2	Cufflinks	exon	44819482	44819607	.	-	.	gene_id GRMZM2G171254; transcript_id TCONS_00013892; exon_number 6; oId CUFF.10486.1; tss_id TSS12552;

2	Cufflinks	exon	44824024	44824217	.	-	.	gene_id GRMZM2G171254; transcript_id TCONS_00013892; exon_number 7; oId CUFF.10486.1; tss_id TSS12552;
2	Cufflinks	exon	44824315	44824385	.	-	.	gene_id GRMZM2G171254; transcript_id TCONS_00013892; exon_number 8; oId CUFF.10486.1; tss_id TSS12552;
2	Cufflinks	exon	44824471	44824545	.	-	.	gene_id GRMZM2G171254; transcript_id TCONS_00013892; exon_number 9; oId CUFF.10486.1; tss_id TSS12552;
2	Cufflinks	exon	44824680	44824949	.	-	.	gene_id GRMZM2G171254; transcript_id TCONS_00013892; exon_number 10; oId CUFF.10486.1; tss_id TSS12552;
2	Cufflinks	exon	45069171	45069681	.	-	.	gene_id GRMZM2G127139; transcript_id TCONS_00013906; exon_number 1; oId CUFF.10502.2; tss_id TSS12564;
2	Cufflinks	exon	45069825	45069912	.	-	.	gene_id GRMZM2G127139; transcript_id TCONS_00013906; exon_number 2; oId CUFF.10502.2; tss_id TSS12564;
2	Cufflinks	exon	45070002	45070090	.	-	.	gene_id GRMZM2G127139; transcript_id TCONS_00013906; exon_number 3; oId CUFF.10502.2; tss_id TSS12564;
2	Cufflinks	exon	45070208	45070262	.	-	.	gene_id GRMZM2G127139; transcript_id TCONS_00013906; exon_number 4; oId CUFF.10502.2; tss_id TSS12564;
2	Cufflinks	exon	45070348	45070437	.	-	.	gene_id GRMZM2G127139; transcript_id TCONS_00013906; exon_number 5; oId CUFF.10502.2; tss_id TSS12564;
2	Cufflinks	exon	45070548	45070612	.	-	.	gene_id GRMZM2G127139; transcript_id TCONS_00013906; exon_number 6; oId CUFF.10502.2; tss_id TSS12564;
2	Cufflinks	exon	45070690	45070735	.	-	.	gene_id GRMZM2G127139; transcript_id TCONS_00013906; exon_number 7; oId CUFF.10502.2; tss_id TSS12564;
2	Cufflinks	exon	45070912	45071015	.	-	.	gene_id GRMZM2G127139; transcript_id TCONS_00013906; exon_number 8; oId CUFF.10502.2; tss_id TSS12564;
2	Cufflinks	exon	45071248	45071362	.	-	.	gene_id GRMZM2G127139; transcript_id TCONS_00013906; exon_number 9; oId CUFF.10502.2; tss_id TSS12564;
2	Cufflinks	exon	45071571	45071662	.	-	.	gene_id GRMZM2G127139; transcript_id TCONS_00013906; exon_number 10; oId CUFF.10502.2; tss_id TSS12564;
2	Cufflinks	exon	45071751	45071962	.	-	.	gene_id GRMZM2G127139; transcript_id TCONS_00013906; exon_number 11; oId CUFF.10502.2; tss_id TSS12564;
2	Cufflinks	exon	45072092	45072207	.	-	.	gene_id GRMZM2G127139; transcript_id TCONS_00013906; exon_number 12; oId CUFF.10502.2; tss_id TSS12564;
2	Cufflinks	exon	45072304	45072404	.	-	.	gene_id GRMZM2G127139; transcript_id TCONS_00013906; exon_number 13; oId CUFF.10502.2; tss_id TSS12564;
2	Cufflinks	exon	45072484	45072558	.	-	.	gene_id GRMZM2G127139; transcript_id TCONS_00013906; exon_number 14; oId CUFF.10502.2; tss_id TSS12564;
2	Cufflinks	exon	45072662	45072824	.	-	.	gene_id GRMZM2G127139; transcript_id TCONS_00013906; exon_number 15; oId CUFF.10502.2; tss_id TSS12564;
2	Cufflinks	exon	45073279	45074209	.	-	.	gene_id GRMZM2G127139; transcript_id TCONS_00013906; exon_number 16; oId CUFF.10502.2; tss_id TSS12564;
2	Cufflinks	exon	48776032	48776812	.	-	.	gene_id XLOC_012207; transcript_id TCONS_00013950; exon_number 1; oId CUFF.10559.1; tss_id TSS12604;
2	Cufflinks	exon	49031132	49031934	.	-	.	gene_id GRMZM2G047681; transcript_id TCONS_00013951; exon_number 1; oId CUFF.10576.1; tss_id TSS12605;
2	Cufflinks	exon	49032027	49032520	.	-	.	gene_id GRMZM2G047681; transcript_id TCONS_00013951; exon_number 2; oId CUFF.10576.1; tss_id TSS12605;
2	Cufflinks	exon	49032635	49034213	.	-	.	gene_id GRMZM2G047681; transcript_id TCONS_00013951; exon_number 3; oId CUFF.10576.1; tss_id TSS12605;
2	Cufflinks	exon	49034319	49034425	.	-	.	gene_id GRMZM2G047681; transcript_id TCONS_00013951; exon_number 4; oId CUFF.10576.1; tss_id TSS12605;
2	Cufflinks	exon	49034518	49035085	.	-	.	gene_id GRMZM2G047681; transcript_id TCONS_00013951; exon_number 5; oId CUFF.10576.1; tss_id TSS12605;
2	Cufflinks	exon	49036037	49036412	.	-	.	gene_id GRMZM2G047681; transcript_id TCONS_00013951; exon_number 6; oId CUFF.10576.1; tss_id TSS12605;
2	Cufflinks	exon	56456421	56457302	.	-	.	gene_id GRMZM2G048793; transcript_id TCONS_00014034; exon_number 1; oId CUFF.10703.1; tss_id TSS12679;
2	Cufflinks	exon	56457410	56457513	.	-	.	gene_id GRMZM2G048793; transcript_id TCONS_00014034; exon_number 2; oId CUFF.10703.1; tss_id TSS12679;
2	Cufflinks	exon	62121451	62122022	.	-	.	gene_id GRMZM2G134668; transcript_id TCONS_00014076; exon_number 1; oId CUFF.10783.1; tss_id TSS12719;
2	Cufflinks	exon	62122113	62122229	.	-	.	gene_id GRMZM2G134668; transcript_id TCONS_00014076; exon_number 2; oId CUFF.10783.1; tss_id TSS12719;
2	Cufflinks	exon	62122324	62122395	.	-	.	gene_id GRMZM2G134668; transcript_id TCONS_00014076; exon_number 3; oId CUFF.10783.1; tss_id TSS12719;
2	Cufflinks	exon	62122495	62123624	.	-	.	gene_id GRMZM2G134668; transcript_id TCONS_00014076; exon_number 4; oId CUFF.10783.1; tss_id TSS12719;
2	Cufflinks	exon	62124703	62124772	.	-	.	gene_id GRMZM2G134668; transcript_id TCONS_00014076; exon_number 5; oId CUFF.10783.1; tss_id TSS12719;
2	Cufflinks	exon	62124877	62125108	.	-	.	gene_id GRMZM2G134668; transcript_id TCONS_00014076; exon_number 6; oId CUFF.10783.1; tss_id TSS12719;
2	Cufflinks	exon	64002611	64003234	.	-	.	gene_id XLOC_012328; transcript_id TCONS_00014087; exon_number 1; oId CUFF.10796.1; tss_id TSS12730;

2	Cufflinks	exon	64298381	64299759	.	-	.	gene_id GRMZM2G136412; transcript_id TCONS_00014089; exon_number 1; oId CUFF.10823.1; tss_id TSS12732;
2	Cufflinks	exon	64300650	64301370	.	-	.	gene_id GRMZM2G136412; transcript_id TCONS_00014089; exon_number 2; oId CUFF.10823.1; tss_id TSS12732;
2	Cufflinks	exon	69807585	69808567	.	-	.	gene_id GRMZM2G155281; transcript_id TCONS_00014121; exon_number 1; oId CUFF.10860.1; tss_id TSS12759;
2	Cufflinks	exon	69808974	69809061	.	-	.	gene_id GRMZM2G155281; transcript_id TCONS_00014121; exon_number 2; oId CUFF.10860.1; tss_id TSS12759;
2	Cufflinks	exon	69809176	69809387	.	-	.	gene_id GRMZM2G155281; transcript_id TCONS_00014121; exon_number 3; oId CUFF.10860.1; tss_id TSS12759;
2	Cufflinks	exon	69809990	69810251	.	-	.	gene_id GRMZM2G155281; transcript_id TCONS_00014121; exon_number 4; oId CUFF.10860.1; tss_id TSS12759;
2	Cufflinks	exon	69810343	69810746	.	-	.	gene_id GRMZM2G155281; transcript_id TCONS_00014121; exon_number 5; oId CUFF.10860.1; tss_id TSS12759;
2	Cufflinks	exon	71189907	71190586	.	-	.	gene_id XLOC_012369; transcript_id TCONS_00014136; exon_number 1; oId CUFF.10869.1; tss_id TSS12771;
2	Cufflinks	exon	127723784	127724444	.	-	.	gene_id GRMZM2G085924; transcript_id TCONS_00014425; exon_number 1; oId CUFF.11375.1; tss_id TSS13043;
2	Cufflinks	exon	127724524	127725392	.	-	.	gene_id GRMZM2G085924; transcript_id TCONS_00014425; exon_number 2; oId CUFF.11375.1; tss_id TSS13043;
2	Cufflinks	exon	128454357	128454710	.	-	.	gene_id XLOC_012641; transcript_id TCONS_00014427; exon_number 1; oId CUFF.11395.1; tss_id TSS13045;
2	Cufflinks	exon	128454809	128457728	.	-	.	gene_id XLOC_012641; transcript_id TCONS_00014427; exon_number 2; oId CUFF.11395.1; tss_id TSS13045;
2	Cufflinks	exon	134469981	134471436	.	-	.	gene_id XLOC_012675; transcript_id TCONS_00014464; exon_number 1; oId CUFF.11448.1; tss_id TSS13079;
2	Cufflinks	exon	143679351	143679969	.	-	.	gene_id GRMZM2G348866; transcript_id TCONS_00014511; exon_number 1; oId CUFF.11548.1; tss_id TSS13123;
2	Cufflinks	exon	143680060	143680515	.	-	.	gene_id GRMZM2G348866; transcript_id TCONS_00014511; exon_number 2; oId CUFF.11548.1; tss_id TSS13123;
2	Cufflinks	exon	143680738	143680861	.	-	.	gene_id GRMZM2G348866; transcript_id TCONS_00014511; exon_number 3; oId CUFF.11548.1; tss_id TSS13123;
2	Cufflinks	exon	143680961	143681017	.	-	.	gene_id GRMZM2G348866; transcript_id TCONS_00014511; exon_number 4; oId CUFF.11548.1; tss_id TSS13123;
2	Cufflinks	exon	143682675	143682819	.	-	.	gene_id GRMZM2G348866; transcript_id TCONS_00014511; exon_number 5; oId CUFF.11548.1; tss_id TSS13123;
2	Cufflinks	exon	143683804	143683853	.	-	.	gene_id GRMZM2G348866; transcript_id TCONS_00014511; exon_number 6; oId CUFF.11548.1; tss_id TSS13123;
2	Cufflinks	exon	143683931	143683971	.	-	.	gene_id GRMZM2G348866; transcript_id TCONS_00014511; exon_number 7; oId CUFF.11548.1; tss_id TSS13123;
2	Cufflinks	exon	143684077	143684440	.	-	.	gene_id GRMZM2G348866; transcript_id TCONS_00014511; exon_number 8; oId CUFF.11548.1; tss_id TSS13123;
2	Cufflinks	exon	143894058	143895355	.	-	.	gene_id XLOC_012718; transcript_id TCONS_00014512; exon_number 1; oId CUFF.11533.1; tss_id TSS13124;
2	Cufflinks	exon	145653249	145653665	.	-	.	gene_id GRMZM2G132000; transcript_id TCONS_00014531; exon_number 1; oId CUFF.11584.2; tss_id TSS13140;
2	Cufflinks	exon	145656332	145656700	.	-	.	gene_id GRMZM2G132000; transcript_id TCONS_00014531; exon_number 2; oId CUFF.11584.2; tss_id TSS13140;
2	Cufflinks	exon	145653249	145653665	.	-	.	gene_id GRMZM2G132000; transcript_id TCONS_00014532; exon_number 1; oId CUFF.11584.1; tss_id TSS13140;
2	Cufflinks	exon	145656337	145656755	.	-	.	gene_id GRMZM2G132000; transcript_id TCONS_00014532; exon_number 2; oId CUFF.11584.1; tss_id TSS13140;
2	Cufflinks	exon	151389889	151390399	.	-	.	gene_id GRMZM2G460138; transcript_id TCONS_00014558; exon_number 1; oId CUFF.11627.1; tss_id TSS13166;
2	Cufflinks	exon	151390519	151390712	.	-	.	gene_id GRMZM2G460138; transcript_id TCONS_00014558; exon_number 2; oId CUFF.11627.1; tss_id TSS13166;
2	Cufflinks	exon	167840064	167840914	.	-	.	gene_id GRMZM2G045706; transcript_id TCONS_00014698; exon_number 1; oId CUFF.11863.1; tss_id TSS13298;
2	Cufflinks	exon	167841090	167841733	.	-	.	gene_id GRMZM2G045706; transcript_id TCONS_00014698; exon_number 2; oId CUFF.11863.1; tss_id TSS13298;
2	Cufflinks	exon	171021988	171022635	.	-	.	gene_id GRMZM2G092648; transcript_id TCONS_00014725; exon_number 1; oId CUFF.11941.2; tss_id TSS13325;
2	Cufflinks	exon	171022757	171022848	.	-	.	gene_id GRMZM2G092648; transcript_id TCONS_00014725; exon_number 2; oId CUFF.11941.2; tss_id TSS13325;
2	Cufflinks	exon	171023342	171023427	.	-	.	gene_id GRMZM2G092648; transcript_id TCONS_00014725; exon_number 3; oId CUFF.11941.2; tss_id TSS13325;
2	Cufflinks	exon	171023502	171023679	.	-	.	gene_id GRMZM2G092648; transcript_id TCONS_00014725; exon_number 4; oId CUFF.11941.2; tss_id TSS13325;
2	Cufflinks	exon	171025405	171025619	.	-	.	gene_id GRMZM2G092648; transcript_id TCONS_00014725; exon_number 5; oId CUFF.11941.2; tss_id TSS13325;
2	Cufflinks	exon	171026398	171027063	.	-	.	gene_id GRMZM2G092648; transcript_id TCONS_00014725; exon_number 6; oId CUFF.11941.2; tss_id TSS13325;

2	Cufflinks	exon	171021988	171022635	.	-	.	gene_id GRMZM2G092648; transcript_id TCONS_00014726; exon_number 1; oId CUFF.11941.1; tss_id TSS13325;
2	Cufflinks	exon	171022757	171022848	.	-	.	gene_id GRMZM2G092648; transcript_id TCONS_00014726; exon_number 2; oId CUFF.11941.1; tss_id TSS13325;
2	Cufflinks	exon	171023342	171023427	.	-	.	gene_id GRMZM2G092648; transcript_id TCONS_00014726; exon_number 3; oId CUFF.11941.1; tss_id TSS13325;
2	Cufflinks	exon	171023502	171023724	.	-	.	gene_id GRMZM2G092648; transcript_id TCONS_00014726; exon_number 4; oId CUFF.11941.1; tss_id TSS13325;
2	Cufflinks	exon	171025405	171025619	.	-	.	gene_id GRMZM2G092648; transcript_id TCONS_00014726; exon_number 5; oId CUFF.11941.1; tss_id TSS13325;
2	Cufflinks	exon	171026398	171027132	.	-	.	gene_id GRMZM2G092648; transcript_id TCONS_00014726; exon_number 6; oId CUFF.11941.1; tss_id TSS13325;
2	Cufflinks	exon	175112138	175112837	.	-	.	gene_id AC203957.3_FG004; transcript_id TCONS_00014758; exon_number 1; oId CUFF.11994.1; tss_id TSS13356;
2	Cufflinks	exon	175112968	175113156	.	-	.	gene_id AC203957.3_FG004; transcript_id TCONS_00014758; exon_number 2; oId CUFF.11994.1; tss_id TSS13356;
2	Cufflinks	exon	177200717	177201068	.	-	.	gene_id XLOC_012973; transcript_id TCONS_00014788; exon_number 1; oId CUFF.12058.1; tss_id TSS13384;
2	Cufflinks	exon	179011169	179012353	.	-	.	gene_id GRMZM2G125775; transcript_id TCONS_00014814; exon_number 1; oId CUFF.12094.1; tss_id TSS13406;
2	Cufflinks	exon	180452055	180453525	.	-	.	gene_id XLOC_013013; transcript_id TCONS_00014834; exon_number 1; oId CUFF.12133.1; tss_id TSS13424;
2	Cufflinks	exon	183946385	183946791	.	-	.	gene_id XLOC_013033; transcript_id TCONS_00014854; exon_number 1; oId CUFF.12165.1; tss_id TSS13444;
2	Cufflinks	exon	189550226	189551687	.	-	.	gene_id GRMZM2G018070; transcript_id TCONS_00014937; exon_number 1; oId CUFF.12314.1; tss_id TSS13521;
2	Cufflinks	exon	190587060	190587720	.	-	.	gene_id GRMZM2G003947; transcript_id TCONS_00014955; exon_number 1; oId CUFF.12353.1; tss_id TSS13539;
2	Cufflinks	exon	190588081	190589608	.	-	.	gene_id GRMZM2G003947; transcript_id TCONS_00014955; exon_number 2; oId CUFF.12353.1; tss_id TSS13539;
2	Cufflinks	exon	196480040	196480887	.	-	.	gene_id GRMZM2G063533; transcript_id TCONS_00015032; exon_number 1; oId CUFF.12486.1; tss_id TSS13605;
2	Cufflinks	exon	196481292	196481414	.	-	.	gene_id GRMZM2G063533; transcript_id TCONS_00015032; exon_number 2; oId CUFF.12486.1; tss_id TSS13605;
2	Cufflinks	exon	196481922	196482182	.	-	.	gene_id GRMZM2G063533; transcript_id TCONS_00015032; exon_number 3; oId CUFF.12486.1; tss_id TSS13605;
2	Cufflinks	exon	196484096	196484796	.	-	.	gene_id GRMZM2G063533; transcript_id TCONS_00015032; exon_number 4; oId CUFF.12486.1; tss_id TSS13605;
2	Cufflinks	exon	196564900	196565266	.	-	.	gene_id XLOC_013192; transcript_id TCONS_00015033; exon_number 1; oId CUFF.12491.1; tss_id TSS13606;
2	Cufflinks	exon	196730795	196731019	.	-	.	gene_id GRMZM2G050714; transcript_id TCONS_00015038; exon_number 1; oId CUFF.12557.2; tss_id TSS13609;
2	Cufflinks	exon	196731120	196731194	.	-	.	gene_id GRMZM2G050714; transcript_id TCONS_00015038; exon_number 2; oId CUFF.12557.2; tss_id TSS13609;
2	Cufflinks	exon	196731286	196731351	.	-	.	gene_id GRMZM2G050714; transcript_id TCONS_00015038; exon_number 3; oId CUFF.12557.2; tss_id TSS13609;
2	Cufflinks	exon	196731709	196731762	.	-	.	gene_id GRMZM2G050714; transcript_id TCONS_00015038; exon_number 4; oId CUFF.12557.2; tss_id TSS13609;
2	Cufflinks	exon	196731848	196731979	.	-	.	gene_id GRMZM2G050714; transcript_id TCONS_00015038; exon_number 5; oId CUFF.12557.2; tss_id TSS13609;
2	Cufflinks	exon	196732079	196732140	.	-	.	gene_id GRMZM2G050714; transcript_id TCONS_00015038; exon_number 6; oId CUFF.12557.2; tss_id TSS13609;
2	Cufflinks	exon	196732247	196732323	.	-	.	gene_id GRMZM2G050714; transcript_id TCONS_00015038; exon_number 7; oId CUFF.12557.2; tss_id TSS13609;
2	Cufflinks	exon	196732426	196732498	.	-	.	gene_id GRMZM2G050714; transcript_id TCONS_00015038; exon_number 8; oId CUFF.12557.2; tss_id TSS13609;
2	Cufflinks	exon	196733336	196733448	.	-	.	gene_id GRMZM2G050714; transcript_id TCONS_00015038; exon_number 9; oId CUFF.12557.2; tss_id TSS13609;
2	Cufflinks	exon	196733542	196733606	.	-	.	gene_id GRMZM2G050714; transcript_id TCONS_00015038; exon_number 10; oId CUFF.12557.2; tss_id TSS13609;
2	Cufflinks	exon	196733714	196733779	.	-	.	gene_id GRMZM2G050714; transcript_id TCONS_00015038; exon_number 11; oId CUFF.12557.2; tss_id TSS13609;
2	Cufflinks	exon	196734288	196734631	.	-	.	gene_id GRMZM2G050714; transcript_id TCONS_00015038; exon_number 12; oId CUFF.12557.2; tss_id TSS13609;
2	Cufflinks	exon	199378450	199379219	.	-	.	gene_id GRMZM2G004619; transcript_id TCONS_00015092; exon_number 1; oId CUFF.12591.1; tss_id TSS13652;
2	Cufflinks	exon	199379477	199379701	.	-	.	gene_id GRMZM2G004619; transcript_id TCONS_00015092; exon_number 2; oId CUFF.12591.1; tss_id TSS13652;
2	Cufflinks	exon	199380358	199380793	.	-	.	gene_id GRMZM2G004619; transcript_id TCONS_00015092; exon_number 3; oId CUFF.12591.1; tss_id TSS13652;
2	Cufflinks	exon	210207281	210208102	.	-	.	gene_id GRMZM2G102138; transcript_id TCONS_00015275; exon_number 1; oId CUFF.12927.1; tss_id TSS13815;

2	Cufflinks	exon	214245816	214246895	.	-	.	gene_id GRMZM2G123212; transcript_id TCONS_00015349; exon_number 1; oId CUFF.13074.1; tss_id TSS13884;
2	Cufflinks	exon	214616867	214617548	.	-	.	gene_id GRMZM2G060160; transcript_id TCONS_00015359; exon_number 1; oId CUFF.13103.1; tss_id TSS13894;
2	Cufflinks	exon	214617638	214617887	.	-	.	gene_id GRMZM2G060160; transcript_id TCONS_00015359; exon_number 2; oId CUFF.13103.1; tss_id TSS13894;
2	Cufflinks	exon	214618597	214618797	.	-	.	gene_id GRMZM2G060160; transcript_id TCONS_00015359; exon_number 3; oId CUFF.13103.1; tss_id TSS13894;
2	Cufflinks	exon	214619142	214619312	.	-	.	gene_id GRMZM2G060160; transcript_id TCONS_00015359; exon_number 4; oId CUFF.13103.1; tss_id TSS13894;
2	Cufflinks	exon	214619409	214619593	.	-	.	gene_id GRMZM2G060160; transcript_id TCONS_00015359; exon_number 5; oId CUFF.13103.1; tss_id TSS13894;
2	Cufflinks	exon	214619691	214620016	.	-	.	gene_id GRMZM2G060160; transcript_id TCONS_00015359; exon_number 6; oId CUFF.13103.1; tss_id TSS13894;
2	Cufflinks	exon	214620773	214622270	.	-	.	gene_id GRMZM2G060160; transcript_id TCONS_00015359; exon_number 7; oId CUFF.13103.1; tss_id TSS13894;
2	Cufflinks	exon	215333156	215333756	.	-	.	gene_id GRMZM2G327686; transcript_id TCONS_00015371; exon_number 1; oId CUFF.13155.1; tss_id TSS13905;
2	Cufflinks	exon	215333913	215334055	.	-	.	gene_id GRMZM2G327686; transcript_id TCONS_00015371; exon_number 2; oId CUFF.13155.1; tss_id TSS13905;
2	Cufflinks	exon	215334180	215334279	.	-	.	gene_id GRMZM2G327686; transcript_id TCONS_00015371; exon_number 3; oId CUFF.13155.1; tss_id TSS13905;
2	Cufflinks	exon	215334374	215334451	.	-	.	gene_id GRMZM2G327686; transcript_id TCONS_00015371; exon_number 4; oId CUFF.13155.1; tss_id TSS13905;
2	Cufflinks	exon	215334549	215334980	.	-	.	gene_id GRMZM2G327686; transcript_id TCONS_00015371; exon_number 5; oId CUFF.13155.1; tss_id TSS13905;
2	Cufflinks	exon	217520133	217522001	.	-	.	gene_id XLOC_013523; transcript_id TCONS_00015420; exon_number 1; oId CUFF.13214.1; tss_id TSS13950;
2	Cufflinks	exon	220397639	220398088	.	-	.	gene_id XLOC_013559; transcript_id TCONS_00015458; exon_number 1; oId CUFF.13297.1; tss_id TSS13986;
2	Cufflinks	exon	220398129	220399809	.	-	.	gene_id XLOC_013559; transcript_id TCONS_00015458; exon_number 2; oId CUFF.13297.1; tss_id TSS13986;
2	Cufflinks	exon	221338800	221339150	.	-	.	gene_id GRMZM2G171818; transcript_id TCONS_00015470; exon_number 1; oId CUFF.13318.1; tss_id TSS13997;
2	Cufflinks	exon	221339231	221339494	.	-	.	gene_id GRMZM2G171818; transcript_id TCONS_00015470; exon_number 2; oId CUFF.13318.1; tss_id TSS13997;
2	Cufflinks	exon	221339635	221339703	.	-	.	gene_id GRMZM2G171818; transcript_id TCONS_00015470; exon_number 3; oId CUFF.13318.1; tss_id TSS13997;
2	Cufflinks	exon	221339791	221339856	.	-	.	gene_id GRMZM2G171818; transcript_id TCONS_00015470; exon_number 4; oId CUFF.13318.1; tss_id TSS13997;
2	Cufflinks	exon	221339968	221340165	.	-	.	gene_id GRMZM2G171818; transcript_id TCONS_00015470; exon_number 5; oId CUFF.13318.1; tss_id TSS13997;
2	Cufflinks	exon	221340252	221341247	.	-	.	gene_id GRMZM2G171818; transcript_id TCONS_00015470; exon_number 6; oId CUFF.13318.1; tss_id TSS13997;
2	Cufflinks	exon	233507197	233507949	.	-	.	gene_id GRMZM2G395128; transcript_id TCONS_00015642; exon_number 1; oId CUFF.13659.1; tss_id TSS14154;
2	Cufflinks	exon	233508077	233508793	.	-	.	gene_id GRMZM2G395128; transcript_id TCONS_00015642; exon_number 2; oId CUFF.13659.1; tss_id TSS14154;
2	Cufflinks	exon	233546257	233548974	.	-	.	gene_id GRMZM2G135013; transcript_id TCONS_00015643; exon_number 1; oId CUFF.13665.1; tss_id TSS14155;
2	Cufflinks	exon	233643319	233643875	.	-	.	gene_id GRMZM2G082853; transcript_id TCONS_00015648; exon_number 1; oId CUFF.13683.1; tss_id TSS14160;
2	Cufflinks	exon	233643965	233644595	.	-	.	gene_id GRMZM2G082853; transcript_id TCONS_00015648; exon_number 2; oId CUFF.13683.1; tss_id TSS14160;
2	Cufflinks	exon	233644690	233645423	.	-	.	gene_id GRMZM2G082853; transcript_id TCONS_00015648; exon_number 3; oId CUFF.13683.1; tss_id TSS14160;
2	Cufflinks	exon	233645620	233646053	.	-	.	gene_id GRMZM2G082853; transcript_id TCONS_00015648; exon_number 4; oId CUFF.13683.1; tss_id TSS14160;
3	Cufflinks	exon	1672575	1672770	.	+	.	gene_id GRMZM2G011636; transcript_id TCONS_00015749; exon_number 1; oId CUFF.13870.1; tss_id TSS14256;
3	Cufflinks	exon	1673376	1673614	.	+	.	gene_id GRMZM2G011636; transcript_id TCONS_00015749; exon_number 2; oId CUFF.13870.1; tss_id TSS14256;
3	Cufflinks	exon	1673988	1674148	.	+	.	gene_id GRMZM2G011636; transcript_id TCONS_00015749; exon_number 3; oId CUFF.13870.1; tss_id TSS14256;
3	Cufflinks	exon	1674242	1674388	.	+	.	gene_id GRMZM2G011636; transcript_id TCONS_00015749; exon_number 4; oId CUFF.13870.1; tss_id TSS14256;
3	Cufflinks	exon	1676015	1676091	.	+	.	gene_id GRMZM2G011636; transcript_id TCONS_00015749; exon_number 5; oId CUFF.13870.1; tss_id TSS14256;
3	Cufflinks	exon	1676491	1677158	.	+	.	gene_id GRMZM2G011636; transcript_id TCONS_00015749; exon_number 6; oId CUFF.13870.1; tss_id TSS14256;
3	Cufflinks	exon	2001563	2001931	.	+	.	gene_id GRMZM2G099678; transcript_id TCONS_00015764; exon_number 1; oId CUFF.13877.1; tss_id TSS14268;

3	Cufflinks	exon	2002839	2002906	.	+	.	gene_id GRMZM2G099678; transcript_id TCONS_00015764; exon_number 2; oId CUFF.13877.1; tss_id TSS14268;
3	Cufflinks	exon	2003013	2003991	.	+	.	gene_id GRMZM2G099678; transcript_id TCONS_00015764; exon_number 3; oId CUFF.13877.1; tss_id TSS14268;
3	Cufflinks	exon	3830012	3832754	.	+	.	gene_id AC233853.1_FG003; transcript_id TCONS_00015804; exon_number 1; oId CUFF.13959.1; tss_id TSS14303;
3	Cufflinks	exon	4674607	4675289	.	+	.	gene_id GRMZM2G055089; transcript_id TCONS_00015819; exon_number 1; oId CUFF.13992.1; tss_id TSS14318;
3	Cufflinks	exon	4676448	4676732	.	+	.	gene_id GRMZM2G055089; transcript_id TCONS_00015819; exon_number 2; oId CUFF.13992.1; tss_id TSS14318;
3	Cufflinks	exon	4677376	4677693	.	+	.	gene_id GRMZM2G055089; transcript_id TCONS_00015819; exon_number 3; oId CUFF.13992.1; tss_id TSS14318;
3	Cufflinks	exon	4677801	4678028	.	+	.	gene_id GRMZM2G055089; transcript_id TCONS_00015819; exon_number 4; oId CUFF.13992.1; tss_id TSS14318;
3	Cufflinks	exon	4678414	4678514	.	+	.	gene_id GRMZM2G055089; transcript_id TCONS_00015819; exon_number 5; oId CUFF.13992.1; tss_id TSS14318;
3	Cufflinks	exon	4679177	4679288	.	+	.	gene_id GRMZM2G055089; transcript_id TCONS_00015819; exon_number 6; oId CUFF.13992.1; tss_id TSS14318;
3	Cufflinks	exon	4679425	4679862	.	+	.	gene_id GRMZM2G055089; transcript_id TCONS_00015819; exon_number 7; oId CUFF.13992.1; tss_id TSS14318;
3	Cufflinks	exon	4680129	4680711	.	+	.	gene_id GRMZM2G055089; transcript_id TCONS_00015819; exon_number 8; oId CUFF.13992.1; tss_id TSS14318;
3	Cufflinks	exon	4707293	4709130	.	+	.	gene_id GRMZM2G055313; transcript_id TCONS_00015820; exon_number 1; oId CUFF.13996.1; tss_id TSS14319;
3	Cufflinks	exon	4709348	4709423	.	+	.	gene_id GRMZM2G055313; transcript_id TCONS_00015820; exon_number 2; oId CUFF.13996.1; tss_id TSS14319;
3	Cufflinks	exon	4709532	4710578	.	+	.	gene_id GRMZM2G055313; transcript_id TCONS_00015820; exon_number 3; oId CUFF.13996.1; tss_id TSS14319;
3	Cufflinks	exon	9045046	9046827	.	+	.	gene_id GRMZM2G158194; transcript_id TCONS_00015897; exon_number 1; oId CUFF.14127.1; tss_id TSS14383;
3	Cufflinks	exon	9047165	9047445	.	+	.	gene_id GRMZM2G158194; transcript_id TCONS_00015897; exon_number 2; oId CUFF.14127.1; tss_id TSS14383;
3	Cufflinks	exon	39477546	39478771	.	+	.	gene_id GRMZM2G132465; transcript_id TCONS_00016255; exon_number 1; oId CUFF.14742.1; tss_id TSS14707;
3	Cufflinks	exon	39478891	39479022	.	+	.	gene_id GRMZM2G132465; transcript_id TCONS_00016255; exon_number 2; oId CUFF.14742.1; tss_id TSS14707;
3	Cufflinks	exon	39480017	39482124	.	+	.	gene_id GRMZM2G132465; transcript_id TCONS_00016255; exon_number 3; oId CUFF.14742.1; tss_id TSS14707;
3	Cufflinks	exon	59327568	59327871	.	+	.	gene_id GRMZM2G107228; transcript_id TCONS_00016401; exon_number 1; oId CUFF.14985.1; tss_id TSS14841;
3	Cufflinks	exon	59328502	59328927	.	+	.	gene_id GRMZM2G107228; transcript_id TCONS_00016401; exon_number 2; oId CUFF.14985.1; tss_id TSS14841;
3	Cufflinks	exon	59329036	59329698	.	+	.	gene_id GRMZM2G107228; transcript_id TCONS_00016401; exon_number 3; oId CUFF.14985.1; tss_id TSS14841;
3	Cufflinks	exon	60645763	60646191	.	+	.	gene_id GRMZM2G174975; transcript_id TCONS_00016410; exon_number 1; oId CUFF.15006.1; tss_id TSS14850;
3	Cufflinks	exon	60647022	60647371	.	+	.	gene_id GRMZM2G174975; transcript_id TCONS_00016410; exon_number 2; oId CUFF.15006.1; tss_id TSS14850;
3	Cufflinks	exon	60647432	60647702	.	+	.	gene_id GRMZM2G174975; transcript_id TCONS_00016410; exon_number 3; oId CUFF.15006.1; tss_id TSS14850;
3	Cufflinks	exon	60647788	60648379	.	+	.	gene_id GRMZM2G174975; transcript_id TCONS_00016410; exon_number 4; oId CUFF.15006.1; tss_id TSS14850;
3	Cufflinks	exon	85672795	85673919	.	+	.	gene_id GRMZM2G044724; transcript_id TCONS_00016506; exon_number 1; oId CUFF.15193.1; tss_id TSS14933;
3	Cufflinks	exon	85674758	85675508	.	+	.	gene_id GRMZM2G044724; transcript_id TCONS_00016506; exon_number 2; oId CUFF.15193.1; tss_id TSS14933;
3	Cufflinks	exon	85675600	85675811	.	+	.	gene_id GRMZM2G044724; transcript_id TCONS_00016506; exon_number 3; oId CUFF.15193.1; tss_id TSS14933;
3	Cufflinks	exon	85675908	85676822	.	+	.	gene_id GRMZM2G044724; transcript_id TCONS_00016506; exon_number 4; oId CUFF.15193.1; tss_id TSS14933;
3	Cufflinks	exon	91614897	91618962	.	+	.	gene_id GRMZM2G391794; transcript_id TCONS_00016539; exon_number 1; oId CUFF.15259.1; tss_id TSS14961;
3	Cufflinks	exon	117277486	117277758	.	+	.	gene_id XLOC_014595; transcript_id TCONS_00016642; exon_number 1; oId CUFF.15471.1; tss_id TSS15057;
3	Cufflinks	exon	117278015	117278440	.	+	.	gene_id XLOC_014595; transcript_id TCONS_00016642; exon_number 2; oId CUFF.15471.1; tss_id TSS15057;
3	Cufflinks	exon	126175759	126176168	.	+	.	gene_id GRMZM2G351387; transcript_id TCONS_00016684; exon_number 1; oId CUFF.15580.1; tss_id TSS15096;
3	Cufflinks	exon	126189603	126189719	.	+	.	gene_id GRMZM2G351387; transcript_id TCONS_00016684; exon_number 2; oId CUFF.15580.1; tss_id TSS15096;
3	Cufflinks	exon	126189809	126190243	.	+	.	gene_id GRMZM2G351387; transcript_id TCONS_00016684; exon_number 3; oId CUFF.15580.1; tss_id TSS15096;

3	Cufflinks	exon	126190346	126191667	.	+	.	gene_id GRMZM2G351387; transcript_id TCONS_00016684; exon_number 4; oId CUFF.15580.1; tss_id TSS15096;
3	Cufflinks	exon	127119556	127121213	.	+	.	gene_id GRMZM2G060680; transcript_id TCONS_00016688; exon_number 1; oId CUFF.15574.1; tss_id TSS15100;
3	Cufflinks	exon	133809662	133812883	.	+	.	gene_id GRMZM2G155998; transcript_id TCONS_00016752; exon_number 1; oId CUFF.15717.1; tss_id TSS15155;
3	Cufflinks	exon	134042812	134043473	.	+	.	gene_id GRMZM2G323936; transcript_id TCONS_00016756; exon_number 1; oId CUFF.15682.1; tss_id TSS15159;
3	Cufflinks	exon	134043978	134044098	.	+	.	gene_id GRMZM2G323936; transcript_id TCONS_00016756; exon_number 2; oId CUFF.15682.1; tss_id TSS15159;
3	Cufflinks	exon	134044205	134044324	.	+	.	gene_id GRMZM2G323936; transcript_id TCONS_00016756; exon_number 3; oId CUFF.15682.1; tss_id TSS15159;
3	Cufflinks	exon	134044470	134044700	.	+	.	gene_id GRMZM2G323936; transcript_id TCONS_00016756; exon_number 4; oId CUFF.15682.1; tss_id TSS15159;
3	Cufflinks	exon	134044840	134045336	.	+	.	gene_id GRMZM2G323936; transcript_id TCONS_00016756; exon_number 5; oId CUFF.15682.1; tss_id TSS15159;
3	Cufflinks	exon	138121498	138122139	.	+	.	gene_id GRMZM2G138003; transcript_id TCONS_00016799; exon_number 1; oId CUFF.15752.1; tss_id TSS15198;
3	Cufflinks	exon	138122306	138123238	.	+	.	gene_id GRMZM2G138003; transcript_id TCONS_00016799; exon_number 2; oId CUFF.15752.1; tss_id TSS15198;
3	Cufflinks	exon	138121520	138122139	.	+	.	gene_id GRMZM2G138003; transcript_id TCONS_00016800; exon_number 1; oId CUFF.15752.2; tss_id TSS15198;
3	Cufflinks	exon	138122332	138123238	.	+	.	gene_id GRMZM2G138003; transcript_id TCONS_00016800; exon_number 2; oId CUFF.15752.2; tss_id TSS15198;
3	Cufflinks	exon	145209774	145210558	.	+	.	gene_id GRMZM2G122431; transcript_id TCONS_00016876; exon_number 1; oId CUFF.15893.1; tss_id TSS15272;
3	Cufflinks	exon	145210884	145210991	.	+	.	gene_id GRMZM2G122431; transcript_id TCONS_00016876; exon_number 2; oId CUFF.15893.1; tss_id TSS15272;
3	Cufflinks	exon	145211216	145211326	.	+	.	gene_id GRMZM2G122431; transcript_id TCONS_00016876; exon_number 3; oId CUFF.15893.1; tss_id TSS15272;
3	Cufflinks	exon	145211417	145211632	.	+	.	gene_id GRMZM2G122431; transcript_id TCONS_00016876; exon_number 4; oId CUFF.15893.1; tss_id TSS15272;
3	Cufflinks	exon	145211727	145213115	.	+	.	gene_id GRMZM2G122431; transcript_id TCONS_00016876; exon_number 5; oId CUFF.15893.1; tss_id TSS15272;
3	Cufflinks	exon	145209776	145210772	.	+	.	gene_id GRMZM2G122431; transcript_id TCONS_00016877; exon_number 1; oId CUFF.15893.2; tss_id TSS15272;
3	Cufflinks	exon	145210884	145210991	.	+	.	gene_id GRMZM2G122431; transcript_id TCONS_00016877; exon_number 2; oId CUFF.15893.2; tss_id TSS15272;
3	Cufflinks	exon	145211216	145211326	.	+	.	gene_id GRMZM2G122431; transcript_id TCONS_00016877; exon_number 3; oId CUFF.15893.2; tss_id TSS15272;
3	Cufflinks	exon	145211417	145211632	.	+	.	gene_id GRMZM2G122431; transcript_id TCONS_00016877; exon_number 4; oId CUFF.15893.2; tss_id TSS15272;
3	Cufflinks	exon	145211727	145213115	.	+	.	gene_id GRMZM2G122431; transcript_id TCONS_00016877; exon_number 5; oId CUFF.15893.2; tss_id TSS15272;
3	Cufflinks	exon	146284010	146287961	.	+	.	gene_id GRMZM2G309512; transcript_id TCONS_00016886; exon_number 1; oId CUFF.15917.1; tss_id TSS15281;
3	Cufflinks	exon	146557193	146558978	.	+	.	gene_id XLOC_014816; transcript_id TCONS_00016892; exon_number 1; oId CUFF.15927.1; tss_id TSS15287;
3	Cufflinks	exon	149437918	149438345	.	+	.	gene_id XLOC_014841; transcript_id TCONS_00016920; exon_number 1; oId CUFF.15986.1; tss_id TSS15313;
3	Cufflinks	exon	154652399	154652873	.	+	.	gene_id GRMZM2G428987; transcript_id TCONS_00016976; exon_number 1; oId CUFF.16127.3; tss_id TSS15366;
3	Cufflinks	exon	154666637	154666774	.	+	.	gene_id GRMZM2G428987; transcript_id TCONS_00016976; exon_number 2; oId CUFF.16127.3; tss_id TSS15366;
3	Cufflinks	exon	154667010	154667340	.	+	.	gene_id GRMZM2G428987; transcript_id TCONS_00016976; exon_number 3; oId CUFF.16127.3; tss_id TSS15366;
3	Cufflinks	exon	154668501	154668983	.	+	.	gene_id GRMZM2G428987; transcript_id TCONS_00016976; exon_number 4; oId CUFF.16127.3; tss_id TSS15366;
3	Cufflinks	exon	154669094	154669702	.	+	.	gene_id GRMZM2G428987; transcript_id TCONS_00016976; exon_number 5; oId CUFF.16127.3; tss_id TSS15366;
3	Cufflinks	exon	157126999	157127629	.	+	.	gene_id GRMZM2G448834; transcript_id TCONS_00017013; exon_number 1; oId CUFF.16154.1; tss_id TSS15397;
3	Cufflinks	exon	157128920	157129550	.	+	.	gene_id GRMZM2G448834; transcript_id TCONS_00017013; exon_number 2; oId CUFF.16154.1; tss_id TSS15397;
3	Cufflinks	exon	157129653	157130351	.	+	.	gene_id GRMZM2G448834; transcript_id TCONS_00017013; exon_number 3; oId CUFF.16154.1; tss_id TSS15397;
3	Cufflinks	exon	164927183	164927705	.	+	.	gene_id GRMZM2G139822; transcript_id TCONS_00017092; exon_number 1; oId CUFF.16318.1; tss_id TSS15470;
3	Cufflinks	exon	164928638	164928886	.	+	.	gene_id GRMZM2G139822; transcript_id TCONS_00017092; exon_number 2; oId CUFF.16318.1; tss_id TSS15470;
3	Cufflinks	exon	164935370	164935498	.	+	.	gene_id GRMZM2G139822; transcript_id TCONS_00017092; exon_number 3; oId CUFF.16318.1; tss_id TSS15470;

3	Cufflinks	exon	164935767	164935909	.	+	.	gene_id GRMZM2G139822; transcript_id TCONS_00017092; exon_number 4; oId CUFF.16318.1; tss_id TSS15470;
3	Cufflinks	exon	164939917	164940052	.	+	.	gene_id GRMZM2G139822; transcript_id TCONS_00017092; exon_number 5; oId CUFF.16318.1; tss_id TSS15470;
3	Cufflinks	exon	164940670	164941378	.	+	.	gene_id GRMZM2G139822; transcript_id TCONS_00017092; exon_number 6; oId CUFF.16318.1; tss_id TSS15470;
3	Cufflinks	exon	165900808	165902750	.	+	.	gene_id XLOC_015002; transcript_id TCONS_00017105; exon_number 1; oId CUFF.16313.1; tss_id TSS15482;
3	Cufflinks	exon	168968535	168969630	.	+	.	gene_id GRMZM2G035092; transcript_id TCONS_00017166; exon_number 1; oId CUFF.16388.1; tss_id TSS15539;
3	Cufflinks	exon	171752389	171752699	.	+	.	gene_id GRMZM2G138176; transcript_id TCONS_00017213; exon_number 1; oId CUFF.16461.1; tss_id TSS15575;
3	Cufflinks	exon	171753779	171754097	.	+	.	gene_id GRMZM2G138176; transcript_id TCONS_00017213; exon_number 2; oId CUFF.16461.1; tss_id TSS15575;
3	Cufflinks	exon	171754956	171755304	.	+	.	gene_id GRMZM2G138176; transcript_id TCONS_00017213; exon_number 3; oId CUFF.16461.1; tss_id TSS15575;
3	Cufflinks	exon	171755386	171755527	.	+	.	gene_id GRMZM2G138176; transcript_id TCONS_00017213; exon_number 4; oId CUFF.16461.1; tss_id TSS15575;
3	Cufflinks	exon	171755647	171756517	.	+	.	gene_id GRMZM2G138176; transcript_id TCONS_00017213; exon_number 5; oId CUFF.16461.1; tss_id TSS15575;
3	Cufflinks	exon	172376539	172377105	.	+	.	gene_id GRMZM2G078500; transcript_id TCONS_00017222; exon_number 1; oId CUFF.16476.1; tss_id TSS15582;
3	Cufflinks	exon	172378603	172378973	.	+	.	gene_id GRMZM2G078500; transcript_id TCONS_00017222; exon_number 2; oId CUFF.16476.1; tss_id TSS15582;
3	Cufflinks	exon	172379080	172379695	.	+	.	gene_id GRMZM2G078500; transcript_id TCONS_00017222; exon_number 3; oId CUFF.16476.1; tss_id TSS15582;
3	Cufflinks	exon	173048324	173049084	.	+	.	gene_id XLOC_015103; transcript_id TCONS_00017229; exon_number 1; oId CUFF.16498.1; tss_id TSS15589;
3	Cufflinks	exon	175729103	175729346	.	+	.	gene_id AC155610.2_FG007; transcript_id TCONS_00017266; exon_number 1; oId CUFF.16558.1; tss_id TSS15623;
3	Cufflinks	exon	175729452	175729922	.	+	.	gene_id AC155610.2_FG007; transcript_id TCONS_00017266; exon_number 2; oId CUFF.16558.1; tss_id TSS15623;
3	Cufflinks	exon	175731257	175732391	.	+	.	gene_id AC155610.2_FG007; transcript_id TCONS_00017266; exon_number 3; oId CUFF.16558.1; tss_id TSS15623;
3	Cufflinks	exon	180092030	180092434	.	+	.	gene_id XLOC_015216; transcript_id TCONS_00017361; exon_number 1; oId CUFF.16702.1; tss_id TSS15710;
3	Cufflinks	exon	180092531	180092931	.	+	.	gene_id XLOC_015216; transcript_id TCONS_00017361; exon_number 2; oId CUFF.16702.1; tss_id TSS15710;
3	Cufflinks	exon	180252370	180255087	.	+	.	gene_id GRMZM2G054807; transcript_id TCONS_00017363; exon_number 1; oId CUFF.16709.1; tss_id TSS15712;
3	Cufflinks	exon	187769141	187769804	.	+	.	gene_id GRMZM2G138074; transcript_id TCONS_00017496; exon_number 1; oId CUFF.16941.1; tss_id TSS15826;
3	Cufflinks	exon	187770391	187770785	.	+	.	gene_id GRMZM2G138074; transcript_id TCONS_00017496; exon_number 2; oId CUFF.16941.1; tss_id TSS15826;
3	Cufflinks	exon	187770928	187771874	.	+	.	gene_id GRMZM2G138074; transcript_id TCONS_00017496; exon_number 3; oId CUFF.16941.1; tss_id TSS15826;
3	Cufflinks	exon	189593197	189594092	.	+	.	gene_id AC233851.1_FG017; transcript_id TCONS_00017523; exon_number 1; oId CUFF.16997.1; tss_id TSS15852;
3	Cufflinks	exon	189594221	189595375	.	+	.	gene_id AC233851.1_FG017; transcript_id TCONS_00017523; exon_number 2; oId CUFF.16997.1; tss_id TSS15852;
3	Cufflinks	exon	195671890	195673197	.	+	.	gene_id XLOC_015438; transcript_id TCONS_00017622; exon_number 1; oId CUFF.17163.1; tss_id TSS15939;
3	Cufflinks	exon	195673278	195674277	.	+	.	gene_id XLOC_015438; transcript_id TCONS_00017622; exon_number 2; oId CUFF.17163.1; tss_id TSS15939;
3	Cufflinks	exon	198458900	198460981	.	+	.	gene_id XLOC_015481; transcript_id TCONS_00017675; exon_number 1; oId CUFF.17260.1; tss_id TSS15985;
3	Cufflinks	exon	200290750	200293283	.	+	.	gene_id XLOC_015519; transcript_id TCONS_00017717; exon_number 1; oId CUFF.17318.1; tss_id TSS16023;
3	Cufflinks	exon	202504869	202505294	.	+	.	gene_id GRMZM2G055276; transcript_id TCONS_00017752; exon_number 1; oId CUFF.17400.1; tss_id TSS16057;
3	Cufflinks	exon	202505568	202505958	.	+	.	gene_id GRMZM2G055276; transcript_id TCONS_00017752; exon_number 2; oId CUFF.17400.1; tss_id TSS16057;
3	Cufflinks	exon	202506051	202506195	.	+	.	gene_id GRMZM2G055276; transcript_id TCONS_00017752; exon_number 3; oId CUFF.17400.1; tss_id TSS16057;
3	Cufflinks	exon	202506674	202506832	.	+	.	gene_id GRMZM2G055276; transcript_id TCONS_00017752; exon_number 4; oId CUFF.17400.1; tss_id TSS16057;
3	Cufflinks	exon	202506917	202506973	.	+	.	gene_id GRMZM2G055276; transcript_id TCONS_00017752; exon_number 5; oId CUFF.17400.1; tss_id TSS16057;
3	Cufflinks	exon	202507455	202507948	.	+	.	gene_id GRMZM2G055276; transcript_id TCONS_00017752; exon_number 6; oId CUFF.17400.1; tss_id TSS16057;
3	Cufflinks	exon	205695411	205695728	.	+	.	gene_id GRMZM2G157317; transcript_id TCONS_00017817; exon_number 1; oId CUFF.17506.1; tss_id TSS16115;

3	Cufflinks	exon	205701156	205702868	.	+	.	gene_id GRMZM2G157317; transcript_id TCONS_00017817; exon_number 2; oId CUFF.17506.1; tss_id TSS16115;
3	Cufflinks	exon	206848092	206848639	.	+	.	gene_id GRMZM2G146913; transcript_id TCONS_00017836; exon_number 1; oId CUFF.17525.1; tss_id TSS16133;
3	Cufflinks	exon	206848746	206849182	.	+	.	gene_id GRMZM2G146913; transcript_id TCONS_00017836; exon_number 2; oId CUFF.17525.1; tss_id TSS16133;
3	Cufflinks	exon	206923316	206923724	.	+	.	gene_id GRMZM2G056388; transcript_id TCONS_00017840; exon_number 1; oId CUFF.17534.2; tss_id TSS16136;
3	Cufflinks	exon	206923820	206924388	.	+	.	gene_id GRMZM2G056388; transcript_id TCONS_00017840; exon_number 2; oId CUFF.17534.2; tss_id TSS16136;
3	Cufflinks	exon	206923316	206923724	.	+	.	gene_id GRMZM2G056388; transcript_id TCONS_00017839; exon_number 1; oId CUFF.17534.1; tss_id TSS16136;
3	Cufflinks	exon	206923849	206924388	.	+	.	gene_id GRMZM2G056388; transcript_id TCONS_00017839; exon_number 2; oId CUFF.17534.1; tss_id TSS16136;
3	Cufflinks	exon	208488885	208490355	.	+	.	gene_id GRMZM2G452807; transcript_id TCONS_00017869; exon_number 1; oId CUFF.17578.1; tss_id TSS16163;
3	Cufflinks	exon	210588285	210589844	.	+	.	gene_id GRMZM2G022931; transcript_id TCONS_00017907; exon_number 1; oId CUFF.17642.1; tss_id TSS16201;
3	Cufflinks	exon	211402697	211403131	.	+	.	gene_id GRMZM2G125044; transcript_id TCONS_00017922; exon_number 1; oId CUFF.17679.1; tss_id TSS16215;
3	Cufflinks	exon	211405224	211405267	.	+	.	gene_id GRMZM2G125044; transcript_id TCONS_00017922; exon_number 2; oId CUFF.17679.1; tss_id TSS16215;
3	Cufflinks	exon	211405615	211405726	.	+	.	gene_id GRMZM2G125044; transcript_id TCONS_00017922; exon_number 3; oId CUFF.17679.1; tss_id TSS16215;
3	Cufflinks	exon	211405822	211405900	.	+	.	gene_id GRMZM2G125044; transcript_id TCONS_00017922; exon_number 4; oId CUFF.17679.1; tss_id TSS16215;
3	Cufflinks	exon	211405990	211406633	.	+	.	gene_id GRMZM2G125044; transcript_id TCONS_00017922; exon_number 5; oId CUFF.17679.1; tss_id TSS16215;
3	Cufflinks	exon	211407189	211407474	.	+	.	gene_id GRMZM2G125044; transcript_id TCONS_00017922; exon_number 6; oId CUFF.17679.1; tss_id TSS16215;
3	Cufflinks	exon	211407549	211407816	.	+	.	gene_id GRMZM2G125044; transcript_id TCONS_00017922; exon_number 7; oId CUFF.17679.1; tss_id TSS16215;
3	Cufflinks	exon	211408717	211408866	.	+	.	gene_id GRMZM2G125044; transcript_id TCONS_00017922; exon_number 8; oId CUFF.17679.1; tss_id TSS16215;
3	Cufflinks	exon	211409326	211409802	.	+	.	gene_id GRMZM2G125044; transcript_id TCONS_00017922; exon_number 9; oId CUFF.17679.1; tss_id TSS16215;
3	Cufflinks	exon	211409946	211410523	.	+	.	gene_id GRMZM2G125044; transcript_id TCONS_00017922; exon_number 10; oId CUFF.17679.1; tss_id TSS16215;
3	Cufflinks	exon	211845106	211846918	.	+	.	gene_id GRMZM2G344848; transcript_id TCONS_00017928; exon_number 1; oId CUFF.17686.1; tss_id TSS16220;
3	Cufflinks	exon	219420009	219420721	.	+	.	gene_id XLOC_015795; transcript_id TCONS_00018034; exon_number 1; oId CUFF.17871.1; tss_id TSS16320;
3	Cufflinks	exon	221653089	221653363	.	+	.	gene_id GRMZM2G087675; transcript_id TCONS_00018072; exon_number 1; oId CUFF.17971.1; tss_id TSS16356;
3	Cufflinks	exon	221654618	221654953	.	+	.	gene_id GRMZM2G087675; transcript_id TCONS_00018072; exon_number 2; oId CUFF.17971.1; tss_id TSS16356;
3	Cufflinks	exon	221718648	221719285	.	+	.	gene_id GRMZM2G019807; transcript_id TCONS_00018077; exon_number 1; oId CUFF.17956.1; tss_id TSS16360;
3	Cufflinks	exon	221719730	221720095	.	+	.	gene_id GRMZM2G019807; transcript_id TCONS_00018077; exon_number 2; oId CUFF.17956.1; tss_id TSS16360;
3	Cufflinks	exon	221771306	221771497	.	+	.	gene_id GRMZM2G071021; transcript_id TCONS_00018081; exon_number 1; oId CUFF.17973.1; tss_id TSS16362;
3	Cufflinks	exon	221773102	221773244	.	+	.	gene_id GRMZM2G071021; transcript_id TCONS_00018081; exon_number 2; oId CUFF.17973.1; tss_id TSS16362;
3	Cufflinks	exon	221773315	221773962	.	+	.	gene_id GRMZM2G071021; transcript_id TCONS_00018081; exon_number 3; oId CUFF.17973.1; tss_id TSS16362;
3	Cufflinks	exon	221774042	221774179	.	+	.	gene_id GRMZM2G071021; transcript_id TCONS_00018081; exon_number 4; oId CUFF.17973.1; tss_id TSS16362;
3	Cufflinks	exon	221774266	221774403	.	+	.	gene_id GRMZM2G071021; transcript_id TCONS_00018081; exon_number 5; oId CUFF.17973.1; tss_id TSS16362;
3	Cufflinks	exon	221774561	221774622	.	+	.	gene_id GRMZM2G071021; transcript_id TCONS_00018081; exon_number 6; oId CUFF.17973.1; tss_id TSS16362;
3	Cufflinks	exon	221774751	221775473	.	+	.	gene_id GRMZM2G071021; transcript_id TCONS_00018081; exon_number 7; oId CUFF.17973.1; tss_id TSS16362;
3	Cufflinks	exon	229218369	229218951	.	+	.	gene_id GRMZM2G001457; transcript_id TCONS_00018232; exon_number 1; oId CUFF.18238.1; tss_id TSS16494;
3	Cufflinks	exon	229220593	229221070	.	+	.	gene_id GRMZM2G001457; transcript_id TCONS_00018232; exon_number 2; oId CUFF.18238.1; tss_id TSS16494;
3	Cufflinks	exon	229221190	229221372	.	+	.	gene_id GRMZM2G001457; transcript_id TCONS_00018232; exon_number 3; oId CUFF.18238.1; tss_id TSS16494;
3	Cufflinks	exon	229221464	229221555	.	+	.	gene_id GRMZM2G001457; transcript_id TCONS_00018232; exon_number 4; oId CUFF.18238.1; tss_id TSS16494;

3	Cufflinks	exon	229221648	229221915	.	+	.	gene_id GRMZM2G001457; transcript_id TCONS_00018232; exon_number 5; oId CUFF.18238.1; tss_id TSS16494;
3	Cufflinks	exon	229222031	229222241	.	+	.	gene_id GRMZM2G001457; transcript_id TCONS_00018232; exon_number 6; oId CUFF.18238.1; tss_id TSS16494;
3	Cufflinks	exon	229222363	229222433	.	+	.	gene_id GRMZM2G001457; transcript_id TCONS_00018232; exon_number 7; oId CUFF.18238.1; tss_id TSS16494;
3	Cufflinks	exon	229222556	229222662	.	+	.	gene_id GRMZM2G001457; transcript_id TCONS_00018232; exon_number 8; oId CUFF.18238.1; tss_id TSS16494;
3	Cufflinks	exon	229222868	229222917	.	+	.	gene_id GRMZM2G001457; transcript_id TCONS_00018232; exon_number 9; oId CUFF.18238.1; tss_id TSS16494;
3	Cufflinks	exon	229223012	229223095	.	+	.	gene_id GRMZM2G001457; transcript_id TCONS_00018232; exon_number 10; oId CUFF.18238.1; tss_id TSS16494;
3	Cufflinks	exon	229223452	229223511	.	+	.	gene_id GRMZM2G001457; transcript_id TCONS_00018232; exon_number 11; oId CUFF.18238.1; tss_id TSS16494;
3	Cufflinks	exon	229223597	229223686	.	+	.	gene_id GRMZM2G001457; transcript_id TCONS_00018232; exon_number 12; oId CUFF.18238.1; tss_id TSS16494;
3	Cufflinks	exon	229238962	229239038	.	+	.	gene_id GRMZM2G001457; transcript_id TCONS_00018232; exon_number 13; oId CUFF.18238.1; tss_id TSS16494;
3	Cufflinks	exon	229239174	229239258	.	+	.	gene_id GRMZM2G001457; transcript_id TCONS_00018232; exon_number 14; oId CUFF.18238.1; tss_id TSS16494;
3	Cufflinks	exon	229239351	229239431	.	+	.	gene_id GRMZM2G001457; transcript_id TCONS_00018232; exon_number 15; oId CUFF.18238.1; tss_id TSS16494;
3	Cufflinks	exon	229239567	229239670	.	+	.	gene_id GRMZM2G001457; transcript_id TCONS_00018232; exon_number 16; oId CUFF.18238.1; tss_id TSS16494;
3	Cufflinks	exon	229239851	229239900	.	+	.	gene_id GRMZM2G001457; transcript_id TCONS_00018232; exon_number 17; oId CUFF.18238.1; tss_id TSS16494;
3	Cufflinks	exon	229240286	229240392	.	+	.	gene_id GRMZM2G001457; transcript_id TCONS_00018232; exon_number 18; oId CUFF.18238.1; tss_id TSS16494;
3	Cufflinks	exon	229240503	229240628	.	+	.	gene_id GRMZM2G001457; transcript_id TCONS_00018232; exon_number 19; oId CUFF.18238.1; tss_id TSS16494;
3	Cufflinks	exon	229240770	229240821	.	+	.	gene_id GRMZM2G001457; transcript_id TCONS_00018232; exon_number 20; oId CUFF.18238.1; tss_id TSS16494;
3	Cufflinks	exon	229240903	229241015	.	+	.	gene_id GRMZM2G001457; transcript_id TCONS_00018232; exon_number 21; oId CUFF.18238.1; tss_id TSS16494;
3	Cufflinks	exon	229241090	229241730	.	+	.	gene_id GRMZM2G001457; transcript_id TCONS_00018232; exon_number 22; oId CUFF.18238.1; tss_id TSS16494;
3	Cufflinks	exon	230522661	230523725	.	+	.	gene_id GRMZM2G054703; transcript_id TCONS_00018264; exon_number 1; oId CUFF.18250.1; tss_id TSS16525;
3	Cufflinks	exon	230525195	230526086	.	+	.	gene_id GRMZM2G054703; transcript_id TCONS_00018264; exon_number 2; oId CUFF.18250.1; tss_id TSS16525;
3	Cufflinks	exon	4550708	4551588	.	-	.	gene_id GRMZM2G035131; transcript_id TCONS_00018392; exon_number 1; oId CUFF.14004.3; tss_id TSS16640;
3	Cufflinks	exon	4551712	4551766	.	-	.	gene_id GRMZM2G035131; transcript_id TCONS_00018392; exon_number 2; oId CUFF.14004.3; tss_id TSS16640;
3	Cufflinks	exon	4551878	4551967	.	-	.	gene_id GRMZM2G035131; transcript_id TCONS_00018392; exon_number 3; oId CUFF.14004.3; tss_id TSS16640;
3	Cufflinks	exon	4552051	4552115	.	-	.	gene_id GRMZM2G035131; transcript_id TCONS_00018392; exon_number 4; oId CUFF.14004.3; tss_id TSS16640;
3	Cufflinks	exon	4552616	4552686	.	-	.	gene_id GRMZM2G035131; transcript_id TCONS_00018392; exon_number 5; oId CUFF.14004.3; tss_id TSS16640;
3	Cufflinks	exon	4552791	4552829	.	-	.	gene_id GRMZM2G035131; transcript_id TCONS_00018392; exon_number 6; oId CUFF.14004.3; tss_id TSS16640;
3	Cufflinks	exon	4554004	4554636	.	-	.	gene_id GRMZM2G035131; transcript_id TCONS_00018392; exon_number 7; oId CUFF.14004.3; tss_id TSS16640;
3	Cufflinks	exon	4550708	4551588	.	-	.	gene_id GRMZM2G035131; transcript_id TCONS_00018394; exon_number 1; oId CUFF.14004.2; tss_id TSS16640;
3	Cufflinks	exon	4551684	4551766	.	-	.	gene_id GRMZM2G035131; transcript_id TCONS_00018394; exon_number 2; oId CUFF.14004.2; tss_id TSS16640;
3	Cufflinks	exon	4551878	4551967	.	-	.	gene_id GRMZM2G035131; transcript_id TCONS_00018394; exon_number 3; oId CUFF.14004.2; tss_id TSS16640;
3	Cufflinks	exon	4552051	4552115	.	-	.	gene_id GRMZM2G035131; transcript_id TCONS_00018394; exon_number 4; oId CUFF.14004.2; tss_id TSS16640;
3	Cufflinks	exon	4552616	4552686	.	-	.	gene_id GRMZM2G035131; transcript_id TCONS_00018394; exon_number 5; oId CUFF.14004.2; tss_id TSS16640;
3	Cufflinks	exon	4552791	4552829	.	-	.	gene_id GRMZM2G035131; transcript_id TCONS_00018394; exon_number 6; oId CUFF.14004.2; tss_id TSS16640;
3	Cufflinks	exon	4554004	4554681	.	-	.	gene_id GRMZM2G035131; transcript_id TCONS_00018394; exon_number 7; oId CUFF.14004.2; tss_id TSS16640;
3	Cufflinks	exon	4678895	4679878	.	-	.	gene_id XLOC_016106; transcript_id TCONS_00018399; exon_number 1; oId CUFF.13993.1; tss_id TSS16644;
3	Cufflinks	exon	4680104	4680599	.	-	.	gene_id XLOC_016106; transcript_id TCONS_00018399; exon_number 2; oId CUFF.13993.1; tss_id TSS16644;

3	Cufflinks	exon	7938344	7940976	.	-	.	gene_id GRMZM2G355572; transcript_id TCONS_00018459; exon_number 1; oId CUFF.14075.1; tss_id TSS16702;
3	Cufflinks	exon	8219031	8220160	.	-	.	gene_id GRMZM2G176489; transcript_id TCONS_00018469; exon_number 1; oId CUFF.14095.1; tss_id TSS16707;
3	Cufflinks	exon	8220318	8221073	.	-	.	gene_id GRMZM2G176489; transcript_id TCONS_00018469; exon_number 2; oId CUFF.14095.1; tss_id TSS16707;
3	Cufflinks	exon	8221259	8221674	.	-	.	gene_id GRMZM2G176489; transcript_id TCONS_00018469; exon_number 3; oId CUFF.14095.1; tss_id TSS16707;
3	Cufflinks	exon	9856999	9857500	.	-	.	gene_id XLOC_016200; transcript_id TCONS_00018502; exon_number 1; oId CUFF.14158.1; tss_id TSS16739;
3	Cufflinks	exon	19973177	19973784	.	-	.	gene_id GRMZM2G467671; transcript_id TCONS_00018631; exon_number 1; oId CUFF.14440.1; tss_id TSS16858;
3	Cufflinks	exon	19973877	19975077	.	-	.	gene_id GRMZM2G467671; transcript_id TCONS_00018631; exon_number 2; oId CUFF.14440.1; tss_id TSS16858;
3	Cufflinks	exon	19975270	19975305	.	-	.	gene_id GRMZM2G467671; transcript_id TCONS_00018631; exon_number 3; oId CUFF.14440.1; tss_id TSS16858;
3	Cufflinks	exon	19976475	19977322	.	-	.	gene_id GRMZM2G467671; transcript_id TCONS_00018631; exon_number 4; oId CUFF.14440.1; tss_id TSS16858;
3	Cufflinks	exon	26309355	26310879	.	-	.	gene_id GRMZM2G104829; transcript_id TCONS_00018690; exon_number 1; oId CUFF.14540.1; tss_id TSS16908;
3	Cufflinks	exon	26311129	26311225	.	-	.	gene_id GRMZM2G104829; transcript_id TCONS_00018690; exon_number 2; oId CUFF.14540.1; tss_id TSS16908;
3	Cufflinks	exon	26311329	26311616	.	-	.	gene_id GRMZM2G104829; transcript_id TCONS_00018690; exon_number 3; oId CUFF.14540.1; tss_id TSS16908;
3	Cufflinks	exon	26311828	26312448	.	-	.	gene_id GRMZM2G104829; transcript_id TCONS_00018690; exon_number 4; oId CUFF.14540.1; tss_id TSS16908;
3	Cufflinks	exon	37359987	37360498	.	-	.	gene_id GRMZM2G132913; transcript_id TCONS_00018805; exon_number 1; oId CUFF.14707.1; tss_id TSS17014;
3	Cufflinks	exon	37360582	37360752	.	-	.	gene_id GRMZM2G132913; transcript_id TCONS_00018805; exon_number 2; oId CUFF.14707.1; tss_id TSS17014;
3	Cufflinks	exon	37360839	37361918	.	-	.	gene_id GRMZM2G132913; transcript_id TCONS_00018805; exon_number 3; oId CUFF.14707.1; tss_id TSS17014;
3	Cufflinks	exon	40529064	40529664	.	-	.	gene_id GRMZM2G089982; transcript_id TCONS_00018829; exon_number 1; oId CUFF.14752.1; tss_id TSS17038;
3	Cufflinks	exon	40529766	40529931	.	-	.	gene_id GRMZM2G089982; transcript_id TCONS_00018829; exon_number 2; oId CUFF.14752.1; tss_id TSS17038;
3	Cufflinks	exon	40530064	40530255	.	-	.	gene_id GRMZM2G089982; transcript_id TCONS_00018829; exon_number 3; oId CUFF.14752.1; tss_id TSS17038;
3	Cufflinks	exon	40530888	40531245	.	-	.	gene_id GRMZM2G089982; transcript_id TCONS_00018829; exon_number 4; oId CUFF.14752.1; tss_id TSS17038;
3	Cufflinks	exon	41742447	41743618	.	-	.	gene_id GRMZM2G144648; transcript_id TCONS_00018853; exon_number 1; oId CUFF.14774.1; tss_id TSS17059;
3	Cufflinks	exon	41744136	41744589	.	-	.	gene_id GRMZM2G144648; transcript_id TCONS_00018853; exon_number 2; oId CUFF.14774.1; tss_id TSS17059;
3	Cufflinks	exon	44224052	44225188	.	-	.	gene_id GRMZM2G003883; transcript_id TCONS_00018869; exon_number 1; oId CUFF.14807.1; tss_id TSS17073;
3	Cufflinks	exon	44225268	44225729	.	-	.	gene_id GRMZM2G003883; transcript_id TCONS_00018869; exon_number 2; oId CUFF.14807.1; tss_id TSS17073;
3	Cufflinks	exon	44229198	44229674	.	-	.	gene_id GRMZM2G003883; transcript_id TCONS_00018869; exon_number 3; oId CUFF.14807.1; tss_id TSS17073;
3	Cufflinks	exon	48494950	48496392	.	-	.	gene_id GRMZM2G017520; transcript_id TCONS_00018893; exon_number 1; oId CUFF.14852.1; tss_id TSS17096;
3	Cufflinks	exon	48496510	48496639	.	-	.	gene_id GRMZM2G017520; transcript_id TCONS_00018893; exon_number 2; oId CUFF.14852.1; tss_id TSS17096;
3	Cufflinks	exon	48496804	48497362	.	-	.	gene_id GRMZM2G017520; transcript_id TCONS_00018893; exon_number 3; oId CUFF.14852.1; tss_id TSS17096;
3	Cufflinks	exon	52452031	52452887	.	-	.	gene_id GRMZM2G363893; transcript_id TCONS_00018921; exon_number 1; oId CUFF.14909.1; tss_id TSS17124;
3	Cufflinks	exon	52453333	52453905	.	-	.	gene_id GRMZM2G363893; transcript_id TCONS_00018921; exon_number 2; oId CUFF.14909.1; tss_id TSS17124;
3	Cufflinks	exon	52453985	52454284	.	-	.	gene_id GRMZM2G363893; transcript_id TCONS_00018921; exon_number 3; oId CUFF.14909.1; tss_id TSS17124;
3	Cufflinks	exon	137557525	137558581	.	-	.	gene_id AC209377.3_FG003; transcript_id TCONS_00019403; exon_number 1; oId CUFF.15731.1; tss_id TSS17569;
3	Cufflinks	exon	138121201	138121870	.	-	.	gene_id XLOC_017014; transcript_id TCONS_00019409; exon_number 1; oId CUFF.15751.2; tss_id TSS17574;
3	Cufflinks	exon	138122357	138123238	.	-	.	gene_id XLOC_017014; transcript_id TCONS_00019409; exon_number 2; oId CUFF.15751.2; tss_id TSS17574;
3	Cufflinks	exon	142724521	142725513	.	-	.	gene_id GRMZM2G152889; transcript_id TCONS_00019454; exon_number 1; oId CUFF.15846.1; tss_id TSS17618;
3	Cufflinks	exon	142725631	142725771	.	-	.	gene_id GRMZM2G152889; transcript_id TCONS_00019454; exon_number 2; oId CUFF.15846.1; tss_id TSS17618;

3	Cufflinks	exon	142725837	142725887	.	-	.	gene_id GRMZM2G152889; transcript_id TCONS_00019454; exon_number 3; oId CUFF.15846.1; tss_id TSS17618;
3	Cufflinks	exon	142725983	142726063	.	-	.	gene_id GRMZM2G152889; transcript_id TCONS_00019454; exon_number 4; oId CUFF.15846.1; tss_id TSS17618;
3	Cufflinks	exon	142726439	142726534	.	-	.	gene_id GRMZM2G152889; transcript_id TCONS_00019454; exon_number 5; oId CUFF.15846.1; tss_id TSS17618;
3	Cufflinks	exon	142726920	142727095	.	-	.	gene_id GRMZM2G152889; transcript_id TCONS_00019454; exon_number 6; oId CUFF.15846.1; tss_id TSS17618;
3	Cufflinks	exon	142727348	142727421	.	-	.	gene_id GRMZM2G152889; transcript_id TCONS_00019454; exon_number 7; oId CUFF.15846.1; tss_id TSS17618;
3	Cufflinks	exon	142727546	142727653	.	-	.	gene_id GRMZM2G152889; transcript_id TCONS_00019454; exon_number 8; oId CUFF.15846.1; tss_id TSS17618;
3	Cufflinks	exon	142727739	142727875	.	-	.	gene_id GRMZM2G152889; transcript_id TCONS_00019454; exon_number 9; oId CUFF.15846.1; tss_id TSS17618;
3	Cufflinks	exon	142728031	142728081	.	-	.	gene_id GRMZM2G152889; transcript_id TCONS_00019454; exon_number 10; oId CUFF.15846.1; tss_id TSS17618;
3	Cufflinks	exon	142730945	142731055	.	-	.	gene_id GRMZM2G152889; transcript_id TCONS_00019454; exon_number 11; oId CUFF.15846.1; tss_id TSS17618;
3	Cufflinks	exon	142731358	142731430	.	-	.	gene_id GRMZM2G152889; transcript_id TCONS_00019454; exon_number 12; oId CUFF.15846.1; tss_id TSS17618;
3	Cufflinks	exon	142732458	142732534	.	-	.	gene_id GRMZM2G152889; transcript_id TCONS_00019454; exon_number 13; oId CUFF.15846.1; tss_id TSS17618;
3	Cufflinks	exon	142733445	142733559	.	-	.	gene_id GRMZM2G152889; transcript_id TCONS_00019454; exon_number 14; oId CUFF.15846.1; tss_id TSS17618;
3	Cufflinks	exon	142734430	142734864	.	-	.	gene_id GRMZM2G152889; transcript_id TCONS_00019454; exon_number 15; oId CUFF.15846.1; tss_id TSS17618;
3	Cufflinks	exon	146286679	146287500	.	-	.	gene_id GRMZM2G309479; transcript_id TCONS_00019508; exon_number 1; oId CUFF.15918.1; tss_id TSS17667;
3	Cufflinks	exon	146556764	146557927	.	-	.	gene_id GRMZM2G103342; transcript_id TCONS_00019511; exon_number 1; oId CUFF.15924.1; tss_id TSS17670;
3	Cufflinks	exon	146558017	146558820	.	-	.	gene_id GRMZM2G103342; transcript_id TCONS_00019511; exon_number 2; oId CUFF.15924.1; tss_id TSS17670;
3	Cufflinks	exon	152240411	152241271	.	-	.	gene_id GRMZM2G050997; transcript_id TCONS_00019580; exon_number 1; oId CUFF.16052.1; tss_id TSS17735;
3	Cufflinks	exon	152241371	152241633	.	-	.	gene_id GRMZM2G050997; transcript_id TCONS_00019580; exon_number 2; oId CUFF.16052.1; tss_id TSS17735;
3	Cufflinks	exon	152241707	152241967	.	-	.	gene_id GRMZM2G050997; transcript_id TCONS_00019580; exon_number 3; oId CUFF.16052.1; tss_id TSS17735;
3	Cufflinks	exon	152242058	152242185	.	-	.	gene_id GRMZM2G050997; transcript_id TCONS_00019580; exon_number 4; oId CUFF.16052.1; tss_id TSS17735;
3	Cufflinks	exon	152242293	152243105	.	-	.	gene_id GRMZM2G050997; transcript_id TCONS_00019580; exon_number 5; oId CUFF.16052.1; tss_id TSS17735;
3	Cufflinks	exon	160370641	160371347	.	-	.	gene_id GRMZM2G129114; transcript_id TCONS_00019678; exon_number 1; oId CUFF.16206.1; tss_id TSS17827;
3	Cufflinks	exon	160371452	160371829	.	-	.	gene_id GRMZM2G129114; transcript_id TCONS_00019678; exon_number 2; oId CUFF.16206.1; tss_id TSS17827;
3	Cufflinks	exon	160371948	160372177	.	-	.	gene_id GRMZM2G129114; transcript_id TCONS_00019678; exon_number 3; oId CUFF.16206.1; tss_id TSS17827;
3	Cufflinks	exon	160372263	160372491	.	-	.	gene_id GRMZM2G129114; transcript_id TCONS_00019678; exon_number 4; oId CUFF.16206.1; tss_id TSS17827;
3	Cufflinks	exon	173048156	173048825	.	-	.	gene_id GRMZM2G049373; transcript_id TCONS_00019847; exon_number 1; oId CUFF.16496.1; tss_id TSS17966;
3	Cufflinks	exon	173049757	173050136	.	-	.	gene_id GRMZM2G049373; transcript_id TCONS_00019847; exon_number 2; oId CUFF.16496.1; tss_id TSS17966;
3	Cufflinks	exon	173050260	173050637	.	-	.	gene_id GRMZM2G049373; transcript_id TCONS_00019847; exon_number 3; oId CUFF.16496.1; tss_id TSS17966;
3	Cufflinks	exon	176347957	176348895	.	-	.	gene_id XLOC_017423; transcript_id TCONS_00019893; exon_number 1; oId CUFF.16579.1; tss_id TSS18006;
3	Cufflinks	exon	178185823	178186744	.	-	.	gene_id GRMZM2G177518; transcript_id TCONS_00019924; exon_number 1; oId CUFF.16651.1; tss_id TSS18034;
3	Cufflinks	exon	178186879	178188427	.	-	.	gene_id GRMZM2G177518; transcript_id TCONS_00019924; exon_number 2; oId CUFF.16651.1; tss_id TSS18034;
3	Cufflinks	exon	179468333	179468806	.	-	.	gene_id XLOC_017468; transcript_id TCONS_00019945; exon_number 1; oId CUFF.16697.1; tss_id TSS18052;
3	Cufflinks	exon	179470545	179470593	.	-	.	gene_id XLOC_017468; transcript_id TCONS_00019945; exon_number 2; oId CUFF.16697.1; tss_id TSS18052;
3	Cufflinks	exon	179470692	179470746	.	-	.	gene_id XLOC_017468; transcript_id TCONS_00019945; exon_number 3; oId CUFF.16697.1; tss_id TSS18052;
3	Cufflinks	exon	179470870	179471039	.	-	.	gene_id XLOC_017468; transcript_id TCONS_00019945; exon_number 4; oId CUFF.16697.1; tss_id TSS18052;
3	Cufflinks	exon	179471443	179471518	.	-	.	gene_id XLOC_017468; transcript_id TCONS_00019945; exon_number 5; oId CUFF.16697.1; tss_id TSS18052;

3	Cufflinks	exon	179471898	179472203	.	-	.	gene_id XLOC_017468; transcript_id TCONS_00019945; exon_number 6; oId CUFF.16697.1; tss_id TSS18052;
3	Cufflinks	exon	179468333	179468806	.	-	.	gene_id XLOC_017468; transcript_id TCONS_00019946; exon_number 1; oId CUFF.16697.2; tss_id TSS18052;
3	Cufflinks	exon	179470545	179470593	.	-	.	gene_id XLOC_017468; transcript_id TCONS_00019946; exon_number 2; oId CUFF.16697.2; tss_id TSS18052;
3	Cufflinks	exon	179470692	179470746	.	-	.	gene_id XLOC_017468; transcript_id TCONS_00019946; exon_number 3; oId CUFF.16697.2; tss_id TSS18052;
3	Cufflinks	exon	179470870	179471039	.	-	.	gene_id XLOC_017468; transcript_id TCONS_00019946; exon_number 4; oId CUFF.16697.2; tss_id TSS18052;
3	Cufflinks	exon	179471440	179471518	.	-	.	gene_id XLOC_017468; transcript_id TCONS_00019946; exon_number 5; oId CUFF.16697.2; tss_id TSS18052;
3	Cufflinks	exon	179471898	179472231	.	-	.	gene_id XLOC_017468; transcript_id TCONS_00019946; exon_number 6; oId CUFF.16697.2; tss_id TSS18052;
3	Cufflinks	exon	180092014	180092401	.	-	.	gene_id XLOC_017482; transcript_id TCONS_00019961; exon_number 1; oId CUFF.16701.1; tss_id TSS18066;
3	Cufflinks	exon	181805519	181805970	.	-	.	gene_id GRMZM2G126077; transcript_id TCONS_00019991; exon_number 1; oId CUFF.16750.1; tss_id TSS18093;
3	Cufflinks	exon	181806047	181806115	.	-	.	gene_id GRMZM2G126077; transcript_id TCONS_00019991; exon_number 2; oId CUFF.16750.1; tss_id TSS18093;
3	Cufflinks	exon	181806219	181806781	.	-	.	gene_id GRMZM2G126077; transcript_id TCONS_00019991; exon_number 3; oId CUFF.16750.1; tss_id TSS18093;
3	Cufflinks	exon	181806874	181807107	.	-	.	gene_id GRMZM2G126077; transcript_id TCONS_00019991; exon_number 4; oId CUFF.16750.1; tss_id TSS18093;
3	Cufflinks	exon	182096591	182096977	.	-	.	gene_id GRMZM2G426953; transcript_id TCONS_00019998; exon_number 1; oId CUFF.16774.1; tss_id TSS18100;
3	Cufflinks	exon	182097062	182097140	.	-	.	gene_id GRMZM2G426953; transcript_id TCONS_00019998; exon_number 2; oId CUFF.16774.1; tss_id TSS18100;
3	Cufflinks	exon	182097224	182097352	.	-	.	gene_id GRMZM2G426953; transcript_id TCONS_00019998; exon_number 3; oId CUFF.16774.1; tss_id TSS18100;
3	Cufflinks	exon	182097432	182097578	.	-	.	gene_id GRMZM2G426953; transcript_id TCONS_00019998; exon_number 4; oId CUFF.16774.1; tss_id TSS18100;
3	Cufflinks	exon	182097662	182097812	.	-	.	gene_id GRMZM2G426953; transcript_id TCONS_00019998; exon_number 5; oId CUFF.16774.1; tss_id TSS18100;
3	Cufflinks	exon	182097924	182098012	.	-	.	gene_id GRMZM2G426953; transcript_id TCONS_00019998; exon_number 6; oId CUFF.16774.1; tss_id TSS18100;
3	Cufflinks	exon	182098124	182098226	.	-	.	gene_id GRMZM2G426953; transcript_id TCONS_00019998; exon_number 7; oId CUFF.16774.1; tss_id TSS18100;
3	Cufflinks	exon	182098309	182098424	.	-	.	gene_id GRMZM2G426953; transcript_id TCONS_00019998; exon_number 8; oId CUFF.16774.1; tss_id TSS18100;
3	Cufflinks	exon	182098508	182098603	.	-	.	gene_id GRMZM2G426953; transcript_id TCONS_00019998; exon_number 9; oId CUFF.16774.1; tss_id TSS18100;
3	Cufflinks	exon	182098710	182099516	.	-	.	gene_id GRMZM2G426953; transcript_id TCONS_00019998; exon_number 10; oId CUFF.16774.1; tss_id TSS18100;
3	Cufflinks	exon	182099610	182099723	.	-	.	gene_id GRMZM2G426953; transcript_id TCONS_00019998; exon_number 11; oId CUFF.16774.1; tss_id TSS18100;
3	Cufflinks	exon	182099833	182099881	.	-	.	gene_id GRMZM2G426953; transcript_id TCONS_00019998; exon_number 12; oId CUFF.16774.1; tss_id TSS18100;
3	Cufflinks	exon	182099974	182100136	.	-	.	gene_id GRMZM2G426953; transcript_id TCONS_00019998; exon_number 13; oId CUFF.16774.1; tss_id TSS18100;
3	Cufflinks	exon	182100753	182101272	.	-	.	gene_id GRMZM2G426953; transcript_id TCONS_00019998; exon_number 14; oId CUFF.16774.1; tss_id TSS18100;
3	Cufflinks	exon	183701067	183702321	.	-	.	gene_id GRMZM2G132169; transcript_id TCONS_00020022; exon_number 1; oId CUFF.16825.1; tss_id TSS18123;
3	Cufflinks	exon	183702416	183702544	.	-	.	gene_id GRMZM2G132169; transcript_id TCONS_00020022; exon_number 2; oId CUFF.16825.1; tss_id TSS18123;
3	Cufflinks	exon	183702629	183702873	.	-	.	gene_id GRMZM2G132169; transcript_id TCONS_00020022; exon_number 3; oId CUFF.16825.1; tss_id TSS18123;
3	Cufflinks	exon	183703016	183703167	.	-	.	gene_id GRMZM2G132169; transcript_id TCONS_00020022; exon_number 4; oId CUFF.16825.1; tss_id TSS18123;
3	Cufflinks	exon	183703337	183703595	.	-	.	gene_id GRMZM2G132169; transcript_id TCONS_00020022; exon_number 5; oId CUFF.16825.1; tss_id TSS18123;
3	Cufflinks	exon	187770273	187771591	.	-	.	gene_id XLOC_017607; transcript_id TCONS_00020105; exon_number 1; oId CUFF.16942.1; tss_id TSS18196;
3	Cufflinks	exon	188728061	188729514	.	-	.	gene_id GRMZM2G310368; transcript_id TCONS_00020130; exon_number 1; oId CUFF.16975.1; tss_id TSS18217;
3	Cufflinks	exon	189832965	189833531	.	-	.	gene_id XLOC_017639; transcript_id TCONS_00020144; exon_number 1; oId CUFF.17009.1; tss_id TSS18230;
3	Cufflinks	exon	197962668	197962731	.	-	.	gene_id XLOC_017756; transcript_id TCONS_00020268; exon_number 1; oId CUFF.17251.1; tss_id TSS18348;
3	Cufflinks	exon	197962867	197963235	.	-	.	gene_id XLOC_017756; transcript_id TCONS_00020268; exon_number 2; oId CUFF.17251.1; tss_id TSS18348;

3	Cufflinks	exon	198462555	198463329	.	-	.	gene_id GRMZM2G358830; transcript_id TCONS_00020276; exon_number 1; oId CUFF.17284.2; tss_id TSS18355;
3	Cufflinks	exon	198463420	198463581	.	-	.	gene_id GRMZM2G358830; transcript_id TCONS_00020276; exon_number 2; oId CUFF.17284.2; tss_id TSS18355;
3	Cufflinks	exon	198463799	198463946	.	-	.	gene_id GRMZM2G358830; transcript_id TCONS_00020276; exon_number 3; oId CUFF.17284.2; tss_id TSS18355;
3	Cufflinks	exon	198464081	198464157	.	-	.	gene_id GRMZM2G358830; transcript_id TCONS_00020276; exon_number 4; oId CUFF.17284.2; tss_id TSS18355;
3	Cufflinks	exon	198464243	198464313	.	-	.	gene_id GRMZM2G358830; transcript_id TCONS_00020276; exon_number 5; oId CUFF.17284.2; tss_id TSS18355;
3	Cufflinks	exon	198464419	198464505	.	-	.	gene_id GRMZM2G358830; transcript_id TCONS_00020276; exon_number 6; oId CUFF.17284.2; tss_id TSS18355;
3	Cufflinks	exon	198464591	198466556	.	-	.	gene_id GRMZM2G358830; transcript_id TCONS_00020276; exon_number 7; oId CUFF.17284.2; tss_id TSS18355;
3	Cufflinks	exon	198462555	198463329	.	-	.	gene_id GRMZM2G358830; transcript_id TCONS_00020275; exon_number 1; oId CUFF.17284.1; tss_id TSS18355;
3	Cufflinks	exon	198463420	198463581	.	-	.	gene_id GRMZM2G358830; transcript_id TCONS_00020275; exon_number 2; oId CUFF.17284.1; tss_id TSS18355;
3	Cufflinks	exon	198463799	198463946	.	-	.	gene_id GRMZM2G358830; transcript_id TCONS_00020275; exon_number 3; oId CUFF.17284.1; tss_id TSS18355;
3	Cufflinks	exon	198464081	198464157	.	-	.	gene_id GRMZM2G358830; transcript_id TCONS_00020275; exon_number 4; oId CUFF.17284.1; tss_id TSS18355;
3	Cufflinks	exon	198464243	198464313	.	-	.	gene_id GRMZM2G358830; transcript_id TCONS_00020275; exon_number 5; oId CUFF.17284.1; tss_id TSS18355;
3	Cufflinks	exon	198464419	198464505	.	-	.	gene_id GRMZM2G358830; transcript_id TCONS_00020275; exon_number 6; oId CUFF.17284.1; tss_id TSS18355;
3	Cufflinks	exon	198464591	198465061	.	-	.	gene_id GRMZM2G358830; transcript_id TCONS_00020275; exon_number 7; oId CUFF.17284.1; tss_id TSS18355;
3	Cufflinks	exon	198465467	198466556	.	-	.	gene_id GRMZM2G358830; transcript_id TCONS_00020275; exon_number 8; oId CUFF.17284.1; tss_id TSS18355;
3	Cufflinks	exon	199322717	199323781	.	-	.	gene_id GRMZM2G177929; transcript_id TCONS_00020290; exon_number 1; oId CUFF.17288.4; tss_id TSS18369;
3	Cufflinks	exon	199323912	199324100	.	-	.	gene_id GRMZM2G177929; transcript_id TCONS_00020290; exon_number 2; oId CUFF.17288.4; tss_id TSS18369;
3	Cufflinks	exon	199324686	199325008	.	-	.	gene_id GRMZM2G177929; transcript_id TCONS_00020290; exon_number 3; oId CUFF.17288.4; tss_id TSS18369;
3	Cufflinks	exon	199325210	199325504	.	-	.	gene_id GRMZM2G177929; transcript_id TCONS_00020290; exon_number 4; oId CUFF.17288.4; tss_id TSS18369;
3	Cufflinks	exon	199322717	199323781	.	-	.	gene_id GRMZM2G177929; transcript_id TCONS_00020291; exon_number 1; oId CUFF.17288.1; tss_id TSS18370;
3	Cufflinks	exon	199323912	199324100	.	-	.	gene_id GRMZM2G177929; transcript_id TCONS_00020291; exon_number 2; oId CUFF.17288.1; tss_id TSS18370;
3	Cufflinks	exon	199324686	199325008	.	-	.	gene_id GRMZM2G177929; transcript_id TCONS_00020291; exon_number 3; oId CUFF.17288.1; tss_id TSS18370;
3	Cufflinks	exon	199325128	199326069	.	-	.	gene_id GRMZM2G177929; transcript_id TCONS_00020291; exon_number 4; oId CUFF.17288.1; tss_id TSS18370;
3	Cufflinks	exon	199322717	199323781	.	-	.	gene_id GRMZM2G177929; transcript_id TCONS_00020293; exon_number 1; oId CUFF.17288.3; tss_id TSS18371;
3	Cufflinks	exon	199323912	199324100	.	-	.	gene_id GRMZM2G177929; transcript_id TCONS_00020293; exon_number 2; oId CUFF.17288.3; tss_id TSS18371;
3	Cufflinks	exon	199324686	199325008	.	-	.	gene_id GRMZM2G177929; transcript_id TCONS_00020293; exon_number 3; oId CUFF.17288.3; tss_id TSS18371;
3	Cufflinks	exon	199326255	199326605	.	-	.	gene_id GRMZM2G177929; transcript_id TCONS_00020293; exon_number 4; oId CUFF.17288.3; tss_id TSS18371;
3	Cufflinks	exon	200263486	200265308	.	-	.	gene_id GRMZM2G381025; transcript_id TCONS_00020311; exon_number 1; oId CUFF.17308.1; tss_id TSS18388;
3	Cufflinks	exon	200290750	200292783	.	-	.	gene_id XLOC_017795; transcript_id TCONS_00020313; exon_number 1; oId CUFF.17319.1; tss_id TSS18390;
3	Cufflinks	exon	200668046	200668710	.	-	.	gene_id XLOC_017798; transcript_id TCONS_00020316; exon_number 1; oId CUFF.17312.1; tss_id TSS18393;
3	Cufflinks	exon	201696573	201700349	.	-	.	gene_id XLOC_017816; transcript_id TCONS_00020336; exon_number 1; oId CUFF.17374.1; tss_id TSS18413;
3	Cufflinks	exon	204542143	204542665	.	-	.	gene_id GRMZM2G111611; transcript_id TCONS_00020385; exon_number 1; oId CUFF.17462.1; tss_id TSS18458;
3	Cufflinks	exon	204545098	204545268	.	-	.	gene_id GRMZM2G111611; transcript_id TCONS_00020385; exon_number 2; oId CUFF.17462.1; tss_id TSS18458;
3	Cufflinks	exon	204547259	204547447	.	-	.	gene_id GRMZM2G111611; transcript_id TCONS_00020385; exon_number 3; oId CUFF.17462.1; tss_id TSS18458;
3	Cufflinks	exon	204547692	204547750	.	-	.	gene_id GRMZM2G111611; transcript_id TCONS_00020385; exon_number 4; oId CUFF.17462.1; tss_id TSS18458;
3	Cufflinks	exon	204547852	204548156	.	-	.	gene_id GRMZM2G111611; transcript_id TCONS_00020385; exon_number 5; oId CUFF.17462.1; tss_id TSS18458;

3	Cufflinks	exon	209050298	209053592	.	-	.	gene_id GRMZM2G423202; transcript_id TCONS_00020474; exon_number 1; oId CUFF.17606.1; tss_id TSS18544;
3	Cufflinks	exon	210617138	210617957	.	-	.	gene_id GRMZM2G023436; transcript_id TCONS_00020500; exon_number 1; oId CUFF.17651.1; tss_id TSS18567;
3	Cufflinks	exon	210618419	210619025	.	-	.	gene_id GRMZM2G023436; transcript_id TCONS_00020500; exon_number 2; oId CUFF.17651.1; tss_id TSS18567;
3	Cufflinks	exon	213469179	213469830	.	-	.	gene_id GRMZM2G330302; transcript_id TCONS_00020539; exon_number 1; oId CUFF.17752.2; tss_id TSS18605;
3	Cufflinks	exon	213469952	213470045	.	-	.	gene_id GRMZM2G330302; transcript_id TCONS_00020539; exon_number 2; oId CUFF.17752.2; tss_id TSS18605;
3	Cufflinks	exon	213470174	213470254	.	-	.	gene_id GRMZM2G330302; transcript_id TCONS_00020539; exon_number 3; oId CUFF.17752.2; tss_id TSS18605;
3	Cufflinks	exon	213470367	213470415	.	-	.	gene_id GRMZM2G330302; transcript_id TCONS_00020539; exon_number 4; oId CUFF.17752.2; tss_id TSS18605;
3	Cufflinks	exon	213470514	213470599	.	-	.	gene_id GRMZM2G330302; transcript_id TCONS_00020539; exon_number 5; oId CUFF.17752.2; tss_id TSS18605;
3	Cufflinks	exon	213470683	213470910	.	-	.	gene_id GRMZM2G330302; transcript_id TCONS_00020539; exon_number 6; oId CUFF.17752.2; tss_id TSS18605;
3	Cufflinks	exon	213470998	213471036	.	-	.	gene_id GRMZM2G330302; transcript_id TCONS_00020539; exon_number 7; oId CUFF.17752.2; tss_id TSS18605;
3	Cufflinks	exon	213471124	213471213	.	-	.	gene_id GRMZM2G330302; transcript_id TCONS_00020539; exon_number 8; oId CUFF.17752.2; tss_id TSS18605;
3	Cufflinks	exon	213471696	213471758	.	-	.	gene_id GRMZM2G330302; transcript_id TCONS_00020539; exon_number 9; oId CUFF.17752.2; tss_id TSS18605;
3	Cufflinks	exon	213471882	213471960	.	-	.	gene_id GRMZM2G330302; transcript_id TCONS_00020539; exon_number 10; oId CUFF.17752.2; tss_id TSS18605;
3	Cufflinks	exon	213472051	213472097	.	-	.	gene_id GRMZM2G330302; transcript_id TCONS_00020539; exon_number 11; oId CUFF.17752.2; tss_id TSS18605;
3	Cufflinks	exon	213472186	213472248	.	-	.	gene_id GRMZM2G330302; transcript_id TCONS_00020539; exon_number 12; oId CUFF.17752.2; tss_id TSS18605;
3	Cufflinks	exon	213472333	213472383	.	-	.	gene_id GRMZM2G330302; transcript_id TCONS_00020539; exon_number 13; oId CUFF.17752.2; tss_id TSS18605;
3	Cufflinks	exon	213472501	213472584	.	-	.	gene_id GRMZM2G330302; transcript_id TCONS_00020539; exon_number 14; oId CUFF.17752.2; tss_id TSS18605;
3	Cufflinks	exon	213472702	213472766	.	-	.	gene_id GRMZM2G330302; transcript_id TCONS_00020539; exon_number 15; oId CUFF.17752.2; tss_id TSS18605;
3	Cufflinks	exon	213472868	213472924	.	-	.	gene_id GRMZM2G330302; transcript_id TCONS_00020539; exon_number 16; oId CUFF.17752.2; tss_id TSS18605;
3	Cufflinks	exon	213473127	213473244	.	-	.	gene_id GRMZM2G330302; transcript_id TCONS_00020539; exon_number 17; oId CUFF.17752.2; tss_id TSS18605;
3	Cufflinks	exon	213473344	213473394	.	-	.	gene_id GRMZM2G330302; transcript_id TCONS_00020539; exon_number 18; oId CUFF.17752.2; tss_id TSS18605;
3	Cufflinks	exon	213474137	213474223	.	-	.	gene_id GRMZM2G330302; transcript_id TCONS_00020539; exon_number 19; oId CUFF.17752.2; tss_id TSS18605;
3	Cufflinks	exon	213474428	213474507	.	-	.	gene_id GRMZM2G330302; transcript_id TCONS_00020539; exon_number 20; oId CUFF.17752.2; tss_id TSS18605;
3	Cufflinks	exon	213475294	213475662	.	-	.	gene_id GRMZM2G330302; transcript_id TCONS_00020539; exon_number 21; oId CUFF.17752.2; tss_id TSS18605;
3	Cufflinks	exon	213469179	213469830	.	-	.	gene_id GRMZM2G330302; transcript_id TCONS_00020538; exon_number 1; oId CUFF.17752.1; tss_id TSS18605;
3	Cufflinks	exon	213469952	213470045	.	-	.	gene_id GRMZM2G330302; transcript_id TCONS_00020538; exon_number 2; oId CUFF.17752.1; tss_id TSS18605;
3	Cufflinks	exon	213470174	213470254	.	-	.	gene_id GRMZM2G330302; transcript_id TCONS_00020538; exon_number 3; oId CUFF.17752.1; tss_id TSS18605;
3	Cufflinks	exon	213470367	213470415	.	-	.	gene_id GRMZM2G330302; transcript_id TCONS_00020538; exon_number 4; oId CUFF.17752.1; tss_id TSS18605;
3	Cufflinks	exon	213470514	213470599	.	-	.	gene_id GRMZM2G330302; transcript_id TCONS_00020538; exon_number 5; oId CUFF.17752.1; tss_id TSS18605;
3	Cufflinks	exon	213470683	213470910	.	-	.	gene_id GRMZM2G330302; transcript_id TCONS_00020538; exon_number 6; oId CUFF.17752.1; tss_id TSS18605;
3	Cufflinks	exon	213470998	213471036	.	-	.	gene_id GRMZM2G330302; transcript_id TCONS_00020538; exon_number 7; oId CUFF.17752.1; tss_id TSS18605;
3	Cufflinks	exon	213471124	213471213	.	-	.	gene_id GRMZM2G330302; transcript_id TCONS_00020538; exon_number 8; oId CUFF.17752.1; tss_id TSS18605;
3	Cufflinks	exon	213471696	213471758	.	-	.	gene_id GRMZM2G330302; transcript_id TCONS_00020538; exon_number 9; oId CUFF.17752.1; tss_id TSS18605;
3	Cufflinks	exon	213471882	213471960	.	-	.	gene_id GRMZM2G330302; transcript_id TCONS_00020538; exon_number 10; oId CUFF.17752.1; tss_id TSS18605;
3	Cufflinks	exon	213472051	213472097	.	-	.	gene_id GRMZM2G330302; transcript_id TCONS_00020538; exon_number 11; oId CUFF.17752.1; tss_id TSS18605;
3	Cufflinks	exon	213472186	213472248	.	-	.	gene_id GRMZM2G330302; transcript_id TCONS_00020538; exon_number 12; oId CUFF.17752.1; tss_id TSS18605;

3	Cufflinks	exon	213472333	213472383	.	-	.	gene_id GRMZM2G330302; transcript_id TCONS_00020538; exon_number 13; oId CUFF.17752.1; tss_id TSS18605;
3	Cufflinks	exon	213472501	213472584	.	-	.	gene_id GRMZM2G330302; transcript_id TCONS_00020538; exon_number 14; oId CUFF.17752.1; tss_id TSS18605;
3	Cufflinks	exon	213472702	213472766	.	-	.	gene_id GRMZM2G330302; transcript_id TCONS_00020538; exon_number 15; oId CUFF.17752.1; tss_id TSS18605;
3	Cufflinks	exon	213472868	213472972	.	-	.	gene_id GRMZM2G330302; transcript_id TCONS_00020538; exon_number 16; oId CUFF.17752.1; tss_id TSS18605;
3	Cufflinks	exon	213473127	213473244	.	-	.	gene_id GRMZM2G330302; transcript_id TCONS_00020538; exon_number 17; oId CUFF.17752.1; tss_id TSS18605;
3	Cufflinks	exon	213473344	213473394	.	-	.	gene_id GRMZM2G330302; transcript_id TCONS_00020538; exon_number 18; oId CUFF.17752.1; tss_id TSS18605;
3	Cufflinks	exon	213474137	213474223	.	-	.	gene_id GRMZM2G330302; transcript_id TCONS_00020538; exon_number 19; oId CUFF.17752.1; tss_id TSS18605;
3	Cufflinks	exon	213474428	213474507	.	-	.	gene_id GRMZM2G330302; transcript_id TCONS_00020538; exon_number 20; oId CUFF.17752.1; tss_id TSS18605;
3	Cufflinks	exon	213475294	213475662	.	-	.	gene_id GRMZM2G330302; transcript_id TCONS_00020538; exon_number 21; oId CUFF.17752.1; tss_id TSS18605;
3	Cufflinks	exon	220604720	220604988	.	-	.	gene_id GRMZM2G116584; transcript_id TCONS_00020653; exon_number 1; oId CUFF.17930.1; tss_id TSS18704;
3	Cufflinks	exon	220605262	220605520	.	-	.	gene_id GRMZM2G116584; transcript_id TCONS_00020653; exon_number 2; oId CUFF.17930.1; tss_id TSS18704;
3	Cufflinks	exon	220605705	220605849	.	-	.	gene_id GRMZM2G116584; transcript_id TCONS_00020653; exon_number 3; oId CUFF.17930.1; tss_id TSS18704;
3	Cufflinks	exon	220605937	220606017	.	-	.	gene_id GRMZM2G116584; transcript_id TCONS_00020653; exon_number 4; oId CUFF.17930.1; tss_id TSS18704;
3	Cufflinks	exon	220606109	220606161	.	-	.	gene_id GRMZM2G116584; transcript_id TCONS_00020653; exon_number 5; oId CUFF.17930.1; tss_id TSS18704;
3	Cufflinks	exon	220606263	220606371	.	-	.	gene_id GRMZM2G116584; transcript_id TCONS_00020653; exon_number 6; oId CUFF.17930.1; tss_id TSS18704;
3	Cufflinks	exon	220606546	220606605	.	-	.	gene_id GRMZM2G116584; transcript_id TCONS_00020653; exon_number 7; oId CUFF.17930.1; tss_id TSS18704;
3	Cufflinks	exon	220607729	220607811	.	-	.	gene_id GRMZM2G116584; transcript_id TCONS_00020653; exon_number 8; oId CUFF.17930.1; tss_id TSS18704;
3	Cufflinks	exon	220607932	220608229	.	-	.	gene_id GRMZM2G116584; transcript_id TCONS_00020653; exon_number 9; oId CUFF.17930.1; tss_id TSS18704;
3	Cufflinks	exon	220608354	220608430	.	-	.	gene_id GRMZM2G116584; transcript_id TCONS_00020653; exon_number 10; oId CUFF.17930.1; tss_id TSS18704;
3	Cufflinks	exon	229070754	229071665	.	-	.	gene_id GRMZM2G344924; transcript_id TCONS_00020802; exon_number 1; oId CUFF.18187.1; tss_id TSS18842;
3	Cufflinks	exon	229071759	229071936	.	-	.	gene_id GRMZM2G344924; transcript_id TCONS_00020802; exon_number 2; oId CUFF.18187.1; tss_id TSS18842;
3	Cufflinks	exon	229072081	229072194	.	-	.	gene_id GRMZM2G344924; transcript_id TCONS_00020802; exon_number 3; oId CUFF.18187.1; tss_id TSS18842;
3	Cufflinks	exon	229072367	229072505	.	-	.	gene_id GRMZM2G344924; transcript_id TCONS_00020802; exon_number 4; oId CUFF.18187.1; tss_id TSS18842;
3	Cufflinks	exon	229072848	229072908	.	-	.	gene_id GRMZM2G344924; transcript_id TCONS_00020802; exon_number 5; oId CUFF.18187.1; tss_id TSS18842;
3	Cufflinks	exon	229073014	229073083	.	-	.	gene_id GRMZM2G344924; transcript_id TCONS_00020802; exon_number 6; oId CUFF.18187.1; tss_id TSS18842;
3	Cufflinks	exon	229073183	229073691	.	-	.	gene_id GRMZM2G344924; transcript_id TCONS_00020802; exon_number 7; oId CUFF.18187.1; tss_id TSS18842;
3	Cufflinks	exon	229073968	229074183	.	-	.	gene_id GRMZM2G344924; transcript_id TCONS_00020802; exon_number 8; oId CUFF.18187.1; tss_id TSS18842;
3	Cufflinks	exon	229074412	229074906	.	-	.	gene_id GRMZM2G344924; transcript_id TCONS_00020802; exon_number 9; oId CUFF.18187.1; tss_id TSS18842;
3	Cufflinks	exon	229075255	229075602	.	-	.	gene_id GRMZM2G344924; transcript_id TCONS_00020802; exon_number 10; oId CUFF.18187.1; tss_id TSS18842;
3	Cufflinks	exon	229076118	229076299	.	-	.	gene_id GRMZM2G344924; transcript_id TCONS_00020802; exon_number 11; oId CUFF.18187.1; tss_id TSS18842;
3	Cufflinks	exon	229218227	229218934	.	-	.	gene_id XLOC_018225; transcript_id TCONS_00020806; exon_number 1; oId CUFF.18237.1; tss_id TSS18846;
3	Cufflinks	exon	229640519	229641139	.	-	.	gene_id XLOC_018233; transcript_id TCONS_00020815; exon_number 1; oId CUFF.18205.1; tss_id TSS18855;
3	Cufflinks	exon	230083958	230084293	.	-	.	gene_id GRMZM2G005834; transcript_id TCONS_00020828; exon_number 1; oId CUFF.18229.1; tss_id TSS18866;
3	Cufflinks	exon	230084690	230085191	.	-	.	gene_id GRMZM2G005834; transcript_id TCONS_00020828; exon_number 2; oId CUFF.18229.1; tss_id TSS18866;
3	Cufflinks	exon	230085746	230086240	.	-	.	gene_id GRMZM2G005834; transcript_id TCONS_00020828; exon_number 3; oId CUFF.18229.1; tss_id TSS18866;
3	Cufflinks	exon	230523385	230523682	.	-	.	gene_id XLOC_018251; transcript_id TCONS_00020837; exon_number 1; oId CUFF.18251.1; tss_id TSS18875;

3	Cufflinks	exon	231198433	231199552	.	-	.	gene_id GRMZM2G092008; transcript_id TCONS_00020854; exon_number 1; oId CUFF.18303.1; tss_id TSS18892;
3	Cufflinks	exon	231199635	231199675	.	-	.	gene_id GRMZM2G092008; transcript_id TCONS_00020854; exon_number 2; oId CUFF.18303.1; tss_id TSS18892;
3	Cufflinks	exon	231199824	231199885	.	-	.	gene_id GRMZM2G092008; transcript_id TCONS_00020854; exon_number 3; oId CUFF.18303.1; tss_id TSS18892;
3	Cufflinks	exon	231199968	231200286	.	-	.	gene_id GRMZM2G092008; transcript_id TCONS_00020854; exon_number 4; oId CUFF.18303.1; tss_id TSS18892;
4	Cufflinks	exon	2611736	2612238	.	+	.	gene_id XLOC_018327; transcript_id TCONS_00020919; exon_number 1; oId CUFF.18394.1; tss_id TSS18952;
4	Cufflinks	exon	2755561	2755793	.	+	.	gene_id GRMZM2G126453; transcript_id TCONS_00020925; exon_number 1; oId CUFF.18435.1; tss_id TSS18957;
4	Cufflinks	exon	2755886	2756009	.	+	.	gene_id GRMZM2G126453; transcript_id TCONS_00020925; exon_number 2; oId CUFF.18435.1; tss_id TSS18957;
4	Cufflinks	exon	2756101	2756216	.	+	.	gene_id GRMZM2G126453; transcript_id TCONS_00020925; exon_number 3; oId CUFF.18435.1; tss_id TSS18957;
4	Cufflinks	exon	2758034	2758150	.	+	.	gene_id GRMZM2G126453; transcript_id TCONS_00020925; exon_number 4; oId CUFF.18435.1; tss_id TSS18957;
4	Cufflinks	exon	2758262	2758402	.	+	.	gene_id GRMZM2G126453; transcript_id TCONS_00020925; exon_number 5; oId CUFF.18435.1; tss_id TSS18957;
4	Cufflinks	exon	2758535	2758976	.	+	.	gene_id GRMZM2G126453; transcript_id TCONS_00020925; exon_number 6; oId CUFF.18435.1; tss_id TSS18957;
4	Cufflinks	exon	2759073	2759229	.	+	.	gene_id GRMZM2G126453; transcript_id TCONS_00020925; exon_number 7; oId CUFF.18435.1; tss_id TSS18957;
4	Cufflinks	exon	2759575	2759696	.	+	.	gene_id GRMZM2G126453; transcript_id TCONS_00020925; exon_number 8; oId CUFF.18435.1; tss_id TSS18957;
4	Cufflinks	exon	2759892	2759983	.	+	.	gene_id GRMZM2G126453; transcript_id TCONS_00020925; exon_number 9; oId CUFF.18435.1; tss_id TSS18957;
4	Cufflinks	exon	2760097	2760352	.	+	.	gene_id GRMZM2G126453; transcript_id TCONS_00020925; exon_number 10; oId CUFF.18435.1; tss_id TSS18957;
4	Cufflinks	exon	2760428	2760479	.	+	.	gene_id GRMZM2G126453; transcript_id TCONS_00020925; exon_number 11; oId CUFF.18435.1; tss_id TSS18957;
4	Cufflinks	exon	2761510	2761658	.	+	.	gene_id GRMZM2G126453; transcript_id TCONS_00020925; exon_number 12; oId CUFF.18435.1; tss_id TSS18957;
4	Cufflinks	exon	2761744	2761814	.	+	.	gene_id GRMZM2G126453; transcript_id TCONS_00020925; exon_number 13; oId CUFF.18435.1; tss_id TSS18957;
4	Cufflinks	exon	2761898	2762071	.	+	.	gene_id GRMZM2G126453; transcript_id TCONS_00020925; exon_number 14; oId CUFF.18435.1; tss_id TSS18957;
4	Cufflinks	exon	2762298	2762366	.	+	.	gene_id GRMZM2G126453; transcript_id TCONS_00020925; exon_number 15; oId CUFF.18435.1; tss_id TSS18957;
4	Cufflinks	exon	2762509	2762613	.	+	.	gene_id GRMZM2G126453; transcript_id TCONS_00020925; exon_number 16; oId CUFF.18435.1; tss_id TSS18957;
4	Cufflinks	exon	2762688	2762777	.	+	.	gene_id GRMZM2G126453; transcript_id TCONS_00020925; exon_number 17; oId CUFF.18435.1; tss_id TSS18957;
4	Cufflinks	exon	2763351	2763426	.	+	.	gene_id GRMZM2G126453; transcript_id TCONS_00020925; exon_number 18; oId CUFF.18435.1; tss_id TSS18957;
4	Cufflinks	exon	2763506	2763602	.	+	.	gene_id GRMZM2G126453; transcript_id TCONS_00020925; exon_number 19; oId CUFF.18435.1; tss_id TSS18957;
4	Cufflinks	exon	2763691	2763760	.	+	.	gene_id GRMZM2G126453; transcript_id TCONS_00020925; exon_number 20; oId CUFF.18435.1; tss_id TSS18957;
4	Cufflinks	exon	2763845	2764024	.	+	.	gene_id GRMZM2G126453; transcript_id TCONS_00020925; exon_number 21; oId CUFF.18435.1; tss_id TSS18957;
4	Cufflinks	exon	2764617	2764739	.	+	.	gene_id GRMZM2G126453; transcript_id TCONS_00020925; exon_number 22; oId CUFF.18435.1; tss_id TSS18957;
4	Cufflinks	exon	2764816	2764899	.	+	.	gene_id GRMZM2G126453; transcript_id TCONS_00020925; exon_number 23; oId CUFF.18435.1; tss_id TSS18957;
4	Cufflinks	exon	2764977	2765161	.	+	.	gene_id GRMZM2G126453; transcript_id TCONS_00020925; exon_number 24; oId CUFF.18435.1; tss_id TSS18957;
4	Cufflinks	exon	2765305	2765443	.	+	.	gene_id GRMZM2G126453; transcript_id TCONS_00020925; exon_number 25; oId CUFF.18435.1; tss_id TSS18957;
4	Cufflinks	exon	2765517	2765576	.	+	.	gene_id GRMZM2G126453; transcript_id TCONS_00020925; exon_number 26; oId CUFF.18435.1; tss_id TSS18957;
4	Cufflinks	exon	2765732	2765836	.	+	.	gene_id GRMZM2G126453; transcript_id TCONS_00020925; exon_number 27; oId CUFF.18435.1; tss_id TSS18957;
4	Cufflinks	exon	2765973	2766689	.	+	.	gene_id GRMZM2G126453; transcript_id TCONS_00020925; exon_number 28; oId CUFF.18435.1; tss_id TSS18957;
4	Cufflinks	exon	3488028	3488607	.	+	.	gene_id XLOC_018349; transcript_id TCONS_00020944; exon_number 1; oId CUFF.18438.1; tss_id TSS18974;
4	Cufflinks	exon	3489161	3489663	.	+	.	gene_id XLOC_018349; transcript_id TCONS_00020944; exon_number 2; oId CUFF.18438.1; tss_id TSS18974;
4	Cufflinks	exon	5006764	5007111	.	+	.	gene_id XLOC_018393; transcript_id TCONS_00020996; exon_number 1; oId CUFF.18501.1; tss_id TSS19020;

4	Cufflinks	exon	17257026	17257894	.	+	.	gene_id GRMZM2G080168; transcript_id TCONS_00021149; exon_number 1; oId CUFF.18739.1; tss_id TSS19156;
4	Cufflinks	exon	17257987	17258187	.	+	.	gene_id GRMZM2G080168; transcript_id TCONS_00021149; exon_number 2; oId CUFF.18739.1; tss_id TSS19156;
4	Cufflinks	exon	17258284	17258349	.	+	.	gene_id GRMZM2G080168; transcript_id TCONS_00021149; exon_number 3; oId CUFF.18739.1; tss_id TSS19156;
4	Cufflinks	exon	17258434	17258502	.	+	.	gene_id GRMZM2G080168; transcript_id TCONS_00021149; exon_number 4; oId CUFF.18739.1; tss_id TSS19156;
4	Cufflinks	exon	17258613	17258690	.	+	.	gene_id GRMZM2G080168; transcript_id TCONS_00021149; exon_number 5; oId CUFF.18739.1; tss_id TSS19156;
4	Cufflinks	exon	17258819	17259097	.	+	.	gene_id GRMZM2G080168; transcript_id TCONS_00021149; exon_number 6; oId CUFF.18739.1; tss_id TSS19156;
4	Cufflinks	exon	17259240	17259706	.	+	.	gene_id GRMZM2G080168; transcript_id TCONS_00021149; exon_number 7; oId CUFF.18739.1; tss_id TSS19156;
4	Cufflinks	exon	31271410	31272316	.	+	.	gene_id GRMZM2G122277; transcript_id TCONS_00021295; exon_number 1; oId CUFF.19004.1; tss_id TSS19288;
4	Cufflinks	exon	31274427	31275171	.	+	.	gene_id GRMZM2G122277; transcript_id TCONS_00021295; exon_number 2; oId CUFF.19004.1; tss_id TSS19288;
4	Cufflinks	exon	31275845	31278209	.	+	.	gene_id GRMZM2G122277; transcript_id TCONS_00021295; exon_number 3; oId CUFF.19004.1; tss_id TSS19288;
4	Cufflinks	exon	36017140	36017520	.	+	.	gene_id GRMZM2G074280; transcript_id TCONS_00021340; exon_number 1; oId CUFF.19057.1; tss_id TSS19328;
4	Cufflinks	exon	36018164	36018235	.	+	.	gene_id GRMZM2G074280; transcript_id TCONS_00021340; exon_number 2; oId CUFF.19057.1; tss_id TSS19328;
4	Cufflinks	exon	36018333	36018384	.	+	.	gene_id GRMZM2G074280; transcript_id TCONS_00021340; exon_number 3; oId CUFF.19057.1; tss_id TSS19328;
4	Cufflinks	exon	36018456	36018542	.	+	.	gene_id GRMZM2G074280; transcript_id TCONS_00021340; exon_number 4; oId CUFF.19057.1; tss_id TSS19328;
4	Cufflinks	exon	36018720	36020195	.	+	.	gene_id GRMZM2G074280; transcript_id TCONS_00021340; exon_number 5; oId CUFF.19057.1; tss_id TSS19328;
4	Cufflinks	exon	38788778	38789052	.	+	.	gene_id GRMZM2G105438; transcript_id TCONS_00021386; exon_number 1; oId CUFF.19133.1; tss_id TSS19369;
4	Cufflinks	exon	38789135	38789237	.	+	.	gene_id GRMZM2G105438; transcript_id TCONS_00021386; exon_number 2; oId CUFF.19133.1; tss_id TSS19369;
4	Cufflinks	exon	38789357	38789500	.	+	.	gene_id GRMZM2G105438; transcript_id TCONS_00021386; exon_number 3; oId CUFF.19133.1; tss_id TSS19369;
4	Cufflinks	exon	38790475	38791422	.	+	.	gene_id GRMZM2G105438; transcript_id TCONS_00021386; exon_number 4; oId CUFF.19133.1; tss_id TSS19369;
4	Cufflinks	exon	40005741	40005959	.	+	.	gene_id GRMZM2G056573; transcript_id TCONS_00021404; exon_number 1; oId CUFF.19162.1; tss_id TSS19380;
4	Cufflinks	exon	40006153	40006211	.	+	.	gene_id GRMZM2G056573; transcript_id TCONS_00021404; exon_number 2; oId CUFF.19162.1; tss_id TSS19380;
4	Cufflinks	exon	40008956	40009103	.	+	.	gene_id GRMZM2G056573; transcript_id TCONS_00021404; exon_number 3; oId CUFF.19162.1; tss_id TSS19380;
4	Cufflinks	exon	40009192	40009265	.	+	.	gene_id GRMZM2G056573; transcript_id TCONS_00021404; exon_number 4; oId CUFF.19162.1; tss_id TSS19380;
4	Cufflinks	exon	40009359	40009853	.	+	.	gene_id GRMZM2G056573; transcript_id TCONS_00021404; exon_number 5; oId CUFF.19162.1; tss_id TSS19380;
4	Cufflinks	exon	40010613	40011077	.	+	.	gene_id GRMZM2G056573; transcript_id TCONS_00021404; exon_number 6; oId CUFF.19162.1; tss_id TSS19380;
4	Cufflinks	exon	40011157	40011369	.	+	.	gene_id GRMZM2G056573; transcript_id TCONS_00021404; exon_number 7; oId CUFF.19162.1; tss_id TSS19380;
4	Cufflinks	exon	40011538	40012885	.	+	.	gene_id GRMZM2G056573; transcript_id TCONS_00021404; exon_number 8; oId CUFF.19162.1; tss_id TSS19380;
4	Cufflinks	exon	46961515	46962122	.	+	.	gene_id GRMZM2G100583; transcript_id TCONS_00021450; exon_number 1; oId CUFF.19258.1; tss_id TSS19423;
4	Cufflinks	exon	46962256	46962536	.	+	.	gene_id GRMZM2G100583; transcript_id TCONS_00021450; exon_number 2; oId CUFF.19258.1; tss_id TSS19423;
4	Cufflinks	exon	46963595	46966149	.	+	.	gene_id GRMZM2G100583; transcript_id TCONS_00021450; exon_number 3; oId CUFF.19258.1; tss_id TSS19423;
4	Cufflinks	exon	63028898	63030916	.	+	.	gene_id GRMZM5G884985; transcript_id TCONS_00021542; exon_number 1; oId CUFF.19420.1; tss_id TSS19503;
4	Cufflinks	exon	63562639	63563315	.	+	.	gene_id XLOC_018862; transcript_id TCONS_00021545; exon_number 1; oId CUFF.19431.1; tss_id TSS19506;
4	Cufflinks	exon	67045579	67046699	.	+	.	gene_id GRMZM2G352132; transcript_id TCONS_00021579; exon_number 1; oId CUFF.19491.1; tss_id TSS19536;
4	Cufflinks	exon	67046817	67046858	.	+	.	gene_id GRMZM2G352132; transcript_id TCONS_00021579; exon_number 2; oId CUFF.19491.1; tss_id TSS19536;
4	Cufflinks	exon	67046969	67047040	.	+	.	gene_id GRMZM2G352132; transcript_id TCONS_00021579; exon_number 3; oId CUFF.19491.1; tss_id TSS19536;
4	Cufflinks	exon	67047158	67047229	.	+	.	gene_id GRMZM2G352132; transcript_id TCONS_00021579; exon_number 4; oId CUFF.19491.1; tss_id TSS19536;

4	Cufflinks	exon	67047304	67047327	.	+	.	gene_id GRMZM2G352132; transcript_id TCONS_00021579; exon_number 5; oId CUFF.19491.1; tss_id TSS19536;
4	Cufflinks	exon	67047349	67047372	.	+	.	gene_id GRMZM2G352132; transcript_id TCONS_00021579; exon_number 6; oId CUFF.19491.1; tss_id TSS19536;
4	Cufflinks	exon	67047498	67047566	.	+	.	gene_id GRMZM2G352132; transcript_id TCONS_00021579; exon_number 7; oId CUFF.19491.1; tss_id TSS19536;
4	Cufflinks	exon	67047646	67047717	.	+	.	gene_id GRMZM2G352132; transcript_id TCONS_00021579; exon_number 8; oId CUFF.19491.1; tss_id TSS19536;
4	Cufflinks	exon	67047819	67047921	.	+	.	gene_id GRMZM2G352132; transcript_id TCONS_00021579; exon_number 9; oId CUFF.19491.1; tss_id TSS19536;
4	Cufflinks	exon	67048020	67048094	.	+	.	gene_id GRMZM2G352132; transcript_id TCONS_00021579; exon_number 10; oId CUFF.19491.1; tss_id TSS19536;
4	Cufflinks	exon	67048188	67048359	.	+	.	gene_id GRMZM2G352132; transcript_id TCONS_00021579; exon_number 11; oId CUFF.19491.1; tss_id TSS19536;
4	Cufflinks	exon	67048533	67048602	.	+	.	gene_id GRMZM2G352132; transcript_id TCONS_00021579; exon_number 12; oId CUFF.19491.1; tss_id TSS19536;
4	Cufflinks	exon	67048920	67048985	.	+	.	gene_id GRMZM2G352132; transcript_id TCONS_00021579; exon_number 13; oId CUFF.19491.1; tss_id TSS19536;
4	Cufflinks	exon	67052089	67052872	.	+	.	gene_id GRMZM2G352132; transcript_id TCONS_00021579; exon_number 14; oId CUFF.19491.1; tss_id TSS19536;
4	Cufflinks	exon	67677536	67677715	.	+	.	gene_id XLOC_018894; transcript_id TCONS_00021584; exon_number 1; oId CUFF.19481.1; tss_id TSS19541;
4	Cufflinks	exon	67677918	67679076	.	+	.	gene_id XLOC_018894; transcript_id TCONS_00021584; exon_number 2; oId CUFF.19481.1; tss_id TSS19541;
4	Cufflinks	exon	71935082	71936234	.	+	.	gene_id GRMZM2G174732; transcript_id TCONS_00021616; exon_number 1; oId CUFF.19544.1; tss_id TSS19573;
4	Cufflinks	exon	71944421	71944619	.	+	.	gene_id GRMZM2G174732; transcript_id TCONS_00021616; exon_number 2; oId CUFF.19544.1; tss_id TSS19573;
4	Cufflinks	exon	71945088	71945301	.	+	.	gene_id GRMZM2G174732; transcript_id TCONS_00021616; exon_number 3; oId CUFF.19544.1; tss_id TSS19573;
4	Cufflinks	exon	71945478	71945592	.	+	.	gene_id GRMZM2G174732; transcript_id TCONS_00021616; exon_number 4; oId CUFF.19544.1; tss_id TSS19573;
4	Cufflinks	exon	71945674	71945964	.	+	.	gene_id GRMZM2G174732; transcript_id TCONS_00021616; exon_number 5; oId CUFF.19544.1; tss_id TSS19573;
4	Cufflinks	exon	125797420	125797602	.	+	.	gene_id GRMZM5G898290; transcript_id TCONS_00021892; exon_number 1; oId CUFF.19995.1; tss_id TSS19817;
4	Cufflinks	exon	125797739	125797988	.	+	.	gene_id GRMZM5G898290; transcript_id TCONS_00021892; exon_number 2; oId CUFF.19995.1; tss_id TSS19817;
4	Cufflinks	exon	125798125	125798423	.	+	.	gene_id GRMZM5G898290; transcript_id TCONS_00021892; exon_number 3; oId CUFF.19995.1; tss_id TSS19817;
4	Cufflinks	exon	125798572	125799414	.	+	.	gene_id GRMZM5G898290; transcript_id TCONS_00021892; exon_number 4; oId CUFF.19995.1; tss_id TSS19817;
4	Cufflinks	exon	143491887	143492100	.	+	.	gene_id XLOC_019252; transcript_id TCONS_00021992; exon_number 1; oId CUFF.20186.1; tss_id TSS19911;
4	Cufflinks	exon	143492226	143494313	.	+	.	gene_id XLOC_019252; transcript_id TCONS_00021992; exon_number 2; oId CUFF.20186.1; tss_id TSS19911;
4	Cufflinks	exon	151675886	151676077	.	+	.	gene_id GRMZM2G145226; transcript_id TCONS_00022056; exon_number 1; oId CUFF.20301.1; tss_id TSS19968;
4	Cufflinks	exon	151676183	151676314	.	+	.	gene_id GRMZM2G145226; transcript_id TCONS_00022056; exon_number 2; oId CUFF.20301.1; tss_id TSS19968;
4	Cufflinks	exon	151676435	151676491	.	+	.	gene_id GRMZM2G145226; transcript_id TCONS_00022056; exon_number 3; oId CUFF.20301.1; tss_id TSS19968;
4	Cufflinks	exon	151677649	151677754	.	+	.	gene_id GRMZM2G145226; transcript_id TCONS_00022056; exon_number 4; oId CUFF.20301.1; tss_id TSS19968;
4	Cufflinks	exon	151678437	151678495	.	+	.	gene_id GRMZM2G145226; transcript_id TCONS_00022056; exon_number 5; oId CUFF.20301.1; tss_id TSS19968;
4	Cufflinks	exon	151678585	151678659	.	+	.	gene_id GRMZM2G145226; transcript_id TCONS_00022056; exon_number 6; oId CUFF.20301.1; tss_id TSS19968;
4	Cufflinks	exon	151679027	151679075	.	+	.	gene_id GRMZM2G145226; transcript_id TCONS_00022056; exon_number 7; oId CUFF.20301.1; tss_id TSS19968;
4	Cufflinks	exon	151679185	151679261	.	+	.	gene_id GRMZM2G145226; transcript_id TCONS_00022056; exon_number 8; oId CUFF.20301.1; tss_id TSS19968;
4	Cufflinks	exon	151681923	151682478	.	+	.	gene_id GRMZM2G145226; transcript_id TCONS_00022056; exon_number 9; oId CUFF.20301.1; tss_id TSS19968;
4	Cufflinks	exon	156439025	156439617	.	+	.	gene_id GRMZM2G528010; transcript_id TCONS_00022118; exon_number 1; oId CUFF.20378.1; tss_id TSS20026;
4	Cufflinks	exon	156441936	156442160	.	+	.	gene_id GRMZM2G528010; transcript_id TCONS_00022118; exon_number 2; oId CUFF.20378.1; tss_id TSS20026;
4	Cufflinks	exon	156442254	156442607	.	+	.	gene_id GRMZM2G528010; transcript_id TCONS_00022118; exon_number 3; oId CUFF.20378.1; tss_id TSS20026;
4	Cufflinks	exon	156443699	156444649	.	+	.	gene_id GRMZM2G528010; transcript_id TCONS_00022118; exon_number 4; oId CUFF.20378.1; tss_id TSS20026;

4	Cufflinks	exon	158364982	158365388	.	+	.	gene_id GRMZM2G066615; transcript_id TCONS_00022153; exon_number 1; oId CUFF.20433.1; tss_id TSS20060;
4	Cufflinks	exon	158365483	158365578	.	+	.	gene_id GRMZM2G066615; transcript_id TCONS_00022153; exon_number 2; oId CUFF.20433.1; tss_id TSS20060;
4	Cufflinks	exon	158369112	158369532	.	+	.	gene_id GRMZM2G066615; transcript_id TCONS_00022153; exon_number 3; oId CUFF.20433.1; tss_id TSS20060;
4	Cufflinks	exon	158369843	158369933	.	+	.	gene_id GRMZM2G066615; transcript_id TCONS_00022153; exon_number 4; oId CUFF.20433.1; tss_id TSS20060;
4	Cufflinks	exon	158370436	158370772	.	+	.	gene_id GRMZM2G066615; transcript_id TCONS_00022153; exon_number 5; oId CUFF.20433.1; tss_id TSS20060;
4	Cufflinks	exon	158370863	158371011	.	+	.	gene_id GRMZM2G066615; transcript_id TCONS_00022153; exon_number 6; oId CUFF.20433.1; tss_id TSS20060;
4	Cufflinks	exon	158371140	158372319	.	+	.	gene_id GRMZM2G066615; transcript_id TCONS_00022153; exon_number 7; oId CUFF.20433.1; tss_id TSS20060;
4	Cufflinks	exon	160982522	160983293	.	+	.	gene_id GRMZM2G342515; transcript_id TCONS_00022185; exon_number 1; oId CUFF.20494.1; tss_id TSS20089;
4	Cufflinks	exon	163674452	163675848	.	+	.	gene_id GRMZM2G309950; transcript_id TCONS_00022225; exon_number 1; oId CUFF.20574.1; tss_id TSS20125;
4	Cufflinks	exon	165861729	165862472	.	+	.	gene_id GRMZM2G021339; transcript_id TCONS_00022244; exon_number 1; oId CUFF.20614.1; tss_id TSS20143;
4	Cufflinks	exon	165862577	165862713	.	+	.	gene_id GRMZM2G021339; transcript_id TCONS_00022244; exon_number 2; oId CUFF.20614.1; tss_id TSS20143;
4	Cufflinks	exon	165862828	165863067	.	+	.	gene_id GRMZM2G021339; transcript_id TCONS_00022244; exon_number 3; oId CUFF.20614.1; tss_id TSS20143;
4	Cufflinks	exon	165863199	165864137	.	+	.	gene_id GRMZM2G021339; transcript_id TCONS_00022244; exon_number 4; oId CUFF.20614.1; tss_id TSS20143;
4	Cufflinks	exon	166895031	166895149	.	+	.	gene_id GRMZM2G471733; transcript_id TCONS_00022253; exon_number 1; oId CUFF.20629.1; tss_id TSS20152;
4	Cufflinks	exon	166895230	166895685	.	+	.	gene_id GRMZM2G471733; transcript_id TCONS_00022253; exon_number 2; oId CUFF.20629.1; tss_id TSS20152;
4	Cufflinks	exon	166895777	166897669	.	+	.	gene_id GRMZM2G471733; transcript_id TCONS_00022253; exon_number 3; oId CUFF.20629.1; tss_id TSS20152;
4	Cufflinks	exon	168574502	168574622	.	+	.	gene_id GRMZM2G017852; transcript_id TCONS_00022276; exon_number 1; oId CUFF.20681.1; tss_id TSS20172;
4	Cufflinks	exon	168574700	168575263	.	+	.	gene_id GRMZM2G017852; transcript_id TCONS_00022276; exon_number 2; oId CUFF.20681.1; tss_id TSS20172;
4	Cufflinks	exon	168575352	168577580	.	+	.	gene_id GRMZM2G017852; transcript_id TCONS_00022276; exon_number 3; oId CUFF.20681.1; tss_id TSS20172;
4	Cufflinks	exon	169987963	169988210	.	+	.	gene_id GRMZM2G082222; transcript_id TCONS_00022295; exon_number 1; oId CUFF.20697.1; tss_id TSS20186;
4	Cufflinks	exon	169988297	169988374	.	+	.	gene_id GRMZM2G082222; transcript_id TCONS_00022295; exon_number 2; oId CUFF.20697.1; tss_id TSS20186;
4	Cufflinks	exon	169989717	169989812	.	+	.	gene_id GRMZM2G082222; transcript_id TCONS_00022295; exon_number 3; oId CUFF.20697.1; tss_id TSS20186;
4	Cufflinks	exon	169989885	169990290	.	+	.	gene_id GRMZM2G082222; transcript_id TCONS_00022295; exon_number 4; oId CUFF.20697.1; tss_id TSS20186;
4	Cufflinks	exon	172777612	172777959	.	+	.	gene_id GRMZM2G178398; transcript_id TCONS_00022350; exon_number 1; oId CUFF.20797.1; tss_id TSS20237;
4	Cufflinks	exon	172778357	172778460	.	+	.	gene_id GRMZM2G178398; transcript_id TCONS_00022350; exon_number 2; oId CUFF.20797.1; tss_id TSS20237;
4	Cufflinks	exon	172778584	172778635	.	+	.	gene_id GRMZM2G178398; transcript_id TCONS_00022350; exon_number 3; oId CUFF.20797.1; tss_id TSS20237;
4	Cufflinks	exon	172778783	172778862	.	+	.	gene_id GRMZM2G178398; transcript_id TCONS_00022350; exon_number 4; oId CUFF.20797.1; tss_id TSS20237;
4	Cufflinks	exon	172778957	172779035	.	+	.	gene_id GRMZM2G178398; transcript_id TCONS_00022350; exon_number 5; oId CUFF.20797.1; tss_id TSS20237;
4	Cufflinks	exon	172779158	172779241	.	+	.	gene_id GRMZM2G178398; transcript_id TCONS_00022350; exon_number 6; oId CUFF.20797.1; tss_id TSS20237;
4	Cufflinks	exon	172779364	172779509	.	+	.	gene_id GRMZM2G178398; transcript_id TCONS_00022350; exon_number 7; oId CUFF.20797.1; tss_id TSS20237;
4	Cufflinks	exon	172779620	172779967	.	+	.	gene_id GRMZM2G178398; transcript_id TCONS_00022350; exon_number 8; oId CUFF.20797.1; tss_id TSS20237;
4	Cufflinks	exon	172780122	172780163	.	+	.	gene_id GRMZM2G178398; transcript_id TCONS_00022350; exon_number 9; oId CUFF.20797.1; tss_id TSS20237;
4	Cufflinks	exon	172780251	172780349	.	+	.	gene_id GRMZM2G178398; transcript_id TCONS_00022350; exon_number 10; oId CUFF.20797.1; tss_id TSS20237;
4	Cufflinks	exon	172780432	172780616	.	+	.	gene_id GRMZM2G178398; transcript_id TCONS_00022350; exon_number 11; oId CUFF.20797.1; tss_id TSS20237;
4	Cufflinks	exon	172780823	172781022	.	+	.	gene_id GRMZM2G178398; transcript_id TCONS_00022350; exon_number 12; oId CUFF.20797.1; tss_id TSS20237;
4	Cufflinks	exon	172781772	172782729	.	+	.	gene_id GRMZM2G178398; transcript_id TCONS_00022350; exon_number 13; oId CUFF.20797.1; tss_id TSS20237;

4	Cufflinks	exon	172782823	172783745	.	+	.	gene_id GRMZM2G178398; transcript_id TCONS_00022350; exon_number 14; oId CUFF.20797.1; tss_id TSS20237;
4	Cufflinks	exon	175361055	175361851	.	+	.	gene_id GRMZM2G314946; transcript_id TCONS_00022390; exon_number 1; oId CUFF.20854.1; tss_id TSS20273;
4	Cufflinks	exon	178702590	178703293	.	+	.	gene_id GRMZM2G015983; transcript_id TCONS_00022443; exon_number 1; oId CUFF.20956.1; tss_id TSS20323;
4	Cufflinks	exon	178704523	178706283	.	+	.	gene_id GRMZM2G015983; transcript_id TCONS_00022443; exon_number 2; oId CUFF.20956.1; tss_id TSS20323;
4	Cufflinks	exon	180401155	180403087	.	+	.	gene_id XLOC_019669; transcript_id TCONS_00022459; exon_number 1; oId CUFF.20992.1; tss_id TSS20338;
4	Cufflinks	exon	180463493	180463570	.	+	.	gene_id GRMZM2G470524; transcript_id TCONS_00022462; exon_number 1; oId CUFF.21042.1; tss_id TSS20341;
4	Cufflinks	exon	180463700	180465143	.	+	.	gene_id GRMZM2G470524; transcript_id TCONS_00022462; exon_number 2; oId CUFF.21042.1; tss_id TSS20341;
4	Cufflinks	exon	181560393	181560782	.	+	.	gene_id GRMZM2G169033; transcript_id TCONS_00022475; exon_number 1; oId CUFF.21025.1; tss_id TSS20353;
4	Cufflinks	exon	181561726	181563214	.	+	.	gene_id GRMZM2G169033; transcript_id TCONS_00022475; exon_number 2; oId CUFF.21025.1; tss_id TSS20353;
4	Cufflinks	exon	181563343	181563791	.	+	.	gene_id GRMZM2G169033; transcript_id TCONS_00022475; exon_number 3; oId CUFF.21025.1; tss_id TSS20353;
4	Cufflinks	exon	181903993	181906445	.	+	.	gene_id GRMZM2G041809; transcript_id TCONS_00022483; exon_number 1; oId CUFF.21035.1; tss_id TSS20361;
4	Cufflinks	exon	181918746	181919655	.	+	.	gene_id GRMZM2G041959; transcript_id TCONS_00022486; exon_number 1; oId CUFF.21049.3; tss_id TSS20364;
4	Cufflinks	exon	181921071	181922939	.	+	.	gene_id GRMZM2G041959; transcript_id TCONS_00022486; exon_number 2; oId CUFF.21049.3; tss_id TSS20364;
4	Cufflinks	exon	184148616	184150320	.	+	.	gene_id GRMZM2G114052; transcript_id TCONS_00022522; exon_number 1; oId CUFF.21101.1; tss_id TSS20395;
4	Cufflinks	exon	184150441	184151366	.	+	.	gene_id GRMZM2G114052; transcript_id TCONS_00022522; exon_number 2; oId CUFF.21101.1; tss_id TSS20395;
4	Cufflinks	exon	184152100	184152991	.	+	.	gene_id GRMZM2G114052; transcript_id TCONS_00022522; exon_number 3; oId CUFF.21101.1; tss_id TSS20395;
4	Cufflinks	exon	185642093	185644023	.	+	.	gene_id XLOC_019750; transcript_id TCONS_00022554; exon_number 1; oId CUFF.21168.1; tss_id TSS20425;
4	Cufflinks	exon	185702432	185703426	.	+	.	gene_id XLOC_019752; transcript_id TCONS_00022556; exon_number 1; oId CUFF.21170.1; tss_id TSS20427;
4	Cufflinks	exon	185704570	185704873	.	+	.	gene_id XLOC_019752; transcript_id TCONS_00022556; exon_number 2; oId CUFF.21170.1; tss_id TSS20427;
4	Cufflinks	exon	186764494	186764899	.	+	.	gene_id GRMZM2G171605; transcript_id TCONS_00022572; exon_number 1; oId CUFF.21200.1; tss_id TSS20442;
4	Cufflinks	exon	186765055	186765187	.	+	.	gene_id GRMZM2G171605; transcript_id TCONS_00022572; exon_number 2; oId CUFF.21200.1; tss_id TSS20442;
4	Cufflinks	exon	186765334	186765572	.	+	.	gene_id GRMZM2G171605; transcript_id TCONS_00022572; exon_number 3; oId CUFF.21200.1; tss_id TSS20442;
4	Cufflinks	exon	186766139	186766310	.	+	.	gene_id GRMZM2G171605; transcript_id TCONS_00022572; exon_number 4; oId CUFF.21200.1; tss_id TSS20442;
4	Cufflinks	exon	186766464	186766965	.	+	.	gene_id GRMZM2G171605; transcript_id TCONS_00022572; exon_number 5; oId CUFF.21200.1; tss_id TSS20442;
4	Cufflinks	exon	197104904	197105217	.	+	.	gene_id GRMZM2G440925; transcript_id TCONS_00022715; exon_number 1; oId CUFF.21468.1; tss_id TSS20575;
4	Cufflinks	exon	197105360	197106971	.	+	.	gene_id GRMZM2G440925; transcript_id TCONS_00022715; exon_number 2; oId CUFF.21468.1; tss_id TSS20575;
4	Cufflinks	exon	197104905	197105220	.	+	.	gene_id GRMZM2G440925; transcript_id TCONS_00022716; exon_number 1; oId CUFF.21468.2; tss_id TSS20575;
4	Cufflinks	exon	197105360	197106971	.	+	.	gene_id GRMZM2G440925; transcript_id TCONS_00022716; exon_number 2; oId CUFF.21468.2; tss_id TSS20575;
4	Cufflinks	exon	197104989	197105214	.	+	.	gene_id GRMZM2G440925; transcript_id TCONS_00022718; exon_number 1; oId CUFF.21468.4; tss_id TSS20575;
4	Cufflinks	exon	197105364	197106971	.	+	.	gene_id GRMZM2G440925; transcript_id TCONS_00022718; exon_number 2; oId CUFF.21468.4; tss_id TSS20575;
4	Cufflinks	exon	197104989	197105214	.	+	.	gene_id GRMZM2G440925; transcript_id TCONS_00022717; exon_number 1; oId CUFF.21468.3; tss_id TSS20575;
4	Cufflinks	exon	197105360	197106971	.	+	.	gene_id GRMZM2G440925; transcript_id TCONS_00022717; exon_number 2; oId CUFF.21468.3; tss_id TSS20575;
4	Cufflinks	exon	201299690	201300168	.	+	.	gene_id GRMZM2G079491; transcript_id TCONS_00022781; exon_number 1; oId CUFF.21567.1; tss_id TSS20630;
4	Cufflinks	exon	201300260	201302345	.	+	.	gene_id GRMZM2G079491; transcript_id TCONS_00022781; exon_number 2; oId CUFF.21567.1; tss_id TSS20630;
4	Cufflinks	exon	207618649	207619296	.	+	.	gene_id XLOC_020010; transcript_id TCONS_00022853; exon_number 1; oId CUFF.21677.1; tss_id TSS20694;
4	Cufflinks	exon	211330470	211333066	.	+	.	gene_id GRMZM2G071478; transcript_id TCONS_00022875; exon_number 1; oId CUFF.21730.1; tss_id TSS20713;

4	Cufflinks	exon	213863768	213864678	.	+	.	gene_id GRMZM2G079365; transcript_id TCONS_00022890; exon_number 1; oId CUFF.21783.1; tss_id TSS20727;
4	Cufflinks	exon	213864837	213864969	.	+	.	gene_id GRMZM2G079365; transcript_id TCONS_00022890; exon_number 2; oId CUFF.21783.1; tss_id TSS20727;
4	Cufflinks	exon	213879167	213879251	.	+	.	gene_id GRMZM2G079365; transcript_id TCONS_00022890; exon_number 3; oId CUFF.21783.1; tss_id TSS20727;
4	Cufflinks	exon	213879488	213880142	.	+	.	gene_id GRMZM2G079365; transcript_id TCONS_00022890; exon_number 4; oId CUFF.21783.1; tss_id TSS20727;
4	Cufflinks	exon	217184907	217185462	.	+	.	gene_id XLOC_020073; transcript_id TCONS_00022922; exon_number 1; oId CUFF.21823.1; tss_id TSS20759;
4	Cufflinks	exon	217185549	217185666	.	+	.	gene_id XLOC_020073; transcript_id TCONS_00022922; exon_number 2; oId CUFF.21823.1; tss_id TSS20759;
4	Cufflinks	exon	217186260	217186659	.	+	.	gene_id XLOC_020073; transcript_id TCONS_00022922; exon_number 3; oId CUFF.21823.1; tss_id TSS20759;
4	Cufflinks	exon	217187382	217187452	.	+	.	gene_id XLOC_020073; transcript_id TCONS_00022922; exon_number 4; oId CUFF.21823.1; tss_id TSS20759;
4	Cufflinks	exon	217187553	217187634	.	+	.	gene_id XLOC_020073; transcript_id TCONS_00022922; exon_number 5; oId CUFF.21823.1; tss_id TSS20759;
4	Cufflinks	exon	217188815	217188907	.	+	.	gene_id XLOC_020073; transcript_id TCONS_00022922; exon_number 6; oId CUFF.21823.1; tss_id TSS20759;
4	Cufflinks	exon	217189312	217189552	.	+	.	gene_id XLOC_020073; transcript_id TCONS_00022922; exon_number 7; oId CUFF.21823.1; tss_id TSS20759;
4	Cufflinks	exon	226108085	226108881	.	+	.	gene_id GRMZM2G052920; transcript_id TCONS_00022997; exon_number 1; oId CUFF.21946.1; tss_id TSS20830;
4	Cufflinks	exon	228425361	228426757	.	+	.	gene_id GRMZM2G017022; transcript_id TCONS_00023016; exon_number 1; oId CUFF.22001.1; tss_id TSS20847;
4	Cufflinks	exon	228427012	228428399	.	+	.	gene_id GRMZM2G017022; transcript_id TCONS_00023016; exon_number 2; oId CUFF.22001.1; tss_id TSS20847;
4	Cufflinks	exon	230038398	230038767	.	+	.	gene_id GRMZM2G069631; transcript_id TCONS_00023041; exon_number 1; oId CUFF.22060.1; tss_id TSS20869;
4	Cufflinks	exon	230040469	230040572	.	+	.	gene_id GRMZM2G069631; transcript_id TCONS_00023041; exon_number 2; oId CUFF.22060.1; tss_id TSS20869;
4	Cufflinks	exon	230040650	230040745	.	+	.	gene_id GRMZM2G069631; transcript_id TCONS_00023041; exon_number 3; oId CUFF.22060.1; tss_id TSS20869;
4	Cufflinks	exon	230041977	230042096	.	+	.	gene_id GRMZM2G069631; transcript_id TCONS_00023041; exon_number 4; oId CUFF.22060.1; tss_id TSS20869;
4	Cufflinks	exon	230042479	230042682	.	+	.	gene_id GRMZM2G069631; transcript_id TCONS_00023041; exon_number 5; oId CUFF.22060.1; tss_id TSS20869;
4	Cufflinks	exon	230042861	230042952	.	+	.	gene_id GRMZM2G069631; transcript_id TCONS_00023041; exon_number 6; oId CUFF.22060.1; tss_id TSS20869;
4	Cufflinks	exon	230044102	230044220	.	+	.	gene_id GRMZM2G069631; transcript_id TCONS_00023041; exon_number 7; oId CUFF.22060.1; tss_id TSS20869;
4	Cufflinks	exon	230045435	230045558	.	+	.	gene_id GRMZM2G069631; transcript_id TCONS_00023041; exon_number 8; oId CUFF.22060.1; tss_id TSS20869;
4	Cufflinks	exon	230045703	230045826	.	+	.	gene_id GRMZM2G069631; transcript_id TCONS_00023041; exon_number 9; oId CUFF.22060.1; tss_id TSS20869;
4	Cufflinks	exon	230046164	230046232	.	+	.	gene_id GRMZM2G069631; transcript_id TCONS_00023041; exon_number 10; oId CUFF.22060.1; tss_id TSS20869;
4	Cufflinks	exon	230046319	230046410	.	+	.	gene_id GRMZM2G069631; transcript_id TCONS_00023041; exon_number 11; oId CUFF.22060.1; tss_id TSS20869;
4	Cufflinks	exon	230046512	230046589	.	+	.	gene_id GRMZM2G069631; transcript_id TCONS_00023041; exon_number 12; oId CUFF.22060.1; tss_id TSS20869;
4	Cufflinks	exon	230046996	230047054	.	+	.	gene_id GRMZM2G069631; transcript_id TCONS_00023041; exon_number 13; oId CUFF.22060.1; tss_id TSS20869;
4	Cufflinks	exon	230047153	230047311	.	+	.	gene_id GRMZM2G069631; transcript_id TCONS_00023041; exon_number 14; oId CUFF.22060.1; tss_id TSS20869;
4	Cufflinks	exon	230047389	230047846	.	+	.	gene_id GRMZM2G069631; transcript_id TCONS_00023041; exon_number 15; oId CUFF.22060.1; tss_id TSS20869;
4	Cufflinks	exon	235227459	235227682	.	+	.	gene_id GRMZM2G125308; transcript_id TCONS_00023121; exon_number 1; oId CUFF.22185.1; tss_id TSS20940;
4	Cufflinks	exon	235227778	235227940	.	+	.	gene_id GRMZM2G125308; transcript_id TCONS_00023121; exon_number 2; oId CUFF.22185.1; tss_id TSS20940;
4	Cufflinks	exon	235228119	235228381	.	+	.	gene_id GRMZM2G125308; transcript_id TCONS_00023121; exon_number 3; oId CUFF.22185.1; tss_id TSS20940;
4	Cufflinks	exon	235228496	235229436	.	+	.	gene_id GRMZM2G125308; transcript_id TCONS_00023121; exon_number 4; oId CUFF.22185.1; tss_id TSS20940;
4	Cufflinks	exon	238872952	238873626	.	+	.	gene_id XLOC_020356; transcript_id TCONS_00023243; exon_number 1; oId CUFF.22384.1; tss_id TSS21046;
4	Cufflinks	exon	239641012	239641380	.	+	.	gene_id XLOC_020374; transcript_id TCONS_00023266; exon_number 1; oId CUFF.22410.1; tss_id TSS21065;
4	Cufflinks	exon	240272545	240272774	.	+	.	gene_id GRMZM2G025127; transcript_id TCONS_00023285; exon_number 1; oId CUFF.22471.1; tss_id TSS21084;

4	Cufflinks	exon	240273241	240273617	.	+	.	gene_id GRMZM2G025127; transcript_id TCONS_00023285; exon_number 2; oId CUFF.22471.1; tss_id TSS21084;
4	Cufflinks	exon	240274271	240274406	.	+	.	gene_id GRMZM2G025127; transcript_id TCONS_00023285; exon_number 3; oId CUFF.22471.1; tss_id TSS21084;
4	Cufflinks	exon	240274679	240274815	.	+	.	gene_id GRMZM2G025127; transcript_id TCONS_00023285; exon_number 4; oId CUFF.22471.1; tss_id TSS21084;
4	Cufflinks	exon	240274906	240275029	.	+	.	gene_id GRMZM2G025127; transcript_id TCONS_00023285; exon_number 5; oId CUFF.22471.1; tss_id TSS21084;
4	Cufflinks	exon	240275746	240276538	.	+	.	gene_id GRMZM2G025127; transcript_id TCONS_00023285; exon_number 6; oId CUFF.22471.1; tss_id TSS21084;
4	Cufflinks	exon	512986	515273	.	-	.	gene_id GRMZM2G018254; transcript_id TCONS_00023329; exon_number 1; oId CUFF.18297.1; tss_id TSS21125;
4	Cufflinks	exon	829560	832103	.	-	.	gene_id GRMZM2G073823; transcript_id TCONS_00023331; exon_number 1; oId CUFF.18310.1; tss_id TSS21127;
4	Cufflinks	exon	832506	835056	.	-	.	gene_id GRMZM2G073805; transcript_id TCONS_00023332; exon_number 1; oId CUFF.18313.1; tss_id TSS21128;
4	Cufflinks	exon	1078823	1079216	.	-	.	gene_id XLOC_020442; transcript_id TCONS_00023339; exon_number 1; oId CUFF.18335.1; tss_id TSS21135;
4	Cufflinks	exon	1079394	1079883	.	-	.	gene_id XLOC_020442; transcript_id TCONS_00023339; exon_number 2; oId CUFF.18335.1; tss_id TSS21135;
4	Cufflinks	exon	5006400	5007189	.	-	.	gene_id GRMZM5G877647; transcript_id TCONS_00023421; exon_number 1; oId CUFF.18500.3; tss_id TSS21209;
4	Cufflinks	exon	5007379	5007784	.	-	.	gene_id GRMZM5G877647; transcript_id TCONS_00023421; exon_number 2; oId CUFF.18500.3; tss_id TSS21209;
4	Cufflinks	exon	5006400	5007194	.	-	.	gene_id GRMZM5G877647; transcript_id TCONS_00023422; exon_number 1; oId CUFF.18500.1; tss_id TSS21209;
4	Cufflinks	exon	5007379	5007839	.	-	.	gene_id GRMZM5G877647; transcript_id TCONS_00023422; exon_number 2; oId CUFF.18500.1; tss_id TSS21209;
4	Cufflinks	exon	6247911	6249250	.	-	.	gene_id AC212755.4_FG001; transcript_id TCONS_00023449; exon_number 1; oId CUFF.18541.1; tss_id TSS21235;
4	Cufflinks	exon	9896505	9896920	.	-	.	gene_id GRMZM2G113470; transcript_id TCONS_00023476; exon_number 1; oId CUFF.18601.1; tss_id TSS21259;
4	Cufflinks	exon	9901727	9901999	.	-	.	gene_id GRMZM2G113470; transcript_id TCONS_00023476; exon_number 2; oId CUFF.18601.1; tss_id TSS21259;
4	Cufflinks	exon	9902086	9902617	.	-	.	gene_id GRMZM2G113470; transcript_id TCONS_00023476; exon_number 3; oId CUFF.18601.1; tss_id TSS21259;
4	Cufflinks	exon	14877039	14877684	.	-	.	gene_id GRMZM2G091481; transcript_id TCONS_00023526; exon_number 1; oId CUFF.18707.1; tss_id TSS21307;
4	Cufflinks	exon	14877773	14877885	.	-	.	gene_id GRMZM2G091481; transcript_id TCONS_00023526; exon_number 2; oId CUFF.18707.1; tss_id TSS21307;
4	Cufflinks	exon	14878061	14878178	.	-	.	gene_id GRMZM2G091481; transcript_id TCONS_00023526; exon_number 3; oId CUFF.18707.1; tss_id TSS21307;
4	Cufflinks	exon	14878271	14878390	.	-	.	gene_id GRMZM2G091481; transcript_id TCONS_00023526; exon_number 4; oId CUFF.18707.1; tss_id TSS21307;
4	Cufflinks	exon	14878542	14878679	.	-	.	gene_id GRMZM2G091481; transcript_id TCONS_00023526; exon_number 5; oId CUFF.18707.1; tss_id TSS21307;
4	Cufflinks	exon	14879872	14879997	.	-	.	gene_id GRMZM2G091481; transcript_id TCONS_00023526; exon_number 6; oId CUFF.18707.1; tss_id TSS21307;
4	Cufflinks	exon	14880137	14880325	.	-	.	gene_id GRMZM2G091481; transcript_id TCONS_00023526; exon_number 7; oId CUFF.18707.1; tss_id TSS21307;
4	Cufflinks	exon	14880432	14880719	.	-	.	gene_id GRMZM2G091481; transcript_id TCONS_00023526; exon_number 8; oId CUFF.18707.1; tss_id TSS21307;
4	Cufflinks	exon	14880824	14880854	.	-	.	gene_id GRMZM2G091481; transcript_id TCONS_00023526; exon_number 9; oId CUFF.18707.1; tss_id TSS21307;
4	Cufflinks	exon	14881450	14881740	.	-	.	gene_id GRMZM2G091481; transcript_id TCONS_00023526; exon_number 10; oId CUFF.18707.1; tss_id TSS21307;
4	Cufflinks	exon	18610025	18610696	.	-	.	gene_id GRMZM2G139894; transcript_id TCONS_00023562; exon_number 1; oId CUFF.18778.1; tss_id TSS21343;
4	Cufflinks	exon	18610782	18610943	.	-	.	gene_id GRMZM2G139894; transcript_id TCONS_00023562; exon_number 2; oId CUFF.18778.1; tss_id TSS21343;
4	Cufflinks	exon	18611231	18611413	.	-	.	gene_id GRMZM2G139894; transcript_id TCONS_00023562; exon_number 3; oId CUFF.18778.1; tss_id TSS21343;
4	Cufflinks	exon	18611526	18611651	.	-	.	gene_id GRMZM2G139894; transcript_id TCONS_00023562; exon_number 4; oId CUFF.18778.1; tss_id TSS21343;
4	Cufflinks	exon	18611731	18611883	.	-	.	gene_id GRMZM2G139894; transcript_id TCONS_00023562; exon_number 5; oId CUFF.18778.1; tss_id TSS21343;
4	Cufflinks	exon	18611968	18612052	.	-	.	gene_id GRMZM2G139894; transcript_id TCONS_00023562; exon_number 6; oId CUFF.18778.1; tss_id TSS21343;
4	Cufflinks	exon	18612137	18612253	.	-	.	gene_id GRMZM2G139894; transcript_id TCONS_00023562; exon_number 7; oId CUFF.18778.1; tss_id TSS21343;
4	Cufflinks	exon	18612340	18612528	.	-	.	gene_id GRMZM2G139894; transcript_id TCONS_00023562; exon_number 8; oId CUFF.18778.1; tss_id TSS21343;

4	Cufflinks	exon	18612592	18612764	.	-	.	gene_id GRMZM2G139894; transcript_id TCONS_00023562; exon_number 9; oId CUFF.18778.1; tss_id TSS21343;
4	Cufflinks	exon	18612844	18613035	.	-	.	gene_id GRMZM2G139894; transcript_id TCONS_00023562; exon_number 10; oId CUFF.18778.1; tss_id TSS21343;
4	Cufflinks	exon	18613118	18613252	.	-	.	gene_id GRMZM2G139894; transcript_id TCONS_00023562; exon_number 11; oId CUFF.18778.1; tss_id TSS21343;
4	Cufflinks	exon	18613396	18613603	.	-	.	gene_id GRMZM2G139894; transcript_id TCONS_00023562; exon_number 12; oId CUFF.18778.1; tss_id TSS21343;
4	Cufflinks	exon	18613685	18613904	.	-	.	gene_id GRMZM2G139894; transcript_id TCONS_00023562; exon_number 13; oId CUFF.18778.1; tss_id TSS21343;
4	Cufflinks	exon	18613982	18614171	.	-	.	gene_id GRMZM2G139894; transcript_id TCONS_00023562; exon_number 14; oId CUFF.18778.1; tss_id TSS21343;
4	Cufflinks	exon	18614305	18614501	.	-	.	gene_id GRMZM2G139894; transcript_id TCONS_00023562; exon_number 15; oId CUFF.18778.1; tss_id TSS21343;
4	Cufflinks	exon	18615127	18615314	.	-	.	gene_id GRMZM2G139894; transcript_id TCONS_00023562; exon_number 16; oId CUFF.18778.1; tss_id TSS21343;
4	Cufflinks	exon	18616118	18616353	.	-	.	gene_id GRMZM2G139894; transcript_id TCONS_00023562; exon_number 17; oId CUFF.18778.1; tss_id TSS21343;
4	Cufflinks	exon	18616454	18616532	.	-	.	gene_id GRMZM2G139894; transcript_id TCONS_00023562; exon_number 18; oId CUFF.18778.1; tss_id TSS21343;
4	Cufflinks	exon	19133772	19136181	.	-	.	gene_id GRMZM2G177668; transcript_id TCONS_00023568; exon_number 1; oId CUFF.18770.1; tss_id TSS21349;
4	Cufflinks	exon	29179242	29181600	.	-	.	gene_id GRMZM2G382323; transcript_id TCONS_00023651; exon_number 1; oId CUFF.18944.1; tss_id TSS21427;
4	Cufflinks	exon	32278497	32279553	.	-	.	gene_id AC196475.3_FG004; transcript_id TCONS_00023679; exon_number 1; oId CUFF.18999.1; tss_id TSS21455;
4	Cufflinks	exon	32280331	32280771	.	-	.	gene_id AC196475.3_FG004; transcript_id TCONS_00023679; exon_number 2; oId CUFF.18999.1; tss_id TSS21455;
4	Cufflinks	exon	32280812	32280957	.	-	.	gene_id AC196475.3_FG004; transcript_id TCONS_00023679; exon_number 3; oId CUFF.18999.1; tss_id TSS21455;
4	Cufflinks	exon	37262877	37263974	.	-	.	gene_id GRMZM2G363540; transcript_id TCONS_00023728; exon_number 1; oId CUFF.19090.1; tss_id TSS21500;
4	Cufflinks	exon	38791036	38791462	.	-	.	gene_id GRMZM2G105387; transcript_id TCONS_00023751; exon_number 1; oId CUFF.19134.1; tss_id TSS21522;
4	Cufflinks	exon	38791611	38791652	.	-	.	gene_id GRMZM2G105387; transcript_id TCONS_00023751; exon_number 2; oId CUFF.19134.1; tss_id TSS21522;
4	Cufflinks	exon	38791743	38791784	.	-	.	gene_id GRMZM2G105387; transcript_id TCONS_00023751; exon_number 3; oId CUFF.19134.1; tss_id TSS21522;
4	Cufflinks	exon	38791860	38791962	.	-	.	gene_id GRMZM2G105387; transcript_id TCONS_00023751; exon_number 4; oId CUFF.19134.1; tss_id TSS21522;
4	Cufflinks	exon	38792059	38792120	.	-	.	gene_id GRMZM2G105387; transcript_id TCONS_00023751; exon_number 5; oId CUFF.19134.1; tss_id TSS21522;
4	Cufflinks	exon	38792222	38792306	.	-	.	gene_id GRMZM2G105387; transcript_id TCONS_00023751; exon_number 6; oId CUFF.19134.1; tss_id TSS21522;
4	Cufflinks	exon	38792399	38792802	.	-	.	gene_id GRMZM2G105387; transcript_id TCONS_00023751; exon_number 7; oId CUFF.19134.1; tss_id TSS21522;
4	Cufflinks	exon	39633113	39633886	.	-	.	gene_id GRMZM2G135568; transcript_id TCONS_00023763; exon_number 1; oId CUFF.19185.1; tss_id TSS21533;
4	Cufflinks	exon	39633963	39634112	.	-	.	gene_id GRMZM2G135568; transcript_id TCONS_00023763; exon_number 2; oId CUFF.19185.1; tss_id TSS21533;
4	Cufflinks	exon	39634247	39634332	.	-	.	gene_id GRMZM2G135568; transcript_id TCONS_00023763; exon_number 3; oId CUFF.19185.1; tss_id TSS21533;
4	Cufflinks	exon	39634487	39634562	.	-	.	gene_id GRMZM2G135568; transcript_id TCONS_00023763; exon_number 4; oId CUFF.19185.1; tss_id TSS21533;
4	Cufflinks	exon	39635111	39635268	.	-	.	gene_id GRMZM2G135568; transcript_id TCONS_00023763; exon_number 5; oId CUFF.19185.1; tss_id TSS21533;
4	Cufflinks	exon	39635681	39635794	.	-	.	gene_id GRMZM2G135568; transcript_id TCONS_00023763; exon_number 6; oId CUFF.19185.1; tss_id TSS21533;
4	Cufflinks	exon	39635889	39635987	.	-	.	gene_id GRMZM2G135568; transcript_id TCONS_00023763; exon_number 7; oId CUFF.19185.1; tss_id TSS21533;
4	Cufflinks	exon	39636485	39636563	.	-	.	gene_id GRMZM2G135568; transcript_id TCONS_00023763; exon_number 8; oId CUFF.19185.1; tss_id TSS21533;
4	Cufflinks	exon	39636867	39637075	.	-	.	gene_id GRMZM2G135568; transcript_id TCONS_00023763; exon_number 9; oId CUFF.19185.1; tss_id TSS21533;
4	Cufflinks	exon	39637242	39637478	.	-	.	gene_id GRMZM2G135568; transcript_id TCONS_00023763; exon_number 10; oId CUFF.19185.1; tss_id TSS21533;
4	Cufflinks	exon	44885720	44886103	.	-	.	gene_id GRMZM2G006953; transcript_id TCONS_00023809; exon_number 1; oId CUFF.19228.1; tss_id TSS21575;
4	Cufflinks	exon	44887920	44888367	.	-	.	gene_id GRMZM2G006953; transcript_id TCONS_00023809; exon_number 2; oId CUFF.19228.1; tss_id TSS21575;
4	Cufflinks	exon	46961562	46962159	.	-	.	gene_id XLOC_020878; transcript_id TCONS_00023823; exon_number 1; oId CUFF.19259.1; tss_id TSS21586;

4	Cufflinks	exon	47746128	47746701	.	-	.	gene_id GRMZM2G107289; transcript_id TCONS_00023830; exon_number 1; oId CUFF.19263.1; tss_id TSS21592;
4	Cufflinks	exon	47747361	47747461	.	-	.	gene_id GRMZM2G107289; transcript_id TCONS_00023830; exon_number 2; oId CUFF.19263.1; tss_id TSS21592;
4	Cufflinks	exon	47747537	47747586	.	-	.	gene_id GRMZM2G107289; transcript_id TCONS_00023830; exon_number 3; oId CUFF.19263.1; tss_id TSS21592;
4	Cufflinks	exon	47747756	47747808	.	-	.	gene_id GRMZM2G107289; transcript_id TCONS_00023830; exon_number 4; oId CUFF.19263.1; tss_id TSS21592;
4	Cufflinks	exon	47749315	47749437	.	-	.	gene_id GRMZM2G107289; transcript_id TCONS_00023830; exon_number 5; oId CUFF.19263.1; tss_id TSS21592;
4	Cufflinks	exon	47749674	47749757	.	-	.	gene_id GRMZM2G107289; transcript_id TCONS_00023830; exon_number 6; oId CUFF.19263.1; tss_id TSS21592;
4	Cufflinks	exon	47749861	47749926	.	-	.	gene_id GRMZM2G107289; transcript_id TCONS_00023830; exon_number 7; oId CUFF.19263.1; tss_id TSS21592;
4	Cufflinks	exon	47751018	47751481	.	-	.	gene_id GRMZM2G107289; transcript_id TCONS_00023830; exon_number 8; oId CUFF.19263.1; tss_id TSS21592;
4	Cufflinks	exon	60384110	60385046	.	-	.	gene_id GRMZM2G121928; transcript_id TCONS_00023902; exon_number 1; oId CUFF.19384.1; tss_id TSS21659;
4	Cufflinks	exon	61742444	61743224	.	-	.	gene_id GRMZM2G011806; transcript_id TCONS_00023909; exon_number 1; oId CUFF.19415.1; tss_id TSS21666;
4	Cufflinks	exon	61743869	61746837	.	-	.	gene_id GRMZM2G011806; transcript_id TCONS_00023909; exon_number 2; oId CUFF.19415.1; tss_id TSS21666;
4	Cufflinks	exon	61747811	61748096	.	-	.	gene_id GRMZM2G011806; transcript_id TCONS_00023909; exon_number 3; oId CUFF.19415.1; tss_id TSS21666;
4	Cufflinks	exon	63562329	63563184	.	-	.	gene_id GRMZM2G082087; transcript_id TCONS_00023926; exon_number 1; oId CUFF.19430.1; tss_id TSS21681;
4	Cufflinks	exon	63563343	63563525	.	-	.	gene_id GRMZM2G082087; transcript_id TCONS_00023926; exon_number 2; oId CUFF.19430.1; tss_id TSS21681;
4	Cufflinks	exon	63564403	63564533	.	-	.	gene_id GRMZM2G082087; transcript_id TCONS_00023926; exon_number 3; oId CUFF.19430.1; tss_id TSS21681;
4	Cufflinks	exon	63565450	63565590	.	-	.	gene_id GRMZM2G082087; transcript_id TCONS_00023926; exon_number 4; oId CUFF.19430.1; tss_id TSS21681;
4	Cufflinks	exon	63565697	63566123	.	-	.	gene_id GRMZM2G082087; transcript_id TCONS_00023926; exon_number 5; oId CUFF.19430.1; tss_id TSS21681;
4	Cufflinks	exon	67675287	67676573	.	-	.	gene_id GRMZM2G359986; transcript_id TCONS_00023950; exon_number 1; oId CUFF.19480.1; tss_id TSS21700;
4	Cufflinks	exon	67677973	67678035	.	-	.	gene_id GRMZM2G359986; transcript_id TCONS_00023950; exon_number 2; oId CUFF.19480.1; tss_id TSS21700;
4	Cufflinks	exon	67678151	67679206	.	-	.	gene_id GRMZM2G359986; transcript_id TCONS_00023950; exon_number 3; oId CUFF.19480.1; tss_id TSS21700;
4	Cufflinks	exon	77234574	77236038	.	-	.	gene_id XLOC_021048; transcript_id TCONS_00024024; exon_number 1; oId CUFF.19586.1; tss_id TSS21757;
4	Cufflinks	exon	86308802	86310197	.	-	.	gene_id GRMZM2G089698; transcript_id TCONS_00024082; exon_number 1; oId CUFF.19679.1; tss_id TSS21807;
4	Cufflinks	exon	86310288	86310939	.	-	.	gene_id GRMZM2G089698; transcript_id TCONS_00024082; exon_number 2; oId CUFF.19679.1; tss_id TSS21807;
4	Cufflinks	exon	92859072	92859601	.	-	.	gene_id XLOC_021125; transcript_id TCONS_00024111; exon_number 1; oId CUFF.19732.1; tss_id TSS21834;
4	Cufflinks	exon	92859682	92859932	.	-	.	gene_id XLOC_021125; transcript_id TCONS_00024111; exon_number 2; oId CUFF.19732.1; tss_id TSS21834;
4	Cufflinks	exon	92860027	92860156	.	-	.	gene_id XLOC_021125; transcript_id TCONS_00024111; exon_number 3; oId CUFF.19732.1; tss_id TSS21834;
4	Cufflinks	exon	92860266	92860700	.	-	.	gene_id XLOC_021125; transcript_id TCONS_00024111; exon_number 4; oId CUFF.19732.1; tss_id TSS21834;
4	Cufflinks	exon	141306653	141307645	.	-	.	gene_id XLOC_021322; transcript_id TCONS_00024342; exon_number 1; oId CUFF.20143.1; tss_id TSS22040;
4	Cufflinks	exon	142662836	142663677	.	-	.	gene_id GRMZM2G127857; transcript_id TCONS_00024351; exon_number 1; oId CUFF.20162.1; tss_id TSS22048;
4	Cufflinks	exon	142663960	142664368	.	-	.	gene_id GRMZM2G127857; transcript_id TCONS_00024351; exon_number 2; oId CUFF.20162.1; tss_id TSS22048;
4	Cufflinks	exon	151682337	151683625	.	-	.	gene_id GRMZM2G145221; transcript_id TCONS_00024407; exon_number 1; oId CUFF.20303.1; tss_id TSS22100;
4	Cufflinks	exon	151683715	151683860	.	-	.	gene_id GRMZM2G145221; transcript_id TCONS_00024407; exon_number 2; oId CUFF.20303.1; tss_id TSS22100;
4	Cufflinks	exon	151683949	151684641	.	-	.	gene_id GRMZM2G145221; transcript_id TCONS_00024407; exon_number 3; oId CUFF.20303.1; tss_id TSS22100;
4	Cufflinks	exon	152425543	152425832	.	-	.	gene_id GRMZM2G026922; transcript_id TCONS_00024415; exon_number 1; oId CUFF.20312.1; tss_id TSS22108;
4	Cufflinks	exon	152426021	152426255	.	-	.	gene_id GRMZM2G026922; transcript_id TCONS_00024415; exon_number 2; oId CUFF.20312.1; tss_id TSS22108;
4	Cufflinks	exon	152426364	152426457	.	-	.	gene_id GRMZM2G026922; transcript_id TCONS_00024415; exon_number 3; oId CUFF.20312.1; tss_id TSS22108;

4	Cufflinks	exon	152426804	152427017	.	-	.	gene_id GRMZM2G026922; transcript_id TCONS_00024415; exon_number 4; oId CUFF.20312.1; tss_id TSS22108;
4	Cufflinks	exon	152433116	152433694	.	-	.	gene_id GRMZM2G026922; transcript_id TCONS_00024415; exon_number 5; oId CUFF.20312.1; tss_id TSS22108;
4	Cufflinks	exon	155035662	155037140	.	-	.	gene_id GRMZM2G114998; transcript_id TCONS_00024445; exon_number 1; oId CUFF.20363.1; tss_id TSS22136;
4	Cufflinks	exon	155038117	155038571	.	-	.	gene_id GRMZM2G114998; transcript_id TCONS_00024445; exon_number 2; oId CUFF.20363.1; tss_id TSS22136;
4	Cufflinks	exon	156986015	156986580	.	-	.	gene_id GRMZM2G081172; transcript_id TCONS_00024466; exon_number 1; oId CUFF.20418.2; tss_id TSS22157;
4	Cufflinks	exon	156986704	156986945	.	-	.	gene_id GRMZM2G081172; transcript_id TCONS_00024466; exon_number 2; oId CUFF.20418.2; tss_id TSS22157;
4	Cufflinks	exon	156987390	156987605	.	-	.	gene_id GRMZM2G081172; transcript_id TCONS_00024466; exon_number 3; oId CUFF.20418.2; tss_id TSS22157;
4	Cufflinks	exon	156986015	156986580	.	-	.	gene_id GRMZM2G081172; transcript_id TCONS_00024467; exon_number 1; oId CUFF.20418.1; tss_id TSS22157;
4	Cufflinks	exon	156986786	156986945	.	-	.	gene_id GRMZM2G081172; transcript_id TCONS_00024467; exon_number 2; oId CUFF.20418.1; tss_id TSS22157;
4	Cufflinks	exon	156987390	156987610	.	-	.	gene_id GRMZM2G081172; transcript_id TCONS_00024467; exon_number 3; oId CUFF.20418.1; tss_id TSS22157;
4	Cufflinks	exon	158326931	158329099	.	-	.	gene_id GRMZM2G350157; transcript_id TCONS_00024483; exon_number 1; oId CUFF.20429.1; tss_id TSS22172;
4	Cufflinks	exon	158585836	158586204	.	-	.	gene_id GRMZM2G121075; transcript_id TCONS_00024489; exon_number 1; oId CUFF.20445.1; tss_id TSS22178;
4	Cufflinks	exon	158586586	158587198	.	-	.	gene_id GRMZM2G121075; transcript_id TCONS_00024489; exon_number 2; oId CUFF.20445.1; tss_id TSS22178;
4	Cufflinks	exon	160367219	160368303	.	-	.	gene_id GRMZM2G119258; transcript_id TCONS_00024521; exon_number 1; oId CUFF.20503.1; tss_id TSS22208;
4	Cufflinks	exon	160371325	160371443	.	-	.	gene_id GRMZM2G119258; transcript_id TCONS_00024521; exon_number 2; oId CUFF.20503.1; tss_id TSS22208;
4	Cufflinks	exon	160371561	160371682	.	-	.	gene_id GRMZM2G119258; transcript_id TCONS_00024521; exon_number 3; oId CUFF.20503.1; tss_id TSS22208;
4	Cufflinks	exon	160374334	160374647	.	-	.	gene_id GRMZM2G119258; transcript_id TCONS_00024521; exon_number 4; oId CUFF.20503.1; tss_id TSS22208;
4	Cufflinks	exon	161579119	161581022	.	-	.	gene_id GRMZM2G022514; transcript_id TCONS_00024533; exon_number 1; oId CUFF.20523.1; tss_id TSS22220;
4	Cufflinks	exon	161581880	161583314	.	-	.	gene_id GRMZM2G022514; transcript_id TCONS_00024533; exon_number 2; oId CUFF.20523.1; tss_id TSS22220;
4	Cufflinks	exon	169989941	169990709	.	-	.	gene_id GRMZM2G082007; transcript_id TCONS_00024626; exon_number 1; oId CUFF.20698.1; tss_id TSS22308;
4	Cufflinks	exon	169990791	169990855	.	-	.	gene_id GRMZM2G082007; transcript_id TCONS_00024626; exon_number 2; oId CUFF.20698.1; tss_id TSS22308;
4	Cufflinks	exon	169992625	169993608	.	-	.	gene_id GRMZM2G082007; transcript_id TCONS_00024626; exon_number 3; oId CUFF.20698.1; tss_id TSS22308;
4	Cufflinks	exon	170004620	170005149	.	-	.	gene_id GRMZM2G081843; transcript_id TCONS_00024628; exon_number 1; oId CUFF.20695.1; tss_id TSS22310;
4	Cufflinks	exon	170005243	170006029	.	-	.	gene_id GRMZM2G081843; transcript_id TCONS_00024628; exon_number 2; oId CUFF.20695.1; tss_id TSS22310;
4	Cufflinks	exon	170006775	170006968	.	-	.	gene_id GRMZM2G081843; transcript_id TCONS_00024628; exon_number 3; oId CUFF.20695.1; tss_id TSS22310;
4	Cufflinks	exon	180464434	180465406	.	-	.	gene_id GRMZM2G169617; transcript_id TCONS_00024804; exon_number 1; oId CUFF.21045.3; tss_id TSS22461;
4	Cufflinks	exon	180465716	180466759	.	-	.	gene_id GRMZM2G169617; transcript_id TCONS_00024804; exon_number 2; oId CUFF.21045.3; tss_id TSS22461;
4	Cufflinks	exon	184154926	184156822	.	-	.	gene_id GRMZM2G113967; transcript_id TCONS_00024873; exon_number 1; oId CUFF.21096.1; tss_id TSS22520;
4	Cufflinks	exon	184157485	184157682	.	-	.	gene_id GRMZM2G113967; transcript_id TCONS_00024873; exon_number 2; oId CUFF.21096.1; tss_id TSS22520;
4	Cufflinks	exon	185209888	185210808	.	-	.	gene_id GRMZM2G151516; transcript_id TCONS_00024897; exon_number 1; oId CUFF.21151.1; tss_id TSS22542;
4	Cufflinks	exon	185210954	185211477	.	-	.	gene_id GRMZM2G151516; transcript_id TCONS_00024897; exon_number 2; oId CUFF.21151.1; tss_id TSS22542;
4	Cufflinks	exon	185702075	185702304	.	-	.	gene_id GRMZM2G179733; transcript_id TCONS_00024910; exon_number 1; oId CUFF.21169.1; tss_id TSS22555;
4	Cufflinks	exon	185702504	185703421	.	-	.	gene_id GRMZM2G179733; transcript_id TCONS_00024910; exon_number 2; oId CUFF.21169.1; tss_id TSS22555;
4	Cufflinks	exon	185703695	185703839	.	-	.	gene_id GRMZM2G179733; transcript_id TCONS_00024910; exon_number 3; oId CUFF.21169.1; tss_id TSS22555;
4	Cufflinks	exon	186214078	186215780	.	-	.	gene_id GRMZM2G146553; transcript_id TCONS_00024926; exon_number 1; oId CUFF.21213.2; tss_id TSS22570;
4	Cufflinks	exon	186215955	186216029	.	-	.	gene_id GRMZM2G146553; transcript_id TCONS_00024926; exon_number 2; oId CUFF.21213.2; tss_id TSS22570;

4	Cufflinks	exon	186216110	186216166	.	-	.	gene_id GRMZM2G146553; transcript_id TCONS_00024926; exon_number 3; oId CUFF.21213.2; tss_id TSS22570;
4	Cufflinks	exon	186216296	186216409	.	-	.	gene_id GRMZM2G146553; transcript_id TCONS_00024926; exon_number 4; oId CUFF.21213.2; tss_id TSS22570;
4	Cufflinks	exon	186216717	186216827	.	-	.	gene_id GRMZM2G146553; transcript_id TCONS_00024926; exon_number 5; oId CUFF.21213.2; tss_id TSS22570;
4	Cufflinks	exon	186216924	186217046	.	-	.	gene_id GRMZM2G146553; transcript_id TCONS_00024926; exon_number 6; oId CUFF.21213.2; tss_id TSS22570;
4	Cufflinks	exon	186217526	186217615	.	-	.	gene_id GRMZM2G146553; transcript_id TCONS_00024926; exon_number 7; oId CUFF.21213.2; tss_id TSS22570;
4	Cufflinks	exon	186217698	186217823	.	-	.	gene_id GRMZM2G146553; transcript_id TCONS_00024926; exon_number 8; oId CUFF.21213.2; tss_id TSS22570;
4	Cufflinks	exon	186217925	186217978	.	-	.	gene_id GRMZM2G146553; transcript_id TCONS_00024926; exon_number 9; oId CUFF.21213.2; tss_id TSS22570;
4	Cufflinks	exon	186218147	186218227	.	-	.	gene_id GRMZM2G146553; transcript_id TCONS_00024926; exon_number 10; oId CUFF.21213.2; tss_id TSS22570;
4	Cufflinks	exon	186218305	186218412	.	-	.	gene_id GRMZM2G146553; transcript_id TCONS_00024926; exon_number 11; oId CUFF.21213.2; tss_id TSS22570;
4	Cufflinks	exon	186218554	186218625	.	-	.	gene_id GRMZM2G146553; transcript_id TCONS_00024926; exon_number 12; oId CUFF.21213.2; tss_id TSS22570;
4	Cufflinks	exon	186218793	186218855	.	-	.	gene_id GRMZM2G146553; transcript_id TCONS_00024926; exon_number 13; oId CUFF.21213.2; tss_id TSS22570;
4	Cufflinks	exon	186219093	186219344	.	-	.	gene_id GRMZM2G146553; transcript_id TCONS_00024926; exon_number 14; oId CUFF.21213.2; tss_id TSS22570;
4	Cufflinks	exon	186220524	186220919	.	-	.	gene_id GRMZM2G146553; transcript_id TCONS_00024926; exon_number 15; oId CUFF.21213.2; tss_id TSS22570;
4	Cufflinks	exon	188665523	188666118	.	-	.	gene_id GRMZM2G030877; transcript_id TCONS_00024982; exon_number 1; oId CUFF.21280.5; tss_id TSS22619;
4	Cufflinks	exon	188667536	188667678	.	-	.	gene_id GRMZM2G030877; transcript_id TCONS_00024982; exon_number 2; oId CUFF.21280.5; tss_id TSS22619;
4	Cufflinks	exon	188668233	188668469	.	-	.	gene_id GRMZM2G030877; transcript_id TCONS_00024982; exon_number 3; oId CUFF.21280.5; tss_id TSS22619;
4	Cufflinks	exon	188668690	188668961	.	-	.	gene_id GRMZM2G030877; transcript_id TCONS_00024982; exon_number 4; oId CUFF.21280.5; tss_id TSS22619;
4	Cufflinks	exon	188669075	188669117	.	-	.	gene_id GRMZM2G030877; transcript_id TCONS_00024982; exon_number 5; oId CUFF.21280.5; tss_id TSS22619;
4	Cufflinks	exon	188669384	188669461	.	-	.	gene_id GRMZM2G030877; transcript_id TCONS_00024982; exon_number 6; oId CUFF.21280.5; tss_id TSS22619;
4	Cufflinks	exon	188669566	188669625	.	-	.	gene_id GRMZM2G030877; transcript_id TCONS_00024982; exon_number 7; oId CUFF.21280.5; tss_id TSS22619;
4	Cufflinks	exon	188669784	188669834	.	-	.	gene_id GRMZM2G030877; transcript_id TCONS_00024982; exon_number 8; oId CUFF.21280.5; tss_id TSS22619;
4	Cufflinks	exon	188670920	188671072	.	-	.	gene_id GRMZM2G030877; transcript_id TCONS_00024982; exon_number 9; oId CUFF.21280.5; tss_id TSS22619;
4	Cufflinks	exon	188671232	188671347	.	-	.	gene_id GRMZM2G030877; transcript_id TCONS_00024982; exon_number 10; oId CUFF.21280.5; tss_id TSS22619;
4	Cufflinks	exon	188671831	188671955	.	-	.	gene_id GRMZM2G030877; transcript_id TCONS_00024982; exon_number 11; oId CUFF.21280.5; tss_id TSS22619;
4	Cufflinks	exon	188672268	188672437	.	-	.	gene_id GRMZM2G030877; transcript_id TCONS_00024982; exon_number 12; oId CUFF.21280.5; tss_id TSS22619;
4	Cufflinks	exon	188672555	188672953	.	-	.	gene_id GRMZM2G030877; transcript_id TCONS_00024982; exon_number 13; oId CUFF.21280.5; tss_id TSS22619;
4	Cufflinks	exon	188665523	188666118	.	-	.	gene_id GRMZM2G030877; transcript_id TCONS_00024983; exon_number 1; oId CUFF.21280.1; tss_id TSS22619;
4	Cufflinks	exon	188667536	188667678	.	-	.	gene_id GRMZM2G030877; transcript_id TCONS_00024983; exon_number 2; oId CUFF.21280.1; tss_id TSS22619;
4	Cufflinks	exon	188668233	188668469	.	-	.	gene_id GRMZM2G030877; transcript_id TCONS_00024983; exon_number 3; oId CUFF.21280.1; tss_id TSS22619;
4	Cufflinks	exon	188668690	188668961	.	-	.	gene_id GRMZM2G030877; transcript_id TCONS_00024983; exon_number 4; oId CUFF.21280.1; tss_id TSS22619;
4	Cufflinks	exon	188669075	188669117	.	-	.	gene_id GRMZM2G030877; transcript_id TCONS_00024983; exon_number 5; oId CUFF.21280.1; tss_id TSS22619;
4	Cufflinks	exon	188669384	188669461	.	-	.	gene_id GRMZM2G030877; transcript_id TCONS_00024983; exon_number 6; oId CUFF.21280.1; tss_id TSS22619;
4	Cufflinks	exon	188669566	188669625	.	-	.	gene_id GRMZM2G030877; transcript_id TCONS_00024983; exon_number 7; oId CUFF.21280.1; tss_id TSS22619;
4	Cufflinks	exon	188669784	188669834	.	-	.	gene_id GRMZM2G030877; transcript_id TCONS_00024983; exon_number 8; oId CUFF.21280.1; tss_id TSS22619;
4	Cufflinks	exon	188670920	188671072	.	-	.	gene_id GRMZM2G030877; transcript_id TCONS_00024983; exon_number 9; oId CUFF.21280.1; tss_id TSS22619;
4	Cufflinks	exon	188671232	188671347	.	-	.	gene_id GRMZM2G030877; transcript_id TCONS_00024983; exon_number 10; oId CUFF.21280.1; tss_id TSS22619;

4	Cufflinks	exon	188671831	188671955	.	-	.	gene_id GRMZM2G030877; transcript_id TCONS_00024983; exon_number 11; oId CUFF.21280.1; tss_id TSS22619;
4	Cufflinks	exon	188672268	188672443	.	-	.	gene_id GRMZM2G030877; transcript_id TCONS_00024983; exon_number 12; oId CUFF.21280.1; tss_id TSS22619;
4	Cufflinks	exon	188672555	188672956	.	-	.	gene_id GRMZM2G030877; transcript_id TCONS_00024983; exon_number 13; oId CUFF.21280.1; tss_id TSS22619;
4	Cufflinks	exon	188665523	188666118	.	-	.	gene_id GRMZM2G030877; transcript_id TCONS_00024984; exon_number 1; oId CUFF.21280.3; tss_id TSS22619;
4	Cufflinks	exon	188667536	188667678	.	-	.	gene_id GRMZM2G030877; transcript_id TCONS_00024984; exon_number 2; oId CUFF.21280.3; tss_id TSS22619;
4	Cufflinks	exon	188668233	188668469	.	-	.	gene_id GRMZM2G030877; transcript_id TCONS_00024984; exon_number 3; oId CUFF.21280.3; tss_id TSS22619;
4	Cufflinks	exon	188668690	188668961	.	-	.	gene_id GRMZM2G030877; transcript_id TCONS_00024984; exon_number 4; oId CUFF.21280.3; tss_id TSS22619;
4	Cufflinks	exon	188669075	188669117	.	-	.	gene_id GRMZM2G030877; transcript_id TCONS_00024984; exon_number 5; oId CUFF.21280.3; tss_id TSS22619;
4	Cufflinks	exon	188669384	188669461	.	-	.	gene_id GRMZM2G030877; transcript_id TCONS_00024984; exon_number 6; oId CUFF.21280.3; tss_id TSS22619;
4	Cufflinks	exon	188669566	188669625	.	-	.	gene_id GRMZM2G030877; transcript_id TCONS_00024984; exon_number 7; oId CUFF.21280.3; tss_id TSS22619;
4	Cufflinks	exon	188669784	188669834	.	-	.	gene_id GRMZM2G030877; transcript_id TCONS_00024984; exon_number 8; oId CUFF.21280.3; tss_id TSS22619;
4	Cufflinks	exon	188670920	188671072	.	-	.	gene_id GRMZM2G030877; transcript_id TCONS_00024984; exon_number 9; oId CUFF.21280.3; tss_id TSS22619;
4	Cufflinks	exon	188671232	188671555	.	-	.	gene_id GRMZM2G030877; transcript_id TCONS_00024984; exon_number 10; oId CUFF.21280.3; tss_id TSS22619;
4	Cufflinks	exon	188671831	188671955	.	-	.	gene_id GRMZM2G030877; transcript_id TCONS_00024984; exon_number 11; oId CUFF.21280.3; tss_id TSS22619;
4	Cufflinks	exon	188672268	188672437	.	-	.	gene_id GRMZM2G030877; transcript_id TCONS_00024984; exon_number 12; oId CUFF.21280.3; tss_id TSS22619;
4	Cufflinks	exon	188672555	188672994	.	-	.	gene_id GRMZM2G030877; transcript_id TCONS_00024984; exon_number 13; oId CUFF.21280.3; tss_id TSS22619;
4	Cufflinks	exon	188665523	188666118	.	-	.	gene_id GRMZM2G030877; transcript_id TCONS_00024985; exon_number 1; oId CUFF.21280.2; tss_id TSS22619;
4	Cufflinks	exon	188667536	188667678	.	-	.	gene_id GRMZM2G030877; transcript_id TCONS_00024985; exon_number 2; oId CUFF.21280.2; tss_id TSS22619;
4	Cufflinks	exon	188668233	188668469	.	-	.	gene_id GRMZM2G030877; transcript_id TCONS_00024985; exon_number 3; oId CUFF.21280.2; tss_id TSS22619;
4	Cufflinks	exon	188668690	188668961	.	-	.	gene_id GRMZM2G030877; transcript_id TCONS_00024985; exon_number 4; oId CUFF.21280.2; tss_id TSS22619;
4	Cufflinks	exon	188669075	188669117	.	-	.	gene_id GRMZM2G030877; transcript_id TCONS_00024985; exon_number 5; oId CUFF.21280.2; tss_id TSS22619;
4	Cufflinks	exon	188669384	188669461	.	-	.	gene_id GRMZM2G030877; transcript_id TCONS_00024985; exon_number 6; oId CUFF.21280.2; tss_id TSS22619;
4	Cufflinks	exon	188669566	188669625	.	-	.	gene_id GRMZM2G030877; transcript_id TCONS_00024985; exon_number 7; oId CUFF.21280.2; tss_id TSS22619;
4	Cufflinks	exon	188669784	188669834	.	-	.	gene_id GRMZM2G030877; transcript_id TCONS_00024985; exon_number 8; oId CUFF.21280.2; tss_id TSS22619;
4	Cufflinks	exon	188670920	188671072	.	-	.	gene_id GRMZM2G030877; transcript_id TCONS_00024985; exon_number 9; oId CUFF.21280.2; tss_id TSS22619;
4	Cufflinks	exon	188671232	188671350	.	-	.	gene_id GRMZM2G030877; transcript_id TCONS_00024985; exon_number 10; oId CUFF.21280.2; tss_id TSS22619;
4	Cufflinks	exon	188671831	188671955	.	-	.	gene_id GRMZM2G030877; transcript_id TCONS_00024985; exon_number 11; oId CUFF.21280.2; tss_id TSS22619;
4	Cufflinks	exon	188672268	188672437	.	-	.	gene_id GRMZM2G030877; transcript_id TCONS_00024985; exon_number 12; oId CUFF.21280.2; tss_id TSS22619;
4	Cufflinks	exon	188672555	188672996	.	-	.	gene_id GRMZM2G030877; transcript_id TCONS_00024985; exon_number 13; oId CUFF.21280.2; tss_id TSS22619;
4	Cufflinks	exon	195975252	195976446	.	-	.	gene_id GRMZM2G163671; transcript_id TCONS_00025081; exon_number 1; oId CUFF.21453.1; tss_id TSS22704;
4	Cufflinks	exon	195976640	195976755	.	-	.	gene_id GRMZM2G163671; transcript_id TCONS_00025081; exon_number 2; oId CUFF.21453.1; tss_id TSS22704;
4	Cufflinks	exon	195978542	195978687	.	-	.	gene_id GRMZM2G163671; transcript_id TCONS_00025081; exon_number 3; oId CUFF.21453.1; tss_id TSS22704;
4	Cufflinks	exon	195978774	195979088	.	-	.	gene_id GRMZM2G163671; transcript_id TCONS_00025081; exon_number 4; oId CUFF.21453.1; tss_id TSS22704;
4	Cufflinks	exon	198256542	198257411	.	-	.	gene_id GRMZM2G332522; transcript_id TCONS_00025111; exon_number 1; oId CUFF.21492.1; tss_id TSS22732;
4	Cufflinks	exon	198257507	198257719	.	-	.	gene_id GRMZM2G332522; transcript_id TCONS_00025111; exon_number 2; oId CUFF.21492.1; tss_id TSS22732;
4	Cufflinks	exon	206672898	206674203	.	-	.	gene_id GRMZM2G059706; transcript_id TCONS_00025209; exon_number 1; oId CUFF.21661.1; tss_id TSS22822;

4	Cufflinks	exon	207618649	207619251	.	-	.	gene_id XLOC_022084; transcript_id TCONS_00025211; exon_number 1; oId CUFF.21678.1; tss_id TSS22824;
4	Cufflinks	exon	209050229	209050827	.	-	.	gene_id GRMZM2G089856; transcript_id TCONS_00025230; exon_number 1; oId CUFF.21694.1; tss_id TSS22838;
4	Cufflinks	exon	209051244	209051586	.	-	.	gene_id GRMZM2G089856; transcript_id TCONS_00025230; exon_number 2; oId CUFF.21694.1; tss_id TSS22838;
4	Cufflinks	exon	209051704	209052176	.	-	.	gene_id GRMZM2G089856; transcript_id TCONS_00025230; exon_number 3; oId CUFF.21694.1; tss_id TSS22838;
4	Cufflinks	exon	215441250	215441753	.	-	.	gene_id GRMZM2G590333; transcript_id TCONS_00025278; exon_number 1; oId CUFF.21801.4; tss_id TSS22883;
4	Cufflinks	exon	215443128	215443236	.	-	.	gene_id GRMZM2G590333; transcript_id TCONS_00025278; exon_number 2; oId CUFF.21801.4; tss_id TSS22883;
4	Cufflinks	exon	215443559	215443713	.	-	.	gene_id GRMZM2G590333; transcript_id TCONS_00025278; exon_number 3; oId CUFF.21801.4; tss_id TSS22883;
4	Cufflinks	exon	215443823	215443862	.	-	.	gene_id GRMZM2G590333; transcript_id TCONS_00025278; exon_number 4; oId CUFF.21801.4; tss_id TSS22883;
4	Cufflinks	exon	215444220	215444272	.	-	.	gene_id GRMZM2G590333; transcript_id TCONS_00025278; exon_number 5; oId CUFF.21801.4; tss_id TSS22883;
4	Cufflinks	exon	215444405	215444478	.	-	.	gene_id GRMZM2G590333; transcript_id TCONS_00025278; exon_number 6; oId CUFF.21801.4; tss_id TSS22883;
4	Cufflinks	exon	215444589	215444671	.	-	.	gene_id GRMZM2G590333; transcript_id TCONS_00025278; exon_number 7; oId CUFF.21801.4; tss_id TSS22883;
4	Cufflinks	exon	215444821	215444889	.	-	.	gene_id GRMZM2G590333; transcript_id TCONS_00025278; exon_number 8; oId CUFF.21801.4; tss_id TSS22883;
4	Cufflinks	exon	215444990	215445772	.	-	.	gene_id GRMZM2G590333; transcript_id TCONS_00025278; exon_number 9; oId CUFF.21801.4; tss_id TSS22883;
4	Cufflinks	exon	215441250	215441753	.	-	.	gene_id GRMZM2G590333; transcript_id TCONS_00025281; exon_number 1; oId CUFF.21801.3; tss_id TSS22883;
4	Cufflinks	exon	215443128	215443224	.	-	.	gene_id GRMZM2G590333; transcript_id TCONS_00025281; exon_number 2; oId CUFF.21801.3; tss_id TSS22883;
4	Cufflinks	exon	215443559	215443713	.	-	.	gene_id GRMZM2G590333; transcript_id TCONS_00025281; exon_number 3; oId CUFF.21801.3; tss_id TSS22883;
4	Cufflinks	exon	215443823	215443862	.	-	.	gene_id GRMZM2G590333; transcript_id TCONS_00025281; exon_number 4; oId CUFF.21801.3; tss_id TSS22883;
4	Cufflinks	exon	215444220	215444275	.	-	.	gene_id GRMZM2G590333; transcript_id TCONS_00025281; exon_number 5; oId CUFF.21801.3; tss_id TSS22883;
4	Cufflinks	exon	215444405	215444478	.	-	.	gene_id GRMZM2G590333; transcript_id TCONS_00025281; exon_number 6; oId CUFF.21801.3; tss_id TSS22883;
4	Cufflinks	exon	215444589	215444671	.	-	.	gene_id GRMZM2G590333; transcript_id TCONS_00025281; exon_number 7; oId CUFF.21801.3; tss_id TSS22883;
4	Cufflinks	exon	215444821	215444889	.	-	.	gene_id GRMZM2G590333; transcript_id TCONS_00025281; exon_number 8; oId CUFF.21801.3; tss_id TSS22883;
4	Cufflinks	exon	215444990	215445850	.	-	.	gene_id GRMZM2G590333; transcript_id TCONS_00025281; exon_number 9; oId CUFF.21801.3; tss_id TSS22883;
4	Cufflinks	exon	215441250	215441753	.	-	.	gene_id GRMZM2G590333; transcript_id TCONS_00025279; exon_number 1; oId CUFF.21801.1; tss_id TSS22883;
4	Cufflinks	exon	215443128	215443224	.	-	.	gene_id GRMZM2G590333; transcript_id TCONS_00025279; exon_number 2; oId CUFF.21801.1; tss_id TSS22883;
4	Cufflinks	exon	215443559	215443713	.	-	.	gene_id GRMZM2G590333; transcript_id TCONS_00025279; exon_number 3; oId CUFF.21801.1; tss_id TSS22883;
4	Cufflinks	exon	215443823	215443862	.	-	.	gene_id GRMZM2G590333; transcript_id TCONS_00025279; exon_number 4; oId CUFF.21801.1; tss_id TSS22883;
4	Cufflinks	exon	215444220	215444272	.	-	.	gene_id GRMZM2G590333; transcript_id TCONS_00025279; exon_number 5; oId CUFF.21801.1; tss_id TSS22883;
4	Cufflinks	exon	215444405	215444478	.	-	.	gene_id GRMZM2G590333; transcript_id TCONS_00025279; exon_number 6; oId CUFF.21801.1; tss_id TSS22883;
4	Cufflinks	exon	215444589	215444671	.	-	.	gene_id GRMZM2G590333; transcript_id TCONS_00025279; exon_number 7; oId CUFF.21801.1; tss_id TSS22883;
4	Cufflinks	exon	215444821	215444889	.	-	.	gene_id GRMZM2G590333; transcript_id TCONS_00025279; exon_number 8; oId CUFF.21801.1; tss_id TSS22883;
4	Cufflinks	exon	215444990	215445850	.	-	.	gene_id GRMZM2G590333; transcript_id TCONS_00025279; exon_number 9; oId CUFF.21801.1; tss_id TSS22883;
4	Cufflinks	exon	225418052	225419587	.	-	.	gene_id XLOC_022215; transcript_id TCONS_00025363; exon_number 1; oId CUFF.21935.1; tss_id TSS22959;
4	Cufflinks	exon	226605254	226607090	.	-	.	gene_id GRMZM2G035077; transcript_id TCONS_00025383; exon_number 1; oId CUFF.21966.1; tss_id TSS22978;
4	Cufflinks	exon	226843625	226844335	.	-	.	gene_id XLOC_022237; transcript_id TCONS_00025386; exon_number 1; oId CUFF.21970.1; tss_id TSS22981;
4	Cufflinks	exon	235108555	235109163	.	-	.	gene_id GRMZM2G008859; transcript_id TCONS_00025507; exon_number 1; oId CUFF.22177.1; tss_id TSS23084;
4	Cufflinks	exon	235109780	235109983	.	-	.	gene_id GRMZM2G008859; transcript_id TCONS_00025507; exon_number 2; oId CUFF.22177.1; tss_id TSS23084;

4	Cufflinks	exon	235110419	235110676	.	-	.	gene_id GRMZM2G008859; transcript_id TCONS_00025507; exon_number 3; oId CUFF.22177.1; tss_id TSS23084;
4	Cufflinks	exon	235111520	235111831	.	-	.	gene_id GRMZM2G008859; transcript_id TCONS_00025507; exon_number 4; oId CUFF.22177.1; tss_id TSS23084;
4	Cufflinks	exon	236262710	236263037	.	-	.	gene_id XLOC_022369; transcript_id TCONS_00025540; exon_number 1; oId CUFF.22233.1; tss_id TSS23116;
4	Cufflinks	exon	236263086	236264243	.	-	.	gene_id XLOC_022369; transcript_id TCONS_00025540; exon_number 2; oId CUFF.22233.1; tss_id TSS23116;
4	Cufflinks	exon	237811718	237812263	.	-	.	gene_id GRMZM2G303374; transcript_id TCONS_00025583; exon_number 1; oId CUFF.22328.1; tss_id TSS23152;
4	Cufflinks	exon	237812374	237812427	.	-	.	gene_id GRMZM2G303374; transcript_id TCONS_00025583; exon_number 2; oId CUFF.22328.1; tss_id TSS23152;
4	Cufflinks	exon	237812518	237812588	.	-	.	gene_id GRMZM2G303374; transcript_id TCONS_00025583; exon_number 3; oId CUFF.22328.1; tss_id TSS23152;
4	Cufflinks	exon	237812668	237812744	.	-	.	gene_id GRMZM2G303374; transcript_id TCONS_00025583; exon_number 4; oId CUFF.22328.1; tss_id TSS23152;
4	Cufflinks	exon	237812844	237812927	.	-	.	gene_id GRMZM2G303374; transcript_id TCONS_00025583; exon_number 5; oId CUFF.22328.1; tss_id TSS23152;
4	Cufflinks	exon	237813045	237813201	.	-	.	gene_id GRMZM2G303374; transcript_id TCONS_00025583; exon_number 6; oId CUFF.22328.1; tss_id TSS23152;
4	Cufflinks	exon	238052080	238052677	.	-	.	gene_id GRMZM2G166035; transcript_id TCONS_00025589; exon_number 1; oId CUFF.22341.1; tss_id TSS23158;
4	Cufflinks	exon	238052762	238052836	.	-	.	gene_id GRMZM2G166035; transcript_id TCONS_00025589; exon_number 2; oId CUFF.22341.1; tss_id TSS23158;
4	Cufflinks	exon	238053024	238053115	.	-	.	gene_id GRMZM2G166035; transcript_id TCONS_00025589; exon_number 3; oId CUFF.22341.1; tss_id TSS23158;
4	Cufflinks	exon	238053213	238053299	.	-	.	gene_id GRMZM2G166035; transcript_id TCONS_00025589; exon_number 4; oId CUFF.22341.1; tss_id TSS23158;
4	Cufflinks	exon	238053598	238053756	.	-	.	gene_id GRMZM2G166035; transcript_id TCONS_00025589; exon_number 5; oId CUFF.22341.1; tss_id TSS23158;
4	Cufflinks	exon	238053849	238053947	.	-	.	gene_id GRMZM2G166035; transcript_id TCONS_00025589; exon_number 6; oId CUFF.22341.1; tss_id TSS23158;
4	Cufflinks	exon	238054070	238054203	.	-	.	gene_id GRMZM2G166035; transcript_id TCONS_00025589; exon_number 7; oId CUFF.22341.1; tss_id TSS23158;
4	Cufflinks	exon	238055198	238055542	.	-	.	gene_id GRMZM2G166035; transcript_id TCONS_00025589; exon_number 8; oId CUFF.22341.1; tss_id TSS23158;
4	Cufflinks	exon	239641083	239641380	.	-	.	gene_id XLOC_022459; transcript_id TCONS_00025650; exon_number 1; oId CUFF.22411.1; tss_id TSS23209;
4	Cufflinks	exon	240049949	240051035	.	-	.	gene_id GRMZM2G169998; transcript_id TCONS_00025680; exon_number 1; oId CUFF.22456.1; tss_id TSS23237;
4	Cufflinks	exon	240051138	240051605	.	-	.	gene_id GRMZM2G169998; transcript_id TCONS_00025680; exon_number 2; oId CUFF.22456.1; tss_id TSS23237;
4	Cufflinks	exon	240052938	240053153	.	-	.	gene_id GRMZM2G169998; transcript_id TCONS_00025680; exon_number 3; oId CUFF.22456.1; tss_id TSS23237;
4	Cufflinks	exon	240053345	240053626	.	-	.	gene_id GRMZM2G169998; transcript_id TCONS_00025680; exon_number 4; oId CUFF.22456.1; tss_id TSS23237;
5	Cufflinks	exon	812882	813298	.	+	.	gene_id GRMZM2G019866; transcript_id TCONS_00025740; exon_number 1; oId CUFF.22563.1; tss_id TSS23287;
5	Cufflinks	exon	813424	813546	.	+	.	gene_id GRMZM2G019866; transcript_id TCONS_00025740; exon_number 2; oId CUFF.22563.1; tss_id TSS23287;
5	Cufflinks	exon	813622	814072	.	+	.	gene_id GRMZM2G019866; transcript_id TCONS_00025740; exon_number 3; oId CUFF.22563.1; tss_id TSS23287;
5	Cufflinks	exon	922448	922706	.	+	.	gene_id GRMZM2G384070; transcript_id TCONS_00025754; exon_number 1; oId CUFF.22574.1; tss_id TSS23299;
5	Cufflinks	exon	923420	924190	.	+	.	gene_id GRMZM2G384070; transcript_id TCONS_00025754; exon_number 2; oId CUFF.22574.1; tss_id TSS23299;
5	Cufflinks	exon	1728200	1728663	.	+	.	gene_id GRMZM2G023081; transcript_id TCONS_00025802; exon_number 1; oId CUFF.22649.1; tss_id TSS23338;
5	Cufflinks	exon	1728768	1729396	.	+	.	gene_id GRMZM2G023081; transcript_id TCONS_00025802; exon_number 2; oId CUFF.22649.1; tss_id TSS23338;
5	Cufflinks	exon	1872661	1872921	.	+	.	gene_id GRMZM2G100524; transcript_id TCONS_00025805; exon_number 1; oId CUFF.22665.1; tss_id TSS23341;
5	Cufflinks	exon	1873064	1874479	.	+	.	gene_id GRMZM2G100524; transcript_id TCONS_00025805; exon_number 2; oId CUFF.22665.1; tss_id TSS23341;
5	Cufflinks	exon	1874610	1874643	.	+	.	gene_id GRMZM2G100524; transcript_id TCONS_00025805; exon_number 3; oId CUFF.22665.1; tss_id TSS23341;
5	Cufflinks	exon	1874791	1875805	.	+	.	gene_id GRMZM2G100524; transcript_id TCONS_00025805; exon_number 4; oId CUFF.22665.1; tss_id TSS23341;
5	Cufflinks	exon	2109630	2110118	.	+	.	gene_id GRMZM2G121715; transcript_id TCONS_00025811; exon_number 1; oId CUFF.22676.2; tss_id TSS23346;
5	Cufflinks	exon	2110233	2110435	.	+	.	gene_id GRMZM2G121715; transcript_id TCONS_00025811; exon_number 2; oId CUFF.22676.2; tss_id TSS23346;

5	Cufflinks	exon	2110525	2110681	.	+	.	gene_id GRMZM2G121715; transcript_id TCONS_00025811; exon_number 3; oId CUFF.22676.2; tss_id TSS23346;
5	Cufflinks	exon	2110894	2110983	.	+	.	gene_id GRMZM2G121715; transcript_id TCONS_00025811; exon_number 4; oId CUFF.22676.2; tss_id TSS23346;
5	Cufflinks	exon	2111151	2111449	.	+	.	gene_id GRMZM2G121715; transcript_id TCONS_00025811; exon_number 5; oId CUFF.22676.2; tss_id TSS23346;
5	Cufflinks	exon	2111532	2112133	.	+	.	gene_id GRMZM2G121715; transcript_id TCONS_00025811; exon_number 6; oId CUFF.22676.2; tss_id TSS23346;
5	Cufflinks	exon	3777982	3778189	.	+	.	gene_id GRMZM2G054224; transcript_id TCONS_00025887; exon_number 1; oId CUFF.22801.1; tss_id TSS23415;
5	Cufflinks	exon	3778293	3778350	.	+	.	gene_id GRMZM2G054224; transcript_id TCONS_00025887; exon_number 2; oId CUFF.22801.1; tss_id TSS23415;
5	Cufflinks	exon	3778477	3778581	.	+	.	gene_id GRMZM2G054224; transcript_id TCONS_00025887; exon_number 3; oId CUFF.22801.1; tss_id TSS23415;
5	Cufflinks	exon	3778806	3778863	.	+	.	gene_id GRMZM2G054224; transcript_id TCONS_00025887; exon_number 4; oId CUFF.22801.1; tss_id TSS23415;
5	Cufflinks	exon	3778951	3779126	.	+	.	gene_id GRMZM2G054224; transcript_id TCONS_00025887; exon_number 5; oId CUFF.22801.1; tss_id TSS23415;
5	Cufflinks	exon	3779214	3779274	.	+	.	gene_id GRMZM2G054224; transcript_id TCONS_00025887; exon_number 6; oId CUFF.22801.1; tss_id TSS23415;
5	Cufflinks	exon	3779361	3779876	.	+	.	gene_id GRMZM2G054224; transcript_id TCONS_00025887; exon_number 7; oId CUFF.22801.1; tss_id TSS23415;
5	Cufflinks	exon	3777988	3778189	.	+	.	gene_id GRMZM2G054224; transcript_id TCONS_00025888; exon_number 1; oId CUFF.22801.2; tss_id TSS23415;
5	Cufflinks	exon	3778293	3778350	.	+	.	gene_id GRMZM2G054224; transcript_id TCONS_00025888; exon_number 2; oId CUFF.22801.2; tss_id TSS23415;
5	Cufflinks	exon	3778477	3778863	.	+	.	gene_id GRMZM2G054224; transcript_id TCONS_00025888; exon_number 3; oId CUFF.22801.2; tss_id TSS23415;
5	Cufflinks	exon	3778951	3779126	.	+	.	gene_id GRMZM2G054224; transcript_id TCONS_00025888; exon_number 4; oId CUFF.22801.2; tss_id TSS23415;
5	Cufflinks	exon	3779214	3779274	.	+	.	gene_id GRMZM2G054224; transcript_id TCONS_00025888; exon_number 5; oId CUFF.22801.2; tss_id TSS23415;
5	Cufflinks	exon	3779361	3779876	.	+	.	gene_id GRMZM2G054224; transcript_id TCONS_00025888; exon_number 6; oId CUFF.22801.2; tss_id TSS23415;
5	Cufflinks	exon	4345261	4345941	.	+	.	gene_id GRMZM2G099615; transcript_id TCONS_00025909; exon_number 1; oId CUFF.22835.1; tss_id TSS23433;
5	Cufflinks	exon	4348846	4349288	.	+	.	gene_id GRMZM2G099615; transcript_id TCONS_00025909; exon_number 2; oId CUFF.22835.1; tss_id TSS23433;
5	Cufflinks	exon	4423409	4423537	.	+	.	gene_id GRMZM2G130544; transcript_id TCONS_00025910; exon_number 1; oId CUFF.22837.1; tss_id TSS23434;
5	Cufflinks	exon	4423626	4423786	.	+	.	gene_id GRMZM2G130544; transcript_id TCONS_00025910; exon_number 2; oId CUFF.22837.1; tss_id TSS23434;
5	Cufflinks	exon	4423877	4424065	.	+	.	gene_id GRMZM2G130544; transcript_id TCONS_00025910; exon_number 3; oId CUFF.22837.1; tss_id TSS23434;
5	Cufflinks	exon	4424992	4425429	.	+	.	gene_id GRMZM2G130544; transcript_id TCONS_00025910; exon_number 4; oId CUFF.22837.1; tss_id TSS23434;
5	Cufflinks	exon	7780703	7781216	.	+	.	gene_id GRMZM2G077356; transcript_id TCONS_00026021; exon_number 1; oId CUFF.23028.1; tss_id TSS23528;
5	Cufflinks	exon	7782211	7782461	.	+	.	gene_id GRMZM2G077356; transcript_id TCONS_00026021; exon_number 2; oId CUFF.23028.1; tss_id TSS23528;
5	Cufflinks	exon	7782615	7782729	.	+	.	gene_id GRMZM2G077356; transcript_id TCONS_00026021; exon_number 3; oId CUFF.23028.1; tss_id TSS23528;
5	Cufflinks	exon	7782837	7782898	.	+	.	gene_id GRMZM2G077356; transcript_id TCONS_00026021; exon_number 4; oId CUFF.23028.1; tss_id TSS23528;
5	Cufflinks	exon	7783007	7783409	.	+	.	gene_id GRMZM2G077356; transcript_id TCONS_00026021; exon_number 5; oId CUFF.23028.1; tss_id TSS23528;
5	Cufflinks	exon	7866128	7866541	.	+	.	gene_id GRMZM2G147809; transcript_id TCONS_00026025; exon_number 1; oId CUFF.23026.1; tss_id TSS23531;
5	Cufflinks	exon	7866652	7867055	.	+	.	gene_id GRMZM2G147809; transcript_id TCONS_00026025; exon_number 2; oId CUFF.23026.1; tss_id TSS23531;
5	Cufflinks	exon	7867194	7867244	.	+	.	gene_id GRMZM2G147809; transcript_id TCONS_00026025; exon_number 3; oId CUFF.23026.1; tss_id TSS23531;
5	Cufflinks	exon	7867582	7867654	.	+	.	gene_id GRMZM2G147809; transcript_id TCONS_00026025; exon_number 4; oId CUFF.23026.1; tss_id TSS23531;
5	Cufflinks	exon	7870252	7870805	.	+	.	gene_id GRMZM2G147809; transcript_id TCONS_00026025; exon_number 5; oId CUFF.23026.1; tss_id TSS23531;
5	Cufflinks	exon	9911089	9911491	.	+	.	gene_id GRMZM2G096596; transcript_id TCONS_00026063; exon_number 1; oId CUFF.23100.1; tss_id TSS23566;
5	Cufflinks	exon	9911790	9911961	.	+	.	gene_id GRMZM2G096596; transcript_id TCONS_00026063; exon_number 2; oId CUFF.23100.1; tss_id TSS23566;
5	Cufflinks	exon	9912051	9912115	.	+	.	gene_id GRMZM2G096596; transcript_id TCONS_00026063; exon_number 3; oId CUFF.23100.1; tss_id TSS23566;

5	Cufflinks	exon	9913449	9913770	.	+	.	gene_id GRMZM2G096596; transcript_id TCONS_00026063; exon_number 4; oId CUFF.23100.1; tss_id TSS23566;
5	Cufflinks	exon	9913857	9914025	.	+	.	gene_id GRMZM2G096596; transcript_id TCONS_00026063; exon_number 5; oId CUFF.23100.1; tss_id TSS23566;
5	Cufflinks	exon	9916148	9916257	.	+	.	gene_id GRMZM2G096596; transcript_id TCONS_00026063; exon_number 6; oId CUFF.23100.1; tss_id TSS23566;
5	Cufflinks	exon	9916328	9916383	.	+	.	gene_id GRMZM2G096596; transcript_id TCONS_00026063; exon_number 7; oId CUFF.23100.1; tss_id TSS23566;
5	Cufflinks	exon	9916473	9916529	.	+	.	gene_id GRMZM2G096596; transcript_id TCONS_00026063; exon_number 8; oId CUFF.23100.1; tss_id TSS23566;
5	Cufflinks	exon	9917360	9917503	.	+	.	gene_id GRMZM2G096596; transcript_id TCONS_00026063; exon_number 9; oId CUFF.23100.1; tss_id TSS23566;
5	Cufflinks	exon	9918618	9919240	.	+	.	gene_id GRMZM2G096596; transcript_id TCONS_00026063; exon_number 10; oId CUFF.23100.1; tss_id TSS23566;
5	Cufflinks	exon	18789107	18789626	.	+	.	gene_id GRMZM2G087326; transcript_id TCONS_00026249; exon_number 1; oId CUFF.23398.1; tss_id TSS23736;
5	Cufflinks	exon	18790592	18790842	.	+	.	gene_id GRMZM2G087326; transcript_id TCONS_00026249; exon_number 2; oId CUFF.23398.1; tss_id TSS23736;
5	Cufflinks	exon	18790929	18791097	.	+	.	gene_id GRMZM2G087326; transcript_id TCONS_00026249; exon_number 3; oId CUFF.23398.1; tss_id TSS23736;
5	Cufflinks	exon	18791203	18791842	.	+	.	gene_id GRMZM2G087326; transcript_id TCONS_00026249; exon_number 4; oId CUFF.23398.1; tss_id TSS23736;
5	Cufflinks	exon	22675530	22676644	.	+	.	gene_id GRMZM2G100158; transcript_id TCONS_00026303; exon_number 1; oId CUFF.23511.1; tss_id TSS23788;
5	Cufflinks	exon	22678008	22679029	.	+	.	gene_id GRMZM2G100158; transcript_id TCONS_00026303; exon_number 2; oId CUFF.23511.1; tss_id TSS23788;
5	Cufflinks	exon	23017949	23018871	.	+	.	gene_id GRMZM2G113135; transcript_id TCONS_00026306; exon_number 1; oId CUFF.23508.1; tss_id TSS23791;
5	Cufflinks	exon	24175048	24175557	.	+	.	gene_id GRMZM2G107444; transcript_id TCONS_00026330; exon_number 1; oId CUFF.23559.1; tss_id TSS23813;
5	Cufflinks	exon	24177470	24178212	.	+	.	gene_id GRMZM2G107444; transcript_id TCONS_00026330; exon_number 2; oId CUFF.23559.1; tss_id TSS23813;
5	Cufflinks	exon	30327205	30327906	.	+	.	gene_id XLOC_023106; transcript_id TCONS_00026400; exon_number 1; oId CUFF.23663.1; tss_id TSS23873;
5	Cufflinks	exon	31464389	31465555	.	+	.	gene_id GRMZM2G154954; transcript_id TCONS_00026413; exon_number 1; oId CUFF.23695.1; tss_id TSS23886;
5	Cufflinks	exon	31900737	31903637	.	+	.	gene_id GRMZM2G326707; transcript_id TCONS_00026416; exon_number 1; oId CUFF.23712.1; tss_id TSS23889;
5	Cufflinks	exon	32245099	32245891	.	+	.	gene_id GRMZM2G337048; transcript_id TCONS_00026418; exon_number 1; oId CUFF.23719.1; tss_id TSS23891;
5	Cufflinks	exon	32246008	32247118	.	+	.	gene_id GRMZM2G337048; transcript_id TCONS_00026418; exon_number 2; oId CUFF.23719.1; tss_id TSS23891;
5	Cufflinks	exon	35290711	35291061	.	+	.	gene_id GRMZM2G017805; transcript_id TCONS_00026459; exon_number 1; oId CUFF.23786.3; tss_id TSS23923;
5	Cufflinks	exon	35291249	35292911	.	+	.	gene_id GRMZM2G017805; transcript_id TCONS_00026459; exon_number 2; oId CUFF.23786.3; tss_id TSS23923;
5	Cufflinks	exon	35294305	35295535	.	+	.	gene_id GRMZM2G017805; transcript_id TCONS_00026459; exon_number 3; oId CUFF.23786.3; tss_id TSS23923;
5	Cufflinks	exon	39895036	39895398	.	+	.	gene_id GRMZM2G012209; transcript_id TCONS_00026504; exon_number 1; oId CUFF.23884.1; tss_id TSS23964;
5	Cufflinks	exon	39901037	39901154	.	+	.	gene_id GRMZM2G012209; transcript_id TCONS_00026504; exon_number 2; oId CUFF.23884.1; tss_id TSS23964;
5	Cufflinks	exon	39901739	39901798	.	+	.	gene_id GRMZM2G012209; transcript_id TCONS_00026504; exon_number 3; oId CUFF.23884.1; tss_id TSS23964;
5	Cufflinks	exon	39901881	39901940	.	+	.	gene_id GRMZM2G012209; transcript_id TCONS_00026504; exon_number 4; oId CUFF.23884.1; tss_id TSS23964;
5	Cufflinks	exon	39902035	39902111	.	+	.	gene_id GRMZM2G012209; transcript_id TCONS_00026504; exon_number 5; oId CUFF.23884.1; tss_id TSS23964;
5	Cufflinks	exon	39906004	39906590	.	+	.	gene_id GRMZM2G012209; transcript_id TCONS_00026504; exon_number 6; oId CUFF.23884.1; tss_id TSS23964;
5	Cufflinks	exon	48477760	48478483	.	+	.	gene_id GRMZM2G463913; transcript_id TCONS_00026568; exon_number 1; oId CUFF.23999.1; tss_id TSS24020;
5	Cufflinks	exon	48479920	48479958	.	+	.	gene_id GRMZM2G463913; transcript_id TCONS_00026568; exon_number 2; oId CUFF.23999.1; tss_id TSS24020;
5	Cufflinks	exon	48480047	48480274	.	+	.	gene_id GRMZM2G463913; transcript_id TCONS_00026568; exon_number 3; oId CUFF.23999.1; tss_id TSS24020;
5	Cufflinks	exon	48480410	48480813	.	+	.	gene_id GRMZM2G463913; transcript_id TCONS_00026568; exon_number 4; oId CUFF.23999.1; tss_id TSS24020;
5	Cufflinks	exon	48600490	48600908	.	+	.	gene_id GRMZM5G848124; transcript_id TCONS_00026570; exon_number 1; oId CUFF.23998.1; tss_id TSS24022;
5	Cufflinks	exon	48601166	48601830	.	+	.	gene_id GRMZM5G848124; transcript_id TCONS_00026570; exon_number 2; oId CUFF.23998.1; tss_id TSS24022;

5	Cufflinks	exon	48902473	48902877	.	+	.	gene_id GRMZM2G140443; transcript_id TCONS_00026573; exon_number 1; oId CUFF.24010.1; tss_id TSS24025;
5	Cufflinks	exon	48907419	48907517	.	+	.	gene_id GRMZM2G140443; transcript_id TCONS_00026573; exon_number 2; oId CUFF.24010.1; tss_id TSS24025;
5	Cufflinks	exon	48908517	48908567	.	+	.	gene_id GRMZM2G140443; transcript_id TCONS_00026573; exon_number 3; oId CUFF.24010.1; tss_id TSS24025;
5	Cufflinks	exon	48908700	48908823	.	+	.	gene_id GRMZM2G140443; transcript_id TCONS_00026573; exon_number 4; oId CUFF.24010.1; tss_id TSS24025;
5	Cufflinks	exon	48909462	48909580	.	+	.	gene_id GRMZM2G140443; transcript_id TCONS_00026573; exon_number 5; oId CUFF.24010.1; tss_id TSS24025;
5	Cufflinks	exon	48909679	48909780	.	+	.	gene_id GRMZM2G140443; transcript_id TCONS_00026573; exon_number 6; oId CUFF.24010.1; tss_id TSS24025;
5	Cufflinks	exon	48909857	48909931	.	+	.	gene_id GRMZM2G140443; transcript_id TCONS_00026573; exon_number 7; oId CUFF.24010.1; tss_id TSS24025;
5	Cufflinks	exon	48910051	48910127	.	+	.	gene_id GRMZM2G140443; transcript_id TCONS_00026573; exon_number 8; oId CUFF.24010.1; tss_id TSS24025;
5	Cufflinks	exon	48910327	48910576	.	+	.	gene_id GRMZM2G140443; transcript_id TCONS_00026573; exon_number 9; oId CUFF.24010.1; tss_id TSS24025;
5	Cufflinks	exon	48910724	48911068	.	+	.	gene_id GRMZM2G140443; transcript_id TCONS_00026573; exon_number 10; oId CUFF.24010.1; tss_id TSS24025;
5	Cufflinks	exon	48911596	48911724	.	+	.	gene_id GRMZM2G140443; transcript_id TCONS_00026573; exon_number 11; oId CUFF.24010.1; tss_id TSS24025;
5	Cufflinks	exon	48911793	48912285	.	+	.	gene_id GRMZM2G140443; transcript_id TCONS_00026573; exon_number 12; oId CUFF.24010.1; tss_id TSS24025;
5	Cufflinks	exon	49575119	49575569	.	+	.	gene_id GRMZM2G141858; transcript_id TCONS_00026580; exon_number 1; oId CUFF.24022.2; tss_id TSS24030;
5	Cufflinks	exon	49577301	49577760	.	+	.	gene_id GRMZM2G141858; transcript_id TCONS_00026580; exon_number 2; oId CUFF.24022.2; tss_id TSS24030;
5	Cufflinks	exon	50372099	50374372	.	+	.	gene_id GRMZM2G382785; transcript_id TCONS_00026589; exon_number 1; oId CUFF.24044.1; tss_id TSS24039;
5	Cufflinks	exon	59338041	59339527	.	+	.	gene_id GRMZM2G085117; transcript_id TCONS_00026679; exon_number 1; oId CUFF.24186.1; tss_id TSS24123;
5	Cufflinks	exon	59753812	59754297	.	+	.	gene_id GRMZM2G102447; transcript_id TCONS_00026690; exon_number 1; oId CUFF.24217.2; tss_id TSS24130;
5	Cufflinks	exon	59754422	59754606	.	+	.	gene_id GRMZM2G102447; transcript_id TCONS_00026690; exon_number 2; oId CUFF.24217.2; tss_id TSS24130;
5	Cufflinks	exon	59754711	59754951	.	+	.	gene_id GRMZM2G102447; transcript_id TCONS_00026690; exon_number 3; oId CUFF.24217.2; tss_id TSS24130;
5	Cufflinks	exon	59755852	59755988	.	+	.	gene_id GRMZM2G102447; transcript_id TCONS_00026690; exon_number 4; oId CUFF.24217.2; tss_id TSS24130;
5	Cufflinks	exon	59756087	59756561	.	+	.	gene_id GRMZM2G102447; transcript_id TCONS_00026690; exon_number 5; oId CUFF.24217.2; tss_id TSS24130;
5	Cufflinks	exon	59753812	59754303	.	+	.	gene_id GRMZM2G102447; transcript_id TCONS_00026689; exon_number 1; oId CUFF.24217.1; tss_id TSS24130;
5	Cufflinks	exon	59754422	59754606	.	+	.	gene_id GRMZM2G102447; transcript_id TCONS_00026689; exon_number 2; oId CUFF.24217.1; tss_id TSS24130;
5	Cufflinks	exon	59754711	59754951	.	+	.	gene_id GRMZM2G102447; transcript_id TCONS_00026689; exon_number 3; oId CUFF.24217.1; tss_id TSS24130;
5	Cufflinks	exon	59755852	59755988	.	+	.	gene_id GRMZM2G102447; transcript_id TCONS_00026689; exon_number 4; oId CUFF.24217.1; tss_id TSS24130;
5	Cufflinks	exon	59756087	59756561	.	+	.	gene_id GRMZM2G102447; transcript_id TCONS_00026689; exon_number 5; oId CUFF.24217.1; tss_id TSS24130;
5	Cufflinks	exon	61455057	61455153	.	+	.	gene_id XLOC_023371; transcript_id TCONS_00026710; exon_number 1; oId CUFF.24264.2; tss_id TSS24148;
5	Cufflinks	exon	61455341	61455410	.	+	.	gene_id XLOC_023371; transcript_id TCONS_00026710; exon_number 2; oId CUFF.24264.2; tss_id TSS24148;
5	Cufflinks	exon	61455483	61456050	.	+	.	gene_id XLOC_023371; transcript_id TCONS_00026710; exon_number 3; oId CUFF.24264.2; tss_id TSS24148;
5	Cufflinks	exon	61456233	61456309	.	+	.	gene_id XLOC_023371; transcript_id TCONS_00026710; exon_number 4; oId CUFF.24264.2; tss_id TSS24148;
5	Cufflinks	exon	61456417	61456461	.	+	.	gene_id XLOC_023371; transcript_id TCONS_00026710; exon_number 5; oId CUFF.24264.2; tss_id TSS24148;
5	Cufflinks	exon	61456600	61457358	.	+	.	gene_id XLOC_023371; transcript_id TCONS_00026710; exon_number 6; oId CUFF.24264.2; tss_id TSS24148;
5	Cufflinks	exon	62142767	62142904	.	+	.	gene_id XLOC_023383; transcript_id TCONS_00026725; exon_number 1; oId CUFF.24300.1; tss_id TSS24161;
5	Cufflinks	exon	62143022	62143464	.	+	.	gene_id XLOC_023383; transcript_id TCONS_00026725; exon_number 2; oId CUFF.24300.1; tss_id TSS24161;
5	Cufflinks	exon	67432374	67433147	.	+	.	gene_id GRMZM2G128929; transcript_id TCONS_00026778; exon_number 1; oId CUFF.24378.1; tss_id TSS24210;
5	Cufflinks	exon	67433443	67434191	.	+	.	gene_id GRMZM2G128929; transcript_id TCONS_00026778; exon_number 2; oId CUFF.24378.1; tss_id TSS24210;

5	Cufflinks	exon	70111506	70111734	.	+	.	gene_id GRMZM2G156824; transcript_id TCONS_00026820; exon_number 1; oId CUFF.24463.1; tss_id TSS24248;
5	Cufflinks	exon	70112195	70112577	.	+	.	gene_id GRMZM2G156824; transcript_id TCONS_00026820; exon_number 2; oId CUFF.24463.1; tss_id TSS24248;
5	Cufflinks	exon	70113419	70113554	.	+	.	gene_id GRMZM2G156824; transcript_id TCONS_00026820; exon_number 3; oId CUFF.24463.1; tss_id TSS24248;
5	Cufflinks	exon	70114340	70114476	.	+	.	gene_id GRMZM2G156824; transcript_id TCONS_00026820; exon_number 4; oId CUFF.24463.1; tss_id TSS24248;
5	Cufflinks	exon	70114561	70114684	.	+	.	gene_id GRMZM2G156824; transcript_id TCONS_00026820; exon_number 5; oId CUFF.24463.1; tss_id TSS24248;
5	Cufflinks	exon	70114842	70115642	.	+	.	gene_id GRMZM2G156824; transcript_id TCONS_00026820; exon_number 6; oId CUFF.24463.1; tss_id TSS24248;
5	Cufflinks	exon	73076939	73077564	.	+	.	gene_id GRMZM2G029879; transcript_id TCONS_00026852; exon_number 1; oId CUFF.24498.1; tss_id TSS24280;
5	Cufflinks	exon	73078358	73079038	.	+	.	gene_id GRMZM2G029879; transcript_id TCONS_00026852; exon_number 2; oId CUFF.24498.1; tss_id TSS24280;
5	Cufflinks	exon	76240628	76241173	.	+	.	gene_id GRMZM6G869379; transcript_id TCONS_00026880; exon_number 1; oId CUFF.24550.1; tss_id TSS24306;
5	Cufflinks	exon	76242673	76243376	.	+	.	gene_id GRMZM6G869379; transcript_id TCONS_00026880; exon_number 2; oId CUFF.24550.1; tss_id TSS24306;
5	Cufflinks	exon	77949479	77950640	.	+	.	gene_id GRMZM2G097207; transcript_id TCONS_00026913; exon_number 1; oId CUFF.24614.1; tss_id TSS24338;
5	Cufflinks	exon	77953066	77954635	.	+	.	gene_id GRMZM2G097207; transcript_id TCONS_00026913; exon_number 2; oId CUFF.24614.1; tss_id TSS24338;
5	Cufflinks	exon	83895830	83897179	.	+	.	gene_id GRMZM2G088396; transcript_id TCONS_00026979; exon_number 1; oId CUFF.24733.1; tss_id TSS24397;
5	Cufflinks	exon	83897587	83898120	.	+	.	gene_id GRMZM2G088396; transcript_id TCONS_00026979; exon_number 2; oId CUFF.24733.1; tss_id TSS24397;
5	Cufflinks	exon	84097690	84098838	.	+	.	gene_id GRMZM2G322129; transcript_id TCONS_00026983; exon_number 1; oId CUFF.24726.2; tss_id TSS24400;
5	Cufflinks	exon	84098926	84099073	.	+	.	gene_id GRMZM2G322129; transcript_id TCONS_00026983; exon_number 2; oId CUFF.24726.2; tss_id TSS24400;
5	Cufflinks	exon	84099636	84100625	.	+	.	gene_id GRMZM2G322129; transcript_id TCONS_00026983; exon_number 3; oId CUFF.24726.2; tss_id TSS24400;
5	Cufflinks	exon	84100740	84100884	.	+	.	gene_id GRMZM2G322129; transcript_id TCONS_00026983; exon_number 4; oId CUFF.24726.2; tss_id TSS24400;
5	Cufflinks	exon	84100970	84101221	.	+	.	gene_id GRMZM2G322129; transcript_id TCONS_00026983; exon_number 5; oId CUFF.24726.2; tss_id TSS24400;
5	Cufflinks	exon	84101320	84101629	.	+	.	gene_id GRMZM2G322129; transcript_id TCONS_00026983; exon_number 6; oId CUFF.24726.2; tss_id TSS24400;
5	Cufflinks	exon	84101721	84102291	.	+	.	gene_id GRMZM2G322129; transcript_id TCONS_00026983; exon_number 7; oId CUFF.24726.2; tss_id TSS24400;
5	Cufflinks	exon	86185411	86185528	.	+	.	gene_id GRMZM2G180218; transcript_id TCONS_00026999; exon_number 1; oId CUFF.24750.1; tss_id TSS24416;
5	Cufflinks	exon	86186121	86186569	.	+	.	gene_id GRMZM2G180218; transcript_id TCONS_00026999; exon_number 2; oId CUFF.24750.1; tss_id TSS24416;
5	Cufflinks	exon	92390468	92391015	.	+	.	gene_id GRMZM2G057328; transcript_id TCONS_00027037; exon_number 1; oId CUFF.24825.1; tss_id TSS24448;
5	Cufflinks	exon	92391203	92391360	.	+	.	gene_id GRMZM2G057328; transcript_id TCONS_00027037; exon_number 2; oId CUFF.24825.1; tss_id TSS24448;
5	Cufflinks	exon	92391490	92391675	.	+	.	gene_id GRMZM2G057328; transcript_id TCONS_00027037; exon_number 3; oId CUFF.24825.1; tss_id TSS24448;
5	Cufflinks	exon	92391793	92392148	.	+	.	gene_id GRMZM2G057328; transcript_id TCONS_00027037; exon_number 4; oId CUFF.24825.1; tss_id TSS24448;
5	Cufflinks	exon	92393050	92393209	.	+	.	gene_id GRMZM2G057328; transcript_id TCONS_00027037; exon_number 5; oId CUFF.24825.1; tss_id TSS24448;
5	Cufflinks	exon	92393316	92393828	.	+	.	gene_id GRMZM2G057328; transcript_id TCONS_00027037; exon_number 6; oId CUFF.24825.1; tss_id TSS24448;
5	Cufflinks	exon	118710432	118710838	.	+	.	gene_id XLOC_023749; transcript_id TCONS_00027133; exon_number 1; oId CUFF.25016.1; tss_id TSS24542;
5	Cufflinks	exon	122676304	122676667	.	+	.	gene_id GRMZM2G029979; transcript_id TCONS_00027148; exon_number 1; oId CUFF.25049.1; tss_id TSS24556;
5	Cufflinks	exon	122677067	122677182	.	+	.	gene_id GRMZM2G029979; transcript_id TCONS_00027148; exon_number 2; oId CUFF.25049.1; tss_id TSS24556;
5	Cufflinks	exon	122678260	122678318	.	+	.	gene_id GRMZM2G029979; transcript_id TCONS_00027148; exon_number 3; oId CUFF.25049.1; tss_id TSS24556;
5	Cufflinks	exon	122678472	122678621	.	+	.	gene_id GRMZM2G029979; transcript_id TCONS_00027148; exon_number 4; oId CUFF.25049.1; tss_id TSS24556;
5	Cufflinks	exon	122678739	122678891	.	+	.	gene_id GRMZM2G029979; transcript_id TCONS_00027148; exon_number 5; oId CUFF.25049.1; tss_id TSS24556;
5	Cufflinks	exon	122679036	122679113	.	+	.	gene_id GRMZM2G029979; transcript_id TCONS_00027148; exon_number 6; oId CUFF.25049.1; tss_id TSS24556;

5	Cufflinks	exon	122679677	122679734	.	+	.	gene_id GRMZM2G029979; transcript_id TCONS_00027148; exon_number 7; oId CUFF.25049.1; tss_id TSS24556;
5	Cufflinks	exon	122679928	122680232	.	+	.	gene_id GRMZM2G029979; transcript_id TCONS_00027148; exon_number 8; oId CUFF.25049.1; tss_id TSS24556;
5	Cufflinks	exon	122688320	122688568	.	+	.	gene_id GRMZM2G029979; transcript_id TCONS_00027148; exon_number 9; oId CUFF.25049.1; tss_id TSS24556;
5	Cufflinks	exon	122688657	122689150	.	+	.	gene_id GRMZM2G029979; transcript_id TCONS_00027148; exon_number 10; oId CUFF.25049.1; tss_id TSS24556;
5	Cufflinks	exon	123239697	123240188	.	+	.	gene_id GRMZM2G009479; transcript_id TCONS_00027150; exon_number 1; oId CUFF.25034.1; tss_id TSS24558;
5	Cufflinks	exon	123240301	123240566	.	+	.	gene_id GRMZM2G009479; transcript_id TCONS_00027150; exon_number 2; oId CUFF.25034.1; tss_id TSS24558;
5	Cufflinks	exon	123240679	123241240	.	+	.	gene_id GRMZM2G009479; transcript_id TCONS_00027150; exon_number 3; oId CUFF.25034.1; tss_id TSS24558;
5	Cufflinks	exon	123241356	123243181	.	+	.	gene_id GRMZM2G009479; transcript_id TCONS_00027150; exon_number 4; oId CUFF.25034.1; tss_id TSS24558;
5	Cufflinks	exon	146924241	146924547	.	+	.	gene_id XLOC_023893; transcript_id TCONS_00027286; exon_number 1; oId CUFF.25297.1; tss_id TSS24688;
5	Cufflinks	exon	146924695	146924930	.	+	.	gene_id XLOC_023893; transcript_id TCONS_00027286; exon_number 2; oId CUFF.25297.1; tss_id TSS24688;
5	Cufflinks	exon	155564693	155565005	.	+	.	gene_id GRMZM2G318633; transcript_id TCONS_00027326; exon_number 1; oId CUFF.25384.2; tss_id TSS24724;
5	Cufflinks	exon	155565268	155565385	.	+	.	gene_id GRMZM2G318633; transcript_id TCONS_00027326; exon_number 2; oId CUFF.25384.2; tss_id TSS24724;
5	Cufflinks	exon	155565578	155565998	.	+	.	gene_id GRMZM2G318633; transcript_id TCONS_00027326; exon_number 3; oId CUFF.25384.2; tss_id TSS24724;
5	Cufflinks	exon	160693221	160693899	.	+	.	gene_id GRMZM2G012046; transcript_id TCONS_00027380; exon_number 1; oId CUFF.25474.1; tss_id TSS24774;
5	Cufflinks	exon	160696189	160696427	.	+	.	gene_id GRMZM2G012046; transcript_id TCONS_00027380; exon_number 2; oId CUFF.25474.1; tss_id TSS24774;
5	Cufflinks	exon	160697005	160697206	.	+	.	gene_id GRMZM2G012046; transcript_id TCONS_00027380; exon_number 3; oId CUFF.25474.1; tss_id TSS24774;
5	Cufflinks	exon	160697285	160697365	.	+	.	gene_id GRMZM2G012046; transcript_id TCONS_00027380; exon_number 4; oId CUFF.25474.1; tss_id TSS24774;
5	Cufflinks	exon	160697450	160697633	.	+	.	gene_id GRMZM2G012046; transcript_id TCONS_00027380; exon_number 5; oId CUFF.25474.1; tss_id TSS24774;
5	Cufflinks	exon	160697770	160697936	.	+	.	gene_id GRMZM2G012046; transcript_id TCONS_00027380; exon_number 6; oId CUFF.25474.1; tss_id TSS24774;
5	Cufflinks	exon	160698204	160699305	.	+	.	gene_id GRMZM2G012046; transcript_id TCONS_00027380; exon_number 7; oId CUFF.25474.1; tss_id TSS24774;
5	Cufflinks	exon	164769971	164770205	.	+	.	gene_id XLOC_024036; transcript_id TCONS_00027443; exon_number 1; oId CUFF.25573.3; tss_id TSS24832;
5	Cufflinks	exon	164770640	164771166	.	+	.	gene_id XLOC_024036; transcript_id TCONS_00027443; exon_number 2; oId CUFF.25573.3; tss_id TSS24832;
5	Cufflinks	exon	167468740	167469535	.	+	.	gene_id XLOC_024053; transcript_id TCONS_00027461; exon_number 1; oId CUFF.25590.1; tss_id TSS24850;
5	Cufflinks	exon	169497565	169497876	.	+	.	gene_id GRMZM2G139300; transcript_id TCONS_00027489; exon_number 1; oId CUFF.25661.1; tss_id TSS24876;
5	Cufflinks	exon	169498511	169498519	.	+	.	gene_id GRMZM2G139300; transcript_id TCONS_00027489; exon_number 2; oId CUFF.25661.1; tss_id TSS24876;
5	Cufflinks	exon	169499087	169499949	.	+	.	gene_id GRMZM2G139300; transcript_id TCONS_00027489; exon_number 3; oId CUFF.25661.1; tss_id TSS24876;
5	Cufflinks	exon	169500212	169500370	.	+	.	gene_id GRMZM2G139300; transcript_id TCONS_00027489; exon_number 4; oId CUFF.25661.1; tss_id TSS24876;
5	Cufflinks	exon	169500996	169501258	.	+	.	gene_id GRMZM2G139300; transcript_id TCONS_00027489; exon_number 5; oId CUFF.25661.1; tss_id TSS24876;
5	Cufflinks	exon	169501373	169501463	.	+	.	gene_id GRMZM2G139300; transcript_id TCONS_00027489; exon_number 6; oId CUFF.25661.1; tss_id TSS24876;
5	Cufflinks	exon	169501555	169502117	.	+	.	gene_id GRMZM2G139300; transcript_id TCONS_00027489; exon_number 7; oId CUFF.25661.1; tss_id TSS24876;
5	Cufflinks	exon	170544174	170544600	.	+	.	gene_id XLOC_024087; transcript_id TCONS_00027500; exon_number 1; oId CUFF.25678.1; tss_id TSS24885;
5	Cufflinks	exon	172849643	172849890	.	+	.	gene_id GRMZM2G119566; transcript_id TCONS_00027529; exon_number 1; oId CUFF.25753.1; tss_id TSS24911;
5	Cufflinks	exon	172850064	172850123	.	+	.	gene_id GRMZM2G119566; transcript_id TCONS_00027529; exon_number 2; oId CUFF.25753.1; tss_id TSS24911;
5	Cufflinks	exon	172850243	172850325	.	+	.	gene_id GRMZM2G119566; transcript_id TCONS_00027529; exon_number 3; oId CUFF.25753.1; tss_id TSS24911;
5	Cufflinks	exon	172850521	172850674	.	+	.	gene_id GRMZM2G119566; transcript_id TCONS_00027529; exon_number 4; oId CUFF.25753.1; tss_id TSS24911;
5	Cufflinks	exon	172850783	172850912	.	+	.	gene_id GRMZM2G119566; transcript_id TCONS_00027529; exon_number 5; oId CUFF.25753.1; tss_id TSS24911;

5	Cufflinks	exon	172850995	172851153	.	+	.	gene_id GRMZM2G119566; transcript_id TCONS_00027529; exon_number 6; oId CUFF.25753.1; tss_id TSS24911;
5	Cufflinks	exon	172851237	172851415	.	+	.	gene_id GRMZM2G119566; transcript_id TCONS_00027529; exon_number 7; oId CUFF.25753.1; tss_id TSS24911;
5	Cufflinks	exon	172851649	172851729	.	+	.	gene_id GRMZM2G119566; transcript_id TCONS_00027529; exon_number 8; oId CUFF.25753.1; tss_id TSS24911;
5	Cufflinks	exon	172851846	172851998	.	+	.	gene_id GRMZM2G119566; transcript_id TCONS_00027529; exon_number 9; oId CUFF.25753.1; tss_id TSS24911;
5	Cufflinks	exon	172852089	172852744	.	+	.	gene_id GRMZM2G119566; transcript_id TCONS_00027529; exon_number 10; oId CUFF.25753.1; tss_id TSS24911;
5	Cufflinks	exon	174664668	174665015	.	+	.	gene_id XLOC_024137; transcript_id TCONS_00027562; exon_number 1; oId CUFF.25779.1; tss_id TSS24939;
5	Cufflinks	exon	176656120	176656714	.	+	.	gene_id GRMZM2G132706; transcript_id TCONS_00027599; exon_number 1; oId CUFF.25837.1; tss_id TSS24971;
5	Cufflinks	exon	176656799	176658005	.	+	.	gene_id GRMZM2G132706; transcript_id TCONS_00027599; exon_number 2; oId CUFF.25837.1; tss_id TSS24971;
5	Cufflinks	exon	180233335	180233589	.	+	.	gene_id GRMZM2G044359; transcript_id TCONS_00027648; exon_number 1; oId CUFF.25925.1; tss_id TSS25017;
5	Cufflinks	exon	180233672	180233741	.	+	.	gene_id GRMZM2G044359; transcript_id TCONS_00027648; exon_number 2; oId CUFF.25925.1; tss_id TSS25017;
5	Cufflinks	exon	180234072	180235265	.	+	.	gene_id GRMZM2G044359; transcript_id TCONS_00027648; exon_number 3; oId CUFF.25925.1; tss_id TSS25017;
5	Cufflinks	exon	180235410	180236245	.	+	.	gene_id GRMZM2G044359; transcript_id TCONS_00027648; exon_number 4; oId CUFF.25925.1; tss_id TSS25017;
5	Cufflinks	exon	182568960	182569502	.	+	.	gene_id GRMZM2G129189; transcript_id TCONS_00027694; exon_number 1; oId CUFF.25993.1; tss_id TSS25058;
5	Cufflinks	exon	182569571	182570172	.	+	.	gene_id GRMZM2G129189; transcript_id TCONS_00027694; exon_number 2; oId CUFF.25993.1; tss_id TSS25058;
5	Cufflinks	exon	185696130	185696413	.	+	.	gene_id GRMZM2G028830; transcript_id TCONS_00027737; exon_number 1; oId CUFF.26073.1; tss_id TSS25095;
5	Cufflinks	exon	185696505	185697035	.	+	.	gene_id GRMZM2G028830; transcript_id TCONS_00027737; exon_number 2; oId CUFF.26073.1; tss_id TSS25095;
5	Cufflinks	exon	189857312	189857457	.	+	.	gene_id XLOC_024338; transcript_id TCONS_00027795; exon_number 1; oId CUFF.26181.1; tss_id TSS25147;
5	Cufflinks	exon	189858567	189858886	.	+	.	gene_id XLOC_024338; transcript_id TCONS_00027795; exon_number 2; oId CUFF.26181.1; tss_id TSS25147;
5	Cufflinks	exon	191419331	191420180	.	+	.	gene_id GRMZM5G840002; transcript_id TCONS_00027819; exon_number 1; oId CUFF.26241.1; tss_id TSS25170;
5	Cufflinks	exon	191422386	191422728	.	+	.	gene_id GRMZM5G840002; transcript_id TCONS_00027819; exon_number 2; oId CUFF.26241.1; tss_id TSS25170;
5	Cufflinks	exon	191419340	191420180	.	+	.	gene_id GRMZM5G840002; transcript_id TCONS_00027820; exon_number 1; oId CUFF.26241.2; tss_id TSS25170;
5	Cufflinks	exon	191422381	191422728	.	+	.	gene_id GRMZM5G840002; transcript_id TCONS_00027820; exon_number 2; oId CUFF.26241.2; tss_id TSS25170;
5	Cufflinks	exon	194886364	194886750	.	+	.	gene_id XLOC_024415; transcript_id TCONS_00027881; exon_number 1; oId CUFF.26332.1; tss_id TSS25226;
5	Cufflinks	exon	194886837	194887629	.	+	.	gene_id XLOC_024415; transcript_id TCONS_00027881; exon_number 2; oId CUFF.26332.1; tss_id TSS25226;
5	Cufflinks	exon	195239858	195240324	.	+	.	gene_id GRMZM2G154628; transcript_id TCONS_00027883; exon_number 1; oId CUFF.26326.1; tss_id TSS25228;
5	Cufflinks	exon	195240449	195240744	.	+	.	gene_id GRMZM2G154628; transcript_id TCONS_00027883; exon_number 2; oId CUFF.26326.1; tss_id TSS25228;
5	Cufflinks	exon	195241811	195241951	.	+	.	gene_id GRMZM2G154628; transcript_id TCONS_00027883; exon_number 3; oId CUFF.26326.1; tss_id TSS25228;
5	Cufflinks	exon	195242277	195242631	.	+	.	gene_id GRMZM2G154628; transcript_id TCONS_00027883; exon_number 4; oId CUFF.26326.1; tss_id TSS25228;
5	Cufflinks	exon	200487573	200488124	.	+	.	gene_id GRMZM2G325019; transcript_id TCONS_00027970; exon_number 1; oId CUFF.26468.1; tss_id TSS25303;
5	Cufflinks	exon	200489012	200491201	.	+	.	gene_id GRMZM2G325019; transcript_id TCONS_00027970; exon_number 2; oId CUFF.26468.1; tss_id TSS25303;
5	Cufflinks	exon	200990577	200991072	.	+	.	gene_id GRMZM2G101287; transcript_id TCONS_00027983; exon_number 1; oId CUFF.26497.1; tss_id TSS25315;
5	Cufflinks	exon	200991609	200991688	.	+	.	gene_id GRMZM2G101287; transcript_id TCONS_00027983; exon_number 2; oId CUFF.26497.1; tss_id TSS25315;
5	Cufflinks	exon	200991931	200992049	.	+	.	gene_id GRMZM2G101287; transcript_id TCONS_00027983; exon_number 3; oId CUFF.26497.1; tss_id TSS25315;
5	Cufflinks	exon	200993313	200993389	.	+	.	gene_id GRMZM2G101287; transcript_id TCONS_00027983; exon_number 4; oId CUFF.26497.1; tss_id TSS25315;
5	Cufflinks	exon	200993624	200993961	.	+	.	gene_id GRMZM2G101287; transcript_id TCONS_00027983; exon_number 5; oId CUFF.26497.1; tss_id TSS25315;
5	Cufflinks	exon	201721167	201721358	.	+	.	gene_id GRMZM2G170602; transcript_id TCONS_00028000; exon_number 1; oId CUFF.26532.1; tss_id TSS25332;

5	Cufflinks	exon	201721477	201721648	.	+	.	gene_id GRMZM2G170602; transcript_id TCONS_00028000; exon_number 2; oId CUFF.26532.1; tss_id TSS25332;
5	Cufflinks	exon	201721754	201722294	.	+	.	gene_id GRMZM2G170602; transcript_id TCONS_00028000; exon_number 3; oId CUFF.26532.1; tss_id TSS25332;
5	Cufflinks	exon	201722409	201722533	.	+	.	gene_id GRMZM2G170602; transcript_id TCONS_00028000; exon_number 4; oId CUFF.26532.1; tss_id TSS25332;
5	Cufflinks	exon	201722714	201722826	.	+	.	gene_id GRMZM2G170602; transcript_id TCONS_00028000; exon_number 5; oId CUFF.26532.1; tss_id TSS25332;
5	Cufflinks	exon	201722933	201723705	.	+	.	gene_id GRMZM2G170602; transcript_id TCONS_00028000; exon_number 6; oId CUFF.26532.1; tss_id TSS25332;
5	Cufflinks	exon	202034268	202034410	.	+	.	gene_id GRMZM2G315176; transcript_id TCONS_00028006; exon_number 1; oId CUFF.26557.1; tss_id TSS25338;
5	Cufflinks	exon	202034538	202034649	.	+	.	gene_id GRMZM2G315176; transcript_id TCONS_00028006; exon_number 2; oId CUFF.26557.1; tss_id TSS25338;
5	Cufflinks	exon	202035383	202035877	.	+	.	gene_id GRMZM2G315176; transcript_id TCONS_00028006; exon_number 3; oId CUFF.26557.1; tss_id TSS25338;
5	Cufflinks	exon	202035963	202036075	.	+	.	gene_id GRMZM2G315176; transcript_id TCONS_00028006; exon_number 4; oId CUFF.26557.1; tss_id TSS25338;
5	Cufflinks	exon	202037737	202037968	.	+	.	gene_id GRMZM2G315176; transcript_id TCONS_00028006; exon_number 5; oId CUFF.26557.1; tss_id TSS25338;
5	Cufflinks	exon	202038477	202038640	.	+	.	gene_id GRMZM2G315176; transcript_id TCONS_00028006; exon_number 6; oId CUFF.26557.1; tss_id TSS25338;
5	Cufflinks	exon	202039036	202039117	.	+	.	gene_id GRMZM2G315176; transcript_id TCONS_00028006; exon_number 7; oId CUFF.26557.1; tss_id TSS25338;
5	Cufflinks	exon	202041025	202041114	.	+	.	gene_id GRMZM2G315176; transcript_id TCONS_00028006; exon_number 8; oId CUFF.26557.1; tss_id TSS25338;
5	Cufflinks	exon	202041541	202041774	.	+	.	gene_id GRMZM2G315176; transcript_id TCONS_00028006; exon_number 9; oId CUFF.26557.1; tss_id TSS25338;
5	Cufflinks	exon	202047321	202047392	.	+	.	gene_id GRMZM2G315176; transcript_id TCONS_00028006; exon_number 10; oId CUFF.26557.1; tss_id TSS25338;
5	Cufflinks	exon	202047562	202047711	.	+	.	gene_id GRMZM2G315176; transcript_id TCONS_00028006; exon_number 11; oId CUFF.26557.1; tss_id TSS25338;
5	Cufflinks	exon	202047799	202047942	.	+	.	gene_id GRMZM2G315176; transcript_id TCONS_00028006; exon_number 12; oId CUFF.26557.1; tss_id TSS25338;
5	Cufflinks	exon	202048265	202048423	.	+	.	gene_id GRMZM2G315176; transcript_id TCONS_00028006; exon_number 13; oId CUFF.26557.1; tss_id TSS25338;
5	Cufflinks	exon	202048779	202048862	.	+	.	gene_id GRMZM2G315176; transcript_id TCONS_00028006; exon_number 14; oId CUFF.26557.1; tss_id TSS25338;
5	Cufflinks	exon	202048993	202049565	.	+	.	gene_id GRMZM2G315176; transcript_id TCONS_00028006; exon_number 15; oId CUFF.26557.1; tss_id TSS25338;
5	Cufflinks	exon	202049668	202050016	.	+	.	gene_id GRMZM2G315176; transcript_id TCONS_00028006; exon_number 16; oId CUFF.26557.1; tss_id TSS25338;
5	Cufflinks	exon	202141025	202141510	.	+	.	gene_id GRMZM2G180983; transcript_id TCONS_00028009; exon_number 1; oId CUFF.26548.1; tss_id TSS25341;
5	Cufflinks	exon	202142050	202142193	.	+	.	gene_id GRMZM2G180983; transcript_id TCONS_00028009; exon_number 2; oId CUFF.26548.1; tss_id TSS25341;
5	Cufflinks	exon	202142296	202142370	.	+	.	gene_id GRMZM2G180983; transcript_id TCONS_00028009; exon_number 3; oId CUFF.26548.1; tss_id TSS25341;
5	Cufflinks	exon	202142446	202142633	.	+	.	gene_id GRMZM2G180983; transcript_id TCONS_00028009; exon_number 4; oId CUFF.26548.1; tss_id TSS25341;
5	Cufflinks	exon	202142739	202142904	.	+	.	gene_id GRMZM2G180983; transcript_id TCONS_00028009; exon_number 5; oId CUFF.26548.1; tss_id TSS25341;
5	Cufflinks	exon	202142985	202143386	.	+	.	gene_id GRMZM2G180983; transcript_id TCONS_00028009; exon_number 6; oId CUFF.26548.1; tss_id TSS25341;
5	Cufflinks	exon	202149840	202150154	.	+	.	gene_id GRMZM2G180983; transcript_id TCONS_00028009; exon_number 7; oId CUFF.26548.1; tss_id TSS25341;
5	Cufflinks	exon	204771774	204772127	.	+	.	gene_id XLOC_024603; transcript_id TCONS_00028094; exon_number 1; oId CUFF.26658.2; tss_id TSS25419;
5	Cufflinks	exon	204772240	204772376	.	+	.	gene_id XLOC_024603; transcript_id TCONS_00028094; exon_number 2; oId CUFF.26658.2; tss_id TSS25419;
5	Cufflinks	exon	204772492	204772556	.	+	.	gene_id XLOC_024603; transcript_id TCONS_00028094; exon_number 3; oId CUFF.26658.2; tss_id TSS25419;
5	Cufflinks	exon	204771774	204772127	.	+	.	gene_id XLOC_024603; transcript_id TCONS_00028093; exon_number 1; oId CUFF.26658.1; tss_id TSS25419;
5	Cufflinks	exon	204772232	204772376	.	+	.	gene_id XLOC_024603; transcript_id TCONS_00028093; exon_number 2; oId CUFF.26658.1; tss_id TSS25419;
5	Cufflinks	exon	204772492	204772556	.	+	.	gene_id XLOC_024603; transcript_id TCONS_00028093; exon_number 3; oId CUFF.26658.1; tss_id TSS25419;
5	Cufflinks	exon	205283735	205284798	.	+	.	gene_id GRMZM2G385999; transcript_id TCONS_00028113; exon_number 1; oId CUFF.26669.1; tss_id TSS25433;
5	Cufflinks	exon	205986559	205988233	.	+	.	gene_id XLOC_024642; transcript_id TCONS_00028142; exon_number 1; oId CUFF.26709.1; tss_id TSS25460;

5	Cufflinks	exon	206668533	206668941	.	+	.	gene_id GRMZM2G140651; transcript_id TCONS_00028156; exon_number 1; oId CUFF.26751.1; tss_id TSS25473;
5	Cufflinks	exon	206669066	206669253	.	+	.	gene_id GRMZM2G140651; transcript_id TCONS_00028156; exon_number 2; oId CUFF.26751.1; tss_id TSS25473;
5	Cufflinks	exon	206669584	206669667	.	+	.	gene_id GRMZM2G140651; transcript_id TCONS_00028156; exon_number 3; oId CUFF.26751.1; tss_id TSS25473;
5	Cufflinks	exon	206670091	206670258	.	+	.	gene_id GRMZM2G140651; transcript_id TCONS_00028156; exon_number 4; oId CUFF.26751.1; tss_id TSS25473;
5	Cufflinks	exon	206670440	206670932	.	+	.	gene_id GRMZM2G140651; transcript_id TCONS_00028156; exon_number 5; oId CUFF.26751.1; tss_id TSS25473;
5	Cufflinks	exon	206972607	206972958	.	+	.	gene_id GRMZM2G028325; transcript_id TCONS_00028162; exon_number 1; oId CUFF.26768.2; tss_id TSS25478;
5	Cufflinks	exon	206973114	206973338	.	+	.	gene_id GRMZM2G028325; transcript_id TCONS_00028162; exon_number 2; oId CUFF.26768.2; tss_id TSS25478;
5	Cufflinks	exon	206974919	206975113	.	+	.	gene_id GRMZM2G028325; transcript_id TCONS_00028162; exon_number 3; oId CUFF.26768.2; tss_id TSS25478;
5	Cufflinks	exon	206975226	206976286	.	+	.	gene_id GRMZM2G028325; transcript_id TCONS_00028162; exon_number 4; oId CUFF.26768.2; tss_id TSS25478;
5	Cufflinks	exon	206972607	206972958	.	+	.	gene_id GRMZM2G028325; transcript_id TCONS_00028161; exon_number 1; oId CUFF.26768.1; tss_id TSS25478;
5	Cufflinks	exon	206973114	206973338	.	+	.	gene_id GRMZM2G028325; transcript_id TCONS_00028161; exon_number 2; oId CUFF.26768.1; tss_id TSS25478;
5	Cufflinks	exon	206974919	206975113	.	+	.	gene_id GRMZM2G028325; transcript_id TCONS_00028161; exon_number 3; oId CUFF.26768.1; tss_id TSS25478;
5	Cufflinks	exon	206975226	206975287	.	+	.	gene_id GRMZM2G028325; transcript_id TCONS_00028161; exon_number 4; oId CUFF.26768.1; tss_id TSS25478;
5	Cufflinks	exon	206975657	206976286	.	+	.	gene_id GRMZM2G028325; transcript_id TCONS_00028161; exon_number 5; oId CUFF.26768.1; tss_id TSS25478;
5	Cufflinks	exon	208973665	208975706	.	+	.	gene_id GRMZM2G323013; transcript_id TCONS_00028216; exon_number 1; oId CUFF.26836.1; tss_id TSS25519;
5	Cufflinks	exon	210525082	210525511	.	+	.	gene_id XLOC_024731; transcript_id TCONS_00028250; exon_number 1; oId CUFF.26913.1; tss_id TSS25550;
5	Cufflinks	exon	210825897	210826088	.	+	.	gene_id GRMZM2G052422; transcript_id TCONS_00028261; exon_number 1; oId CUFF.26934.1; tss_id TSS25558;
5	Cufflinks	exon	210826646	210826875	.	+	.	gene_id GRMZM2G052422; transcript_id TCONS_00028261; exon_number 2; oId CUFF.26934.1; tss_id TSS25558;
5	Cufflinks	exon	210827446	210828454	.	+	.	gene_id GRMZM2G052422; transcript_id TCONS_00028261; exon_number 3; oId CUFF.26934.1; tss_id TSS25558;
5	Cufflinks	exon	212213297	212213464	.	+	.	gene_id GRMZM2G124377; transcript_id TCONS_00028292; exon_number 1; oId CUFF.27013.1; tss_id TSS25586;
5	Cufflinks	exon	212213563	212213659	.	+	.	gene_id GRMZM2G124377; transcript_id TCONS_00028292; exon_number 2; oId CUFF.27013.1; tss_id TSS25586;
5	Cufflinks	exon	212213891	212213935	.	+	.	gene_id GRMZM2G124377; transcript_id TCONS_00028292; exon_number 3; oId CUFF.27013.1; tss_id TSS25586;
5	Cufflinks	exon	212214060	212214227	.	+	.	gene_id GRMZM2G124377; transcript_id TCONS_00028292; exon_number 4; oId CUFF.27013.1; tss_id TSS25586;
5	Cufflinks	exon	212214296	212214380	.	+	.	gene_id GRMZM2G124377; transcript_id TCONS_00028292; exon_number 5; oId CUFF.27013.1; tss_id TSS25586;
5	Cufflinks	exon	212214533	212214639	.	+	.	gene_id GRMZM2G124377; transcript_id TCONS_00028292; exon_number 6; oId CUFF.27013.1; tss_id TSS25586;
5	Cufflinks	exon	212215104	212215184	.	+	.	gene_id GRMZM2G124377; transcript_id TCONS_00028292; exon_number 7; oId CUFF.27013.1; tss_id TSS25586;
5	Cufflinks	exon	212218770	212218822	.	+	.	gene_id GRMZM2G124377; transcript_id TCONS_00028292; exon_number 8; oId CUFF.27013.1; tss_id TSS25586;
5	Cufflinks	exon	212218898	212218991	.	+	.	gene_id GRMZM2G124377; transcript_id TCONS_00028292; exon_number 9; oId CUFF.27013.1; tss_id TSS25586;
5	Cufflinks	exon	212219314	212219457	.	+	.	gene_id GRMZM2G124377; transcript_id TCONS_00028292; exon_number 10; oId CUFF.27013.1; tss_id TSS25586;
5	Cufflinks	exon	212219672	212219768	.	+	.	gene_id GRMZM2G124377; transcript_id TCONS_00028292; exon_number 11; oId CUFF.27013.1; tss_id TSS25586;
5	Cufflinks	exon	212219983	212220202	.	+	.	gene_id GRMZM2G124377; transcript_id TCONS_00028292; exon_number 12; oId CUFF.27013.1; tss_id TSS25586;
5	Cufflinks	exon	212220276	212220369	.	+	.	gene_id GRMZM2G124377; transcript_id TCONS_00028292; exon_number 13; oId CUFF.27013.1; tss_id TSS25586;
5	Cufflinks	exon	212220466	212221506	.	+	.	gene_id GRMZM2G124377; transcript_id TCONS_00028292; exon_number 14; oId CUFF.27013.1; tss_id TSS25586;
5	Cufflinks	exon	212221639	212221731	.	+	.	gene_id GRMZM2G124377; transcript_id TCONS_00028292; exon_number 15; oId CUFF.27013.1; tss_id TSS25586;
5	Cufflinks	exon	212221808	212222091	.	+	.	gene_id GRMZM2G124377; transcript_id TCONS_00028292; exon_number 16; oId CUFF.27013.1; tss_id TSS25586;
5	Cufflinks	exon	212222654	212223198	.	+	.	gene_id GRMZM2G124377; transcript_id TCONS_00028292; exon_number 17; oId CUFF.27013.1; tss_id TSS25586;

5	Cufflinks	exon	213088218	213088476	.	+	.	gene_id XLOC_024786; transcript_id TCONS_00028321; exon_number 1; oId CUFF.27017.1; tss_id TSS25611;
5	Cufflinks	exon	213088607	213090006	.	+	.	gene_id XLOC_024786; transcript_id TCONS_00028321; exon_number 2; oId CUFF.27017.1; tss_id TSS25611;
5	Cufflinks	exon	214192037	214192267	.	+	.	gene_id GRMZM2G180384; transcript_id TCONS_00028350; exon_number 1; oId CUFF.27081.1; tss_id TSS25638;
5	Cufflinks	exon	214192378	214192478	.	+	.	gene_id GRMZM2G180384; transcript_id TCONS_00028350; exon_number 2; oId CUFF.27081.1; tss_id TSS25638;
5	Cufflinks	exon	214193731	214193828	.	+	.	gene_id GRMZM2G180384; transcript_id TCONS_00028350; exon_number 3; oId CUFF.27081.1; tss_id TSS25638;
5	Cufflinks	exon	214194715	214195300	.	+	.	gene_id GRMZM2G180384; transcript_id TCONS_00028350; exon_number 4; oId CUFF.27081.1; tss_id TSS25638;
5	Cufflinks	exon	214574405	214575446	.	+	.	gene_id GRMZM2G163200; transcript_id TCONS_00028357; exon_number 1; oId CUFF.27111.1; tss_id TSS25645;
5	Cufflinks	exon	214577944	214578265	.	+	.	gene_id GRMZM2G163200; transcript_id TCONS_00028357; exon_number 2; oId CUFF.27111.1; tss_id TSS25645;
5	Cufflinks	exon	214578927	214580589	.	+	.	gene_id GRMZM2G163200; transcript_id TCONS_00028357; exon_number 3; oId CUFF.27111.1; tss_id TSS25645;
5	Cufflinks	exon	215100492	215102606	.	+	.	gene_id GRMZM2G058289; transcript_id TCONS_00028363; exon_number 1; oId CUFF.27121.1; tss_id TSS25651;
5	Cufflinks	exon	215103859	215104749	.	+	.	gene_id GRMZM2G058289; transcript_id TCONS_00028363; exon_number 2; oId CUFF.27121.1; tss_id TSS25651;
5	Cufflinks	exon	215314528	215315100	.	+	.	gene_id GRMZM5G862817; transcript_id TCONS_00028370; exon_number 1; oId CUFF.27132.1; tss_id TSS25658;
5	Cufflinks	exon	215315193	215315311	.	+	.	gene_id GRMZM5G862817; transcript_id TCONS_00028370; exon_number 2; oId CUFF.27132.1; tss_id TSS25658;
5	Cufflinks	exon	215315610	215316506	.	+	.	gene_id GRMZM5G862817; transcript_id TCONS_00028370; exon_number 3; oId CUFF.27132.1; tss_id TSS25658;
5	Cufflinks	exon	215890994	215891587	.	+	.	gene_id GRMZM5G894916; transcript_id TCONS_00028397; exon_number 1; oId CUFF.27194.2; tss_id TSS25682;
5	Cufflinks	exon	215891772	215891895	.	+	.	gene_id GRMZM5G894916; transcript_id TCONS_00028397; exon_number 2; oId CUFF.27194.2; tss_id TSS25682;
5	Cufflinks	exon	215891989	215892148	.	+	.	gene_id GRMZM5G894916; transcript_id TCONS_00028397; exon_number 3; oId CUFF.27194.2; tss_id TSS25682;
5	Cufflinks	exon	215892170	215892185	.	+	.	gene_id GRMZM5G894916; transcript_id TCONS_00028397; exon_number 4; oId CUFF.27194.2; tss_id TSS25682;
5	Cufflinks	exon	215892510	215893442	.	+	.	gene_id GRMZM5G894916; transcript_id TCONS_00028397; exon_number 5; oId CUFF.27194.2; tss_id TSS25682;
5	Cufflinks	exon	217060938	217062596	.	+	.	gene_id GRMZM2G048497; transcript_id TCONS_00028429; exon_number 1; oId CUFF.27230.1; tss_id TSS25710;
5	Cufflinks	exon	217062720	217063036	.	+	.	gene_id GRMZM2G048497; transcript_id TCONS_00028429; exon_number 2; oId CUFF.27230.1; tss_id TSS25710;
5	Cufflinks	exon	803571	803808	.	-	.	gene_id GRMZM2G014170; transcript_id TCONS_00028465; exon_number 1; oId CUFF.22579.2; tss_id TSS25737;
5	Cufflinks	exon	803896	804013	.	-	.	gene_id GRMZM2G014170; transcript_id TCONS_00028465; exon_number 2; oId CUFF.22579.2; tss_id TSS25737;
5	Cufflinks	exon	804387	804456	.	-	.	gene_id GRMZM2G014170; transcript_id TCONS_00028465; exon_number 3; oId CUFF.22579.2; tss_id TSS25737;
5	Cufflinks	exon	804554	804617	.	-	.	gene_id GRMZM2G014170; transcript_id TCONS_00028465; exon_number 4; oId CUFF.22579.2; tss_id TSS25737;
5	Cufflinks	exon	804797	804970	.	-	.	gene_id GRMZM2G014170; transcript_id TCONS_00028465; exon_number 5; oId CUFF.22579.2; tss_id TSS25737;
5	Cufflinks	exon	805064	805208	.	-	.	gene_id GRMZM2G014170; transcript_id TCONS_00028465; exon_number 6; oId CUFF.22579.2; tss_id TSS25737;
5	Cufflinks	exon	806111806242	.	-	.	gene_id GRMZM2G014170; transcript_id TCONS_00028465; exon_number 7; oId CUFF.22579.2; tss_id TSS25737;	
5	Cufflinks	exon	806942	807031	.	-	.	gene_id GRMZM2G014170; transcript_id TCONS_00028465; exon_number 8; oId CUFF.22579.2; tss_id TSS25737;
5	Cufflinks	exon	807789	807848	.	-	.	gene_id GRMZM2G014170; transcript_id TCONS_00028465; exon_number 9; oId CUFF.22579.2; tss_id TSS25737;
5	Cufflinks	exon	807937	808071	.	-	.	gene_id GRMZM2G014170; transcript_id TCONS_00028465; exon_number 10; oId CUFF.22579.2; tss_id TSS25737;
5	Cufflinks	exon	808336	808521	.	-	.	gene_id GRMZM2G014170; transcript_id TCONS_00028465; exon_number 11; oId CUFF.22579.2; tss_id TSS25737;
5	Cufflinks	exon	808971	810274	.	-	.	gene_id GRMZM2G014170; transcript_id TCONS_00028465; exon_number 12; oId CUFF.22579.2; tss_id TSS25737;
5	Cufflinks	exon	813840	815338	.	-	.	gene_id AC190636.3_FG005; transcript_id TCONS_00028467; exon_number 1; oId CUFF.22565.1; tss_id TSS25738;
5	Cufflinks	exon	2593362	2594082	.	-	.	gene_id XLOC_024980; transcript_id TCONS_00028565; exon_number 1; oId CUFF.22722.1; tss_id TSS25818;
5	Cufflinks	exon	4345145	4346123	.	-	.	gene_id XLOC_025031; transcript_id TCONS_00028620; exon_number 1; oId CUFF.22834.1; tss_id TSS25870;

5	Cufflinks	exon	6146163	6146769	.	-	.	gene_id GRMZM2G171468; transcript_id TCONS_00028665; exon_number 1; oId CUFF.22943.1; tss_id TSS25912;
5	Cufflinks	exon	6146913	6146989	.	-	.	gene_id GRMZM2G171468; transcript_id TCONS_00028665; exon_number 2; oId CUFF.22943.1; tss_id TSS25912;
5	Cufflinks	exon	6147109	6147526	.	-	.	gene_id GRMZM2G171468; transcript_id TCONS_00028665; exon_number 3; oId CUFF.22943.1; tss_id TSS25912;
5	Cufflinks	exon	6147689	6147825	.	-	.	gene_id GRMZM2G171468; transcript_id TCONS_00028665; exon_number 4; oId CUFF.22943.1; tss_id TSS25912;
5	Cufflinks	exon	6147961	6149014	.	-	.	gene_id GRMZM2G171468; transcript_id TCONS_00028665; exon_number 5; oId CUFF.22943.1; tss_id TSS25912;
5	Cufflinks	exon	6321319	6321822	.	-	.	gene_id XLOC_025077; transcript_id TCONS_00028669; exon_number 1; oId CUFF.22938.1; tss_id TSS25916;
5	Cufflinks	exon	6918683	6918977	.	-	.	gene_id XLOC_025097; transcript_id TCONS_00028691; exon_number 1; oId CUFF.22973.2; tss_id TSS25936;
5	Cufflinks	exon	6919811	6919913	.	-	.	gene_id XLOC_025097; transcript_id TCONS_00028691; exon_number 2; oId CUFF.22973.2; tss_id TSS25936;
5	Cufflinks	exon	7772722	7774847	.	-	.	gene_id GRMZM2G077389; transcript_id TCONS_00028724; exon_number 1; oId CUFF.23016.1; tss_id TSS25964;
5	Cufflinks	exon	10796873	10797414	.	-	.	gene_id GRMZM2G145827; transcript_id TCONS_00028794; exon_number 1; oId CUFF.23152.1; tss_id TSS26027;
5	Cufflinks	exon	10797877	10798016	.	-	.	gene_id GRMZM2G145827; transcript_id TCONS_00028794; exon_number 2; oId CUFF.23152.1; tss_id TSS26027;
5	Cufflinks	exon	10798902	10799130	.	-	.	gene_id GRMZM2G145827; transcript_id TCONS_00028794; exon_number 3; oId CUFF.23152.1; tss_id TSS26027;
5	Cufflinks	exon	10799218	10799268	.	-	.	gene_id GRMZM2G145827; transcript_id TCONS_00028794; exon_number 4; oId CUFF.23152.1; tss_id TSS26027;
5	Cufflinks	exon	10800189	10800414	.	-	.	gene_id GRMZM2G145827; transcript_id TCONS_00028794; exon_number 5; oId CUFF.23152.1; tss_id TSS26027;
5	Cufflinks	exon	10800586	10800656	.	-	.	gene_id GRMZM2G145827; transcript_id TCONS_00028794; exon_number 6; oId CUFF.23152.1; tss_id TSS26027;
5	Cufflinks	exon	10800842	10800961	.	-	.	gene_id GRMZM2G145827; transcript_id TCONS_00028794; exon_number 7; oId CUFF.23152.1; tss_id TSS26027;
5	Cufflinks	exon	10801054	10801248	.	-	.	gene_id GRMZM2G145827; transcript_id TCONS_00028794; exon_number 8; oId CUFF.23152.1; tss_id TSS26027;
5	Cufflinks	exon	10801327	10801391	.	-	.	gene_id GRMZM2G145827; transcript_id TCONS_00028794; exon_number 9; oId CUFF.23152.1; tss_id TSS26027;
5	Cufflinks	exon	10801536	10801626	.	-	.	gene_id GRMZM2G145827; transcript_id TCONS_00028794; exon_number 10; oId CUFF.23152.1; tss_id TSS26027;
5	Cufflinks	exon	10802040	10802101	.	-	.	gene_id GRMZM2G145827; transcript_id TCONS_00028794; exon_number 11; oId CUFF.23152.1; tss_id TSS26027;
5	Cufflinks	exon	10802192	10802242	.	-	.	gene_id GRMZM2G145827; transcript_id TCONS_00028794; exon_number 12; oId CUFF.23152.1; tss_id TSS26027;
5	Cufflinks	exon	10803395	10803496	.	-	.	gene_id GRMZM2G145827; transcript_id TCONS_00028794; exon_number 13; oId CUFF.23152.1; tss_id TSS26027;
5	Cufflinks	exon	10804217	10804321	.	-	.	gene_id GRMZM2G145827; transcript_id TCONS_00028794; exon_number 14; oId CUFF.23152.1; tss_id TSS26027;
5	Cufflinks	exon	10804404	10804467	.	-	.	gene_id GRMZM2G145827; transcript_id TCONS_00028794; exon_number 15; oId CUFF.23152.1; tss_id TSS26027;
5	Cufflinks	exon	10804581	10804954	.	-	.	gene_id GRMZM2G145827; transcript_id TCONS_00028794; exon_number 16; oId CUFF.23152.1; tss_id TSS26027;
5	Cufflinks	exon	12237732	12237984	.	-	.	gene_id GRMZM2G102616; transcript_id TCONS_00028817; exon_number 1; oId CUFF.23185.1; tss_id TSS26050;
5	Cufflinks	exon	12238101	12238164	.	-	.	gene_id GRMZM2G102616; transcript_id TCONS_00028817; exon_number 2; oId CUFF.23185.1; tss_id TSS26050;
5	Cufflinks	exon	12285642	12286645	.	-	.	gene_id GRMZM2G102760; transcript_id TCONS_00028820; exon_number 1; oId CUFF.23207.1; tss_id TSS26052;
5	Cufflinks	exon	12286735	12286998	.	-	.	gene_id GRMZM2G102760; transcript_id TCONS_00028820; exon_number 2; oId CUFF.23207.1; tss_id TSS26052;
5	Cufflinks	exon	12287111	12287418	.	-	.	gene_id GRMZM2G102760; transcript_id TCONS_00028820; exon_number 3; oId CUFF.23207.1; tss_id TSS26052;
5	Cufflinks	exon	12287511	12287618	.	-	.	gene_id GRMZM2G102760; transcript_id TCONS_00028820; exon_number 4; oId CUFF.23207.1; tss_id TSS26052;
5	Cufflinks	exon	12287727	12287833	.	-	.	gene_id GRMZM2G102760; transcript_id TCONS_00028820; exon_number 5; oId CUFF.23207.1; tss_id TSS26052;
5	Cufflinks	exon	12287946	12288290	.	-	.	gene_id GRMZM2G102760; transcript_id TCONS_00028820; exon_number 6; oId CUFF.23207.1; tss_id TSS26052;
5	Cufflinks	exon	12288382	12288625	.	-	.	gene_id GRMZM2G102760; transcript_id TCONS_00028820; exon_number 7; oId CUFF.23207.1; tss_id TSS26052;
5	Cufflinks	exon	12289136	12289425	.	-	.	gene_id GRMZM2G102760; transcript_id TCONS_00028820; exon_number 8; oId CUFF.23207.1; tss_id TSS26052;
5	Cufflinks	exon	12290129	12290523	.	-	.	gene_id GRMZM2G102760; transcript_id TCONS_00028820; exon_number 9; oId CUFF.23207.1; tss_id TSS26052;

5	Cufflinks	exon	14609748	14610160	.	-	.	gene_id GRMZM2G176723; transcript_id TCONS_00028861; exon_number 1; oId CUFF.23276.1; tss_id TSS26089;
5	Cufflinks	exon	14610245	14610757	.	-	.	gene_id GRMZM2G176723; transcript_id TCONS_00028861; exon_number 2; oId CUFF.23276.1; tss_id TSS26089;
5	Cufflinks	exon	14625518	14625973	.	-	.	gene_id GRMZM5G822180; transcript_id TCONS_00028863; exon_number 1; oId CUFF.23318.1; tss_id TSS26091;
5	Cufflinks	exon	14626068	14626540	.	-	.	gene_id GRMZM5G822180; transcript_id TCONS_00028863; exon_number 2; oId CUFF.23318.1; tss_id TSS26091;
5	Cufflinks	exon	14870256	14871310	.	-	.	gene_id GRMZM2G066191; transcript_id TCONS_00028868; exon_number 1; oId CUFF.23293.1; tss_id TSS26096;
5	Cufflinks	exon	14871405	14871674	.	-	.	gene_id GRMZM2G066191; transcript_id TCONS_00028868; exon_number 2; oId CUFF.23293.1; tss_id TSS26096;
5	Cufflinks	exon	14873498	14874075	.	-	.	gene_id GRMZM2G066191; transcript_id TCONS_00028868; exon_number 3; oId CUFF.23293.1; tss_id TSS26096;
5	Cufflinks	exon	19698812	19699146	.	-	.	gene_id GRMZM2G124918; transcript_id TCONS_00028938; exon_number 1; oId CUFF.23472.2; tss_id TSS26159;
5	Cufflinks	exon	19699239	19699304	.	-	.	gene_id GRMZM2G124918; transcript_id TCONS_00028938; exon_number 2; oId CUFF.23472.2; tss_id TSS26159;
5	Cufflinks	exon	19699475	19699523	.	-	.	gene_id GRMZM2G124918; transcript_id TCONS_00028938; exon_number 3; oId CUFF.23472.2; tss_id TSS26159;
5	Cufflinks	exon	19699649	19699716	.	-	.	gene_id GRMZM2G124918; transcript_id TCONS_00028938; exon_number 4; oId CUFF.23472.2; tss_id TSS26159;
5	Cufflinks	exon	19699803	19699848	.	-	.	gene_id GRMZM2G124918; transcript_id TCONS_00028938; exon_number 5; oId CUFF.23472.2; tss_id TSS26159;
5	Cufflinks	exon	19699962	19700055	.	-	.	gene_id GRMZM2G124918; transcript_id TCONS_00028938; exon_number 6; oId CUFF.23472.2; tss_id TSS26159;
5	Cufflinks	exon	19700233	19700295	.	-	.	gene_id GRMZM2G124918; transcript_id TCONS_00028938; exon_number 7; oId CUFF.23472.2; tss_id TSS26159;
5	Cufflinks	exon	19700536	19700658	.	-	.	gene_id GRMZM2G124918; transcript_id TCONS_00028938; exon_number 8; oId CUFF.23472.2; tss_id TSS26159;
5	Cufflinks	exon	19700776	19700809	.	-	.	gene_id GRMZM2G124918; transcript_id TCONS_00028938; exon_number 9; oId CUFF.23472.2; tss_id TSS26159;
5	Cufflinks	exon	19700938	19701000	.	-	.	gene_id GRMZM2G124918; transcript_id TCONS_00028938; exon_number 10; oId CUFF.23472.2; tss_id TSS26159;
5	Cufflinks	exon	19701101	19701217	.	-	.	gene_id GRMZM2G124918; transcript_id TCONS_00028938; exon_number 11; oId CUFF.23472.2; tss_id TSS26159;
5	Cufflinks	exon	19701328	19701393	.	-	.	gene_id GRMZM2G124918; transcript_id TCONS_00028938; exon_number 12; oId CUFF.23472.2; tss_id TSS26159;
5	Cufflinks	exon	19702526	19702780	.	-	.	gene_id GRMZM2G124918; transcript_id TCONS_00028938; exon_number 13; oId CUFF.23472.2; tss_id TSS26159;
5	Cufflinks	exon	19698812	19699146	.	-	.	gene_id GRMZM2G124918; transcript_id TCONS_00028937; exon_number 1; oId CUFF.23472.1; tss_id TSS26159;
5	Cufflinks	exon	19699239	19699304	.	-	.	gene_id GRMZM2G124918; transcript_id TCONS_00028937; exon_number 2; oId CUFF.23472.1; tss_id TSS26159;
5	Cufflinks	exon	19699475	19699523	.	-	.	gene_id GRMZM2G124918; transcript_id TCONS_00028937; exon_number 3; oId CUFF.23472.1; tss_id TSS26159;
5	Cufflinks	exon	19699649	19699716	.	-	.	gene_id GRMZM2G124918; transcript_id TCONS_00028937; exon_number 4; oId CUFF.23472.1; tss_id TSS26159;
5	Cufflinks	exon	19699803	19699848	.	-	.	gene_id GRMZM2G124918; transcript_id TCONS_00028937; exon_number 5; oId CUFF.23472.1; tss_id TSS26159;
5	Cufflinks	exon	19699962	19700055	.	-	.	gene_id GRMZM2G124918; transcript_id TCONS_00028937; exon_number 6; oId CUFF.23472.1; tss_id TSS26159;
5	Cufflinks	exon	19700233	19700295	.	-	.	gene_id GRMZM2G124918; transcript_id TCONS_00028937; exon_number 7; oId CUFF.23472.1; tss_id TSS26159;
5	Cufflinks	exon	19700536	19700658	.	-	.	gene_id GRMZM2G124918; transcript_id TCONS_00028937; exon_number 8; oId CUFF.23472.1; tss_id TSS26159;
5	Cufflinks	exon	19700776	19700809	.	-	.	gene_id GRMZM2G124918; transcript_id TCONS_00028937; exon_number 9; oId CUFF.23472.1; tss_id TSS26159;
5	Cufflinks	exon	19700938	19701000	.	-	.	gene_id GRMZM2G124918; transcript_id TCONS_00028937; exon_number 10; oId CUFF.23472.1; tss_id TSS26159;
5	Cufflinks	exon	19701101	19701220	.	-	.	gene_id GRMZM2G124918; transcript_id TCONS_00028937; exon_number 11; oId CUFF.23472.1; tss_id TSS26159;
5	Cufflinks	exon	19701328	19701393	.	-	.	gene_id GRMZM2G124918; transcript_id TCONS_00028937; exon_number 12; oId CUFF.23472.1; tss_id TSS26159;
5	Cufflinks	exon	19702526	19702780	.	-	.	gene_id GRMZM2G124918; transcript_id TCONS_00028937; exon_number 13; oId CUFF.23472.1; tss_id TSS26159;
5	Cufflinks	exon	20622861	20623200	.	-	.	gene_id GRMZM2G170128; transcript_id TCONS_00028953; exon_number 1; oId CUFF.23452.1; tss_id TSS26174;
5	Cufflinks	exon	20623363	20623449	.	-	.	gene_id GRMZM2G170128; transcript_id TCONS_00028953; exon_number 2; oId CUFF.23452.1; tss_id TSS26174;
5	Cufflinks	exon	20623530	20623648	.	-	.	gene_id GRMZM2G170128; transcript_id TCONS_00028953; exon_number 3; oId CUFF.23452.1; tss_id TSS26174;

5	Cufflinks	exon	20623795	20624039	.	-	.	gene_id GRMZM2G170128; transcript_id TCONS_00028953; exon_number 4; oId CUFF.23452.1; tss_id TSS26174;
5	Cufflinks	exon	20624140	20624196	.	-	.	gene_id GRMZM2G170128; transcript_id TCONS_00028953; exon_number 5; oId CUFF.23452.1; tss_id TSS26174;
5	Cufflinks	exon	20624319	20624950	.	-	.	gene_id GRMZM2G170128; transcript_id TCONS_00028953; exon_number 6; oId CUFF.23452.1; tss_id TSS26174;
5	Cufflinks	exon	20625980	20626562	.	-	.	gene_id GRMZM2G170128; transcript_id TCONS_00028953; exon_number 7; oId CUFF.23452.1; tss_id TSS26174;
5	Cufflinks	exon	20929213	20930183	.	-	.	gene_id GRMZM2G132036; transcript_id TCONS_00028958; exon_number 1; oId CUFF.23460.1; tss_id TSS26179;
5	Cufflinks	exon	20930273	20930377	.	-	.	gene_id GRMZM2G132036; transcript_id TCONS_00028958; exon_number 2; oId CUFF.23460.1; tss_id TSS26179;
5	Cufflinks	exon	20930581	20930707	.	-	.	gene_id GRMZM2G132036; transcript_id TCONS_00028958; exon_number 3; oId CUFF.23460.1; tss_id TSS26179;
5	Cufflinks	exon	20934029	20934078	.	-	.	gene_id GRMZM2G132036; transcript_id TCONS_00028958; exon_number 4; oId CUFF.23460.1; tss_id TSS26179;
5	Cufflinks	exon	20934175	20934778	.	-	.	gene_id GRMZM2G132036; transcript_id TCONS_00028958; exon_number 5; oId CUFF.23460.1; tss_id TSS26179;
5	Cufflinks	exon	31900586	31902800	.	-	.	gene_id XLOC_025458; transcript_id TCONS_00029101; exon_number 1; oId CUFF.23711.1; tss_id TSS26310;
5	Cufflinks	exon	34430419	34430697	.	-	.	gene_id XLOC_025491; transcript_id TCONS_00029140; exon_number 1; oId CUFF.23766.1; tss_id TSS26344;
5	Cufflinks	exon	39980153	39982962	.	-	.	gene_id GRMZM2G134597; transcript_id TCONS_00029207; exon_number 1; oId CUFF.23871.1; tss_id TSS26406;
5	Cufflinks	exon	41225477	41225885	.	-	.	gene_id XLOC_025566; transcript_id TCONS_00029235; exon_number 1; oId CUFF.23898.1; tss_id TSS26423;
5	Cufflinks	exon	46975199	46975950	.	-	.	gene_id AC209987.4_FG010; transcript_id TCONS_00029280; exon_number 1; oId CUFF.23987.1; tss_id TSS26464;
5	Cufflinks	exon	46976058	46976425	.	-	.	gene_id AC209987.4_FG010; transcript_id TCONS_00029280; exon_number 2; oId CUFF.23987.1; tss_id TSS26464;
5	Cufflinks	exon	48600426	48601183	.	-	.	gene_id XLOC_025618; transcript_id TCONS_00029296; exon_number 1; oId CUFF.23997.1; tss_id TSS26479;
5	Cufflinks	exon	48601252	48601438	.	-	.	gene_id XLOC_025618; transcript_id TCONS_00029296; exon_number 2; oId CUFF.23997.1; tss_id TSS26479;
5	Cufflinks	exon	50115509	50116018	.	-	.	gene_id GRMZM2G113655; transcript_id TCONS_00029311; exon_number 1; oId CUFF.24026.1; tss_id TSS26494;
5	Cufflinks	exon	50118593	50119141	.	-	.	gene_id GRMZM2G113655; transcript_id TCONS_00029311; exon_number 2; oId CUFF.24026.1; tss_id TSS26494;
5	Cufflinks	exon	50119233	50120425	.	-	.	gene_id GRMZM2G113655; transcript_id TCONS_00029311; exon_number 3; oId CUFF.24026.1; tss_id TSS26494;
5	Cufflinks	exon	50371817	50373787	.	-	.	gene_id XLOC_025635; transcript_id TCONS_00029313; exon_number 1; oId CUFF.24043.1; tss_id TSS26496;
5	Cufflinks	exon	50478793	50480241	.	-	.	gene_id GRMZM2G026881; transcript_id TCONS_00029314; exon_number 1; oId CUFF.24039.1; tss_id TSS26497;
5	Cufflinks	exon	50480367	50481470	.	-	.	gene_id GRMZM2G026881; transcript_id TCONS_00029314; exon_number 2; oId CUFF.24039.1; tss_id TSS26497;
5	Cufflinks	exon	53425033	53425806	.	-	.	gene_id GRMZM2G080183; transcript_id TCONS_00029335; exon_number 1; oId CUFF.24079.2; tss_id TSS26515;
5	Cufflinks	exon	53425952	53426117	.	-	.	gene_id GRMZM2G080183; transcript_id TCONS_00029335; exon_number 2; oId CUFF.24079.2; tss_id TSS26515;
5	Cufflinks	exon	53427155	53427346	.	-	.	gene_id GRMZM2G080183; transcript_id TCONS_00029335; exon_number 3; oId CUFF.24079.2; tss_id TSS26515;
5	Cufflinks	exon	53427445	53427956	.	-	.	gene_id GRMZM2G080183; transcript_id TCONS_00029335; exon_number 4; oId CUFF.24079.2; tss_id TSS26515;
5	Cufflinks	exon	59299682	59300279	.	-	.	gene_id XLOC_025710; transcript_id TCONS_00029394; exon_number 1; oId CUFF.24181.1; tss_id TSS26572;
5	Cufflinks	exon	59337823	59339561	.	-	.	gene_id XLOC_025713; transcript_id TCONS_00029397; exon_number 1; oId CUFF.24185.1; tss_id TSS26575;
5	Cufflinks	exon	61555926	61556425	.	-	.	gene_id GRMZM2G399073; transcript_id TCONS_00029437; exon_number 1; oId CUFF.24281.1; tss_id TSS26614;
5	Cufflinks	exon	61556560	61556964	.	-	.	gene_id GRMZM2G399073; transcript_id TCONS_00029437; exon_number 2; oId CUFF.24281.1; tss_id TSS26614;
5	Cufflinks	exon	61557079	61557229	.	-	.	gene_id GRMZM2G399073; transcript_id TCONS_00029437; exon_number 3; oId CUFF.24281.1; tss_id TSS26614;
5	Cufflinks	exon	61557325	61557463	.	-	.	gene_id GRMZM2G399073; transcript_id TCONS_00029437; exon_number 4; oId CUFF.24281.1; tss_id TSS26614;
5	Cufflinks	exon	61557552	61557717	.	-	.	gene_id GRMZM2G399073; transcript_id TCONS_00029437; exon_number 5; oId CUFF.24281.1; tss_id TSS26614;
5	Cufflinks	exon	61557804	61558019	.	-	.	gene_id GRMZM2G399073; transcript_id TCONS_00029437; exon_number 6; oId CUFF.24281.1; tss_id TSS26614;
5	Cufflinks	exon	61558145	61558329	.	-	.	gene_id GRMZM2G399073; transcript_id TCONS_00029437; exon_number 7; oId CUFF.24281.1; tss_id TSS26614;

5	Cufflinks	exon	61558412	61558529	.	-	.	gene_id GRMZM2G399073; transcript_id TCONS_00029437; exon_number 8; oId CUFF.24281.1; tss_id TSS26614;
5	Cufflinks	exon	61559001	61559139	.	-	.	gene_id GRMZM2G399073; transcript_id TCONS_00029437; exon_number 9; oId CUFF.24281.1; tss_id TSS26614;
5	Cufflinks	exon	61559226	61559352	.	-	.	gene_id GRMZM2G399073; transcript_id TCONS_00029437; exon_number 10; oId CUFF.24281.1; tss_id TSS26614;
5	Cufflinks	exon	61559491	61559602	.	-	.	gene_id GRMZM2G399073; transcript_id TCONS_00029437; exon_number 11; oId CUFF.24281.1; tss_id TSS26614;
5	Cufflinks	exon	61559697	61559777	.	-	.	gene_id GRMZM2G399073; transcript_id TCONS_00029437; exon_number 12; oId CUFF.24281.1; tss_id TSS26614;
5	Cufflinks	exon	61559858	61560011	.	-	.	gene_id GRMZM2G399073; transcript_id TCONS_00029437; exon_number 13; oId CUFF.24281.1; tss_id TSS26614;
5	Cufflinks	exon	61560088	61560208	.	-	.	gene_id GRMZM2G399073; transcript_id TCONS_00029437; exon_number 14; oId CUFF.24281.1; tss_id TSS26614;
5	Cufflinks	exon	61560831	61560984	.	-	.	gene_id GRMZM2G399073; transcript_id TCONS_00029437; exon_number 15; oId CUFF.24281.1; tss_id TSS26614;
5	Cufflinks	exon	62142491	62143212	.	-	.	gene_id GRMZM2G065125; transcript_id TCONS_00029444; exon_number 1; oId CUFF.24301.1; tss_id TSS26621;
5	Cufflinks	exon	62143314	62143498	.	-	.	gene_id GRMZM2G065125; transcript_id TCONS_00029444; exon_number 2; oId CUFF.24301.1; tss_id TSS26621;
5	Cufflinks	exon	62143577	62143992	.	-	.	gene_id GRMZM2G065125; transcript_id TCONS_00029444; exon_number 3; oId CUFF.24301.1; tss_id TSS26621;
5	Cufflinks	exon	62144444	62144631	.	-	.	gene_id GRMZM2G065125; transcript_id TCONS_00029444; exon_number 4; oId CUFF.24301.1; tss_id TSS26621;
5	Cufflinks	exon	62144715	62144786	.	-	.	gene_id GRMZM2G065125; transcript_id TCONS_00029444; exon_number 5; oId CUFF.24301.1; tss_id TSS26621;
5	Cufflinks	exon	62146834	62147010	.	-	.	gene_id GRMZM2G065125; transcript_id TCONS_00029444; exon_number 6; oId CUFF.24301.1; tss_id TSS26621;
5	Cufflinks	exon	62147108	62147249	.	-	.	gene_id GRMZM2G065125; transcript_id TCONS_00029444; exon_number 7; oId CUFF.24301.1; tss_id TSS26621;
5	Cufflinks	exon	62624370	62624982	.	-	.	gene_id GRMZM2G048073; transcript_id TCONS_00029452; exon_number 1; oId CUFF.24292.1; tss_id TSS26629;
5	Cufflinks	exon	62625075	62625326	.	-	.	gene_id GRMZM2G048073; transcript_id TCONS_00029452; exon_number 2; oId CUFF.24292.1; tss_id TSS26629;
5	Cufflinks	exon	62625445	62625575	.	-	.	gene_id GRMZM2G048073; transcript_id TCONS_00029452; exon_number 3; oId CUFF.24292.1; tss_id TSS26629;
5	Cufflinks	exon	62625722	62626140	.	-	.	gene_id GRMZM2G048073; transcript_id TCONS_00029452; exon_number 4; oId CUFF.24292.1; tss_id TSS26629;
5	Cufflinks	exon	62711015	62712046	.	-	.	gene_id GRMZM2G132331; transcript_id TCONS_00029454; exon_number 1; oId CUFF.24286.1; tss_id TSS26631;
5	Cufflinks	exon	62712145	62712290	.	-	.	gene_id GRMZM2G132331; transcript_id TCONS_00029454; exon_number 2; oId CUFF.24286.1; tss_id TSS26631;
5	Cufflinks	exon	62712398	62712701	.	-	.	gene_id GRMZM2G132331; transcript_id TCONS_00029454; exon_number 3; oId CUFF.24286.1; tss_id TSS26631;
5	Cufflinks	exon	68336691	68338762	.	-	.	gene_id GRMZM2G035073; transcript_id TCONS_00029523; exon_number 1; oId CUFF.24394.1; tss_id TSS26693;
5	Cufflinks	exon	70802312	70803695	.	-	.	gene_id GRMZM2G164202; transcript_id TCONS_00029554; exon_number 1; oId CUFF.24527.1; tss_id TSS26724;
5	Cufflinks	exon	70803816	70804163	.	-	.	gene_id GRMZM2G164202; transcript_id TCONS_00029554; exon_number 2; oId CUFF.24527.1; tss_id TSS26724;
5	Cufflinks	exon	76252113	76255338	.	-	.	gene_id XLOC_025907; transcript_id TCONS_00029614; exon_number 1; oId CUFF.24568.1; tss_id TSS26775;
5	Cufflinks	exon	76714970	76716099	.	-	.	gene_id GRMZM2G042278; transcript_id TCONS_00029622; exon_number 1; oId CUFF.24583.1; tss_id TSS26781;
5	Cufflinks	exon	76716226	76717162	.	-	.	gene_id GRMZM2G042278; transcript_id TCONS_00029622; exon_number 2; oId CUFF.24583.1; tss_id TSS26781;
5	Cufflinks	exon	76717302	76717349	.	-	.	gene_id GRMZM2G042278; transcript_id TCONS_00029622; exon_number 3; oId CUFF.24583.1; tss_id TSS26781;
5	Cufflinks	exon	76717579	76718117	.	-	.	gene_id GRMZM2G042278; transcript_id TCONS_00029622; exon_number 4; oId CUFF.24583.1; tss_id TSS26781;
5	Cufflinks	exon	76718211	76718433	.	-	.	gene_id GRMZM2G042278; transcript_id TCONS_00029622; exon_number 5; oId CUFF.24583.1; tss_id TSS26781;
5	Cufflinks	exon	83900910	83902051	.	-	.	gene_id GRMZM2G088235; transcript_id TCONS_00029694; exon_number 1; oId CUFF.24734.4; tss_id TSS26843;
5	Cufflinks	exon	83902166	83902358	.	-	.	gene_id GRMZM2G088235; transcript_id TCONS_00029694; exon_number 2; oId CUFF.24734.4; tss_id TSS26843;
5	Cufflinks	exon	87452681	87453022	.	-	.	gene_id XLOC_025998; transcript_id TCONS_00029721; exon_number 1; oId CUFF.24758.1; tss_id TSS26868;
5	Cufflinks	exon	89189606	89190223	.	-	.	gene_id GRMZM2G075333; transcript_id TCONS_00029733; exon_number 1; oId CUFF.24788.1; tss_id TSS26880;
5	Cufflinks	exon	89190429	89190531	.	-	.	gene_id GRMZM2G075333; transcript_id TCONS_00029733; exon_number 2; oId CUFF.24788.1; tss_id TSS26880;

5	Cufflinks	exon	89190689	89190902	.	-	.	gene_id GRMZM2G075333; transcript_id TCONS_00029733; exon_number 3; oId CUFF.24788.1; tss_id TSS26880;
5	Cufflinks	exon	89192377	89192575	.	-	.	gene_id GRMZM2G075333; transcript_id TCONS_00029733; exon_number 4; oId CUFF.24788.1; tss_id TSS26880;
5	Cufflinks	exon	89192672	89193025	.	-	.	gene_id GRMZM2G075333; transcript_id TCONS_00029733; exon_number 5; oId CUFF.24788.1; tss_id TSS26880;
5	Cufflinks	exon	89193446	89194342	.	-	.	gene_id GRMZM2G075333; transcript_id TCONS_00029733; exon_number 6; oId CUFF.24788.1; tss_id TSS26880;
5	Cufflinks	exon	93274116	93274685	.	-	.	gene_id GRMZM2G060485; transcript_id TCONS_00029757; exon_number 1; oId CUFF.24824.1; tss_id TSS26903;
5	Cufflinks	exon	93274781	93275842	.	-	.	gene_id GRMZM2G060485; transcript_id TCONS_00029757; exon_number 2; oId CUFF.24824.1; tss_id TSS26903;
5	Cufflinks	exon	93276401	93276477	.	-	.	gene_id GRMZM2G060485; transcript_id TCONS_00029757; exon_number 3; oId CUFF.24824.1; tss_id TSS26903;
5	Cufflinks	exon	93276591	93276858	.	-	.	gene_id GRMZM2G060485; transcript_id TCONS_00029757; exon_number 4; oId CUFF.24824.1; tss_id TSS26903;
5	Cufflinks	exon	93551459	93553187	.	-	.	gene_id GRMZM2G117544; transcript_id TCONS_00029759; exon_number 1; oId CUFF.24830.1; tss_id TSS26905;
5	Cufflinks	exon	93553412	93553506	.	-	.	gene_id GRMZM2G117544; transcript_id TCONS_00029759; exon_number 2; oId CUFF.24830.1; tss_id TSS26905;
5	Cufflinks	exon	93554007	93554091	.	-	.	gene_id GRMZM2G117544; transcript_id TCONS_00029759; exon_number 3; oId CUFF.24830.1; tss_id TSS26905;
5	Cufflinks	exon	93554835	93554924	.	-	.	gene_id GRMZM2G117544; transcript_id TCONS_00029759; exon_number 4; oId CUFF.24830.1; tss_id TSS26905;
5	Cufflinks	exon	93555288	93555395	.	-	.	gene_id GRMZM2G117544; transcript_id TCONS_00029759; exon_number 5; oId CUFF.24830.1; tss_id TSS26905;
5	Cufflinks	exon	93555508	93555689	.	-	.	gene_id GRMZM2G117544; transcript_id TCONS_00029759; exon_number 6; oId CUFF.24830.1; tss_id TSS26905;
5	Cufflinks	exon	93555814	93555969	.	-	.	gene_id GRMZM2G117544; transcript_id TCONS_00029759; exon_number 7; oId CUFF.24830.1; tss_id TSS26905;
5	Cufflinks	exon	147664347	147665442	.	-	.	gene_id GRMZM2G026490; transcript_id TCONS_00030036; exon_number 1; oId CUFF.25313.1; tss_id TSS27156;
5	Cufflinks	exon	147665646	147665731	.	-	.	gene_id GRMZM2G026490; transcript_id TCONS_00030036; exon_number 2; oId CUFF.25313.1; tss_id TSS27156;
5	Cufflinks	exon	147667652	147667861	.	-	.	gene_id GRMZM2G026490; transcript_id TCONS_00030036; exon_number 3; oId CUFF.25313.1; tss_id TSS27156;
5	Cufflinks	exon	147667944	147668114	.	-	.	gene_id GRMZM2G026490; transcript_id TCONS_00030036; exon_number 4; oId CUFF.25313.1; tss_id TSS27156;
5	Cufflinks	exon	147668898	147669080	.	-	.	gene_id GRMZM2G026490; transcript_id TCONS_00030036; exon_number 5; oId CUFF.25313.1; tss_id TSS27156;
5	Cufflinks	exon	147669652	147669747	.	-	.	gene_id GRMZM2G026490; transcript_id TCONS_00030036; exon_number 6; oId CUFF.25313.1; tss_id TSS27156;
5	Cufflinks	exon	147669871	147670122	.	-	.	gene_id GRMZM2G026490; transcript_id TCONS_00030036; exon_number 7; oId CUFF.25313.1; tss_id TSS27156;
5	Cufflinks	exon	163455311	163456332	.	-	.	gene_id GRMZM2G004856; transcript_id TCONS_00030166; exon_number 1; oId CUFF.25550.3; tss_id TSS27268;
5	Cufflinks	exon	163456447	163456660	.	-	.	gene_id GRMZM2G004856; transcript_id TCONS_00030166; exon_number 2; oId CUFF.25550.3; tss_id TSS27268;
5	Cufflinks	exon	168572548	168575048	.	-	.	gene_id GRMZM5G876303; transcript_id TCONS_00030226; exon_number 1; oId CUFF.25621.1; tss_id TSS27320;
5	Cufflinks	exon	169501558	169502071	.	-	.	gene_id XLOC_026443; transcript_id TCONS_00030237; exon_number 1; oId CUFF.25663.1; tss_id TSS27330;
5	Cufflinks	exon	170458785	170459633	.	-	.	gene_id GRMZM2G451314; transcript_id TCONS_00030252; exon_number 1; oId CUFF.25686.1; tss_id TSS27345;
5	Cufflinks	exon	170459744	170459877	.	-	.	gene_id GRMZM2G451314; transcript_id TCONS_00030252; exon_number 2; oId CUFF.25686.1; tss_id TSS27345;
5	Cufflinks	exon	170459954	170460147	.	-	.	gene_id GRMZM2G451314; transcript_id TCONS_00030252; exon_number 3; oId CUFF.25686.1; tss_id TSS27345;
5	Cufflinks	exon	170460234	170460465	.	-	.	gene_id GRMZM2G451314; transcript_id TCONS_00030252; exon_number 4; oId CUFF.25686.1; tss_id TSS27345;
5	Cufflinks	exon	170462788	170463276	.	-	.	gene_id GRMZM2G451314; transcript_id TCONS_00030252; exon_number 5; oId CUFF.25686.1; tss_id TSS27345;
5	Cufflinks	exon	171563296	171564018	.	-	.	gene_id XLOC_026470; transcript_id TCONS_00030265; exon_number 1; oId CUFF.25712.1; tss_id TSS27357;
5	Cufflinks	exon	171565038	171567359	.	-	.	gene_id XLOC_026470; transcript_id TCONS_00030265; exon_number 2; oId CUFF.25712.1; tss_id TSS27357;
5	Cufflinks	exon	171567455	171567672	.	-	.	gene_id XLOC_026470; transcript_id TCONS_00030265; exon_number 3; oId CUFF.25712.1; tss_id TSS27357;
5	Cufflinks	exon	174638160	174638454	.	-	.	gene_id GRMZM2G421231; transcript_id TCONS_00030317; exon_number 1; oId CUFF.25777.1; tss_id TSS27405;
5	Cufflinks	exon	175731393	175734369	.	-	.	gene_id GRMZM2G023988; transcript_id TCONS_00030336; exon_number 1; oId CUFF.25817.2; tss_id TSS27420;

5	Cufflinks	exon	175735194	175736451	.	-	.	gene_id GRMZM2G023988; transcript_id TCONS_00030336; exon_number 2; oId CUFF.25817.2; tss_id TSS27420;
5	Cufflinks	exon	176985247	176986274	.	-	.	gene_id GRMZM2G146380; transcript_id TCONS_00030349; exon_number 1; oId CUFF.25855.2; tss_id TSS27430;
5	Cufflinks	exon	176986450	176986711	.	-	.	gene_id GRMZM2G146380; transcript_id TCONS_00030349; exon_number 2; oId CUFF.25855.2; tss_id TSS27430;
5	Cufflinks	exon	176985247	176986277	.	-	.	gene_id GRMZM2G146380; transcript_id TCONS_00030348; exon_number 1; oId CUFF.25855.1; tss_id TSS27430;
5	Cufflinks	exon	176986450	176986711	.	-	.	gene_id GRMZM2G146380; transcript_id TCONS_00030348; exon_number 2; oId CUFF.25855.1; tss_id TSS27430;
5	Cufflinks	exon	185696058	185696877	.	-	.	gene_id XLOC_026656; transcript_id TCONS_00030489; exon_number 1; oId CUFF.26072.1; tss_id TSS27551;
5	Cufflinks	exon	187620886	187622518	.	-	.	gene_id GRMZM2G141411; transcript_id TCONS_00030517; exon_number 1; oId CUFF.26114.1; tss_id TSS27578;
5	Cufflinks	exon	188716613	188718822	.	-	.	gene_id GRMZM2G139765; transcript_id TCONS_00030541; exon_number 1; oId CUFF.26148.1; tss_id TSS27599;
5	Cufflinks	exon	191180794	191182091	.	-	.	gene_id GRMZM2G094768; transcript_id TCONS_00030579; exon_number 1; oId CUFF.26243.1; tss_id TSS27633;
5	Cufflinks	exon	191182236	191182379	.	-	.	gene_id GRMZM2G094768; transcript_id TCONS_00030579; exon_number 2; oId CUFF.26243.1; tss_id TSS27633;
5	Cufflinks	exon	191182498	191183354	.	-	.	gene_id GRMZM2G094768; transcript_id TCONS_00030579; exon_number 3; oId CUFF.26243.1; tss_id TSS27633;
5	Cufflinks	exon	191180794	191182091	.	-	.	gene_id GRMZM2G094768; transcript_id TCONS_00030580; exon_number 1; oId CUFF.26243.2; tss_id TSS27633;
5	Cufflinks	exon	191182236	191182387	.	-	.	gene_id GRMZM2G094768; transcript_id TCONS_00030580; exon_number 2; oId CUFF.26243.2; tss_id TSS27633;
5	Cufflinks	exon	191182500	191183392	.	-	.	gene_id GRMZM2G094768; transcript_id TCONS_00030580; exon_number 3; oId CUFF.26243.2; tss_id TSS27633;
5	Cufflinks	exon	200490849	200491838	.	-	.	gene_id GRMZM2G325038; transcript_id TCONS_00030709; exon_number 1; oId CUFF.26470.1; tss_id TSS27753;
5	Cufflinks	exon	200491957	200492345	.	-	.	gene_id GRMZM2G325038; transcript_id TCONS_00030709; exon_number 2; oId CUFF.26470.1; tss_id TSS27753;
5	Cufflinks	exon	201723129	201723777	.	-	.	gene_id GRMZM2G170595; transcript_id TCONS_00030730; exon_number 1; oId CUFF.26534.1; tss_id TSS27771;
5	Cufflinks	exon	201723952	201724092	.	-	.	gene_id GRMZM2G170595; transcript_id TCONS_00030730; exon_number 2; oId CUFF.26534.1; tss_id TSS27771;
5	Cufflinks	exon	201724186	201724328	.	-	.	gene_id GRMZM2G170595; transcript_id TCONS_00030730; exon_number 3; oId CUFF.26534.1; tss_id TSS27771;
5	Cufflinks	exon	201724670	201724827	.	-	.	gene_id GRMZM2G170595; transcript_id TCONS_00030730; exon_number 4; oId CUFF.26534.1; tss_id TSS27771;
5	Cufflinks	exon	201724923	201725031	.	-	.	gene_id GRMZM2G170595; transcript_id TCONS_00030730; exon_number 5; oId CUFF.26534.1; tss_id TSS27771;
5	Cufflinks	exon	201725183	201725280	.	-	.	gene_id GRMZM2G170595; transcript_id TCONS_00030730; exon_number 6; oId CUFF.26534.1; tss_id TSS27771;
5	Cufflinks	exon	201725373	201725527	.	-	.	gene_id GRMZM2G170595; transcript_id TCONS_00030730; exon_number 7; oId CUFF.26534.1; tss_id TSS27771;
5	Cufflinks	exon	201725786	201725869	.	-	.	gene_id GRMZM2G170595; transcript_id TCONS_00030730; exon_number 8; oId CUFF.26534.1; tss_id TSS27771;
5	Cufflinks	exon	201726010	201726317	.	-	.	gene_id GRMZM2G170595; transcript_id TCONS_00030730; exon_number 9; oId CUFF.26534.1; tss_id TSS27771;
5	Cufflinks	exon	202139172	202141517	.	-	.	gene_id GRMZM2G481604; transcript_id TCONS_00030737; exon_number 1; oId CUFF.26547.1; tss_id TSS27776;
5	Cufflinks	exon	203730871	203731291	.	-	.	gene_id XLOC_026900; transcript_id TCONS_00030769; exon_number 1; oId CUFF.26584.1; tss_id TSS27805;
5	Cufflinks	exon	203755612	203756080	.	-	.	gene_id XLOC_026903; transcript_id TCONS_00030772; exon_number 1; oId CUFF.26604.1; tss_id TSS27808;
5	Cufflinks	exon	204771774	204772556	.	-	.	gene_id GRMZM6G946111; transcript_id TCONS_00030791; exon_number 1; oId CUFF.26659.1; tss_id TSS27825;
5	Cufflinks	exon	205803187	205805607	.	-	.	gene_id GRMZM2G142709; transcript_id TCONS_00030816; exon_number 1; oId CUFF.26698.1; tss_id TSS27848;
5	Cufflinks	exon	208137640	208138739	.	-	.	gene_id GRMZM2G035503; transcript_id TCONS_00030866; exon_number 1; oId CUFF.26814.1; tss_id TSS27897;
5	Cufflinks	exon	208138824	208139594	.	-	.	gene_id GRMZM2G035503; transcript_id TCONS_00030866; exon_number 2; oId CUFF.26814.1; tss_id TSS27897;
5	Cufflinks	exon	208140193	208140290	.	-	.	gene_id GRMZM2G035503; transcript_id TCONS_00030866; exon_number 3; oId CUFF.26814.1; tss_id TSS27897;
5	Cufflinks	exon	208140469	208140638	.	-	.	gene_id GRMZM2G035503; transcript_id TCONS_00030866; exon_number 4; oId CUFF.26814.1; tss_id TSS27897;
5	Cufflinks	exon	208140757	208141056	.	-	.	gene_id GRMZM2G035503; transcript_id TCONS_00030866; exon_number 5; oId CUFF.26814.1; tss_id TSS27897;
5	Cufflinks	exon	208141691	208142328	.	-	.	gene_id GRMZM2G035503; transcript_id TCONS_00030866; exon_number 6; oId CUFF.26814.1; tss_id TSS27897;

5	Cufflinks	exon	210350135	210350510	.	-	.	gene_id GRMZM2G414621; transcript_id TCONS_00030930; exon_number 1; oId CUFF.26909.1; tss_id TSS27956;
5	Cufflinks	exon	210350710	210351223	.	-	.	gene_id GRMZM2G414621; transcript_id TCONS_00030930; exon_number 2; oId CUFF.26909.1; tss_id TSS27956;
5	Cufflinks	exon	210351347	210351715	.	-	.	gene_id GRMZM2G414621; transcript_id TCONS_00030930; exon_number 3; oId CUFF.26909.1; tss_id TSS27956;
5	Cufflinks	exon	210351814	210351920	.	-	.	gene_id GRMZM2G414621; transcript_id TCONS_00030930; exon_number 4; oId CUFF.26909.1; tss_id TSS27956;
5	Cufflinks	exon	210352256	210352786	.	-	.	gene_id GRMZM2G414621; transcript_id TCONS_00030930; exon_number 5; oId CUFF.26909.1; tss_id TSS27956;
5	Cufflinks	exon	212075870	212078778	.	-	.	gene_id GRMZM2G163749; transcript_id TCONS_00030970; exon_number 1; oId CUFF.26977.1; tss_id TSS27992;
5	Cufflinks	exon	215883819	215884214	.	-	.	gene_id GRMZM2G013981; transcript_id TCONS_00031097; exon_number 1; oId CUFF.27193.1; tss_id TSS28101;
5	Cufflinks	exon	215884320	215884412	.	-	.	gene_id GRMZM2G013981; transcript_id TCONS_00031097; exon_number 2; oId CUFF.27193.1; tss_id TSS28101;
5	Cufflinks	exon	215884495	215884569	.	-	.	gene_id GRMZM2G013981; transcript_id TCONS_00031097; exon_number 3; oId CUFF.27193.1; tss_id TSS28101;
5	Cufflinks	exon	215885019	215885119	.	-	.	gene_id GRMZM2G013981; transcript_id TCONS_00031097; exon_number 4; oId CUFF.27193.1; tss_id TSS28101;
5	Cufflinks	exon	215885295	215885411	.	-	.	gene_id GRMZM2G013981; transcript_id TCONS_00031097; exon_number 5; oId CUFF.27193.1; tss_id TSS28101;
5	Cufflinks	exon	215885500	215885593	.	-	.	gene_id GRMZM2G013981; transcript_id TCONS_00031097; exon_number 6; oId CUFF.27193.1; tss_id TSS28101;
5	Cufflinks	exon	215885805	215885870	.	-	.	gene_id GRMZM2G013981; transcript_id TCONS_00031097; exon_number 7; oId CUFF.27193.1; tss_id TSS28101;
5	Cufflinks	exon	215886044	215886110	.	-	.	gene_id GRMZM2G013981; transcript_id TCONS_00031097; exon_number 8; oId CUFF.27193.1; tss_id TSS28101;
5	Cufflinks	exon	215886755	215887609	.	-	.	gene_id GRMZM2G013981; transcript_id TCONS_00031097; exon_number 9; oId CUFF.27193.1; tss_id TSS28101;
5	Cufflinks	exon	217664134	217664804	.	-	.	gene_id GRMZM2G073609; transcript_id TCONS_00031143; exon_number 1; oId CUFF.27254.1; tss_id TSS28144;
5	Cufflinks	exon	217664878	217665072	.	-	.	gene_id GRMZM2G073609; transcript_id TCONS_00031143; exon_number 2; oId CUFF.27254.1; tss_id TSS28144;
6	Cufflinks	exon	1280395	1282028	.	+	.	gene_id GRMZM2G098174; transcript_id TCONS_00031154; exon_number 1; oId CUFF.27304.2; tss_id TSS28153;
6	Cufflinks	exon	1282230	1282290	.	+	.	gene_id GRMZM2G098174; transcript_id TCONS_00031154; exon_number 2; oId CUFF.27304.2; tss_id TSS28153;
6	Cufflinks	exon	1283303	1283436	.	+	.	gene_id GRMZM2G098174; transcript_id TCONS_00031154; exon_number 3; oId CUFF.27304.2; tss_id TSS28153;
6	Cufflinks	exon	1283532	1283605	.	+	.	gene_id GRMZM2G098174; transcript_id TCONS_00031154; exon_number 4; oId CUFF.27304.2; tss_id TSS28153;
6	Cufflinks	exon	1283699	1284196	.	+	.	gene_id GRMZM2G098174; transcript_id TCONS_00031154; exon_number 5; oId CUFF.27304.2; tss_id TSS28153;
6	Cufflinks	exon	1286112	1286576	.	+	.	gene_id GRMZM2G098174; transcript_id TCONS_00031154; exon_number 6; oId CUFF.27304.2; tss_id TSS28153;
6	Cufflinks	exon	1286658	1286870	.	+	.	gene_id GRMZM2G098174; transcript_id TCONS_00031154; exon_number 7; oId CUFF.27304.2; tss_id TSS28153;
6	Cufflinks	exon	1287048	1288498	.	+	.	gene_id GRMZM2G098174; transcript_id TCONS_00031154; exon_number 8; oId CUFF.27304.2; tss_id TSS28153;
6	Cufflinks	exon	3237181	3237651	.	+	.	gene_id GRMZM2G117961; transcript_id TCONS_00031184; exon_number 1; oId CUFF.27335.1; tss_id TSS28178;
6	Cufflinks	exon	3237775	3237856	.	+	.	gene_id GRMZM2G117961; transcript_id TCONS_00031184; exon_number 2; oId CUFF.27335.1; tss_id TSS28178;
6	Cufflinks	exon	3238097	3238158	.	+	.	gene_id GRMZM2G117961; transcript_id TCONS_00031184; exon_number 3; oId CUFF.27335.1; tss_id TSS28178;
6	Cufflinks	exon	3238688	3238790	.	+	.	gene_id GRMZM2G117961; transcript_id TCONS_00031184; exon_number 4; oId CUFF.27335.1; tss_id TSS28178;
6	Cufflinks	exon	3238879	3238920	.	+	.	gene_id GRMZM2G117961; transcript_id TCONS_00031184; exon_number 5; oId CUFF.27335.1; tss_id TSS28178;
6	Cufflinks	exon	3239327	3239368	.	+	.	gene_id GRMZM2G117961; transcript_id TCONS_00031184; exon_number 6; oId CUFF.27335.1; tss_id TSS28178;
6	Cufflinks	exon	3239465	3239818	.	+	.	gene_id GRMZM2G117961; transcript_id TCONS_00031184; exon_number 7; oId CUFF.27335.1; tss_id TSS28178;
6	Cufflinks	exon	6186552	6190122	.	+	.	gene_id GRMZM2G364977; transcript_id TCONS_00031211; exon_number 1; oId CUFF.27389.1; tss_id TSS28202;
6	Cufflinks	exon	8109337	8109567	.	+	.	gene_id GRMZM2G068955; transcript_id TCONS_00031232; exon_number 1; oId CUFF.27414.1; tss_id TSS28223;
6	Cufflinks	exon	8110278	8110724	.	+	.	gene_id GRMZM2G068955; transcript_id TCONS_00031232; exon_number 2; oId CUFF.27414.1; tss_id TSS28223;
6	Cufflinks	exon	8110825	8111137	.	+	.	gene_id GRMZM2G068955; transcript_id TCONS_00031232; exon_number 3; oId CUFF.27414.1; tss_id TSS28223;

6	Cufflinks	exon	8111224	8111820	.	+	.	gene_id GRMZM2G068955; transcript_id TCONS_00031232; exon_number 4; oId CUFF.27414.1; tss_id TSS28223;
6	Cufflinks	exon	14969927	14974172	.	+	.	gene_id GRMZM2G172980; transcript_id TCONS_00031370; exon_number 1; oId CUFF.27722.1; tss_id TSS28359;
6	Cufflinks	exon	25702321	25703777	.	+	.	gene_id XLOC_027546; transcript_id TCONS_00031480; exon_number 1; oId CUFF.27898.1; tss_id TSS28461;
6	Cufflinks	exon	28140500	28140859	.	+	.	gene_id GRMZM2G313448; transcript_id TCONS_00031499; exon_number 1; oId CUFF.28026.1; tss_id TSS28478;
6	Cufflinks	exon	28142384	28142441	.	+	.	gene_id GRMZM2G313448; transcript_id TCONS_00031499; exon_number 2; oId CUFF.28026.1; tss_id TSS28478;
6	Cufflinks	exon	28144133	28144307	.	+	.	gene_id GRMZM2G313448; transcript_id TCONS_00031499; exon_number 3; oId CUFF.28026.1; tss_id TSS28478;
6	Cufflinks	exon	28144559	28146597	.	+	.	gene_id GRMZM2G313448; transcript_id TCONS_00031499; exon_number 4; oId CUFF.28026.1; tss_id TSS28478;
6	Cufflinks	exon	28141374	28141589	.	+	.	gene_id GRMZM2G313448; transcript_id TCONS_00031500; exon_number 1; oId CUFF.28026.2; tss_id TSS28479;
6	Cufflinks	exon	28142384	28142441	.	+	.	gene_id GRMZM2G313448; transcript_id TCONS_00031500; exon_number 2; oId CUFF.28026.2; tss_id TSS28479;
6	Cufflinks	exon	28144133	28144307	.	+	.	gene_id GRMZM2G313448; transcript_id TCONS_00031500; exon_number 3; oId CUFF.28026.2; tss_id TSS28479;
6	Cufflinks	exon	28144559	28146597	.	+	.	gene_id GRMZM2G313448; transcript_id TCONS_00031500; exon_number 4; oId CUFF.28026.2; tss_id TSS28479;
6	Cufflinks	exon	31415780	31415954	.	+	.	gene_id GRMZM2G099317; transcript_id TCONS_00031514; exon_number 1; oId CUFF.27977.1; tss_id TSS28491;
6	Cufflinks	exon	31417295	31417406	.	+	.	gene_id GRMZM2G099317; transcript_id TCONS_00031514; exon_number 2; oId CUFF.27977.1; tss_id TSS28491;
6	Cufflinks	exon	31418170	31418209	.	+	.	gene_id GRMZM2G099317; transcript_id TCONS_00031514; exon_number 3; oId CUFF.27977.1; tss_id TSS28491;
6	Cufflinks	exon	31418349	31418444	.	+	.	gene_id GRMZM2G099317; transcript_id TCONS_00031514; exon_number 4; oId CUFF.27977.1; tss_id TSS28491;
6	Cufflinks	exon	31418571	31418767	.	+	.	gene_id GRMZM2G099317; transcript_id TCONS_00031514; exon_number 5; oId CUFF.27977.1; tss_id TSS28491;
6	Cufflinks	exon	31418863	31418964	.	+	.	gene_id GRMZM2G099317; transcript_id TCONS_00031514; exon_number 6; oId CUFF.27977.1; tss_id TSS28491;
6	Cufflinks	exon	31419051	31420025	.	+	.	gene_id GRMZM2G099317; transcript_id TCONS_00031514; exon_number 7; oId CUFF.27977.1; tss_id TSS28491;
6	Cufflinks	exon	34889334	34891116	.	+	.	gene_id XLOC_027591; transcript_id TCONS_00031532; exon_number 1; oId CUFF.28022.1; tss_id TSS28507;
6	Cufflinks	exon	35061175	35062828	.	+	.	gene_id GRMZM2G001205; transcript_id TCONS_00031535; exon_number 1; oId CUFF.27998.1; tss_id TSS28510;
6	Cufflinks	exon	35892962	35893489	.	+	.	gene_id GRMZM2G035741; transcript_id TCONS_00031545; exon_number 1; oId CUFF.28045.5; tss_id TSS28516;
6	Cufflinks	exon	35893817	35894269	.	+	.	gene_id GRMZM2G035741; transcript_id TCONS_00031545; exon_number 2; oId CUFF.28045.5; tss_id TSS28516;
6	Cufflinks	exon	35895810	35896087	.	+	.	gene_id GRMZM2G035741; transcript_id TCONS_00031545; exon_number 3; oId CUFF.28045.5; tss_id TSS28516;
6	Cufflinks	exon	35896178	35896659	.	+	.	gene_id GRMZM2G035741; transcript_id TCONS_00031545; exon_number 4; oId CUFF.28045.5; tss_id TSS28516;
6	Cufflinks	exon	35892962	35893489	.	+	.	gene_id GRMZM2G035741; transcript_id TCONS_00031544; exon_number 1; oId CUFF.28045.4; tss_id TSS28516;
6	Cufflinks	exon	35893754	35894269	.	+	.	gene_id GRMZM2G035741; transcript_id TCONS_00031544; exon_number 2; oId CUFF.28045.4; tss_id TSS28516;
6	Cufflinks	exon	35895810	35896087	.	+	.	gene_id GRMZM2G035741; transcript_id TCONS_00031544; exon_number 3; oId CUFF.28045.4; tss_id TSS28516;
6	Cufflinks	exon	35896178	35896659	.	+	.	gene_id GRMZM2G035741; transcript_id TCONS_00031544; exon_number 4; oId CUFF.28045.4; tss_id TSS28516;
6	Cufflinks	exon	35892962	35894269	.	+	.	gene_id GRMZM2G035741; transcript_id TCONS_00031542; exon_number 1; oId CUFF.28045.2; tss_id TSS28516;
6	Cufflinks	exon	35895807	35896087	.	+	.	gene_id GRMZM2G035741; transcript_id TCONS_00031542; exon_number 2; oId CUFF.28045.2; tss_id TSS28516;
6	Cufflinks	exon	35896178	35896659	.	+	.	gene_id GRMZM2G035741; transcript_id TCONS_00031542; exon_number 3; oId CUFF.28045.2; tss_id TSS28516;
6	Cufflinks	exon	35892970	35893489	.	+	.	gene_id GRMZM2G035741; transcript_id TCONS_00031546; exon_number 1; oId CUFF.28045.6; tss_id TSS28516;
6	Cufflinks	exon	35893817	35894269	.	+	.	gene_id GRMZM2G035741; transcript_id TCONS_00031546; exon_number 2; oId CUFF.28045.6; tss_id TSS28516;
6	Cufflinks	exon	35895807	35896087	.	+	.	gene_id GRMZM2G035741; transcript_id TCONS_00031546; exon_number 3; oId CUFF.28045.6; tss_id TSS28516;
6	Cufflinks	exon	35896178	35896659	.	+	.	gene_id GRMZM2G035741; transcript_id TCONS_00031546; exon_number 4; oId CUFF.28045.6; tss_id TSS28516;
6	Cufflinks	exon	62703857	62704026	.	+	.	gene_id GRMZM2G077837; transcript_id TCONS_00031692; exon_number 1; oId CUFF.28303.1; tss_id TSS28651;

6	Cufflinks	exon	62704496	62704715	.	+	.	gene_id GRMZM2G077837; transcript_id TCONS_00031692; exon_number 2; oId CUFF.28303.1; tss_id TSS28651;
6	Cufflinks	exon	62704797	62704871	.	+	.	gene_id GRMZM2G077837; transcript_id TCONS_00031692; exon_number 3; oId CUFF.28303.1; tss_id TSS28651;
6	Cufflinks	exon	62704972	62705046	.	+	.	gene_id GRMZM2G077837; transcript_id TCONS_00031692; exon_number 4; oId CUFF.28303.1; tss_id TSS28651;
6	Cufflinks	exon	62705151	62705231	.	+	.	gene_id GRMZM2G077837; transcript_id TCONS_00031692; exon_number 5; oId CUFF.28303.1; tss_id TSS28651;
6	Cufflinks	exon	62705309	62705383	.	+	.	gene_id GRMZM2G077837; transcript_id TCONS_00031692; exon_number 6; oId CUFF.28303.1; tss_id TSS28651;
6	Cufflinks	exon	62705485	62705658	.	+	.	gene_id GRMZM2G077837; transcript_id TCONS_00031692; exon_number 7; oId CUFF.28303.1; tss_id TSS28651;
6	Cufflinks	exon	62705729	62705991	.	+	.	gene_id GRMZM2G077837; transcript_id TCONS_00031692; exon_number 8; oId CUFF.28303.1; tss_id TSS28651;
6	Cufflinks	exon	62703867	62704026	.	+	.	gene_id GRMZM2G077837; transcript_id TCONS_00031693; exon_number 1; oId CUFF.28303.2; tss_id TSS28651;
6	Cufflinks	exon	62704428	62704715	.	+	.	gene_id GRMZM2G077837; transcript_id TCONS_00031693; exon_number 2; oId CUFF.28303.2; tss_id TSS28651;
6	Cufflinks	exon	62704797	62704871	.	+	.	gene_id GRMZM2G077837; transcript_id TCONS_00031693; exon_number 3; oId CUFF.28303.2; tss_id TSS28651;
6	Cufflinks	exon	62704972	62705046	.	+	.	gene_id GRMZM2G077837; transcript_id TCONS_00031693; exon_number 4; oId CUFF.28303.2; tss_id TSS28651;
6	Cufflinks	exon	62705151	62705231	.	+	.	gene_id GRMZM2G077837; transcript_id TCONS_00031693; exon_number 5; oId CUFF.28303.2; tss_id TSS28651;
6	Cufflinks	exon	62705309	62705383	.	+	.	gene_id GRMZM2G077837; transcript_id TCONS_00031693; exon_number 6; oId CUFF.28303.2; tss_id TSS28651;
6	Cufflinks	exon	62705485	62705658	.	+	.	gene_id GRMZM2G077837; transcript_id TCONS_00031693; exon_number 7; oId CUFF.28303.2; tss_id TSS28651;
6	Cufflinks	exon	62705729	62705991	.	+	.	gene_id GRMZM2G077837; transcript_id TCONS_00031693; exon_number 8; oId CUFF.28303.2; tss_id TSS28651;
6	Cufflinks	exon	67615156	67615294	.	+	.	gene_id GRMZM2G430902; transcript_id TCONS_00031732; exon_number 1; oId CUFF.28350.1; tss_id TSS28687;
6	Cufflinks	exon	67615385	67615798	.	+	.	gene_id GRMZM2G430902; transcript_id TCONS_00031732; exon_number 2; oId CUFF.28350.1; tss_id TSS28687;
6	Cufflinks	exon	67615869	67617122	.	+	.	gene_id GRMZM2G430902; transcript_id TCONS_00031732; exon_number 3; oId CUFF.28350.1; tss_id TSS28687;
6	Cufflinks	exon	67617201	67617583	.	+	.	gene_id GRMZM2G430902; transcript_id TCONS_00031732; exon_number 4; oId CUFF.28350.1; tss_id TSS28687;
6	Cufflinks	exon	74071296	74073124	.	+	.	gene_id GRMZM2G362298; transcript_id TCONS_00031779; exon_number 1; oId CUFF.28440.1; tss_id TSS28733;
6	Cufflinks	exon	74073217	74073747	.	+	.	gene_id GRMZM2G362298; transcript_id TCONS_00031779; exon_number 2; oId CUFF.28440.1; tss_id TSS28733;
6	Cufflinks	exon	82128247	82128399	.	+	.	gene_id GRMZM2G020912; transcript_id TCONS_00031838; exon_number 1; oId CUFF.28535.1; tss_id TSS28787;
6	Cufflinks	exon	82128500	82128552	.	+	.	gene_id GRMZM2G020912; transcript_id TCONS_00031838; exon_number 2; oId CUFF.28535.1; tss_id TSS28787;
6	Cufflinks	exon	82129915	82130028	.	+	.	gene_id GRMZM2G020912; transcript_id TCONS_00031838; exon_number 3; oId CUFF.28535.1; tss_id TSS28787;
6	Cufflinks	exon	82130130	82130173	.	+	.	gene_id GRMZM2G020912; transcript_id TCONS_00031838; exon_number 4; oId CUFF.28535.1; tss_id TSS28787;
6	Cufflinks	exon	82130748	82130835	.	+	.	gene_id GRMZM2G020912; transcript_id TCONS_00031838; exon_number 5; oId CUFF.28535.1; tss_id TSS28787;
6	Cufflinks	exon	82130923	82133582	.	+	.	gene_id GRMZM2G020912; transcript_id TCONS_00031838; exon_number 6; oId CUFF.28535.1; tss_id TSS28787;
6	Cufflinks	exon	82133852	82133953	.	+	.	gene_id GRMZM2G020912; transcript_id TCONS_00031838; exon_number 7; oId CUFF.28535.1; tss_id TSS28787;
6	Cufflinks	exon	82134073	82134232	.	+	.	gene_id GRMZM2G020912; transcript_id TCONS_00031838; exon_number 8; oId CUFF.28535.1; tss_id TSS28787;
6	Cufflinks	exon	82134469	82135184	.	+	.	gene_id GRMZM2G020912; transcript_id TCONS_00031838; exon_number 9; oId CUFF.28535.1; tss_id TSS28787;
6	Cufflinks	exon	84094029	84094460	.	+	.	gene_id GRMZM2G065105; transcript_id TCONS_00031869; exon_number 1; oId CUFF.28586.1; tss_id TSS28813;
6	Cufflinks	exon	84094676	84094768	.	+	.	gene_id GRMZM2G065105; transcript_id TCONS_00031869; exon_number 2; oId CUFF.28586.1; tss_id TSS28813;
6	Cufflinks	exon	84095655	84095748	.	+	.	gene_id GRMZM2G065105; transcript_id TCONS_00031869; exon_number 3; oId CUFF.28586.1; tss_id TSS28813;
6	Cufflinks	exon	84095856	84095893	.	+	.	gene_id GRMZM2G065105; transcript_id TCONS_00031869; exon_number 4; oId CUFF.28586.1; tss_id TSS28813;
6	Cufflinks	exon	84096107	84096195	.	+	.	gene_id GRMZM2G065105; transcript_id TCONS_00031869; exon_number 5; oId CUFF.28586.1; tss_id TSS28813;
6	Cufflinks	exon	84096387	84096466	.	+	.	gene_id GRMZM2G065105; transcript_id TCONS_00031869; exon_number 6; oId CUFF.28586.1; tss_id TSS28813;

6	Cufflinks	exon	84096958	84097014	.	+	.	gene_id GRMZM2G065105; transcript_id TCONS_00031869; exon_number 7; oId CUFF.28586.1; tss_id TSS28813;
6	Cufflinks	exon	84097114	84097206	.	+	.	gene_id GRMZM2G065105; transcript_id TCONS_00031869; exon_number 8; oId CUFF.28586.1; tss_id TSS28813;
6	Cufflinks	exon	84097280	84097354	.	+	.	gene_id GRMZM2G065105; transcript_id TCONS_00031869; exon_number 9; oId CUFF.28586.1; tss_id TSS28813;
6	Cufflinks	exon	84098019	84098506	.	+	.	gene_id GRMZM2G065105; transcript_id TCONS_00031869; exon_number 10; oId CUFF.28586.1; tss_id TSS28813;
6	Cufflinks	exon	86051785	86052435	.	+	.	gene_id XLOC_027911; transcript_id TCONS_00031891; exon_number 1; oId CUFF.28606.1; tss_id TSS28833;
6	Cufflinks	exon	89061130	89061421	.	+	.	gene_id GRMZM2G150209; transcript_id TCONS_00031942; exon_number 1; oId CUFF.28691.1; tss_id TSS28877;
6	Cufflinks	exon	89061530	89061642	.	+	.	gene_id GRMZM2G150209; transcript_id TCONS_00031942; exon_number 2; oId CUFF.28691.1; tss_id TSS28877;
6	Cufflinks	exon	89061722	89061873	.	+	.	gene_id GRMZM2G150209; transcript_id TCONS_00031942; exon_number 3; oId CUFF.28691.1; tss_id TSS28877;
6	Cufflinks	exon	89061978	89062105	.	+	.	gene_id GRMZM2G150209; transcript_id TCONS_00031942; exon_number 4; oId CUFF.28691.1; tss_id TSS28877;
6	Cufflinks	exon	89062471	89062569	.	+	.	gene_id GRMZM2G150209; transcript_id TCONS_00031942; exon_number 5; oId CUFF.28691.1; tss_id TSS28877;
6	Cufflinks	exon	89063190	89063342	.	+	.	gene_id GRMZM2G150209; transcript_id TCONS_00031942; exon_number 6; oId CUFF.28691.1; tss_id TSS28877;
6	Cufflinks	exon	89063542	89063645	.	+	.	gene_id GRMZM2G150209; transcript_id TCONS_00031942; exon_number 7; oId CUFF.28691.1; tss_id TSS28877;
6	Cufflinks	exon	89063852	89063919	.	+	.	gene_id GRMZM2G150209; transcript_id TCONS_00031942; exon_number 8; oId CUFF.28691.1; tss_id TSS28877;
6	Cufflinks	exon	89063994	89064359	.	+	.	gene_id GRMZM2G150209; transcript_id TCONS_00031942; exon_number 9; oId CUFF.28691.1; tss_id TSS28877;
6	Cufflinks	exon	89084484	89085224	.	+	.	gene_id XLOC_027958; transcript_id TCONS_00031949; exon_number 1; oId CUFF.28702.1; tss_id TSS28884;
6	Cufflinks	exon	110002951	110003303	.	+	.	gene_id GRMZM2G136058; transcript_id TCONS_00032205; exon_number 1; oId CUFF.29151.1; tss_id TSS29122;
6	Cufflinks	exon	110006713	110006922	.	+	.	gene_id GRMZM2G136058; transcript_id TCONS_00032205; exon_number 2; oId CUFF.29151.1; tss_id TSS29122;
6	Cufflinks	exon	110007009	110007085	.	+	.	gene_id GRMZM2G136058; transcript_id TCONS_00032205; exon_number 3; oId CUFF.29151.1; tss_id TSS29122;
6	Cufflinks	exon	110007818	110008161	.	+	.	gene_id GRMZM2G136058; transcript_id TCONS_00032205; exon_number 4; oId CUFF.29151.1; tss_id TSS29122;
6	Cufflinks	exon	110052982	110053982	.	+	.	gene_id GRMZM2G135970; transcript_id TCONS_00032206; exon_number 1; oId CUFF.29148.1; tss_id TSS29123;
6	Cufflinks	exon	110054297	110054434	.	+	.	gene_id GRMZM2G135970; transcript_id TCONS_00032206; exon_number 2; oId CUFF.29148.1; tss_id TSS29123;
6	Cufflinks	exon	110054532	110054771	.	+	.	gene_id GRMZM2G135970; transcript_id TCONS_00032206; exon_number 3; oId CUFF.29148.1; tss_id TSS29123;
6	Cufflinks	exon	110054891	110055216	.	+	.	gene_id GRMZM2G135970; transcript_id TCONS_00032206; exon_number 4; oId CUFF.29148.1; tss_id TSS29123;
6	Cufflinks	exon	112149529	112150144	.	+	.	gene_id GRMZM2G118657; transcript_id TCONS_00032238; exon_number 1; oId CUFF.29210.1; tss_id TSS29154;
6	Cufflinks	exon	112150254	112151755	.	+	.	gene_id GRMZM2G118657; transcript_id TCONS_00032238; exon_number 2; oId CUFF.29210.1; tss_id TSS29154;
6	Cufflinks	exon	113900332	113901471	.	+	.	gene_id AC202439.3_FG001; transcript_id TCONS_00032253; exon_number 1; oId CUFF.29219.1; tss_id TSS29168;
6	Cufflinks	exon	116206148	116207201	.	+	.	gene_id GRMZM2G324375; transcript_id TCONS_00032281; exon_number 1; oId CUFF.29269.1; tss_id TSS29194;
6	Cufflinks	exon	125194477	125194792	.	+	.	gene_id GRMZM2G135108; transcript_id TCONS_00032385; exon_number 1; oId CUFF.29465.1; tss_id TSS29288;
6	Cufflinks	exon	125194908	125195366	.	+	.	gene_id GRMZM2G135108; transcript_id TCONS_00032385; exon_number 2; oId CUFF.29465.1; tss_id TSS29288;
6	Cufflinks	exon	125195466	125196129	.	+	.	gene_id GRMZM2G135108; transcript_id TCONS_00032385; exon_number 3; oId CUFF.29465.1; tss_id TSS29288;
6	Cufflinks	exon	127773719	127774046	.	+	.	gene_id GRMZM2G163054; transcript_id TCONS_00032404; exon_number 1; oId CUFF.29517.2; tss_id TSS29306;
6	Cufflinks	exon	127774173	127774313	.	+	.	gene_id GRMZM2G163054; transcript_id TCONS_00032404; exon_number 2; oId CUFF.29517.2; tss_id TSS29306;
6	Cufflinks	exon	127774475	127775141	.	+	.	gene_id GRMZM2G163054; transcript_id TCONS_00032404; exon_number 3; oId CUFF.29517.2; tss_id TSS29306;
6	Cufflinks	exon	127773719	127774046	.	+	.	gene_id GRMZM2G163054; transcript_id TCONS_00032403; exon_number 1; oId CUFF.29517.1; tss_id TSS29306;
6	Cufflinks	exon	127774176	127774313	.	+	.	gene_id GRMZM2G163054; transcript_id TCONS_00032403; exon_number 2; oId CUFF.29517.1; tss_id TSS29306;
6	Cufflinks	exon	127774475	127775141	.	+	.	gene_id GRMZM2G163054; transcript_id TCONS_00032403; exon_number 3; oId CUFF.29517.1; tss_id TSS29306;

6	Cufflinks	exon	127870096	127870493	.	+	.	gene_id GRMZM2G167387; transcript_id TCONS_00032407; exon_number 1; oId CUFF.29521.2; tss_id TSS29308;
6	Cufflinks	exon	127870571	127870710	.	+	.	gene_id GRMZM2G167387; transcript_id TCONS_00032407; exon_number 2; oId CUFF.29521.2; tss_id TSS29308;
6	Cufflinks	exon	127870856	127871796	.	+	.	gene_id GRMZM2G167387; transcript_id TCONS_00032407; exon_number 3; oId CUFF.29521.2; tss_id TSS29308;
6	Cufflinks	exon	127870096	127870493	.	+	.	gene_id GRMZM2G167387; transcript_id TCONS_00032406; exon_number 1; oId CUFF.29521.1; tss_id TSS29308;
6	Cufflinks	exon	127870616	127870710	.	+	.	gene_id GRMZM2G167387; transcript_id TCONS_00032406; exon_number 2; oId CUFF.29521.1; tss_id TSS29308;
6	Cufflinks	exon	127870856	127871796	.	+	.	gene_id GRMZM2G167387; transcript_id TCONS_00032406; exon_number 3; oId CUFF.29521.1; tss_id TSS29308;
6	Cufflinks	exon	128637993	128638947	.	+	.	gene_id GRMZM2G352618; transcript_id TCONS_00032412; exon_number 1; oId CUFF.29531.1; tss_id TSS29313;
6	Cufflinks	exon	135723001	135723954	.	+	.	gene_id GRMZM2G147245; transcript_id TCONS_00032509; exon_number 1; oId CUFF.29717.1; tss_id TSS29404;
6	Cufflinks	exon	135724231	135724352	.	+	.	gene_id GRMZM2G147245; transcript_id TCONS_00032509; exon_number 2; oId CUFF.29717.1; tss_id TSS29404;
6	Cufflinks	exon	135724625	135725403	.	+	.	gene_id GRMZM2G147245; transcript_id TCONS_00032509; exon_number 3; oId CUFF.29717.1; tss_id TSS29404;
6	Cufflinks	exon	139464334	139464559	.	+	.	gene_id GRMZM2G122479; transcript_id TCONS_00032545; exon_number 1; oId CUFF.29810.1; tss_id TSS29438;
6	Cufflinks	exon	139464779	139465047	.	+	.	gene_id GRMZM2G122479; transcript_id TCONS_00032545; exon_number 2; oId CUFF.29810.1; tss_id TSS29438;
6	Cufflinks	exon	139466261	139466381	.	+	.	gene_id GRMZM2G122479; transcript_id TCONS_00032545; exon_number 3; oId CUFF.29810.1; tss_id TSS29438;
6	Cufflinks	exon	139466475	139466549	.	+	.	gene_id GRMZM2G122479; transcript_id TCONS_00032545; exon_number 4; oId CUFF.29810.1; tss_id TSS29438;
6	Cufflinks	exon	139466675	139466826	.	+	.	gene_id GRMZM2G122479; transcript_id TCONS_00032545; exon_number 5; oId CUFF.29810.1; tss_id TSS29438;
6	Cufflinks	exon	139466921	139467026	.	+	.	gene_id GRMZM2G122479; transcript_id TCONS_00032545; exon_number 6; oId CUFF.29810.1; tss_id TSS29438;
6	Cufflinks	exon	139467096	139467161	.	+	.	gene_id GRMZM2G122479; transcript_id TCONS_00032545; exon_number 7; oId CUFF.29810.1; tss_id TSS29438;
6	Cufflinks	exon	139467277	139467372	.	+	.	gene_id GRMZM2G122479; transcript_id TCONS_00032545; exon_number 8; oId CUFF.29810.1; tss_id TSS29438;
6	Cufflinks	exon	139467470	139467541	.	+	.	gene_id GRMZM2G122479; transcript_id TCONS_00032545; exon_number 9; oId CUFF.29810.1; tss_id TSS29438;
6	Cufflinks	exon	139467627	139467710	.	+	.	gene_id GRMZM2G122479; transcript_id TCONS_00032545; exon_number 10; oId CUFF.29810.1; tss_id TSS29438;
6	Cufflinks	exon	139467847	139467945	.	+	.	gene_id GRMZM2G122479; transcript_id TCONS_00032545; exon_number 11; oId CUFF.29810.1; tss_id TSS29438;
6	Cufflinks	exon	139468113	139468160	.	+	.	gene_id GRMZM2G122479; transcript_id TCONS_00032545; exon_number 12; oId CUFF.29810.1; tss_id TSS29438;
6	Cufflinks	exon	139468256	139468309	.	+	.	gene_id GRMZM2G122479; transcript_id TCONS_00032545; exon_number 13; oId CUFF.29810.1; tss_id TSS29438;
6	Cufflinks	exon	139468398	139468496	.	+	.	gene_id GRMZM2G122479; transcript_id TCONS_00032545; exon_number 14; oId CUFF.29810.1; tss_id TSS29438;
6	Cufflinks	exon	139468619	139468708	.	+	.	gene_id GRMZM2G122479; transcript_id TCONS_00032545; exon_number 15; oId CUFF.29810.1; tss_id TSS29438;
6	Cufflinks	exon	139468797	139468874	.	+	.	gene_id GRMZM2G122479; transcript_id TCONS_00032545; exon_number 16; oId CUFF.29810.1; tss_id TSS29438;
6	Cufflinks	exon	139469080	139469154	.	+	.	gene_id GRMZM2G122479; transcript_id TCONS_00032545; exon_number 17; oId CUFF.29810.1; tss_id TSS29438;
6	Cufflinks	exon	139469255	139469345	.	+	.	gene_id GRMZM2G122479; transcript_id TCONS_00032545; exon_number 18; oId CUFF.29810.1; tss_id TSS29438;
6	Cufflinks	exon	139469420	139469548	.	+	.	gene_id GRMZM2G122479; transcript_id TCONS_00032545; exon_number 19; oId CUFF.29810.1; tss_id TSS29438;
6	Cufflinks	exon	139469634	139470019	.	+	.	gene_id GRMZM2G122479; transcript_id TCONS_00032545; exon_number 20; oId CUFF.29810.1; tss_id TSS29438;
6	Cufflinks	exon	142833016	142835191	.	+	.	gene_id GRMZM2G132478; transcript_id TCONS_00032594; exon_number 1; oId CUFF.29897.1; tss_id TSS29483;
6	Cufflinks	exon	146310907	146311358	.	+	.	gene_id GRMZM2G173630; transcript_id TCONS_00032648; exon_number 1; oId CUFF.29989.1; tss_id TSS29534;
6	Cufflinks	exon	146311882	146312944	.	+	.	gene_id GRMZM2G173630; transcript_id TCONS_00032648; exon_number 2; oId CUFF.29989.1; tss_id TSS29534;
6	Cufflinks	exon	146313099	146313590	.	+	.	gene_id GRMZM2G173630; transcript_id TCONS_00032648; exon_number 3; oId CUFF.29989.1; tss_id TSS29534;
6	Cufflinks	exon	152066590	152067328	.	+	.	gene_id XLOC_028691; transcript_id TCONS_00032756; exon_number 1; oId CUFF.30187.1; tss_id TSS29638;
6	Cufflinks	exon	153503421	153503766	.	+	.	gene_id GRMZM2G127297; transcript_id TCONS_00032781; exon_number 1; oId CUFF.30251.1; tss_id TSS29660;

6	Cufflinks	exon	153504755	153504833	.	+	.	gene_id GRMZM2G127297; transcript_id TCONS_00032781; exon_number 2; oId CUFF.30251.1; tss_id TSS29660;
6	Cufflinks	exon	153504955	153505053	.	+	.	gene_id GRMZM2G127297; transcript_id TCONS_00032781; exon_number 3; oId CUFF.30251.1; tss_id TSS29660;
6	Cufflinks	exon	153505870	153505983	.	+	.	gene_id GRMZM2G127297; transcript_id TCONS_00032781; exon_number 4; oId CUFF.30251.1; tss_id TSS29660;
6	Cufflinks	exon	153506197	153506339	.	+	.	gene_id GRMZM2G127297; transcript_id TCONS_00032781; exon_number 5; oId CUFF.30251.1; tss_id TSS29660;
6	Cufflinks	exon	153506447	153506614	.	+	.	gene_id GRMZM2G127297; transcript_id TCONS_00032781; exon_number 6; oId CUFF.30251.1; tss_id TSS29660;
6	Cufflinks	exon	153506752	153506976	.	+	.	gene_id GRMZM2G127297; transcript_id TCONS_00032781; exon_number 7; oId CUFF.30251.1; tss_id TSS29660;
6	Cufflinks	exon	153507078	153508370	.	+	.	gene_id GRMZM2G127297; transcript_id TCONS_00032781; exon_number 8; oId CUFF.30251.1; tss_id TSS29660;
6	Cufflinks	exon	155074658	155076625	.	+	.	gene_id XLOC_028750; transcript_id TCONS_00032822; exon_number 1; oId CUFF.30309.1; tss_id TSS29698;
6	Cufflinks	exon	158380660	158380948	.	+	.	gene_id GRMZM2G375904; transcript_id TCONS_00032899; exon_number 1; oId CUFF.30480.1; tss_id TSS29767;
6	Cufflinks	exon	158381576	158381971	.	+	.	gene_id GRMZM2G375904; transcript_id TCONS_00032899; exon_number 2; oId CUFF.30480.1; tss_id TSS29767;
6	Cufflinks	exon	158382691	158382865	.	+	.	gene_id GRMZM2G375904; transcript_id TCONS_00032899; exon_number 3; oId CUFF.30480.1; tss_id TSS29767;
6	Cufflinks	exon	158383872	158383954	.	+	.	gene_id GRMZM2G375904; transcript_id TCONS_00032899; exon_number 4; oId CUFF.30480.1; tss_id TSS29767;
6	Cufflinks	exon	158384041	158384282	.	+	.	gene_id GRMZM2G375904; transcript_id TCONS_00032899; exon_number 5; oId CUFF.30480.1; tss_id TSS29767;
6	Cufflinks	exon	158384374	158384608	.	+	.	gene_id GRMZM2G375904; transcript_id TCONS_00032899; exon_number 6; oId CUFF.30480.1; tss_id TSS29767;
6	Cufflinks	exon	158385250	158385329	.	+	.	gene_id GRMZM2G375904; transcript_id TCONS_00032899; exon_number 7; oId CUFF.30480.1; tss_id TSS29767;
6	Cufflinks	exon	158385442	158385526	.	+	.	gene_id GRMZM2G375904; transcript_id TCONS_00032899; exon_number 8; oId CUFF.30480.1; tss_id TSS29767;
6	Cufflinks	exon	158386565	158386696	.	+	.	gene_id GRMZM2G375904; transcript_id TCONS_00032899; exon_number 9; oId CUFF.30480.1; tss_id TSS29767;
6	Cufflinks	exon	158386783	158386896	.	+	.	gene_id GRMZM2G375904; transcript_id TCONS_00032899; exon_number 10; oId CUFF.30480.1; tss_id TSS29767;
6	Cufflinks	exon	158387544	158387591	.	+	.	gene_id GRMZM2G375904; transcript_id TCONS_00032899; exon_number 11; oId CUFF.30480.1; tss_id TSS29767;
6	Cufflinks	exon	158387983	158388067	.	+	.	gene_id GRMZM2G375904; transcript_id TCONS_00032899; exon_number 12; oId CUFF.30480.1; tss_id TSS29767;
6	Cufflinks	exon	158388175	158388231	.	+	.	gene_id GRMZM2G375904; transcript_id TCONS_00032899; exon_number 13; oId CUFF.30480.1; tss_id TSS29767;
6	Cufflinks	exon	158388325	158388837	.	+	.	gene_id GRMZM2G375904; transcript_id TCONS_00032899; exon_number 14; oId CUFF.30480.1; tss_id TSS29767;
6	Cufflinks	exon	158417573	158418960	.	+	.	gene_id GRMZM2G074792; transcript_id TCONS_00032902; exon_number 1; oId CUFF.30457.1; tss_id TSS29770;
6	Cufflinks	exon	158419530	158419838	.	+	.	gene_id GRMZM2G074792; transcript_id TCONS_00032902; exon_number 2; oId CUFF.30457.1; tss_id TSS29770;
6	Cufflinks	exon	158419926	158420216	.	+	.	gene_id GRMZM2G074792; transcript_id TCONS_00032902; exon_number 3; oId CUFF.30457.1; tss_id TSS29770;
6	Cufflinks	exon	158420319	158420432	.	+	.	gene_id GRMZM2G074792; transcript_id TCONS_00032902; exon_number 4; oId CUFF.30457.1; tss_id TSS29770;
6	Cufflinks	exon	158420876	158422014	.	+	.	gene_id GRMZM2G074792; transcript_id TCONS_00032902; exon_number 5; oId CUFF.30457.1; tss_id TSS29770;
6	Cufflinks	exon	160191073	160191402	.	+	.	gene_id GRMZM2G004349; transcript_id TCONS_00032951; exon_number 1; oId CUFF.30532.1; tss_id TSS29817;
6	Cufflinks	exon	160191989	160192169	.	+	.	gene_id GRMZM2G004349; transcript_id TCONS_00032951; exon_number 2; oId CUFF.30532.1; tss_id TSS29817;
6	Cufflinks	exon	160192390	160192531	.	+	.	gene_id GRMZM2G004349; transcript_id TCONS_00032951; exon_number 3; oId CUFF.30532.1; tss_id TSS29817;
6	Cufflinks	exon	160192626	160192695	.	+	.	gene_id GRMZM2G004349; transcript_id TCONS_00032951; exon_number 4; oId CUFF.30532.1; tss_id TSS29817;
6	Cufflinks	exon	160192772	160193306	.	+	.	gene_id GRMZM2G004349; transcript_id TCONS_00032951; exon_number 5; oId CUFF.30532.1; tss_id TSS29817;
6	Cufflinks	exon	161647173	161648309	.	+	.	gene_id XLOC_028905; transcript_id TCONS_00032993; exon_number 1; oId CUFF.30604.1; tss_id TSS29856;
6	Cufflinks	exon	162746428	162748871	.	+	.	gene_id GRMZM2G179797; transcript_id TCONS_00033032; exon_number 1; oId CUFF.30665.1; tss_id TSS29891;
6	Cufflinks	exon	163081648	163083193	.	+	.	gene_id GRMZM2G037998; transcript_id TCONS_00033045; exon_number 1; oId CUFF.30692.1; tss_id TSS29903;
6	Cufflinks	exon	167082887	167083041	.	+	.	gene_id GRMZM2G159028; transcript_id TCONS_00033155; exon_number 1; oId CUFF.30884.1; tss_id TSS30005;

6	Cufflinks	exon	167084398	167085534	.	+	.	gene_id GRMZM2G159028; transcript_id TCONS_00033155; exon_number 2; oId CUFF.30884.1; tss_id TSS30005;
6	Cufflinks	exon	167774471	167774632	.	+	.	gene_id GRMZM2G070659; transcript_id TCONS_00033170; exon_number 1; oId CUFF.30931.3; tss_id TSS30017;
6	Cufflinks	exon	167775803	167776029	.	+	.	gene_id GRMZM2G070659; transcript_id TCONS_00033170; exon_number 2; oId CUFF.30931.3; tss_id TSS30017;
6	Cufflinks	exon	167776103	167776223	.	+	.	gene_id GRMZM2G070659; transcript_id TCONS_00033170; exon_number 3; oId CUFF.30931.3; tss_id TSS30017;
6	Cufflinks	exon	167776288	167777115	.	+	.	gene_id GRMZM2G070659; transcript_id TCONS_00033170; exon_number 4; oId CUFF.30931.3; tss_id TSS30017;
6	Cufflinks	exon	167775104	167775337	.	+	.	gene_id GRMZM2G070659; transcript_id TCONS_00033171; exon_number 1; oId CUFF.30931.4; tss_id TSS30018;
6	Cufflinks	exon	167775791	167776029	.	+	.	gene_id GRMZM2G070659; transcript_id TCONS_00033171; exon_number 2; oId CUFF.30931.4; tss_id TSS30018;
6	Cufflinks	exon	167776103	167776223	.	+	.	gene_id GRMZM2G070659; transcript_id TCONS_00033171; exon_number 3; oId CUFF.30931.4; tss_id TSS30018;
6	Cufflinks	exon	167776288	167777115	.	+	.	gene_id GRMZM2G070659; transcript_id TCONS_00033171; exon_number 4; oId CUFF.30931.4; tss_id TSS30018;
6	Cufflinks	exon	167775117	167775341	.	+	.	gene_id GRMZM2G070659; transcript_id TCONS_00033172; exon_number 1; oId CUFF.30931.5; tss_id TSS30018;
6	Cufflinks	exon	167775803	167776029	.	+	.	gene_id GRMZM2G070659; transcript_id TCONS_00033172; exon_number 2; oId CUFF.30931.5; tss_id TSS30018;
6	Cufflinks	exon	167776103	167776223	.	+	.	gene_id GRMZM2G070659; transcript_id TCONS_00033172; exon_number 3; oId CUFF.30931.5; tss_id TSS30018;
6	Cufflinks	exon	167776288	167777115	.	+	.	gene_id GRMZM2G070659; transcript_id TCONS_00033172; exon_number 4; oId CUFF.30931.5; tss_id TSS30018;
6	Cufflinks	exon	167775135	167775337	.	+	.	gene_id GRMZM2G070659; transcript_id TCONS_00033173; exon_number 1; oId CUFF.30931.6; tss_id TSS30018;
6	Cufflinks	exon	167775803	167776029	.	+	.	gene_id GRMZM2G070659; transcript_id TCONS_00033173; exon_number 2; oId CUFF.30931.6; tss_id TSS30018;
6	Cufflinks	exon	167776103	167776223	.	+	.	gene_id GRMZM2G070659; transcript_id TCONS_00033173; exon_number 3; oId CUFF.30931.6; tss_id TSS30018;
6	Cufflinks	exon	167776288	167777115	.	+	.	gene_id GRMZM2G070659; transcript_id TCONS_00033173; exon_number 4; oId CUFF.30931.6; tss_id TSS30018;
6	Cufflinks	exon	168897219	168898900	.	+	.	gene_id XLOC_029073; transcript_id TCONS_00033189; exon_number 1; oId CUFF.30937.1; tss_id TSS30032;
6	Cufflinks	exon	169124326	169124842	.	+	.	gene_id GRMZM5G833747; transcript_id TCONS_00033192; exon_number 1; oId CUFF.30943.1; tss_id TSS30034;
6	Cufflinks	exon	169124998	169125123	.	+	.	gene_id GRMZM5G833747; transcript_id TCONS_00033192; exon_number 2; oId CUFF.30943.1; tss_id TSS30034;
6	Cufflinks	exon	169125220	169125257	.	+	.	gene_id GRMZM5G833747; transcript_id TCONS_00033192; exon_number 3; oId CUFF.30943.1; tss_id TSS30034;
6	Cufflinks	exon	169125451	169125501	.	+	.	gene_id GRMZM5G833747; transcript_id TCONS_00033192; exon_number 4; oId CUFF.30943.1; tss_id TSS30034;
6	Cufflinks	exon	169125643	169125810	.	+	.	gene_id GRMZM5G833747; transcript_id TCONS_00033192; exon_number 5; oId CUFF.30943.1; tss_id TSS30034;
6	Cufflinks	exon	169125889	169126001	.	+	.	gene_id GRMZM5G833747; transcript_id TCONS_00033192; exon_number 6; oId CUFF.30943.1; tss_id TSS30034;
6	Cufflinks	exon	169126093	169127492	.	+	.	gene_id GRMZM5G833747; transcript_id TCONS_00033192; exon_number 7; oId CUFF.30943.1; tss_id TSS30034;
6	Cufflinks	exon	655906	657038	.	-	.	gene_id GRMZM2G358540; transcript_id TCONS_00033212; exon_number 1; oId CUFF.27260.1; tss_id TSS30050;
6	Cufflinks	exon	2724260	2725338	.	-	.	gene_id GRMZM2G367941; transcript_id TCONS_00033238; exon_number 1; oId CUFF.27302.1; tss_id TSS30074;
6	Cufflinks	exon	2725567	2725829	.	-	.	gene_id GRMZM2G367941; transcript_id TCONS_00033238; exon_number 2; oId CUFF.27302.1; tss_id TSS30074;
6	Cufflinks	exon	3239342	3240135	.	-	.	gene_id GRMZM2G117984; transcript_id TCONS_00033254; exon_number 1; oId CUFF.27336.1; tss_id TSS30084;
6	Cufflinks	exon	3251477	3251620	.	-	.	gene_id GRMZM2G117984; transcript_id TCONS_00033254; exon_number 2; oId CUFF.27336.1; tss_id TSS30084;
6	Cufflinks	exon	3251754	3251856	.	-	.	gene_id GRMZM2G117984; transcript_id TCONS_00033254; exon_number 3; oId CUFF.27336.1; tss_id TSS30084;
6	Cufflinks	exon	3251931	3252177	.	-	.	gene_id GRMZM2G117984; transcript_id TCONS_00033254; exon_number 4; oId CUFF.27336.1; tss_id TSS30084;
6	Cufflinks	exon	6434660	6435755	.	-	.	gene_id GRMZM2G009661; transcript_id TCONS_00033294; exon_number 1; oId CUFF.27394.1; tss_id TSS30119;
6	Cufflinks	exon	6436774	6438078	.	-	.	gene_id GRMZM2G009661; transcript_id TCONS_00033294; exon_number 2; oId CUFF.27394.1; tss_id TSS30119;
6	Cufflinks	exon	6438138	6438643	.	-	.	gene_id GRMZM2G009661; transcript_id TCONS_00033294; exon_number 3; oId CUFF.27394.1; tss_id TSS30119;
6	Cufflinks	exon	8112897	8113352	.	-	.	gene_id GRMZM2G136662; transcript_id TCONS_00033312; exon_number 1; oId CUFF.27443.1; tss_id TSS30135;

6	Cufflinks	exon	8113781	8113873	.	-	.	gene_id GRMZM2G136662; transcript_id TCONS_00033312; exon_number 2; oId CUFF.27443.1; tss_id TSS30135;
6	Cufflinks	exon	8113998	8114123	.	-	.	gene_id GRMZM2G136662; transcript_id TCONS_00033312; exon_number 3; oId CUFF.27443.1; tss_id TSS30135;
6	Cufflinks	exon	8114213	8114323	.	-	.	gene_id GRMZM2G136662; transcript_id TCONS_00033312; exon_number 4; oId CUFF.27443.1; tss_id TSS30135;
6	Cufflinks	exon	8115932	8116051	.	-	.	gene_id GRMZM2G136662; transcript_id TCONS_00033312; exon_number 5; oId CUFF.27443.1; tss_id TSS30135;
6	Cufflinks	exon	8116417	8116509	.	-	.	gene_id GRMZM2G136662; transcript_id TCONS_00033312; exon_number 6; oId CUFF.27443.1; tss_id TSS30135;
6	Cufflinks	exon	8134304	8134450	.	-	.	gene_id GRMZM2G136662; transcript_id TCONS_00033312; exon_number 7; oId CUFF.27443.1; tss_id TSS30135;
6	Cufflinks	exon	8134853	8135107	.	-	.	gene_id GRMZM2G136662; transcript_id TCONS_00033312; exon_number 8; oId CUFF.27443.1; tss_id TSS30135;
6	Cufflinks	exon	8135183	8135417	.	-	.	gene_id GRMZM2G136662; transcript_id TCONS_00033312; exon_number 9; oId CUFF.27443.1; tss_id TSS30135;
6	Cufflinks	exon	8136561	8136923	.	-	.	gene_id GRMZM2G136662; transcript_id TCONS_00033312; exon_number 10; oId CUFF.27443.1; tss_id TSS30135;
6	Cufflinks	exon	9496444	9497226	.	-	.	gene_id XLOC_029184; transcript_id TCONS_00033328; exon_number 1; oId CUFF.27442.1; tss_id TSS30148;
6	Cufflinks	exon	9497383	9497703	.	-	.	gene_id XLOC_029184; transcript_id TCONS_00033328; exon_number 2; oId CUFF.27442.1; tss_id TSS30148;
6	Cufflinks	exon	9497808	9497921	.	-	.	gene_id XLOC_029184; transcript_id TCONS_00033328; exon_number 3; oId CUFF.27442.1; tss_id TSS30148;
6	Cufflinks	exon	9498229	9498281	.	-	.	gene_id XLOC_029184; transcript_id TCONS_00033328; exon_number 4; oId CUFF.27442.1; tss_id TSS30148;
6	Cufflinks	exon	9498406	9498477	.	-	.	gene_id XLOC_029184; transcript_id TCONS_00033328; exon_number 5; oId CUFF.27442.1; tss_id TSS30148;
6	Cufflinks	exon	9498611	9498691	.	-	.	gene_id XLOC_029184; transcript_id TCONS_00033328; exon_number 6; oId CUFF.27442.1; tss_id TSS30148;
6	Cufflinks	exon	9498877	9499331	.	-	.	gene_id XLOC_029184; transcript_id TCONS_00033328; exon_number 7; oId CUFF.27442.1; tss_id TSS30148;
6	Cufflinks	exon	21463563	21465430	.	-	.	gene_id GRMZM2G155252; transcript_id TCONS_00033549; exon_number 1; oId CUFF.27851.1; tss_id TSS30358;
6	Cufflinks	exon	24318350	24319143	.	-	.	gene_id GRMZM2G119583; transcript_id TCONS_00033567; exon_number 1; oId CUFF.27895.1; tss_id TSS30373;
6	Cufflinks	exon	24319468	24319902	.	-	.	gene_id GRMZM2G119583; transcript_id TCONS_00033567; exon_number 2; oId CUFF.27895.1; tss_id TSS30373;
6	Cufflinks	exon	24320023	24320654	.	-	.	gene_id GRMZM2G119583; transcript_id TCONS_00033567; exon_number 3; oId CUFF.27895.1; tss_id TSS30373;
6	Cufflinks	exon	24549334	24549972	.	-	.	gene_id GRMZM2G111579; transcript_id TCONS_00033568; exon_number 1; oId CUFF.27899.1; tss_id TSS30374;
6	Cufflinks	exon	24550058	24550127	.	-	.	gene_id GRMZM2G111579; transcript_id TCONS_00033568; exon_number 2; oId CUFF.27899.1; tss_id TSS30374;
6	Cufflinks	exon	24550202	24550282	.	-	.	gene_id GRMZM2G111579; transcript_id TCONS_00033568; exon_number 3; oId CUFF.27899.1; tss_id TSS30374;
6	Cufflinks	exon	24550584	24550676	.	-	.	gene_id GRMZM2G111579; transcript_id TCONS_00033568; exon_number 4; oId CUFF.27899.1; tss_id TSS30374;
6	Cufflinks	exon	24550838	24550918	.	-	.	gene_id GRMZM2G111579; transcript_id TCONS_00033568; exon_number 5; oId CUFF.27899.1; tss_id TSS30374;
6	Cufflinks	exon	24551005	24551094	.	-	.	gene_id GRMZM2G111579; transcript_id TCONS_00033568; exon_number 6; oId CUFF.27899.1; tss_id TSS30374;
6	Cufflinks	exon	24551250	24551343	.	-	.	gene_id GRMZM2G111579; transcript_id TCONS_00033568; exon_number 7; oId CUFF.27899.1; tss_id TSS30374;
6	Cufflinks	exon	24551466	24551534	.	-	.	gene_id GRMZM2G111579; transcript_id TCONS_00033568; exon_number 8; oId CUFF.27899.1; tss_id TSS30374;
6	Cufflinks	exon	24551616	24551686	.	-	.	gene_id GRMZM2G111579; transcript_id TCONS_00033568; exon_number 9; oId CUFF.27899.1; tss_id TSS30374;
6	Cufflinks	exon	24551760	24551828	.	-	.	gene_id GRMZM2G111579; transcript_id TCONS_00033568; exon_number 10; oId CUFF.27899.1; tss_id TSS30374;
6	Cufflinks	exon	24552005	24552088	.	-	.	gene_id GRMZM2G111579; transcript_id TCONS_00033568; exon_number 11; oId CUFF.27899.1; tss_id TSS30374;
6	Cufflinks	exon	24552624	24552716	.	-	.	gene_id GRMZM2G111579; transcript_id TCONS_00033568; exon_number 12; oId CUFF.27899.1; tss_id TSS30374;
6	Cufflinks	exon	24553063	24553170	.	-	.	gene_id GRMZM2G111579; transcript_id TCONS_00033568; exon_number 13; oId CUFF.27899.1; tss_id TSS30374;
6	Cufflinks	exon	24553395	24553545	.	-	.	gene_id GRMZM2G111579; transcript_id TCONS_00033568; exon_number 14; oId CUFF.27899.1; tss_id TSS30374;
6	Cufflinks	exon	24553943	24554137	.	-	.	gene_id GRMZM2G111579; transcript_id TCONS_00033568; exon_number 15; oId CUFF.27899.1; tss_id TSS30374;
6	Cufflinks	exon	24554165	24554223	.	-	.	gene_id GRMZM2G111579; transcript_id TCONS_00033568; exon_number 16; oId CUFF.27899.1; tss_id TSS30374;

6	Cufflinks	exon	24554306	24554738	.	-	.	gene_id GRMZM2G111579; transcript_id TCONS_00033568; exon_number 17; oId CUFF.27899.1; tss_id TSS30374;
6	Cufflinks	exon	25700899	25703378	.	-	.	gene_id GRMZM2G016637; transcript_id TCONS_00033575; exon_number 1; oId CUFF.27897.1; tss_id TSS30381;
6	Cufflinks	exon	25703594	25705088	.	-	.	gene_id GRMZM2G016637; transcript_id TCONS_00033575; exon_number 2; oId CUFF.27897.1; tss_id TSS30381;
6	Cufflinks	exon	26720745	26721236	.	-	.	gene_id GRMZM2G531804; transcript_id TCONS_00033584; exon_number 1; oId CUFF.27913.2; tss_id TSS30389;
6	Cufflinks	exon	26721944	26722230	.	-	.	gene_id GRMZM2G531804; transcript_id TCONS_00033584; exon_number 2; oId CUFF.27913.2; tss_id TSS30389;
6	Cufflinks	exon	34888892	34890020	.	-	.	gene_id GRMZM2G305856; transcript_id TCONS_00033649; exon_number 1; oId CUFF.28021.2; tss_id TSS30446;
6	Cufflinks	exon	34890220	34890920	.	-	.	gene_id GRMZM2G305856; transcript_id TCONS_00033649; exon_number 2; oId CUFF.28021.2; tss_id TSS30446;
6	Cufflinks	exon	34888892	34890020	.	-	.	gene_id GRMZM2G305856; transcript_id TCONS_00033648; exon_number 1; oId CUFF.28021.1; tss_id TSS30446;
6	Cufflinks	exon	34890278	34890920	.	-	.	gene_id GRMZM2G305856; transcript_id TCONS_00033648; exon_number 2; oId CUFF.28021.1; tss_id TSS30446;
6	Cufflinks	exon	39529258	39529737	.	-	.	gene_id GRMZM2G066326; transcript_id TCONS_00033679; exon_number 1; oId CUFF.28065.1; tss_id TSS30476;
6	Cufflinks	exon	39529819	39530806	.	-	.	gene_id GRMZM2G066326; transcript_id TCONS_00033679; exon_number 2; oId CUFF.28065.1; tss_id TSS30476;
6	Cufflinks	exon	56261420	56263747	.	-	.	gene_id GRMZM2G107073; transcript_id TCONS_00033771; exon_number 1; oId CUFF.28241.1; tss_id TSS30556;
6	Cufflinks	exon	56264102	56264391	.	-	.	gene_id GRMZM2G107073; transcript_id TCONS_00033771; exon_number 2; oId CUFF.28241.1; tss_id TSS30556;
6	Cufflinks	exon	56376617	56376860	.	-	.	gene_id GRMZM2G385287; transcript_id TCONS_00033772; exon_number 1; oId CUFF.28219.1; tss_id TSS30557;
6	Cufflinks	exon	56377551	56377673	.	-	.	gene_id GRMZM2G385287; transcript_id TCONS_00033772; exon_number 2; oId CUFF.28219.1; tss_id TSS30557;
6	Cufflinks	exon	56377796	56378293	.	-	.	gene_id GRMZM2G385287; transcript_id TCONS_00033772; exon_number 3; oId CUFF.28219.1; tss_id TSS30557;
6	Cufflinks	exon	57903594	57905295	.	-	.	gene_id GRMZM2G127798; transcript_id TCONS_00033778; exon_number 1; oId CUFF.28246.2; tss_id TSS30562;
6	Cufflinks	exon	57905959	57906115	.	-	.	gene_id GRMZM2G127798; transcript_id TCONS_00033778; exon_number 2; oId CUFF.28246.2; tss_id TSS30562;
6	Cufflinks	exon	57903594	57905300	.	-	.	gene_id GRMZM2G127798; transcript_id TCONS_00033777; exon_number 1; oId CUFF.28246.1; tss_id TSS30562;
6	Cufflinks	exon	57905959	57906115	.	-	.	gene_id GRMZM2G127798; transcript_id TCONS_00033777; exon_number 2; oId CUFF.28246.1; tss_id TSS30562;
6	Cufflinks	exon	82129100	82129534	.	-	.	gene_id XLOC_029728; transcript_id TCONS_00033936; exon_number 1; oId CUFF.28536.1; tss_id TSS30708;
6	Cufflinks	exon	83989254	83989982	.	-	.	gene_id GRMZM2G065888; transcript_id TCONS_00033954; exon_number 1; oId CUFF.28594.2; tss_id TSS30722;
6	Cufflinks	exon	83990091	83990167	.	-	.	gene_id GRMZM2G065888; transcript_id TCONS_00033954; exon_number 2; oId CUFF.28594.2; tss_id TSS30722;
6	Cufflinks	exon	83990362	83990478	.	-	.	gene_id GRMZM2G065888; transcript_id TCONS_00033954; exon_number 3; oId CUFF.28594.2; tss_id TSS30722;
6	Cufflinks	exon	83990647	83990747	.	-	.	gene_id GRMZM2G065888; transcript_id TCONS_00033954; exon_number 4; oId CUFF.28594.2; tss_id TSS30722;
6	Cufflinks	exon	83990825	83990894	.	-	.	gene_id GRMZM2G065888; transcript_id TCONS_00033954; exon_number 5; oId CUFF.28594.2; tss_id TSS30722;
6	Cufflinks	exon	83991085	83991179	.	-	.	gene_id GRMZM2G065888; transcript_id TCONS_00033954; exon_number 6; oId CUFF.28594.2; tss_id TSS30722;
6	Cufflinks	exon	83991733	83991826	.	-	.	gene_id GRMZM2G065888; transcript_id TCONS_00033954; exon_number 7; oId CUFF.28594.2; tss_id TSS30722;
6	Cufflinks	exon	83992121	83992211	.	-	.	gene_id GRMZM2G065888; transcript_id TCONS_00033954; exon_number 8; oId CUFF.28594.2; tss_id TSS30722;
6	Cufflinks	exon	83992290	83992397	.	-	.	gene_id GRMZM2G065888; transcript_id TCONS_00033954; exon_number 9; oId CUFF.28594.2; tss_id TSS30722;
6	Cufflinks	exon	83992495	83992808	.	-	.	gene_id GRMZM2G065888; transcript_id TCONS_00033954; exon_number 10; oId CUFF.28594.2; tss_id TSS30722;
6	Cufflinks	exon	83994068	83994612	.	-	.	gene_id GRMZM2G065888; transcript_id TCONS_00033954; exon_number 11; oId CUFF.28594.2; tss_id TSS30722;
6	Cufflinks	exon	85681129	85681774	.	-	.	gene_id GRMZM2G308689; transcript_id TCONS_00033969; exon_number 1; oId CUFF.28601.1; tss_id TSS30737;
6	Cufflinks	exon	85681867	85682003	.	-	.	gene_id GRMZM2G308689; transcript_id TCONS_00033969; exon_number 2; oId CUFF.28601.1; tss_id TSS30737;
6	Cufflinks	exon	85682741	85682981	.	-	.	gene_id GRMZM2G308689; transcript_id TCONS_00033969; exon_number 3; oId CUFF.28601.1; tss_id TSS30737;
6	Cufflinks	exon	85683076	85683260	.	-	.	gene_id GRMZM2G308689; transcript_id TCONS_00033969; exon_number 4; oId CUFF.28601.1; tss_id TSS30737;

6	Cufflinks	exon	85683397	85683710	.	-	.	gene_id GRMZM2G308689; transcript_id TCONS_00033969; exon_number 5; oId CUFF.28601.1; tss_id TSS30737;
6	Cufflinks	exon	88008788	88009697	.	-	.	gene_id GRMZM2G434935; transcript_id TCONS_00033994; exon_number 1; oId CUFF.28653.1; tss_id TSS30761;
6	Cufflinks	exon	88009832	88010435	.	-	.	gene_id GRMZM2G434935; transcript_id TCONS_00033994; exon_number 2; oId CUFF.28653.1; tss_id TSS30761;
6	Cufflinks	exon	89066375	89066755	.	-	.	gene_id GRMZM2G150256; transcript_id TCONS_00034012; exon_number 1; oId CUFF.28704.2; tss_id TSS30778;
6	Cufflinks	exon	89068336	89068782	.	-	.	gene_id GRMZM2G150256; transcript_id TCONS_00034012; exon_number 2; oId CUFF.28704.2; tss_id TSS30778;
6	Cufflinks	exon	89069847	89069987	.	-	.	gene_id GRMZM2G150256; transcript_id TCONS_00034012; exon_number 3; oId CUFF.28704.2; tss_id TSS30778;
6	Cufflinks	exon	89070081	89071249	.	-	.	gene_id GRMZM2G150256; transcript_id TCONS_00034012; exon_number 4; oId CUFF.28704.2; tss_id TSS30778;
6	Cufflinks	exon	89066375	89066755	.	-	.	gene_id GRMZM2G150256; transcript_id TCONS_00034013; exon_number 1; oId CUFF.28704.1; tss_id TSS30778;
6	Cufflinks	exon	89068336	89068782	.	-	.	gene_id GRMZM2G150256; transcript_id TCONS_00034013; exon_number 2; oId CUFF.28704.1; tss_id TSS30778;
6	Cufflinks	exon	89069847	89069987	.	-	.	gene_id GRMZM2G150256; transcript_id TCONS_00034013; exon_number 3; oId CUFF.28704.1; tss_id TSS30778;
6	Cufflinks	exon	89070081	89070316	.	-	.	gene_id GRMZM2G150256; transcript_id TCONS_00034013; exon_number 4; oId CUFF.28704.1; tss_id TSS30778;
6	Cufflinks	exon	89070611	89071273	.	-	.	gene_id GRMZM2G150256; transcript_id TCONS_00034013; exon_number 5; oId CUFF.28704.1; tss_id TSS30778;
6	Cufflinks	exon	89072232	89072748	.	-	.	gene_id GRMZM2G150276; transcript_id TCONS_00034014; exon_number 1; oId CUFF.28693.1; tss_id TSS30779;
6	Cufflinks	exon	89072900	89073043	.	-	.	gene_id GRMZM2G150276; transcript_id TCONS_00034014; exon_number 2; oId CUFF.28693.1; tss_id TSS30779;
6	Cufflinks	exon	89073173	89073405	.	-	.	gene_id GRMZM2G150276; transcript_id TCONS_00034014; exon_number 3; oId CUFF.28693.1; tss_id TSS30779;
6	Cufflinks	exon	89073544	89074190	.	-	.	gene_id GRMZM2G150276; transcript_id TCONS_00034014; exon_number 4; oId CUFF.28693.1; tss_id TSS30779;
6	Cufflinks	exon	89228410	89228829	.	-	.	gene_id GRMZM2G089400; transcript_id TCONS_00034016; exon_number 1; oId CUFF.28697.1; tss_id TSS30781;
6	Cufflinks	exon	89229073	89229099	.	-	.	gene_id GRMZM2G089400; transcript_id TCONS_00034016; exon_number 2; oId CUFF.28697.1; tss_id TSS30781;
6	Cufflinks	exon	89230197	89230672	.	-	.	gene_id GRMZM2G089400; transcript_id TCONS_00034016; exon_number 3; oId CUFF.28697.1; tss_id TSS30781;
6	Cufflinks	exon	91731581	91733115	.	-	.	gene_id XLOC_029829; transcript_id TCONS_00034050; exon_number 1; oId CUFF.28765.1; tss_id TSS30810;
6	Cufflinks	exon	98420496	98421100	.	-	.	gene_id XLOC_029909; transcript_id TCONS_00034144; exon_number 1; oId CUFF.28921.1; tss_id TSS30891;
6	Cufflinks	exon	98421182	98422255	.	-	.	gene_id XLOC_029909; transcript_id TCONS_00034144; exon_number 2; oId CUFF.28921.1; tss_id TSS30891;
6	Cufflinks	exon	103569642	103570142	.	-	.	gene_id GRMZM2G310144; transcript_id TCONS_00034179; exon_number 1; oId CUFF.28990.1; tss_id TSS30922;
6	Cufflinks	exon	103570236	103570324	.	-	.	gene_id GRMZM2G310144; transcript_id TCONS_00034179; exon_number 2; oId CUFF.28990.1; tss_id TSS30922;
6	Cufflinks	exon	103570420	103570525	.	-	.	gene_id GRMZM2G310144; transcript_id TCONS_00034179; exon_number 3; oId CUFF.28990.1; tss_id TSS30922;
6	Cufflinks	exon	103570708	103570836	.	-	.	gene_id GRMZM2G310144; transcript_id TCONS_00034179; exon_number 4; oId CUFF.28990.1; tss_id TSS30922;
6	Cufflinks	exon	103571263	103571347	.	-	.	gene_id GRMZM2G310144; transcript_id TCONS_00034179; exon_number 5; oId CUFF.28990.1; tss_id TSS30922;
6	Cufflinks	exon	103571449	103571558	.	-	.	gene_id GRMZM2G310144; transcript_id TCONS_00034179; exon_number 6; oId CUFF.28990.1; tss_id TSS30922;
6	Cufflinks	exon	103572457	103572660	.	-	.	gene_id GRMZM2G310144; transcript_id TCONS_00034179; exon_number 7; oId CUFF.28990.1; tss_id TSS30922;
6	Cufflinks	exon	103572923	103573284	.	-	.	gene_id GRMZM2G310144; transcript_id TCONS_00034179; exon_number 8; oId CUFF.28990.1; tss_id TSS30922;
6	Cufflinks	exon	103573618	103573701	.	-	.	gene_id GRMZM2G310144; transcript_id TCONS_00034179; exon_number 9; oId CUFF.28990.1; tss_id TSS30922;
6	Cufflinks	exon	103573835	103573960	.	-	.	gene_id GRMZM2G310144; transcript_id TCONS_00034179; exon_number 10; oId CUFF.28990.1; tss_id TSS30922;
6	Cufflinks	exon	103574042	103574204	.	-	.	gene_id GRMZM2G310144; transcript_id TCONS_00034179; exon_number 11; oId CUFF.28990.1; tss_id TSS30922;
6	Cufflinks	exon	103574331	103574489	.	-	.	gene_id GRMZM2G310144; transcript_id TCONS_00034179; exon_number 12; oId CUFF.28990.1; tss_id TSS30922;
6	Cufflinks	exon	103574847	103574974	.	-	.	gene_id GRMZM2G310144; transcript_id TCONS_00034179; exon_number 13; oId CUFF.28990.1; tss_id TSS30922;
6	Cufflinks	exon	103575069	103575188	.	-	.	gene_id GRMZM2G310144; transcript_id TCONS_00034179; exon_number 14; oId CUFF.28990.1; tss_id TSS30922;

6	Cufflinks	exon	103575865	103576009	.	-	.	gene_id GRMZM2G310144; transcript_id TCONS_00034179; exon_number 15; oId CUFF.28990.1; tss_id TSS30922;
6	Cufflinks	exon	103576101	103576177	.	-	.	gene_id GRMZM2G310144; transcript_id TCONS_00034179; exon_number 16; oId CUFF.28990.1; tss_id TSS30922;
6	Cufflinks	exon	103576368	103576755	.	-	.	gene_id GRMZM2G310144; transcript_id TCONS_00034179; exon_number 17; oId CUFF.28990.1; tss_id TSS30922;
6	Cufflinks	exon	103577545	103577833	.	-	.	gene_id GRMZM2G310144; transcript_id TCONS_00034179; exon_number 18; oId CUFF.28990.1; tss_id TSS30922;
6	Cufflinks	exon	104030544	104031931	.	-	.	gene_id GRMZM2G032028; transcript_id TCONS_00034184; exon_number 1; oId CUFF.29001.1; tss_id TSS30927;
6	Cufflinks	exon	104032996	104033188	.	-	.	gene_id GRMZM2G032028; transcript_id TCONS_00034184; exon_number 2; oId CUFF.29001.1; tss_id TSS30927;
6	Cufflinks	exon	104838658	104840135	.	-	.	gene_id GRMZM2G449817; transcript_id TCONS_00034193; exon_number 1; oId CUFF.29011.1; tss_id TSS30933;
6	Cufflinks	exon	104840236	104842995	.	-	.	gene_id GRMZM2G449817; transcript_id TCONS_00034193; exon_number 2; oId CUFF.29011.1; tss_id TSS30933;
6	Cufflinks	exon	116206088	116206943	.	-	.	gene_id XLOC_030074; transcript_id TCONS_00034335; exon_number 1; oId CUFF.29268.1; tss_id TSS31062;
6	Cufflinks	exon	125424088	125424971	.	-	.	gene_id GRMZM2G357296; transcript_id TCONS_00034448; exon_number 1; oId CUFF.29462.1; tss_id TSS31163;
6	Cufflinks	exon	128638235	128638848	.	-	.	gene_id XLOC_030216; transcript_id TCONS_00034504; exon_number 1; oId CUFF.29532.1; tss_id TSS31208;
6	Cufflinks	exon	132244350	132244827	.	-	.	gene_id GRMZM2G032896; transcript_id TCONS_00034568; exon_number 1; oId CUFF.29637.1; tss_id TSS31270;
6	Cufflinks	exon	132245209	132245537	.	-	.	gene_id GRMZM2G032896; transcript_id TCONS_00034568; exon_number 2; oId CUFF.29637.1; tss_id TSS31270;
6	Cufflinks	exon	132245649	132245738	.	-	.	gene_id GRMZM2G032896; transcript_id TCONS_00034568; exon_number 3; oId CUFF.29637.1; tss_id TSS31270;
6	Cufflinks	exon	132246326	132246595	.	-	.	gene_id GRMZM2G032896; transcript_id TCONS_00034568; exon_number 4; oId CUFF.29637.1; tss_id TSS31270;
6	Cufflinks	exon	132246772	132246921	.	-	.	gene_id GRMZM2G032896; transcript_id TCONS_00034568; exon_number 5; oId CUFF.29637.1; tss_id TSS31270;
6	Cufflinks	exon	132247970	132248169	.	-	.	gene_id GRMZM2G032896; transcript_id TCONS_00034568; exon_number 6; oId CUFF.29637.1; tss_id TSS31270;
6	Cufflinks	exon	132248264	132248327	.	-	.	gene_id GRMZM2G032896; transcript_id TCONS_00034568; exon_number 7; oId CUFF.29637.1; tss_id TSS31270;
6	Cufflinks	exon	132248478	132248959	.	-	.	gene_id GRMZM2G032896; transcript_id TCONS_00034568; exon_number 8; oId CUFF.29637.1; tss_id TSS31270;
6	Cufflinks	exon	138474315	138475901	.	-	.	gene_id GRMZM2G447480; transcript_id TCONS_00034646; exon_number 1; oId CUFF.29784.1; tss_id TSS31336;
6	Cufflinks	exon	138476009	138476165	.	-	.	gene_id GRMZM2G447480; transcript_id TCONS_00034646; exon_number 2; oId CUFF.29784.1; tss_id TSS31336;
6	Cufflinks	exon	139641367	139641722	.	-	.	gene_id GRMZM2G423863; transcript_id TCONS_00034666; exon_number 1; oId CUFF.29804.1; tss_id TSS31354;
6	Cufflinks	exon	139643176	139643511	.	-	.	gene_id GRMZM2G423863; transcript_id TCONS_00034666; exon_number 2; oId CUFF.29804.1; tss_id TSS31354;
6	Cufflinks	exon	139643620	139643749	.	-	.	gene_id GRMZM2G423863; transcript_id TCONS_00034666; exon_number 3; oId CUFF.29804.1; tss_id TSS31354;
6	Cufflinks	exon	142836658	142837321	.	-	.	gene_id GRMZM2G132461; transcript_id TCONS_00034726; exon_number 1; oId CUFF.29909.1; tss_id TSS31410;
6	Cufflinks	exon	142838051	142838379	.	-	.	gene_id GRMZM2G132461; transcript_id TCONS_00034726; exon_number 2; oId CUFF.29909.1; tss_id TSS31410;
6	Cufflinks	exon	142838483	142838785	.	-	.	gene_id GRMZM2G132461; transcript_id TCONS_00034726; exon_number 3; oId CUFF.29909.1; tss_id TSS31410;
6	Cufflinks	exon	142838900	142839312	.	-	.	gene_id GRMZM2G132461; transcript_id TCONS_00034726; exon_number 4; oId CUFF.29909.1; tss_id TSS31410;
6	Cufflinks	exon	142839413	142839545	.	-	.	gene_id GRMZM2G132461; transcript_id TCONS_00034726; exon_number 5; oId CUFF.29909.1; tss_id TSS31410;
6	Cufflinks	exon	144147804	144148882	.	-	.	gene_id GRMZM5G869788; transcript_id TCONS_00034749; exon_number 1; oId CUFF.29945.1; tss_id TSS31430;
6	Cufflinks	exon	144151377	144152000	.	-	.	gene_id GRMZM5G869788; transcript_id TCONS_00034749; exon_number 2; oId CUFF.29945.1; tss_id TSS31430;
6	Cufflinks	exon	144152109	144152234	.	-	.	gene_id GRMZM5G869788; transcript_id TCONS_00034749; exon_number 3; oId CUFF.29945.1; tss_id TSS31430;
6	Cufflinks	exon	144152368	144153012	.	-	.	gene_id GRMZM5G869788; transcript_id TCONS_00034749; exon_number 4; oId CUFF.29945.1; tss_id TSS31430;
6	Cufflinks	exon	146885515	146886750	.	-	.	gene_id GRMZM5G827496; transcript_id TCONS_00034788; exon_number 1; oId CUFF.30014.1; tss_id TSS31468;
6	Cufflinks	exon	146887097	146887913	.	-	.	gene_id GRMZM5G827496; transcript_id TCONS_00034788; exon_number 2; oId CUFF.30014.1; tss_id TSS31468;
6	Cufflinks	exon	146888649	146888876	.	-	.	gene_id GRMZM5G827496; transcript_id TCONS_00034788; exon_number 3; oId CUFF.30014.1; tss_id TSS31468;

6	Cufflinks	exon	155208311	155209003	.	-	.	gene_id GRMZM2G429714; transcript_id TCONS_00034973; exon_number 1; oId CUFF.30337.1; tss_id TSS31628;
6	Cufflinks	exon	155209111	155209343	.	-	.	gene_id GRMZM2G429714; transcript_id TCONS_00034973; exon_number 2; oId CUFF.30337.1; tss_id TSS31628;
6	Cufflinks	exon	155209483	155209702	.	-	.	gene_id GRMZM2G429714; transcript_id TCONS_00034973; exon_number 3; oId CUFF.30337.1; tss_id TSS31628;
6	Cufflinks	exon	155209869	155210008	.	-	.	gene_id GRMZM2G429714; transcript_id TCONS_00034973; exon_number 4; oId CUFF.30337.1; tss_id TSS31628;
6	Cufflinks	exon	155210244	155210408	.	-	.	gene_id GRMZM2G429714; transcript_id TCONS_00034973; exon_number 5; oId CUFF.30337.1; tss_id TSS31628;
6	Cufflinks	exon	155210523	155210998	.	-	.	gene_id GRMZM2G429714; transcript_id TCONS_00034973; exon_number 6; oId CUFF.30337.1; tss_id TSS31628;
6	Cufflinks	exon	155211102	155211236	.	-	.	gene_id GRMZM2G429714; transcript_id TCONS_00034973; exon_number 7; oId CUFF.30337.1; tss_id TSS31628;
6	Cufflinks	exon	155211336	155211407	.	-	.	gene_id GRMZM2G429714; transcript_id TCONS_00034973; exon_number 8; oId CUFF.30337.1; tss_id TSS31628;
6	Cufflinks	exon	155211496	155211570	.	-	.	gene_id GRMZM2G429714; transcript_id TCONS_00034973; exon_number 9; oId CUFF.30337.1; tss_id TSS31628;
6	Cufflinks	exon	155211674	155211742	.	-	.	gene_id GRMZM2G429714; transcript_id TCONS_00034973; exon_number 10; oId CUFF.30337.1; tss_id TSS31628;
6	Cufflinks	exon	155211830	155211901	.	-	.	gene_id GRMZM2G429714; transcript_id TCONS_00034973; exon_number 11; oId CUFF.30337.1; tss_id TSS31628;
6	Cufflinks	exon	155212012	155212083	.	-	.	gene_id GRMZM2G429714; transcript_id TCONS_00034973; exon_number 12; oId CUFF.30337.1; tss_id TSS31628;
6	Cufflinks	exon	155212184	155212255	.	-	.	gene_id GRMZM2G429714; transcript_id TCONS_00034973; exon_number 13; oId CUFF.30337.1; tss_id TSS31628;
6	Cufflinks	exon	155212362	155212523	.	-	.	gene_id GRMZM2G429714; transcript_id TCONS_00034973; exon_number 14; oId CUFF.30337.1; tss_id TSS31628;
6	Cufflinks	exon	155212674	155212745	.	-	.	gene_id GRMZM2G429714; transcript_id TCONS_00034973; exon_number 15; oId CUFF.30337.1; tss_id TSS31628;
6	Cufflinks	exon	155212841	155212915	.	-	.	gene_id GRMZM2G429714; transcript_id TCONS_00034973; exon_number 16; oId CUFF.30337.1; tss_id TSS31628;
6	Cufflinks	exon	155213245	155213316	.	-	.	gene_id GRMZM2G429714; transcript_id TCONS_00034973; exon_number 17; oId CUFF.30337.1; tss_id TSS31628;
6	Cufflinks	exon	155213459	155213582	.	-	.	gene_id GRMZM2G429714; transcript_id TCONS_00034973; exon_number 18; oId CUFF.30337.1; tss_id TSS31628;
6	Cufflinks	exon	155213807	155214291	.	-	.	gene_id GRMZM2G429714; transcript_id TCONS_00034973; exon_number 19; oId CUFF.30337.1; tss_id TSS31628;
6	Cufflinks	exon	155783970	155785350	.	-	.	gene_id GRMZM2G116258; transcript_id TCONS_00034991; exon_number 1; oId CUFF.30354.1; tss_id TSS31644;
6	Cufflinks	exon	155786166	155786339	.	-	.	gene_id GRMZM2G116258; transcript_id TCONS_00034991; exon_number 2; oId CUFF.30354.1; tss_id TSS31644;
6	Cufflinks	exon	155788026	155788502	.	-	.	gene_id GRMZM2G116258; transcript_id TCONS_00034991; exon_number 3; oId CUFF.30354.1; tss_id TSS31644;
6	Cufflinks	exon	155861586	155861802	.	-	.	gene_id GRMZM2G440968; transcript_id TCONS_00034996; exon_number 1; oId CUFF.30375.1; tss_id TSS31648;
6	Cufflinks	exon	155861966	155862167	.	-	.	gene_id GRMZM2G440968; transcript_id TCONS_00034996; exon_number 2; oId CUFF.30375.1; tss_id TSS31648;
6	Cufflinks	exon	155862244	155862536	.	-	.	gene_id GRMZM2G440968; transcript_id TCONS_00034996; exon_number 3; oId CUFF.30375.1; tss_id TSS31648;
6	Cufflinks	exon	155951341	155951701	.	-	.	gene_id GRMZM2G090300; transcript_id TCONS_00034997; exon_number 1; oId CUFF.30360.1; tss_id TSS31649;
6	Cufflinks	exon	155952063	155953332	.	-	.	gene_id GRMZM2G090300; transcript_id TCONS_00034997; exon_number 2; oId CUFF.30360.1; tss_id TSS31649;
6	Cufflinks	exon	160229718	160230328	.	-	.	gene_id GRMZM2G004932; transcript_id TCONS_00035097; exon_number 1; oId CUFF.30544.1; tss_id TSS31737;
6	Cufflinks	exon	160230547	160230698	.	-	.	gene_id GRMZM2G004932; transcript_id TCONS_00035097; exon_number 2; oId CUFF.30544.1; tss_id TSS31737;
6	Cufflinks	exon	160230789	160230834	.	-	.	gene_id GRMZM2G004932; transcript_id TCONS_00035097; exon_number 3; oId CUFF.30544.1; tss_id TSS31737;
6	Cufflinks	exon	160231006	160231230	.	-	.	gene_id GRMZM2G004932; transcript_id TCONS_00035097; exon_number 4; oId CUFF.30544.1; tss_id TSS31737;
6	Cufflinks	exon	160231361	160231401	.	-	.	gene_id GRMZM2G004932; transcript_id TCONS_00035097; exon_number 5; oId CUFF.30544.1; tss_id TSS31737;
6	Cufflinks	exon	160231622	160231751	.	-	.	gene_id GRMZM2G004932; transcript_id TCONS_00035097; exon_number 6; oId CUFF.30544.1; tss_id TSS31737;
6	Cufflinks	exon	160231858	160231926	.	-	.	gene_id GRMZM2G004932; transcript_id TCONS_00035097; exon_number 7; oId CUFF.30544.1; tss_id TSS31737;
6	Cufflinks	exon	160232169	160232222	.	-	.	gene_id GRMZM2G004932; transcript_id TCONS_00035097; exon_number 8; oId CUFF.30544.1; tss_id TSS31737;
6	Cufflinks	exon	160232306	160232464	.	-	.	gene_id GRMZM2G004932; transcript_id TCONS_00035097; exon_number 9; oId CUFF.30544.1; tss_id TSS31737;

6	Cufflinks	exon	160233335	160233472	.	-	.	gene_id GRMZM2G004932; transcript_id TCONS_00035097; exon_number 10; oId CUFF.30544.1; tss_id TSS31737;
6	Cufflinks	exon	160233563	160233625	.	-	.	gene_id GRMZM2G004932; transcript_id TCONS_00035097; exon_number 11; oId CUFF.30544.1; tss_id TSS31737;
6	Cufflinks	exon	160233737	160233776	.	-	.	gene_id GRMZM2G004932; transcript_id TCONS_00035097; exon_number 12; oId CUFF.30544.1; tss_id TSS31737;
6	Cufflinks	exon	160233893	160233939	.	-	.	gene_id GRMZM2G004932; transcript_id TCONS_00035097; exon_number 13; oId CUFF.30544.1; tss_id TSS31737;
6	Cufflinks	exon	160234822	160235410	.	-	.	gene_id GRMZM2G004932; transcript_id TCONS_00035097; exon_number 14; oId CUFF.30544.1; tss_id TSS31737;
6	Cufflinks	exon	160433610	160434347	.	-	.	gene_id GRMZM2G432801; transcript_id TCONS_00035101; exon_number 1; oId CUFF.30552.1; tss_id TSS31741;
6	Cufflinks	exon	160434423	160434504	.	-	.	gene_id GRMZM2G432801; transcript_id TCONS_00035101; exon_number 2; oId CUFF.30552.1; tss_id TSS31741;
6	Cufflinks	exon	160435392	160435522	.	-	.	gene_id GRMZM2G432801; transcript_id TCONS_00035101; exon_number 3; oId CUFF.30552.1; tss_id TSS31741;
6	Cufflinks	exon	160435617	160435715	.	-	.	gene_id GRMZM2G432801; transcript_id TCONS_00035101; exon_number 4; oId CUFF.30552.1; tss_id TSS31741;
6	Cufflinks	exon	160435868	160436023	.	-	.	gene_id GRMZM2G432801; transcript_id TCONS_00035101; exon_number 5; oId CUFF.30552.1; tss_id TSS31741;
6	Cufflinks	exon	160436210	160436284	.	-	.	gene_id GRMZM2G432801; transcript_id TCONS_00035101; exon_number 6; oId CUFF.30552.1; tss_id TSS31741;
6	Cufflinks	exon	160436938	160437120	.	-	.	gene_id GRMZM2G432801; transcript_id TCONS_00035101; exon_number 7; oId CUFF.30552.1; tss_id TSS31741;
6	Cufflinks	exon	160437207	160437357	.	-	.	gene_id GRMZM2G432801; transcript_id TCONS_00035101; exon_number 8; oId CUFF.30552.1; tss_id TSS31741;
6	Cufflinks	exon	160439501	160439979	.	-	.	gene_id GRMZM2G432801; transcript_id TCONS_00035101; exon_number 9; oId CUFF.30552.1; tss_id TSS31741;
6	Cufflinks	exon	161413828	161414351	.	-	.	gene_id GRMZM2G158972; transcript_id TCONS_00035122; exon_number 1; oId CUFF.30620.2; tss_id TSS31762;
6	Cufflinks	exon	161414430	161414572	.	-	.	gene_id GRMZM2G158972; transcript_id TCONS_00035122; exon_number 2; oId CUFF.30620.2; tss_id TSS31762;
6	Cufflinks	exon	161414642	161414750	.	-	.	gene_id GRMZM2G158972; transcript_id TCONS_00035122; exon_number 3; oId CUFF.30620.2; tss_id TSS31762;
6	Cufflinks	exon	161414862	161415484	.	-	.	gene_id GRMZM2G158972; transcript_id TCONS_00035122; exon_number 4; oId CUFF.30620.2; tss_id TSS31762;
6	Cufflinks	exon	161413828	161414351	.	-	.	gene_id GRMZM2G158972; transcript_id TCONS_00035123; exon_number 1; oId CUFF.30620.1; tss_id TSS31763;
6	Cufflinks	exon	161414430	161414572	.	-	.	gene_id GRMZM2G158972; transcript_id TCONS_00035123; exon_number 2; oId CUFF.30620.1; tss_id TSS31763;
6	Cufflinks	exon	161414642	161414750	.	-	.	gene_id GRMZM2G158972; transcript_id TCONS_00035123; exon_number 3; oId CUFF.30620.1; tss_id TSS31763;
6	Cufflinks	exon	161414862	161415046	.	-	.	gene_id GRMZM2G158972; transcript_id TCONS_00035123; exon_number 4; oId CUFF.30620.1; tss_id TSS31763;
6	Cufflinks	exon	161415558	161416062	.	-	.	gene_id GRMZM2G158972; transcript_id TCONS_00035123; exon_number 5; oId CUFF.30620.1; tss_id TSS31763;
6	Cufflinks	exon	161417648	161417752	.	-	.	gene_id GRMZM2G158972; transcript_id TCONS_00035123; exon_number 6; oId CUFF.30620.1; tss_id TSS31763;
6	Cufflinks	exon	161417986	161418073	.	-	.	gene_id GRMZM2G158972; transcript_id TCONS_00035123; exon_number 7; oId CUFF.30620.1; tss_id TSS31763;
6	Cufflinks	exon	161418737	161418808	.	-	.	gene_id GRMZM2G158972; transcript_id TCONS_00035123; exon_number 8; oId CUFF.30620.1; tss_id TSS31763;
6	Cufflinks	exon	161419765	161419945	.	-	.	gene_id GRMZM2G158972; transcript_id TCONS_00035123; exon_number 9; oId CUFF.30620.1; tss_id TSS31763;
6	Cufflinks	exon	161420058	161420521	.	-	.	gene_id GRMZM2G158972; transcript_id TCONS_00035123; exon_number 10; oId CUFF.30620.1; tss_id TSS31763;
6	Cufflinks	exon	161647137	161648173	.	-	.	gene_id GRMZM2G090245; transcript_id TCONS_00035130; exon_number 1; oId CUFF.30603.1; tss_id TSS31770;
6	Cufflinks	exon	163081613	163083105	.	-	.	gene_id XLOC_030801; transcript_id TCONS_00035175; exon_number 1; oId CUFF.30691.1; tss_id TSS31808;
6	Cufflinks	exon	164760362	164761040	.	-	.	gene_id GRMZM2G021549; transcript_id TCONS_00035222; exon_number 1; oId CUFF.30784.1; tss_id TSS31852;
6	Cufflinks	exon	164761190	164762305	.	-	.	gene_id GRMZM2G021549; transcript_id TCONS_00035222; exon_number 2; oId CUFF.30784.1; tss_id TSS31852;
6	Cufflinks	exon	164765627	164765731	.	-	.	gene_id GRMZM2G021549; transcript_id TCONS_00035222; exon_number 3; oId CUFF.30784.1; tss_id TSS31852;
6	Cufflinks	exon	164766090	164766199	.	-	.	gene_id GRMZM2G021549; transcript_id TCONS_00035222; exon_number 4; oId CUFF.30784.1; tss_id TSS31852;
6	Cufflinks	exon	164766687	164767127	.	-	.	gene_id GRMZM2G021549; transcript_id TCONS_00035222; exon_number 5; oId CUFF.30784.1; tss_id TSS31852;
6	Cufflinks	exon	168642899	168643689	.	-	.	gene_id GRMZM2G141818; transcript_id TCONS_00035317; exon_number 1; oId CUFF.30950.1; tss_id TSS31938;

6	Cufflinks	exon	168643775	168643806	.	-	.	gene_id GRMZM2G141818; transcript_id TCONS_00035317; exon_number 2; oId CUFF.30950.1; tss_id TSS31938;
6	Cufflinks	exon	168643889	168643983	.	-	.	gene_id GRMZM2G141818; transcript_id TCONS_00035317; exon_number 3; oId CUFF.30950.1; tss_id TSS31938;
6	Cufflinks	exon	168644064	168644137	.	-	.	gene_id GRMZM2G141818; transcript_id TCONS_00035317; exon_number 4; oId CUFF.30950.1; tss_id TSS31938;
6	Cufflinks	exon	168644219	168644330	.	-	.	gene_id GRMZM2G141818; transcript_id TCONS_00035317; exon_number 5; oId CUFF.30950.1; tss_id TSS31938;
6	Cufflinks	exon	168644421	168644484	.	-	.	gene_id GRMZM2G141818; transcript_id TCONS_00035317; exon_number 6; oId CUFF.30950.1; tss_id TSS31938;
6	Cufflinks	exon	168644657	168644722	.	-	.	gene_id GRMZM2G141818; transcript_id TCONS_00035317; exon_number 7; oId CUFF.30950.1; tss_id TSS31938;
6	Cufflinks	exon	168644810	168644937	.	-	.	gene_id GRMZM2G141818; transcript_id TCONS_00035317; exon_number 8; oId CUFF.30950.1; tss_id TSS31938;
6	Cufflinks	exon	168645062	168645196	.	-	.	gene_id GRMZM2G141818; transcript_id TCONS_00035317; exon_number 9; oId CUFF.30950.1; tss_id TSS31938;
6	Cufflinks	exon	168645284	168645396	.	-	.	gene_id GRMZM2G141818; transcript_id TCONS_00035317; exon_number 10; oId CUFF.30950.1; tss_id TSS31938;
6	Cufflinks	exon	168645474	168645639	.	-	.	gene_id GRMZM2G141818; transcript_id TCONS_00035317; exon_number 11; oId CUFF.30950.1; tss_id TSS31938;
6	Cufflinks	exon	168645708	168645821	.	-	.	gene_id GRMZM2G141818; transcript_id TCONS_00035317; exon_number 12; oId CUFF.30950.1; tss_id TSS31938;
6	Cufflinks	exon	168645918	168645980	.	-	.	gene_id GRMZM2G141818; transcript_id TCONS_00035317; exon_number 13; oId CUFF.30950.1; tss_id TSS31938;
6	Cufflinks	exon	168646255	168646362	.	-	.	gene_id GRMZM2G141818; transcript_id TCONS_00035317; exon_number 14; oId CUFF.30950.1; tss_id TSS31938;
6	Cufflinks	exon	168646468	168646584	.	-	.	gene_id GRMZM2G141818; transcript_id TCONS_00035317; exon_number 15; oId CUFF.30950.1; tss_id TSS31938;
6	Cufflinks	exon	168646821	168646983	.	-	.	gene_id GRMZM2G141818; transcript_id TCONS_00035317; exon_number 16; oId CUFF.30950.1; tss_id TSS31938;
6	Cufflinks	exon	168647196	168647293	.	-	.	gene_id GRMZM2G141818; transcript_id TCONS_00035317; exon_number 17; oId CUFF.30950.1; tss_id TSS31938;
6	Cufflinks	exon	168647402	168647499	.	-	.	gene_id GRMZM2G141818; transcript_id TCONS_00035317; exon_number 18; oId CUFF.30950.1; tss_id TSS31938;
6	Cufflinks	exon	168647608	168647744	.	-	.	gene_id GRMZM2G141818; transcript_id TCONS_00035317; exon_number 19; oId CUFF.30950.1; tss_id TSS31938;
6	Cufflinks	exon	168647835	168648059	.	-	.	gene_id GRMZM2G141818; transcript_id TCONS_00035317; exon_number 20; oId CUFF.30950.1; tss_id TSS31938;
6	Cufflinks	exon	168648145	168648350	.	-	.	gene_id GRMZM2G141818; transcript_id TCONS_00035317; exon_number 21; oId CUFF.30950.1; tss_id TSS31938;
6	Cufflinks	exon	168648460	168648725	.	-	.	gene_id GRMZM2G141818; transcript_id TCONS_00035317; exon_number 22; oId CUFF.30950.1; tss_id TSS31938;
6	Cufflinks	exon	168650295	168650480	.	-	.	gene_id GRMZM2G141818; transcript_id TCONS_00035317; exon_number 23; oId CUFF.30950.1; tss_id TSS31938;
7	Cufflinks	exon	272954	273522	.	+	.	gene_id GRMZM2G424147; transcript_id TCONS_00035324; exon_number 1; oId CUFF.30977.1; tss_id TSS31944;
7	Cufflinks	exon	955586	955860	.	+	.	gene_id GRMZM2G020281; transcript_id TCONS_00035326; exon_number 1; oId CUFF.30999.1; tss_id TSS31946;
7	Cufflinks	exon	956140	956230	.	+	.	gene_id GRMZM2G020281; transcript_id TCONS_00035326; exon_number 2; oId CUFF.30999.1; tss_id TSS31946;
7	Cufflinks	exon	956346	956398	.	+	.	gene_id GRMZM2G020281; transcript_id TCONS_00035326; exon_number 3; oId CUFF.30999.1; tss_id TSS31946;
7	Cufflinks	exon	956504	956596	.	+	.	gene_id GRMZM2G020281; transcript_id TCONS_00035326; exon_number 4; oId CUFF.30999.1; tss_id TSS31946;
7	Cufflinks	exon	956734	956805	.	+	.	gene_id GRMZM2G020281; transcript_id TCONS_00035326; exon_number 5; oId CUFF.30999.1; tss_id TSS31946;
7	Cufflinks	exon	956974	957092	.	+	.	gene_id GRMZM2G020281; transcript_id TCONS_00035326; exon_number 6; oId CUFF.30999.1; tss_id TSS31946;
7	Cufflinks	exon	957184	957247	.	+	.	gene_id GRMZM2G020281; transcript_id TCONS_00035326; exon_number 7; oId CUFF.30999.1; tss_id TSS31946;
7	Cufflinks	exon	960199	960252	.	+	.	gene_id GRMZM2G020281; transcript_id TCONS_00035326; exon_number 8; oId CUFF.30999.1; tss_id TSS31946;
7	Cufflinks	exon	960520	960597	.	+	.	gene_id GRMZM2G020281; transcript_id TCONS_00035326; exon_number 9; oId CUFF.30999.1; tss_id TSS31946;
7	Cufflinks	exon	960690	961591	.	+	.	gene_id GRMZM2G020281; transcript_id TCONS_00035326; exon_number 10; oId CUFF.30999.1; tss_id TSS31946;
7	Cufflinks	exon	955595	955860	.	+	.	gene_id GRMZM2G020281; transcript_id TCONS_00035327; exon_number 1; oId CUFF.30999.2; tss_id TSS31946;
7	Cufflinks	exon	960199	960252	.	+	.	gene_id GRMZM2G020281; transcript_id TCONS_00035327; exon_number 2; oId CUFF.30999.2; tss_id TSS31946;
7	Cufflinks	exon	960520	960597	.	+	.	gene_id GRMZM2G020281; transcript_id TCONS_00035327; exon_number 3; oId CUFF.30999.2; tss_id TSS31946;

7	Cufflinks	exon	960690	961591	.	+	.	gene_id GRMZM2G020281; transcript_id TCONS_00035327; exon_number 4; oId CUFF.30999.2; tss_id TSS31946;
7	Cufflinks	exon	955599	955860	.	+	.	gene_id GRMZM2G020281; transcript_id TCONS_00035328; exon_number 1; oId CUFF.30999.3; tss_id TSS31946;
7	Cufflinks	exon	956734	956805	.	+	.	gene_id GRMZM2G020281; transcript_id TCONS_00035328; exon_number 2; oId CUFF.30999.3; tss_id TSS31946;
7	Cufflinks	exon	956974	957092	.	+	.	gene_id GRMZM2G020281; transcript_id TCONS_00035328; exon_number 3; oId CUFF.30999.3; tss_id TSS31946;
7	Cufflinks	exon	957184	957247	.	+	.	gene_id GRMZM2G020281; transcript_id TCONS_00035328; exon_number 4; oId CUFF.30999.3; tss_id TSS31946;
7	Cufflinks	exon	960199	960252	.	+	.	gene_id GRMZM2G020281; transcript_id TCONS_00035328; exon_number 5; oId CUFF.30999.3; tss_id TSS31946;
7	Cufflinks	exon	960520	960597	.	+	.	gene_id GRMZM2G020281; transcript_id TCONS_00035328; exon_number 6; oId CUFF.30999.3; tss_id TSS31946;
7	Cufflinks	exon	960690	961591	.	+	.	gene_id GRMZM2G020281; transcript_id TCONS_00035328; exon_number 7; oId CUFF.30999.3; tss_id TSS31946;
7	Cufflinks	exon	6073356	6075719	.	+	.	gene_id AC205320.3_FG004; transcript_id TCONS_00035422; exon_number 1; oId CUFF.31141.1; tss_id TSS32035;
7	Cufflinks	exon	19063721	19064681	.	+	.	gene_id GRMZM2G340656; transcript_id TCONS_00035577; exon_number 1; oId CUFF.31408.2; tss_id TSS32180;
7	Cufflinks	exon	19064775	19065006	.	+	.	gene_id GRMZM2G340656; transcript_id TCONS_00035577; exon_number 2; oId CUFF.31408.2; tss_id TSS32180;
7	Cufflinks	exon	19065096	19065206	.	+	.	gene_id GRMZM2G340656; transcript_id TCONS_00035577; exon_number 3; oId CUFF.31408.2; tss_id TSS32180;
7	Cufflinks	exon	19065308	19065998	.	+	.	gene_id GRMZM2G340656; transcript_id TCONS_00035577; exon_number 4; oId CUFF.31408.2; tss_id TSS32180;
7	Cufflinks	exon	19066096	19066298	.	+	.	gene_id GRMZM2G340656; transcript_id TCONS_00035577; exon_number 5; oId CUFF.31408.2; tss_id TSS32180;
7	Cufflinks	exon	19066424	19067235	.	+	.	gene_id GRMZM2G340656; transcript_id TCONS_00035577; exon_number 6; oId CUFF.31408.2; tss_id TSS32180;
7	Cufflinks	exon	19063721	19064212	.	+	.	gene_id GRMZM2G340656; transcript_id TCONS_00035576; exon_number 1; oId CUFF.31408.1; tss_id TSS32180;
7	Cufflinks	exon	19064443	19064681	.	+	.	gene_id GRMZM2G340656; transcript_id TCONS_00035576; exon_number 2; oId CUFF.31408.1; tss_id TSS32180;
7	Cufflinks	exon	19064775	19065006	.	+	.	gene_id GRMZM2G340656; transcript_id TCONS_00035576; exon_number 3; oId CUFF.31408.1; tss_id TSS32180;
7	Cufflinks	exon	19065096	19065206	.	+	.	gene_id GRMZM2G340656; transcript_id TCONS_00035576; exon_number 4; oId CUFF.31408.1; tss_id TSS32180;
7	Cufflinks	exon	19065308	19065998	.	+	.	gene_id GRMZM2G340656; transcript_id TCONS_00035576; exon_number 5; oId CUFF.31408.1; tss_id TSS32180;
7	Cufflinks	exon	19066096	19066298	.	+	.	gene_id GRMZM2G340656; transcript_id TCONS_00035576; exon_number 6; oId CUFF.31408.1; tss_id TSS32180;
7	Cufflinks	exon	19066424	19067235	.	+	.	gene_id GRMZM2G340656; transcript_id TCONS_00035576; exon_number 7; oId CUFF.31408.1; tss_id TSS32180;
7	Cufflinks	exon	19641007	19641568	.	+	.	gene_id XLOC_031169; transcript_id TCONS_00035582; exon_number 1; oId CUFF.31425.1; tss_id TSS32185;
7	Cufflinks	exon	25552651	25552834	.	+	.	gene_id XLOC_031212; transcript_id TCONS_00035628; exon_number 1; oId CUFF.31510.1; tss_id TSS32228;
7	Cufflinks	exon	25553093	25553257	.	+	.	gene_id XLOC_031212; transcript_id TCONS_00035628; exon_number 2; oId CUFF.31510.1; tss_id TSS32228;
7	Cufflinks	exon	25819517	25820504	.	+	.	gene_id GRMZM2G303829; transcript_id TCONS_00035630; exon_number 1; oId CUFF.31512.1; tss_id TSS32230;
7	Cufflinks	exon	31098634	31099387	.	+	.	gene_id GRMZM2G000221; transcript_id TCONS_00035665; exon_number 1; oId CUFF.31571.1; tss_id TSS32262;
7	Cufflinks	exon	31781244	31782056	.	+	.	gene_id GRMZM2G104231; transcript_id TCONS_00035672; exon_number 1; oId CUFF.31599.1; tss_id TSS32269;
7	Cufflinks	exon	31782575	31783659	.	+	.	gene_id GRMZM2G104231; transcript_id TCONS_00035672; exon_number 2; oId CUFF.31599.1; tss_id TSS32269;
7	Cufflinks	exon	31781247	31781327	.	+	.	gene_id GRMZM2G104231; transcript_id TCONS_00035674; exon_number 1; oId CUFF.31599.3; tss_id TSS32269;
7	Cufflinks	exon	31781600	31783659	.	+	.	gene_id GRMZM2G104231; transcript_id TCONS_00035674; exon_number 2; oId CUFF.31599.3; tss_id TSS32269;
7	Cufflinks	exon	51667897	51668499	.	+	.	gene_id XLOC_031361; transcript_id TCONS_00035793; exon_number 1; oId CUFF.31778.1; tss_id TSS32379;
7	Cufflinks	exon	52269164	52270135	.	+	.	gene_id GRMZM2G027198; transcript_id TCONS_00035799; exon_number 1; oId CUFF.31793.1; tss_id TSS32383;
7	Cufflinks	exon	54345908	54347701	.	+	.	gene_id GRMZM2G318299; transcript_id TCONS_00035805; exon_number 1; oId CUFF.31809.1; tss_id TSS32389;
7	Cufflinks	exon	85288907	85289449	.	+	.	gene_id GRMZM2G117878; transcript_id TCONS_00035912; exon_number 1; oId CUFF.32000.1; tss_id TSS32485;
7	Cufflinks	exon	85289551	85290654	.	+	.	gene_id GRMZM2G117878; transcript_id TCONS_00035912; exon_number 2; oId CUFF.32000.1; tss_id TSS32485;

7	Cufflinks	exon	99233022	99234662	.	+	.	gene_id GRMZM2G162396; transcript_id TCONS_00035998; exon_number 1; oId CUFF.32155.1; tss_id TSS32564;
7	Cufflinks	exon	107954326	107957193	.	+	.	gene_id GRMZM2G019183; transcript_id TCONS_00036047; exon_number 1; oId CUFF.32269.1; tss_id TSS32611;
7	Cufflinks	exon	107957292	107957565	.	+	.	gene_id GRMZM2G019183; transcript_id TCONS_00036047; exon_number 2; oId CUFF.32269.1; tss_id TSS32611;
7	Cufflinks	exon	107957693	107958837	.	+	.	gene_id GRMZM2G019183; transcript_id TCONS_00036047; exon_number 3; oId CUFF.32269.1; tss_id TSS32611;
7	Cufflinks	exon	110084378	110084765	.	+	.	gene_id GRMZM2G121189; transcript_id TCONS_00036068; exon_number 1; oId CUFF.32317.1; tss_id TSS32632;
7	Cufflinks	exon	110084879	110084963	.	+	.	gene_id GRMZM2G121189; transcript_id TCONS_00036068; exon_number 2; oId CUFF.32317.1; tss_id TSS32632;
7	Cufflinks	exon	110085061	110085727	.	+	.	gene_id GRMZM2G121189; transcript_id TCONS_00036068; exon_number 3; oId CUFF.32317.1; tss_id TSS32632;
7	Cufflinks	exon	117727876	117728391	.	+	.	gene_id GRMZM2G142918; transcript_id TCONS_00036116; exon_number 1; oId CUFF.32389.1; tss_id TSS32677;
7	Cufflinks	exon	117729051	117730526	.	+	.	gene_id GRMZM2G142918; transcript_id TCONS_00036116; exon_number 2; oId CUFF.32389.1; tss_id TSS32677;
7	Cufflinks	exon	121285089	121286003	.	+	.	gene_id GRMZM2G135763; transcript_id TCONS_00036147; exon_number 1; oId CUFF.32458.1; tss_id TSS32706;
7	Cufflinks	exon	121287001	121287121	.	+	.	gene_id GRMZM2G135763; transcript_id TCONS_00036147; exon_number 2; oId CUFF.32458.1; tss_id TSS32706;
7	Cufflinks	exon	121287475	121287723	.	+	.	gene_id GRMZM2G135763; transcript_id TCONS_00036147; exon_number 3; oId CUFF.32458.1; tss_id TSS32706;
7	Cufflinks	exon	121287948	121288101	.	+	.	gene_id GRMZM2G135763; transcript_id TCONS_00036147; exon_number 4; oId CUFF.32458.1; tss_id TSS32706;
7	Cufflinks	exon	121288479	121289636	.	+	.	gene_id GRMZM2G135763; transcript_id TCONS_00036147; exon_number 5; oId CUFF.32458.1; tss_id TSS32706;
7	Cufflinks	exon	121850282	121851010	.	+	.	gene_id GRMZM2G004207; transcript_id TCONS_00036160; exon_number 1; oId CUFF.32469.1; tss_id TSS32718;
7	Cufflinks	exon	121851109	121851244	.	+	.	gene_id GRMZM2G004207; transcript_id TCONS_00036160; exon_number 2; oId CUFF.32469.1; tss_id TSS32718;
7	Cufflinks	exon	121851518	121851654	.	+	.	gene_id GRMZM2G004207; transcript_id TCONS_00036160; exon_number 3; oId CUFF.32469.1; tss_id TSS32718;
7	Cufflinks	exon	121852259	121852444	.	+	.	gene_id GRMZM2G004207; transcript_id TCONS_00036160; exon_number 4; oId CUFF.32469.1; tss_id TSS32718;
7	Cufflinks	exon	128406301	128406767	.	+	.	gene_id GRMZM2G416632; transcript_id TCONS_00036234; exon_number 1; oId CUFF.32582.1; tss_id TSS32787;
7	Cufflinks	exon	128406868	128408544	.	+	.	gene_id GRMZM2G416632; transcript_id TCONS_00036234; exon_number 2; oId CUFF.32582.1; tss_id TSS32787;
7	Cufflinks	exon	128679213	128681250	.	+	.	gene_id XLOC_031757; transcript_id TCONS_00036237; exon_number 1; oId CUFF.32576.1; tss_id TSS32790;
7	Cufflinks	exon	132922501	132923371	.	+	.	gene_id GRMZM2G085467; transcript_id TCONS_00036291; exon_number 1; oId CUFF.32690.1; tss_id TSS32838;
7	Cufflinks	exon	132923537	132924039	.	+	.	gene_id GRMZM2G085467; transcript_id TCONS_00036291; exon_number 2; oId CUFF.32690.1; tss_id TSS32838;
7	Cufflinks	exon	132924177	132924242	.	+	.	gene_id GRMZM2G085467; transcript_id TCONS_00036291; exon_number 3; oId CUFF.32690.1; tss_id TSS32838;
7	Cufflinks	exon	132924378	132924446	.	+	.	gene_id GRMZM2G085467; transcript_id TCONS_00036291; exon_number 4; oId CUFF.32690.1; tss_id TSS32838;
7	Cufflinks	exon	132924810	132925523	.	+	.	gene_id GRMZM2G085467; transcript_id TCONS_00036291; exon_number 5; oId CUFF.32690.1; tss_id TSS32838;
7	Cufflinks	exon	134168481	134169179	.	+	.	gene_id GRMZM2G168364; transcript_id TCONS_00036317; exon_number 1; oId CUFF.32730.1; tss_id TSS32858;
7	Cufflinks	exon	134169261	134169417	.	+	.	gene_id GRMZM2G168364; transcript_id TCONS_00036317; exon_number 2; oId CUFF.32730.1; tss_id TSS32858;
7	Cufflinks	exon	134169512	134170279	.	+	.	gene_id GRMZM2G168364; transcript_id TCONS_00036317; exon_number 3; oId CUFF.32730.1; tss_id TSS32858;
7	Cufflinks	exon	139144164	139144566	.	+	.	gene_id GRMZM2G081048; transcript_id TCONS_00036372; exon_number 1; oId CUFF.32845.1; tss_id TSS32911;
7	Cufflinks	exon	139144693	139144845	.	+	.	gene_id GRMZM2G081048; transcript_id TCONS_00036372; exon_number 2; oId CUFF.32845.1; tss_id TSS32911;
7	Cufflinks	exon	139145233	139145447	.	+	.	gene_id GRMZM2G081048; transcript_id TCONS_00036372; exon_number 3; oId CUFF.32845.1; tss_id TSS32911;
7	Cufflinks	exon	139145540	139145610	.	+	.	gene_id GRMZM2G081048; transcript_id TCONS_00036372; exon_number 4; oId CUFF.32845.1; tss_id TSS32911;
7	Cufflinks	exon	139145781	139145940	.	+	.	gene_id GRMZM2G081048; transcript_id TCONS_00036372; exon_number 5; oId CUFF.32845.1; tss_id TSS32911;
7	Cufflinks	exon	139146664	139146856	.	+	.	gene_id GRMZM2G081048; transcript_id TCONS_00036372; exon_number 6; oId CUFF.32845.1; tss_id TSS32911;
7	Cufflinks	exon	139146983	139147138	.	+	.	gene_id GRMZM2G081048; transcript_id TCONS_00036372; exon_number 7; oId CUFF.32845.1; tss_id TSS32911;

7	Cufflinks	exon	139147252	139147340	.	+	.	gene_id GRMZM2G081048; transcript_id TCONS_00036372; exon_number 8; oId CUFF.32845.1; tss_id TSS32911;
7	Cufflinks	exon	139152252	139152345	.	+	.	gene_id GRMZM2G081048; transcript_id TCONS_00036372; exon_number 9; oId CUFF.32845.1; tss_id TSS32911;
7	Cufflinks	exon	139154262	139155145	.	+	.	gene_id GRMZM2G081048; transcript_id TCONS_00036372; exon_number 10; oId CUFF.32845.1; tss_id TSS32911;
7	Cufflinks	exon	140268784	140269784	.	+	.	gene_id XLOC_031883; transcript_id TCONS_00036386; exon_number 1; oId CUFF.32844.1; tss_id TSS32922;
7	Cufflinks	exon	141630989	141632442	.	+	.	gene_id GRMZM2G001639; transcript_id TCONS_00036410; exon_number 1; oId CUFF.32891.2; tss_id TSS32944;
7	Cufflinks	exon	141632771	141633508	.	+	.	gene_id GRMZM2G001639; transcript_id TCONS_00036410; exon_number 2; oId CUFF.32891.2; tss_id TSS32944;
7	Cufflinks	exon	141917927	141918223	.	+	.	gene_id GRMZM2G052610; transcript_id TCONS_00036417; exon_number 1; oId CUFF.32919.1; tss_id TSS32951;
7	Cufflinks	exon	141918309	141918355	.	+	.	gene_id GRMZM2G052610; transcript_id TCONS_00036417; exon_number 2; oId CUFF.32919.1; tss_id TSS32951;
7	Cufflinks	exon	141918643	141918735	.	+	.	gene_id GRMZM2G052610; transcript_id TCONS_00036417; exon_number 3; oId CUFF.32919.1; tss_id TSS32951;
7	Cufflinks	exon	141919119	141919257	.	+	.	gene_id GRMZM2G052610; transcript_id TCONS_00036417; exon_number 4; oId CUFF.32919.1; tss_id TSS32951;
7	Cufflinks	exon	141919352	141919454	.	+	.	gene_id GRMZM2G052610; transcript_id TCONS_00036417; exon_number 5; oId CUFF.32919.1; tss_id TSS32951;
7	Cufflinks	exon	141921371	141921808	.	+	.	gene_id GRMZM2G052610; transcript_id TCONS_00036417; exon_number 6; oId CUFF.32919.1; tss_id TSS32951;
7	Cufflinks	exon	150455168	150455568	.	+	.	gene_id GRMZM2G075719; transcript_id TCONS_00036562; exon_number 1; oId CUFF.33148.1; tss_id TSS33077;
7	Cufflinks	exon	150456648	150456718	.	+	.	gene_id GRMZM2G075719; transcript_id TCONS_00036562; exon_number 2; oId CUFF.33148.1; tss_id TSS33077;
7	Cufflinks	exon	150456810	150456898	.	+	.	gene_id GRMZM2G075719; transcript_id TCONS_00036562; exon_number 3; oId CUFF.33148.1; tss_id TSS33077;
7	Cufflinks	exon	150457067	150457178	.	+	.	gene_id GRMZM2G075719; transcript_id TCONS_00036562; exon_number 4; oId CUFF.33148.1; tss_id TSS33077;
7	Cufflinks	exon	150457857	150457947	.	+	.	gene_id GRMZM2G075719; transcript_id TCONS_00036562; exon_number 5; oId CUFF.33148.1; tss_id TSS33077;
7	Cufflinks	exon	150458403	150458991	.	+	.	gene_id GRMZM2G075719; transcript_id TCONS_00036562; exon_number 6; oId CUFF.33148.1; tss_id TSS33077;
7	Cufflinks	exon	151226660	151228687	.	+	.	gene_id GRMZM2G155911; transcript_id TCONS_00036578; exon_number 1; oId CUFF.33179.1; tss_id TSS33091;
7	Cufflinks	exon	152808921	152809072	.	+	.	gene_id GRMZM2G108849; transcript_id TCONS_00036599; exon_number 1; oId CUFF.33223.1; tss_id TSS33110;
7	Cufflinks	exon	152809232	152809329	.	+	.	gene_id GRMZM2G108849; transcript_id TCONS_00036599; exon_number 2; oId CUFF.33223.1; tss_id TSS33110;
7	Cufflinks	exon	152810338	152810382	.	+	.	gene_id GRMZM2G108849; transcript_id TCONS_00036599; exon_number 3; oId CUFF.33223.1; tss_id TSS33110;
7	Cufflinks	exon	152810476	152810556	.	+	.	gene_id GRMZM2G108849; transcript_id TCONS_00036599; exon_number 4; oId CUFF.33223.1; tss_id TSS33110;
7	Cufflinks	exon	152810666	152810822	.	+	.	gene_id GRMZM2G108849; transcript_id TCONS_00036599; exon_number 5; oId CUFF.33223.1; tss_id TSS33110;
7	Cufflinks	exon	152811029	152811108	.	+	.	gene_id GRMZM2G108849; transcript_id TCONS_00036599; exon_number 6; oId CUFF.33223.1; tss_id TSS33110;
7	Cufflinks	exon	152811220	152811345	.	+	.	gene_id GRMZM2G108849; transcript_id TCONS_00036599; exon_number 7; oId CUFF.33223.1; tss_id TSS33110;
7	Cufflinks	exon	152811845	152812063	.	+	.	gene_id GRMZM2G108849; transcript_id TCONS_00036599; exon_number 8; oId CUFF.33223.1; tss_id TSS33110;
7	Cufflinks	exon	152812393	152812506	.	+	.	gene_id GRMZM2G108849; transcript_id TCONS_00036599; exon_number 9; oId CUFF.33223.1; tss_id TSS33110;
7	Cufflinks	exon	152812595	152812630	.	+	.	gene_id GRMZM2G108849; transcript_id TCONS_00036599; exon_number 10; oId CUFF.33223.1; tss_id TSS33110;
7	Cufflinks	exon	152812722	152813134	.	+	.	gene_id GRMZM2G108849; transcript_id TCONS_00036599; exon_number 11; oId CUFF.33223.1; tss_id TSS33110;
7	Cufflinks	exon	153330905	153331175	.	+	.	gene_id GRMZM2G429396; transcript_id TCONS_00036611; exon_number 1; oId CUFF.33230.1; tss_id TSS33119;
7	Cufflinks	exon	153331291	153331987	.	+	.	gene_id GRMZM2G429396; transcript_id TCONS_00036611; exon_number 2; oId CUFF.33230.1; tss_id TSS33119;
7	Cufflinks	exon	153692958	153693106	.	+	.	gene_id GRMZM2G167591; transcript_id TCONS_00036614; exon_number 1; oId CUFF.33244.1; tss_id TSS33122;
7	Cufflinks	exon	153695189	153695938	.	+	.	gene_id GRMZM2G167591; transcript_id TCONS_00036614; exon_number 2; oId CUFF.33244.1; tss_id TSS33122;
7	Cufflinks	exon	155396590	155396875	.	+	.	gene_id GRMZM2G058310; transcript_id TCONS_00036654; exon_number 1; oId CUFF.33327.1; tss_id TSS33160;
7	Cufflinks	exon	155397073	155397471	.	+	.	gene_id GRMZM2G058310; transcript_id TCONS_00036654; exon_number 2; oId CUFF.33327.1; tss_id TSS33160;

7	Cufflinks	exon	155397573	155397737	.	+	.	gene_id GRMZM2G058310; transcript_id TCONS_00036654; exon_number 3; oId CUFF.33327.1; tss_id TSS33160;
7	Cufflinks	exon	155398218	155398481	.	+	.	gene_id GRMZM2G058310; transcript_id TCONS_00036654; exon_number 4; oId CUFF.33327.1; tss_id TSS33160;
7	Cufflinks	exon	155398665	155398874	.	+	.	gene_id GRMZM2G058310; transcript_id TCONS_00036654; exon_number 5; oId CUFF.33327.1; tss_id TSS33160;
7	Cufflinks	exon	155398978	155399217	.	+	.	gene_id GRMZM2G058310; transcript_id TCONS_00036654; exon_number 6; oId CUFF.33327.1; tss_id TSS33160;
7	Cufflinks	exon	155399308	155399705	.	+	.	gene_id GRMZM2G058310; transcript_id TCONS_00036654; exon_number 7; oId CUFF.33327.1; tss_id TSS33160;
7	Cufflinks	exon	160189515	160190226	.	+	.	gene_id GRMZM5G889052; transcript_id TCONS_00036753; exon_number 1; oId CUFF.33491.1; tss_id TSS33246;
7	Cufflinks	exon	160190898	160193189	.	+	.	gene_id GRMZM5G889052; transcript_id TCONS_00036753; exon_number 2; oId CUFF.33491.1; tss_id TSS33246;
7	Cufflinks	exon	160854635	160854839	.	+	.	gene_id GRMZM2G302604; transcript_id TCONS_00036767; exon_number 1; oId CUFF.33513.1; tss_id TSS33259;
7	Cufflinks	exon	160854931	160856784	.	+	.	gene_id GRMZM2G302604; transcript_id TCONS_00036767; exon_number 2; oId CUFF.33513.1; tss_id TSS33259;
7	Cufflinks	exon	161376313	161376913	.	+	.	gene_id GRMZM2G023444; transcript_id TCONS_00036771; exon_number 1; oId CUFF.33517.1; tss_id TSS33262;
7	Cufflinks	exon	161377648	161379215	.	+	.	gene_id GRMZM2G023444; transcript_id TCONS_00036771; exon_number 2; oId CUFF.33517.1; tss_id TSS33262;
7	Cufflinks	exon	161386057	161386853	.	+	.	gene_id GRMZM2G023346; transcript_id TCONS_00036773; exon_number 1; oId CUFF.33529.1; tss_id TSS33264;
7	Cufflinks	exon	161386948	161387017	.	+	.	gene_id GRMZM2G023346; transcript_id TCONS_00036773; exon_number 2; oId CUFF.33529.1; tss_id TSS33264;
7	Cufflinks	exon	161387095	161388246	.	+	.	gene_id GRMZM2G023346; transcript_id TCONS_00036773; exon_number 3; oId CUFF.33529.1; tss_id TSS33264;
7	Cufflinks	exon	161388345	161389217	.	+	.	gene_id GRMZM2G023346; transcript_id TCONS_00036773; exon_number 4; oId CUFF.33529.1; tss_id TSS33264;
7	Cufflinks	exon	161941447	161944260	.	+	.	gene_id GRMZM2G172657; transcript_id TCONS_00036782; exon_number 1; oId CUFF.33545.1; tss_id TSS33272;
7	Cufflinks	exon	162108103	162109600	.	+	.	gene_id GRMZM2G057247; transcript_id TCONS_00036785; exon_number 1; oId CUFF.33568.1; tss_id TSS33275;
7	Cufflinks	exon	162109690	162109886	.	+	.	gene_id GRMZM2G057247; transcript_id TCONS_00036785; exon_number 2; oId CUFF.33568.1; tss_id TSS33275;
7	Cufflinks	exon	162112729	162112967	.	+	.	gene_id GRMZM2G057247; transcript_id TCONS_00036785; exon_number 3; oId CUFF.33568.1; tss_id TSS33275;
7	Cufflinks	exon	162113500	162114970	.	+	.	gene_id GRMZM2G057247; transcript_id TCONS_00036785; exon_number 4; oId CUFF.33568.1; tss_id TSS33275;
7	Cufflinks	exon	163042101	163042441	.	+	.	gene_id XLOC_032238; transcript_id TCONS_00036801; exon_number 1; oId CUFF.33593.1; tss_id TSS33290;
7	Cufflinks	exon	165496269	165496647	.	+	.	gene_id GRMZM2G089736; transcript_id TCONS_00036858; exon_number 1; oId CUFF.33679.1; tss_id TSS33344;
7	Cufflinks	exon	165496889	165497030	.	+	.	gene_id GRMZM2G089736; transcript_id TCONS_00036858; exon_number 2; oId CUFF.33679.1; tss_id TSS33344;
7	Cufflinks	exon	165497570	165497823	.	+	.	gene_id GRMZM2G089736; transcript_id TCONS_00036858; exon_number 3; oId CUFF.33679.1; tss_id TSS33344;
7	Cufflinks	exon	165497934	165497991	.	+	.	gene_id GRMZM2G089736; transcript_id TCONS_00036858; exon_number 4; oId CUFF.33679.1; tss_id TSS33344;
7	Cufflinks	exon	165498093	165498636	.	+	.	gene_id GRMZM2G089736; transcript_id TCONS_00036858; exon_number 5; oId CUFF.33679.1; tss_id TSS33344;
7	Cufflinks	exon	166410307	166410751	.	+	.	gene_id GRMZM2G056732; transcript_id TCONS_00036877; exon_number 1; oId CUFF.33714.1; tss_id TSS33363;
7	Cufflinks	exon	166412381	166412455	.	+	.	gene_id GRMZM2G056732; transcript_id TCONS_00036877; exon_number 2; oId CUFF.33714.1; tss_id TSS33363;
7	Cufflinks	exon	166414117	166414218	.	+	.	gene_id GRMZM2G056732; transcript_id TCONS_00036877; exon_number 3; oId CUFF.33714.1; tss_id TSS33363;
7	Cufflinks	exon	166414303	166414356	.	+	.	gene_id GRMZM2G056732; transcript_id TCONS_00036877; exon_number 4; oId CUFF.33714.1; tss_id TSS33363;
7	Cufflinks	exon	166414553	166414645	.	+	.	gene_id GRMZM2G056732; transcript_id TCONS_00036877; exon_number 5; oId CUFF.33714.1; tss_id TSS33363;
7	Cufflinks	exon	166414722	166414814	.	+	.	gene_id GRMZM2G056732; transcript_id TCONS_00036877; exon_number 6; oId CUFF.33714.1; tss_id TSS33363;
7	Cufflinks	exon	166415116	166415220	.	+	.	gene_id GRMZM2G056732; transcript_id TCONS_00036877; exon_number 7; oId CUFF.33714.1; tss_id TSS33363;
7	Cufflinks	exon	166415304	166415402	.	+	.	gene_id GRMZM2G056732; transcript_id TCONS_00036877; exon_number 8; oId CUFF.33714.1; tss_id TSS33363;
7	Cufflinks	exon	166415475	166416117	.	+	.	gene_id GRMZM2G056732; transcript_id TCONS_00036877; exon_number 9; oId CUFF.33714.1; tss_id TSS33363;
7	Cufflinks	exon	167674297	167674491	.	+	.	gene_id GRMZM2G141322; transcript_id TCONS_00036902; exon_number 1; oId CUFF.33758.1; tss_id TSS33384;

7	Cufflinks	exon	167674645	167675623	.	+	.	gene_id GRMZM2G141322; transcript_id TCONS_00036902; exon_number 2; oId CUFF.33758.1; tss_id TSS33384;
7	Cufflinks	exon	167946724	167947210	.	+	.	gene_id GRMZM2G150758; transcript_id TCONS_00036909; exon_number 1; oId CUFF.33789.1; tss_id TSS33390;
7	Cufflinks	exon	167947325	167947411	.	+	.	gene_id GRMZM2G150758; transcript_id TCONS_00036909; exon_number 2; oId CUFF.33789.1; tss_id TSS33390;
7	Cufflinks	exon	167947501	167947608	.	+	.	gene_id GRMZM2G150758; transcript_id TCONS_00036909; exon_number 3; oId CUFF.33789.1; tss_id TSS33390;
7	Cufflinks	exon	167947707	167948218	.	+	.	gene_id GRMZM2G150758; transcript_id TCONS_00036909; exon_number 4; oId CUFF.33789.1; tss_id TSS33390;
7	Cufflinks	exon	168019762	168020329	.	+	.	gene_id GRMZM2G318843; transcript_id TCONS_00036913; exon_number 1; oId CUFF.33775.1; tss_id TSS33392;
7	Cufflinks	exon	168020452	168020566	.	+	.	gene_id GRMZM2G318843; transcript_id TCONS_00036913; exon_number 2; oId CUFF.33775.1; tss_id TSS33392;
7	Cufflinks	exon	168020663	168020728	.	+	.	gene_id GRMZM2G318843; transcript_id TCONS_00036913; exon_number 3; oId CUFF.33775.1; tss_id TSS33392;
7	Cufflinks	exon	168020818	168020906	.	+	.	gene_id GRMZM2G318843; transcript_id TCONS_00036913; exon_number 4; oId CUFF.33775.1; tss_id TSS33392;
7	Cufflinks	exon	168021001	168021088	.	+	.	gene_id GRMZM2G318843; transcript_id TCONS_00036913; exon_number 5; oId CUFF.33775.1; tss_id TSS33392;
7	Cufflinks	exon	168021199	168021345	.	+	.	gene_id GRMZM2G318843; transcript_id TCONS_00036913; exon_number 6; oId CUFF.33775.1; tss_id TSS33392;
7	Cufflinks	exon	168021451	168021543	.	+	.	gene_id GRMZM2G318843; transcript_id TCONS_00036913; exon_number 7; oId CUFF.33775.1; tss_id TSS33392;
7	Cufflinks	exon	168021684	168021785	.	+	.	gene_id GRMZM2G318843; transcript_id TCONS_00036913; exon_number 8; oId CUFF.33775.1; tss_id TSS33392;
7	Cufflinks	exon	168021873	168022899	.	+	.	gene_id GRMZM2G318843; transcript_id TCONS_00036913; exon_number 9; oId CUFF.33775.1; tss_id TSS33392;
7	Cufflinks	exon	168441397	168441886	.	+	.	gene_id GRMZM2G477325; transcript_id TCONS_00036926; exon_number 1; oId CUFF.33795.1; tss_id TSS33405;
7	Cufflinks	exon	168441910	168442008	.	+	.	gene_id GRMZM2G477325; transcript_id TCONS_00036926; exon_number 2; oId CUFF.33795.1; tss_id TSS33405;
7	Cufflinks	exon	168442120	168442283	.	+	.	gene_id GRMZM2G477325; transcript_id TCONS_00036926; exon_number 3; oId CUFF.33795.1; tss_id TSS33405;
7	Cufflinks	exon	168442326	168442596	.	+	.	gene_id GRMZM2G477325; transcript_id TCONS_00036926; exon_number 4; oId CUFF.33795.1; tss_id TSS33405;
7	Cufflinks	exon	170071170	170071427	.	+	.	gene_id GRMZM2G055607; transcript_id TCONS_00036961; exon_number 1; oId CUFF.33887.1; tss_id TSS33440;
7	Cufflinks	exon	170072076	170072207	.	+	.	gene_id GRMZM2G055607; transcript_id TCONS_00036961; exon_number 2; oId CUFF.33887.1; tss_id TSS33440;
7	Cufflinks	exon	170072304	170072542	.	+	.	gene_id GRMZM2G055607; transcript_id TCONS_00036961; exon_number 3; oId CUFF.33887.1; tss_id TSS33440;
7	Cufflinks	exon	170072653	170072911	.	+	.	gene_id GRMZM2G055607; transcript_id TCONS_00036961; exon_number 4; oId CUFF.33887.1; tss_id TSS33440;
7	Cufflinks	exon	170073337	170073516	.	+	.	gene_id GRMZM2G055607; transcript_id TCONS_00036961; exon_number 5; oId CUFF.33887.1; tss_id TSS33440;
7	Cufflinks	exon	170073774	170073929	.	+	.	gene_id GRMZM2G055607; transcript_id TCONS_00036961; exon_number 6; oId CUFF.33887.1; tss_id TSS33440;
7	Cufflinks	exon	170074027	170074488	.	+	.	gene_id GRMZM2G055607; transcript_id TCONS_00036961; exon_number 7; oId CUFF.33887.1; tss_id TSS33440;
7	Cufflinks	exon	170071549	170071796	.	+	.	gene_id GRMZM2G055607; transcript_id TCONS_00036962; exon_number 1; oId CUFF.33887.2; tss_id TSS33441;
7	Cufflinks	exon	170072076	170072207	.	+	.	gene_id GRMZM2G055607; transcript_id TCONS_00036962; exon_number 2; oId CUFF.33887.2; tss_id TSS33441;
7	Cufflinks	exon	170072304	170072542	.	+	.	gene_id GRMZM2G055607; transcript_id TCONS_00036962; exon_number 3; oId CUFF.33887.2; tss_id TSS33441;
7	Cufflinks	exon	170072653	170072911	.	+	.	gene_id GRMZM2G055607; transcript_id TCONS_00036962; exon_number 4; oId CUFF.33887.2; tss_id TSS33441;
7	Cufflinks	exon	170073337	170073516	.	+	.	gene_id GRMZM2G055607; transcript_id TCONS_00036962; exon_number 5; oId CUFF.33887.2; tss_id TSS33441;
7	Cufflinks	exon	170073774	170073929	.	+	.	gene_id GRMZM2G055607; transcript_id TCONS_00036962; exon_number 6; oId CUFF.33887.2; tss_id TSS33441;
7	Cufflinks	exon	170074027	170074488	.	+	.	gene_id GRMZM2G055607; transcript_id TCONS_00036962; exon_number 7; oId CUFF.33887.2; tss_id TSS33441;
7	Cufflinks	exon	171863190	171864506	.	+	.	gene_id GRMZM2G414423; transcript_id TCONS_00037013; exon_number 1; oId CUFF.33934.1; tss_id TSS33488;
7	Cufflinks	exon	171864624	171864934	.	+	.	gene_id GRMZM2G414423; transcript_id TCONS_00037013; exon_number 2; oId CUFF.33934.1; tss_id TSS33488;
7	Cufflinks	exon	171865048	171865343	.	+	.	gene_id GRMZM2G414423; transcript_id TCONS_00037013; exon_number 3; oId CUFF.33934.1; tss_id TSS33488;
7	Cufflinks	exon	172809428	172809761	.	+	.	gene_id GRMZM2G427815; transcript_id TCONS_00037043; exon_number 1; oId CUFF.33998.1; tss_id TSS33517;

7	Cufflinks	exon	172809970	172810167	.	+	.	gene_id GRMZM2G427815; transcript_id TCONS_00037043; exon_number 2; oId CUFF.33998.1; tss_id TSS33517;
7	Cufflinks	exon	172810280	172811102	.	+	.	gene_id GRMZM2G427815; transcript_id TCONS_00037043; exon_number 3; oId CUFF.33998.1; tss_id TSS33517;
7	Cufflinks	exon	174614466	174615537	.	+	.	gene_id GRMZM2G056772; transcript_id TCONS_00037091; exon_number 1; oId CUFF.34089.1; tss_id TSS33559;
7	Cufflinks	exon	175611974	175613300	.	+	.	gene_id GRMZM2G032376; transcript_id TCONS_00037115; exon_number 1; oId CUFF.34174.1; tss_id TSS33580;
7	Cufflinks	exon	175613789	175613978	.	+	.	gene_id GRMZM2G032376; transcript_id TCONS_00037115; exon_number 2; oId CUFF.34174.1; tss_id TSS33580;
7	Cufflinks	exon	175614124	175614512	.	+	.	gene_id GRMZM2G032376; transcript_id TCONS_00037115; exon_number 3; oId CUFF.34174.1; tss_id TSS33580;
7	Cufflinks	exon	175614596	175614726	.	+	.	gene_id GRMZM2G032376; transcript_id TCONS_00037115; exon_number 4; oId CUFF.34174.1; tss_id TSS33580;
7	Cufflinks	exon	175614771	175615007	.	+	.	gene_id GRMZM2G032376; transcript_id TCONS_00037115; exon_number 5; oId CUFF.34174.1; tss_id TSS33580;
7	Cufflinks	exon	175615100	175615355	.	+	.	gene_id GRMZM2G032376; transcript_id TCONS_00037115; exon_number 6; oId CUFF.34174.1; tss_id TSS33580;
7	Cufflinks	exon	175615445	175615934	.	+	.	gene_id GRMZM2G032376; transcript_id TCONS_00037115; exon_number 7; oId CUFF.34174.1; tss_id TSS33580;
7	Cufflinks	exon	272380	273997	.	-	.	gene_id GRMZM5G894233; transcript_id TCONS_00037134; exon_number 1; oId CUFF.30976.3; tss_id TSS33595;
7	Cufflinks	exon	274085	274300	.	-	.	gene_id GRMZM5G894233; transcript_id TCONS_00037134; exon_number 2; oId CUFF.30976.3; tss_id TSS33595;
7	Cufflinks	exon	274372	274689	.	-	.	gene_id GRMZM5G894233; transcript_id TCONS_00037134; exon_number 3; oId CUFF.30976.3; tss_id TSS33595;
7	Cufflinks	exon	274797	274891	.	-	.	gene_id GRMZM5G894233; transcript_id TCONS_00037134; exon_number 4; oId CUFF.30976.3; tss_id TSS33595;
7	Cufflinks	exon	274999	275223	.	-	.	gene_id GRMZM5G894233; transcript_id TCONS_00037134; exon_number 5; oId CUFF.30976.3; tss_id TSS33595;
7	Cufflinks	exon	275308	275529	.	-	.	gene_id GRMZM5G894233; transcript_id TCONS_00037134; exon_number 6; oId CUFF.30976.3; tss_id TSS33595;
7	Cufflinks	exon	276041	276643	.	-	.	gene_id GRMZM5G894233; transcript_id TCONS_00037134; exon_number 7; oId CUFF.30976.3; tss_id TSS33595;
7	Cufflinks	exon	272380	273997	.	-	.	gene_id GRMZM5G894233; transcript_id TCONS_00037133; exon_number 1; oId CUFF.30976.2; tss_id TSS33595;
7	Cufflinks	exon	274085	274300	.	-	.	gene_id GRMZM5G894233; transcript_id TCONS_00037133; exon_number 2; oId CUFF.30976.2; tss_id TSS33595;
7	Cufflinks	exon	274372	274689	.	-	.	gene_id GRMZM5G894233; transcript_id TCONS_00037133; exon_number 3; oId CUFF.30976.2; tss_id TSS33595;
7	Cufflinks	exon	274797	274891	.	-	.	gene_id GRMZM5G894233; transcript_id TCONS_00037133; exon_number 4; oId CUFF.30976.2; tss_id TSS33595;
7	Cufflinks	exon	274999	275223	.	-	.	gene_id GRMZM5G894233; transcript_id TCONS_00037133; exon_number 5; oId CUFF.30976.2; tss_id TSS33595;
7	Cufflinks	exon	275308	275461	.	-	.	gene_id GRMZM5G894233; transcript_id TCONS_00037133; exon_number 6; oId CUFF.30976.2; tss_id TSS33595;
7	Cufflinks	exon	276041	276643	.	-	.	gene_id GRMZM5G894233; transcript_id TCONS_00037133; exon_number 7; oId CUFF.30976.2; tss_id TSS33595;
7	Cufflinks	exon	1272770	1273988	.	-	.	gene_id GRMZM2G420733; transcript_id TCONS_00037153; exon_number 1; oId CUFF.30989.1; tss_id TSS33612;
7	Cufflinks	exon	3252792	3253074	.	-	.	gene_id XLOC_032592; transcript_id TCONS_00037202; exon_number 1; oId CUFF.31067.1; tss_id TSS33658;
7	Cufflinks	exon	6073362	6073696	.	-	.	gene_id GRMZM2G026614; transcript_id TCONS_00037236; exon_number 1; oId CUFF.31142.1; tss_id TSS33692;
7	Cufflinks	exon	6073829	6073930	.	-	.	gene_id GRMZM2G026614; transcript_id TCONS_00037236; exon_number 2; oId CUFF.31142.1; tss_id TSS33692;
7	Cufflinks	exon	6074040	6074529	.	-	.	gene_id GRMZM2G026614; transcript_id TCONS_00037236; exon_number 3; oId CUFF.31142.1; tss_id TSS33692;
7	Cufflinks	exon	9547566	9548174	.	-	.	gene_id GRMZM2G132568; transcript_id TCONS_00037284; exon_number 1; oId CUFF.31229.1; tss_id TSS33739;
7	Cufflinks	exon	9548296	9548453	.	-	.	gene_id GRMZM2G132568; transcript_id TCONS_00037284; exon_number 2; oId CUFF.31229.1; tss_id TSS33739;
7	Cufflinks	exon	9550390	9550503	.	-	.	gene_id GRMZM2G132568; transcript_id TCONS_00037284; exon_number 3; oId CUFF.31229.1; tss_id TSS33739;
7	Cufflinks	exon	9551262	9551755	.	-	.	gene_id GRMZM2G132568; transcript_id TCONS_00037284; exon_number 4; oId CUFF.31229.1; tss_id TSS33739;
7	Cufflinks	exon	10656435	10657883	.	-	.	gene_id XLOC_032682; transcript_id TCONS_00037294; exon_number 1; oId CUFF.31250.1; tss_id TSS33749;
7	Cufflinks	exon	11446449	11447265	.	-	.	gene_id GRMZM2G401308; transcript_id TCONS_00037301; exon_number 1; oId CUFF.31274.1; tss_id TSS33756;
7	Cufflinks	exon	11447425	11447690	.	-	.	gene_id GRMZM2G401308; transcript_id TCONS_00037301; exon_number 2; oId CUFF.31274.1; tss_id TSS33756;

7	Cufflinks	exon	13623866	13626771	.	-	.	gene_id GRMZM2G458494; transcript_id TCONS_00037330; exon_number 1; oId CUFF.31315.1; tss_id TSS33774;
7	Cufflinks	exon	15013266	15014357	.	-	.	gene_id GRMZM2G028129; transcript_id TCONS_00037338; exon_number 1; oId CUFF.31351.1; tss_id TSS33781;
7	Cufflinks	exon	15014439	15015247	.	-	.	gene_id GRMZM2G028129; transcript_id TCONS_00037338; exon_number 2; oId CUFF.31351.1; tss_id TSS33781;
7	Cufflinks	exon	18620430	18621416	.	-	.	gene_id GRMZM2G424832; transcript_id TCONS_00037371; exon_number 1; oId CUFF.31403.1; tss_id TSS33811;
7	Cufflinks	exon	18621522	18621872	.	-	.	gene_id GRMZM2G424832; transcript_id TCONS_00037371; exon_number 2; oId CUFF.31403.1; tss_id TSS33811;
7	Cufflinks	exon	18621989	18622191	.	-	.	gene_id GRMZM2G424832; transcript_id TCONS_00037371; exon_number 3; oId CUFF.31403.1; tss_id TSS33811;
7	Cufflinks	exon	18622348	18622600	.	-	.	gene_id GRMZM2G424832; transcript_id TCONS_00037371; exon_number 4; oId CUFF.31403.1; tss_id TSS33811;
7	Cufflinks	exon	18622710	18622922	.	-	.	gene_id GRMZM2G424832; transcript_id TCONS_00037371; exon_number 5; oId CUFF.31403.1; tss_id TSS33811;
7	Cufflinks	exon	18623014	18623139	.	-	.	gene_id GRMZM2G424832; transcript_id TCONS_00037371; exon_number 6; oId CUFF.31403.1; tss_id TSS33811;
7	Cufflinks	exon	18623267	18623404	.	-	.	gene_id GRMZM2G424832; transcript_id TCONS_00037371; exon_number 7; oId CUFF.31403.1; tss_id TSS33811;
7	Cufflinks	exon	18623485	18623830	.	-	.	gene_id GRMZM2G424832; transcript_id TCONS_00037371; exon_number 8; oId CUFF.31403.1; tss_id TSS33811;
7	Cufflinks	exon	18623925	18624191	.	-	.	gene_id GRMZM2G424832; transcript_id TCONS_00037371; exon_number 9; oId CUFF.31403.1; tss_id TSS33811;
7	Cufflinks	exon	18624642	18624831	.	-	.	gene_id GRMZM2G424832; transcript_id TCONS_00037371; exon_number 10; oId CUFF.31403.1; tss_id TSS33811;
7	Cufflinks	exon	18624922	18625021	.	-	.	gene_id GRMZM2G424832; transcript_id TCONS_00037371; exon_number 11; oId CUFF.31403.1; tss_id TSS33811;
7	Cufflinks	exon	18625106	18625350	.	-	.	gene_id GRMZM2G424832; transcript_id TCONS_00037371; exon_number 12; oId CUFF.31403.1; tss_id TSS33811;
7	Cufflinks	exon	18625699	18625891	.	-	.	gene_id GRMZM2G424832; transcript_id TCONS_00037371; exon_number 13; oId CUFF.31403.1; tss_id TSS33811;
7	Cufflinks	exon	18625999	18626288	.	-	.	gene_id GRMZM2G424832; transcript_id TCONS_00037371; exon_number 14; oId CUFF.31403.1; tss_id TSS33811;
7	Cufflinks	exon	19636888	19639754	.	-	.	gene_id XLOC_032757; transcript_id TCONS_00037385; exon_number 1; oId CUFF.31423.2; tss_id TSS33824;
7	Cufflinks	exon	19639863	19639943	.	-	.	gene_id XLOC_032757; transcript_id TCONS_00037385; exon_number 2; oId CUFF.31423.2; tss_id TSS33824;
7	Cufflinks	exon	19640186	19640242	.	-	.	gene_id XLOC_032757; transcript_id TCONS_00037385; exon_number 3; oId CUFF.31423.2; tss_id TSS33824;
7	Cufflinks	exon	19640344	19642833	.	-	.	gene_id XLOC_032757; transcript_id TCONS_00037385; exon_number 4; oId CUFF.31423.2; tss_id TSS33824;
7	Cufflinks	exon	23364784	23365989	.	-	.	gene_id GRMZM5G892627; transcript_id TCONS_00037417; exon_number 1; oId CUFF.31478.2; tss_id TSS33853;
7	Cufflinks	exon	23366083	23366680	.	-	.	gene_id GRMZM5G892627; transcript_id TCONS_00037417; exon_number 2; oId CUFF.31478.2; tss_id TSS33853;
7	Cufflinks	exon	23364784	23365981	.	-	.	gene_id GRMZM5G892627; transcript_id TCONS_00037416; exon_number 1; oId CUFF.31478.1; tss_id TSS33853;
7	Cufflinks	exon	23366083	23366680	.	-	.	gene_id GRMZM5G892627; transcript_id TCONS_00037416; exon_number 2; oId CUFF.31478.1; tss_id TSS33853;
7	Cufflinks	exon	23364784	23365995	.	-	.	gene_id GRMZM5G892627; transcript_id TCONS_00037418; exon_number 1; oId CUFF.31478.3; tss_id TSS33853;
7	Cufflinks	exon	23366083	23366705	.	-	.	gene_id GRMZM5G892627; transcript_id TCONS_00037418; exon_number 2; oId CUFF.31478.3; tss_id TSS33853;
7	Cufflinks	exon	25552619	25552831	.	-	.	gene_id XLOC_032804; transcript_id TCONS_00037438; exon_number 1; oId CUFF.31509.1; tss_id TSS33872;
7	Cufflinks	exon	25553116	25553236	.	-	.	gene_id XLOC_032804; transcript_id TCONS_00037438; exon_number 2; oId CUFF.31509.1; tss_id TSS33872;
7	Cufflinks	exon	25819517	25820377	.	-	.	gene_id XLOC_032807; transcript_id TCONS_00037441; exon_number 1; oId CUFF.31513.1; tss_id TSS33875;
7	Cufflinks	exon	41435269	41435742	.	-	.	gene_id GRMZM2G014914; transcript_id TCONS_00037516; exon_number 1; oId CUFF.31681.1; tss_id TSS33947;
7	Cufflinks	exon	41435821	41435961	.	-	.	gene_id GRMZM2G014914; transcript_id TCONS_00037516; exon_number 2; oId CUFF.31681.1; tss_id TSS33947;
7	Cufflinks	exon	41437734	41438511	.	-	.	gene_id GRMZM2G014914; transcript_id TCONS_00037516; exon_number 3; oId CUFF.31681.1; tss_id TSS33947;
7	Cufflinks	exon	52269319	52269859	.	-	.	gene_id XLOC_032933; transcript_id TCONS_00037583; exon_number 1; oId CUFF.31794.1; tss_id TSS34006;
7	Cufflinks	exon	54345911	54348241	.	-	.	gene_id GRMZM2G318292; transcript_id TCONS_00037590; exon_number 1; oId CUFF.31810.1; tss_id TSS34013;
7	Cufflinks	exon	89458280	89458635	.	-	.	gene_id GRMZM2G047592; transcript_id TCONS_00037733; exon_number 1; oId CUFF.32063.1; tss_id TSS34143;

7	Cufflinks	exon	89458753	89458834	.	-	.	gene_id GRMZM2G047592; transcript_id TCONS_00037733; exon_number 2; oId CUFF.32063.1; tss_id TSS34143;
7	Cufflinks	exon	89458919	89459026	.	-	.	gene_id GRMZM2G047592; transcript_id TCONS_00037733; exon_number 3; oId CUFF.32063.1; tss_id TSS34143;
7	Cufflinks	exon	89459317	89459414	.	-	.	gene_id GRMZM2G047592; transcript_id TCONS_00037733; exon_number 4; oId CUFF.32063.1; tss_id TSS34143;
7	Cufflinks	exon	89459491	89459589	.	-	.	gene_id GRMZM2G047592; transcript_id TCONS_00037733; exon_number 5; oId CUFF.32063.1; tss_id TSS34143;
7	Cufflinks	exon	89459729	89459866	.	-	.	gene_id GRMZM2G047592; transcript_id TCONS_00037733; exon_number 6; oId CUFF.32063.1; tss_id TSS34143;
7	Cufflinks	exon	89460237	89460288	.	-	.	gene_id GRMZM2G047592; transcript_id TCONS_00037733; exon_number 7; oId CUFF.32063.1; tss_id TSS34143;
7	Cufflinks	exon	89460374	89460487	.	-	.	gene_id GRMZM2G047592; transcript_id TCONS_00037733; exon_number 8; oId CUFF.32063.1; tss_id TSS34143;
7	Cufflinks	exon	89461249	89461365	.	-	.	gene_id GRMZM2G047592; transcript_id TCONS_00037733; exon_number 9; oId CUFF.32063.1; tss_id TSS34143;
7	Cufflinks	exon	89461475	89461662	.	-	.	gene_id GRMZM2G047592; transcript_id TCONS_00037733; exon_number 10; oId CUFF.32063.1; tss_id TSS34143;
7	Cufflinks	exon	101604486	101605006	.	-	.	gene_id GRMZM2G016660; transcript_id TCONS_00037816; exon_number 1; oId CUFF.32246.2; tss_id TSS34219;
7	Cufflinks	exon	101605544	101605668	.	-	.	gene_id GRMZM2G016660; transcript_id TCONS_00037816; exon_number 2; oId CUFF.32246.2; tss_id TSS34219;
7	Cufflinks	exon	101605950	101606135	.	-	.	gene_id GRMZM2G016660; transcript_id TCONS_00037816; exon_number 3; oId CUFF.32246.2; tss_id TSS34219;
7	Cufflinks	exon	101606687	101606818	.	-	.	gene_id GRMZM2G016660; transcript_id TCONS_00037816; exon_number 4; oId CUFF.32246.2; tss_id TSS34219;
7	Cufflinks	exon	101613248	101613390	.	-	.	gene_id GRMZM2G016660; transcript_id TCONS_00037816; exon_number 5; oId CUFF.32246.2; tss_id TSS34219;
7	Cufflinks	exon	101613665	101613981	.	-	.	gene_id GRMZM2G016660; transcript_id TCONS_00037816; exon_number 6; oId CUFF.32246.2; tss_id TSS34219;
7	Cufflinks	exon	101604486	101605006	.	-	.	gene_id GRMZM2G016660; transcript_id TCONS_00037815; exon_number 1; oId CUFF.32246.1; tss_id TSS34219;
7	Cufflinks	exon	101605544	101605668	.	-	.	gene_id GRMZM2G016660; transcript_id TCONS_00037815; exon_number 2; oId CUFF.32246.1; tss_id TSS34219;
7	Cufflinks	exon	101605950	101606135	.	-	.	gene_id GRMZM2G016660; transcript_id TCONS_00037815; exon_number 3; oId CUFF.32246.1; tss_id TSS34219;
7	Cufflinks	exon	101606687	101606827	.	-	.	gene_id GRMZM2G016660; transcript_id TCONS_00037815; exon_number 4; oId CUFF.32246.1; tss_id TSS34219;
7	Cufflinks	exon	101613248	101613390	.	-	.	gene_id GRMZM2G016660; transcript_id TCONS_00037815; exon_number 5; oId CUFF.32246.1; tss_id TSS34219;
7	Cufflinks	exon	101613665	101613981	.	-	.	gene_id GRMZM2G016660; transcript_id TCONS_00037815; exon_number 6; oId CUFF.32246.1; tss_id TSS34219;
7	Cufflinks	exon	103534410	103535834	.	-	.	gene_id GRMZM2G036916; transcript_id TCONS_00037827; exon_number 1; oId CUFF.32226.1; tss_id TSS34230;
7	Cufflinks	exon	103536116	103536370	.	-	.	gene_id GRMZM2G036916; transcript_id TCONS_00037827; exon_number 2; oId CUFF.32226.1; tss_id TSS34230;
7	Cufflinks	exon	103536450	103536564	.	-	.	gene_id GRMZM2G036916; transcript_id TCONS_00037827; exon_number 3; oId CUFF.32226.1; tss_id TSS34230;
7	Cufflinks	exon	103536672	103536724	.	-	.	gene_id GRMZM2G036916; transcript_id TCONS_00037827; exon_number 4; oId CUFF.32226.1; tss_id TSS34230;
7	Cufflinks	exon	103537156	103537416	.	-	.	gene_id GRMZM2G036916; transcript_id TCONS_00037827; exon_number 5; oId CUFF.32226.1; tss_id TSS34230;
7	Cufflinks	exon	103537567	103537617	.	-	.	gene_id GRMZM2G036916; transcript_id TCONS_00037827; exon_number 6; oId CUFF.32226.1; tss_id TSS34230;
7	Cufflinks	exon	103537712	103537960	.	-	.	gene_id GRMZM2G036916; transcript_id TCONS_00037827; exon_number 7; oId CUFF.32226.1; tss_id TSS34230;
7	Cufflinks	exon	103538090	103538318	.	-	.	gene_id GRMZM2G036916; transcript_id TCONS_00037827; exon_number 8; oId CUFF.32226.1; tss_id TSS34230;
7	Cufflinks	exon	103538700	103539199	.	-	.	gene_id GRMZM2G036916; transcript_id TCONS_00037827; exon_number 9; oId CUFF.32226.1; tss_id TSS34230;
7	Cufflinks	exon	108330462	108331560	.	-	.	gene_id XLOC_033185; transcript_id TCONS_00037863; exon_number 1; oId CUFF.32277.1; tss_id TSS34262;
7	Cufflinks	exon	108331687	108331800	.	-	.	gene_id XLOC_033185; transcript_id TCONS_00037863; exon_number 2; oId CUFF.32277.1; tss_id TSS34262;
7	Cufflinks	exon	108331891	108332092	.	-	.	gene_id XLOC_033185; transcript_id TCONS_00037863; exon_number 3; oId CUFF.32277.1; tss_id TSS34262;
7	Cufflinks	exon	108414550	108415148	.	-	.	gene_id XLOC_033186; transcript_id TCONS_00037864; exon_number 1; oId CUFF.32274.1; tss_id TSS34263;
7	Cufflinks	exon	108415252	108415771	.	-	.	gene_id XLOC_033186; transcript_id TCONS_00037864; exon_number 2; oId CUFF.32274.1; tss_id TSS34263;
7	Cufflinks	exon	108415874	108415987	.	-	.	gene_id XLOC_033186; transcript_id TCONS_00037864; exon_number 3; oId CUFF.32274.1; tss_id TSS34263;

7	Cufflinks	exon	108416197	108416494	.	-	.	gene_id XLOC_033186; transcript_id TCONS_00037864; exon_number 4; oId CUFF.32274.1; tss_id TSS34263;
7	Cufflinks	exon	109353827	109354997	.	-	.	gene_id GRMZM2G092525; transcript_id TCONS_00037876; exon_number 1; oId CUFF.32303.1; tss_id TSS34274;
7	Cufflinks	exon	109356590	109357542	.	-	.	gene_id GRMZM2G092525; transcript_id TCONS_00037876; exon_number 2; oId CUFF.32303.1; tss_id TSS34274;
7	Cufflinks	exon	110085353	110085924	.	-	.	gene_id GRMZM2G121200; transcript_id TCONS_00037879; exon_number 1; oId CUFF.32318.1; tss_id TSS34277;
7	Cufflinks	exon	110086728	110086811	.	-	.	gene_id GRMZM2G121200; transcript_id TCONS_00037879; exon_number 2; oId CUFF.32318.1; tss_id TSS34277;
7	Cufflinks	exon	110086978	110087048	.	-	.	gene_id GRMZM2G121200; transcript_id TCONS_00037879; exon_number 3; oId CUFF.32318.1; tss_id TSS34277;
7	Cufflinks	exon	110087128	110087215	.	-	.	gene_id GRMZM2G121200; transcript_id TCONS_00037879; exon_number 4; oId CUFF.32318.1; tss_id TSS34277;
7	Cufflinks	exon	110087327	110087518	.	-	.	gene_id GRMZM2G121200; transcript_id TCONS_00037879; exon_number 5; oId CUFF.32318.1; tss_id TSS34277;
7	Cufflinks	exon	110087602	110088614	.	-	.	gene_id GRMZM2G121200; transcript_id TCONS_00037879; exon_number 6; oId CUFF.32318.1; tss_id TSS34277;
7	Cufflinks	exon	115719658	115722055	.	-	.	gene_id GRMZM2G129018; transcript_id TCONS_00037907; exon_number 1; oId CUFF.32366.1; tss_id TSS34304;
7	Cufflinks	exon	124779178	124779599	.	-	.	gene_id GRMZM2G024865; transcript_id TCONS_00037981; exon_number 1; oId CUFF.32528.1; tss_id TSS34375;
7	Cufflinks	exon	124779694	124779761	.	-	.	gene_id GRMZM2G024865; transcript_id TCONS_00037981; exon_number 2; oId CUFF.32528.1; tss_id TSS34375;
7	Cufflinks	exon	124780131	124780217	.	-	.	gene_id GRMZM2G024865; transcript_id TCONS_00037981; exon_number 3; oId CUFF.32528.1; tss_id TSS34375;
7	Cufflinks	exon	124780339	124780407	.	-	.	gene_id GRMZM2G024865; transcript_id TCONS_00037981; exon_number 4; oId CUFF.32528.1; tss_id TSS34375;
7	Cufflinks	exon	124780491	124780559	.	-	.	gene_id GRMZM2G024865; transcript_id TCONS_00037981; exon_number 5; oId CUFF.32528.1; tss_id TSS34375;
7	Cufflinks	exon	124780664	124781786	.	-	.	gene_id GRMZM2G024865; transcript_id TCONS_00037981; exon_number 6; oId CUFF.32528.1; tss_id TSS34375;
7	Cufflinks	exon	128407526	128408178	.	-	.	gene_id GRMZM2G416625; transcript_id TCONS_00038004; exon_number 1; oId CUFF.32584.1; tss_id TSS34397;
7	Cufflinks	exon	128411222	128412102	.	-	.	gene_id GRMZM2G416625; transcript_id TCONS_00038004; exon_number 2; oId CUFF.32584.1; tss_id TSS34397;
7	Cufflinks	exon	128679183	128680735	.	-	.	gene_id GRMZM2G082653; transcript_id TCONS_00038009; exon_number 1; oId CUFF.32575.1; tss_id TSS34400;
7	Cufflinks	exon	134168847	134169179	.	-	.	gene_id XLOC_033407; transcript_id TCONS_00038100; exon_number 1; oId CUFF.32731.1; tss_id TSS34486;
7	Cufflinks	exon	134169261	134169418	.	-	.	gene_id XLOC_033407; transcript_id TCONS_00038100; exon_number 2; oId CUFF.32731.1; tss_id TSS34486;
7	Cufflinks	exon	135625837	135626563	.	-	.	gene_id GRMZM2G131281; transcript_id TCONS_00038110; exon_number 1; oId CUFF.32747.1; tss_id TSS34496;
7	Cufflinks	exon	135626659	135626999	.	-	.	gene_id GRMZM2G131281; transcript_id TCONS_00038110; exon_number 2; oId CUFF.32747.1; tss_id TSS34496;
7	Cufflinks	exon	140268676	140269704	.	-	.	gene_id GRMZM2G132486; transcript_id TCONS_00038157; exon_number 1; oId CUFF.32843.1; tss_id TSS34541;
7	Cufflinks	exon	140269983	140270227	.	-	.	gene_id GRMZM2G132486; transcript_id TCONS_00038157; exon_number 2; oId CUFF.32843.1; tss_id TSS34541;
7	Cufflinks	exon	140270392	140270591	.	-	.	gene_id GRMZM2G132486; transcript_id TCONS_00038157; exon_number 3; oId CUFF.32843.1; tss_id TSS34541;
7	Cufflinks	exon	140270697	140271906	.	-	.	gene_id GRMZM2G132486; transcript_id TCONS_00038157; exon_number 4; oId CUFF.32843.1; tss_id TSS34541;
7	Cufflinks	exon	140847928	140848482	.	-	.	gene_id GRMZM2G149520; transcript_id TCONS_00038164; exon_number 1; oId CUFF.32890.1; tss_id TSS34547;
7	Cufflinks	exon	140848824	140848957	.	-	.	gene_id GRMZM2G149520; transcript_id TCONS_00038164; exon_number 2; oId CUFF.32890.1; tss_id TSS34547;
7	Cufflinks	exon	140849030	140849124	.	-	.	gene_id GRMZM2G149520; transcript_id TCONS_00038164; exon_number 3; oId CUFF.32890.1; tss_id TSS34547;
7	Cufflinks	exon	140849211	140849307	.	-	.	gene_id GRMZM2G149520; transcript_id TCONS_00038164; exon_number 4; oId CUFF.32890.1; tss_id TSS34547;
7	Cufflinks	exon	140849395	140849566	.	-	.	gene_id GRMZM2G149520; transcript_id TCONS_00038164; exon_number 5; oId CUFF.32890.1; tss_id TSS34547;
7	Cufflinks	exon	140850018	140850078	.	-	.	gene_id GRMZM2G149520; transcript_id TCONS_00038164; exon_number 6; oId CUFF.32890.1; tss_id TSS34547;
7	Cufflinks	exon	140850164	140850265	.	-	.	gene_id GRMZM2G149520; transcript_id TCONS_00038164; exon_number 7; oId CUFF.32890.1; tss_id TSS34547;
7	Cufflinks	exon	140850594	140850705	.	-	.	gene_id GRMZM2G149520; transcript_id TCONS_00038164; exon_number 8; oId CUFF.32890.1; tss_id TSS34547;
7	Cufflinks	exon	140850786	140850940	.	-	.	gene_id GRMZM2G149520; transcript_id TCONS_00038164; exon_number 9; oId CUFF.32890.1; tss_id TSS34547;

7	Cufflinks	exon	140851028	140851098	.	-	.	gene_id GRMZM2G149520; transcript_id TCONS_00038164; exon_number 10; oId CUFF.32890.1; tss_id TSS34547;
7	Cufflinks	exon	140851328	140851439	.	-	.	gene_id GRMZM2G149520; transcript_id TCONS_00038164; exon_number 11; oId CUFF.32890.1; tss_id TSS34547;
7	Cufflinks	exon	140851526	140851666	.	-	.	gene_id GRMZM2G149520; transcript_id TCONS_00038164; exon_number 12; oId CUFF.32890.1; tss_id TSS34547;
7	Cufflinks	exon	140851757	140851891	.	-	.	gene_id GRMZM2G149520; transcript_id TCONS_00038164; exon_number 13; oId CUFF.32890.1; tss_id TSS34547;
7	Cufflinks	exon	140852356	140853032	.	-	.	gene_id GRMZM2G149520; transcript_id TCONS_00038164; exon_number 14; oId CUFF.32890.1; tss_id TSS34547;
7	Cufflinks	exon	141921207	141922185	.	-	.	gene_id GRMZM2G052509; transcript_id TCONS_00038188; exon_number 1; oId CUFF.32923.1; tss_id TSS34571;
7	Cufflinks	exon	141923341	141924166	.	-	.	gene_id GRMZM2G052509; transcript_id TCONS_00038188; exon_number 2; oId CUFF.32923.1; tss_id TSS34571;
7	Cufflinks	exon	150906366	150906852	.	-	.	gene_id XLOC_033625; transcript_id TCONS_00038341; exon_number 1; oId CUFF.33155.1; tss_id TSS34709;
7	Cufflinks	exon	151545591	151545949	.	-	.	gene_id GRMZM2G010298; transcript_id TCONS_00038355; exon_number 1; oId CUFF.33192.1; tss_id TSS34723;
7	Cufflinks	exon	151546181	151546335	.	-	.	gene_id GRMZM2G010298; transcript_id TCONS_00038355; exon_number 2; oId CUFF.33192.1; tss_id TSS34723;
7	Cufflinks	exon	151547441	151547726	.	-	.	gene_id GRMZM2G010298; transcript_id TCONS_00038355; exon_number 3; oId CUFF.33192.1; tss_id TSS34723;
7	Cufflinks	exon	151548228	151548479	.	-	.	gene_id GRMZM2G010298; transcript_id TCONS_00038355; exon_number 4; oId CUFF.33192.1; tss_id TSS34723;
7	Cufflinks	exon	151555276	151555845	.	-	.	gene_id GRMZM2G010298; transcript_id TCONS_00038355; exon_number 5; oId CUFF.33192.1; tss_id TSS34723;
7	Cufflinks	exon	155396211	155398094	.	-	.	gene_id GRMZM2G358467; transcript_id TCONS_00038418; exon_number 1; oId CUFF.33325.1; tss_id TSS34782;
7	Cufflinks	exon	156219834	156220357	.	-	.	gene_id GRMZM5G889999; transcript_id TCONS_00038434; exon_number 1; oId CUFF.33338.1; tss_id TSS34798;
7	Cufflinks	exon	156220476	156220626	.	-	.	gene_id GRMZM5G889999; transcript_id TCONS_00038434; exon_number 2; oId CUFF.33338.1; tss_id TSS34798;
7	Cufflinks	exon	156220705	156220942	.	-	.	gene_id GRMZM5G889999; transcript_id TCONS_00038434; exon_number 3; oId CUFF.33338.1; tss_id TSS34798;
7	Cufflinks	exon	156221031	156221241	.	-	.	gene_id GRMZM5G889999; transcript_id TCONS_00038434; exon_number 4; oId CUFF.33338.1; tss_id TSS34798;
7	Cufflinks	exon	156221339	156221559	.	-	.	gene_id GRMZM5G889999; transcript_id TCONS_00038434; exon_number 5; oId CUFF.33338.1; tss_id TSS34798;
7	Cufflinks	exon	156222009	156222116	.	-	.	gene_id GRMZM5G889999; transcript_id TCONS_00038434; exon_number 6; oId CUFF.33338.1; tss_id TSS34798;
7	Cufflinks	exon	156222226	156222555	.	-	.	gene_id GRMZM5G889999; transcript_id TCONS_00038434; exon_number 7; oId CUFF.33338.1; tss_id TSS34798;
7	Cufflinks	exon	156223040	156223350	.	-	.	gene_id GRMZM5G889999; transcript_id TCONS_00038434; exon_number 8; oId CUFF.33338.1; tss_id TSS34798;
7	Cufflinks	exon	156224828	156225721	.	-	.	gene_id GRMZM5G889999; transcript_id TCONS_00038434; exon_number 9; oId CUFF.33338.1; tss_id TSS34798;
7	Cufflinks	exon	157049353	157051304	.	-	.	gene_id GRMZM2G060993; transcript_id TCONS_00038447; exon_number 1; oId CUFF.33358.1; tss_id TSS34810;
7	Cufflinks	exon	157477093	157478805	.	-	.	gene_id GRMZM5G801949; transcript_id TCONS_00038459; exon_number 1; oId CUFF.33381.1; tss_id TSS34822;
7	Cufflinks	exon	157479343	157479679	.	-	.	gene_id GRMZM5G801949; transcript_id TCONS_00038459; exon_number 2; oId CUFF.33381.1; tss_id TSS34822;
7	Cufflinks	exon	159570727	159571366	.	-	.	gene_id XLOC_033774; transcript_id TCONS_00038504; exon_number 1; oId CUFF.33457.1; tss_id TSS34866;
7	Cufflinks	exon	159572151	159573245	.	-	.	gene_id XLOC_033774; transcript_id TCONS_00038504; exon_number 2; oId CUFF.33457.1; tss_id TSS34866;
7	Cufflinks	exon	161158692	161160144	.	-	.	gene_id XLOC_033803; transcript_id TCONS_00038536; exon_number 1; oId CUFF.33514.1; tss_id TSS34897;
7	Cufflinks	exon	161160236	161160676	.	-	.	gene_id XLOC_033803; transcript_id TCONS_00038536; exon_number 2; oId CUFF.33514.1; tss_id TSS34897;
7	Cufflinks	exon	165382432	165385059	.	-	.	gene_id GRMZM2G080549; transcript_id TCONS_00038618; exon_number 1; oId CUFF.33690.1; tss_id TSS34975;
7	Cufflinks	exon	165385159	165385218	.	-	.	gene_id GRMZM2G080549; transcript_id TCONS_00038618; exon_number 2; oId CUFF.33690.1; tss_id TSS34975;
7	Cufflinks	exon	165385977	165386072	.	-	.	gene_id GRMZM2G080549; transcript_id TCONS_00038618; exon_number 3; oId CUFF.33690.1; tss_id TSS34975;
7	Cufflinks	exon	165386756	165386841	.	-	.	gene_id GRMZM2G080549; transcript_id TCONS_00038618; exon_number 4; oId CUFF.33690.1; tss_id TSS34975;
7	Cufflinks	exon	165386991	165387049	.	-	.	gene_id GRMZM2G080549; transcript_id TCONS_00038618; exon_number 5; oId CUFF.33690.1; tss_id TSS34975;
7	Cufflinks	exon	165387491	165387587	.	-	.	gene_id GRMZM2G080549; transcript_id TCONS_00038618; exon_number 6; oId CUFF.33690.1; tss_id TSS34975;

7	Cufflinks	exon	165388818	165388899	.	-	.	gene_id GRMZM2G080549; transcript_id TCONS_00038618; exon_number 7; oId CUFF.33690.1; tss_id TSS34975;
7	Cufflinks	exon	165389006	165389115	.	-	.	gene_id GRMZM2G080549; transcript_id TCONS_00038618; exon_number 8; oId CUFF.33690.1; tss_id TSS34975;
7	Cufflinks	exon	165389222	165389417	.	-	.	gene_id GRMZM2G080549; transcript_id TCONS_00038618; exon_number 9; oId CUFF.33690.1; tss_id TSS34975;
7	Cufflinks	exon	166186897	166187412	.	-	.	gene_id GRMZM2G079226; transcript_id TCONS_00038636; exon_number 1; oId CUFF.33711.1; tss_id TSS34991;
7	Cufflinks	exon	166189170	166189273	.	-	.	gene_id GRMZM2G079226; transcript_id TCONS_00038636; exon_number 2; oId CUFF.33711.1; tss_id TSS34991;
7	Cufflinks	exon	166189801	166189862	.	-	.	gene_id GRMZM2G079226; transcript_id TCONS_00038636; exon_number 3; oId CUFF.33711.1; tss_id TSS34991;
7	Cufflinks	exon	166190039	166190147	.	-	.	gene_id GRMZM2G079226; transcript_id TCONS_00038636; exon_number 4; oId CUFF.33711.1; tss_id TSS34991;
7	Cufflinks	exon	166190693	166190833	.	-	.	gene_id GRMZM2G079226; transcript_id TCONS_00038636; exon_number 5; oId CUFF.33711.1; tss_id TSS34991;
7	Cufflinks	exon	166190977	166191116	.	-	.	gene_id GRMZM2G079226; transcript_id TCONS_00038636; exon_number 6; oId CUFF.33711.1; tss_id TSS34991;
7	Cufflinks	exon	166191435	166191764	.	-	.	gene_id GRMZM2G079226; transcript_id TCONS_00038636; exon_number 7; oId CUFF.33711.1; tss_id TSS34991;
7	Cufflinks	exon	167541463	167542348	.	-	.	gene_id GRMZM2G017145; transcript_id TCONS_00038662; exon_number 1; oId CUFF.33759.1; tss_id TSS35015;
7	Cufflinks	exon	167542712	167542839	.	-	.	gene_id GRMZM2G017145; transcript_id TCONS_00038662; exon_number 2; oId CUFF.33759.1; tss_id TSS35015;
7	Cufflinks	exon	167543249	167543367	.	-	.	gene_id GRMZM2G017145; transcript_id TCONS_00038662; exon_number 3; oId CUFF.33759.1; tss_id TSS35015;
7	Cufflinks	exon	167543903	167544005	.	-	.	gene_id GRMZM2G017145; transcript_id TCONS_00038662; exon_number 4; oId CUFF.33759.1; tss_id TSS35015;
7	Cufflinks	exon	167544122	167544286	.	-	.	gene_id GRMZM2G017145; transcript_id TCONS_00038662; exon_number 5; oId CUFF.33759.1; tss_id TSS35015;
7	Cufflinks	exon	167674835	167675315	.	-	.	gene_id XLOC_033929; transcript_id TCONS_00038671; exon_number 1; oId CUFF.33760.1; tss_id TSS35024;
7	Cufflinks	exon	168441795	168442008	.	-	.	gene_id XLOC_033944; transcript_id TCONS_00038689; exon_number 1; oId CUFF.33796.1; tss_id TSS35041;
7	Cufflinks	exon	168442120	168442543	.	-	.	gene_id XLOC_033944; transcript_id TCONS_00038689; exon_number 2; oId CUFF.33796.1; tss_id TSS35041;
7	Cufflinks	exon	169977821	169979383	.	-	.	gene_id GRMZM2G301934; transcript_id TCONS_00038715; exon_number 1; oId CUFF.33850.1; tss_id TSS35066;
7	Cufflinks	exon	170074356	170075064	.	-	.	gene_id GRMZM2G356894; transcript_id TCONS_00038719; exon_number 1; oId CUFF.33888.1; tss_id TSS35070;
7	Cufflinks	exon	170075154	170075402	.	-	.	gene_id GRMZM2G356894; transcript_id TCONS_00038719; exon_number 2; oId CUFF.33888.1; tss_id TSS35070;
7	Cufflinks	exon	170076054	170076254	.	-	.	gene_id GRMZM2G356894; transcript_id TCONS_00038719; exon_number 3; oId CUFF.33888.1; tss_id TSS35070;
7	Cufflinks	exon	170076716	170076886	.	-	.	gene_id GRMZM2G356894; transcript_id TCONS_00038719; exon_number 4; oId CUFF.33888.1; tss_id TSS35070;
7	Cufflinks	exon	170076984	170077168	.	-	.	gene_id GRMZM2G356894; transcript_id TCONS_00038719; exon_number 5; oId CUFF.33888.1; tss_id TSS35070;
7	Cufflinks	exon	170077255	170077580	.	-	.	gene_id GRMZM2G356894; transcript_id TCONS_00038719; exon_number 6; oId CUFF.33888.1; tss_id TSS35070;
7	Cufflinks	exon	170078479	170080041	.	-	.	gene_id GRMZM2G356894; transcript_id TCONS_00038719; exon_number 7; oId CUFF.33888.1; tss_id TSS35070;
7	Cufflinks	exon	170696934	170697438	.	-	.	gene_id GRMZM2G433767; transcript_id TCONS_00038735; exon_number 1; oId CUFF.33894.1; tss_id TSS35084;
7	Cufflinks	exon	170697581	170697686	.	-	.	gene_id GRMZM2G433767; transcript_id TCONS_00038735; exon_number 2; oId CUFF.33894.1; tss_id TSS35084;
7	Cufflinks	exon	170697803	170697895	.	-	.	gene_id GRMZM2G433767; transcript_id TCONS_00038735; exon_number 3; oId CUFF.33894.1; tss_id TSS35084;
7	Cufflinks	exon	170698015	170698133	.	-	.	gene_id GRMZM2G433767; transcript_id TCONS_00038735; exon_number 4; oId CUFF.33894.1; tss_id TSS35084;
7	Cufflinks	exon	170698226	170698498	.	-	.	gene_id GRMZM2G433767; transcript_id TCONS_00038735; exon_number 5; oId CUFF.33894.1; tss_id TSS35084;
7	Cufflinks	exon	170698641	170699032	.	-	.	gene_id GRMZM2G433767; transcript_id TCONS_00038735; exon_number 6; oId CUFF.33894.1; tss_id TSS35084;
7	Cufflinks	exon	170699198	170699642	.	-	.	gene_id GRMZM2G433767; transcript_id TCONS_00038735; exon_number 7; oId CUFF.33894.1; tss_id TSS35084;
7	Cufflinks	exon	171577707	171578957	.	-	.	gene_id GRMZM2G037189; transcript_id TCONS_00038753; exon_number 1; oId CUFF.33918.1; tss_id TSS35102;
7	Cufflinks	exon	171775066	171775374	.	-	.	gene_id GRMZM2G025992; transcript_id TCONS_00038759; exon_number 1; oId CUFF.33949.1; tss_id TSS35108;
7	Cufflinks	exon	171775554	171775607	.	-	.	gene_id GRMZM2G025992; transcript_id TCONS_00038759; exon_number 2; oId CUFF.33949.1; tss_id TSS35108;

7	Cufflinks	exon	171776633	171776708	.	-	.	gene_id GRMZM2G025992; transcript_id TCONS_00038759; exon_number 3; oId CUFF.33949.1; tss_id TSS35108;
7	Cufflinks	exon	171776819	171776850	.	-	.	gene_id GRMZM2G025992; transcript_id TCONS_00038759; exon_number 4; oId CUFF.33949.1; tss_id TSS35108;
7	Cufflinks	exon	171777023	171777118	.	-	.	gene_id GRMZM2G025992; transcript_id TCONS_00038759; exon_number 5; oId CUFF.33949.1; tss_id TSS35108;
7	Cufflinks	exon	171777194	171777295	.	-	.	gene_id GRMZM2G025992; transcript_id TCONS_00038759; exon_number 6; oId CUFF.33949.1; tss_id TSS35108;
7	Cufflinks	exon	171777371	171777455	.	-	.	gene_id GRMZM2G025992; transcript_id TCONS_00038759; exon_number 7; oId CUFF.33949.1; tss_id TSS35108;
7	Cufflinks	exon	171777959	171778156	.	-	.	gene_id GRMZM2G025992; transcript_id TCONS_00038759; exon_number 8; oId CUFF.33949.1; tss_id TSS35108;
7	Cufflinks	exon	171775066	171775374	.	-	.	gene_id GRMZM2G025992; transcript_id TCONS_00038760; exon_number 1; oId CUFF.33949.2; tss_id TSS35108;
7	Cufflinks	exon	171775554	171775607	.	-	.	gene_id GRMZM2G025992; transcript_id TCONS_00038760; exon_number 2; oId CUFF.33949.2; tss_id TSS35108;
7	Cufflinks	exon	171776633	171776708	.	-	.	gene_id GRMZM2G025992; transcript_id TCONS_00038760; exon_number 3; oId CUFF.33949.2; tss_id TSS35108;
7	Cufflinks	exon	171776819	171776850	.	-	.	gene_id GRMZM2G025992; transcript_id TCONS_00038760; exon_number 4; oId CUFF.33949.2; tss_id TSS35108;
7	Cufflinks	exon	171777023	171777118	.	-	.	gene_id GRMZM2G025992; transcript_id TCONS_00038760; exon_number 5; oId CUFF.33949.2; tss_id TSS35108;
7	Cufflinks	exon	171777194	171777295	.	-	.	gene_id GRMZM2G025992; transcript_id TCONS_00038760; exon_number 6; oId CUFF.33949.2; tss_id TSS35108;
7	Cufflinks	exon	171777371	171777455	.	-	.	gene_id GRMZM2G025992; transcript_id TCONS_00038760; exon_number 7; oId CUFF.33949.2; tss_id TSS35108;
7	Cufflinks	exon	171778026	171778176	.	-	.	gene_id GRMZM2G025992; transcript_id TCONS_00038760; exon_number 8; oId CUFF.33949.2; tss_id TSS35108;
7	Cufflinks	exon	172301215	172302771	.	-	.	gene_id GRMZM2G020766; transcript_id TCONS_00038771; exon_number 1; oId CUFF.33964.1; tss_id TSS35118;
7	Cufflinks	exon	172303326	172303580	.	-	.	gene_id GRMZM2G020766; transcript_id TCONS_00038771; exon_number 2; oId CUFF.33964.1; tss_id TSS35118;
7	Cufflinks	exon	172303678	172303792	.	-	.	gene_id GRMZM2G020766; transcript_id TCONS_00038771; exon_number 3; oId CUFF.33964.1; tss_id TSS35118;
7	Cufflinks	exon	172303878	172303930	.	-	.	gene_id GRMZM2G020766; transcript_id TCONS_00038771; exon_number 4; oId CUFF.33964.1; tss_id TSS35118;
7	Cufflinks	exon	172304008	172304268	.	-	.	gene_id GRMZM2G020766; transcript_id TCONS_00038771; exon_number 5; oId CUFF.33964.1; tss_id TSS35118;
7	Cufflinks	exon	172310535	172310588	.	-	.	gene_id GRMZM2G020766; transcript_id TCONS_00038771; exon_number 6; oId CUFF.33964.1; tss_id TSS35118;
7	Cufflinks	exon	172310681	172310926	.	-	.	gene_id GRMZM2G020766; transcript_id TCONS_00038771; exon_number 7; oId CUFF.33964.1; tss_id TSS35118;
7	Cufflinks	exon	172311227	172311470	.	-	.	gene_id GRMZM2G020766; transcript_id TCONS_00038771; exon_number 8; oId CUFF.33964.1; tss_id TSS35118;
7	Cufflinks	exon	172312348	172312681	.	-	.	gene_id GRMZM2G020766; transcript_id TCONS_00038771; exon_number 9; oId CUFF.33964.1; tss_id TSS35118;
7	Cufflinks	exon	172543068	172543892	.	-	.	gene_id GRMZM2G414460; transcript_id TCONS_00038776; exon_number 1; oId CUFF.33981.1; tss_id TSS35123;
7	Cufflinks	exon	172543988	172544802	.	-	.	gene_id GRMZM2G414460; transcript_id TCONS_00038776; exon_number 2; oId CUFF.33981.1; tss_id TSS35123;
7	Cufflinks	exon	172545617	172545718	.	-	.	gene_id GRMZM2G414460; transcript_id TCONS_00038776; exon_number 3; oId CUFF.33981.1; tss_id TSS35123;
7	Cufflinks	exon	172546921	172547171	.	-	.	gene_id GRMZM2G414460; transcript_id TCONS_00038776; exon_number 4; oId CUFF.33981.1; tss_id TSS35123;
7	Cufflinks	exon	172810282	172810699	.	-	.	gene_id XLOC_034040; transcript_id TCONS_00038795; exon_number 1; oId CUFF.33999.1; tss_id TSS35142;
7	Cufflinks	exon	172977273	172978903	.	-	.	gene_id GRMZM2G166738; transcript_id TCONS_00038800; exon_number 1; oId CUFF.34011.1; tss_id TSS35146;
7	Cufflinks	exon	172978996	172979250	.	-	.	gene_id GRMZM2G166738; transcript_id TCONS_00038800; exon_number 2; oId CUFF.34011.1; tss_id TSS35146;
7	Cufflinks	exon	172979345	172979468	.	-	.	gene_id GRMZM2G166738; transcript_id TCONS_00038800; exon_number 3; oId CUFF.34011.1; tss_id TSS35146;
7	Cufflinks	exon	172979592	172979644	.	-	.	gene_id GRMZM2G166738; transcript_id TCONS_00038800; exon_number 4; oId CUFF.34011.1; tss_id TSS35146;
7	Cufflinks	exon	172979787	172980047	.	-	.	gene_id GRMZM2G166738; transcript_id TCONS_00038800; exon_number 5; oId CUFF.34011.1; tss_id TSS35146;
7	Cufflinks	exon	172980259	172980312	.	-	.	gene_id GRMZM2G166738; transcript_id TCONS_00038800; exon_number 6; oId CUFF.34011.1; tss_id TSS35146;
7	Cufflinks	exon	172980404	172980652	.	-	.	gene_id GRMZM2G166738; transcript_id TCONS_00038800; exon_number 7; oId CUFF.34011.1; tss_id TSS35146;
7	Cufflinks	exon	172980747	172980984	.	-	.	gene_id GRMZM2G166738; transcript_id TCONS_00038800; exon_number 8; oId CUFF.34011.1; tss_id TSS35146;

7	Cufflinks	exon	172981093	172981385	.	-	.	gene_id GRMZM2G166738; transcript_id TCONS_00038800; exon_number 9; oId CUFF.34011.1; tss_id TSS35146;
7	Cufflinks	exon	173556079	173558212	.	-	.	gene_id GRMZM2G430849; transcript_id TCONS_00038819; exon_number 1; oId CUFF.34047.2; tss_id TSS35162;
7	Cufflinks	exon	173558305	173558510	.	-	.	gene_id GRMZM2G430849; transcript_id TCONS_00038819; exon_number 2; oId CUFF.34047.2; tss_id TSS35162;
7	Cufflinks	exon	173558618	173558804	.	-	.	gene_id GRMZM2G430849; transcript_id TCONS_00038819; exon_number 3; oId CUFF.34047.2; tss_id TSS35162;
7	Cufflinks	exon	173556079	173557268	.	-	.	gene_id GRMZM2G430849; transcript_id TCONS_00038818; exon_number 1; oId CUFF.34047.1; tss_id TSS35162;
7	Cufflinks	exon	173557896	173558212	.	-	.	gene_id GRMZM2G430849; transcript_id TCONS_00038818; exon_number 2; oId CUFF.34047.1; tss_id TSS35162;
7	Cufflinks	exon	173558305	173558510	.	-	.	gene_id GRMZM2G430849; transcript_id TCONS_00038818; exon_number 3; oId CUFF.34047.1; tss_id TSS35162;
7	Cufflinks	exon	173558618	173558804	.	-	.	gene_id GRMZM2G430849; transcript_id TCONS_00038818; exon_number 4; oId CUFF.34047.1; tss_id TSS35162;
8	Cufflinks	exon	1146664	1147130	.	+	.	gene_id XLOC_034134; transcript_id TCONS_00038904; exon_number 1; oId CUFF.34183.1; tss_id TSS35239;
8	Cufflinks	exon	2511344	2511526	.	+	.	gene_id GRMZM2G589579; transcript_id TCONS_00038922; exon_number 1; oId CUFF.34235.1; tss_id TSS35255;
8	Cufflinks	exon	2512519	2512564	.	+	.	gene_id GRMZM2G589579; transcript_id TCONS_00038922; exon_number 2; oId CUFF.34235.1; tss_id TSS35255;
8	Cufflinks	exon	2513373	2513604	.	+	.	gene_id GRMZM2G589579; transcript_id TCONS_00038922; exon_number 3; oId CUFF.34235.1; tss_id TSS35255;
8	Cufflinks	exon	2513693	2513898	.	+	.	gene_id GRMZM2G589579; transcript_id TCONS_00038922; exon_number 4; oId CUFF.34235.1; tss_id TSS35255;
8	Cufflinks	exon	2513988	2514209	.	+	.	gene_id GRMZM2G589579; transcript_id TCONS_00038922; exon_number 5; oId CUFF.34235.1; tss_id TSS35255;
8	Cufflinks	exon	2514326	2514462	.	+	.	gene_id GRMZM2G589579; transcript_id TCONS_00038922; exon_number 6; oId CUFF.34235.1; tss_id TSS35255;
8	Cufflinks	exon	2514563	2514660	.	+	.	gene_id GRMZM2G589579; transcript_id TCONS_00038922; exon_number 7; oId CUFF.34235.1; tss_id TSS35255;
8	Cufflinks	exon	2514764	2514861	.	+	.	gene_id GRMZM2G589579; transcript_id TCONS_00038922; exon_number 8; oId CUFF.34235.1; tss_id TSS35255;
8	Cufflinks	exon	2514998	2515148	.	+	.	gene_id GRMZM2G589579; transcript_id TCONS_00038922; exon_number 9; oId CUFF.34235.1; tss_id TSS35255;
8	Cufflinks	exon	2515241	2515357	.	+	.	gene_id GRMZM2G589579; transcript_id TCONS_00038922; exon_number 10; oId CUFF.34235.1; tss_id TSS35255;
8	Cufflinks	exon	2515500	2515607	.	+	.	gene_id GRMZM2G589579; transcript_id TCONS_00038922; exon_number 11; oId CUFF.34235.1; tss_id TSS35255;
8	Cufflinks	exon	2515700	2515762	.	+	.	gene_id GRMZM2G589579; transcript_id TCONS_00038922; exon_number 12; oId CUFF.34235.1; tss_id TSS35255;
8	Cufflinks	exon	2515844	2515957	.	+	.	gene_id GRMZM2G589579; transcript_id TCONS_00038922; exon_number 13; oId CUFF.34235.1; tss_id TSS35255;
8	Cufflinks	exon	2516062	2516227	.	+	.	gene_id GRMZM2G589579; transcript_id TCONS_00038922; exon_number 14; oId CUFF.34235.1; tss_id TSS35255;
8	Cufflinks	exon	2516309	2516421	.	+	.	gene_id GRMZM2G589579; transcript_id TCONS_00038922; exon_number 15; oId CUFF.34235.1; tss_id TSS35255;
8	Cufflinks	exon	2516505	2516642	.	+	.	gene_id GRMZM2G589579; transcript_id TCONS_00038922; exon_number 16; oId CUFF.34235.1; tss_id TSS35255;
8	Cufflinks	exon	2516719	2516846	.	+	.	gene_id GRMZM2G589579; transcript_id TCONS_00038922; exon_number 17; oId CUFF.34235.1; tss_id TSS35255;
8	Cufflinks	exon	2516960	2517025	.	+	.	gene_id GRMZM2G589579; transcript_id TCONS_00038922; exon_number 18; oId CUFF.34235.1; tss_id TSS35255;
8	Cufflinks	exon	2517169	2517232	.	+	.	gene_id GRMZM2G589579; transcript_id TCONS_00038922; exon_number 19; oId CUFF.34235.1; tss_id TSS35255;
8	Cufflinks	exon	2517357	2517468	.	+	.	gene_id GRMZM2G589579; transcript_id TCONS_00038922; exon_number 20; oId CUFF.34235.1; tss_id TSS35255;
8	Cufflinks	exon	2517569	2517642	.	+	.	gene_id GRMZM2G589579; transcript_id TCONS_00038922; exon_number 21; oId CUFF.34235.1; tss_id TSS35255;
8	Cufflinks	exon	2518015	2518109	.	+	.	gene_id GRMZM2G589579; transcript_id TCONS_00038922; exon_number 22; oId CUFF.34235.1; tss_id TSS35255;
8	Cufflinks	exon	2518213	2518244	.	+	.	gene_id GRMZM2G589579; transcript_id TCONS_00038922; exon_number 23; oId CUFF.34235.1; tss_id TSS35255;
8	Cufflinks	exon	2518335	2518808	.	+	.	gene_id GRMZM2G589579; transcript_id TCONS_00038922; exon_number 24; oId CUFF.34235.1; tss_id TSS35255;
8	Cufflinks	exon	11486497	11488203	.	+	.	gene_id XLOC_034286; transcript_id TCONS_00039082; exon_number 1; oId CUFF.34448.1; tss_id TSS35395;
8	Cufflinks	exon	14796888	14797534	.	+	.	gene_id GRMZM2G139600; transcript_id TCONS_00039144; exon_number 1; oId CUFF.34537.1; tss_id TSS35449;
8	Cufflinks	exon	14797645	14797880	.	+	.	gene_id GRMZM2G139600; transcript_id TCONS_00039144; exon_number 2; oId CUFF.34537.1; tss_id TSS35449;

8	Cufflinks	exon	14797983	14798247	.	+	.	gene_id GRMZM2G139600; transcript_id TCONS_00039144; exon_number 3; oId CUFF.34537.1; tss_id TSS35449;
8	Cufflinks	exon	14798383	14798520	.	+	.	gene_id GRMZM2G139600; transcript_id TCONS_00039144; exon_number 4; oId CUFF.34537.1; tss_id TSS35449;
8	Cufflinks	exon	14798622	14799245	.	+	.	gene_id GRMZM2G139600; transcript_id TCONS_00039144; exon_number 5; oId CUFF.34537.1; tss_id TSS35449;
8	Cufflinks	exon	20696323	20696578	.	+	.	gene_id GRMZM2G080588; transcript_id TCONS_00039212; exon_number 1; oId CUFF.34709.1; tss_id TSS35514;
8	Cufflinks	exon	20697653	20697763	.	+	.	gene_id GRMZM2G080588; transcript_id TCONS_00039212; exon_number 2; oId CUFF.34709.1; tss_id TSS35514;
8	Cufflinks	exon	20701649	20701795	.	+	.	gene_id GRMZM2G080588; transcript_id TCONS_00039212; exon_number 3; oId CUFF.34709.1; tss_id TSS35514;
8	Cufflinks	exon	20701891	20701986	.	+	.	gene_id GRMZM2G080588; transcript_id TCONS_00039212; exon_number 4; oId CUFF.34709.1; tss_id TSS35514;
8	Cufflinks	exon	20702061	20702156	.	+	.	gene_id GRMZM2G080588; transcript_id TCONS_00039212; exon_number 5; oId CUFF.34709.1; tss_id TSS35514;
8	Cufflinks	exon	20702271	20702367	.	+	.	gene_id GRMZM2G080588; transcript_id TCONS_00039212; exon_number 6; oId CUFF.34709.1; tss_id TSS35514;
8	Cufflinks	exon	20702482	20702599	.	+	.	gene_id GRMZM2G080588; transcript_id TCONS_00039212; exon_number 7; oId CUFF.34709.1; tss_id TSS35514;
8	Cufflinks	exon	20702909	20702981	.	+	.	gene_id GRMZM2G080588; transcript_id TCONS_00039212; exon_number 8; oId CUFF.34709.1; tss_id TSS35514;
8	Cufflinks	exon	20703059	20703544	.	+	.	gene_id GRMZM2G080588; transcript_id TCONS_00039212; exon_number 9; oId CUFF.34709.1; tss_id TSS35514;
8	Cufflinks	exon	23892754	23895063	.	+	.	gene_id GRMZM2G099483; transcript_id TCONS_00039248; exon_number 1; oId CUFF.34757.1; tss_id TSS35548;
8	Cufflinks	exon	27670562	27671633	.	+	.	gene_id GRMZM2G014580; transcript_id TCONS_00039286; exon_number 1; oId CUFF.34837.1; tss_id TSS35581;
8	Cufflinks	exon	27671727	27672733	.	+	.	gene_id GRMZM2G014580; transcript_id TCONS_00039286; exon_number 2; oId CUFF.34837.1; tss_id TSS35581;
8	Cufflinks	exon	52818656	52820059	.	+	.	gene_id XLOC_034566; transcript_id TCONS_00039393; exon_number 1; oId CUFF.35033.1; tss_id TSS35680;
8	Cufflinks	exon	55520438	55520785	.	+	.	gene_id GRMZM6G514393; transcript_id TCONS_00039405; exon_number 1; oId CUFF.35058.1; tss_id TSS35692;
8	Cufflinks	exon	55521384	55522327	.	+	.	gene_id GRMZM6G514393; transcript_id TCONS_00039405; exon_number 2; oId CUFF.35058.1; tss_id TSS35692;
8	Cufflinks	exon	69651928	69652291	.	+	.	gene_id GRMZM2G031827; transcript_id TCONS_00039517; exon_number 1; oId CUFF.35239.1; tss_id TSS35790;
8	Cufflinks	exon	69659375	69660583	.	+	.	gene_id GRMZM2G031827; transcript_id TCONS_00039517; exon_number 2; oId CUFF.35239.1; tss_id TSS35790;
8	Cufflinks	exon	74456822	74457598	.	+	.	gene_id XLOC_034712; transcript_id TCONS_00039568; exon_number 1; oId CUFF.35322.1; tss_id TSS35832;
8	Cufflinks	exon	86549083	86549404	.	+	.	gene_id GRMZM2G306851; transcript_id TCONS_00039661; exon_number 1; oId CUFF.35497.2; tss_id TSS35914;
8	Cufflinks	exon	86549585	86549684	.	+	.	gene_id GRMZM2G306851; transcript_id TCONS_00039661; exon_number 2; oId CUFF.35497.2; tss_id TSS35914;
8	Cufflinks	exon	86549888	86549985	.	+	.	gene_id GRMZM2G306851; transcript_id TCONS_00039661; exon_number 3; oId CUFF.35497.2; tss_id TSS35914;
8	Cufflinks	exon	86554078	86554224	.	+	.	gene_id GRMZM2G306851; transcript_id TCONS_00039661; exon_number 4; oId CUFF.35497.2; tss_id TSS35914;
8	Cufflinks	exon	86554313	86554443	.	+	.	gene_id GRMZM2G306851; transcript_id TCONS_00039661; exon_number 5; oId CUFF.35497.2; tss_id TSS35914;
8	Cufflinks	exon	86554511	86554648	.	+	.	gene_id GRMZM2G306851; transcript_id TCONS_00039661; exon_number 6; oId CUFF.35497.2; tss_id TSS35914;
8	Cufflinks	exon	86554742	86554840	.	+	.	gene_id GRMZM2G306851; transcript_id TCONS_00039661; exon_number 7; oId CUFF.35497.2; tss_id TSS35914;
8	Cufflinks	exon	86554953	86555756	.	+	.	gene_id GRMZM2G306851; transcript_id TCONS_00039661; exon_number 8; oId CUFF.35497.2; tss_id TSS35914;
8	Cufflinks	exon	93946368	93946636	.	+	.	gene_id GRMZM2G023755; transcript_id TCONS_00039712; exon_number 1; oId CUFF.35569.1; tss_id TSS35965;
8	Cufflinks	exon	93946750	93946821	.	+	.	gene_id GRMZM2G023755; transcript_id TCONS_00039712; exon_number 2; oId CUFF.35569.1; tss_id TSS35965;
8	Cufflinks	exon	93947273	93947340	.	+	.	gene_id GRMZM2G023755; transcript_id TCONS_00039712; exon_number 3; oId CUFF.35569.1; tss_id TSS35965;
8	Cufflinks	exon	93948058	93948178	.	+	.	gene_id GRMZM2G023755; transcript_id TCONS_00039712; exon_number 4; oId CUFF.35569.1; tss_id TSS35965;
8	Cufflinks	exon	93948274	93948357	.	+	.	gene_id GRMZM2G023755; transcript_id TCONS_00039712; exon_number 5; oId CUFF.35569.1; tss_id TSS35965;
8	Cufflinks	exon	93948455	93948565	.	+	.	gene_id GRMZM2G023755; transcript_id TCONS_00039712; exon_number 6; oId CUFF.35569.1; tss_id TSS35965;
8	Cufflinks	exon	93948666	93949244	.	+	.	gene_id GRMZM2G023755; transcript_id TCONS_00039712; exon_number 7; oId CUFF.35569.1; tss_id TSS35965;

8	Cufflinks	exon	106153653	106154246	.	+	.	gene_id GRMZM2G016605; transcript_id TCONS_00039832; exon_number 1; oId CUFF.35783.1; tss_id TSS36075;
8	Cufflinks	exon	106154823	106156495	.	+	.	gene_id GRMZM2G016605; transcript_id TCONS_00039832; exon_number 2; oId CUFF.35783.1; tss_id TSS36075;
8	Cufflinks	exon	119544238	119544607	.	+	.	gene_id XLOC_035083; transcript_id TCONS_00039981; exon_number 1; oId CUFF.36047.1; tss_id TSS36210;
8	Cufflinks	exon	131576700	131577925	.	+	.	gene_id GRMZM2G700665; transcript_id TCONS_00040135; exon_number 1; oId CUFF.36327.1; tss_id TSS36352;
8	Cufflinks	exon	131578034	131578059	.	+	.	gene_id GRMZM2G700665; transcript_id TCONS_00040135; exon_number 2; oId CUFF.36327.1; tss_id TSS36352;
8	Cufflinks	exon	131578187	131578217	.	+	.	gene_id GRMZM2G700665; transcript_id TCONS_00040135; exon_number 3; oId CUFF.36327.1; tss_id TSS36352;
8	Cufflinks	exon	131578311	131578544	.	+	.	gene_id GRMZM2G700665; transcript_id TCONS_00040135; exon_number 4; oId CUFF.36327.1; tss_id TSS36352;
8	Cufflinks	exon	131578987	131579078	.	+	.	gene_id GRMZM2G700665; transcript_id TCONS_00040135; exon_number 5; oId CUFF.36327.1; tss_id TSS36352;
8	Cufflinks	exon	131579161	131579282	.	+	.	gene_id GRMZM2G700665; transcript_id TCONS_00040135; exon_number 6; oId CUFF.36327.1; tss_id TSS36352;
8	Cufflinks	exon	131579440	131579562	.	+	.	gene_id GRMZM2G700665; transcript_id TCONS_00040135; exon_number 7; oId CUFF.36327.1; tss_id TSS36352;
8	Cufflinks	exon	131579905	131580312	.	+	.	gene_id GRMZM2G700665; transcript_id TCONS_00040135; exon_number 8; oId CUFF.36327.1; tss_id TSS36352;
8	Cufflinks	exon	131727361	131728157	.	+	.	gene_id GRMZM2G474726; transcript_id TCONS_00040139; exon_number 1; oId CUFF.36345.2; tss_id TSS36354;
8	Cufflinks	exon	131728484	131728596	.	+	.	gene_id GRMZM2G474726; transcript_id TCONS_00040139; exon_number 2; oId CUFF.36345.2; tss_id TSS36354;
8	Cufflinks	exon	131728696	131728759	.	+	.	gene_id GRMZM2G474726; transcript_id TCONS_00040139; exon_number 3; oId CUFF.36345.2; tss_id TSS36354;
8	Cufflinks	exon	131729164	131729251	.	+	.	gene_id GRMZM2G474726; transcript_id TCONS_00040139; exon_number 4; oId CUFF.36345.2; tss_id TSS36354;
8	Cufflinks	exon	131729334	131729414	.	+	.	gene_id GRMZM2G474726; transcript_id TCONS_00040139; exon_number 5; oId CUFF.36345.2; tss_id TSS36354;
8	Cufflinks	exon	131729498	131729554	.	+	.	gene_id GRMZM2G474726; transcript_id TCONS_00040139; exon_number 6; oId CUFF.36345.2; tss_id TSS36354;
8	Cufflinks	exon	131730606	131730687	.	+	.	gene_id GRMZM2G474726; transcript_id TCONS_00040139; exon_number 7; oId CUFF.36345.2; tss_id TSS36354;
8	Cufflinks	exon	131730911	131730960	.	+	.	gene_id GRMZM2G474726; transcript_id TCONS_00040139; exon_number 8; oId CUFF.36345.2; tss_id TSS36354;
8	Cufflinks	exon	131731209	131731265	.	+	.	gene_id GRMZM2G474726; transcript_id TCONS_00040139; exon_number 9; oId CUFF.36345.2; tss_id TSS36354;
8	Cufflinks	exon	131731355	131731433	.	+	.	gene_id GRMZM2G474726; transcript_id TCONS_00040139; exon_number 10; oId CUFF.36345.2; tss_id TSS36354;
8	Cufflinks	exon	131731599	131731657	.	+	.	gene_id GRMZM2G474726; transcript_id TCONS_00040139; exon_number 11; oId CUFF.36345.2; tss_id TSS36354;
8	Cufflinks	exon	131731969	131732026	.	+	.	gene_id GRMZM2G474726; transcript_id TCONS_00040139; exon_number 12; oId CUFF.36345.2; tss_id TSS36354;
8	Cufflinks	exon	131732167	131732295	.	+	.	gene_id GRMZM2G474726; transcript_id TCONS_00040139; exon_number 13; oId CUFF.36345.2; tss_id TSS36354;
8	Cufflinks	exon	131733185	131733498	.	+	.	gene_id GRMZM2G474726; transcript_id TCONS_00040139; exon_number 14; oId CUFF.36345.2; tss_id TSS36354;
8	Cufflinks	exon	134376621	134376831	.	+	.	gene_id GRMZM2G470513; transcript_id TCONS_00040180; exon_number 1; oId CUFF.36382.1; tss_id TSS36388;
8	Cufflinks	exon	134376961	134377099	.	+	.	gene_id GRMZM2G470513; transcript_id TCONS_00040180; exon_number 2; oId CUFF.36382.1; tss_id TSS36388;
8	Cufflinks	exon	134377194	134377244	.	+	.	gene_id GRMZM2G470513; transcript_id TCONS_00040180; exon_number 3; oId CUFF.36382.1; tss_id TSS36388;
8	Cufflinks	exon	134378693	134379299	.	+	.	gene_id GRMZM2G470513; transcript_id TCONS_00040180; exon_number 4; oId CUFF.36382.1; tss_id TSS36388;
8	Cufflinks	exon	135947268	135948122	.	+	.	gene_id AC207342.3_FG008; transcript_id TCONS_00040208; exon_number 1; oId CUFF.36433.1; tss_id TSS36415;
8	Cufflinks	exon	135948286	135948386	.	+	.	gene_id AC207342.3_FG008; transcript_id TCONS_00040208; exon_number 2; oId CUFF.36433.1; tss_id TSS36415;
8	Cufflinks	exon	135948484	135948567	.	+	.	gene_id AC207342.3_FG008; transcript_id TCONS_00040208; exon_number 3; oId CUFF.36433.1; tss_id TSS36415;
8	Cufflinks	exon	135949330	135949742	.	+	.	gene_id AC207342.3_FG008; transcript_id TCONS_00040208; exon_number 4; oId CUFF.36433.1; tss_id TSS36415;
8	Cufflinks	exon	142535900	142536724	.	+	.	gene_id GRMZM2G117742; transcript_id TCONS_00040302; exon_number 1; oId CUFF.36565.1; tss_id TSS36490;
8	Cufflinks	exon	142536824	142537004	.	+	.	gene_id GRMZM2G117742; transcript_id TCONS_00040302; exon_number 2; oId CUFF.36565.1; tss_id TSS36490;
8	Cufflinks	exon	142537938	142538119	.	+	.	gene_id GRMZM2G117742; transcript_id TCONS_00040302; exon_number 3; oId CUFF.36565.1; tss_id TSS36490;

8	Cufflinks	exon	142538187	142538342	.	+	.	gene_id GRMZM2G117742; transcript_id TCONS_00040302; exon_number 4; oId CUFF.36565.1; tss_id TSS36490;
8	Cufflinks	exon	142538415	142538931	.	+	.	gene_id GRMZM2G117742; transcript_id TCONS_00040302; exon_number 5; oId CUFF.36565.1; tss_id TSS36490;
8	Cufflinks	exon	145218849	145219519	.	+	.	gene_id GRMZM2G021436; transcript_id TCONS_00040336; exon_number 1; oId CUFF.36618.1; tss_id TSS36520;
8	Cufflinks	exon	145219624	145219874	.	+	.	gene_id GRMZM2G021436; transcript_id TCONS_00040336; exon_number 2; oId CUFF.36618.1; tss_id TSS36520;
8	Cufflinks	exon	145220026	145220392	.	+	.	gene_id GRMZM2G021436; transcript_id TCONS_00040336; exon_number 3; oId CUFF.36618.1; tss_id TSS36520;
8	Cufflinks	exon	145220536	145221114	.	+	.	gene_id GRMZM2G021436; transcript_id TCONS_00040336; exon_number 4; oId CUFF.36618.1; tss_id TSS36520;
8	Cufflinks	exon	146150403	146151619	.	+	.	gene_id GRMZM2G063672; transcript_id TCONS_00040371; exon_number 1; oId CUFF.36667.1; tss_id TSS36548;
8	Cufflinks	exon	146152770	146153728	.	+	.	gene_id GRMZM2G063672; transcript_id TCONS_00040371; exon_number 2; oId CUFF.36667.1; tss_id TSS36548;
8	Cufflinks	exon	152285732	152287245	.	+	.	gene_id XLOC_035503; transcript_id TCONS_00040481; exon_number 1; oId CUFF.36868.2; tss_id TSS36645;
8	Cufflinks	exon	152288899	152289126	.	+	.	gene_id XLOC_035503; transcript_id TCONS_00040481; exon_number 2; oId CUFF.36868.2; tss_id TSS36645;
8	Cufflinks	exon	152291156	152291342	.	+	.	gene_id XLOC_035503; transcript_id TCONS_00040481; exon_number 3; oId CUFF.36868.2; tss_id TSS36645;
8	Cufflinks	exon	152291445	152293012	.	+	.	gene_id XLOC_035503; transcript_id TCONS_00040481; exon_number 4; oId CUFF.36868.2; tss_id TSS36645;
8	Cufflinks	exon	152285732	152287245	.	+	.	gene_id XLOC_035503; transcript_id TCONS_00040480; exon_number 1; oId CUFF.36868.1; tss_id TSS36645;
8	Cufflinks	exon	152288899	152289126	.	+	.	gene_id XLOC_035503; transcript_id TCONS_00040480; exon_number 2; oId CUFF.36868.1; tss_id TSS36645;
8	Cufflinks	exon	152291166	152291342	.	+	.	gene_id XLOC_035503; transcript_id TCONS_00040480; exon_number 3; oId CUFF.36868.1; tss_id TSS36645;
8	Cufflinks	exon	152291445	152293012	.	+	.	gene_id XLOC_035503; transcript_id TCONS_00040480; exon_number 4; oId CUFF.36868.1; tss_id TSS36645;
8	Cufflinks	exon	152285736	152287695	.	+	.	gene_id XLOC_035503; transcript_id TCONS_00040482; exon_number 1; oId CUFF.36868.3; tss_id TSS36645;
8	Cufflinks	exon	155020506	155021940	.	+	.	gene_id GRMZM2G069203; transcript_id TCONS_00040528; exon_number 1; oId CUFF.36912.1; tss_id TSS36681;
8	Cufflinks	exon	159531387	159532365	.	+	.	gene_id GRMZM2G010176; transcript_id TCONS_00040632; exon_number 1; oId CUFF.37125.1; tss_id TSS36777;
8	Cufflinks	exon	159532617	159532667	.	+	.	gene_id GRMZM2G010176; transcript_id TCONS_00040632; exon_number 2; oId CUFF.37125.1; tss_id TSS36777;
8	Cufflinks	exon	159532772	159532844	.	+	.	gene_id GRMZM2G010176; transcript_id TCONS_00040632; exon_number 3; oId CUFF.37125.1; tss_id TSS36777;
8	Cufflinks	exon	159534791	159535110	.	+	.	gene_id GRMZM2G010176; transcript_id TCONS_00040632; exon_number 4; oId CUFF.37125.1; tss_id TSS36777;
8	Cufflinks	exon	159952116	159952424	.	+	.	gene_id GRMZM2G134162; transcript_id TCONS_00040642; exon_number 1; oId CUFF.37134.1; tss_id TSS36785;
8	Cufflinks	exon	159953808	159954113	.	+	.	gene_id GRMZM2G134162; transcript_id TCONS_00040642; exon_number 2; oId CUFF.37134.1; tss_id TSS36785;
8	Cufflinks	exon	159955187	159955504	.	+	.	gene_id GRMZM2G134162; transcript_id TCONS_00040642; exon_number 3; oId CUFF.37134.1; tss_id TSS36785;
8	Cufflinks	exon	159955610	159955825	.	+	.	gene_id GRMZM2G134162; transcript_id TCONS_00040642; exon_number 4; oId CUFF.37134.1; tss_id TSS36785;
8	Cufflinks	exon	159956049	159956149	.	+	.	gene_id GRMZM2G134162; transcript_id TCONS_00040642; exon_number 5; oId CUFF.37134.1; tss_id TSS36785;
8	Cufflinks	exon	159956220	159956337	.	+	.	gene_id GRMZM2G134162; transcript_id TCONS_00040642; exon_number 6; oId CUFF.37134.1; tss_id TSS36785;
8	Cufflinks	exon	159956556	159957434	.	+	.	gene_id GRMZM2G134162; transcript_id TCONS_00040642; exon_number 7; oId CUFF.37134.1; tss_id TSS36785;
8	Cufflinks	exon	162305650	162306050	.	+	.	gene_id XLOC_035676; transcript_id TCONS_00040686; exon_number 1; oId CUFF.37195.1; tss_id TSS36822;
8	Cufflinks	exon	162335276	162335492	.	+	.	gene_id GRMZM2G470942; transcript_id TCONS_00040687; exon_number 1; oId CUFF.37217.1; tss_id TSS36823;
8	Cufflinks	exon	162335939	162336127	.	+	.	gene_id GRMZM2G470942; transcript_id TCONS_00040687; exon_number 2; oId CUFF.37217.1; tss_id TSS36823;
8	Cufflinks	exon	162336348	162336512	.	+	.	gene_id GRMZM2G470942; transcript_id TCONS_00040687; exon_number 3; oId CUFF.37217.1; tss_id TSS36823;
8	Cufflinks	exon	162336837	162337118	.	+	.	gene_id GRMZM2G470942; transcript_id TCONS_00040687; exon_number 4; oId CUFF.37217.1; tss_id TSS36823;
8	Cufflinks	exon	162335330	162335492	.	+	.	gene_id GRMZM2G470942; transcript_id TCONS_00040688; exon_number 1; oId CUFF.37217.2; tss_id TSS36823;
8	Cufflinks	exon	162335939	162336127	.	+	.	gene_id GRMZM2G470942; transcript_id TCONS_00040688; exon_number 2; oId CUFF.37217.2; tss_id TSS36823;

8	Cufflinks	exon	162336348	162336503	.	+	.	gene_id GRMZM2G470942; transcript_id TCONS_00040688; exon_number 3; oId CUFF.37217.2; tss_id TSS36823;
8	Cufflinks	exon	162336837	162337118	.	+	.	gene_id GRMZM2G470942; transcript_id TCONS_00040688; exon_number 4; oId CUFF.37217.2; tss_id TSS36823;
8	Cufflinks	exon	162588168	162589986	.	+	.	gene_id GRMZM2G175406; transcript_id TCONS_00040695; exon_number 1; oId CUFF.37210.1; tss_id TSS36829;
8	Cufflinks	exon	163128903	163129317	.	+	.	gene_id GRMZM2G100318; transcript_id TCONS_00040709; exon_number 1; oId CUFF.37241.2; tss_id TSS36841;
8	Cufflinks	exon	163130338	163130490	.	+	.	gene_id GRMZM2G100318; transcript_id TCONS_00040709; exon_number 2; oId CUFF.37241.2; tss_id TSS36841;
8	Cufflinks	exon	163130585	163130971	.	+	.	gene_id GRMZM2G100318; transcript_id TCONS_00040709; exon_number 3; oId CUFF.37241.2; tss_id TSS36841;
8	Cufflinks	exon	163131079	163131155	.	+	.	gene_id GRMZM2G100318; transcript_id TCONS_00040709; exon_number 4; oId CUFF.37241.2; tss_id TSS36841;
8	Cufflinks	exon	163131813	163133067	.	+	.	gene_id GRMZM2G100318; transcript_id TCONS_00040709; exon_number 5; oId CUFF.37241.2; tss_id TSS36841;
8	Cufflinks	exon	163203559	163204199	.	+	.	gene_id XLOC_035697; transcript_id TCONS_00040712; exon_number 1; oId CUFF.37237.1; tss_id TSS36844;
8	Cufflinks	exon	164285571	164285708	.	+	.	gene_id XLOC_035726; transcript_id TCONS_00040744; exon_number 1; oId CUFF.37281.1; tss_id TSS36873;
8	Cufflinks	exon	164285774	164286601	.	+	.	gene_id XLOC_035726; transcript_id TCONS_00040744; exon_number 2; oId CUFF.37281.1; tss_id TSS36873;
8	Cufflinks	exon	164450478	164451426	.	+	.	gene_id GRMZM2G068361; transcript_id TCONS_00040753; exon_number 1; oId CUFF.37294.1; tss_id TSS36880;
8	Cufflinks	exon	164451989	164452474	.	+	.	gene_id GRMZM2G068361; transcript_id TCONS_00040753; exon_number 2; oId CUFF.37294.1; tss_id TSS36880;
8	Cufflinks	exon	164822797	164823361	.	+	.	gene_id GRMZM2G092817; transcript_id TCONS_00040760; exon_number 1; oId CUFF.37317.1; tss_id TSS36886;
8	Cufflinks	exon	164825198	164825568	.	+	.	gene_id GRMZM2G092817; transcript_id TCONS_00040760; exon_number 2; oId CUFF.37317.1; tss_id TSS36886;
8	Cufflinks	exon	164825639	164826113	.	+	.	gene_id GRMZM2G092817; transcript_id TCONS_00040760; exon_number 3; oId CUFF.37317.1; tss_id TSS36886;
8	Cufflinks	exon	166450330	166453602	.	+	.	gene_id GRMZM2G396248; transcript_id TCONS_00040799; exon_number 1; oId CUFF.37393.1; tss_id TSS36922;
8	Cufflinks	exon	166704257	166706406	.	+	.	gene_id GRMZM2G123394; transcript_id TCONS_00040805; exon_number 1; oId CUFF.37401.1; tss_id TSS36927;
8	Cufflinks	exon	168615332	168615693	.	+	.	gene_id GRMZM2G112836; transcript_id TCONS_00040846; exon_number 1; oId CUFF.37495.2; tss_id TSS36967;
8	Cufflinks	exon	168615806	168615875	.	+	.	gene_id GRMZM2G112836; transcript_id TCONS_00040846; exon_number 2; oId CUFF.37495.2; tss_id TSS36967;
8	Cufflinks	exon	168616003	168617500	.	+	.	gene_id GRMZM2G112836; transcript_id TCONS_00040846; exon_number 3; oId CUFF.37495.2; tss_id TSS36967;
8	Cufflinks	exon	169068643	169069208	.	+	.	gene_id GRMZM5G840487; transcript_id TCONS_00040854; exon_number 1; oId CUFF.37526.1; tss_id TSS36974;
8	Cufflinks	exon	169070468	169070606	.	+	.	gene_id GRMZM5G840487; transcript_id TCONS_00040854; exon_number 2; oId CUFF.37526.1; tss_id TSS36974;
8	Cufflinks	exon	169071534	169073066	.	+	.	gene_id GRMZM5G840487; transcript_id TCONS_00040854; exon_number 3; oId CUFF.37526.1; tss_id TSS36974;
8	Cufflinks	exon	169069683	169070348	.	+	.	gene_id GRMZM5G840487; transcript_id TCONS_00040855; exon_number 1; oId CUFF.37526.2; tss_id TSS36975;
8	Cufflinks	exon	169070468	169070606	.	+	.	gene_id GRMZM5G840487; transcript_id TCONS_00040855; exon_number 2; oId CUFF.37526.2; tss_id TSS36975;
8	Cufflinks	exon	169071534	169073066	.	+	.	gene_id GRMZM5G840487; transcript_id TCONS_00040855; exon_number 3; oId CUFF.37526.2; tss_id TSS36975;
8	Cufflinks	exon	169175095	169175629	.	+	.	gene_id GRMZM2G066755; transcript_id TCONS_00040856; exon_number 1; oId CUFF.37521.1; tss_id TSS36976;
8	Cufflinks	exon	169176464	169176592	.	+	.	gene_id GRMZM2G066755; transcript_id TCONS_00040856; exon_number 2; oId CUFF.37521.1; tss_id TSS36976;
8	Cufflinks	exon	169176686	169176806	.	+	.	gene_id GRMZM2G066755; transcript_id TCONS_00040856; exon_number 3; oId CUFF.37521.1; tss_id TSS36976;
8	Cufflinks	exon	169176895	169176976	.	+	.	gene_id GRMZM2G066755; transcript_id TCONS_00040856; exon_number 4; oId CUFF.37521.1; tss_id TSS36976;
8	Cufflinks	exon	169177469	169177622	.	+	.	gene_id GRMZM2G066755; transcript_id TCONS_00040856; exon_number 5; oId CUFF.37521.1; tss_id TSS36976;
8	Cufflinks	exon	169177711	169178292	.	+	.	gene_id GRMZM2G066755; transcript_id TCONS_00040856; exon_number 6; oId CUFF.37521.1; tss_id TSS36976;
8	Cufflinks	exon	169677765	169678824	.	+	.	gene_id GRMZM2G036448; transcript_id TCONS_00040873; exon_number 1; oId CUFF.37557.2; tss_id TSS36993;
8	Cufflinks	exon	169678958	169679107	.	+	.	gene_id GRMZM2G036448; transcript_id TCONS_00040873; exon_number 2; oId CUFF.37557.2; tss_id TSS36993;
8	Cufflinks	exon	169679278	169679481	.	+	.	gene_id GRMZM2G036448; transcript_id TCONS_00040873; exon_number 3; oId CUFF.37557.2; tss_id TSS36993;

8	Cufflinks	exon	169679586	169680051	.	+	.	gene_id GRMZM2G036448; transcript_id TCONS_00040873; exon_number 4; oId CUFF.37557.2; tss_id TSS36993;
8	Cufflinks	exon	169680153	169680749	.	+	.	gene_id GRMZM2G036448; transcript_id TCONS_00040873; exon_number 5; oId CUFF.37557.2; tss_id TSS36993;
8	Cufflinks	exon	171307877	171308490	.	+	.	gene_id GRMZM2G139374; transcript_id TCONS_00040921; exon_number 1; oId CUFF.37639.1; tss_id TSS37040;
8	Cufflinks	exon	171309821	171309961	.	+	.	gene_id GRMZM2G139374; transcript_id TCONS_00040921; exon_number 2; oId CUFF.37639.1; tss_id TSS37040;
8	Cufflinks	exon	171310096	171310461	.	+	.	gene_id GRMZM2G139374; transcript_id TCONS_00040921; exon_number 3; oId CUFF.37639.1; tss_id TSS37040;
8	Cufflinks	exon	171311468	171311713	.	+	.	gene_id GRMZM2G139374; transcript_id TCONS_00040921; exon_number 4; oId CUFF.37639.1; tss_id TSS37040;
8	Cufflinks	exon	171312010	171312102	.	+	.	gene_id GRMZM2G139374; transcript_id TCONS_00040921; exon_number 5; oId CUFF.37639.1; tss_id TSS37040;
8	Cufflinks	exon	171312594	171312794	.	+	.	gene_id GRMZM2G139374; transcript_id TCONS_00040921; exon_number 6; oId CUFF.37639.1; tss_id TSS37040;
8	Cufflinks	exon	171313031	171313576	.	+	.	gene_id GRMZM2G139374; transcript_id TCONS_00040921; exon_number 7; oId CUFF.37639.1; tss_id TSS37040;
8	Cufflinks	exon	172112785	172113031	.	+	.	gene_id GRMZM2G116086; transcript_id TCONS_00040949; exon_number 1; oId CUFF.37689.1; tss_id TSS37065;
8	Cufflinks	exon	172113688	172114106	.	+	.	gene_id GRMZM2G116086; transcript_id TCONS_00040949; exon_number 2; oId CUFF.37689.1; tss_id TSS37065;
8	Cufflinks	exon	172116610	172116735	.	+	.	gene_id GRMZM2G116086; transcript_id TCONS_00040949; exon_number 3; oId CUFF.37689.1; tss_id TSS37065;
8	Cufflinks	exon	172116814	172116887	.	+	.	gene_id GRMZM2G116086; transcript_id TCONS_00040949; exon_number 4; oId CUFF.37689.1; tss_id TSS37065;
8	Cufflinks	exon	172117256	172117325	.	+	.	gene_id GRMZM2G116086; transcript_id TCONS_00040949; exon_number 5; oId CUFF.37689.1; tss_id TSS37065;
8	Cufflinks	exon	172117413	172117468	.	+	.	gene_id GRMZM2G116086; transcript_id TCONS_00040949; exon_number 6; oId CUFF.37689.1; tss_id TSS37065;
8	Cufflinks	exon	172117547	172117618	.	+	.	gene_id GRMZM2G116086; transcript_id TCONS_00040949; exon_number 7; oId CUFF.37689.1; tss_id TSS37065;
8	Cufflinks	exon	172117695	172117789	.	+	.	gene_id GRMZM2G116086; transcript_id TCONS_00040949; exon_number 8; oId CUFF.37689.1; tss_id TSS37065;
8	Cufflinks	exon	172117982	172118051	.	+	.	gene_id GRMZM2G116086; transcript_id TCONS_00040949; exon_number 9; oId CUFF.37689.1; tss_id TSS37065;
8	Cufflinks	exon	172118126	172118255	.	+	.	gene_id GRMZM2G116086; transcript_id TCONS_00040949; exon_number 10; oId CUFF.37689.1; tss_id TSS37065;
8	Cufflinks	exon	172118592	172118807	.	+	.	gene_id GRMZM2G116086; transcript_id TCONS_00040949; exon_number 11; oId CUFF.37689.1; tss_id TSS37065;
8	Cufflinks	exon	172118925	172119055	.	+	.	gene_id GRMZM2G116086; transcript_id TCONS_00040949; exon_number 12; oId CUFF.37689.1; tss_id TSS37065;
8	Cufflinks	exon	172119532	172119706	.	+	.	gene_id GRMZM2G116086; transcript_id TCONS_00040949; exon_number 13; oId CUFF.37689.1; tss_id TSS37065;
8	Cufflinks	exon	172119790	172119880	.	+	.	gene_id GRMZM2G116086; transcript_id TCONS_00040949; exon_number 14; oId CUFF.37689.1; tss_id TSS37065;
8	Cufflinks	exon	172120674	172120831	.	+	.	gene_id GRMZM2G116086; transcript_id TCONS_00040949; exon_number 15; oId CUFF.37689.1; tss_id TSS37065;
8	Cufflinks	exon	172121037	172121225	.	+	.	gene_id GRMZM2G116086; transcript_id TCONS_00040949; exon_number 16; oId CUFF.37689.1; tss_id TSS37065;
8	Cufflinks	exon	172121309	172121960	.	+	.	gene_id GRMZM2G116086; transcript_id TCONS_00040949; exon_number 17; oId CUFF.37689.1; tss_id TSS37065;
8	Cufflinks	exon	705086	705419	.	-	.	gene_id XLOC_035994; transcript_id TCONS_00041043; exon_number 1; oId CUFF.34158.1; tss_id TSS37153;
8	Cufflinks	exon	1145660	1146015	.	-	.	gene_id GRMZM2G063223; transcript_id TCONS_00041052; exon_number 1; oId CUFF.34182.1; tss_id TSS37161;
8	Cufflinks	exon	1146801	1147184	.	-	.	gene_id GRMZM2G063223; transcript_id TCONS_00041052; exon_number 2; oId CUFF.34182.1; tss_id TSS37161;
8	Cufflinks	exon	2901206	2901712	.	-	.	gene_id GRMZM2G019515; transcript_id TCONS_00041074; exon_number 1; oId CUFF.34213.1; tss_id TSS37182;
8	Cufflinks	exon	2901989	2902348	.	-	.	gene_id GRMZM2G019515; transcript_id TCONS_00041074; exon_number 2; oId CUFF.34213.1; tss_id TSS37182;
8	Cufflinks	exon	2903517	2904218	.	-	.	gene_id GRMZM2G019515; transcript_id TCONS_00041074; exon_number 3; oId CUFF.34213.1; tss_id TSS37182;
8	Cufflinks	exon	11486313	11487605	.	-	.	gene_id GRMZM2G096358; transcript_id TCONS_00041179; exon_number 1; oId CUFF.34447.1; tss_id TSS37283;
8	Cufflinks	exon	11487744	11488153	.	-	.	gene_id GRMZM2G096358; transcript_id TCONS_00041179; exon_number 2; oId CUFF.34447.1; tss_id TSS37283;
8	Cufflinks	exon	18444281	18446241	.	-	.	gene_id GRMZM2G375159; transcript_id TCONS_00041297; exon_number 1; oId CUFF.34636.1; tss_id TSS37386;
8	Cufflinks	exon	20703355	20703741	.	-	.	gene_id GRMZM2G080375; transcript_id TCONS_00041319; exon_number 1; oId CUFF.34710.1; tss_id TSS37406;

8	Cufflinks	exon	20703833	20704036	.	-	.	gene_id GRMZM2G080375; transcript_id TCONS_00041319; exon_number 2; oId CUFF.34710.1; tss_id TSS37406;
8	Cufflinks	exon	20704236	20704460	.	-	.	gene_id GRMZM2G080375; transcript_id TCONS_00041319; exon_number 3; oId CUFF.34710.1; tss_id TSS37406;
8	Cufflinks	exon	20704568	20704608	.	-	.	gene_id GRMZM2G080375; transcript_id TCONS_00041319; exon_number 4; oId CUFF.34710.1; tss_id TSS37406;
8	Cufflinks	exon	20704724	20704853	.	-	.	gene_id GRMZM2G080375; transcript_id TCONS_00041319; exon_number 5; oId CUFF.34710.1; tss_id TSS37406;
8	Cufflinks	exon	20704944	20705012	.	-	.	gene_id GRMZM2G080375; transcript_id TCONS_00041319; exon_number 6; oId CUFF.34710.1; tss_id TSS37406;
8	Cufflinks	exon	20705099	20705311	.	-	.	gene_id GRMZM2G080375; transcript_id TCONS_00041319; exon_number 7; oId CUFF.34710.1; tss_id TSS37406;
8	Cufflinks	exon	20705616	20705816	.	-	.	gene_id GRMZM2G080375; transcript_id TCONS_00041319; exon_number 8; oId CUFF.34710.1; tss_id TSS37406;
8	Cufflinks	exon	20705916	20705955	.	-	.	gene_id GRMZM2G080375; transcript_id TCONS_00041319; exon_number 9; oId CUFF.34710.1; tss_id TSS37406;
8	Cufflinks	exon	20706091	20706137	.	-	.	gene_id GRMZM2G080375; transcript_id TCONS_00041319; exon_number 10; oId CUFF.34710.1; tss_id TSS37406;
8	Cufflinks	exon	20706482	20706994	.	-	.	gene_id GRMZM2G080375; transcript_id TCONS_00041319; exon_number 11; oId CUFF.34710.1; tss_id TSS37406;
8	Cufflinks	exon	22180368	22180764	.	-	.	gene_id GRMZM2G004083; transcript_id TCONS_00041345; exon_number 1; oId CUFF.34721.1; tss_id TSS37426;
8	Cufflinks	exon	22181123	22181282	.	-	.	gene_id GRMZM2G004083; transcript_id TCONS_00041345; exon_number 2; oId CUFF.34721.1; tss_id TSS37426;
8	Cufflinks	exon	22181364	22181485	.	-	.	gene_id GRMZM2G004083; transcript_id TCONS_00041345; exon_number 3; oId CUFF.34721.1; tss_id TSS37426;
8	Cufflinks	exon	22181587	22182467	.	-	.	gene_id GRMZM2G004083; transcript_id TCONS_00041345; exon_number 4; oId CUFF.34721.1; tss_id TSS37426;
8	Cufflinks	exon	26863089	26864155	.	-	.	gene_id GRMZM2G127687; transcript_id TCONS_00041398; exon_number 1; oId CUFF.34812.1; tss_id TSS37477;
8	Cufflinks	exon	26865836	26867549	.	-	.	gene_id GRMZM2G127687; transcript_id TCONS_00041398; exon_number 2; oId CUFF.34812.1; tss_id TSS37477;
8	Cufflinks	exon	27218394	27219064	.	-	.	gene_id GRMZM2G010596; transcript_id TCONS_00041406; exon_number 1; oId CUFF.34823.1; tss_id TSS37484;
8	Cufflinks	exon	27219154	27219207	.	-	.	gene_id GRMZM2G010596; transcript_id TCONS_00041406; exon_number 2; oId CUFF.34823.1; tss_id TSS37484;
8	Cufflinks	exon	27219474	27219620	.	-	.	gene_id GRMZM2G010596; transcript_id TCONS_00041406; exon_number 3; oId CUFF.34823.1; tss_id TSS37484;
8	Cufflinks	exon	27219737	27219943	.	-	.	gene_id GRMZM2G010596; transcript_id TCONS_00041406; exon_number 4; oId CUFF.34823.1; tss_id TSS37484;
8	Cufflinks	exon	27220569	27221078	.	-	.	gene_id GRMZM2G010596; transcript_id TCONS_00041406; exon_number 5; oId CUFF.34823.1; tss_id TSS37484;
8	Cufflinks	exon	27670252	27671597	.	-	.	gene_id XLOC_036317; transcript_id TCONS_00041409; exon_number 1; oId CUFF.34835.1; tss_id TSS37486;
8	Cufflinks	exon	27671725	27672389	.	-	.	gene_id XLOC_036317; transcript_id TCONS_00041409; exon_number 2; oId CUFF.34835.1; tss_id TSS37486;
8	Cufflinks	exon	36144983	36145351	.	-	.	gene_id GRMZM5G842965; transcript_id TCONS_00041465; exon_number 1; oId CUFF.34925.4; tss_id TSS37531;
8	Cufflinks	exon	36145477	36145542	.	-	.	gene_id GRMZM5G842965; transcript_id TCONS_00041465; exon_number 2; oId CUFF.34925.4; tss_id TSS37531;
8	Cufflinks	exon	36145662	36145766	.	-	.	gene_id GRMZM5G842965; transcript_id TCONS_00041465; exon_number 3; oId CUFF.34925.4; tss_id TSS37531;
8	Cufflinks	exon	36146764	36146867	.	-	.	gene_id GRMZM5G842965; transcript_id TCONS_00041465; exon_number 4; oId CUFF.34925.4; tss_id TSS37531;
8	Cufflinks	exon	36146993	36147389	.	-	.	gene_id GRMZM5G842965; transcript_id TCONS_00041465; exon_number 5; oId CUFF.34925.4; tss_id TSS37531;
8	Cufflinks	exon	37813042	37814105	.	-	.	gene_id GRMZM2G061088; transcript_id TCONS_00041478; exon_number 1; oId CUFF.34942.1; tss_id TSS37544;
8	Cufflinks	exon	37816462	37816875	.	-	.	gene_id GRMZM2G061088; transcript_id TCONS_00041478; exon_number 2; oId CUFF.34942.1; tss_id TSS37544;
8	Cufflinks	exon	51816339	51817463	.	-	.	gene_id AC205274.3_FG001; transcript_id TCONS_00041536; exon_number 1; oId CUFF.35030.1; tss_id TSS37594;
8	Cufflinks	exon	67245670	67246224	.	-	.	gene_id GRMZM2G345039; transcript_id TCONS_00041625; exon_number 1; oId CUFF.35237.2; tss_id TSS37677;
8	Cufflinks	exon	67246321	67246374	.	-	.	gene_id GRMZM2G345039; transcript_id TCONS_00041625; exon_number 2; oId CUFF.35237.2; tss_id TSS37677;
8	Cufflinks	exon	67246490	67246579	.	-	.	gene_id GRMZM2G345039; transcript_id TCONS_00041625; exon_number 3; oId CUFF.35237.2; tss_id TSS37677;
8	Cufflinks	exon	67246756	67246841	.	-	.	gene_id GRMZM2G345039; transcript_id TCONS_00041625; exon_number 4; oId CUFF.35237.2; tss_id TSS37677;
8	Cufflinks	exon	67246927	67247043	.	-	.	gene_id GRMZM2G345039; transcript_id TCONS_00041625; exon_number 5; oId CUFF.35237.2; tss_id TSS37677;

8	Cufflinks	exon	67247921	67247957	.	-	.	gene_id GRMZM2G345039; transcript_id TCONS_00041625; exon_number 6; oId CUFF.35237.2; tss_id TSS37677;
8	Cufflinks	exon	67248032	67248110	.	-	.	gene_id GRMZM2G345039; transcript_id TCONS_00041625; exon_number 7; oId CUFF.35237.2; tss_id TSS37677;
8	Cufflinks	exon	67249665	67249732	.	-	.	gene_id GRMZM2G345039; transcript_id TCONS_00041625; exon_number 8; oId CUFF.35237.2; tss_id TSS37677;
8	Cufflinks	exon	67249814	67249855	.	-	.	gene_id GRMZM2G345039; transcript_id TCONS_00041625; exon_number 9; oId CUFF.35237.2; tss_id TSS37677;
8	Cufflinks	exon	67250342	67250402	.	-	.	gene_id GRMZM2G345039; transcript_id TCONS_00041625; exon_number 10; oId CUFF.35237.2; tss_id TSS37677;
8	Cufflinks	exon	67250533	67250579	.	-	.	gene_id GRMZM2G345039; transcript_id TCONS_00041625; exon_number 11; oId CUFF.35237.2; tss_id TSS37677;
8	Cufflinks	exon	67250660	67250897	.	-	.	gene_id GRMZM2G345039; transcript_id TCONS_00041625; exon_number 12; oId CUFF.35237.2; tss_id TSS37677;
8	Cufflinks	exon	67245670	67246228	.	-	.	gene_id GRMZM2G345039; transcript_id TCONS_00041624; exon_number 1; oId CUFF.35237.1; tss_id TSS37677;
8	Cufflinks	exon	67246321	67246374	.	-	.	gene_id GRMZM2G345039; transcript_id TCONS_00041624; exon_number 2; oId CUFF.35237.1; tss_id TSS37677;
8	Cufflinks	exon	67246490	67246579	.	-	.	gene_id GRMZM2G345039; transcript_id TCONS_00041624; exon_number 3; oId CUFF.35237.1; tss_id TSS37677;
8	Cufflinks	exon	67246756	67246841	.	-	.	gene_id GRMZM2G345039; transcript_id TCONS_00041624; exon_number 4; oId CUFF.35237.1; tss_id TSS37677;
8	Cufflinks	exon	67246927	67247043	.	-	.	gene_id GRMZM2G345039; transcript_id TCONS_00041624; exon_number 5; oId CUFF.35237.1; tss_id TSS37677;
8	Cufflinks	exon	67247921	67247957	.	-	.	gene_id GRMZM2G345039; transcript_id TCONS_00041624; exon_number 6; oId CUFF.35237.1; tss_id TSS37677;
8	Cufflinks	exon	67248032	67248110	.	-	.	gene_id GRMZM2G345039; transcript_id TCONS_00041624; exon_number 7; oId CUFF.35237.1; tss_id TSS37677;
8	Cufflinks	exon	67249665	67249732	.	-	.	gene_id GRMZM2G345039; transcript_id TCONS_00041624; exon_number 8; oId CUFF.35237.1; tss_id TSS37677;
8	Cufflinks	exon	67249814	67249855	.	-	.	gene_id GRMZM2G345039; transcript_id TCONS_00041624; exon_number 9; oId CUFF.35237.1; tss_id TSS37677;
8	Cufflinks	exon	67250342	67250402	.	-	.	gene_id GRMZM2G345039; transcript_id TCONS_00041624; exon_number 10; oId CUFF.35237.1; tss_id TSS37677;
8	Cufflinks	exon	67250533	67250579	.	-	.	gene_id GRMZM2G345039; transcript_id TCONS_00041624; exon_number 11; oId CUFF.35237.1; tss_id TSS37677;
8	Cufflinks	exon	67250660	67250897	.	-	.	gene_id GRMZM2G345039; transcript_id TCONS_00041624; exon_number 12; oId CUFF.35237.1; tss_id TSS37677;
8	Cufflinks	exon	69273990	69274639	.	-	.	gene_id XLOC_036521; transcript_id TCONS_00041645; exon_number 1; oId CUFF.35234.1; tss_id TSS37696;
8	Cufflinks	exon	74456610	74457885	.	-	.	gene_id GRMZM2G045664; transcript_id TCONS_00041685; exon_number 1; oId CUFF.35321.1; tss_id TSS37734;
8	Cufflinks	exon	86555339	86556140	.	-	.	gene_id GRMZM2G007404; transcript_id TCONS_00041759; exon_number 1; oId CUFF.35498.1; tss_id TSS37803;
8	Cufflinks	exon	86556341	86556415	.	-	.	gene_id GRMZM2G007404; transcript_id TCONS_00041759; exon_number 2; oId CUFF.35498.1; tss_id TSS37803;
8	Cufflinks	exon	86556504	86556587	.	-	.	gene_id GRMZM2G007404; transcript_id TCONS_00041759; exon_number 3; oId CUFF.35498.1; tss_id TSS37803;
8	Cufflinks	exon	86556684	86556763	.	-	.	gene_id GRMZM2G007404; transcript_id TCONS_00041759; exon_number 4; oId CUFF.35498.1; tss_id TSS37803;
8	Cufflinks	exon	86557611	86557758	.	-	.	gene_id GRMZM2G007404; transcript_id TCONS_00041759; exon_number 5; oId CUFF.35498.1; tss_id TSS37803;
8	Cufflinks	exon	86558808	86559750	.	-	.	gene_id GRMZM2G007404; transcript_id TCONS_00041759; exon_number 6; oId CUFF.35498.1; tss_id TSS37803;
8	Cufflinks	exon	94309687	94310894	.	-	.	gene_id GRMZM2G358606; transcript_id TCONS_00041821; exon_number 1; oId CUFF.35565.1; tss_id TSS37858;
8	Cufflinks	exon	109359888	109360346	.	-	.	gene_id GRMZM2G116238; transcript_id TCONS_00041962; exon_number 1; oId CUFF.35841.2; tss_id TSS37990;
8	Cufflinks	exon	109360431	109361490	.	-	.	gene_id GRMZM2G116238; transcript_id TCONS_00041962; exon_number 2; oId CUFF.35841.2; tss_id TSS37990;
8	Cufflinks	exon	113237540	113238297	.	-	.	gene_id GRMZM2G029756; transcript_id TCONS_00041997; exon_number 1; oId CUFF.35895.1; tss_id TSS38020;
8	Cufflinks	exon	113240810	113242322	.	-	.	gene_id GRMZM2G029756; transcript_id TCONS_00041997; exon_number 2; oId CUFF.35895.1; tss_id TSS38020;
8	Cufflinks	exon	118087800	118088310	.	-	.	gene_id XLOC_036878; transcript_id TCONS_00042041; exon_number 1; oId CUFF.35998.1; tss_id TSS38062;
8	Cufflinks	exon	119461575	119462915	.	-	.	gene_id GRMZM2G073347; transcript_id TCONS_00042069; exon_number 1; oId CUFF.36034.1; tss_id TSS38088;
8	Cufflinks	exon	131331979	131332862	.	-	.	gene_id AC214648.3_FG003; transcript_id TCONS_00042228; exon_number 1; oId CUFF.36308.1; tss_id TSS38234;
8	Cufflinks	exon	131335924	131336740	.	-	.	gene_id AC214648.3_FG003; transcript_id TCONS_00042228; exon_number 2; oId CUFF.36308.1; tss_id TSS38234;

8	Cufflinks	exon	131733467	131737823	.	-	.	gene_id GRMZM2G173763; transcript_id TCONS_00042230; exon_number 1; oId CUFF.36346.1; tss_id TSS38236;
8	Cufflinks	exon	131737966	131738126	.	-	.	gene_id GRMZM2G173763; transcript_id TCONS_00042230; exon_number 2; oId CUFF.36346.1; tss_id TSS38236;
8	Cufflinks	exon	133998505	133999042	.	-	.	gene_id GRMZM2G028986; transcript_id TCONS_00042261; exon_number 1; oId CUFF.36373.1; tss_id TSS38265;
8	Cufflinks	exon	133999138	133999204	.	-	.	gene_id GRMZM2G028986; transcript_id TCONS_00042261; exon_number 2; oId CUFF.36373.1; tss_id TSS38265;
8	Cufflinks	exon	134000509	134000685	.	-	.	gene_id GRMZM2G028986; transcript_id TCONS_00042261; exon_number 3; oId CUFF.36373.1; tss_id TSS38265;
8	Cufflinks	exon	135310261	135311594	.	-	.	gene_id XLOC_037089; transcript_id TCONS_00042291; exon_number 1; oId CUFF.36423.3; tss_id TSS38281;
8	Cufflinks	exon	135311941	135312158	.	-	.	gene_id XLOC_037089; transcript_id TCONS_00042291; exon_number 2; oId CUFF.36423.3; tss_id TSS38281;
8	Cufflinks	exon	135310261	135311609	.	-	.	gene_id XLOC_037089; transcript_id TCONS_00042293; exon_number 1; oId CUFF.36423.2; tss_id TSS38281;
8	Cufflinks	exon	135311941	135312197	.	-	.	gene_id XLOC_037089; transcript_id TCONS_00042293; exon_number 2; oId CUFF.36423.2; tss_id TSS38281;
8	Cufflinks	exon	142537765	142540628	.	-	.	gene_id GRMZM5G803433; transcript_id TCONS_00042372; exon_number 1; oId CUFF.36566.1; tss_id TSS38349;
8	Cufflinks	exon	142542465	142543929	.	-	.	gene_id GRMZM5G803433; transcript_id TCONS_00042372; exon_number 2; oId CUFF.36566.1; tss_id TSS38349;
8	Cufflinks	exon	144765266	144765848	.	-	.	gene_id GRMZM2G149683; transcript_id TCONS_00042398; exon_number 1; oId CUFF.36611.1; tss_id TSS38374;
8	Cufflinks	exon	144766048	144766173	.	-	.	gene_id GRMZM2G149683; transcript_id TCONS_00042398; exon_number 2; oId CUFF.36611.1; tss_id TSS38374;
8	Cufflinks	exon	144766344	144766964	.	-	.	gene_id GRMZM2G149683; transcript_id TCONS_00042398; exon_number 3; oId CUFF.36611.1; tss_id TSS38374;
8	Cufflinks	exon	151798812	151799399	.	-	.	gene_id GRMZM5G843389; transcript_id TCONS_00042511; exon_number 1; oId CUFF.36823.1; tss_id TSS38475;
8	Cufflinks	exon	151799501	151799650	.	-	.	gene_id GRMZM5G843389; transcript_id TCONS_00042511; exon_number 2; oId CUFF.36823.1; tss_id TSS38475;
8	Cufflinks	exon	151799724	151799819	.	-	.	gene_id GRMZM5G843389; transcript_id TCONS_00042511; exon_number 3; oId CUFF.36823.1; tss_id TSS38475;
8	Cufflinks	exon	151801952	151802122	.	-	.	gene_id GRMZM5G843389; transcript_id TCONS_00042511; exon_number 4; oId CUFF.36823.1; tss_id TSS38475;
8	Cufflinks	exon	151802525	151803317	.	-	.	gene_id GRMZM5G843389; transcript_id TCONS_00042511; exon_number 5; oId CUFF.36823.1; tss_id TSS38475;
8	Cufflinks	exon	152285587	152287295	.	-	.	gene_id XLOC_037286; transcript_id TCONS_00042517; exon_number 1; oId CUFF.36867.1; tss_id TSS38481;
8	Cufflinks	exon	153934934	153935113	.	-	.	gene_id XLOC_037303; transcript_id TCONS_00042535; exon_number 1; oId CUFF.36876.1; tss_id TSS38499;
8	Cufflinks	exon	153935644	153935775	.	-	.	gene_id XLOC_037303; transcript_id TCONS_00042535; exon_number 2; oId CUFF.36876.1; tss_id TSS38499;
8	Cufflinks	exon	153936804	153937499	.	-	.	gene_id XLOC_037303; transcript_id TCONS_00042535; exon_number 3; oId CUFF.36876.1; tss_id TSS38499;
8	Cufflinks	exon	154561144	154562088	.	-	.	gene_id GRMZM2G107985; transcript_id TCONS_00042554; exon_number 1; oId CUFF.36917.3; tss_id TSS38516;
8	Cufflinks	exon	154562201	154562565	.	-	.	gene_id GRMZM2G107985; transcript_id TCONS_00042554; exon_number 2; oId CUFF.36917.3; tss_id TSS38516;
8	Cufflinks	exon	156268171	156269856	.	-	.	gene_id GRMZM2G367701; transcript_id TCONS_00042592; exon_number 1; oId CUFF.36969.1; tss_id TSS38552;
8	Cufflinks	exon	159956854	159957468	.	-	.	gene_id GRMZM2G433959; transcript_id TCONS_00042683; exon_number 1; oId CUFF.37137.1; tss_id TSS38633;
8	Cufflinks	exon	159957537	159957601	.	-	.	gene_id GRMZM2G433959; transcript_id TCONS_00042683; exon_number 2; oId CUFF.37137.1; tss_id TSS38633;
8	Cufflinks	exon	159957714	159957921	.	-	.	gene_id GRMZM2G433959; transcript_id TCONS_00042683; exon_number 3; oId CUFF.37137.1; tss_id TSS38633;
8	Cufflinks	exon	163203210	163204952	.	-	.	gene_id XLOC_037490; transcript_id TCONS_00042748; exon_number 1; oId CUFF.37236.1; tss_id TSS38694;
8	Cufflinks	exon	164285437	164286435	.	-	.	gene_id GRMZM2G171996; transcript_id TCONS_00042773; exon_number 1; oId CUFF.37280.1; tss_id TSS38713;
8	Cufflinks	exon	166202041	166202906	.	-	.	gene_id GRMZM2G143640; transcript_id TCONS_00042828; exon_number 1; oId CUFF.37374.1; tss_id TSS38759;
8	Cufflinks	exon	166203002	166203641	.	-	.	gene_id GRMZM2G143640; transcript_id TCONS_00042828; exon_number 2; oId CUFF.37374.1; tss_id TSS38759;
8	Cufflinks	exon	166741560	166742308	.	-	.	gene_id GRMZM2G147544; transcript_id TCONS_00042856; exon_number 1; oId CUFF.37410.1; tss_id TSS38784;
8	Cufflinks	exon	166742408	166743934	.	-	.	gene_id GRMZM2G147544; transcript_id TCONS_00042856; exon_number 2; oId CUFF.37410.1; tss_id TSS38784;
8	Cufflinks	exon	168042162	168044284	.	-	.	gene_id XLOC_037608; transcript_id TCONS_00042890; exon_number 1; oId CUFF.37477.1; tss_id TSS38815;

8	Cufflinks	exon	170179327	170180802	.	-	.	gene_id AC218972.3_FG002; transcript_id TCONS_00042949; exon_number 1; oId CUFF.37563.1; tss_id TSS38868;
8	Cufflinks	exon	170180924	170181072	.	-	.	gene_id AC218972.3_FG002; transcript_id TCONS_00042949; exon_number 2; oId CUFF.37563.1; tss_id TSS38868;
8	Cufflinks	exon	170181150	170181277	.	-	.	gene_id AC218972.3_FG002; transcript_id TCONS_00042949; exon_number 3; oId CUFF.37563.1; tss_id TSS38868;
8	Cufflinks	exon	170197942	170198883	.	-	.	gene_id AC218972.3_FG004; transcript_id TCONS_00042951; exon_number 1; oId CUFF.37570.1; tss_id TSS38870;
8	Cufflinks	exon	170199787	170201141	.	-	.	gene_id AC218972.3_FG004; transcript_id TCONS_00042951; exon_number 2; oId CUFF.37570.1; tss_id TSS38870;
9	Cufflinks	exon	7393694	7394149	.	+	.	gene_id GRMZM2G106303; transcript_id TCONS_00043176; exon_number 1; oId CUFF.37947.1; tss_id TSS39076;
9	Cufflinks	exon	7394243	7395273	.	+	.	gene_id GRMZM2G106303; transcript_id TCONS_00043176; exon_number 2; oId CUFF.37947.1; tss_id TSS39076;
9	Cufflinks	exon	9510044	9510759	.	+	.	gene_id GRMZM5G864689; transcript_id TCONS_00043206; exon_number 1; oId CUFF.38008.1; tss_id TSS39103;
9	Cufflinks	exon	9511244	9511384	.	+	.	gene_id GRMZM5G864689; transcript_id TCONS_00043206; exon_number 2; oId CUFF.38008.1; tss_id TSS39103;
9	Cufflinks	exon	9511466	9511570	.	+	.	gene_id GRMZM5G864689; transcript_id TCONS_00043206; exon_number 3; oId CUFF.38008.1; tss_id TSS39103;
9	Cufflinks	exon	9511641	9511691	.	+	.	gene_id GRMZM5G864689; transcript_id TCONS_00043206; exon_number 4; oId CUFF.38008.1; tss_id TSS39103;
9	Cufflinks	exon	9512032	9512079	.	+	.	gene_id GRMZM5G864689; transcript_id TCONS_00043206; exon_number 5; oId CUFF.38008.1; tss_id TSS39103;
9	Cufflinks	exon	9512153	9512356	.	+	.	gene_id GRMZM5G864689; transcript_id TCONS_00043206; exon_number 6; oId CUFF.38008.1; tss_id TSS39103;
9	Cufflinks	exon	9512513	9513407	.	+	.	gene_id GRMZM5G864689; transcript_id TCONS_00043206; exon_number 7; oId CUFF.38008.1; tss_id TSS39103;
9	Cufflinks	exon	14119204	14121439	.	+	.	gene_id GRMZM2G009139; transcript_id TCONS_00043268; exon_number 1; oId CUFF.38143.1; tss_id TSS39159;
9	Cufflinks	exon	15616216	15616513	.	+	.	gene_id GRMZM5G831993; transcript_id TCONS_00043291; exon_number 1; oId CUFF.38187.1; tss_id TSS39181;
9	Cufflinks	exon	15617383	15617652	.	+	.	gene_id GRMZM5G831993; transcript_id TCONS_00043291; exon_number 2; oId CUFF.38187.1; tss_id TSS39181;
9	Cufflinks	exon	15618095	15618318	.	+	.	gene_id GRMZM5G831993; transcript_id TCONS_00043291; exon_number 3; oId CUFF.38187.1; tss_id TSS39181;
9	Cufflinks	exon	15620114	15621318	.	+	.	gene_id GRMZM5G831993; transcript_id TCONS_00043291; exon_number 4; oId CUFF.38187.1; tss_id TSS39181;
9	Cufflinks	exon	16001623	16002878	.	+	.	gene_id GRMZM2G005080; transcript_id TCONS_00043298; exon_number 1; oId CUFF.38196.1; tss_id TSS39188;
9	Cufflinks	exon	16685283	16685705	.	+	.	gene_id GRMZM2G075488; transcript_id TCONS_00043311; exon_number 1; oId CUFF.38258.1; tss_id TSS39200;
9	Cufflinks	exon	16686444	16686580	.	+	.	gene_id GRMZM2G075488; transcript_id TCONS_00043311; exon_number 2; oId CUFF.38258.1; tss_id TSS39200;
9	Cufflinks	exon	16686686	16686859	.	+	.	gene_id GRMZM2G075488; transcript_id TCONS_00043311; exon_number 3; oId CUFF.38258.1; tss_id TSS39200;
9	Cufflinks	exon	16687443	16687679	.	+	.	gene_id GRMZM2G075488; transcript_id TCONS_00043311; exon_number 4; oId CUFF.38258.1; tss_id TSS39200;
9	Cufflinks	exon	16687751	16687851	.	+	.	gene_id GRMZM2G075488; transcript_id TCONS_00043311; exon_number 5; oId CUFF.38258.1; tss_id TSS39200;
9	Cufflinks	exon	16687952	16688024	.	+	.	gene_id GRMZM2G075488; transcript_id TCONS_00043311; exon_number 6; oId CUFF.38258.1; tss_id TSS39200;
9	Cufflinks	exon	16688104	16688172	.	+	.	gene_id GRMZM2G075488; transcript_id TCONS_00043311; exon_number 7; oId CUFF.38258.1; tss_id TSS39200;
9	Cufflinks	exon	16688263	16688370	.	+	.	gene_id GRMZM2G075488; transcript_id TCONS_00043311; exon_number 8; oId CUFF.38258.1; tss_id TSS39200;
9	Cufflinks	exon	16688584	16688709	.	+	.	gene_id GRMZM2G075488; transcript_id TCONS_00043311; exon_number 9; oId CUFF.38258.1; tss_id TSS39200;
9	Cufflinks	exon	16688987	16689432	.	+	.	gene_id GRMZM2G075488; transcript_id TCONS_00043311; exon_number 10; oId CUFF.38258.1; tss_id TSS39200;
9	Cufflinks	exon	24486952	24487411	.	+	.	gene_id GRMZM2G050166; transcript_id TCONS_00043444; exon_number 1; oId CUFF.38469.1; tss_id TSS39319;
9	Cufflinks	exon	24489164	24489369	.	+	.	gene_id GRMZM2G050166; transcript_id TCONS_00043444; exon_number 2; oId CUFF.38469.1; tss_id TSS39319;
9	Cufflinks	exon	24489600	24489698	.	+	.	gene_id GRMZM2G050166; transcript_id TCONS_00043444; exon_number 3; oId CUFF.38469.1; tss_id TSS39319;
9	Cufflinks	exon	24490060	24490133	.	+	.	gene_id GRMZM2G050166; transcript_id TCONS_00043444; exon_number 4; oId CUFF.38469.1; tss_id TSS39319;
9	Cufflinks	exon	24490538	24491050	.	+	.	gene_id GRMZM2G050166; transcript_id TCONS_00043444; exon_number 5; oId CUFF.38469.1; tss_id TSS39319;
9	Cufflinks	exon	25266852	25267368	.	+	.	gene_id GRMZM2G071491; transcript_id TCONS_00043461; exon_number 1; oId CUFF.38486.2; tss_id TSS39335;

9	Cufflinks	exon	25267521	25267589	.	+	.	gene_id GRMZM2G071491; transcript_id TCONS_00043461; exon_number 2; oId CUFF.38486.2; tss_id TSS39335;
9	Cufflinks	exon	25268335	25268435	.	+	.	gene_id GRMZM2G071491; transcript_id TCONS_00043461; exon_number 3; oId CUFF.38486.2; tss_id TSS39335;
9	Cufflinks	exon	25268561	25268654	.	+	.	gene_id GRMZM2G071491; transcript_id TCONS_00043461; exon_number 4; oId CUFF.38486.2; tss_id TSS39335;
9	Cufflinks	exon	25268741	25268810	.	+	.	gene_id GRMZM2G071491; transcript_id TCONS_00043461; exon_number 5; oId CUFF.38486.2; tss_id TSS39335;
9	Cufflinks	exon	25268923	25270126	.	+	.	gene_id GRMZM2G071491; transcript_id TCONS_00043461; exon_number 6; oId CUFF.38486.2; tss_id TSS39335;
9	Cufflinks	exon	25533695	25534397	.	+	.	gene_id GRMZM2G083711; transcript_id TCONS_00043466; exon_number 1; oId CUFF.38473.1; tss_id TSS39340;
9	Cufflinks	exon	25534534	25535338	.	+	.	gene_id GRMZM2G083711; transcript_id TCONS_00043466; exon_number 2; oId CUFF.38473.1; tss_id TSS39340;
9	Cufflinks	exon	26833575	26833754	.	+	.	gene_id GRMZM2G475293; transcript_id TCONS_00043487; exon_number 1; oId CUFF.38513.2; tss_id TSS39359;
9	Cufflinks	exon	26833964	26834967	.	+	.	gene_id GRMZM2G475293; transcript_id TCONS_00043487; exon_number 2; oId CUFF.38513.2; tss_id TSS39359;
9	Cufflinks	exon	34129749	34130129	.	+	.	gene_id GRMZM2G058675; transcript_id TCONS_00043549; exon_number 1; oId CUFF.38623.1; tss_id TSS39413;
9	Cufflinks	exon	34132209	34132355	.	+	.	gene_id GRMZM2G058675; transcript_id TCONS_00043549; exon_number 2; oId CUFF.38623.1; tss_id TSS39413;
9	Cufflinks	exon	34132882	34133024	.	+	.	gene_id GRMZM2G058675; transcript_id TCONS_00043549; exon_number 3; oId CUFF.38623.1; tss_id TSS39413;
9	Cufflinks	exon	34133112	34133265	.	+	.	gene_id GRMZM2G058675; transcript_id TCONS_00043549; exon_number 4; oId CUFF.38623.1; tss_id TSS39413;
9	Cufflinks	exon	34133373	34133602	.	+	.	gene_id GRMZM2G058675; transcript_id TCONS_00043549; exon_number 5; oId CUFF.38623.1; tss_id TSS39413;
9	Cufflinks	exon	34141787	34141876	.	+	.	gene_id GRMZM2G058675; transcript_id TCONS_00043549; exon_number 6; oId CUFF.38623.1; tss_id TSS39413;
9	Cufflinks	exon	34141974	34142147	.	+	.	gene_id GRMZM2G058675; transcript_id TCONS_00043549; exon_number 7; oId CUFF.38623.1; tss_id TSS39413;
9	Cufflinks	exon	34142240	34142377	.	+	.	gene_id GRMZM2G058675; transcript_id TCONS_00043549; exon_number 8; oId CUFF.38623.1; tss_id TSS39413;
9	Cufflinks	exon	34142722	34142859	.	+	.	gene_id GRMZM2G058675; transcript_id TCONS_00043549; exon_number 9; oId CUFF.38623.1; tss_id TSS39413;
9	Cufflinks	exon	34142978	34143039	.	+	.	gene_id GRMZM2G058675; transcript_id TCONS_00043549; exon_number 10; oId CUFF.38623.1; tss_id TSS39413;
9	Cufflinks	exon	34143163	34143684	.	+	.	gene_id GRMZM2G058675; transcript_id TCONS_00043549; exon_number 11; oId CUFF.38623.1; tss_id TSS39413;
9	Cufflinks	exon	38545694	38547957	.	+	.	gene_id XLOC_038209; transcript_id TCONS_00043580; exon_number 1; oId CUFF.38660.1; tss_id TSS39442;
9	Cufflinks	exon	43626080	43627895	.	+	.	gene_id XLOC_038228; transcript_id TCONS_00043604; exon_number 1; oId CUFF.38699.1; tss_id TSS39464;
9	Cufflinks	exon	46856491	46856618	.	+	.	gene_id XLOC_038252; transcript_id TCONS_00043632; exon_number 1; oId CUFF.38739.1; tss_id TSS39488;
9	Cufflinks	exon	46856758	46857697	.	+	.	gene_id XLOC_038252; transcript_id TCONS_00043632; exon_number 2; oId CUFF.38739.1; tss_id TSS39488;
9	Cufflinks	exon	69385542	69386024	.	+	.	gene_id XLOC_038357; transcript_id TCONS_00043749; exon_number 1; oId CUFF.38949.1; tss_id TSS39597;
9	Cufflinks	exon	78160268	78162284	.	+	.	gene_id GRMZM2G080839; transcript_id TCONS_00043797; exon_number 1; oId CUFF.39032.1; tss_id TSS39645;
9	Cufflinks	exon	87869539	87869715	.	+	.	gene_id GRMZM2G022558; transcript_id TCONS_00043849; exon_number 1; oId CUFF.39123.1; tss_id TSS39690;
9	Cufflinks	exon	87869863	87872095	.	+	.	gene_id GRMZM2G022558; transcript_id TCONS_00043849; exon_number 2; oId CUFF.39123.1; tss_id TSS39690;
9	Cufflinks	exon	97192447	97194746	.	+	.	gene_id GRMZM2G141288; transcript_id TCONS_00043923; exon_number 1; oId CUFF.39263.1; tss_id TSS39756;
9	Cufflinks	exon	97195489	97196646	.	+	.	gene_id GRMZM2G141288; transcript_id TCONS_00043923; exon_number 2; oId CUFF.39263.1; tss_id TSS39756;
9	Cufflinks	exon	100416810	100417202	.	+	.	gene_id GRMZM2G033555; transcript_id TCONS_00043947; exon_number 1; oId CUFF.39322.1; tss_id TSS39779;
9	Cufflinks	exon	100421334	100421503	.	+	.	gene_id GRMZM2G033555; transcript_id TCONS_00043947; exon_number 2; oId CUFF.39322.1; tss_id TSS39779;
9	Cufflinks	exon	100422065	100422250	.	+	.	gene_id GRMZM2G033555; transcript_id TCONS_00043947; exon_number 3; oId CUFF.39322.1; tss_id TSS39779;
9	Cufflinks	exon	100422368	100422530	.	+	.	gene_id GRMZM2G033555; transcript_id TCONS_00043947; exon_number 4; oId CUFF.39322.1; tss_id TSS39779;
9	Cufflinks	exon	100422681	100422867	.	+	.	gene_id GRMZM2G033555; transcript_id TCONS_00043947; exon_number 5; oId CUFF.39322.1; tss_id TSS39779;
9	Cufflinks	exon	100423261	100423707	.	+	.	gene_id GRMZM2G033555; transcript_id TCONS_00043947; exon_number 6; oId CUFF.39322.1; tss_id TSS39779;

9	Cufflinks	exon	101311298	101312871	.	+	.	gene_id GRMZM2G122447; transcript_id TCONS_00043962; exon_number 1; oId CUFF.39332.1; tss_id TSS39794;
9	Cufflinks	exon	104241255	104241444	.	+	.	gene_id GRMZM2G086773; transcript_id TCONS_00043993; exon_number 1; oId CUFF.39395.2; tss_id TSS39823;
9	Cufflinks	exon	104241535	104241715	.	+	.	gene_id GRMZM2G086773; transcript_id TCONS_00043993; exon_number 2; oId CUFF.39395.2; tss_id TSS39823;
9	Cufflinks	exon	104241897	104242124	.	+	.	gene_id GRMZM2G086773; transcript_id TCONS_00043993; exon_number 3; oId CUFF.39395.2; tss_id TSS39823;
9	Cufflinks	exon	104242256	104242635	.	+	.	gene_id GRMZM2G086773; transcript_id TCONS_00043993; exon_number 4; oId CUFF.39395.2; tss_id TSS39823;
9	Cufflinks	exon	104244053	104244459	.	+	.	gene_id GRMZM2G086773; transcript_id TCONS_00043993; exon_number 5; oId CUFF.39395.2; tss_id TSS39823;
9	Cufflinks	exon	107641650	107641920	.	+	.	gene_id GRMZM2G139082; transcript_id TCONS_00044015; exon_number 1; oId CUFF.39440.1; tss_id TSS39843;
9	Cufflinks	exon	107642090	107642411	.	+	.	gene_id GRMZM2G139082; transcript_id TCONS_00044015; exon_number 2; oId CUFF.39440.1; tss_id TSS39843;
9	Cufflinks	exon	107642937	107643019	.	+	.	gene_id GRMZM2G139082; transcript_id TCONS_00044015; exon_number 3; oId CUFF.39440.1; tss_id TSS39843;
9	Cufflinks	exon	107643149	107643157	.	+	.	gene_id GRMZM2G139082; transcript_id TCONS_00044015; exon_number 4; oId CUFF.39440.1; tss_id TSS39843;
9	Cufflinks	exon	107643316	107643404	.	+	.	gene_id GRMZM2G139082; transcript_id TCONS_00044015; exon_number 5; oId CUFF.39440.1; tss_id TSS39843;
9	Cufflinks	exon	107643500	107643573	.	+	.	gene_id GRMZM2G139082; transcript_id TCONS_00044015; exon_number 6; oId CUFF.39440.1; tss_id TSS39843;
9	Cufflinks	exon	107643791	107643841	.	+	.	gene_id GRMZM2G139082; transcript_id TCONS_00044015; exon_number 7; oId CUFF.39440.1; tss_id TSS39843;
9	Cufflinks	exon	107644057	107644133	.	+	.	gene_id GRMZM2G139082; transcript_id TCONS_00044015; exon_number 8; oId CUFF.39440.1; tss_id TSS39843;
9	Cufflinks	exon	107644245	107645156	.	+	.	gene_id GRMZM2G139082; transcript_id TCONS_00044015; exon_number 9; oId CUFF.39440.1; tss_id TSS39843;
9	Cufflinks	exon	108447914	108448771	.	+	.	gene_id GRMZM2G038783; transcript_id TCONS_00044023; exon_number 1; oId CUFF.39466.1; tss_id TSS39851;
9	Cufflinks	exon	108448879	108449016	.	+	.	gene_id GRMZM2G038783; transcript_id TCONS_00044023; exon_number 2; oId CUFF.39466.1; tss_id TSS39851;
9	Cufflinks	exon	108449121	108449734	.	+	.	gene_id GRMZM2G038783; transcript_id TCONS_00044023; exon_number 3; oId CUFF.39466.1; tss_id TSS39851;
9	Cufflinks	exon	109332530	109333186	.	+	.	gene_id GRMZM2G095302; transcript_id TCONS_00044039; exon_number 1; oId CUFF.39495.1; tss_id TSS39863;
9	Cufflinks	exon	109333395	109333457	.	+	.	gene_id GRMZM2G095302; transcript_id TCONS_00044039; exon_number 2; oId CUFF.39495.1; tss_id TSS39863;
9	Cufflinks	exon	109334233	109334904	.	+	.	gene_id GRMZM2G095302; transcript_id TCONS_00044039; exon_number 3; oId CUFF.39495.1; tss_id TSS39863;
9	Cufflinks	exon	109335378	109335632	.	+	.	gene_id GRMZM2G095302; transcript_id TCONS_00044039; exon_number 4; oId CUFF.39495.1; tss_id TSS39863;
9	Cufflinks	exon	109336046	109336200	.	+	.	gene_id GRMZM2G095302; transcript_id TCONS_00044039; exon_number 5; oId CUFF.39495.1; tss_id TSS39863;
9	Cufflinks	exon	109336291	109336370	.	+	.	gene_id GRMZM2G095302; transcript_id TCONS_00044039; exon_number 6; oId CUFF.39495.1; tss_id TSS39863;
9	Cufflinks	exon	109336459	109336594	.	+	.	gene_id GRMZM2G095302; transcript_id TCONS_00044039; exon_number 7; oId CUFF.39495.1; tss_id TSS39863;
9	Cufflinks	exon	109336794	109336943	.	+	.	gene_id GRMZM2G095302; transcript_id TCONS_00044039; exon_number 8; oId CUFF.39495.1; tss_id TSS39863;
9	Cufflinks	exon	109337019	109337572	.	+	.	gene_id GRMZM2G095302; transcript_id TCONS_00044039; exon_number 9; oId CUFF.39495.1; tss_id TSS39863;
9	Cufflinks	exon	113514930	113515472	.	+	.	gene_id GRMZM2G144841; transcript_id TCONS_00044086; exon_number 1; oId CUFF.39576.1; tss_id TSS39904;
9	Cufflinks	exon	113516635	113516700	.	+	.	gene_id GRMZM2G144841; transcript_id TCONS_00044086; exon_number 2; oId CUFF.39576.1; tss_id TSS39904;
9	Cufflinks	exon	113516774	113516838	.	+	.	gene_id GRMZM2G144841; transcript_id TCONS_00044086; exon_number 3; oId CUFF.39576.1; tss_id TSS39904;
9	Cufflinks	exon	113517311	113517932	.	+	.	gene_id GRMZM2G144841; transcript_id TCONS_00044086; exon_number 4; oId CUFF.39576.1; tss_id TSS39904;
9	Cufflinks	exon	113514975	113515472	.	+	.	gene_id GRMZM2G144841; transcript_id TCONS_00044087; exon_number 1; oId CUFF.39576.2; tss_id TSS39904;
9	Cufflinks	exon	113516774	113516838	.	+	.	gene_id GRMZM2G144841; transcript_id TCONS_00044087; exon_number 2; oId CUFF.39576.2; tss_id TSS39904;
9	Cufflinks	exon	113517311	113517932	.	+	.	gene_id GRMZM2G144841; transcript_id TCONS_00044087; exon_number 3; oId CUFF.39576.2; tss_id TSS39904;
9	Cufflinks	exon	117079453	117080654	.	+	.	gene_id XLOC_038710; transcript_id TCONS_00044144; exon_number 1; oId CUFF.39654.1; tss_id TSS39961;
9	Cufflinks	exon	124820597	124822010	.	+	.	gene_id GRMZM2G359033; transcript_id TCONS_00044243; exon_number 1; oId CUFF.39806.1; tss_id TSS40044;

9	Cufflinks	exon	124924627	124925337	.	+	.	gene_id XLOC_038795; transcript_id TCONS_00044247; exon_number 1; oId CUFF.39810.1; tss_id TSS40047;
9	Cufflinks	exon	127611784	127613690	.	+	.	gene_id GRMZM2G030458; transcript_id TCONS_00044282; exon_number 1; oId CUFF.39870.1; tss_id TSS40076;
9	Cufflinks	exon	128348131	128348315	.	+	.	gene_id GRMZM2G157683; transcript_id TCONS_00044292; exon_number 1; oId CUFF.39898.1; tss_id TSS40086;
9	Cufflinks	exon	128348393	128348590	.	+	.	gene_id GRMZM2G157683; transcript_id TCONS_00044292; exon_number 2; oId CUFF.39898.1; tss_id TSS40086;
9	Cufflinks	exon	128348692	128349850	.	+	.	gene_id GRMZM2G157683; transcript_id TCONS_00044292; exon_number 3; oId CUFF.39898.1; tss_id TSS40086;
9	Cufflinks	exon	130353591	130353740	.	+	.	gene_id GRMZM2G121360; transcript_id TCONS_00044330; exon_number 1; oId CUFF.39964.1; tss_id TSS40119;
9	Cufflinks	exon	130353877	130353952	.	+	.	gene_id GRMZM2G121360; transcript_id TCONS_00044330; exon_number 2; oId CUFF.39964.1; tss_id TSS40119;
9	Cufflinks	exon	130354057	130355317	.	+	.	gene_id GRMZM2G121360; transcript_id TCONS_00044330; exon_number 3; oId CUFF.39964.1; tss_id TSS40119;
9	Cufflinks	exon	130759495	130759878	.	+	.	gene_id GRMZM2G134107; transcript_id TCONS_00044332; exon_number 1; oId CUFF.39968.1; tss_id TSS40121;
9	Cufflinks	exon	130760380	130760703	.	+	.	gene_id GRMZM2G134107; transcript_id TCONS_00044332; exon_number 2; oId CUFF.39968.1; tss_id TSS40121;
9	Cufflinks	exon	139138144	139138561	.	+	.	gene_id XLOC_038971; transcript_id TCONS_00044447; exon_number 1; oId CUFF.40201.1; tss_id TSS40226;
9	Cufflinks	exon	139445962	139446689	.	+	.	gene_id XLOC_038977; transcript_id TCONS_00044453; exon_number 1; oId CUFF.40215.1; tss_id TSS40232;
9	Cufflinks	exon	139870591	139870925	.	+	.	gene_id GRMZM2G103490; transcript_id TCONS_00044465; exon_number 1; oId CUFF.40228.1; tss_id TSS40241;
9	Cufflinks	exon	139871289	139873524	.	+	.	gene_id GRMZM2G103490; transcript_id TCONS_00044465; exon_number 2; oId CUFF.40228.1; tss_id TSS40241;
9	Cufflinks	exon	144209790	144210072	.	+	.	gene_id GRMZM2G178279; transcript_id TCONS_00044556; exon_number 1; oId CUFF.40388.1; tss_id TSS40322;
9	Cufflinks	exon	144210278	144210513	.	+	.	gene_id GRMZM2G178279; transcript_id TCONS_00044556; exon_number 2; oId CUFF.40388.1; tss_id TSS40322;
9	Cufflinks	exon	144210699	144211194	.	+	.	gene_id GRMZM2G178279; transcript_id TCONS_00044556; exon_number 3; oId CUFF.40388.1; tss_id TSS40322;
9	Cufflinks	exon	144211290	144211480	.	+	.	gene_id GRMZM2G178279; transcript_id TCONS_00044556; exon_number 4; oId CUFF.40388.1; tss_id TSS40322;
9	Cufflinks	exon	144212401	144213235	.	+	.	gene_id GRMZM2G178279; transcript_id TCONS_00044556; exon_number 5; oId CUFF.40388.1; tss_id TSS40322;
9	Cufflinks	exon	144855111	144855542	.	+	.	gene_id GRMZM2G177914; transcript_id TCONS_00044566; exon_number 1; oId CUFF.40414.1; tss_id TSS40332;
9	Cufflinks	exon	144855742	144855784	.	+	.	gene_id GRMZM2G177914; transcript_id TCONS_00044566; exon_number 2; oId CUFF.40414.1; tss_id TSS40332;
9	Cufflinks	exon	144855858	144855919	.	+	.	gene_id GRMZM2G177914; transcript_id TCONS_00044566; exon_number 3; oId CUFF.40414.1; tss_id TSS40332;
9	Cufflinks	exon	144856135	144856231	.	+	.	gene_id GRMZM2G177914; transcript_id TCONS_00044566; exon_number 4; oId CUFF.40414.1; tss_id TSS40332;
9	Cufflinks	exon	144856631	144856701	.	+	.	gene_id GRMZM2G177914; transcript_id TCONS_00044566; exon_number 5; oId CUFF.40414.1; tss_id TSS40332;
9	Cufflinks	exon	144857724	144857815	.	+	.	gene_id GRMZM2G177914; transcript_id TCONS_00044566; exon_number 6; oId CUFF.40414.1; tss_id TSS40332;
9	Cufflinks	exon	144857900	144858225	.	+	.	gene_id GRMZM2G177914; transcript_id TCONS_00044566; exon_number 7; oId CUFF.40414.1; tss_id TSS40332;
9	Cufflinks	exon	145045717	145046621	.	+	.	gene_id GRMZM2G105834; transcript_id TCONS_00044570; exon_number 1; oId CUFF.40419.2; tss_id TSS40334;
9	Cufflinks	exon	145046899	145046978	.	+	.	gene_id GRMZM2G105834; transcript_id TCONS_00044570; exon_number 2; oId CUFF.40419.2; tss_id TSS40334;
9	Cufflinks	exon	145047159	145047357	.	+	.	gene_id GRMZM2G105834; transcript_id TCONS_00044570; exon_number 3; oId CUFF.40419.2; tss_id TSS40334;
9	Cufflinks	exon	145045717	145046978	.	+	.	gene_id GRMZM2G105834; transcript_id TCONS_00044569; exon_number 1; oId CUFF.40419.1; tss_id TSS40334;
9	Cufflinks	exon	145047159	145047357	.	+	.	gene_id GRMZM2G105834; transcript_id TCONS_00044569; exon_number 2; oId CUFF.40419.1; tss_id TSS40334;
9	Cufflinks	exon	145424529	145425175	.	+	.	gene_id GRMZM2G091742; transcript_id TCONS_00044574; exon_number 1; oId CUFF.40425.1; tss_id TSS40338;
9	Cufflinks	exon	145425317	145426536	.	+	.	gene_id GRMZM2G091742; transcript_id TCONS_00044574; exon_number 2; oId CUFF.40425.1; tss_id TSS40338;
9	Cufflinks	exon	149512257	149512858	.	+	.	gene_id GRMZM2G004466; transcript_id TCONS_00044659; exon_number 1; oId CUFF.40587.1; tss_id TSS40411;
9	Cufflinks	exon	149512955	149512987	.	+	.	gene_id GRMZM2G004466; transcript_id TCONS_00044659; exon_number 2; oId CUFF.40587.1; tss_id TSS40411;
9	Cufflinks	exon	149513057	149513861	.	+	.	gene_id GRMZM2G004466; transcript_id TCONS_00044659; exon_number 3; oId CUFF.40587.1; tss_id TSS40411;

9	Cufflinks	exon	151382817	151383310	.	+	.	gene_id XLOC_039183; transcript_id TCONS_00044702; exon_number 1; oId CUFF.40666.1; tss_id TSS40451;
9	Cufflinks	exon	151539092	151540031	.	+	.	gene_id GRMZM2G178787; transcript_id TCONS_00044709; exon_number 1; oId CUFF.40717.2; tss_id TSS40457;
9	Cufflinks	exon	151540677	151541035	.	+	.	gene_id GRMZM2G178787; transcript_id TCONS_00044709; exon_number 2; oId CUFF.40717.2; tss_id TSS40457;
9	Cufflinks	exon	151541133	151541268	.	+	.	gene_id GRMZM2G178787; transcript_id TCONS_00044709; exon_number 3; oId CUFF.40717.2; tss_id TSS40457;
9	Cufflinks	exon	151541367	151541503	.	+	.	gene_id GRMZM2G178787; transcript_id TCONS_00044709; exon_number 4; oId CUFF.40717.2; tss_id TSS40457;
9	Cufflinks	exon	151541606	151543047	.	+	.	gene_id GRMZM2G178787; transcript_id TCONS_00044709; exon_number 5; oId CUFF.40717.2; tss_id TSS40457;
9	Cufflinks	exon	151692379	151692756	.	+	.	gene_id XLOC_039195; transcript_id TCONS_00044718; exon_number 1; oId CUFF.40702.1; tss_id TSS40464;
9	Cufflinks	exon	151998333	152000250	.	+	.	gene_id GRMZM2G126682; transcript_id TCONS_00044733; exon_number 1; oId CUFF.40720.1; tss_id TSS40477;
9	Cufflinks	exon	154723964	154724276	.	+	.	gene_id GRMZM2G393334; transcript_id TCONS_00044815; exon_number 1; oId CUFF.40901.1; tss_id TSS40547;
9	Cufflinks	exon	154724427	154724557	.	+	.	gene_id GRMZM2G393334; transcript_id TCONS_00044815; exon_number 2; oId CUFF.40901.1; tss_id TSS40547;
9	Cufflinks	exon	154724673	154724738	.	+	.	gene_id GRMZM2G393334; transcript_id TCONS_00044815; exon_number 3; oId CUFF.40901.1; tss_id TSS40547;
9	Cufflinks	exon	154725084	154725193	.	+	.	gene_id GRMZM2G393334; transcript_id TCONS_00044815; exon_number 4; oId CUFF.40901.1; tss_id TSS40547;
9	Cufflinks	exon	154725389	154725449	.	+	.	gene_id GRMZM2G393334; transcript_id TCONS_00044815; exon_number 5; oId CUFF.40901.1; tss_id TSS40547;
9	Cufflinks	exon	154725648	154725731	.	+	.	gene_id GRMZM2G393334; transcript_id TCONS_00044815; exon_number 6; oId CUFF.40901.1; tss_id TSS40547;
9	Cufflinks	exon	154725967	154726023	.	+	.	gene_id GRMZM2G393334; transcript_id TCONS_00044815; exon_number 7; oId CUFF.40901.1; tss_id TSS40547;
9	Cufflinks	exon	154726667	154726727	.	+	.	gene_id GRMZM2G393334; transcript_id TCONS_00044815; exon_number 8; oId CUFF.40901.1; tss_id TSS40547;
9	Cufflinks	exon	154726894	154726940	.	+	.	gene_id GRMZM2G393334; transcript_id TCONS_00044815; exon_number 9; oId CUFF.40901.1; tss_id TSS40547;
9	Cufflinks	exon	154727022	154727099	.	+	.	gene_id GRMZM2G393334; transcript_id TCONS_00044815; exon_number 10; oId CUFF.40901.1; tss_id TSS40547;
9	Cufflinks	exon	154727185	154727349	.	+	.	gene_id GRMZM2G393334; transcript_id TCONS_00044815; exon_number 11; oId CUFF.40901.1; tss_id TSS40547;
9	Cufflinks	exon	154727425	154727670	.	+	.	gene_id GRMZM2G393334; transcript_id TCONS_00044815; exon_number 12; oId CUFF.40901.1; tss_id TSS40547;
9	Cufflinks	exon	154727797	154727875	.	+	.	gene_id GRMZM2G393334; transcript_id TCONS_00044815; exon_number 13; oId CUFF.40901.1; tss_id TSS40547;
9	Cufflinks	exon	154728115	154728267	.	+	.	gene_id GRMZM2G393334; transcript_id TCONS_00044815; exon_number 14; oId CUFF.40901.1; tss_id TSS40547;
9	Cufflinks	exon	154728401	154728510	.	+	.	gene_id GRMZM2G393334; transcript_id TCONS_00044815; exon_number 15; oId CUFF.40901.1; tss_id TSS40547;
9	Cufflinks	exon	154728872	154729849	.	+	.	gene_id GRMZM2G393334; transcript_id TCONS_00044815; exon_number 16; oId CUFF.40901.1; tss_id TSS40547;
9	Cufflinks	exon	155855551	155856182	.	+	.	gene_id GRMZM2G462243; transcript_id TCONS_00044845; exon_number 1; oId CUFF.40903.1; tss_id TSS40573;
9	Cufflinks	exon	155866514	155867635	.	+	.	gene_id GRMZM2G161905; transcript_id TCONS_00044848; exon_number 1; oId CUFF.40918.1; tss_id TSS40576;
9	Cufflinks	exon	9955287	9956481	.	-	.	gene_id XLOC_039408; transcript_id TCONS_00044966; exon_number 1; oId CUFF.38013.1; tss_id TSS40683;
9	Cufflinks	exon	11715793	11716391	.	-	.	gene_id GRMZM2G177231; transcript_id TCONS_00045010; exon_number 1; oId CUFF.38073.1; tss_id TSS40726;
9	Cufflinks	exon	11716416	11716637	.	-	.	gene_id GRMZM2G177231; transcript_id TCONS_00045010; exon_number 2; oId CUFF.38073.1; tss_id TSS40726;
9	Cufflinks	exon	11718004	11718138	.	-	.	gene_id GRMZM2G177231; transcript_id TCONS_00045010; exon_number 3; oId CUFF.38073.1; tss_id TSS40726;
9	Cufflinks	exon	11718221	11718341	.	-	.	gene_id GRMZM2G177231; transcript_id TCONS_00045010; exon_number 4; oId CUFF.38073.1; tss_id TSS40726;
9	Cufflinks	exon	11718443	11718467	.	-	.	gene_id GRMZM2G177231; transcript_id TCONS_00045010; exon_number 5; oId CUFF.38073.1; tss_id TSS40726;
9	Cufflinks	exon	11718616	11718713	.	-	.	gene_id GRMZM2G177231; transcript_id TCONS_00045010; exon_number 6; oId CUFF.38073.1; tss_id TSS40726;
9	Cufflinks	exon	11718977	11719506	.	-	.	gene_id GRMZM2G177231; transcript_id TCONS_00045010; exon_number 7; oId CUFF.38073.1; tss_id TSS40726;
9	Cufflinks	exon	11719601	11720000	.	-	.	gene_id GRMZM2G177231; transcript_id TCONS_00045010; exon_number 8; oId CUFF.38073.1; tss_id TSS40726;
9	Cufflinks	exon	16001752	16002137	.	-	.	gene_id XLOC_039514; transcript_id TCONS_00045089; exon_number 1; oId CUFF.38197.1; tss_id TSS40791;

9	Cufflinks	exon	19034946	19035553	.	-	.	gene_id GRMZM2G028594; transcript_id TCONS_00045135; exon_number 1; oId CUFF.38294.1; tss_id TSS40830;
9	Cufflinks	exon	19036124	19036729	.	-	.	gene_id GRMZM2G028594; transcript_id TCONS_00045135; exon_number 2; oId CUFF.38294.1; tss_id TSS40830;
9	Cufflinks	exon	19040560	19040945	.	-	.	gene_id GRMZM2G028594; transcript_id TCONS_00045135; exon_number 3; oId CUFF.38294.1; tss_id TSS40830;
9	Cufflinks	exon	24558416	24559236	.	-	.	gene_id XLOC_039625; transcript_id TCONS_00045218; exon_number 1; oId CUFF.38438.1; tss_id TSS40906;
9	Cufflinks	exon	25268948	25270538	.	-	.	gene_id GRMZM2G071602; transcript_id TCONS_00045232; exon_number 1; oId CUFF.38487.2; tss_id TSS40917;
9	Cufflinks	exon	25270655	25270763	.	-	.	gene_id GRMZM2G071602; transcript_id TCONS_00045232; exon_number 2; oId CUFF.38487.2; tss_id TSS40917;
9	Cufflinks	exon	25270982	25271109	.	-	.	gene_id GRMZM2G071602; transcript_id TCONS_00045232; exon_number 3; oId CUFF.38487.2; tss_id TSS40917;
9	Cufflinks	exon	25271277	25271333	.	-	.	gene_id GRMZM2G071602; transcript_id TCONS_00045232; exon_number 4; oId CUFF.38487.2; tss_id TSS40917;
9	Cufflinks	exon	25271430	25271503	.	-	.	gene_id GRMZM2G071602; transcript_id TCONS_00045232; exon_number 5; oId CUFF.38487.2; tss_id TSS40917;
9	Cufflinks	exon	25271693	25271954	.	-	.	gene_id GRMZM2G071602; transcript_id TCONS_00045232; exon_number 6; oId CUFF.38487.2; tss_id TSS40917;
9	Cufflinks	exon	25272156	25272348	.	-	.	gene_id GRMZM2G071602; transcript_id TCONS_00045232; exon_number 7; oId CUFF.38487.2; tss_id TSS40917;
9	Cufflinks	exon	25272731	25272807	.	-	.	gene_id GRMZM2G071602; transcript_id TCONS_00045232; exon_number 8; oId CUFF.38487.2; tss_id TSS40917;
9	Cufflinks	exon	25268948	25270538	.	-	.	gene_id GRMZM2G071602; transcript_id TCONS_00045233; exon_number 1; oId CUFF.38487.1; tss_id TSS40917;
9	Cufflinks	exon	25270655	25270763	.	-	.	gene_id GRMZM2G071602; transcript_id TCONS_00045233; exon_number 2; oId CUFF.38487.1; tss_id TSS40917;
9	Cufflinks	exon	25270982	25271109	.	-	.	gene_id GRMZM2G071602; transcript_id TCONS_00045233; exon_number 3; oId CUFF.38487.1; tss_id TSS40917;
9	Cufflinks	exon	25271277	25271333	.	-	.	gene_id GRMZM2G071602; transcript_id TCONS_00045233; exon_number 4; oId CUFF.38487.1; tss_id TSS40917;
9	Cufflinks	exon	25271430	25271503	.	-	.	gene_id GRMZM2G071602; transcript_id TCONS_00045233; exon_number 5; oId CUFF.38487.1; tss_id TSS40917;
9	Cufflinks	exon	25271693	25271939	.	-	.	gene_id GRMZM2G071602; transcript_id TCONS_00045233; exon_number 6; oId CUFF.38487.1; tss_id TSS40917;
9	Cufflinks	exon	25272156	25272348	.	-	.	gene_id GRMZM2G071602; transcript_id TCONS_00045233; exon_number 7; oId CUFF.38487.1; tss_id TSS40917;
9	Cufflinks	exon	25272731	25272830	.	-	.	gene_id GRMZM2G071602; transcript_id TCONS_00045233; exon_number 8; oId CUFF.38487.1; tss_id TSS40917;
9	Cufflinks	exon	25535087	25535599	.	-	.	gene_id GRMZM2G383540; transcript_id TCONS_00045236; exon_number 1; oId CUFF.38474.1; tss_id TSS40920;
9	Cufflinks	exon	25536479	25536544	.	-	.	gene_id GRMZM2G383540; transcript_id TCONS_00045236; exon_number 2; oId CUFF.38474.1; tss_id TSS40920;
9	Cufflinks	exon	25536650	25536747	.	-	.	gene_id GRMZM2G383540; transcript_id TCONS_00045236; exon_number 3; oId CUFF.38474.1; tss_id TSS40920;
9	Cufflinks	exon	25536878	25537349	.	-	.	gene_id GRMZM2G383540; transcript_id TCONS_00045236; exon_number 4; oId CUFF.38474.1; tss_id TSS40920;
9	Cufflinks	exon	38545391	38547608	.	-	.	gene_id GRMZM2G027958; transcript_id TCONS_00045326; exon_number 1; oId CUFF.38659.1; tss_id TSS41004;
9	Cufflinks	exon	43625799	43627733	.	-	.	gene_id GRMZM2G099740; transcript_id TCONS_00045346; exon_number 1; oId CUFF.38698.1; tss_id TSS41022;
9	Cufflinks	exon	46856634	46857418	.	-	.	gene_id XLOC_039757; transcript_id TCONS_00045365; exon_number 1; oId CUFF.38740.1; tss_id TSS41040;
9	Cufflinks	exon	69385549	69386024	.	-	.	gene_id XLOC_039870; transcript_id TCONS_00045489; exon_number 1; oId CUFF.38950.1; tss_id TSS41157;
9	Cufflinks	exon	75178361	75179280	.	-	.	gene_id GRMZM2G136300; transcript_id TCONS_00045512; exon_number 1; oId CUFF.39028.1; tss_id TSS41180;
9	Cufflinks	exon	75179361	75179500	.	-	.	gene_id GRMZM2G136300; transcript_id TCONS_00045512; exon_number 2; oId CUFF.39028.1; tss_id TSS41180;
9	Cufflinks	exon	75179586	75179803	.	-	.	gene_id GRMZM2G136300; transcript_id TCONS_00045512; exon_number 3; oId CUFF.39028.1; tss_id TSS41180;
9	Cufflinks	exon	75179911	75180004	.	-	.	gene_id GRMZM2G136300; transcript_id TCONS_00045512; exon_number 4; oId CUFF.39028.1; tss_id TSS41180;
9	Cufflinks	exon	75180114	75180347	.	-	.	gene_id GRMZM2G136300; transcript_id TCONS_00045512; exon_number 5; oId CUFF.39028.1; tss_id TSS41180;
9	Cufflinks	exon	75189002	75189267	.	-	.	gene_id GRMZM2G136300; transcript_id TCONS_00045512; exon_number 6; oId CUFF.39028.1; tss_id TSS41180;
9	Cufflinks	exon	88365936	88367357	.	-	.	gene_id GRMZM2G159641; transcript_id TCONS_00045579; exon_number 1; oId CUFF.39136.1; tss_id TSS41245;
9	Cufflinks	exon	88367477	88368071	.	-	.	gene_id GRMZM2G159641; transcript_id TCONS_00045579; exon_number 2; oId CUFF.39136.1; tss_id TSS41245;

9	Cufflinks	exon	89922996	89925114	.	-	.	gene_id GRMZM2G101004; transcript_id TCONS_00045595; exon_number 1; oId CUFF.39179.1; tss_id TSS41258;
9	Cufflinks	exon	89925227	89925334	.	-	.	gene_id GRMZM2G101004; transcript_id TCONS_00045595; exon_number 2; oId CUFF.39179.1; tss_id TSS41258;
9	Cufflinks	exon	89925681	89925904	.	-	.	gene_id GRMZM2G101004; transcript_id TCONS_00045595; exon_number 3; oId CUFF.39179.1; tss_id TSS41258;
9	Cufflinks	exon	89933109	89933844	.	-	.	gene_id GRMZM2G101004; transcript_id TCONS_00045595; exon_number 4; oId CUFF.39179.1; tss_id TSS41258;
9	Cufflinks	exon	97195479	97195816	.	-	.	gene_id XLOC_040016; transcript_id TCONS_00045645; exon_number 1; oId CUFF.39264.1; tss_id TSS41305;
9	Cufflinks	exon	98256395	98257044	.	-	.	gene_id XLOC_040027; transcript_id TCONS_00045656; exon_number 1; oId CUFF.39281.1; tss_id TSS41316;
9	Cufflinks	exon	98257226	98257480	.	-	.	gene_id XLOC_040027; transcript_id TCONS_00045656; exon_number 2; oId CUFF.39281.1; tss_id TSS41316;
9	Cufflinks	exon	107644186	107644947	.	-	.	gene_id XLOC_040115; transcript_id TCONS_00045752; exon_number 1; oId CUFF.39441.1; tss_id TSS41405;
9	Cufflinks	exon	109162497	109163371	.	-	.	gene_id GRMZM2G103647; transcript_id TCONS_00045773; exon_number 1; oId CUFF.39490.2; tss_id TSS41426;
9	Cufflinks	exon	109163463	109163588	.	-	.	gene_id GRMZM2G103647; transcript_id TCONS_00045773; exon_number 2; oId CUFF.39490.2; tss_id TSS41426;
9	Cufflinks	exon	109163690	109163765	.	-	.	gene_id GRMZM2G103647; transcript_id TCONS_00045773; exon_number 3; oId CUFF.39490.2; tss_id TSS41426;
9	Cufflinks	exon	109163871	109164006	.	-	.	gene_id GRMZM2G103647; transcript_id TCONS_00045773; exon_number 4; oId CUFF.39490.2; tss_id TSS41426;
9	Cufflinks	exon	109164404	109164506	.	-	.	gene_id GRMZM2G103647; transcript_id TCONS_00045773; exon_number 5; oId CUFF.39490.2; tss_id TSS41426;
9	Cufflinks	exon	109164638	109164922	.	-	.	gene_id GRMZM2G103647; transcript_id TCONS_00045773; exon_number 6; oId CUFF.39490.2; tss_id TSS41426;
9	Cufflinks	exon	109162497	109163371	.	-	.	gene_id GRMZM2G103647; transcript_id TCONS_00045774; exon_number 1; oId CUFF.39490.1; tss_id TSS41426;
9	Cufflinks	exon	109163463	109163588	.	-	.	gene_id GRMZM2G103647; transcript_id TCONS_00045774; exon_number 2; oId CUFF.39490.1; tss_id TSS41426;
9	Cufflinks	exon	109163690	109163765	.	-	.	gene_id GRMZM2G103647; transcript_id TCONS_00045774; exon_number 3; oId CUFF.39490.1; tss_id TSS41426;
9	Cufflinks	exon	109163871	109164006	.	-	.	gene_id GRMZM2G103647; transcript_id TCONS_00045774; exon_number 4; oId CUFF.39490.1; tss_id TSS41426;
9	Cufflinks	exon	109164404	109164485	.	-	.	gene_id GRMZM2G103647; transcript_id TCONS_00045774; exon_number 5; oId CUFF.39490.1; tss_id TSS41426;
9	Cufflinks	exon	109164638	109164977	.	-	.	gene_id GRMZM2G103647; transcript_id TCONS_00045774; exon_number 6; oId CUFF.39490.1; tss_id TSS41426;
9	Cufflinks	exon	109336954	109338379	.	-	.	gene_id GRMZM2G095287; transcript_id TCONS_00045779; exon_number 1; oId CUFF.39496.1; tss_id TSS41431;
9	Cufflinks	exon	109338710	109339106	.	-	.	gene_id GRMZM2G095287; transcript_id TCONS_00045779; exon_number 2; oId CUFF.39496.1; tss_id TSS41431;
9	Cufflinks	exon	109339586	109340083	.	-	.	gene_id GRMZM2G095287; transcript_id TCONS_00045779; exon_number 3; oId CUFF.39496.1; tss_id TSS41431;
9	Cufflinks	exon	111500037	111500591	.	-	.	gene_id GRMZM2G017685; transcript_id TCONS_00045807; exon_number 1; oId CUFF.39517.1; tss_id TSS41457;
9	Cufflinks	exon	112024912	112028149	.	-	.	gene_id GRMZM2G049229; transcript_id TCONS_00045812; exon_number 1; oId CUFF.39532.1; tss_id TSS41462;
9	Cufflinks	exon	113509124	113509715	.	-	.	gene_id GRMZM2G144818; transcript_id TCONS_00045824; exon_number 1; oId CUFF.39575.1; tss_id TSS41474;
9	Cufflinks	exon	113509940	113510040	.	-	.	gene_id GRMZM2G144818; transcript_id TCONS_00045824; exon_number 2; oId CUFF.39575.1; tss_id TSS41474;
9	Cufflinks	exon	113510422	113510456	.	-	.	gene_id GRMZM2G144818; transcript_id TCONS_00045824; exon_number 3; oId CUFF.39575.1; tss_id TSS41474;
9	Cufflinks	exon	113510680	113510796	.	-	.	gene_id GRMZM2G144818; transcript_id TCONS_00045824; exon_number 4; oId CUFF.39575.1; tss_id TSS41474;
9	Cufflinks	exon	113514838	113514909	.	-	.	gene_id GRMZM2G144818; transcript_id TCONS_00045824; exon_number 5; oId CUFF.39575.1; tss_id TSS41474;
9	Cufflinks	exon	113515007	113515269	.	-	.	gene_id GRMZM2G144818; transcript_id TCONS_00045824; exon_number 6; oId CUFF.39575.1; tss_id TSS41474;
9	Cufflinks	exon	114574678	114576847	.	-	.	gene_id GRMZM2G428470; transcript_id TCONS_00045846; exon_number 1; oId CUFF.39601.1; tss_id TSS41491;
9	Cufflinks	exon	122479087	122479747	.	-	.	gene_id GRMZM2G152908; transcript_id TCONS_00045920; exon_number 1; oId CUFF.39784.1; tss_id TSS41559;
9	Cufflinks	exon	122479828	122480072	.	-	.	gene_id GRMZM2G152908; transcript_id TCONS_00045920; exon_number 2; oId CUFF.39784.1; tss_id TSS41559;
9	Cufflinks	exon	122480164	122480482	.	-	.	gene_id GRMZM2G152908; transcript_id TCONS_00045920; exon_number 3; oId CUFF.39784.1; tss_id TSS41559;
9	Cufflinks	exon	122480575	122480799	.	-	.	gene_id GRMZM2G152908; transcript_id TCONS_00045920; exon_number 4; oId CUFF.39784.1; tss_id TSS41559;

9	Cufflinks	exon	122480894	122481060	.	-	.	gene_id GRMZM2G152908; transcript_id TCONS_00045920; exon_number 5; oId CUFF.39784.1; tss_id TSS41559;
9	Cufflinks	exon	122481162	122481278	.	-	.	gene_id GRMZM2G152908; transcript_id TCONS_00045920; exon_number 6; oId CUFF.39784.1; tss_id TSS41559;
9	Cufflinks	exon	122481373	122481546	.	-	.	gene_id GRMZM2G152908; transcript_id TCONS_00045920; exon_number 7; oId CUFF.39784.1; tss_id TSS41559;
9	Cufflinks	exon	122481638	122481733	.	-	.	gene_id GRMZM2G152908; transcript_id TCONS_00045920; exon_number 8; oId CUFF.39784.1; tss_id TSS41559;
9	Cufflinks	exon	122481818	122482034	.	-	.	gene_id GRMZM2G152908; transcript_id TCONS_00045920; exon_number 9; oId CUFF.39784.1; tss_id TSS41559;
9	Cufflinks	exon	122482138	122482256	.	-	.	gene_id GRMZM2G152908; transcript_id TCONS_00045920; exon_number 10; oId CUFF.39784.1; tss_id TSS41559;
9	Cufflinks	exon	122482520	122482712	.	-	.	gene_id GRMZM2G152908; transcript_id TCONS_00045920; exon_number 11; oId CUFF.39784.1; tss_id TSS41559;
9	Cufflinks	exon	122482838	122482989	.	-	.	gene_id GRMZM2G152908; transcript_id TCONS_00045920; exon_number 12; oId CUFF.39784.1; tss_id TSS41559;
9	Cufflinks	exon	122483079	122483208	.	-	.	gene_id GRMZM2G152908; transcript_id TCONS_00045920; exon_number 13; oId CUFF.39784.1; tss_id TSS41559;
9	Cufflinks	exon	122483918	122484054	.	-	.	gene_id GRMZM2G152908; transcript_id TCONS_00045920; exon_number 14; oId CUFF.39784.1; tss_id TSS41559;
9	Cufflinks	exon	122485564	122485767	.	-	.	gene_id GRMZM2G152908; transcript_id TCONS_00045920; exon_number 15; oId CUFF.39784.1; tss_id TSS41559;
9	Cufflinks	exon	126705611	126706390	.	-	.	gene_id GRMZM2G145794; transcript_id TCONS_00045979; exon_number 1; oId CUFF.39863.1; tss_id TSS41612;
9	Cufflinks	exon	126706692	126706976	.	-	.	gene_id GRMZM2G145794; transcript_id TCONS_00045979; exon_number 2; oId CUFF.39863.1; tss_id TSS41612;
9	Cufflinks	exon	126707641	126708065	.	-	.	gene_id GRMZM2G145794; transcript_id TCONS_00045979; exon_number 3; oId CUFF.39863.1; tss_id TSS41612;
9	Cufflinks	exon	126708194	126709017	.	-	.	gene_id GRMZM2G145794; transcript_id TCONS_00045979; exon_number 4; oId CUFF.39863.1; tss_id TSS41612;
9	Cufflinks	exon	129107641	129108309	.	-	.	gene_id GRMZM2G017460; transcript_id TCONS_00046019; exon_number 1; oId CUFF.39932.1; tss_id TSS41648;
9	Cufflinks	exon	129108414	129108543	.	-	.	gene_id GRMZM2G017460; transcript_id TCONS_00046019; exon_number 2; oId CUFF.39932.1; tss_id TSS41648;
9	Cufflinks	exon	129109607	129109712	.	-	.	gene_id GRMZM2G017460; transcript_id TCONS_00046019; exon_number 3; oId CUFF.39932.1; tss_id TSS41648;
9	Cufflinks	exon	129109887	129110218	.	-	.	gene_id GRMZM2G017460; transcript_id TCONS_00046019; exon_number 4; oId CUFF.39932.1; tss_id TSS41648;
9	Cufflinks	exon	138040992	138041596	.	-	.	gene_id GRMZM2G078472; transcript_id TCONS_00046184; exon_number 1; oId CUFF.40182.1; tss_id TSS41795;
9	Cufflinks	exon	138041725	138041832	.	-	.	gene_id GRMZM2G078472; transcript_id TCONS_00046184; exon_number 2; oId CUFF.40182.1; tss_id TSS41795;
9	Cufflinks	exon	138041890	138041976	.	-	.	gene_id GRMZM2G078472; transcript_id TCONS_00046184; exon_number 3; oId CUFF.40182.1; tss_id TSS41795;
9	Cufflinks	exon	138042071	138042151	.	-	.	gene_id GRMZM2G078472; transcript_id TCONS_00046184; exon_number 4; oId CUFF.40182.1; tss_id TSS41795;
9	Cufflinks	exon	138042272	138042628	.	-	.	gene_id GRMZM2G078472; transcript_id TCONS_00046184; exon_number 5; oId CUFF.40182.1; tss_id TSS41795;
9	Cufflinks	exon	138042726	138042806	.	-	.	gene_id GRMZM2G078472; transcript_id TCONS_00046184; exon_number 6; oId CUFF.40182.1; tss_id TSS41795;
9	Cufflinks	exon	138042906	138043067	.	-	.	gene_id GRMZM2G078472; transcript_id TCONS_00046184; exon_number 7; oId CUFF.40182.1; tss_id TSS41795;
9	Cufflinks	exon	138043173	138043363	.	-	.	gene_id GRMZM2G078472; transcript_id TCONS_00046184; exon_number 8; oId CUFF.40182.1; tss_id TSS41795;
9	Cufflinks	exon	138043501	138043642	.	-	.	gene_id GRMZM2G078472; transcript_id TCONS_00046184; exon_number 9; oId CUFF.40182.1; tss_id TSS41795;
9	Cufflinks	exon	138043779	138043877	.	-	.	gene_id GRMZM2G078472; transcript_id TCONS_00046184; exon_number 10; oId CUFF.40182.1; tss_id TSS41795;
9	Cufflinks	exon	138044012	138044150	.	-	.	gene_id GRMZM2G078472; transcript_id TCONS_00046184; exon_number 11; oId CUFF.40182.1; tss_id TSS41795;
9	Cufflinks	exon	138044426	138044636	.	-	.	gene_id GRMZM2G078472; transcript_id TCONS_00046184; exon_number 12; oId CUFF.40182.1; tss_id TSS41795;
9	Cufflinks	exon	138684219	138684370	.	-	.	gene_id GRMZM2G053987; transcript_id TCONS_00046196; exon_number 1; oId CUFF.40191.1; tss_id TSS41805;
9	Cufflinks	exon	138684479	138684687	.	-	.	gene_id GRMZM2G053987; transcript_id TCONS_00046196; exon_number 2; oId CUFF.40191.1; tss_id TSS41805;
9	Cufflinks	exon	138685822	138686005	.	-	.	gene_id GRMZM2G053987; transcript_id TCONS_00046196; exon_number 3; oId CUFF.40191.1; tss_id TSS41805;
9	Cufflinks	exon	138686096	138686566	.	-	.	gene_id GRMZM2G053987; transcript_id TCONS_00046196; exon_number 4; oId CUFF.40191.1; tss_id TSS41805;
9	Cufflinks	exon	138686660	138686789	.	-	.	gene_id GRMZM2G053987; transcript_id TCONS_00046196; exon_number 5; oId CUFF.40191.1; tss_id TSS41805;

9	Cufflinks	exon	138687080	138687449	.	-	.	gene_id GRMZM2G053987; transcript_id TCONS_00046196; exon_number 6; oId CUFF.40191.1; tss_id TSS41805;
9	Cufflinks	exon	139137817	139139408	.	-	.	gene_id GRMZM2G049318; transcript_id TCONS_00046203; exon_number 1; oId CUFF.40200.1; tss_id TSS41812;
9	Cufflinks	exon	139445735	139446720	.	-	.	gene_id GRMZM2G305027; transcript_id TCONS_00046210; exon_number 1; oId CUFF.40214.1; tss_id TSS41819;
9	Cufflinks	exon	140100585	140101088	.	-	.	gene_id GRMZM2G151992; transcript_id TCONS_00046227; exon_number 1; oId CUFF.40241.1; tss_id TSS41832;
9	Cufflinks	exon	140101239	140101508	.	-	.	gene_id GRMZM2G151992; transcript_id TCONS_00046227; exon_number 2; oId CUFF.40241.1; tss_id TSS41832;
9	Cufflinks	exon	140101615	140101740	.	-	.	gene_id GRMZM2G151992; transcript_id TCONS_00046227; exon_number 3; oId CUFF.40241.1; tss_id TSS41832;
9	Cufflinks	exon	140101840	140101937	.	-	.	gene_id GRMZM2G151992; transcript_id TCONS_00046227; exon_number 4; oId CUFF.40241.1; tss_id TSS41832;
9	Cufflinks	exon	140102068	140102133	.	-	.	gene_id GRMZM2G151992; transcript_id TCONS_00046227; exon_number 5; oId CUFF.40241.1; tss_id TSS41832;
9	Cufflinks	exon	140102270	140102356	.	-	.	gene_id GRMZM2G151992; transcript_id TCONS_00046227; exon_number 6; oId CUFF.40241.1; tss_id TSS41832;
9	Cufflinks	exon	140102605	140102820	.	-	.	gene_id GRMZM2G151992; transcript_id TCONS_00046227; exon_number 7; oId CUFF.40241.1; tss_id TSS41832;
9	Cufflinks	exon	141795025	141797141	.	-	.	gene_id GRMZM2G031447; transcript_id TCONS_00046270; exon_number 1; oId CUFF.40299.1; tss_id TSS41868;
9	Cufflinks	exon	142281711	142282929	.	-	.	gene_id XLOC_040562; transcript_id TCONS_00046276; exon_number 1; oId CUFF.40312.1; tss_id TSS41874;
9	Cufflinks	exon	147665984	147666434	.	-	.	gene_id GRMZM2G113205; transcript_id TCONS_00046400; exon_number 1; oId CUFF.40531.1; tss_id TSS41984;
9	Cufflinks	exon	147666514	147666610	.	-	.	gene_id GRMZM2G113205; transcript_id TCONS_00046400; exon_number 2; oId CUFF.40531.1; tss_id TSS41984;
9	Cufflinks	exon	147666740	147666829	.	-	.	gene_id GRMZM2G113205; transcript_id TCONS_00046400; exon_number 3; oId CUFF.40531.1; tss_id TSS41984;
9	Cufflinks	exon	147666999	147667439	.	-	.	gene_id GRMZM2G113205; transcript_id TCONS_00046400; exon_number 4; oId CUFF.40531.1; tss_id TSS41984;
9	Cufflinks	exon	149280835	149281531	.	-	.	gene_id GRMZM2G050159; transcript_id TCONS_00046437; exon_number 1; oId CUFF.40572.1; tss_id TSS42018;
9	Cufflinks	exon	149282127	149282968	.	-	.	gene_id GRMZM2G050159; transcript_id TCONS_00046437; exon_number 2; oId CUFF.40572.1; tss_id TSS42018;
9	Cufflinks	exon	151919604	151920602	.	-	.	gene_id XLOC_040778; transcript_id TCONS_00046524; exon_number 1; oId CUFF.40745.1; tss_id TSS42100;
9	Cufflinks	exon	152081653	152082635	.	-	.	gene_id XLOC_040788; transcript_id TCONS_00046534; exon_number 1; oId CUFF.40755.1; tss_id TSS42110;
9	Cufflinks	exon	154211297	154212031	.	-	.	gene_id GRMZM2G472696; transcript_id TCONS_00046597; exon_number 1; oId CUFF.40836.1; tss_id TSS42168;
9	Cufflinks	exon	154729257	154730168	.	-	.	gene_id GRMZM2G092497; transcript_id TCONS_00046627; exon_number 1; oId CUFF.40902.1; tss_id TSS42189;
9	Cufflinks	exon	154730251	154730294	.	-	.	gene_id GRMZM2G092497; transcript_id TCONS_00046627; exon_number 2; oId CUFF.40902.1; tss_id TSS42189;
9	Cufflinks	exon	154730410	154730472	.	-	.	gene_id GRMZM2G092497; transcript_id TCONS_00046627; exon_number 3; oId CUFF.40902.1; tss_id TSS42189;
9	Cufflinks	exon	154730579	154730717	.	-	.	gene_id GRMZM2G092497; transcript_id TCONS_00046627; exon_number 4; oId CUFF.40902.1; tss_id TSS42189;
9	Cufflinks	exon	154730812	154730880	.	-	.	gene_id GRMZM2G092497; transcript_id TCONS_00046627; exon_number 5; oId CUFF.40902.1; tss_id TSS42189;
9	Cufflinks	exon	154730996	154731263	.	-	.	gene_id GRMZM2G092497; transcript_id TCONS_00046627; exon_number 6; oId CUFF.40902.1; tss_id TSS42189;
9	Cufflinks	exon	154731339	154731438	.	-	.	gene_id GRMZM2G092497; transcript_id TCONS_00046627; exon_number 7; oId CUFF.40902.1; tss_id TSS42189;
9	Cufflinks	exon	154731521	154731780	.	-	.	gene_id GRMZM2G092497; transcript_id TCONS_00046627; exon_number 8; oId CUFF.40902.1; tss_id TSS42189;
9	Cufflinks	exon	155707392	155708469	.	-	.	gene_id GRMZM2G168355; transcript_id TCONS_00046645; exon_number 1; oId CUFF.40908.1; tss_id TSS42206;
9	Cufflinks	exon	155708565	155708760	.	-	.	gene_id GRMZM2G168355; transcript_id TCONS_00046645; exon_number 2; oId CUFF.40908.1; tss_id TSS42206;
9	Cufflinks	exon	155708889	155709337	.	-	.	gene_id GRMZM2G168355; transcript_id TCONS_00046645; exon_number 3; oId CUFF.40908.1; tss_id TSS42206;
9	Cufflinks	exon	155855551	155856028	.	-	.	gene_id XLOC_040883; transcript_id TCONS_00046650; exon_number 1; oId CUFF.40905.1; tss_id TSS42210;
1	Cufflinks	exon	2839306	2840779	.	+	.	gene_id GRMZM5G802392; transcript_id TCONS_00000045; exon_number 1; oId CUFF.189.1; tss_id TSS95;
1	Cufflinks	exon	2871385	2871794	.	+	.	gene_id GRMZM2G007025; transcript_id TCONS_00000047; exon_number 1; oId CUFF.270.1; tss_id TSS97;
1	Cufflinks	exon	2871954	2872084	.	+	.	gene_id GRMZM2G007025; transcript_id TCONS_00000047; exon_number 2; oId CUFF.270.1; tss_id TSS97;

1	Cufflinks	exon	2873169	2873326	.	+	.	gene_id GRMZM2G007025; transcript_id TCONS_00000047; exon_number 3; oId CUFF.270.1; tss_id TSS97;
1	Cufflinks	exon	2873407	2875166	.	+	.	gene_id GRMZM2G007025; transcript_id TCONS_00000047; exon_number 4; oId CUFF.270.1; tss_id TSS97;
1	Cufflinks	exon	5039291	5040037	.	+	.	gene_id GRMZM2G159477; transcript_id TCONS_00000115; exon_number 1; oId CUFF.421.1; tss_id TSS232;
1	Cufflinks	exon	5041094	5042641	.	+	.	gene_id GRMZM2G159477; transcript_id TCONS_00000115; exon_number 2; oId CUFF.421.1; tss_id TSS232;
1	Cufflinks	exon	6217891	6218279	.	+	.	gene_id XLOC_000123; transcript_id TCONS_00000142; exon_number 1; oId CUFF.516.1; tss_id TSS279;
1	Cufflinks	exon	7078162	7078239	.	+	.	gene_id GRMZM2G409976; transcript_id TCONS_00000157; exon_number 1; oId CUFF.593.3; tss_id TSS298;
1	Cufflinks	exon	7078393	7078709	.	+	.	gene_id GRMZM2G409976; transcript_id TCONS_00000157; exon_number 2; oId CUFF.593.3; tss_id TSS298;
1	Cufflinks	exon	7078940	7079085	.	+	.	gene_id GRMZM2G409976; transcript_id TCONS_00000157; exon_number 3; oId CUFF.593.3; tss_id TSS298;
1	Cufflinks	exon	7079208	7079350	.	+	.	gene_id GRMZM2G409976; transcript_id TCONS_00000157; exon_number 4; oId CUFF.593.3; tss_id TSS298;
1	Cufflinks	exon	7079430	7079553	.	+	.	gene_id GRMZM2G409976; transcript_id TCONS_00000157; exon_number 5; oId CUFF.593.3; tss_id TSS298;
1	Cufflinks	exon	7079659	7082865	.	+	.	gene_id GRMZM2G409976; transcript_id TCONS_00000157; exon_number 6; oId CUFF.593.3; tss_id TSS298;
1	Cufflinks	exon	7353118	7353430	.	+	.	gene_id GRMZM2G095968; transcript_id TCONS_00000165; exon_number 1; oId CUFF.585.1; tss_id TSS316;
1	Cufflinks	exon	7353514	7353802	.	+	.	gene_id GRMZM2G095968; transcript_id TCONS_00000165; exon_number 2; oId CUFF.585.1; tss_id TSS316;
1	Cufflinks	exon	7353906	7354050	.	+	.	gene_id GRMZM2G095968; transcript_id TCONS_00000165; exon_number 3; oId CUFF.585.1; tss_id TSS316;
1	Cufflinks	exon	7354198	7354749	.	+	.	gene_id GRMZM2G095968; transcript_id TCONS_00000165; exon_number 4; oId CUFF.585.1; tss_id TSS316;
1	Cufflinks	exon	9575028	9576757	.	+	.	gene_id GRMZM2G415390; transcript_id TCONS_00000213; exon_number 1; oId CUFF.769.1; tss_id TSS408;
1	Cufflinks	exon	10069879	10071167	.	+	.	gene_id XLOC_000190; transcript_id TCONS_00000219; exon_number 1; oId CUFF.786.1; tss_id TSS424;
1	Cufflinks	exon	15168071	15168574	.	+	.	gene_id GRMZM2G035243; transcript_id TCONS_00000323; exon_number 1; oId CUFF.1177.1; tss_id TSS654;
1	Cufflinks	exon	15168692	15168755	.	+	.	gene_id GRMZM2G035243; transcript_id TCONS_00000323; exon_number 2; oId CUFF.1177.1; tss_id TSS654;
1	Cufflinks	exon	15168926	15171112	.	+	.	gene_id GRMZM2G035243; transcript_id TCONS_00000323; exon_number 3; oId CUFF.1177.1; tss_id TSS654;
1	Cufflinks	exon	15168071	15168574	.	+	.	gene_id GRMZM2G035243; transcript_id TCONS_00000324; exon_number 1; oId CUFF.1177.2; tss_id TSS654;
1	Cufflinks	exon	15168692	15168755	.	+	.	gene_id GRMZM2G035243; transcript_id TCONS_00000324; exon_number 2; oId CUFF.1177.2; tss_id TSS654;
1	Cufflinks	exon	15168861	15171112	.	+	.	gene_id GRMZM2G035243; transcript_id TCONS_00000324; exon_number 3; oId CUFF.1177.2; tss_id TSS654;
1	Cufflinks	exon	15989656	15990015	.	+	.	gene_id GRMZM2G156486; transcript_id TCONS_00000336; exon_number 1; oId CUFF.1206.1; tss_id TSS678;
1	Cufflinks	exon	15990113	15990229	.	+	.	gene_id GRMZM2G156486; transcript_id TCONS_00000336; exon_number 2; oId CUFF.1206.1; tss_id TSS678;
1	Cufflinks	exon	15991328	15991552	.	+	.	gene_id GRMZM2G156486; transcript_id TCONS_00000336; exon_number 3; oId CUFF.1206.1; tss_id TSS678;
1	Cufflinks	exon	15991929	15992022	.	+	.	gene_id GRMZM2G156486; transcript_id TCONS_00000336; exon_number 4; oId CUFF.1206.1; tss_id TSS678;
1	Cufflinks	exon	15992313	15992387	.	+	.	gene_id GRMZM2G156486; transcript_id TCONS_00000336; exon_number 5; oId CUFF.1206.1; tss_id TSS678;
1	Cufflinks	exon	15992696	15992774	.	+	.	gene_id GRMZM2G156486; transcript_id TCONS_00000336; exon_number 6; oId CUFF.1206.1; tss_id TSS678;
1	Cufflinks	exon	15992995	15993010	.	+	.	gene_id GRMZM2G156486; transcript_id TCONS_00000336; exon_number 7; oId CUFF.1206.1; tss_id TSS678;
1	Cufflinks	exon	15993123	15993203	.	+	.	gene_id GRMZM2G156486; transcript_id TCONS_00000336; exon_number 8; oId CUFF.1206.1; tss_id TSS678;
1	Cufflinks	exon	15993399	15993481	.	+	.	gene_id GRMZM2G156486; transcript_id TCONS_00000336; exon_number 9; oId CUFF.1206.1; tss_id TSS678;
1	Cufflinks	exon	15993566	15993930	.	+	.	gene_id GRMZM2G156486; transcript_id TCONS_00000336; exon_number 10; oId CUFF.1206.1; tss_id TSS678;
1	Cufflinks	exon	15994434	15994613	.	+	.	gene_id GRMZM2G156486; transcript_id TCONS_00000336; exon_number 11; oId CUFF.1206.1; tss_id TSS678;
1	Cufflinks	exon	16202286	16202881	.	+	.	gene_id XLOC_000289; transcript_id TCONS_00000337; exon_number 1; oId CUFF.1199.1; tss_id TSS680;
1	Cufflinks	exon	21249523	21250167	.	+	.	gene_id GRMZM2G464515; transcript_id TCONS_00000419; exon_number 1; oId CUFF.1505.1; tss_id TSS846;

1	Cufflinks	exon	21250273	21251201	.	+	.	gene_id GRMZM2G464515; transcript_id TCONS_00000419; exon_number 2; oId CUFF.1505.1; tss_id TSS846;
1	Cufflinks	exon	30748051	30748514	.	+	.	gene_id GRMZM2G012758; transcript_id TCONS_00000564; exon_number 1; oId CUFF.2076.1; tss_id TSS1126;
1	Cufflinks	exon	30756454	30756833	.	+	.	gene_id GRMZM2G012758; transcript_id TCONS_00000564; exon_number 2; oId CUFF.2076.1; tss_id TSS1126;
1	Cufflinks	exon	30756978	30757607	.	+	.	gene_id GRMZM2G012758; transcript_id TCONS_00000564; exon_number 3; oId CUFF.2076.1; tss_id TSS1126;
1	Cufflinks	exon	30757698	30758493	.	+	.	gene_id GRMZM2G012758; transcript_id TCONS_00000564; exon_number 4; oId CUFF.2076.1; tss_id TSS1126;
1	Cufflinks	exon	32095243	32097436	.	+	.	gene_id GRMZM2G152663; transcript_id TCONS_00000586; exon_number 1; oId CUFF.2098.1; tss_id TSS1171;
1	Cufflinks	exon	32098120	32098991	.	+	.	gene_id GRMZM2G152663; transcript_id TCONS_00000586; exon_number 2; oId CUFF.2098.1; tss_id TSS1171;
1	Cufflinks	exon	34974445	34975174	.	+	.	gene_id XLOC_000539; transcript_id TCONS_00000629; exon_number 1; oId CUFF.2235.1; tss_id TSS1246;
1	Cufflinks	exon	41766299	41766912	.	+	.	gene_id XLOC_000625; transcript_id TCONS_00000724; exon_number 1; oId CUFF.2601.1; tss_id TSS1449;
1	Cufflinks	exon	46845162	46845951	.	+	.	gene_id GRMZM2G313934; transcript_id TCONS_00000807; exon_number 1; oId CUFF.2889.1; tss_id TSS1612;
1	Cufflinks	exon	48358219	48359262	.	+	.	gene_id XLOC_000719; transcript_id TCONS_00000831; exon_number 1; oId CUFF.2953.1; tss_id TSS1667;
1	Cufflinks	exon	52562240	52564454	.	+	.	gene_id XLOC_000769; transcript_id TCONS_00000889; exon_number 1; oId CUFF.3191.1; tss_id TSS1814;
1	Cufflinks	exon	61615166	61615576	.	+	.	gene_id AC204530.4_FG002; transcript_id TCONS_00000991; exon_number 1; oId CUFF.3681.1; tss_id TSS2080;
1	Cufflinks	exon	61616027	61616141	.	+	.	gene_id AC204530.4_FG002; transcript_id TCONS_00000991; exon_number 2; oId CUFF.3681.1; tss_id TSS2080;
1	Cufflinks	exon	61616244	61616447	.	+	.	gene_id AC204530.4_FG002; transcript_id TCONS_00000991; exon_number 3; oId CUFF.3681.1; tss_id TSS2080;
1	Cufflinks	exon	61616714	61616842	.	+	.	gene_id AC204530.4_FG002; transcript_id TCONS_00000991; exon_number 4; oId CUFF.3681.1; tss_id TSS2080;
1	Cufflinks	exon	61616919	61617083	.	+	.	gene_id AC204530.4_FG002; transcript_id TCONS_00000991; exon_number 5; oId CUFF.3681.1; tss_id TSS2080;
1	Cufflinks	exon	61617758	61617952	.	+	.	gene_id AC204530.4_FG002; transcript_id TCONS_00000991; exon_number 6; oId CUFF.3681.1; tss_id TSS2080;
1	Cufflinks	exon	61619151	61619251	.	+	.	gene_id AC204530.4_FG002; transcript_id TCONS_00000991; exon_number 7; oId CUFF.3681.1; tss_id TSS2080;
1	Cufflinks	exon	61619874	61619963	.	+	.	gene_id AC204530.4_FG002; transcript_id TCONS_00000991; exon_number 8; oId CUFF.3681.1; tss_id TSS2080;
1	Cufflinks	exon	61621080	61621152	.	+	.	gene_id AC204530.4_FG002; transcript_id TCONS_00000991; exon_number 9; oId CUFF.3681.1; tss_id TSS2080;
1	Cufflinks	exon	61621233	61621261	.	+	.	gene_id AC204530.4_FG002; transcript_id TCONS_00000991; exon_number 10; oId CUFF.3681.1; tss_id TSS2080;
1	Cufflinks	exon	61621361	61621484	.	+	.	gene_id AC204530.4_FG002; transcript_id TCONS_00000991; exon_number 11; oId CUFF.3681.1; tss_id TSS2080;
1	Cufflinks	exon	61621716	61621826	.	+	.	gene_id AC204530.4_FG002; transcript_id TCONS_00000991; exon_number 12; oId CUFF.3681.1; tss_id TSS2080;
1	Cufflinks	exon	61622835	61622935	.	+	.	gene_id AC204530.4_FG002; transcript_id TCONS_00000991; exon_number 13; oId CUFF.3681.1; tss_id TSS2080;
1	Cufflinks	exon	61623014	61623614	.	+	.	gene_id AC204530.4_FG002; transcript_id TCONS_00000991; exon_number 14; oId CUFF.3681.1; tss_id TSS2080;
1	Cufflinks	exon	62561406	62562377	.	+	.	gene_id GRMZM5G822100; transcript_id TCONS_00001010; exon_number 1; oId CUFF.3726.1; tss_id TSS2108;
1	Cufflinks	exon	62563133	62563927	.	+	.	gene_id GRMZM5G822100; transcript_id TCONS_00001010; exon_number 2; oId CUFF.3726.1; tss_id TSS2108;
1	Cufflinks	exon	67777631	67778053	.	+	.	gene_id GRMZM2G179792; transcript_id TCONS_00001096; exon_number 1; oId CUFF.3974.1; tss_id TSS2239;
1	Cufflinks	exon	67778134	67778156	.	+	.	gene_id GRMZM2G179792; transcript_id TCONS_00001096; exon_number 2; oId CUFF.3974.1; tss_id TSS2239;
1	Cufflinks	exon	67778178	67778411	.	+	.	gene_id GRMZM2G179792; transcript_id TCONS_00001096; exon_number 3; oId CUFF.3974.1; tss_id TSS2239;
1	Cufflinks	exon	67778887	67778946	.	+	.	gene_id GRMZM2G179792; transcript_id TCONS_00001096; exon_number 4; oId CUFF.3974.1; tss_id TSS2239;
1	Cufflinks	exon	67779053	67780949	.	+	.	gene_id GRMZM2G179792; transcript_id TCONS_00001096; exon_number 5; oId CUFF.3974.1; tss_id TSS2239;
1	Cufflinks	exon	67781024	67781637	.	+	.	gene_id GRMZM2G179792; transcript_id TCONS_00001096; exon_number 6; oId CUFF.3974.1; tss_id TSS2239;
1	Cufflinks	exon	67777631	67778053	.	+	.	gene_id GRMZM2G179792; transcript_id TCONS_00001098; exon_number 1; oId CUFF.3974.2; tss_id TSS2239;
1	Cufflinks	exon	67778138	67778156	.	+	.	gene_id GRMZM2G179792; transcript_id TCONS_00001098; exon_number 2; oId CUFF.3974.2; tss_id TSS2239;

1	Cufflinks	exon	67778178	67778411	.	+	.	gene_id GRMZM2G179792; transcript_id TCONS_00001098; exon_number 3; oId CUFF.3974.2; tss_id TSS2239;
1	Cufflinks	exon	67778887	67778946	.	+	.	gene_id GRMZM2G179792; transcript_id TCONS_00001098; exon_number 4; oId CUFF.3974.2; tss_id TSS2239;
1	Cufflinks	exon	67779053	67780949	.	+	.	gene_id GRMZM2G179792; transcript_id TCONS_00001098; exon_number 5; oId CUFF.3974.2; tss_id TSS2239;
1	Cufflinks	exon	67781024	67781637	.	+	.	gene_id GRMZM2G179792; transcript_id TCONS_00001098; exon_number 6; oId CUFF.3974.2; tss_id TSS2239;
1	Cufflinks	exon	68121352	68121966	.	+	.	gene_id XLOC_000949; transcript_id TCONS_00001106; exon_number 1; oId CUFF.3961.1; tss_id TSS2253;
1	Cufflinks	exon	68568365	68569907	.	+	.	gene_id XLOC_000952; transcript_id TCONS_00001109; exon_number 1; oId CUFF.3988.1; tss_id TSS2262;
1	Cufflinks	exon	68884287	68884520	.	+	.	gene_id GRMZM2G022061; transcript_id TCONS_00001114; exon_number 1; oId CUFF.4040.2; tss_id TSS2270;
1	Cufflinks	exon	68884600	68884752	.	+	.	gene_id GRMZM2G022061; transcript_id TCONS_00001114; exon_number 2; oId CUFF.4040.2; tss_id TSS2270;
1	Cufflinks	exon	68884940	68885015	.	+	.	gene_id GRMZM2G022061; transcript_id TCONS_00001114; exon_number 3; oId CUFF.4040.2; tss_id TSS2270;
1	Cufflinks	exon	68887040	68887334	.	+	.	gene_id GRMZM2G022061; transcript_id TCONS_00001114; exon_number 4; oId CUFF.4040.2; tss_id TSS2270;
1	Cufflinks	exon	68887418	68887524	.	+	.	gene_id GRMZM2G022061; transcript_id TCONS_00001114; exon_number 5; oId CUFF.4040.2; tss_id TSS2270;
1	Cufflinks	exon	68887602	68887649	.	+	.	gene_id GRMZM2G022061; transcript_id TCONS_00001114; exon_number 6; oId CUFF.4040.2; tss_id TSS2270;
1	Cufflinks	exon	68887729	68887935	.	+	.	gene_id GRMZM2G022061; transcript_id TCONS_00001114; exon_number 7; oId CUFF.4040.2; tss_id TSS2270;
1	Cufflinks	exon	68888250	68888351	.	+	.	gene_id GRMZM2G022061; transcript_id TCONS_00001114; exon_number 8; oId CUFF.4040.2; tss_id TSS2270;
1	Cufflinks	exon	68888528	68888658	.	+	.	gene_id GRMZM2G022061; transcript_id TCONS_00001114; exon_number 9; oId CUFF.4040.2; tss_id TSS2270;
1	Cufflinks	exon	68888757	68888886	.	+	.	gene_id GRMZM2G022061; transcript_id TCONS_00001114; exon_number 10; oId CUFF.4040.2; tss_id TSS2270;
1	Cufflinks	exon	68889462	68889560	.	+	.	gene_id GRMZM2G022061; transcript_id TCONS_00001114; exon_number 11; oId CUFF.4040.2; tss_id TSS2270;
1	Cufflinks	exon	68889651	68889980	.	+	.	gene_id GRMZM2G022061; transcript_id TCONS_00001114; exon_number 12; oId CUFF.4040.2; tss_id TSS2270;
1	Cufflinks	exon	74719104	74719886	.	+	.	gene_id XLOC_001011; transcript_id TCONS_00001179; exon_number 1; oId CUFF.4225.1; tss_id TSS2399;
1	Cufflinks	exon	76878489	76878700	.	+	.	gene_id GRMZM2G084587; transcript_id TCONS_00001201; exon_number 1; oId CUFF.4397.1; tss_id TSS2444;
1	Cufflinks	exon	76878874	76878945	.	+	.	gene_id GRMZM2G084587; transcript_id TCONS_00001201; exon_number 2; oId CUFF.4397.1; tss_id TSS2444;
1	Cufflinks	exon	76879029	76879172	.	+	.	gene_id GRMZM2G084587; transcript_id TCONS_00001201; exon_number 3; oId CUFF.4397.1; tss_id TSS2444;
1	Cufflinks	exon	76879405	76879476	.	+	.	gene_id GRMZM2G084587; transcript_id TCONS_00001201; exon_number 4; oId CUFF.4397.1; tss_id TSS2444;
1	Cufflinks	exon	76879558	76879629	.	+	.	gene_id GRMZM2G084587; transcript_id TCONS_00001201; exon_number 5; oId CUFF.4397.1; tss_id TSS2444;
1	Cufflinks	exon	76889541	76889924	.	+	.	gene_id GRMZM2G084587; transcript_id TCONS_00001201; exon_number 6; oId CUFF.4397.1; tss_id TSS2444;
1	Cufflinks	exon	80173019	80173673	.	+	.	gene_id GRMZM2G143703; transcript_id TCONS_00001221; exon_number 1; oId CUFF.4516.1; tss_id TSS2502;
1	Cufflinks	exon	80174180	80174659	.	+	.	gene_id GRMZM2G143703; transcript_id TCONS_00001221; exon_number 2; oId CUFF.4516.1; tss_id TSS2502;
1	Cufflinks	exon	80174752	80176153	.	+	.	gene_id GRMZM2G143703; transcript_id TCONS_00001221; exon_number 3; oId CUFF.4516.1; tss_id TSS2502;
1	Cufflinks	exon	80173019	80174659	.	+	.	gene_id GRMZM2G143703; transcript_id TCONS_00001222; exon_number 1; oId CUFF.4516.2; tss_id TSS2502;
1	Cufflinks	exon	80174752	80176153	.	+	.	gene_id GRMZM2G143703; transcript_id TCONS_00001222; exon_number 2; oId CUFF.4516.2; tss_id TSS2502;
1	Cufflinks	exon	81450983	81451418	.	+	.	gene_id GRMZM2G025190; transcript_id TCONS_00001240; exon_number 1; oId CUFF.4523.1; tss_id TSS2537;
1	Cufflinks	exon	81451538	81452132	.	+	.	gene_id GRMZM2G025190; transcript_id TCONS_00001240; exon_number 2; oId CUFF.4523.1; tss_id TSS2537;
1	Cufflinks	exon	81559325	81559768	.	+	.	gene_id GRMZM2G302373; transcript_id TCONS_00001242; exon_number 1; oId CUFF.4540.1; tss_id TSS2540;
1	Cufflinks	exon	81559889	81560291	.	+	.	gene_id GRMZM2G302373; transcript_id TCONS_00001242; exon_number 2; oId CUFF.4540.1; tss_id TSS2540;
1	Cufflinks	exon	81612836	81613690	.	+	.	gene_id XLOC_001068; transcript_id TCONS_00001245; exon_number 1; oId CUFF.4544.1; tss_id TSS2543;
1	Cufflinks	exon	88490036	88491131	.	+	.	gene_id XLOC_001134; transcript_id TCONS_00001323; exon_number 1; oId CUFF.4780.1; tss_id TSS2680;

1	Cufflinks	exon	92248217	92248435	.	+	.	gene_id GRMZM2G114772; transcript_id TCONS_00001346; exon_number 1; oId CUFF.4883.1; tss_id TSS2725;
1	Cufflinks	exon	92249243	92249504	.	+	.	gene_id GRMZM2G114772; transcript_id TCONS_00001346; exon_number 2; oId CUFF.4883.1; tss_id TSS2725;
1	Cufflinks	exon	92249834	92250035	.	+	.	gene_id GRMZM2G114772; transcript_id TCONS_00001346; exon_number 3; oId CUFF.4883.1; tss_id TSS2725;
1	Cufflinks	exon	92250131	92250278	.	+	.	gene_id GRMZM2G114772; transcript_id TCONS_00001346; exon_number 4; oId CUFF.4883.1; tss_id TSS2725;
1	Cufflinks	exon	92250382	92252805	.	+	.	gene_id GRMZM2G114772; transcript_id TCONS_00001346; exon_number 5; oId CUFF.4883.1; tss_id TSS2725;
1	Cufflinks	exon	99547343	99547922	.	+	.	gene_id XLOC_001211; transcript_id TCONS_00001406; exon_number 1; oId CUFF.5022.1; tss_id TSS2832;
1	Cufflinks	exon	106902765	106903710	.	+	.	gene_id GRMZM2G012306; transcript_id TCONS_00001442; exon_number 1; oId CUFF.5189.1; tss_id TSS2901;
1	Cufflinks	exon	106903794	106904011	.	+	.	gene_id GRMZM2G012306; transcript_id TCONS_00001442; exon_number 2; oId CUFF.5189.1; tss_id TSS2901;
1	Cufflinks	exon	106904139	106905799	.	+	.	gene_id GRMZM2G012306; transcript_id TCONS_00001442; exon_number 3; oId CUFF.5189.1; tss_id TSS2901;
1	Cufflinks	exon	137254903	137255792	.	+	.	gene_id XLOC_001339; transcript_id TCONS_00001547; exon_number 1; oId CUFF.5769.1; tss_id TSS3197;
1	Cufflinks	exon	147208824	147209541	.	+	.	gene_id XLOC_001374; transcript_id TCONS_00001586; exon_number 1; oId CUFF.6046.1; tss_id TSS3315;
1	Cufflinks	exon	151744602	151744708	.	+	.	gene_id GRMZM2G138419; transcript_id TCONS_00001615; exon_number 1; oId CUFF.6219.1; tss_id TSS3387;
1	Cufflinks	exon	151745992	151748418	.	+	.	gene_id GRMZM2G138419; transcript_id TCONS_00001615; exon_number 2; oId CUFF.6219.1; tss_id TSS3387;
1	Cufflinks	exon	151748522	151751395	.	+	.	gene_id GRMZM2G138419; transcript_id TCONS_00001615; exon_number 3; oId CUFF.6219.1; tss_id TSS3387;
1	Cufflinks	exon	154092672	154093005	.	+	.	gene_id GRMZM2G056039; transcript_id TCONS_00001629; exon_number 1; oId CUFF.6232.1; tss_id TSS3401;
1	Cufflinks	exon	154094632	154096549	.	+	.	gene_id GRMZM2G056039; transcript_id TCONS_00001629; exon_number 2; oId CUFF.6232.1; tss_id TSS3401;
1	Cufflinks	exon	161322448	161323383	.	+	.	gene_id XLOC_001442; transcript_id TCONS_00001664; exon_number 1; oId CUFF.6400.1; tss_id TSS3475;
1	Cufflinks	exon	165420861	165422748	.	+	.	gene_id GRMZM2G428216; transcript_id TCONS_00001700; exon_number 1; oId CUFF.6503.1; tss_id TSS3541;
1	Cufflinks	exon	176865344	176865454	.	+	.	gene_id GRMZM2G149751; transcript_id TCONS_00001811; exon_number 1; oId CUFF.6955.1; tss_id TSS3752;
1	Cufflinks	exon	176866830	176867183	.	+	.	gene_id GRMZM2G149751; transcript_id TCONS_00001811; exon_number 2; oId CUFF.6955.1; tss_id TSS3752;
1	Cufflinks	exon	176867274	176867361	.	+	.	gene_id GRMZM2G149751; transcript_id TCONS_00001811; exon_number 3; oId CUFF.6955.1; tss_id TSS3752;
1	Cufflinks	exon	176868034	176868161	.	+	.	gene_id GRMZM2G149751; transcript_id TCONS_00001811; exon_number 4; oId CUFF.6955.1; tss_id TSS3752;
1	Cufflinks	exon	176868238	176868429	.	+	.	gene_id GRMZM2G149751; transcript_id TCONS_00001811; exon_number 5; oId CUFF.6955.1; tss_id TSS3752;
1	Cufflinks	exon	176868527	176868729	.	+	.	gene_id GRMZM2G149751; transcript_id TCONS_00001811; exon_number 6; oId CUFF.6955.1; tss_id TSS3752;
1	Cufflinks	exon	176868815	176868978	.	+	.	gene_id GRMZM2G149751; transcript_id TCONS_00001811; exon_number 7; oId CUFF.6955.1; tss_id TSS3752;
1	Cufflinks	exon	176869095	176869175	.	+	.	gene_id GRMZM2G149751; transcript_id TCONS_00001811; exon_number 8; oId CUFF.6955.1; tss_id TSS3752;
1	Cufflinks	exon	176869261	176869427	.	+	.	gene_id GRMZM2G149751; transcript_id TCONS_00001811; exon_number 9; oId CUFF.6955.1; tss_id TSS3752;
1	Cufflinks	exon	176869503	176869608	.	+	.	gene_id GRMZM2G149751; transcript_id TCONS_00001811; exon_number 10; oId CUFF.6955.1; tss_id TSS3752;
1	Cufflinks	exon	176869717	176869967	.	+	.	gene_id GRMZM2G149751; transcript_id TCONS_00001811; exon_number 11; oId CUFF.6955.1; tss_id TSS3752;
1	Cufflinks	exon	176870065	176870266	.	+	.	gene_id GRMZM2G149751; transcript_id TCONS_00001811; exon_number 12; oId CUFF.6955.1; tss_id TSS3752;
1	Cufflinks	exon	176870368	176871673	.	+	.	gene_id GRMZM2G149751; transcript_id TCONS_00001811; exon_number 13; oId CUFF.6955.1; tss_id TSS3752;
1	Cufflinks	exon	180635595	180637502	.	+	.	gene_id XLOC_001589; transcript_id TCONS_00001843; exon_number 1; oId CUFF.7047.1; tss_id TSS3808;
1	Cufflinks	exon	180651917	180653172	.	+	.	gene_id XLOC_001590; transcript_id TCONS_00001844; exon_number 1; oId CUFF.7055.1; tss_id TSS3809;
1	Cufflinks	exon	195218148	195218716	.	+	.	gene_id GRMZM2G054252; transcript_id TCONS_00002050; exon_number 1; oId CUFF.7730.2; tss_id TSS4186;
1	Cufflinks	exon	195218865	195219163	.	+	.	gene_id GRMZM2G054252; transcript_id TCONS_00002050; exon_number 2; oId CUFF.7730.2; tss_id TSS4186;
1	Cufflinks	exon	195220750	195221731	.	+	.	gene_id GRMZM2G054252; transcript_id TCONS_00002050; exon_number 3; oId CUFF.7730.2; tss_id TSS4186;

1	Cufflinks	exon	195218148	195218716	.	+	.	gene_id GRMZM2G054252; transcript_id TCONS_00002051; exon_number 1; oId CUFF.7730.3; tss_id TSS4186;
1	Cufflinks	exon	195218862	195219163	.	+	.	gene_id GRMZM2G054252; transcript_id TCONS_00002051; exon_number 2; oId CUFF.7730.3; tss_id TSS4186;
1	Cufflinks	exon	195220750	195221731	.	+	.	gene_id GRMZM2G054252; transcript_id TCONS_00002051; exon_number 3; oId CUFF.7730.3; tss_id TSS4186;
1	Cufflinks	exon	199788867	199789152	.	+	.	gene_id GRMZM5G881887; transcript_id TCONS_00002120; exon_number 1; oId CUFF.7984.2; tss_id TSS4326;
1	Cufflinks	exon	199789299	199789471	.	+	.	gene_id GRMZM5G881887; transcript_id TCONS_00002120; exon_number 2; oId CUFF.7984.2; tss_id TSS4326;
1	Cufflinks	exon	199789758	199789985	.	+	.	gene_id GRMZM5G881887; transcript_id TCONS_00002120; exon_number 3; oId CUFF.7984.2; tss_id TSS4326;
1	Cufflinks	exon	199790079	199790461	.	+	.	gene_id GRMZM5G881887; transcript_id TCONS_00002120; exon_number 4; oId CUFF.7984.2; tss_id TSS4326;
1	Cufflinks	exon	199790562	199791107	.	+	.	gene_id GRMZM5G881887; transcript_id TCONS_00002120; exon_number 5; oId CUFF.7984.2; tss_id TSS4326;
1	Cufflinks	exon	199788867	199789152	.	+	.	gene_id GRMZM5G881887; transcript_id TCONS_00002121; exon_number 1; oId CUFF.7984.1; tss_id TSS4326;
1	Cufflinks	exon	199789299	199789471	.	+	.	gene_id GRMZM5G881887; transcript_id TCONS_00002121; exon_number 2; oId CUFF.7984.1; tss_id TSS4326;
1	Cufflinks	exon	199789755	199789985	.	+	.	gene_id GRMZM5G881887; transcript_id TCONS_00002121; exon_number 3; oId CUFF.7984.1; tss_id TSS4326;
1	Cufflinks	exon	199790079	199790461	.	+	.	gene_id GRMZM5G881887; transcript_id TCONS_00002121; exon_number 4; oId CUFF.7984.1; tss_id TSS4326;
1	Cufflinks	exon	199790562	199791107	.	+	.	gene_id GRMZM5G881887; transcript_id TCONS_00002121; exon_number 5; oId CUFF.7984.1; tss_id TSS4326;
1	Cufflinks	exon	199788867	199789152	.	+	.	gene_id GRMZM5G881887; transcript_id TCONS_00002122; exon_number 1; oId CUFF.7984.3; tss_id TSS4326;
1	Cufflinks	exon	199789299	199789985	.	+	.	gene_id GRMZM5G881887; transcript_id TCONS_00002122; exon_number 2; oId CUFF.7984.3; tss_id TSS4326;
1	Cufflinks	exon	199790079	199790461	.	+	.	gene_id GRMZM5G881887; transcript_id TCONS_00002122; exon_number 3; oId CUFF.7984.3; tss_id TSS4326;
1	Cufflinks	exon	199790562	199791107	.	+	.	gene_id GRMZM5G881887; transcript_id TCONS_00002122; exon_number 4; oId CUFF.7984.3; tss_id TSS4326;
1	Cufflinks	exon	202334807	202335597	.	+	.	gene_id GRMZM2G315375; transcript_id TCONS_00002162; exon_number 1; oId CUFF.8069.1; tss_id TSS4389;
1	Cufflinks	exon	202335741	202336377	.	+	.	gene_id GRMZM2G315375; transcript_id TCONS_00002162; exon_number 2; oId CUFF.8069.1; tss_id TSS4389;
1	Cufflinks	exon	202336473	202336783	.	+	.	gene_id GRMZM2G315375; transcript_id TCONS_00002162; exon_number 3; oId CUFF.8069.1; tss_id TSS4389;
1	Cufflinks	exon	202337397	202337626	.	+	.	gene_id GRMZM2G315375; transcript_id TCONS_00002162; exon_number 4; oId CUFF.8069.1; tss_id TSS4389;
1	Cufflinks	exon	202339289	202342249	.	+	.	gene_id GRMZM2G315375; transcript_id TCONS_00002162; exon_number 5; oId CUFF.8069.1; tss_id TSS4389;
1	Cufflinks	exon	202955781	202956675	.	+	.	gene_id GRMZM2G179703; transcript_id TCONS_00002175; exon_number 1; oId CUFF.8121.1; tss_id TSS4409;
1	Cufflinks	exon	202957125	202958669	.	+	.	gene_id GRMZM2G179703; transcript_id TCONS_00002175; exon_number 2; oId CUFF.8121.1; tss_id TSS4409;
1	Cufflinks	exon	208340735	208341691	.	+	.	gene_id GRMZM2G401147; transcript_id TCONS_00002257; exon_number 1; oId CUFF.8355.1; tss_id TSS4540;
1	Cufflinks	exon	211567120	211567647	.	+	.	gene_id GRMZM2G131205; transcript_id TCONS_00002294; exon_number 1; oId CUFF.8552.1; tss_id TSS4618;
1	Cufflinks	exon	211567759	211567913	.	+	.	gene_id GRMZM2G131205; transcript_id TCONS_00002294; exon_number 2; oId CUFF.8552.1; tss_id TSS4618;
1	Cufflinks	exon	211568015	211568203	.	+	.	gene_id GRMZM2G131205; transcript_id TCONS_00002294; exon_number 3; oId CUFF.8552.1; tss_id TSS4618;
1	Cufflinks	exon	211570194	211570546	.	+	.	gene_id GRMZM2G131205; transcript_id TCONS_00002294; exon_number 4; oId CUFF.8552.1; tss_id TSS4618;
1	Cufflinks	exon	211572458	211572922	.	+	.	gene_id GRMZM2G131205; transcript_id TCONS_00002294; exon_number 5; oId CUFF.8552.1; tss_id TSS4618;
1	Cufflinks	exon	212088923	212089444	.	+	.	gene_id GRMZM2G556078; transcript_id TCONS_00002299; exon_number 1; oId CUFF.8541.1; tss_id TSS4632;
1	Cufflinks	exon	216747936	216748356	.	+	.	gene_id GRMZM2G167669; transcript_id TCONS_00002343; exon_number 1; oId CUFF.8768.1; tss_id TSS4741;
1	Cufflinks	exon	220147969	220148422	.	+	.	gene_id GRMZM2G136439; transcript_id TCONS_00002386; exon_number 1; oId CUFF.8915.1; tss_id TSS4834;
1	Cufflinks	exon	220150843	220150934	.	+	.	gene_id GRMZM2G136439; transcript_id TCONS_00002386; exon_number 2; oId CUFF.8915.1; tss_id TSS4834;
1	Cufflinks	exon	223849490	223850364	.	+	.	gene_id XLOC_002108; transcript_id TCONS_00002444; exon_number 1; oId CUFF.9136.1; tss_id TSS4958;
1	Cufflinks	exon	233293225	233293591	.	+	.	gene_id XLOC_002206; transcript_id TCONS_00002562; exon_number 1; oId CUFF.9683.1; tss_id TSS5200;

1	Cufflinks	exon	234322570	234322750	.	+	.	gene_id GRMZM2G445905; transcript_id TCONS_00002573; exon_number 1; oId CUFF.9769.1; tss_id TSS5233;
1	Cufflinks	exon	234322825	234323023	.	+	.	gene_id GRMZM2G445905; transcript_id TCONS_00002573; exon_number 2; oId CUFF.9769.1; tss_id TSS5233;
1	Cufflinks	exon	234323112	234323300	.	+	.	gene_id GRMZM2G445905; transcript_id TCONS_00002573; exon_number 3; oId CUFF.9769.1; tss_id TSS5233;
1	Cufflinks	exon	234323382	234323538	.	+	.	gene_id GRMZM2G445905; transcript_id TCONS_00002573; exon_number 4; oId CUFF.9769.1; tss_id TSS5233;
1	Cufflinks	exon	234323633	234324722	.	+	.	gene_id GRMZM2G445905; transcript_id TCONS_00002573; exon_number 5; oId CUFF.9769.1; tss_id TSS5233;
1	Cufflinks	exon	234324836	234325414	.	+	.	gene_id GRMZM2G445905; transcript_id TCONS_00002573; exon_number 6; oId CUFF.9769.1; tss_id TSS5233;
1	Cufflinks	exon	234325511	234325861	.	+	.	gene_id GRMZM2G445905; transcript_id TCONS_00002573; exon_number 7; oId CUFF.9769.1; tss_id TSS5233;
1	Cufflinks	exon	234325951	234326740	.	+	.	gene_id GRMZM2G445905; transcript_id TCONS_00002573; exon_number 8; oId CUFF.9769.1; tss_id TSS5233;
1	Cufflinks	exon	234715321	234717256	.	+	.	gene_id GRMZM2G450762; transcript_id TCONS_00002585; exon_number 1; oId CUFF.9899.1; tss_id TSS5283;
1	Cufflinks	exon	235420397	235420657	.	+	.	gene_id GRMZM2G020295; transcript_id TCONS_00002590; exon_number 1; oId CUFF.9943.1; tss_id TSS5308;
1	Cufflinks	exon	235423426	235423490	.	+	.	gene_id GRMZM2G020295; transcript_id TCONS_00002590; exon_number 2; oId CUFF.9943.1; tss_id TSS5308;
1	Cufflinks	exon	235423596	235423659	.	+	.	gene_id GRMZM2G020295; transcript_id TCONS_00002590; exon_number 3; oId CUFF.9943.1; tss_id TSS5308;
1	Cufflinks	exon	235423742	235423751	.	+	.	gene_id GRMZM2G020295; transcript_id TCONS_00002590; exon_number 4; oId CUFF.9943.1; tss_id TSS5308;
1	Cufflinks	exon	235423929	235423960	.	+	.	gene_id GRMZM2G020295; transcript_id TCONS_00002590; exon_number 5; oId CUFF.9943.1; tss_id TSS5308;
1	Cufflinks	exon	235424047	235424159	.	+	.	gene_id GRMZM2G020295; transcript_id TCONS_00002590; exon_number 6; oId CUFF.9943.1; tss_id TSS5308;
1	Cufflinks	exon	235424540	235424616	.	+	.	gene_id GRMZM2G020295; transcript_id TCONS_00002590; exon_number 7; oId CUFF.9943.1; tss_id TSS5308;
1	Cufflinks	exon	235424696	235425098	.	+	.	gene_id GRMZM2G020295; transcript_id TCONS_00002590; exon_number 8; oId CUFF.9943.1; tss_id TSS5308;
1	Cufflinks	exon	235769118	235771246	.	+	.	gene_id XLOC_002237; transcript_id TCONS_00002596; exon_number 1; oId CUFF.9980.1; tss_id TSS5320;
1	Cufflinks	exon	237544777	237544932	.	+	.	gene_id GRMZM2G003724; transcript_id TCONS_00002615; exon_number 1; oId CUFF.10043.1; tss_id TSS5372;
1	Cufflinks	exon	237545681	237545779	.	+	.	gene_id GRMZM2G003724; transcript_id TCONS_00002615; exon_number 2; oId CUFF.10043.1; tss_id TSS5372;
1	Cufflinks	exon	237545926	237546713	.	+	.	gene_id GRMZM2G003724; transcript_id TCONS_00002615; exon_number 3; oId CUFF.10043.1; tss_id TSS5372;
1	Cufflinks	exon	246768933	246769376	.	+	.	gene_id GRMZM2G146486; transcript_id TCONS_00002720; exon_number 1; oId CUFF.10556.1; tss_id TSS5655;
1	Cufflinks	exon	246769446	246769538	.	+	.	gene_id GRMZM2G146486; transcript_id TCONS_00002720; exon_number 2; oId CUFF.10556.1; tss_id TSS5655;
1	Cufflinks	exon	246769679	246769747	.	+	.	gene_id GRMZM2G146486; transcript_id TCONS_00002720; exon_number 3; oId CUFF.10556.1; tss_id TSS5655;
1	Cufflinks	exon	246770040	246770129	.	+	.	gene_id GRMZM2G146486; transcript_id TCONS_00002720; exon_number 4; oId CUFF.10556.1; tss_id TSS5655;
1	Cufflinks	exon	246770218	246770301	.	+	.	gene_id GRMZM2G146486; transcript_id TCONS_00002720; exon_number 5; oId CUFF.10556.1; tss_id TSS5655;
1	Cufflinks	exon	246770544	246770708	.	+	.	gene_id GRMZM2G146486; transcript_id TCONS_00002720; exon_number 6; oId CUFF.10556.1; tss_id TSS5655;
1	Cufflinks	exon	246772758	246772945	.	+	.	gene_id GRMZM2G146486; transcript_id TCONS_00002720; exon_number 7; oId CUFF.10556.1; tss_id TSS5655;
1	Cufflinks	exon	246773147	246773219	.	+	.	gene_id GRMZM2G146486; transcript_id TCONS_00002720; exon_number 8; oId CUFF.10556.1; tss_id TSS5655;
1	Cufflinks	exon	246774105	246774158	.	+	.	gene_id GRMZM2G146486; transcript_id TCONS_00002720; exon_number 9; oId CUFF.10556.1; tss_id TSS5655;
1	Cufflinks	exon	246774562	246774819	.	+	.	gene_id GRMZM2G146486; transcript_id TCONS_00002720; exon_number 10; oId CUFF.10556.1; tss_id TSS5655;
1	Cufflinks	exon	246780483	246780689	.	+	.	gene_id GRMZM2G146486; transcript_id TCONS_00002720; exon_number 11; oId CUFF.10556.1; tss_id TSS5655;
1	Cufflinks	exon	246780790	246780893	.	+	.	gene_id GRMZM2G146486; transcript_id TCONS_00002720; exon_number 12; oId CUFF.10556.1; tss_id TSS5655;
1	Cufflinks	exon	246781185	246781329	.	+	.	gene_id GRMZM2G146486; transcript_id TCONS_00002720; exon_number 13; oId CUFF.10556.1; tss_id TSS5655;
1	Cufflinks	exon	246781648	246781782	.	+	.	gene_id GRMZM2G146486; transcript_id TCONS_00002720; exon_number 14; oId CUFF.10556.1; tss_id TSS5655;
1	Cufflinks	exon	246782207	246782639	.	+	.	gene_id GRMZM2G146486; transcript_id TCONS_00002720; exon_number 15; oId CUFF.10556.1; tss_id TSS5655;

1	Cufflinks	exon	264577362	264577882	.	+	.	gene_id GRMZM2G033787; transcript_id TCONS_00002958; exon_number 1; oId CUFF.11498.1; tss_id TSS6224;
1	Cufflinks	exon	264577981	264578144	.	+	.	gene_id GRMZM2G033787; transcript_id TCONS_00002958; exon_number 2; oId CUFF.11498.1; tss_id TSS6224;
1	Cufflinks	exon	264578265	264578302	.	+	.	gene_id GRMZM2G033787; transcript_id TCONS_00002958; exon_number 3; oId CUFF.11498.1; tss_id TSS6224;
1	Cufflinks	exon	264578412	264578499	.	+	.	gene_id GRMZM2G033787; transcript_id TCONS_00002958; exon_number 4; oId CUFF.11498.1; tss_id TSS6224;
1	Cufflinks	exon	264578613	264578828	.	+	.	gene_id GRMZM2G033787; transcript_id TCONS_00002958; exon_number 5; oId CUFF.11498.1; tss_id TSS6224;
1	Cufflinks	exon	264579134	264579199	.	+	.	gene_id GRMZM2G033787; transcript_id TCONS_00002958; exon_number 6; oId CUFF.11498.1; tss_id TSS6224;
1	Cufflinks	exon	264579928	264580333	.	+	.	gene_id GRMZM2G033787; transcript_id TCONS_00002958; exon_number 7; oId CUFF.11498.1; tss_id TSS6224;
1	Cufflinks	exon	270898329	270898894	.	+	.	gene_id XLOC_002634; transcript_id TCONS_00003050; exon_number 1; oId CUFF.11853.1; tss_id TSS6414;
1	Cufflinks	exon	276371897	276372033	.	+	.	gene_id GRMZM2G147687; transcript_id TCONS_00003140; exon_number 1; oId CUFF.12215.1; tss_id TSS6572;
1	Cufflinks	exon	276373971	276374240	.	+	.	gene_id GRMZM2G147687; transcript_id TCONS_00003140; exon_number 2; oId CUFF.12215.1; tss_id TSS6572;
1	Cufflinks	exon	276374342	276374546	.	+	.	gene_id GRMZM2G147687; transcript_id TCONS_00003140; exon_number 3; oId CUFF.12215.1; tss_id TSS6572;
1	Cufflinks	exon	276374674	276374764	.	+	.	gene_id GRMZM2G147687; transcript_id TCONS_00003140; exon_number 4; oId CUFF.12215.1; tss_id TSS6572;
1	Cufflinks	exon	276374875	276375017	.	+	.	gene_id GRMZM2G147687; transcript_id TCONS_00003140; exon_number 5; oId CUFF.12215.1; tss_id TSS6572;
1	Cufflinks	exon	276375160	276375403	.	+	.	gene_id GRMZM2G147687; transcript_id TCONS_00003140; exon_number 6; oId CUFF.12215.1; tss_id TSS6572;
1	Cufflinks	exon	276375498	276375602	.	+	.	gene_id GRMZM2G147687; transcript_id TCONS_00003140; exon_number 7; oId CUFF.12215.1; tss_id TSS6572;
1	Cufflinks	exon	276375910	276376092	.	+	.	gene_id GRMZM2G147687; transcript_id TCONS_00003140; exon_number 8; oId CUFF.12215.1; tss_id TSS6572;
1	Cufflinks	exon	276376179	276376380	.	+	.	gene_id GRMZM2G147687; transcript_id TCONS_00003140; exon_number 9; oId CUFF.12215.1; tss_id TSS6572;
1	Cufflinks	exon	276376490	276377338	.	+	.	gene_id GRMZM2G147687; transcript_id TCONS_00003140; exon_number 10; oId CUFF.12215.1; tss_id TSS6572;
1	Cufflinks	exon	276371927	276372026	.	+	.	gene_id GRMZM2G147687; transcript_id TCONS_00003141; exon_number 1; oId CUFF.12215.2; tss_id TSS6572;
1	Cufflinks	exon	276373971	276374240	.	+	.	gene_id GRMZM2G147687; transcript_id TCONS_00003141; exon_number 2; oId CUFF.12215.2; tss_id TSS6572;
1	Cufflinks	exon	276374342	276374546	.	+	.	gene_id GRMZM2G147687; transcript_id TCONS_00003141; exon_number 3; oId CUFF.12215.2; tss_id TSS6572;
1	Cufflinks	exon	276374674	276374764	.	+	.	gene_id GRMZM2G147687; transcript_id TCONS_00003141; exon_number 4; oId CUFF.12215.2; tss_id TSS6572;
1	Cufflinks	exon	276374875	276375017	.	+	.	gene_id GRMZM2G147687; transcript_id TCONS_00003141; exon_number 5; oId CUFF.12215.2; tss_id TSS6572;
1	Cufflinks	exon	276375160	276375403	.	+	.	gene_id GRMZM2G147687; transcript_id TCONS_00003141; exon_number 6; oId CUFF.12215.2; tss_id TSS6572;
1	Cufflinks	exon	276375498	276375602	.	+	.	gene_id GRMZM2G147687; transcript_id TCONS_00003141; exon_number 7; oId CUFF.12215.2; tss_id TSS6572;
1	Cufflinks	exon	276375910	276376092	.	+	.	gene_id GRMZM2G147687; transcript_id TCONS_00003141; exon_number 8; oId CUFF.12215.2; tss_id TSS6572;
1	Cufflinks	exon	276376179	276376380	.	+	.	gene_id GRMZM2G147687; transcript_id TCONS_00003141; exon_number 9; oId CUFF.12215.2; tss_id TSS6572;
1	Cufflinks	exon	276376490	276377338	.	+	.	gene_id GRMZM2G147687; transcript_id TCONS_00003141; exon_number 10; oId CUFF.12215.2; tss_id TSS6572;
1	Cufflinks	exon	276389960	276390742	.	+	.	gene_id GRMZM2G017186; transcript_id TCONS_00003144; exon_number 1; oId CUFF.12202.1; tss_id TSS6576;
1	Cufflinks	exon	276392640	276393121	.	+	.	gene_id GRMZM2G017186; transcript_id TCONS_00003144; exon_number 2; oId CUFF.12202.1; tss_id TSS6576;
1	Cufflinks	exon	276393220	276393310	.	+	.	gene_id GRMZM2G017186; transcript_id TCONS_00003144; exon_number 3; oId CUFF.12202.1; tss_id TSS6576;
1	Cufflinks	exon	276393412	276393557	.	+	.	gene_id GRMZM2G017186; transcript_id TCONS_00003144; exon_number 4; oId CUFF.12202.1; tss_id TSS6576;
1	Cufflinks	exon	276393644	276393887	.	+	.	gene_id GRMZM2G017186; transcript_id TCONS_00003144; exon_number 5; oId CUFF.12202.1; tss_id TSS6576;
1	Cufflinks	exon	276393965	276394069	.	+	.	gene_id GRMZM2G017186; transcript_id TCONS_00003144; exon_number 6; oId CUFF.12202.1; tss_id TSS6576;
1	Cufflinks	exon	276394149	276395199	.	+	.	gene_id GRMZM2G017186; transcript_id TCONS_00003144; exon_number 7; oId CUFF.12202.1; tss_id TSS6576;
1	Cufflinks	exon	277055488	277056094	.	+	.	gene_id GRMZM2G058032; transcript_id TCONS_00003158; exon_number 1; oId CUFF.12241.2; tss_id TSS6607;

1	Cufflinks	exon	277056234	277056323	.	+	.	gene_id GRMZM2G058032; transcript_id TCONS_00003158; exon_number 2; oId CUFF.12241.2; tss_id TSS6607;
1	Cufflinks	exon	277056462	277057838	.	+	.	gene_id GRMZM2G058032; transcript_id TCONS_00003158; exon_number 3; oId CUFF.12241.2; tss_id TSS6607;
1	Cufflinks	exon	277055488	277056094	.	+	.	gene_id GRMZM2G058032; transcript_id TCONS_00003159; exon_number 1; oId CUFF.12241.1; tss_id TSS6607;
1	Cufflinks	exon	277056239	277056323	.	+	.	gene_id GRMZM2G058032; transcript_id TCONS_00003159; exon_number 2; oId CUFF.12241.1; tss_id TSS6607;
1	Cufflinks	exon	277056462	277057838	.	+	.	gene_id GRMZM2G058032; transcript_id TCONS_00003159; exon_number 3; oId CUFF.12241.1; tss_id TSS6607;
1	Cufflinks	exon	277063140	277064199	.	+	.	gene_id XLOC_002732; transcript_id TCONS_00003161; exon_number 1; oId CUFF.12250.1; tss_id TSS6608;
1	Cufflinks	exon	277449320	277450131	.	+	.	gene_id XLOC_002740; transcript_id TCONS_00003173; exon_number 1; oId CUFF.12286.4; tss_id TSS6624;
1	Cufflinks	exon	285855288	285857095	.	+	.	gene_id GRMZM2G414540; transcript_id TCONS_00003291; exon_number 1; oId CUFF.12698.1; tss_id TSS6859;
1	Cufflinks	exon	286045297	286046742	.	+	.	gene_id GRMZM2G028926; transcript_id TCONS_00003299; exon_number 1; oId CUFF.12719.1; tss_id TSS6868;
1	Cufflinks	exon	286047605	286047748	.	+	.	gene_id GRMZM2G028926; transcript_id TCONS_00003299; exon_number 2; oId CUFF.12719.1; tss_id TSS6868;
1	Cufflinks	exon	286047876	286048028	.	+	.	gene_id GRMZM2G028926; transcript_id TCONS_00003299; exon_number 3; oId CUFF.12719.1; tss_id TSS6868;
1	Cufflinks	exon	286048128	286048243	.	+	.	gene_id GRMZM2G028926; transcript_id TCONS_00003299; exon_number 4; oId CUFF.12719.1; tss_id TSS6868;
1	Cufflinks	exon	286048442	286048609	.	+	.	gene_id GRMZM2G028926; transcript_id TCONS_00003299; exon_number 5; oId CUFF.12719.1; tss_id TSS6868;
1	Cufflinks	exon	286048698	286048922	.	+	.	gene_id GRMZM2G028926; transcript_id TCONS_00003299; exon_number 6; oId CUFF.12719.1; tss_id TSS6868;
1	Cufflinks	exon	286049055	286050143	.	+	.	gene_id GRMZM2G028926; transcript_id TCONS_00003299; exon_number 7; oId CUFF.12719.1; tss_id TSS6868;
1	Cufflinks	exon	286428657	286429831	.	+	.	gene_id XLOC_002859; transcript_id TCONS_00003304; exon_number 1; oId CUFF.12778.1; tss_id TSS6875;
1	Cufflinks	exon	286730567	286730962	.	+	.	gene_id GRMZM2G060924; transcript_id TCONS_00003310; exon_number 1; oId CUFF.12789.1; tss_id TSS6894;
1	Cufflinks	exon	286731054	286731164	.	+	.	gene_id GRMZM2G060924; transcript_id TCONS_00003310; exon_number 2; oId CUFF.12789.1; tss_id TSS6894;
1	Cufflinks	exon	286731272	286731304	.	+	.	gene_id GRMZM2G060924; transcript_id TCONS_00003310; exon_number 3; oId CUFF.12789.1; tss_id TSS6894;
1	Cufflinks	exon	286734066	286734234	.	+	.	gene_id GRMZM2G060924; transcript_id TCONS_00003310; exon_number 4; oId CUFF.12789.1; tss_id TSS6894;
1	Cufflinks	exon	286734762	286734943	.	+	.	gene_id GRMZM2G060924; transcript_id TCONS_00003310; exon_number 5; oId CUFF.12789.1; tss_id TSS6894;
1	Cufflinks	exon	286735160	286735296	.	+	.	gene_id GRMZM2G060924; transcript_id TCONS_00003310; exon_number 6; oId CUFF.12789.1; tss_id TSS6894;
1	Cufflinks	exon	286735436	286735534	.	+	.	gene_id GRMZM2G060924; transcript_id TCONS_00003310; exon_number 7; oId CUFF.12789.1; tss_id TSS6894;
1	Cufflinks	exon	286736360	286736429	.	+	.	gene_id GRMZM2G060924; transcript_id TCONS_00003310; exon_number 8; oId CUFF.12789.1; tss_id TSS6894;
1	Cufflinks	exon	286736518	286736592	.	+	.	gene_id GRMZM2G060924; transcript_id TCONS_00003310; exon_number 9; oId CUFF.12789.1; tss_id TSS6894;
1	Cufflinks	exon	286736829	286736874	.	+	.	gene_id GRMZM2G060924; transcript_id TCONS_00003310; exon_number 10; oId CUFF.12789.1; tss_id TSS6894;
1	Cufflinks	exon	286737060	286737443	.	+	.	gene_id GRMZM2G060924; transcript_id TCONS_00003310; exon_number 11; oId CUFF.12789.1; tss_id TSS6894;
1	Cufflinks	exon	286916349	286916756	.	+	.	gene_id GRMZM2G120674; transcript_id TCONS_00003313; exon_number 1; oId CUFF.12765.1; tss_id TSS6896;
1	Cufflinks	exon	286916890	286916915	.	+	.	gene_id GRMZM2G120674; transcript_id TCONS_00003313; exon_number 2; oId CUFF.12765.1; tss_id TSS6896;
1	Cufflinks	exon	286918853	286918942	.	+	.	gene_id GRMZM2G120674; transcript_id TCONS_00003313; exon_number 3; oId CUFF.12765.1; tss_id TSS6896;
1	Cufflinks	exon	286919037	286919125	.	+	.	gene_id GRMZM2G120674; transcript_id TCONS_00003313; exon_number 4; oId CUFF.12765.1; tss_id TSS6896;
1	Cufflinks	exon	286920419	286921522	.	+	.	gene_id GRMZM2G120674; transcript_id TCONS_00003313; exon_number 5; oId CUFF.12765.1; tss_id TSS6896;
1	Cufflinks	exon	288992277	288993039	.	+	.	gene_id GRMZM2G453320; transcript_id TCONS_00003357; exon_number 1; oId CUFF.12890.1; tss_id TSS6970;
1	Cufflinks	exon	292162383	292163166	.	+	.	gene_id GRMZM2G347043; transcript_id TCONS_00003415; exon_number 1; oId CUFF.13134.1; tss_id TSS7087;
1	Cufflinks	exon	292163269	292164080	.	+	.	gene_id GRMZM2G347043; transcript_id TCONS_00003415; exon_number 2; oId CUFF.13134.1; tss_id TSS7087;
1	Cufflinks	exon	292412705	292413447	.	+	.	gene_id XLOC_002964; transcript_id TCONS_00003422; exon_number 1; oId CUFF.13158.1; tss_id TSS7102;

1	Cufflinks	exon	293722839	293724323	.	+	.	gene_id GRMZM2G068443; transcript_id TCONS_00003447; exon_number 1; oId CUFF.13291.1; tss_id TSS7154;
1	Cufflinks	exon	293724410	293724496	.	+	.	gene_id GRMZM2G068443; transcript_id TCONS_00003447; exon_number 2; oId CUFF.13291.1; tss_id TSS7154;
1	Cufflinks	exon	293724609	293724671	.	+	.	gene_id GRMZM2G068443; transcript_id TCONS_00003447; exon_number 3; oId CUFF.13291.1; tss_id TSS7154;
1	Cufflinks	exon	293724763	293724864	.	+	.	gene_id GRMZM2G068443; transcript_id TCONS_00003447; exon_number 4; oId CUFF.13291.1; tss_id TSS7154;
1	Cufflinks	exon	293725635	293725893	.	+	.	gene_id GRMZM2G068443; transcript_id TCONS_00003447; exon_number 5; oId CUFF.13291.1; tss_id TSS7154;
1	Cufflinks	exon	293726038	293726477	.	+	.	gene_id GRMZM2G068443; transcript_id TCONS_00003447; exon_number 6; oId CUFF.13291.1; tss_id TSS7154;
1	Cufflinks	exon	295100532	295101411	.	+	.	gene_id GRMZM2G139123; transcript_id TCONS_00003474; exon_number 1; oId CUFF.13357.1; tss_id TSS7198;
1	Cufflinks	exon	296062422	296065979	.	+	.	gene_id GRMZM2G448523; transcript_id TCONS_00003491; exon_number 1; oId CUFF.13432.1; tss_id TSS7226;
1	Cufflinks	exon	296066946	296069083	.	+	.	gene_id GRMZM2G448523; transcript_id TCONS_00003491; exon_number 2; oId CUFF.13432.1; tss_id TSS7226;
1	Cufflinks	exon	296076874	296077091	.	+	.	gene_id GRMZM2G053916; transcript_id TCONS_00003492; exon_number 1; oId CUFF.13557.1; tss_id TSS7229;
1	Cufflinks	exon	296077200	296077382	.	+	.	gene_id GRMZM2G053916; transcript_id TCONS_00003492; exon_number 2; oId CUFF.13557.1; tss_id TSS7229;
1	Cufflinks	exon	296077569	296077780	.	+	.	gene_id GRMZM2G053916; transcript_id TCONS_00003492; exon_number 3; oId CUFF.13557.1; tss_id TSS7229;
1	Cufflinks	exon	296080196	296080356	.	+	.	gene_id GRMZM2G053916; transcript_id TCONS_00003492; exon_number 4; oId CUFF.13557.1; tss_id TSS7229;
1	Cufflinks	exon	296080633	296080782	.	+	.	gene_id GRMZM2G053916; transcript_id TCONS_00003492; exon_number 5; oId CUFF.13557.1; tss_id TSS7229;
1	Cufflinks	exon	296081057	296081109	.	+	.	gene_id GRMZM2G053916; transcript_id TCONS_00003492; exon_number 6; oId CUFF.13557.1; tss_id TSS7229;
1	Cufflinks	exon	296081293	296081433	.	+	.	gene_id GRMZM2G053916; transcript_id TCONS_00003492; exon_number 7; oId CUFF.13557.1; tss_id TSS7229;
1	Cufflinks	exon	296081533	296085483	.	+	.	gene_id GRMZM2G053916; transcript_id TCONS_00003492; exon_number 8; oId CUFF.13557.1; tss_id TSS7229;
1	Cufflinks	exon	296088128	296089220	.	+	.	gene_id GRMZM2G351160; transcript_id TCONS_00003495; exon_number 1; oId CUFF.13558.2; tss_id TSS7232;
1	Cufflinks	exon	296089341	296089438	.	+	.	gene_id GRMZM2G351160; transcript_id TCONS_00003495; exon_number 2; oId CUFF.13558.2; tss_id TSS7232;
1	Cufflinks	exon	296089672	296089810	.	+	.	gene_id GRMZM2G351160; transcript_id TCONS_00003495; exon_number 3; oId CUFF.13558.2; tss_id TSS7232;
1	Cufflinks	exon	296089902	296089930	.	+	.	gene_id GRMZM2G351160; transcript_id TCONS_00003495; exon_number 4; oId CUFF.13558.2; tss_id TSS7232;
1	Cufflinks	exon	296090038	296090134	.	+	.	gene_id GRMZM2G351160; transcript_id TCONS_00003495; exon_number 5; oId CUFF.13558.2; tss_id TSS7232;
1	Cufflinks	exon	296090213	296090268	.	+	.	gene_id GRMZM2G351160; transcript_id TCONS_00003495; exon_number 6; oId CUFF.13558.2; tss_id TSS7232;
1	Cufflinks	exon	296090485	296090592	.	+	.	gene_id GRMZM2G351160; transcript_id TCONS_00003495; exon_number 7; oId CUFF.13558.2; tss_id TSS7232;
1	Cufflinks	exon	296090876	296090932	.	+	.	gene_id GRMZM2G351160; transcript_id TCONS_00003495; exon_number 8; oId CUFF.13558.2; tss_id TSS7232;
1	Cufflinks	exon	296091410	296091501	.	+	.	gene_id GRMZM2G351160; transcript_id TCONS_00003495; exon_number 9; oId CUFF.13558.2; tss_id TSS7232;
1	Cufflinks	exon	296092872	296092956	.	+	.	gene_id GRMZM2G351160; transcript_id TCONS_00003495; exon_number 10; oId CUFF.13558.2; tss_id TSS7232;
1	Cufflinks	exon	296094168	296094320	.	+	.	gene_id GRMZM2G351160; transcript_id TCONS_00003495; exon_number 11; oId CUFF.13558.2; tss_id TSS7232;
1	Cufflinks	exon	296094726	296094820	.	+	.	gene_id GRMZM2G351160; transcript_id TCONS_00003495; exon_number 12; oId CUFF.13558.2; tss_id TSS7232;
1	Cufflinks	exon	296094899	296095075	.	+	.	gene_id GRMZM2G351160; transcript_id TCONS_00003495; exon_number 13; oId CUFF.13558.2; tss_id TSS7232;
1	Cufflinks	exon	296095162	296095265	.	+	.	gene_id GRMZM2G351160; transcript_id TCONS_00003495; exon_number 14; oId CUFF.13558.2; tss_id TSS7232;
1	Cufflinks	exon	296095814	296095883	.	+	.	gene_id GRMZM2G351160; transcript_id TCONS_00003495; exon_number 15; oId CUFF.13558.2; tss_id TSS7232;
1	Cufflinks	exon	296096024	296096116	.	+	.	gene_id GRMZM2G351160; transcript_id TCONS_00003495; exon_number 16; oId CUFF.13558.2; tss_id TSS7232;
1	Cufflinks	exon	296096234	296096856	.	+	.	gene_id GRMZM2G351160; transcript_id TCONS_00003495; exon_number 17; oId CUFF.13558.2; tss_id TSS7232;
1	Cufflinks	exon	296091950	296092519	.	+	.	gene_id GRMZM2G351160; transcript_id TCONS_00003496; exon_number 1; oId CUFF.13558.4; tss_id TSS7234;
1	Cufflinks	exon	296092872	296092956	.	+	.	gene_id GRMZM2G351160; transcript_id TCONS_00003496; exon_number 2; oId CUFF.13558.4; tss_id TSS7234;

1	Cufflinks	exon	296094168	296094320	.	+	.	gene_id GRMZM2G351160; transcript_id TCONS_00003496; exon_number 3; oId CUFF.13558.4; tss_id TSS7234;
1	Cufflinks	exon	296094726	296094820	.	+	.	gene_id GRMZM2G351160; transcript_id TCONS_00003496; exon_number 4; oId CUFF.13558.4; tss_id TSS7234;
1	Cufflinks	exon	296094899	296095075	.	+	.	gene_id GRMZM2G351160; transcript_id TCONS_00003496; exon_number 5; oId CUFF.13558.4; tss_id TSS7234;
1	Cufflinks	exon	296095162	296095265	.	+	.	gene_id GRMZM2G351160; transcript_id TCONS_00003496; exon_number 6; oId CUFF.13558.4; tss_id TSS7234;
1	Cufflinks	exon	296095814	296095883	.	+	.	gene_id GRMZM2G351160; transcript_id TCONS_00003496; exon_number 7; oId CUFF.13558.4; tss_id TSS7234;
1	Cufflinks	exon	296096024	296096116	.	+	.	gene_id GRMZM2G351160; transcript_id TCONS_00003496; exon_number 8; oId CUFF.13558.4; tss_id TSS7234;
1	Cufflinks	exon	296096234	296096856	.	+	.	gene_id GRMZM2G351160; transcript_id TCONS_00003496; exon_number 9; oId CUFF.13558.4; tss_id TSS7234;
1	Cufflinks	exon	298212453	298212781	.	+	.	gene_id XLOC_003067; transcript_id TCONS_00003553; exon_number 1; oId CUFF.13592.1; tss_id TSS7343;
1	Cufflinks	exon	298819416	298819576	.	+	.	gene_id XLOC_003086; transcript_id TCONS_00003575; exon_number 1; oId CUFF.13650.1; tss_id TSS7373;
1	Cufflinks	exon	298819709	298820340	.	+	.	gene_id XLOC_003086; transcript_id TCONS_00003575; exon_number 2; oId CUFF.13650.1; tss_id TSS7373;
1	Cufflinks	exon	299980674	299981223	.	+	.	gene_id XLOC_003103; transcript_id TCONS_00003593; exon_number 1; oId CUFF.13781.1; tss_id TSS7415;
1	Cufflinks	exon	645004	646000	.	-	.	gene_id GRMZM2G032104; transcript_id TCONS_00003634; exon_number 1; oId CUFF.75.1; tss_id TSS7522;
1	Cufflinks	exon	646083	646153	.	-	.	gene_id GRMZM2G032104; transcript_id TCONS_00003634; exon_number 2; oId CUFF.75.1; tss_id TSS7522;
1	Cufflinks	exon	646225	646376	.	-	.	gene_id GRMZM2G032104; transcript_id TCONS_00003634; exon_number 3; oId CUFF.75.1; tss_id TSS7522;
1	Cufflinks	exon	646465	646711.	-	.	gene_id GRMZM2G032104; transcript_id TCONS_00003634; exon_number 4; oId CUFF.75.1; tss_id TSS7522;	
1	Cufflinks	exon	646783	646842	.	-	.	gene_id GRMZM2G032104; transcript_id TCONS_00003634; exon_number 5; oId CUFF.75.1; tss_id TSS7522;
1	Cufflinks	exon	647379	647466	.	-	.	gene_id GRMZM2G032104; transcript_id TCONS_00003634; exon_number 6; oId CUFF.75.1; tss_id TSS7522;
1	Cufflinks	exon	647626	647756	.	-	.	gene_id GRMZM2G032104; transcript_id TCONS_00003634; exon_number 7; oId CUFF.75.1; tss_id TSS7522;
1	Cufflinks	exon	647844	647987	.	-	.	gene_id GRMZM2G032104; transcript_id TCONS_00003634; exon_number 8; oId CUFF.75.1; tss_id TSS7522;
1	Cufflinks	exon	648061	648111.	-	.	gene_id GRMZM2G032104; transcript_id TCONS_00003634; exon_number 9; oId CUFF.75.1; tss_id TSS7522;	
1	Cufflinks	exon	648732	648806	.	-	.	gene_id GRMZM2G032104; transcript_id TCONS_00003634; exon_number 10; oId CUFF.75.1; tss_id TSS7522;
1	Cufflinks	exon	648924	649031	.	-	.	gene_id GRMZM2G032104; transcript_id TCONS_00003634; exon_number 11; oId CUFF.75.1; tss_id TSS7522;
1	Cufflinks	exon	649134	649181	.	-	.	gene_id GRMZM2G032104; transcript_id TCONS_00003634; exon_number 12; oId CUFF.75.1; tss_id TSS7522;
1	Cufflinks	exon	649506	649655	.	-	.	gene_id GRMZM2G032104; transcript_id TCONS_00003634; exon_number 13; oId CUFF.75.1; tss_id TSS7522;
1	Cufflinks	exon	649848	649948	.	-	.	gene_id GRMZM2G032104; transcript_id TCONS_00003634; exon_number 14; oId CUFF.75.1; tss_id TSS7522;
1	Cufflinks	exon	650103	650250	.	-	.	gene_id GRMZM2G032104; transcript_id TCONS_00003634; exon_number 15; oId CUFF.75.1; tss_id TSS7522;
1	Cufflinks	exon	650696	652666	.	-	.	gene_id GRMZM2G032104; transcript_id TCONS_00003634; exon_number 16; oId CUFF.75.1; tss_id TSS7522;
1	Cufflinks	exon	2873956	2874561	.	-	.	gene_id GRMZM2G015384; transcript_id TCONS_00003676; exon_number 1; oId CUFF.272.1; tss_id TSS7619;
1	Cufflinks	exon	2874646	2874714	.	-	.	gene_id GRMZM2G015384; transcript_id TCONS_00003676; exon_number 2; oId CUFF.272.1; tss_id TSS7619;
1	Cufflinks	exon	2874797	2874861	.	-	.	gene_id GRMZM2G015384; transcript_id TCONS_00003676; exon_number 3; oId CUFF.272.1; tss_id TSS7619;
1	Cufflinks	exon	2887065	2887127	.	-	.	gene_id GRMZM2G015384; transcript_id TCONS_00003676; exon_number 4; oId CUFF.272.1; tss_id TSS7619;
1	Cufflinks	exon	2887228	2887290	.	-	.	gene_id GRMZM2G015384; transcript_id TCONS_00003676; exon_number 5; oId CUFF.272.1; tss_id TSS7619;
1	Cufflinks	exon	2887387	2887446	.	-	.	gene_id GRMZM2G015384; transcript_id TCONS_00003676; exon_number 6; oId CUFF.272.1; tss_id TSS7619;
1	Cufflinks	exon	2887870	2887936	.	-	.	gene_id GRMZM2G015384; transcript_id TCONS_00003676; exon_number 7; oId CUFF.272.1; tss_id TSS7619;
1	Cufflinks	exon	2888393	2888444	.	-	.	gene_id GRMZM2G015384; transcript_id TCONS_00003676; exon_number 8; oId CUFF.272.1; tss_id TSS7619;
1	Cufflinks	exon	2888654	2888714	.	-	.	gene_id GRMZM2G015384; transcript_id TCONS_00003676; exon_number 9; oId CUFF.272.1; tss_id TSS7619;

1	Cufflinks	exon	2888800	2888853	.	-	.	gene_id GRMZM2G015384; transcript_id TCONS_00003676; exon_number 10; oId CUFF.272.1; tss_id TSS7619;
1	Cufflinks	exon	2890120	2890173	.	-	.	gene_id GRMZM2G015384; transcript_id TCONS_00003676; exon_number 11; oId CUFF.272.1; tss_id TSS7619;
1	Cufflinks	exon	2890258	2890335	.	-	.	gene_id GRMZM2G015384; transcript_id TCONS_00003676; exon_number 12; oId CUFF.272.1; tss_id TSS7619;
1	Cufflinks	exon	2890525	2890579	.	-	.	gene_id GRMZM2G015384; transcript_id TCONS_00003676; exon_number 13; oId CUFF.272.1; tss_id TSS7619;
1	Cufflinks	exon	2891285	2891334	.	-	.	gene_id GRMZM2G015384; transcript_id TCONS_00003676; exon_number 14; oId CUFF.272.1; tss_id TSS7619;
1	Cufflinks	exon	2891413	2891473	.	-	.	gene_id GRMZM2G015384; transcript_id TCONS_00003676; exon_number 15; oId CUFF.272.1; tss_id TSS7619;
1	Cufflinks	exon	2892150	2892230	.	-	.	gene_id GRMZM2G015384; transcript_id TCONS_00003676; exon_number 16; oId CUFF.272.1; tss_id TSS7619;
1	Cufflinks	exon	2892605	2892678	.	-	.	gene_id GRMZM2G015384; transcript_id TCONS_00003676; exon_number 17; oId CUFF.272.1; tss_id TSS7619;
1	Cufflinks	exon	2892771	2892944	.	-	.	gene_id GRMZM2G015384; transcript_id TCONS_00003676; exon_number 18; oId CUFF.272.1; tss_id TSS7619;
1	Cufflinks	exon	2893943	2894034	.	-	.	gene_id GRMZM2G015384; transcript_id TCONS_00003676; exon_number 19; oId CUFF.272.1; tss_id TSS7619;
1	Cufflinks	exon	2894111	2894310	.	-	.	gene_id GRMZM2G015384; transcript_id TCONS_00003676; exon_number 20; oId CUFF.272.1; tss_id TSS7619;
1	Cufflinks	exon	3130242	3130740	.	-	.	gene_id XLOC_003180; transcript_id TCONS_00003681; exon_number 1; oId CUFF.222.1; tss_id TSS7639;
1	Cufflinks	exon	4855668	4856246	.	-	.	gene_id GRMZM2G026223; transcript_id TCONS_00003721; exon_number 1; oId CUFF.476.1; tss_id TSS7700;
1	Cufflinks	exon	4856444	4856485	.	-	.	gene_id GRMZM2G026223; transcript_id TCONS_00003721; exon_number 2; oId CUFF.476.1; tss_id TSS7700;
1	Cufflinks	exon	4856578	4856619	.	-	.	gene_id GRMZM2G026223; transcript_id TCONS_00003721; exon_number 3; oId CUFF.476.1; tss_id TSS7700;
1	Cufflinks	exon	4856733	4856832	.	-	.	gene_id GRMZM2G026223; transcript_id TCONS_00003721; exon_number 4; oId CUFF.476.1; tss_id TSS7700;
1	Cufflinks	exon	4856906	4856967	.	-	.	gene_id GRMZM2G026223; transcript_id TCONS_00003721; exon_number 5; oId CUFF.476.1; tss_id TSS7700;
1	Cufflinks	exon	4857530	4857608	.	-	.	gene_id GRMZM2G026223; transcript_id TCONS_00003721; exon_number 6; oId CUFF.476.1; tss_id TSS7700;
1	Cufflinks	exon	4870586	4870868	.	-	.	gene_id GRMZM2G026223; transcript_id TCONS_00003721; exon_number 7; oId CUFF.476.1; tss_id TSS7700;
1	Cufflinks	exon	4870986	4871367	.	-	.	gene_id GRMZM2G026223; transcript_id TCONS_00003721; exon_number 8; oId CUFF.476.1; tss_id TSS7700;
1	Cufflinks	exon	5435129	5436156	.	-	.	gene_id XLOC_003222; transcript_id TCONS_00003731; exon_number 1; oId CUFF.427.1; tss_id TSS7716;
1	Cufflinks	exon	10069879	10070768	.	-	.	gene_id XLOC_003309; transcript_id TCONS_00003830; exon_number 1; oId CUFF.787.1; tss_id TSS7892;
1	Cufflinks	exon	15991596	15992246	.	-	.	gene_id XLOC_003390; transcript_id TCONS_00003926; exon_number 1; oId CUFF.1207.2; tss_id TSS8067;
1	Cufflinks	exon	17459873	17461086	.	-	.	gene_id GRMZM2G129674; transcript_id TCONS_00003947; exon_number 1; oId CUFF.1297.1; tss_id TSS8121;
1	Cufflinks	exon	17461269	17461915	.	-	.	gene_id GRMZM2G129674; transcript_id TCONS_00003947; exon_number 2; oId CUFF.1297.1; tss_id TSS8121;
1	Cufflinks	exon	17462504	17462949	.	-	.	gene_id GRMZM2G129674; transcript_id TCONS_00003947; exon_number 3; oId CUFF.1297.1; tss_id TSS8121;
1	Cufflinks	exon	17459873	17461086	.	-	.	gene_id GRMZM2G129674; transcript_id TCONS_00003948; exon_number 1; oId CUFF.1297.2; tss_id TSS8121;
1	Cufflinks	exon	17461269	17461915	.	-	.	gene_id GRMZM2G129674; transcript_id TCONS_00003948; exon_number 2; oId CUFF.1297.2; tss_id TSS8121;
1	Cufflinks	exon	17462033	17462395	.	-	.	gene_id GRMZM2G129674; transcript_id TCONS_00003948; exon_number 3; oId CUFF.1297.2; tss_id TSS8121;
1	Cufflinks	exon	17462504	17462949	.	-	.	gene_id GRMZM2G129674; transcript_id TCONS_00003948; exon_number 4; oId CUFF.1297.2; tss_id TSS8121;
1	Cufflinks	exon	21247887	21250509	.	-	.	gene_id GRMZM2G464510; transcript_id TCONS_00003997; exon_number 1; oId CUFF.1504.1; tss_id TSS8224;
1	Cufflinks	exon	22435812	22437908	.	-	.	gene_id GRMZM2G077127; transcript_id TCONS_00004011; exon_number 1; oId CUFF.1570.1; tss_id TSS8254;
1	Cufflinks	exon	22438023	22439519	.	-	.	gene_id GRMZM2G077127; transcript_id TCONS_00004011; exon_number 2; oId CUFF.1570.1; tss_id TSS8254;
1	Cufflinks	exon	27099652	27100161	.	-	.	gene_id GRMZM2G163154; transcript_id TCONS_00004058; exon_number 1; oId CUFF.1822.1; tss_id TSS8364;
1	Cufflinks	exon	27100260	27100355	.	-	.	gene_id GRMZM2G163154; transcript_id TCONS_00004058; exon_number 2; oId CUFF.1822.1; tss_id TSS8364;
1	Cufflinks	exon	27100458	27100574	.	-	.	gene_id GRMZM2G163154; transcript_id TCONS_00004058; exon_number 3; oId CUFF.1822.1; tss_id TSS8364;

1	Cufflinks	exon	27100683	27100754	.	-	.	gene_id GRMZM2G163154; transcript_id TCONS_00004058; exon_number 4; oId CUFF.1822.1; tss_id TSS8364;
1	Cufflinks	exon	27100867	27101030	.	-	.	gene_id GRMZM2G163154; transcript_id TCONS_00004058; exon_number 5; oId CUFF.1822.1; tss_id TSS8364;
1	Cufflinks	exon	27101112	27101212	.	-	.	gene_id GRMZM2G163154; transcript_id TCONS_00004058; exon_number 6; oId CUFF.1822.1; tss_id TSS8364;
1	Cufflinks	exon	27101335	27101431	.	-	.	gene_id GRMZM2G163154; transcript_id TCONS_00004058; exon_number 7; oId CUFF.1822.1; tss_id TSS8364;
1	Cufflinks	exon	27101542	27101677	.	-	.	gene_id GRMZM2G163154; transcript_id TCONS_00004058; exon_number 8; oId CUFF.1822.1; tss_id TSS8364;
1	Cufflinks	exon	27101784	27101989	.	-	.	gene_id GRMZM2G163154; transcript_id TCONS_00004058; exon_number 9; oId CUFF.1822.1; tss_id TSS8364;
1	Cufflinks	exon	27102113	27102331	.	-	.	gene_id GRMZM2G163154; transcript_id TCONS_00004058; exon_number 10; oId CUFF.1822.1; tss_id TSS8364;
1	Cufflinks	exon	27102888	27103009	.	-	.	gene_id GRMZM2G163154; transcript_id TCONS_00004058; exon_number 11; oId CUFF.1822.1; tss_id TSS8364;
1	Cufflinks	exon	27103116	27108024	.	-	.	gene_id GRMZM2G163154; transcript_id TCONS_00004058; exon_number 12; oId CUFF.1822.1; tss_id TSS8364;
1	Cufflinks	exon	27125856	27126315	.	-	.	gene_id AC208897.3_FG004; transcript_id TCONS_00004060; exon_number 1; oId CUFF.1786.1; tss_id TSS8367;
1	Cufflinks	exon	27128033	27128302	.	-	.	gene_id AC208897.3_FG004; transcript_id TCONS_00004060; exon_number 2; oId CUFF.1786.1; tss_id TSS8367;
1	Cufflinks	exon	29540914	29543011	.	-	.	gene_id GRMZM2G126808; transcript_id TCONS_00004097; exon_number 1; oId CUFF.1955.1; tss_id TSS8450;
1	Cufflinks	exon	29543060	29543201	.	-	.	gene_id GRMZM2G126808; transcript_id TCONS_00004097; exon_number 2; oId CUFF.1955.1; tss_id TSS8450;
1	Cufflinks	exon	29543435	29543514	.	-	.	gene_id GRMZM2G126808; transcript_id TCONS_00004097; exon_number 3; oId CUFF.1955.1; tss_id TSS8450;
1	Cufflinks	exon	29543942	29544577	.	-	.	gene_id GRMZM2G126808; transcript_id TCONS_00004097; exon_number 4; oId CUFF.1955.1; tss_id TSS8450;
1	Cufflinks	exon	31253767	31254533	.	-	.	gene_id GRMZM2G116902; transcript_id TCONS_00004115; exon_number 1; oId CUFF.2031.1; tss_id TSS8500;
1	Cufflinks	exon	31254672	31254837	.	-	.	gene_id GRMZM2G116902; transcript_id TCONS_00004115; exon_number 2; oId CUFF.2031.1; tss_id TSS8500;
1	Cufflinks	exon	31254954	31255136	.	-	.	gene_id GRMZM2G116902; transcript_id TCONS_00004115; exon_number 3; oId CUFF.2031.1; tss_id TSS8500;
1	Cufflinks	exon	31255316	31255665	.	-	.	gene_id GRMZM2G116902; transcript_id TCONS_00004115; exon_number 4; oId CUFF.2031.1; tss_id TSS8500;
1	Cufflinks	exon	32322594	32323169	.	-	.	gene_id GRMZM2G099528; transcript_id TCONS_00004134; exon_number 1; oId CUFF.2115.1; tss_id TSS8539;
1	Cufflinks	exon	32324095	32324190	.	-	.	gene_id GRMZM2G099528; transcript_id TCONS_00004134; exon_number 2; oId CUFF.2115.1; tss_id TSS8539;
1	Cufflinks	exon	32324287	32324342	.	-	.	gene_id GRMZM2G099528; transcript_id TCONS_00004134; exon_number 3; oId CUFF.2115.1; tss_id TSS8539;
1	Cufflinks	exon	32326158	32326240	.	-	.	gene_id GRMZM2G099528; transcript_id TCONS_00004134; exon_number 4; oId CUFF.2115.1; tss_id TSS8539;
1	Cufflinks	exon	32326338	32326736	.	-	.	gene_id GRMZM2G099528; transcript_id TCONS_00004134; exon_number 5; oId CUFF.2115.1; tss_id TSS8539;
1	Cufflinks	exon	36160110	36162737	.	-	.	gene_id GRMZM2G338702; transcript_id TCONS_00004187; exon_number 1; oId CUFF.2297.1; tss_id TSS8654;
1	Cufflinks	exon	36164251	36164398	.	-	.	gene_id GRMZM2G338702; transcript_id TCONS_00004187; exon_number 2; oId CUFF.2297.1; tss_id TSS8654;
1	Cufflinks	exon	36165679	36165881	.	-	.	gene_id GRMZM2G338702; transcript_id TCONS_00004187; exon_number 3; oId CUFF.2297.1; tss_id TSS8654;
1	Cufflinks	exon	42851939	42853975	.	-	.	gene_id GRMZM2G141704; transcript_id TCONS_00004287; exon_number 1; oId CUFF.2652.1; tss_id TSS8832;
1	Cufflinks	exon	42854693	42854921	.	-	.	gene_id GRMZM2G141704; transcript_id TCONS_00004287; exon_number 2; oId CUFF.2652.1; tss_id TSS8832;
1	Cufflinks	exon	42855014	42855123	.	-	.	gene_id GRMZM2G141704; transcript_id TCONS_00004287; exon_number 3; oId CUFF.2652.1; tss_id TSS8832;
1	Cufflinks	exon	42855204	42855499	.	-	.	gene_id GRMZM2G141704; transcript_id TCONS_00004287; exon_number 4; oId CUFF.2652.1; tss_id TSS8832;
1	Cufflinks	exon	44959567	44960104	.	-	.	gene_id AC212219.3_FG005; transcript_id TCONS_00004310; exon_number 1; oId CUFF.2767.1; tss_id TSS8888;
1	Cufflinks	exon	44964047	44964383	.	-	.	gene_id AC212219.3_FG005; transcript_id TCONS_00004310; exon_number 2; oId CUFF.2767.1; tss_id TSS8888;
1	Cufflinks	exon	44964532	44965488	.	-	.	gene_id AC212219.3_FG005; transcript_id TCONS_00004310; exon_number 3; oId CUFF.2767.1; tss_id TSS8888;
1	Cufflinks	exon	45371092	45371148	.	-	.	gene_id XLOC_003727; transcript_id TCONS_00004317; exon_number 1; oId CUFF.2778.1; tss_id TSS8897;
1	Cufflinks	exon	45371302	45372046	.	-	.	gene_id XLOC_003727; transcript_id TCONS_00004317; exon_number 2; oId CUFF.2778.1; tss_id TSS8897;

1	Cufflinks	exon	45640504	45641402	.	-	.	gene_id XLOC_003731; transcript_id TCONS_00004321; exon_number 1; oId CUFF.2828.4; tss_id TSS8908;
1	Cufflinks	exon	47329448	47330074	.	-	.	gene_id GRMZM2G016734; transcript_id TCONS_00004358; exon_number 1; oId CUFF.2905.1; tss_id TSS8952;
1	Cufflinks	exon	47330171	47330227	.	-	.	gene_id GRMZM2G016734; transcript_id TCONS_00004358; exon_number 2; oId CUFF.2905.1; tss_id TSS8952;
1	Cufflinks	exon	47330422	47330492	.	-	.	gene_id GRMZM2G016734; transcript_id TCONS_00004358; exon_number 3; oId CUFF.2905.1; tss_id TSS8952;
1	Cufflinks	exon	47330581	47330668	.	-	.	gene_id GRMZM2G016734; transcript_id TCONS_00004358; exon_number 4; oId CUFF.2905.1; tss_id TSS8952;
1	Cufflinks	exon	47330815	47331142	.	-	.	gene_id GRMZM2G016734; transcript_id TCONS_00004358; exon_number 5; oId CUFF.2905.1; tss_id TSS8952;
1	Cufflinks	exon	47510283	47511240	.	-	.	gene_id XLOC_003761; transcript_id TCONS_00004361; exon_number 1; oId CUFF.2910.1; tss_id TSS8959;
1	Cufflinks	exon	48358409	48358808	.	-	.	gene_id XLOC_003766; transcript_id TCONS_00004366; exon_number 1; oId CUFF.2954.1; tss_id TSS8965;
1	Cufflinks	exon	52392189	52393041	.	-	.	gene_id GRMZM2G009060; transcript_id TCONS_00004411; exon_number 1; oId CUFF.3216.1; tss_id TSS9072;
1	Cufflinks	exon	52394008	52394077	.	-	.	gene_id GRMZM2G009060; transcript_id TCONS_00004411; exon_number 2; oId CUFF.3216.1; tss_id TSS9072;
1	Cufflinks	exon	52394146	52394233	.	-	.	gene_id GRMZM2G009060; transcript_id TCONS_00004411; exon_number 3; oId CUFF.3216.1; tss_id TSS9072;
1	Cufflinks	exon	52394315	52394372	.	-	.	gene_id GRMZM2G009060; transcript_id TCONS_00004411; exon_number 4; oId CUFF.3216.1; tss_id TSS9072;
1	Cufflinks	exon	52394452	52394528	.	-	.	gene_id GRMZM2G009060; transcript_id TCONS_00004411; exon_number 5; oId CUFF.3216.1; tss_id TSS9072;
1	Cufflinks	exon	52394609	52394822	.	-	.	gene_id GRMZM2G009060; transcript_id TCONS_00004411; exon_number 6; oId CUFF.3216.1; tss_id TSS9072;
1	Cufflinks	exon	52394932	52395169	.	-	.	gene_id GRMZM2G009060; transcript_id TCONS_00004411; exon_number 7; oId CUFF.3216.1; tss_id TSS9072;
1	Cufflinks	exon	52392189	52393062	.	-	.	gene_id GRMZM2G009060; transcript_id TCONS_00004412; exon_number 1; oId CUFF.3216.3; tss_id TSS9072;
1	Cufflinks	exon	52394014	52394077	.	-	.	gene_id GRMZM2G009060; transcript_id TCONS_00004412; exon_number 2; oId CUFF.3216.3; tss_id TSS9072;
1	Cufflinks	exon	52394146	52394233	.	-	.	gene_id GRMZM2G009060; transcript_id TCONS_00004412; exon_number 3; oId CUFF.3216.3; tss_id TSS9072;
1	Cufflinks	exon	52394315	52394372	.	-	.	gene_id GRMZM2G009060; transcript_id TCONS_00004412; exon_number 4; oId CUFF.3216.3; tss_id TSS9072;
1	Cufflinks	exon	52394452	52394528	.	-	.	gene_id GRMZM2G009060; transcript_id TCONS_00004412; exon_number 5; oId CUFF.3216.3; tss_id TSS9072;
1	Cufflinks	exon	52394609	52394822	.	-	.	gene_id GRMZM2G009060; transcript_id TCONS_00004412; exon_number 6; oId CUFF.3216.3; tss_id TSS9072;
1	Cufflinks	exon	52394932	52395169	.	-	.	gene_id GRMZM2G009060; transcript_id TCONS_00004412; exon_number 7; oId CUFF.3216.3; tss_id TSS9072;
1	Cufflinks	exon	54602876	54603329	.	-	.	gene_id GRMZM2G312661; transcript_id TCONS_00004459; exon_number 1; oId CUFF.3335.3; tss_id TSS9144;
1	Cufflinks	exon	54604626	54605395	.	-	.	gene_id GRMZM2G312661; transcript_id TCONS_00004459; exon_number 2; oId CUFF.3335.3; tss_id TSS9144;
1	Cufflinks	exon	61943533	61944858	.	-	.	gene_id XLOC_003919; transcript_id TCONS_00004544; exon_number 1; oId CUFF.3703.1; tss_id TSS9341;
1	Cufflinks	exon	74718500	74719924	.	-	.	gene_id GRMZM2G304442; transcript_id TCONS_00004680; exon_number 1; oId CUFF.4224.1; tss_id TSS9612;
1	Cufflinks	exon	81589129	81589704	.	-	.	gene_id XLOC_004105; transcript_id TCONS_00004758; exon_number 1; oId CUFF.4539.1; tss_id TSS9798;
1	Cufflinks	exon	88487882	88491179	.	-	.	gene_id GRMZM2G079470; transcript_id TCONS_00004820; exon_number 1; oId CUFF.4777.1; tss_id TSS9923;
1	Cufflinks	exon	92793547	92794797	.	-	.	gene_id GRMZM2G161459; transcript_id TCONS_00004847; exon_number 1; oId CUFF.4879.1; tss_id TSS9980;
1	Cufflinks	exon	92796474	92797054	.	-	.	gene_id GRMZM2G161459; transcript_id TCONS_00004847; exon_number 2; oId CUFF.4879.1; tss_id TSS9980;
1	Cufflinks	exon	92797162	92797379	.	-	.	gene_id GRMZM2G161459; transcript_id TCONS_00004847; exon_number 3; oId CUFF.4879.1; tss_id TSS9980;
1	Cufflinks	exon	92797553	92797895	.	-	.	gene_id GRMZM2G161459; transcript_id TCONS_00004847; exon_number 4; oId CUFF.4879.1; tss_id TSS9980;
1	Cufflinks	exon	103834561	103835137	.	-	.	gene_id GRMZM5G866843; transcript_id TCONS_00004896; exon_number 1; oId CUFF.5148.4; tss_id TSS10077;
1	Cufflinks	exon	103835402	103835497	.	-	.	gene_id GRMZM5G866843; transcript_id TCONS_00004896; exon_number 2; oId CUFF.5148.4; tss_id TSS10077;
1	Cufflinks	exon	103835595	103835777	.	-	.	gene_id GRMZM5G866843; transcript_id TCONS_00004896; exon_number 3; oId CUFF.5148.4; tss_id TSS10077;
1	Cufflinks	exon	103836075	103836160	.	-	.	gene_id GRMZM5G866843; transcript_id TCONS_00004896; exon_number 4; oId CUFF.5148.4; tss_id TSS10077;

1	Cufflinks	exon	103836262	103836503	.	-	.	gene_id GRMZM5G866843; transcript_id TCONS_00004896; exon_number 5; oId CUFF.5148.4; tss_id TSS10077;
1	Cufflinks	exon	103836829	103838119	.	-	.	gene_id GRMZM5G866843; transcript_id TCONS_00004896; exon_number 6; oId CUFF.5148.4; tss_id TSS10077;
1	Cufflinks	exon	103834561	103835134	.	-	.	gene_id GRMZM5G866843; transcript_id TCONS_00004897; exon_number 1; oId CUFF.5148.1; tss_id TSS10078;
1	Cufflinks	exon	103835402	103835497	.	-	.	gene_id GRMZM5G866843; transcript_id TCONS_00004897; exon_number 2; oId CUFF.5148.1; tss_id TSS10078;
1	Cufflinks	exon	103835595	103835777	.	-	.	gene_id GRMZM5G866843; transcript_id TCONS_00004897; exon_number 3; oId CUFF.5148.1; tss_id TSS10078;
1	Cufflinks	exon	103836075	103836160	.	-	.	gene_id GRMZM5G866843; transcript_id TCONS_00004897; exon_number 4; oId CUFF.5148.1; tss_id TSS10078;
1	Cufflinks	exon	103836262	103836503	.	-	.	gene_id GRMZM5G866843; transcript_id TCONS_00004897; exon_number 5; oId CUFF.5148.1; tss_id TSS10078;
1	Cufflinks	exon	103836829	103838408	.	-	.	gene_id GRMZM5G866843; transcript_id TCONS_00004897; exon_number 6; oId CUFF.5148.1; tss_id TSS10078;
1	Cufflinks	exon	111555606	111556464	.	-	.	gene_id XLOC_004266; transcript_id TCONS_00004941; exon_number 1; oId CUFF.5259.2; tss_id TSS10180;
1	Cufflinks	exon	126122680	126124282	.	-	.	gene_id XLOC_004341; transcript_id TCONS_00005018; exon_number 1; oId CUFF.5582.1; tss_id TSS10355;
1	Cufflinks	exon	140725406	140726375	.	-	.	gene_id GRMZM2G159587; transcript_id TCONS_00005066; exon_number 1; oId CUFF.5866.1; tss_id TSS10512;
1	Cufflinks	exon	140729166	140729780	.	-	.	gene_id GRMZM2G159587; transcript_id TCONS_00005066; exon_number 2; oId CUFF.5866.1; tss_id TSS10512;
1	Cufflinks	exon	165420894	165421789	.	-	.	gene_id XLOC_004506; transcript_id TCONS_00005197; exon_number 1; oId CUFF.6505.1; tss_id TSS10844;
1	Cufflinks	exon	180651899	180653885	.	-	.	gene_id GRMZM2G310947; transcript_id TCONS_00005346; exon_number 1; oId CUFF.7054.1; tss_id TSS11169;
1	Cufflinks	exon	180717585	180718436	.	-	.	gene_id XLOC_004639; transcript_id TCONS_00005347; exon_number 1; oId CUFF.7083.1; tss_id TSS11170;
1	Cufflinks	exon	183477478	183479191	.	-	.	gene_id GRMZM2G042488; transcript_id TCONS_00005377; exon_number 1; oId CUFF.7222.1; tss_id TSS11237;
1	Cufflinks	exon	187433111	187434213	.	-	.	gene_id XLOC_004709; transcript_id TCONS_00005421; exon_number 1; oId CUFF.7352.1; tss_id TSS11315;
1	Cufflinks	exon	188151948	188153420	.	-	.	gene_id XLOC_004719; transcript_id TCONS_00005431; exon_number 1; oId CUFF.7403.1; tss_id TSS11334;
1	Cufflinks	exon	197105380	197106330	.	-	.	gene_id XLOC_004799; transcript_id TCONS_00005524; exon_number 1; oId CUFF.7845.1; tss_id TSS11525;
1	Cufflinks	exon	200504383	200506023	.	-	.	gene_id XLOC_004841; transcript_id TCONS_00005569; exon_number 1; oId CUFF.7977.1; tss_id TSS11611;
1	Cufflinks	exon	208340744	208341268	.	-	.	gene_id GRMZM2G101268; transcript_id TCONS_00005686; exon_number 1; oId CUFF.8356.1; tss_id TSS11814;
1	Cufflinks	exon	216924085	216924463	.	-	.	gene_id XLOC_005022; transcript_id TCONS_00005783; exon_number 1; oId CUFF.8788.1; tss_id TSS12045;
1	Cufflinks	exon	218469225	218469942	.	-	.	gene_id GRMZM2G114093; transcript_id TCONS_00005796; exon_number 1; oId CUFF.8867.1; tss_id TSS12073;
1	Cufflinks	exon	218470177	218470632	.	-	.	gene_id GRMZM2G114093; transcript_id TCONS_00005796; exon_number 2; oId CUFF.8867.1; tss_id TSS12073;
1	Cufflinks	exon	218471436	218472813	.	-	.	gene_id GRMZM2G114093; transcript_id TCONS_00005796; exon_number 3; oId CUFF.8867.1; tss_id TSS12073;
1	Cufflinks	exon	218473395	218473811	.	-	.	gene_id GRMZM2G114093; transcript_id TCONS_00005796; exon_number 4; oId CUFF.8867.1; tss_id TSS12073;
1	Cufflinks	exon	220148133	220148722	.	-	.	gene_id XLOC_005054; transcript_id TCONS_00005821; exon_number 1; oId CUFF.8916.1; tss_id TSS12111;
1	Cufflinks	exon	227894417	227895044	.	-	.	gene_id XLOC_005176; transcript_id TCONS_00005977; exon_number 1; oId CUFF.9403.1; tss_id TSS12379;
1	Cufflinks	exon	233290453	233290765	.	-	.	gene_id GRMZM2G134747; transcript_id TCONS_00006050; exon_number 1; oId CUFF.9681.1; tss_id TSS12565;
1	Cufflinks	exon	233291049	233291157	.	-	.	gene_id GRMZM2G134747; transcript_id TCONS_00006050; exon_number 2; oId CUFF.9681.1; tss_id TSS12565;
1	Cufflinks	exon	233291582	233291653	.	-	.	gene_id GRMZM2G134747; transcript_id TCONS_00006050; exon_number 3; oId CUFF.9681.1; tss_id TSS12565;
1	Cufflinks	exon	233293210	233293630	.	-	.	gene_id GRMZM2G134747; transcript_id TCONS_00006050; exon_number 4; oId CUFF.9681.1; tss_id TSS12565;
1	Cufflinks	exon	233875216	233877958	.	-	.	gene_id GRMZM2G398198; transcript_id TCONS_00006065; exon_number 1; oId CUFF.9713.1; tss_id TSS12590;
1	Cufflinks	exon	234706337	234718755	.	-	.	gene_id GRMZM2G450825; transcript_id TCONS_00006080; exon_number 1; oId CUFF.9903.1; tss_id TSS12654;
1	Cufflinks	exon	235769492	235770313	.	-	.	gene_id XLOC_005293; transcript_id TCONS_00006108; exon_number 1; oId CUFF.9983.1; tss_id TSS12722;
1	Cufflinks	exon	235770659	235770778	.	-	.	gene_id XLOC_005293; transcript_id TCONS_00006108; exon_number 2; oId CUFF.9983.1; tss_id TSS12722;

1	Cufflinks	exon	235771507	235771572	.	-	.	gene_id XLOC_005293; transcript_id TCONS_00006108; exon_number 3; oId CUFF.9983.1; tss_id TSS12722;
1	Cufflinks	exon	235771661	235771709	.	-	.	gene_id XLOC_005293; transcript_id TCONS_00006108; exon_number 4; oId CUFF.9983.1; tss_id TSS12722;
1	Cufflinks	exon	235773520	235773657	.	-	.	gene_id XLOC_005293; transcript_id TCONS_00006108; exon_number 5; oId CUFF.9983.1; tss_id TSS12722;
1	Cufflinks	exon	235774368	235774468	.	-	.	gene_id XLOC_005293; transcript_id TCONS_00006108; exon_number 6; oId CUFF.9983.1; tss_id TSS12722;
1	Cufflinks	exon	235774558	235774668	.	-	.	gene_id XLOC_005293; transcript_id TCONS_00006108; exon_number 7; oId CUFF.9983.1; tss_id TSS12722;
1	Cufflinks	exon	235775200	235775364	.	-	.	gene_id XLOC_005293; transcript_id TCONS_00006108; exon_number 8; oId CUFF.9983.1; tss_id TSS12722;
1	Cufflinks	exon	235775653	235775747	.	-	.	gene_id XLOC_005293; transcript_id TCONS_00006108; exon_number 9; oId CUFF.9983.1; tss_id TSS12722;
1	Cufflinks	exon	235775833	235776073	.	-	.	gene_id XLOC_005293; transcript_id TCONS_00006108; exon_number 10; oId CUFF.9983.1; tss_id TSS12722;
1	Cufflinks	exon	235922074	235923485	.	-	.	gene_id GRMZM2G029518; transcript_id TCONS_00006114; exon_number 1; oId CUFF.9949.1; tss_id TSS12728;
1	Cufflinks	exon	237545346	237548821	.	-	.	gene_id GRMZM2G303965; transcript_id TCONS_00006131; exon_number 1; oId CUFF.10044.1; tss_id TSS12763;
1	Cufflinks	exon	237715089	237715921	.	-	.	gene_id GRMZM2G008290; transcript_id TCONS_00006134; exon_number 1; oId CUFF.10054.1; tss_id TSS12766;
1	Cufflinks	exon	237716057	237716135	.	-	.	gene_id GRMZM2G008290; transcript_id TCONS_00006134; exon_number 2; oId CUFF.10054.1; tss_id TSS12766;
1	Cufflinks	exon	237716236	237716292	.	-	.	gene_id GRMZM2G008290; transcript_id TCONS_00006134; exon_number 3; oId CUFF.10054.1; tss_id TSS12766;
1	Cufflinks	exon	237716401	237716600	.	-	.	gene_id GRMZM2G008290; transcript_id TCONS_00006134; exon_number 4; oId CUFF.10054.1; tss_id TSS12766;
1	Cufflinks	exon	237716720	237717335	.	-	.	gene_id GRMZM2G008290; transcript_id TCONS_00006134; exon_number 5; oId CUFF.10054.1; tss_id TSS12766;
1	Cufflinks	exon	238877136	238878198	.	-	.	gene_id GRMZM2G000540; transcript_id TCONS_00006145; exon_number 1; oId CUFF.10120.1; tss_id TSS12790;
1	Cufflinks	exon	238878865	238879120	.	-	.	gene_id GRMZM2G000540; transcript_id TCONS_00006145; exon_number 2; oId CUFF.10120.1; tss_id TSS12790;
1	Cufflinks	exon	238880077	238880205	.	-	.	gene_id GRMZM2G000540; transcript_id TCONS_00006145; exon_number 3; oId CUFF.10120.1; tss_id TSS12790;
1	Cufflinks	exon	238880498	238880750	.	-	.	gene_id GRMZM2G000540; transcript_id TCONS_00006145; exon_number 4; oId CUFF.10120.1; tss_id TSS12790;
1	Cufflinks	exon	238880847	238880891	.	-	.	gene_id GRMZM2G000540; transcript_id TCONS_00006145; exon_number 5; oId CUFF.10120.1; tss_id TSS12790;
1	Cufflinks	exon	238881947	238882237	.	-	.	gene_id GRMZM2G000540; transcript_id TCONS_00006145; exon_number 6; oId CUFF.10120.1; tss_id TSS12790;
1	Cufflinks	exon	238882311	238882478	.	-	.	gene_id GRMZM2G000540; transcript_id TCONS_00006145; exon_number 7; oId CUFF.10120.1; tss_id TSS12790;
1	Cufflinks	exon	238882663	238882811	.	-	.	gene_id GRMZM2G000540; transcript_id TCONS_00006145; exon_number 8; oId CUFF.10120.1; tss_id TSS12790;
1	Cufflinks	exon	238882913	238883069	.	-	.	gene_id GRMZM2G000540; transcript_id TCONS_00006145; exon_number 9; oId CUFF.10120.1; tss_id TSS12790;
1	Cufflinks	exon	238883276	238883338	.	-	.	gene_id GRMZM2G000540; transcript_id TCONS_00006145; exon_number 10; oId CUFF.10120.1; tss_id TSS12790;
1	Cufflinks	exon	238883598	238883677	.	-	.	gene_id GRMZM2G000540; transcript_id TCONS_00006145; exon_number 11; oId CUFF.10120.1; tss_id TSS12790;
1	Cufflinks	exon	238883889	238884049	.	-	.	gene_id GRMZM2G000540; transcript_id TCONS_00006145; exon_number 12; oId CUFF.10120.1; tss_id TSS12790;
1	Cufflinks	exon	238884189	238884267	.	-	.	gene_id GRMZM2G000540; transcript_id TCONS_00006145; exon_number 13; oId CUFF.10120.1; tss_id TSS12790;
1	Cufflinks	exon	238884460	238884559	.	-	.	gene_id GRMZM2G000540; transcript_id TCONS_00006145; exon_number 14; oId CUFF.10120.1; tss_id TSS12790;
1	Cufflinks	exon	238885196	238886130	.	-	.	gene_id GRMZM2G000540; transcript_id TCONS_00006145; exon_number 15; oId CUFF.10120.1; tss_id TSS12790;
1	Cufflinks	exon	246761616	246761989	.	-	.	gene_id GRMZM2G146589; transcript_id TCONS_00006208; exon_number 1; oId CUFF.10555.2; tss_id TSS12961;
1	Cufflinks	exon	246762766	246762831	.	-	.	gene_id GRMZM2G146589; transcript_id TCONS_00006208; exon_number 2; oId CUFF.10555.2; tss_id TSS12961;
1	Cufflinks	exon	246762949	246763092	.	-	.	gene_id GRMZM2G146589; transcript_id TCONS_00006208; exon_number 3; oId CUFF.10555.2; tss_id TSS12961;
1	Cufflinks	exon	246763388	246763456	.	-	.	gene_id GRMZM2G146589; transcript_id TCONS_00006208; exon_number 4; oId CUFF.10555.2; tss_id TSS12961;
1	Cufflinks	exon	246763535	246763678	.	-	.	gene_id GRMZM2G146589; transcript_id TCONS_00006208; exon_number 5; oId CUFF.10555.2; tss_id TSS12961;
1	Cufflinks	exon	246764109	246764265	.	-	.	gene_id GRMZM2G146589; transcript_id TCONS_00006208; exon_number 6; oId CUFF.10555.2; tss_id TSS12961;

1	Cufflinks	exon	246764387	246764483	.	-	.	gene_id GRMZM2G146589; transcript_id TCONS_00006208; exon_number 7; oId CUFF.10555.2; tss_id TSS12961;
1	Cufflinks	exon	246764590	246764872	.	-	.	gene_id GRMZM2G146589; transcript_id TCONS_00006208; exon_number 8; oId CUFF.10555.2; tss_id TSS12961;
1	Cufflinks	exon	246764940	246765065	.	-	.	gene_id GRMZM2G146589; transcript_id TCONS_00006208; exon_number 9; oId CUFF.10555.2; tss_id TSS12961;
1	Cufflinks	exon	246765679	246765759	.	-	.	gene_id GRMZM2G146589; transcript_id TCONS_00006208; exon_number 10; oId CUFF.10555.2; tss_id TSS12961;
1	Cufflinks	exon	246766039	246766121	.	-	.	gene_id GRMZM2G146589; transcript_id TCONS_00006208; exon_number 11; oId CUFF.10555.2; tss_id TSS12961;
1	Cufflinks	exon	246766438	246766523	.	-	.	gene_id GRMZM2G146589; transcript_id TCONS_00006208; exon_number 12; oId CUFF.10555.2; tss_id TSS12961;
1	Cufflinks	exon	246767965	246768058	.	-	.	gene_id GRMZM2G146589; transcript_id TCONS_00006208; exon_number 13; oId CUFF.10555.2; tss_id TSS12961;
1	Cufflinks	exon	246768137	246768302	.	-	.	gene_id GRMZM2G146589; transcript_id TCONS_00006208; exon_number 14; oId CUFF.10555.2; tss_id TSS12961;
1	Cufflinks	exon	246768393	246768458	.	-	.	gene_id GRMZM2G146589; transcript_id TCONS_00006208; exon_number 15; oId CUFF.10555.2; tss_id TSS12961;
1	Cufflinks	exon	246768628	246768703	.	-	.	gene_id GRMZM2G146589; transcript_id TCONS_00006208; exon_number 16; oId CUFF.10555.2; tss_id TSS12961;
1	Cufflinks	exon	246768809	246769046	.	-	.	gene_id GRMZM2G146589; transcript_id TCONS_00006208; exon_number 17; oId CUFF.10555.2; tss_id TSS12961;
1	Cufflinks	exon	248480724	248480862	.	-	.	gene_id GRMZM2G153928; transcript_id TCONS_00006227; exon_number 1; oId CUFF.10659.1; tss_id TSS12993;
1	Cufflinks	exon	248482467	248482763	.	-	.	gene_id GRMZM2G153928; transcript_id TCONS_00006227; exon_number 2; oId CUFF.10659.1; tss_id TSS12993;
1	Cufflinks	exon	248487690	248487869	.	-	.	gene_id GRMZM2G153928; transcript_id TCONS_00006227; exon_number 3; oId CUFF.10659.1; tss_id TSS12993;
1	Cufflinks	exon	248488183	248488299	.	-	.	gene_id GRMZM2G153928; transcript_id TCONS_00006227; exon_number 4; oId CUFF.10659.1; tss_id TSS12993;
1	Cufflinks	exon	248505987	248506051	.	-	.	gene_id GRMZM2G153928; transcript_id TCONS_00006227; exon_number 5; oId CUFF.10659.1; tss_id TSS12993;
1	Cufflinks	exon	248508463	248508554	.	-	.	gene_id GRMZM2G153928; transcript_id TCONS_00006227; exon_number 6; oId CUFF.10659.1; tss_id TSS12993;
1	Cufflinks	exon	248508748	248508811	.	-	.	gene_id GRMZM2G153928; transcript_id TCONS_00006227; exon_number 7; oId CUFF.10659.1; tss_id TSS12993;
1	Cufflinks	exon	248508921	248508999	.	-	.	gene_id GRMZM2G153928; transcript_id TCONS_00006227; exon_number 8; oId CUFF.10659.1; tss_id TSS12993;
1	Cufflinks	exon	248509074	248509681	.	-	.	gene_id GRMZM2G153928; transcript_id TCONS_00006227; exon_number 9; oId CUFF.10659.1; tss_id TSS12993;
1	Cufflinks	exon	248481851	248482230	.	-	.	gene_id GRMZM2G153928; transcript_id TCONS_00006228; exon_number 1; oId CUFF.10659.3; tss_id TSS12993;
1	Cufflinks	exon	248482467	248482763	.	-	.	gene_id GRMZM2G153928; transcript_id TCONS_00006228; exon_number 2; oId CUFF.10659.3; tss_id TSS12993;
1	Cufflinks	exon	248487690	248487869	.	-	.	gene_id GRMZM2G153928; transcript_id TCONS_00006228; exon_number 3; oId CUFF.10659.3; tss_id TSS12993;
1	Cufflinks	exon	248488183	248488299	.	-	.	gene_id GRMZM2G153928; transcript_id TCONS_00006228; exon_number 4; oId CUFF.10659.3; tss_id TSS12993;
1	Cufflinks	exon	248505987	248506051	.	-	.	gene_id GRMZM2G153928; transcript_id TCONS_00006228; exon_number 5; oId CUFF.10659.3; tss_id TSS12993;
1	Cufflinks	exon	248508463	248508554	.	-	.	gene_id GRMZM2G153928; transcript_id TCONS_00006228; exon_number 6; oId CUFF.10659.3; tss_id TSS12993;
1	Cufflinks	exon	248508748	248508811	.	-	.	gene_id GRMZM2G153928; transcript_id TCONS_00006228; exon_number 7; oId CUFF.10659.3; tss_id TSS12993;
1	Cufflinks	exon	248508921	248508999	.	-	.	gene_id GRMZM2G153928; transcript_id TCONS_00006228; exon_number 8; oId CUFF.10659.3; tss_id TSS12993;
1	Cufflinks	exon	248509074	248509681	.	-	.	gene_id GRMZM2G153928; transcript_id TCONS_00006228; exon_number 9; oId CUFF.10659.3; tss_id TSS12993;
1	Cufflinks	exon	248481851	248482763	.	-	.	gene_id GRMZM2G153928; transcript_id TCONS_00006229; exon_number 1; oId CUFF.10659.2; tss_id TSS12993;
1	Cufflinks	exon	248487690	248487869	.	-	.	gene_id GRMZM2G153928; transcript_id TCONS_00006229; exon_number 2; oId CUFF.10659.2; tss_id TSS12993;
1	Cufflinks	exon	248488183	248488299	.	-	.	gene_id GRMZM2G153928; transcript_id TCONS_00006229; exon_number 3; oId CUFF.10659.2; tss_id TSS12993;
1	Cufflinks	exon	248505987	248506051	.	-	.	gene_id GRMZM2G153928; transcript_id TCONS_00006229; exon_number 4; oId CUFF.10659.2; tss_id TSS12993;
1	Cufflinks	exon	248508463	248508554	.	-	.	gene_id GRMZM2G153928; transcript_id TCONS_00006229; exon_number 5; oId CUFF.10659.2; tss_id TSS12993;
1	Cufflinks	exon	248508748	248508811	.	-	.	gene_id GRMZM2G153928; transcript_id TCONS_00006229; exon_number 6; oId CUFF.10659.2; tss_id TSS12993;
1	Cufflinks	exon	248508921	248508999	.	-	.	gene_id GRMZM2G153928; transcript_id TCONS_00006229; exon_number 7; oId CUFF.10659.2; tss_id TSS12993;

1	Cufflinks	exon	248509074	248509681	.	-	.	gene_id GRMZM2G153928; transcript_id TCONS_00006229; exon_number 8; oId CUFF.10659.2; tss_id TSS12993;
1	Cufflinks	exon	251503953	251504683	.	-	.	gene_id XLOC_005429; transcript_id TCONS_00006263; exon_number 1; oId CUFF.10753.1; tss_id TSS13069;
1	Cufflinks	exon	256142498	256143727	.	-	.	gene_id XLOC_005479; transcript_id TCONS_00006322; exon_number 1; oId CUFF.11003.1; tss_id TSS13187;
1	Cufflinks	exon	263064702	263065147	.	-	.	gene_id GRMZM5G858417; transcript_id TCONS_00006422; exon_number 1; oId CUFF.11423.1; tss_id TSS13412;
1	Cufflinks	exon	263065232	263065392	.	-	.	gene_id GRMZM5G858417; transcript_id TCONS_00006422; exon_number 2; oId CUFF.11423.1; tss_id TSS13412;
1	Cufflinks	exon	263065515	263065592	.	-	.	gene_id GRMZM5G858417; transcript_id TCONS_00006422; exon_number 3; oId CUFF.11423.1; tss_id TSS13412;
1	Cufflinks	exon	263065743	263065834	.	-	.	gene_id GRMZM5G858417; transcript_id TCONS_00006422; exon_number 4; oId CUFF.11423.1; tss_id TSS13412;
1	Cufflinks	exon	263065941	263065999	.	-	.	gene_id GRMZM5G858417; transcript_id TCONS_00006422; exon_number 5; oId CUFF.11423.1; tss_id TSS13412;
1	Cufflinks	exon	263066313	263066399	.	-	.	gene_id GRMZM5G858417; transcript_id TCONS_00006422; exon_number 6; oId CUFF.11423.1; tss_id TSS13412;
1	Cufflinks	exon	263066648	263066744	.	-	.	gene_id GRMZM5G858417; transcript_id TCONS_00006422; exon_number 7; oId CUFF.11423.1; tss_id TSS13412;
1	Cufflinks	exon	263066834	263067018	.	-	.	gene_id GRMZM5G858417; transcript_id TCONS_00006422; exon_number 8; oId CUFF.11423.1; tss_id TSS13412;
1	Cufflinks	exon	263067104	263067163	.	-	.	gene_id GRMZM5G858417; transcript_id TCONS_00006422; exon_number 9; oId CUFF.11423.1; tss_id TSS13412;
1	Cufflinks	exon	263067258	263067333	.	-	.	gene_id GRMZM5G858417; transcript_id TCONS_00006422; exon_number 10; oId CUFF.11423.1; tss_id TSS13412;
1	Cufflinks	exon	263067452	263067552	.	-	.	gene_id GRMZM5G858417; transcript_id TCONS_00006422; exon_number 11; oId CUFF.11423.1; tss_id TSS13412;
1	Cufflinks	exon	263067666	263067842	.	-	.	gene_id GRMZM5G858417; transcript_id TCONS_00006422; exon_number 12; oId CUFF.11423.1; tss_id TSS13412;
1	Cufflinks	exon	263067943	263068040	.	-	.	gene_id GRMZM5G858417; transcript_id TCONS_00006422; exon_number 13; oId CUFF.11423.1; tss_id TSS13412;
1	Cufflinks	exon	263068188	263068643	.	-	.	gene_id GRMZM5G858417; transcript_id TCONS_00006422; exon_number 14; oId CUFF.11423.1; tss_id TSS13412;
1	Cufflinks	exon	264593352	264595682	.	-	.	gene_id XLOC_005589; transcript_id TCONS_00006442; exon_number 1; oId CUFF.11485.1; tss_id TSS13446;
1	Cufflinks	exon	275290498	275291000	.	-	.	gene_id GRMZM2G169671; transcript_id TCONS_00006604; exon_number 1; oId CUFF.12099.1; tss_id TSS13795;
1	Cufflinks	exon	275291089	275291209	.	-	.	gene_id GRMZM2G169671; transcript_id TCONS_00006604; exon_number 2; oId CUFF.12099.1; tss_id TSS13795;
1	Cufflinks	exon	275291307	275291547	.	-	.	gene_id GRMZM2G169671; transcript_id TCONS_00006604; exon_number 3; oId CUFF.12099.1; tss_id TSS13795;
1	Cufflinks	exon	275293889	275294240	.	-	.	gene_id GRMZM2G169671; transcript_id TCONS_00006604; exon_number 4; oId CUFF.12099.1; tss_id TSS13795;
1	Cufflinks	exon	277056474	277057254	.	-	.	gene_id XLOC_005776; transcript_id TCONS_00006648; exon_number 1; oId CUFF.12242.1; tss_id TSS13876;
1	Cufflinks	exon	277059425	277060124	.	-	.	gene_id GRMZM2G057935; transcript_id TCONS_00006649; exon_number 1; oId CUFF.12249.1; tss_id TSS13877;
1	Cufflinks	exon	277060583	277060876	.	-	.	gene_id GRMZM2G057935; transcript_id TCONS_00006649; exon_number 2; oId CUFF.12249.1; tss_id TSS13877;
1	Cufflinks	exon	277061172	277061988	.	-	.	gene_id GRMZM2G057935; transcript_id TCONS_00006649; exon_number 3; oId CUFF.12249.1; tss_id TSS13877;
1	Cufflinks	exon	277062089	277064749	.	-	.	gene_id GRMZM2G057935; transcript_id TCONS_00006649; exon_number 4; oId CUFF.12249.1; tss_id TSS13877;
1	Cufflinks	exon	277446604	277447376	.	-	.	gene_id GRMZM5G826714; transcript_id TCONS_00006660; exon_number 1; oId CUFF.12287.1; tss_id TSS13893;
1	Cufflinks	exon	277447459	277447739	.	-	.	gene_id GRMZM5G826714; transcript_id TCONS_00006660; exon_number 2; oId CUFF.12287.1; tss_id TSS13893;
1	Cufflinks	exon	277447833	277447986	.	-	.	gene_id GRMZM5G826714; transcript_id TCONS_00006660; exon_number 3; oId CUFF.12287.1; tss_id TSS13893;
1	Cufflinks	exon	277448389	277448845	.	-	.	gene_id GRMZM5G826714; transcript_id TCONS_00006660; exon_number 4; oId CUFF.12287.1; tss_id TSS13893;
1	Cufflinks	exon	277449080	277449159	.	-	.	gene_id GRMZM5G826714; transcript_id TCONS_00006660; exon_number 5; oId CUFF.12287.1; tss_id TSS13893;
1	Cufflinks	exon	277449733	277450109	.	-	.	gene_id GRMZM5G826714; transcript_id TCONS_00006660; exon_number 6; oId CUFF.12287.1; tss_id TSS13893;
1	Cufflinks	exon	278197373	278199188	.	-	.	gene_id XLOC_005789; transcript_id TCONS_00006664; exon_number 1; oId CUFF.12324.1; tss_id TSS13917;
1	Cufflinks	exon	279251625	279254873	.	-	.	gene_id GRMZM2G091578; transcript_id TCONS_00006681; exon_number 1; oId CUFF.12387.1; tss_id TSS13952;
1	Cufflinks	exon	279633156	279634477	.	-	.	gene_id XLOC_005809; transcript_id TCONS_00006686; exon_number 1; oId CUFF.12439.2; tss_id TSS13960;

1	Cufflinks	exon	285280649	285281991	.	-	.	gene_id GRMZM2G040145; transcript_id TCONS_00006790; exon_number 1; oId CUFF.12671.1; tss_id TSS14101;
1	Cufflinks	exon	285410446	285411691	.	-	.	gene_id GRMZM2G308687; transcript_id TCONS_00006793; exon_number 1; oId CUFF.12676.1; tss_id TSS14106;
1	Cufflinks	exon	285855548	285856928	.	-	.	gene_id XLOC_005910; transcript_id TCONS_00006797; exon_number 1; oId CUFF.12699.1; tss_id TSS14111;
1	Cufflinks	exon	287361438	287361864	.	-	.	gene_id GRMZM2G178415; transcript_id TCONS_00006824; exon_number 1; oId CUFF.12839.2; tss_id TSS14149;
1	Cufflinks	exon	287361967	287362053	.	-	.	gene_id GRMZM2G178415; transcript_id TCONS_00006824; exon_number 2; oId CUFF.12839.2; tss_id TSS14149;
1	Cufflinks	exon	287362132	287362207	.	-	.	gene_id GRMZM2G178415; transcript_id TCONS_00006824; exon_number 3; oId CUFF.12839.2; tss_id TSS14149;
1	Cufflinks	exon	287362346	287362596	.	-	.	gene_id GRMZM2G178415; transcript_id TCONS_00006824; exon_number 4; oId CUFF.12839.2; tss_id TSS14149;
1	Cufflinks	exon	287362826	287362942	.	-	.	gene_id GRMZM2G178415; transcript_id TCONS_00006824; exon_number 5; oId CUFF.12839.2; tss_id TSS14149;
1	Cufflinks	exon	287363208	287363282	.	-	.	gene_id GRMZM2G178415; transcript_id TCONS_00006824; exon_number 6; oId CUFF.12839.2; tss_id TSS14149;
1	Cufflinks	exon	287363391	287363612	.	-	.	gene_id GRMZM2G178415; transcript_id TCONS_00006824; exon_number 7; oId CUFF.12839.2; tss_id TSS14149;
1	Cufflinks	exon	287364295	287364408	.	-	.	gene_id GRMZM2G178415; transcript_id TCONS_00006824; exon_number 8; oId CUFF.12839.2; tss_id TSS14149;
1	Cufflinks	exon	287364651	287364803	.	-	.	gene_id GRMZM2G178415; transcript_id TCONS_00006824; exon_number 9; oId CUFF.12839.2; tss_id TSS14149;
1	Cufflinks	exon	287364877	287366888	.	-	.	gene_id GRMZM2G178415; transcript_id TCONS_00006824; exon_number 10; oId CUFF.12839.2; tss_id TSS14149;
1	Cufflinks	exon	288992430	288992879	.	-	.	gene_id XLOC_005960; transcript_id TCONS_00006851; exon_number 1; oId CUFF.12892.1; tss_id TSS14203;
1	Cufflinks	exon	290273163	290275009	.	-	.	gene_id XLOC_005986; transcript_id TCONS_00006881; exon_number 1; oId CUFF.12978.1; tss_id TSS14257;
1	Cufflinks	exon	290344248	290345563	.	-	.	gene_id GRMZM2G432560; transcript_id TCONS_00006882; exon_number 1; oId CUFF.13061.1; tss_id TSS14261;
1	Cufflinks	exon	290345676	290345753	.	-	.	gene_id GRMZM2G432560; transcript_id TCONS_00006882; exon_number 2; oId CUFF.13061.1; tss_id TSS14261;
1	Cufflinks	exon	290345861	290346241	.	-	.	gene_id GRMZM2G432560; transcript_id TCONS_00006882; exon_number 3; oId CUFF.13061.1; tss_id TSS14261;
1	Cufflinks	exon	295097968	295098274	.	-	.	gene_id XLOC_006075; transcript_id TCONS_00006978; exon_number 1; oId CUFF.13361.1; tss_id TSS14458;
1	Cufflinks	exon	296067223	296069043	.	-	.	gene_id GRMZM2G366681; transcript_id TCONS_00007003; exon_number 1; oId CUFF.13433.1; tss_id TSS14502;
1	Cufflinks	exon	298814031	298814417	.	-	.	gene_id XLOC_006147; transcript_id TCONS_00007066; exon_number 1; oId CUFF.13657.1; tss_id TSS14604;
1	Cufflinks	exon	299276941	299277767	.	-	.	gene_id GRMZM2G417843; transcript_id TCONS_00007078; exon_number 1; oId CUFF.13703.1; tss_id TSS14635;
1	Cufflinks	exon	299277883	299277986	.	-	.	gene_id GRMZM2G417843; transcript_id TCONS_00007078; exon_number 2; oId CUFF.13703.1; tss_id TSS14635;
1	Cufflinks	exon	299278104	299278203	.	-	.	gene_id GRMZM2G417843; transcript_id TCONS_00007078; exon_number 3; oId CUFF.13703.1; tss_id TSS14635;
1	Cufflinks	exon	299278298	299278395	.	-	.	gene_id GRMZM2G417843; transcript_id TCONS_00007078; exon_number 4; oId CUFF.13703.1; tss_id TSS14635;
1	Cufflinks	exon	299279197	299279249	.	-	.	gene_id GRMZM2G417843; transcript_id TCONS_00007078; exon_number 5; oId CUFF.13703.1; tss_id TSS14635;
1	Cufflinks	exon	299279344	299279403	.	-	.	gene_id GRMZM2G417843; transcript_id TCONS_00007078; exon_number 6; oId CUFF.13703.1; tss_id TSS14635;
1	Cufflinks	exon	299280102	299280173	.	-	.	gene_id GRMZM2G417843; transcript_id TCONS_00007078; exon_number 7; oId CUFF.13703.1; tss_id TSS14635;
1	Cufflinks	exon	299280587	299280658	.	-	.	gene_id GRMZM2G417843; transcript_id TCONS_00007078; exon_number 8; oId CUFF.13703.1; tss_id TSS14635;
1	Cufflinks	exon	299280736	299280828	.	-	.	gene_id GRMZM2G417843; transcript_id TCONS_00007078; exon_number 9; oId CUFF.13703.1; tss_id TSS14635;
1	Cufflinks	exon	299281382	299281449	.	-	.	gene_id GRMZM2G417843; transcript_id TCONS_00007078; exon_number 10; oId CUFF.13703.1; tss_id TSS14635;
1	Cufflinks	exon	299281567	299281868	.	-	.	gene_id GRMZM2G417843; transcript_id TCONS_00007078; exon_number 11; oId CUFF.13703.1; tss_id TSS14635;
1	Cufflinks	exon	300823694	300824035	.	-	.	gene_id GRMZM2G415077; transcript_id TCONS_00007113; exon_number 1; oId CUFF.13857.1; tss_id TSS14741;
1	Cufflinks	exon	300951291	300951966	.	-	.	gene_id GRMZM2G131939; transcript_id TCONS_00007115; exon_number 1; oId CUFF.13894.1; tss_id TSS14746;
1	Cufflinks	exon	300952097	300952190	.	-	.	gene_id GRMZM2G131939; transcript_id TCONS_00007115; exon_number 2; oId CUFF.13894.1; tss_id TSS14746;
1	Cufflinks	exon	300958386	300958459	.	-	.	gene_id GRMZM2G131939; transcript_id TCONS_00007115; exon_number 3; oId CUFF.13894.1; tss_id TSS14746;

1	Cufflinks	exon	300960085	300960133	.	-	.	gene_id GRMZM2G131939; transcript_id TCONS_00007115; exon_number 4; oId CUFF.13894.1; tss_id TSS14746;
1	Cufflinks	exon	300962813	300962980	.	-	.	gene_id GRMZM2G131939; transcript_id TCONS_00007115; exon_number 5; oId CUFF.13894.1; tss_id TSS14746;
1	Cufflinks	exon	300963060	300963137	.	-	.	gene_id GRMZM2G131939; transcript_id TCONS_00007115; exon_number 6; oId CUFF.13894.1; tss_id TSS14746;
1	Cufflinks	exon	300963642	300963853	.	-	.	gene_id GRMZM2G131939; transcript_id TCONS_00007115; exon_number 7; oId CUFF.13894.1; tss_id TSS14746;
10	Cufflinks	exon	1948867	1949015	.	+	.	gene_id GRMZM2G043119; transcript_id TCONS_00007137; exon_number 1; oId CUFF.14045.2; tss_id TSS14821;
10	Cufflinks	exon	1949102	1949430	.	+	.	gene_id GRMZM2G043119; transcript_id TCONS_00007137; exon_number 2; oId CUFF.14045.2; tss_id TSS14821;
10	Cufflinks	exon	1950010	1951755	.	+	.	gene_id GRMZM2G043119; transcript_id TCONS_00007137; exon_number 3; oId CUFF.14045.2; tss_id TSS14821;
10	Cufflinks	exon	4531087	4531735	.	+	.	gene_id XLOC_006267; transcript_id TCONS_00007193; exon_number 1; oId CUFF.14246.1; tss_id TSS14933;
10	Cufflinks	exon	5645825	5646958	.	+	.	gene_id GRMZM2G464328; transcript_id TCONS_00007215; exon_number 1; oId CUFF.14402.1; tss_id TSS14991;
10	Cufflinks	exon	12930630	12930765	.	+	.	gene_id XLOC_006355; transcript_id TCONS_00007288; exon_number 1; oId CUFF.14688.1; tss_id TSS15136;
10	Cufflinks	exon	12932871	12933118	.	+	.	gene_id XLOC_006355; transcript_id TCONS_00007288; exon_number 2; oId CUFF.14688.1; tss_id TSS15136;
10	Cufflinks	exon	25565234	25565562	.	+	.	gene_id GRMZM2G054393; transcript_id TCONS_00007371; exon_number 1; oId CUFF.15120.2; tss_id TSS15334;
10	Cufflinks	exon	25567144	25567232	.	+	.	gene_id GRMZM2G054393; transcript_id TCONS_00007371; exon_number 2; oId CUFF.15120.2; tss_id TSS15334;
10	Cufflinks	exon	25568840	25568926	.	+	.	gene_id GRMZM2G054393; transcript_id TCONS_00007371; exon_number 3; oId CUFF.15120.2; tss_id TSS15334;
10	Cufflinks	exon	25570015	25570061	.	+	.	gene_id GRMZM2G054393; transcript_id TCONS_00007371; exon_number 4; oId CUFF.15120.2; tss_id TSS15334;
10	Cufflinks	exon	25570145	25570202	.	+	.	gene_id GRMZM2G054393; transcript_id TCONS_00007371; exon_number 5; oId CUFF.15120.2; tss_id TSS15334;
10	Cufflinks	exon	25570795	25570905	.	+	.	gene_id GRMZM2G054393; transcript_id TCONS_00007371; exon_number 6; oId CUFF.15120.2; tss_id TSS15334;
10	Cufflinks	exon	25571071	25571187	.	+	.	gene_id GRMZM2G054393; transcript_id TCONS_00007371; exon_number 7; oId CUFF.15120.2; tss_id TSS15334;
10	Cufflinks	exon	25572218	25572277	.	+	.	gene_id GRMZM2G054393; transcript_id TCONS_00007371; exon_number 8; oId CUFF.15120.2; tss_id TSS15334;
10	Cufflinks	exon	25573045	25573265	.	+	.	gene_id GRMZM2G054393; transcript_id TCONS_00007371; exon_number 9; oId CUFF.15120.2; tss_id TSS15334;
10	Cufflinks	exon	25573352	25573505	.	+	.	gene_id GRMZM2G054393; transcript_id TCONS_00007371; exon_number 10; oId CUFF.15120.2; tss_id TSS15334;
10	Cufflinks	exon	25574114	25574189	.	+	.	gene_id GRMZM2G054393; transcript_id TCONS_00007371; exon_number 11; oId CUFF.15120.2; tss_id TSS15334;
10	Cufflinks	exon	25574264	25574385	.	+	.	gene_id GRMZM2G054393; transcript_id TCONS_00007371; exon_number 12; oId CUFF.15120.2; tss_id TSS15334;
10	Cufflinks	exon	25574500	25575047	.	+	.	gene_id GRMZM2G054393; transcript_id TCONS_00007371; exon_number 13; oId CUFF.15120.2; tss_id TSS15334;
10	Cufflinks	exon	35929292	35930296	.	+	.	gene_id GRMZM2G565911; transcript_id TCONS_00007415; exon_number 1; oId CUFF.15270.1; tss_id TSS15420;
10	Cufflinks	exon	68937698	68938766	.	+	.	gene_id XLOC_006623; transcript_id TCONS_00007588; exon_number 1; oId CUFF.16000.1; tss_id TSS15824;
10	Cufflinks	exon	75157846	75158315	.	+	.	gene_id XLOC_006656; transcript_id TCONS_00007624; exon_number 1; oId CUFF.16184.1; tss_id TSS15897;
10	Cufflinks	exon	86066482	86066878	.	+	.	gene_id XLOC_006759; transcript_id TCONS_00007741; exon_number 1; oId CUFF.16559.1; tss_id TSS16109;
10	Cufflinks	exon	86998288	86998965	.	+	.	gene_id GRMZM2G028104; transcript_id TCONS_00007746; exon_number 1; oId CUFF.16592.2; tss_id TSS16116;
10	Cufflinks	exon	86999374	87000589	.	+	.	gene_id GRMZM2G028104; transcript_id TCONS_00007746; exon_number 2; oId CUFF.16592.2; tss_id TSS16116;
10	Cufflinks	exon	87284105	87284585	.	+	.	gene_id XLOC_006771; transcript_id TCONS_00007755; exon_number 1; oId CUFF.16610.1; tss_id TSS16127;
10	Cufflinks	exon	98012816	98013047	.	+	.	gene_id GRMZM2G075315; transcript_id TCONS_00007850; exon_number 1; oId CUFF.17063.1; tss_id TSS16359;
10	Cufflinks	exon	98013170	98014124	.	+	.	gene_id GRMZM2G075315; transcript_id TCONS_00007850; exon_number 2; oId CUFF.17063.1; tss_id TSS16359;
10	Cufflinks	exon	99220564	99221170	.	+	.	gene_id XLOC_006863; transcript_id TCONS_00007860; exon_number 1; oId CUFF.17107.1; tss_id TSS16397;
10	Cufflinks	exon	99624013	99624641	.	+	.	gene_id GRMZM2G119127; transcript_id TCONS_00007864; exon_number 1; oId CUFF.17119.1; tss_id TSS16403;
10	Cufflinks	exon	111444985	111445522	.	+	.	gene_id GRMZM2G451716; transcript_id TCONS_00007976; exon_number 1; oId CUFF.17728.1; tss_id TSS16699;

10	Cufflinks	exon	111446138	111446264	.	+	.	gene_id GRMZM2G451716; transcript_id TCONS_00007976; exon_number 2; oId CUFF.17728.1; tss_id TSS16699;
10	Cufflinks	exon	111446480	111446525	.	+	.	gene_id GRMZM2G451716; transcript_id TCONS_00007976; exon_number 3; oId CUFF.17728.1; tss_id TSS16699;
10	Cufflinks	exon	111447020	111447325	.	+	.	gene_id GRMZM2G451716; transcript_id TCONS_00007976; exon_number 4; oId CUFF.17728.1; tss_id TSS16699;
10	Cufflinks	exon	111447673	111448065	.	+	.	gene_id GRMZM2G451716; transcript_id TCONS_00007976; exon_number 5; oId CUFF.17728.1; tss_id TSS16699;
10	Cufflinks	exon	111448149	111448307	.	+	.	gene_id GRMZM2G451716; transcript_id TCONS_00007976; exon_number 6; oId CUFF.17728.1; tss_id TSS16699;
10	Cufflinks	exon	111448528	111448641	.	+	.	gene_id GRMZM2G451716; transcript_id TCONS_00007976; exon_number 7; oId CUFF.17728.1; tss_id TSS16699;
10	Cufflinks	exon	111448725	111448873	.	+	.	gene_id GRMZM2G451716; transcript_id TCONS_00007976; exon_number 8; oId CUFF.17728.1; tss_id TSS16699;
10	Cufflinks	exon	111449026	111449114	.	+	.	gene_id GRMZM2G451716; transcript_id TCONS_00007976; exon_number 9; oId CUFF.17728.1; tss_id TSS16699;
10	Cufflinks	exon	111449188	111449646	.	+	.	gene_id GRMZM2G451716; transcript_id TCONS_00007976; exon_number 10; oId CUFF.17728.1; tss_id TSS16699;
10	Cufflinks	exon	111449883	111450537	.	+	.	gene_id GRMZM2G451716; transcript_id TCONS_00007976; exon_number 11; oId CUFF.17728.1; tss_id TSS16699;
10	Cufflinks	exon	111450637	111450721	.	+	.	gene_id GRMZM2G451716; transcript_id TCONS_00007976; exon_number 12; oId CUFF.17728.1; tss_id TSS16699;
10	Cufflinks	exon	111450830	111450898	.	+	.	gene_id GRMZM2G451716; transcript_id TCONS_00007976; exon_number 13; oId CUFF.17728.1; tss_id TSS16699;
10	Cufflinks	exon	111451541	111451720	.	+	.	gene_id GRMZM2G451716; transcript_id TCONS_00007976; exon_number 14; oId CUFF.17728.1; tss_id TSS16699;
10	Cufflinks	exon	111452402	111453558	.	+	.	gene_id GRMZM2G451716; transcript_id TCONS_00007976; exon_number 15; oId CUFF.17728.1; tss_id TSS16699;
10	Cufflinks	exon	117641432	117641727	.	+	.	gene_id GRMZM2G136508; transcript_id TCONS_00008034; exon_number 1; oId CUFF.17941.1; tss_id TSS16834;
10	Cufflinks	exon	117642087	117642269	.	+	.	gene_id GRMZM2G136508; transcript_id TCONS_00008034; exon_number 2; oId CUFF.17941.1; tss_id TSS16834;
10	Cufflinks	exon	117642753	117642872	.	+	.	gene_id GRMZM2G136508; transcript_id TCONS_00008034; exon_number 3; oId CUFF.17941.1; tss_id TSS16834;
10	Cufflinks	exon	117643373	117643564	.	+	.	gene_id GRMZM2G136508; transcript_id TCONS_00008034; exon_number 4; oId CUFF.17941.1; tss_id TSS16834;
10	Cufflinks	exon	117643685	117643990	.	+	.	gene_id GRMZM2G136508; transcript_id TCONS_00008034; exon_number 5; oId CUFF.17941.1; tss_id TSS16834;
10	Cufflinks	exon	117644077	117645132	.	+	.	gene_id GRMZM2G136508; transcript_id TCONS_00008034; exon_number 6; oId CUFF.17941.1; tss_id TSS16834;
10	Cufflinks	exon	128580520	128581374	.	+	.	gene_id XLOC_007148; transcript_id TCONS_00008178; exon_number 1; oId CUFF.18519.1; tss_id TSS17120;
10	Cufflinks	exon	132446577	132447208	.	+	.	gene_id XLOC_007203; transcript_id TCONS_00008246; exon_number 1; oId CUFF.18769.1; tss_id TSS17259;
10	Cufflinks	exon	134830087	134830456	.	+	.	gene_id XLOC_007252; transcript_id TCONS_00008305; exon_number 1; oId CUFF.18919.1; tss_id TSS17352;
10	Cufflinks	exon	136041276	136043126	.	+	.	gene_id GRMZM2G098676; transcript_id TCONS_00008328; exon_number 1; oId CUFF.18995.1; tss_id TSS17394;
10	Cufflinks	exon	136043650	136044237	.	+	.	gene_id GRMZM2G098676; transcript_id TCONS_00008328; exon_number 2; oId CUFF.18995.1; tss_id TSS17394;
10	Cufflinks	exon	137853902	137854077	.	+	.	gene_id GRMZM2G142832; transcript_id TCONS_00008359; exon_number 1; oId CUFF.19133.7; tss_id TSS17450;
10	Cufflinks	exon	137854297	137854742	.	+	.	gene_id GRMZM2G142832; transcript_id TCONS_00008359; exon_number 2; oId CUFF.19133.7; tss_id TSS17450;
10	Cufflinks	exon	137855164	137855299	.	+	.	gene_id GRMZM2G142832; transcript_id TCONS_00008359; exon_number 3; oId CUFF.19133.7; tss_id TSS17450;
10	Cufflinks	exon	137855605	137855747	.	+	.	gene_id GRMZM2G142832; transcript_id TCONS_00008359; exon_number 4; oId CUFF.19133.7; tss_id TSS17450;
10	Cufflinks	exon	137855856	137855979	.	+	.	gene_id GRMZM2G142832; transcript_id TCONS_00008359; exon_number 5; oId CUFF.19133.7; tss_id TSS17450;
10	Cufflinks	exon	137856096	137856863	.	+	.	gene_id GRMZM2G142832; transcript_id TCONS_00008359; exon_number 6; oId CUFF.19133.7; tss_id TSS17450;
10	Cufflinks	exon	140355466	140355933	.	+	.	gene_id GRMZM2G109753; transcript_id TCONS_00008398; exon_number 1; oId CUFF.19422.1; tss_id TSS17547;
10	Cufflinks	exon	140359809	140359895	.	+	.	gene_id GRMZM2G109753; transcript_id TCONS_00008398; exon_number 2; oId CUFF.19422.1; tss_id TSS17547;
10	Cufflinks	exon	140359998	140360039	.	+	.	gene_id GRMZM2G109753; transcript_id TCONS_00008398; exon_number 3; oId CUFF.19422.1; tss_id TSS17547;
10	Cufflinks	exon	140360152	140360196	.	+	.	gene_id GRMZM2G109753; transcript_id TCONS_00008398; exon_number 4; oId CUFF.19422.1; tss_id TSS17547;
10	Cufflinks	exon	140360531	140360596	.	+	.	gene_id GRMZM2G109753; transcript_id TCONS_00008398; exon_number 5; oId CUFF.19422.1; tss_id TSS17547;

10	Cufflinks	exon	140360665	140360721	.	+	.	gene_id GRMZM2G109753; transcript_id TCONS_00008398; exon_number 6; oId CUFF.19422.1; tss_id TSS17547;
10	Cufflinks	exon	140360829	140360890	.	+	.	gene_id GRMZM2G109753; transcript_id TCONS_00008398; exon_number 7; oId CUFF.19422.1; tss_id TSS17547;
10	Cufflinks	exon	140422854	140422974	.	+	.	gene_id GRMZM2G109753; transcript_id TCONS_00008398; exon_number 8; oId CUFF.19422.1; tss_id TSS17547;
10	Cufflinks	exon	140426108	140426185	.	+	.	gene_id GRMZM2G109753; transcript_id TCONS_00008398; exon_number 9; oId CUFF.19422.1; tss_id TSS17547;
10	Cufflinks	exon	140426276	140426382	.	+	.	gene_id GRMZM2G109753; transcript_id TCONS_00008398; exon_number 10; oId CUFF.19422.1; tss_id TSS17547;
10	Cufflinks	exon	140426499	140426941	.	+	.	gene_id GRMZM2G109753; transcript_id TCONS_00008398; exon_number 11; oId CUFF.19422.1; tss_id TSS17547;
10	Cufflinks	exon	141312471	141313203	.	+	.	gene_id XLOC_007349; transcript_id TCONS_00008417; exon_number 1; oId CUFF.19315.1; tss_id TSS17583;
10	Cufflinks	exon	144346275	144346895	.	+	.	gene_id XLOC_007429; transcript_id TCONS_00008510; exon_number 1; oId CUFF.19614.1; tss_id TSS17746;
10	Cufflinks	exon	145054951	145056959	.	+	.	gene_id GRMZM2G153017; transcript_id TCONS_00008533; exon_number 1; oId CUFF.19672.3; tss_id TSS17775;
10	Cufflinks	exon	145330728	145330914	.	+	.	gene_id GRMZM2G143480; transcript_id TCONS_00008542; exon_number 1; oId CUFF.19712.2; tss_id TSS17788;
10	Cufflinks	exon	145331753	145331936	.	+	.	gene_id GRMZM2G143480; transcript_id TCONS_00008542; exon_number 2; oId CUFF.19712.2; tss_id TSS17788;
10	Cufflinks	exon	145332021	145332142	.	+	.	gene_id GRMZM2G143480; transcript_id TCONS_00008542; exon_number 3; oId CUFF.19712.2; tss_id TSS17788;
10	Cufflinks	exon	145332343	145332438	.	+	.	gene_id GRMZM2G143480; transcript_id TCONS_00008542; exon_number 4; oId CUFF.19712.2; tss_id TSS17788;
10	Cufflinks	exon	145333210	145333305	.	+	.	gene_id GRMZM2G143480; transcript_id TCONS_00008542; exon_number 5; oId CUFF.19712.2; tss_id TSS17788;
10	Cufflinks	exon	145334441	145334519	.	+	.	gene_id GRMZM2G143480; transcript_id TCONS_00008542; exon_number 6; oId CUFF.19712.2; tss_id TSS17788;
10	Cufflinks	exon	145334987	145335033	.	+	.	gene_id GRMZM2G143480; transcript_id TCONS_00008542; exon_number 7; oId CUFF.19712.2; tss_id TSS17788;
10	Cufflinks	exon	145335245	145335304	.	+	.	gene_id GRMZM2G143480; transcript_id TCONS_00008542; exon_number 8; oId CUFF.19712.2; tss_id TSS17788;
10	Cufflinks	exon	145335557	145335704	.	+	.	gene_id GRMZM2G143480; transcript_id TCONS_00008542; exon_number 9; oId CUFF.19712.2; tss_id TSS17788;
10	Cufflinks	exon	145338985	145339330	.	+	.	gene_id GRMZM2G143480; transcript_id TCONS_00008542; exon_number 10; oId CUFF.19712.2; tss_id TSS17788;
10	Cufflinks	exon	145369441	145369658	.	+	.	gene_id GRMZM2G143402; transcript_id TCONS_00008544; exon_number 1; oId CUFF.19690.1; tss_id TSS17792;
10	Cufflinks	exon	145371401	145371503	.	+	.	gene_id GRMZM2G143402; transcript_id TCONS_00008544; exon_number 2; oId CUFF.19690.1; tss_id TSS17792;
10	Cufflinks	exon	145371595	145371681	.	+	.	gene_id GRMZM2G143402; transcript_id TCONS_00008544; exon_number 3; oId CUFF.19690.1; tss_id TSS17792;
10	Cufflinks	exon	145371751	145371820	.	+	.	gene_id GRMZM2G143402; transcript_id TCONS_00008544; exon_number 4; oId CUFF.19690.1; tss_id TSS17792;
10	Cufflinks	exon	145371923	145371980	.	+	.	gene_id GRMZM2G143402; transcript_id TCONS_00008544; exon_number 5; oId CUFF.19690.1; tss_id TSS17792;
10	Cufflinks	exon	145372737	145373354	.	+	.	gene_id GRMZM2G143402; transcript_id TCONS_00008544; exon_number 6; oId CUFF.19690.1; tss_id TSS17792;
10	Cufflinks	exon	147045247	147045370	.	+	.	gene_id XLOC_007494; transcript_id TCONS_00008579; exon_number 1; oId CUFF.19912.1; tss_id TSS17900;
10	Cufflinks	exon	147045451	147045660	.	+	.	gene_id XLOC_007494; transcript_id TCONS_00008579; exon_number 2; oId CUFF.19912.1; tss_id TSS17900;
10	Cufflinks	exon	148075099	148076695	.	+	.	gene_id GRMZM2G074718; transcript_id TCONS_00008613; exon_number 1; oId CUFF.20005.1; tss_id TSS17952;
10	Cufflinks	exon	148313887	148314859	.	+	.	gene_id XLOC_007535; transcript_id TCONS_00008623; exon_number 1; oId CUFF.20040.1; tss_id TSS17971;
10	Cufflinks	exon	148737011	148737290	.	+	.	gene_id GRMZM2G110908; transcript_id TCONS_00008635; exon_number 1; oId CUFF.20071.1; tss_id TSS17991;
10	Cufflinks	exon	148737359	148739143	.	+	.	gene_id GRMZM2G110908; transcript_id TCONS_00008635; exon_number 2; oId CUFF.20071.1; tss_id TSS17991;
10	Cufflinks	exon	148821382	148821923	.	+	.	gene_id GRMZM2G011590; transcript_id TCONS_00008638; exon_number 1; oId CUFF.20116.1; tss_id TSS17998;
10	Cufflinks	exon	148822010	148822227	.	+	.	gene_id GRMZM2G011590; transcript_id TCONS_00008638; exon_number 2; oId CUFF.20116.1; tss_id TSS17998;
10	Cufflinks	exon	148828096	148830411	.	+	.	gene_id GRMZM2G011590; transcript_id TCONS_00008638; exon_number 3; oId CUFF.20116.1; tss_id TSS17998;
10	Cufflinks	exon	1078071	1079418	.	-	.	gene_id GRMZM2G066165; transcript_id TCONS_00008648; exon_number 1; oId CUFF.13947.1; tss_id TSS18034;
10	Cufflinks	exon	1082298	1082453	.	-	.	gene_id GRMZM2G066165; transcript_id TCONS_00008648; exon_number 2; oId CUFF.13947.1; tss_id TSS18034;

10	Cufflinks	exon	1082567	1082938	.	-	.	gene_id GRMZM2G066165; transcript_id TCONS_00008648; exon_number 3; oId CUFF.13947.1; tss_id TSS18034;
10	Cufflinks	exon	1083019	1084308	.	-	.	gene_id GRMZM2G066165; transcript_id TCONS_00008648; exon_number 4; oId CUFF.13947.1; tss_id TSS18034;
10	Cufflinks	exon	2436258	2437872	.	-	.	gene_id XLOC_007601; transcript_id TCONS_00008696; exon_number 1; oId CUFF.14102.1; tss_id TSS18107;
10	Cufflinks	exon	2449057	2449866	.	-	.	gene_id XLOC_007603; transcript_id TCONS_00008698; exon_number 1; oId CUFF.14077.1; tss_id TSS18111;
10	Cufflinks	exon	3176413	3176925	.	-	.	gene_id XLOC_007620; transcript_id TCONS_00008715; exon_number 1; oId CUFF.14127.1; tss_id TSS18133;
10	Cufflinks	exon	4056048	4057136	.	-	.	gene_id XLOC_007649; transcript_id TCONS_00008745; exon_number 1; oId CUFF.14239.1; tss_id TSS18194;
10	Cufflinks	exon	12927390	12927996	.	-	.	gene_id GRMZM2G015869; transcript_id TCONS_00008885; exon_number 1; oId CUFF.14685.1; tss_id TSS18462;
10	Cufflinks	exon	12928120	12928169	.	-	.	gene_id GRMZM2G015869; transcript_id TCONS_00008885; exon_number 2; oId CUFF.14685.1; tss_id TSS18462;
10	Cufflinks	exon	12928255	12928372	.	-	.	gene_id GRMZM2G015869; transcript_id TCONS_00008885; exon_number 3; oId CUFF.14685.1; tss_id TSS18462;
10	Cufflinks	exon	12929005	12929047	.	-	.	gene_id GRMZM2G015869; transcript_id TCONS_00008885; exon_number 4; oId CUFF.14685.1; tss_id TSS18462;
10	Cufflinks	exon	12929186	12929310	.	-	.	gene_id GRMZM2G015869; transcript_id TCONS_00008885; exon_number 5; oId CUFF.14685.1; tss_id TSS18462;
10	Cufflinks	exon	12929401	12929490	.	-	.	gene_id GRMZM2G015869; transcript_id TCONS_00008885; exon_number 6; oId CUFF.14685.1; tss_id TSS18462;
10	Cufflinks	exon	12930633	12930765	.	-	.	gene_id GRMZM2G015869; transcript_id TCONS_00008885; exon_number 7; oId CUFF.14685.1; tss_id TSS18462;
10	Cufflinks	exon	12932871	12933138	.	-	.	gene_id GRMZM2G015869; transcript_id TCONS_00008885; exon_number 8; oId CUFF.14685.1; tss_id TSS18462;
10	Cufflinks	exon	20327689	20328985	.	-	.	gene_id XLOC_007823; transcript_id TCONS_00008943; exon_number 1; oId CUFF.14909.1; tss_id TSS18588;
10	Cufflinks	exon	29230376	29232031	.	-	.	gene_id GRMZM2G120530; transcript_id TCONS_00009025; exon_number 1; oId CUFF.15198.1; tss_id TSS18745;
10	Cufflinks	exon	29233425	29233478	.	-	.	gene_id GRMZM2G120530; transcript_id TCONS_00009025; exon_number 2; oId CUFF.15198.1; tss_id TSS18745;
10	Cufflinks	exon	29233589	29233661	.	-	.	gene_id GRMZM2G120530; transcript_id TCONS_00009025; exon_number 3; oId CUFF.15198.1; tss_id TSS18745;
10	Cufflinks	exon	29233752	29233816	.	-	.	gene_id GRMZM2G120530; transcript_id TCONS_00009025; exon_number 4; oId CUFF.15198.1; tss_id TSS18745;
10	Cufflinks	exon	29235765	29236119	.	-	.	gene_id GRMZM2G120530; transcript_id TCONS_00009025; exon_number 5; oId CUFF.15198.1; tss_id TSS18745;
10	Cufflinks	exon	61960807	61961374	.	-	.	gene_id XLOC_008016; transcript_id TCONS_00009161; exon_number 1; oId CUFF.15824.1; tss_id TSS19063;
10	Cufflinks	exon	77287726	77288880	.	-	.	gene_id XLOC_008118; transcript_id TCONS_00009271; exon_number 1; oId CUFF.16245.1; tss_id TSS19285;
10	Cufflinks	exon	90258469	90259237	.	-	.	gene_id XLOC_008230; transcript_id TCONS_00009389; exon_number 1; oId CUFF.16730.1; tss_id TSS19540;
10	Cufflinks	exon	99619669	99620332	.	-	.	gene_id GRMZM2G119116; transcript_id TCONS_00009483; exon_number 1; oId CUFF.17118.1; tss_id TSS19737;
10	Cufflinks	exon	99620434	99620755	.	-	.	gene_id GRMZM2G119116; transcript_id TCONS_00009483; exon_number 2; oId CUFF.17118.1; tss_id TSS19737;
10	Cufflinks	exon	99624108	99624690	.	-	.	gene_id GRMZM2G119116; transcript_id TCONS_00009483; exon_number 3; oId CUFF.17118.1; tss_id TSS19737;
10	Cufflinks	exon	109915061	109915553	.	-	.	gene_id XLOC_008380; transcript_id TCONS_00009563; exon_number 1; oId CUFF.17645.1; tss_id TSS19996;
10	Cufflinks	exon	111445144	111445552	.	-	.	gene_id XLOC_008393; transcript_id TCONS_00009578; exon_number 1; oId CUFF.17729.1; tss_id TSS20029;
10	Cufflinks	exon	112222537	112223557	.	-	.	gene_id XLOC_008397; transcript_id TCONS_00009582; exon_number 1; oId CUFF.17725.1; tss_id TSS20042;
10	Cufflinks	exon	132120374	132121425	.	-	.	gene_id GRMZM2G046952; transcript_id TCONS_00009856; exon_number 1; oId CUFF.18731.1; tss_id TSS20607;
10	Cufflinks	exon	132121578	132122335	.	-	.	gene_id GRMZM2G046952; transcript_id TCONS_00009856; exon_number 2; oId CUFF.18731.1; tss_id TSS20607;
10	Cufflinks	exon	143178937	143179364	.	-	.	gene_id XLOC_008824; transcript_id TCONS_00010086; exon_number 1; oId CUFF.19496.1; tss_id TSS20978;
10	Cufflinks	exon	144340337	144341784	.	-	.	gene_id GRMZM2G085236; transcript_id TCONS_00010102; exon_number 1; oId CUFF.19613.1; tss_id TSS21010;
10	Cufflinks	exon	144341965	144342206	.	-	.	gene_id GRMZM2G085236; transcript_id TCONS_00010102; exon_number 2; oId CUFF.19613.1; tss_id TSS21010;
10	Cufflinks	exon	144342287	144343112	.	-	.	gene_id GRMZM2G085236; transcript_id TCONS_00010102; exon_number 3; oId CUFF.19613.1; tss_id TSS21010;
10	Cufflinks	exon	144343195	144343418	.	-	.	gene_id GRMZM2G085236; transcript_id TCONS_00010102; exon_number 4; oId CUFF.19613.1; tss_id TSS21010;

10	Cufflinks	exon	144343508	144343824	.	-	.	gene_id GRMZM2G085236; transcript_id TCONS_00010102; exon_number 5; oId CUFF.19613.1; tss_id TSS21010;
10	Cufflinks	exon	144343938	144344197	.	-	.	gene_id GRMZM2G085236; transcript_id TCONS_00010102; exon_number 6; oId CUFF.19613.1; tss_id TSS21010;
10	Cufflinks	exon	144346299	144346675	.	-	.	gene_id GRMZM2G085236; transcript_id TCONS_00010102; exon_number 7; oId CUFF.19613.1; tss_id TSS21010;
10	Cufflinks	exon	144347105	144347159	.	-	.	gene_id GRMZM2G085236; transcript_id TCONS_00010102; exon_number 8; oId CUFF.19613.1; tss_id TSS21010;
10	Cufflinks	exon	144347299	144347928	.	-	.	gene_id GRMZM2G085236; transcript_id TCONS_00010102; exon_number 9; oId CUFF.19613.1; tss_id TSS21010;
10	Cufflinks	exon	145055233	145056650	.	-	.	gene_id XLOC_008854; transcript_id TCONS_00010117; exon_number 1; oId CUFF.19676.1; tss_id TSS21032;
10	Cufflinks	exon	145327308	145327696	.	-	.	gene_id GRMZM2G143499; transcript_id TCONS_00010120; exon_number 1; oId CUFF.19711.1; tss_id TSS21037;
10	Cufflinks	exon	145327782	145327808	.	-	.	gene_id GRMZM2G143499; transcript_id TCONS_00010120; exon_number 2; oId CUFF.19711.1; tss_id TSS21037;
10	Cufflinks	exon	145327901	145327970	.	-	.	gene_id GRMZM2G143499; transcript_id TCONS_00010120; exon_number 3; oId CUFF.19711.1; tss_id TSS21037;
10	Cufflinks	exon	145328662	145328731	.	-	.	gene_id GRMZM2G143499; transcript_id TCONS_00010120; exon_number 4; oId CUFF.19711.1; tss_id TSS21037;
10	Cufflinks	exon	145328863	145328922	.	-	.	gene_id GRMZM2G143499; transcript_id TCONS_00010120; exon_number 5; oId CUFF.19711.1; tss_id TSS21037;
10	Cufflinks	exon	145329945	145330003	.	-	.	gene_id GRMZM2G143499; transcript_id TCONS_00010120; exon_number 6; oId CUFF.19711.1; tss_id TSS21037;
10	Cufflinks	exon	145330115	145330955	.	-	.	gene_id GRMZM2G143499; transcript_id TCONS_00010120; exon_number 7; oId CUFF.19711.1; tss_id TSS21037;
10	Cufflinks	exon	146745094	146746414	.	-	.	gene_id XLOC_008900; transcript_id TCONS_00010180; exon_number 1; oId CUFF.19824.1; tss_id TSS21137;
10	Cufflinks	exon	148074813	148078937	.	-	.	gene_id GRMZM2G074107; transcript_id TCONS_00010243; exon_number 1; oId CUFF.20010.1; tss_id TSS21248;
10	Cufflinks	exon	148079023	148079202	.	-	.	gene_id GRMZM2G074107; transcript_id TCONS_00010243; exon_number 2; oId CUFF.20010.1; tss_id TSS21248;
10	Cufflinks	exon	148079289	148079462	.	-	.	gene_id GRMZM2G074107; transcript_id TCONS_00010243; exon_number 3; oId CUFF.20010.1; tss_id TSS21248;
10	Cufflinks	exon	148079573	148079640	.	-	.	gene_id GRMZM2G074107; transcript_id TCONS_00010243; exon_number 4; oId CUFF.20010.1; tss_id TSS21248;
10	Cufflinks	exon	148080184	148081156	.	-	.	gene_id GRMZM2G074107; transcript_id TCONS_00010243; exon_number 5; oId CUFF.20010.1; tss_id TSS21248;
10	Cufflinks	exon	148283968	148286266	.	-	.	gene_id GRMZM2G406099; transcript_id TCONS_00010246; exon_number 1; oId CUFF.20027.1; tss_id TSS21253;
10	Cufflinks	exon	148737480	148739495	.	-	.	gene_id GRMZM2G110869; transcript_id TCONS_00010259; exon_number 1; oId CUFF.20072.1; tss_id TSS21280;
10	Cufflinks	exon	148740064	148741164	.	-	.	gene_id GRMZM2G110869; transcript_id TCONS_00010259; exon_number 2; oId CUFF.20072.1; tss_id TSS21280;
2	Cufflinks	exon	2549740	2550717	.	+	.	gene_id GRMZM2G010257; transcript_id TCONS_00010345; exon_number 1; oId CUFF.20414.1; tss_id TSS21434;
2	Cufflinks	exon	2551194	2551282	.	+	.	gene_id GRMZM2G010257; transcript_id TCONS_00010345; exon_number 2; oId CUFF.20414.1; tss_id TSS21434;
2	Cufflinks	exon	2551812	2552038	.	+	.	gene_id GRMZM2G010257; transcript_id TCONS_00010345; exon_number 3; oId CUFF.20414.1; tss_id TSS21434;
2	Cufflinks	exon	2552274	2552578	.	+	.	gene_id GRMZM2G010257; transcript_id TCONS_00010345; exon_number 4; oId CUFF.20414.1; tss_id TSS21434;
2	Cufflinks	exon	2787752	2787773	.	+	.	gene_id GRMZM2G102349; transcript_id TCONS_00010351; exon_number 1; oId CUFF.20393.1; tss_id TSS21445;
2	Cufflinks	exon	2787866	2788058	.	+	.	gene_id GRMZM2G102349; transcript_id TCONS_00010351; exon_number 2; oId CUFF.20393.1; tss_id TSS21445;
2	Cufflinks	exon	2788185	2788278	.	+	.	gene_id GRMZM2G102349; transcript_id TCONS_00010351; exon_number 3; oId CUFF.20393.1; tss_id TSS21445;
2	Cufflinks	exon	2788407	2788522	.	+	.	gene_id GRMZM2G102349; transcript_id TCONS_00010351; exon_number 4; oId CUFF.20393.1; tss_id TSS21445;
2	Cufflinks	exon	2788712	2788820	.	+	.	gene_id GRMZM2G102349; transcript_id TCONS_00010351; exon_number 5; oId CUFF.20393.1; tss_id TSS21445;
2	Cufflinks	exon	2788914	2788982	.	+	.	gene_id GRMZM2G102349; transcript_id TCONS_00010351; exon_number 6; oId CUFF.20393.1; tss_id TSS21445;
2	Cufflinks	exon	2790092	2790163	.	+	.	gene_id GRMZM2G102349; transcript_id TCONS_00010351; exon_number 7; oId CUFF.20393.1; tss_id TSS21445;
2	Cufflinks	exon	2790253	2790300	.	+	.	gene_id GRMZM2G102349; transcript_id TCONS_00010351; exon_number 8; oId CUFF.20393.1; tss_id TSS21445;
2	Cufflinks	exon	2790555	2791301	.	+	.	gene_id GRMZM2G102349; transcript_id TCONS_00010351; exon_number 9; oId CUFF.20393.1; tss_id TSS21445;
2	Cufflinks	exon	3978982	3979406	.	+	.	gene_id XLOC_009093; transcript_id TCONS_00010405; exon_number 1; oId CUFF.20534.1; tss_id TSS21531;

2	Cufflinks	exon	5306604	5307559	.	+	.	gene_id XLOC_009137; transcript_id TCONS_00010453; exon_number 1; oId CUFF.20682.1; tss_id TSS21611;
2	Cufflinks	exon	6058782	6059605	.	+	.	gene_id XLOC_009154; transcript_id TCONS_00010472; exon_number 1; oId CUFF.20743.1; tss_id TSS21649;
2	Cufflinks	exon	6991269	6991847	.	+	.	gene_id XLOC_009175; transcript_id TCONS_00010500; exon_number 1; oId CUFF.20830.1; tss_id TSS21698;
2	Cufflinks	exon	7049348	7050124	.	+	.	gene_id XLOC_009182; transcript_id TCONS_00010509; exon_number 1; oId CUFF.20858.1; tss_id TSS21706;
2	Cufflinks	exon	7519507	7519827	.	+	.	gene_id GRMZM2G164400; transcript_id TCONS_00010527; exon_number 1; oId CUFF.20879.1; tss_id TSS21733;
2	Cufflinks	exon	7520576	7520806	.	+	.	gene_id GRMZM2G164400; transcript_id TCONS_00010527; exon_number 2; oId CUFF.20879.1; tss_id TSS21733;
2	Cufflinks	exon	7520898	7520945	.	+	.	gene_id GRMZM2G164400; transcript_id TCONS_00010527; exon_number 3; oId CUFF.20879.1; tss_id TSS21733;
2	Cufflinks	exon	7521031	7521087	.	+	.	gene_id GRMZM2G164400; transcript_id TCONS_00010527; exon_number 4; oId CUFF.20879.1; tss_id TSS21733;
2	Cufflinks	exon	7521164	7521265	.	+	.	gene_id GRMZM2G164400; transcript_id TCONS_00010527; exon_number 5; oId CUFF.20879.1; tss_id TSS21733;
2	Cufflinks	exon	7521408	7521473	.	+	.	gene_id GRMZM2G164400; transcript_id TCONS_00010527; exon_number 6; oId CUFF.20879.1; tss_id TSS21733;
2	Cufflinks	exon	7526980	7527081	.	+	.	gene_id GRMZM2G164400; transcript_id TCONS_00010527; exon_number 7; oId CUFF.20879.1; tss_id TSS21733;
2	Cufflinks	exon	7527169	7527284	.	+	.	gene_id GRMZM2G164400; transcript_id TCONS_00010527; exon_number 8; oId CUFF.20879.1; tss_id TSS21733;
2	Cufflinks	exon	7527470	7527867	.	+	.	gene_id GRMZM2G164400; transcript_id TCONS_00010527; exon_number 9; oId CUFF.20879.1; tss_id TSS21733;
2	Cufflinks	exon	9117236	9117438	.	+	.	gene_id GRMZM2G084181; transcript_id TCONS_00010568; exon_number 1; oId CUFF.21009.1; tss_id TSS21787;
2	Cufflinks	exon	9118345	9118728	.	+	.	gene_id GRMZM2G084181; transcript_id TCONS_00010568; exon_number 2; oId CUFF.21009.1; tss_id TSS21787;
2	Cufflinks	exon	9119039	9119220	.	+	.	gene_id GRMZM2G084181; transcript_id TCONS_00010568; exon_number 3; oId CUFF.21009.1; tss_id TSS21787;
2	Cufflinks	exon	9119338	9119377	.	+	.	gene_id GRMZM2G084181; transcript_id TCONS_00010568; exon_number 4; oId CUFF.21009.1; tss_id TSS21787;
2	Cufflinks	exon	9119720	9119879	.	+	.	gene_id GRMZM2G084181; transcript_id TCONS_00010568; exon_number 5; oId CUFF.21009.1; tss_id TSS21787;
2	Cufflinks	exon	9119969	9120083	.	+	.	gene_id GRMZM2G084181; transcript_id TCONS_00010568; exon_number 6; oId CUFF.21009.1; tss_id TSS21787;
2	Cufflinks	exon	9120160	9120240	.	+	.	gene_id GRMZM2G084181; transcript_id TCONS_00010568; exon_number 7; oId CUFF.21009.1; tss_id TSS21787;
2	Cufflinks	exon	9120321	9120345	.	+	.	gene_id GRMZM2G084181; transcript_id TCONS_00010568; exon_number 8; oId CUFF.21009.1; tss_id TSS21787;
2	Cufflinks	exon	9120454	9120510	.	+	.	gene_id GRMZM2G084181; transcript_id TCONS_00010568; exon_number 9; oId CUFF.21009.1; tss_id TSS21787;
2	Cufflinks	exon	9120660	9120725	.	+	.	gene_id GRMZM2G084181; transcript_id TCONS_00010568; exon_number 10; oId CUFF.21009.1; tss_id TSS21787;
2	Cufflinks	exon	9120829	9120903	.	+	.	gene_id GRMZM2G084181; transcript_id TCONS_00010568; exon_number 11; oId CUFF.21009.1; tss_id TSS21787;
2	Cufflinks	exon	9121332	9121439	.	+	.	gene_id GRMZM2G084181; transcript_id TCONS_00010568; exon_number 12; oId CUFF.21009.1; tss_id TSS21787;
2	Cufflinks	exon	9121586	9121720	.	+	.	gene_id GRMZM2G084181; transcript_id TCONS_00010568; exon_number 13; oId CUFF.21009.1; tss_id TSS21787;
2	Cufflinks	exon	9122635	9122741	.	+	.	gene_id GRMZM2G084181; transcript_id TCONS_00010568; exon_number 14; oId CUFF.21009.1; tss_id TSS21787;
2	Cufflinks	exon	9122830	9122923	.	+	.	gene_id GRMZM2G084181; transcript_id TCONS_00010568; exon_number 15; oId CUFF.21009.1; tss_id TSS21787;
2	Cufflinks	exon	9123062	9123229	.	+	.	gene_id GRMZM2G084181; transcript_id TCONS_00010568; exon_number 16; oId CUFF.21009.1; tss_id TSS21787;
2	Cufflinks	exon	9123547	9123687	.	+	.	gene_id GRMZM2G084181; transcript_id TCONS_00010568; exon_number 17; oId CUFF.21009.1; tss_id TSS21787;
2	Cufflinks	exon	9124158	9124364	.	+	.	gene_id GRMZM2G084181; transcript_id TCONS_00010568; exon_number 18; oId CUFF.21009.1; tss_id TSS21787;
2	Cufflinks	exon	9124442	9124540	.	+	.	gene_id GRMZM2G084181; transcript_id TCONS_00010568; exon_number 19; oId CUFF.21009.1; tss_id TSS21787;
2	Cufflinks	exon	9124759	9124911	.	+	.	gene_id GRMZM2G084181; transcript_id TCONS_00010568; exon_number 20; oId CUFF.21009.1; tss_id TSS21787;
2	Cufflinks	exon	9125624	9126012	.	+	.	gene_id GRMZM2G084181; transcript_id TCONS_00010568; exon_number 21; oId CUFF.21009.1; tss_id TSS21787;
2	Cufflinks	exon	9126101	9126281	.	+	.	gene_id GRMZM2G084181; transcript_id TCONS_00010568; exon_number 22; oId CUFF.21009.1; tss_id TSS21787;
2	Cufflinks	exon	9126380	9126703	.	+	.	gene_id GRMZM2G084181; transcript_id TCONS_00010568; exon_number 23; oId CUFF.21009.1; tss_id TSS21787;

2	Cufflinks	exon	9127035	9127791	.	+	.	gene_id GRMZM2G084181; transcript_id TCONS_00010568; exon_number 24; oId CUFF.21009.1; tss_id TSS21787;
2	Cufflinks	exon	9127880	9128001	.	+	.	gene_id GRMZM2G084181; transcript_id TCONS_00010568; exon_number 25; oId CUFF.21009.1; tss_id TSS21787;
2	Cufflinks	exon	9128535	9128780	.	+	.	gene_id GRMZM2G084181; transcript_id TCONS_00010568; exon_number 26; oId CUFF.21009.1; tss_id TSS21787;
2	Cufflinks	exon	9129132	9129233	.	+	.	gene_id GRMZM2G084181; transcript_id TCONS_00010568; exon_number 27; oId CUFF.21009.1; tss_id TSS21787;
2	Cufflinks	exon	9129396	9129717	.	+	.	gene_id GRMZM2G084181; transcript_id TCONS_00010568; exon_number 28; oId CUFF.21009.1; tss_id TSS21787;
2	Cufflinks	exon	9130119	9130542	.	+	.	gene_id GRMZM2G084181; transcript_id TCONS_00010568; exon_number 29; oId CUFF.21009.1; tss_id TSS21787;
2	Cufflinks	exon	9551436	9551633	.	+	.	gene_id GRMZM2G094328; transcript_id TCONS_00010574; exon_number 1; oId CUFF.21005.1; tss_id TSS21800;
2	Cufflinks	exon	9551747	9552864	.	+	.	gene_id GRMZM2G094328; transcript_id TCONS_00010574; exon_number 2; oId CUFF.21005.1; tss_id TSS21800;
2	Cufflinks	exon	13715062	13717432	.	+	.	gene_id GRMZM2G300111; transcript_id TCONS_00010683; exon_number 1; oId CUFF.21367.1; tss_id TSS22009;
2	Cufflinks	exon	18735506	18737183	.	+	.	gene_id GRMZM2G175504; transcript_id TCONS_00010776; exon_number 1; oId CUFF.21715.1; tss_id TSS22206;
2	Cufflinks	exon	21891133	21893168	.	+	.	gene_id GRMZM2G320827; transcript_id TCONS_00010826; exon_number 1; oId CUFF.21904.1; tss_id TSS22324;
2	Cufflinks	exon	22221051	22221648	.	+	.	gene_id GRMZM2G351330; transcript_id TCONS_00010830; exon_number 1; oId CUFF.21929.1; tss_id TSS22331;
2	Cufflinks	exon	22221780	22222694	.	+	.	gene_id GRMZM2G351330; transcript_id TCONS_00010830; exon_number 2; oId CUFF.21929.1; tss_id TSS22331;
2	Cufflinks	exon	25628639	25629596	.	+	.	gene_id XLOC_009507; transcript_id TCONS_00010890; exon_number 1; oId CUFF.22164.1; tss_id TSS22446;
2	Cufflinks	exon	26759318	26763396	.	+	.	gene_id GRMZM2G123122; transcript_id TCONS_00010909; exon_number 1; oId CUFF.22204.1; tss_id TSS22477;
2	Cufflinks	exon	27500744	27502059	.	+	.	gene_id GRMZM2G362413; transcript_id TCONS_00010924; exon_number 1; oId CUFF.22242.1; tss_id TSS22499;
2	Cufflinks	exon	28494828	28495455	.	+	.	gene_id XLOC_009548; transcript_id TCONS_00010942; exon_number 1; oId CUFF.22329.1; tss_id TSS22528;
2	Cufflinks	exon	30170685	30171385	.	+	.	gene_id XLOC_009576; transcript_id TCONS_00010972; exon_number 1; oId CUFF.22423.1; tss_id TSS22572;
2	Cufflinks	exon	32571968	32573911	.	+	.	gene_id XLOC_009602; transcript_id TCONS_00011000; exon_number 1; oId CUFF.22558.1; tss_id TSS22634;
2	Cufflinks	exon	37392043	37392784	.	+	.	gene_id GRMZM2G063262; transcript_id TCONS_00011060; exon_number 1; oId CUFF.22816.3; tss_id TSS22740;
2	Cufflinks	exon	37392858	37393077	.	+	.	gene_id GRMZM2G063262; transcript_id TCONS_00011060; exon_number 2; oId CUFF.22816.3; tss_id TSS22740;
2	Cufflinks	exon	37393690	37393798	.	+	.	gene_id GRMZM2G063262; transcript_id TCONS_00011060; exon_number 3; oId CUFF.22816.3; tss_id TSS22740;
2	Cufflinks	exon	37393898	37394090	.	+	.	gene_id GRMZM2G063262; transcript_id TCONS_00011060; exon_number 4; oId CUFF.22816.3; tss_id TSS22740;
2	Cufflinks	exon	37394193	37394329	.	+	.	gene_id GRMZM2G063262; transcript_id TCONS_00011060; exon_number 5; oId CUFF.22816.3; tss_id TSS22740;
2	Cufflinks	exon	37394600	37394888	.	+	.	gene_id GRMZM2G063262; transcript_id TCONS_00011060; exon_number 6; oId CUFF.22816.3; tss_id TSS22740;
2	Cufflinks	exon	37395058	37395164	.	+	.	gene_id GRMZM2G063262; transcript_id TCONS_00011060; exon_number 7; oId CUFF.22816.3; tss_id TSS22740;
2	Cufflinks	exon	37395242	37395398	.	+	.	gene_id GRMZM2G063262; transcript_id TCONS_00011060; exon_number 8; oId CUFF.22816.3; tss_id TSS22740;
2	Cufflinks	exon	37395547	37395745	.	+	.	gene_id GRMZM2G063262; transcript_id TCONS_00011060; exon_number 9; oId CUFF.22816.3; tss_id TSS22740;
2	Cufflinks	exon	37395869	37396109	.	+	.	gene_id GRMZM2G063262; transcript_id TCONS_00011060; exon_number 10; oId CUFF.22816.3; tss_id TSS22740;
2	Cufflinks	exon	37396216	37396674	.	+	.	gene_id GRMZM2G063262; transcript_id TCONS_00011060; exon_number 11; oId CUFF.22816.3; tss_id TSS22740;
2	Cufflinks	exon	37392043	37392784	.	+	.	gene_id GRMZM2G063262; transcript_id TCONS_00011061; exon_number 1; oId CUFF.22816.2; tss_id TSS22740;
2	Cufflinks	exon	37392841	37393077	.	+	.	gene_id GRMZM2G063262; transcript_id TCONS_00011061; exon_number 2; oId CUFF.22816.2; tss_id TSS22740;
2	Cufflinks	exon	37393690	37393798	.	+	.	gene_id GRMZM2G063262; transcript_id TCONS_00011061; exon_number 3; oId CUFF.22816.2; tss_id TSS22740;
2	Cufflinks	exon	37393898	37394090	.	+	.	gene_id GRMZM2G063262; transcript_id TCONS_00011061; exon_number 4; oId CUFF.22816.2; tss_id TSS22740;
2	Cufflinks	exon	37394193	37394329	.	+	.	gene_id GRMZM2G063262; transcript_id TCONS_00011061; exon_number 5; oId CUFF.22816.2; tss_id TSS22740;
2	Cufflinks	exon	37394600	37394888	.	+	.	gene_id GRMZM2G063262; transcript_id TCONS_00011061; exon_number 6; oId CUFF.22816.2; tss_id TSS22740;

2	Cufflinks	exon	37395058	37395164	.	+	.	gene_id GRMZM2G063262; transcript_id TCONS_00011061; exon_number 7; oId CUFF.22816.2; tss_id TSS22740;
2	Cufflinks	exon	37395242	37395398	.	+	.	gene_id GRMZM2G063262; transcript_id TCONS_00011061; exon_number 8; oId CUFF.22816.2; tss_id TSS22740;
2	Cufflinks	exon	37395547	37395745	.	+	.	gene_id GRMZM2G063262; transcript_id TCONS_00011061; exon_number 9; oId CUFF.22816.2; tss_id TSS22740;
2	Cufflinks	exon	37395869	37396109	.	+	.	gene_id GRMZM2G063262; transcript_id TCONS_00011061; exon_number 10; oId CUFF.22816.2; tss_id TSS22740;
2	Cufflinks	exon	37396216	37396674	.	+	.	gene_id GRMZM2G063262; transcript_id TCONS_00011061; exon_number 11; oId CUFF.22816.2; tss_id TSS22740;
2	Cufflinks	exon	38760903	38761507	.	+	.	gene_id GRMZM2G147884; transcript_id TCONS_00011084; exon_number 1; oId CUFF.22862.1; tss_id TSS22776;
2	Cufflinks	exon	38763510	38763619	.	+	.	gene_id GRMZM2G147884; transcript_id TCONS_00011084; exon_number 2; oId CUFF.22862.1; tss_id TSS22776;
2	Cufflinks	exon	38763702	38764007	.	+	.	gene_id GRMZM2G147884; transcript_id TCONS_00011084; exon_number 3; oId CUFF.22862.1; tss_id TSS22776;
2	Cufflinks	exon	38764265	38764377	.	+	.	gene_id GRMZM2G147884; transcript_id TCONS_00011084; exon_number 4; oId CUFF.22862.1; tss_id TSS22776;
2	Cufflinks	exon	38764485	38764815	.	+	.	gene_id GRMZM2G147884; transcript_id TCONS_00011084; exon_number 5; oId CUFF.22862.1; tss_id TSS22776;
2	Cufflinks	exon	38764893	38765108	.	+	.	gene_id GRMZM2G147884; transcript_id TCONS_00011084; exon_number 6; oId CUFF.22862.1; tss_id TSS22776;
2	Cufflinks	exon	38765195	38765431	.	+	.	gene_id GRMZM2G147884; transcript_id TCONS_00011084; exon_number 7; oId CUFF.22862.1; tss_id TSS22776;
2	Cufflinks	exon	38766022	38766207	.	+	.	gene_id GRMZM2G147884; transcript_id TCONS_00011084; exon_number 8; oId CUFF.22862.1; tss_id TSS22776;
2	Cufflinks	exon	38766518	38766658	.	+	.	gene_id GRMZM2G147884; transcript_id TCONS_00011084; exon_number 9; oId CUFF.22862.1; tss_id TSS22776;
2	Cufflinks	exon	38767213	38767481	.	+	.	gene_id GRMZM2G147884; transcript_id TCONS_00011084; exon_number 10; oId CUFF.22862.1; tss_id TSS22776;
2	Cufflinks	exon	38767580	38767661	.	+	.	gene_id GRMZM2G147884; transcript_id TCONS_00011084; exon_number 11; oId CUFF.22862.1; tss_id TSS22776;
2	Cufflinks	exon	38767801	38768270	.	+	.	gene_id GRMZM2G147884; transcript_id TCONS_00011084; exon_number 12; oId CUFF.22862.1; tss_id TSS22776;
2	Cufflinks	exon	40051916	40052322	.	+	.	gene_id XLOC_009682; transcript_id TCONS_00011098; exon_number 1; oId CUFF.22907.2; tss_id TSS22803;
2	Cufflinks	exon	40053975	40054726	.	+	.	gene_id XLOC_009682; transcript_id TCONS_00011098; exon_number 2; oId CUFF.22907.2; tss_id TSS22803;
2	Cufflinks	exon	46616631	46616958	.	+	.	gene_id GRMZM2G028258; transcript_id TCONS_00011193; exon_number 1; oId CUFF.23236.1; tss_id TSS22957;
2	Cufflinks	exon	46617346	46617567	.	+	.	gene_id GRMZM2G028258; transcript_id TCONS_00011193; exon_number 2; oId CUFF.23236.1; tss_id TSS22957;
2	Cufflinks	exon	46617726	46618425	.	+	.	gene_id GRMZM2G028258; transcript_id TCONS_00011193; exon_number 3; oId CUFF.23236.1; tss_id TSS22957;
2	Cufflinks	exon	46618517	46618597	.	+	.	gene_id GRMZM2G028258; transcript_id TCONS_00011193; exon_number 4; oId CUFF.23236.1; tss_id TSS22957;
2	Cufflinks	exon	46618683	46618781	.	+	.	gene_id GRMZM2G028258; transcript_id TCONS_00011193; exon_number 5; oId CUFF.23236.1; tss_id TSS22957;
2	Cufflinks	exon	46618878	46619099	.	+	.	gene_id GRMZM2G028258; transcript_id TCONS_00011193; exon_number 6; oId CUFF.23236.1; tss_id TSS22957;
2	Cufflinks	exon	46619184	46619375	.	+	.	gene_id GRMZM2G028258; transcript_id TCONS_00011193; exon_number 7; oId CUFF.23236.1; tss_id TSS22957;
2	Cufflinks	exon	46619462	46619537	.	+	.	gene_id GRMZM2G028258; transcript_id TCONS_00011193; exon_number 8; oId CUFF.23236.1; tss_id TSS22957;
2	Cufflinks	exon	46619651	46619945	.	+	.	gene_id GRMZM2G028258; transcript_id TCONS_00011193; exon_number 9; oId CUFF.23236.1; tss_id TSS22957;
2	Cufflinks	exon	46620076	46620668	.	+	.	gene_id GRMZM2G028258; transcript_id TCONS_00011193; exon_number 10; oId CUFF.23236.1; tss_id TSS22957;
2	Cufflinks	exon	62122873	62123618	.	+	.	gene_id XLOC_009891; transcript_id TCONS_00011322; exon_number 1; oId CUFF.23746.1; tss_id TSS23229;
2	Cufflinks	exon	64294902	64296104	.	+	.	gene_id GRMZM2G436533; transcript_id TCONS_00011336; exon_number 1; oId CUFF.23817.1; tss_id TSS23255;
2	Cufflinks	exon	64296926	64296976	.	+	.	gene_id GRMZM2G436533; transcript_id TCONS_00011336; exon_number 2; oId CUFF.23817.1; tss_id TSS23255;
2	Cufflinks	exon	64297997	64298511	.	+	.	gene_id GRMZM2G436533; transcript_id TCONS_00011336; exon_number 3; oId CUFF.23817.1; tss_id TSS23255;
2	Cufflinks	exon	86977308	86977770	.	+	.	gene_id GRMZM2G027307; transcript_id TCONS_00011466; exon_number 1; oId CUFF.24439.1; tss_id TSS23580;
2	Cufflinks	exon	86978279	86978522	.	+	.	gene_id GRMZM2G027307; transcript_id TCONS_00011466; exon_number 2; oId CUFF.24439.1; tss_id TSS23580;
2	Cufflinks	exon	86978611	86978702	.	+	.	gene_id GRMZM2G027307; transcript_id TCONS_00011466; exon_number 3; oId CUFF.24439.1; tss_id TSS23580;

2	Cufflinks	exon	86989451	86989574	.	+	.	gene_id GRMZM2G027307; transcript_id TCONS_00011466; exon_number 4; oId CUFF.24439.1; tss_id TSS23580;
2	Cufflinks	exon	86989651	86989736	.	+	.	gene_id GRMZM2G027307; transcript_id TCONS_00011466; exon_number 5; oId CUFF.24439.1; tss_id TSS23580;
2	Cufflinks	exon	86989812	86989877	.	+	.	gene_id GRMZM2G027307; transcript_id TCONS_00011466; exon_number 6; oId CUFF.24439.1; tss_id TSS23580;
2	Cufflinks	exon	86990746	86991387	.	+	.	gene_id GRMZM2G027307; transcript_id TCONS_00011466; exon_number 7; oId CUFF.24439.1; tss_id TSS23580;
2	Cufflinks	exon	113942575	113944157	.	+	.	gene_id GRMZM2G076343; transcript_id TCONS_00011591; exon_number 1; oId CUFF.25109.1; tss_id TSS23875;
2	Cufflinks	exon	157931493	157932381	.	+	.	gene_id XLOC_010349; transcript_id TCONS_00011843; exon_number 1; oId CUFF.26212.1; tss_id TSS24432;
2	Cufflinks	exon	162045456	162047512	.	+	.	gene_id XLOC_010373; transcript_id TCONS_00011871; exon_number 1; oId CUFF.26441.1; tss_id TSS24500;
2	Cufflinks	exon	173494956	173495067	.	+	.	gene_id XLOC_010473; transcript_id TCONS_00011987; exon_number 1; oId CUFF.26774.2; tss_id TSS24752;
2	Cufflinks	exon	173495509	173495587	.	+	.	gene_id XLOC_010473; transcript_id TCONS_00011987; exon_number 2; oId CUFF.26774.2; tss_id TSS24752;
2	Cufflinks	exon	173495704	173495774	.	+	.	gene_id XLOC_010473; transcript_id TCONS_00011987; exon_number 3; oId CUFF.26774.2; tss_id TSS24752;
2	Cufflinks	exon	173495869	173496273	.	+	.	gene_id XLOC_010473; transcript_id TCONS_00011987; exon_number 4; oId CUFF.26774.2; tss_id TSS24752;
2	Cufflinks	exon	173494956	173495405	.	+	.	gene_id XLOC_010473; transcript_id TCONS_00011988; exon_number 1; oId CUFF.26774.1; tss_id TSS24752;
2	Cufflinks	exon	173495509	173495587	.	+	.	gene_id XLOC_010473; transcript_id TCONS_00011988; exon_number 2; oId CUFF.26774.1; tss_id TSS24752;
2	Cufflinks	exon	173495704	173495774	.	+	.	gene_id XLOC_010473; transcript_id TCONS_00011988; exon_number 3; oId CUFF.26774.1; tss_id TSS24752;
2	Cufflinks	exon	173495869	173496273	.	+	.	gene_id XLOC_010473; transcript_id TCONS_00011988; exon_number 4; oId CUFF.26774.1; tss_id TSS24752;
2	Cufflinks	exon	179011357	179012135	.	+	.	gene_id XLOC_010532; transcript_id TCONS_00012053; exon_number 1; oId CUFF.27009.1; tss_id TSS24885;
2	Cufflinks	exon	186829385	186830074	.	+	.	gene_id GRMZM2G002131; transcript_id TCONS_00012131; exon_number 1; oId CUFF.27329.1; tss_id TSS25029;
2	Cufflinks	exon	186832431	186833823	.	+	.	gene_id GRMZM2G002131; transcript_id TCONS_00012131; exon_number 2; oId CUFF.27329.1; tss_id TSS25029;
2	Cufflinks	exon	189927099	189928613	.	+	.	gene_id XLOC_010651; transcript_id TCONS_00012186; exon_number 1; oId CUFF.27502.1; tss_id TSS25133;
2	Cufflinks	exon	196564846	196565285	.	+	.	gene_id GRMZM2G002828; transcript_id TCONS_00012281; exon_number 1; oId CUFF.27845.1; tss_id TSS25315;
2	Cufflinks	exon	196565994	196566018	.	+	.	gene_id GRMZM2G002828; transcript_id TCONS_00012281; exon_number 2; oId CUFF.27845.1; tss_id TSS25315;
2	Cufflinks	exon	196567461	196567590	.	+	.	gene_id GRMZM2G002828; transcript_id TCONS_00012281; exon_number 3; oId CUFF.27845.1; tss_id TSS25315;
2	Cufflinks	exon	196567690	196567820	.	+	.	gene_id GRMZM2G002828; transcript_id TCONS_00012281; exon_number 4; oId CUFF.27845.1; tss_id TSS25315;
2	Cufflinks	exon	196567918	196568006	.	+	.	gene_id GRMZM2G002828; transcript_id TCONS_00012281; exon_number 5; oId CUFF.27845.1; tss_id TSS25315;
2	Cufflinks	exon	196568410	196568486	.	+	.	gene_id GRMZM2G002828; transcript_id TCONS_00012281; exon_number 6; oId CUFF.27845.1; tss_id TSS25315;
2	Cufflinks	exon	196568561	196569244	.	+	.	gene_id GRMZM2G002828; transcript_id TCONS_00012281; exon_number 7; oId CUFF.27845.1; tss_id TSS25315;
2	Cufflinks	exon	196700585	196700931	.	+	.	gene_id GRMZM2G058105; transcript_id TCONS_00012283; exon_number 1; oId CUFF.27964.1; tss_id TSS25320;
2	Cufflinks	exon	196701018	196701375	.	+	.	gene_id GRMZM2G058105; transcript_id TCONS_00012283; exon_number 2; oId CUFF.27964.1; tss_id TSS25320;
2	Cufflinks	exon	196701461	196701510	.	+	.	gene_id GRMZM2G058105; transcript_id TCONS_00012283; exon_number 3; oId CUFF.27964.1; tss_id TSS25320;
2	Cufflinks	exon	196702573	196702614	.	+	.	gene_id GRMZM2G058105; transcript_id TCONS_00012283; exon_number 4; oId CUFF.27964.1; tss_id TSS25320;
2	Cufflinks	exon	196702717	196702838	.	+	.	gene_id GRMZM2G058105; transcript_id TCONS_00012283; exon_number 5; oId CUFF.27964.1; tss_id TSS25320;
2	Cufflinks	exon	196702924	196703032	.	+	.	gene_id GRMZM2G058105; transcript_id TCONS_00012283; exon_number 6; oId CUFF.27964.1; tss_id TSS25320;
2	Cufflinks	exon	196747072	196747523	.	+	.	gene_id GRMZM2G058105; transcript_id TCONS_00012283; exon_number 7; oId CUFF.27964.1; tss_id TSS25320;
2	Cufflinks	exon	199224685	199225129	.	+	.	gene_id GRMZM2G081571; transcript_id TCONS_00012336; exon_number 1; oId CUFF.28035.1; tss_id TSS25414;
2	Cufflinks	exon	199225252	199225401	.	+	.	gene_id GRMZM2G081571; transcript_id TCONS_00012336; exon_number 2; oId CUFF.28035.1; tss_id TSS25414;
2	Cufflinks	exon	199229058	199229234	.	+	.	gene_id GRMZM2G081571; transcript_id TCONS_00012336; exon_number 3; oId CUFF.28035.1; tss_id TSS25414;

2	Cufflinks	exon	199229335	199229423	.	+	.	gene_id GRMZM2G081571; transcript_id TCONS_00012336; exon_number 4; oId CUFF.28035.1; tss_id TSS25414;
2	Cufflinks	exon	199229523	199229587	.	+	.	gene_id GRMZM2G081571; transcript_id TCONS_00012336; exon_number 5; oId CUFF.28035.1; tss_id TSS25414;
2	Cufflinks	exon	199229727	199229829	.	+	.	gene_id GRMZM2G081571; transcript_id TCONS_00012336; exon_number 6; oId CUFF.28035.1; tss_id TSS25414;
2	Cufflinks	exon	199230276	199230386	.	+	.	gene_id GRMZM2G081571; transcript_id TCONS_00012336; exon_number 7; oId CUFF.28035.1; tss_id TSS25414;
2	Cufflinks	exon	199230522	199231534	.	+	.	gene_id GRMZM2G081571; transcript_id TCONS_00012336; exon_number 8; oId CUFF.28035.1; tss_id TSS25414;
2	Cufflinks	exon	202063279	202063720	.	+	.	gene_id GRMZM2G169694; transcript_id TCONS_00012378; exon_number 1; oId CUFF.28250.1; tss_id TSS25525;
2	Cufflinks	exon	202070819	202070889	.	+	.	gene_id GRMZM2G169694; transcript_id TCONS_00012378; exon_number 2; oId CUFF.28250.1; tss_id TSS25525;
2	Cufflinks	exon	202070978	202071066	.	+	.	gene_id GRMZM2G169694; transcript_id TCONS_00012378; exon_number 3; oId CUFF.28250.1; tss_id TSS25525;
2	Cufflinks	exon	202071228	202071339	.	+	.	gene_id GRMZM2G169694; transcript_id TCONS_00012378; exon_number 4; oId CUFF.28250.1; tss_id TSS25525;
2	Cufflinks	exon	202072488	202072578	.	+	.	gene_id GRMZM2G169694; transcript_id TCONS_00012378; exon_number 5; oId CUFF.28250.1; tss_id TSS25525;
2	Cufflinks	exon	202072771	202073506	.	+	.	gene_id GRMZM2G169694; transcript_id TCONS_00012378; exon_number 6; oId CUFF.28250.1; tss_id TSS25525;
2	Cufflinks	exon	208500848	208501066	.	+	.	gene_id GRMZM2G431309; transcript_id TCONS_00012513; exon_number 1; oId CUFF.28696.1; tss_id TSS25783;
2	Cufflinks	exon	208502780	208505599	.	+	.	gene_id GRMZM2G431309; transcript_id TCONS_00012513; exon_number 2; oId CUFF.28696.1; tss_id TSS25783;
2	Cufflinks	exon	210207604	210208075	.	+	.	gene_id XLOC_010974; transcript_id TCONS_00012547; exon_number 1; oId CUFF.28771.1; tss_id TSS25841;
2	Cufflinks	exon	211499169	211499602	.	+	.	gene_id GRMZM2G412888; transcript_id TCONS_00012562; exon_number 1; oId CUFF.28857.1; tss_id TSS25874;
2	Cufflinks	exon	211501508	211501543	.	+	.	gene_id GRMZM2G412888; transcript_id TCONS_00012562; exon_number 2; oId CUFF.28857.1; tss_id TSS25874;
2	Cufflinks	exon	211501636	211501714	.	+	.	gene_id GRMZM2G412888; transcript_id TCONS_00012562; exon_number 3; oId CUFF.28857.1; tss_id TSS25874;
2	Cufflinks	exon	211501838	211501892	.	+	.	gene_id GRMZM2G412888; transcript_id TCONS_00012562; exon_number 4; oId CUFF.28857.1; tss_id TSS25874;
2	Cufflinks	exon	211501967	211502083	.	+	.	gene_id GRMZM2G412888; transcript_id TCONS_00012562; exon_number 5; oId CUFF.28857.1; tss_id TSS25874;
2	Cufflinks	exon	211503363	211503527	.	+	.	gene_id GRMZM2G412888; transcript_id TCONS_00012562; exon_number 6; oId CUFF.28857.1; tss_id TSS25874;
2	Cufflinks	exon	211503892	211504147	.	+	.	gene_id GRMZM2G412888; transcript_id TCONS_00012562; exon_number 7; oId CUFF.28857.1; tss_id TSS25874;
2	Cufflinks	exon	211504457	211505370	.	+	.	gene_id GRMZM2G412888; transcript_id TCONS_00012562; exon_number 8; oId CUFF.28857.1; tss_id TSS25874;
2	Cufflinks	exon	211875412	211875733	.	+	.	gene_id GRMZM2G101769; transcript_id TCONS_00012575; exon_number 1; oId CUFF.28885.1; tss_id TSS25893;
2	Cufflinks	exon	211875973	211876117	.	+	.	gene_id GRMZM2G101769; transcript_id TCONS_00012575; exon_number 2; oId CUFF.28885.1; tss_id TSS25893;
2	Cufflinks	exon	211876872	211877134	.	+	.	gene_id GRMZM2G101769; transcript_id TCONS_00012575; exon_number 3; oId CUFF.28885.1; tss_id TSS25893;
2	Cufflinks	exon	211877242	211877299	.	+	.	gene_id GRMZM2G101769; transcript_id TCONS_00012575; exon_number 4; oId CUFF.28885.1; tss_id TSS25893;
2	Cufflinks	exon	211877404	211877983	.	+	.	gene_id GRMZM2G101769; transcript_id TCONS_00012575; exon_number 5; oId CUFF.28885.1; tss_id TSS25893;
2	Cufflinks	exon	214483242	214483828	.	+	.	gene_id XLOC_011057; transcript_id TCONS_00012644; exon_number 1; oId CUFF.29059.1; tss_id TSS25997;
2	Cufflinks	exon	215589169	215591897	.	+	.	gene_id GRMZM2G459363; transcript_id TCONS_00012675; exon_number 1; oId CUFF.29149.1; tss_id TSS26062;
2	Cufflinks	exon	221314181	221314378	.	+	.	gene_id GRMZM5G843434; transcript_id TCONS_00012781; exon_number 1; oId CUFF.29542.1; tss_id TSS26280;
2	Cufflinks	exon	221314586	221314620	.	+	.	gene_id GRMZM5G843434; transcript_id TCONS_00012781; exon_number 2; oId CUFF.29542.1; tss_id TSS26280;
2	Cufflinks	exon	221314728	221316190	.	+	.	gene_id GRMZM5G843434; transcript_id TCONS_00012781; exon_number 3; oId CUFF.29542.1; tss_id TSS26280;
2	Cufflinks	exon	221314181	221314620	.	+	.	gene_id GRMZM5G843434; transcript_id TCONS_00012782; exon_number 1; oId CUFF.29542.2; tss_id TSS26280;
2	Cufflinks	exon	221314728	221316190	.	+	.	gene_id GRMZM5G843434; transcript_id TCONS_00012782; exon_number 2; oId CUFF.29542.2; tss_id TSS26280;
2	Cufflinks	exon	232655972	232657479	.	+	.	gene_id XLOC_011352; transcript_id TCONS_00012966; exon_number 1; oId CUFF.30180.1; tss_id TSS26649;
2	Cufflinks	exon	233546506	233548765	.	+	.	gene_id XLOC_011373; transcript_id TCONS_00012991; exon_number 1; oId CUFF.30256.1; tss_id TSS26686;

2	Cufflinks	exon	235377544	235378343	.	+	.	gene_id GRMZM2G324544; transcript_id TCONS_00013043; exon_number 1; oId CUFF.30440.1; tss_id TSS26770;
2	Cufflinks	exon	1439756	1440808	.	-	.	gene_id GRMZM2G019500; transcript_id TCONS_00013101; exon_number 1; oId CUFF.20260.1; tss_id TSS26914;
2	Cufflinks	exon	1440904	1441057	.	-	.	gene_id GRMZM2G019500; transcript_id TCONS_00013101; exon_number 2; oId CUFF.20260.1; tss_id TSS26914;
2	Cufflinks	exon	1443633	1444066	.	-	.	gene_id GRMZM2G019500; transcript_id TCONS_00013101; exon_number 3; oId CUFF.20260.1; tss_id TSS26914;
2	Cufflinks	exon	2544036	2544539	.	-	.	gene_id GRMZM2G009994; transcript_id TCONS_00013134; exon_number 1; oId CUFF.20413.2; tss_id TSS26986;
2	Cufflinks	exon	2544774	2544937	.	-	.	gene_id GRMZM2G009994; transcript_id TCONS_00013134; exon_number 2; oId CUFF.20413.2; tss_id TSS26986;
2	Cufflinks	exon	2545236	2545319	.	-	.	gene_id GRMZM2G009994; transcript_id TCONS_00013134; exon_number 3; oId CUFF.20413.2; tss_id TSS26986;
2	Cufflinks	exon	2545463	2545516	.	-	.	gene_id GRMZM2G009994; transcript_id TCONS_00013134; exon_number 4; oId CUFF.20413.2; tss_id TSS26986;
2	Cufflinks	exon	2545590	2545696	.	-	.	gene_id GRMZM2G009994; transcript_id TCONS_00013134; exon_number 5; oId CUFF.20413.2; tss_id TSS26986;
2	Cufflinks	exon	2545793	2545933	.	-	.	gene_id GRMZM2G009994; transcript_id TCONS_00013134; exon_number 6; oId CUFF.20413.2; tss_id TSS26986;
2	Cufflinks	exon	2546732	2546987	.	-	.	gene_id GRMZM2G009994; transcript_id TCONS_00013134; exon_number 7; oId CUFF.20413.2; tss_id TSS26986;
2	Cufflinks	exon	2547228	2547304	.	-	.	gene_id GRMZM2G009994; transcript_id TCONS_00013134; exon_number 8; oId CUFF.20413.2; tss_id TSS26986;
2	Cufflinks	exon	2547423	2547486	.	-	.	gene_id GRMZM2G009994; transcript_id TCONS_00013134; exon_number 9; oId CUFF.20413.2; tss_id TSS26986;
2	Cufflinks	exon	2548063	2548220	.	-	.	gene_id GRMZM2G009994; transcript_id TCONS_00013134; exon_number 10; oId CUFF.20413.2; tss_id TSS26986;
2	Cufflinks	exon	2549309	2549386	.	-	.	gene_id GRMZM2G009994; transcript_id TCONS_00013134; exon_number 11; oId CUFF.20413.2; tss_id TSS26986;
2	Cufflinks	exon	2549746	2550144	.	-	.	gene_id GRMZM2G009994; transcript_id TCONS_00013134; exon_number 12; oId CUFF.20413.2; tss_id TSS26986;
2	Cufflinks	exon	2550363	2550544	.	-	.	gene_id GRMZM2G009994; transcript_id TCONS_00013134; exon_number 13; oId CUFF.20413.2; tss_id TSS26986;
2	Cufflinks	exon	2544036	2544539	.	-	.	gene_id GRMZM2G009994; transcript_id TCONS_00013135; exon_number 1; oId CUFF.20413.3; tss_id TSS26986;
2	Cufflinks	exon	2544774	2544937	.	-	.	gene_id GRMZM2G009994; transcript_id TCONS_00013135; exon_number 2; oId CUFF.20413.3; tss_id TSS26986;
2	Cufflinks	exon	2545236	2545319	.	-	.	gene_id GRMZM2G009994; transcript_id TCONS_00013135; exon_number 3; oId CUFF.20413.3; tss_id TSS26986;
2	Cufflinks	exon	2545463	2545516	.	-	.	gene_id GRMZM2G009994; transcript_id TCONS_00013135; exon_number 4; oId CUFF.20413.3; tss_id TSS26986;
2	Cufflinks	exon	2545590	2545696	.	-	.	gene_id GRMZM2G009994; transcript_id TCONS_00013135; exon_number 5; oId CUFF.20413.3; tss_id TSS26986;
2	Cufflinks	exon	2545793	2545933	.	-	.	gene_id GRMZM2G009994; transcript_id TCONS_00013135; exon_number 6; oId CUFF.20413.3; tss_id TSS26986;
2	Cufflinks	exon	2546732	2546987	.	-	.	gene_id GRMZM2G009994; transcript_id TCONS_00013135; exon_number 7; oId CUFF.20413.3; tss_id TSS26986;
2	Cufflinks	exon	2547228	2547295	.	-	.	gene_id GRMZM2G009994; transcript_id TCONS_00013135; exon_number 8; oId CUFF.20413.3; tss_id TSS26986;
2	Cufflinks	exon	2547423	2547486	.	-	.	gene_id GRMZM2G009994; transcript_id TCONS_00013135; exon_number 9; oId CUFF.20413.3; tss_id TSS26986;
2	Cufflinks	exon	2548063	2548220	.	-	.	gene_id GRMZM2G009994; transcript_id TCONS_00013135; exon_number 10; oId CUFF.20413.3; tss_id TSS26986;
2	Cufflinks	exon	2549309	2549386	.	-	.	gene_id GRMZM2G009994; transcript_id TCONS_00013135; exon_number 11; oId CUFF.20413.3; tss_id TSS26986;
2	Cufflinks	exon	2549746	2550137	.	-	.	gene_id GRMZM2G009994; transcript_id TCONS_00013135; exon_number 12; oId CUFF.20413.3; tss_id TSS26986;
2	Cufflinks	exon	2550363	2550541	.	-	.	gene_id GRMZM2G009994; transcript_id TCONS_00013135; exon_number 13; oId CUFF.20413.3; tss_id TSS26986;
2	Cufflinks	exon	2544036	2544539	.	-	.	gene_id GRMZM2G009994; transcript_id TCONS_00013136; exon_number 1; oId CUFF.20413.1; tss_id TSS26986;
2	Cufflinks	exon	2544774	2544937	.	-	.	gene_id GRMZM2G009994; transcript_id TCONS_00013136; exon_number 2; oId CUFF.20413.1; tss_id TSS26986;
2	Cufflinks	exon	2545236	2545319	.	-	.	gene_id GRMZM2G009994; transcript_id TCONS_00013136; exon_number 3; oId CUFF.20413.1; tss_id TSS26986;
2	Cufflinks	exon	2545463	2545516	.	-	.	gene_id GRMZM2G009994; transcript_id TCONS_00013136; exon_number 4; oId CUFF.20413.1; tss_id TSS26986;
2	Cufflinks	exon	2545590	2545696	.	-	.	gene_id GRMZM2G009994; transcript_id TCONS_00013136; exon_number 5; oId CUFF.20413.1; tss_id TSS26986;
2	Cufflinks	exon	2545793	2545933	.	-	.	gene_id GRMZM2G009994; transcript_id TCONS_00013136; exon_number 6; oId CUFF.20413.1; tss_id TSS26986;

2	Cufflinks	exon	2546732	2546987	.	-	.	gene_id GRMZM2G009994; transcript_id TCONS_00013136; exon_number 7; oId CUFF.20413.1; tss_id TSS26986;
2	Cufflinks	exon	2547228	2547304	.	-	.	gene_id GRMZM2G009994; transcript_id TCONS_00013136; exon_number 8; oId CUFF.20413.1; tss_id TSS26986;
2	Cufflinks	exon	2547423	2547486	.	-	.	gene_id GRMZM2G009994; transcript_id TCONS_00013136; exon_number 9; oId CUFF.20413.1; tss_id TSS26986;
2	Cufflinks	exon	2548063	2548220	.	-	.	gene_id GRMZM2G009994; transcript_id TCONS_00013136; exon_number 10; oId CUFF.20413.1; tss_id TSS26986;
2	Cufflinks	exon	2549309	2549386	.	-	.	gene_id GRMZM2G009994; transcript_id TCONS_00013136; exon_number 11; oId CUFF.20413.1; tss_id TSS26986;
2	Cufflinks	exon	2549746	2550137	.	-	.	gene_id GRMZM2G009994; transcript_id TCONS_00013136; exon_number 12; oId CUFF.20413.1; tss_id TSS26986;
2	Cufflinks	exon	2550363	2550544	.	-	.	gene_id GRMZM2G009994; transcript_id TCONS_00013136; exon_number 13; oId CUFF.20413.1; tss_id TSS26986;
2	Cufflinks	exon	3018653	3019449	.	-	.	gene_id XLOC_011511; transcript_id TCONS_00013144; exon_number 1; oId CUFF.20421.4; tss_id TSS27005;
2	Cufflinks	exon	3019519	3020854	.	-	.	gene_id XLOC_011511; transcript_id TCONS_00013144; exon_number 2; oId CUFF.20421.4; tss_id TSS27005;
2	Cufflinks	exon	3018653	3019454	.	-	.	gene_id XLOC_011511; transcript_id TCONS_00013145; exon_number 1; oId CUFF.20421.2; tss_id TSS27005;
2	Cufflinks	exon	3019519	3020854	.	-	.	gene_id XLOC_011511; transcript_id TCONS_00013145; exon_number 2; oId CUFF.20421.2; tss_id TSS27005;
2	Cufflinks	exon	3192839	3193216	.	-	.	gene_id XLOC_011518; transcript_id TCONS_00013154; exon_number 1; oId CUFF.20432.1; tss_id TSS27022;
2	Cufflinks	exon	3978709	3979346	.	-	.	gene_id GRMZM2G056513; transcript_id TCONS_00013176; exon_number 1; oId CUFF.20533.1; tss_id TSS27065;
2	Cufflinks	exon	3979583	3979948	.	-	.	gene_id GRMZM2G056513; transcript_id TCONS_00013176; exon_number 2; oId CUFF.20533.1; tss_id TSS27065;
2	Cufflinks	exon	4333730	4334080	.	-	.	gene_id XLOC_011543; transcript_id TCONS_00013185; exon_number 1; oId CUFF.20573.1; tss_id TSS27076;
2	Cufflinks	exon	4852765	4853213	.	-	.	gene_id GRMZM2G032977; transcript_id TCONS_00013205; exon_number 1; oId CUFF.20631.1; tss_id TSS27105;
2	Cufflinks	exon	4853354	4853424	.	-	.	gene_id GRMZM2G032977; transcript_id TCONS_00013205; exon_number 2; oId CUFF.20631.1; tss_id TSS27105;
2	Cufflinks	exon	4853541	4853608	.	-	.	gene_id GRMZM2G032977; transcript_id TCONS_00013205; exon_number 3; oId CUFF.20631.1; tss_id TSS27105;
2	Cufflinks	exon	4853741	4853833	.	-	.	gene_id GRMZM2G032977; transcript_id TCONS_00013205; exon_number 4; oId CUFF.20631.1; tss_id TSS27105;
2	Cufflinks	exon	4853922	4854061	.	-	.	gene_id GRMZM2G032977; transcript_id TCONS_00013205; exon_number 5; oId CUFF.20631.1; tss_id TSS27105;
2	Cufflinks	exon	4854139	4854231	.	-	.	gene_id GRMZM2G032977; transcript_id TCONS_00013205; exon_number 6; oId CUFF.20631.1; tss_id TSS27105;
2	Cufflinks	exon	4855027	4855198	.	-	.	gene_id GRMZM2G032977; transcript_id TCONS_00013205; exon_number 7; oId CUFF.20631.1; tss_id TSS27105;
2	Cufflinks	exon	4855338	4855560	.	-	.	gene_id GRMZM2G032977; transcript_id TCONS_00013205; exon_number 8; oId CUFF.20631.1; tss_id TSS27105;
2	Cufflinks	exon	6642342	6643179	.	-	.	gene_id GRMZM5G829840; transcript_id TCONS_00013265; exon_number 1; oId CUFF.20803.2; tss_id TSS27201;
2	Cufflinks	exon	6645112	6645200	.	-	.	gene_id GRMZM5G829840; transcript_id TCONS_00013265; exon_number 2; oId CUFF.20803.2; tss_id TSS27201;
2	Cufflinks	exon	6645732	6646369	.	-	.	gene_id GRMZM5G829840; transcript_id TCONS_00013265; exon_number 3; oId CUFF.20803.2; tss_id TSS27201;
2	Cufflinks	exon	6642342	6643179	.	-	.	gene_id GRMZM5G829840; transcript_id TCONS_00013266; exon_number 1; oId CUFF.20803.1; tss_id TSS27201;
2	Cufflinks	exon	6645732	6646369	.	-	.	gene_id GRMZM5G829840; transcript_id TCONS_00013266; exon_number 2; oId CUFF.20803.1; tss_id TSS27201;
2	Cufflinks	exon	6988897	6989336	.	-	.	gene_id GRMZM2G106245; transcript_id TCONS_00013277; exon_number 1; oId CUFF.20829.1; tss_id TSS27214;
2	Cufflinks	exon	6991192	6991891	.	-	.	gene_id GRMZM2G106245; transcript_id TCONS_00013277; exon_number 2; oId CUFF.20829.1; tss_id TSS27214;
2	Cufflinks	exon	7047927	7049325	.	-	.	gene_id XLOC_011624; transcript_id TCONS_00013282; exon_number 1; oId CUFF.20855.3; tss_id TSS27220;
2	Cufflinks	exon	7050889	7051492	.	-	.	gene_id XLOC_011624; transcript_id TCONS_00013282; exon_number 2; oId CUFF.20855.3; tss_id TSS27220;
2	Cufflinks	exon	7527470	7528083	.	-	.	gene_id GRMZM2G164277; transcript_id TCONS_00013287; exon_number 1; oId CUFF.20880.1; tss_id TSS27230;
2	Cufflinks	exon	7528194	7528250	.	-	.	gene_id GRMZM2G164277; transcript_id TCONS_00013287; exon_number 2; oId CUFF.20880.1; tss_id TSS27230;
2	Cufflinks	exon	7528345	7528421	.	-	.	gene_id GRMZM2G164277; transcript_id TCONS_00013287; exon_number 3; oId CUFF.20880.1; tss_id TSS27230;
2	Cufflinks	exon	7528511	7528580	.	-	.	gene_id GRMZM2G164277; transcript_id TCONS_00013287; exon_number 4; oId CUFF.20880.1; tss_id TSS27230;

2	Cufflinks	exon	7528668	7528737	.	-	.	gene_id GRMZM2G164277; transcript_id TCONS_00013287; exon_number 5; oId CUFF.20880.1; tss_id TSS27230;
2	Cufflinks	exon	7528881	7528935	.	-	.	gene_id GRMZM2G164277; transcript_id TCONS_00013287; exon_number 6; oId CUFF.20880.1; tss_id TSS27230;
2	Cufflinks	exon	7529034	7529082	.	-	.	gene_id GRMZM2G164277; transcript_id TCONS_00013287; exon_number 7; oId CUFF.20880.1; tss_id TSS27230;
2	Cufflinks	exon	7529315	7529402	.	-	.	gene_id GRMZM2G164277; transcript_id TCONS_00013287; exon_number 8; oId CUFF.20880.1; tss_id TSS27230;
2	Cufflinks	exon	7529761	7529879	.	-	.	gene_id GRMZM2G164277; transcript_id TCONS_00013287; exon_number 9; oId CUFF.20880.1; tss_id TSS27230;
2	Cufflinks	exon	7530265	7530296	.	-	.	gene_id GRMZM2G164277; transcript_id TCONS_00013287; exon_number 10; oId CUFF.20880.1; tss_id TSS27230;
2	Cufflinks	exon	7530409	7530521	.	-	.	gene_id GRMZM2G164277; transcript_id TCONS_00013287; exon_number 11; oId CUFF.20880.1; tss_id TSS27230;
2	Cufflinks	exon	7530772	7531079	.	-	.	gene_id GRMZM2G164277; transcript_id TCONS_00013287; exon_number 12; oId CUFF.20880.1; tss_id TSS27230;
2	Cufflinks	exon	7531170	7531255	.	-	.	gene_id GRMZM2G164277; transcript_id TCONS_00013287; exon_number 13; oId CUFF.20880.1; tss_id TSS27230;
2	Cufflinks	exon	7531364	7531439	.	-	.	gene_id GRMZM2G164277; transcript_id TCONS_00013287; exon_number 14; oId CUFF.20880.1; tss_id TSS27230;
2	Cufflinks	exon	7531702	7531925	.	-	.	gene_id GRMZM2G164277; transcript_id TCONS_00013287; exon_number 15; oId CUFF.20880.1; tss_id TSS27230;
2	Cufflinks	exon	9551732	9552554	.	-	.	gene_id XLOC_011660; transcript_id TCONS_00013323; exon_number 1; oId CUFF.21006.1; tss_id TSS27295;
2	Cufflinks	exon	10348422	10348804	.	-	.	gene_id XLOC_011677; transcript_id TCONS_00013342; exon_number 1; oId CUFF.21108.1; tss_id TSS27329;
2	Cufflinks	exon	13554335	13554617	.	-	.	gene_id XLOC_011739; transcript_id TCONS_00013410; exon_number 1; oId CUFF.21382.1; tss_id TSS27486;
2	Cufflinks	exon	13554719	13555152	.	-	.	gene_id XLOC_011740; transcript_id TCONS_00013411; exon_number 1; oId CUFF.21383.1; tss_id TSS27487;
2	Cufflinks	exon	13715840	13716398	.	-	.	gene_id XLOC_011741; transcript_id TCONS_00013412; exon_number 1; oId CUFF.21368.1; tss_id TSS27492;
2	Cufflinks	exon	14548496	14548908	.	-	.	gene_id GRMZM2G314396; transcript_id TCONS_00013425; exon_number 1; oId CUFF.21472.1; tss_id TSS27530;
2	Cufflinks	exon	14550072	14550296	.	-	.	gene_id GRMZM2G314396; transcript_id TCONS_00013425; exon_number 2; oId CUFF.21472.1; tss_id TSS27530;
2	Cufflinks	exon	14550870	14551037	.	-	.	gene_id GRMZM2G314396; transcript_id TCONS_00013425; exon_number 3; oId CUFF.21472.1; tss_id TSS27530;
2	Cufflinks	exon	14551239	14551354	.	-	.	gene_id GRMZM2G314396; transcript_id TCONS_00013425; exon_number 4; oId CUFF.21472.1; tss_id TSS27530;
2	Cufflinks	exon	14551477	14551629	.	-	.	gene_id GRMZM2G314396; transcript_id TCONS_00013425; exon_number 5; oId CUFF.21472.1; tss_id TSS27530;
2	Cufflinks	exon	14551733	14551876	.	-	.	gene_id GRMZM2G314396; transcript_id TCONS_00013425; exon_number 6; oId CUFF.21472.1; tss_id TSS27530;
2	Cufflinks	exon	14552836	14554289	.	-	.	gene_id GRMZM2G314396; transcript_id TCONS_00013425; exon_number 7; oId CUFF.21472.1; tss_id TSS27530;
2	Cufflinks	exon	16654034	16656556	.	-	.	gene_id GRMZM2G080992; transcript_id TCONS_00013470; exon_number 1; oId CUFF.21592.1; tss_id TSS27613;
2	Cufflinks	exon	17085454	17091232	.	-	.	gene_id GRMZM2G379540; transcript_id TCONS_00013481; exon_number 1; oId CUFF.21623.1; tss_id TSS27625;
2	Cufflinks	exon	17091316	17091557	.	-	.	gene_id GRMZM2G379540; transcript_id TCONS_00013481; exon_number 2; oId CUFF.21623.1; tss_id TSS27625;
2	Cufflinks	exon	17091643	17091806	.	-	.	gene_id GRMZM2G379540; transcript_id TCONS_00013481; exon_number 3; oId CUFF.21623.1; tss_id TSS27625;
2	Cufflinks	exon	17091944	17092107	.	-	.	gene_id GRMZM2G379540; transcript_id TCONS_00013481; exon_number 4; oId CUFF.21623.1; tss_id TSS27625;
2	Cufflinks	exon	17092251	17092794	.	-	.	gene_id GRMZM2G379540; transcript_id TCONS_00013481; exon_number 5; oId CUFF.21623.1; tss_id TSS27625;
2	Cufflinks	exon	17972445	17972889	.	-	.	gene_id GRMZM2G145968; transcript_id TCONS_00013489; exon_number 1; oId CUFF.21660.1; tss_id TSS27642;
2	Cufflinks	exon	17972977	17973087	.	-	.	gene_id GRMZM2G145968; transcript_id TCONS_00013489; exon_number 2; oId CUFF.21660.1; tss_id TSS27642;
2	Cufflinks	exon	17979477	17979530	.	-	.	gene_id GRMZM2G145968; transcript_id TCONS_00013489; exon_number 3; oId CUFF.21660.1; tss_id TSS27642;
2	Cufflinks	exon	17979647	17979691	.	-	.	gene_id GRMZM2G145968; transcript_id TCONS_00013489; exon_number 4; oId CUFF.21660.1; tss_id TSS27642;
2	Cufflinks	exon	17979782	17979839	.	-	.	gene_id GRMZM2G145968; transcript_id TCONS_00013489; exon_number 5; oId CUFF.21660.1; tss_id TSS27642;
2	Cufflinks	exon	17979946	17980020	.	-	.	gene_id GRMZM2G145968; transcript_id TCONS_00013489; exon_number 6; oId CUFF.21660.1; tss_id TSS27642;
2	Cufflinks	exon	17980142	17980306	.	-	.	gene_id GRMZM2G145968; transcript_id TCONS_00013489; exon_number 7; oId CUFF.21660.1; tss_id TSS27642;

2	Cufflinks	exon	25437808	25438697	.	-	.	gene_id GRMZM2G108219; transcript_id TCONS_00013615; exon_number 1; oId CUFF.22143.1; tss_id TSS27878;
2	Cufflinks	exon	25438810	25438995	.	-	.	gene_id GRMZM2G108219; transcript_id TCONS_00013615; exon_number 2; oId CUFF.22143.1; tss_id TSS27878;
2	Cufflinks	exon	25439094	25439460	.	-	.	gene_id GRMZM2G108219; transcript_id TCONS_00013615; exon_number 3; oId CUFF.22143.1; tss_id TSS27878;
2	Cufflinks	exon	27501203	27501645	.	-	.	gene_id XLOC_011944; transcript_id TCONS_00013648; exon_number 1; oId CUFF.22244.1; tss_id TSS27955;
2	Cufflinks	exon	43419169	43419719	.	-	.	gene_id XLOC_012141; transcript_id TCONS_00013875; exon_number 1; oId CUFF.23048.1; tss_id TSS28390;
2	Cufflinks	exon	44830075	44831590	.	-	.	gene_id XLOC_012161; transcript_id TCONS_00013895; exon_number 1; oId CUFF.23094.1; tss_id TSS28429;
2	Cufflinks	exon	45069108	45069681	.	-	.	gene_id GRMZM2G127139; transcript_id TCONS_00013907; exon_number 1; oId CUFF.23138.2; tss_id TSS28446;
2	Cufflinks	exon	45069825	45069912	.	-	.	gene_id GRMZM2G127139; transcript_id TCONS_00013907; exon_number 2; oId CUFF.23138.2; tss_id TSS28446;
2	Cufflinks	exon	45070002	45070090	.	-	.	gene_id GRMZM2G127139; transcript_id TCONS_00013907; exon_number 3; oId CUFF.23138.2; tss_id TSS28446;
2	Cufflinks	exon	45070208	45070262	.	-	.	gene_id GRMZM2G127139; transcript_id TCONS_00013907; exon_number 4; oId CUFF.23138.2; tss_id TSS28446;
2	Cufflinks	exon	45070348	45070437	.	-	.	gene_id GRMZM2G127139; transcript_id TCONS_00013907; exon_number 5; oId CUFF.23138.2; tss_id TSS28446;
2	Cufflinks	exon	45070548	45070612	.	-	.	gene_id GRMZM2G127139; transcript_id TCONS_00013907; exon_number 6; oId CUFF.23138.2; tss_id TSS28446;
2	Cufflinks	exon	45070690	45070735	.	-	.	gene_id GRMZM2G127139; transcript_id TCONS_00013907; exon_number 7; oId CUFF.23138.2; tss_id TSS28446;
2	Cufflinks	exon	45070912	45071015	.	-	.	gene_id GRMZM2G127139; transcript_id TCONS_00013907; exon_number 8; oId CUFF.23138.2; tss_id TSS28446;
2	Cufflinks	exon	45071248	45071362	.	-	.	gene_id GRMZM2G127139; transcript_id TCONS_00013907; exon_number 9; oId CUFF.23138.2; tss_id TSS28446;
2	Cufflinks	exon	45071571	45071662	.	-	.	gene_id GRMZM2G127139; transcript_id TCONS_00013907; exon_number 10; oId CUFF.23138.2; tss_id TSS28446;
2	Cufflinks	exon	45071751	45071962	.	-	.	gene_id GRMZM2G127139; transcript_id TCONS_00013907; exon_number 11; oId CUFF.23138.2; tss_id TSS28446;
2	Cufflinks	exon	45072092	45072207	.	-	.	gene_id GRMZM2G127139; transcript_id TCONS_00013907; exon_number 12; oId CUFF.23138.2; tss_id TSS28446;
2	Cufflinks	exon	45072304	45072404	.	-	.	gene_id GRMZM2G127139; transcript_id TCONS_00013907; exon_number 13; oId CUFF.23138.2; tss_id TSS28446;
2	Cufflinks	exon	45072484	45072558	.	-	.	gene_id GRMZM2G127139; transcript_id TCONS_00013907; exon_number 14; oId CUFF.23138.2; tss_id TSS28446;
2	Cufflinks	exon	45072662	45072824	.	-	.	gene_id GRMZM2G127139; transcript_id TCONS_00013907; exon_number 15; oId CUFF.23138.2; tss_id TSS28446;
2	Cufflinks	exon	45077194	45078053	.	-	.	gene_id GRMZM2G127139; transcript_id TCONS_00013907; exon_number 16; oId CUFF.23138.2; tss_id TSS28446;
2	Cufflinks	exon	46620242	46621123	.	-	.	gene_id GRMZM2G028139; transcript_id TCONS_00013927; exon_number 1; oId CUFF.23237.1; tss_id TSS28477;
2	Cufflinks	exon	46621968	46622117	.	-	.	gene_id GRMZM2G028139; transcript_id TCONS_00013927; exon_number 2; oId CUFF.23237.1; tss_id TSS28477;
2	Cufflinks	exon	46622309	46623343	.	-	.	gene_id GRMZM2G028139; transcript_id TCONS_00013927; exon_number 3; oId CUFF.23237.1; tss_id TSS28477;
2	Cufflinks	exon	46630100	46630433	.	-	.	gene_id GRMZM2G028139; transcript_id TCONS_00013927; exon_number 4; oId CUFF.23237.1; tss_id TSS28477;
2	Cufflinks	exon	46631426	46631492	.	-	.	gene_id GRMZM2G028139; transcript_id TCONS_00013927; exon_number 5; oId CUFF.23237.1; tss_id TSS28477;
2	Cufflinks	exon	46631591	46631681	.	-	.	gene_id GRMZM2G028139; transcript_id TCONS_00013927; exon_number 6; oId CUFF.23237.1; tss_id TSS28477;
2	Cufflinks	exon	46631805	46632084	.	-	.	gene_id GRMZM2G028139; transcript_id TCONS_00013927; exon_number 7; oId CUFF.23237.1; tss_id TSS28477;
2	Cufflinks	exon	51867500	51868326	.	-	.	gene_id GRMZM2G131155; transcript_id TCONS_00013978; exon_number 1; oId CUFF.23421.1; tss_id TSS28606;
2	Cufflinks	exon	51868798	51869605	.	-	.	gene_id GRMZM2G131155; transcript_id TCONS_00013978; exon_number 2; oId CUFF.23421.1; tss_id TSS28606;
2	Cufflinks	exon	54244859	54245494	.	-	.	gene_id GRMZM2G069093; transcript_id TCONS_00014000; exon_number 1; oId CUFF.23490.1; tss_id TSS28654;
2	Cufflinks	exon	54245618	54245783	.	-	.	gene_id GRMZM2G069093; transcript_id TCONS_00014000; exon_number 2; oId CUFF.23490.1; tss_id TSS28654;
2	Cufflinks	exon	54245890	54246513	.	-	.	gene_id GRMZM2G069093; transcript_id TCONS_00014000; exon_number 3; oId CUFF.23490.1; tss_id TSS28654;
2	Cufflinks	exon	74719597	74720215	.	-	.	gene_id GRMZM2G027272; transcript_id TCONS_00014152; exon_number 1; oId CUFF.24087.2; tss_id TSS28959;
2	Cufflinks	exon	74721343	74721510	.	-	.	gene_id GRMZM2G027272; transcript_id TCONS_00014152; exon_number 2; oId CUFF.24087.2; tss_id TSS28959;

2	Cufflinks	exon	74721616	74722116	.	-	.	gene_id GRMZM2G027272; transcript_id TCONS_00014152; exon_number 3; oId CUFF.24087.2; tss_id TSS28959;
2	Cufflinks	exon	74723387	74723421	.	-	.	gene_id GRMZM2G027272; transcript_id TCONS_00014152; exon_number 4; oId CUFF.24087.2; tss_id TSS28959;
2	Cufflinks	exon	74724198	74724317	.	-	.	gene_id GRMZM2G027272; transcript_id TCONS_00014152; exon_number 5; oId CUFF.24087.2; tss_id TSS28959;
2	Cufflinks	exon	101895519	101896323	.	-	.	gene_id GRMZM5G803404; transcript_id TCONS_00014265; exon_number 1; oId CUFF.24769.1; tss_id TSS29342;
2	Cufflinks	exon	101896449	101896573	.	-	.	gene_id GRMZM5G803404; transcript_id TCONS_00014265; exon_number 2; oId CUFF.24769.1; tss_id TSS29342;
2	Cufflinks	exon	101896661	101896950	.	-	.	gene_id GRMZM5G803404; transcript_id TCONS_00014265; exon_number 3; oId CUFF.24769.1; tss_id TSS29342;
2	Cufflinks	exon	101897035	101897183	.	-	.	gene_id GRMZM5G803404; transcript_id TCONS_00014265; exon_number 4; oId CUFF.24769.1; tss_id TSS29342;
2	Cufflinks	exon	101897252	101898019	.	-	.	gene_id GRMZM5G803404; transcript_id TCONS_00014265; exon_number 5; oId CUFF.24769.1; tss_id TSS29342;
2	Cufflinks	exon	101898100	101898998	.	-	.	gene_id GRMZM5G803404; transcript_id TCONS_00014265; exon_number 6; oId CUFF.24769.1; tss_id TSS29342;
2	Cufflinks	exon	101899100	101899173	.	-	.	gene_id GRMZM5G803404; transcript_id TCONS_00014265; exon_number 7; oId CUFF.24769.1; tss_id TSS29342;
2	Cufflinks	exon	101899250	101899284	.	-	.	gene_id GRMZM5G803404; transcript_id TCONS_00014265; exon_number 8; oId CUFF.24769.1; tss_id TSS29342;
2	Cufflinks	exon	101899394	101899537	.	-	.	gene_id GRMZM5G803404; transcript_id TCONS_00014265; exon_number 9; oId CUFF.24769.1; tss_id TSS29342;
2	Cufflinks	exon	101899638	101899701	.	-	.	gene_id GRMZM5G803404; transcript_id TCONS_00014265; exon_number 10; oId CUFF.24769.1; tss_id TSS29342;
2	Cufflinks	exon	101899805	101900077	.	-	.	gene_id GRMZM5G803404; transcript_id TCONS_00014265; exon_number 11; oId CUFF.24769.1; tss_id TSS29342;
2	Cufflinks	exon	101900699	101900775	.	-	.	gene_id GRMZM5G803404; transcript_id TCONS_00014265; exon_number 12; oId CUFF.24769.1; tss_id TSS29342;
2	Cufflinks	exon	101900857	101901259	.	-	.	gene_id GRMZM5G803404; transcript_id TCONS_00014265; exon_number 13; oId CUFF.24769.1; tss_id TSS29342;
2	Cufflinks	exon	104805122	104806195	.	-	.	gene_id XLOC_012500; transcript_id TCONS_00014277; exon_number 1; oId CUFF.24759.1; tss_id TSS29373;
2	Cufflinks	exon	136743919	136745286	.	-	.	gene_id XLOC_012684; transcript_id TCONS_00014473; exon_number 1; oId CUFF.25616.1; tss_id TSS29849;
2	Cufflinks	exon	136745390	136745759	.	-	.	gene_id XLOC_012684; transcript_id TCONS_00014473; exon_number 2; oId CUFF.25616.1; tss_id TSS29849;
2	Cufflinks	exon	138609364	138610408	.	-	.	gene_id GRMZM2G146472; transcript_id TCONS_00014478; exon_number 1; oId CUFF.25691.1; tss_id TSS29874;
2	Cufflinks	exon	138610507	138610761	.	-	.	gene_id GRMZM2G146472; transcript_id TCONS_00014478; exon_number 2; oId CUFF.25691.1; tss_id TSS29874;
2	Cufflinks	exon	138611093	138611152	.	-	.	gene_id GRMZM2G146472; transcript_id TCONS_00014478; exon_number 3; oId CUFF.25691.1; tss_id TSS29874;
2	Cufflinks	exon	138611236	138611294	.	-	.	gene_id GRMZM2G146472; transcript_id TCONS_00014478; exon_number 4; oId CUFF.25691.1; tss_id TSS29874;
2	Cufflinks	exon	138611436	138611510	.	-	.	gene_id GRMZM2G146472; transcript_id TCONS_00014478; exon_number 5; oId CUFF.25691.1; tss_id TSS29874;
2	Cufflinks	exon	138611578	138611666	.	-	.	gene_id GRMZM2G146472; transcript_id TCONS_00014478; exon_number 6; oId CUFF.25691.1; tss_id TSS29874;
2	Cufflinks	exon	138611762	138611842	.	-	.	gene_id GRMZM2G146472; transcript_id TCONS_00014478; exon_number 7; oId CUFF.25691.1; tss_id TSS29874;
2	Cufflinks	exon	138612583	138612707	.	-	.	gene_id GRMZM2G146472; transcript_id TCONS_00014478; exon_number 8; oId CUFF.25691.1; tss_id TSS29874;
2	Cufflinks	exon	138613132	138613172	.	-	.	gene_id GRMZM2G146472; transcript_id TCONS_00014478; exon_number 9; oId CUFF.25691.1; tss_id TSS29874;
2	Cufflinks	exon	138619845	138620146	.	-	.	gene_id GRMZM2G146472; transcript_id TCONS_00014478; exon_number 10; oId CUFF.25691.1; tss_id TSS29874;
2	Cufflinks	exon	138609364	138610408	.	-	.	gene_id GRMZM2G146472; transcript_id TCONS_00014479; exon_number 1; oId CUFF.25691.3; tss_id TSS29874;
2	Cufflinks	exon	138610507	138610761	.	-	.	gene_id GRMZM2G146472; transcript_id TCONS_00014479; exon_number 2; oId CUFF.25691.3; tss_id TSS29874;
2	Cufflinks	exon	138611093	138611152	.	-	.	gene_id GRMZM2G146472; transcript_id TCONS_00014479; exon_number 3; oId CUFF.25691.3; tss_id TSS29874;
2	Cufflinks	exon	138611236	138611294	.	-	.	gene_id GRMZM2G146472; transcript_id TCONS_00014479; exon_number 4; oId CUFF.25691.3; tss_id TSS29874;
2	Cufflinks	exon	138611436	138611510	.	-	.	gene_id GRMZM2G146472; transcript_id TCONS_00014479; exon_number 5; oId CUFF.25691.3; tss_id TSS29874;
2	Cufflinks	exon	138611578	138611666	.	-	.	gene_id GRMZM2G146472; transcript_id TCONS_00014479; exon_number 6; oId CUFF.25691.3; tss_id TSS29874;
2	Cufflinks	exon	138611762	138611842	.	-	.	gene_id GRMZM2G146472; transcript_id TCONS_00014479; exon_number 7; oId CUFF.25691.3; tss_id TSS29874;

2	Cufflinks	exon	138612583	138612707	.	-	.	gene_id GRMZM2G146472; transcript_id TCONS_00014479; exon_number 8; oId CUFF.25691.3; tss_id TSS29874;
2	Cufflinks	exon	138619845	138620146	.	-	.	gene_id GRMZM2G146472; transcript_id TCONS_00014479; exon_number 9; oId CUFF.25691.3; tss_id TSS29874;
2	Cufflinks	exon	157924539	157924880	.	-	.	gene_id GRMZM2G365035; transcript_id TCONS_00014612; exon_number 1; oId CUFF.26211.1; tss_id TSS30159;
2	Cufflinks	exon	157925047	157925220	.	-	.	gene_id GRMZM2G365035; transcript_id TCONS_00014612; exon_number 2; oId CUFF.26211.1; tss_id TSS30159;
2	Cufflinks	exon	157925619	157925681	.	-	.	gene_id GRMZM2G365035; transcript_id TCONS_00014612; exon_number 3; oId CUFF.26211.1; tss_id TSS30159;
2	Cufflinks	exon	157926509	157926616	.	-	.	gene_id GRMZM2G365035; transcript_id TCONS_00014612; exon_number 4; oId CUFF.26211.1; tss_id TSS30159;
2	Cufflinks	exon	157926707	157926822	.	-	.	gene_id GRMZM2G365035; transcript_id TCONS_00014612; exon_number 5; oId CUFF.26211.1; tss_id TSS30159;
2	Cufflinks	exon	157927536	157927662	.	-	.	gene_id GRMZM2G365035; transcript_id TCONS_00014612; exon_number 6; oId CUFF.26211.1; tss_id TSS30159;
2	Cufflinks	exon	157929159	157929328	.	-	.	gene_id GRMZM2G365035; transcript_id TCONS_00014612; exon_number 7; oId CUFF.26211.1; tss_id TSS30159;
2	Cufflinks	exon	157929440	157929518	.	-	.	gene_id GRMZM2G365035; transcript_id TCONS_00014612; exon_number 8; oId CUFF.26211.1; tss_id TSS30159;
2	Cufflinks	exon	157929616	157929727	.	-	.	gene_id GRMZM2G365035; transcript_id TCONS_00014612; exon_number 9; oId CUFF.26211.1; tss_id TSS30159;
2	Cufflinks	exon	157930346	157930387	.	-	.	gene_id GRMZM2G365035; transcript_id TCONS_00014612; exon_number 10; oId CUFF.26211.1; tss_id TSS30159;
2	Cufflinks	exon	157930814	157930944	.	-	.	gene_id GRMZM2G365035; transcript_id TCONS_00014612; exon_number 11; oId CUFF.26211.1; tss_id TSS30159;
2	Cufflinks	exon	157931865	157932534	.	-	.	gene_id GRMZM2G365035; transcript_id TCONS_00014612; exon_number 12; oId CUFF.26211.1; tss_id TSS30159;
2	Cufflinks	exon	159085004	159085643	.	-	.	gene_id XLOC_012822; transcript_id TCONS_00014625; exon_number 1; oId CUFF.26241.1; tss_id TSS30184;
2	Cufflinks	exon	169189856	169190340	.	-	.	gene_id XLOC_012902; transcript_id TCONS_00014713; exon_number 1; oId CUFF.26590.1; tss_id TSS30357;
2	Cufflinks	exon	176669016	176670075	.	-	.	gene_id GRMZM2G037150; transcript_id TCONS_00014782; exon_number 1; oId CUFF.26914.1; tss_id TSS30518;
2	Cufflinks	exon	176670166	176670285	.	-	.	gene_id GRMZM2G037150; transcript_id TCONS_00014782; exon_number 2; oId CUFF.26914.1; tss_id TSS30518;
2	Cufflinks	exon	176670457	176670588	.	-	.	gene_id GRMZM2G037150; transcript_id TCONS_00014782; exon_number 3; oId CUFF.26914.1; tss_id TSS30518;
2	Cufflinks	exon	176673102	176673281	.	-	.	gene_id GRMZM2G037150; transcript_id TCONS_00014782; exon_number 4; oId CUFF.26914.1; tss_id TSS30518;
2	Cufflinks	exon	177562929	177563476	.	-	.	gene_id XLOC_012976; transcript_id TCONS_00014791; exon_number 1; oId CUFF.26933.1; tss_id TSS30536;
2	Cufflinks	exon	178670884	178671554	.	-	.	gene_id XLOC_012986; transcript_id TCONS_00014805; exon_number 1; oId CUFF.26994.1; tss_id TSS30563;
2	Cufflinks	exon	181476797	181477422	.	-	.	gene_id XLOC_013019; transcript_id TCONS_00014840; exon_number 1; oId CUFF.27102.1; tss_id TSS30633;
2	Cufflinks	exon	183846951	183847432	.	-	.	gene_id XLOC_013031; transcript_id TCONS_00014852; exon_number 1; oId CUFF.27170.1; tss_id TSS30664;
2	Cufflinks	exon	187110216	187110887	.	-	.	gene_id XLOC_013079; transcript_id TCONS_00014905; exon_number 1; oId CUFF.27327.1; tss_id TSS30756;
2	Cufflinks	exon	192310632	192311327	.	-	.	gene_id GRMZM2G013892; transcript_id TCONS_00014982; exon_number 1; oId CUFF.27642.4; tss_id TSS30891;
2	Cufflinks	exon	192311654	192311727	.	-	.	gene_id GRMZM2G013892; transcript_id TCONS_00014982; exon_number 2; oId CUFF.27642.4; tss_id TSS30891;
2	Cufflinks	exon	192312155	192312236	.	-	.	gene_id GRMZM2G013892; transcript_id TCONS_00014982; exon_number 3; oId CUFF.27642.4; tss_id TSS30891;
2	Cufflinks	exon	192312457	192312715	.	-	.	gene_id GRMZM2G013892; transcript_id TCONS_00014982; exon_number 4; oId CUFF.27642.4; tss_id TSS30891;
2	Cufflinks	exon	192312958	192313168	.	-	.	gene_id GRMZM2G013892; transcript_id TCONS_00014982; exon_number 5; oId CUFF.27642.4; tss_id TSS30891;
2	Cufflinks	exon	192310632	192311327	.	-	.	gene_id GRMZM2G013892; transcript_id TCONS_00014983; exon_number 1; oId CUFF.27642.1; tss_id TSS30891;
2	Cufflinks	exon	192311654	192311727	.	-	.	gene_id GRMZM2G013892; transcript_id TCONS_00014983; exon_number 2; oId CUFF.27642.1; tss_id TSS30891;
2	Cufflinks	exon	192312155	192312224	.	-	.	gene_id GRMZM2G013892; transcript_id TCONS_00014983; exon_number 3; oId CUFF.27642.1; tss_id TSS30891;
2	Cufflinks	exon	192312457	192312715	.	-	.	gene_id GRMZM2G013892; transcript_id TCONS_00014983; exon_number 4; oId CUFF.27642.1; tss_id TSS30891;
2	Cufflinks	exon	192312958	192313184	.	-	.	gene_id GRMZM2G013892; transcript_id TCONS_00014983; exon_number 5; oId CUFF.27642.1; tss_id TSS30891;
2	Cufflinks	exon	196730804	196731019	.	-	.	gene_id GRMZM2G050714; transcript_id TCONS_00015037; exon_number 1; oId CUFF.27969.4; tss_id TSS31016;

2	Cufflinks	exon	196731120	196731194	.	-	.	gene_id GRMZM2G050714; transcript_id TCONS_00015037; exon_number 2; oId CUFF.27969.4; tss_id TSS31016;
2	Cufflinks	exon	196731286	196731351	.	-	.	gene_id GRMZM2G050714; transcript_id TCONS_00015037; exon_number 3; oId CUFF.27969.4; tss_id TSS31016;
2	Cufflinks	exon	196731709	196731762	.	-	.	gene_id GRMZM2G050714; transcript_id TCONS_00015037; exon_number 4; oId CUFF.27969.4; tss_id TSS31016;
2	Cufflinks	exon	196731848	196731979	.	-	.	gene_id GRMZM2G050714; transcript_id TCONS_00015037; exon_number 5; oId CUFF.27969.4; tss_id TSS31016;
2	Cufflinks	exon	196732079	196732140	.	-	.	gene_id GRMZM2G050714; transcript_id TCONS_00015037; exon_number 6; oId CUFF.27969.4; tss_id TSS31016;
2	Cufflinks	exon	196732247	196732323	.	-	.	gene_id GRMZM2G050714; transcript_id TCONS_00015037; exon_number 7; oId CUFF.27969.4; tss_id TSS31016;
2	Cufflinks	exon	196732426	196732495	.	-	.	gene_id GRMZM2G050714; transcript_id TCONS_00015037; exon_number 8; oId CUFF.27969.4; tss_id TSS31016;
2	Cufflinks	exon	196733336	196733448	.	-	.	gene_id GRMZM2G050714; transcript_id TCONS_00015037; exon_number 9; oId CUFF.27969.4; tss_id TSS31016;
2	Cufflinks	exon	196733542	196733606	.	-	.	gene_id GRMZM2G050714; transcript_id TCONS_00015037; exon_number 10; oId CUFF.27969.4; tss_id TSS31016;
2	Cufflinks	exon	196733714	196733779	.	-	.	gene_id GRMZM2G050714; transcript_id TCONS_00015037; exon_number 11; oId CUFF.27969.4; tss_id TSS31016;
2	Cufflinks	exon	196734288	196734606	.	-	.	gene_id GRMZM2G050714; transcript_id TCONS_00015037; exon_number 12; oId CUFF.27969.4; tss_id TSS31016;
2	Cufflinks	exon	198178361	198178755	.	-	.	gene_id GRMZM2G312501; transcript_id TCONS_00015057; exon_number 1; oId CUFF.27958.2; tss_id TSS31077;
2	Cufflinks	exon	198178861	198179380	.	-	.	gene_id GRMZM2G312501; transcript_id TCONS_00015057; exon_number 2; oId CUFF.27958.2; tss_id TSS31077;
2	Cufflinks	exon	198179508	198179662	.	-	.	gene_id GRMZM2G312501; transcript_id TCONS_00015057; exon_number 3; oId CUFF.27958.2; tss_id TSS31077;
2	Cufflinks	exon	198179800	198180190	.	-	.	gene_id GRMZM2G312501; transcript_id TCONS_00015057; exon_number 4; oId CUFF.27958.2; tss_id TSS31077;
2	Cufflinks	exon	198180276	198180344	.	-	.	gene_id GRMZM2G312501; transcript_id TCONS_00015057; exon_number 5; oId CUFF.27958.2; tss_id TSS31077;
2	Cufflinks	exon	198181348	198181446	.	-	.	gene_id GRMZM2G312501; transcript_id TCONS_00015057; exon_number 6; oId CUFF.27958.2; tss_id TSS31077;
2	Cufflinks	exon	198181543	198181588	.	-	.	gene_id GRMZM2G312501; transcript_id TCONS_00015057; exon_number 7; oId CUFF.27958.2; tss_id TSS31077;
2	Cufflinks	exon	198181680	198181755	.	-	.	gene_id GRMZM2G312501; transcript_id TCONS_00015057; exon_number 8; oId CUFF.27958.2; tss_id TSS31077;
2	Cufflinks	exon	198181948	198181984	.	-	.	gene_id GRMZM2G312501; transcript_id TCONS_00015057; exon_number 9; oId CUFF.27958.2; tss_id TSS31077;
2	Cufflinks	exon	198182580	198183017	.	-	.	gene_id GRMZM2G312501; transcript_id TCONS_00015057; exon_number 10; oId CUFF.27958.2; tss_id TSS31077;
2	Cufflinks	exon	198178361	198178755	.	-	.	gene_id GRMZM2G312501; transcript_id TCONS_00015058; exon_number 1; oId CUFF.27958.3; tss_id TSS31077;
2	Cufflinks	exon	198178861	198179380	.	-	.	gene_id GRMZM2G312501; transcript_id TCONS_00015058; exon_number 2; oId CUFF.27958.3; tss_id TSS31077;
2	Cufflinks	exon	198179508	198179662	.	-	.	gene_id GRMZM2G312501; transcript_id TCONS_00015058; exon_number 3; oId CUFF.27958.3; tss_id TSS31077;
2	Cufflinks	exon	198179800	198180190	.	-	.	gene_id GRMZM2G312501; transcript_id TCONS_00015058; exon_number 4; oId CUFF.27958.3; tss_id TSS31077;
2	Cufflinks	exon	198180276	198180344	.	-	.	gene_id GRMZM2G312501; transcript_id TCONS_00015058; exon_number 5; oId CUFF.27958.3; tss_id TSS31077;
2	Cufflinks	exon	198181348	198181446	.	-	.	gene_id GRMZM2G312501; transcript_id TCONS_00015058; exon_number 6; oId CUFF.27958.3; tss_id TSS31077;
2	Cufflinks	exon	198181543	198181588	.	-	.	gene_id GRMZM2G312501; transcript_id TCONS_00015058; exon_number 7; oId CUFF.27958.3; tss_id TSS31077;
2	Cufflinks	exon	198181680	198181755	.	-	.	gene_id GRMZM2G312501; transcript_id TCONS_00015058; exon_number 8; oId CUFF.27958.3; tss_id TSS31077;
2	Cufflinks	exon	198181948	198181987	.	-	.	gene_id GRMZM2G312501; transcript_id TCONS_00015058; exon_number 9; oId CUFF.27958.3; tss_id TSS31077;
2	Cufflinks	exon	198182580	198183070	.	-	.	gene_id GRMZM2G312501; transcript_id TCONS_00015058; exon_number 10; oId CUFF.27958.3; tss_id TSS31077;
2	Cufflinks	exon	208504875	208505919	.	-	.	gene_id GRMZM2G130854; transcript_id TCONS_00015247; exon_number 1; oId CUFF.28698.1; tss_id TSS31441;
2	Cufflinks	exon	208507043	208507201	.	-	.	gene_id GRMZM2G130854; transcript_id TCONS_00015247; exon_number 2; oId CUFF.28698.1; tss_id TSS31441;
2	Cufflinks	exon	208507292	208507454	.	-	.	gene_id GRMZM2G130854; transcript_id TCONS_00015247; exon_number 3; oId CUFF.28698.1; tss_id TSS31441;
2	Cufflinks	exon	208507726	208508266	.	-	.	gene_id GRMZM2G130854; transcript_id TCONS_00015247; exon_number 4; oId CUFF.28698.1; tss_id TSS31441;
2	Cufflinks	exon	208509760	208509927	.	-	.	gene_id GRMZM2G130854; transcript_id TCONS_00015247; exon_number 5; oId CUFF.28698.1; tss_id TSS31441;

2	Cufflinks	exon	208510960	208511227	.	-	.	gene_id GRMZM2G130854; transcript_id TCONS_00015247; exon_number 6; oId CUFF.28698.1; tss_id TSS31441;
2	Cufflinks	exon	209675368	209676189	.	-	.	gene_id GRMZM2G376432; transcript_id TCONS_00015259; exon_number 1; oId CUFF.28739.1; tss_id TSS31472;
2	Cufflinks	exon	209676558	209676655	.	-	.	gene_id GRMZM2G376432; transcript_id TCONS_00015259; exon_number 2; oId CUFF.28739.1; tss_id TSS31472;
2	Cufflinks	exon	209676742	209676843	.	-	.	gene_id GRMZM2G376432; transcript_id TCONS_00015259; exon_number 3; oId CUFF.28739.1; tss_id TSS31472;
2	Cufflinks	exon	209677149	209677270	.	-	.	gene_id GRMZM2G376432; transcript_id TCONS_00015259; exon_number 4; oId CUFF.28739.1; tss_id TSS31472;
2	Cufflinks	exon	209678100	209678600	.	-	.	gene_id GRMZM2G376432; transcript_id TCONS_00015259; exon_number 5; oId CUFF.28739.1; tss_id TSS31472;
2	Cufflinks	exon	209789137	209791372	.	-	.	gene_id GRMZM2G079082; transcript_id TCONS_00015265; exon_number 1; oId CUFF.28745.2; tss_id TSS31479;
2	Cufflinks	exon	209791919	209793396	.	-	.	gene_id GRMZM2G079082; transcript_id TCONS_00015265; exon_number 2; oId CUFF.28745.2; tss_id TSS31479;
2	Cufflinks	exon	213830523	213830956	.	-	.	gene_id GRMZM2G075002; transcript_id TCONS_00015341; exon_number 1; oId CUFF.29038.1; tss_id TSS31625;
2	Cufflinks	exon	213831253	213831327	.	-	.	gene_id GRMZM2G075002; transcript_id TCONS_00015341; exon_number 2; oId CUFF.29038.1; tss_id TSS31625;
2	Cufflinks	exon	213831468	213831539	.	-	.	gene_id GRMZM2G075002; transcript_id TCONS_00015341; exon_number 3; oId CUFF.29038.1; tss_id TSS31625;
2	Cufflinks	exon	213831635	213831751	.	-	.	gene_id GRMZM2G075002; transcript_id TCONS_00015341; exon_number 4; oId CUFF.29038.1; tss_id TSS31625;
2	Cufflinks	exon	213832095	213832196	.	-	.	gene_id GRMZM2G075002; transcript_id TCONS_00015341; exon_number 5; oId CUFF.29038.1; tss_id TSS31625;
2	Cufflinks	exon	213832296	213832508	.	-	.	gene_id GRMZM2G075002; transcript_id TCONS_00015341; exon_number 6; oId CUFF.29038.1; tss_id TSS31625;
2	Cufflinks	exon	213832607	213832732	.	-	.	gene_id GRMZM2G075002; transcript_id TCONS_00015341; exon_number 7; oId CUFF.29038.1; tss_id TSS31625;
2	Cufflinks	exon	213832865	213832918	.	-	.	gene_id GRMZM2G075002; transcript_id TCONS_00015341; exon_number 8; oId CUFF.29038.1; tss_id TSS31625;
2	Cufflinks	exon	213833009	213833089	.	-	.	gene_id GRMZM2G075002; transcript_id TCONS_00015341; exon_number 9; oId CUFF.29038.1; tss_id TSS31625;
2	Cufflinks	exon	213833193	213833300	.	-	.	gene_id GRMZM2G075002; transcript_id TCONS_00015341; exon_number 10; oId CUFF.29038.1; tss_id TSS31625;
2	Cufflinks	exon	213833402	213833473	.	-	.	gene_id GRMZM2G075002; transcript_id TCONS_00015341; exon_number 11; oId CUFF.29038.1; tss_id TSS31625;
2	Cufflinks	exon	213834689	213834751	.	-	.	gene_id GRMZM2G075002; transcript_id TCONS_00015341; exon_number 12; oId CUFF.29038.1; tss_id TSS31625;
2	Cufflinks	exon	213834848	213835155	.	-	.	gene_id GRMZM2G075002; transcript_id TCONS_00015341; exon_number 13; oId CUFF.29038.1; tss_id TSS31625;
2	Cufflinks	exon	215949546	215952234	.	-	.	gene_id XLOC_013492; transcript_id TCONS_00015385; exon_number 1; oId CUFF.29195.1; tss_id TSS31711;
2	Cufflinks	exon	215953968	215955328	.	-	.	gene_id XLOC_013493; transcript_id TCONS_00015386; exon_number 1; oId CUFF.29197.1; tss_id TSS31713;
2	Cufflinks	exon	219256203	219258554	.	-	.	gene_id GRMZM2G140667; transcript_id TCONS_00015445; exon_number 1; oId CUFF.29448.1; tss_id TSS31831;
2	Cufflinks	exon	219258645	219258703	.	-	.	gene_id GRMZM2G140667; transcript_id TCONS_00015445; exon_number 2; oId CUFF.29448.1; tss_id TSS31831;
2	Cufflinks	exon	219258783	219258885	.	-	.	gene_id GRMZM2G140667; transcript_id TCONS_00015445; exon_number 3; oId CUFF.29448.1; tss_id TSS31831;
2	Cufflinks	exon	219258953	219259032	.	-	.	gene_id GRMZM2G140667; transcript_id TCONS_00015445; exon_number 4; oId CUFF.29448.1; tss_id TSS31831;
2	Cufflinks	exon	219259107	219259192	.	-	.	gene_id GRMZM2G140667; transcript_id TCONS_00015445; exon_number 5; oId CUFF.29448.1; tss_id TSS31831;
2	Cufflinks	exon	219259544	219259592	.	-	.	gene_id GRMZM2G140667; transcript_id TCONS_00015445; exon_number 6; oId CUFF.29448.1; tss_id TSS31831;
2	Cufflinks	exon	219260351	219260416	.	-	.	gene_id GRMZM2G140667; transcript_id TCONS_00015445; exon_number 7; oId CUFF.29448.1; tss_id TSS31831;
2	Cufflinks	exon	219260510	219260684	.	-	.	gene_id GRMZM2G140667; transcript_id TCONS_00015445; exon_number 8; oId CUFF.29448.1; tss_id TSS31831;
2	Cufflinks	exon	219260783	219261002	.	-	.	gene_id GRMZM2G140667; transcript_id TCONS_00015445; exon_number 9; oId CUFF.29448.1; tss_id TSS31831;
2	Cufflinks	exon	219606971	219607483	.	-	.	gene_id XLOC_013550; transcript_id TCONS_00015449; exon_number 1; oId CUFF.29452.1; tss_id TSS31839;
2	Cufflinks	exon	221314265	221314378	.	-	.	gene_id XLOC_013568; transcript_id TCONS_00015468; exon_number 1; oId CUFF.29544.1; tss_id TSS31874;
2	Cufflinks	exon	221314586	221314620	.	-	.	gene_id XLOC_013568; transcript_id TCONS_00015468; exon_number 2; oId CUFF.29544.1; tss_id TSS31874;
2	Cufflinks	exon	221314728	221316032	.	-	.	gene_id XLOC_013568; transcript_id TCONS_00015468; exon_number 3; oId CUFF.29544.1; tss_id TSS31874;

2	Cufflinks	exon	232655940	232657533	.	-	.	gene_id GRMZM2G053206; transcript_id TCONS_00015620; exon_number 1; oId CUFF.30179.1; tss_id TSS32180;
3	Cufflinks	exon	655360	657996	.	+	.	gene_id XLOC_013797; transcript_id TCONS_00015729; exon_number 1; oId CUFF.30575.1; tss_id TSS32386;
3	Cufflinks	exon	7939013	7940694	.	+	.	gene_id XLOC_013910; transcript_id TCONS_00015861; exon_number 1; oId CUFF.31087.1; tss_id TSS32627;
3	Cufflinks	exon	8220468	8221022	.	+	.	gene_id XLOC_013917; transcript_id TCONS_00015874; exon_number 1; oId CUFF.31130.1; tss_id TSS32644;
3	Cufflinks	exon	15137688	15138387	.	+	.	gene_id GRMZM2G452564; transcript_id TCONS_00016021; exon_number 1; oId CUFF.31561.1; tss_id TSS32890;
3	Cufflinks	exon	15138543	15139240	.	+	.	gene_id GRMZM2G452564; transcript_id TCONS_00016021; exon_number 2; oId CUFF.31561.1; tss_id TSS32890;
3	Cufflinks	exon	19973505	19974270	.	+	.	gene_id XLOC_014117; transcript_id TCONS_00016105; exon_number 1; oId CUFF.31807.1; tss_id TSS33016;
3	Cufflinks	exon	26258962	26259354	.	+	.	gene_id GRMZM2G090584; transcript_id TCONS_00016162; exon_number 1; oId CUFF.32046.1; tss_id TSS33143;
3	Cufflinks	exon	26259500	26259592	.	+	.	gene_id GRMZM2G090584; transcript_id TCONS_00016162; exon_number 2; oId CUFF.32046.1; tss_id TSS33143;
3	Cufflinks	exon	26259739	26259864	.	+	.	gene_id GRMZM2G090584; transcript_id TCONS_00016162; exon_number 3; oId CUFF.32046.1; tss_id TSS33143;
3	Cufflinks	exon	26260084	26261458	.	+	.	gene_id GRMZM2G090584; transcript_id TCONS_00016162; exon_number 4; oId CUFF.32046.1; tss_id TSS33143;
3	Cufflinks	exon	36311860	36313187	.	+	.	gene_id XLOC_014230; transcript_id TCONS_00016230; exon_number 1; oId CUFF.32409.1; tss_id TSS33287;
3	Cufflinks	exon	41742767	41744902	.	+	.	gene_id XLOC_014266; transcript_id TCONS_00016269; exon_number 1; oId CUFF.32592.1; tss_id TSS33367;
3	Cufflinks	exon	52448552	52448884	.	+	.	gene_id GRMZM2G363908; transcript_id TCONS_00016337; exon_number 1; oId CUFF.32909.1; tss_id TSS33530;
3	Cufflinks	exon	52448967	52449118	.	+	.	gene_id GRMZM2G363908; transcript_id TCONS_00016337; exon_number 2; oId CUFF.32909.1; tss_id TSS33530;
3	Cufflinks	exon	52451390	52452420	.	+	.	gene_id GRMZM2G363908; transcript_id TCONS_00016337; exon_number 3; oId CUFF.32909.1; tss_id TSS33530;
3	Cufflinks	exon	66708373	66708927	.	+	.	gene_id XLOC_014417; transcript_id TCONS_00016440; exon_number 1; oId CUFF.33248.1; tss_id TSS33764;
3	Cufflinks	exon	95281733	95282364	.	+	.	gene_id GRMZM2G017835; transcript_id TCONS_00016552; exon_number 1; oId CUFF.33897.1; tss_id TSS34115;
3	Cufflinks	exon	95282487	95282692	.	+	.	gene_id GRMZM2G017835; transcript_id TCONS_00016552; exon_number 2; oId CUFF.33897.1; tss_id TSS34115;
3	Cufflinks	exon	95282805	95282874	.	+	.	gene_id GRMZM2G017835; transcript_id TCONS_00016552; exon_number 3; oId CUFF.33897.1; tss_id TSS34115;
3	Cufflinks	exon	95283077	95283132	.	+	.	gene_id GRMZM2G017835; transcript_id TCONS_00016552; exon_number 4; oId CUFF.33897.1; tss_id TSS34115;
3	Cufflinks	exon	95283244	95283315	.	+	.	gene_id GRMZM2G017835; transcript_id TCONS_00016552; exon_number 5; oId CUFF.33897.1; tss_id TSS34115;
3	Cufflinks	exon	95283400	95283494	.	+	.	gene_id GRMZM2G017835; transcript_id TCONS_00016552; exon_number 6; oId CUFF.33897.1; tss_id TSS34115;
3	Cufflinks	exon	95283634	95283700	.	+	.	gene_id GRMZM2G017835; transcript_id TCONS_00016552; exon_number 7; oId CUFF.33897.1; tss_id TSS34115;
3	Cufflinks	exon	95283803	95283932	.	+	.	gene_id GRMZM2G017835; transcript_id TCONS_00016552; exon_number 8; oId CUFF.33897.1; tss_id TSS34115;
3	Cufflinks	exon	95284040	95284258	.	+	.	gene_id GRMZM2G017835; transcript_id TCONS_00016552; exon_number 9; oId CUFF.33897.1; tss_id TSS34115;
3	Cufflinks	exon	95284365	95284489	.	+	.	gene_id GRMZM2G017835; transcript_id TCONS_00016552; exon_number 10; oId CUFF.33897.1; tss_id TSS34115;
3	Cufflinks	exon	95284864	95285038	.	+	.	gene_id GRMZM2G017835; transcript_id TCONS_00016552; exon_number 11; oId CUFF.33897.1; tss_id TSS34115;
3	Cufflinks	exon	95285119	95285209	.	+	.	gene_id GRMZM2G017835; transcript_id TCONS_00016552; exon_number 12; oId CUFF.33897.1; tss_id TSS34115;
3	Cufflinks	exon	95285795	95285952	.	+	.	gene_id GRMZM2G017835; transcript_id TCONS_00016552; exon_number 13; oId CUFF.33897.1; tss_id TSS34115;
3	Cufflinks	exon	95286420	95286608	.	+	.	gene_id GRMZM2G017835; transcript_id TCONS_00016552; exon_number 14; oId CUFF.33897.1; tss_id TSS34115;
3	Cufflinks	exon	95286684	95287244	.	+	.	gene_id GRMZM2G017835; transcript_id TCONS_00016552; exon_number 15; oId CUFF.33897.1; tss_id TSS34115;
3	Cufflinks	exon	109717705	109718299	.	+	.	gene_id XLOC_014550; transcript_id TCONS_00016588; exon_number 1; oId CUFF.34210.1; tss_id TSS34261;
3	Cufflinks	exon	119994992	119997548	.	+	.	gene_id XLOC_014607; transcript_id TCONS_00016654; exon_number 1; oId CUFF.34484.1; tss_id TSS34393;
3	Cufflinks	exon	127876049	127877357	.	+	.	gene_id XLOC_014644; transcript_id TCONS_00016701; exon_number 1; oId CUFF.34699.1; tss_id TSS34501;
3	Cufflinks	exon	129109128	129109343	.	+	.	gene_id GRMZM2G046061; transcript_id TCONS_00016716; exon_number 1; oId CUFF.34751.1; tss_id TSS34531;

3	Cufflinks	exon	129109467	129109542	.	+	.	gene_id GRMZM2G046061; transcript_id TCONS_00016716; exon_number 2; oId CUFF.34751.1; tss_id TSS34531;
3	Cufflinks	exon	129109665	129109823	.	+	.	gene_id GRMZM2G046061; transcript_id TCONS_00016716; exon_number 3; oId CUFF.34751.1; tss_id TSS34531;
3	Cufflinks	exon	129109970	129111686	.	+	.	gene_id GRMZM2G046061; transcript_id TCONS_00016716; exon_number 4; oId CUFF.34751.1; tss_id TSS34531;
3	Cufflinks	exon	133812942	133813453	.	+	.	gene_id GRMZM2G155974; transcript_id TCONS_00016753; exon_number 1; oId CUFF.34930.2; tss_id TSS34607;
3	Cufflinks	exon	133813826	133813995	.	+	.	gene_id GRMZM2G155974; transcript_id TCONS_00016753; exon_number 2; oId CUFF.34930.2; tss_id TSS34607;
3	Cufflinks	exon	133814302	133814377	.	+	.	gene_id GRMZM2G155974; transcript_id TCONS_00016753; exon_number 3; oId CUFF.34930.2; tss_id TSS34607;
3	Cufflinks	exon	133814631	133814761	.	+	.	gene_id GRMZM2G155974; transcript_id TCONS_00016753; exon_number 4; oId CUFF.34930.2; tss_id TSS34607;
3	Cufflinks	exon	133815549	133815671	.	+	.	gene_id GRMZM2G155974; transcript_id TCONS_00016753; exon_number 5; oId CUFF.34930.2; tss_id TSS34607;
3	Cufflinks	exon	133815761	133815841	.	+	.	gene_id GRMZM2G155974; transcript_id TCONS_00016753; exon_number 6; oId CUFF.34930.2; tss_id TSS34607;
3	Cufflinks	exon	133816125	133816205	.	+	.	gene_id GRMZM2G155974; transcript_id TCONS_00016753; exon_number 7; oId CUFF.34930.2; tss_id TSS34607;
3	Cufflinks	exon	133816351	133816420	.	+	.	gene_id GRMZM2G155974; transcript_id TCONS_00016753; exon_number 8; oId CUFF.34930.2; tss_id TSS34607;
3	Cufflinks	exon	133816904	133817025	.	+	.	gene_id GRMZM2G155974; transcript_id TCONS_00016753; exon_number 9; oId CUFF.34930.2; tss_id TSS34607;
3	Cufflinks	exon	133824712	133824848	.	+	.	gene_id GRMZM2G155974; transcript_id TCONS_00016753; exon_number 10; oId CUFF.34930.2; tss_id TSS34607;
3	Cufflinks	exon	133824914	133825106	.	+	.	gene_id GRMZM2G155974; transcript_id TCONS_00016753; exon_number 11; oId CUFF.34930.2; tss_id TSS34607;
3	Cufflinks	exon	133825211	133825337	.	+	.	gene_id GRMZM2G155974; transcript_id TCONS_00016753; exon_number 12; oId CUFF.34930.2; tss_id TSS34607;
3	Cufflinks	exon	133825728	133826472	.	+	.	gene_id GRMZM2G155974; transcript_id TCONS_00016753; exon_number 13; oId CUFF.34930.2; tss_id TSS34607;
3	Cufflinks	exon	133813069	133813453	.	+	.	gene_id GRMZM2G155974; transcript_id TCONS_00016754; exon_number 1; oId CUFF.34930.3; tss_id TSS34608;
3	Cufflinks	exon	133813826	133813995	.	+	.	gene_id GRMZM2G155974; transcript_id TCONS_00016754; exon_number 2; oId CUFF.34930.3; tss_id TSS34608;
3	Cufflinks	exon	133814302	133814377	.	+	.	gene_id GRMZM2G155974; transcript_id TCONS_00016754; exon_number 3; oId CUFF.34930.3; tss_id TSS34608;
3	Cufflinks	exon	133814631	133814761	.	+	.	gene_id GRMZM2G155974; transcript_id TCONS_00016754; exon_number 4; oId CUFF.34930.3; tss_id TSS34608;
3	Cufflinks	exon	133815549	133815671	.	+	.	gene_id GRMZM2G155974; transcript_id TCONS_00016754; exon_number 5; oId CUFF.34930.3; tss_id TSS34608;
3	Cufflinks	exon	133815761	133816205	.	+	.	gene_id GRMZM2G155974; transcript_id TCONS_00016754; exon_number 6; oId CUFF.34930.3; tss_id TSS34608;
3	Cufflinks	exon	133816351	133816420	.	+	.	gene_id GRMZM2G155974; transcript_id TCONS_00016754; exon_number 7; oId CUFF.34930.3; tss_id TSS34608;
3	Cufflinks	exon	133816904	133817025	.	+	.	gene_id GRMZM2G155974; transcript_id TCONS_00016754; exon_number 8; oId CUFF.34930.3; tss_id TSS34608;
3	Cufflinks	exon	133824712	133824848	.	+	.	gene_id GRMZM2G155974; transcript_id TCONS_00016754; exon_number 9; oId CUFF.34930.3; tss_id TSS34608;
3	Cufflinks	exon	133824914	133825106	.	+	.	gene_id GRMZM2G155974; transcript_id TCONS_00016754; exon_number 10; oId CUFF.34930.3; tss_id TSS34608;
3	Cufflinks	exon	133825211	133825337	.	+	.	gene_id GRMZM2G155974; transcript_id TCONS_00016754; exon_number 11; oId CUFF.34930.3; tss_id TSS34608;
3	Cufflinks	exon	133825728	133826472	.	+	.	gene_id GRMZM2G155974; transcript_id TCONS_00016754; exon_number 12; oId CUFF.34930.3; tss_id TSS34608;
3	Cufflinks	exon	137557685	137558726	.	+	.	gene_id XLOC_014721; transcript_id TCONS_00016791; exon_number 1; oId CUFF.34983.1; tss_id TSS34680;
3	Cufflinks	exon	142722457	142722577	.	+	.	gene_id GRMZM2G152952; transcript_id TCONS_00016852; exon_number 1; oId CUFF.35215.1; tss_id TSS34824;
3	Cufflinks	exon	142723598	142723682	.	+	.	gene_id GRMZM2G152952; transcript_id TCONS_00016852; exon_number 2; oId CUFF.35215.1; tss_id TSS34824;
3	Cufflinks	exon	142724898	142724987	.	+	.	gene_id GRMZM2G152952; transcript_id TCONS_00016852; exon_number 3; oId CUFF.35215.1; tss_id TSS34824;
3	Cufflinks	exon	142725086	142726219	.	+	.	gene_id GRMZM2G152952; transcript_id TCONS_00016852; exon_number 4; oId CUFF.35215.1; tss_id TSS34824;
3	Cufflinks	exon	153898814	153900122	.	+	.	gene_id GRMZM2G145840; transcript_id TCONS_00016963; exon_number 1; oId CUFF.35699.1; tss_id TSS35067;
3	Cufflinks	exon	154652380	154652873	.	+	.	gene_id GRMZM2G428987; transcript_id TCONS_00016974; exon_number 1; oId CUFF.35739.1; tss_id TSS35089;
3	Cufflinks	exon	154666637	154666774	.	+	.	gene_id GRMZM2G428987; transcript_id TCONS_00016974; exon_number 2; oId CUFF.35739.1; tss_id TSS35089;

3	Cufflinks	exon	154667010	154667340	.	+	.	gene_id GRMZM2G428987; transcript_id TCONS_00016974; exon_number 3; oId CUFF.35739.1; tss_id TSS35089;
3	Cufflinks	exon	154667982	154668306	.	+	.	gene_id GRMZM2G428987; transcript_id TCONS_00016974; exon_number 4; oId CUFF.35739.1; tss_id TSS35089;
3	Cufflinks	exon	160370919	160372685	.	+	.	gene_id XLOC_014944; transcript_id TCONS_00017042; exon_number 1; oId CUFF.35981.1; tss_id TSS35203;
3	Cufflinks	exon	172093718	172094315	.	+	.	gene_id XLOC_015094; transcript_id TCONS_00017218; exon_number 1; oId CUFF.36474.1; tss_id TSS35494;
3	Cufflinks	exon	176088170	176090219	.	+	.	gene_id GRMZM2G061321; transcript_id TCONS_00017277; exon_number 1; oId CUFF.36678.1; tss_id TSS35635;
3	Cufflinks	exon	178149296	178152013	.	+	.	gene_id GRMZM2G003682; transcript_id TCONS_00017330; exon_number 1; oId CUFF.36841.1; tss_id TSS35736;
3	Cufflinks	exon	179467655	179468057	.	+	.	gene_id GRMZM2G065557; transcript_id TCONS_00017349; exon_number 1; oId CUFF.36923.1; tss_id TSS35772;
3	Cufflinks	exon	179468186	179468810	.	+	.	gene_id GRMZM2G065557; transcript_id TCONS_00017349; exon_number 2; oId CUFF.36923.1; tss_id TSS35772;
3	Cufflinks	exon	181805644	181805986	.	+	.	gene_id XLOC_015243; transcript_id TCONS_00017391; exon_number 1; oId CUFF.37083.1; tss_id TSS35858;
3	Cufflinks	exon	182091206	182091351	.	+	.	gene_id GRMZM2G127034; transcript_id TCONS_00017396; exon_number 1; oId CUFF.37122.1; tss_id TSS35868;
3	Cufflinks	exon	182095812	182097283	.	+	.	gene_id GRMZM2G127034; transcript_id TCONS_00017396; exon_number 2; oId CUFF.37122.1; tss_id TSS35868;
3	Cufflinks	exon	188039626	188040339	.	+	.	gene_id GRMZM2G702059; transcript_id TCONS_00017502; exon_number 1; oId CUFF.37499.1; tss_id TSS36088;
3	Cufflinks	exon	188041971	188042160	.	+	.	gene_id GRMZM2G702059; transcript_id TCONS_00017502; exon_number 2; oId CUFF.37499.1; tss_id TSS36088;
3	Cufflinks	exon	188042542	188043090	.	+	.	gene_id GRMZM2G702059; transcript_id TCONS_00017502; exon_number 3; oId CUFF.37499.1; tss_id TSS36088;
3	Cufflinks	exon	188387394	188388307	.	+	.	gene_id XLOC_015342; transcript_id TCONS_00017508; exon_number 1; oId CUFF.37517.1; tss_id TSS36101;
3	Cufflinks	exon	189832965	189837084	.	+	.	gene_id GRMZM2G173647; transcript_id TCONS_00017531; exon_number 1; oId CUFF.37612.1; tss_id TSS36136;
3	Cufflinks	exon	190499330	190500455	.	+	.	gene_id GRMZM2G322723; transcript_id TCONS_00017543; exon_number 1; oId CUFF.37660.1; tss_id TSS36147;
3	Cufflinks	exon	190500540	190501128	.	+	.	gene_id GRMZM2G322723; transcript_id TCONS_00017543; exon_number 2; oId CUFF.37660.1; tss_id TSS36147;
3	Cufflinks	exon	190505367	190505501	.	+	.	gene_id GRMZM2G322723; transcript_id TCONS_00017543; exon_number 3; oId CUFF.37660.1; tss_id TSS36147;
3	Cufflinks	exon	190505588	190505757	.	+	.	gene_id GRMZM2G322723; transcript_id TCONS_00017543; exon_number 4; oId CUFF.37660.1; tss_id TSS36147;
3	Cufflinks	exon	190506024	190506234	.	+	.	gene_id GRMZM2G322723; transcript_id TCONS_00017543; exon_number 5; oId CUFF.37660.1; tss_id TSS36147;
3	Cufflinks	exon	190506328	190506562	.	+	.	gene_id GRMZM2G322723; transcript_id TCONS_00017543; exon_number 6; oId CUFF.37660.1; tss_id TSS36147;
3	Cufflinks	exon	190506656	190506806	.	+	.	gene_id GRMZM2G322723; transcript_id TCONS_00017543; exon_number 7; oId CUFF.37660.1; tss_id TSS36147;
3	Cufflinks	exon	190506908	190507302	.	+	.	gene_id GRMZM2G322723; transcript_id TCONS_00017543; exon_number 8; oId CUFF.37660.1; tss_id TSS36147;
3	Cufflinks	exon	197962850	197963438	.	+	.	gene_id XLOC_015471; transcript_id TCONS_00017665; exon_number 1; oId CUFF.38164.1; tss_id TSS36428;
3	Cufflinks	exon	198667643	198668930	.	+	.	gene_id GRMZM2G474326; transcript_id TCONS_00017681; exon_number 1; oId CUFF.38220.1; tss_id TSS36462;
3	Cufflinks	exon	199722345	199722661	.	+	.	gene_id GRMZM2G051151; transcript_id TCONS_00017706; exon_number 1; oId CUFF.38333.1; tss_id TSS36507;
3	Cufflinks	exon	199722749	199723907	.	+	.	gene_id GRMZM2G051151; transcript_id TCONS_00017706; exon_number 2; oId CUFF.38333.1; tss_id TSS36507;
3	Cufflinks	exon	200668046	200668721	.	+	.	gene_id XLOC_015521; transcript_id TCONS_00017719; exon_number 1; oId CUFF.38351.1; tss_id TSS36542;
3	Cufflinks	exon	201694605	201694965	.	+	.	gene_id GRMZM2G025860; transcript_id TCONS_00017737; exon_number 1; oId CUFF.38446.1; tss_id TSS36574;
3	Cufflinks	exon	201695132	201695186	.	+	.	gene_id GRMZM2G025860; transcript_id TCONS_00017737; exon_number 2; oId CUFF.38446.1; tss_id TSS36574;
3	Cufflinks	exon	201695627	201695805	.	+	.	gene_id GRMZM2G025860; transcript_id TCONS_00017737; exon_number 3; oId CUFF.38446.1; tss_id TSS36574;
3	Cufflinks	exon	201697055	201697512	.	+	.	gene_id GRMZM2G025860; transcript_id TCONS_00017737; exon_number 4; oId CUFF.38446.1; tss_id TSS36574;
3	Cufflinks	exon	201697616	201698156	.	+	.	gene_id GRMZM2G025860; transcript_id TCONS_00017737; exon_number 5; oId CUFF.38446.1; tss_id TSS36574;
3	Cufflinks	exon	201698237	201698416	.	+	.	gene_id GRMZM2G025860; transcript_id TCONS_00017737; exon_number 6; oId CUFF.38446.1; tss_id TSS36574;
3	Cufflinks	exon	201698553	201699830	.	+	.	gene_id GRMZM2G025860; transcript_id TCONS_00017737; exon_number 7; oId CUFF.38446.1; tss_id TSS36574;

3	Cufflinks	exon	201699951	201700357	.	+	.	gene_id GRMZM2G025860; transcript_id TCONS_00017737; exon_number 8; oId CUFF.38446.1; tss_id TSS36574;
3	Cufflinks	exon	201700467	201701003	.	+	.	gene_id GRMZM2G025860; transcript_id TCONS_00017737; exon_number 9; oId CUFF.38446.1; tss_id TSS36574;
3	Cufflinks	exon	205769378	205770850	.	+	.	gene_id XLOC_015609; transcript_id TCONS_00017818; exon_number 1; oId CUFF.38698.1; tss_id TSS36708;
3	Cufflinks	exon	205915987	205917102	.	+	.	gene_id XLOC_015611; transcript_id TCONS_00017820; exon_number 1; oId CUFF.38737.1; tss_id TSS36713;
3	Cufflinks	exon	206949878	206950071	.	+	.	gene_id GRMZM2G447632; transcript_id TCONS_00017842; exon_number 1; oId CUFF.38775.1; tss_id TSS36742;
3	Cufflinks	exon	206950167	206950721	.	+	.	gene_id GRMZM2G447632; transcript_id TCONS_00017842; exon_number 2; oId CUFF.38775.1; tss_id TSS36742;
3	Cufflinks	exon	209050410	209053433	.	+	.	gene_id XLOC_015662; transcript_id TCONS_00017883; exon_number 1; oId CUFF.38967.1; tss_id TSS36834;
3	Cufflinks	exon	211203728	211204276	.	+	.	gene_id GRMZM2G122362; transcript_id TCONS_00017919; exon_number 1; oId CUFF.39122.1; tss_id TSS36896;
3	Cufflinks	exon	211204892	211205095	.	+	.	gene_id GRMZM2G122362; transcript_id TCONS_00017919; exon_number 2; oId CUFF.39122.1; tss_id TSS36896;
3	Cufflinks	exon	211205520	211205614	.	+	.	gene_id GRMZM2G122362; transcript_id TCONS_00017919; exon_number 3; oId CUFF.39122.1; tss_id TSS36896;
3	Cufflinks	exon	211206377	211206482	.	+	.	gene_id GRMZM2G122362; transcript_id TCONS_00017919; exon_number 4; oId CUFF.39122.1; tss_id TSS36896;
3	Cufflinks	exon	211207327	211207782	.	+	.	gene_id GRMZM2G122362; transcript_id TCONS_00017919; exon_number 5; oId CUFF.39122.1; tss_id TSS36896;
3	Cufflinks	exon	211207881	211208218	.	+	.	gene_id GRMZM2G122362; transcript_id TCONS_00017919; exon_number 6; oId CUFF.39122.1; tss_id TSS36896;
3	Cufflinks	exon	211208404	211209219	.	+	.	gene_id GRMZM2G122362; transcript_id TCONS_00017919; exon_number 7; oId CUFF.39122.1; tss_id TSS36896;
3	Cufflinks	exon	212517687	212518002	.	+	.	gene_id GRMZM5G814314; transcript_id TCONS_00017943; exon_number 1; oId CUFF.39196.2; tss_id TSS36938;
3	Cufflinks	exon	212518132	212518221	.	+	.	gene_id GRMZM5G814314; transcript_id TCONS_00017943; exon_number 2; oId CUFF.39196.2; tss_id TSS36938;
3	Cufflinks	exon	212519926	212520053	.	+	.	gene_id GRMZM5G814314; transcript_id TCONS_00017943; exon_number 3; oId CUFF.39196.2; tss_id TSS36938;
3	Cufflinks	exon	212520977	212521081	.	+	.	gene_id GRMZM5G814314; transcript_id TCONS_00017943; exon_number 4; oId CUFF.39196.2; tss_id TSS36938;
3	Cufflinks	exon	212521176	212521608	.	+	.	gene_id GRMZM5G814314; transcript_id TCONS_00017943; exon_number 5; oId CUFF.39196.2; tss_id TSS36938;
3	Cufflinks	exon	1349760	1350781	.	-	.	gene_id GRMZM5G816891; transcript_id TCONS_00018306; exon_number 1; oId CUFF.30652.1; tss_id TSS37681;
3	Cufflinks	exon	1350860	1351084	.	-	.	gene_id GRMZM5G816891; transcript_id TCONS_00018306; exon_number 2; oId CUFF.30652.1; tss_id TSS37681;
3	Cufflinks	exon	1351170	1351290	.	-	.	gene_id GRMZM5G816891; transcript_id TCONS_00018306; exon_number 3; oId CUFF.30652.1; tss_id TSS37681;
3	Cufflinks	exon	1351760	1351951	.	-	.	gene_id GRMZM5G816891; transcript_id TCONS_00018306; exon_number 4; oId CUFF.30652.1; tss_id TSS37681;
3	Cufflinks	exon	1352900	1352972	.	-	.	gene_id GRMZM5G816891; transcript_id TCONS_00018306; exon_number 5; oId CUFF.30652.1; tss_id TSS37681;
3	Cufflinks	exon	1353171	1353300	.	-	.	gene_id GRMZM5G816891; transcript_id TCONS_00018306; exon_number 6; oId CUFF.30652.1; tss_id TSS37681;
3	Cufflinks	exon	1353383	1353456	.	-	.	gene_id GRMZM5G816891; transcript_id TCONS_00018306; exon_number 7; oId CUFF.30652.1; tss_id TSS37681;
3	Cufflinks	exon	1353538	1353642	.	-	.	gene_id GRMZM5G816891; transcript_id TCONS_00018306; exon_number 8; oId CUFF.30652.1; tss_id TSS37681;
3	Cufflinks	exon	1353855	1353957	.	-	.	gene_id GRMZM5G816891; transcript_id TCONS_00018306; exon_number 9; oId CUFF.30652.1; tss_id TSS37681;
3	Cufflinks	exon	1354052	1354114	.	-	.	gene_id GRMZM5G816891; transcript_id TCONS_00018306; exon_number 10; oId CUFF.30652.1; tss_id TSS37681;
3	Cufflinks	exon	1354224	1354413	.	-	.	gene_id GRMZM5G816891; transcript_id TCONS_00018306; exon_number 11; oId CUFF.30652.1; tss_id TSS37681;
3	Cufflinks	exon	1354820	1354995	.	-	.	gene_id GRMZM5G816891; transcript_id TCONS_00018306; exon_number 12; oId CUFF.30652.1; tss_id TSS37681;
3	Cufflinks	exon	2003027	2003644	.	-	.	gene_id XLOC_016051; transcript_id TCONS_00018331; exon_number 1; oId CUFF.30674.1; tss_id TSS37722;
3	Cufflinks	exon	3452011	3452668	.	-	.	gene_id GRMZM2G400907; transcript_id TCONS_00018372; exon_number 1; oId CUFF.30830.1; tss_id TSS37814;
3	Cufflinks	exon	3455132	3455207	.	-	.	gene_id GRMZM2G400907; transcript_id TCONS_00018372; exon_number 2; oId CUFF.30830.1; tss_id TSS37814;
3	Cufflinks	exon	3455335	3455457	.	-	.	gene_id GRMZM2G400907; transcript_id TCONS_00018372; exon_number 3; oId CUFF.30830.1; tss_id TSS37814;
3	Cufflinks	exon	3455630	3455681	.	-	.	gene_id GRMZM2G400907; transcript_id TCONS_00018372; exon_number 4; oId CUFF.30830.1; tss_id TSS37814;

3	Cufflinks	exon	3455771	3457360	.	-	.	gene_id GRMZM2G400907; transcript_id TCONS_00018372; exon_number 5; oId CUFF.30830.1; tss_id TSS37814;
3	Cufflinks	exon	3831343	3832351	.	-	.	gene_id XLOC_016089; transcript_id TCONS_00018379; exon_number 1; oId CUFF.30854.1; tss_id TSS37830;
3	Cufflinks	exon	9808882	9809577	.	-	.	gene_id GRMZM2G083418; transcript_id TCONS_00018500; exon_number 1; oId CUFF.31270.1; tss_id TSS38080;
3	Cufflinks	exon	9809685	9809934	.	-	.	gene_id GRMZM2G083418; transcript_id TCONS_00018500; exon_number 2; oId CUFF.31270.1; tss_id TSS38080;
3	Cufflinks	exon	9810973	9811705	.	-	.	gene_id GRMZM2G083418; transcript_id TCONS_00018500; exon_number 3; oId CUFF.31270.1; tss_id TSS38080;
3	Cufflinks	exon	39477668	39478338	.	-	.	gene_id XLOC_016483; transcript_id TCONS_00018823; exon_number 1; oId CUFF.32511.1; tss_id TSS38781;
3	Cufflinks	exon	48806998	48807974	.	-	.	gene_id GRMZM2G115960; transcript_id TCONS_00018895; exon_number 1; oId CUFF.32766.2; tss_id TSS38916;
3	Cufflinks	exon	48808072	48808137	.	-	.	gene_id GRMZM2G115960; transcript_id TCONS_00018895; exon_number 2; oId CUFF.32766.2; tss_id TSS38916;
3	Cufflinks	exon	48808608	48808673	.	-	.	gene_id GRMZM2G115960; transcript_id TCONS_00018895; exon_number 3; oId CUFF.32766.2; tss_id TSS38916;
3	Cufflinks	exon	48808759	48808851	.	-	.	gene_id GRMZM2G115960; transcript_id TCONS_00018895; exon_number 4; oId CUFF.32766.2; tss_id TSS38916;
3	Cufflinks	exon	48808946	48809936	.	-	.	gene_id GRMZM2G115960; transcript_id TCONS_00018895; exon_number 5; oId CUFF.32766.2; tss_id TSS38916;
3	Cufflinks	exon	48810012	48810388	.	-	.	gene_id GRMZM2G115960; transcript_id TCONS_00018895; exon_number 6; oId CUFF.32766.2; tss_id TSS38916;
3	Cufflinks	exon	57097944	57098813	.	-	.	gene_id GRMZM2G381473; transcript_id TCONS_00018938; exon_number 1; oId CUFF.33041.1; tss_id TSS39021;
3	Cufflinks	exon	57099056	57099130	.	-	.	gene_id GRMZM2G381473; transcript_id TCONS_00018938; exon_number 2; oId CUFF.33041.1; tss_id TSS39021;
3	Cufflinks	exon	57099226	57099309	.	-	.	gene_id GRMZM2G381473; transcript_id TCONS_00018938; exon_number 3; oId CUFF.33041.1; tss_id TSS39021;
3	Cufflinks	exon	57099415	57099494	.	-	.	gene_id GRMZM2G381473; transcript_id TCONS_00018938; exon_number 4; oId CUFF.33041.1; tss_id TSS39021;
3	Cufflinks	exon	57100234	57100381	.	-	.	gene_id GRMZM2G381473; transcript_id TCONS_00018938; exon_number 5; oId CUFF.33041.1; tss_id TSS39021;
3	Cufflinks	exon	57101060	57101742	.	-	.	gene_id GRMZM2G381473; transcript_id TCONS_00018938; exon_number 6; oId CUFF.33041.1; tss_id TSS39021;
3	Cufflinks	exon	91617008	91618186	.	-	.	gene_id XLOC_016738; transcript_id TCONS_00019106; exon_number 1; oId CUFF.33811.1; tss_id TSS39407;
3	Cufflinks	exon	95281770	95282337	.	-	.	gene_id XLOC_016757; transcript_id TCONS_00019125; exon_number 1; oId CUFF.33898.1; tss_id TSS39450;
3	Cufflinks	exon	109716966	109717972	.	-	.	gene_id XLOC_016818; transcript_id TCONS_00019190; exon_number 1; oId CUFF.34208.1; tss_id TSS39654;
3	Cufflinks	exon	117275976	117277417	.	-	.	gene_id GRMZM2G022629; transcript_id TCONS_00019242; exon_number 1; oId CUFF.34437.1; tss_id TSS39753;
3	Cufflinks	exon	117277507	117277758	.	-	.	gene_id GRMZM2G022629; transcript_id TCONS_00019242; exon_number 2; oId CUFF.34437.1; tss_id TSS39753;
3	Cufflinks	exon	117278015	117278216	.	-	.	gene_id GRMZM2G022629; transcript_id TCONS_00019242; exon_number 3; oId CUFF.34437.1; tss_id TSS39753;
3	Cufflinks	exon	126190434	126191618	.	-	.	gene_id XLOC_016921; transcript_id TCONS_00019304; exon_number 1; oId CUFF.34644.1; tss_id TSS39864;
3	Cufflinks	exon	127874065	127877164	.	-	.	gene_id XLOC_016942; transcript_id TCONS_00019327; exon_number 1; oId CUFF.34700.1; tss_id TSS39905;
3	Cufflinks	exon	129112111	129113876	.	-	.	gene_id GRMZM2G046088; transcript_id TCONS_00019341; exon_number 1; oId CUFF.34736.1; tss_id TSS39929;
3	Cufflinks	exon	149351183	149353705	.	-	.	gene_id GRMZM2G428168; transcript_id TCONS_00019558; exon_number 1; oId CUFF.35515.1; tss_id TSS40333;
3	Cufflinks	exon	149437930	149438304	.	-	.	gene_id XLOC_017149; transcript_id TCONS_00019561; exon_number 1; oId CUFF.35517.1; tss_id TSS40339;
3	Cufflinks	exon	153899269	153900406	.	-	.	gene_id XLOC_017183; transcript_id TCONS_00019597; exon_number 1; oId CUFF.35700.1; tss_id TSS40412;
3	Cufflinks	exon	154781735	154782216	.	-	.	gene_id XLOC_017197; transcript_id TCONS_00019614; exon_number 1; oId CUFF.35755.1; tss_id TSS40443;
3	Cufflinks	exon	161889843	161890571	.	-	.	gene_id XLOC_017267; transcript_id TCONS_00019696; exon_number 1; oId CUFF.36055.1; tss_id TSS40601;
3	Cufflinks	exon	164927342	164927696	.	-	.	gene_id XLOC_017296; transcript_id TCONS_00019736; exon_number 1; oId CUFF.36117.1; tss_id TSS40644;
3	Cufflinks	exon	165900055	165901778	.	-	.	gene_id GRMZM2G177227; transcript_id TCONS_00019744; exon_number 1; oId CUFF.36149.1; tss_id TSS40664;
3	Cufflinks	exon	165901870	165902629	.	-	.	gene_id GRMZM2G177227; transcript_id TCONS_00019744; exon_number 2; oId CUFF.36149.1; tss_id TSS40664;
3	Cufflinks	exon	171485569	171487165	.	-	.	gene_id GRMZM2G110398; transcript_id TCONS_00019823; exon_number 1; oId CUFF.36429.1; tss_id TSS40801;

3	Cufflinks	exon	171756041	171758403	.	-	.	gene_id GRMZM2G138161; transcript_id TCONS_00019826; exon_number 1; oId CUFF.36466.1; tss_id TSS40804;
3	Cufflinks	exon	171758763	171758963	.	-	.	gene_id GRMZM2G138161; transcript_id TCONS_00019826; exon_number 2; oId CUFF.36466.1; tss_id TSS40804;
3	Cufflinks	exon	171759069	171759280	.	-	.	gene_id GRMZM2G138161; transcript_id TCONS_00019826; exon_number 3; oId CUFF.36466.1; tss_id TSS40804;
3	Cufflinks	exon	171759571	171759676	.	-	.	gene_id GRMZM2G138161; transcript_id TCONS_00019826; exon_number 4; oId CUFF.36466.1; tss_id TSS40804;
3	Cufflinks	exon	171759776	171760110	.	-	.	gene_id GRMZM2G138161; transcript_id TCONS_00019826; exon_number 5; oId CUFF.36466.1; tss_id TSS40804;
3	Cufflinks	exon	175675750	175676373	.	-	.	gene_id GRMZM5G893764; transcript_id TCONS_00019877; exon_number 1; oId CUFF.36708.1; tss_id TSS40896;
3	Cufflinks	exon	175676539	175676616	.	-	.	gene_id GRMZM5G893764; transcript_id TCONS_00019877; exon_number 2; oId CUFF.36708.1; tss_id TSS40896;
3	Cufflinks	exon	175677137	175677202	.	-	.	gene_id GRMZM5G893764; transcript_id TCONS_00019877; exon_number 3; oId CUFF.36708.1; tss_id TSS40896;
3	Cufflinks	exon	175677359	175677401	.	-	.	gene_id GRMZM5G893764; transcript_id TCONS_00019877; exon_number 4; oId CUFF.36708.1; tss_id TSS40896;
3	Cufflinks	exon	175677605	175677762	.	-	.	gene_id GRMZM5G893764; transcript_id TCONS_00019877; exon_number 5; oId CUFF.36708.1; tss_id TSS40896;
3	Cufflinks	exon	175677833	175677923	.	-	.	gene_id GRMZM5G893764; transcript_id TCONS_00019877; exon_number 6; oId CUFF.36708.1; tss_id TSS40896;
3	Cufflinks	exon	175678268	175678341	.	-	.	gene_id GRMZM5G893764; transcript_id TCONS_00019877; exon_number 7; oId CUFF.36708.1; tss_id TSS40896;
3	Cufflinks	exon	175678508	175678605	.	-	.	gene_id GRMZM5G893764; transcript_id TCONS_00019877; exon_number 8; oId CUFF.36708.1; tss_id TSS40896;
3	Cufflinks	exon	175678973	175679047	.	-	.	gene_id GRMZM5G893764; transcript_id TCONS_00019877; exon_number 9; oId CUFF.36708.1; tss_id TSS40896;
3	Cufflinks	exon	175679470	175679545	.	-	.	gene_id GRMZM5G893764; transcript_id TCONS_00019877; exon_number 10; oId CUFF.36708.1; tss_id TSS40896;
3	Cufflinks	exon	175679634	175679702	.	-	.	gene_id GRMZM5G893764; transcript_id TCONS_00019877; exon_number 11; oId CUFF.36708.1; tss_id TSS40896;
3	Cufflinks	exon	175680062	175680127	.	-	.	gene_id GRMZM5G893764; transcript_id TCONS_00019877; exon_number 12; oId CUFF.36708.1; tss_id TSS40896;
3	Cufflinks	exon	175680263	175680444	.	-	.	gene_id GRMZM5G893764; transcript_id TCONS_00019877; exon_number 13; oId CUFF.36708.1; tss_id TSS40896;
3	Cufflinks	exon	176088318	176089631	.	-	.	gene_id XLOC_017421; transcript_id TCONS_00019891; exon_number 1; oId CUFF.36679.1; tss_id TSS40914;
3	Cufflinks	exon	176607781	176609067	.	-	.	gene_id GRMZM2G127117; transcript_id TCONS_00019902; exon_number 1; oId CUFF.36752.1; tss_id TSS40928;
3	Cufflinks	exon	176610153	176610780	.	-	.	gene_id GRMZM2G127117; transcript_id TCONS_00019902; exon_number 2; oId CUFF.36752.1; tss_id TSS40928;
3	Cufflinks	exon	178150170	178151372	.	-	.	gene_id XLOC_017449; transcript_id TCONS_00019923; exon_number 1; oId CUFF.36843.1; tss_id TSS40966;
3	Cufflinks	exon	178923328	178923663	.	-	.	gene_id XLOC_017459; transcript_id TCONS_00019933; exon_number 1; oId CUFF.36882.1; tss_id TSS40981;
3	Cufflinks	exon	180252317	180254703	.	-	.	gene_id XLOC_017485; transcript_id TCONS_00019964; exon_number 1; oId CUFF.36978.1; tss_id TSS41034;
3	Cufflinks	exon	181844862	181845535	.	-	.	gene_id XLOC_017510; transcript_id TCONS_00019993; exon_number 1; oId CUFF.37095.1; tss_id TSS41093;
3	Cufflinks	exon	186273476	186274094	.	-	.	gene_id XLOC_017571; transcript_id TCONS_00020062; exon_number 1; oId CUFF.37343.1; tss_id TSS41225;
3	Cufflinks	exon	188042731	188043031	.	-	.	gene_id XLOC_017614; transcript_id TCONS_00020113; exon_number 1; oId CUFF.37500.1; tss_id TSS41300;
3	Cufflinks	exon	188387390	188388582	.	-	.	gene_id GRMZM2G047274; transcript_id TCONS_00020118; exon_number 1; oId CUFF.37516.1; tss_id TSS41310;
3	Cufflinks	exon	188389727	188390373	.	-	.	gene_id GRMZM2G047274; transcript_id TCONS_00020118; exon_number 2; oId CUFF.37516.1; tss_id TSS41310;
3	Cufflinks	exon	189594096	189594783	.	-	.	gene_id XLOC_017636; transcript_id TCONS_00020141; exon_number 1; oId CUFF.37592.1; tss_id TSS41347;
3	Cufflinks	exon	190499018	190501286	.	-	.	gene_id XLOC_017645; transcript_id TCONS_00020150; exon_number 1; oId CUFF.37659.1; tss_id TSS41370;
3	Cufflinks	exon	190513119	190513303	.	-	.	gene_id GRMZM2G322728; transcript_id TCONS_00020151; exon_number 1; oId CUFF.37663.1; tss_id TSS41372;
3	Cufflinks	exon	190513565	190513956	.	-	.	gene_id GRMZM2G322728; transcript_id TCONS_00020151; exon_number 2; oId CUFF.37663.1; tss_id TSS41372;
3	Cufflinks	exon	190514055	190514205	.	-	.	gene_id GRMZM2G322728; transcript_id TCONS_00020151; exon_number 3; oId CUFF.37663.1; tss_id TSS41372;
3	Cufflinks	exon	190514325	190514562	.	-	.	gene_id GRMZM2G322728; transcript_id TCONS_00020151; exon_number 4; oId CUFF.37663.1; tss_id TSS41372;
3	Cufflinks	exon	190514676	190514886	.	-	.	gene_id GRMZM2G322728; transcript_id TCONS_00020151; exon_number 5; oId CUFF.37663.1; tss_id TSS41372;

3	Cufflinks	exon	190514975	190515318	.	-	.	gene_id GRMZM2G322728; transcript_id TCONS_00020151; exon_number 6; oId CUFF.37663.1; tss_id TSS41372;
3	Cufflinks	exon	190516881	190518692	.	-	.	gene_id GRMZM2G322728; transcript_id TCONS_00020151; exon_number 7; oId CUFF.37663.1; tss_id TSS41372;
3	Cufflinks	exon	191183824	191184296	.	-	.	gene_id GRMZM2G054905; transcript_id TCONS_00020166; exon_number 1; oId CUFF.37771.1; tss_id TSS41413;
3	Cufflinks	exon	191184507	191184629	.	-	.	gene_id GRMZM2G054905; transcript_id TCONS_00020166; exon_number 2; oId CUFF.37771.1; tss_id TSS41413;
3	Cufflinks	exon	191184979	191185065	.	-	.	gene_id GRMZM2G054905; transcript_id TCONS_00020166; exon_number 3; oId CUFF.37771.1; tss_id TSS41413;
3	Cufflinks	exon	191185165	191185216	.	-	.	gene_id GRMZM2G054905; transcript_id TCONS_00020166; exon_number 4; oId CUFF.37771.1; tss_id TSS41413;
3	Cufflinks	exon	191185347	191185471	.	-	.	gene_id GRMZM2G054905; transcript_id TCONS_00020166; exon_number 5; oId CUFF.37771.1; tss_id TSS41413;
3	Cufflinks	exon	191194679	191194887	.	-	.	gene_id GRMZM2G054905; transcript_id TCONS_00020166; exon_number 6; oId CUFF.37771.1; tss_id TSS41413;
3	Cufflinks	exon	191194982	191195183	.	-	.	gene_id GRMZM2G054905; transcript_id TCONS_00020166; exon_number 7; oId CUFF.37771.1; tss_id TSS41413;
3	Cufflinks	exon	191202902	191203006	.	-	.	gene_id GRMZM2G054905; transcript_id TCONS_00020166; exon_number 8; oId CUFF.37771.1; tss_id TSS41413;
3	Cufflinks	exon	191203384	191203445	.	-	.	gene_id GRMZM2G054905; transcript_id TCONS_00020166; exon_number 9; oId CUFF.37771.1; tss_id TSS41413;
3	Cufflinks	exon	191203557	191203746	.	-	.	gene_id GRMZM2G054905; transcript_id TCONS_00020166; exon_number 10; oId CUFF.37771.1; tss_id TSS41413;
3	Cufflinks	exon	191203892	191203987	.	-	.	gene_id GRMZM2G054905; transcript_id TCONS_00020166; exon_number 11; oId CUFF.37771.1; tss_id TSS41413;
3	Cufflinks	exon	191204105	191204207	.	-	.	gene_id GRMZM2G054905; transcript_id TCONS_00020166; exon_number 12; oId CUFF.37771.1; tss_id TSS41413;
3	Cufflinks	exon	191216372	191216475	.	-	.	gene_id GRMZM2G054905; transcript_id TCONS_00020166; exon_number 13; oId CUFF.37771.1; tss_id TSS41413;
3	Cufflinks	exon	191216836	191216922	.	-	.	gene_id GRMZM2G054905; transcript_id TCONS_00020166; exon_number 14; oId CUFF.37771.1; tss_id TSS41413;
3	Cufflinks	exon	191217150	191217235	.	-	.	gene_id GRMZM2G054905; transcript_id TCONS_00020166; exon_number 15; oId CUFF.37771.1; tss_id TSS41413;
3	Cufflinks	exon	191217342	191217434	.	-	.	gene_id GRMZM2G054905; transcript_id TCONS_00020166; exon_number 16; oId CUFF.37771.1; tss_id TSS41413;
3	Cufflinks	exon	191217679	191217841	.	-	.	gene_id GRMZM2G054905; transcript_id TCONS_00020166; exon_number 17; oId CUFF.37771.1; tss_id TSS41413;
3	Cufflinks	exon	191217938	191218050	.	-	.	gene_id GRMZM2G054905; transcript_id TCONS_00020166; exon_number 18; oId CUFF.37771.1; tss_id TSS41413;
3	Cufflinks	exon	191218118	191218237	.	-	.	gene_id GRMZM2G054905; transcript_id TCONS_00020166; exon_number 19; oId CUFF.37771.1; tss_id TSS41413;
3	Cufflinks	exon	191218773	191218824	.	-	.	gene_id GRMZM2G054905; transcript_id TCONS_00020166; exon_number 20; oId CUFF.37771.1; tss_id TSS41413;
3	Cufflinks	exon	191232565	191232608	.	-	.	gene_id GRMZM2G054905; transcript_id TCONS_00020166; exon_number 21; oId CUFF.37771.1; tss_id TSS41413;
3	Cufflinks	exon	191233077	191233150	.	-	.	gene_id GRMZM2G054905; transcript_id TCONS_00020166; exon_number 22; oId CUFF.37771.1; tss_id TSS41413;
3	Cufflinks	exon	191233272	191233363	.	-	.	gene_id GRMZM2G054905; transcript_id TCONS_00020166; exon_number 23; oId CUFF.37771.1; tss_id TSS41413;
3	Cufflinks	exon	191242001	191242068	.	-	.	gene_id GRMZM2G054905; transcript_id TCONS_00020166; exon_number 24; oId CUFF.37771.1; tss_id TSS41413;
3	Cufflinks	exon	191243677	191243752	.	-	.	gene_id GRMZM2G054905; transcript_id TCONS_00020166; exon_number 25; oId CUFF.37771.1; tss_id TSS41413;
3	Cufflinks	exon	191243919	191244226	.	-	.	gene_id GRMZM2G054905; transcript_id TCONS_00020166; exon_number 26; oId CUFF.37771.1; tss_id TSS41413;
3	Cufflinks	exon	198284004	198285254	.	-	.	gene_id GRMZM2G141299; transcript_id TCONS_00020271; exon_number 1; oId CUFF.38206.1; tss_id TSS41664;
3	Cufflinks	exon	198285393	198285545	.	-	.	gene_id GRMZM2G141299; transcript_id TCONS_00020271; exon_number 2; oId CUFF.38206.1; tss_id TSS41664;
3	Cufflinks	exon	198285731	198286691	.	-	.	gene_id GRMZM2G141299; transcript_id TCONS_00020271; exon_number 3; oId CUFF.38206.1; tss_id TSS41664;
3	Cufflinks	exon	199377243	199378043	.	-	.	gene_id GRMZM2G057067; transcript_id TCONS_00020297; exon_number 1; oId CUFF.38281.1; tss_id TSS41714;
3	Cufflinks	exon	199378142	199378218	.	-	.	gene_id GRMZM2G057067; transcript_id TCONS_00020297; exon_number 2; oId CUFF.38281.1; tss_id TSS41714;
3	Cufflinks	exon	199378299	199378383	.	-	.	gene_id GRMZM2G057067; transcript_id TCONS_00020297; exon_number 3; oId CUFF.38281.1; tss_id TSS41714;
3	Cufflinks	exon	199378549	199378675	.	-	.	gene_id GRMZM2G057067; transcript_id TCONS_00020297; exon_number 4; oId CUFF.38281.1; tss_id TSS41714;
3	Cufflinks	exon	199378947	199379218	.	-	.	gene_id GRMZM2G057067; transcript_id TCONS_00020297; exon_number 5; oId CUFF.38281.1; tss_id TSS41714;

3	Cufflinks	exon	199379387	199379570	.	-	.	gene_id GRMZM2G057067; transcript_id TCONS_00020297; exon_number 6; oId CUFF.38281.1; tss_id TSS41714;
3	Cufflinks	exon	199379763	199380242	.	-	.	gene_id GRMZM2G057067; transcript_id TCONS_00020297; exon_number 7; oId CUFF.38281.1; tss_id TSS41714;
3	Cufflinks	exon	201085643	201087293	.	-	.	gene_id XLOC_017804; transcript_id TCONS_00020322; exon_number 1; oId CUFF.38401.1; tss_id TSS41764;
3	Cufflinks	exon	201756915	201757140	.	-	.	gene_id GRMZM2G159724; transcript_id TCONS_00020339; exon_number 1; oId CUFF.38449.1; tss_id TSS41797;
3	Cufflinks	exon	201757241	201757369	.	-	.	gene_id GRMZM2G159724; transcript_id TCONS_00020339; exon_number 2; oId CUFF.38449.1; tss_id TSS41797;
3	Cufflinks	exon	201757626	201757716	.	-	.	gene_id GRMZM2G159724; transcript_id TCONS_00020339; exon_number 3; oId CUFF.38449.1; tss_id TSS41797;
3	Cufflinks	exon	201757798	201757872	.	-	.	gene_id GRMZM2G159724; transcript_id TCONS_00020339; exon_number 4; oId CUFF.38449.1; tss_id TSS41797;
3	Cufflinks	exon	201757952	201758029	.	-	.	gene_id GRMZM2G159724; transcript_id TCONS_00020339; exon_number 5; oId CUFF.38449.1; tss_id TSS41797;
3	Cufflinks	exon	201758113	201758202	.	-	.	gene_id GRMZM2G159724; transcript_id TCONS_00020339; exon_number 6; oId CUFF.38449.1; tss_id TSS41797;
3	Cufflinks	exon	201758288	201758386	.	-	.	gene_id GRMZM2G159724; transcript_id TCONS_00020339; exon_number 7; oId CUFF.38449.1; tss_id TSS41797;
3	Cufflinks	exon	201758492	201758545	.	-	.	gene_id GRMZM2G159724; transcript_id TCONS_00020339; exon_number 8; oId CUFF.38449.1; tss_id TSS41797;
3	Cufflinks	exon	201758635	201758682	.	-	.	gene_id GRMZM2G159724; transcript_id TCONS_00020339; exon_number 9; oId CUFF.38449.1; tss_id TSS41797;
3	Cufflinks	exon	201759032	201759130	.	-	.	gene_id GRMZM2G159724; transcript_id TCONS_00020339; exon_number 10; oId CUFF.38449.1; tss_id TSS41797;
3	Cufflinks	exon	201759261	201759344	.	-	.	gene_id GRMZM2G159724; transcript_id TCONS_00020339; exon_number 11; oId CUFF.38449.1; tss_id TSS41797;
3	Cufflinks	exon	201759493	201759564	.	-	.	gene_id GRMZM2G159724; transcript_id TCONS_00020339; exon_number 12; oId CUFF.38449.1; tss_id TSS41797;
3	Cufflinks	exon	201759659	201759754	.	-	.	gene_id GRMZM2G159724; transcript_id TCONS_00020339; exon_number 13; oId CUFF.38449.1; tss_id TSS41797;
3	Cufflinks	exon	201759850	201759915	.	-	.	gene_id GRMZM2G159724; transcript_id TCONS_00020339; exon_number 14; oId CUFF.38449.1; tss_id TSS41797;
3	Cufflinks	exon	201760004	201760109	.	-	.	gene_id GRMZM2G159724; transcript_id TCONS_00020339; exon_number 15; oId CUFF.38449.1; tss_id TSS41797;
3	Cufflinks	exon	201760195	201760346	.	-	.	gene_id GRMZM2G159724; transcript_id TCONS_00020339; exon_number 16; oId CUFF.38449.1; tss_id TSS41797;
3	Cufflinks	exon	201760441	201760515	.	-	.	gene_id GRMZM2G159724; transcript_id TCONS_00020339; exon_number 17; oId CUFF.38449.1; tss_id TSS41797;
3	Cufflinks	exon	201760601	201760721	.	-	.	gene_id GRMZM2G159724; transcript_id TCONS_00020339; exon_number 18; oId CUFF.38449.1; tss_id TSS41797;
3	Cufflinks	exon	201761100	201761523	.	-	.	gene_id GRMZM2G159724; transcript_id TCONS_00020339; exon_number 19; oId CUFF.38449.1; tss_id TSS41797;
3	Cufflinks	exon	205701185	205702703	.	-	.	gene_id XLOC_017881; transcript_id TCONS_00020410; exon_number 1; oId CUFF.38700.1; tss_id TSS41940;
3	Cufflinks	exon	205915252	205917259	.	-	.	gene_id AC233882.1_FG003; transcript_id TCONS_00020418; exon_number 1; oId CUFF.38736.2; tss_id TSS41964;
3	Cufflinks	exon	205917920	205918183	.	-	.	gene_id AC233882.1_FG003; transcript_id TCONS_00020418; exon_number 2; oId CUFF.38736.2; tss_id TSS41964;
3	Cufflinks	exon	205918260	205918526	.	-	.	gene_id AC233882.1_FG003; transcript_id TCONS_00020418; exon_number 3; oId CUFF.38736.2; tss_id TSS41964;
3	Cufflinks	exon	205918757	205918985	.	-	.	gene_id AC233882.1_FG003; transcript_id TCONS_00020418; exon_number 4; oId CUFF.38736.2; tss_id TSS41964;
3	Cufflinks	exon	205919080	205919713	.	-	.	gene_id AC233882.1_FG003; transcript_id TCONS_00020418; exon_number 5; oId CUFF.38736.2; tss_id TSS41964;
3	Cufflinks	exon	205919846	205920119	.	-	.	gene_id AC233882.1_FG003; transcript_id TCONS_00020418; exon_number 6; oId CUFF.38736.2; tss_id TSS41964;
3	Cufflinks	exon	205920199	205920724	.	-	.	gene_id AC233882.1_FG003; transcript_id TCONS_00020418; exon_number 7; oId CUFF.38736.2; tss_id TSS41964;
3	Cufflinks	exon	205920962	205921200	.	-	.	gene_id AC233882.1_FG003; transcript_id TCONS_00020418; exon_number 8; oId CUFF.38736.2; tss_id TSS41964;
3	Cufflinks	exon	205921291	205921512	.	-	.	gene_id AC233882.1_FG003; transcript_id TCONS_00020418; exon_number 9; oId CUFF.38736.2; tss_id TSS41964;
3	Cufflinks	exon	205921833	205922008	.	-	.	gene_id AC233882.1_FG003; transcript_id TCONS_00020418; exon_number 10; oId CUFF.38736.2; tss_id TSS41964;
3	Cufflinks	exon	205922541	205922597	.	-	.	gene_id AC233882.1_FG003; transcript_id TCONS_00020418; exon_number 11; oId CUFF.38736.2; tss_id TSS41964;
3	Cufflinks	exon	206848752	206849130	.	-	.	gene_id XLOC_017899; transcript_id TCONS_00020432; exon_number 1; oId CUFF.38765.1; tss_id TSS41991;
3	Cufflinks	exon	206923316	206924346	.	-	.	gene_id XLOC_017900; transcript_id TCONS_00020433; exon_number 1; oId CUFF.38784.1; tss_id TSS41994;

3	Cufflinks	exon	206950153	206950714	.	-	.	gene_id XLOC_017903; transcript_id TCONS_00020436; exon_number 1; oId CUFF.38776.1; tss_id TSS41997;
3	Cufflinks	exon	210588766	210589321	.	-	.	gene_id XLOC_017959; transcript_id TCONS_00020499; exon_number 1; oId CUFF.39057.1; tss_id TSS42150;
3	Cufflinks	exon	211203731	211204235	.	-	.	gene_id XLOC_017967; transcript_id TCONS_00020508; exon_number 1; oId CUFF.39123.1; tss_id TSS42189;
3	Cufflinks	exon	211845283	211846698	.	-	.	gene_id XLOC_017976; transcript_id TCONS_00020517; exon_number 1; oId CUFF.39146.1; tss_id TSS42209;
3	Cufflinks	exon	220604720	220604988	.	-	.	gene_id GRMZM2G116584; transcript_id TCONS_00020655; exon_number 1; oId CUFF.39709.2; tss_id TSS42502;
3	Cufflinks	exon	220605262	220605520	.	-	.	gene_id GRMZM2G116584; transcript_id TCONS_00020655; exon_number 2; oId CUFF.39709.2; tss_id TSS42502;
3	Cufflinks	exon	220605737	220605849	.	-	.	gene_id GRMZM2G116584; transcript_id TCONS_00020655; exon_number 3; oId CUFF.39709.2; tss_id TSS42502;
3	Cufflinks	exon	220605937	220606017	.	-	.	gene_id GRMZM2G116584; transcript_id TCONS_00020655; exon_number 4; oId CUFF.39709.2; tss_id TSS42502;
3	Cufflinks	exon	220606109	220606161	.	-	.	gene_id GRMZM2G116584; transcript_id TCONS_00020655; exon_number 5; oId CUFF.39709.2; tss_id TSS42502;
3	Cufflinks	exon	220606263	220606371	.	-	.	gene_id GRMZM2G116584; transcript_id TCONS_00020655; exon_number 6; oId CUFF.39709.2; tss_id TSS42502;
3	Cufflinks	exon	220606546	220606605	.	-	.	gene_id GRMZM2G116584; transcript_id TCONS_00020655; exon_number 7; oId CUFF.39709.2; tss_id TSS42502;
3	Cufflinks	exon	220607729	220607811	.	-	.	gene_id GRMZM2G116584; transcript_id TCONS_00020655; exon_number 8; oId CUFF.39709.2; tss_id TSS42502;
3	Cufflinks	exon	220607932	220608229	.	-	.	gene_id GRMZM2G116584; transcript_id TCONS_00020655; exon_number 9; oId CUFF.39709.2; tss_id TSS42502;
3	Cufflinks	exon	220608354	220608431	.	-	.	gene_id GRMZM2G116584; transcript_id TCONS_00020655; exon_number 10; oId CUFF.39709.2; tss_id TSS42502;
3	Cufflinks	exon	226500327	226500904	.	-	.	gene_id XLOC_018198; transcript_id TCONS_00020778; exon_number 1; oId CUFF.40066.1; tss_id TSS42741;
3	Cufflinks	exon	226856807	226857659	.	-	.	gene_id GRMZM2G071154; transcript_id TCONS_00020781; exon_number 1; oId CUFF.40150.1; tss_id TSS42751;
3	Cufflinks	exon	226858154	226858237	.	-	.	gene_id GRMZM2G071154; transcript_id TCONS_00020781; exon_number 2; oId CUFF.40150.1; tss_id TSS42751;
3	Cufflinks	exon	226858378	226858478	.	-	.	gene_id GRMZM2G071154; transcript_id TCONS_00020781; exon_number 3; oId CUFF.40150.1; tss_id TSS42751;
3	Cufflinks	exon	226858590	226859571	.	-	.	gene_id GRMZM2G071154; transcript_id TCONS_00020781; exon_number 4; oId CUFF.40150.1; tss_id TSS42751;
4	Cufflinks	exon	32363	33792	.	+	.	gene_id GRMZM2G397661; transcript_id TCONS_00020871; exon_number 1; oId CUFF.40594.1; tss_id TSS42946;
4	Cufflinks	exon	832823	835056	.	+	.	gene_id XLOC_018293; transcript_id TCONS_00020883; exon_number 1; oId CUFF.40551.1; tss_id TSS42971;
4	Cufflinks	exon	2432796	2433635	.	+	.	gene_id XLOC_018321; transcript_id TCONS_00020912; exon_number 1; oId CUFF.40664.1; tss_id TSS43018;
4	Cufflinks	exon	3487958	3488996	.	+	.	gene_id XLOC_018349; transcript_id TCONS_00020945; exon_number 1; oId CUFF.40787.1; tss_id TSS43078;
4	Cufflinks	exon	4790674	4791438	.	+	.	gene_id GRMZM2G165325; transcript_id TCONS_00020990; exon_number 1; oId CUFF.40853.1; tss_id TSS43148;
4	Cufflinks	exon	4791620	4792253	.	+	.	gene_id GRMZM2G165325; transcript_id TCONS_00020990; exon_number 2; oId CUFF.40853.1; tss_id TSS43148;
4	Cufflinks	exon	6248121	6248792	.	+	.	gene_id XLOC_018421; transcript_id TCONS_00021033; exon_number 1; oId CUFF.40942.1; tss_id TSS43199;
4	Cufflinks	exon	9490215	9490772	.	+	.	gene_id GRMZM2G027021; transcript_id TCONS_00021060; exon_number 1; oId CUFF.41073.1; tss_id TSS43244;
4	Cufflinks	exon	9491045	9491187	.	+	.	gene_id GRMZM2G027021; transcript_id TCONS_00021060; exon_number 2; oId CUFF.41073.1; tss_id TSS43244;
4	Cufflinks	exon	9493671	9493787	.	+	.	gene_id GRMZM2G027021; transcript_id TCONS_00021060; exon_number 3; oId CUFF.41073.1; tss_id TSS43244;
4	Cufflinks	exon	9494475	9494589	.	+	.	gene_id GRMZM2G027021; transcript_id TCONS_00021060; exon_number 4; oId CUFF.41073.1; tss_id TSS43244;
4	Cufflinks	exon	9494727	9494864	.	+	.	gene_id GRMZM2G027021; transcript_id TCONS_00021060; exon_number 5; oId CUFF.41073.1; tss_id TSS43244;
4	Cufflinks	exon	9495146	9495241	.	+	.	gene_id GRMZM2G027021; transcript_id TCONS_00021060; exon_number 6; oId CUFF.41073.1; tss_id TSS43244;
4	Cufflinks	exon	9495328	9495441	.	+	.	gene_id GRMZM2G027021; transcript_id TCONS_00021060; exon_number 7; oId CUFF.41073.1; tss_id TSS43244;
4	Cufflinks	exon	9495641	9495709	.	+	.	gene_id GRMZM2G027021; transcript_id TCONS_00021060; exon_number 8; oId CUFF.41073.1; tss_id TSS43244;
4	Cufflinks	exon	9495788	9495858	.	+	.	gene_id GRMZM2G027021; transcript_id TCONS_00021060; exon_number 9; oId CUFF.41073.1; tss_id TSS43244;
4	Cufflinks	exon	9495957	9496050	.	+	.	gene_id GRMZM2G027021; transcript_id TCONS_00021060; exon_number 10; oId CUFF.41073.1; tss_id TSS43244;

4	Cufflinks	exon	9496650	9496775	.	+	.	gene_id GRMZM2G027021; transcript_id TCONS_00021060; exon_number 11; oId CUFF.41073.1; tss_id TSS43244;
4	Cufflinks	exon	9498381	9498470	.	+	.	gene_id GRMZM2G027021; transcript_id TCONS_00021060; exon_number 12; oId CUFF.41073.1; tss_id TSS43244;
4	Cufflinks	exon	9499004	9499465	.	+	.	gene_id GRMZM2G027021; transcript_id TCONS_00021060; exon_number 13; oId CUFF.41073.1; tss_id TSS43244;
4	Cufflinks	exon	14054454	14055226	.	+	.	gene_id GRMZM2G581155; transcript_id TCONS_00021107; exon_number 1; oId CUFF.41235.1; tss_id TSS43343;
4	Cufflinks	exon	14055393	14055897	.	+	.	gene_id GRMZM2G581155; transcript_id TCONS_00021107; exon_number 2; oId CUFF.41235.1; tss_id TSS43343;
4	Cufflinks	exon	14058099	14058300	.	+	.	gene_id GRMZM2G581155; transcript_id TCONS_00021107; exon_number 3; oId CUFF.41235.1; tss_id TSS43343;
4	Cufflinks	exon	14058592	14059644	.	+	.	gene_id GRMZM2G581155; transcript_id TCONS_00021107; exon_number 4; oId CUFF.41235.1; tss_id TSS43343;
4	Cufflinks	exon	18698719	18699241	.	+	.	gene_id GRMZM2G574782; transcript_id TCONS_00021165; exon_number 1; oId CUFF.41453.1; tss_id TSS43451;
4	Cufflinks	exon	18700155	18700291	.	+	.	gene_id GRMZM2G574782; transcript_id TCONS_00021165; exon_number 2; oId CUFF.41453.1; tss_id TSS43451;
4	Cufflinks	exon	18700379	18700474	.	+	.	gene_id GRMZM2G574782; transcript_id TCONS_00021165; exon_number 3; oId CUFF.41453.1; tss_id TSS43451;
4	Cufflinks	exon	18701611	18701730	.	+	.	gene_id GRMZM2G574782; transcript_id TCONS_00021165; exon_number 4; oId CUFF.41453.1; tss_id TSS43451;
4	Cufflinks	exon	18702200	18702289	.	+	.	gene_id GRMZM2G574782; transcript_id TCONS_00021165; exon_number 5; oId CUFF.41453.1; tss_id TSS43451;
4	Cufflinks	exon	18703132	18703293	.	+	.	gene_id GRMZM2G574782; transcript_id TCONS_00021165; exon_number 6; oId CUFF.41453.1; tss_id TSS43451;
4	Cufflinks	exon	18703473	18703625	.	+	.	gene_id GRMZM2G574782; transcript_id TCONS_00021165; exon_number 7; oId CUFF.41453.1; tss_id TSS43451;
4	Cufflinks	exon	18703775	18703924	.	+	.	gene_id GRMZM2G574782; transcript_id TCONS_00021165; exon_number 8; oId CUFF.41453.1; tss_id TSS43451;
4	Cufflinks	exon	18704020	18704121	.	+	.	gene_id GRMZM2G574782; transcript_id TCONS_00021165; exon_number 9; oId CUFF.41453.1; tss_id TSS43451;
4	Cufflinks	exon	18704517	18704626	.	+	.	gene_id GRMZM2G574782; transcript_id TCONS_00021165; exon_number 10; oId CUFF.41453.1; tss_id TSS43451;
4	Cufflinks	exon	18704787	18704905	.	+	.	gene_id GRMZM2G574782; transcript_id TCONS_00021165; exon_number 11; oId CUFF.41453.1; tss_id TSS43451;
4	Cufflinks	exon	18705192	18705550	.	+	.	gene_id GRMZM2G574782; transcript_id TCONS_00021165; exon_number 12; oId CUFF.41453.1; tss_id TSS43451;
4	Cufflinks	exon	29179242	29181499	.	+	.	gene_id XLOC_018633; transcript_id TCONS_00021274; exon_number 1; oId CUFF.41896.1; tss_id TSS43690;
4	Cufflinks	exon	39629171	39629328	.	+	.	gene_id GRMZM2G135547; transcript_id TCONS_00021398; exon_number 1; oId CUFF.42322.3; tss_id TSS43909;
4	Cufflinks	exon	39630821	39630890	.	+	.	gene_id GRMZM2G135547; transcript_id TCONS_00021398; exon_number 2; oId CUFF.42322.3; tss_id TSS43909;
4	Cufflinks	exon	39630980	39631007	.	+	.	gene_id GRMZM2G135547; transcript_id TCONS_00021398; exon_number 3; oId CUFF.42322.3; tss_id TSS43909;
4	Cufflinks	exon	39631355	39631483	.	+	.	gene_id GRMZM2G135547; transcript_id TCONS_00021398; exon_number 4; oId CUFF.42322.3; tss_id TSS43909;
4	Cufflinks	exon	39631550	39633705	.	+	.	gene_id GRMZM2G135547; transcript_id TCONS_00021398; exon_number 5; oId CUFF.42322.3; tss_id TSS43909;
4	Cufflinks	exon	39629171	39629328	.	+	.	gene_id GRMZM2G135547; transcript_id TCONS_00021399; exon_number 1; oId CUFF.42322.2; tss_id TSS43909;
4	Cufflinks	exon	39630821	39630890	.	+	.	gene_id GRMZM2G135547; transcript_id TCONS_00021399; exon_number 2; oId CUFF.42322.2; tss_id TSS43909;
4	Cufflinks	exon	39630980	39631007	.	+	.	gene_id GRMZM2G135547; transcript_id TCONS_00021399; exon_number 3; oId CUFF.42322.2; tss_id TSS43909;
4	Cufflinks	exon	39631355	39631483	.	+	.	gene_id GRMZM2G135547; transcript_id TCONS_00021399; exon_number 4; oId CUFF.42322.2; tss_id TSS43909;
4	Cufflinks	exon	39631553	39633705	.	+	.	gene_id GRMZM2G135547; transcript_id TCONS_00021399; exon_number 5; oId CUFF.42322.2; tss_id TSS43909;
4	Cufflinks	exon	61742780	61743475	.	+	.	gene_id XLOC_018851; transcript_id TCONS_00021532; exon_number 1; oId CUFF.42912.2; tss_id TSS44241;
4	Cufflinks	exon	63119243	63119725	.	+	.	gene_id XLOC_018861; transcript_id TCONS_00021544; exon_number 1; oId CUFF.42942.1; tss_id TSS44264;
4	Cufflinks	exon	86309261	86310675	.	+	.	gene_id XLOC_018991; transcript_id TCONS_00021692; exon_number 1; oId CUFF.43592.1; tss_id TSS44618;
4	Cufflinks	exon	92860154	92860755	.	+	.	gene_id XLOC_019017; transcript_id TCONS_00021723; exon_number 1; oId CUFF.43699.1; tss_id TSS44693;
4	Cufflinks	exon	131204417	131205991	.	+	.	gene_id GRMZM2G407996; transcript_id TCONS_00021928; exon_number 1; oId CUFF.44607.1; tss_id TSS45172;
4	Cufflinks	exon	131206650	131207606	.	+	.	gene_id GRMZM2G407996; transcript_id TCONS_00021928; exon_number 2; oId CUFF.44607.1; tss_id TSS45172;

4	Cufflinks	exon	141306798	141307103	.	+	.	gene_id GRMZM2G159700; transcript_id TCONS_00021976; exon_number 1; oId CUFF.44852.1; tss_id TSS45273;
4	Cufflinks	exon	141307209	141308561	.	+	.	gene_id GRMZM2G159700; transcript_id TCONS_00021976; exon_number 2; oId CUFF.44852.1; tss_id TSS45273;
4	Cufflinks	exon	149311160	149312065	.	+	.	gene_id XLOC_019297; transcript_id TCONS_00022045; exon_number 1; oId CUFF.45080.1; tss_id TSS45399;
4	Cufflinks	exon	153654778	153655224	.	+	.	gene_id GRMZM2G392975; transcript_id TCONS_00022090; exon_number 1; oId CUFF.45236.1; tss_id TSS45473;
4	Cufflinks	exon	153656367	153656662	.	+	.	gene_id GRMZM2G392975; transcript_id TCONS_00022090; exon_number 2; oId CUFF.45236.1; tss_id TSS45473;
4	Cufflinks	exon	153657203	153657343	.	+	.	gene_id GRMZM2G392975; transcript_id TCONS_00022090; exon_number 3; oId CUFF.45236.1; tss_id TSS45473;
4	Cufflinks	exon	153657766	153659027	.	+	.	gene_id GRMZM2G392975; transcript_id TCONS_00022090; exon_number 4; oId CUFF.45236.1; tss_id TSS45473;
4	Cufflinks	exon	158166368	158167870	.	+	.	gene_id GRMZM2G054354; transcript_id TCONS_00022150; exon_number 1; oId CUFF.45400.2; tss_id TSS45579;
4	Cufflinks	exon	158168526	158168768	.	+	.	gene_id GRMZM2G054354; transcript_id TCONS_00022150; exon_number 2; oId CUFF.45400.2; tss_id TSS45579;
4	Cufflinks	exon	158169329	158170289	.	+	.	gene_id GRMZM2G054354; transcript_id TCONS_00022150; exon_number 3; oId CUFF.45400.2; tss_id TSS45579;
4	Cufflinks	exon	167105924	167108481	.	+	.	gene_id GRMZM2G014854; transcript_id TCONS_00022265; exon_number 1; oId CUFF.45832.1; tss_id TSS45789;
4	Cufflinks	exon	170880755	170882453	.	+	.	gene_id GRMZM2G146207; transcript_id TCONS_00022305; exon_number 1; oId CUFF.46023.1; tss_id TSS45877;
4	Cufflinks	exon	170884325	170885518	.	+	.	gene_id GRMZM2G146207; transcript_id TCONS_00022305; exon_number 2; oId CUFF.46023.1; tss_id TSS45877;
4	Cufflinks	exon	175765088	175765360	.	+	.	gene_id GRMZM2G181359; transcript_id TCONS_00022393; exon_number 1; oId CUFF.46433.1; tss_id TSS46068;
4	Cufflinks	exon	175765611	175765692	.	+	.	gene_id GRMZM2G181359; transcript_id TCONS_00022393; exon_number 2; oId CUFF.46433.1; tss_id TSS46068;
4	Cufflinks	exon	175766540	175766631	.	+	.	gene_id GRMZM2G181359; transcript_id TCONS_00022393; exon_number 3; oId CUFF.46433.1; tss_id TSS46068;
4	Cufflinks	exon	175766786	175766863	.	+	.	gene_id GRMZM2G181359; transcript_id TCONS_00022393; exon_number 4; oId CUFF.46433.1; tss_id TSS46068;
4	Cufflinks	exon	175766947	175767008	.	+	.	gene_id GRMZM2G181359; transcript_id TCONS_00022393; exon_number 5; oId CUFF.46433.1; tss_id TSS46068;
4	Cufflinks	exon	175767654	175767798	.	+	.	gene_id GRMZM2G181359; transcript_id TCONS_00022393; exon_number 6; oId CUFF.46433.1; tss_id TSS46068;
4	Cufflinks	exon	175767897	175768115	.	+	.	gene_id GRMZM2G181359; transcript_id TCONS_00022393; exon_number 7; oId CUFF.46433.1; tss_id TSS46068;
4	Cufflinks	exon	175768208	175768389	.	+	.	gene_id GRMZM2G181359; transcript_id TCONS_00022393; exon_number 8; oId CUFF.46433.1; tss_id TSS46068;
4	Cufflinks	exon	175768629	175768804	.	+	.	gene_id GRMZM2G181359; transcript_id TCONS_00022393; exon_number 9; oId CUFF.46433.1; tss_id TSS46068;
4	Cufflinks	exon	175768922	175769387	.	+	.	gene_id GRMZM2G181359; transcript_id TCONS_00022393; exon_number 10; oId CUFF.46433.1; tss_id TSS46068;
4	Cufflinks	exon	180679954	180681492	.	+	.	gene_id XLOC_019677; transcript_id TCONS_00022467; exon_number 1; oId CUFF.46632.1; tss_id TSS46224;
4	Cufflinks	exon	181917370	181918108	.	+	.	gene_id GRMZM2G041959; transcript_id TCONS_00022484; exon_number 1; oId CUFF.46775.1; tss_id TSS46298;
4	Cufflinks	exon	181921071	181923296	.	+	.	gene_id GRMZM2G041959; transcript_id TCONS_00022484; exon_number 2; oId CUFF.46775.1; tss_id TSS46298;
4	Cufflinks	exon	183213169	183220384	.	+	.	gene_id GRMZM2G318033; transcript_id TCONS_00022504; exon_number 1; oId CUFF.46826.1; tss_id TSS46328;
4	Cufflinks	exon	185030955	185031280	.	+	.	gene_id GRMZM2G060470; transcript_id TCONS_00022538; exon_number 1; oId CUFF.47005.1; tss_id TSS46427;
4	Cufflinks	exon	185031686	185031819	.	+	.	gene_id GRMZM2G060470; transcript_id TCONS_00022538; exon_number 2; oId CUFF.47005.1; tss_id TSS46427;
4	Cufflinks	exon	185032208	185032367	.	+	.	gene_id GRMZM2G060470; transcript_id TCONS_00022538; exon_number 3; oId CUFF.47005.1; tss_id TSS46427;
4	Cufflinks	exon	185032747	185032933	.	+	.	gene_id GRMZM2G060470; transcript_id TCONS_00022538; exon_number 4; oId CUFF.47005.1; tss_id TSS46427;
4	Cufflinks	exon	185033186	185033331	.	+	.	gene_id GRMZM2G060470; transcript_id TCONS_00022538; exon_number 5; oId CUFF.47005.1; tss_id TSS46427;
4	Cufflinks	exon	185033423	185033495	.	+	.	gene_id GRMZM2G060470; transcript_id TCONS_00022538; exon_number 6; oId CUFF.47005.1; tss_id TSS46427;
4	Cufflinks	exon	185033616	185033679	.	+	.	gene_id GRMZM2G060470; transcript_id TCONS_00022538; exon_number 7; oId CUFF.47005.1; tss_id TSS46427;
4	Cufflinks	exon	185034178	185034277	.	+	.	gene_id GRMZM2G060470; transcript_id TCONS_00022538; exon_number 8; oId CUFF.47005.1; tss_id TSS46427;
4	Cufflinks	exon	185034356	185034514	.	+	.	gene_id GRMZM2G060470; transcript_id TCONS_00022538; exon_number 9; oId CUFF.47005.1; tss_id TSS46427;

4	Cufflinks	exon	185034673	185035131	.	+	.	gene_id GRMZM2G060470; transcript_id TCONS_00022538; exon_number 10; oId CUFF.47005.1; tss_id TSS46427;
4	Cufflinks	exon	186832765	186833127	.	+	.	gene_id GRMZM2G095141; transcript_id TCONS_00022574; exon_number 1; oId CUFF.47183.1; tss_id TSS46485;
4	Cufflinks	exon	186833333	186833465	.	+	.	gene_id GRMZM2G095141; transcript_id TCONS_00022574; exon_number 2; oId CUFF.47183.1; tss_id TSS46485;
4	Cufflinks	exon	186833646	186833790	.	+	.	gene_id GRMZM2G095141; transcript_id TCONS_00022574; exon_number 3; oId CUFF.47183.1; tss_id TSS46485;
4	Cufflinks	exon	186833872	186834051	.	+	.	gene_id GRMZM2G095141; transcript_id TCONS_00022574; exon_number 4; oId CUFF.47183.1; tss_id TSS46485;
4	Cufflinks	exon	186834151	186834231	.	+	.	gene_id GRMZM2G095141; transcript_id TCONS_00022574; exon_number 5; oId CUFF.47183.1; tss_id TSS46485;
4	Cufflinks	exon	186834366	186834529	.	+	.	gene_id GRMZM2G095141; transcript_id TCONS_00022574; exon_number 6; oId CUFF.47183.1; tss_id TSS46485;
4	Cufflinks	exon	186834618	186834754	.	+	.	gene_id GRMZM2G095141; transcript_id TCONS_00022574; exon_number 7; oId CUFF.47183.1; tss_id TSS46485;
4	Cufflinks	exon	186834885	186835000	.	+	.	gene_id GRMZM2G095141; transcript_id TCONS_00022574; exon_number 8; oId CUFF.47183.1; tss_id TSS46485;
4	Cufflinks	exon	186835101	186835999	.	+	.	gene_id GRMZM2G095141; transcript_id TCONS_00022574; exon_number 9; oId CUFF.47183.1; tss_id TSS46485;
4	Cufflinks	exon	195797433	195797835	.	+	.	gene_id GRMZM2G149843; transcript_id TCONS_00022697; exon_number 1; oId CUFF.47584.1; tss_id TSS46721;
4	Cufflinks	exon	195798055	195798245	.	+	.	gene_id GRMZM2G149843; transcript_id TCONS_00022697; exon_number 2; oId CUFF.47584.1; tss_id TSS46721;
4	Cufflinks	exon	195798331	195798494	.	+	.	gene_id GRMZM2G149843; transcript_id TCONS_00022697; exon_number 3; oId CUFF.47584.1; tss_id TSS46721;
4	Cufflinks	exon	195799613	195801279	.	+	.	gene_id GRMZM2G149843; transcript_id TCONS_00022697; exon_number 4; oId CUFF.47584.1; tss_id TSS46721;
4	Cufflinks	exon	195975699	195976927	.	+	.	gene_id XLOC_019888; transcript_id TCONS_00022708; exon_number 1; oId CUFF.47594.1; tss_id TSS46731;
4	Cufflinks	exon	212126276	212126539	.	+	.	gene_id GRMZM2G014071; transcript_id TCONS_00022880; exon_number 1; oId CUFF.48196.1; tss_id TSS47046;
4	Cufflinks	exon	212126735	212127095	.	+	.	gene_id GRMZM2G014071; transcript_id TCONS_00022880; exon_number 2; oId CUFF.48196.1; tss_id TSS47046;
4	Cufflinks	exon	212128550	212129323	.	+	.	gene_id GRMZM2G014071; transcript_id TCONS_00022880; exon_number 3; oId CUFF.48196.1; tss_id TSS47046;
4	Cufflinks	exon	212129423	212130478	.	+	.	gene_id GRMZM2G014071; transcript_id TCONS_00022880; exon_number 4; oId CUFF.48196.1; tss_id TSS47046;
4	Cufflinks	exon	215442637	215445193	.	+	.	gene_id XLOC_020056; transcript_id TCONS_00022905; exon_number 1; oId CUFF.48328.1; tss_id TSS47100;
4	Cufflinks	exon	217184999	217185666	.	+	.	gene_id XLOC_020073; transcript_id TCONS_00022923; exon_number 1; oId CUFF.48365.1; tss_id TSS47137;
4	Cufflinks	exon	217186218	217186659	.	+	.	gene_id XLOC_020073; transcript_id TCONS_00022923; exon_number 2; oId CUFF.48365.1; tss_id TSS47137;
4	Cufflinks	exon	217187382	217187452	.	+	.	gene_id XLOC_020073; transcript_id TCONS_00022923; exon_number 3; oId CUFF.48365.1; tss_id TSS47137;
4	Cufflinks	exon	217187553	217187634	.	+	.	gene_id XLOC_020073; transcript_id TCONS_00022923; exon_number 4; oId CUFF.48365.1; tss_id TSS47137;
4	Cufflinks	exon	217188815	217188907	.	+	.	gene_id XLOC_020073; transcript_id TCONS_00022923; exon_number 5; oId CUFF.48365.1; tss_id TSS47137;
4	Cufflinks	exon	217189312	217189560	.	+	.	gene_id XLOC_020073; transcript_id TCONS_00022923; exon_number 6; oId CUFF.48365.1; tss_id TSS47137;
4	Cufflinks	exon	234164884	234165478	.	+	.	gene_id GRMZM2G015419; transcript_id TCONS_00023095; exon_number 1; oId CUFF.49080.1; tss_id TSS47501;
4	Cufflinks	exon	234165957	234166222	.	+	.	gene_id GRMZM2G015419; transcript_id TCONS_00023095; exon_number 2; oId CUFF.49080.1; tss_id TSS47501;
4	Cufflinks	exon	234166304	234166868	.	+	.	gene_id GRMZM2G015419; transcript_id TCONS_00023095; exon_number 3; oId CUFF.49080.1; tss_id TSS47501;
4	Cufflinks	exon	234166950	234168656	.	+	.	gene_id GRMZM2G015419; transcript_id TCONS_00023095; exon_number 4; oId CUFF.49080.1; tss_id TSS47501;
4	Cufflinks	exon	236263993	236264323	.	+	.	gene_id XLOC_020286; transcript_id TCONS_00023163; exon_number 1; oId CUFF.49260.1; tss_id TSS47589;
4	Cufflinks	exon	238874856	238875709	.	+	.	gene_id XLOC_020358; transcript_id TCONS_00023245; exon_number 1; oId CUFF.49519.1; tss_id TSS47736;
4	Cufflinks	exon	240053313	240053631	.	+	.	gene_id XLOC_020387; transcript_id TCONS_00023280; exon_number 1; oId CUFF.49665.1; tss_id TSS47796;
4	Cufflinks	exon	32440	33510	.	-	.	gene_id GRMZM2G000510; transcript_id TCONS_00023328; exon_number 1; oId CUFF.40596.1; tss_id TSS47891;
4	Cufflinks	exon	33593	33679	.	-	.	gene_id GRMZM2G000510; transcript_id TCONS_00023328; exon_number 2; oId CUFF.40596.1; tss_id TSS47891;
4	Cufflinks	exon	34480	34554	.	-	.	gene_id GRMZM2G000510; transcript_id TCONS_00023328; exon_number 3; oId CUFF.40596.1; tss_id TSS47891;

4	Cufflinks	exon	35147	35251	.	-	.	gene_id GRMZM2G000510; transcript_id TCONS_00023328; exon_number 4; oId CUFF.40596.1; tss_id TSS47891;
4	Cufflinks	exon	35335	35433	.	-	.	gene_id GRMZM2G000510; transcript_id TCONS_00023328; exon_number 5; oId CUFF.40596.1; tss_id TSS47891;
4	Cufflinks	exon	35551	35684	.	-	.	gene_id GRMZM2G000510; transcript_id TCONS_00023328; exon_number 6; oId CUFF.40596.1; tss_id TSS47891;
4	Cufflinks	exon	35888	35909	.	-	.	gene_id GRMZM2G000510; transcript_id TCONS_00023328; exon_number 7; oId CUFF.40596.1; tss_id TSS47891;
4	Cufflinks	exon	62778	62834	.	-	.	gene_id GRMZM2G000510; transcript_id TCONS_00023328; exon_number 8; oId CUFF.40596.1; tss_id TSS47891;
4	Cufflinks	exon	62978	63211	.	-	.	gene_id GRMZM2G000510; transcript_id TCONS_00023328; exon_number 9; oId CUFF.40596.1; tss_id TSS47891;
4	Cufflinks	exon	63434	63818	.	-	.	gene_id GRMZM2G000510; transcript_id TCONS_00023328; exon_number 10; oId CUFF.40596.1; tss_id TSS47891;
4	Cufflinks	exon	2431594	2433746	.	-	.	gene_id GRMZM2G448213; transcript_id TCONS_00023377; exon_number 1; oId CUFF.40663.1; tss_id TSS47957;
4	Cufflinks	exon	2611736	2612297	.	-	.	gene_id XLOC_020483; transcript_id TCONS_00023382; exon_number 1; oId CUFF.40674.1; tss_id TSS47967;
4	Cufflinks	exon	26852157	26852616	.	-	.	gene_id XLOC_020709; transcript_id TCONS_00023634; exon_number 1; oId CUFF.41771.1; tss_id TSS48541;
4	Cufflinks	exon	31276128	31277846	.	-	.	gene_id XLOC_020746; transcript_id TCONS_00023671; exon_number 1; oId CUFF.41958.1; tss_id TSS48626;
4	Cufflinks	exon	42127982	42129254	.	-	.	gene_id GRMZM2G075496; transcript_id TCONS_00023786; exon_number 1; oId CUFF.42405.1; tss_id TSS48846;
4	Cufflinks	exon	42129351	42130115	.	-	.	gene_id GRMZM2G075496; transcript_id TCONS_00023786; exon_number 2; oId CUFF.42405.1; tss_id TSS48846;
4	Cufflinks	exon	42130211	42131460	.	-	.	gene_id GRMZM2G075496; transcript_id TCONS_00023786; exon_number 3; oId CUFF.42405.1; tss_id TSS48846;
4	Cufflinks	exon	42127982	42129254	.	-	.	gene_id GRMZM2G075496; transcript_id TCONS_00023787; exon_number 1; oId CUFF.42405.2; tss_id TSS48846;
4	Cufflinks	exon	42129351	42130115	.	-	.	gene_id GRMZM2G075496; transcript_id TCONS_00023787; exon_number 2; oId CUFF.42405.2; tss_id TSS48846;
4	Cufflinks	exon	42130211	42130375	.	-	.	gene_id GRMZM2G075496; transcript_id TCONS_00023787; exon_number 3; oId CUFF.42405.2; tss_id TSS48846;
4	Cufflinks	exon	42131272	42131478	.	-	.	gene_id GRMZM2G075496; transcript_id TCONS_00023787; exon_number 4; oId CUFF.42405.2; tss_id TSS48846;
4	Cufflinks	exon	63028730	63030827	.	-	.	gene_id XLOC_020967; transcript_id TCONS_00023920; exon_number 1; oId CUFF.42934.1; tss_id TSS49101;
4	Cufflinks	exon	81729034	81729642	.	-	.	gene_id GRMZM2G091155; transcript_id TCONS_00024048; exon_number 1; oId CUFF.43463.1; tss_id TSS49334;
4	Cufflinks	exon	81729746	81729862	.	-	.	gene_id GRMZM2G091155; transcript_id TCONS_00024048; exon_number 2; oId CUFF.43463.1; tss_id TSS49334;
4	Cufflinks	exon	81730461	81730583	.	-	.	gene_id GRMZM2G091155; transcript_id TCONS_00024048; exon_number 3; oId CUFF.43463.1; tss_id TSS49334;
4	Cufflinks	exon	81730729	81730807	.	-	.	gene_id GRMZM2G091155; transcript_id TCONS_00024048; exon_number 4; oId CUFF.43463.1; tss_id TSS49334;
4	Cufflinks	exon	81730914	81731326	.	-	.	gene_id GRMZM2G091155; transcript_id TCONS_00024048; exon_number 5; oId CUFF.43463.1; tss_id TSS49334;
4	Cufflinks	exon	81732467	81732613	.	-	.	gene_id GRMZM2G091155; transcript_id TCONS_00024048; exon_number 6; oId CUFF.43463.1; tss_id TSS49334;
4	Cufflinks	exon	116891237	116892701	.	-	.	gene_id GRMZM2G147279; transcript_id TCONS_00024197; exon_number 1; oId CUFF.44228.1; tss_id TSS49720;
4	Cufflinks	exon	116892844	116892978	.	-	.	gene_id GRMZM2G147279; transcript_id TCONS_00024197; exon_number 2; oId CUFF.44228.1; tss_id TSS49720;
4	Cufflinks	exon	116893133	116893515	.	-	.	gene_id GRMZM2G147279; transcript_id TCONS_00024197; exon_number 3; oId CUFF.44228.1; tss_id TSS49720;
4	Cufflinks	exon	135677987	135678739	.	-	.	gene_id GRMZM2G107896; transcript_id TCONS_00024317; exon_number 1; oId CUFF.44761.1; tss_id TSS49984;
4	Cufflinks	exon	135679429	135679678	.	-	.	gene_id GRMZM2G107896; transcript_id TCONS_00024317; exon_number 2; oId CUFF.44761.1; tss_id TSS49984;
4	Cufflinks	exon	135704617	135704705	.	-	.	gene_id GRMZM2G107896; transcript_id TCONS_00024317; exon_number 3; oId CUFF.44761.1; tss_id TSS49984;
4	Cufflinks	exon	135706780	135706877	.	-	.	gene_id GRMZM2G107896; transcript_id TCONS_00024317; exon_number 4; oId CUFF.44761.1; tss_id TSS49984;
4	Cufflinks	exon	135707051	135707206	.	-	.	gene_id GRMZM2G107896; transcript_id TCONS_00024317; exon_number 5; oId CUFF.44761.1; tss_id TSS49984;
4	Cufflinks	exon	143491590	143493973	.	-	.	gene_id GRMZM2G081582; transcript_id TCONS_00024357; exon_number 1; oId CUFF.44902.1; tss_id TSS50080;
4	Cufflinks	exon	143496369	143497192	.	-	.	gene_id GRMZM2G081582; transcript_id TCONS_00024357; exon_number 2; oId CUFF.44902.1; tss_id TSS50080;
4	Cufflinks	exon	152813342	152814079	.	-	.	gene_id GRMZM2G102502; transcript_id TCONS_00024419; exon_number 1; oId CUFF.45148.1; tss_id TSS50209;

4	Cufflinks	exon	152815160	152815476	.	-	.	gene_id GRMZM2G102502; transcript_id TCONS_00024419; exon_number 2; oId CUFF.45148.1; tss_id TSS50209;
4	Cufflinks	exon	152817609	152817882	.	-	.	gene_id GRMZM2G102502; transcript_id TCONS_00024419; exon_number 3; oId CUFF.45148.1; tss_id TSS50209;
4	Cufflinks	exon	152817973	152818202	.	-	.	gene_id GRMZM2G102502; transcript_id TCONS_00024419; exon_number 4; oId CUFF.45148.1; tss_id TSS50209;
4	Cufflinks	exon	156217617	156219742	.	-	.	gene_id GRMZM2G134023; transcript_id TCONS_00024456; exon_number 1; oId CUFF.45281.1; tss_id TSS50280;
4	Cufflinks	exon	156443715	156445719	.	-	.	gene_id XLOC_021429; transcript_id TCONS_00024459; exon_number 1; oId CUFF.45292.1; tss_id TSS50286;
4	Cufflinks	exon	158166397	158166669	.	-	.	gene_id XLOC_021449; transcript_id TCONS_00024481; exon_number 1; oId CUFF.45401.1; tss_id TSS50342;
4	Cufflinks	exon	158167346	158167576	.	-	.	gene_id XLOC_021449; transcript_id TCONS_00024481; exon_number 2; oId CUFF.45401.1; tss_id TSS50342;
4	Cufflinks	exon	158370468	158372295	.	-	.	gene_id XLOC_021453; transcript_id TCONS_00024485; exon_number 1; oId CUFF.45406.1; tss_id TSS50349;
4	Cufflinks	exon	167111209	167111781	.	-	.	gene_id GRMZM2G036464; transcript_id TCONS_00024592; exon_number 1; oId CUFF.45836.1; tss_id TSS50587;
4	Cufflinks	exon	167111870	167112121	.	-	.	gene_id GRMZM2G036464; transcript_id TCONS_00024592; exon_number 2; oId CUFF.45836.1; tss_id TSS50587;
4	Cufflinks	exon	167112233	167112307	.	-	.	gene_id GRMZM2G036464; transcript_id TCONS_00024592; exon_number 3; oId CUFF.45836.1; tss_id TSS50587;
4	Cufflinks	exon	167112400	167112528	.	-	.	gene_id GRMZM2G036464; transcript_id TCONS_00024592; exon_number 4; oId CUFF.45836.1; tss_id TSS50587;
4	Cufflinks	exon	167112614	167112701	.	-	.	gene_id GRMZM2G036464; transcript_id TCONS_00024592; exon_number 5; oId CUFF.45836.1; tss_id TSS50587;
4	Cufflinks	exon	167112802	167112908	.	-	.	gene_id GRMZM2G036464; transcript_id TCONS_00024592; exon_number 6; oId CUFF.45836.1; tss_id TSS50587;
4	Cufflinks	exon	167113355	167113403	.	-	.	gene_id GRMZM2G036464; transcript_id TCONS_00024592; exon_number 7; oId CUFF.45836.1; tss_id TSS50587;
4	Cufflinks	exon	167113489	167113592	.	-	.	gene_id GRMZM2G036464; transcript_id TCONS_00024592; exon_number 8; oId CUFF.45836.1; tss_id TSS50587;
4	Cufflinks	exon	167113808	167113847	.	-	.	gene_id GRMZM2G036464; transcript_id TCONS_00024592; exon_number 9; oId CUFF.45836.1; tss_id TSS50587;
4	Cufflinks	exon	167114329	167114536	.	-	.	gene_id GRMZM2G036464; transcript_id TCONS_00024592; exon_number 10; oId CUFF.45836.1; tss_id TSS50587;
4	Cufflinks	exon	171951028	171952275	.	-	.	gene_id GRMZM2G164854; transcript_id TCONS_00024672; exon_number 1; oId CUFF.46132.1; tss_id TSS50731;
4	Cufflinks	exon	171952524	171953143	.	-	.	gene_id GRMZM2G164854; transcript_id TCONS_00024672; exon_number 2; oId CUFF.46132.1; tss_id TSS50731;
4	Cufflinks	exon	171953245	171955087	.	-	.	gene_id GRMZM2G164854; transcript_id TCONS_00024672; exon_number 3; oId CUFF.46132.1; tss_id TSS50731;
4	Cufflinks	exon	174991068	174992665	.	-	.	gene_id GRMZM2G312521; transcript_id TCONS_00024720; exon_number 1; oId CUFF.46365.1; tss_id TSS50859;
4	Cufflinks	exon	174993121	174993394	.	-	.	gene_id GRMZM2G312521; transcript_id TCONS_00024720; exon_number 2; oId CUFF.46365.1; tss_id TSS50859;
4	Cufflinks	exon	174993936	174996208	.	-	.	gene_id GRMZM2G312521; transcript_id TCONS_00024720; exon_number 3; oId CUFF.46365.1; tss_id TSS50859;
4	Cufflinks	exon	174991068	174993394	.	-	.	gene_id GRMZM2G312521; transcript_id TCONS_00024721; exon_number 1; oId CUFF.46365.2; tss_id TSS50859;
4	Cufflinks	exon	174993936	174996208	.	-	.	gene_id GRMZM2G312521; transcript_id TCONS_00024721; exon_number 2; oId CUFF.46365.2; tss_id TSS50859;
4	Cufflinks	exon	175361439	175361691	.	-	.	gene_id XLOC_021670; transcript_id TCONS_00024724; exon_number 1; oId CUFF.46341.1; tss_id TSS50866;
4	Cufflinks	exon	180401112	180402233	.	-	.	gene_id GRMZM2G041699; transcript_id TCONS_00024800; exon_number 1; oId CUFF.46621.1; tss_id TSS51022;
4	Cufflinks	exon	180402366	180403035	.	-	.	gene_id GRMZM2G041699; transcript_id TCONS_00024800; exon_number 2; oId CUFF.46621.1; tss_id TSS51022;
4	Cufflinks	exon	180464258	180465403	.	-	.	gene_id GRMZM2G169617; transcript_id TCONS_00024806; exon_number 1; oId CUFF.46683.4; tss_id TSS51026;
4	Cufflinks	exon	180465716	180466342	.	-	.	gene_id GRMZM2G169617; transcript_id TCONS_00024806; exon_number 2; oId CUFF.46683.4; tss_id TSS51026;
4	Cufflinks	exon	180471902	180472043	.	-	.	gene_id GRMZM2G169617; transcript_id TCONS_00024806; exon_number 3; oId CUFF.46683.4; tss_id TSS51026;
4	Cufflinks	exon	180473669	180473792	.	-	.	gene_id GRMZM2G169617; transcript_id TCONS_00024806; exon_number 4; oId CUFF.46683.4; tss_id TSS51026;
4	Cufflinks	exon	180473923	180475317	.	-	.	gene_id GRMZM2G169617; transcript_id TCONS_00024806; exon_number 5; oId CUFF.46683.4; tss_id TSS51026;
4	Cufflinks	exon	180464258	180465403	.	-	.	gene_id GRMZM2G169617; transcript_id TCONS_00024807; exon_number 1; oId CUFF.46683.2; tss_id TSS51025;
4	Cufflinks	exon	180465716	180465974	.	-	.	gene_id GRMZM2G169617; transcript_id TCONS_00024807; exon_number 2; oId CUFF.46683.2; tss_id TSS51025;

4	Cufflinks	exon	180466309	180466342	.	-	.	gene_id GRMZM2G169617; transcript_id TCONS_00024807; exon_number 3; oId CUFF.46683.2; tss_id TSS51025;
4	Cufflinks	exon	180471902	180472043	.	-	.	gene_id GRMZM2G169617; transcript_id TCONS_00024807; exon_number 4; oId CUFF.46683.2; tss_id TSS51025;
4	Cufflinks	exon	180473669	180473792	.	-	.	gene_id GRMZM2G169617; transcript_id TCONS_00024807; exon_number 5; oId CUFF.46683.2; tss_id TSS51025;
4	Cufflinks	exon	180473923	180474697	.	-	.	gene_id GRMZM2G169617; transcript_id TCONS_00024807; exon_number 6; oId CUFF.46683.2; tss_id TSS51025;
4	Cufflinks	exon	180679873	180681240	.	-	.	gene_id GRMZM2G322950; transcript_id TCONS_00024811; exon_number 1; oId CUFF.46630.1; tss_id TSS51031;
4	Cufflinks	exon	181921872	181922751	.	-	.	gene_id XLOC_021759; transcript_id TCONS_00024835; exon_number 1; oId CUFF.46777.1; tss_id TSS51088;
4	Cufflinks	exon	183213856	183214933	.	-	.	gene_id GRMZM2G020996; transcript_id TCONS_00024847; exon_number 1; oId CUFF.46830.1; tss_id TSS51108;
4	Cufflinks	exon	183215007	183215788	.	-	.	gene_id GRMZM2G020996; transcript_id TCONS_00024847; exon_number 2; oId CUFF.46830.1; tss_id TSS51108;
4	Cufflinks	exon	183224182	183225003	.	-	.	gene_id GRMZM2G020996; transcript_id TCONS_00024847; exon_number 3; oId CUFF.46830.1; tss_id TSS51108;
4	Cufflinks	exon	184149627	184150231	.	-	.	gene_id XLOC_021790; transcript_id TCONS_00024872; exon_number 1; oId CUFF.46874.1; tss_id TSS51162;
4	Cufflinks	exon	185209930	185210811	.	-	.	gene_id GRMZM2G151516; transcript_id TCONS_00024895; exon_number 1; oId CUFF.47018.2; tss_id TSS51208;
4	Cufflinks	exon	185210954	185211510	.	-	.	gene_id GRMZM2G151516; transcript_id TCONS_00024895; exon_number 2; oId CUFF.47018.2; tss_id TSS51208;
4	Cufflinks	exon	185209930	185210871	.	-	.	gene_id GRMZM2G151516; transcript_id TCONS_00024896; exon_number 1; oId CUFF.47018.3; tss_id TSS51208;
4	Cufflinks	exon	185210954	185211510	.	-	.	gene_id GRMZM2G151516; transcript_id TCONS_00024896; exon_number 2; oId CUFF.47018.3; tss_id TSS51208;
4	Cufflinks	exon	195800111	195801572	.	-	.	gene_id GRMZM2G451366; transcript_id TCONS_00025076; exon_number 1; oId CUFF.47587.1; tss_id TSS51517;
4	Cufflinks	exon	195801661	195801750	.	-	.	gene_id GRMZM2G451366; transcript_id TCONS_00025076; exon_number 2; oId CUFF.47587.1; tss_id TSS51517;
4	Cufflinks	exon	195801837	195801938	.	-	.	gene_id GRMZM2G451366; transcript_id TCONS_00025076; exon_number 3; oId CUFF.47587.1; tss_id TSS51517;
4	Cufflinks	exon	195802019	195802186	.	-	.	gene_id GRMZM2G451366; transcript_id TCONS_00025076; exon_number 4; oId CUFF.47587.1; tss_id TSS51517;
4	Cufflinks	exon	195802389	195802449	.	-	.	gene_id GRMZM2G451366; transcript_id TCONS_00025076; exon_number 5; oId CUFF.47587.1; tss_id TSS51517;
4	Cufflinks	exon	195802544	195802656	.	-	.	gene_id GRMZM2G451366; transcript_id TCONS_00025076; exon_number 6; oId CUFF.47587.1; tss_id TSS51517;
4	Cufflinks	exon	195803004	195803081	.	-	.	gene_id GRMZM2G451366; transcript_id TCONS_00025076; exon_number 7; oId CUFF.47587.1; tss_id TSS51517;
4	Cufflinks	exon	195803174	195803314	.	-	.	gene_id GRMZM2G451366; transcript_id TCONS_00025076; exon_number 8; oId CUFF.47587.1; tss_id TSS51517;
4	Cufflinks	exon	195803406	195803570	.	-	.	gene_id GRMZM2G451366; transcript_id TCONS_00025076; exon_number 9; oId CUFF.47587.1; tss_id TSS51517;
4	Cufflinks	exon	195803648	195803740	.	-	.	gene_id GRMZM2G451366; transcript_id TCONS_00025076; exon_number 10; oId CUFF.47587.1; tss_id TSS51517;
4	Cufflinks	exon	195803955	195804021	.	-	.	gene_id GRMZM2G451366; transcript_id TCONS_00025076; exon_number 11; oId CUFF.47587.1; tss_id TSS51517;
4	Cufflinks	exon	195804162	195804265	.	-	.	gene_id GRMZM2G451366; transcript_id TCONS_00025076; exon_number 12; oId CUFF.47587.1; tss_id TSS51517;
4	Cufflinks	exon	195804611	195804756	.	-	.	gene_id GRMZM2G451366; transcript_id TCONS_00025076; exon_number 13; oId CUFF.47587.1; tss_id TSS51517;
4	Cufflinks	exon	195805283	195805721	.	-	.	gene_id GRMZM2G451366; transcript_id TCONS_00025076; exon_number 14; oId CUFF.47587.1; tss_id TSS51517;
4	Cufflinks	exon	197104888	197106605	.	-	.	gene_id XLOC_021978; transcript_id TCONS_00025091; exon_number 1; oId CUFF.47613.1; tss_id TSS51550;
4	Cufflinks	exon	204522509	204523059	.	-	.	gene_id AC216067.3_FG002; transcript_id TCONS_00025189; exon_number 1; oId CUFF.47974.1; tss_id TSS51748;
4	Cufflinks	exon	204524238	204524523	.	-	.	gene_id AC216067.3_FG002; transcript_id TCONS_00025189; exon_number 2; oId CUFF.47974.1; tss_id TSS51748;
4	Cufflinks	exon	204524846	204525059	.	-	.	gene_id AC216067.3_FG002; transcript_id TCONS_00025189; exon_number 3; oId CUFF.47974.1; tss_id TSS51748;
4	Cufflinks	exon	231270481	231270977	.	-	.	gene_id XLOC_022280; transcript_id TCONS_00025433; exon_number 1; oId CUFF.48951.1; tss_id TSS52239;
4	Cufflinks	exon	231271965	231272402	.	-	.	gene_id XLOC_022280; transcript_id TCONS_00025433; exon_number 2; oId CUFF.48951.1; tss_id TSS52239;
5	Cufflinks	exon	1524444	1524718	.	+	.	gene_id GRMZM2G447976; transcript_id TCONS_00025785; exon_number 1; oId CUFF.49992.1; tss_id TSS52817;
5	Cufflinks	exon	1525788	1527639	.	+	.	gene_id GRMZM2G447976; transcript_id TCONS_00025785; exon_number 2; oId CUFF.49992.1; tss_id TSS52817;

5	Cufflinks	exon	1545003	1545310	.	+	.	gene_id XLOC_022572; transcript_id TCONS_00025788; exon_number 1; oId CUFF.49981.1; tss_id TSS52820;
5	Cufflinks	exon	4606404	4608549	.	+	.	gene_id GRMZM2G124229; transcript_id TCONS_00025918; exon_number 1; oId CUFF.50413.1; tss_id TSS53047;
5	Cufflinks	exon	6914005	6914427	.	+	.	gene_id GRMZM2G071970; transcript_id TCONS_00026005; exon_number 1; oId CUFF.50706.1; tss_id TSS53198;
5	Cufflinks	exon	6915164	6915243	.	+	.	gene_id GRMZM2G071970; transcript_id TCONS_00026005; exon_number 2; oId CUFF.50706.1; tss_id TSS53198;
5	Cufflinks	exon	6915477	6915933	.	+	.	gene_id GRMZM2G071970; transcript_id TCONS_00026005; exon_number 3; oId CUFF.50706.1; tss_id TSS53198;
5	Cufflinks	exon	6916310	6916463	.	+	.	gene_id GRMZM2G071970; transcript_id TCONS_00026005; exon_number 4; oId CUFF.50706.1; tss_id TSS53198;
5	Cufflinks	exon	6916550	6916830	.	+	.	gene_id GRMZM2G071970; transcript_id TCONS_00026005; exon_number 5; oId CUFF.50706.1; tss_id TSS53198;
5	Cufflinks	exon	6916919	6917726	.	+	.	gene_id GRMZM2G071970; transcript_id TCONS_00026005; exon_number 6; oId CUFF.50706.1; tss_id TSS53198;
5	Cufflinks	exon	10796396	10796572	.	+	.	gene_id GRMZM2G145850; transcript_id TCONS_00026086; exon_number 1; oId CUFF.51012.1; tss_id TSS53323;
5	Cufflinks	exon	10796668	10797296	.	+	.	gene_id GRMZM2G145850; transcript_id TCONS_00026086; exon_number 2; oId CUFF.51012.1; tss_id TSS53323;
5	Cufflinks	exon	12237732	12238164	.	+	.	gene_id XLOC_022856; transcript_id TCONS_00026116; exon_number 1; oId CUFF.51064.1; tss_id TSS53384;
5	Cufflinks	exon	12289155	12289430	.	+	.	gene_id XLOC_022858; transcript_id TCONS_00026118; exon_number 1; oId CUFF.51106.1; tss_id TSS53391;
5	Cufflinks	exon	14609902	14610741	.	+	.	gene_id XLOC_022911; transcript_id TCONS_00026177; exon_number 1; oId CUFF.51258.1; tss_id TSS53503;
5	Cufflinks	exon	14870816	14871240	.	+	.	gene_id XLOC_022917; transcript_id TCONS_00026184; exon_number 1; oId CUFF.51288.1; tss_id TSS53521;
5	Cufflinks	exon	20929481	20930339	.	+	.	gene_id XLOC_023002; transcript_id TCONS_00026279; exon_number 1; oId CUFF.51597.1; tss_id TSS53688;
5	Cufflinks	exon	21362862	21363809	.	+	.	gene_id GRMZM5G851862; transcript_id TCONS_00026290; exon_number 1; oId CUFF.51641.1; tss_id TSS53730;
5	Cufflinks	exon	21365017	21366235	.	+	.	gene_id GRMZM5G851862; transcript_id TCONS_00026290; exon_number 2; oId CUFF.51641.1; tss_id TSS53730;
5	Cufflinks	exon	35290719	35291061	.	+	.	gene_id GRMZM2G017805; transcript_id TCONS_00026458; exon_number 1; oId CUFF.52373.1; tss_id TSS54136;
5	Cufflinks	exon	35291249	35295480	.	+	.	gene_id GRMZM2G017805; transcript_id TCONS_00026458; exon_number 2; oId CUFF.52373.1; tss_id TSS54136;
5	Cufflinks	exon	35731107	35732791	.	+	.	gene_id GRMZM2G043182; transcript_id TCONS_00026463; exon_number 1; oId CUFF.52398.1; tss_id TSS54143;
5	Cufflinks	exon	50479714	50480691	.	+	.	gene_id XLOC_023268; transcript_id TCONS_00026590; exon_number 1; oId CUFF.52928.1; tss_id TSS54439;
5	Cufflinks	exon	53935017	53935504	.	+	.	gene_id GRMZM2G026980; transcript_id TCONS_00026627; exon_number 1; oId CUFF.53116.1; tss_id TSS54554;
5	Cufflinks	exon	53935630	53936879	.	+	.	gene_id GRMZM2G026980; transcript_id TCONS_00026627; exon_number 2; oId CUFF.53116.1; tss_id TSS54554;
5	Cufflinks	exon	59063729	59064198	.	+	.	gene_id GRMZM2G135904; transcript_id TCONS_00026673; exon_number 1; oId CUFF.53357.1; tss_id TSS54674;
5	Cufflinks	exon	59065133	59065541	.	+	.	gene_id GRMZM2G135904; transcript_id TCONS_00026673; exon_number 2; oId CUFF.53357.1; tss_id TSS54674;
5	Cufflinks	exon	59065701	59065828	.	+	.	gene_id GRMZM2G135904; transcript_id TCONS_00026673; exon_number 3; oId CUFF.53357.1; tss_id TSS54674;
5	Cufflinks	exon	59065908	59065967	.	+	.	gene_id GRMZM2G135904; transcript_id TCONS_00026673; exon_number 4; oId CUFF.53357.1; tss_id TSS54674;
5	Cufflinks	exon	59066245	59066394	.	+	.	gene_id GRMZM2G135904; transcript_id TCONS_00026673; exon_number 5; oId CUFF.53357.1; tss_id TSS54674;
5	Cufflinks	exon	59066857	59067012	.	+	.	gene_id GRMZM2G135904; transcript_id TCONS_00026673; exon_number 6; oId CUFF.53357.1; tss_id TSS54674;
5	Cufflinks	exon	59067132	59067269	.	+	.	gene_id GRMZM2G135904; transcript_id TCONS_00026673; exon_number 7; oId CUFF.53357.1; tss_id TSS54674;
5	Cufflinks	exon	59067369	59067442	.	+	.	gene_id GRMZM2G135904; transcript_id TCONS_00026673; exon_number 8; oId CUFF.53357.1; tss_id TSS54674;
5	Cufflinks	exon	59067892	59067993	.	+	.	gene_id GRMZM2G135904; transcript_id TCONS_00026673; exon_number 9; oId CUFF.53357.1; tss_id TSS54674;
5	Cufflinks	exon	59068077	59068302	.	+	.	gene_id GRMZM2G135904; transcript_id TCONS_00026673; exon_number 10; oId CUFF.53357.1; tss_id TSS54674;
5	Cufflinks	exon	59068553	59069485	.	+	.	gene_id GRMZM2G135904; transcript_id TCONS_00026673; exon_number 11; oId CUFF.53357.1; tss_id TSS54674;
5	Cufflinks	exon	59299608	59301346	.	+	.	gene_id GRMZM2G385945; transcript_id TCONS_00026677; exon_number 1; oId CUFF.53314.1; tss_id TSS54682;
5	Cufflinks	exon	61454332	61455153	.	+	.	gene_id XLOC_023371; transcript_id TCONS_00026709; exon_number 1; oId CUFF.53448.1; tss_id TSS54733;

5	Cufflinks	exon	61455341	61455410	.	+	.	gene_id XLOC_023371; transcript_id TCONS_00026709; exon_number 2; oId CUFF.53448.1; tss_id TSS54733;
5	Cufflinks	exon	61455483	61455583	.	+	.	gene_id XLOC_023371; transcript_id TCONS_00026709; exon_number 3; oId CUFF.53448.1; tss_id TSS54733;
5	Cufflinks	exon	61455934	61456050	.	+	.	gene_id XLOC_023371; transcript_id TCONS_00026709; exon_number 4; oId CUFF.53448.1; tss_id TSS54733;
5	Cufflinks	exon	61456233	61456309	.	+	.	gene_id XLOC_023371; transcript_id TCONS_00026709; exon_number 5; oId CUFF.53448.1; tss_id TSS54733;
5	Cufflinks	exon	61456417	61456461	.	+	.	gene_id XLOC_023371; transcript_id TCONS_00026709; exon_number 6; oId CUFF.53448.1; tss_id TSS54733;
5	Cufflinks	exon	61456600	61457289	.	+	.	gene_id XLOC_023371; transcript_id TCONS_00026709; exon_number 7; oId CUFF.53448.1; tss_id TSS54733;
5	Cufflinks	exon	62625055	62625575	.	+	.	gene_id XLOC_023388; transcript_id TCONS_00026731; exon_number 1; oId CUFF.53510.1; tss_id TSS54763;
5	Cufflinks	exon	62625722	62626051	.	+	.	gene_id XLOC_023388; transcript_id TCONS_00026731; exon_number 2; oId CUFF.53510.1; tss_id TSS54763;
5	Cufflinks	exon	63305844	63307092	.	+	.	gene_id XLOC_023396; transcript_id TCONS_00026739; exon_number 1; oId CUFF.53508.1; tss_id TSS54769;
5	Cufflinks	exon	65138689	65138879	.	+	.	gene_id GRMZM2G057000; transcript_id TCONS_00026756; exon_number 1; oId CUFF.53598.1; tss_id TSS54806;
5	Cufflinks	exon	65140549	65141805	.	+	.	gene_id GRMZM2G057000; transcript_id TCONS_00026756; exon_number 2; oId CUFF.53598.1; tss_id TSS54806;
5	Cufflinks	exon	65141897	65142574	.	+	.	gene_id GRMZM2G057000; transcript_id TCONS_00026756; exon_number 3; oId CUFF.53598.1; tss_id TSS54806;
5	Cufflinks	exon	65230942	65231216	.	+	.	gene_id GRMZM2G055682; transcript_id TCONS_00026760; exon_number 1; oId CUFF.53627.1; tss_id TSS54810;
5	Cufflinks	exon	65231882	65232001	.	+	.	gene_id GRMZM2G055682; transcript_id TCONS_00026760; exon_number 2; oId CUFF.53627.1; tss_id TSS54810;
5	Cufflinks	exon	65232263	65232398	.	+	.	gene_id GRMZM2G055682; transcript_id TCONS_00026760; exon_number 3; oId CUFF.53627.1; tss_id TSS54810;
5	Cufflinks	exon	65232496	65232610	.	+	.	gene_id GRMZM2G055682; transcript_id TCONS_00026760; exon_number 4; oId CUFF.53627.1; tss_id TSS54810;
5	Cufflinks	exon	65232696	65233276	.	+	.	gene_id GRMZM2G055682; transcript_id TCONS_00026760; exon_number 5; oId CUFF.53627.1; tss_id TSS54810;
5	Cufflinks	exon	70795837	70796455	.	+	.	gene_id GRMZM2G466281; transcript_id TCONS_00026831; exon_number 1; oId CUFF.53936.1; tss_id TSS54958;
5	Cufflinks	exon	70797019	70797028	.	+	.	gene_id GRMZM2G466281; transcript_id TCONS_00026831; exon_number 2; oId CUFF.53936.1; tss_id TSS54958;
5	Cufflinks	exon	70797111	70797161	.	+	.	gene_id GRMZM2G466281; transcript_id TCONS_00026831; exon_number 3; oId CUFF.53936.1; tss_id TSS54958;
5	Cufflinks	exon	70797315	70797392	.	+	.	gene_id GRMZM2G466281; transcript_id TCONS_00026831; exon_number 4; oId CUFF.53936.1; tss_id TSS54958;
5	Cufflinks	exon	70797625	70797700	.	+	.	gene_id GRMZM2G466281; transcript_id TCONS_00026831; exon_number 5; oId CUFF.53936.1; tss_id TSS54958;
5	Cufflinks	exon	70797783	70797805	.	+	.	gene_id GRMZM2G466281; transcript_id TCONS_00026831; exon_number 6; oId CUFF.53936.1; tss_id TSS54958;
5	Cufflinks	exon	70797886	70797986	.	+	.	gene_id GRMZM2G466281; transcript_id TCONS_00026831; exon_number 7; oId CUFF.53936.1; tss_id TSS54958;
5	Cufflinks	exon	70798567	70798661	.	+	.	gene_id GRMZM2G466281; transcript_id TCONS_00026831; exon_number 8; oId CUFF.53936.1; tss_id TSS54958;
5	Cufflinks	exon	70798827	70798895	.	+	.	gene_id GRMZM2G466281; transcript_id TCONS_00026831; exon_number 9; oId CUFF.53936.1; tss_id TSS54958;
5	Cufflinks	exon	70799083	70799152	.	+	.	gene_id GRMZM2G466281; transcript_id TCONS_00026831; exon_number 10; oId CUFF.53936.1; tss_id TSS54958;
5	Cufflinks	exon	70799966	70800029	.	+	.	gene_id GRMZM2G466281; transcript_id TCONS_00026831; exon_number 11; oId CUFF.53936.1; tss_id TSS54958;
5	Cufflinks	exon	70800110	70800150	.	+	.	gene_id GRMZM2G466281; transcript_id TCONS_00026831; exon_number 12; oId CUFF.53936.1; tss_id TSS54958;
5	Cufflinks	exon	70800299	70800383	.	+	.	gene_id GRMZM2G466281; transcript_id TCONS_00026831; exon_number 13; oId CUFF.53936.1; tss_id TSS54958;
5	Cufflinks	exon	70800641	70800703	.	+	.	gene_id GRMZM2G466281; transcript_id TCONS_00026831; exon_number 14; oId CUFF.53936.1; tss_id TSS54958;
5	Cufflinks	exon	70800796	70800854	.	+	.	gene_id GRMZM2G466281; transcript_id TCONS_00026831; exon_number 15; oId CUFF.53936.1; tss_id TSS54958;
5	Cufflinks	exon	70800942	70800994	.	+	.	gene_id GRMZM2G466281; transcript_id TCONS_00026831; exon_number 16; oId CUFF.53936.1; tss_id TSS54958;
5	Cufflinks	exon	70801813	70801885	.	+	.	gene_id GRMZM2G466281; transcript_id TCONS_00026831; exon_number 17; oId CUFF.53936.1; tss_id TSS54958;
5	Cufflinks	exon	70801963	70802844	.	+	.	gene_id GRMZM2G466281; transcript_id TCONS_00026831; exon_number 18; oId CUFF.53936.1; tss_id TSS54958;
5	Cufflinks	exon	76249702	76250026	.	+	.	gene_id AC192244.3_FG007; transcript_id TCONS_00026882; exon_number 1; oId CUFF.54126.1; tss_id TSS55066;

5	Cufflinks	exon	76250306	76250377	.	+	.	gene_id AC192244.3_FG007; transcript_id TCONS_00026882; exon_number 2; oId CUFF.54126.1; tss_id TSS55066;
5	Cufflinks	exon	76251560	76251619	.	+	.	gene_id AC192244.3_FG007; transcript_id TCONS_00026882; exon_number 3; oId CUFF.54126.1; tss_id TSS55066;
5	Cufflinks	exon	76251780	76251874	.	+	.	gene_id AC192244.3_FG007; transcript_id TCONS_00026882; exon_number 4; oId CUFF.54126.1; tss_id TSS55066;
5	Cufflinks	exon	76251955	76252024	.	+	.	gene_id AC192244.3_FG007; transcript_id TCONS_00026882; exon_number 5; oId CUFF.54126.1; tss_id TSS55066;
5	Cufflinks	exon	76252105	76252730	.	+	.	gene_id AC192244.3_FG007; transcript_id TCONS_00026882; exon_number 6; oId CUFF.54126.1; tss_id TSS55066;
5	Cufflinks	exon	81799333	81801056	.	+	.	gene_id XLOC_023583; transcript_id TCONS_00026955; exon_number 1; oId CUFF.54305.1; tss_id TSS55206;
5	Cufflinks	exon	87452593	87453027	.	+	.	gene_id GRMZM2G138527; transcript_id TCONS_00027012; exon_number 1; oId CUFF.54548.1; tss_id TSS55345;
5	Cufflinks	exon	87456748	87457026	.	+	.	gene_id GRMZM2G138527; transcript_id TCONS_00027012; exon_number 2; oId CUFF.54548.1; tss_id TSS55345;
5	Cufflinks	exon	92841722	92842097	.	+	.	gene_id GRMZM2G120320; transcript_id TCONS_00027038; exon_number 1; oId CUFF.54716.1; tss_id TSS55432;
5	Cufflinks	exon	92842232	92844459	.	+	.	gene_id GRMZM2G120320; transcript_id TCONS_00027038; exon_number 2; oId CUFF.54716.1; tss_id TSS55432;
5	Cufflinks	exon	106298211	106299383	.	+	.	gene_id GRMZM2G344163; transcript_id TCONS_00027091; exon_number 1; oId CUFF.55037.1; tss_id TSS55620;
5	Cufflinks	exon	106299499	106300636	.	+	.	gene_id GRMZM2G344163; transcript_id TCONS_00027091; exon_number 2; oId CUFF.55037.1; tss_id TSS55620;
5	Cufflinks	exon	106300724	106301969	.	+	.	gene_id GRMZM2G344163; transcript_id TCONS_00027091; exon_number 3; oId CUFF.55037.1; tss_id TSS55620;
5	Cufflinks	exon	146922694	146923762	.	+	.	gene_id GRMZM2G129713; transcript_id TCONS_00027285; exon_number 1; oId CUFF.56036.1; tss_id TSS56116;
5	Cufflinks	exon	162094523	162094828	.	+	.	gene_id GRMZM2G149115; transcript_id TCONS_00027391; exon_number 1; oId CUFF.56501.1; tss_id TSS56333;
5	Cufflinks	exon	162097538	162098211	.	+	.	gene_id GRMZM2G149115; transcript_id TCONS_00027391; exon_number 2; oId CUFF.56501.1; tss_id TSS56333;
5	Cufflinks	exon	162742238	162743559	.	+	.	gene_id XLOC_023995; transcript_id TCONS_00027398; exon_number 1; oId CUFF.56563.1; tss_id TSS56345;
5	Cufflinks	exon	163455309	163455697	.	+	.	gene_id XLOC_024013; transcript_id TCONS_00027418; exon_number 1; oId CUFF.56616.1; tss_id TSS56382;
5	Cufflinks	exon	164769816	164770205	.	+	.	gene_id XLOC_024036; transcript_id TCONS_00027441; exon_number 1; oId CUFF.56662.3; tss_id TSS56404;
5	Cufflinks	exon	164770284	164771069	.	+	.	gene_id XLOC_024036; transcript_id TCONS_00027441; exon_number 2; oId CUFF.56662.3; tss_id TSS56404;
5	Cufflinks	exon	170545309	170545691	.	+	.	gene_id XLOC_024088; transcript_id TCONS_00027501; exon_number 1; oId CUFF.56846.1; tss_id TSS56515;
5	Cufflinks	exon	172660909	172661295	.	+	.	gene_id GRMZM2G031581; transcript_id TCONS_00027526; exon_number 1; oId CUFF.56937.1; tss_id TSS56567;
5	Cufflinks	exon	172661406	172663831	.	+	.	gene_id GRMZM2G031581; transcript_id TCONS_00027526; exon_number 2; oId CUFF.56937.1; tss_id TSS56567;
5	Cufflinks	exon	175735743	175736375	.	+	.	gene_id XLOC_024153; transcript_id TCONS_00027581; exon_number 1; oId CUFF.57151.1; tss_id TSS56666;
5	Cufflinks	exon	176155032	176155421	.	+	.	gene_id XLOC_024159; transcript_id TCONS_00027588; exon_number 1; oId CUFF.57147.1; tss_id TSS56675;
5	Cufflinks	exon	176985833	176986251	.	+	.	gene_id XLOC_024174; transcript_id TCONS_00027604; exon_number 1; oId CUFF.57189.1; tss_id TSS56694;
5	Cufflinks	exon	177275115	177276989	.	+	.	gene_id GRMZM2G492156; transcript_id TCONS_00027606; exon_number 1; oId CUFF.57233.2; tss_id TSS56702;
5	Cufflinks	exon	177277795	177277894	.	+	.	gene_id GRMZM2G492156; transcript_id TCONS_00027606; exon_number 2; oId CUFF.57233.2; tss_id TSS56702;
5	Cufflinks	exon	177277976	177278017	.	+	.	gene_id GRMZM2G492156; transcript_id TCONS_00027606; exon_number 3; oId CUFF.57233.2; tss_id TSS56702;
5	Cufflinks	exon	177278102	177278185	.	+	.	gene_id GRMZM2G492156; transcript_id TCONS_00027606; exon_number 4; oId CUFF.57233.2; tss_id TSS56702;
5	Cufflinks	exon	177278263	177278417	.	+	.	gene_id GRMZM2G492156; transcript_id TCONS_00027606; exon_number 5; oId CUFF.57233.2; tss_id TSS56702;
5	Cufflinks	exon	177278500	177279005	.	+	.	gene_id GRMZM2G492156; transcript_id TCONS_00027606; exon_number 6; oId CUFF.57233.2; tss_id TSS56702;
5	Cufflinks	exon	177873141	177873462	.	+	.	gene_id XLOC_024185; transcript_id TCONS_00027616; exon_number 1; oId CUFF.57227.1; tss_id TSS56712;
5	Cufflinks	exon	183658189	183659274	.	+	.	gene_id XLOC_024267; transcript_id TCONS_00027713; exon_number 1; oId CUFF.57581.1; tss_id TSS56897;
5	Cufflinks	exon	183662191	183662680	.	+	.	gene_id XLOC_024267; transcript_id TCONS_00027713; exon_number 2; oId CUFF.57581.1; tss_id TSS56897;
5	Cufflinks	exon	187621682	187622099	.	+	.	gene_id XLOC_024305; transcript_id TCONS_00027758; exon_number 1; oId CUFF.57758.1; tss_id TSS56998;

5	Cufflinks	exon	188717132	188717767	.	+	.	gene_id XLOC_024320; transcript_id TCONS_00027776; exon_number 1; oId CUFF.57835.1; tss_id TSS57030;
5	Cufflinks	exon	188718037	188718626	.	+	.	gene_id XLOC_024321; transcript_id TCONS_00027777; exon_number 1; oId CUFF.57836.1; tss_id TSS57031;
5	Cufflinks	exon	191730938	191731148	.	+	.	gene_id GRMZM2G170591; transcript_id TCONS_00027824; exon_number 1; oId CUFF.58008.1; tss_id TSS57116;
5	Cufflinks	exon	191731216	191731585	.	+	.	gene_id GRMZM2G170591; transcript_id TCONS_00027824; exon_number 2; oId CUFF.58008.1; tss_id TSS57116;
5	Cufflinks	exon	191732940	191734190	.	+	.	gene_id GRMZM2G170591; transcript_id TCONS_00027824; exon_number 3; oId CUFF.58008.1; tss_id TSS57116;
5	Cufflinks	exon	191734509	191735780	.	+	.	gene_id GRMZM2G170591; transcript_id TCONS_00027824; exon_number 4; oId CUFF.58008.1; tss_id TSS57116;
5	Cufflinks	exon	193359086	193359677	.	+	.	gene_id AC209208.3_FG002; transcript_id TCONS_00027850; exon_number 1; oId CUFF.58143.1; tss_id TSS57171;
5	Cufflinks	exon	193360879	193361174	.	+	.	gene_id AC209208.3_FG002; transcript_id TCONS_00027850; exon_number 2; oId CUFF.58143.1; tss_id TSS57171;
5	Cufflinks	exon	193361599	193361739	.	+	.	gene_id AC209208.3_FG002; transcript_id TCONS_00027850; exon_number 3; oId CUFF.58143.1; tss_id TSS57171;
5	Cufflinks	exon	193362095	193363241	.	+	.	gene_id AC209208.3_FG002; transcript_id TCONS_00027850; exon_number 4; oId CUFF.58143.1; tss_id TSS57171;
5	Cufflinks	exon	194144303	194146501	.	+	.	gene_id GRMZM2G074053; transcript_id TCONS_00027871; exon_number 1; oId CUFF.58139.1; tss_id TSS57196;
5	Cufflinks	exon	194884662	194885678	.	+	.	gene_id XLOC_024414; transcript_id TCONS_00027880; exon_number 1; oId CUFF.58181.1; tss_id TSS57211;
5	Cufflinks	exon	199913016	199913887	.	+	.	gene_id XLOC_024483; transcript_id TCONS_00027958; exon_number 1; oId CUFF.58480.1; tss_id TSS57359;
5	Cufflinks	exon	203730336	203731019	.	+	.	gene_id GRMZM5G848822; transcript_id TCONS_00028046; exon_number 1; oId CUFF.58708.1; tss_id TSS57511;
5	Cufflinks	exon	203731160	203732571	.	+	.	gene_id GRMZM5G848822; transcript_id TCONS_00028046; exon_number 2; oId CUFF.58708.1; tss_id TSS57511;
5	Cufflinks	exon	203754997	203755409	.	+	.	gene_id GRMZM2G128992; transcript_id TCONS_00028052; exon_number 1; oId CUFF.58719.1; tss_id TSS57517;
5	Cufflinks	exon	203755540	203756242	.	+	.	gene_id GRMZM2G128992; transcript_id TCONS_00028052; exon_number 2; oId CUFF.58719.1; tss_id TSS57517;
5	Cufflinks	exon	205385840	205387859	.	+	.	gene_id GRMZM2G481730; transcript_id TCONS_00028121; exon_number 1; oId CUFF.58915.1; tss_id TSS57623;
5	Cufflinks	exon	205388056	205388206	.	+	.	gene_id GRMZM2G481730; transcript_id TCONS_00028121; exon_number 2; oId CUFF.58915.1; tss_id TSS57623;
5	Cufflinks	exon	205388502	205390104	.	+	.	gene_id GRMZM2G481730; transcript_id TCONS_00028121; exon_number 3; oId CUFF.58915.1; tss_id TSS57623;
5	Cufflinks	exon	205782086	205783762	.	+	.	gene_id XLOC_024635; transcript_id TCONS_00028135; exon_number 1; oId CUFF.58906.1; tss_id TSS57640;
5	Cufflinks	exon	205896279	205896680	.	+	.	gene_id XLOC_024638; transcript_id TCONS_00028138; exon_number 1; oId CUFF.58928.1; tss_id TSS57645;
5	Cufflinks	exon	210349025	210349341	.	+	.	gene_id GRMZM2G113418; transcript_id TCONS_00028246; exon_number 1; oId CUFF.59378.1; tss_id TSS57888;
5	Cufflinks	exon	210349450	210349539	.	+	.	gene_id GRMZM2G113418; transcript_id TCONS_00028246; exon_number 2; oId CUFF.59378.1; tss_id TSS57888;
5	Cufflinks	exon	210349720	210351808	.	+	.	gene_id GRMZM2G113418; transcript_id TCONS_00028246; exon_number 3; oId CUFF.59378.1; tss_id TSS57888;
5	Cufflinks	exon	213103814	213105936	.	+	.	gene_id GRMZM2G122231; transcript_id TCONS_00028322; exon_number 1; oId CUFF.59650.1; tss_id TSS58038;
5	Cufflinks	exon	213106300	213106573	.	+	.	gene_id GRMZM2G122231; transcript_id TCONS_00028322; exon_number 2; oId CUFF.59650.1; tss_id TSS58038;
5	Cufflinks	exon	213107031	213107810	.	+	.	gene_id GRMZM2G122231; transcript_id TCONS_00028322; exon_number 3; oId CUFF.59650.1; tss_id TSS58038;
5	Cufflinks	exon	213103814	213105936	.	+	.	gene_id GRMZM2G122231; transcript_id TCONS_00028323; exon_number 1; oId CUFF.59650.2; tss_id TSS58038;
5	Cufflinks	exon	213106300	213107810	.	+	.	gene_id GRMZM2G122231; transcript_id TCONS_00028323; exon_number 2; oId CUFF.59650.2; tss_id TSS58038;
5	Cufflinks	exon	215337121	215337685	.	+	.	gene_id GRMZM5G806449; transcript_id TCONS_00028371; exon_number 1; oId CUFF.59897.1; tss_id TSS58149;
5	Cufflinks	exon	215337810	215337984	.	+	.	gene_id GRMZM5G806449; transcript_id TCONS_00028371; exon_number 2; oId CUFF.59897.1; tss_id TSS58149;
5	Cufflinks	exon	215338495	215338559	.	+	.	gene_id GRMZM5G806449; transcript_id TCONS_00028371; exon_number 3; oId CUFF.59897.1; tss_id TSS58149;
5	Cufflinks	exon	215338683	215338752	.	+	.	gene_id GRMZM5G806449; transcript_id TCONS_00028371; exon_number 4; oId CUFF.59897.1; tss_id TSS58149;
5	Cufflinks	exon	215338892	215338963	.	+	.	gene_id GRMZM5G806449; transcript_id TCONS_00028371; exon_number 5; oId CUFF.59897.1; tss_id TSS58149;
5	Cufflinks	exon	215339070	215339273	.	+	.	gene_id GRMZM5G806449; transcript_id TCONS_00028371; exon_number 6; oId CUFF.59897.1; tss_id TSS58149;

5	Cufflinks	exon	215339603	215339676	.	+	.	gene_id GRMZM5G806449; transcript_id TCONS_00028371; exon_number 7; oId CUFF.59897.1; tss_id TSS58149;
5	Cufflinks	exon	215339765	215339848	.	+	.	gene_id GRMZM5G806449; transcript_id TCONS_00028371; exon_number 8; oId CUFF.59897.1; tss_id TSS58149;
5	Cufflinks	exon	215343668	215343755	.	+	.	gene_id GRMZM5G806449; transcript_id TCONS_00028371; exon_number 9; oId CUFF.59897.1; tss_id TSS58149;
5	Cufflinks	exon	215343840	215343921	.	+	.	gene_id GRMZM5G806449; transcript_id TCONS_00028371; exon_number 10; oId CUFF.59897.1; tss_id TSS58149;
5	Cufflinks	exon	215344208	215344281	.	+	.	gene_id GRMZM5G806449; transcript_id TCONS_00028371; exon_number 11; oId CUFF.59897.1; tss_id TSS58149;
5	Cufflinks	exon	215344452	215344559	.	+	.	gene_id GRMZM5G806449; transcript_id TCONS_00028371; exon_number 12; oId CUFF.59897.1; tss_id TSS58149;
5	Cufflinks	exon	215344637	215344701	.	+	.	gene_id GRMZM5G806449; transcript_id TCONS_00028371; exon_number 13; oId CUFF.59897.1; tss_id TSS58149;
5	Cufflinks	exon	215345574	215345687	.	+	.	gene_id GRMZM5G806449; transcript_id TCONS_00028371; exon_number 14; oId CUFF.59897.1; tss_id TSS58149;
5	Cufflinks	exon	215345881	215345980	.	+	.	gene_id GRMZM5G806449; transcript_id TCONS_00028371; exon_number 15; oId CUFF.59897.1; tss_id TSS58149;
5	Cufflinks	exon	215346065	215346118	.	+	.	gene_id GRMZM5G806449; transcript_id TCONS_00028371; exon_number 16; oId CUFF.59897.1; tss_id TSS58149;
5	Cufflinks	exon	215346208	215346503	.	+	.	gene_id GRMZM5G806449; transcript_id TCONS_00028371; exon_number 17; oId CUFF.59897.1; tss_id TSS58149;
5	Cufflinks	exon	217656072	217656403	.	+	.	gene_id GRMZM2G375002; transcript_id TCONS_00028436; exon_number 1; oId CUFF.60125.1; tss_id TSS58277;
5	Cufflinks	exon	217656921	217657008	.	+	.	gene_id GRMZM2G375002; transcript_id TCONS_00028436; exon_number 2; oId CUFF.60125.1; tss_id TSS58277;
5	Cufflinks	exon	217657098	217657207	.	+	.	gene_id GRMZM2G375002; transcript_id TCONS_00028436; exon_number 3; oId CUFF.60125.1; tss_id TSS58277;
5	Cufflinks	exon	217657325	217657388	.	+	.	gene_id GRMZM2G375002; transcript_id TCONS_00028436; exon_number 4; oId CUFF.60125.1; tss_id TSS58277;
5	Cufflinks	exon	217663440	217663504	.	+	.	gene_id GRMZM2G375002; transcript_id TCONS_00028436; exon_number 5; oId CUFF.60125.1; tss_id TSS58277;
5	Cufflinks	exon	217663583	217663648	.	+	.	gene_id GRMZM2G375002; transcript_id TCONS_00028436; exon_number 6; oId CUFF.60125.1; tss_id TSS58277;
5	Cufflinks	exon	217663741	217664485	.	+	.	gene_id GRMZM2G375002; transcript_id TCONS_00028436; exon_number 7; oId CUFF.60125.1; tss_id TSS58277;
5	Cufflinks	exon	888240	890892	.	-	.	gene_id GRMZM2G384339; transcript_id TCONS_00028469; exon_number 1; oId CUFF.49943.2; tss_id TSS58331;
5	Cufflinks	exon	892380	893249	.	-	.	gene_id GRMZM2G384339; transcript_id TCONS_00028469; exon_number 2; oId CUFF.49943.2; tss_id TSS58331;
5	Cufflinks	exon	1846419	1847142	.	-	.	gene_id GRMZM2G138421; transcript_id TCONS_00028527; exon_number 1; oId CUFF.50079.1; tss_id TSS58407;
5	Cufflinks	exon	1847419	1847997	.	-	.	gene_id GRMZM2G138421; transcript_id TCONS_00028527; exon_number 2; oId CUFF.50079.1; tss_id TSS58407;
5	Cufflinks	exon	1848161	1848305	.	-	.	gene_id GRMZM2G138421; transcript_id TCONS_00028527; exon_number 3; oId CUFF.50079.1; tss_id TSS58407;
5	Cufflinks	exon	1848429	1848570	.	-	.	gene_id GRMZM2G138421; transcript_id TCONS_00028527; exon_number 4; oId CUFF.50079.1; tss_id TSS58407;
5	Cufflinks	exon	1848692	1848809	.	-	.	gene_id GRMZM2G138421; transcript_id TCONS_00028527; exon_number 5; oId CUFF.50079.1; tss_id TSS58407;
5	Cufflinks	exon	1848889	1848996	.	-	.	gene_id GRMZM2G138421; transcript_id TCONS_00028527; exon_number 6; oId CUFF.50079.1; tss_id TSS58407;
5	Cufflinks	exon	1849071	1849145	.	-	.	gene_id GRMZM2G138421; transcript_id TCONS_00028527; exon_number 7; oId CUFF.50079.1; tss_id TSS58407;
5	Cufflinks	exon	1850329	1850558	.	-	.	gene_id GRMZM2G138421; transcript_id TCONS_00028527; exon_number 8; oId CUFF.50079.1; tss_id TSS58407;
5	Cufflinks	exon	1850645	1850932	.	-	.	gene_id GRMZM2G138421; transcript_id TCONS_00028527; exon_number 9; oId CUFF.50079.1; tss_id TSS58407;
5	Cufflinks	exon	1851557	1851738	.	-	.	gene_id GRMZM2G138421; transcript_id TCONS_00028527; exon_number 10; oId CUFF.50079.1; tss_id TSS58407;
5	Cufflinks	exon	1852013	1853070	.	-	.	gene_id GRMZM2G138421; transcript_id TCONS_00028527; exon_number 11; oId CUFF.50079.1; tss_id TSS58407;
5	Cufflinks	exon	3145962	3147116	.	-	.	gene_id GRMZM2G332976; transcript_id TCONS_00028581; exon_number 1; oId CUFF.50252.1; tss_id TSS58506;
5	Cufflinks	exon	3147607	3147816	.	-	.	gene_id GRMZM2G332976; transcript_id TCONS_00028581; exon_number 2; oId CUFF.50252.1; tss_id TSS58506;
5	Cufflinks	exon	3779249	3781015	.	-	.	gene_id XLOC_025015; transcript_id TCONS_00028602; exon_number 1; oId CUFF.50317.1; tss_id TSS58543;
5	Cufflinks	exon	3953124	3953974	.	-	.	gene_id XLOC_025021; transcript_id TCONS_00028609; exon_number 1; oId CUFF.50362.2; tss_id TSS58556;
5	Cufflinks	exon	4312135	4312844	.	-	.	gene_id GRMZM2G099474; transcript_id TCONS_00028619; exon_number 1; oId CUFF.50399.1; tss_id TSS58579;

5	Cufflinks	exon	4313469	4313546	.	-	.	gene_id GRMZM2G099474; transcript_id TCONS_00028619; exon_number 2; oId CUFF.50399.1; tss_id TSS58579;
5	Cufflinks	exon	4313628	4313674	.	-	.	gene_id GRMZM2G099474; transcript_id TCONS_00028619; exon_number 3; oId CUFF.50399.1; tss_id TSS58579;
5	Cufflinks	exon	4313786	4313846	.	-	.	gene_id GRMZM2G099474; transcript_id TCONS_00028619; exon_number 4; oId CUFF.50399.1; tss_id TSS58579;
5	Cufflinks	exon	4313947	4313995	.	-	.	gene_id GRMZM2G099474; transcript_id TCONS_00028619; exon_number 5; oId CUFF.50399.1; tss_id TSS58579;
5	Cufflinks	exon	4316544	4316667	.	-	.	gene_id GRMZM2G099474; transcript_id TCONS_00028619; exon_number 6; oId CUFF.50399.1; tss_id TSS58579;
5	Cufflinks	exon	4316916	4317196	.	-	.	gene_id GRMZM2G099474; transcript_id TCONS_00028619; exon_number 7; oId CUFF.50399.1; tss_id TSS58579;
5	Cufflinks	exon	6918690	6919909	.	-	.	gene_id XLOC_025097; transcript_id TCONS_00028692; exon_number 1; oId CUFF.50710.4; tss_id TSS58737;
5	Cufflinks	exon	9918543	9919311	.	-	.	gene_id GRMZM2G096591; transcript_id TCONS_00028769; exon_number 1; oId CUFF.50952.1; tss_id TSS58877;
5	Cufflinks	exon	9919411	9920468	.	-	.	gene_id GRMZM2G096591; transcript_id TCONS_00028769; exon_number 2; oId CUFF.50952.1; tss_id TSS58877;
5	Cufflinks	exon	9921349	9921805	.	-	.	gene_id GRMZM2G096591; transcript_id TCONS_00028769; exon_number 3; oId CUFF.50952.1; tss_id TSS58877;
5	Cufflinks	exon	10240536	10241878	.	-	.	gene_id GRMZM2G152548; transcript_id TCONS_00028790; exon_number 1; oId CUFF.50948.1; tss_id TSS58905;
5	Cufflinks	exon	10242601	10242899	.	-	.	gene_id GRMZM2G152548; transcript_id TCONS_00028790; exon_number 2; oId CUFF.50948.1; tss_id TSS58905;
5	Cufflinks	exon	10243205	10243482	.	-	.	gene_id GRMZM2G152548; transcript_id TCONS_00028790; exon_number 3; oId CUFF.50948.1; tss_id TSS58905;
5	Cufflinks	exon	16709490	16709635	.	-	.	gene_id XLOC_025279; transcript_id TCONS_00028899; exon_number 1; oId CUFF.51397.1; tss_id TSS59104;
5	Cufflinks	exon	16709763	16709870	.	-	.	gene_id XLOC_025279; transcript_id TCONS_00028899; exon_number 2; oId CUFF.51397.1; tss_id TSS59104;
5	Cufflinks	exon	16709960	16710036	.	-	.	gene_id XLOC_025279; transcript_id TCONS_00028899; exon_number 3; oId CUFF.51397.1; tss_id TSS59104;
5	Cufflinks	exon	16710118	16710190	.	-	.	gene_id XLOC_025279; transcript_id TCONS_00028899; exon_number 4; oId CUFF.51397.1; tss_id TSS59104;
5	Cufflinks	exon	16710295	16710432	.	-	.	gene_id XLOC_025279; transcript_id TCONS_00028899; exon_number 5; oId CUFF.51397.1; tss_id TSS59104;
5	Cufflinks	exon	16710522	16710566	.	-	.	gene_id XLOC_025279; transcript_id TCONS_00028899; exon_number 6; oId CUFF.51397.1; tss_id TSS59104;
5	Cufflinks	exon	16710655	16710775	.	-	.	gene_id XLOC_025279; transcript_id TCONS_00028899; exon_number 7; oId CUFF.51397.1; tss_id TSS59104;
5	Cufflinks	exon	16710876	16710962	.	-	.	gene_id XLOC_025279; transcript_id TCONS_00028899; exon_number 8; oId CUFF.51397.1; tss_id TSS59104;
5	Cufflinks	exon	16711048	16711112	.	-	.	gene_id XLOC_025279; transcript_id TCONS_00028899; exon_number 9; oId CUFF.51397.1; tss_id TSS59104;
5	Cufflinks	exon	16711271	16711468	.	-	.	gene_id XLOC_025279; transcript_id TCONS_00028899; exon_number 10; oId CUFF.51397.1; tss_id TSS59104;
5	Cufflinks	exon	16711608	16711898	.	-	.	gene_id XLOC_025279; transcript_id TCONS_00028899; exon_number 11; oId CUFF.51397.1; tss_id TSS59104;
5	Cufflinks	exon	16711972	16712193	.	-	.	gene_id XLOC_025279; transcript_id TCONS_00028899; exon_number 12; oId CUFF.51397.1; tss_id TSS59104;
5	Cufflinks	exon	16713427	16713961	.	-	.	gene_id XLOC_025279; transcript_id TCONS_00028899; exon_number 13; oId CUFF.51397.1; tss_id TSS59104;
5	Cufflinks	exon	21362636	21363839	.	-	.	gene_id XLOC_025335; transcript_id TCONS_00028964; exon_number 1; oId CUFF.51640.1; tss_id TSS59223;
5	Cufflinks	exon	23018093	23018717	.	-	.	gene_id XLOC_025361; transcript_id TCONS_00028993; exon_number 1; oId CUFF.51731.1; tss_id TSS59293;
5	Cufflinks	exon	35018140	35019008	.	-	.	gene_id GRMZM2G084021; transcript_id TCONS_00029147; exon_number 1; oId CUFF.52360.1; tss_id TSS59593;
5	Cufflinks	exon	35019328	35019439	.	-	.	gene_id GRMZM2G084021; transcript_id TCONS_00029147; exon_number 2; oId CUFF.52360.1; tss_id TSS59593;
5	Cufflinks	exon	35020735	35021031	.	-	.	gene_id GRMZM2G084021; transcript_id TCONS_00029147; exon_number 3; oId CUFF.52360.1; tss_id TSS59593;
5	Cufflinks	exon	35021140	35021326	.	-	.	gene_id GRMZM2G084021; transcript_id TCONS_00029147; exon_number 4; oId CUFF.52360.1; tss_id TSS59593;
5	Cufflinks	exon	43684627	43686211	.	-	.	gene_id XLOC_025579; transcript_id TCONS_00029248; exon_number 1; oId CUFF.52708.1; tss_id TSS59800;
5	Cufflinks	exon	49575144	49575550	.	-	.	gene_id XLOC_025626; transcript_id TCONS_00029304; exon_number 1; oId CUFF.52894.1; tss_id TSS59880;
5	Cufflinks	exon	59063826	59064502	.	-	.	gene_id XLOC_025700; transcript_id TCONS_00029384; exon_number 1; oId CUFF.53358.2; tss_id TSS60061;
5	Cufflinks	exon	61455740	61457120	.	-	.	gene_id GRMZM2G417454; transcript_id TCONS_00029435; exon_number 1; oId CUFF.53449.1; tss_id TSS60135;

5	Cufflinks	exon	65233060	65233886	.	-	.	gene_id GRMZM2G055619; transcript_id TCONS_00029491; exon_number 1; oId CUFF.53630.1; tss_id TSS60242;
5	Cufflinks	exon	65233979	65234173	.	-	.	gene_id GRMZM2G055619; transcript_id TCONS_00029491; exon_number 2; oId CUFF.53630.1; tss_id TSS60242;
5	Cufflinks	exon	65234377	65234471	.	-	.	gene_id GRMZM2G055619; transcript_id TCONS_00029491; exon_number 3; oId CUFF.53630.1; tss_id TSS60242;
5	Cufflinks	exon	65234598	65234952	.	-	.	gene_id GRMZM2G055619; transcript_id TCONS_00029491; exon_number 4; oId CUFF.53630.1; tss_id TSS60242;
5	Cufflinks	exon	65235029	65235608	.	-	.	gene_id GRMZM2G055619; transcript_id TCONS_00029491; exon_number 5; oId CUFF.53630.1; tss_id TSS60242;
5	Cufflinks	exon	65235883	65236112	.	-	.	gene_id GRMZM2G055619; transcript_id TCONS_00029491; exon_number 6; oId CUFF.53630.1; tss_id TSS60242;
5	Cufflinks	exon	65236312	65236881	.	-	.	gene_id GRMZM2G055619; transcript_id TCONS_00029491; exon_number 7; oId CUFF.53630.1; tss_id TSS60242;
5	Cufflinks	exon	65233060	65233886	.	-	.	gene_id GRMZM2G055619; transcript_id TCONS_00029492; exon_number 1; oId CUFF.53630.2; tss_id TSS60242;
5	Cufflinks	exon	65233979	65234173	.	-	.	gene_id GRMZM2G055619; transcript_id TCONS_00029492; exon_number 2; oId CUFF.53630.2; tss_id TSS60242;
5	Cufflinks	exon	65234377	65234471	.	-	.	gene_id GRMZM2G055619; transcript_id TCONS_00029492; exon_number 3; oId CUFF.53630.2; tss_id TSS60242;
5	Cufflinks	exon	65234598	65234952	.	-	.	gene_id GRMZM2G055619; transcript_id TCONS_00029492; exon_number 4; oId CUFF.53630.2; tss_id TSS60242;
5	Cufflinks	exon	65235029	65235608	.	-	.	gene_id GRMZM2G055619; transcript_id TCONS_00029492; exon_number 5; oId CUFF.53630.2; tss_id TSS60242;
5	Cufflinks	exon	65235883	65236204	.	-	.	gene_id GRMZM2G055619; transcript_id TCONS_00029492; exon_number 6; oId CUFF.53630.2; tss_id TSS60242;
5	Cufflinks	exon	65236312	65236881	.	-	.	gene_id GRMZM2G055619; transcript_id TCONS_00029492; exon_number 7; oId CUFF.53630.2; tss_id TSS60242;
5	Cufflinks	exon	67702344	67703449	.	-	.	gene_id GRMZM2G159047; transcript_id TCONS_00029519; exon_number 1; oId CUFF.53713.1; tss_id TSS60320;
5	Cufflinks	exon	68048954	68049256	.	-	.	gene_id GRMZM2G108474; transcript_id TCONS_00029522; exon_number 1; oId CUFF.53750.1; tss_id TSS60329;
5	Cufflinks	exon	68049913	68050070	.	-	.	gene_id GRMZM2G108474; transcript_id TCONS_00029522; exon_number 2; oId CUFF.53750.1; tss_id TSS60329;
5	Cufflinks	exon	68050168	68050293	.	-	.	gene_id GRMZM2G108474; transcript_id TCONS_00029522; exon_number 3; oId CUFF.53750.1; tss_id TSS60329;
5	Cufflinks	exon	68051243	68051316	.	-	.	gene_id GRMZM2G108474; transcript_id TCONS_00029522; exon_number 4; oId CUFF.53750.1; tss_id TSS60329;
5	Cufflinks	exon	68051536	68051724	.	-	.	gene_id GRMZM2G108474; transcript_id TCONS_00029522; exon_number 5; oId CUFF.53750.1; tss_id TSS60329;
5	Cufflinks	exon	70805268	70805994	.	-	.	gene_id GRMZM2G164242; transcript_id TCONS_00029555; exon_number 1; oId CUFF.53940.2; tss_id TSS60413;
5	Cufflinks	exon	70806154	70806288	.	-	.	gene_id GRMZM2G164242; transcript_id TCONS_00029555; exon_number 2; oId CUFF.53940.2; tss_id TSS60413;
5	Cufflinks	exon	70806927	70806980	.	-	.	gene_id GRMZM2G164242; transcript_id TCONS_00029555; exon_number 3; oId CUFF.53940.2; tss_id TSS60413;
5	Cufflinks	exon	70807101	70807181	.	-	.	gene_id GRMZM2G164242; transcript_id TCONS_00029555; exon_number 4; oId CUFF.53940.2; tss_id TSS60413;
5	Cufflinks	exon	70807268	70807363	.	-	.	gene_id GRMZM2G164242; transcript_id TCONS_00029555; exon_number 5; oId CUFF.53940.2; tss_id TSS60413;
5	Cufflinks	exon	70807670	70807845	.	-	.	gene_id GRMZM2G164242; transcript_id TCONS_00029555; exon_number 6; oId CUFF.53940.2; tss_id TSS60413;
5	Cufflinks	exon	70807943	70808016	.	-	.	gene_id GRMZM2G164242; transcript_id TCONS_00029555; exon_number 7; oId CUFF.53940.2; tss_id TSS60413;
5	Cufflinks	exon	70808197	70808304	.	-	.	gene_id GRMZM2G164242; transcript_id TCONS_00029555; exon_number 8; oId CUFF.53940.2; tss_id TSS60413;
5	Cufflinks	exon	70808384	70808559	.	-	.	gene_id GRMZM2G164242; transcript_id TCONS_00029555; exon_number 9; oId CUFF.53940.2; tss_id TSS60413;
5	Cufflinks	exon	70809240	70809290	.	-	.	gene_id GRMZM2G164242; transcript_id TCONS_00029555; exon_number 10; oId CUFF.53940.2; tss_id TSS60413;
5	Cufflinks	exon	70809426	70809536	.	-	.	gene_id GRMZM2G164242; transcript_id TCONS_00029555; exon_number 11; oId CUFF.53940.2; tss_id TSS60413;
5	Cufflinks	exon	70810009	70810063	.	-	.	gene_id GRMZM2G164242; transcript_id TCONS_00029555; exon_number 12; oId CUFF.53940.2; tss_id TSS60413;
5	Cufflinks	exon	70810688	70810807	.	-	.	gene_id GRMZM2G164242; transcript_id TCONS_00029555; exon_number 13; oId CUFF.53940.2; tss_id TSS60413;
5	Cufflinks	exon	70811202	70811290	.	-	.	gene_id GRMZM2G164242; transcript_id TCONS_00029555; exon_number 14; oId CUFF.53940.2; tss_id TSS60413;
5	Cufflinks	exon	70811753	70811867	.	-	.	gene_id GRMZM2G164242; transcript_id TCONS_00029555; exon_number 15; oId CUFF.53940.2; tss_id TSS60413;
5	Cufflinks	exon	70811971	70812495	.	-	.	gene_id GRMZM2G164242; transcript_id TCONS_00029555; exon_number 16; oId CUFF.53940.2; tss_id TSS60413;

5	Cufflinks	exon	70805268	70805994	.	-	.	gene_id GRMZM2G164242; transcript_id TCONS_00029556; exon_number 1; oId CUFF.53940.1; tss_id TSS60413;
5	Cufflinks	exon	70806154	70806288	.	-	.	gene_id GRMZM2G164242; transcript_id TCONS_00029556; exon_number 2; oId CUFF.53940.1; tss_id TSS60413;
5	Cufflinks	exon	70806927	70806980	.	-	.	gene_id GRMZM2G164242; transcript_id TCONS_00029556; exon_number 3; oId CUFF.53940.1; tss_id TSS60413;
5	Cufflinks	exon	70807101	70807181	.	-	.	gene_id GRMZM2G164242; transcript_id TCONS_00029556; exon_number 4; oId CUFF.53940.1; tss_id TSS60413;
5	Cufflinks	exon	70807268	70807363	.	-	.	gene_id GRMZM2G164242; transcript_id TCONS_00029556; exon_number 5; oId CUFF.53940.1; tss_id TSS60413;
5	Cufflinks	exon	70807670	70807845	.	-	.	gene_id GRMZM2G164242; transcript_id TCONS_00029556; exon_number 6; oId CUFF.53940.1; tss_id TSS60413;
5	Cufflinks	exon	70807943	70808016	.	-	.	gene_id GRMZM2G164242; transcript_id TCONS_00029556; exon_number 7; oId CUFF.53940.1; tss_id TSS60413;
5	Cufflinks	exon	70808197	70808304	.	-	.	gene_id GRMZM2G164242; transcript_id TCONS_00029556; exon_number 8; oId CUFF.53940.1; tss_id TSS60413;
5	Cufflinks	exon	70808384	70808571	.	-	.	gene_id GRMZM2G164242; transcript_id TCONS_00029556; exon_number 9; oId CUFF.53940.1; tss_id TSS60413;
5	Cufflinks	exon	70809240	70809290	.	-	.	gene_id GRMZM2G164242; transcript_id TCONS_00029556; exon_number 10; oId CUFF.53940.1; tss_id TSS60413;
5	Cufflinks	exon	70809426	70809536	.	-	.	gene_id GRMZM2G164242; transcript_id TCONS_00029556; exon_number 11; oId CUFF.53940.1; tss_id TSS60413;
5	Cufflinks	exon	70810009	70810063	.	-	.	gene_id GRMZM2G164242; transcript_id TCONS_00029556; exon_number 12; oId CUFF.53940.1; tss_id TSS60413;
5	Cufflinks	exon	70810688	70810807	.	-	.	gene_id GRMZM2G164242; transcript_id TCONS_00029556; exon_number 13; oId CUFF.53940.1; tss_id TSS60413;
5	Cufflinks	exon	70811202	70811290	.	-	.	gene_id GRMZM2G164242; transcript_id TCONS_00029556; exon_number 14; oId CUFF.53940.1; tss_id TSS60413;
5	Cufflinks	exon	70811753	70811867	.	-	.	gene_id GRMZM2G164242; transcript_id TCONS_00029556; exon_number 15; oId CUFF.53940.1; tss_id TSS60413;
5	Cufflinks	exon	70811971	70812495	.	-	.	gene_id GRMZM2G164242; transcript_id TCONS_00029556; exon_number 16; oId CUFF.53940.1; tss_id TSS60413;
5	Cufflinks	exon	76240730	76241160	.	-	.	gene_id XLOC_025906; transcript_id TCONS_00029613; exon_number 1; oId CUFF.54106.1; tss_id TSS60530;
5	Cufflinks	exon	81799469	81799920	.	-	.	gene_id XLOC_025954; transcript_id TCONS_00029669; exon_number 1; oId CUFF.54306.1; tss_id TSS60621;
5	Cufflinks	exon	83896631	83898155	.	-	.	gene_id GRMZM2G088235; transcript_id TCONS_00029692; exon_number 1; oId CUFF.54457.1; tss_id TSS60668;
5	Cufflinks	exon	83901212	83902051	.	-	.	gene_id GRMZM2G088235; transcript_id TCONS_00029692; exon_number 2; oId CUFF.54457.1; tss_id TSS60668;
5	Cufflinks	exon	83902162	83902358	.	-	.	gene_id GRMZM2G088235; transcript_id TCONS_00029692; exon_number 3; oId CUFF.54457.1; tss_id TSS60668;
5	Cufflinks	exon	83896631	83898113	.	-	.	gene_id GRMZM2G088235; transcript_id TCONS_00029693; exon_number 1; oId CUFF.54457.3; tss_id TSS60668;
5	Cufflinks	exon	83901212	83902051	.	-	.	gene_id GRMZM2G088235; transcript_id TCONS_00029693; exon_number 2; oId CUFF.54457.3; tss_id TSS60668;
5	Cufflinks	exon	83902162	83902358	.	-	.	gene_id GRMZM2G088235; transcript_id TCONS_00029693; exon_number 3; oId CUFF.54457.3; tss_id TSS60668;
5	Cufflinks	exon	84098409	84099212	.	-	.	gene_id XLOC_025974; transcript_id TCONS_00029696; exon_number 1; oId CUFF.54449.1; tss_id TSS60672;
5	Cufflinks	exon	86185986	86186689	.	-	.	gene_id GRMZM2G180211; transcript_id TCONS_00029712; exon_number 1; oId CUFF.54518.2; tss_id TSS60705;
5	Cufflinks	exon	86186816	86187105	.	-	.	gene_id GRMZM2G180211; transcript_id TCONS_00029712; exon_number 2; oId CUFF.54518.2; tss_id TSS60705;
5	Cufflinks	exon	86187287	86187587	.	-	.	gene_id GRMZM2G180211; transcript_id TCONS_00029712; exon_number 3; oId CUFF.54518.2; tss_id TSS60705;
5	Cufflinks	exon	86187709	86187779	.	-	.	gene_id GRMZM2G180211; transcript_id TCONS_00029712; exon_number 4; oId CUFF.54518.2; tss_id TSS60705;
5	Cufflinks	exon	86187953	86188066	.	-	.	gene_id GRMZM2G180211; transcript_id TCONS_00029712; exon_number 5; oId CUFF.54518.2; tss_id TSS60705;
5	Cufflinks	exon	86188147	86188221	.	-	.	gene_id GRMZM2G180211; transcript_id TCONS_00029712; exon_number 6; oId CUFF.54518.2; tss_id TSS60705;
5	Cufflinks	exon	86188316	86188374	.	-	.	gene_id GRMZM2G180211; transcript_id TCONS_00029712; exon_number 7; oId CUFF.54518.2; tss_id TSS60705;
5	Cufflinks	exon	86188984	86189477	.	-	.	gene_id GRMZM2G180211; transcript_id TCONS_00029712; exon_number 8; oId CUFF.54518.2; tss_id TSS60705;
5	Cufflinks	exon	92392744	92393218	.	-	.	gene_id XLOC_026024; transcript_id TCONS_00029748; exon_number 1; oId CUFF.54739.1; tss_id TSS60795;
5	Cufflinks	exon	92393292	92393411	.	-	.	gene_id XLOC_026024; transcript_id TCONS_00029748; exon_number 2; oId CUFF.54739.1; tss_id TSS60795;
5	Cufflinks	exon	92393489	92393814	.	-	.	gene_id XLOC_026024; transcript_id TCONS_00029748; exon_number 3; oId CUFF.54739.1; tss_id TSS60795;

5	Cufflinks	exon	118709138	118709890	.	-	.	gene_id GRMZM2G138349; transcript_id TCONS_00029858; exon_number 1; oId CUFF.55366.2; tss_id TSS61109;
5	Cufflinks	exon	118710026	118710121	.	-	.	gene_id GRMZM2G138349; transcript_id TCONS_00029858; exon_number 2; oId CUFF.55366.2; tss_id TSS61109;
5	Cufflinks	exon	118710206	118710322	.	-	.	gene_id GRMZM2G138349; transcript_id TCONS_00029858; exon_number 3; oId CUFF.55366.2; tss_id TSS61109;
5	Cufflinks	exon	118710418	118710535	.	-	.	gene_id GRMZM2G138349; transcript_id TCONS_00029858; exon_number 4; oId CUFF.55366.2; tss_id TSS61109;
5	Cufflinks	exon	118710729	118710892	.	-	.	gene_id GRMZM2G138349; transcript_id TCONS_00029858; exon_number 5; oId CUFF.55366.2; tss_id TSS61109;
5	Cufflinks	exon	118711125	118711200	.	-	.	gene_id GRMZM2G138349; transcript_id TCONS_00029858; exon_number 6; oId CUFF.55366.2; tss_id TSS61109;
5	Cufflinks	exon	118711507	118711591	.	-	.	gene_id GRMZM2G138349; transcript_id TCONS_00029858; exon_number 7; oId CUFF.55366.2; tss_id TSS61109;
5	Cufflinks	exon	118711890	118711969	.	-	.	gene_id GRMZM2G138349; transcript_id TCONS_00029858; exon_number 8; oId CUFF.55366.2; tss_id TSS61109;
5	Cufflinks	exon	118712316	118712441	.	-	.	gene_id GRMZM2G138349; transcript_id TCONS_00029858; exon_number 9; oId CUFF.55366.2; tss_id TSS61109;
5	Cufflinks	exon	151922535	151923503	.	-	.	gene_id GRMZM2G176206; transcript_id TCONS_00030058; exon_number 1; oId CUFF.56160.1; tss_id TSS61558;
5	Cufflinks	exon	151923619	151926261	.	-	.	gene_id GRMZM2G176206; transcript_id TCONS_00030058; exon_number 2; oId CUFF.56160.1; tss_id TSS61558;
5	Cufflinks	exon	152470035	152471158	.	-	.	gene_id GRMZM2G161299; transcript_id TCONS_00030063; exon_number 1; oId CUFF.56210.7; tss_id TSS61570;
5	Cufflinks	exon	152473373	152473492	.	-	.	gene_id GRMZM2G161299; transcript_id TCONS_00030063; exon_number 2; oId CUFF.56210.7; tss_id TSS61570;
5	Cufflinks	exon	152473567	152473751	.	-	.	gene_id GRMZM2G161299; transcript_id TCONS_00030063; exon_number 3; oId CUFF.56210.7; tss_id TSS61570;
5	Cufflinks	exon	152473836	152473962	.	-	.	gene_id GRMZM2G161299; transcript_id TCONS_00030063; exon_number 4; oId CUFF.56210.7; tss_id TSS61570;
5	Cufflinks	exon	152474146	152474353	.	-	.	gene_id GRMZM2G161299; transcript_id TCONS_00030063; exon_number 5; oId CUFF.56210.7; tss_id TSS61570;
5	Cufflinks	exon	152474443	152474528	.	-	.	gene_id GRMZM2G161299; transcript_id TCONS_00030063; exon_number 6; oId CUFF.56210.7; tss_id TSS61570;
5	Cufflinks	exon	152479558	152479785	.	-	.	gene_id GRMZM2G161299; transcript_id TCONS_00030063; exon_number 7; oId CUFF.56210.7; tss_id TSS61570;
5	Cufflinks	exon	162737531	162738936	.	-	.	gene_id GRMZM2G430526; transcript_id TCONS_00030147; exon_number 1; oId CUFF.56565.4; tss_id TSS61773;
5	Cufflinks	exon	162739126	162739178	.	-	.	gene_id GRMZM2G430526; transcript_id TCONS_00030147; exon_number 2; oId CUFF.56565.4; tss_id TSS61773;
5	Cufflinks	exon	162739279	162739428	.	-	.	gene_id GRMZM2G430526; transcript_id TCONS_00030147; exon_number 3; oId CUFF.56565.4; tss_id TSS61773;
5	Cufflinks	exon	162739676	162740885	.	-	.	gene_id GRMZM2G430526; transcript_id TCONS_00030147; exon_number 4; oId CUFF.56565.4; tss_id TSS61773;
5	Cufflinks	exon	162737531	162738936	.	-	.	gene_id GRMZM2G430526; transcript_id TCONS_00030148; exon_number 1; oId CUFF.56565.7; tss_id TSS61773;
5	Cufflinks	exon	162739126	162739178	.	-	.	gene_id GRMZM2G430526; transcript_id TCONS_00030148; exon_number 2; oId CUFF.56565.7; tss_id TSS61773;
5	Cufflinks	exon	162739279	162739428	.	-	.	gene_id GRMZM2G430526; transcript_id TCONS_00030148; exon_number 3; oId CUFF.56565.7; tss_id TSS61773;
5	Cufflinks	exon	162739701	162740885	.	-	.	gene_id GRMZM2G430526; transcript_id TCONS_00030148; exon_number 4; oId CUFF.56565.7; tss_id TSS61773;
5	Cufflinks	exon	162737531	162738752	.	-	.	gene_id GRMZM2G430526; transcript_id TCONS_00030150; exon_number 1; oId CUFF.56565.1; tss_id TSS61774;
5	Cufflinks	exon	162738841	162738936	.	-	.	gene_id GRMZM2G430526; transcript_id TCONS_00030150; exon_number 2; oId CUFF.56565.1; tss_id TSS61774;
5	Cufflinks	exon	162739126	162739178	.	-	.	gene_id GRMZM2G430526; transcript_id TCONS_00030150; exon_number 3; oId CUFF.56565.1; tss_id TSS61774;
5	Cufflinks	exon	162739279	162739428	.	-	.	gene_id GRMZM2G430526; transcript_id TCONS_00030150; exon_number 4; oId CUFF.56565.1; tss_id TSS61774;
5	Cufflinks	exon	162739701	162739836	.	-	.	gene_id GRMZM2G430526; transcript_id TCONS_00030150; exon_number 5; oId CUFF.56565.1; tss_id TSS61774;
5	Cufflinks	exon	162740461	162740605	.	-	.	gene_id GRMZM2G430526; transcript_id TCONS_00030150; exon_number 6; oId CUFF.56565.1; tss_id TSS61774;
5	Cufflinks	exon	162741521	162743559	.	-	.	gene_id GRMZM2G430526; transcript_id TCONS_00030150; exon_number 7; oId CUFF.56565.1; tss_id TSS61774;
5	Cufflinks	exon	162737531	162738752	.	-	.	gene_id GRMZM2G430526; transcript_id TCONS_00030151; exon_number 1; oId CUFF.56565.10; tss_id TSS61774;
5	Cufflinks	exon	162738841	162738936	.	-	.	gene_id GRMZM2G430526; transcript_id TCONS_00030151; exon_number 2; oId CUFF.56565.10; tss_id TSS61774;
5	Cufflinks	exon	162739126	162739178	.	-	.	gene_id GRMZM2G430526; transcript_id TCONS_00030151; exon_number 3; oId CUFF.56565.10; tss_id TSS61774;

5	Cufflinks	exon	162739279	162739428	.	-	.	gene_id GRMZM2G430526; transcript_id TCONS_00030151; exon_number 4; oId CUFF.56565.10; tss_id TSS61774;
5	Cufflinks	exon	162739676	162739836	.	-	.	gene_id GRMZM2G430526; transcript_id TCONS_00030151; exon_number 5; oId CUFF.56565.10; tss_id TSS61774;
5	Cufflinks	exon	162740461	162740605	.	-	.	gene_id GRMZM2G430526; transcript_id TCONS_00030151; exon_number 6; oId CUFF.56565.10; tss_id TSS61774;
5	Cufflinks	exon	162741521	162741683	.	-	.	gene_id GRMZM2G430526; transcript_id TCONS_00030151; exon_number 7; oId CUFF.56565.10; tss_id TSS61774;
5	Cufflinks	exon	162742961	162743559	.	-	.	gene_id GRMZM2G430526; transcript_id TCONS_00030151; exon_number 8; oId CUFF.56565.10; tss_id TSS61774;
5	Cufflinks	exon	167625007	167625814	.	-	.	gene_id GRMZM2G020840; transcript_id TCONS_00030206; exon_number 1; oId CUFF.56724.1; tss_id TSS61877;
5	Cufflinks	exon	167626208	167626599	.	-	.	gene_id GRMZM2G020840; transcript_id TCONS_00030206; exon_number 2; oId CUFF.56724.1; tss_id TSS61877;
5	Cufflinks	exon	175731393	175731623	.	-	.	gene_id GRMZM2G023988; transcript_id TCONS_00030335; exon_number 1; oId CUFF.57150.1; tss_id TSS62109;
5	Cufflinks	exon	175731813	175731877	.	-	.	gene_id GRMZM2G023988; transcript_id TCONS_00030335; exon_number 2; oId CUFF.57150.1; tss_id TSS62109;
5	Cufflinks	exon	175735194	175736518	.	-	.	gene_id GRMZM2G023988; transcript_id TCONS_00030335; exon_number 3; oId CUFF.57150.1; tss_id TSS62109;
5	Cufflinks	exon	177275738	177276159	.	-	.	gene_id XLOC_026542; transcript_id TCONS_00030353; exon_number 1; oId CUFF.57234.1; tss_id TSS62146;
5	Cufflinks	exon	177276278	177278150	.	-	.	gene_id XLOC_026542; transcript_id TCONS_00030353; exon_number 2; oId CUFF.57234.1; tss_id TSS62146;
5	Cufflinks	exon	182505927	182506522	.	-	.	gene_id GRMZM2G095147; transcript_id TCONS_00030441; exon_number 1; oId CUFF.57505.1; tss_id TSS62286;
5	Cufflinks	exon	183657809	183659274	.	-	.	gene_id GRMZM2G035584; transcript_id TCONS_00030462; exon_number 1; oId CUFF.57580.1; tss_id TSS62330;
5	Cufflinks	exon	183662191	183662628	.	-	.	gene_id GRMZM2G035584; transcript_id TCONS_00030462; exon_number 2; oId CUFF.57580.1; tss_id TSS62330;
5	Cufflinks	exon	183662706	183662949	.	-	.	gene_id GRMZM2G035584; transcript_id TCONS_00030462; exon_number 3; oId CUFF.57580.1; tss_id TSS62330;
5	Cufflinks	exon	190526842	190527999	.	-	.	gene_id GRMZM2G117164; transcript_id TCONS_00030565; exon_number 1; oId CUFF.57942.1; tss_id TSS62548;
5	Cufflinks	exon	190528089	190528626	.	-	.	gene_id GRMZM2G117164; transcript_id TCONS_00030565; exon_number 2; oId CUFF.57942.1; tss_id TSS62548;
5	Cufflinks	exon	190726076	190727295	.	-	.	gene_id GRMZM2G156599; transcript_id TCONS_00030569; exon_number 1; oId CUFF.57971.2; tss_id TSS62557;
5	Cufflinks	exon	190727400	190727517	.	-	.	gene_id GRMZM2G156599; transcript_id TCONS_00030569; exon_number 2; oId CUFF.57971.2; tss_id TSS62557;
5	Cufflinks	exon	190727675	190727864	.	-	.	gene_id GRMZM2G156599; transcript_id TCONS_00030569; exon_number 3; oId CUFF.57971.2; tss_id TSS62557;
5	Cufflinks	exon	190727955	190728089	.	-	.	gene_id GRMZM2G156599; transcript_id TCONS_00030569; exon_number 4; oId CUFF.57971.2; tss_id TSS62557;
5	Cufflinks	exon	190728170	190728267	.	-	.	gene_id GRMZM2G156599; transcript_id TCONS_00030569; exon_number 5; oId CUFF.57971.2; tss_id TSS62557;
5	Cufflinks	exon	190728349	190728518	.	-	.	gene_id GRMZM2G156599; transcript_id TCONS_00030569; exon_number 6; oId CUFF.57971.2; tss_id TSS62557;
5	Cufflinks	exon	190728602	190729027	.	-	.	gene_id GRMZM2G156599; transcript_id TCONS_00030569; exon_number 7; oId CUFF.57971.2; tss_id TSS62557;
5	Cufflinks	exon	190729161	190729337	.	-	.	gene_id GRMZM2G156599; transcript_id TCONS_00030569; exon_number 8; oId CUFF.57971.2; tss_id TSS62557;
5	Cufflinks	exon	194144551	194146414	.	-	.	gene_id XLOC_026772; transcript_id TCONS_00030624; exon_number 1; oId CUFF.58140.1; tss_id TSS62644;
5	Cufflinks	exon	194884329	194885888	.	-	.	gene_id XLOC_026779; transcript_id TCONS_00030634; exon_number 1; oId CUFF.58180.1; tss_id TSS62661;
5	Cufflinks	exon	194886849	194887265	.	-	.	gene_id XLOC_026779; transcript_id TCONS_00030634; exon_number 2; oId CUFF.58180.1; tss_id TSS62661;
5	Cufflinks	exon	198928222	198928999	.	-	.	gene_id XLOC_026825; transcript_id TCONS_00030684; exon_number 1; oId CUFF.58375.1; tss_id TSS62768;
5	Cufflinks	exon	200704676	200706175	.	-	.	gene_id GRMZM2G179147; transcript_id TCONS_00030714; exon_number 1; oId CUFF.58526.1; tss_id TSS62826;
5	Cufflinks	exon	200706256	200706362	.	-	.	gene_id GRMZM2G179147; transcript_id TCONS_00030714; exon_number 2; oId CUFF.58526.1; tss_id TSS62826;
5	Cufflinks	exon	200706566	200706734	.	-	.	gene_id GRMZM2G179147; transcript_id TCONS_00030714; exon_number 3; oId CUFF.58526.1; tss_id TSS62826;
5	Cufflinks	exon	200706829	200707218	.	-	.	gene_id GRMZM2G179147; transcript_id TCONS_00030714; exon_number 4; oId CUFF.58526.1; tss_id TSS62826;
5	Cufflinks	exon	200707325	200707817	.	-	.	gene_id GRMZM2G179147; transcript_id TCONS_00030714; exon_number 5; oId CUFF.58526.1; tss_id TSS62826;
5	Cufflinks	exon	201995204	201995662	.	-	.	gene_id XLOC_026868; transcript_id TCONS_00030732; exon_number 1; oId CUFF.58596.1; tss_id TSS62861;

5	Cufflinks	exon	203092250	203093914	.	-	.	gene_id GRMZM2G056252; transcript_id TCONS_00030757; exon_number 1; oId CUFF.58693.1; tss_id TSS62904;
5	Cufflinks	exon	203097014	203097186	.	-	.	gene_id GRMZM2G056252; transcript_id TCONS_00030757; exon_number 2; oId CUFF.58693.1; tss_id TSS62904;
5	Cufflinks	exon	203092250	203093910	.	-	.	gene_id GRMZM2G056252; transcript_id TCONS_00030758; exon_number 1; oId CUFF.58693.2; tss_id TSS62904;
5	Cufflinks	exon	203097014	203097186	.	-	.	gene_id GRMZM2G056252; transcript_id TCONS_00030758; exon_number 2; oId CUFF.58693.2; tss_id TSS62904;
5	Cufflinks	exon	203731859	203732426	.	-	.	gene_id XLOC_026901; transcript_id TCONS_00030770; exon_number 1; oId CUFF.58710.1; tss_id TSS62924;
5	Cufflinks	exon	205782086	205784030	.	-	.	gene_id GRMZM2G142779; transcript_id TCONS_00030815; exon_number 1; oId CUFF.58907.1; tss_id TSS63012;
5	Cufflinks	exon	205785469	205786901	.	-	.	gene_id GRMZM2G142779; transcript_id TCONS_00030815; exon_number 2; oId CUFF.58907.1; tss_id TSS63012;
5	Cufflinks	exon	205983909	205985356	.	-	.	gene_id GRMZM2G480364; transcript_id TCONS_00030822; exon_number 1; oId CUFF.58909.1; tss_id TSS63023;
5	Cufflinks	exon	205986279	205987943	.	-	.	gene_id GRMZM2G179768; transcript_id TCONS_00030823; exon_number 1; oId CUFF.58912.1; tss_id TSS63024;
5	Cufflinks	exon	210458456	210459126	.	-	.	gene_id GRMZM2G149452; transcript_id TCONS_00030932; exon_number 1; oId CUFF.59376.1; tss_id TSS63258;
5	Cufflinks	exon	210459288	210459439	.	-	.	gene_id GRMZM2G149452; transcript_id TCONS_00030932; exon_number 2; oId CUFF.59376.1; tss_id TSS63258;
5	Cufflinks	exon	210459543	210460077	.	-	.	gene_id GRMZM2G149452; transcript_id TCONS_00030932; exon_number 3; oId CUFF.59376.1; tss_id TSS63258;
5	Cufflinks	exon	210460161	210460223	.	-	.	gene_id GRMZM2G149452; transcript_id TCONS_00030932; exon_number 4; oId CUFF.59376.1; tss_id TSS63258;
5	Cufflinks	exon	210460968	210461256	.	-	.	gene_id GRMZM2G149452; transcript_id TCONS_00030932; exon_number 5; oId CUFF.59376.1; tss_id TSS63258;
5	Cufflinks	exon	212222159	212223644	.	-	.	gene_id GRMZM2G124416; transcript_id TCONS_00030978; exon_number 1; oId CUFF.59606.2; tss_id TSS63344;
5	Cufflinks	exon	212224377	212224485	.	-	.	gene_id GRMZM2G124416; transcript_id TCONS_00030978; exon_number 2; oId CUFF.59606.2; tss_id TSS63344;
5	Cufflinks	exon	212224573	212224600	.	-	.	gene_id GRMZM2G124416; transcript_id TCONS_00030978; exon_number 3; oId CUFF.59606.2; tss_id TSS63344;
5	Cufflinks	exon	212224915	212225087	.	-	.	gene_id GRMZM2G124416; transcript_id TCONS_00030978; exon_number 4; oId CUFF.59606.2; tss_id TSS63344;
5	Cufflinks	exon	212225255	212225345	.	-	.	gene_id GRMZM2G124416; transcript_id TCONS_00030978; exon_number 5; oId CUFF.59606.2; tss_id TSS63344;
5	Cufflinks	exon	212226970	212227637	.	-	.	gene_id GRMZM2G124416; transcript_id TCONS_00030978; exon_number 6; oId CUFF.59606.2; tss_id TSS63344;
5	Cufflinks	exon	213106063	213106920	.	-	.	gene_id XLOC_027112; transcript_id TCONS_00031005; exon_number 1; oId CUFF.59651.3; tss_id TSS63389;
5	Cufflinks	exon	213993280	213996291	.	-	.	gene_id GRMZM2G469111; transcript_id TCONS_00031033; exon_number 1; oId CUFF.59722.1; tss_id TSS63443;
5	Cufflinks	exon	214192115	214192267	.	-	.	gene_id XLOC_027142; transcript_id TCONS_00031040; exon_number 1; oId CUFF.59753.1; tss_id TSS63454;
5	Cufflinks	exon	214192378	214192485	.	-	.	gene_id XLOC_027142; transcript_id TCONS_00031040; exon_number 2; oId CUFF.59753.1; tss_id TSS63454;
5	Cufflinks	exon	215101686	215102542	.	-	.	gene_id XLOC_027171; transcript_id TCONS_00031074; exon_number 1; oId CUFF.59821.1; tss_id TSS63519;
5	Cufflinks	exon	215112799	215112945	.	-	.	gene_id GRMZM5G851655; transcript_id TCONS_00031077; exon_number 1; oId CUFF.59843.2; tss_id TSS63523;
5	Cufflinks	exon	215113041	215113170	.	-	.	gene_id GRMZM5G851655; transcript_id TCONS_00031077; exon_number 2; oId CUFF.59843.2; tss_id TSS63523;
5	Cufflinks	exon	215113283	215113357	.	-	.	gene_id GRMZM5G851655; transcript_id TCONS_00031077; exon_number 3; oId CUFF.59843.2; tss_id TSS63523;
5	Cufflinks	exon	215113460	215113508	.	-	.	gene_id GRMZM5G851655; transcript_id TCONS_00031077; exon_number 4; oId CUFF.59843.2; tss_id TSS63523;
5	Cufflinks	exon	215113770	215114152	.	-	.	gene_id GRMZM5G851655; transcript_id TCONS_00031077; exon_number 5; oId CUFF.59843.2; tss_id TSS63523;
5	Cufflinks	exon	215114338	215114629	.	-	.	gene_id GRMZM5G851655; transcript_id TCONS_00031077; exon_number 6; oId CUFF.59843.2; tss_id TSS63523;
5	Cufflinks	exon	215114863	215114937	.	-	.	gene_id GRMZM5G851655; transcript_id TCONS_00031077; exon_number 7; oId CUFF.59843.2; tss_id TSS63523;
5	Cufflinks	exon	215115125	215116277	.	-	.	gene_id GRMZM5G851655; transcript_id TCONS_00031077; exon_number 8; oId CUFF.59843.2; tss_id TSS63523;
5	Cufflinks	exon	215315528	215316236	.	-	.	gene_id XLOC_027174; transcript_id TCONS_00031078; exon_number 1; oId CUFF.59858.1; tss_id TSS63527;
5	Cufflinks	exon	215406418	215407368	.	-	.	gene_id GRMZM2G078887; transcript_id TCONS_00031083; exon_number 1; oId CUFF.59865.1; tss_id TSS63538;
5	Cufflinks	exon	215407464	215407561	.	-	.	gene_id GRMZM2G078887; transcript_id TCONS_00031083; exon_number 2; oId CUFF.59865.1; tss_id TSS63538;

5	Cufflinks	exon	215407662	215407900	.	-	.	gene_id GRMZM2G078887; transcript_id TCONS_00031083; exon_number 3; oId CUFF.59865.1; tss_id TSS63538;
5	Cufflinks	exon	215868272	215868675	.	-	.	gene_id GRMZM5G848768; transcript_id TCONS_00031094; exon_number 1; oId CUFF.59948.2; tss_id TSS63566;
5	Cufflinks	exon	215868850	215868933	.	-	.	gene_id GRMZM5G848768; transcript_id TCONS_00031094; exon_number 2; oId CUFF.59948.2; tss_id TSS63566;
5	Cufflinks	exon	215869055	215869164	.	-	.	gene_id GRMZM5G848768; transcript_id TCONS_00031094; exon_number 3; oId CUFF.59948.2; tss_id TSS63566;
5	Cufflinks	exon	215869248	215869371	.	-	.	gene_id GRMZM5G848768; transcript_id TCONS_00031094; exon_number 4; oId CUFF.59948.2; tss_id TSS63566;
5	Cufflinks	exon	215869484	215869580	.	-	.	gene_id GRMZM5G848768; transcript_id TCONS_00031094; exon_number 5; oId CUFF.59948.2; tss_id TSS63566;
5	Cufflinks	exon	215869668	215870093	.	-	.	gene_id GRMZM5G848768; transcript_id TCONS_00031094; exon_number 6; oId CUFF.59948.2; tss_id TSS63566;
5	Cufflinks	exon	215870180	215870287	.	-	.	gene_id GRMZM5G848768; transcript_id TCONS_00031094; exon_number 7; oId CUFF.59948.2; tss_id TSS63566;
5	Cufflinks	exon	215870381	215870448	.	-	.	gene_id GRMZM5G848768; transcript_id TCONS_00031094; exon_number 8; oId CUFF.59948.2; tss_id TSS63566;
5	Cufflinks	exon	215870531	215870629	.	-	.	gene_id GRMZM5G848768; transcript_id TCONS_00031094; exon_number 9; oId CUFF.59948.2; tss_id TSS63566;
5	Cufflinks	exon	215870752	215870878	.	-	.	gene_id GRMZM5G848768; transcript_id TCONS_00031094; exon_number 10; oId CUFF.59948.2; tss_id TSS63566;
5	Cufflinks	exon	215871050	215871134	.	-	.	gene_id GRMZM5G848768; transcript_id TCONS_00031094; exon_number 11; oId CUFF.59948.2; tss_id TSS63566;
5	Cufflinks	exon	215871616	215871692	.	-	.	gene_id GRMZM5G848768; transcript_id TCONS_00031094; exon_number 12; oId CUFF.59948.2; tss_id TSS63566;
5	Cufflinks	exon	215871807	215872212	.	-	.	gene_id GRMZM5G848768; transcript_id TCONS_00031094; exon_number 13; oId CUFF.59948.2; tss_id TSS63566;
5	Cufflinks	exon	215868272	215868675	.	-	.	gene_id GRMZM5G848768; transcript_id TCONS_00031095; exon_number 1; oId CUFF.59948.1; tss_id TSS63566;
5	Cufflinks	exon	215868850	215868933	.	-	.	gene_id GRMZM5G848768; transcript_id TCONS_00031095; exon_number 2; oId CUFF.59948.1; tss_id TSS63566;
5	Cufflinks	exon	215869055	215869164	.	-	.	gene_id GRMZM5G848768; transcript_id TCONS_00031095; exon_number 3; oId CUFF.59948.1; tss_id TSS63566;
5	Cufflinks	exon	215869248	215869371	.	-	.	gene_id GRMZM5G848768; transcript_id TCONS_00031095; exon_number 4; oId CUFF.59948.1; tss_id TSS63566;
5	Cufflinks	exon	215869484	215869580	.	-	.	gene_id GRMZM5G848768; transcript_id TCONS_00031095; exon_number 5; oId CUFF.59948.1; tss_id TSS63566;
5	Cufflinks	exon	215869668	215869800	.	-	.	gene_id GRMZM5G848768; transcript_id TCONS_00031095; exon_number 6; oId CUFF.59948.1; tss_id TSS63566;
5	Cufflinks	exon	215870016	215870093	.	-	.	gene_id GRMZM5G848768; transcript_id TCONS_00031095; exon_number 7; oId CUFF.59948.1; tss_id TSS63566;
5	Cufflinks	exon	215870180	215870287	.	-	.	gene_id GRMZM5G848768; transcript_id TCONS_00031095; exon_number 8; oId CUFF.59948.1; tss_id TSS63566;
5	Cufflinks	exon	215870381	215870448	.	-	.	gene_id GRMZM5G848768; transcript_id TCONS_00031095; exon_number 9; oId CUFF.59948.1; tss_id TSS63566;
5	Cufflinks	exon	215870531	215870629	.	-	.	gene_id GRMZM5G848768; transcript_id TCONS_00031095; exon_number 10; oId CUFF.59948.1; tss_id TSS63566;
5	Cufflinks	exon	215870752	215870878	.	-	.	gene_id GRMZM5G848768; transcript_id TCONS_00031095; exon_number 11; oId CUFF.59948.1; tss_id TSS63566;
5	Cufflinks	exon	215871050	215871134	.	-	.	gene_id GRMZM5G848768; transcript_id TCONS_00031095; exon_number 12; oId CUFF.59948.1; tss_id TSS63566;
5	Cufflinks	exon	215871616	215871692	.	-	.	gene_id GRMZM5G848768; transcript_id TCONS_00031095; exon_number 13; oId CUFF.59948.1; tss_id TSS63566;
5	Cufflinks	exon	215871807	215872218	.	-	.	gene_id GRMZM5G848768; transcript_id TCONS_00031095; exon_number 14; oId CUFF.59948.1; tss_id TSS63566;
5	Cufflinks	exon	217060912	217061698	.	-	.	gene_id GRMZM2G048470; transcript_id TCONS_00031137; exon_number 1; oId CUFF.60044.1; tss_id TSS63644;
6	Cufflinks	exon	3195407	3195736	.	+	.	gene_id GRMZM2G157296; transcript_id TCONS_00031183; exon_number 1; oId CUFF.60273.1; tss_id TSS63763;
6	Cufflinks	exon	3196839	3196953	.	+	.	gene_id GRMZM2G157296; transcript_id TCONS_00031183; exon_number 2; oId CUFF.60273.1; tss_id TSS63763;
6	Cufflinks	exon	3197493	3198606	.	+	.	gene_id GRMZM2G157296; transcript_id TCONS_00031183; exon_number 3; oId CUFF.60273.1; tss_id TSS63763;
6	Cufflinks	exon	8116573	8118254	.	+	.	gene_id XLOC_027310; transcript_id TCONS_00031233; exon_number 1; oId CUFF.60531.1; tss_id TSS63866;
6	Cufflinks	exon	9493165	9493347	.	+	.	gene_id GRMZM2G301884; transcript_id TCONS_00031238; exon_number 1; oId CUFF.60582.1; tss_id TSS63890;
6	Cufflinks	exon	9493443	9493581	.	+	.	gene_id GRMZM2G301884; transcript_id TCONS_00031238; exon_number 2; oId CUFF.60582.1; tss_id TSS63890;
6	Cufflinks	exon	9494117	9494304	.	+	.	gene_id GRMZM2G301884; transcript_id TCONS_00031238; exon_number 3; oId CUFF.60582.1; tss_id TSS63890;

6	Cufflinks	exon	9494411	9494582	.	+	.	gene_id GRMZM2G301884; transcript_id TCONS_00031238; exon_number 4; oId CUFF.60582.1; tss_id TSS63890;
6	Cufflinks	exon	9495165	9495367	.	+	.	gene_id GRMZM2G301884; transcript_id TCONS_00031238; exon_number 5; oId CUFF.60582.1; tss_id TSS63890;
6	Cufflinks	exon	9495668	9495790	.	+	.	gene_id GRMZM2G301884; transcript_id TCONS_00031238; exon_number 6; oId CUFF.60582.1; tss_id TSS63890;
6	Cufflinks	exon	9495869	9495972	.	+	.	gene_id GRMZM2G301884; transcript_id TCONS_00031238; exon_number 7; oId CUFF.60582.1; tss_id TSS63890;
6	Cufflinks	exon	9496218	9496796	.	+	.	gene_id GRMZM2G301884; transcript_id TCONS_00031238; exon_number 8; oId CUFF.60582.1; tss_id TSS63890;
6	Cufflinks	exon	21464815	21465424	.	+	.	gene_id XLOC_027524; transcript_id TCONS_00031456; exon_number 1; oId CUFF.61285.1; tss_id TSS64192;
6	Cufflinks	exon	24553394	24553545	.	+	.	gene_id XLOC_027544; transcript_id TCONS_00031477; exon_number 1; oId CUFF.61363.1; tss_id TSS64222;
6	Cufflinks	exon	24553943	24554144	.	+	.	gene_id XLOC_027544; transcript_id TCONS_00031477; exon_number 2; oId CUFF.61363.1; tss_id TSS64222;
6	Cufflinks	exon	27107889	27108204	.	+	.	gene_id GRMZM2G381051; transcript_id TCONS_00031491; exon_number 1; oId CUFF.61460.1; tss_id TSS64251;
6	Cufflinks	exon	27108286	27108407	.	+	.	gene_id GRMZM2G381051; transcript_id TCONS_00031491; exon_number 2; oId CUFF.61460.1; tss_id TSS64251;
6	Cufflinks	exon	27108527	27108793	.	+	.	gene_id GRMZM2G381051; transcript_id TCONS_00031491; exon_number 3; oId CUFF.61460.1; tss_id TSS64251;
6	Cufflinks	exon	27108904	27108948	.	+	.	gene_id GRMZM2G381051; transcript_id TCONS_00031491; exon_number 4; oId CUFF.61460.1; tss_id TSS64251;
6	Cufflinks	exon	27109112	27109160	.	+	.	gene_id GRMZM2G381051; transcript_id TCONS_00031491; exon_number 5; oId CUFF.61460.1; tss_id TSS64251;
6	Cufflinks	exon	27109251	27109360	.	+	.	gene_id GRMZM2G381051; transcript_id TCONS_00031491; exon_number 6; oId CUFF.61460.1; tss_id TSS64251;
6	Cufflinks	exon	27109446	27109522	.	+	.	gene_id GRMZM2G381051; transcript_id TCONS_00031491; exon_number 7; oId CUFF.61460.1; tss_id TSS64251;
6	Cufflinks	exon	27109630	27109680	.	+	.	gene_id GRMZM2G381051; transcript_id TCONS_00031491; exon_number 8; oId CUFF.61460.1; tss_id TSS64251;
6	Cufflinks	exon	27109758	27109822	.	+	.	gene_id GRMZM2G381051; transcript_id TCONS_00031491; exon_number 9; oId CUFF.61460.1; tss_id TSS64251;
6	Cufflinks	exon	27110190	27110344	.	+	.	gene_id GRMZM2G381051; transcript_id TCONS_00031491; exon_number 10; oId CUFF.61460.1; tss_id TSS64251;
6	Cufflinks	exon	27110452	27110492	.	+	.	gene_id GRMZM2G381051; transcript_id TCONS_00031491; exon_number 11; oId CUFF.61460.1; tss_id TSS64251;
6	Cufflinks	exon	27110565	27110619	.	+	.	gene_id GRMZM2G381051; transcript_id TCONS_00031491; exon_number 12; oId CUFF.61460.1; tss_id TSS64251;
6	Cufflinks	exon	27110781	27110837	.	+	.	gene_id GRMZM2G381051; transcript_id TCONS_00031491; exon_number 13; oId CUFF.61460.1; tss_id TSS64251;
6	Cufflinks	exon	27111167	27118645	.	+	.	gene_id GRMZM2G381051; transcript_id TCONS_00031491; exon_number 14; oId CUFF.61460.1; tss_id TSS64251;
6	Cufflinks	exon	34825142	34825650	.	+	.	gene_id GRMZM2G013581; transcript_id TCONS_00031530; exon_number 1; oId CUFF.61627.2; tss_id TSS64353;
6	Cufflinks	exon	34825837	34827192	.	+	.	gene_id GRMZM2G013581; transcript_id TCONS_00031530; exon_number 2; oId CUFF.61627.2; tss_id TSS64353;
6	Cufflinks	exon	34825142	34825592	.	+	.	gene_id GRMZM2G013581; transcript_id TCONS_00031531; exon_number 1; oId CUFF.61627.1; tss_id TSS64353;
6	Cufflinks	exon	34825837	34827192	.	+	.	gene_id GRMZM2G013581; transcript_id TCONS_00031531; exon_number 2; oId CUFF.61627.1; tss_id TSS64353;
6	Cufflinks	exon	36088959	36089225	.	+	.	gene_id GRMZM2G109821; transcript_id TCONS_00031549; exon_number 1; oId CUFF.61681.1; tss_id TSS64372;
6	Cufflinks	exon	36089310	36089415	.	+	.	gene_id GRMZM2G109821; transcript_id TCONS_00031549; exon_number 2; oId CUFF.61681.1; tss_id TSS64372;
6	Cufflinks	exon	36089515	36089860	.	+	.	gene_id GRMZM2G109821; transcript_id TCONS_00031549; exon_number 3; oId CUFF.61681.1; tss_id TSS64372;
6	Cufflinks	exon	36089960	36090265	.	+	.	gene_id GRMZM2G109821; transcript_id TCONS_00031549; exon_number 4; oId CUFF.61681.1; tss_id TSS64372;
6	Cufflinks	exon	36090365	36091188	.	+	.	gene_id GRMZM2G109821; transcript_id TCONS_00031549; exon_number 5; oId CUFF.61681.1; tss_id TSS64372;
6	Cufflinks	exon	78785552	78788014	.	+	.	gene_id GRMZM2G700969; transcript_id TCONS_00031814; exon_number 1; oId CUFF.62807.1; tss_id TSS64991;
6	Cufflinks	exon	78789565	78789971	.	+	.	gene_id GRMZM2G700969; transcript_id TCONS_00031814; exon_number 2; oId CUFF.62807.1; tss_id TSS64991;
6	Cufflinks	exon	78790278	78790300	.	+	.	gene_id GRMZM2G700969; transcript_id TCONS_00031814; exon_number 3; oId CUFF.62807.1; tss_id TSS64991;
6	Cufflinks	exon	85959795	85961400	.	+	.	gene_id GRMZM2G365961; transcript_id TCONS_00031888; exon_number 1; oId CUFF.63063.1; tss_id TSS65167;
6	Cufflinks	exon	86045729	86046130	.	+	.	gene_id GRMZM2G134930; transcript_id TCONS_00031889; exon_number 1; oId CUFF.63075.3; tss_id TSS65168;

6	Cufflinks	exon	86046365	86046427	.	+	.	gene_id GRMZM2G134930; transcript_id TCONS_00031889; exon_number 2; oId CUFF.63075.3; tss_id TSS65168;
6	Cufflinks	exon	86046569	86046685	.	+	.	gene_id GRMZM2G134930; transcript_id TCONS_00031889; exon_number 3; oId CUFF.63075.3; tss_id TSS65168;
6	Cufflinks	exon	86046796	86047090	.	+	.	gene_id GRMZM2G134930; transcript_id TCONS_00031889; exon_number 4; oId CUFF.63075.3; tss_id TSS65168;
6	Cufflinks	exon	86047273	86047428	.	+	.	gene_id GRMZM2G134930; transcript_id TCONS_00031889; exon_number 5; oId CUFF.63075.3; tss_id TSS65168;
6	Cufflinks	exon	86047511	86047662	.	+	.	gene_id GRMZM2G134930; transcript_id TCONS_00031889; exon_number 6; oId CUFF.63075.3; tss_id TSS65168;
6	Cufflinks	exon	86047741	86048261	.	+	.	gene_id GRMZM2G134930; transcript_id TCONS_00031889; exon_number 7; oId CUFF.63075.3; tss_id TSS65168;
6	Cufflinks	exon	86048455	86048942	.	+	.	gene_id GRMZM2G134930; transcript_id TCONS_00031889; exon_number 8; oId CUFF.63075.3; tss_id TSS65168;
6	Cufflinks	exon	93749599	93750313	.	+	.	gene_id XLOC_028005; transcript_id TCONS_00031999; exon_number 1; oId CUFF.63443.1; tss_id TSS65356;
6	Cufflinks	exon	94661977	94662538	.	+	.	gene_id GRMZM2G172413; transcript_id TCONS_00032012; exon_number 1; oId CUFF.63492.1; tss_id TSS65377;
6	Cufflinks	exon	94663049	94664066	.	+	.	gene_id GRMZM2G172413; transcript_id TCONS_00032012; exon_number 2; oId CUFF.63492.1; tss_id TSS65377;
6	Cufflinks	exon	97274349	97274902	.	+	.	gene_id GRMZM2G131020; transcript_id TCONS_00032043; exon_number 1; oId CUFF.63610.1; tss_id TSS65442;
6	Cufflinks	exon	97275967	97276123	.	+	.	gene_id GRMZM2G131020; transcript_id TCONS_00032043; exon_number 2; oId CUFF.63610.1; tss_id TSS65442;
6	Cufflinks	exon	97276774	97276997	.	+	.	gene_id GRMZM2G131020; transcript_id TCONS_00032043; exon_number 3; oId CUFF.63610.1; tss_id TSS65442;
6	Cufflinks	exon	97277099	97277272	.	+	.	gene_id GRMZM2G131020; transcript_id TCONS_00032043; exon_number 4; oId CUFF.63610.1; tss_id TSS65442;
6	Cufflinks	exon	97277383	97277738	.	+	.	gene_id GRMZM2G131020; transcript_id TCONS_00032043; exon_number 5; oId CUFF.63610.1; tss_id TSS65442;
6	Cufflinks	exon	104021788	104022587	.	+	.	gene_id XLOC_028114; transcript_id TCONS_00032120; exon_number 1; oId CUFF.63850.1; tss_id TSS65569;
6	Cufflinks	exon	121567042	121568491	.	+	.	gene_id XLOC_028309; transcript_id TCONS_00032333; exon_number 1; oId CUFF.64708.1; tss_id TSS66000;
6	Cufflinks	exon	125424395	125424938	.	+	.	gene_id XLOC_028357; transcript_id TCONS_00032388; exon_number 1; oId CUFF.64898.1; tss_id TSS66090;
6	Cufflinks	exon	126329458	126329901	.	+	.	gene_id GRMZM2G341729; transcript_id TCONS_00032395; exon_number 1; oId CUFF.64958.1; tss_id TSS66099;
6	Cufflinks	exon	126331422	126331531	.	+	.	gene_id GRMZM2G341729; transcript_id TCONS_00032395; exon_number 2; oId CUFF.64958.1; tss_id TSS66099;
6	Cufflinks	exon	126331620	126331738	.	+	.	gene_id GRMZM2G341729; transcript_id TCONS_00032395; exon_number 3; oId CUFF.64958.1; tss_id TSS66099;
6	Cufflinks	exon	126331839	126332466	.	+	.	gene_id GRMZM2G341729; transcript_id TCONS_00032395; exon_number 4; oId CUFF.64958.1; tss_id TSS66099;
6	Cufflinks	exon	128235624	128236497	.	+	.	gene_id GRMZM2G108714; transcript_id TCONS_00032408; exon_number 1; oId CUFF.65022.1; tss_id TSS66130;
6	Cufflinks	exon	128236623	128237977	.	+	.	gene_id GRMZM2G108714; transcript_id TCONS_00032408; exon_number 2; oId CUFF.65022.1; tss_id TSS66130;
6	Cufflinks	exon	147917374	147917661	.	+	.	gene_id GRMZM2G070199; transcript_id TCONS_00032677; exon_number 1; oId CUFF.66205.1; tss_id TSS66696;
6	Cufflinks	exon	147918307	147918440	.	+	.	gene_id GRMZM2G070199; transcript_id TCONS_00032677; exon_number 2; oId CUFF.66205.1; tss_id TSS66696;
6	Cufflinks	exon	147922388	147922817	.	+	.	gene_id GRMZM2G070199; transcript_id TCONS_00032677; exon_number 3; oId CUFF.66205.1; tss_id TSS66696;
6	Cufflinks	exon	148062224	148062563	.	+	.	gene_id GRMZM2G180328; transcript_id TCONS_00032681; exon_number 1; oId CUFF.66216.2; tss_id TSS66700;
6	Cufflinks	exon	148062670	148062947	.	+	.	gene_id GRMZM2G180328; transcript_id TCONS_00032681; exon_number 2; oId CUFF.66216.2; tss_id TSS66700;
6	Cufflinks	exon	148063442	148064507	.	+	.	gene_id GRMZM2G180328; transcript_id TCONS_00032681; exon_number 3; oId CUFF.66216.2; tss_id TSS66700;
6	Cufflinks	exon	148627957	148629694	.	+	.	gene_id XLOC_028633; transcript_id TCONS_00032690; exon_number 1; oId CUFF.66249.3; tss_id TSS66723;
6	Cufflinks	exon	151319337	151319654	.	+	.	gene_id GRMZM2G061906; transcript_id TCONS_00032740; exon_number 1; oId CUFF.66438.1; tss_id TSS66814;
6	Cufflinks	exon	151319790	151319858	.	+	.	gene_id GRMZM2G061906; transcript_id TCONS_00032740; exon_number 2; oId CUFF.66438.1; tss_id TSS66814;
6	Cufflinks	exon	151320384	151320461	.	+	.	gene_id GRMZM2G061906; transcript_id TCONS_00032740; exon_number 3; oId CUFF.66438.1; tss_id TSS66814;
6	Cufflinks	exon	151320547	151320709	.	+	.	gene_id GRMZM2G061906; transcript_id TCONS_00032740; exon_number 4; oId CUFF.66438.1; tss_id TSS66814;
6	Cufflinks	exon	151320804	151321751	.	+	.	gene_id GRMZM2G061906; transcript_id TCONS_00032740; exon_number 5; oId CUFF.66438.1; tss_id TSS66814;

6	Cufflinks	exon	151977873	151978127	.	+	.	gene_id GRMZM2G130239; transcript_id TCONS_00032754; exon_number 1; oId CUFF.66483.1; tss_id TSS66841;
6	Cufflinks	exon	151980168	151981648	.	+	.	gene_id GRMZM2G130239; transcript_id TCONS_00032754; exon_number 2; oId CUFF.66483.1; tss_id TSS66841;
6	Cufflinks	exon	156405022	156405355	.	+	.	gene_id GRMZM2G305159; transcript_id TCONS_00032856; exon_number 1; oId CUFF.66856.2; tss_id TSS67028;
6	Cufflinks	exon	156408340	156408394	.	+	.	gene_id GRMZM2G305159; transcript_id TCONS_00032856; exon_number 2; oId CUFF.66856.2; tss_id TSS67028;
6	Cufflinks	exon	156408483	156410419	.	+	.	gene_id GRMZM2G305159; transcript_id TCONS_00032856; exon_number 3; oId CUFF.66856.2; tss_id TSS67028;
6	Cufflinks	exon	156410512	156410670	.	+	.	gene_id GRMZM2G305159; transcript_id TCONS_00032856; exon_number 4; oId CUFF.66856.2; tss_id TSS67028;
6	Cufflinks	exon	156411675	156411973	.	+	.	gene_id GRMZM2G305159; transcript_id TCONS_00032856; exon_number 5; oId CUFF.66856.2; tss_id TSS67028;
6	Cufflinks	exon	156412345	156413226	.	+	.	gene_id GRMZM2G305159; transcript_id TCONS_00032856; exon_number 6; oId CUFF.66856.2; tss_id TSS67028;
6	Cufflinks	exon	156405012	156405355	.	+	.	gene_id GRMZM2G305159; transcript_id TCONS_00032857; exon_number 1; oId CUFF.66856.1; tss_id TSS67028;
6	Cufflinks	exon	156408340	156408394	.	+	.	gene_id GRMZM2G305159; transcript_id TCONS_00032857; exon_number 2; oId CUFF.66856.1; tss_id TSS67028;
6	Cufflinks	exon	156408483	156410419	.	+	.	gene_id GRMZM2G305159; transcript_id TCONS_00032857; exon_number 3; oId CUFF.66856.1; tss_id TSS67028;
6	Cufflinks	exon	156410512	156410670	.	+	.	gene_id GRMZM2G305159; transcript_id TCONS_00032857; exon_number 4; oId CUFF.66856.1; tss_id TSS67028;
6	Cufflinks	exon	156411405	156411576	.	+	.	gene_id GRMZM2G305159; transcript_id TCONS_00032857; exon_number 5; oId CUFF.66856.1; tss_id TSS67028;
6	Cufflinks	exon	156411675	156411973	.	+	.	gene_id GRMZM2G305159; transcript_id TCONS_00032857; exon_number 6; oId CUFF.66856.1; tss_id TSS67028;
6	Cufflinks	exon	156412345	156413226	.	+	.	gene_id GRMZM2G305159; transcript_id TCONS_00032857; exon_number 7; oId CUFF.66856.1; tss_id TSS67028;
6	Cufflinks	exon	160429758	160432862	.	+	.	gene_id GRMZM2G132212; transcript_id TCONS_00032963; exon_number 1; oId CUFF.67182.1; tss_id TSS67228;
6	Cufflinks	exon	160432954	160434506	.	+	.	gene_id GRMZM2G132212; transcript_id TCONS_00032963; exon_number 2; oId CUFF.67182.1; tss_id TSS67228;
6	Cufflinks	exon	161419537	161420979	.	+	.	gene_id XLOC_028899; transcript_id TCONS_00032987; exon_number 1; oId CUFF.67286.1; tss_id TSS67273;
6	Cufflinks	exon	164760526	164762818	.	+	.	gene_id XLOC_028987; transcript_id TCONS_00033084; exon_number 1; oId CUFF.67646.1; tss_id TSS67469;
6	Cufflinks	exon	167774318	167774632	.	+	.	gene_id GRMZM2G070659; transcript_id TCONS_00033169; exon_number 1; oId CUFF.67878.1; tss_id TSS67611;
6	Cufflinks	exon	167775791	167776029	.	+	.	gene_id GRMZM2G070659; transcript_id TCONS_00033169; exon_number 2; oId CUFF.67878.1; tss_id TSS67611;
6	Cufflinks	exon	167776103	167776223	.	+	.	gene_id GRMZM2G070659; transcript_id TCONS_00033169; exon_number 3; oId CUFF.67878.1; tss_id TSS67611;
6	Cufflinks	exon	167776288	167777084	.	+	.	gene_id GRMZM2G070659; transcript_id TCONS_00033169; exon_number 4; oId CUFF.67878.1; tss_id TSS67611;
6	Cufflinks	exon	168168646	168169311	.	+	.	gene_id XLOC_029066; transcript_id TCONS_00033180; exon_number 1; oId CUFF.67907.1; tss_id TSS67627;
6	Cufflinks	exon	168642436	168642571	.	+	.	gene_id GRMZM2G141925; transcript_id TCONS_00033186; exon_number 1; oId CUFF.67989.2; tss_id TSS67641;
6	Cufflinks	exon	168642684	168643842	.	+	.	gene_id GRMZM2G141925; transcript_id TCONS_00033186; exon_number 2; oId CUFF.67989.2; tss_id TSS67641;
6	Cufflinks	exon	168642324	168642571	.	+	.	gene_id GRMZM2G141925; transcript_id TCONS_00033187; exon_number 1; oId CUFF.67989.1; tss_id TSS67640;
6	Cufflinks	exon	168642681	168643842	.	+	.	gene_id GRMZM2G141925; transcript_id TCONS_00033187; exon_number 2; oId CUFF.67989.1; tss_id TSS67640;
6	Cufflinks	exon	3041977	3043750	.	-	.	gene_id GRMZM2G374827; transcript_id TCONS_00033242; exon_number 1; oId CUFF.60326.3; tss_id TSS67738;
6	Cufflinks	exon	3043837	3043898	.	-	.	gene_id GRMZM2G374827; transcript_id TCONS_00033242; exon_number 2; oId CUFF.60326.3; tss_id TSS67738;
6	Cufflinks	exon	3043990	3044062	.	-	.	gene_id GRMZM2G374827; transcript_id TCONS_00033242; exon_number 3; oId CUFF.60326.3; tss_id TSS67738;
6	Cufflinks	exon	3044155	3044225	.	-	.	gene_id GRMZM2G374827; transcript_id TCONS_00033242; exon_number 4; oId CUFF.60326.3; tss_id TSS67738;
6	Cufflinks	exon	3044309	3044502	.	-	.	gene_id GRMZM2G374827; transcript_id TCONS_00033242; exon_number 5; oId CUFF.60326.3; tss_id TSS67738;
6	Cufflinks	exon	3044608	3044764	.	-	.	gene_id GRMZM2G374827; transcript_id TCONS_00033242; exon_number 6; oId CUFF.60326.3; tss_id TSS67738;
6	Cufflinks	exon	3044868	3044906	.	-	.	gene_id GRMZM2G374827; transcript_id TCONS_00033242; exon_number 7; oId CUFF.60326.3; tss_id TSS67738;
6	Cufflinks	exon	3045000	3045055	.	-	.	gene_id GRMZM2G374827; transcript_id TCONS_00033242; exon_number 8; oId CUFF.60326.3; tss_id TSS67738;

6	Cufflinks	exon	3045138	3045191	.	-	.	gene_id GRMZM2G374827; transcript_id TCONS_00033242; exon_number 9; oId CUFF.60326.3; tss_id TSS67738;
6	Cufflinks	exon	3045287	3045390	.	-	.	gene_id GRMZM2G374827; transcript_id TCONS_00033242; exon_number 10; oId CUFF.60326.3; tss_id TSS67738;
6	Cufflinks	exon	3045555	3045655	.	-	.	gene_id GRMZM2G374827; transcript_id TCONS_00033242; exon_number 11; oId CUFF.60326.3; tss_id TSS67738;
6	Cufflinks	exon	3045732	3045802	.	-	.	gene_id GRMZM2G374827; transcript_id TCONS_00033242; exon_number 12; oId CUFF.60326.3; tss_id TSS67738;
6	Cufflinks	exon	3045914	3046013	.	-	.	gene_id GRMZM2G374827; transcript_id TCONS_00033242; exon_number 13; oId CUFF.60326.3; tss_id TSS67738;
6	Cufflinks	exon	3046112	3046182	.	-	.	gene_id GRMZM2G374827; transcript_id TCONS_00033242; exon_number 14; oId CUFF.60326.3; tss_id TSS67738;
6	Cufflinks	exon	3046344	3046400	.	-	.	gene_id GRMZM2G374827; transcript_id TCONS_00033242; exon_number 15; oId CUFF.60326.3; tss_id TSS67738;
6	Cufflinks	exon	3046494	3046616	.	-	.	gene_id GRMZM2G374827; transcript_id TCONS_00033242; exon_number 16; oId CUFF.60326.3; tss_id TSS67738;
6	Cufflinks	exon	3046861	3046911	.	-	.	gene_id GRMZM2G374827; transcript_id TCONS_00033242; exon_number 17; oId CUFF.60326.3; tss_id TSS67738;
6	Cufflinks	exon	3047003	3049466	.	-	.	gene_id GRMZM2G374827; transcript_id TCONS_00033242; exon_number 18; oId CUFF.60326.3; tss_id TSS67738;
6	Cufflinks	exon	3049542	3049676	.	-	.	gene_id GRMZM2G374827; transcript_id TCONS_00033242; exon_number 19; oId CUFF.60326.3; tss_id TSS67738;
6	Cufflinks	exon	3049890	3049943	.	-	.	gene_id GRMZM2G374827; transcript_id TCONS_00033242; exon_number 20; oId CUFF.60326.3; tss_id TSS67738;
6	Cufflinks	exon	3050081	3050158	.	-	.	gene_id GRMZM2G374827; transcript_id TCONS_00033242; exon_number 21; oId CUFF.60326.3; tss_id TSS67738;
6	Cufflinks	exon	3051122	3051204	.	-	.	gene_id GRMZM2G374827; transcript_id TCONS_00033242; exon_number 22; oId CUFF.60326.3; tss_id TSS67738;
6	Cufflinks	exon	3051465	3051510	.	-	.	gene_id GRMZM2G374827; transcript_id TCONS_00033242; exon_number 23; oId CUFF.60326.3; tss_id TSS67738;
6	Cufflinks	exon	3051648	3051773	.	-	.	gene_id GRMZM2G374827; transcript_id TCONS_00033242; exon_number 24; oId CUFF.60326.3; tss_id TSS67738;
6	Cufflinks	exon	3052383	3052667	.	-	.	gene_id GRMZM2G374827; transcript_id TCONS_00033242; exon_number 25; oId CUFF.60326.3; tss_id TSS67738;
6	Cufflinks	exon	3053069	3053364	.	-	.	gene_id GRMZM2G374827; transcript_id TCONS_00033242; exon_number 26; oId CUFF.60326.3; tss_id TSS67738;
6	Cufflinks	exon	3041977	3043750	.	-	.	gene_id GRMZM2G374827; transcript_id TCONS_00033245; exon_number 1; oId CUFF.60326.1; tss_id TSS67738;
6	Cufflinks	exon	3043837	3043898	.	-	.	gene_id GRMZM2G374827; transcript_id TCONS_00033245; exon_number 2; oId CUFF.60326.1; tss_id TSS67738;
6	Cufflinks	exon	3043990	3044062	.	-	.	gene_id GRMZM2G374827; transcript_id TCONS_00033245; exon_number 3; oId CUFF.60326.1; tss_id TSS67738;
6	Cufflinks	exon	3044155	3044225	.	-	.	gene_id GRMZM2G374827; transcript_id TCONS_00033245; exon_number 4; oId CUFF.60326.1; tss_id TSS67738;
6	Cufflinks	exon	3044309	3044502	.	-	.	gene_id GRMZM2G374827; transcript_id TCONS_00033245; exon_number 5; oId CUFF.60326.1; tss_id TSS67738;
6	Cufflinks	exon	3044608	3044764	.	-	.	gene_id GRMZM2G374827; transcript_id TCONS_00033245; exon_number 6; oId CUFF.60326.1; tss_id TSS67738;
6	Cufflinks	exon	3044868	3044906	.	-	.	gene_id GRMZM2G374827; transcript_id TCONS_00033245; exon_number 7; oId CUFF.60326.1; tss_id TSS67738;
6	Cufflinks	exon	3045000	3045055	.	-	.	gene_id GRMZM2G374827; transcript_id TCONS_00033245; exon_number 8; oId CUFF.60326.1; tss_id TSS67738;
6	Cufflinks	exon	3045138	3045191	.	-	.	gene_id GRMZM2G374827; transcript_id TCONS_00033245; exon_number 9; oId CUFF.60326.1; tss_id TSS67738;
6	Cufflinks	exon	3045287	3045413	.	-	.	gene_id GRMZM2G374827; transcript_id TCONS_00033245; exon_number 10; oId CUFF.60326.1; tss_id TSS67738;
6	Cufflinks	exon	3045555	3045655	.	-	.	gene_id GRMZM2G374827; transcript_id TCONS_00033245; exon_number 11; oId CUFF.60326.1; tss_id TSS67738;
6	Cufflinks	exon	3045732	3045802	.	-	.	gene_id GRMZM2G374827; transcript_id TCONS_00033245; exon_number 12; oId CUFF.60326.1; tss_id TSS67738;
6	Cufflinks	exon	3045914	3046013	.	-	.	gene_id GRMZM2G374827; transcript_id TCONS_00033245; exon_number 13; oId CUFF.60326.1; tss_id TSS67738;
6	Cufflinks	exon	3046112	3046182	.	-	.	gene_id GRMZM2G374827; transcript_id TCONS_00033245; exon_number 14; oId CUFF.60326.1; tss_id TSS67738;
6	Cufflinks	exon	3046344	3046400	.	-	.	gene_id GRMZM2G374827; transcript_id TCONS_00033245; exon_number 15; oId CUFF.60326.1; tss_id TSS67738;
6	Cufflinks	exon	3046494	3046616	.	-	.	gene_id GRMZM2G374827; transcript_id TCONS_00033245; exon_number 16; oId CUFF.60326.1; tss_id TSS67738;
6	Cufflinks	exon	3046861	3046911	.	-	.	gene_id GRMZM2G374827; transcript_id TCONS_00033245; exon_number 17; oId CUFF.60326.1; tss_id TSS67738;
6	Cufflinks	exon	3047003	3049466	.	-	.	gene_id GRMZM2G374827; transcript_id TCONS_00033245; exon_number 18; oId CUFF.60326.1; tss_id TSS67738;

6	Cufflinks	exon	3049542	3049676	.	-	.	gene_id GRMZM2G374827; transcript_id TCONS_00033245; exon_number 19; oId CUFF.60326.1; tss_id TSS67738;
6	Cufflinks	exon	3049890	3049943	.	-	.	gene_id GRMZM2G374827; transcript_id TCONS_00033245; exon_number 20; oId CUFF.60326.1; tss_id TSS67738;
6	Cufflinks	exon	3050081	3050158	.	-	.	gene_id GRMZM2G374827; transcript_id TCONS_00033245; exon_number 21; oId CUFF.60326.1; tss_id TSS67738;
6	Cufflinks	exon	3051122	3051204	.	-	.	gene_id GRMZM2G374827; transcript_id TCONS_00033245; exon_number 22; oId CUFF.60326.1; tss_id TSS67738;
6	Cufflinks	exon	3051465	3051510	.	-	.	gene_id GRMZM2G374827; transcript_id TCONS_00033245; exon_number 23; oId CUFF.60326.1; tss_id TSS67738;
6	Cufflinks	exon	3051648	3051773	.	-	.	gene_id GRMZM2G374827; transcript_id TCONS_00033245; exon_number 24; oId CUFF.60326.1; tss_id TSS67738;
6	Cufflinks	exon	3052383	3052667	.	-	.	gene_id GRMZM2G374827; transcript_id TCONS_00033245; exon_number 25; oId CUFF.60326.1; tss_id TSS67738;
6	Cufflinks	exon	3053069	3053364	.	-	.	gene_id GRMZM2G374827; transcript_id TCONS_00033245; exon_number 26; oId CUFF.60326.1; tss_id TSS67738;
6	Cufflinks	exon	6186556	6190745	.	-	.	gene_id XLOC_029150; transcript_id TCONS_00033285; exon_number 1; oId CUFF.60447.1; tss_id TSS67834;
6	Cufflinks	exon	13653532	13654945	.	-	.	gene_id XLOC_029297; transcript_id TCONS_00033446; exon_number 1; oId CUFF.60878.1; tss_id TSS68122;
6	Cufflinks	exon	14021636	14022577	.	-	.	gene_id XLOC_029304; transcript_id TCONS_00033453; exon_number 1; oId CUFF.60922.1; tss_id TSS68145;
6	Cufflinks	exon	35062195	35062742	.	-	.	gene_id XLOC_029472; transcript_id TCONS_00033650; exon_number 1; oId CUFF.61624.1; tss_id TSS68567;
6	Cufflinks	exon	36483546	36484012	.	-	.	gene_id XLOC_029485; transcript_id TCONS_00033663; exon_number 1; oId CUFF.61676.1; tss_id TSS68588;
6	Cufflinks	exon	59260577	59261040	.	-	.	gene_id XLOC_029599; transcript_id TCONS_00033793; exon_number 1; oId CUFF.62205.1; tss_id TSS68846;
6	Cufflinks	exon	67615942	67616607	.	-	.	gene_id XLOC_029644; transcript_id TCONS_00033844; exon_number 1; oId CUFF.62468.1; tss_id TSS68967;
6	Cufflinks	exon	70452079	70453918	.	-	.	gene_id GRMZM2G447347; transcript_id TCONS_00033861; exon_number 1; oId CUFF.62576.1; tss_id TSS69026;
6	Cufflinks	exon	70454064	70454153	.	-	.	gene_id GRMZM2G447347; transcript_id TCONS_00033861; exon_number 2; oId CUFF.62576.1; tss_id TSS69026;
6	Cufflinks	exon	70454241	70454503	.	-	.	gene_id GRMZM2G447347; transcript_id TCONS_00033861; exon_number 3; oId CUFF.62576.1; tss_id TSS69026;
6	Cufflinks	exon	74511770	74512313	.	-	.	gene_id XLOC_029685; transcript_id TCONS_00033888; exon_number 1; oId CUFF.62681.1; tss_id TSS69098;
6	Cufflinks	exon	78786923	78787431	.	-	.	gene_id GRMZM2G700968; transcript_id TCONS_00033912; exon_number 1; oId CUFF.62809.1; tss_id TSS69149;
6	Cufflinks	exon	83989255	83989982	.	-	.	gene_id GRMZM2G065888; transcript_id TCONS_00033953; exon_number 1; oId CUFF.63002.6; tss_id TSS69217;
6	Cufflinks	exon	83990091	83990167	.	-	.	gene_id GRMZM2G065888; transcript_id TCONS_00033953; exon_number 2; oId CUFF.63002.6; tss_id TSS69217;
6	Cufflinks	exon	83990362	83990478	.	-	.	gene_id GRMZM2G065888; transcript_id TCONS_00033953; exon_number 3; oId CUFF.63002.6; tss_id TSS69217;
6	Cufflinks	exon	83990647	83990747	.	-	.	gene_id GRMZM2G065888; transcript_id TCONS_00033953; exon_number 4; oId CUFF.63002.6; tss_id TSS69217;
6	Cufflinks	exon	83990825	83990894	.	-	.	gene_id GRMZM2G065888; transcript_id TCONS_00033953; exon_number 5; oId CUFF.63002.6; tss_id TSS69217;
6	Cufflinks	exon	83991085	83991179	.	-	.	gene_id GRMZM2G065888; transcript_id TCONS_00033953; exon_number 6; oId CUFF.63002.6; tss_id TSS69217;
6	Cufflinks	exon	83991733	83991794	.	-	.	gene_id GRMZM2G065888; transcript_id TCONS_00033953; exon_number 7; oId CUFF.63002.6; tss_id TSS69217;
6	Cufflinks	exon	83992121	83992211	.	-	.	gene_id GRMZM2G065888; transcript_id TCONS_00033953; exon_number 8; oId CUFF.63002.6; tss_id TSS69217;
6	Cufflinks	exon	83992290	83992397	.	-	.	gene_id GRMZM2G065888; transcript_id TCONS_00033953; exon_number 9; oId CUFF.63002.6; tss_id TSS69217;
6	Cufflinks	exon	83992495	83992808	.	-	.	gene_id GRMZM2G065888; transcript_id TCONS_00033953; exon_number 10; oId CUFF.63002.6; tss_id TSS69217;
6	Cufflinks	exon	83994068	83994599	.	-	.	gene_id GRMZM2G065888; transcript_id TCONS_00033953; exon_number 11; oId CUFF.63002.6; tss_id TSS69217;
6	Cufflinks	exon	86050769	86052508	.	-	.	gene_id GRMZM2G134759; transcript_id TCONS_00033973; exon_number 1; oId CUFF.63066.1; tss_id TSS69254;
6	Cufflinks	exon	92976168	92977409	.	-	.	gene_id GRMZM2G043822; transcript_id TCONS_00034058; exon_number 1; oId CUFF.63394.2; tss_id TSS69454;
6	Cufflinks	exon	92977991	92978998	.	-	.	gene_id GRMZM2G043822; transcript_id TCONS_00034058; exon_number 2; oId CUFF.63394.2; tss_id TSS69454;
6	Cufflinks	exon	92976168	92977409	.	-	.	gene_id GRMZM2G043822; transcript_id TCONS_00034059; exon_number 1; oId CUFF.63394.1; tss_id TSS69454;
6	Cufflinks	exon	92977991	92978260	.	-	.	gene_id GRMZM2G043822; transcript_id TCONS_00034059; exon_number 2; oId CUFF.63394.1; tss_id TSS69454;

6	Cufflinks	exon	92978524	92979012	.	-	.	gene_id GRMZM2G043822; transcript_id TCONS_00034059; exon_number 3; oId CUFF.63394.1; tss_id TSS69454;
6	Cufflinks	exon	93375032	93376082	.	-	.	gene_id GRMZM2G048092; transcript_id TCONS_00034075; exon_number 1; oId CUFF.63433.1; tss_id TSS69483;
6	Cufflinks	exon	93376165	93376275	.	-	.	gene_id GRMZM2G048092; transcript_id TCONS_00034075; exon_number 2; oId CUFF.63433.1; tss_id TSS69483;
6	Cufflinks	exon	93376970	93377100	.	-	.	gene_id GRMZM2G048092; transcript_id TCONS_00034075; exon_number 3; oId CUFF.63433.1; tss_id TSS69483;
6	Cufflinks	exon	93377325	93377483	.	-	.	gene_id GRMZM2G048092; transcript_id TCONS_00034075; exon_number 4; oId CUFF.63433.1; tss_id TSS69483;
6	Cufflinks	exon	100364390	100365022	.	-	.	gene_id GRMZM2G041714; transcript_id TCONS_00034159; exon_number 1; oId CUFF.63711.1; tss_id TSS69634;
6	Cufflinks	exon	100365167	100365388	.	-	.	gene_id GRMZM2G041714; transcript_id TCONS_00034159; exon_number 2; oId CUFF.63711.1; tss_id TSS69634;
6	Cufflinks	exon	102760900	102761948	.	-	.	gene_id GRMZM2G043310; transcript_id TCONS_00034169; exon_number 1; oId CUFF.63772.1; tss_id TSS69662;
6	Cufflinks	exon	102763547	102763662	.	-	.	gene_id GRMZM2G043310; transcript_id TCONS_00034169; exon_number 2; oId CUFF.63772.1; tss_id TSS69662;
6	Cufflinks	exon	102763747	102763822	.	-	.	gene_id GRMZM2G043310; transcript_id TCONS_00034169; exon_number 3; oId CUFF.63772.1; tss_id TSS69662;
6	Cufflinks	exon	102764387	102765449	.	-	.	gene_id GRMZM2G043310; transcript_id TCONS_00034169; exon_number 4; oId CUFF.63772.1; tss_id TSS69662;
6	Cufflinks	exon	108237703	108238301	.	-	.	gene_id XLOC_029999; transcript_id TCONS_00034252; exon_number 1; oId CUFF.64079.1; tss_id TSS69838;
6	Cufflinks	exon	116979182	116981091	.	-	.	gene_id GRMZM2G157113; transcript_id TCONS_00034346; exon_number 1; oId CUFF.64490.1; tss_id TSS70061;
6	Cufflinks	exon	116981174	116981588	.	-	.	gene_id GRMZM2G157113; transcript_id TCONS_00034346; exon_number 2; oId CUFF.64490.1; tss_id TSS70061;
6	Cufflinks	exon	124546088	124546690	.	-	.	gene_id GRMZM2G176910; transcript_id TCONS_00034434; exon_number 1; oId CUFF.64847.1; tss_id TSS70268;
6	Cufflinks	exon	124546840	124546929	.	-	.	gene_id GRMZM2G176910; transcript_id TCONS_00034434; exon_number 2; oId CUFF.64847.1; tss_id TSS70268;
6	Cufflinks	exon	124547347	124547514	.	-	.	gene_id GRMZM2G176910; transcript_id TCONS_00034434; exon_number 3; oId CUFF.64847.1; tss_id TSS70268;
6	Cufflinks	exon	124547596	124547868	.	-	.	gene_id GRMZM2G176910; transcript_id TCONS_00034434; exon_number 4; oId CUFF.64847.1; tss_id TSS70268;
6	Cufflinks	exon	124547968	124548074	.	-	.	gene_id GRMZM2G176910; transcript_id TCONS_00034434; exon_number 5; oId CUFF.64847.1; tss_id TSS70268;
6	Cufflinks	exon	124548576	124548966	.	-	.	gene_id GRMZM2G176910; transcript_id TCONS_00034434; exon_number 6; oId CUFF.64847.1; tss_id TSS70268;
6	Cufflinks	exon	134280739	134281131	.	-	.	gene_id GRMZM2G339091; transcript_id TCONS_00034588; exon_number 1; oId CUFF.65354.1; tss_id TSS70568;
6	Cufflinks	exon	134281247	134281355	.	-	.	gene_id GRMZM2G339091; transcript_id TCONS_00034588; exon_number 2; oId CUFF.65354.1; tss_id TSS70568;
6	Cufflinks	exon	134283123	134283218	.	-	.	gene_id GRMZM2G339091; transcript_id TCONS_00034588; exon_number 3; oId CUFF.65354.1; tss_id TSS70568;
6	Cufflinks	exon	134283299	134283444	.	-	.	gene_id GRMZM2G339091; transcript_id TCONS_00034588; exon_number 4; oId CUFF.65354.1; tss_id TSS70568;
6	Cufflinks	exon	134283701	134283982	.	-	.	gene_id GRMZM2G339091; transcript_id TCONS_00034588; exon_number 5; oId CUFF.65354.1; tss_id TSS70568;
6	Cufflinks	exon	134284137	134284222	.	-	.	gene_id GRMZM2G339091; transcript_id TCONS_00034588; exon_number 6; oId CUFF.65354.1; tss_id TSS70568;
6	Cufflinks	exon	134284343	134284531	.	-	.	gene_id GRMZM2G339091; transcript_id TCONS_00034588; exon_number 7; oId CUFF.65354.1; tss_id TSS70568;
6	Cufflinks	exon	134284650	134285176	.	-	.	gene_id GRMZM2G339091; transcript_id TCONS_00034588; exon_number 8; oId CUFF.65354.1; tss_id TSS70568;
6	Cufflinks	exon	134280739	134281125	.	-	.	gene_id GRMZM2G339091; transcript_id TCONS_00034589; exon_number 1; oId CUFF.65354.4; tss_id TSS70568;
6	Cufflinks	exon	134281247	134281355	.	-	.	gene_id GRMZM2G339091; transcript_id TCONS_00034589; exon_number 2; oId CUFF.65354.4; tss_id TSS70568;
6	Cufflinks	exon	134283123	134283218	.	-	.	gene_id GRMZM2G339091; transcript_id TCONS_00034589; exon_number 3; oId CUFF.65354.4; tss_id TSS70568;
6	Cufflinks	exon	134283299	134283444	.	-	.	gene_id GRMZM2G339091; transcript_id TCONS_00034589; exon_number 4; oId CUFF.65354.4; tss_id TSS70568;
6	Cufflinks	exon	134283701	134283982	.	-	.	gene_id GRMZM2G339091; transcript_id TCONS_00034589; exon_number 5; oId CUFF.65354.4; tss_id TSS70568;
6	Cufflinks	exon	134284137	134284222	.	-	.	gene_id GRMZM2G339091; transcript_id TCONS_00034589; exon_number 6; oId CUFF.65354.4; tss_id TSS70568;
6	Cufflinks	exon	134284343	134284531	.	-	.	gene_id GRMZM2G339091; transcript_id TCONS_00034589; exon_number 7; oId CUFF.65354.4; tss_id TSS70568;
6	Cufflinks	exon	134284650	134285174	.	-	.	gene_id GRMZM2G339091; transcript_id TCONS_00034589; exon_number 8; oId CUFF.65354.4; tss_id TSS70568;

6	Cufflinks	exon	134280739	134281122	.	-	.	gene_id GRMZM2G339091; transcript_id TCONS_00034590; exon_number 1; oId CUFF.65354.3; tss_id TSS70568;
6	Cufflinks	exon	134281247	134281355	.	-	.	gene_id GRMZM2G339091; transcript_id TCONS_00034590; exon_number 2; oId CUFF.65354.3; tss_id TSS70568;
6	Cufflinks	exon	134283123	134283218	.	-	.	gene_id GRMZM2G339091; transcript_id TCONS_00034590; exon_number 3; oId CUFF.65354.3; tss_id TSS70568;
6	Cufflinks	exon	134283299	134283444	.	-	.	gene_id GRMZM2G339091; transcript_id TCONS_00034590; exon_number 4; oId CUFF.65354.3; tss_id TSS70568;
6	Cufflinks	exon	134283701	134283982	.	-	.	gene_id GRMZM2G339091; transcript_id TCONS_00034590; exon_number 5; oId CUFF.65354.3; tss_id TSS70568;
6	Cufflinks	exon	134284137	134284222	.	-	.	gene_id GRMZM2G339091; transcript_id TCONS_00034590; exon_number 6; oId CUFF.65354.3; tss_id TSS70568;
6	Cufflinks	exon	134284343	134284531	.	-	.	gene_id GRMZM2G339091; transcript_id TCONS_00034590; exon_number 7; oId CUFF.65354.3; tss_id TSS70568;
6	Cufflinks	exon	134284650	134285168	.	-	.	gene_id GRMZM2G339091; transcript_id TCONS_00034590; exon_number 8; oId CUFF.65354.3; tss_id TSS70568;
6	Cufflinks	exon	140914068	140915319	.	-	.	gene_id GRMZM2G093325; transcript_id TCONS_00034690; exon_number 1; oId CUFF.65708.1; tss_id TSS70803;
6	Cufflinks	exon	140915440	140915871	.	-	.	gene_id GRMZM2G093325; transcript_id TCONS_00034690; exon_number 2; oId CUFF.65708.1; tss_id TSS70803;
6	Cufflinks	exon	142832661	142834858	.	-	.	gene_id XLOC_030415; transcript_id TCONS_00034725; exon_number 1; oId CUFF.65834.1; tss_id TSS70874;
6	Cufflinks	exon	145511301	145511757	.	-	.	gene_id GRMZM2G479608; transcript_id TCONS_00034765; exon_number 1; oId CUFF.66037.1; tss_id TSS70979;
6	Cufflinks	exon	145511859	145512016	.	-	.	gene_id GRMZM2G479608; transcript_id TCONS_00034765; exon_number 2; oId CUFF.66037.1; tss_id TSS70979;
6	Cufflinks	exon	145512116	145512229	.	-	.	gene_id GRMZM2G479608; transcript_id TCONS_00034765; exon_number 3; oId CUFF.66037.1; tss_id TSS70979;
6	Cufflinks	exon	145515561	145516044	.	-	.	gene_id GRMZM2G479608; transcript_id TCONS_00034765; exon_number 4; oId CUFF.66037.1; tss_id TSS70979;
6	Cufflinks	exon	147921865	147922850	.	-	.	gene_id XLOC_030488; transcript_id TCONS_00034808; exon_number 1; oId CUFF.66207.1; tss_id TSS71083;
6	Cufflinks	exon	148628022	148628730	.	-	.	gene_id GRMZM2G111955; transcript_id TCONS_00034824; exon_number 1; oId CUFF.66250.1; tss_id TSS71114;
6	Cufflinks	exon	148628845	148628910	.	-	.	gene_id GRMZM2G111955; transcript_id TCONS_00034824; exon_number 2; oId CUFF.66250.1; tss_id TSS71114;
6	Cufflinks	exon	148629005	148629067	.	-	.	gene_id GRMZM2G111955; transcript_id TCONS_00034824; exon_number 3; oId CUFF.66250.1; tss_id TSS71114;
6	Cufflinks	exon	148629252	148629394	.	-	.	gene_id GRMZM2G111955; transcript_id TCONS_00034824; exon_number 4; oId CUFF.66250.1; tss_id TSS71114;
6	Cufflinks	exon	148629912	148630038	.	-	.	gene_id GRMZM2G111955; transcript_id TCONS_00034824; exon_number 5; oId CUFF.66250.1; tss_id TSS71114;
6	Cufflinks	exon	148632129	148632519	.	-	.	gene_id GRMZM2G111955; transcript_id TCONS_00034824; exon_number 6; oId CUFF.66250.1; tss_id TSS71114;
6	Cufflinks	exon	151980700	151981737	.	-	.	gene_id GRMZM2G431350; transcript_id TCONS_00034896; exon_number 1; oId CUFF.66484.1; tss_id TSS71225;
6	Cufflinks	exon	151981823	151981904	.	-	.	gene_id GRMZM2G431350; transcript_id TCONS_00034896; exon_number 2; oId CUFF.66484.1; tss_id TSS71225;
6	Cufflinks	exon	151982208	151982285	.	-	.	gene_id GRMZM2G431350; transcript_id TCONS_00034896; exon_number 3; oId CUFF.66484.1; tss_id TSS71225;
6	Cufflinks	exon	151982398	151982545	.	-	.	gene_id GRMZM2G431350; transcript_id TCONS_00034896; exon_number 4; oId CUFF.66484.1; tss_id TSS71225;
6	Cufflinks	exon	151983196	151983355	.	-	.	gene_id GRMZM2G431350; transcript_id TCONS_00034896; exon_number 5; oId CUFF.66484.1; tss_id TSS71225;
6	Cufflinks	exon	151983618	151983784	.	-	.	gene_id GRMZM2G431350; transcript_id TCONS_00034896; exon_number 6; oId CUFF.66484.1; tss_id TSS71225;
6	Cufflinks	exon	153507939	153508736	.	-	.	gene_id GRMZM2G127350; transcript_id TCONS_00034940; exon_number 1; oId CUFF.66687.2; tss_id TSS71316;
6	Cufflinks	exon	153508832	153508918	.	-	.	gene_id GRMZM2G127350; transcript_id TCONS_00034940; exon_number 2; oId CUFF.66687.2; tss_id TSS71316;
6	Cufflinks	exon	153509023	153509136	.	-	.	gene_id GRMZM2G127350; transcript_id TCONS_00034940; exon_number 3; oId CUFF.66687.2; tss_id TSS71316;
6	Cufflinks	exon	153509219	153509444	.	-	.	gene_id GRMZM2G127350; transcript_id TCONS_00034940; exon_number 4; oId CUFF.66687.2; tss_id TSS71316;
6	Cufflinks	exon	153509576	153509755	.	-	.	gene_id GRMZM2G127350; transcript_id TCONS_00034940; exon_number 5; oId CUFF.66687.2; tss_id TSS71316;
6	Cufflinks	exon	153509849	153509992	.	-	.	gene_id GRMZM2G127350; transcript_id TCONS_00034940; exon_number 6; oId CUFF.66687.2; tss_id TSS71316;
6	Cufflinks	exon	153510084	153510254	.	-	.	gene_id GRMZM2G127350; transcript_id TCONS_00034940; exon_number 7; oId CUFF.66687.2; tss_id TSS71316;
6	Cufflinks	exon	153510363	153510593	.	-	.	gene_id GRMZM2G127350; transcript_id TCONS_00034940; exon_number 8; oId CUFF.66687.2; tss_id TSS71316;

6	Cufflinks	exon	153512285	153512599	.	-	.	gene_id GRMZM2G127350; transcript_id TCONS_00034940; exon_number 9; oId CUFF.66687.2; tss_id TSS71316;
6	Cufflinks	exon	153978666	153979619	.	-	.	gene_id XLOC_030598; transcript_id TCONS_00034943; exon_number 1; oId CUFF.66622.1; tss_id TSS71324;
6	Cufflinks	exon	153980084	153981320	.	-	.	gene_id XLOC_030599; transcript_id TCONS_00034944; exon_number 1; oId CUFF.66624.1; tss_id TSS71325;
6	Cufflinks	exon	158380664	158381595	.	-	.	gene_id XLOC_030694; transcript_id TCONS_00035055; exon_number 1; oId CUFF.67003.1; tss_id TSS71511;
6	Cufflinks	exon	160084716	160087818	.	-	.	gene_id GRMZM2G058314; transcript_id TCONS_00035087; exon_number 1; oId CUFF.67142.1; tss_id TSS71565;
6	Cufflinks	exon	164572072	164572883	.	-	.	gene_id GRMZM2G038284; transcript_id TCONS_00035213; exon_number 1; oId CUFF.67619.1; tss_id TSS71818;
6	Cufflinks	exon	164573372	164573661	.	-	.	gene_id GRMZM2G038284; transcript_id TCONS_00035213; exon_number 2; oId CUFF.67619.1; tss_id TSS71818;
6	Cufflinks	exon	164573807	164573884	.	-	.	gene_id GRMZM2G038284; transcript_id TCONS_00035213; exon_number 3; oId CUFF.67619.1; tss_id TSS71818;
6	Cufflinks	exon	164574921	164575060	.	-	.	gene_id GRMZM2G038284; transcript_id TCONS_00035213; exon_number 4; oId CUFF.67619.1; tss_id TSS71818;
6	Cufflinks	exon	164575169	164575526	.	-	.	gene_id GRMZM2G038284; transcript_id TCONS_00035213; exon_number 5; oId CUFF.67619.1; tss_id TSS71818;
6	Cufflinks	exon	164757491	164758850	.	-	.	gene_id XLOC_030842; transcript_id TCONS_00035220; exon_number 1; oId CUFF.67611.1; tss_id TSS71831;
7	Cufflinks	exon	1272958	1274081	.	+	.	gene_id XLOC_030952; transcript_id TCONS_00035343; exon_number 1; oId CUFF.68031.1; tss_id TSS72058;
7	Cufflinks	exon	2137542	2137745	.	+	.	gene_id AC205122.4_FG003; transcript_id TCONS_00035354; exon_number 1; oId CUFF.68105.1; tss_id TSS72089;
7	Cufflinks	exon	2137836	2137872	.	+	.	gene_id AC205122.4_FG003; transcript_id TCONS_00035354; exon_number 2; oId CUFF.68105.1; tss_id TSS72089;
7	Cufflinks	exon	2138481	2139233	.	+	.	gene_id AC205122.4_FG003; transcript_id TCONS_00035354; exon_number 3; oId CUFF.68105.1; tss_id TSS72089;
7	Cufflinks	exon	9551283	9551687	.	+	.	gene_id XLOC_031066; transcript_id TCONS_00035471; exon_number 1; oId CUFF.68519.1; tss_id TSS72313;
7	Cufflinks	exon	10699818	10700185	.	+	.	gene_id XLOC_031080; transcript_id TCONS_00035485; exon_number 1; oId CUFF.68575.1; tss_id TSS72348;
7	Cufflinks	exon	18621004	18621402	.	+	.	gene_id XLOC_031162; transcript_id TCONS_00035574; exon_number 1; oId CUFF.68883.1; tss_id TSS72494;
7	Cufflinks	exon	46202870	46203190	.	+	.	gene_id GRMZM5G893913; transcript_id TCONS_00035763; exon_number 1; oId CUFF.69654.1; tss_id TSS72933;
7	Cufflinks	exon	46204169	46204469	.	+	.	gene_id GRMZM5G893913; transcript_id TCONS_00035763; exon_number 2; oId CUFF.69654.1; tss_id TSS72933;
7	Cufflinks	exon	46205084	46205200	.	+	.	gene_id GRMZM5G893913; transcript_id TCONS_00035763; exon_number 3; oId CUFF.69654.1; tss_id TSS72933;
7	Cufflinks	exon	46205286	46207496	.	+	.	gene_id GRMZM5G893913; transcript_id TCONS_00035763; exon_number 4; oId CUFF.69654.1; tss_id TSS72933;
7	Cufflinks	exon	50166355	50166973	.	+	.	gene_id GRMZM2G061562; transcript_id TCONS_00035787; exon_number 1; oId CUFF.69789.1; tss_id TSS73016;
7	Cufflinks	exon	50167823	50167887	.	+	.	gene_id GRMZM2G061562; transcript_id TCONS_00035787; exon_number 2; oId CUFF.69789.1; tss_id TSS73016;
7	Cufflinks	exon	50167981	50170023	.	+	.	gene_id GRMZM2G061562; transcript_id TCONS_00035787; exon_number 3; oId CUFF.69789.1; tss_id TSS73016;
7	Cufflinks	exon	50170271	50170421	.	+	.	gene_id GRMZM2G061562; transcript_id TCONS_00035787; exon_number 4; oId CUFF.69789.1; tss_id TSS73016;
7	Cufflinks	exon	50171335	50172435	.	+	.	gene_id GRMZM2G061562; transcript_id TCONS_00035787; exon_number 5; oId CUFF.69789.1; tss_id TSS73016;
7	Cufflinks	exon	83788520	83789843	.	+	.	gene_id GRMZM2G092137; transcript_id TCONS_00035904; exon_number 1; oId CUFF.70513.1; tss_id TSS73375;
7	Cufflinks	exon	83790423	83791400	.	+	.	gene_id GRMZM2G092137; transcript_id TCONS_00035904; exon_number 2; oId CUFF.70513.1; tss_id TSS73375;
7	Cufflinks	exon	115996804	115997993	.	+	.	gene_id GRMZM2G125853; transcript_id TCONS_00036098; exon_number 1; oId CUFF.71400.2; tss_id TSS73813;
7	Cufflinks	exon	115999455	115999522	.	+	.	gene_id GRMZM2G125853; transcript_id TCONS_00036098; exon_number 2; oId CUFF.71400.2; tss_id TSS73813;
7	Cufflinks	exon	116009987	116010105	.	+	.	gene_id GRMZM2G125853; transcript_id TCONS_00036098; exon_number 3; oId CUFF.71400.2; tss_id TSS73813;
7	Cufflinks	exon	116010253	116010834	.	+	.	gene_id GRMZM2G125853; transcript_id TCONS_00036098; exon_number 4; oId CUFF.71400.2; tss_id TSS73813;
7	Cufflinks	exon	119983090	119984190	.	+	.	gene_id XLOC_031663; transcript_id TCONS_00036131; exon_number 1; oId CUFF.71557.1; tss_id TSS73896;
7	Cufflinks	exon	124780337	124781632	.	+	.	gene_id XLOC_031723; transcript_id TCONS_00036197; exon_number 1; oId CUFF.71798.1; tss_id TSS74056;
7	Cufflinks	exon	129753381	129754241	.	+	.	gene_id GRMZM2G096952; transcript_id TCONS_00036248; exon_number 1; oId CUFF.72017.1; tss_id TSS74174;

7	Cufflinks	exon	129754525	129754604	.	+	.	gene_id GRMZM2G096952; transcript_id TCONS_00036248; exon_number 2; oId CUFF.72017.1; tss_id TSS74174;
7	Cufflinks	exon	129754830	129755294	.	+	.	gene_id GRMZM2G096952; transcript_id TCONS_00036248; exon_number 3; oId CUFF.72017.1; tss_id TSS74174;
7	Cufflinks	exon	129756488	129756574	.	+	.	gene_id GRMZM2G096952; transcript_id TCONS_00036248; exon_number 4; oId CUFF.72017.1; tss_id TSS74174;
7	Cufflinks	exon	129756703	129756834	.	+	.	gene_id GRMZM2G096952; transcript_id TCONS_00036248; exon_number 5; oId CUFF.72017.1; tss_id TSS74174;
7	Cufflinks	exon	129756974	129757165	.	+	.	gene_id GRMZM2G096952; transcript_id TCONS_00036248; exon_number 6; oId CUFF.72017.1; tss_id TSS74174;
7	Cufflinks	exon	129757290	129758428	.	+	.	gene_id GRMZM2G096952; transcript_id TCONS_00036248; exon_number 7; oId CUFF.72017.1; tss_id TSS74174;
7	Cufflinks	exon	132423389	132423996	.	+	.	gene_id GRMZM2G055469; transcript_id TCONS_00036283; exon_number 1; oId CUFF.72171.1; tss_id TSS74247;
7	Cufflinks	exon	132424174	132424223	.	+	.	gene_id GRMZM2G055469; transcript_id TCONS_00036283; exon_number 2; oId CUFF.72171.1; tss_id TSS74247;
7	Cufflinks	exon	132424325	132424392	.	+	.	gene_id GRMZM2G055469; transcript_id TCONS_00036283; exon_number 3; oId CUFF.72171.1; tss_id TSS74247;
7	Cufflinks	exon	132425188	132425354	.	+	.	gene_id GRMZM2G055469; transcript_id TCONS_00036283; exon_number 4; oId CUFF.72171.1; tss_id TSS74247;
7	Cufflinks	exon	132425459	132426153	.	+	.	gene_id GRMZM2G055469; transcript_id TCONS_00036283; exon_number 5; oId CUFF.72171.1; tss_id TSS74247;
7	Cufflinks	exon	132548737	132549369	.	+	.	gene_id GRMZM2G176430; transcript_id TCONS_00036286; exon_number 1; oId CUFF.72181.1; tss_id TSS74255;
7	Cufflinks	exon	132549496	132549988	.	+	.	gene_id GRMZM2G176430; transcript_id TCONS_00036286; exon_number 2; oId CUFF.72181.1; tss_id TSS74255;
7	Cufflinks	exon	132550158	132550201	.	+	.	gene_id GRMZM2G176430; transcript_id TCONS_00036286; exon_number 3; oId CUFF.72181.1; tss_id TSS74255;
7	Cufflinks	exon	132550283	132550378	.	+	.	gene_id GRMZM2G176430; transcript_id TCONS_00036286; exon_number 4; oId CUFF.72181.1; tss_id TSS74255;
7	Cufflinks	exon	132550472	132550982	.	+	.	gene_id GRMZM2G176430; transcript_id TCONS_00036286; exon_number 5; oId CUFF.72181.1; tss_id TSS74255;
7	Cufflinks	exon	132551085	132551662	.	+	.	gene_id GRMZM2G176430; transcript_id TCONS_00036286; exon_number 6; oId CUFF.72181.1; tss_id TSS74255;
7	Cufflinks	exon	140848023	140848380	.	+	.	gene_id XLOC_031896; transcript_id TCONS_00036400; exon_number 1; oId CUFF.72663.1; tss_id TSS74515;
7	Cufflinks	exon	141630856	141631979	.	+	.	gene_id GRMZM2G001639; transcript_id TCONS_00036409; exon_number 1; oId CUFF.72708.1; tss_id TSS74544;
7	Cufflinks	exon	141632324	141632442	.	+	.	gene_id GRMZM2G001639; transcript_id TCONS_00036409; exon_number 2; oId CUFF.72708.1; tss_id TSS74544;
7	Cufflinks	exon	141632771	141633496	.	+	.	gene_id GRMZM2G001639; transcript_id TCONS_00036409; exon_number 3; oId CUFF.72708.1; tss_id TSS74544;
7	Cufflinks	exon	142270553	142270955	.	+	.	gene_id GRMZM2G165272; transcript_id TCONS_00036429; exon_number 1; oId CUFF.72794.1; tss_id TSS74592;
7	Cufflinks	exon	142271089	142272208	.	+	.	gene_id GRMZM2G165272; transcript_id TCONS_00036429; exon_number 2; oId CUFF.72794.1; tss_id TSS74592;
7	Cufflinks	exon	143244178	143246692	.	+	.	gene_id XLOC_031931; transcript_id TCONS_00036442; exon_number 1; oId CUFF.72851.1; tss_id TSS74625;
7	Cufflinks	exon	143286301	143287016	.	+	.	gene_id XLOC_031935; transcript_id TCONS_00036446; exon_number 1; oId CUFF.72868.1; tss_id TSS74639;
7	Cufflinks	exon	143287112	143287306	.	+	.	gene_id XLOC_031935; transcript_id TCONS_00036446; exon_number 2; oId CUFF.72868.1; tss_id TSS74639;
7	Cufflinks	exon	143287412	143287483	.	+	.	gene_id XLOC_031935; transcript_id TCONS_00036446; exon_number 3; oId CUFF.72868.1; tss_id TSS74639;
7	Cufflinks	exon	143287869	143287981	.	+	.	gene_id XLOC_031935; transcript_id TCONS_00036446; exon_number 4; oId CUFF.72868.1; tss_id TSS74639;
7	Cufflinks	exon	143288076	143288804	.	+	.	gene_id XLOC_031935; transcript_id TCONS_00036446; exon_number 5; oId CUFF.72868.1; tss_id TSS74639;
7	Cufflinks	exon	143286931	143287016	.	+	.	gene_id XLOC_031935; transcript_id TCONS_00036447; exon_number 1; oId CUFF.72868.2; tss_id TSS74640;
7	Cufflinks	exon	143287112	143287306	.	+	.	gene_id XLOC_031935; transcript_id TCONS_00036447; exon_number 2; oId CUFF.72868.2; tss_id TSS74640;
7	Cufflinks	exon	143287412	143287483	.	+	.	gene_id XLOC_031935; transcript_id TCONS_00036447; exon_number 3; oId CUFF.72868.2; tss_id TSS74640;
7	Cufflinks	exon	143287869	143287981	.	+	.	gene_id XLOC_031935; transcript_id TCONS_00036447; exon_number 4; oId CUFF.72868.2; tss_id TSS74640;
7	Cufflinks	exon	143288060	143288804	.	+	.	gene_id XLOC_031935; transcript_id TCONS_00036447; exon_number 5; oId CUFF.72868.2; tss_id TSS74640;
7	Cufflinks	exon	143551214	143551688	.	+	.	gene_id GRMZM2G091586; transcript_id TCONS_00036452; exon_number 1; oId CUFF.72889.2; tss_id TSS74649;
7	Cufflinks	exon	143552384	143552408	.	+	.	gene_id GRMZM2G091586; transcript_id TCONS_00036452; exon_number 2; oId CUFF.72889.2; tss_id TSS74649;

7	Cufflinks	exon	143555670	143555799	.	+	.	gene_id GRMZM2G091586; transcript_id TCONS_00036452; exon_number 3; oId CUFF.72889.2; tss_id TSS74649;
7	Cufflinks	exon	143555893	143556023	.	+	.	gene_id GRMZM2G091586; transcript_id TCONS_00036452; exon_number 4; oId CUFF.72889.2; tss_id TSS74649;
7	Cufflinks	exon	143556120	143556208	.	+	.	gene_id GRMZM2G091586; transcript_id TCONS_00036452; exon_number 5; oId CUFF.72889.2; tss_id TSS74649;
7	Cufflinks	exon	143556636	143556712	.	+	.	gene_id GRMZM2G091586; transcript_id TCONS_00036452; exon_number 6; oId CUFF.72889.2; tss_id TSS74649;
7	Cufflinks	exon	143556787	143557231	.	+	.	gene_id GRMZM2G091586; transcript_id TCONS_00036452; exon_number 7; oId CUFF.72889.2; tss_id TSS74649;
7	Cufflinks	exon	145809169	145809829	.	+	.	gene_id XLOC_031968; transcript_id TCONS_00036487; exon_number 1; oId CUFF.73003.1; tss_id TSS74718;
7	Cufflinks	exon	150906444	150909453	.	+	.	gene_id GRMZM2G038449; transcript_id TCONS_00036571; exon_number 1; oId CUFF.73386.1; tss_id TSS74942;
7	Cufflinks	exon	152431465	152432095	.	+	.	gene_id GRMZM2G325008; transcript_id TCONS_00036591; exon_number 1; oId CUFF.73487.3; tss_id TSS74990;
7	Cufflinks	exon	152432238	152433566	.	+	.	gene_id GRMZM2G325008; transcript_id TCONS_00036591; exon_number 2; oId CUFF.73487.3; tss_id TSS74990;
7	Cufflinks	exon	152437016	152437043	.	+	.	gene_id GRMZM2G325008; transcript_id TCONS_00036591; exon_number 3; oId CUFF.73487.3; tss_id TSS74990;
7	Cufflinks	exon	152437879	152438328	.	+	.	gene_id GRMZM2G325008; transcript_id TCONS_00036591; exon_number 4; oId CUFF.73487.3; tss_id TSS74990;
7	Cufflinks	exon	153211728	153212562	.	+	.	gene_id XLOC_032067; transcript_id TCONS_00036603; exon_number 1; oId CUFF.73543.1; tss_id TSS75021;
7	Cufflinks	exon	156224860	156225621	.	+	.	gene_id XLOC_032125; transcript_id TCONS_00036666; exon_number 1; oId CUFF.73788.1; tss_id TSS75130;
7	Cufflinks	exon	157477186	157478848	.	+	.	gene_id XLOC_032147; transcript_id TCONS_00036695; exon_number 1; oId CUFF.73840.1; tss_id TSS75173;
7	Cufflinks	exon	166191360	166191945	.	+	.	gene_id GRMZM2G079397; transcript_id TCONS_00036874; exon_number 1; oId CUFF.74499.1; tss_id TSS75512;
7	Cufflinks	exon	166196500	166197002	.	+	.	gene_id GRMZM2G079397; transcript_id TCONS_00036874; exon_number 2; oId CUFF.74499.1; tss_id TSS75512;
7	Cufflinks	exon	171577972	171578887	.	+	.	gene_id XLOC_032421; transcript_id TCONS_00037003; exon_number 1; oId CUFF.74895.1; tss_id TSS75726;
7	Cufflinks	exon	172301623	172302947	.	+	.	gene_id XLOC_032437; transcript_id TCONS_00037020; exon_number 1; oId CUFF.74985.1; tss_id TSS75759;
7	Cufflinks	exon	172980469	172980652	.	+	.	gene_id XLOC_032461; transcript_id TCONS_00037046; exon_number 1; oId CUFF.75074.1; tss_id TSS75802;
7	Cufflinks	exon	172980747	172981129	.	+	.	gene_id XLOC_032461; transcript_id TCONS_00037046; exon_number 2; oId CUFF.75074.1; tss_id TSS75802;
7	Cufflinks	exon	173490053	173490328	.	+	.	gene_id XLOC_032475; transcript_id TCONS_00037062; exon_number 1; oId CUFF.75138.1; tss_id TSS75830;
7	Cufflinks	exon	173556354	173557829	.	+	.	gene_id XLOC_032477; transcript_id TCONS_00037064; exon_number 1; oId CUFF.75141.1; tss_id TSS75833;
7	Cufflinks	exon	174834523	174834980	.	+	.	gene_id GRMZM2G134284; transcript_id TCONS_00037099; exon_number 1; oId CUFF.75299.3; tss_id TSS75897;
7	Cufflinks	exon	174835253	174835412	.	+	.	gene_id GRMZM2G134284; transcript_id TCONS_00037099; exon_number 2; oId CUFF.75299.3; tss_id TSS75897;
7	Cufflinks	exon	174835493	174835588	.	+	.	gene_id GRMZM2G134284; transcript_id TCONS_00037099; exon_number 3; oId CUFF.75299.3; tss_id TSS75897;
7	Cufflinks	exon	174835669	174835783	.	+	.	gene_id GRMZM2G134284; transcript_id TCONS_00037099; exon_number 4; oId CUFF.75299.3; tss_id TSS75897;
7	Cufflinks	exon	174835884	174836046	.	+	.	gene_id GRMZM2G134284; transcript_id TCONS_00037099; exon_number 5; oId CUFF.75299.3; tss_id TSS75897;
7	Cufflinks	exon	174836266	174836437	.	+	.	gene_id GRMZM2G134284; transcript_id TCONS_00037099; exon_number 6; oId CUFF.75299.3; tss_id TSS75897;
7	Cufflinks	exon	174837029	174837151	.	+	.	gene_id GRMZM2G134284; transcript_id TCONS_00037099; exon_number 7; oId CUFF.75299.3; tss_id TSS75897;
7	Cufflinks	exon	174837227	174837676	.	+	.	gene_id GRMZM2G134284; transcript_id TCONS_00037099; exon_number 8; oId CUFF.75299.3; tss_id TSS75897;
7	Cufflinks	exon	174837752	174838330	.	+	.	gene_id GRMZM2G134284; transcript_id TCONS_00037099; exon_number 9; oId CUFF.75299.3; tss_id TSS75897;
7	Cufflinks	exon	174838581	174838649	.	+	.	gene_id GRMZM2G134284; transcript_id TCONS_00037099; exon_number 10; oId CUFF.75299.3; tss_id TSS75897;
7	Cufflinks	exon	174839212	174839328	.	+	.	gene_id GRMZM2G134284; transcript_id TCONS_00037099; exon_number 11; oId CUFF.75299.3; tss_id TSS75897;
7	Cufflinks	exon	174839407	174839652	.	+	.	gene_id GRMZM2G134284; transcript_id TCONS_00037099; exon_number 12; oId CUFF.75299.3; tss_id TSS75897;
7	Cufflinks	exon	174840852	174840899	.	+	.	gene_id GRMZM2G134284; transcript_id TCONS_00037099; exon_number 13; oId CUFF.75299.3; tss_id TSS75897;
7	Cufflinks	exon	174840980	174841120	.	+	.	gene_id GRMZM2G134284; transcript_id TCONS_00037099; exon_number 14; oId CUFF.75299.3; tss_id TSS75897;

7	Cufflinks	exon	174841203	174841283	.	+	.	gene_id GRMZM2G134284; transcript_id TCONS_00037099; exon_number 15; oId CUFF.75299.3; tss_id TSS75897;
7	Cufflinks	exon	174841363	174841425	.	+	.	gene_id GRMZM2G134284; transcript_id TCONS_00037099; exon_number 16; oId CUFF.75299.3; tss_id TSS75897;
7	Cufflinks	exon	174841530	174841637	.	+	.	gene_id GRMZM2G134284; transcript_id TCONS_00037099; exon_number 17; oId CUFF.75299.3; tss_id TSS75897;
7	Cufflinks	exon	174844427	174844507	.	+	.	gene_id GRMZM2G134284; transcript_id TCONS_00037099; exon_number 18; oId CUFF.75299.3; tss_id TSS75897;
7	Cufflinks	exon	174844611	174844658	.	+	.	gene_id GRMZM2G134284; transcript_id TCONS_00037099; exon_number 19; oId CUFF.75299.3; tss_id TSS75897;
7	Cufflinks	exon	174845630	174845704	.	+	.	gene_id GRMZM2G134284; transcript_id TCONS_00037099; exon_number 20; oId CUFF.75299.3; tss_id TSS75897;
7	Cufflinks	exon	174845995	174846090	.	+	.	gene_id GRMZM2G134284; transcript_id TCONS_00037099; exon_number 21; oId CUFF.75299.3; tss_id TSS75897;
7	Cufflinks	exon	174846172	174846252	.	+	.	gene_id GRMZM2G134284; transcript_id TCONS_00037099; exon_number 22; oId CUFF.75299.3; tss_id TSS75897;
7	Cufflinks	exon	174846332	174847147	.	+	.	gene_id GRMZM2G134284; transcript_id TCONS_00037099; exon_number 23; oId CUFF.75299.3; tss_id TSS75897;
7	Cufflinks	exon	175612069	175614512	.	+	.	gene_id GRMZM2G032376; transcript_id TCONS_00037117; exon_number 1; oId CUFF.75339.3; tss_id TSS75928;
7	Cufflinks	exon	175614596	175614726	.	+	.	gene_id GRMZM2G032376; transcript_id TCONS_00037117; exon_number 2; oId CUFF.75339.3; tss_id TSS75928;
7	Cufflinks	exon	175614789	175615007	.	+	.	gene_id GRMZM2G032376; transcript_id TCONS_00037117; exon_number 3; oId CUFF.75339.3; tss_id TSS75928;
7	Cufflinks	exon	175615100	175615355	.	+	.	gene_id GRMZM2G032376; transcript_id TCONS_00037117; exon_number 4; oId CUFF.75339.3; tss_id TSS75928;
7	Cufflinks	exon	175615445	175615786	.	+	.	gene_id GRMZM2G032376; transcript_id TCONS_00037117; exon_number 5; oId CUFF.75339.3; tss_id TSS75928;
7	Cufflinks	exon	176182074	176182480	.	+	.	gene_id GRMZM2G000818; transcript_id TCONS_00037125; exon_number 1; oId CUFF.75317.1; tss_id TSS75950;
7	Cufflinks	exon	176182569	176183077	.	+	.	gene_id GRMZM2G000818; transcript_id TCONS_00037125; exon_number 2; oId CUFF.75317.1; tss_id TSS75950;
7	Cufflinks	exon	176436743	176438053	.	+	.	gene_id XLOC_032527; transcript_id TCONS_00037126; exon_number 1; oId CUFF.75328.1; tss_id TSS75953;
7	Cufflinks	exon	176438150	176439281	.	+	.	gene_id XLOC_032527; transcript_id TCONS_00037126; exon_number 2; oId CUFF.75328.1; tss_id TSS75953;
7	Cufflinks	exon	1270762	1272128	.	-	.	gene_id GRMZM2G420743; transcript_id TCONS_00037152; exon_number 1; oId CUFF.68025.1; tss_id TSS75993;
7	Cufflinks	exon	10578984	10581443	.	-	.	gene_id GRMZM2G328171; transcript_id TCONS_00037293; exon_number 1; oId CUFF.68585.1; tss_id TSS76265;
7	Cufflinks	exon	15013267	15014342	.	-	.	gene_id GRMZM2G028129; transcript_id TCONS_00037337; exon_number 1; oId CUFF.68760.1; tss_id TSS76359;
7	Cufflinks	exon	15014439	15015214	.	-	.	gene_id GRMZM2G028129; transcript_id TCONS_00037337; exon_number 2; oId CUFF.68760.1; tss_id TSS76359;
7	Cufflinks	exon	50170951	50171841	.	-	.	gene_id XLOC_032918; transcript_id TCONS_00037567; exon_number 1; oId CUFF.69791.1; tss_id TSS76886;
7	Cufflinks	exon	50171941	50172266	.	-	.	gene_id XLOC_032918; transcript_id TCONS_00037567; exon_number 2; oId CUFF.69791.1; tss_id TSS76886;
7	Cufflinks	exon	99233077	99234124	.	-	.	gene_id XLOC_033120; transcript_id TCONS_00037789; exon_number 1; oId CUFF.70961.1; tss_id TSS77482;
7	Cufflinks	exon	108871160	108872024	.	-	.	gene_id GRMZM2G126507; transcript_id TCONS_00037868; exon_number 1; oId CUFF.71218.1; tss_id TSS77644;
7	Cufflinks	exon	108872164	108872221	.	-	.	gene_id GRMZM2G126507; transcript_id TCONS_00037868; exon_number 2; oId CUFF.71218.1; tss_id TSS77644;
7	Cufflinks	exon	108872330	108872687	.	-	.	gene_id GRMZM2G126507; transcript_id TCONS_00037868; exon_number 3; oId CUFF.71218.1; tss_id TSS77644;
7	Cufflinks	exon	108872770	108872854	.	-	.	gene_id GRMZM2G126507; transcript_id TCONS_00037868; exon_number 4; oId CUFF.71218.1; tss_id TSS77644;
7	Cufflinks	exon	108872948	108873174	.	-	.	gene_id GRMZM2G126507; transcript_id TCONS_00037868; exon_number 5; oId CUFF.71218.1; tss_id TSS77644;
7	Cufflinks	exon	108873275	108873444	.	-	.	gene_id GRMZM2G126507; transcript_id TCONS_00037868; exon_number 6; oId CUFF.71218.1; tss_id TSS77644;
7	Cufflinks	exon	108873966	108874537	.	-	.	gene_id GRMZM2G126507; transcript_id TCONS_00037868; exon_number 7; oId CUFF.71218.1; tss_id TSS77644;
7	Cufflinks	exon	111685454	111686024	.	-	.	gene_id GRMZM2G159221; transcript_id TCONS_00037890; exon_number 1; oId CUFF.71307.1; tss_id TSS77693;
7	Cufflinks	exon	111686153	111686251	.	-	.	gene_id GRMZM2G159221; transcript_id TCONS_00037890; exon_number 2; oId CUFF.71307.1; tss_id TSS77693;
7	Cufflinks	exon	111686684	111687316	.	-	.	gene_id GRMZM2G159221; transcript_id TCONS_00037890; exon_number 3; oId CUFF.71307.1; tss_id TSS77693;
7	Cufflinks	exon	111688247	111688840	.	-	.	gene_id GRMZM2G159221; transcript_id TCONS_00037890; exon_number 4; oId CUFF.71307.1; tss_id TSS77693;

7	Cufflinks	exon	119983118	119983535	.	-	.	gene_id XLOC_033259; transcript_id TCONS_00037943; exon_number 1; oId CUFF.71558.1; tss_id TSS77831;
7	Cufflinks	exon	129753354	129754232	.	-	.	gene_id XLOC_033326; transcript_id TCONS_00038014; exon_number 1; oId CUFF.72016.1; tss_id TSS78034;
7	Cufflinks	exon	141674424	141674758	.	-	.	gene_id GRMZM2G073228; transcript_id TCONS_00038178; exon_number 1; oId CUFF.72717.1; tss_id TSS78399;
7	Cufflinks	exon	141675165	141675273	.	-	.	gene_id GRMZM2G073228; transcript_id TCONS_00038178; exon_number 2; oId CUFF.72717.1; tss_id TSS78399;
7	Cufflinks	exon	141675393	141675517	.	-	.	gene_id GRMZM2G073228; transcript_id TCONS_00038178; exon_number 3; oId CUFF.72717.1; tss_id TSS78399;
7	Cufflinks	exon	141677111	141677155	.	-	.	gene_id GRMZM2G073228; transcript_id TCONS_00038178; exon_number 4; oId CUFF.72717.1; tss_id TSS78399;
7	Cufflinks	exon	141677317	141677381	.	-	.	gene_id GRMZM2G073228; transcript_id TCONS_00038178; exon_number 5; oId CUFF.72717.1; tss_id TSS78399;
7	Cufflinks	exon	141677493	141677659	.	-	.	gene_id GRMZM2G073228; transcript_id TCONS_00038178; exon_number 6; oId CUFF.72717.1; tss_id TSS78399;
7	Cufflinks	exon	141678196	141678723	.	-	.	gene_id GRMZM2G073228; transcript_id TCONS_00038178; exon_number 7; oId CUFF.72717.1; tss_id TSS78399;
7	Cufflinks	exon	142270740	142271931	.	-	.	gene_id GRMZM2G465685; transcript_id TCONS_00038198; exon_number 1; oId CUFF.72793.1; tss_id TSS78432;
7	Cufflinks	exon	143551214	143551708	.	-	.	gene_id XLOC_033518; transcript_id TCONS_00038218; exon_number 1; oId CUFF.72890.1; tss_id TSS78474;
7	Cufflinks	exon	145808406	145810041	.	-	.	gene_id GRMZM2G006117; transcript_id TCONS_00038261; exon_number 1; oId CUFF.73002.1; tss_id TSS78537;
7	Cufflinks	exon	145810151	145810304	.	-	.	gene_id GRMZM2G006117; transcript_id TCONS_00038261; exon_number 2; oId CUFF.73002.1; tss_id TSS78537;
7	Cufflinks	exon	145810392	145810504	.	-	.	gene_id GRMZM2G006117; transcript_id TCONS_00038261; exon_number 3; oId CUFF.73002.1; tss_id TSS78537;
7	Cufflinks	exon	145810624	145810729	.	-	.	gene_id GRMZM2G006117; transcript_id TCONS_00038261; exon_number 4; oId CUFF.73002.1; tss_id TSS78537;
7	Cufflinks	exon	145811487	145811596	.	-	.	gene_id GRMZM2G006117; transcript_id TCONS_00038261; exon_number 5; oId CUFF.73002.1; tss_id TSS78537;
7	Cufflinks	exon	145811694	145811858	.	-	.	gene_id GRMZM2G006117; transcript_id TCONS_00038261; exon_number 6; oId CUFF.73002.1; tss_id TSS78537;
7	Cufflinks	exon	145812601	145812881	.	-	.	gene_id GRMZM2G006117; transcript_id TCONS_00038261; exon_number 7; oId CUFF.73002.1; tss_id TSS78537;
7	Cufflinks	exon	160190641	160190959	.	-	.	gene_id XLOC_033788; transcript_id TCONS_00038521; exon_number 1; oId CUFF.74036.1; tss_id TSS79113;
7	Cufflinks	exon	160854309	160856519	.	-	.	gene_id XLOC_033801; transcript_id TCONS_00038534; exon_number 1; oId CUFF.74098.1; tss_id TSS79134;
7	Cufflinks	exon	161376439	161376899	.	-	.	gene_id XLOC_033806; transcript_id TCONS_00038539; exon_number 1; oId CUFF.74109.1; tss_id TSS79153;
7	Cufflinks	exon	162113500	162114498	.	-	.	gene_id XLOC_033829; transcript_id TCONS_00038563; exon_number 1; oId CUFF.74213.1; tss_id TSS79208;
7	Cufflinks	exon	163042057	163042441	.	-	.	gene_id GRMZM2G113696; transcript_id TCONS_00038583; exon_number 1; oId CUFF.74267.1; tss_id TSS79256;
7	Cufflinks	exon	163042909	163042983	.	-	.	gene_id GRMZM2G113696; transcript_id TCONS_00038583; exon_number 2; oId CUFF.74267.1; tss_id TSS79256;
7	Cufflinks	exon	163043069	163043125	.	-	.	gene_id GRMZM2G113696; transcript_id TCONS_00038583; exon_number 3; oId CUFF.74267.1; tss_id TSS79256;
7	Cufflinks	exon	163043206	163043328	.	-	.	gene_id GRMZM2G113696; transcript_id TCONS_00038583; exon_number 4; oId CUFF.74267.1; tss_id TSS79256;
7	Cufflinks	exon	163044736	163044869	.	-	.	gene_id GRMZM2G113696; transcript_id TCONS_00038583; exon_number 5; oId CUFF.74267.1; tss_id TSS79256;
7	Cufflinks	exon	163045021	163045208	.	-	.	gene_id GRMZM2G113696; transcript_id TCONS_00038583; exon_number 6; oId CUFF.74267.1; tss_id TSS79256;
7	Cufflinks	exon	166502692	166503314	.	-	.	gene_id GRMZM2G117707; transcript_id TCONS_00038642; exon_number 1; oId CUFF.74517.1; tss_id TSS79363;
7	Cufflinks	exon	166503943	166504188	.	-	.	gene_id GRMZM2G117707; transcript_id TCONS_00038642; exon_number 2; oId CUFF.74517.1; tss_id TSS79363;
7	Cufflinks	exon	166504291	166504567	.	-	.	gene_id GRMZM2G117707; transcript_id TCONS_00038642; exon_number 3; oId CUFF.74517.1; tss_id TSS79363;
7	Cufflinks	exon	166505532	166505809	.	-	.	gene_id GRMZM2G117707; transcript_id TCONS_00038642; exon_number 4; oId CUFF.74517.1; tss_id TSS79363;
7	Cufflinks	exon	166505900	166506595	.	-	.	gene_id GRMZM2G117707; transcript_id TCONS_00038642; exon_number 5; oId CUFF.74517.1; tss_id TSS79363;
7	Cufflinks	exon	167947896	167948665	.	-	.	gene_id GRMZM2G150683; transcript_id TCONS_00038678; exon_number 1; oId CUFF.74619.1; tss_id TSS79427;
7	Cufflinks	exon	167948781	167948808	.	-	.	gene_id GRMZM2G150683; transcript_id TCONS_00038678; exon_number 2; oId CUFF.74619.1; tss_id TSS79427;
7	Cufflinks	exon	167953941	167954255	.	-	.	gene_id GRMZM2G150683; transcript_id TCONS_00038678; exon_number 3; oId CUFF.74619.1; tss_id TSS79427;

7	Cufflinks	exon	167954645	167954751	.	-	.	gene_id GRMZM2G150683; transcript_id TCONS_00038678; exon_number 4; oId CUFF.74619.1; tss_id TSS79427;
7	Cufflinks	exon	167955752	167955945	.	-	.	gene_id GRMZM2G150683; transcript_id TCONS_00038678; exon_number 5; oId CUFF.74619.1; tss_id TSS79427;
7	Cufflinks	exon	173595180	173595504	.	-	.	gene_id GRMZM2G469380; transcript_id TCONS_00038820; exon_number 1; oId CUFF.75115.1; tss_id TSS79701;
7	Cufflinks	exon	173595630	173596356	.	-	.	gene_id GRMZM2G469380; transcript_id TCONS_00038820; exon_number 2; oId CUFF.75115.1; tss_id TSS79701;
7	Cufflinks	exon	173596452	173596955	.	-	.	gene_id GRMZM2G469380; transcript_id TCONS_00038820; exon_number 3; oId CUFF.75115.1; tss_id TSS79701;
7	Cufflinks	exon	174614465	174615416	.	-	.	gene_id XLOC_034093; transcript_id TCONS_00038857; exon_number 1; oId CUFF.75210.1; tss_id TSS79751;
7	Cufflinks	exon	174627232	174627851	.	-	.	gene_id GRMZM2G056569; transcript_id TCONS_00038858; exon_number 1; oId CUFF.75224.1; tss_id TSS79753;
7	Cufflinks	exon	174628101	174628241	.	-	.	gene_id GRMZM2G056569; transcript_id TCONS_00038858; exon_number 2; oId CUFF.75224.1; tss_id TSS79753;
7	Cufflinks	exon	174628331	174628717	.	-	.	gene_id GRMZM2G056569; transcript_id TCONS_00038858; exon_number 3; oId CUFF.75224.1; tss_id TSS79753;
7	Cufflinks	exon	174628888	174629133	.	-	.	gene_id GRMZM2G056569; transcript_id TCONS_00038858; exon_number 4; oId CUFF.75224.1; tss_id TSS79753;
7	Cufflinks	exon	174629929	174631734	.	-	.	gene_id GRMZM2G056569; transcript_id TCONS_00038858; exon_number 5; oId CUFF.75224.1; tss_id TSS79753;
7	Cufflinks	exon	174832020	174832278	.	-	.	gene_id GRMZM2G134295; transcript_id TCONS_00038862; exon_number 1; oId CUFF.75297.1; tss_id TSS79763;
7	Cufflinks	exon	174832629	174832781	.	-	.	gene_id GRMZM2G134295; transcript_id TCONS_00038862; exon_number 2; oId CUFF.75297.1; tss_id TSS79763;
7	Cufflinks	exon	174833242	174833334	.	-	.	gene_id GRMZM2G134295; transcript_id TCONS_00038862; exon_number 3; oId CUFF.75297.1; tss_id TSS79763;
7	Cufflinks	exon	174833680	174833710	.	-	.	gene_id GRMZM2G134295; transcript_id TCONS_00038862; exon_number 4; oId CUFF.75297.1; tss_id TSS79763;
7	Cufflinks	exon	174833803	174833902	.	-	.	gene_id GRMZM2G134295; transcript_id TCONS_00038862; exon_number 5; oId CUFF.75297.1; tss_id TSS79763;
7	Cufflinks	exon	174833993	174834107	.	-	.	gene_id GRMZM2G134295; transcript_id TCONS_00038862; exon_number 6; oId CUFF.75297.1; tss_id TSS79763;
7	Cufflinks	exon	174834211	174834285	.	-	.	gene_id GRMZM2G134295; transcript_id TCONS_00038862; exon_number 7; oId CUFF.75297.1; tss_id TSS79763;
7	Cufflinks	exon	174834377	174835057	.	-	.	gene_id GRMZM2G134295; transcript_id TCONS_00038862; exon_number 8; oId CUFF.75297.1; tss_id TSS79763;
7	Cufflinks	exon	175605863	175606058	.	-	.	gene_id GRMZM2G032711; transcript_id TCONS_00038875; exon_number 1; oId CUFF.75338.1; tss_id TSS79789;
7	Cufflinks	exon	175606154	175606385	.	-	.	gene_id GRMZM2G032711; transcript_id TCONS_00038875; exon_number 2; oId CUFF.75338.1; tss_id TSS79789;
7	Cufflinks	exon	175606737	175606832	.	-	.	gene_id GRMZM2G032711; transcript_id TCONS_00038875; exon_number 3; oId CUFF.75338.1; tss_id TSS79789;
7	Cufflinks	exon	175608845	175608969	.	-	.	gene_id GRMZM2G032711; transcript_id TCONS_00038875; exon_number 4; oId CUFF.75338.1; tss_id TSS79789;
7	Cufflinks	exon	175609421	175609564	.	-	.	gene_id GRMZM2G032711; transcript_id TCONS_00038875; exon_number 5; oId CUFF.75338.1; tss_id TSS79789;
7	Cufflinks	exon	175611168	175613266	.	-	.	gene_id GRMZM2G032711; transcript_id TCONS_00038875; exon_number 6; oId CUFF.75338.1; tss_id TSS79789;
8	Cufflinks	exon	2903301	2903831	.	+	.	gene_id XLOC_034151; transcript_id TCONS_00038923; exon_number 1; oId CUFF.75475.1; tss_id TSS79889;
8	Cufflinks	exon	3921958	3922099	.	+	.	gene_id GRMZM2G050325; transcript_id TCONS_00038946; exon_number 1; oId CUFF.75559.2; tss_id TSS79917;
8	Cufflinks	exon	3922189	3922398	.	+	.	gene_id GRMZM2G050325; transcript_id TCONS_00038946; exon_number 2; oId CUFF.75559.2; tss_id TSS79917;
8	Cufflinks	exon	3922471	3922767	.	+	.	gene_id GRMZM2G050325; transcript_id TCONS_00038946; exon_number 3; oId CUFF.75559.2; tss_id TSS79917;
8	Cufflinks	exon	3923474	3923635	.	+	.	gene_id GRMZM2G050325; transcript_id TCONS_00038946; exon_number 4; oId CUFF.75559.2; tss_id TSS79917;
8	Cufflinks	exon	3924058	3924141	.	+	.	gene_id GRMZM2G050325; transcript_id TCONS_00038946; exon_number 5; oId CUFF.75559.2; tss_id TSS79917;
8	Cufflinks	exon	3924453	3925143	.	+	.	gene_id GRMZM2G050325; transcript_id TCONS_00038946; exon_number 6; oId CUFF.75559.2; tss_id TSS79917;
8	Cufflinks	exon	15108641	15109157	.	+	.	gene_id XLOC_034340; transcript_id TCONS_00039149; exon_number 1; oId CUFF.76250.1; tss_id TSS80334;
8	Cufflinks	exon	17177806	17178198	.	+	.	gene_id GRMZM2G095595; transcript_id TCONS_00039173; exon_number 1; oId CUFF.76332.2; tss_id TSS80402;
8	Cufflinks	exon	17179525	17179608	.	+	.	gene_id GRMZM2G095595; transcript_id TCONS_00039173; exon_number 2; oId CUFF.76332.2; tss_id TSS80402;
8	Cufflinks	exon	17180143	17180360	.	+	.	gene_id GRMZM2G095595; transcript_id TCONS_00039173; exon_number 3; oId CUFF.76332.2; tss_id TSS80402;

8	Cufflinks	exon	17180531	17180929	.	+	.	gene_id GRMZM2G095595; transcript_id TCONS_00039173; exon_number 4; oId CUFF.76332.2; tss_id TSS80402;
8	Cufflinks	exon	18444771	18445411	.	+	.	gene_id XLOC_034371; transcript_id TCONS_00039182; exon_number 1; oId CUFF.76381.1; tss_id TSS80423;
8	Cufflinks	exon	18612350	18613070	.	+	.	gene_id GRMZM2G032190; transcript_id TCONS_00039186; exon_number 1; oId CUFF.76399.1; tss_id TSS80428;
8	Cufflinks	exon	18614914	18615154	.	+	.	gene_id GRMZM2G032190; transcript_id TCONS_00039186; exon_number 2; oId CUFF.76399.1; tss_id TSS80428;
8	Cufflinks	exon	18615260	18616112	.	+	.	gene_id GRMZM2G032190; transcript_id TCONS_00039186; exon_number 3; oId CUFF.76399.1; tss_id TSS80428;
8	Cufflinks	exon	22175678	22175848	.	+	.	gene_id GRMZM2G004182; transcript_id TCONS_00039231; exon_number 1; oId CUFF.76524.1; tss_id TSS80503;
8	Cufflinks	exon	22178866	22178918	.	+	.	gene_id GRMZM2G004182; transcript_id TCONS_00039231; exon_number 2; oId CUFF.76524.1; tss_id TSS80503;
8	Cufflinks	exon	22179016	22179109	.	+	.	gene_id GRMZM2G004182; transcript_id TCONS_00039231; exon_number 3; oId CUFF.76524.1; tss_id TSS80503;
8	Cufflinks	exon	22179234	22179332	.	+	.	gene_id GRMZM2G004182; transcript_id TCONS_00039231; exon_number 4; oId CUFF.76524.1; tss_id TSS80503;
8	Cufflinks	exon	22179419	22180874	.	+	.	gene_id GRMZM2G004182; transcript_id TCONS_00039231; exon_number 5; oId CUFF.76524.1; tss_id TSS80503;
8	Cufflinks	exon	24724777	24725184	.	+	.	gene_id GRMZM2G047139; transcript_id TCONS_00039259; exon_number 1; oId CUFF.76654.2; tss_id TSS80566;
8	Cufflinks	exon	24725316	24728219	.	+	.	gene_id GRMZM2G047139; transcript_id TCONS_00039259; exon_number 2; oId CUFF.76654.2; tss_id TSS80566;
8	Cufflinks	exon	24724776	24725184	.	+	.	gene_id GRMZM2G047139; transcript_id TCONS_00039260; exon_number 1; oId CUFF.76654.1; tss_id TSS80566;
8	Cufflinks	exon	24725316	24725957	.	+	.	gene_id GRMZM2G047139; transcript_id TCONS_00039260; exon_number 2; oId CUFF.76654.1; tss_id TSS80566;
8	Cufflinks	exon	24726374	24728219	.	+	.	gene_id GRMZM2G047139; transcript_id TCONS_00039260; exon_number 3; oId CUFF.76654.1; tss_id TSS80566;
8	Cufflinks	exon	25881226	25882741	.	+	.	gene_id GRMZM2G369182; transcript_id TCONS_00039266; exon_number 1; oId CUFF.76668.1; tss_id TSS80583;
8	Cufflinks	exon	27218363	27218894	.	+	.	gene_id XLOC_034461; transcript_id TCONS_00039280; exon_number 1; oId CUFF.76738.1; tss_id TSS80610;
8	Cufflinks	exon	69107919	69110163	.	+	.	gene_id GRMZM2G001514; transcript_id TCONS_00039513; exon_number 1; oId CUFF.77839.1; tss_id TSS81205;
8	Cufflinks	exon	72494681	72496570	.	+	.	gene_id GRMZM2G023872; transcript_id TCONS_00039546; exon_number 1; oId CUFF.77960.1; tss_id TSS81269;
8	Cufflinks	exon	73423869	73424293	.	+	.	gene_id XLOC_034703; transcript_id TCONS_00039554; exon_number 1; oId CUFF.78001.1; tss_id TSS81288;
8	Cufflinks	exon	75116592	75117310	.	+	.	gene_id GRMZM2G058456; transcript_id TCONS_00039571; exon_number 1; oId CUFF.78068.1; tss_id TSS81315;
8	Cufflinks	exon	75117451	75117533	.	+	.	gene_id GRMZM2G058456; transcript_id TCONS_00039571; exon_number 2; oId CUFF.78068.1; tss_id TSS81315;
8	Cufflinks	exon	75118180	75118330	.	+	.	gene_id GRMZM2G058456; transcript_id TCONS_00039571; exon_number 3; oId CUFF.78068.1; tss_id TSS81315;
8	Cufflinks	exon	75118418	75118517	.	+	.	gene_id GRMZM2G058456; transcript_id TCONS_00039571; exon_number 4; oId CUFF.78068.1; tss_id TSS81315;
8	Cufflinks	exon	75118612	75118695	.	+	.	gene_id GRMZM2G058456; transcript_id TCONS_00039571; exon_number 5; oId CUFF.78068.1; tss_id TSS81315;
8	Cufflinks	exon	75118806	75118875	.	+	.	gene_id GRMZM2G058456; transcript_id TCONS_00039571; exon_number 6; oId CUFF.78068.1; tss_id TSS81315;
8	Cufflinks	exon	75118994	75119028	.	+	.	gene_id GRMZM2G058456; transcript_id TCONS_00039571; exon_number 7; oId CUFF.78068.1; tss_id TSS81315;
8	Cufflinks	exon	75119145	75119233	.	+	.	gene_id GRMZM2G058456; transcript_id TCONS_00039571; exon_number 8; oId CUFF.78068.1; tss_id TSS81315;
8	Cufflinks	exon	75119359	75119705	.	+	.	gene_id GRMZM2G058456; transcript_id TCONS_00039571; exon_number 9; oId CUFF.78068.1; tss_id TSS81315;
8	Cufflinks	exon	78161466	78161726	.	+	.	gene_id GRMZM5G892645; transcript_id TCONS_00039606; exon_number 1; oId CUFF.78194.2; tss_id TSS81375;
8	Cufflinks	exon	78161825	78161879	.	+	.	gene_id GRMZM5G892645; transcript_id TCONS_00039606; exon_number 2; oId CUFF.78194.2; tss_id TSS81375;
8	Cufflinks	exon	78162003	78162119	.	+	.	gene_id GRMZM5G892645; transcript_id TCONS_00039606; exon_number 3; oId CUFF.78194.2; tss_id TSS81375;
8	Cufflinks	exon	78162240	78162340	.	+	.	gene_id GRMZM5G892645; transcript_id TCONS_00039606; exon_number 4; oId CUFF.78194.2; tss_id TSS81375;
8	Cufflinks	exon	78163391	78163563	.	+	.	gene_id GRMZM5G892645; transcript_id TCONS_00039606; exon_number 5; oId CUFF.78194.2; tss_id TSS81375;
8	Cufflinks	exon	78163649	78163880	.	+	.	gene_id GRMZM5G892645; transcript_id TCONS_00039606; exon_number 6; oId CUFF.78194.2; tss_id TSS81375;
8	Cufflinks	exon	78163966	78164084	.	+	.	gene_id GRMZM5G892645; transcript_id TCONS_00039606; exon_number 7; oId CUFF.78194.2; tss_id TSS81375;

8	Cufflinks	exon	78164726	78164782	.	+	.	gene_id GRMZM5G892645; transcript_id TCONS_00039606; exon_number 8; oId CUFF.78194.2; tss_id TSS81375;
8	Cufflinks	exon	78164867	78165051	.	+	.	gene_id GRMZM5G892645; transcript_id TCONS_00039606; exon_number 9; oId CUFF.78194.2; tss_id TSS81375;
8	Cufflinks	exon	78165528	78165672	.	+	.	gene_id GRMZM5G892645; transcript_id TCONS_00039606; exon_number 10; oId CUFF.78194.2; tss_id TSS81375;
8	Cufflinks	exon	78165765	78165848	.	+	.	gene_id GRMZM5G892645; transcript_id TCONS_00039606; exon_number 11; oId CUFF.78194.2; tss_id TSS81375;
8	Cufflinks	exon	78165916	78165979	.	+	.	gene_id GRMZM5G892645; transcript_id TCONS_00039606; exon_number 12; oId CUFF.78194.2; tss_id TSS81375;
8	Cufflinks	exon	78166076	78166379	.	+	.	gene_id GRMZM5G892645; transcript_id TCONS_00039606; exon_number 13; oId CUFF.78194.2; tss_id TSS81375;
8	Cufflinks	exon	86105887	86107005	.	+	.	gene_id GRMZM2G128682; transcript_id TCONS_00039657; exon_number 1; oId CUFF.78354.1; tss_id TSS81488;
8	Cufflinks	exon	86107083	86107965	.	+	.	gene_id GRMZM2G128682; transcript_id TCONS_00039657; exon_number 2; oId CUFF.78354.1; tss_id TSS81488;
8	Cufflinks	exon	96331102	96331558	.	+	.	gene_id XLOC_034863; transcript_id TCONS_00039741; exon_number 1; oId CUFF.78715.1; tss_id TSS81664;
8	Cufflinks	exon	97452226	97452612	.	+	.	gene_id XLOC_034876; transcript_id TCONS_00039755; exon_number 1; oId CUFF.78752.1; tss_id TSS81682;
8	Cufflinks	exon	118087730	118088559	.	+	.	gene_id XLOC_035059; transcript_id TCONS_00039954; exon_number 1; oId CUFF.79574.1; tss_id TSS82132;
8	Cufflinks	exon	118088643	118088907	.	+	.	gene_id XLOC_035059; transcript_id TCONS_00039954; exon_number 2; oId CUFF.79574.1; tss_id TSS82132;
8	Cufflinks	exon	118087730	118088537	.	+	.	gene_id XLOC_035059; transcript_id TCONS_00039955; exon_number 1; oId CUFF.79574.3; tss_id TSS82132;
8	Cufflinks	exon	118088643	118088907	.	+	.	gene_id XLOC_035059; transcript_id TCONS_00039955; exon_number 2; oId CUFF.79574.3; tss_id TSS82132;
8	Cufflinks	exon	118795173	118797211	.	+	.	gene_id XLOC_035077; transcript_id TCONS_00039974; exon_number 1; oId CUFF.79609.1; tss_id TSS82160;
8	Cufflinks	exon	119745323	119746151	.	+	.	gene_id XLOC_035084; transcript_id TCONS_00039982; exon_number 1; oId CUFF.79670.1; tss_id TSS82184;
8	Cufflinks	exon	119747479	119747929	.	+	.	gene_id XLOC_035084; transcript_id TCONS_00039982; exon_number 2; oId CUFF.79670.1; tss_id TSS82184;
8	Cufflinks	exon	126229305	126229540	.	+	.	gene_id GRMZM2G150367; transcript_id TCONS_00040065; exon_number 1; oId CUFF.79967.1; tss_id TSS82331;
8	Cufflinks	exon	126229619	126229876	.	+	.	gene_id GRMZM2G150367; transcript_id TCONS_00040065; exon_number 2; oId CUFF.79967.1; tss_id TSS82331;
8	Cufflinks	exon	126230683	126232019	.	+	.	gene_id GRMZM2G150367; transcript_id TCONS_00040065; exon_number 3; oId CUFF.79967.1; tss_id TSS82331;
8	Cufflinks	exon	131336046	131336543	.	+	.	gene_id XLOC_035215; transcript_id TCONS_00040131; exon_number 1; oId CUFF.80174.1; tss_id TSS82455;
8	Cufflinks	exon	131904461	131905210	.	+	.	gene_id GRMZM2G053991; transcript_id TCONS_00040140; exon_number 1; oId CUFF.80197.1; tss_id TSS82471;
8	Cufflinks	exon	131905289	131905991	.	+	.	gene_id GRMZM2G053991; transcript_id TCONS_00040140; exon_number 2; oId CUFF.80197.1; tss_id TSS82471;
8	Cufflinks	exon	144697840	144698053	.	+	.	gene_id GRMZM2G074735; transcript_id TCONS_00040326; exon_number 1; oId CUFF.80822.1; tss_id TSS82820;
8	Cufflinks	exon	144698315	144698410	.	+	.	gene_id GRMZM2G074735; transcript_id TCONS_00040326; exon_number 2; oId CUFF.80822.1; tss_id TSS82820;
8	Cufflinks	exon	144699058	144699628	.	+	.	gene_id GRMZM2G074735; transcript_id TCONS_00040326; exon_number 3; oId CUFF.80822.1; tss_id TSS82820;
8	Cufflinks	exon	154562135	154562609	.	+	.	gene_id XLOC_035530; transcript_id TCONS_00040520; exon_number 1; oId CUFF.81405.1; tss_id TSS83117;
8	Cufflinks	exon	155182657	155183352	.	+	.	gene_id GRMZM2G151245; transcript_id TCONS_00040533; exon_number 1; oId CUFF.81415.1; tss_id TSS83132;
8	Cufflinks	exon	155183482	155183606	.	+	.	gene_id GRMZM2G151245; transcript_id TCONS_00040533; exon_number 2; oId CUFF.81415.1; tss_id TSS83132;
8	Cufflinks	exon	155183703	155183869	.	+	.	gene_id GRMZM2G151245; transcript_id TCONS_00040533; exon_number 3; oId CUFF.81415.1; tss_id TSS83132;
8	Cufflinks	exon	155183988	155184072	.	+	.	gene_id GRMZM2G151245; transcript_id TCONS_00040533; exon_number 4; oId CUFF.81415.1; tss_id TSS83132;
8	Cufflinks	exon	155184144	155184839	.	+	.	gene_id GRMZM2G151245; transcript_id TCONS_00040533; exon_number 5; oId CUFF.81415.1; tss_id TSS83132;
8	Cufflinks	exon	156268512	156269644	.	+	.	gene_id XLOC_035566; transcript_id TCONS_00040559; exon_number 1; oId CUFF.81513.1; tss_id TSS83196;
8	Cufflinks	exon	156423920	156424168	.	+	.	gene_id GRMZM2G167758; transcript_id TCONS_00040566; exon_number 1; oId CUFF.81547.1; tss_id TSS83213;
8	Cufflinks	exon	156424768	156424851	.	+	.	gene_id GRMZM2G167758; transcript_id TCONS_00040566; exon_number 2; oId CUFF.81547.1; tss_id TSS83213;
8	Cufflinks	exon	156424962	156425001	.	+	.	gene_id GRMZM2G167758; transcript_id TCONS_00040566; exon_number 3; oId CUFF.81547.1; tss_id TSS83213;

8	Cufflinks	exon	156425112	156425180	.	+	.	gene_id GRMZM2G167758; transcript_id TCONS_00040566; exon_number 4; oId CUFF.81547.1; tss_id TSS83213;
8	Cufflinks	exon	156425257	156425366	.	+	.	gene_id GRMZM2G167758; transcript_id TCONS_00040566; exon_number 5; oId CUFF.81547.1; tss_id TSS83213;
8	Cufflinks	exon	156425474	156425734	.	+	.	gene_id GRMZM2G167758; transcript_id TCONS_00040566; exon_number 6; oId CUFF.81547.1; tss_id TSS83213;
8	Cufflinks	exon	166202461	166203023	.	+	.	gene_id XLOC_035766; transcript_id TCONS_00040791; exon_number 1; oId CUFF.82309.1; tss_id TSS83640;
8	Cufflinks	exon	168814382	168814995	.	+	.	gene_id XLOC_035823; transcript_id TCONS_00040851; exon_number 1; oId CUFF.82555.1; tss_id TSS83750;
8	Cufflinks	exon	169675588	169676148	.	+	.	gene_id GRMZM2G036448; transcript_id TCONS_00040872; exon_number 1; oId CUFF.82627.1; tss_id TSS83784;
8	Cufflinks	exon	169676229	169676381	.	+	.	gene_id GRMZM2G036448; transcript_id TCONS_00040872; exon_number 2; oId CUFF.82627.1; tss_id TSS83784;
8	Cufflinks	exon	169679278	169679481	.	+	.	gene_id GRMZM2G036448; transcript_id TCONS_00040872; exon_number 3; oId CUFF.82627.1; tss_id TSS83784;
8	Cufflinks	exon	169679586	169680051	.	+	.	gene_id GRMZM2G036448; transcript_id TCONS_00040872; exon_number 4; oId CUFF.82627.1; tss_id TSS83784;
8	Cufflinks	exon	169680153	169681153	.	+	.	gene_id GRMZM2G036448; transcript_id TCONS_00040872; exon_number 5; oId CUFF.82627.1; tss_id TSS83784;
8	Cufflinks	exon	170198174	170198543	.	+	.	gene_id XLOC_035858; transcript_id TCONS_00040889; exon_number 1; oId CUFF.82661.1; tss_id TSS83809;
8	Cufflinks	exon	170218672	170218956	.	+	.	gene_id XLOC_035859; transcript_id TCONS_00040890; exon_number 1; oId CUFF.82671.1; tss_id TSS83810;
8	Cufflinks	exon	170219553	170219673	.	+	.	gene_id XLOC_035859; transcript_id TCONS_00040890; exon_number 2; oId CUFF.82671.1; tss_id TSS83810;
8	Cufflinks	exon	170221113	170221170	.	+	.	gene_id XLOC_035859; transcript_id TCONS_00040890; exon_number 3; oId CUFF.82671.1; tss_id TSS83810;
8	Cufflinks	exon	170885667	170886009	.	+	.	gene_id GRMZM2G476009; transcript_id TCONS_00040907; exon_number 1; oId CUFF.82724.1; tss_id TSS83840;
8	Cufflinks	exon	170886680	170886859	.	+	.	gene_id GRMZM2G476009; transcript_id TCONS_00040907; exon_number 2; oId CUFF.82724.1; tss_id TSS83840;
8	Cufflinks	exon	170886997	170887110	.	+	.	gene_id GRMZM2G476009; transcript_id TCONS_00040907; exon_number 3; oId CUFF.82724.1; tss_id TSS83840;
8	Cufflinks	exon	170888071	170888437	.	+	.	gene_id GRMZM2G476009; transcript_id TCONS_00040907; exon_number 4; oId CUFF.82724.1; tss_id TSS83840;
8	Cufflinks	exon	173488595	173488860	.	+	.	gene_id XLOC_035950; transcript_id TCONS_00040996; exon_number 1; oId CUFF.83002.1; tss_id TSS83987;
8	Cufflinks	exon	2511400	2512210	.	-	.	gene_id XLOC_036018; transcript_id TCONS_00041071; exon_number 1; oId CUFF.75518.1; tss_id TSS84143;
8	Cufflinks	exon	13801218	13802898	.	-	.	gene_id GRMZM2G079458; transcript_id TCONS_00041207; exon_number 1; oId CUFF.76187.1; tss_id TSS84444;
8	Cufflinks	exon	13803205	13803704	.	-	.	gene_id GRMZM2G079458; transcript_id TCONS_00041207; exon_number 2; oId CUFF.76187.1; tss_id TSS84444;
8	Cufflinks	exon	13804195	13804482	.	-	.	gene_id GRMZM2G079458; transcript_id TCONS_00041207; exon_number 3; oId CUFF.76187.1; tss_id TSS84444;
8	Cufflinks	exon	13801218	13802898	.	-	.	gene_id GRMZM2G079458; transcript_id TCONS_00041208; exon_number 1; oId CUFF.76187.3; tss_id TSS84444;
8	Cufflinks	exon	13803205	13803687	.	-	.	gene_id GRMZM2G079458; transcript_id TCONS_00041208; exon_number 2; oId CUFF.76187.3; tss_id TSS84444;
8	Cufflinks	exon	13804195	13804482	.	-	.	gene_id GRMZM2G079458; transcript_id TCONS_00041208; exon_number 3; oId CUFF.76187.3; tss_id TSS84444;
8	Cufflinks	exon	14797419	14799093	.	-	.	gene_id XLOC_036161; transcript_id TCONS_00041227; exon_number 1; oId CUFF.76210.1; tss_id TSS84465;
8	Cufflinks	exon	15987340	15988139	.	-	.	gene_id GRMZM2G083328; transcript_id TCONS_00041244; exon_number 1; oId CUFF.76264.2; tss_id TSS84497;
8	Cufflinks	exon	15988349	15989474	.	-	.	gene_id GRMZM2G083328; transcript_id TCONS_00041244; exon_number 2; oId CUFF.76264.2; tss_id TSS84497;
8	Cufflinks	exon	16431439	16431659	.	-	.	gene_id GRMZM2G081848; transcript_id TCONS_00041252; exon_number 1; oId CUFF.76287.1; tss_id TSS84504;
8	Cufflinks	exon	16433110	16433340	.	-	.	gene_id GRMZM2G081848; transcript_id TCONS_00041252; exon_number 2; oId CUFF.76287.1; tss_id TSS84504;
8	Cufflinks	exon	16433441	16433808	.	-	.	gene_id GRMZM2G081848; transcript_id TCONS_00041252; exon_number 3; oId CUFF.76287.1; tss_id TSS84504;
8	Cufflinks	exon	18612463	18613061	.	-	.	gene_id XLOC_036220; transcript_id TCONS_00041299; exon_number 1; oId CUFF.76400.1; tss_id TSS84559;
8	Cufflinks	exon	23427704	23428763	.	-	.	gene_id GRMZM2G472625; transcript_id TCONS_00041360; exon_number 1; oId CUFF.76581.1; tss_id TSS84643;
8	Cufflinks	exon	23428905	23429006	.	-	.	gene_id GRMZM2G472625; transcript_id TCONS_00041360; exon_number 2; oId CUFF.76581.1; tss_id TSS84643;
8	Cufflinks	exon	23429521	23429604	.	-	.	gene_id GRMZM2G472625; transcript_id TCONS_00041360; exon_number 3; oId CUFF.76581.1; tss_id TSS84643;

8	Cufflinks	exon	23429808	23429903	.	-	.	gene_id GRMZM2G472625; transcript_id TCONS_00041360; exon_number 4; oId CUFF.76581.1; tss_id TSS84643;
8	Cufflinks	exon	23429981	23430031	.	-	.	gene_id GRMZM2G472625; transcript_id TCONS_00041360; exon_number 5; oId CUFF.76581.1; tss_id TSS84643;
8	Cufflinks	exon	23430140	23430280	.	-	.	gene_id GRMZM2G472625; transcript_id TCONS_00041360; exon_number 6; oId CUFF.76581.1; tss_id TSS84643;
8	Cufflinks	exon	23430937	23430993	.	-	.	gene_id GRMZM2G472625; transcript_id TCONS_00041360; exon_number 7; oId CUFF.76581.1; tss_id TSS84643;
8	Cufflinks	exon	23431078	23431152	.	-	.	gene_id GRMZM2G472625; transcript_id TCONS_00041360; exon_number 8; oId CUFF.76581.1; tss_id TSS84643;
8	Cufflinks	exon	23431243	23431515	.	-	.	gene_id GRMZM2G472625; transcript_id TCONS_00041360; exon_number 9; oId CUFF.76581.1; tss_id TSS84643;
8	Cufflinks	exon	23431602	23431661	.	-	.	gene_id GRMZM2G472625; transcript_id TCONS_00041360; exon_number 10; oId CUFF.76581.1; tss_id TSS84643;
8	Cufflinks	exon	23432241	23432324	.	-	.	gene_id GRMZM2G472625; transcript_id TCONS_00041360; exon_number 11; oId CUFF.76581.1; tss_id TSS84643;
8	Cufflinks	exon	23432475	23432837	.	-	.	gene_id GRMZM2G472625; transcript_id TCONS_00041360; exon_number 12; oId CUFF.76581.1; tss_id TSS84643;
8	Cufflinks	exon	36145034	36145351	.	-	.	gene_id GRMZM5G842965; transcript_id TCONS_00041463; exon_number 1; oId CUFF.76955.1; tss_id TSS84860;
8	Cufflinks	exon	36145477	36145545	.	-	.	gene_id GRMZM5G842965; transcript_id TCONS_00041463; exon_number 2; oId CUFF.76955.1; tss_id TSS84860;
8	Cufflinks	exon	36145662	36145766	.	-	.	gene_id GRMZM5G842965; transcript_id TCONS_00041463; exon_number 3; oId CUFF.76955.1; tss_id TSS84860;
8	Cufflinks	exon	36146764	36146867	.	-	.	gene_id GRMZM5G842965; transcript_id TCONS_00041463; exon_number 4; oId CUFF.76955.1; tss_id TSS84860;
8	Cufflinks	exon	36146989	36147483	.	-	.	gene_id GRMZM5G842965; transcript_id TCONS_00041463; exon_number 5; oId CUFF.76955.1; tss_id TSS84860;
8	Cufflinks	exon	36145034	36145351	.	-	.	gene_id GRMZM5G842965; transcript_id TCONS_00041464; exon_number 1; oId CUFF.76955.3; tss_id TSS84860;
8	Cufflinks	exon	36145477	36145545	.	-	.	gene_id GRMZM5G842965; transcript_id TCONS_00041464; exon_number 2; oId CUFF.76955.3; tss_id TSS84860;
8	Cufflinks	exon	36145662	36145766	.	-	.	gene_id GRMZM5G842965; transcript_id TCONS_00041464; exon_number 3; oId CUFF.76955.3; tss_id TSS84860;
8	Cufflinks	exon	36146764	36146867	.	-	.	gene_id GRMZM5G842965; transcript_id TCONS_00041464; exon_number 4; oId CUFF.76955.3; tss_id TSS84860;
8	Cufflinks	exon	36146993	36147538	.	-	.	gene_id GRMZM5G842965; transcript_id TCONS_00041464; exon_number 5; oId CUFF.76955.3; tss_id TSS84860;
8	Cufflinks	exon	43077873	43078436	.	-	.	gene_id GRMZM2G035542; transcript_id TCONS_00041510; exon_number 1; oId CUFF.77163.1; tss_id TSS84952;
8	Cufflinks	exon	43080762	43080821	.	-	.	gene_id GRMZM2G035542; transcript_id TCONS_00041510; exon_number 2; oId CUFF.77163.1; tss_id TSS84952;
8	Cufflinks	exon	43080900	43081310	.	-	.	gene_id GRMZM2G035542; transcript_id TCONS_00041510; exon_number 3; oId CUFF.77163.1; tss_id TSS84952;
8	Cufflinks	exon	43081380	43081710	.	-	.	gene_id GRMZM2G035542; transcript_id TCONS_00041510; exon_number 4; oId CUFF.77163.1; tss_id TSS84952;
8	Cufflinks	exon	43081795	43081881	.	-	.	gene_id GRMZM2G035542; transcript_id TCONS_00041510; exon_number 5; oId CUFF.77163.1; tss_id TSS84952;
8	Cufflinks	exon	43081962	43082916	.	-	.	gene_id GRMZM2G035542; transcript_id TCONS_00041510; exon_number 6; oId CUFF.77163.1; tss_id TSS84952;
8	Cufflinks	exon	43086101	43086649	.	-	.	gene_id GRMZM2G035542; transcript_id TCONS_00041510; exon_number 7; oId CUFF.77163.1; tss_id TSS84952;
8	Cufflinks	exon	52817772	52819178	.	-	.	gene_id XLOC_036426; transcript_id TCONS_00041540; exon_number 1; oId CUFF.77299.1; tss_id TSS85026;
8	Cufflinks	exon	69108973	69109970	.	-	.	gene_id XLOC_036518; transcript_id TCONS_00041642; exon_number 1; oId CUFF.77840.1; tss_id TSS85309;
8	Cufflinks	exon	69659485	69660454	.	-	.	gene_id XLOC_036522; transcript_id TCONS_00041646; exon_number 1; oId CUFF.77875.1; tss_id TSS85316;
8	Cufflinks	exon	73418363	73419074	.	-	.	gene_id GRMZM2G097768; transcript_id TCONS_00041678; exon_number 1; oId CUFF.78000.2; tss_id TSS85390;
8	Cufflinks	exon	73419168	73419282	.	-	.	gene_id GRMZM2G097768; transcript_id TCONS_00041678; exon_number 2; oId CUFF.78000.2; tss_id TSS85390;
8	Cufflinks	exon	73419844	73419903	.	-	.	gene_id GRMZM2G097768; transcript_id TCONS_00041678; exon_number 3; oId CUFF.78000.2; tss_id TSS85390;
8	Cufflinks	exon	73419985	73420050	.	-	.	gene_id GRMZM2G097768; transcript_id TCONS_00041678; exon_number 4; oId CUFF.78000.2; tss_id TSS85390;
8	Cufflinks	exon	73420184	73420243	.	-	.	gene_id GRMZM2G097768; transcript_id TCONS_00041678; exon_number 5; oId CUFF.78000.2; tss_id TSS85390;
8	Cufflinks	exon	73420320	73420382	.	-	.	gene_id GRMZM2G097768; transcript_id TCONS_00041678; exon_number 6; oId CUFF.78000.2; tss_id TSS85390;
8	Cufflinks	exon	73420483	73420529	.	-	.	gene_id GRMZM2G097768; transcript_id TCONS_00041678; exon_number 7; oId CUFF.78000.2; tss_id TSS85390;

8	Cufflinks	exon	73420770	73420854	.	-	.	gene_id GRMZM2G097768; transcript_id TCONS_00041678; exon_number 8; oId CUFF.78000.2; tss_id TSS85390;
8	Cufflinks	exon	73420935	73421015	.	-	.	gene_id GRMZM2G097768; transcript_id TCONS_00041678; exon_number 9; oId CUFF.78000.2; tss_id TSS85390;
8	Cufflinks	exon	73421482	73421574	.	-	.	gene_id GRMZM2G097768; transcript_id TCONS_00041678; exon_number 10; oId CUFF.78000.2; tss_id TSS85390;
8	Cufflinks	exon	73421648	73421712	.	-	.	gene_id GRMZM2G097768; transcript_id TCONS_00041678; exon_number 11; oId CUFF.78000.2; tss_id TSS85390;
8	Cufflinks	exon	73421855	73421930	.	-	.	gene_id GRMZM2G097768; transcript_id TCONS_00041678; exon_number 12; oId CUFF.78000.2; tss_id TSS85390;
8	Cufflinks	exon	73422045	73422110	.	-	.	gene_id GRMZM2G097768; transcript_id TCONS_00041678; exon_number 13; oId CUFF.78000.2; tss_id TSS85390;
8	Cufflinks	exon	73423402	73423467	.	-	.	gene_id GRMZM2G097768; transcript_id TCONS_00041678; exon_number 14; oId CUFF.78000.2; tss_id TSS85390;
8	Cufflinks	exon	73423582	73423641	.	-	.	gene_id GRMZM2G097768; transcript_id TCONS_00041678; exon_number 15; oId CUFF.78000.2; tss_id TSS85390;
8	Cufflinks	exon	73423841	73423930	.	-	.	gene_id GRMZM2G097768; transcript_id TCONS_00041678; exon_number 16; oId CUFF.78000.2; tss_id TSS85390;
8	Cufflinks	exon	73424019	73424464	.	-	.	gene_id GRMZM2G097768; transcript_id TCONS_00041678; exon_number 17; oId CUFF.78000.2; tss_id TSS85390;
8	Cufflinks	exon	73424527	73424793	.	-	.	gene_id GRMZM2G097768; transcript_id TCONS_00041678; exon_number 18; oId CUFF.78000.2; tss_id TSS85390;
8	Cufflinks	exon	78524069	78524547	.	-	.	gene_id GRMZM2G456000; transcript_id TCONS_00041719; exon_number 1; oId CUFF.78188.1; tss_id TSS85481;
8	Cufflinks	exon	78524635	78524868	.	-	.	gene_id GRMZM2G456000; transcript_id TCONS_00041719; exon_number 2; oId CUFF.78188.1; tss_id TSS85481;
8	Cufflinks	exon	78524947	78525271	.	-	.	gene_id GRMZM2G456000; transcript_id TCONS_00041719; exon_number 3; oId CUFF.78188.1; tss_id TSS85481;
8	Cufflinks	exon	78525720	78525965	.	-	.	gene_id GRMZM2G456000; transcript_id TCONS_00041719; exon_number 4; oId CUFF.78188.1; tss_id TSS85481;
8	Cufflinks	exon	78526091	78526431	.	-	.	gene_id GRMZM2G456000; transcript_id TCONS_00041719; exon_number 5; oId CUFF.78188.1; tss_id TSS85481;
8	Cufflinks	exon	78526605	78526685	.	-	.	gene_id GRMZM2G456000; transcript_id TCONS_00041719; exon_number 6; oId CUFF.78188.1; tss_id TSS85481;
8	Cufflinks	exon	78526818	78526937	.	-	.	gene_id GRMZM2G456000; transcript_id TCONS_00041719; exon_number 7; oId CUFF.78188.1; tss_id TSS85481;
8	Cufflinks	exon	78527254	78527355	.	-	.	gene_id GRMZM2G456000; transcript_id TCONS_00041719; exon_number 8; oId CUFF.78188.1; tss_id TSS85481;
8	Cufflinks	exon	78527773	78527908	.	-	.	gene_id GRMZM2G456000; transcript_id TCONS_00041719; exon_number 9; oId CUFF.78188.1; tss_id TSS85481;
8	Cufflinks	exon	78528037	78528183	.	-	.	gene_id GRMZM2G456000; transcript_id TCONS_00041719; exon_number 10; oId CUFF.78188.1; tss_id TSS85481;
8	Cufflinks	exon	78528324	78528819	.	-	.	gene_id GRMZM2G456000; transcript_id TCONS_00041719; exon_number 11; oId CUFF.78188.1; tss_id TSS85481;
8	Cufflinks	exon	96330315	96332969	.	-	.	gene_id GRMZM2G094017; transcript_id TCONS_00041839; exon_number 1; oId CUFF.78714.1; tss_id TSS85789;
8	Cufflinks	exon	119542597	119543133	.	-	.	gene_id GRMZM2G062555; transcript_id TCONS_00042072; exon_number 1; oId CUFF.79665.2; tss_id TSS86263;
8	Cufflinks	exon	119543474	119544810	.	-	.	gene_id GRMZM2G062555; transcript_id TCONS_00042072; exon_number 2; oId CUFF.79665.2; tss_id TSS86263;
8	Cufflinks	exon	119745262	119745600	.	-	.	gene_id GRMZM2G025459; transcript_id TCONS_00042078; exon_number 1; oId CUFF.79669.2; tss_id TSS86267;
8	Cufflinks	exon	119745697	119745859	.	-	.	gene_id GRMZM2G025459; transcript_id TCONS_00042078; exon_number 2; oId CUFF.79669.2; tss_id TSS86267;
8	Cufflinks	exon	119745976	119746151	.	-	.	gene_id GRMZM2G025459; transcript_id TCONS_00042078; exon_number 3; oId CUFF.79669.2; tss_id TSS86267;
8	Cufflinks	exon	119747479	119748192	.	-	.	gene_id GRMZM2G025459; transcript_id TCONS_00042078; exon_number 4; oId CUFF.79669.2; tss_id TSS86267;
8	Cufflinks	exon	119745262	119745600	.	-	.	gene_id GRMZM2G025459; transcript_id TCONS_00042079; exon_number 1; oId CUFF.79669.1; tss_id TSS86267;
8	Cufflinks	exon	119745697	119745859	.	-	.	gene_id GRMZM2G025459; transcript_id TCONS_00042079; exon_number 2; oId CUFF.79669.1; tss_id TSS86267;
8	Cufflinks	exon	119745976	119746151	.	-	.	gene_id GRMZM2G025459; transcript_id TCONS_00042079; exon_number 3; oId CUFF.79669.1; tss_id TSS86267;
8	Cufflinks	exon	119747876	119748192	.	-	.	gene_id GRMZM2G025459; transcript_id TCONS_00042079; exon_number 4; oId CUFF.79669.1; tss_id TSS86267;
8	Cufflinks	exon	132973314	132973657	.	-	.	gene_id XLOC_037056; transcript_id TCONS_00042242; exon_number 1; oId CUFF.80247.1; tss_id TSS86584;
8	Cufflinks	exon	133091531	133091829	.	-	.	gene_id XLOC_037059; transcript_id TCONS_00042245; exon_number 1; oId CUFF.80257.1; tss_id TSS86587;
8	Cufflinks	exon	134444295	134444960	.	-	.	gene_id XLOC_037080; transcript_id TCONS_00042268; exon_number 1; oId CUFF.80324.1; tss_id TSS86627;

8	Cufflinks	exon	136652554	136652953	.	-	.	gene_id XLOC_037103; transcript_id TCONS_00042313; exon_number 1; oId CUFF.80443.1; tss_id TSS86681;
8	Cufflinks	exon	142813533	142814761	.	-	.	gene_id GRMZM2G050270; transcript_id TCONS_00042376; exon_number 1; oId CUFF.80722.1; tss_id TSS86805;
8	Cufflinks	exon	142817035	142817608	.	-	.	gene_id GRMZM2G050270; transcript_id TCONS_00042376; exon_number 2; oId CUFF.80722.1; tss_id TSS86805;
8	Cufflinks	exon	145219676	145219874	.	-	.	gene_id XLOC_037185; transcript_id TCONS_00042403; exon_number 1; oId CUFF.80860.1; tss_id TSS86873;
8	Cufflinks	exon	145220026	145220486	.	-	.	gene_id XLOC_037185; transcript_id TCONS_00042403; exon_number 2; oId CUFF.80860.1; tss_id TSS86873;
8	Cufflinks	exon	151441785	151442939	.	-	.	gene_id GRMZM2G144028; transcript_id TCONS_00042502; exon_number 1; oId CUFF.81225.2; tss_id TSS87063;
8	Cufflinks	exon	151443068	151443518	.	-	.	gene_id GRMZM2G144028; transcript_id TCONS_00042502; exon_number 2; oId CUFF.81225.2; tss_id TSS87063;
8	Cufflinks	exon	151443661	151443948	.	-	.	gene_id GRMZM2G144028; transcript_id TCONS_00042502; exon_number 3; oId CUFF.81225.2; tss_id TSS87063;
8	Cufflinks	exon	151444374	151444403	.	-	.	gene_id GRMZM2G144028; transcript_id TCONS_00042502; exon_number 4; oId CUFF.81225.2; tss_id TSS87063;
8	Cufflinks	exon	151449120	151450139	.	-	.	gene_id GRMZM2G144028; transcript_id TCONS_00042502; exon_number 5; oId CUFF.81225.2; tss_id TSS87063;
8	Cufflinks	exon	151669118	151669267	.	-	.	gene_id GRMZM2G092018; transcript_id TCONS_00042510; exon_number 1; oId CUFF.81237.1; tss_id TSS87070;
8	Cufflinks	exon	151669292	151669525	.	-	.	gene_id GRMZM2G092018; transcript_id TCONS_00042510; exon_number 2; oId CUFF.81237.1; tss_id TSS87070;
8	Cufflinks	exon	151669897	151670071	.	-	.	gene_id GRMZM2G092018; transcript_id TCONS_00042510; exon_number 3; oId CUFF.81237.1; tss_id TSS87070;
8	Cufflinks	exon	151670165	151670275	.	-	.	gene_id GRMZM2G092018; transcript_id TCONS_00042510; exon_number 4; oId CUFF.81237.1; tss_id TSS87070;
8	Cufflinks	exon	151678026	151678132	.	-	.	gene_id GRMZM2G092018; transcript_id TCONS_00042510; exon_number 5; oId CUFF.81237.1; tss_id TSS87070;
8	Cufflinks	exon	151678494	151678603	.	-	.	gene_id GRMZM2G092018; transcript_id TCONS_00042510; exon_number 6; oId CUFF.81237.1; tss_id TSS87070;
8	Cufflinks	exon	151678935	151679031	.	-	.	gene_id GRMZM2G092018; transcript_id TCONS_00042510; exon_number 7; oId CUFF.81237.1; tss_id TSS87070;
8	Cufflinks	exon	151679171	151679324	.	-	.	gene_id GRMZM2G092018; transcript_id TCONS_00042510; exon_number 8; oId CUFF.81237.1; tss_id TSS87070;
8	Cufflinks	exon	151679530	151679583	.	-	.	gene_id GRMZM2G092018; transcript_id TCONS_00042510; exon_number 9; oId CUFF.81237.1; tss_id TSS87070;
8	Cufflinks	exon	151679739	151679799	.	-	.	gene_id GRMZM2G092018; transcript_id TCONS_00042510; exon_number 10; oId CUFF.81237.1; tss_id TSS87070;
8	Cufflinks	exon	151679996	151680065	.	-	.	gene_id GRMZM2G092018; transcript_id TCONS_00042510; exon_number 11; oId CUFF.81237.1; tss_id TSS87070;
8	Cufflinks	exon	151680207	151680291	.	-	.	gene_id GRMZM2G092018; transcript_id TCONS_00042510; exon_number 12; oId CUFF.81237.1; tss_id TSS87070;
8	Cufflinks	exon	151680390	151680488	.	-	.	gene_id GRMZM2G092018; transcript_id TCONS_00042510; exon_number 13; oId CUFF.81237.1; tss_id TSS87070;
8	Cufflinks	exon	151680584	151680781	.	-	.	gene_id GRMZM2G092018; transcript_id TCONS_00042510; exon_number 14; oId CUFF.81237.1; tss_id TSS87070;
8	Cufflinks	exon	154553618	154554612	.	-	.	gene_id GRMZM2G107985; transcript_id TCONS_00042553; exon_number 1; oId CUFF.81401.1; tss_id TSS87171;
8	Cufflinks	exon	154554753	154554938	.	-	.	gene_id GRMZM2G107985; transcript_id TCONS_00042553; exon_number 2; oId CUFF.81401.1; tss_id TSS87171;
8	Cufflinks	exon	154555760	154556075	.	-	.	gene_id GRMZM2G107985; transcript_id TCONS_00042553; exon_number 3; oId CUFF.81401.1; tss_id TSS87171;
8	Cufflinks	exon	154556169	154556283	.	-	.	gene_id GRMZM2G107985; transcript_id TCONS_00042553; exon_number 4; oId CUFF.81401.1; tss_id TSS87171;
8	Cufflinks	exon	154562201	154562574	.	-	.	gene_id GRMZM2G107985; transcript_id TCONS_00042553; exon_number 5; oId CUFF.81401.1; tss_id TSS87171;
8	Cufflinks	exon	155184005	155184905	.	-	.	gene_id GRMZM2G151252; transcript_id TCONS_00042562; exon_number 1; oId CUFF.81417.1; tss_id TSS87187;
8	Cufflinks	exon	155184995	155185081	.	-	.	gene_id GRMZM2G151252; transcript_id TCONS_00042562; exon_number 2; oId CUFF.81417.1; tss_id TSS87187;
8	Cufflinks	exon	155185835	155186005	.	-	.	gene_id GRMZM2G151252; transcript_id TCONS_00042562; exon_number 3; oId CUFF.81417.1; tss_id TSS87187;
8	Cufflinks	exon	155186100	155186141	.	-	.	gene_id GRMZM2G151252; transcript_id TCONS_00042562; exon_number 4; oId CUFF.81417.1; tss_id TSS87187;
8	Cufflinks	exon	155186248	155186489	.	-	.	gene_id GRMZM2G151252; transcript_id TCONS_00042562; exon_number 5; oId CUFF.81417.1; tss_id TSS87187;
8	Cufflinks	exon	163131948	163132447	.	-	.	gene_id XLOC_037489; transcript_id TCONS_00042747; exon_number 1; oId CUFF.82056.1; tss_id TSS87507;
8	Cufflinks	exon	166705309	166706025	.	-	.	gene_id XLOC_037576; transcript_id TCONS_00042854; exon_number 1; oId CUFF.82377.1; tss_id TSS87682;

8	Cufflinks	exon	169071358	169072345	.	-	.	gene_id XLOC_037635; transcript_id TCONS_00042917; exon_number 1; oId CUFF.82572.1; tss_id TSS87781;
8	Cufflinks	exon	169208422	169209009	.	-	.	gene_id GRMZM2G311220; transcript_id TCONS_00042930; exon_number 1; oId CUFF.82624.1; tss_id TSS87795;
8	Cufflinks	exon	169209114	169209347	.	-	.	gene_id GRMZM2G311220; transcript_id TCONS_00042930; exon_number 2; oId CUFF.82624.1; tss_id TSS87795;
8	Cufflinks	exon	169210760	169210927	.	-	.	gene_id GRMZM2G311220; transcript_id TCONS_00042930; exon_number 3; oId CUFF.82624.1; tss_id TSS87795;
8	Cufflinks	exon	169211043	169211158	.	-	.	gene_id GRMZM2G311220; transcript_id TCONS_00042930; exon_number 4; oId CUFF.82624.1; tss_id TSS87795;
8	Cufflinks	exon	169211618	169211770	.	-	.	gene_id GRMZM2G311220; transcript_id TCONS_00042930; exon_number 5; oId CUFF.82624.1; tss_id TSS87795;
8	Cufflinks	exon	169211848	169211991	.	-	.	gene_id GRMZM2G311220; transcript_id TCONS_00042930; exon_number 6; oId CUFF.82624.1; tss_id TSS87795;
8	Cufflinks	exon	169214533	169215320	.	-	.	gene_id GRMZM2G311220; transcript_id TCONS_00042930; exon_number 7; oId CUFF.82624.1; tss_id TSS87795;
8	Cufflinks	exon	170218080	170218956	.	-	.	gene_id XLOC_037665; transcript_id TCONS_00042954; exon_number 1; oId CUFF.82670.2; tss_id TSS87837;
8	Cufflinks	exon	170219553	170219673	.	-	.	gene_id XLOC_037665; transcript_id TCONS_00042954; exon_number 2; oId CUFF.82670.2; tss_id TSS87837;
8	Cufflinks	exon	170221113	170221215	.	-	.	gene_id XLOC_037665; transcript_id TCONS_00042954; exon_number 3; oId CUFF.82670.2; tss_id TSS87837;
8	Cufflinks	exon	170221379	170221722	.	-	.	gene_id XLOC_037665; transcript_id TCONS_00042954; exon_number 4; oId CUFF.82670.2; tss_id TSS87837;
8	Cufflinks	exon	170218080	170218956	.	-	.	gene_id XLOC_037665; transcript_id TCONS_00042955; exon_number 1; oId CUFF.82670.1; tss_id TSS87837;
8	Cufflinks	exon	170219553	170219673	.	-	.	gene_id XLOC_037665; transcript_id TCONS_00042955; exon_number 2; oId CUFF.82670.1; tss_id TSS87837;
8	Cufflinks	exon	170221113	170221215	.	-	.	gene_id XLOC_037665; transcript_id TCONS_00042955; exon_number 3; oId CUFF.82670.1; tss_id TSS87837;
8	Cufflinks	exon	170221326	170221722	.	-	.	gene_id XLOC_037665; transcript_id TCONS_00042955; exon_number 4; oId CUFF.82670.1; tss_id TSS87837;
8	Cufflinks	exon	170516288	170516967	.	-	.	gene_id XLOC_037673; transcript_id TCONS_00042963; exon_number 1; oId CUFF.82699.1; tss_id TSS87849;
8	Cufflinks	exon	171685242	171685998	.	-	.	gene_id GRMZM5G852779; transcript_id TCONS_00043005; exon_number 1; oId CUFF.82832.1; tss_id TSS87927;
8	Cufflinks	exon	171686211	171686333	.	-	.	gene_id GRMZM5G852779; transcript_id TCONS_00043005; exon_number 2; oId CUFF.82832.1; tss_id TSS87927;
8	Cufflinks	exon	171686864	171686950	.	-	.	gene_id GRMZM5G852779; transcript_id TCONS_00043005; exon_number 3; oId CUFF.82832.1; tss_id TSS87927;
8	Cufflinks	exon	171687038	171687089	.	-	.	gene_id GRMZM5G852779; transcript_id TCONS_00043005; exon_number 4; oId CUFF.82832.1; tss_id TSS87927;
8	Cufflinks	exon	171687225	171687353	.	-	.	gene_id GRMZM5G852779; transcript_id TCONS_00043005; exon_number 5; oId CUFF.82832.1; tss_id TSS87927;
8	Cufflinks	exon	171688126	171688330	.	-	.	gene_id GRMZM5G852779; transcript_id TCONS_00043005; exon_number 6; oId CUFF.82832.1; tss_id TSS87927;
8	Cufflinks	exon	171688424	171688996	.	-	.	gene_id GRMZM5G852779; transcript_id TCONS_00043005; exon_number 7; oId CUFF.82832.1; tss_id TSS87927;
8	Cufflinks	exon	172961557	172961959	.	-	.	gene_id GRMZM2G302639; transcript_id TCONS_00043043; exon_number 1; oId CUFF.82962.1; tss_id TSS88011;
8	Cufflinks	exon	172962260	172962297	.	-	.	gene_id GRMZM2G302639; transcript_id TCONS_00043043; exon_number 2; oId CUFF.82962.1; tss_id TSS88011;
8	Cufflinks	exon	172962429	172962491	.	-	.	gene_id GRMZM2G302639; transcript_id TCONS_00043043; exon_number 3; oId CUFF.82962.1; tss_id TSS88011;
8	Cufflinks	exon	172962590	172962676	.	-	.	gene_id GRMZM2G302639; transcript_id TCONS_00043043; exon_number 4; oId CUFF.82962.1; tss_id TSS88011;
8	Cufflinks	exon	172962981	172963078	.	-	.	gene_id GRMZM2G302639; transcript_id TCONS_00043043; exon_number 5; oId CUFF.82962.1; tss_id TSS88011;
8	Cufflinks	exon	172963121	172963511	.	-	.	gene_id GRMZM2G302639; transcript_id TCONS_00043043; exon_number 6; oId CUFF.82962.1; tss_id TSS88011;
9	Cufflinks	exon	6948280	6961033	.	+	.	gene_id GRMZM2G469593; transcript_id TCONS_00043171; exon_number 1; oId CUFF.83452.1; tss_id TSS88239;
9	Cufflinks	exon	8239976	8240287	.	+	.	gene_id XLOC_037868; transcript_id TCONS_00043194; exon_number 1; oId CUFF.83508.1; tss_id TSS88285;
9	Cufflinks	exon	10947383	10948025	.	+	.	gene_id XLOC_037888; transcript_id TCONS_00043215; exon_number 1; oId CUFF.83633.1; tss_id TSS88338;
9	Cufflinks	exon	12797426	12798671	.	+	.	gene_id XLOC_037912; transcript_id TCONS_00043244; exon_number 1; oId CUFF.83754.1; tss_id TSS88389;
9	Cufflinks	exon	14925130	14925915	.	+	.	gene_id XLOC_037946; transcript_id TCONS_00043281; exon_number 1; oId CUFF.83901.1; tss_id TSS88462;
9	Cufflinks	exon	15385595	15386863	.	+	.	gene_id XLOC_037949; transcript_id TCONS_00043284; exon_number 1; oId CUFF.83941.1; tss_id TSS88465;

9	Cufflinks	exon	18328876	18329310	.	+	.	gene_id GRMZM5G838414; transcript_id TCONS_00043347; exon_number 1; oId CUFF.84141.3; tss_id TSS88580;
9	Cufflinks	exon	18330601	18330753	.	+	.	gene_id GRMZM5G838414; transcript_id TCONS_00043347; exon_number 2; oId CUFF.84141.3; tss_id TSS88580;
9	Cufflinks	exon	18330826	18330908	.	+	.	gene_id GRMZM5G838414; transcript_id TCONS_00043347; exon_number 3; oId CUFF.84141.3; tss_id TSS88580;
9	Cufflinks	exon	18331011	18331110	.	+	.	gene_id GRMZM5G838414; transcript_id TCONS_00043347; exon_number 4; oId CUFF.84141.3; tss_id TSS88580;
9	Cufflinks	exon	18333598	18333711	.	+	.	gene_id GRMZM5G838414; transcript_id TCONS_00043347; exon_number 5; oId CUFF.84141.3; tss_id TSS88580;
9	Cufflinks	exon	18334445	18334587	.	+	.	gene_id GRMZM5G838414; transcript_id TCONS_00043347; exon_number 6; oId CUFF.84141.3; tss_id TSS88580;
9	Cufflinks	exon	18335404	18337267	.	+	.	gene_id GRMZM5G838414; transcript_id TCONS_00043347; exon_number 7; oId CUFF.84141.3; tss_id TSS88580;
9	Cufflinks	exon	19035099	19035555	.	+	.	gene_id XLOC_038017; transcript_id TCONS_00043363; exon_number 1; oId CUFF.84123.1; tss_id TSS88606;
9	Cufflinks	exon	32305933	32306735	.	+	.	gene_id XLOC_038172; transcript_id TCONS_00043540; exon_number 1; oId CUFF.84768.2; tss_id TSS88972;
9	Cufflinks	exon	32306866	32308115	.	+	.	gene_id XLOC_038172; transcript_id TCONS_00043540; exon_number 2; oId CUFF.84768.2; tss_id TSS88972;
9	Cufflinks	exon	48763025	48764394	.	+	.	gene_id GRMZM2G027860; transcript_id TCONS_00043640; exon_number 1; oId CUFF.85179.2; tss_id TSS89200;
9	Cufflinks	exon	48765748	48767093	.	+	.	gene_id GRMZM2G027860; transcript_id TCONS_00043640; exon_number 2; oId CUFF.85179.2; tss_id TSS89200;
9	Cufflinks	exon	48767174	48767290	.	+	.	gene_id GRMZM2G027860; transcript_id TCONS_00043640; exon_number 3; oId CUFF.85179.2; tss_id TSS89200;
9	Cufflinks	exon	48767365	48767641	.	+	.	gene_id GRMZM2G027860; transcript_id TCONS_00043640; exon_number 4; oId CUFF.85179.2; tss_id TSS89200;
9	Cufflinks	exon	48767840	48767928	.	+	.	gene_id GRMZM2G027860; transcript_id TCONS_00043640; exon_number 5; oId CUFF.85179.2; tss_id TSS89200;
9	Cufflinks	exon	48768019	48768127	.	+	.	gene_id GRMZM2G027860; transcript_id TCONS_00043640; exon_number 6; oId CUFF.85179.2; tss_id TSS89200;
9	Cufflinks	exon	48769136	48769348	.	+	.	gene_id GRMZM2G027860; transcript_id TCONS_00043640; exon_number 7; oId CUFF.85179.2; tss_id TSS89200;
9	Cufflinks	exon	48769450	48769575	.	+	.	gene_id GRMZM2G027860; transcript_id TCONS_00043640; exon_number 8; oId CUFF.85179.2; tss_id TSS89200;
9	Cufflinks	exon	48769658	48769756	.	+	.	gene_id GRMZM2G027860; transcript_id TCONS_00043640; exon_number 9; oId CUFF.85179.2; tss_id TSS89200;
9	Cufflinks	exon	48770338	48770529	.	+	.	gene_id GRMZM2G027860; transcript_id TCONS_00043640; exon_number 10; oId CUFF.85179.2; tss_id TSS89200;
9	Cufflinks	exon	48771098	48772161	.	+	.	gene_id GRMZM2G027860; transcript_id TCONS_00043640; exon_number 11; oId CUFF.85179.2; tss_id TSS89200;
9	Cufflinks	exon	73801048	73812636	.	+	.	gene_id XLOC_038379; transcript_id TCONS_00043772; exon_number 1; oId CUFF.85908.3; tss_id TSS89572;
9	Cufflinks	exon	73803691	73808103	.	+	.	gene_id XLOC_038380; transcript_id TCONS_00043773; exon_number 1; oId CUFF.85908.6; tss_id TSS89573;
9	Cufflinks	exon	73809795	73812636	.	+	.	gene_id XLOC_038380; transcript_id TCONS_00043773; exon_number 2; oId CUFF.85908.6; tss_id TSS89573;
9	Cufflinks	exon	79630453	79630867	.	+	.	gene_id XLOC_038410; transcript_id TCONS_00043810; exon_number 1; oId CUFF.85997.1; tss_id TSS89652;
9	Cufflinks	exon	79630991	79632390	.	+	.	gene_id XLOC_038410; transcript_id TCONS_00043810; exon_number 2; oId CUFF.85997.1; tss_id TSS89652;
9	Cufflinks	exon	89166309	89166572	.	+	.	gene_id GRMZM2G155868; transcript_id TCONS_00043863; exon_number 1; oId CUFF.86236.2; tss_id TSS89757;
9	Cufflinks	exon	89166704	89166834	.	+	.	gene_id GRMZM2G155868; transcript_id TCONS_00043863; exon_number 2; oId CUFF.86236.2; tss_id TSS89757;
9	Cufflinks	exon	89167951	89168284	.	+	.	gene_id GRMZM2G155868; transcript_id TCONS_00043863; exon_number 3; oId CUFF.86236.2; tss_id TSS89757;
9	Cufflinks	exon	89168384	89169976	.	+	.	gene_id GRMZM2G155868; transcript_id TCONS_00043863; exon_number 4; oId CUFF.86236.2; tss_id TSS89757;
9	Cufflinks	exon	107810066	107811122	.	+	.	gene_id GRMZM2G055320; transcript_id TCONS_00044016; exon_number 1; oId CUFF.86896.1; tss_id TSS90090;
9	Cufflinks	exon	107811594	107811947	.	+	.	gene_id GRMZM2G055320; transcript_id TCONS_00044016; exon_number 2; oId CUFF.86896.1; tss_id TSS90090;
9	Cufflinks	exon	107812543	107812741	.	+	.	gene_id GRMZM2G055320; transcript_id TCONS_00044016; exon_number 3; oId CUFF.86896.1; tss_id TSS90090;
9	Cufflinks	exon	107815759	107815972	.	+	.	gene_id GRMZM2G055320; transcript_id TCONS_00044016; exon_number 4; oId CUFF.86896.1; tss_id TSS90090;
9	Cufflinks	exon	107816069	107816171	.	+	.	gene_id GRMZM2G055320; transcript_id TCONS_00044016; exon_number 5; oId CUFF.86896.1; tss_id TSS90090;
9	Cufflinks	exon	107816348	107816743	.	+	.	gene_id GRMZM2G055320; transcript_id TCONS_00044016; exon_number 6; oId CUFF.86896.1; tss_id TSS90090;

9	Cufflinks	exon	109150958	109151675	.	+	.	gene_id XLOC_038611; transcript_id TCONS_00044035; exon_number 1; oId CUFF.86972.1; tss_id TSS90130;
9	Cufflinks	exon	111500012	111500553	.	+	.	gene_id XLOC_038630; transcript_id TCONS_00044056; exon_number 1; oId CUFF.87042.1; tss_id TSS90189;
9	Cufflinks	exon	143586299	143587418	.	+	.	gene_id GRMZM2G081175; transcript_id TCONS_00044545; exon_number 1; oId CUFF.88883.1; tss_id TSS91136;
9	Cufflinks	exon	143588328	143588494	.	+	.	gene_id GRMZM2G081175; transcript_id TCONS_00044545; exon_number 2; oId CUFF.88883.1; tss_id TSS91136;
9	Cufflinks	exon	143588727	143588811	.	+	.	gene_id GRMZM2G081175; transcript_id TCONS_00044545; exon_number 3; oId CUFF.88883.1; tss_id TSS91136;
9	Cufflinks	exon	143589613	143590618	.	+	.	gene_id GRMZM2G081175; transcript_id TCONS_00044545; exon_number 4; oId CUFF.88883.1; tss_id TSS91136;
9	Cufflinks	exon	143586299	143587418	.	+	.	gene_id GRMZM2G081175; transcript_id TCONS_00044546; exon_number 1; oId CUFF.88883.2; tss_id TSS91136;
9	Cufflinks	exon	143588328	143588494	.	+	.	gene_id GRMZM2G081175; transcript_id TCONS_00044546; exon_number 2; oId CUFF.88883.2; tss_id TSS91136;
9	Cufflinks	exon	143588727	143590618	.	+	.	gene_id GRMZM2G081175; transcript_id TCONS_00044546; exon_number 3; oId CUFF.88883.2; tss_id TSS91136;
9	Cufflinks	exon	144855170	144855542	.	+	.	gene_id GRMZM2G177914; transcript_id TCONS_00044567; exon_number 1; oId CUFF.88981.2; tss_id TSS91188;
9	Cufflinks	exon	144855742	144855784	.	+	.	gene_id GRMZM2G177914; transcript_id TCONS_00044567; exon_number 2; oId CUFF.88981.2; tss_id TSS91188;
9	Cufflinks	exon	144855858	144856231	.	+	.	gene_id GRMZM2G177914; transcript_id TCONS_00044567; exon_number 3; oId CUFF.88981.2; tss_id TSS91188;
9	Cufflinks	exon	144856631	144856701	.	+	.	gene_id GRMZM2G177914; transcript_id TCONS_00044567; exon_number 4; oId CUFF.88981.2; tss_id TSS91188;
9	Cufflinks	exon	144857724	144857815	.	+	.	gene_id GRMZM2G177914; transcript_id TCONS_00044567; exon_number 5; oId CUFF.88981.2; tss_id TSS91188;
9	Cufflinks	exon	144857900	144858225	.	+	.	gene_id GRMZM2G177914; transcript_id TCONS_00044567; exon_number 6; oId CUFF.88981.2; tss_id TSS91188;
9	Cufflinks	exon	146319637	146319803	.	+	.	gene_id GRMZM2G046601; transcript_id TCONS_00044591; exon_number 1; oId CUFF.89091.2; tss_id TSS91235;
9	Cufflinks	exon	146319999	146320038	.	+	.	gene_id GRMZM2G046601; transcript_id TCONS_00044591; exon_number 2; oId CUFF.89091.2; tss_id TSS91235;
9	Cufflinks	exon	146320197	146320526	.	+	.	gene_id GRMZM2G046601; transcript_id TCONS_00044591; exon_number 3; oId CUFF.89091.2; tss_id TSS91235;
9	Cufflinks	exon	146319637	146319803	.	+	.	gene_id GRMZM2G046601; transcript_id TCONS_00044592; exon_number 1; oId CUFF.89091.1; tss_id TSS91235;
9	Cufflinks	exon	146319999	146320038	.	+	.	gene_id GRMZM2G046601; transcript_id TCONS_00044592; exon_number 2; oId CUFF.89091.1; tss_id TSS91235;
9	Cufflinks	exon	146325595	146325701	.	+	.	gene_id GRMZM2G046601; transcript_id TCONS_00044592; exon_number 3; oId CUFF.89091.1; tss_id TSS91235;
9	Cufflinks	exon	146325972	146326059	.	+	.	gene_id GRMZM2G046601; transcript_id TCONS_00044592; exon_number 4; oId CUFF.89091.1; tss_id TSS91235;
9	Cufflinks	exon	146326187	146326315	.	+	.	gene_id GRMZM2G046601; transcript_id TCONS_00044592; exon_number 5; oId CUFF.89091.1; tss_id TSS91235;
9	Cufflinks	exon	146326399	146326473	.	+	.	gene_id GRMZM2G046601; transcript_id TCONS_00044592; exon_number 6; oId CUFF.89091.1; tss_id TSS91235;
9	Cufflinks	exon	146326625	146326678	.	+	.	gene_id GRMZM2G046601; transcript_id TCONS_00044592; exon_number 7; oId CUFF.89091.1; tss_id TSS91235;
9	Cufflinks	exon	146326748	146326945	.	+	.	gene_id GRMZM2G046601; transcript_id TCONS_00044592; exon_number 8; oId CUFF.89091.1; tss_id TSS91235;
9	Cufflinks	exon	146327025	146328720	.	+	.	gene_id GRMZM2G046601; transcript_id TCONS_00044592; exon_number 9; oId CUFF.89091.1; tss_id TSS91235;
9	Cufflinks	exon	152081582	152082290	.	+	.	gene_id GRMZM2G010017; transcript_id TCONS_00044735; exon_number 1; oId CUFF.89561.1; tss_id TSS91473;
9	Cufflinks	exon	152082604	152083045	.	+	.	gene_id GRMZM2G010017; transcript_id TCONS_00044735; exon_number 2; oId CUFF.89561.1; tss_id TSS91473;
9	Cufflinks	exon	152083649	152084016	.	+	.	gene_id GRMZM2G010017; transcript_id TCONS_00044735; exon_number 3; oId CUFF.89561.1; tss_id TSS91473;
9	Cufflinks	exon	152084604	152084840	.	+	.	gene_id GRMZM2G010017; transcript_id TCONS_00044735; exon_number 4; oId CUFF.89561.1; tss_id TSS91473;
9	Cufflinks	exon	152085387	152086034	.	+	.	gene_id GRMZM2G010017; transcript_id TCONS_00044735; exon_number 5; oId CUFF.89561.1; tss_id TSS91473;
9	Cufflinks	exon	152081582	152083045	.	+	.	gene_id GRMZM2G010017; transcript_id TCONS_00044736; exon_number 1; oId CUFF.89561.2; tss_id TSS91473;
9	Cufflinks	exon	152083649	152084016	.	+	.	gene_id GRMZM2G010017; transcript_id TCONS_00044736; exon_number 2; oId CUFF.89561.2; tss_id TSS91473;
9	Cufflinks	exon	152084604	152084840	.	+	.	gene_id GRMZM2G010017; transcript_id TCONS_00044736; exon_number 3; oId CUFF.89561.2; tss_id TSS91473;
9	Cufflinks	exon	152085387	152086034	.	+	.	gene_id GRMZM2G010017; transcript_id TCONS_00044736; exon_number 4; oId CUFF.89561.2; tss_id TSS91473;

9	Cufflinks	exon	154211427	154211993	.	+	.	gene_id XLOC_039267; transcript_id TCONS_00044803; exon_number 1; oId CUFF.89769.1; tss_id TSS91591;
9	Cufflinks	exon	6950322	6961103	.	-	.	gene_id GRMZM2G469502; transcript_id TCONS_00044912; exon_number 1; oId CUFF.83454.1; tss_id TSS91854;
9	Cufflinks	exon	8229971	8230501	.	-	.	gene_id GRMZM2G033413; transcript_id TCONS_00044939; exon_number 1; oId CUFF.83506.1; tss_id TSS91898;
9	Cufflinks	exon	8232489	8232518	.	-	.	gene_id GRMZM2G033413; transcript_id TCONS_00044939; exon_number 2; oId CUFF.83506.1; tss_id TSS91898;
9	Cufflinks	exon	8238629	8238700	.	-	.	gene_id GRMZM2G033413; transcript_id TCONS_00044939; exon_number 3; oId CUFF.83506.1; tss_id TSS91898;
9	Cufflinks	exon	8239418	8240288	.	-	.	gene_id GRMZM2G033413; transcript_id TCONS_00044939; exon_number 4; oId CUFF.83506.1; tss_id TSS91898;
9	Cufflinks	exon	14917204	14917939	.	-	.	gene_id GRMZM2G007683; transcript_id TCONS_00045072; exon_number 1; oId CUFF.83899.1; tss_id TSS92145;
9	Cufflinks	exon	14918015	14918108	.	-	.	gene_id GRMZM2G007683; transcript_id TCONS_00045072; exon_number 2; oId CUFF.83899.1; tss_id TSS92145;
9	Cufflinks	exon	14918214	14918247	.	-	.	gene_id GRMZM2G007683; transcript_id TCONS_00045072; exon_number 3; oId CUFF.83899.1; tss_id TSS92145;
9	Cufflinks	exon	14918943	14919109	.	-	.	gene_id GRMZM2G007683; transcript_id TCONS_00045072; exon_number 4; oId CUFF.83899.1; tss_id TSS92145;
9	Cufflinks	exon	14925248	14925544	.	-	.	gene_id GRMZM2G007683; transcript_id TCONS_00045072; exon_number 5; oId CUFF.83899.1; tss_id TSS92145;
9	Cufflinks	exon	15590769	15592249	.	-	.	gene_id GRMZM2G398736; transcript_id TCONS_00045081; exon_number 1; oId CUFF.83939.3; tss_id TSS92160;
9	Cufflinks	exon	15592389	15592492	.	-	.	gene_id GRMZM2G398736; transcript_id TCONS_00045081; exon_number 2; oId CUFF.83939.3; tss_id TSS92160;
9	Cufflinks	exon	15592579	15592652	.	-	.	gene_id GRMZM2G398736; transcript_id TCONS_00045081; exon_number 3; oId CUFF.83939.3; tss_id TSS92160;
9	Cufflinks	exon	15592756	15592948	.	-	.	gene_id GRMZM2G398736; transcript_id TCONS_00045081; exon_number 4; oId CUFF.83939.3; tss_id TSS92160;
9	Cufflinks	exon	15590769	15591582	.	-	.	gene_id GRMZM2G398736; transcript_id TCONS_00045082; exon_number 1; oId CUFF.83939.1; tss_id TSS92160;
9	Cufflinks	exon	15592181	15592249	.	-	.	gene_id GRMZM2G398736; transcript_id TCONS_00045082; exon_number 2; oId CUFF.83939.1; tss_id TSS92160;
9	Cufflinks	exon	15592389	15592492	.	-	.	gene_id GRMZM2G398736; transcript_id TCONS_00045082; exon_number 3; oId CUFF.83939.1; tss_id TSS92160;
9	Cufflinks	exon	15592579	15592652	.	-	.	gene_id GRMZM2G398736; transcript_id TCONS_00045082; exon_number 4; oId CUFF.83939.1; tss_id TSS92160;
9	Cufflinks	exon	15592756	15592948	.	-	.	gene_id GRMZM2G398736; transcript_id TCONS_00045082; exon_number 5; oId CUFF.83939.1; tss_id TSS92160;
9	Cufflinks	exon	15590769	15591564	.	-	.	gene_id GRMZM2G398736; transcript_id TCONS_00045083; exon_number 1; oId CUFF.83939.2; tss_id TSS92160;
9	Cufflinks	exon	15592181	15592249	.	-	.	gene_id GRMZM2G398736; transcript_id TCONS_00045083; exon_number 2; oId CUFF.83939.2; tss_id TSS92160;
9	Cufflinks	exon	15592389	15592492	.	-	.	gene_id GRMZM2G398736; transcript_id TCONS_00045083; exon_number 3; oId CUFF.83939.2; tss_id TSS92160;
9	Cufflinks	exon	15592579	15592652	.	-	.	gene_id GRMZM2G398736; transcript_id TCONS_00045083; exon_number 4; oId CUFF.83939.2; tss_id TSS92160;
9	Cufflinks	exon	15592756	15592948	.	-	.	gene_id GRMZM2G398736; transcript_id TCONS_00045083; exon_number 5; oId CUFF.83939.2; tss_id TSS92160;
9	Cufflinks	exon	15620220	15622664	.	-	.	gene_id GRMZM5G865595; transcript_id TCONS_00045085; exon_number 1; oId CUFF.83931.1; tss_id TSS92165;
9	Cufflinks	exon	15622779	15622901	.	-	.	gene_id GRMZM5G865595; transcript_id TCONS_00045085; exon_number 2; oId CUFF.83931.1; tss_id TSS92165;
9	Cufflinks	exon	15622995	15623481	.	-	.	gene_id GRMZM5G865595; transcript_id TCONS_00045085; exon_number 3; oId CUFF.83931.1; tss_id TSS92165;
9	Cufflinks	exon	15623580	15624084	.	-	.	gene_id GRMZM5G865595; transcript_id TCONS_00045085; exon_number 4; oId CUFF.83931.1; tss_id TSS92165;
9	Cufflinks	exon	18335677	18336787	.	-	.	gene_id AC231745.1_FG003; transcript_id TCONS_00045113; exon_number 1; oId CUFF.84142.2; tss_id TSS92246;
9	Cufflinks	exon	18337443	18337865	.	-	.	gene_id AC231745.1_FG003; transcript_id TCONS_00045113; exon_number 2; oId CUFF.84142.2; tss_id TSS92246;
9	Cufflinks	exon	18338031	18338579	.	-	.	gene_id AC231745.1_FG003; transcript_id TCONS_00045113; exon_number 3; oId CUFF.84142.2; tss_id TSS92246;
9	Cufflinks	exon	18885308	18885947	.	-	.	gene_id GRMZM2G103137; transcript_id TCONS_00045119; exon_number 1; oId CUFF.84200.6; tss_id TSS92254;
9	Cufflinks	exon	18886048	18886145	.	-	.	gene_id GRMZM2G103137; transcript_id TCONS_00045119; exon_number 2; oId CUFF.84200.6; tss_id TSS92254;
9	Cufflinks	exon	18886225	18886393	.	-	.	gene_id GRMZM2G103137; transcript_id TCONS_00045119; exon_number 3; oId CUFF.84200.6; tss_id TSS92254;
9	Cufflinks	exon	18886499	18886551	.	-	.	gene_id GRMZM2G103137; transcript_id TCONS_00045119; exon_number 4; oId CUFF.84200.6; tss_id TSS92254;

9	Cufflinks	exon	18886657	18886712	.	-	.	gene_id GRMZM2G103137; transcript_id TCONS_00045119; exon_number 5; oId CUFF.84200.6; tss_id TSS92254;
9	Cufflinks	exon	18886793	18887527	.	-	.	gene_id GRMZM2G103137; transcript_id TCONS_00045119; exon_number 6; oId CUFF.84200.6; tss_id TSS92254;
9	Cufflinks	exon	18887655	18887725	.	-	.	gene_id GRMZM2G103137; transcript_id TCONS_00045119; exon_number 7; oId CUFF.84200.6; tss_id TSS92254;
9	Cufflinks	exon	18887868	18887935	.	-	.	gene_id GRMZM2G103137; transcript_id TCONS_00045119; exon_number 8; oId CUFF.84200.6; tss_id TSS92254;
9	Cufflinks	exon	18888093	18888376	.	-	.	gene_id GRMZM2G103137; transcript_id TCONS_00045119; exon_number 9; oId CUFF.84200.6; tss_id TSS92254;
9	Cufflinks	exon	18885308	18885947	.	-	.	gene_id GRMZM2G103137; transcript_id TCONS_00045120; exon_number 1; oId CUFF.84200.4; tss_id TSS92254;
9	Cufflinks	exon	18886048	18886145	.	-	.	gene_id GRMZM2G103137; transcript_id TCONS_00045120; exon_number 2; oId CUFF.84200.4; tss_id TSS92254;
9	Cufflinks	exon	18886225	18886393	.	-	.	gene_id GRMZM2G103137; transcript_id TCONS_00045120; exon_number 3; oId CUFF.84200.4; tss_id TSS92254;
9	Cufflinks	exon	18886499	18886551	.	-	.	gene_id GRMZM2G103137; transcript_id TCONS_00045120; exon_number 4; oId CUFF.84200.4; tss_id TSS92254;
9	Cufflinks	exon	18886657	18886712	.	-	.	gene_id GRMZM2G103137; transcript_id TCONS_00045120; exon_number 5; oId CUFF.84200.4; tss_id TSS92254;
9	Cufflinks	exon	18886793	18886956	.	-	.	gene_id GRMZM2G103137; transcript_id TCONS_00045120; exon_number 6; oId CUFF.84200.4; tss_id TSS92254;
9	Cufflinks	exon	18887252	18887527	.	-	.	gene_id GRMZM2G103137; transcript_id TCONS_00045120; exon_number 7; oId CUFF.84200.4; tss_id TSS92254;
9	Cufflinks	exon	18887655	18887725	.	-	.	gene_id GRMZM2G103137; transcript_id TCONS_00045120; exon_number 8; oId CUFF.84200.4; tss_id TSS92254;
9	Cufflinks	exon	18887868	18887935	.	-	.	gene_id GRMZM2G103137; transcript_id TCONS_00045120; exon_number 9; oId CUFF.84200.4; tss_id TSS92254;
9	Cufflinks	exon	18888093	18888376	.	-	.	gene_id GRMZM2G103137; transcript_id TCONS_00045120; exon_number 10; oId CUFF.84200.4; tss_id TSS92254;
9	Cufflinks	exon	18885308	18885947	.	-	.	gene_id GRMZM2G103137; transcript_id TCONS_00045121; exon_number 1; oId CUFF.84200.12; tss_id TSS92254;
9	Cufflinks	exon	18886048	18886145	.	-	.	gene_id GRMZM2G103137; transcript_id TCONS_00045121; exon_number 2; oId CUFF.84200.12; tss_id TSS92254;
9	Cufflinks	exon	18886225	18886393	.	-	.	gene_id GRMZM2G103137; transcript_id TCONS_00045121; exon_number 3; oId CUFF.84200.12; tss_id TSS92254;
9	Cufflinks	exon	18886499	18886551	.	-	.	gene_id GRMZM2G103137; transcript_id TCONS_00045121; exon_number 4; oId CUFF.84200.12; tss_id TSS92254;
9	Cufflinks	exon	18886657	18886712	.	-	.	gene_id GRMZM2G103137; transcript_id TCONS_00045121; exon_number 5; oId CUFF.84200.12; tss_id TSS92254;
9	Cufflinks	exon	18886793	18886956	.	-	.	gene_id GRMZM2G103137; transcript_id TCONS_00045121; exon_number 6; oId CUFF.84200.12; tss_id TSS92254;
9	Cufflinks	exon	18887252	18887527	.	-	.	gene_id GRMZM2G103137; transcript_id TCONS_00045121; exon_number 7; oId CUFF.84200.12; tss_id TSS92254;
9	Cufflinks	exon	18887668	18887725	.	-	.	gene_id GRMZM2G103137; transcript_id TCONS_00045121; exon_number 8; oId CUFF.84200.12; tss_id TSS92254;
9	Cufflinks	exon	18887868	18887935	.	-	.	gene_id GRMZM2G103137; transcript_id TCONS_00045121; exon_number 9; oId CUFF.84200.12; tss_id TSS92254;
9	Cufflinks	exon	18888093	18888376	.	-	.	gene_id GRMZM2G103137; transcript_id TCONS_00045121; exon_number 10; oId CUFF.84200.12; tss_id TSS92254;
9	Cufflinks	exon	18885308	18885947	.	-	.	gene_id GRMZM2G103137; transcript_id TCONS_00045122; exon_number 1; oId CUFF.84200.14; tss_id TSS92254;
9	Cufflinks	exon	18886048	18886145	.	-	.	gene_id GRMZM2G103137; transcript_id TCONS_00045122; exon_number 2; oId CUFF.84200.14; tss_id TSS92254;
9	Cufflinks	exon	18886225	18886393	.	-	.	gene_id GRMZM2G103137; transcript_id TCONS_00045122; exon_number 3; oId CUFF.84200.14; tss_id TSS92254;
9	Cufflinks	exon	18886499	18886551	.	-	.	gene_id GRMZM2G103137; transcript_id TCONS_00045122; exon_number 4; oId CUFF.84200.14; tss_id TSS92254;
9	Cufflinks	exon	18886657	18886712	.	-	.	gene_id GRMZM2G103137; transcript_id TCONS_00045122; exon_number 5; oId CUFF.84200.14; tss_id TSS92254;
9	Cufflinks	exon	18886793	18886956	.	-	.	gene_id GRMZM2G103137; transcript_id TCONS_00045122; exon_number 6; oId CUFF.84200.14; tss_id TSS92254;
9	Cufflinks	exon	18887252	18887527	.	-	.	gene_id GRMZM2G103137; transcript_id TCONS_00045122; exon_number 7; oId CUFF.84200.14; tss_id TSS92254;
9	Cufflinks	exon	18887655	18887785	.	-	.	gene_id GRMZM2G103137; transcript_id TCONS_00045122; exon_number 8; oId CUFF.84200.14; tss_id TSS92254;
9	Cufflinks	exon	18887868	18887935	.	-	.	gene_id GRMZM2G103137; transcript_id TCONS_00045122; exon_number 9; oId CUFF.84200.14; tss_id TSS92254;
9	Cufflinks	exon	18888093	18888376	.	-	.	gene_id GRMZM2G103137; transcript_id TCONS_00045122; exon_number 10; oId CUFF.84200.14; tss_id TSS92254;
9	Cufflinks	exon	18885308	18885947	.	-	.	gene_id GRMZM2G103137; transcript_id TCONS_00045123; exon_number 1; oId CUFF.84200.1; tss_id TSS92254;

9	Cufflinks	exon	18886048	18886145	.	-	.	gene_id GRMZM2G103137; transcript_id TCONS_00045123; exon_number 2; oId CUFF.84200.1; tss_id TSS92254;
9	Cufflinks	exon	18886225	18886393	.	-	.	gene_id GRMZM2G103137; transcript_id TCONS_00045123; exon_number 3; oId CUFF.84200.1; tss_id TSS92254;
9	Cufflinks	exon	18886499	18886551	.	-	.	gene_id GRMZM2G103137; transcript_id TCONS_00045123; exon_number 4; oId CUFF.84200.1; tss_id TSS92254;
9	Cufflinks	exon	18886657	18886712	.	-	.	gene_id GRMZM2G103137; transcript_id TCONS_00045123; exon_number 5; oId CUFF.84200.1; tss_id TSS92254;
9	Cufflinks	exon	18886793	18886956	.	-	.	gene_id GRMZM2G103137; transcript_id TCONS_00045123; exon_number 6; oId CUFF.84200.1; tss_id TSS92254;
9	Cufflinks	exon	18887252	18887527	.	-	.	gene_id GRMZM2G103137; transcript_id TCONS_00045123; exon_number 7; oId CUFF.84200.1; tss_id TSS92254;
9	Cufflinks	exon	18887668	18887785	.	-	.	gene_id GRMZM2G103137; transcript_id TCONS_00045123; exon_number 8; oId CUFF.84200.1; tss_id TSS92254;
9	Cufflinks	exon	18887868	18887935	.	-	.	gene_id GRMZM2G103137; transcript_id TCONS_00045123; exon_number 9; oId CUFF.84200.1; tss_id TSS92254;
9	Cufflinks	exon	18888093	18888376	.	-	.	gene_id GRMZM2G103137; transcript_id TCONS_00045123; exon_number 10; oId CUFF.84200.1; tss_id TSS92254;
9	Cufflinks	exon	23765379	23767204	.	-	.	gene_id GRMZM2G118690; transcript_id TCONS_00045201; exon_number 1; oId CUFF.84411.2; tss_id TSS92397;
9	Cufflinks	exon	23768528	23768649	.	-	.	gene_id GRMZM2G118690; transcript_id TCONS_00045201; exon_number 2; oId CUFF.84411.2; tss_id TSS92397;
9	Cufflinks	exon	23771076	23771219	.	-	.	gene_id GRMZM2G118690; transcript_id TCONS_00045201; exon_number 3; oId CUFF.84411.2; tss_id TSS92397;
9	Cufflinks	exon	23765379	23767204	.	-	.	gene_id GRMZM2G118690; transcript_id TCONS_00045202; exon_number 1; oId CUFF.84411.1; tss_id TSS92397;
9	Cufflinks	exon	23768528	23768649	.	-	.	gene_id GRMZM2G118690; transcript_id TCONS_00045202; exon_number 2; oId CUFF.84411.1; tss_id TSS92397;
9	Cufflinks	exon	23771006	23771206	.	-	.	gene_id GRMZM2G118690; transcript_id TCONS_00045202; exon_number 3; oId CUFF.84411.1; tss_id TSS92397;
9	Cufflinks	exon	26833946	26834965	.	-	.	gene_id GRMZM2G475360; transcript_id TCONS_00045250; exon_number 1; oId CUFF.84593.1; tss_id TSS92473;
9	Cufflinks	exon	26835003	26835117	.	-	.	gene_id GRMZM2G475360; transcript_id TCONS_00045250; exon_number 2; oId CUFF.84593.1; tss_id TSS92473;
9	Cufflinks	exon	26835230	26835373	.	-	.	gene_id GRMZM2G475360; transcript_id TCONS_00045250; exon_number 3; oId CUFF.84593.1; tss_id TSS92473;
9	Cufflinks	exon	26835514	26835715	.	-	.	gene_id GRMZM2G475360; transcript_id TCONS_00045250; exon_number 4; oId CUFF.84593.1; tss_id TSS92473;
9	Cufflinks	exon	26835807	26835871	.	-	.	gene_id GRMZM2G475360; transcript_id TCONS_00045250; exon_number 5; oId CUFF.84593.1; tss_id TSS92473;
9	Cufflinks	exon	26835962	26836027	.	-	.	gene_id GRMZM2G475360; transcript_id TCONS_00045250; exon_number 6; oId CUFF.84593.1; tss_id TSS92473;
9	Cufflinks	exon	26836199	26836303	.	-	.	gene_id GRMZM2G475360; transcript_id TCONS_00045250; exon_number 7; oId CUFF.84593.1; tss_id TSS92473;
9	Cufflinks	exon	26836394	26836510	.	-	.	gene_id GRMZM2G475360; transcript_id TCONS_00045250; exon_number 8; oId CUFF.84593.1; tss_id TSS92473;
9	Cufflinks	exon	26836724	26836876	.	-	.	gene_id GRMZM2G475360; transcript_id TCONS_00045250; exon_number 9; oId CUFF.84593.1; tss_id TSS92473;
9	Cufflinks	exon	26837180	26837793	.	-	.	gene_id GRMZM2G475360; transcript_id TCONS_00045250; exon_number 10; oId CUFF.84593.1; tss_id TSS92473;
9	Cufflinks	exon	26838267	26839205	.	-	.	gene_id GRMZM2G475360; transcript_id TCONS_00045250; exon_number 11; oId CUFF.84593.1; tss_id TSS92473;
9	Cufflinks	exon	32305077	32306671	.	-	.	gene_id XLOC_039692; transcript_id TCONS_00045296; exon_number 1; oId CUFF.84767.1; tss_id TSS92566;
9	Cufflinks	exon	32308239	32309434	.	-	.	gene_id XLOC_039694; transcript_id TCONS_00045298; exon_number 1; oId CUFF.84736.1; tss_id TSS92568;
9	Cufflinks	exon	73804250	73806195	.	-	.	gene_id XLOC_039882; transcript_id TCONS_00045501; exon_number 1; oId CUFF.85909.2; tss_id TSS93089;
9	Cufflinks	exon	78160997	78162100	.	-	.	gene_id XLOC_039902; transcript_id TCONS_00045522; exon_number 1; oId CUFF.85962.1; tss_id TSS93171;
9	Cufflinks	exon	79629909	79630363	.	-	.	gene_id GRMZM2G161472; transcript_id TCONS_00045525; exon_number 1; oId CUFF.85996.1; tss_id TSS93186;
9	Cufflinks	exon	79630442	79630636	.	-	.	gene_id GRMZM2G161472; transcript_id TCONS_00045525; exon_number 2; oId CUFF.85996.1; tss_id TSS93186;
9	Cufflinks	exon	79630724	79630882	.	-	.	gene_id GRMZM2G161472; transcript_id TCONS_00045525; exon_number 3; oId CUFF.85996.1; tss_id TSS93186;
9	Cufflinks	exon	79630995	79631249	.	-	.	gene_id GRMZM2G161472; transcript_id TCONS_00045525; exon_number 4; oId CUFF.85996.1; tss_id TSS93186;
9	Cufflinks	exon	79631352	79631492	.	-	.	gene_id GRMZM2G161472; transcript_id TCONS_00045525; exon_number 5; oId CUFF.85996.1; tss_id TSS93186;
9	Cufflinks	exon	79631608	79631769	.	-	.	gene_id GRMZM2G161472; transcript_id TCONS_00045525; exon_number 6; oId CUFF.85996.1; tss_id TSS93186;

9	Cufflinks	exon	79632221	79632381	.	-	.	gene_id GRMZM2G161472; transcript_id TCONS_00045525; exon_number 7; oId CUFF.85996.1; tss_id TSS93186;
9	Cufflinks	exon	79633829	79634177	.	-	.	gene_id GRMZM2G161472; transcript_id TCONS_00045525; exon_number 8; oId CUFF.85996.1; tss_id TSS93186;
9	Cufflinks	exon	102482867	102484265	.	-	.	gene_id GRMZM5G836222; transcript_id TCONS_00045700; exon_number 1; oId CUFF.86718.1; tss_id TSS93565;
9	Cufflinks	exon	102484911	102484953	.	-	.	gene_id GRMZM5G836222; transcript_id TCONS_00045700; exon_number 2; oId CUFF.86718.1; tss_id TSS93565;
9	Cufflinks	exon	102485111	102485230	.	-	.	gene_id GRMZM5G836222; transcript_id TCONS_00045700; exon_number 3; oId CUFF.86718.1; tss_id TSS93565;
9	Cufflinks	exon	103112503	103113053	.	-	.	gene_id XLOC_040069; transcript_id TCONS_00045703; exon_number 1; oId CUFF.86713.1; tss_id TSS93568;
9	Cufflinks	exon	104241829	104242613	.	-	.	gene_id XLOC_040083; transcript_id TCONS_00045717; exon_number 1; oId CUFF.86755.1; tss_id TSS93586;
9	Cufflinks	exon	108449125	108449560	.	-	.	gene_id XLOC_040129; transcript_id TCONS_00045766; exon_number 1; oId CUFF.86919.1; tss_id TSS93689;
9	Cufflinks	exon	109146317	109146870	.	-	.	gene_id GRMZM2G103805; transcript_id TCONS_00045771; exon_number 1; oId CUFF.86970.1; tss_id TSS93708;
9	Cufflinks	exon	109147058	109147096	.	-	.	gene_id GRMZM2G103805; transcript_id TCONS_00045771; exon_number 2; oId CUFF.86970.1; tss_id TSS93708;
9	Cufflinks	exon	109147177	109147299	.	-	.	gene_id GRMZM2G103805; transcript_id TCONS_00045771; exon_number 3; oId CUFF.86970.1; tss_id TSS93708;
9	Cufflinks	exon	109147380	109147436	.	-	.	gene_id GRMZM2G103805; transcript_id TCONS_00045771; exon_number 4; oId CUFF.86970.1; tss_id TSS93708;
9	Cufflinks	exon	109148007	109148087	.	-	.	gene_id GRMZM2G103805; transcript_id TCONS_00045771; exon_number 5; oId CUFF.86970.1; tss_id TSS93708;
9	Cufflinks	exon	109148231	109148321	.	-	.	gene_id GRMZM2G103805; transcript_id TCONS_00045771; exon_number 6; oId CUFF.86970.1; tss_id TSS93708;
9	Cufflinks	exon	109148448	109148519	.	-	.	gene_id GRMZM2G103805; transcript_id TCONS_00045771; exon_number 7; oId CUFF.86970.1; tss_id TSS93708;
9	Cufflinks	exon	109148600	109148700	.	-	.	gene_id GRMZM2G103805; transcript_id TCONS_00045771; exon_number 8; oId CUFF.86970.1; tss_id TSS93708;
9	Cufflinks	exon	109149642	109149740	.	-	.	gene_id GRMZM2G103805; transcript_id TCONS_00045771; exon_number 9; oId CUFF.86970.1; tss_id TSS93708;
9	Cufflinks	exon	109150290	109150779	.	-	.	gene_id GRMZM2G103805; transcript_id TCONS_00045771; exon_number 10; oId CUFF.86970.1; tss_id TSS93708;
9	Cufflinks	exon	109150880	109151812	.	-	.	gene_id GRMZM2G103805; transcript_id TCONS_00045771; exon_number 11; oId CUFF.86970.1; tss_id TSS93708;
9	Cufflinks	exon	109146317	109146870	.	-	.	gene_id GRMZM2G103805; transcript_id TCONS_00045772; exon_number 1; oId CUFF.86970.2; tss_id TSS93708;
9	Cufflinks	exon	109147058	109147096	.	-	.	gene_id GRMZM2G103805; transcript_id TCONS_00045772; exon_number 2; oId CUFF.86970.2; tss_id TSS93708;
9	Cufflinks	exon	109147177	109147299	.	-	.	gene_id GRMZM2G103805; transcript_id TCONS_00045772; exon_number 3; oId CUFF.86970.2; tss_id TSS93708;
9	Cufflinks	exon	109147380	109147436	.	-	.	gene_id GRMZM2G103805; transcript_id TCONS_00045772; exon_number 4; oId CUFF.86970.2; tss_id TSS93708;
9	Cufflinks	exon	109148007	109148087	.	-	.	gene_id GRMZM2G103805; transcript_id TCONS_00045772; exon_number 5; oId CUFF.86970.2; tss_id TSS93708;
9	Cufflinks	exon	109148231	109148321	.	-	.	gene_id GRMZM2G103805; transcript_id TCONS_00045772; exon_number 6; oId CUFF.86970.2; tss_id TSS93708;
9	Cufflinks	exon	109148448	109148519	.	-	.	gene_id GRMZM2G103805; transcript_id TCONS_00045772; exon_number 7; oId CUFF.86970.2; tss_id TSS93708;
9	Cufflinks	exon	109148600	109148700	.	-	.	gene_id GRMZM2G103805; transcript_id TCONS_00045772; exon_number 8; oId CUFF.86970.2; tss_id TSS93708;
9	Cufflinks	exon	109149642	109149740	.	-	.	gene_id GRMZM2G103805; transcript_id TCONS_00045772; exon_number 9; oId CUFF.86970.2; tss_id TSS93708;
9	Cufflinks	exon	109150290	109150779	.	-	.	gene_id GRMZM2G103805; transcript_id TCONS_00045772; exon_number 10; oId CUFF.86970.2; tss_id TSS93708;
9	Cufflinks	exon	109151450	109151812	.	-	.	gene_id GRMZM2G103805; transcript_id TCONS_00045772; exon_number 11; oId CUFF.86970.2; tss_id TSS93708;
9	Cufflinks	exon	109311410	109312702	.	-	.	gene_id GRMZM2G157115; transcript_id TCONS_00045776; exon_number 1; oId CUFF.86968.1; tss_id TSS93715;
9	Cufflinks	exon	109312840	109312963	.	-	.	gene_id GRMZM2G157115; transcript_id TCONS_00045776; exon_number 2; oId CUFF.86968.1; tss_id TSS93715;
9	Cufflinks	exon	109314341	109314477	.	-	.	gene_id GRMZM2G157115; transcript_id TCONS_00045776; exon_number 3; oId CUFF.86968.1; tss_id TSS93715;
9	Cufflinks	exon	109315107	109315242	.	-	.	gene_id GRMZM2G157115; transcript_id TCONS_00045776; exon_number 4; oId CUFF.86968.1; tss_id TSS93715;
9	Cufflinks	exon	109315570	109315880	.	-	.	gene_id GRMZM2G157115; transcript_id TCONS_00045776; exon_number 5; oId CUFF.86968.1; tss_id TSS93715;
9	Cufflinks	exon	109316960	109317567	.	-	.	gene_id GRMZM2G157115; transcript_id TCONS_00045776; exon_number 6; oId CUFF.86968.1; tss_id TSS93715;

9	Cufflinks	exon	111478933	111480601	.	-	.	gene_id GRMZM2G017739; transcript_id TCONS_00045805; exon_number 1; oId CUFF.87055.2; tss_id TSS93761;
9	Cufflinks	exon	111480780	111480939	.	-	.	gene_id GRMZM2G017739; transcript_id TCONS_00045805; exon_number 2; oId CUFF.87055.2; tss_id TSS93761;
9	Cufflinks	exon	111481992	111482476	.	-	.	gene_id GRMZM2G017739; transcript_id TCONS_00045805; exon_number 3; oId CUFF.87055.2; tss_id TSS93761;
9	Cufflinks	exon	114715073	114715420	.	-	.	gene_id XLOC_040198; transcript_id TCONS_00045847; exon_number 1; oId CUFF.87252.1; tss_id TSS93851;
9	Cufflinks	exon	117079224	117080432	.	-	.	gene_id GRMZM2G409245; transcript_id TCONS_00045864; exon_number 1; oId CUFF.87367.1; tss_id TSS93911;
9	Cufflinks	exon	120202329	120202687	.	-	.	gene_id XLOC_040245; transcript_id TCONS_00045900; exon_number 1; oId CUFF.87529.1; tss_id TSS93982;
9	Cufflinks	exon	120203875	120204097	.	-	.	gene_id XLOC_040245; transcript_id TCONS_00045900; exon_number 2; oId CUFF.87529.1; tss_id TSS93982;
9	Cufflinks	exon	120202329	120202763	.	-	.	gene_id XLOC_040245; transcript_id TCONS_00045901; exon_number 1; oId CUFF.87529.2; tss_id TSS93982;
9	Cufflinks	exon	120203875	120204023	.	-	.	gene_id XLOC_040245; transcript_id TCONS_00045901; exon_number 2; oId CUFF.87529.2; tss_id TSS93982;
9	Cufflinks	exon	124924237	124925395	.	-	.	gene_id GRMZM2G105092; transcript_id TCONS_00045952; exon_number 1; oId CUFF.87740.1; tss_id TSS94102;
9	Cufflinks	exon	127611832	127613195	.	-	.	gene_id XLOC_040325; transcript_id TCONS_00045993; exon_number 1; oId CUFF.87866.1; tss_id TSS94181;
9	Cufflinks	exon	136728264	136729499	.	-	.	gene_id GRMZM5G889372; transcript_id TCONS_00046146; exon_number 1; oId CUFF.88390.1; tss_id TSS94482;
9	Cufflinks	exon	136729665	136730464	.	-	.	gene_id GRMZM5G889372; transcript_id TCONS_00046146; exon_number 2; oId CUFF.88390.1; tss_id TSS94482;
9	Cufflinks	exon	136731673	136733501	.	-	.	gene_id GRMZM5G889372; transcript_id TCONS_00046146; exon_number 3; oId CUFF.88390.1; tss_id TSS94482;
9	Cufflinks	exon	136733613	136734142	.	-	.	gene_id GRMZM5G889372; transcript_id TCONS_00046146; exon_number 4; oId CUFF.88390.1; tss_id TSS94482;
9	Cufflinks	exon	143586334	143587418	.	-	.	gene_id XLOC_040581; transcript_id TCONS_00046298; exon_number 1; oId CUFF.88884.2; tss_id TSS94760;
9	Cufflinks	exon	143588328	143588750	.	-	.	gene_id XLOC_040581; transcript_id TCONS_00046298; exon_number 2; oId CUFF.88884.2; tss_id TSS94760;
9	Cufflinks	exon	144212381	144213018	.	-	.	gene_id XLOC_040591; transcript_id TCONS_00046309; exon_number 1; oId CUFF.88930.1; tss_id TSS94781;
9	Cufflinks	exon	145046440	145046931	.	-	.	gene_id XLOC_040607; transcript_id TCONS_00046326; exon_number 1; oId CUFF.88990.1; tss_id TSS94822;
9	Cufflinks	exon	145047052	145047353	.	-	.	gene_id XLOC_040607; transcript_id TCONS_00046326; exon_number 2; oId CUFF.88990.1; tss_id TSS94822;
9	Cufflinks	exon	149513489	149513867	.	-	.	gene_id GRMZM2G004377; transcript_id TCONS_00046448; exon_number 1; oId CUFF.89326.1; tss_id TSS95006;
9	Cufflinks	exon	149513969	149514097	.	-	.	gene_id GRMZM2G004377; transcript_id TCONS_00046448; exon_number 2; oId CUFF.89326.1; tss_id TSS95006;
9	Cufflinks	exon	149514180	149514323	.	-	.	gene_id GRMZM2G004377; transcript_id TCONS_00046448; exon_number 3; oId CUFF.89326.1; tss_id TSS95006;
9	Cufflinks	exon	149514460	149514629	.	-	.	gene_id GRMZM2G004377; transcript_id TCONS_00046448; exon_number 4; oId CUFF.89326.1; tss_id TSS95006;
9	Cufflinks	exon	149516025	149516112	.	-	.	gene_id GRMZM2G004377; transcript_id TCONS_00046448; exon_number 5; oId CUFF.89326.1; tss_id TSS95006;
9	Cufflinks	exon	149516738	149516807	.	-	.	gene_id GRMZM2G004377; transcript_id TCONS_00046448; exon_number 6; oId CUFF.89326.1; tss_id TSS95006;
9	Cufflinks	exon	149517017	149517078	.	-	.	gene_id GRMZM2G004377; transcript_id TCONS_00046448; exon_number 7; oId CUFF.89326.1; tss_id TSS95006;
9	Cufflinks	exon	149517865	149518715	.	-	.	gene_id GRMZM2G004377; transcript_id TCONS_00046448; exon_number 8; oId CUFF.89326.1; tss_id TSS95006;
9	Cufflinks	exon	149518877	149519493	.	-	.	gene_id GRMZM2G004377; transcript_id TCONS_00046448; exon_number 9; oId CUFF.89326.1; tss_id TSS95006;
9	Cufflinks	exon	151381654	151383564	.	-	.	gene_id AC149829.2_FG002; transcript_id TCONS_00046499; exon_number 1; oId CUFF.89462.1; tss_id TSS95107;
9	Cufflinks	exon	151414935	151417236	.	-	.	gene_id XLOC_040760; transcript_id TCONS_00046504; exon_number 1; oId CUFF.89486.2; tss_id TSS95113;
9	Cufflinks	exon	151417519	151418059	.	-	.	gene_id XLOC_040760; transcript_id TCONS_00046504; exon_number 2; oId CUFF.89486.2; tss_id TSS95113;
9	Cufflinks	exon	151414935	151417236	.	-	.	gene_id XLOC_040760; transcript_id TCONS_00046505; exon_number 1; oId CUFF.89486.1; tss_id TSS95113;
9	Cufflinks	exon	151417341	151417410	.	-	.	gene_id XLOC_040760; transcript_id TCONS_00046505; exon_number 2; oId CUFF.89486.1; tss_id TSS95113;
9	Cufflinks	exon	151417519	151418059	.	-	.	gene_id XLOC_040760; transcript_id TCONS_00046505; exon_number 3; oId CUFF.89486.1; tss_id TSS95113;
9	Cufflinks	exon	151542239	151543232	.	-	.	gene_id GRMZM2G178826; transcript_id TCONS_00046508; exon_number 1; oId CUFF.89517.3; tss_id TSS95116;

9	Cufflinks	exon	151543847	151543922	.	-	.	gene_id GRMZM2G178826; transcript_id TCONS_00046508; exon_number 2; oId CUFF.89517.3; tss_id TSS95116;
9	Cufflinks	exon	151544005	151544172	.	-	.	gene_id GRMZM2G178826; transcript_id TCONS_00046508; exon_number 3; oId CUFF.89517.3; tss_id TSS95116;
9	Cufflinks	exon	151544277	151544366	.	-	.	gene_id GRMZM2G178826; transcript_id TCONS_00046508; exon_number 4; oId CUFF.89517.3; tss_id TSS95116;
9	Cufflinks	exon	151544588	151544728	.	-	.	gene_id GRMZM2G178826; transcript_id TCONS_00046508; exon_number 5; oId CUFF.89517.3; tss_id TSS95116;
9	Cufflinks	exon	151544933	151545001	.	-	.	gene_id GRMZM2G178826; transcript_id TCONS_00046508; exon_number 6; oId CUFF.89517.3; tss_id TSS95116;
9	Cufflinks	exon	151545118	151545216	.	-	.	gene_id GRMZM2G178826; transcript_id TCONS_00046508; exon_number 7; oId CUFF.89517.3; tss_id TSS95116;
9	Cufflinks	exon	151545537	151545706	.	-	.	gene_id GRMZM2G178826; transcript_id TCONS_00046508; exon_number 8; oId CUFF.89517.3; tss_id TSS95116;
9	Cufflinks	exon	151545873	151546061	.	-	.	gene_id GRMZM2G178826; transcript_id TCONS_00046508; exon_number 9; oId CUFF.89517.3; tss_id TSS95116;
9	Cufflinks	exon	151546281	151546401	.	-	.	gene_id GRMZM2G178826; transcript_id TCONS_00046508; exon_number 10; oId CUFF.89517.3; tss_id TSS95116;
9	Cufflinks	exon	151998593	151999043	.	-	.	gene_id XLOC_040785; transcript_id TCONS_00046531; exon_number 1; oId CUFF.89543.1; tss_id TSS95149;
9	Cufflinks	exon	151999349	152000146	.	-	.	gene_id XLOC_040786; transcript_id TCONS_00046532; exon_number 1; oId CUFF.89544.1; tss_id TSS95150;
9	Cufflinks	exon	153128005	153128595	.	-	.	gene_id GRMZM2G330019; transcript_id TCONS_00046564; exon_number 1; oId CUFF.89678.1; tss_id TSS95208;
9	Cufflinks	exon	153128701	153128847	.	-	.	gene_id GRMZM2G330019; transcript_id TCONS_00046564; exon_number 2; oId CUFF.89678.1; tss_id TSS95208;
9	Cufflinks	exon	153128941	153129153	.	-	.	gene_id GRMZM2G330019; transcript_id TCONS_00046564; exon_number 3; oId CUFF.89678.1; tss_id TSS95208;
9	Cufflinks	exon	153129241	153129825	.	-	.	gene_id GRMZM2G330019; transcript_id TCONS_00046564; exon_number 4; oId CUFF.89678.1; tss_id TSS95208;
9	Cufflinks	exon	153130492	153131037	.	-	.	gene_id GRMZM2G330019; transcript_id TCONS_00046564; exon_number 5; oId CUFF.89678.1; tss_id TSS95208;
9	Cufflinks	exon	153131550	153131623	.	-	.	gene_id GRMZM2G330019; transcript_id TCONS_00046564; exon_number 6; oId CUFF.89678.1; tss_id TSS95208;
9	Cufflinks	exon	153131794	153131914	.	-	.	gene_id GRMZM2G330019; transcript_id TCONS_00046564; exon_number 7; oId CUFF.89678.1; tss_id TSS95208;
9	Cufflinks	exon	153132010	153132095	.	-	.	gene_id GRMZM2G330019; transcript_id TCONS_00046564; exon_number 8; oId CUFF.89678.1; tss_id TSS95208;
9	Cufflinks	exon	153132484	153133217	.	-	.	gene_id GRMZM2G330019; transcript_id TCONS_00046564; exon_number 9; oId CUFF.89678.1; tss_id TSS95208;
1	Cufflinks	exon	5080787	5080992	.	+	.	gene_id GRMZM2G327059; transcript_id TCONS_00000116; exon_number 1; oId CUFF.240.1; tss_id TSS109;
1	Cufflinks	exon	5081612	5081720	.	+	.	gene_id GRMZM2G327059; transcript_id TCONS_00000116; exon_number 2; oId CUFF.240.1; tss_id TSS109;
1	Cufflinks	exon	5081860	5082635	.	+	.	gene_id GRMZM2G327059; transcript_id TCONS_00000116; exon_number 3; oId CUFF.240.1; tss_id TSS109;
1	Cufflinks	exon	5083676	5084061	.	+	.	gene_id GRMZM2G327059; transcript_id TCONS_00000116; exon_number 4; oId CUFF.240.1; tss_id TSS109;
1	Cufflinks	exon	5084895	5084955	.	+	.	gene_id GRMZM2G327059; transcript_id TCONS_00000116; exon_number 5; oId CUFF.240.1; tss_id TSS109;
1	Cufflinks	exon	5085393	5086610	.	+	.	gene_id GRMZM2G327059; transcript_id TCONS_00000116; exon_number 6; oId CUFF.240.1; tss_id TSS109;
1	Cufflinks	exon	5080788	5081720	.	+	.	gene_id GRMZM2G327059; transcript_id TCONS_00000119; exon_number 1; oId CUFF.240.4; tss_id TSS109;
1	Cufflinks	exon	5081860	5082635	.	+	.	gene_id GRMZM2G327059; transcript_id TCONS_00000119; exon_number 2; oId CUFF.240.4; tss_id TSS109;
1	Cufflinks	exon	5083676	5084061	.	+	.	gene_id GRMZM2G327059; transcript_id TCONS_00000119; exon_number 3; oId CUFF.240.4; tss_id TSS109;
1	Cufflinks	exon	5084895	5084955	.	+	.	gene_id GRMZM2G327059; transcript_id TCONS_00000119; exon_number 4; oId CUFF.240.4; tss_id TSS109;
1	Cufflinks	exon	5085393	5086610	.	+	.	gene_id GRMZM2G327059; transcript_id TCONS_00000119; exon_number 5; oId CUFF.240.4; tss_id TSS109;
1	Cufflinks	exon	17519366	17520530	.	+	.	gene_id XLOC_000312; transcript_id TCONS_00000364; exon_number 1; oId CUFF.592.1; tss_id TSS324;
1	Cufflinks	exon	18984003	18985420	.	+	.	gene_id XLOC_000340; transcript_id TCONS_00000395; exon_number 1; oId CUFF.633.1; tss_id TSS353;
1	Cufflinks	exon	39144595	39145057	.	+	.	gene_id XLOC_000585; transcript_id TCONS_00000680; exon_number 1; oId CUFF.1118.1; tss_id TSS609;
1	Cufflinks	exon	39145209	39145544	.	+	.	gene_id XLOC_000585; transcript_id TCONS_00000680; exon_number 2; oId CUFF.1118.1; tss_id TSS609;
1	Cufflinks	exon	39145644	39146126	.	+	.	gene_id XLOC_000585; transcript_id TCONS_00000680; exon_number 3; oId CUFF.1118.1; tss_id TSS609;

1	Cufflinks	exon	42849835	42854421	.	+	.	gene_id GRMZM2G442277; transcript_id TCONS_00000740; exon_number 1; oId CUFF.1226.1; tss_id TSS664;
1	Cufflinks	exon	46236163	46237825	.	+	.	gene_id XLOC_000689; transcript_id TCONS_00000798; exon_number 1; oId CUFF.1309.1; tss_id TSS717;
1	Cufflinks	exon	51892116	51893672	.	+	.	gene_id GRMZM2G318652; transcript_id TCONS_00000874; exon_number 1; oId CUFF.1430.1; tss_id TSS785;
1	Cufflinks	exon	62678621	62679843	.	+	.	gene_id GRMZM2G168665; transcript_id TCONS_00001011; exon_number 1; oId CUFF.1682.1; tss_id TSS909;
1	Cufflinks	exon	64021299	64021898	.	+	.	gene_id XLOC_000900; transcript_id TCONS_00001039; exon_number 1; oId CUFF.1727.1; tss_id TSS936;
1	Cufflinks	exon	69295636	69297279	.	+	.	gene_id GRMZM2G049070; transcript_id TCONS_00001119; exon_number 1; oId CUFF.1810.1; tss_id TSS1001;
1	Cufflinks	exon	70779206	70780258	.	+	.	gene_id XLOC_000969; transcript_id TCONS_00001129; exon_number 1; oId CUFF.1837.1; tss_id TSS1011;
1	Cufflinks	exon	78064712	78065532	.	+	.	gene_id XLOC_001037; transcript_id TCONS_00001208; exon_number 1; oId CUFF.1982.1; tss_id TSS1080;
1	Cufflinks	exon	92248218	92248822	.	+	.	gene_id GRMZM2G114772; transcript_id TCONS_00001347; exon_number 1; oId CUFF.2220.2; tss_id TSS1207;
1	Cufflinks	exon	92473694	92474849	.	+	.	gene_id GRMZM2G094639; transcript_id TCONS_00001351; exon_number 1; oId CUFF.2218.1; tss_id TSS1210;
1	Cufflinks	exon	126123363	126124398	.	+	.	gene_id AC211467.4_FG002; transcript_id TCONS_00001514; exon_number 1; oId CUFF.2523.1; tss_id TSS1363;
1	Cufflinks	exon	151734008	151734328	.	+	.	gene_id GRMZM6G924006; transcript_id TCONS_00001614; exon_number 1; oId CUFF.2708.1; tss_id TSS1453;
1	Cufflinks	exon	151734894	151734948	.	+	.	gene_id GRMZM6G924006; transcript_id TCONS_00001614; exon_number 2; oId CUFF.2708.1; tss_id TSS1453;
1	Cufflinks	exon	151735048	151735098	.	+	.	gene_id GRMZM6G924006; transcript_id TCONS_00001614; exon_number 3; oId CUFF.2708.1; tss_id TSS1453;
1	Cufflinks	exon	151735390	151735811	.	+	.	gene_id GRMZM6G924006; transcript_id TCONS_00001614; exon_number 4; oId CUFF.2708.1; tss_id TSS1453;
1	Cufflinks	exon	151735910	151736151	.	+	.	gene_id GRMZM6G924006; transcript_id TCONS_00001614; exon_number 5; oId CUFF.2708.1; tss_id TSS1453;
1	Cufflinks	exon	175321975	175323121	.	+	.	gene_id AC212976.3_FG003; transcript_id TCONS_00001805; exon_number 1; oId CUFF.3019.1; tss_id TSS1620;
1	Cufflinks	exon	187917466	187919447	.	+	.	gene_id XLOC_001677; transcript_id TCONS_00001945; exon_number 1; oId CUFF.3268.1; tss_id TSS1748;
1	Cufflinks	exon	188493609	188494765	.	+	.	gene_id XLOC_001686; transcript_id TCONS_00001958; exon_number 1; oId CUFF.3297.1; tss_id TSS1759;
1	Cufflinks	exon	197743016	197743467	.	+	.	gene_id XLOC_001799; transcript_id TCONS_00002086; exon_number 1; oId CUFF.3475.1; tss_id TSS1877;
1	Cufflinks	exon	199871050	199872477	.	+	.	gene_id GRMZM2G104938; transcript_id TCONS_00002123; exon_number 1; oId CUFF.3547.1; tss_id TSS1912;
1	Cufflinks	exon	208873436	208874144	.	+	.	gene_id XLOC_001947; transcript_id TCONS_00002262; exon_number 1; oId CUFF.3772.1; tss_id TSS2034;
1	Cufflinks	exon	218473265	218474165	.	+	.	gene_id XLOC_002039; transcript_id TCONS_00002365; exon_number 1; oId CUFF.3974.1; tss_id TSS2128;
1	Cufflinks	exon	233875715	233878017	.	+	.	gene_id XLOC_002210; transcript_id TCONS_00002566; exon_number 1; oId CUFF.4346.1; tss_id TSS2305;
1	Cufflinks	exon	235922447	235923497	.	+	.	gene_id XLOC_002238; transcript_id TCONS_00002597; exon_number 1; oId CUFF.4417.1; tss_id TSS2335;
1	Cufflinks	exon	248481668	248482632	.	+	.	gene_id GRMZM2G153887; transcript_id TCONS_00002743; exon_number 1; oId CUFF.4709.1; tss_id TSS2468;
1	Cufflinks	exon	255803441	255804699	.	+	.	gene_id GRMZM2G082686; transcript_id TCONS_00002837; exon_number 1; oId CUFF.4800.1; tss_id TSS2550;
1	Cufflinks	exon	275694984	275696172	.	+	.	gene_id XLOC_002702; transcript_id TCONS_00003123; exon_number 1; oId CUFF.5323.1; tss_id TSS2812;
1	Cufflinks	exon	279251712	279254882	.	+	.	gene_id XLOC_002767; transcript_id TCONS_00003206; exon_number 1; oId CUFF.5455.1; tss_id TSS2882;
1	Cufflinks	exon	280846114	280846636	.	+	.	gene_id XLOC_002789; transcript_id TCONS_00003231; exon_number 1; oId CUFF.5491.1; tss_id TSS2904;
1	Cufflinks	exon	280894065	280895067	.	+	.	gene_id GRMZM2G072814; transcript_id TCONS_00003232; exon_number 1; oId CUFF.5498.1; tss_id TSS2905;
1	Cufflinks	exon	281977000	281979054	.	+	.	gene_id XLOC_002804; transcript_id TCONS_00003247; exon_number 1; oId CUFF.5541.1; tss_id TSS2919;
1	Cufflinks	exon	282015389	282016975	.	+	.	gene_id XLOC_002805; transcript_id TCONS_00003248; exon_number 1; oId CUFF.5533.1; tss_id TSS2920;
1	Cufflinks	exon	285410613	285412021	.	+	.	gene_id XLOC_002841; transcript_id TCONS_00003286; exon_number 1; oId CUFF.5624.1; tss_id TSS2956;
1	Cufflinks	exon	287360747	287361598	.	+	.	gene_id XLOC_002877; transcript_id TCONS_00003326; exon_number 1; oId CUFF.5718.1; tss_id TSS2992;
1	Cufflinks	exon	293724242	293724323	.	+	.	gene_id GRMZM2G068443; transcript_id TCONS_00003448; exon_number 1; oId CUFF.5928.2; tss_id TSS3103;

1	Cufflinks	exon	293724410	293724496	.	+	.	gene_id GRMZM2G068443; transcript_id TCONS_00003448; exon_number 2; oId CUFF.5928.2; tss_id TSS3103;
1	Cufflinks	exon	293724609	293724671	.	+	.	gene_id GRMZM2G068443; transcript_id TCONS_00003448; exon_number 3; oId CUFF.5928.2; tss_id TSS3103;
1	Cufflinks	exon	293724763	293725893	.	+	.	gene_id GRMZM2G068443; transcript_id TCONS_00003448; exon_number 4; oId CUFF.5928.2; tss_id TSS3103;
1	Cufflinks	exon	293726038	293726484	.	+	.	gene_id GRMZM2G068443; transcript_id TCONS_00003448; exon_number 5; oId CUFF.5928.2; tss_id TSS3103;
1	Cufflinks	exon	2839365	2840264	.	-	.	gene_id XLOC_003174; transcript_id TCONS_00003675; exon_number 1; oId CUFF.84.1; tss_id TSS3305;
1	Cufflinks	exon	3127903	3129982	.	-	.	gene_id XLOC_003179; transcript_id TCONS_00003680; exon_number 1; oId CUFF.102.1; tss_id TSS3310;
1	Cufflinks	exon	5038799	5040058	.	-	.	gene_id XLOC_003216; transcript_id TCONS_00003724; exon_number 1; oId CUFF.191.1; tss_id TSS3348;
1	Cufflinks	exon	8025460	8025864	.	-	.	gene_id XLOC_003278; transcript_id TCONS_00003797; exon_number 1; oId CUFF.306.1; tss_id TSS3413;
1	Cufflinks	exon	8093433	8097320	.	-	.	gene_id GRMZM2G176523; transcript_id TCONS_00003799; exon_number 1; oId CUFF.312.1; tss_id TSS3415;
1	Cufflinks	exon	17518667	17520030	.	-	.	gene_id AC206951.3_FG016; transcript_id TCONS_00003950; exon_number 1; oId CUFF.591.1; tss_id TSS3548;
1	Cufflinks	exon	18983737	18985312	.	-	.	gene_id GRMZM2G148706; transcript_id TCONS_00003967; exon_number 1; oId CUFF.632.1; tss_id TSS3564;
1	Cufflinks	exon	29400429	29401250	.	-	.	gene_id XLOC_003535; transcript_id TCONS_00004094; exon_number 1; oId CUFF.881.1; tss_id TSS3678;
1	Cufflinks	exon	46236692	46238160	.	-	.	gene_id GRMZM2G144224; transcript_id TCONS_00004340; exon_number 1; oId CUFF.1311.1; tss_id TSS3896;
1	Cufflinks	exon	51891410	51893253	.	-	.	gene_id XLOC_003796; transcript_id TCONS_00004401; exon_number 1; oId CUFF.1429.1; tss_id TSS3950;
1	Cufflinks	exon	62678619	62679687	.	-	.	gene_id XLOC_003932; transcript_id TCONS_00004560; exon_number 1; oId CUFF.1681.1; tss_id TSS4091;
1	Cufflinks	exon	62719153	62720477	.	-	.	gene_id XLOC_003934; transcript_id TCONS_00004562; exon_number 1; oId CUFF.1684.1; tss_id TSS4093;
1	Cufflinks	exon	67780993	67781504	.	-	.	gene_id XLOC_003969; transcript_id TCONS_00004605; exon_number 1; oId CUFF.1830.1; tss_id TSS4128;
1	Cufflinks	exon	69295619	69296188	.	-	.	gene_id XLOC_003979; transcript_id TCONS_00004616; exon_number 1; oId CUFF.1809.1; tss_id TSS4139;
1	Cufflinks	exon	79336553	79338285	.	-	.	gene_id XLOC_004082; transcript_id TCONS_00004730; exon_number 1; oId CUFF.2004.1; tss_id TSS4243;
1	Cufflinks	exon	85029194	85029701	.	-	.	gene_id XLOC_004131; transcript_id TCONS_00004786; exon_number 1; oId CUFF.2116.1; tss_id TSS4293;
1	Cufflinks	exon	89004822	89006240	.	-	.	gene_id XLOC_004165; transcript_id TCONS_00004825; exon_number 1; oId CUFF.2177.1; tss_id TSS4329;
1	Cufflinks	exon	139800143	139801008	.	-	.	gene_id GRMZM2G167649; transcript_id TCONS_00005061; exon_number 1; oId CUFF.2601.1; tss_id TSS4549;
1	Cufflinks	exon	147170825	147172007	.	-	.	gene_id XLOC_004417; transcript_id TCONS_00005102; exon_number 1; oId CUFF.2659.1; tss_id TSS4587;
1	Cufflinks	exon	147208726	147209953	.	-	.	gene_id GRMZM2G006704; transcript_id TCONS_00005103; exon_number 1; oId CUFF.2663.1; tss_id TSS4588;
1	Cufflinks	exon	151749886	151751089	.	-	.	gene_id XLOC_004442; transcript_id TCONS_00005133; exon_number 1; oId CUFF.2722.1; tss_id TSS4613;
1	Cufflinks	exon	166942218	166945246	.	-	.	gene_id XLOC_004512; transcript_id TCONS_00005203; exon_number 1; oId CUFF.2882.1; tss_id TSS4683;
1	Cufflinks	exon	187378034	187378819	.	-	.	gene_id AC213857.4_FG003; transcript_id TCONS_00005420; exon_number 1; oId CUFF.3288.1; tss_id TSS4884;
1	Cufflinks	exon	187917195	187919314	.	-	.	gene_id GRMZM2G121312; transcript_id TCONS_00005426; exon_number 1; oId CUFF.3267.1; tss_id TSS4890;
1	Cufflinks	exon	195218610	195219235	.	-	.	gene_id XLOC_004778; transcript_id TCONS_00005502; exon_number 1; oId CUFF.3430.1; tss_id TSS4957;
1	Cufflinks	exon	201668928	201669258	.	-	.	gene_id XLOC_004853; transcript_id TCONS_00005582; exon_number 1; oId CUFF.3584.1; tss_id TSS5034;
1	Cufflinks	exon	204909826	204911008	.	-	.	gene_id GRMZM2G320708; transcript_id TCONS_00005625; exon_number 1; oId CUFF.3674.1; tss_id TSS5072;
1	Cufflinks	exon	217332510	217332972	.	-	.	gene_id XLOC_005026; transcript_id TCONS_00005787; exon_number 1; oId CUFF.3926.1; tss_id TSS5217;
1	Cufflinks	exon	219952350	219953886	.	-	.	gene_id GRMZM2G321010; transcript_id TCONS_00005819; exon_number 1; oId CUFF.3998.1; tss_id TSS5246;
1	Cufflinks	exon	224090231	224091493	.	-	.	gene_id XLOC_005098; transcript_id TCONS_00005878; exon_number 1; oId CUFF.4086.1; tss_id TSS5294;
1	Cufflinks	exon	224436921	224438023	.	-	.	gene_id XLOC_005105; transcript_id TCONS_00005886; exon_number 1; oId CUFF.4101.1; tss_id TSS5301;
1	Cufflinks	exon	224484800	224485876	.	-	.	gene_id GRMZM2G475956; transcript_id TCONS_00005888; exon_number 1; oId CUFF.4116.1; tss_id TSS5303;

1	Cufflinks	exon	226230644	226231367	.	-	.	gene_id XLOC_005127; transcript_id TCONS_00005910; exon_number 1; oId CUFF.4149.1; tss_id TSS5323;
1	Cufflinks	exon	226822640	226823502	.	-	.	gene_id XLOC_005148; transcript_id TCONS_00005937; exon_number 1; oId CUFF.4172.1; tss_id TSS5344;
1	Cufflinks	exon	234403212	234403769	.	-	.	gene_id XLOC_005260; transcript_id TCONS_00006074; exon_number 1; oId CUFF.4368.1; tss_id TSS5462;
1	Cufflinks	exon	235420172	235420870	.	-	.	gene_id XLOC_005283; transcript_id TCONS_00006097; exon_number 1; oId CUFF.4404.1; tss_id TSS5485;
1	Cufflinks	exon	253775607	253776040	.	-	.	gene_id XLOC_005451; transcript_id TCONS_00006290; exon_number 1; oId CUFF.4763.1; tss_id TSS5657;
1	Cufflinks	exon	255803514	255804904	.	-	.	gene_id XLOC_005475; transcript_id TCONS_00006318; exon_number 1; oId CUFF.4801.1; tss_id TSS5682;
1	Cufflinks	exon	270898020	270899100	.	-	.	gene_id GRMZM2G026780; transcript_id TCONS_00006531; exon_number 1; oId CUFF.5178.1; tss_id TSS5878;
1	Cufflinks	exon	280845925	280846624	.	-	.	gene_id XLOC_005828; transcript_id TCONS_00006708; exon_number 1; oId CUFF.5490.1; tss_id TSS6040;
1	Cufflinks	exon	280894123	280894576	.	-	.	gene_id XLOC_005831; transcript_id TCONS_00006711; exon_number 1; oId CUFF.5496.1; tss_id TSS6043;
1	Cufflinks	exon	282015181	282016843	.	-	.	gene_id GRMZM2G165709; transcript_id TCONS_00006730; exon_number 1; oId CUFF.5532.1; tss_id TSS6062;
1	Cufflinks	exon	295100872	295101411	.	-	.	gene_id XLOC_006076; transcript_id TCONS_00006979; exon_number 1; oId CUFF.5964.1; tss_id TSS6294;
1	Cufflinks	exon	296789724	296790547	.	-	.	gene_id GRMZM5G883969; transcript_id TCONS_00007015; exon_number 1; oId CUFF.6022.1; tss_id TSS6324;
1	Cufflinks	exon	297095291	297096113	.	-	.	gene_id GRMZM2G352415; transcript_id TCONS_00007030; exon_number 1; oId CUFF.6058.2; tss_id TSS6337;
1	Cufflinks	exon	297097285	297098257	.	-	.	gene_id GRMZM2G352415; transcript_id TCONS_00007030; exon_number 2; oId CUFF.6058.2; tss_id TSS6337;
1	Cufflinks	exon	297098334	297098628	.	-	.	gene_id GRMZM2G352415; transcript_id TCONS_00007030; exon_number 3; oId CUFF.6058.2; tss_id TSS6337;
10	Cufflinks	exon	1083998	1085635	.	+	.	gene_id GRMZM2G066142; transcript_id TCONS_00007123; exon_number 1; oId CUFF.6203.1; tss_id TSS6424;
10	Cufflinks	exon	3176422	3177326	.	+	.	gene_id GRMZM2G058481; transcript_id TCONS_00007172; exon_number 1; oId CUFF.6320.1; tss_id TSS6471;
10	Cufflinks	exon	6533858	6535307	.	+	.	gene_id AC202107.3_FG001; transcript_id TCONS_00007230; exon_number 1; oId CUFF.6468.1; tss_id TSS6527;
10	Cufflinks	exon	49989787	49992026	.	+	.	gene_id XLOC_006523; transcript_id TCONS_00007476; exon_number 1; oId CUFF.6950.1; tss_id TSS6755;
10	Cufflinks	exon	78074137	78076764	.	+	.	gene_id GRMZM2G078906; transcript_id TCONS_00007644; exon_number 1; oId CUFF.7248.1; tss_id TSS6906;
10	Cufflinks	exon	79422812	79423830	.	+	.	gene_id XLOC_006681; transcript_id TCONS_00007655; exon_number 1; oId CUFF.7272.1; tss_id TSS6916;
10	Cufflinks	exon	79424059	79424883	.	+	.	gene_id XLOC_006682; transcript_id TCONS_00007656; exon_number 1; oId CUFF.7268.1; tss_id TSS6917;
10	Cufflinks	exon	95139127	95139940	.	+	.	gene_id XLOC_006835; transcript_id TCONS_00007826; exon_number 1; oId CUFF.7554.1; tss_id TSS7073;
10	Cufflinks	exon	131913905	131914803	.	+	.	gene_id XLOC_007189; transcript_id TCONS_00008227; exon_number 1; oId CUFF.8267.1; tss_id TSS7434;
10	Cufflinks	exon	146078469	146080245	.	+	.	gene_id GRMZM2G067424; transcript_id TCONS_00008558; exon_number 1; oId CUFF.8799.1; tss_id TSS7731;
10	Cufflinks	exon	149246609	149248076	.	+	.	gene_id XLOC_007556; transcript_id TCONS_00008644; exon_number 1; oId CUFF.9004.1; tss_id TSS7814;
10	Cufflinks	exon	1950526	1951117	.	-	.	gene_id GRMZM2G043150; transcript_id TCONS_00008671; exon_number 1; oId CUFF.6260.2; tss_id TSS7838;
10	Cufflinks	exon	1951648	1951727	.	-	.	gene_id GRMZM2G043150; transcript_id TCONS_00008671; exon_number 2; oId CUFF.6260.2; tss_id TSS7838;
10	Cufflinks	exon	1956973	1957196	.	-	.	gene_id GRMZM2G043150; transcript_id TCONS_00008671; exon_number 3; oId CUFF.6260.2; tss_id TSS7838;
10	Cufflinks	exon	1957285	1957489	.	-	.	gene_id GRMZM2G043150; transcript_id TCONS_00008671; exon_number 4; oId CUFF.6260.2; tss_id TSS7838;
10	Cufflinks	exon	1957572	1957745	.	-	.	gene_id GRMZM2G043150; transcript_id TCONS_00008671; exon_number 5; oId CUFF.6260.2; tss_id TSS7838;
10	Cufflinks	exon	1957832	1957972	.	-	.	gene_id GRMZM2G043150; transcript_id TCONS_00008671; exon_number 6; oId CUFF.6260.2; tss_id TSS7838;
10	Cufflinks	exon	1958554	1958658	.	-	.	gene_id GRMZM2G043150; transcript_id TCONS_00008671; exon_number 7; oId CUFF.6260.2; tss_id TSS7838;
10	Cufflinks	exon	1958746	1959838	.	-	.	gene_id GRMZM2G043150; transcript_id TCONS_00008671; exon_number 8; oId CUFF.6260.2; tss_id TSS7838;
10	Cufflinks	exon	4529937	4530266	.	-	.	gene_id GRMZM2G054481; transcript_id TCONS_00008753; exon_number 1; oId CUFF.6382.1; tss_id TSS7916;
10	Cufflinks	exon	4530349	4530570	.	-	.	gene_id GRMZM2G054481; transcript_id TCONS_00008753; exon_number 2; oId CUFF.6382.1; tss_id TSS7916;

10	Cufflinks	exon	4531070	4531806	.	-	.	gene_id GRMZM2G054481; transcript_id TCONS_00008753; exon_number 3; oId CUFF.6382.1; tss_id TSS7916;
10	Cufflinks	exon	5226560	5227949	.	-	.	gene_id XLOC_007686; transcript_id TCONS_00008788; exon_number 1; oId CUFF.6428.1; tss_id TSS7945;
10	Cufflinks	exon	6402289	6402853	.	-	.	gene_id XLOC_007707; transcript_id TCONS_00008810; exon_number 1; oId CUFF.6460.1; tss_id TSS7966;
10	Cufflinks	exon	17614876	17615435	.	-	.	gene_id XLOC_007808; transcript_id TCONS_00008923; exon_number 1; oId CUFF.6647.1; tss_id TSS8070;
10	Cufflinks	exon	35929743	35930125	.	-	.	gene_id XLOC_007917; transcript_id TCONS_00009052; exon_number 1; oId CUFF.6836.1; tss_id TSS8187;
10	Cufflinks	exon	78074671	78075747	.	-	.	gene_id XLOC_008126; transcript_id TCONS_00009279; exon_number 1; oId CUFF.7245.1; tss_id TSS8398;
10	Cufflinks	exon	86998117	87000393	.	-	.	gene_id XLOC_008201; transcript_id TCONS_00009360; exon_number 1; oId CUFF.7422.2; tss_id TSS8475;
10	Cufflinks	exon	87283734	87284881	.	-	.	gene_id GRMZM2G015605; transcript_id TCONS_00009369; exon_number 1; oId CUFF.7432.1; tss_id TSS8484;
10	Cufflinks	exon	100699105	100699594	.	-	.	gene_id XLOC_008322; transcript_id TCONS_00009492; exon_number 1; oId CUFF.7647.1; tss_id TSS8599;
10	Cufflinks	exon	126160350	126160680	.	-	.	gene_id XLOC_008552; transcript_id TCONS_00009762; exon_number 1; oId CUFF.8123.1; tss_id TSS8838;
10	Cufflinks	exon	128580332	128582775	.	-	.	gene_id GRMZM2G000261; transcript_id TCONS_00009803; exon_number 1; oId CUFF.8179.1; tss_id TSS8865;
10	Cufflinks	exon	134829905	134830675	.	-	.	gene_id XLOC_008661; transcript_id TCONS_00009898; exon_number 1; oId CUFF.8371.1; tss_id TSS8948;
10	Cufflinks	exon	143743661	143744585	.	-	.	gene_id XLOC_008830; transcript_id TCONS_00010092; exon_number 1; oId CUFF.8699.1; tss_id TSS9125;
10	Cufflinks	exon	146078896	146080074	.	-	.	gene_id XLOC_008876; transcript_id TCONS_00010149; exon_number 1; oId CUFF.8800.1; tss_id TSS9171;
2	Cufflinks	exon	177859	179378	.	+	.	gene_id XLOC_008977; transcript_id TCONS_00010272; exon_number 1; oId CUFF.8996.1; tss_id TSS9275;
2	Cufflinks	exon	529436	529968	.	+	.	gene_id XLOC_008979; transcript_id TCONS_00010274; exon_number 1; oId CUFF.9000.1; tss_id TSS9277;
2	Cufflinks	exon	16654335	16656671	.	+	.	gene_id XLOC_009380; transcript_id TCONS_00010737; exon_number 1; oId CUFF.9735.1; tss_id TSS9690;
2	Cufflinks	exon	33977709	33979429	.	+	.	gene_id XLOC_009621; transcript_id TCONS_00011021; exon_number 1; oId CUFF.10219.1; tss_id TSS9944;
2	Cufflinks	exon	43413441	43413641	.	+	.	gene_id GRMZM2G065073; transcript_id TCONS_00011145; exon_number 1; oId CUFF.10444.1; tss_id TSS10052;
2	Cufflinks	exon	43413739	43414097	.	+	.	gene_id GRMZM2G065073; transcript_id TCONS_00011145; exon_number 2; oId CUFF.10444.1; tss_id TSS10052;
2	Cufflinks	exon	43418941	43419456	.	+	.	gene_id GRMZM2G065073; transcript_id TCONS_00011145; exon_number 3; oId CUFF.10444.1; tss_id TSS10052;
2	Cufflinks	exon	43419544	43419779	.	+	.	gene_id GRMZM2G065073; transcript_id TCONS_00011145; exon_number 4; oId CUFF.10444.1; tss_id TSS10052;
2	Cufflinks	exon	43419956	43420220	.	+	.	gene_id GRMZM2G065073; transcript_id TCONS_00011145; exon_number 5; oId CUFF.10444.1; tss_id TSS10052;
2	Cufflinks	exon	43420323	43420460	.	+	.	gene_id GRMZM2G065073; transcript_id TCONS_00011145; exon_number 6; oId CUFF.10444.1; tss_id TSS10052;
2	Cufflinks	exon	43420589	43421006	.	+	.	gene_id GRMZM2G065073; transcript_id TCONS_00011145; exon_number 7; oId CUFF.10444.1; tss_id TSS10052;
2	Cufflinks	exon	51868677	51870074	.	+	.	gene_id XLOC_009826; transcript_id TCONS_00011254; exon_number 1; oId CUFF.10625.1; tss_id TSS10154;
2	Cufflinks	exon	69807982	69808578	.	+	.	gene_id XLOC_009935; transcript_id TCONS_00011377; exon_number 1; oId CUFF.10861.1; tss_id TSS10265;
2	Cufflinks	exon	104803852	104806393	.	+	.	gene_id GRMZM2G172695; transcript_id TCONS_00011524; exon_number 1; oId CUFF.11142.1; tss_id TSS10403;
2	Cufflinks	exon	130329090	130329738	.	+	.	gene_id XLOC_010183; transcript_id TCONS_00011653; exon_number 1; oId CUFF.11406.1; tss_id TSS10517;
2	Cufflinks	exon	130778699	130779350	.	+	.	gene_id XLOC_010187; transcript_id TCONS_00011657; exon_number 1; oId CUFF.11415.1; tss_id TSS10521;
2	Cufflinks	exon	143678133	143679858	.	+	.	gene_id GRMZM2G348855; transcript_id TCONS_00011725; exon_number 1; oId CUFF.11547.1; tss_id TSS10581;
2	Cufflinks	exon	144155245	144156375	.	+	.	gene_id GRMZM2G450044; transcript_id TCONS_00011735; exon_number 1; oId CUFF.11535.1; tss_id TSS10588;
2	Cufflinks	exon	178377065	178378920	.	+	.	gene_id GRMZM2G340933; transcript_id TCONS_00012048; exon_number 1; oId CUFF.12075.1; tss_id TSS10869;
2	Cufflinks	exon	181475801	181477592	.	+	.	gene_id GRMZM2G359521; transcript_id TCONS_00012077; exon_number 1; oId CUFF.12136.1; tss_id TSS10895;
2	Cufflinks	exon	183946317	183947096	.	+	.	gene_id XLOC_010571; transcript_id TCONS_00012097; exon_number 1; oId CUFF.12164.1; tss_id TSS10915;
2	Cufflinks	exon	189550546	189551309	.	+	.	gene_id XLOC_010643; transcript_id TCONS_00012178; exon_number 1; oId CUFF.12315.1; tss_id TSS10991;

2	Cufflinks	exon	190588589	190589744	.	+	.	gene_id XLOC_010664; transcript_id TCONS_00012202; exon_number 1; oId CUFF.12354.1; tss_id TSS11012;
2	Cufflinks	exon	194492414	194494026	.	+	.	gene_id XLOC_010713; transcript_id TCONS_00012258; exon_number 1; oId CUFF.12446.1; tss_id TSS11062;
2	Cufflinks	exon	214245985	214246562	.	+	.	gene_id XLOC_011054; transcript_id TCONS_00012641; exon_number 1; oId CUFF.13075.1; tss_id TSS11411;
2	Cufflinks	exon	215926301	215927938	.	+	.	gene_id XLOC_011092; transcript_id TCONS_00012683; exon_number 1; oId CUFF.13139.1; tss_id TSS11451;
2	Cufflinks	exon	217520351	217522200	.	+	.	gene_id GRMZM2G308752; transcript_id TCONS_00012722; exon_number 1; oId CUFF.13215.1; tss_id TSS11488;
2	Cufflinks	exon	177675	179550	.	-	.	gene_id XLOC_011451; transcript_id TCONS_00013070; exon_number 1; oId CUFF.8995.1; tss_id TSS11816;
2	Cufflinks	exon	179732	179791	.	-	.	gene_id XLOC_011451; transcript_id TCONS_00013070; exon_number 2; oId CUFF.8995.1; tss_id TSS11816;
2	Cufflinks	exon	3230346	3230889	.	-	.	gene_id XLOC_011519; transcript_id TCONS_00013155; exon_number 1; oId CUFF.9158.1; tss_id TSS11886;
2	Cufflinks	exon	4194864	4195911	.	-	.	gene_id XLOC_011539; transcript_id TCONS_00013181; exon_number 1; oId CUFF.9206.1; tss_id TSS11906;
2	Cufflinks	exon	9126849	9127558	.	-	.	gene_id XLOC_011646; transcript_id TCONS_00013306; exon_number 1; oId CUFF.9485.1; tss_id TSS12016;
2	Cufflinks	exon	10387374	10389429	.	-	.	gene_id XLOC_011679; transcript_id TCONS_00013345; exon_number 1; oId CUFF.9500.1; tss_id TSS12051;
2	Cufflinks	exon	11769111	11771067	.	-	.	gene_id XLOC_011714; transcript_id TCONS_00013383; exon_number 1; oId CUFF.9559.1; tss_id TSS12088;
2	Cufflinks	exon	21891777	21892731	.	-	.	gene_id XLOC_011854; transcript_id TCONS_00013542; exon_number 1; oId CUFF.9879.1; tss_id TSS12233;
2	Cufflinks	exon	22220989	22221632	.	-	.	gene_id XLOC_011863; transcript_id TCONS_00013555; exon_number 1; oId CUFF.9886.1; tss_id TSS12244;
2	Cufflinks	exon	45069171	45069681	.	-	.	gene_id GRMZM2G127139; transcript_id TCONS_00013905; exon_number 1; oId CUFF.10502.3; tss_id TSS12564;
2	Cufflinks	exon	45069825	45069912	.	-	.	gene_id GRMZM2G127139; transcript_id TCONS_00013905; exon_number 2; oId CUFF.10502.3; tss_id TSS12564;
2	Cufflinks	exon	45070002	45070090	.	-	.	gene_id GRMZM2G127139; transcript_id TCONS_00013905; exon_number 3; oId CUFF.10502.3; tss_id TSS12564;
2	Cufflinks	exon	45070208	45070262	.	-	.	gene_id GRMZM2G127139; transcript_id TCONS_00013905; exon_number 4; oId CUFF.10502.3; tss_id TSS12564;
2	Cufflinks	exon	45070348	45070437	.	-	.	gene_id GRMZM2G127139; transcript_id TCONS_00013905; exon_number 5; oId CUFF.10502.3; tss_id TSS12564;
2	Cufflinks	exon	45070548	45070612	.	-	.	gene_id GRMZM2G127139; transcript_id TCONS_00013905; exon_number 6; oId CUFF.10502.3; tss_id TSS12564;
2	Cufflinks	exon	45070690	45070814	.	-	.	gene_id GRMZM2G127139; transcript_id TCONS_00013905; exon_number 7; oId CUFF.10502.3; tss_id TSS12564;
2	Cufflinks	exon	45070912	45071015	.	-	.	gene_id GRMZM2G127139; transcript_id TCONS_00013905; exon_number 8; oId CUFF.10502.3; tss_id TSS12564;
2	Cufflinks	exon	45071248	45071362	.	-	.	gene_id GRMZM2G127139; transcript_id TCONS_00013905; exon_number 9; oId CUFF.10502.3; tss_id TSS12564;
2	Cufflinks	exon	45071571	45071662	.	-	.	gene_id GRMZM2G127139; transcript_id TCONS_00013905; exon_number 10; oId CUFF.10502.3; tss_id TSS12564;
2	Cufflinks	exon	45071751	45071962	.	-	.	gene_id GRMZM2G127139; transcript_id TCONS_00013905; exon_number 11; oId CUFF.10502.3; tss_id TSS12564;
2	Cufflinks	exon	45072092	45072207	.	-	.	gene_id GRMZM2G127139; transcript_id TCONS_00013905; exon_number 12; oId CUFF.10502.3; tss_id TSS12564;
2	Cufflinks	exon	45072304	45072404	.	-	.	gene_id GRMZM2G127139; transcript_id TCONS_00013905; exon_number 13; oId CUFF.10502.3; tss_id TSS12564;
2	Cufflinks	exon	45072484	45072558	.	-	.	gene_id GRMZM2G127139; transcript_id TCONS_00013905; exon_number 14; oId CUFF.10502.3; tss_id TSS12564;
2	Cufflinks	exon	45072662	45072824	.	-	.	gene_id GRMZM2G127139; transcript_id TCONS_00013905; exon_number 15; oId CUFF.10502.3; tss_id TSS12564;
2	Cufflinks	exon	45073279	45074202	.	-	.	gene_id GRMZM2G127139; transcript_id TCONS_00013905; exon_number 16; oId CUFF.10502.3; tss_id TSS12564;
2	Cufflinks	exon	113942348	113943804	.	-	.	gene_id XLOC_012567; transcript_id TCONS_00014352; exon_number 1; oId CUFF.11257.1; tss_id TSS12971;
2	Cufflinks	exon	130328542	130329737	.	-	.	gene_id GRMZM2G143397; transcript_id TCONS_00014447; exon_number 1; oId CUFF.11405.1; tss_id TSS13062;
2	Cufflinks	exon	130778406	130779394	.	-	.	gene_id GRMZM2G164064; transcript_id TCONS_00014452; exon_number 1; oId CUFF.11414.1; tss_id TSS13067;
2	Cufflinks	exon	140149765	140151440	.	-	.	gene_id XLOC_012696; transcript_id TCONS_00014486; exon_number 1; oId CUFF.11499.1; tss_id TSS13100;
2	Cufflinks	exon	142057146	142058616	.	-	.	gene_id XLOC_012711; transcript_id TCONS_00014505; exon_number 1; oId CUFF.11518.1; tss_id TSS13117;
2	Cufflinks	exon	144155308	144156067	.	-	.	gene_id XLOC_012719; transcript_id TCONS_00014513; exon_number 1; oId CUFF.11536.1; tss_id TSS13125;

2	Cufflinks	exon	178376981	178378697	.	-	.	gene_id XLOC_012981; transcript_id TCONS_00014799; exon_number 1; oId CUFF.12074.1; tss_id TSS13392;
2	Cufflinks	exon	186829842	186830387	.	-	.	gene_id XLOC_013076; transcript_id TCONS_00014902; exon_number 1; oId CUFF.12249.1; tss_id TSS13488;
2	Cufflinks	exon	190221109	190222380	.	-	.	gene_id XLOC_013120; transcript_id TCONS_00014950; exon_number 1; oId CUFF.12345.1; tss_id TSS13534;
2	Cufflinks	exon	194155565	194156945	.	-	.	gene_id XLOC_013165; transcript_id TCONS_00015004; exon_number 1; oId CUFF.12432.1; tss_id TSS13579;
2	Cufflinks	exon	194491687	194493980	.	-	.	gene_id GRMZM2G170253; transcript_id TCONS_00015009; exon_number 1; oId CUFF.12445.1; tss_id TSS13583;
2	Cufflinks	exon	198426229	198427884	.	-	.	gene_id AC233910.1_FG008; transcript_id TCONS_00015071; exon_number 1; oId CUFF.12545.1; tss_id TSS13635;
2	Cufflinks	exon	199230719	199231132	.	-	.	gene_id GRMZM2G081790; transcript_id TCONS_00015087; exon_number 1; oId CUFF.12604.1; tss_id TSS13648;
2	Cufflinks	exon	199231258	199232772	.	-	.	gene_id GRMZM2G081790; transcript_id TCONS_00015087; exon_number 2; oId CUFF.12604.1; tss_id TSS13648;
2	Cufflinks	exon	199233698	199233993	.	-	.	gene_id GRMZM2G081790; transcript_id TCONS_00015087; exon_number 3; oId CUFF.12604.1; tss_id TSS13648;
2	Cufflinks	exon	206709530	206711963	.	-	.	gene_id GRMZM2G060271; transcript_id TCONS_00015222; exon_number 1; oId CUFF.12831.1; tss_id TSS13769;
2	Cufflinks	exon	215752840	215753423	.	-	.	gene_id XLOC_013486; transcript_id TCONS_00015376; exon_number 1; oId CUFF.13136.1; tss_id TSS13910;
2	Cufflinks	exon	215925940	215926278	.	-	.	gene_id XLOC_013491; transcript_id TCONS_00015384; exon_number 1; oId CUFF.13138.1; tss_id TSS13917;
2	Cufflinks	exon	215926302	215927828	.	-	.	gene_id XLOC_013491; transcript_id TCONS_00015384; exon_number 2; oId CUFF.13138.1; tss_id TSS13917;
2	Cufflinks	exon	221286814	221287471	.	-	.	gene_id XLOC_013567; transcript_id TCONS_00015467; exon_number 1; oId CUFF.13325.1; tss_id TSS13994;
2	Cufflinks	exon	224404275	224405053	.	-	.	gene_id XLOC_013593; transcript_id TCONS_00015494; exon_number 1; oId CUFF.13391.1; tss_id TSS14020;
2	Cufflinks	exon	225012370	225012954	.	-	.	gene_id XLOC_013606; transcript_id TCONS_00015510; exon_number 1; oId CUFF.13436.1; tss_id TSS14034;
2	Cufflinks	exon	234413745	234415092	.	-	.	gene_id XLOC_013747; transcript_id TCONS_00015674; exon_number 1; oId CUFF.13713.1; tss_id TSS14185;
3	Cufflinks	exon	4344330	4345391	.	+	.	gene_id GRMZM2G045330; transcript_id TCONS_00015814; exon_number 1; oId CUFF.13972.1; tss_id TSS14313;
3	Cufflinks	exon	7966853	7968441	.	+	.	gene_id XLOC_013911; transcript_id TCONS_00015862; exon_number 1; oId CUFF.14073.1; tss_id TSS14357;
3	Cufflinks	exon	9855605	9858253	.	+	.	gene_id GRMZM2G305900; transcript_id TCONS_00015925; exon_number 1; oId CUFF.14157.1; tss_id TSS14404;
3	Cufflinks	exon	13278182	13279001	.	+	.	gene_id GRMZM2G021410; transcript_id TCONS_00015988; exon_number 1; oId CUFF.14242.1; tss_id TSS14460;
3	Cufflinks	exon	13280250	13280339	.	+	.	gene_id GRMZM2G021410; transcript_id TCONS_00015988; exon_number 2; oId CUFF.14242.1; tss_id TSS14460;
3	Cufflinks	exon	13280431	13280498	.	+	.	gene_id GRMZM2G021410; transcript_id TCONS_00015988; exon_number 3; oId CUFF.14242.1; tss_id TSS14460;
3	Cufflinks	exon	13280582	13280956	.	+	.	gene_id GRMZM2G021410; transcript_id TCONS_00015988; exon_number 4; oId CUFF.14242.1; tss_id TSS14460;
3	Cufflinks	exon	48496982	48497546	.	+	.	gene_id XLOC_014298; transcript_id TCONS_00016302; exon_number 1; oId CUFF.14853.1; tss_id TSS14752;
3	Cufflinks	exon	93102813	93104274	.	+	.	gene_id XLOC_014507; transcript_id TCONS_00016543; exon_number 1; oId CUFF.15261.1; tss_id TSS14965;
3	Cufflinks	exon	128958274	128959485	.	+	.	gene_id XLOC_014656; transcript_id TCONS_00016714; exon_number 1; oId CUFF.15612.1; tss_id TSS15121;
3	Cufflinks	exon	147559407	147561340	.	+	.	gene_id GRMZM2G102056; transcript_id TCONS_00016904; exon_number 1; oId CUFF.15956.1; tss_id TSS15298;
3	Cufflinks	exon	171482926	171483084	.	+	.	gene_id GRMZM2G110277; transcript_id TCONS_00017206; exon_number 1; oId CUFF.16463.1; tss_id TSS15569;
3	Cufflinks	exon	171483967	171484022	.	+	.	gene_id GRMZM2G110277; transcript_id TCONS_00017206; exon_number 2; oId CUFF.16463.1; tss_id TSS15569;
3	Cufflinks	exon	171485267	171485809	.	+	.	gene_id GRMZM2G110277; transcript_id TCONS_00017206; exon_number 3; oId CUFF.16463.1; tss_id TSS15569;
3	Cufflinks	exon	171486180	171486296	.	+	.	gene_id GRMZM2G110277; transcript_id TCONS_00017206; exon_number 4; oId CUFF.16463.1; tss_id TSS15569;
3	Cufflinks	exon	171486409	171486494	.	+	.	gene_id GRMZM2G110277; transcript_id TCONS_00017206; exon_number 5; oId CUFF.16463.1; tss_id TSS15569;
3	Cufflinks	exon	171486600	171486892	.	+	.	gene_id GRMZM2G110277; transcript_id TCONS_00017206; exon_number 6; oId CUFF.16463.1; tss_id TSS15569;
3	Cufflinks	exon	176346351	176349021	.	+	.	gene_id GRMZM2G074248; transcript_id TCONS_00017280; exon_number 1; oId CUFF.16577.1; tss_id TSS15637;
3	Cufflinks	exon	178186779	178188651	.	+	.	gene_id XLOC_015190; transcript_id TCONS_00017331; exon_number 1; oId CUFF.16652.1; tss_id TSS15683;

3	Cufflinks	exon	188728781	188729273	.	+	.	gene_id XLOC_015345; transcript_id TCONS_00017511; exon_number 1; oId CUFF.16976.1; tss_id TSS15841;
3	Cufflinks	exon	190516912	190519079	.	+	.	gene_id XLOC_015373; transcript_id TCONS_00017544; exon_number 1; oId CUFF.17036.1; tss_id TSS15869;
3	Cufflinks	exon	191183128	191184336	.	+	.	gene_id XLOC_015379; transcript_id TCONS_00017555; exon_number 1; oId CUFF.17132.1; tss_id TSS15877;
3	Cufflinks	exon	213639551	213641474	.	+	.	gene_id XLOC_015728; transcript_id TCONS_00017957; exon_number 1; oId CUFF.17722.1; tss_id TSS16247;
3	Cufflinks	exon	226500341	226500972	.	+	.	gene_id XLOC_015910; transcript_id TCONS_00018173; exon_number 1; oId CUFF.18101.1; tss_id TSS16441;
3	Cufflinks	exon	227566067	227567279	.	+	.	gene_id XLOC_015929; transcript_id TCONS_00018195; exon_number 1; oId CUFF.18139.1; tss_id TSS16461;
3	Cufflinks	exon	229070973	229071445	.	+	.	gene_id XLOC_015957; transcript_id TCONS_00018229; exon_number 1; oId CUFF.18188.1; tss_id TSS16491;
3	Cufflinks	exon	231024532	231025858	.	+	.	gene_id GRMZM2G152781; transcript_id TCONS_00018279; exon_number 1; oId CUFF.18269.1; tss_id TSS16537;
3	Cufflinks	exon	231562330	231563531	.	+	.	gene_id XLOC_016006; transcript_id TCONS_00018283; exon_number 1; oId CUFF.18284.1; tss_id TSS16541;
3	Cufflinks	exon	4709572	4709965	.	-	.	gene_id XLOC_016108; transcript_id TCONS_00018401; exon_number 1; oId CUFF.13995.1; tss_id TSS16646;
3	Cufflinks	exon	7966861	7967903	.	-	.	gene_id GRMZM2G367898; transcript_id TCONS_00018465; exon_number 1; oId CUFF.14074.1; tss_id TSS16704;
3	Cufflinks	exon	9044823	9046830	.	-	.	gene_id XLOC_016189; transcript_id TCONS_00018490; exon_number 1; oId CUFF.14126.1; tss_id TSS16728;
3	Cufflinks	exon	13278194	13278623	.	-	.	gene_id XLOC_016230; transcript_id TCONS_00018535; exon_number 1; oId CUFF.14243.1; tss_id TSS16770;
3	Cufflinks	exon	15136784	15136881	.	-	.	gene_id XLOC_016255; transcript_id TCONS_00018564; exon_number 1; oId CUFF.14292.1; tss_id TSS16796;
3	Cufflinks	exon	15137385	15138327	.	-	.	gene_id XLOC_016255; transcript_id TCONS_00018564; exon_number 2; oId CUFF.14292.1; tss_id TSS16796;
3	Cufflinks	exon	15138432	15139075	.	-	.	gene_id XLOC_016255; transcript_id TCONS_00018564; exon_number 3; oId CUFF.14292.1; tss_id TSS16796;
3	Cufflinks	exon	18085278	18085649	.	-	.	gene_id XLOC_016291; transcript_id TCONS_00018606; exon_number 1; oId CUFF.14390.1; tss_id TSS16833;
3	Cufflinks	exon	19762167	19764374	.	-	.	gene_id XLOC_016312; transcript_id TCONS_00018627; exon_number 1; oId CUFF.14428.1; tss_id TSS16854;
3	Cufflinks	exon	22005530	22007136	.	-	.	gene_id GRMZM2G156950; transcript_id TCONS_00018654; exon_number 1; oId CUFF.14475.1; tss_id TSS16879;
3	Cufflinks	exon	38438067	38440389	.	-	.	gene_id GRMZM2G368388; transcript_id TCONS_00018818; exon_number 1; oId CUFF.14724.1; tss_id TSS17027;
3	Cufflinks	exon	93102434	93104203	.	-	.	gene_id GRMZM2G133793; transcript_id TCONS_00019114; exon_number 1; oId CUFF.15260.1; tss_id TSS17300;
3	Cufflinks	exon	128957613	128959445	.	-	.	gene_id GRMZM2G160902; transcript_id TCONS_00019336; exon_number 1; oId CUFF.15611.1; tss_id TSS17508;
3	Cufflinks	exon	129110506	129111661	.	-	.	gene_id GRMZM2G046088; transcript_id TCONS_00019340; exon_number 1; oId CUFF.15635.1; tss_id TSS17511;
3	Cufflinks	exon	129113517	129113876	.	-	.	gene_id GRMZM2G046088; transcript_id TCONS_00019340; exon_number 2; oId CUFF.15635.1; tss_id TSS17511;
3	Cufflinks	exon	147559395	147561086	.	-	.	gene_id XLOC_017124; transcript_id TCONS_00019532; exon_number 1; oId CUFF.15955.1; tss_id TSS17690;
3	Cufflinks	exon	154666729	154667335	.	-	.	gene_id XLOC_017193; transcript_id TCONS_00019608; exon_number 1; oId CUFF.16128.1; tss_id TSS17763;
3	Cufflinks	exon	168968523	168969254	.	-	.	gene_id XLOC_017336; transcript_id TCONS_00019783; exon_number 1; oId CUFF.16387.1; tss_id TSS17914;
3	Cufflinks	exon	172092917	172094704	.	-	.	gene_id GRMZM2G135743; transcript_id TCONS_00019831; exon_number 1; oId CUFF.16465.1; tss_id TSS17952;
3	Cufflinks	exon	176346802	176347318	.	-	.	gene_id XLOC_017422; transcript_id TCONS_00019892; exon_number 1; oId CUFF.16578.1; tss_id TSS18005;
3	Cufflinks	exon	197959705	197959966	.	-	.	gene_id XLOC_017755; transcript_id TCONS_00020267; exon_number 1; oId CUFF.17250.1; tss_id TSS18347;
3	Cufflinks	exon	197964318	197965147	.	-	.	gene_id XLOC_017755; transcript_id TCONS_00020267; exon_number 2; oId CUFF.17250.1; tss_id TSS18347;
3	Cufflinks	exon	197965739	197966012	.	-	.	gene_id XLOC_017755; transcript_id TCONS_00020267; exon_number 3; oId CUFF.17250.1; tss_id TSS18347;
3	Cufflinks	exon	197972859	197974998	.	-	.	gene_id XLOC_017755; transcript_id TCONS_00020267; exon_number 4; oId CUFF.17250.1; tss_id TSS18347;
3	Cufflinks	exon	205448996	205451333	.	-	.	gene_id GRMZM2G165099; transcript_id TCONS_00020403; exon_number 1; oId CUFF.17481.1; tss_id TSS18475;
3	Cufflinks	exon	208488417	208489596	.	-	.	gene_id XLOC_017927; transcript_id TCONS_00020464; exon_number 1; oId CUFF.17577.1; tss_id TSS18534;
3	Cufflinks	exon	213639419	213641365	.	-	.	gene_id GRMZM2G022311; transcript_id TCONS_00020540; exon_number 1; oId CUFF.17721.1; tss_id TSS18606;

3	Cufflinks	exon	219419497	219420765	.	-	.	gene_id GRMZM2G079089; transcript_id TCONS_00020634; exon_number 1; oId CUFF.17870.1; tss_id TSS18686;
3	Cufflinks	exon	221718415	221719225	.	-	.	gene_id XLOC_018120; transcript_id TCONS_00020691; exon_number 1; oId CUFF.17955.1; tss_id TSS18737;
3	Cufflinks	exon	223930441	223931772	.	-	.	gene_id GRMZM2G058021; transcript_id TCONS_00020729; exon_number 1; oId CUFF.18039.1; tss_id TSS18773;
3	Cufflinks	exon	227565909	227567701	.	-	.	gene_id GRMZM2G368491; transcript_id TCONS_00020788; exon_number 1; oId CUFF.18137.1; tss_id TSS18828;
3	Cufflinks	exon	231024891	231025397	.	-	.	gene_id XLOC_018266; transcript_id TCONS_00020852; exon_number 1; oId CUFF.18270.1; tss_id TSS18890;
3	Cufflinks	exon	231562253	231563381	.	-	.	gene_id GRMZM2G313320; transcript_id TCONS_00020859; exon_number 1; oId CUFF.18282.1; tss_id TSS18896;
4	Cufflinks	exon	831188832279	.	+	.	gene_id XLOC_018292; transcript_id TCONS_00020882; exon_number 1; oId CUFF.18311.1; tss_id TSS18916;	
4	Cufflinks	exon	2386082	2387319	.	+	.	gene_id GRMZM2G171036; transcript_id TCONS_00020907; exon_number 1; oId CUFF.18373.1; tss_id TSS18940;
4	Cufflinks	exon	19134262	19135158	.	+	.	gene_id XLOC_018538; transcript_id TCONS_00021167; exon_number 1; oId CUFF.18771.1; tss_id TSS19174;
4	Cufflinks	exon	26450748	26452606	.	+	.	gene_id GRMZM2G089995; transcript_id TCONS_00021227; exon_number 1; oId CUFF.18877.1; tss_id TSS19232;
4	Cufflinks	exon	37603564	37604830	.	+	.	gene_id GRMZM2G324643; transcript_id TCONS_00021375; exon_number 1; oId CUFF.19107.1; tss_id TSS19360;
4	Cufflinks	exon	60384206	60385041	.	+	.	gene_id XLOC_018846; transcript_id TCONS_00021527; exon_number 1; oId CUFF.19385.1; tss_id TSS19489;
4	Cufflinks	exon	76410462	76412170	.	+	.	gene_id GRMZM2G066599; transcript_id TCONS_00021644; exon_number 1; oId CUFF.19580.1; tss_id TSS19601;
4	Cufflinks	exon	151683084	151684683	.	+	.	gene_id XLOC_019309; transcript_id TCONS_00022057; exon_number 1; oId CUFF.20302.1; tss_id TSS19969;
4	Cufflinks	exon	152424058	152425840	.	+	.	gene_id GRMZM2G323719; transcript_id TCONS_00022065; exon_number 1; oId CUFF.20311.1; tss_id TSS19977;
4	Cufflinks	exon	156050218	156052167	.	+	.	gene_id XLOC_019360; transcript_id TCONS_00022115; exon_number 1; oId CUFF.20373.1; tss_id TSS20023;
4	Cufflinks	exon	156218446	156219862	.	+	.	gene_id XLOC_019362; transcript_id TCONS_00022117; exon_number 1; oId CUFF.20375.1; tss_id TSS20025;
4	Cufflinks	exon	158586700	158587145	.	+	.	gene_id XLOC_019399; transcript_id TCONS_00022155; exon_number 1; oId CUFF.20446.1; tss_id TSS20062;
4	Cufflinks	exon	160367021	160368068	.	+	.	gene_id XLOC_019416; transcript_id TCONS_00022175; exon_number 1; oId CUFF.20502.1; tss_id TSS20079;
4	Cufflinks	exon	174072810	174073935	.	+	.	gene_id GRMZM2G316787; transcript_id TCONS_00022376; exon_number 1; oId CUFF.20833.1; tss_id TSS20260;
4	Cufflinks	exon	180924991	180926213	.	+	.	gene_id GRMZM2G151107; transcript_id TCONS_00022469; exon_number 1; oId CUFF.21000.1; tss_id TSS20348;
4	Cufflinks	exon	184155293	184156738	.	+	.	gene_id XLOC_019721; transcript_id TCONS_00022523; exon_number 1; oId CUFF.21097.1; tss_id TSS20396;
4	Cufflinks	exon	185210476	185210873	.	+	.	gene_id XLOC_019743; transcript_id TCONS_00022547; exon_number 1; oId CUFF.21153.1; tss_id TSS20418;
4	Cufflinks	exon	188475513	188478292	.	+	.	gene_id XLOC_019790; transcript_id TCONS_00022601; exon_number 1; oId CUFF.21259.1; tss_id TSS20467;
4	Cufflinks	exon	201141912	201143147	.	+	.	gene_id GRMZM2G046111; transcript_id TCONS_00022777; exon_number 1; oId CUFF.21559.1; tss_id TSS20626;
4	Cufflinks	exon	212857831	212859418	.	+	.	gene_id XLOC_020036; transcript_id TCONS_00022885; exon_number 1; oId CUFF.21805.1; tss_id TSS20722;
4	Cufflinks	exon	226843654	226844563	.	+	.	gene_id GRMZM2G033855; transcript_id TCONS_00023003; exon_number 1; oId CUFF.21971.1; tss_id TSS20836;
4	Cufflinks	exon	237855481	237858140	.	+	.	gene_id XLOC_020330; transcript_id TCONS_00023216; exon_number 1; oId CUFF.22320.1; tss_id TSS21020;
4	Cufflinks	exon	238665605	238666775	.	+	.	gene_id XLOC_020354; transcript_id TCONS_00023241; exon_number 1; oId CUFF.22354.1; tss_id TSS21044;
4	Cufflinks	exon	239528888	239529910	.	+	.	gene_id GRMZM2G324956; transcript_id TCONS_00023256; exon_number 1; oId CUFF.22399.1; tss_id TSS21059;
4	Cufflinks	exon	2385958	2386696	.	-	.	gene_id XLOC_020472; transcript_id TCONS_00023371; exon_number 1; oId CUFF.18372.1; tss_id TSS21165;
4	Cufflinks	exon	9490202	9491088	.	-	.	gene_id XLOC_020556; transcript_id TCONS_00023469; exon_number 1; oId CUFF.18604.1; tss_id TSS21253;
4	Cufflinks	exon	15037278	15038658	.	-	.	gene_id XLOC_020611; transcript_id TCONS_00023528; exon_number 1; oId CUFF.18696.1; tss_id TSS21309;
4	Cufflinks	exon	37603712	37604136	.	-	.	gene_id XLOC_020801; transcript_id TCONS_00023735; exon_number 1; oId CUFF.19108.1; tss_id TSS21507;
4	Cufflinks	exon	40012198	40012616	.	-	.	gene_id XLOC_020835; transcript_id TCONS_00023771; exon_number 1; oId CUFF.19163.1; tss_id TSS21541;
4	Cufflinks	exon	71934793	71936351	.	-	.	gene_id XLOC_021018; transcript_id TCONS_00023978; exon_number 1; oId CUFF.19543.1; tss_id TSS21726;

4	Cufflinks	exon	131205067	131205607	.	-	.	gene_id XLOC_021276; transcript_id TCONS_00024286; exon_number 1; oId CUFF.20062.1; tss_id TSS21992;
4	Cufflinks	exon	156049488	156052142	.	-	.	gene_id GRMZM2G028438; transcript_id TCONS_00024455; exon_number 1; oId CUFF.20372.1; tss_id TSS22146;
4	Cufflinks	exon	167106377	167106872	.	-	.	gene_id XLOC_021554; transcript_id TCONS_00024591; exon_number 1; oId CUFF.20638.1; tss_id TSS22276;
4	Cufflinks	exon	170884819	170886073	.	-	.	gene_id GRMZM2G146228; transcript_id TCONS_00024645; exon_number 1; oId CUFF.20724.1; tss_id TSS22324;
4	Cufflinks	exon	174072841	174074369	.	-	.	gene_id GRMZM2G316778; transcript_id TCONS_00024709; exon_number 1; oId CUFF.20835.1; tss_id TSS22380;
4	Cufflinks	exon	180924869	180925861	.	-	.	gene_id XLOC_021744; transcript_id TCONS_00024816; exon_number 1; oId CUFF.20999.1; tss_id TSS22471;
4	Cufflinks	exon	181903925	181905990	.	-	.	gene_id XLOC_021758; transcript_id TCONS_00024834; exon_number 1; oId CUFF.21034.1; tss_id TSS22485;
4	Cufflinks	exon	201141953	201142723	.	-	.	gene_id XLOC_022029; transcript_id TCONS_00025152; exon_number 1; oId CUFF.21560.1; tss_id TSS22767;
4	Cufflinks	exon	212126428	212127100	.	-	.	gene_id XLOC_022119; transcript_id TCONS_00025253; exon_number 1; oId CUFF.21741.1; tss_id TSS22860;
4	Cufflinks	exon	212853674	212855001	.	-	.	gene_id GRMZM2G458728; transcript_id TCONS_00025262; exon_number 1; oId CUFF.21806.3; tss_id TSS22867;
4	Cufflinks	exon	212855109	212855169	.	-	.	gene_id GRMZM2G458728; transcript_id TCONS_00025262; exon_number 2; oId CUFF.21806.3; tss_id TSS22867;
4	Cufflinks	exon	212855452	212855834	.	-	.	gene_id GRMZM2G458728; transcript_id TCONS_00025262; exon_number 3; oId CUFF.21806.3; tss_id TSS22867;
4	Cufflinks	exon	212857214	212858099	.	-	.	gene_id GRMZM2G458728; transcript_id TCONS_00025262; exon_number 4; oId CUFF.21806.3; tss_id TSS22867;
4	Cufflinks	exon	212858849	212859386	.	-	.	gene_id GRMZM2G458728; transcript_id TCONS_00025262; exon_number 5; oId CUFF.21806.3; tss_id TSS22867;
4	Cufflinks	exon	212853674	212855001	.	-	.	gene_id GRMZM2G458728; transcript_id TCONS_00025260; exon_number 1; oId CUFF.21806.1; tss_id TSS22867;
4	Cufflinks	exon	212855109	212855169	.	-	.	gene_id GRMZM2G458728; transcript_id TCONS_00025260; exon_number 2; oId CUFF.21806.1; tss_id TSS22867;
4	Cufflinks	exon	212855452	212855834	.	-	.	gene_id GRMZM2G458728; transcript_id TCONS_00025260; exon_number 3; oId CUFF.21806.1; tss_id TSS22867;
4	Cufflinks	exon	212857214	212858099	.	-	.	gene_id GRMZM2G458728; transcript_id TCONS_00025260; exon_number 4; oId CUFF.21806.1; tss_id TSS22867;
4	Cufflinks	exon	212858598	212859386	.	-	.	gene_id GRMZM2G458728; transcript_id TCONS_00025260; exon_number 5; oId CUFF.21806.1; tss_id TSS22867;
4	Cufflinks	exon	217185342	217185795	.	-	.	gene_id XLOC_022156; transcript_id TCONS_00025296; exon_number 1; oId CUFF.21824.1; tss_id TSS22898;
4	Cufflinks	exon	226107900	226108706	.	-	.	gene_id XLOC_022221; transcript_id TCONS_00025369; exon_number 1; oId CUFF.21945.1; tss_id TSS22965;
4	Cufflinks	exon	228426856	228427885	.	-	.	gene_id XLOC_022248; transcript_id TCONS_00025398; exon_number 1; oId CUFF.22002.1; tss_id TSS22992;
4	Cufflinks	exon	235658484	235659038	.	-	.	gene_id XLOC_022355; transcript_id TCONS_00025524; exon_number 1; oId CUFF.22202.1; tss_id TSS23101;
4	Cufflinks	exon	237855270	237857865	.	-	.	gene_id XLOC_022406; transcript_id TCONS_00025585; exon_number 1; oId CUFF.22319.1; tss_id TSS23154;
4	Cufflinks	exon	239288712	239289401	.	-	.	gene_id GRMZM2G027209; transcript_id TCONS_00025634; exon_number 1; oId CUFF.22418.1; tss_id TSS23199;
4	Cufflinks	exon	239292958	239293053	.	-	.	gene_id GRMZM2G027209; transcript_id TCONS_00025634; exon_number 2; oId CUFF.22418.1; tss_id TSS23199;
4	Cufflinks	exon	239293234	239293310	.	-	.	gene_id GRMZM2G027209; transcript_id TCONS_00025634; exon_number 3; oId CUFF.22418.1; tss_id TSS23199;
4	Cufflinks	exon	239298794	239298862	.	-	.	gene_id GRMZM2G027209; transcript_id TCONS_00025634; exon_number 4; oId CUFF.22418.1; tss_id TSS23199;
4	Cufflinks	exon	239298954	239299007	.	-	.	gene_id GRMZM2G027209; transcript_id TCONS_00025634; exon_number 5; oId CUFF.22418.1; tss_id TSS23199;
4	Cufflinks	exon	239299416	239299461	.	-	.	gene_id GRMZM2G027209; transcript_id TCONS_00025634; exon_number 6; oId CUFF.22418.1; tss_id TSS23199;
4	Cufflinks	exon	239299540	239299577	.	-	.	gene_id GRMZM2G027209; transcript_id TCONS_00025634; exon_number 7; oId CUFF.22418.1; tss_id TSS23199;
4	Cufflinks	exon	239299719	239299765	.	-	.	gene_id GRMZM2G027209; transcript_id TCONS_00025634; exon_number 8; oId CUFF.22418.1; tss_id TSS23199;
4	Cufflinks	exon	239299850	239300120	.	-	.	gene_id GRMZM2G027209; transcript_id TCONS_00025634; exon_number 9; oId CUFF.22418.1; tss_id TSS23199;
4	Cufflinks	exon	239300271	239300359	.	-	.	gene_id GRMZM2G027209; transcript_id TCONS_00025634; exon_number 10; oId CUFF.22418.1; tss_id TSS23199;
4	Cufflinks	exon	239300462	239300856	.	-	.	gene_id GRMZM2G027209; transcript_id TCONS_00025634; exon_number 11; oId CUFF.22418.1; tss_id TSS23199;
4	Cufflinks	exon	239529220	239529624	.	-	.	gene_id XLOC_022453; transcript_id TCONS_00025641; exon_number 1; oId CUFF.22400.1; tss_id TSS23203;

4	Cufflinks	exon	240275454	240276216	.	-	.	gene_id XLOC_022492; transcript_id TCONS_00025688; exon_number 1; oId CUFF.22472.1; tss_id TSS23244;
5	Cufflinks	exon	814586	815398	.	+	.	gene_id XLOC_022536; transcript_id TCONS_00025741; exon_number 1; oId CUFF.22564.1; tss_id TSS23288;
5	Cufflinks	exon	892919	893892	.	+	.	gene_id GRMZM2G384338; transcript_id TCONS_00025749; exon_number 1; oId CUFF.22573.1; tss_id TSS23294;
5	Cufflinks	exon	1582617	1583214	.	+	.	gene_id XLOC_022577; transcript_id TCONS_00025795; exon_number 1; oId CUFF.22629.1; tss_id TSS23332;
5	Cufflinks	exon	6320889	6322687	.	+	.	gene_id GRMZM2G035103; transcript_id TCONS_00025980; exon_number 1; oId CUFF.22937.1; tss_id TSS23493;
5	Cufflinks	exon	7773900	7774829	.	+	.	gene_id XLOC_022769; transcript_id TCONS_00026020; exon_number 1; oId CUFF.23017.1; tss_id TSS23527;
5	Cufflinks	exon	23677896	23679481	.	+	.	gene_id XLOC_023042; transcript_id TCONS_00026321; exon_number 1; oId CUFF.23550.1; tss_id TSS23805;
5	Cufflinks	exon	41225312	41226535	.	+	.	gene_id GRMZM2G366077; transcript_id TCONS_00026516; exon_number 1; oId CUFF.23896.1; tss_id TSS23974;
5	Cufflinks	exon	53425395	53426300	.	+	.	gene_id XLOC_023299; transcript_id TCONS_00026625; exon_number 1; oId CUFF.24080.1; tss_id TSS24074;
5	Cufflinks	exon	62711807	62712234	.	+	.	gene_id XLOC_023389; transcript_id TCONS_00026732; exon_number 1; oId CUFF.24287.1; tss_id TSS24167;
5	Cufflinks	exon	86050120	86052355	.	+	.	gene_id AC207043.3_FG002; transcript_id TCONS_00026995; exon_number 1; oId CUFF.24739.1; tss_id TSS24412;
5	Cufflinks	exon	93551777	93552691	.	+	.	gene_id XLOC_023660; transcript_id TCONS_00027041; exon_number 1; oId CUFF.24831.1; tss_id TSS24452;
5	Cufflinks	exon	147664563	147665713	.	+	.	gene_id XLOC_023896; transcript_id TCONS_00027290; exon_number 1; oId CUFF.25314.1; tss_id TSS24691;
5	Cufflinks	exon	151923737	151926498	.	+	.	gene_id XLOC_023914; transcript_id TCONS_00027309; exon_number 1; oId CUFF.25350.1; tss_id TSS24709;
5	Cufflinks	exon	152472231	152472631	.	+	.	gene_id XLOC_023917; transcript_id TCONS_00027312; exon_number 1; oId CUFF.25368.1; tss_id TSS24712;
5	Cufflinks	exon	153416709	153417683	.	+	.	gene_id XLOC_023924; transcript_id TCONS_00027319; exon_number 1; oId CUFF.25362.1; tss_id TSS24719;
5	Cufflinks	exon	171317220	171318479	.	+	.	gene_id XLOC_024096; transcript_id TCONS_00027509; exon_number 1; oId CUFF.25694.1; tss_id TSS24894;
5	Cufflinks	exon	191140510	191142976	.	+	.	gene_id GRMZM2G097704; transcript_id TCONS_00027813; exon_number 1; oId CUFF.26215.1; tss_id TSS25164;
5	Cufflinks	exon	196336155	196336999	.	+	.	gene_id XLOC_024432; transcript_id TCONS_00027898; exon_number 1; oId CUFF.26386.1; tss_id TSS25243;
5	Cufflinks	exon	198928604	198930118	.	+	.	gene_id GRMZM2G413829; transcript_id TCONS_00027925; exon_number 1; oId CUFF.26409.1; tss_id TSS25269;
5	Cufflinks	exon	205591579	205593459	.	+	.	gene_id XLOC_024629; transcript_id TCONS_00028128; exon_number 1; oId CUFF.26694.1; tss_id TSS25447;
5	Cufflinks	exon	205804786	205805352	.	+	.	gene_id XLOC_024636; transcript_id TCONS_00028136; exon_number 1; oId CUFF.26700.1; tss_id TSS25454;
5	Cufflinks	exon	205984369	205984925	.	+	.	gene_id XLOC_024641; transcript_id TCONS_00028141; exon_number 1; oId CUFF.26705.1; tss_id TSS25459;
5	Cufflinks	exon	208141589	208142230	.	+	.	gene_id XLOC_024678; transcript_id TCONS_00028182; exon_number 1; oId CUFF.26816.1; tss_id TSS25496;
5	Cufflinks	exon	214577054	214577732	.	+	.	gene_id GRMZM2G163200; transcript_id TCONS_00028358; exon_number 1; oId CUFF.27111.2; tss_id TSS25646;
5	Cufflinks	exon	214577944	214578265	.	+	.	gene_id GRMZM2G163200; transcript_id TCONS_00028358; exon_number 2; oId CUFF.27111.2; tss_id TSS25646;
5	Cufflinks	exon	214578927	214580589	.	+	.	gene_id GRMZM2G163200; transcript_id TCONS_00028358; exon_number 3; oId CUFF.27111.2; tss_id TSS25646;
5	Cufflinks	exon	215072718	215073186	.	+	.	gene_id XLOC_024823; transcript_id TCONS_00028362; exon_number 1; oId CUFF.27120.1; tss_id TSS25650;
5	Cufflinks	exon	216124627	216126640	.	+	.	gene_id GRMZM2G002124; transcript_id TCONS_00028402; exon_number 1; oId CUFF.27182.1; tss_id TSS25687;
5	Cufflinks	exon	216749139	216751299	.	+	.	gene_id AC208348.3_FG012; transcript_id TCONS_00028424; exon_number 1; oId CUFF.27250.1; tss_id TSS25705;
5	Cufflinks	exon	1526661	1529125	.	-	.	gene_id GRMZM2G448001; transcript_id TCONS_00028499; exon_number 1; oId CUFF.22633.1; tss_id TSS25762;
5	Cufflinks	exon	1582563	1583065	.	-	.	gene_id GRMZM2G125784; transcript_id TCONS_00028506; exon_number 1; oId CUFF.22628.1; tss_id TSS25769;
5	Cufflinks	exon	4768343	4769519	.	-	.	gene_id XLOC_025045; transcript_id TCONS_00028636; exon_number 1; oId CUFF.22855.1; tss_id TSS25884;
5	Cufflinks	exon	7786550	7787521	.	-	.	gene_id GRMZM2G077295; transcript_id TCONS_00028728; exon_number 1; oId CUFF.23029.3; tss_id TSS25966;
5	Cufflinks	exon	23677551	23679570	.	-	.	gene_id GRMZM2G018695; transcript_id TCONS_00029009; exon_number 1; oId CUFF.23549.1; tss_id TSS26224;
5	Cufflinks	exon	31464541	31465344	.	-	.	gene_id XLOC_025452; transcript_id TCONS_00029094; exon_number 1; oId CUFF.23697.1; tss_id TSS26304;

5	Cufflinks	exon	32244735	32247057	.	-	.	gene_id XLOC_025464; transcript_id TCONS_00029107; exon_number 1; oId CUFF.23718.1; tss_id TSS26316;
5	Cufflinks	exon	35731101	35732701	.	-	.	gene_id XLOC_025508; transcript_id TCONS_00029161; exon_number 1; oId CUFF.23798.1; tss_id TSS26363;
5	Cufflinks	exon	48902394	48902868	.	-	.	gene_id XLOC_025621; transcript_id TCONS_00029299; exon_number 1; oId CUFF.24009.1; tss_id TSS26482;
5	Cufflinks	exon	56392398	56393951	.	-	.	gene_id XLOC_025682; transcript_id TCONS_00029365; exon_number 1; oId CUFF.24124.1; tss_id TSS26544;
5	Cufflinks	exon	60683906	60685891	.	-	.	gene_id XLOC_025737; transcript_id TCONS_00029421; exon_number 1; oId CUFF.24220.1; tss_id TSS26599;
5	Cufflinks	exon	63306785	63308362	.	-	.	gene_id XLOC_025771; transcript_id TCONS_00029456; exon_number 1; oId CUFF.24296.1; tss_id TSS26633;
5	Cufflinks	exon	70114064	70115222	.	-	.	gene_id XLOC_025853; transcript_id TCONS_00029547; exon_number 1; oId CUFF.24464.1; tss_id TSS26717;
5	Cufflinks	exon	73078048	73079005	.	-	.	gene_id XLOC_025877; transcript_id TCONS_00029576; exon_number 1; oId CUFF.24499.1; tss_id TSS26743;
5	Cufflinks	exon	77952734	77954985	.	-	.	gene_id XLOC_025924; transcript_id TCONS_00029633; exon_number 1; oId CUFF.24615.1; tss_id TSS26792;
5	Cufflinks	exon	86050854	86051671	.	-	.	gene_id XLOC_025989; transcript_id TCONS_00029711; exon_number 1; oId CUFF.24740.1; tss_id TSS26859;
5	Cufflinks	exon	160692953	160693951	.	-	.	gene_id XLOC_026357; transcript_id TCONS_00030124; exon_number 1; oId CUFF.25473.1; tss_id TSS27239;
5	Cufflinks	exon	163455311	163456332	.	-	.	gene_id GRMZM2G004856; transcript_id TCONS_00030168; exon_number 1; oId CUFF.25550.2; tss_id TSS27269;
5	Cufflinks	exon	163456974	163457561	.	-	.	gene_id GRMZM2G004856; transcript_id TCONS_00030168; exon_number 2; oId CUFF.25550.2; tss_id TSS27269;
5	Cufflinks	exon	163457785	163457941	.	-	.	gene_id GRMZM2G004856; transcript_id TCONS_00030168; exon_number 3; oId CUFF.25550.2; tss_id TSS27269;
5	Cufflinks	exon	164401022	164401850	.	-	.	gene_id XLOC_026392; transcript_id TCONS_00030175; exon_number 1; oId CUFF.25543.1; tss_id TSS27276;
5	Cufflinks	exon	170543803	170545707	.	-	.	gene_id GRMZM2G303964; transcript_id TCONS_00030253; exon_number 1; oId CUFF.25677.1; tss_id TSS27346;
5	Cufflinks	exon	171317073	171318365	.	-	.	gene_id GRMZM2G118825; transcript_id TCONS_00030261; exon_number 1; oId CUFF.25693.1; tss_id TSS27354;
5	Cufflinks	exon	172660909	172663243	.	-	.	gene_id XLOC_026481; transcript_id TCONS_00030277; exon_number 1; oId CUFF.25720.1; tss_id TSS27368;
5	Cufflinks	exon	172851847	172853370	.	-	.	gene_id GRMZM2G119517; transcript_id TCONS_00030278; exon_number 1; oId CUFF.25754.3; tss_id TSS27369;
5	Cufflinks	exon	172853516	172853617	.	-	.	gene_id GRMZM2G119517; transcript_id TCONS_00030278; exon_number 2; oId CUFF.25754.3; tss_id TSS27369;
5	Cufflinks	exon	172853788	172854441	.	-	.	gene_id GRMZM2G119517; transcript_id TCONS_00030278; exon_number 3; oId CUFF.25754.3; tss_id TSS27369;
5	Cufflinks	exon	172854616	172854683	.	-	.	gene_id GRMZM2G119517; transcript_id TCONS_00030278; exon_number 4; oId CUFF.25754.3; tss_id TSS27369;
5	Cufflinks	exon	172854787	172854901	.	-	.	gene_id GRMZM2G119517; transcript_id TCONS_00030278; exon_number 5; oId CUFF.25754.3; tss_id TSS27369;
5	Cufflinks	exon	172855046	172855091	.	-	.	gene_id GRMZM2G119517; transcript_id TCONS_00030278; exon_number 6; oId CUFF.25754.3; tss_id TSS27369;
5	Cufflinks	exon	172855184	172855274	.	-	.	gene_id GRMZM2G119517; transcript_id TCONS_00030278; exon_number 7; oId CUFF.25754.3; tss_id TSS27369;
5	Cufflinks	exon	172855496	172855542	.	-	.	gene_id GRMZM2G119517; transcript_id TCONS_00030278; exon_number 8; oId CUFF.25754.3; tss_id TSS27369;
5	Cufflinks	exon	172855768	172856315	.	-	.	gene_id GRMZM2G119517; transcript_id TCONS_00030278; exon_number 9; oId CUFF.25754.3; tss_id TSS27369;
5	Cufflinks	exon	172851847	172853370	.	-	.	gene_id GRMZM2G119517; transcript_id TCONS_00030280; exon_number 1; oId CUFF.25754.2; tss_id TSS27370;
5	Cufflinks	exon	172853516	172853617	.	-	.	gene_id GRMZM2G119517; transcript_id TCONS_00030280; exon_number 2; oId CUFF.25754.2; tss_id TSS27370;
5	Cufflinks	exon	172853788	172854441	.	-	.	gene_id GRMZM2G119517; transcript_id TCONS_00030280; exon_number 3; oId CUFF.25754.2; tss_id TSS27370;
5	Cufflinks	exon	172854616	172854683	.	-	.	gene_id GRMZM2G119517; transcript_id TCONS_00030280; exon_number 4; oId CUFF.25754.2; tss_id TSS27370;
5	Cufflinks	exon	172854787	172854901	.	-	.	gene_id GRMZM2G119517; transcript_id TCONS_00030280; exon_number 5; oId CUFF.25754.2; tss_id TSS27370;
5	Cufflinks	exon	172855046	172855091	.	-	.	gene_id GRMZM2G119517; transcript_id TCONS_00030280; exon_number 6; oId CUFF.25754.2; tss_id TSS27370;
5	Cufflinks	exon	172855184	172855274	.	-	.	gene_id GRMZM2G119517; transcript_id TCONS_00030280; exon_number 7; oId CUFF.25754.2; tss_id TSS27370;
5	Cufflinks	exon	172855496	172855542	.	-	.	gene_id GRMZM2G119517; transcript_id TCONS_00030280; exon_number 8; oId CUFF.25754.2; tss_id TSS27370;
5	Cufflinks	exon	172858104	172858249	.	-	.	gene_id GRMZM2G119517; transcript_id TCONS_00030280; exon_number 9; oId CUFF.25754.2; tss_id TSS27370;

5	Cufflinks	exon	172858477	172858649	.	-	.	gene_id GRMZM2G119517; transcript_id TCONS_00030280; exon_number 10; oId CUFF.25754.2; tss_id TSS27370;
5	Cufflinks	exon	172851847	172853370	.	-	.	gene_id GRMZM2G119517; transcript_id TCONS_00030279; exon_number 1; oId CUFF.25754.1; tss_id TSS27370;
5	Cufflinks	exon	172853516	172853617	.	-	.	gene_id GRMZM2G119517; transcript_id TCONS_00030279; exon_number 2; oId CUFF.25754.1; tss_id TSS27370;
5	Cufflinks	exon	172853788	172854441	.	-	.	gene_id GRMZM2G119517; transcript_id TCONS_00030279; exon_number 3; oId CUFF.25754.1; tss_id TSS27370;
5	Cufflinks	exon	172854616	172854683	.	-	.	gene_id GRMZM2G119517; transcript_id TCONS_00030279; exon_number 4; oId CUFF.25754.1; tss_id TSS27370;
5	Cufflinks	exon	172854787	172854901	.	-	.	gene_id GRMZM2G119517; transcript_id TCONS_00030279; exon_number 5; oId CUFF.25754.1; tss_id TSS27370;
5	Cufflinks	exon	172855046	172855091	.	-	.	gene_id GRMZM2G119517; transcript_id TCONS_00030279; exon_number 6; oId CUFF.25754.1; tss_id TSS27370;
5	Cufflinks	exon	172855184	172855274	.	-	.	gene_id GRMZM2G119517; transcript_id TCONS_00030279; exon_number 7; oId CUFF.25754.1; tss_id TSS27370;
5	Cufflinks	exon	172855496	172855542	.	-	.	gene_id GRMZM2G119517; transcript_id TCONS_00030279; exon_number 8; oId CUFF.25754.1; tss_id TSS27370;
5	Cufflinks	exon	172858104	172858649	.	-	.	gene_id GRMZM2G119517; transcript_id TCONS_00030279; exon_number 9; oId CUFF.25754.1; tss_id TSS27370;
5	Cufflinks	exon	176155022	176156880	.	-	.	gene_id XLOC_026536; transcript_id TCONS_00030343; exon_number 1; oId CUFF.25826.1; tss_id TSS27426;
5	Cufflinks	exon	176656189	176657863	.	-	.	gene_id XLOC_026539; transcript_id TCONS_00030347; exon_number 1; oId CUFF.25839.1; tss_id TSS27429;
5	Cufflinks	exon	180235632	180236130	.	-	.	gene_id XLOC_026582; transcript_id TCONS_00030400; exon_number 1; oId CUFF.25926.1; tss_id TSS27475;
5	Cufflinks	exon	191141025	191142528	.	-	.	gene_id XLOC_026733; transcript_id TCONS_00030578; exon_number 1; oId CUFF.26216.1; tss_id TSS27632;
5	Cufflinks	exon	191734943	191735815	.	-	.	gene_id XLOC_026742; transcript_id TCONS_00030589; exon_number 1; oId CUFF.26237.1; tss_id TSS27642;
5	Cufflinks	exon	196333393	196334419	.	-	.	gene_id GRMZM5G833032; transcript_id TCONS_00030643; exon_number 1; oId CUFF.26385.2; tss_id TSS27688;
5	Cufflinks	exon	205389374	205390454	.	-	.	gene_id GRMZM2G181507; transcript_id TCONS_00030809; exon_number 1; oId CUFF.26721.3; tss_id TSS27841;
5	Cufflinks	exon	205390674	205390738	.	-	.	gene_id GRMZM2G181507; transcript_id TCONS_00030809; exon_number 2; oId CUFF.26721.3; tss_id TSS27841;
5	Cufflinks	exon	205390817	205390945	.	-	.	gene_id GRMZM2G181507; transcript_id TCONS_00030809; exon_number 3; oId CUFF.26721.3; tss_id TSS27841;
5	Cufflinks	exon	205391085	205391268	.	-	.	gene_id GRMZM2G181507; transcript_id TCONS_00030809; exon_number 4; oId CUFF.26721.3; tss_id TSS27841;
5	Cufflinks	exon	205391378	205391473	.	-	.	gene_id GRMZM2G181507; transcript_id TCONS_00030809; exon_number 5; oId CUFF.26721.3; tss_id TSS27841;
5	Cufflinks	exon	205391591	205391789	.	-	.	gene_id GRMZM2G181507; transcript_id TCONS_00030809; exon_number 6; oId CUFF.26721.3; tss_id TSS27841;
5	Cufflinks	exon	205392881	205393122	.	-	.	gene_id GRMZM2G181507; transcript_id TCONS_00030809; exon_number 7; oId CUFF.26721.3; tss_id TSS27841;
5	Cufflinks	exon	205394491	205395997	.	-	.	gene_id GRMZM2G181507; transcript_id TCONS_00030809; exon_number 8; oId CUFF.26721.3; tss_id TSS27841;
5	Cufflinks	exon	205397234	205397343	.	-	.	gene_id GRMZM2G181507; transcript_id TCONS_00030809; exon_number 9; oId CUFF.26721.3; tss_id TSS27841;
5	Cufflinks	exon	205397525	205397708	.	-	.	gene_id GRMZM2G181507; transcript_id TCONS_00030809; exon_number 10; oId CUFF.26721.3; tss_id TSS27841;
5	Cufflinks	exon	205397790	205397902	.	-	.	gene_id GRMZM2G181507; transcript_id TCONS_00030809; exon_number 11; oId CUFF.26721.3; tss_id TSS27841;
5	Cufflinks	exon	205399151	205399321	.	-	.	gene_id GRMZM2G181507; transcript_id TCONS_00030809; exon_number 12; oId CUFF.26721.3; tss_id TSS27841;
5	Cufflinks	exon	205399405	205399668	.	-	.	gene_id GRMZM2G181507; transcript_id TCONS_00030809; exon_number 13; oId CUFF.26721.3; tss_id TSS27841;
5	Cufflinks	exon	205400802	205401139	.	-	.	gene_id GRMZM2G181507; transcript_id TCONS_00030809; exon_number 14; oId CUFF.26721.3; tss_id TSS27841;
5	Cufflinks	exon	205389374	205390454	.	-	.	gene_id GRMZM2G181507; transcript_id TCONS_00030808; exon_number 1; oId CUFF.26721.2; tss_id TSS27841;
5	Cufflinks	exon	205390674	205390738	.	-	.	gene_id GRMZM2G181507; transcript_id TCONS_00030808; exon_number 2; oId CUFF.26721.2; tss_id TSS27841;
5	Cufflinks	exon	205390817	205390989	.	-	.	gene_id GRMZM2G181507; transcript_id TCONS_00030808; exon_number 3; oId CUFF.26721.2; tss_id TSS27841;
5	Cufflinks	exon	205391085	205391268	.	-	.	gene_id GRMZM2G181507; transcript_id TCONS_00030808; exon_number 4; oId CUFF.26721.2; tss_id TSS27841;
5	Cufflinks	exon	205391378	205391473	.	-	.	gene_id GRMZM2G181507; transcript_id TCONS_00030808; exon_number 5; oId CUFF.26721.2; tss_id TSS27841;
5	Cufflinks	exon	205391591	205391789	.	-	.	gene_id GRMZM2G181507; transcript_id TCONS_00030808; exon_number 6; oId CUFF.26721.2; tss_id TSS27841;

5	Cufflinks	exon	205392881	205393122	.	-	.	gene_id GRMZM2G181507; transcript_id TCONS_00030808; exon_number 7; oId CUFF.26721.2; tss_id TSS27841;
5	Cufflinks	exon	205394491	205395997	.	-	.	gene_id GRMZM2G181507; transcript_id TCONS_00030808; exon_number 8; oId CUFF.26721.2; tss_id TSS27841;
5	Cufflinks	exon	205397234	205397343	.	-	.	gene_id GRMZM2G181507; transcript_id TCONS_00030808; exon_number 9; oId CUFF.26721.2; tss_id TSS27841;
5	Cufflinks	exon	205397525	205397708	.	-	.	gene_id GRMZM2G181507; transcript_id TCONS_00030808; exon_number 10; oId CUFF.26721.2; tss_id TSS27841;
5	Cufflinks	exon	205397790	205397902	.	-	.	gene_id GRMZM2G181507; transcript_id TCONS_00030808; exon_number 11; oId CUFF.26721.2; tss_id TSS27841;
5	Cufflinks	exon	205399151	205399321	.	-	.	gene_id GRMZM2G181507; transcript_id TCONS_00030808; exon_number 12; oId CUFF.26721.2; tss_id TSS27841;
5	Cufflinks	exon	205399405	205399668	.	-	.	gene_id GRMZM2G181507; transcript_id TCONS_00030808; exon_number 13; oId CUFF.26721.2; tss_id TSS27841;
5	Cufflinks	exon	205400802	205401139	.	-	.	gene_id GRMZM2G181507; transcript_id TCONS_00030808; exon_number 14; oId CUFF.26721.2; tss_id TSS27841;
5	Cufflinks	exon	206668390	206669348	.	-	.	gene_id XLOC_026963; transcript_id TCONS_00030836; exon_number 1; oId CUFF.26749.1; tss_id TSS27868;
5	Cufflinks	exon	208973552	208975257	.	-	.	gene_id XLOC_027011; transcript_id TCONS_00030888; exon_number 1; oId CUFF.26835.1; tss_id TSS27917;
5	Cufflinks	exon	210524837	210526166	.	-	.	gene_id GRMZM2G154332; transcript_id TCONS_00030934; exon_number 1; oId CUFF.26912.1; tss_id TSS27960;
5	Cufflinks	exon	210827628	210828045	.	-	.	gene_id XLOC_027066; transcript_id TCONS_00030948; exon_number 1; oId CUFF.26937.1; tss_id TSS27974;
5	Cufflinks	exon	213088484	213089266	.	-	.	gene_id XLOC_027111; transcript_id TCONS_00031003; exon_number 1; oId CUFF.27018.1; tss_id TSS28019;
6	Cufflinks	exon	2724149	2724788	.	+	.	gene_id XLOC_027260; transcript_id TCONS_00031177; exon_number 1; oId CUFF.27301.1; tss_id TSS28173;
6	Cufflinks	exon	14021973	14022731	.	+	.	gene_id XLOC_027435; transcript_id TCONS_00031359; exon_number 1; oId CUFF.27679.1; tss_id TSS28349;
6	Cufflinks	exon	14236270	14236911	.	+	.	gene_id XLOC_027436; transcript_id TCONS_00031360; exon_number 1; oId CUFF.27683.1; tss_id TSS28350;
6	Cufflinks	exon	24281335	24281874	.	+	.	gene_id XLOC_027540; transcript_id TCONS_00031473; exon_number 1; oId CUFF.27882.1; tss_id TSS28454;
6	Cufflinks	exon	56251970	56252630	.	+	.	gene_id GRMZM2G453388; transcript_id TCONS_00031653; exon_number 1; oId CUFF.28240.1; tss_id TSS28615;
6	Cufflinks	exon	56261688	56263608	.	+	.	gene_id GRMZM2G453388; transcript_id TCONS_00031653; exon_number 2; oId CUFF.28240.1; tss_id TSS28615;
6	Cufflinks	exon	63697116	63697832	.	+	.	gene_id XLOC_027748; transcript_id TCONS_00031708; exon_number 1; oId CUFF.28306.1; tss_id TSS28666;
6	Cufflinks	exon	76846012	76848163	.	+	.	gene_id GRMZM2G419542; transcript_id TCONS_00031807; exon_number 1; oId CUFF.28471.1; tss_id TSS28759;
6	Cufflinks	exon	81785309	81785930	.	+	.	gene_id XLOC_027860; transcript_id TCONS_00031832; exon_number 1; oId CUFF.28517.1; tss_id TSS28781;
6	Cufflinks	exon	83989400	83990186	.	+	.	gene_id XLOC_027890; transcript_id TCONS_00031868; exon_number 1; oId CUFF.28596.1; tss_id TSS28812;
6	Cufflinks	exon	98420954	98422447	.	+	.	gene_id GRMZM2G082118; transcript_id TCONS_00032070; exon_number 1; oId CUFF.28922.1; tss_id TSS28999;
6	Cufflinks	exon	102764632	102765222	.	+	.	gene_id XLOC_028096; transcript_id TCONS_00032101; exon_number 1; oId CUFF.28970.1; tss_id TSS29025;
6	Cufflinks	exon	104839450	104840850	.	+	.	gene_id XLOC_028123; transcript_id TCONS_00032132; exon_number 1; oId CUFF.29012.1; tss_id TSS29055;
6	Cufflinks	exon	105706618	105708350	.	+	.	gene_id GRMZM2G348846; transcript_id TCONS_00032147; exon_number 1; oId CUFF.29040.1; tss_id TSS29069;
6	Cufflinks	exon	106612472	106613000	.	+	.	gene_id XLOC_028149; transcript_id TCONS_00032162; exon_number 1; oId CUFF.29055.1; tss_id TSS29082;
6	Cufflinks	exon	109558799	109560880	.	+	.	gene_id GRMZM2G025491; transcript_id TCONS_00032203; exon_number 1; oId CUFF.29133.1; tss_id TSS29120;
6	Cufflinks	exon	129268752	129270611	.	+	.	gene_id GRMZM2G447795; transcript_id TCONS_00032418; exon_number 1; oId CUFF.29550.1; tss_id TSS29319;
6	Cufflinks	exon	134284618	134285372	.	+	.	gene_id XLOC_028447; transcript_id TCONS_00032486; exon_number 1; oId CUFF.29697.1; tss_id TSS29382;
6	Cufflinks	exon	138474734	138476438	.	+	.	gene_id XLOC_028494; transcript_id TCONS_00032537; exon_number 1; oId CUFF.29785.1; tss_id TSS29430;
6	Cufflinks	exon	138775095	138776992	.	+	.	gene_id GRMZM2G340444; transcript_id TCONS_00032539; exon_number 1; oId CUFF.29783.1; tss_id TSS29432;
6	Cufflinks	exon	139642333	139643913	.	+	.	gene_id GRMZM2G423898; transcript_id TCONS_00032548; exon_number 1; oId CUFF.29805.1; tss_id TSS29441;
6	Cufflinks	exon	142758863	142760970	.	+	.	gene_id XLOC_028541; transcript_id TCONS_00032591; exon_number 1; oId CUFF.29892.1; tss_id TSS29480;
6	Cufflinks	exon	148160727	148161594	.	+	.	gene_id XLOC_028627; transcript_id TCONS_00032684; exon_number 1; oId CUFF.30059.1; tss_id TSS29570;

6	Cufflinks	exon	153977792	153980864	.	+	.	gene_id GRMZM2G125034; transcript_id TCONS_00032791; exon_number 1; oId CUFF.30262.1; tss_id TSS29669;
6	Cufflinks	exon	154431415	154432970	.	+	.	gene_id XLOC_028736; transcript_id TCONS_00032806; exon_number 1; oId CUFF.30286.1; tss_id TSS29684;
6	Cufflinks	exon	160053924	160055102	.	+	.	gene_id GRMZM2G043695; transcript_id TCONS_00032949; exon_number 1; oId CUFF.30520.1; tss_id TSS29815;
6	Cufflinks	exon	160084248	160087779	.	+	.	gene_id AC206788.3_FG015; transcript_id TCONS_00032950; exon_number 1; oId CUFF.30535.1; tss_id TSS29816;
6	Cufflinks	exon	165444350	165445593	.	+	.	gene_id GRMZM2G048892; transcript_id TCONS_00033109; exon_number 1; oId CUFF.30801.1; tss_id TSS29963;
6	Cufflinks	exon	167024418	167025382	.	+	.	gene_id XLOC_029045; transcript_id TCONS_00033151; exon_number 1; oId CUFF.30879.1; tss_id TSS30001;
6	Cufflinks	exon	1280515	1281668	.	-	.	gene_id XLOC_029097; transcript_id TCONS_00033219; exon_number 1; oId CUFF.27305.1; tss_id TSS30057;
6	Cufflinks	exon	8109205	8109622	.	-	.	gene_id XLOC_029170; transcript_id TCONS_00033311; exon_number 1; oId CUFF.27413.1; tss_id TSS30134;
6	Cufflinks	exon	28129083	28129780	.	-	.	gene_id GRMZM2G020091; transcript_id TCONS_00033602; exon_number 1; oId CUFF.28024.1; tss_id TSS30403;
6	Cufflinks	exon	28172729	28173808	.	-	.	gene_id GRMZM2G020091; transcript_id TCONS_00033602; exon_number 2; oId CUFF.28024.1; tss_id TSS30403;
6	Cufflinks	exon	61126233	61127035	.	-	.	gene_id XLOC_029613; transcript_id TCONS_00033810; exon_number 1; oId CUFF.28310.1; tss_id TSS30589;
6	Cufflinks	exon	63696202	63697776	.	-	.	gene_id GRMZM2G069335; transcript_id TCONS_00033824; exon_number 1; oId CUFF.28304.1; tss_id TSS30603;
6	Cufflinks	exon	74071067	74072057	.	-	.	gene_id XLOC_029680; transcript_id TCONS_00033883; exon_number 1; oId CUFF.28438.1; tss_id TSS30659;
6	Cufflinks	exon	74072319	74072936	.	-	.	gene_id XLOC_029681; transcript_id TCONS_00033884; exon_number 1; oId CUFF.28439.1; tss_id TSS30660;
6	Cufflinks	exon	76847017	76847473	.	-	.	gene_id XLOC_029697; transcript_id TCONS_00033900; exon_number 1; oId CUFF.28472.1; tss_id TSS30676;
6	Cufflinks	exon	85959727	85961669	.	-	.	gene_id XLOC_029759; transcript_id TCONS_00033971; exon_number 1; oId CUFF.28603.1; tss_id TSS30739;
6	Cufflinks	exon	86345343	86347299	.	-	.	gene_id XLOC_029764; transcript_id TCONS_00033976; exon_number 1; oId CUFF.28608.1; tss_id TSS30744;
6	Cufflinks	exon	89081149	89081326	.	-	.	gene_id GRMZM2G150302; transcript_id TCONS_00034015; exon_number 1; oId CUFF.28701.1; tss_id TSS30780;
6	Cufflinks	exon	89081496	89082020	.	-	.	gene_id GRMZM2G150302; transcript_id TCONS_00034015; exon_number 2; oId CUFF.28701.1; tss_id TSS30780;
6	Cufflinks	exon	89083794	89084342	.	-	.	gene_id GRMZM2G150302; transcript_id TCONS_00034015; exon_number 3; oId CUFF.28701.1; tss_id TSS30780;
6	Cufflinks	exon	89084448	89085692	.	-	.	gene_id GRMZM2G150302; transcript_id TCONS_00034015; exon_number 4; oId CUFF.28701.1; tss_id TSS30780;
6	Cufflinks	exon	97274338	97274901	.	-	.	gene_id XLOC_029895; transcript_id TCONS_00034129; exon_number 1; oId CUFF.28883.1; tss_id TSS30877;
6	Cufflinks	exon	106612044	106612947	.	-	.	gene_id XLOC_029975; transcript_id TCONS_00034223; exon_number 1; oId CUFF.29054.1; tss_id TSS30960;
6	Cufflinks	exon	109558845	109560431	.	-	.	gene_id XLOC_030013; transcript_id TCONS_00034267; exon_number 1; oId CUFF.29134.1; tss_id TSS31000;
6	Cufflinks	exon	113900300	113901293	.	-	.	gene_id XLOC_030054; transcript_id TCONS_00034314; exon_number 1; oId CUFF.29218.1; tss_id TSS31042;
6	Cufflinks	exon	121567118	121567490	.	-	.	gene_id XLOC_030139; transcript_id TCONS_00034406; exon_number 1; oId CUFF.29385.1; tss_id TSS31128;
6	Cufflinks	exon	129268701	129270059	.	-	.	gene_id XLOC_030226; transcript_id TCONS_00034515; exon_number 1; oId CUFF.29549.1; tss_id TSS31219;
6	Cufflinks	exon	132410072	132411648	.	-	.	gene_id XLOC_030279; transcript_id TCONS_00034571; exon_number 1; oId CUFF.29636.1; tss_id TSS31273;
6	Cufflinks	exon	138774979	138776272	.	-	.	gene_id XLOC_030347; transcript_id TCONS_00034652; exon_number 1; oId CUFF.29781.1; tss_id TSS31341;
6	Cufflinks	exon	148063742	148064148	.	-	.	gene_id XLOC_030490; transcript_id TCONS_00034810; exon_number 1; oId CUFF.30051.1; tss_id TSS31488;
6	Cufflinks	exon	149279487	149282523	.	-	.	gene_id XLOC_030512; transcript_id TCONS_00034835; exon_number 1; oId CUFF.30097.1; tss_id TSS31510;
6	Cufflinks	exon	152066408	152067533	.	-	.	gene_id GRMZM2G143745; transcript_id TCONS_00034900; exon_number 1; oId CUFF.30186.1; tss_id TSS31564;
6	Cufflinks	exon	154430572	154432792	.	-	.	gene_id GRMZM2G390436; transcript_id TCONS_00034951; exon_number 1; oId CUFF.30285.1; tss_id TSS31609;
6	Cufflinks	exon	155074555	155076308	.	-	.	gene_id XLOC_030619; transcript_id TCONS_00034964; exon_number 1; oId CUFF.30308.1; tss_id TSS31622;
6	Cufflinks	exon	156404946	156405356	.	-	.	gene_id XLOC_030654; transcript_id TCONS_00035006; exon_number 1; oId CUFF.30390.1; tss_id TSS31657;
6	Cufflinks	exon	160053752	160055101	.	-	.	gene_id XLOC_030722; transcript_id TCONS_00035086; exon_number 1; oId CUFF.30519.1; tss_id TSS31728;

6	Cufflinks	exon	165443968	165445433	.	-	.	gene_id XLOC_030858; transcript_id TCONS_00035237; exon_number 1; oId CUFF.30800.1; tss_id TSS31866;
6	Cufflinks	exon	167776365	167776826	.	-	.	gene_id XLOC_030910; transcript_id TCONS_00035296; exon_number 1; oId CUFF.30932.1; tss_id TSS31919;
6	Cufflinks	exon	168167423	168170200	.	-	.	gene_id GRMZM2G156905; transcript_id TCONS_00035309; exon_number 1; oId CUFF.30927.2; tss_id TSS31930;
7	Cufflinks	exon	3252351	3253259	.	+	.	gene_id GRMZM2G456997; transcript_id TCONS_00035381; exon_number 1; oId CUFF.31066.1; tss_id TSS31997;
7	Cufflinks	exon	6922475	6924247	.	+	.	gene_id GRMZM2G406014; transcript_id TCONS_00035436; exon_number 1; oId CUFF.31159.1; tss_id TSS32049;
7	Cufflinks	exon	13208122	13208779	.	+	.	gene_id XLOC_031109; transcript_id TCONS_00035516; exon_number 1; oId CUFF.31364.1; tss_id TSS32125;
7	Cufflinks	exon	13624455	13625156	.	+	.	gene_id XLOC_031116; transcript_id TCONS_00035524; exon_number 1; oId CUFF.31316.1; tss_id TSS32132;
7	Cufflinks	exon	23365115	23366572	.	+	.	gene_id XLOC_031193; transcript_id TCONS_00035606; exon_number 1; oId CUFF.31481.1; tss_id TSS32209;
7	Cufflinks	exon	103538461	103539271	.	+	.	gene_id XLOC_031561; transcript_id TCONS_00036022; exon_number 1; oId CUFF.32227.1; tss_id TSS32587;
7	Cufflinks	exon	104202160	104204790	.	+	.	gene_id XLOC_031565; transcript_id TCONS_00036026; exon_number 1; oId CUFF.32224.1; tss_id TSS32591;
7	Cufflinks	exon	109356210	109357565	.	+	.	gene_id XLOC_031596; transcript_id TCONS_00036058; exon_number 1; oId CUFF.32304.1; tss_id TSS32622;
7	Cufflinks	exon	115719873	115722227	.	+	.	gene_id XLOC_031629; transcript_id TCONS_00036093; exon_number 1; oId CUFF.32367.1; tss_id TSS32655;
7	Cufflinks	exon	119943201	119944169	.	+	.	gene_id GRMZM2G071959; transcript_id TCONS_00036130; exon_number 1; oId CUFF.32422.1; tss_id TSS32690;
7	Cufflinks	exon	121804250	121804694	.	+	.	gene_id XLOC_031687; transcript_id TCONS_00036159; exon_number 1; oId CUFF.32451.1; tss_id TSS32717;
7	Cufflinks	exon	132660472	132661613	.	+	.	gene_id GRMZM2G083932; transcript_id TCONS_00036288; exon_number 1; oId CUFF.32665.1; tss_id TSS32835;
7	Cufflinks	exon	135625966	135627055	.	+	.	gene_id XLOC_031833; transcript_id TCONS_00036329; exon_number 1; oId CUFF.32748.1; tss_id TSS32870;
7	Cufflinks	exon	151560171	151562048	.	+	.	gene_id XLOC_032049; transcript_id TCONS_00036582; exon_number 1; oId CUFF.33189.1; tss_id TSS33095;
7	Cufflinks	exon	158406545	158409322	.	+	.	gene_id GRMZM2G106548; transcript_id TCONS_00036715; exon_number 1; oId CUFF.33419.1; tss_id TSS33211;
7	Cufflinks	exon	165383657	165384304	.	+	.	gene_id XLOC_032287; transcript_id TCONS_00036854; exon_number 1; oId CUFF.33692.1; tss_id TSS33340;
7	Cufflinks	exon	167539708	167541966	.	+	.	gene_id AC195864.3_FG017; transcript_id TCONS_00036901; exon_number 1; oId CUFF.33757.1; tss_id TSS33383;
7	Cufflinks	exon	169977981	169978650	.	+	.	gene_id XLOC_032383; transcript_id TCONS_00036959; exon_number 1; oId CUFF.33851.1; tss_id TSS33438;
7	Cufflinks	exon	172543187	172544780	.	+	.	gene_id XLOC_032447; transcript_id TCONS_00037032; exon_number 1; oId CUFF.33982.1; tss_id TSS33506;
7	Cufflinks	exon	174630036	174630579	.	+	.	gene_id XLOC_032498; transcript_id TCONS_00037092; exon_number 1; oId CUFF.34093.1; tss_id TSS33560;
7	Cufflinks	exon	2296054	2297237	.	-	.	gene_id GRMZM2G458082; transcript_id TCONS_00037176; exon_number 1; oId CUFF.31021.1; tss_id TSS33633;
7	Cufflinks	exon	6922617	6923867	.	-	.	gene_id XLOC_032635; transcript_id TCONS_00037245; exon_number 1; oId CUFF.31160.1; tss_id TSS33701;
7	Cufflinks	exon	31098451	31099106	.	-	.	gene_id XLOC_032835; transcript_id TCONS_00037471; exon_number 1; oId CUFF.31570.1; tss_id TSS33905;
7	Cufflinks	exon	38403781	38404431	.	-	.	gene_id GRMZM2G140394; transcript_id TCONS_00037502; exon_number 1; oId CUFF.31654.2; tss_id TSS33935;
7	Cufflinks	exon	38406109	38407836	.	-	.	gene_id GRMZM2G140394; transcript_id TCONS_00037502; exon_number 2; oId CUFF.31654.2; tss_id TSS33935;
7	Cufflinks	exon	38407943	38408311	.	-	.	gene_id GRMZM2G140394; transcript_id TCONS_00037502; exon_number 3; oId CUFF.31654.2; tss_id TSS33935;
7	Cufflinks	exon	38403781	38404431	.	-	.	gene_id GRMZM2G140394; transcript_id TCONS_00037503; exon_number 1; oId CUFF.31654.1; tss_id TSS33935;
7	Cufflinks	exon	38406109	38407836	.	-	.	gene_id GRMZM2G140394; transcript_id TCONS_00037503; exon_number 2; oId CUFF.31654.1; tss_id TSS33935;
7	Cufflinks	exon	38407935	38408314	.	-	.	gene_id GRMZM2G140394; transcript_id TCONS_00037503; exon_number 3; oId CUFF.31654.1; tss_id TSS33935;
7	Cufflinks	exon	51486818	51488870	.	-	.	gene_id XLOC_032924; transcript_id TCONS_00037574; exon_number 1; oId CUFF.31782.1; tss_id TSS33997;
7	Cufflinks	exon	85288917	85290497	.	-	.	gene_id XLOC_033047; transcript_id TCONS_00037705; exon_number 1; oId CUFF.32001.1; tss_id TSS34121;
7	Cufflinks	exon	117728776	117730447	.	-	.	gene_id XLOC_033238; transcript_id TCONS_00037920; exon_number 1; oId CUFF.32390.1; tss_id TSS34317;
7	Cufflinks	exon	119942997	119943754	.	-	.	gene_id XLOC_033257; transcript_id TCONS_00037941; exon_number 1; oId CUFF.32421.1; tss_id TSS34336;

7	Cufflinks	exon	121804250	121804951	.	-	.	gene_id XLOC_033273; transcript_id TCONS_00037958; exon_number 1; oId CUFF.32452.1; tss_id TSS34352;
7	Cufflinks	exon	132660271	132661577	.	-	.	gene_id XLOC_033371; transcript_id TCONS_00038061; exon_number 1; oId CUFF.32664.1; tss_id TSS34450;
7	Cufflinks	exon	140460238	140461566	.	-	.	gene_id XLOC_033463; transcript_id TCONS_00038160; exon_number 1; oId CUFF.32857.1; tss_id TSS34544;
7	Cufflinks	exon	143288306	143289828	.	-	.	gene_id GRMZM2G335111; transcript_id TCONS_00038212; exon_number 1; oId CUFF.32954.1; tss_id TSS34593;
7	Cufflinks	exon	151122730	151124888	.	-	.	gene_id XLOC_033631; transcript_id TCONS_00038347; exon_number 1; oId CUFF.33177.1; tss_id TSS34715;
7	Cufflinks	exon	153211543	153213159	.	-	.	gene_id GRMZM2G449709; transcript_id TCONS_00038377; exon_number 1; oId CUFF.33214.1; tss_id TSS34745;
7	Cufflinks	exon	153330572	153331553	.	-	.	gene_id XLOC_033662; transcript_id TCONS_00038381; exon_number 1; oId CUFF.33229.1; tss_id TSS34749;
7	Cufflinks	exon	154224100	154225746	.	-	.	gene_id GRMZM2G357631; transcript_id TCONS_00038396; exon_number 1; oId CUFF.33258.1; tss_id TSS34763;
7	Cufflinks	exon	158405601	158408516	.	-	.	gene_id GRMZM5G897592; transcript_id TCONS_00038482; exon_number 1; oId CUFF.33416.1; tss_id TSS34845;
7	Cufflinks	exon	160254346	160255890	.	-	.	gene_id XLOC_033790; transcript_id TCONS_00038523; exon_number 1; oId CUFF.33485.1; tss_id TSS34884;
7	Cufflinks	exon	161941387	161943936	.	-	.	gene_id XLOC_033820; transcript_id TCONS_00038554; exon_number 1; oId CUFF.33544.1; tss_id TSS34914;
7	Cufflinks	exon	171769839	171771033	.	-	.	gene_id XLOC_034006; transcript_id TCONS_00038758; exon_number 1; oId CUFF.33933.1; tss_id TSS35107;
7	Cufflinks	exon	171863280	171865189	.	-	.	gene_id XLOC_034009; transcript_id TCONS_00038762; exon_number 1; oId CUFF.33935.1; tss_id TSS35110;
7	Cufflinks	exon	172543068	172543892	.	-	.	gene_id GRMZM2G414460; transcript_id TCONS_00038775; exon_number 1; oId CUFF.33981.2; tss_id TSS35122;
7	Cufflinks	exon	172543988	172544802	.	-	.	gene_id GRMZM2G414460; transcript_id TCONS_00038775; exon_number 2; oId CUFF.33981.2; tss_id TSS35122;
7	Cufflinks	exon	172545617	172546675	.	-	.	gene_id GRMZM2G414460; transcript_id TCONS_00038775; exon_number 3; oId CUFF.33981.2; tss_id TSS35122;
8	Cufflinks	exon	704959	705716	.	+	.	gene_id GRMZM2G386987; transcript_id TCONS_00038896; exon_number 1; oId CUFF.34157.1; tss_id TSS35231;
8	Cufflinks	exon	4969642	4970267	.	+	.	gene_id GRMZM2G134941; transcript_id TCONS_00038966; exon_number 1; oId CUFF.34267.1; tss_id TSS35289;
8	Cufflinks	exon	6359315	6360734	.	+	.	gene_id XLOC_034207; transcript_id TCONS_00038995; exon_number 1; oId CUFF.34303.1; tss_id TSS35314;
8	Cufflinks	exon	6920014	6921235	.	+	.	gene_id GRMZM2G110504; transcript_id TCONS_00039013; exon_number 1; oId CUFF.34323.1; tss_id TSS35330;
8	Cufflinks	exon	13374182	13374574	.	+	.	gene_id XLOC_034316; transcript_id TCONS_00039116; exon_number 1; oId CUFF.34505.1; tss_id TSS35427;
8	Cufflinks	exon	14730093	14730739	.	+	.	gene_id GRMZM2G110063; transcript_id TCONS_00039143; exon_number 1; oId CUFF.34596.2; tss_id TSS35448;
8	Cufflinks	exon	14777752	14777862	.	+	.	gene_id GRMZM2G110063; transcript_id TCONS_00039143; exon_number 2; oId CUFF.34596.2; tss_id TSS35448;
8	Cufflinks	exon	14777957	14778031	.	+	.	gene_id GRMZM2G110063; transcript_id TCONS_00039143; exon_number 3; oId CUFF.34596.2; tss_id TSS35448;
8	Cufflinks	exon	14778157	14778217	.	+	.	gene_id GRMZM2G110063; transcript_id TCONS_00039143; exon_number 4; oId CUFF.34596.2; tss_id TSS35448;
8	Cufflinks	exon	14778398	14778471	.	+	.	gene_id GRMZM2G110063; transcript_id TCONS_00039143; exon_number 5; oId CUFF.34596.2; tss_id TSS35448;
8	Cufflinks	exon	14778548	14778652	.	+	.	gene_id GRMZM2G110063; transcript_id TCONS_00039143; exon_number 6; oId CUFF.34596.2; tss_id TSS35448;
8	Cufflinks	exon	14779050	14779157	.	+	.	gene_id GRMZM2G110063; transcript_id TCONS_00039143; exon_number 7; oId CUFF.34596.2; tss_id TSS35448;
8	Cufflinks	exon	14779238	14779341	.	+	.	gene_id GRMZM2G110063; transcript_id TCONS_00039143; exon_number 8; oId CUFF.34596.2; tss_id TSS35448;
8	Cufflinks	exon	14779518	14779661	.	+	.	gene_id GRMZM2G110063; transcript_id TCONS_00039143; exon_number 9; oId CUFF.34596.2; tss_id TSS35448;
8	Cufflinks	exon	14779745	14779874	.	+	.	gene_id GRMZM2G110063; transcript_id TCONS_00039143; exon_number 10; oId CUFF.34596.2; tss_id TSS35448;
8	Cufflinks	exon	14780018	14780164	.	+	.	gene_id GRMZM2G110063; transcript_id TCONS_00039143; exon_number 11; oId CUFF.34596.2; tss_id TSS35448;
8	Cufflinks	exon	14780357	14780789	.	+	.	gene_id GRMZM2G110063; transcript_id TCONS_00039143; exon_number 12; oId CUFF.34596.2; tss_id TSS35448;
8	Cufflinks	exon	14730093	14730739	.	+	.	gene_id GRMZM2G110063; transcript_id TCONS_00039142; exon_number 1; oId CUFF.34596.1; tss_id TSS35448;
8	Cufflinks	exon	14777752	14777862	.	+	.	gene_id GRMZM2G110063; transcript_id TCONS_00039142; exon_number 2; oId CUFF.34596.1; tss_id TSS35448;
8	Cufflinks	exon	14777957	14778031	.	+	.	gene_id GRMZM2G110063; transcript_id TCONS_00039142; exon_number 3; oId CUFF.34596.1; tss_id TSS35448;

8	Cufflinks	exon	14778157	14778217	.	+	.	gene_id GRMZM2G110063; transcript_id TCONS_00039142; exon_number 4; oId CUFF.34596.1; tss_id TSS35448;
8	Cufflinks	exon	14778398	14778471	.	+	.	gene_id GRMZM2G110063; transcript_id TCONS_00039142; exon_number 5; oId CUFF.34596.1; tss_id TSS35448;
8	Cufflinks	exon	14778548	14778652	.	+	.	gene_id GRMZM2G110063; transcript_id TCONS_00039142; exon_number 6; oId CUFF.34596.1; tss_id TSS35448;
8	Cufflinks	exon	14779050	14779157	.	+	.	gene_id GRMZM2G110063; transcript_id TCONS_00039142; exon_number 7; oId CUFF.34596.1; tss_id TSS35448;
8	Cufflinks	exon	14779238	14779341	.	+	.	gene_id GRMZM2G110063; transcript_id TCONS_00039142; exon_number 8; oId CUFF.34596.1; tss_id TSS35448;
8	Cufflinks	exon	14779518	14779661	.	+	.	gene_id GRMZM2G110063; transcript_id TCONS_00039142; exon_number 9; oId CUFF.34596.1; tss_id TSS35448;
8	Cufflinks	exon	14779745	14779874	.	+	.	gene_id GRMZM2G110063; transcript_id TCONS_00039142; exon_number 10; oId CUFF.34596.1; tss_id TSS35448;
8	Cufflinks	exon	14780021	14780164	.	+	.	gene_id GRMZM2G110063; transcript_id TCONS_00039142; exon_number 11; oId CUFF.34596.1; tss_id TSS35448;
8	Cufflinks	exon	14780357	14780789	.	+	.	gene_id GRMZM2G110063; transcript_id TCONS_00039142; exon_number 12; oId CUFF.34596.1; tss_id TSS35448;
8	Cufflinks	exon	23427241	23428339	.	+	.	gene_id GRMZM2G472654; transcript_id TCONS_00039243; exon_number 1; oId CUFF.34781.1; tss_id TSS35543;
8	Cufflinks	exon	26635509	26636651	.	+	.	gene_id GRMZM2G413717; transcript_id TCONS_00039273; exon_number 1; oId CUFF.34807.1; tss_id TSS35568;
8	Cufflinks	exon	26865699	26866324	.	+	.	gene_id XLOC_034457; transcript_id TCONS_00039276; exon_number 1; oId CUFF.34814.1; tss_id TSS35571;
8	Cufflinks	exon	51816725	51817436	.	+	.	gene_id XLOC_034562; transcript_id TCONS_00039389; exon_number 1; oId CUFF.35031.1; tss_id TSS35676;
8	Cufflinks	exon	64222114	64223758	.	+	.	gene_id GRMZM2G159592; transcript_id TCONS_00039459; exon_number 1; oId CUFF.35147.1; tss_id TSS35743;
8	Cufflinks	exon	94310000	94311063	.	+	.	gene_id XLOC_034847; transcript_id TCONS_00039718; exon_number 1; oId CUFF.35566.1; tss_id TSS35970;
8	Cufflinks	exon	96331887	96333277	.	+	.	gene_id XLOC_034864; transcript_id TCONS_00039742; exon_number 1; oId CUFF.35608.1; tss_id TSS35988;
8	Cufflinks	exon	109361001	109361451	.	+	.	gene_id XLOC_034971; transcript_id TCONS_00039856; exon_number 1; oId CUFF.35842.1; tss_id TSS36097;
8	Cufflinks	exon	119461775	119462949	.	+	.	gene_id XLOC_035081; transcript_id TCONS_00039979; exon_number 1; oId CUFF.36035.1; tss_id TSS36208;
8	Cufflinks	exon	128075569	128075748	.	+	.	gene_id GRMZM2G700655; transcript_id TCONS_00040082; exon_number 1; oId CUFF.36225.1; tss_id TSS36304;
8	Cufflinks	exon	128077285	128077736	.	+	.	gene_id GRMZM2G700655; transcript_id TCONS_00040082; exon_number 2; oId CUFF.36225.1; tss_id TSS36304;
8	Cufflinks	exon	128077814	128078863	.	+	.	gene_id GRMZM2G700655; transcript_id TCONS_00040082; exon_number 3; oId CUFF.36225.1; tss_id TSS36304;
8	Cufflinks	exon	128079309	128081020	.	+	.	gene_id GRMZM2G700655; transcript_id TCONS_00040082; exon_number 4; oId CUFF.36225.1; tss_id TSS36304;
8	Cufflinks	exon	128081126	128081851	.	+	.	gene_id GRMZM2G700655; transcript_id TCONS_00040082; exon_number 5; oId CUFF.36225.1; tss_id TSS36304;
8	Cufflinks	exon	133999615	134000856	.	+	.	gene_id XLOC_035246; transcript_id TCONS_00040167; exon_number 1; oId CUFF.36372.2; tss_id TSS36380;
8	Cufflinks	exon	151802508	151802839	.	+	.	gene_id XLOC_035495; transcript_id TCONS_00040471; exon_number 1; oId CUFF.36825.1; tss_id TSS36637;
8	Cufflinks	exon	152497093	152498604	.	+	.	gene_id GRMZM2G439350; transcript_id TCONS_00040493; exon_number 1; oId CUFF.36834.1; tss_id TSS36651;
8	Cufflinks	exon	163927153	163928646	.	+	.	gene_id XLOC_035716; transcript_id TCONS_00040732; exon_number 1; oId CUFF.37286.1; tss_id TSS36863;
8	Cufflinks	exon	166203213	166204219	.	+	.	gene_id XLOC_035767; transcript_id TCONS_00040792; exon_number 1; oId CUFF.37376.1; tss_id TSS36915;
8	Cufflinks	exon	166742338	166743981	.	+	.	gene_id XLOC_035780; transcript_id TCONS_00040806; exon_number 1; oId CUFF.37411.1; tss_id TSS36928;
8	Cufflinks	exon	168604741	168605617	.	+	.	gene_id GRMZM2G112912; transcript_id TCONS_00040845; exon_number 1; oId CUFF.37486.1; tss_id TSS36966;
8	Cufflinks	exon	170180913	170181318	.	+	.	gene_id XLOC_035857; transcript_id TCONS_00040888; exon_number 1; oId CUFF.37566.1; tss_id TSS37008;
8	Cufflinks	exon	171684881	171685894	.	+	.	gene_id XLOC_035902; transcript_id TCONS_00040938; exon_number 1; oId CUFF.37668.1; tss_id TSS37056;
8	Cufflinks	exon	6359079	6360548	.	-	.	gene_id GRMZM2G058404; transcript_id TCONS_00041111; exon_number 1; oId CUFF.34302.1; tss_id TSS37218;
8	Cufflinks	exon	6920162	6920992	.	-	.	gene_id XLOC_036063; transcript_id TCONS_00041117; exon_number 1; oId CUFF.34324.1; tss_id TSS37224;
8	Cufflinks	exon	15108533	15109664	.	-	.	gene_id AC217965.2_FG012; transcript_id TCONS_00041232; exon_number 1; oId CUFF.34549.1; tss_id TSS37332;
8	Cufflinks	exon	17178910	17179303	.	-	.	gene_id XLOC_036196; transcript_id TCONS_00041265; exon_number 1; oId CUFF.34613.1; tss_id TSS37363;

8	Cufflinks	exon	26635609	26636105	.	-	.	gene_id XLOC_036307; transcript_id TCONS_00041397; exon_number 1; oId CUFF.34808.1; tss_id TSS37476;
8	Cufflinks	exon	55843374	55846170	.	-	.	gene_id GRMZM2G339206; transcript_id TCONS_00041554; exon_number 1; oId CUFF.35079.1; tss_id TSS37610;
8	Cufflinks	exon	64222075	64223671	.	-	.	gene_id XLOC_036481; transcript_id TCONS_00041603; exon_number 1; oId CUFF.35146.1; tss_id TSS37656;
8	Cufflinks	exon	73369546	73370340	.	-	.	gene_id GRMZM2G002034; transcript_id TCONS_00041677; exon_number 1; oId CUFF.35314.1; tss_id TSS37727;
8	Cufflinks	exon	73370419	73370609	.	-	.	gene_id GRMZM2G002034; transcript_id TCONS_00041677; exon_number 2; oId CUFF.35314.1; tss_id TSS37727;
8	Cufflinks	exon	73370693	73370900	.	-	.	gene_id GRMZM2G002034; transcript_id TCONS_00041677; exon_number 3; oId CUFF.35314.1; tss_id TSS37727;
8	Cufflinks	exon	73371001	73371210	.	-	.	gene_id GRMZM2G002034; transcript_id TCONS_00041677; exon_number 4; oId CUFF.35314.1; tss_id TSS37727;
8	Cufflinks	exon	73371711	73371842	.	-	.	gene_id GRMZM2G002034; transcript_id TCONS_00041677; exon_number 5; oId CUFF.35314.1; tss_id TSS37727;
8	Cufflinks	exon	73372048	73372613	.	-	.	gene_id GRMZM2G002034; transcript_id TCONS_00041677; exon_number 6; oId CUFF.35314.1; tss_id TSS37727;
8	Cufflinks	exon	118795818	118797252	.	-	.	gene_id GRMZM2G138817; transcript_id TCONS_00042057; exon_number 1; oId CUFF.36018.1; tss_id TSS38077;
8	Cufflinks	exon	121416852	121418183	.	-	.	gene_id XLOC_036937; transcript_id TCONS_00042104; exon_number 1; oId CUFF.36082.1; tss_id TSS38121;
8	Cufflinks	exon	126231541	126232916	.	-	.	gene_id GRMZM2G150434; transcript_id TCONS_00042168; exon_number 1; oId CUFF.36191.1; tss_id TSS38180;
8	Cufflinks	exon	132540044	132541658	.	-	.	gene_id GRMZM2G080191; transcript_id TCONS_00042237; exon_number 1; oId CUFF.36320.1; tss_id TSS38242;
8	Cufflinks	exon	152497944	152498597	.	-	.	gene_id XLOC_037288; transcript_id TCONS_00042519; exon_number 1; oId CUFF.36835.1; tss_id TSS38483;
8	Cufflinks	exon	162305487	162306280	.	-	.	gene_id GRMZM2G170969; transcript_id TCONS_00042725; exon_number 1; oId CUFF.37194.1; tss_id TSS38674;
8	Cufflinks	exon	162588472	162589576	.	-	.	gene_id XLOC_037480; transcript_id TCONS_00042735; exon_number 1; oId CUFF.37211.1; tss_id TSS38684;
8	Cufflinks	exon	166450439	166452505	.	-	.	gene_id XLOC_037564; transcript_id TCONS_00042842; exon_number 1; oId CUFF.37395.1; tss_id TSS38770;
8	Cufflinks	exon	168604901	168605361	.	-	.	gene_id XLOC_037618; transcript_id TCONS_00042900; exon_number 1; oId CUFF.37487.1; tss_id TSS38825;
8	Cufflinks	exon	168614934	168616983	.	-	.	gene_id XLOC_037620; transcript_id TCONS_00042902; exon_number 1; oId CUFF.37493.1; tss_id TSS38827;
8	Cufflinks	exon	169208525	169209009	.	-	.	gene_id GRMZM2G311220; transcript_id TCONS_00042929; exon_number 1; oId CUFF.37545.2; tss_id TSS38850;
8	Cufflinks	exon	169209114	169210927	.	-	.	gene_id GRMZM2G311220; transcript_id TCONS_00042929; exon_number 2; oId CUFF.37545.2; tss_id TSS38850;
8	Cufflinks	exon	169211043	169211158	.	-	.	gene_id GRMZM2G311220; transcript_id TCONS_00042929; exon_number 3; oId CUFF.37545.2; tss_id TSS38850;
8	Cufflinks	exon	169211618	169211770	.	-	.	gene_id GRMZM2G311220; transcript_id TCONS_00042929; exon_number 4; oId CUFF.37545.2; tss_id TSS38850;
8	Cufflinks	exon	169211848	169211991	.	-	.	gene_id GRMZM2G311220; transcript_id TCONS_00042929; exon_number 5; oId CUFF.37545.2; tss_id TSS38850;
8	Cufflinks	exon	169214533	169215320	.	-	.	gene_id GRMZM2G311220; transcript_id TCONS_00042929; exon_number 6; oId CUFF.37545.2; tss_id TSS38850;
8	Cufflinks	exon	170417531	170420149	.	-	.	gene_id GRMZM2G069078; transcript_id TCONS_00042961; exon_number 1; oId CUFF.37585.1; tss_id TSS38879;
8	Cufflinks	exon	171307811	171308656	.	-	.	gene_id XLOC_037695; transcript_id TCONS_00042989; exon_number 1; oId CUFF.37638.1; tss_id TSS38905;
8	Cufflinks	exon	172627115	172627558	.	-	.	gene_id XLOC_037736; transcript_id TCONS_00043039; exon_number 1; oId CUFF.37709.1; tss_id TSS38950;
8	Cufflinks	exon	173488303	173489765	.	-	.	gene_id GRMZM2G428179; transcript_id TCONS_00043053; exon_number 1; oId CUFF.37748.1; tss_id TSS38964;
9	Cufflinks	exon	9955397	9956538	.	+	.	gene_id XLOC_037884; transcript_id TCONS_00043210; exon_number 1; oId CUFF.38014.1; tss_id TSS39107;
9	Cufflinks	exon	13663138	13664819	.	+	.	gene_id GRMZM2G008305; transcript_id TCONS_00043256; exon_number 1; oId CUFF.38113.1; tss_id TSS39147;
9	Cufflinks	exon	18958599	18959447	.	+	.	gene_id XLOC_038013; transcript_id TCONS_00043357; exon_number 1; oId CUFF.38278.1; tss_id TSS39241;
9	Cufflinks	exon	22170770	22173445	.	+	.	gene_id XLOC_038068; transcript_id TCONS_00043421; exon_number 1; oId CUFF.38388.1; tss_id TSS39298;
9	Cufflinks	exon	24558570	24560564	.	+	.	gene_id GRMZM2G304528; transcript_id TCONS_00043448; exon_number 1; oId CUFF.38439.1; tss_id TSS39323;
9	Cufflinks	exon	32305175	32305327	.	+	.	gene_id XLOC_038172; transcript_id TCONS_00043539; exon_number 1; oId CUFF.38606.1; tss_id TSS39403;
9	Cufflinks	exon	32305421	32305638	.	+	.	gene_id XLOC_038172; transcript_id TCONS_00043539; exon_number 2; oId CUFF.38606.1; tss_id TSS39403;

9	Cufflinks	exon	32305754	32306322	.	+	.	gene_id XLOC_038172; transcript_id TCONS_00043539; exon_number 3; oId CUFF.38606.1; tss_id TSS39403;
9	Cufflinks	exon	32306866	32308115	.	+	.	gene_id XLOC_038172; transcript_id TCONS_00043539; exon_number 4; oId CUFF.38606.1; tss_id TSS39403;
9	Cufflinks	exon	32308200	32308972	.	+	.	gene_id XLOC_038173; transcript_id TCONS_00043541; exon_number 1; oId CUFF.38591.1; tss_id TSS39405;
9	Cufflinks	exon	100766352	100766946	.	+	.	gene_id GRMZM2G092493; transcript_id TCONS_00043955; exon_number 1; oId CUFF.39343.1; tss_id TSS39787;
9	Cufflinks	exon	100767538	100767661	.	+	.	gene_id GRMZM2G092493; transcript_id TCONS_00043955; exon_number 2; oId CUFF.39343.1; tss_id TSS39787;
9	Cufflinks	exon	100769020	100769146	.	+	.	gene_id GRMZM2G092493; transcript_id TCONS_00043955; exon_number 3; oId CUFF.39343.1; tss_id TSS39787;
9	Cufflinks	exon	100769369	100769405	.	+	.	gene_id GRMZM2G092493; transcript_id TCONS_00043955; exon_number 4; oId CUFF.39343.1; tss_id TSS39787;
9	Cufflinks	exon	100769480	100769705	.	+	.	gene_id GRMZM2G092493; transcript_id TCONS_00043955; exon_number 5; oId CUFF.39343.1; tss_id TSS39787;
9	Cufflinks	exon	100769829	100769946	.	+	.	gene_id GRMZM2G092493; transcript_id TCONS_00043955; exon_number 6; oId CUFF.39343.1; tss_id TSS39787;
9	Cufflinks	exon	100770020	100770117	.	+	.	gene_id GRMZM2G092493; transcript_id TCONS_00043955; exon_number 7; oId CUFF.39343.1; tss_id TSS39787;
9	Cufflinks	exon	100770208	100770684	.	+	.	gene_id GRMZM2G092493; transcript_id TCONS_00043955; exon_number 8; oId CUFF.39343.1; tss_id TSS39787;
9	Cufflinks	exon	104241227	104241444	.	+	.	gene_id GRMZM2G086773; transcript_id TCONS_00043992; exon_number 1; oId CUFF.39395.1; tss_id TSS39823;
9	Cufflinks	exon	104241535	104241715	.	+	.	gene_id GRMZM2G086773; transcript_id TCONS_00043992; exon_number 2; oId CUFF.39395.1; tss_id TSS39823;
9	Cufflinks	exon	104241897	104242124	.	+	.	gene_id GRMZM2G086773; transcript_id TCONS_00043992; exon_number 3; oId CUFF.39395.1; tss_id TSS39823;
9	Cufflinks	exon	104242256	104244459	.	+	.	gene_id GRMZM2G086773; transcript_id TCONS_00043992; exon_number 4; oId CUFF.39395.1; tss_id TSS39823;
9	Cufflinks	exon	108225231	108228267	.	+	.	gene_id GRMZM2G405474; transcript_id TCONS_00044019; exon_number 1; oId CUFF.39457.1; tss_id TSS39847;
9	Cufflinks	exon	108316263	108318243	.	+	.	gene_id GRMZM2G151496; transcript_id TCONS_00044021; exon_number 1; oId CUFF.39454.1; tss_id TSS39849;
9	Cufflinks	exon	112025237	112028773	.	+	.	gene_id XLOC_038635; transcript_id TCONS_00044063; exon_number 1; oId CUFF.39533.1; tss_id TSS39884;
9	Cufflinks	exon	115403001	115405100	.	+	.	gene_id XLOC_038688; transcript_id TCONS_00044120; exon_number 1; oId CUFF.39617.1; tss_id TSS39937;
9	Cufflinks	exon	136732038	136733527	.	+	.	gene_id XLOC_038931; transcript_id TCONS_00044402; exon_number 1; oId CUFF.40131.1; tss_id TSS40185;
9	Cufflinks	exon	141795550	141797599	.	+	.	gene_id XLOC_039018; transcript_id TCONS_00044506; exon_number 1; oId CUFF.40300.1; tss_id TSS40276;
9	Cufflinks	exon	145604342	145606676	.	+	.	gene_id XLOC_039083; transcript_id TCONS_00044579; exon_number 1; oId CUFF.40440.1; tss_id TSS40343;
9	Cufflinks	exon	150509633	150510254	.	+	.	gene_id XLOC_039167; transcript_id TCONS_00044684; exon_number 1; oId CUFF.40631.1; tss_id TSS40434;
9	Cufflinks	exon	151417535	151418083	.	+	.	gene_id XLOC_039184; transcript_id TCONS_00044703; exon_number 1; oId CUFF.40688.1; tss_id TSS40452;
9	Cufflinks	exon	7393341	7394579	.	-	.	gene_id XLOC_039370; transcript_id TCONS_00044924; exon_number 1; oId CUFF.37946.1; tss_id TSS40645;
9	Cufflinks	exon	9512594	9512978	.	-	.	gene_id XLOC_039403; transcript_id TCONS_00044959; exon_number 1; oId CUFF.38009.1; tss_id TSS40678;
9	Cufflinks	exon	13663717	13664603	.	-	.	gene_id XLOC_039478; transcript_id TCONS_00045048; exon_number 1; oId CUFF.38114.1; tss_id TSS40755;
9	Cufflinks	exon	19040034	19040929	.	-	.	gene_id GRMZM2G028594; transcript_id TCONS_00045137; exon_number 1; oId CUFF.38294.2; tss_id TSS40830;
9	Cufflinks	exon	23221405	23221809	.	-	.	gene_id XLOC_039600; transcript_id TCONS_00045189; exon_number 1; oId CUFF.38403.1; tss_id TSS40881;
9	Cufflinks	exon	100416596	100417256	.	-	.	gene_id XLOC_040048; transcript_id TCONS_00045680; exon_number 1; oId CUFF.39321.1; tss_id TSS41337;
9	Cufflinks	exon	101311215	101312056	.	-	.	gene_id XLOC_040057; transcript_id TCONS_00045690; exon_number 1; oId CUFF.39331.1; tss_id TSS41346;
9	Cufflinks	exon	107809927	107811180	.	-	.	gene_id XLOC_040120; transcript_id TCONS_00045757; exon_number 1; oId CUFF.39460.1; tss_id TSS41410;
9	Cufflinks	exon	108317269	108317880	.	-	.	gene_id XLOC_040126; transcript_id TCONS_00045763; exon_number 1; oId CUFF.39455.1; tss_id TSS41416;
9	Cufflinks	exon	111478926	111479586	.	-	.	gene_id GRMZM2G017739; transcript_id TCONS_00045804; exon_number 1; oId CUFF.39526.1; tss_id TSS41454;
9	Cufflinks	exon	111480458	111480601	.	-	.	gene_id GRMZM2G017739; transcript_id TCONS_00045804; exon_number 2; oId CUFF.39526.1; tss_id TSS41454;
9	Cufflinks	exon	111480780	111481648	.	-	.	gene_id GRMZM2G017739; transcript_id TCONS_00045804; exon_number 3; oId CUFF.39526.1; tss_id TSS41454;

9	Cufflinks	exon	111481719	111485379	.	-	.	gene_id GRMZM2G017739; transcript_id TCONS_00045806; exon_number 1; oId CUFF.39526.4; tss_id TSS41456;
9	Cufflinks	exon	115402677	115404899	.	-	.	gene_id GRMZM2G069807; transcript_id TCONS_00045852; exon_number 1; oId CUFF.39616.1; tss_id TSS41497;
9	Cufflinks	exon	124820087	124821692	.	-	.	gene_id XLOC_040284; transcript_id TCONS_00045949; exon_number 1; oId CUFF.39805.1; tss_id TSS41582;
9	Cufflinks	exon	150509518	150510116	.	-	.	gene_id GRMZM2G036411; transcript_id TCONS_00046479; exon_number 1; oId CUFF.40629.1; tss_id TSS42059;
10	Cufflinks	exon	23221601	23221949	.	-	.	gene_id XLOC_007842; transcript_id TCONS_00008965; exon_number 1; oId CUFF.15019.1; tss_id TSS18646;
10	Cufflinks	exon	23222052	23222141	.	-	.	gene_id XLOC_007842; transcript_id TCONS_00008965; exon_number 2; oId CUFF.15019.1; tss_id TSS18646;
2	Cufflinks	exon	198424098	198424191	.	+	.	gene_id GRMZM5G887647; transcript_id TCONS_00012317; exon_number 1; oId CUFF.27997.4; tss_id TSS25380;
2	Cufflinks	exon	198424391	198424567	.	+	.	gene_id GRMZM5G887647; transcript_id TCONS_00012317; exon_number 2; oId CUFF.27997.4; tss_id TSS25380;
2	Cufflinks	exon	198424746	198424813	.	+	.	gene_id GRMZM5G887647; transcript_id TCONS_00012317; exon_number 3; oId CUFF.27997.4; tss_id TSS25380;
2	Cufflinks	exon	198424980	198425118	.	+	.	gene_id GRMZM5G887647; transcript_id TCONS_00012317; exon_number 4; oId CUFF.27997.4; tss_id TSS25380;
2	Cufflinks	exon	198425194	198427142	.	+	.	gene_id GRMZM5G887647; transcript_id TCONS_00012317; exon_number 5; oId CUFF.27997.4; tss_id TSS25380;
3	Cufflinks	exon	211402750	211403115	.	-	.	gene_id XLOC_017970; transcript_id TCONS_00020511; exon_number 1; oId CUFF.39133.1; tss_id TSS42195;
5	Cufflinks	exon	162097544	162097925	.	-	.	gene_id XLOC_026373; transcript_id TCONS_00030143; exon_number 1; oId CUFF.56502.1; tss_id TSS61767;
7	Cufflinks	exon	166410567	166410751	.	-	.	gene_id XLOC_033901; transcript_id TCONS_00038640; exon_number 1; oId CUFF.74523.1; tss_id TSS79362;
7	Cufflinks	exon	166412381	166412455	.	-	.	gene_id XLOC_033901; transcript_id TCONS_00038640; exon_number 2; oId CUFF.74523.1; tss_id TSS79362;
7	Cufflinks	exon	166414117	166414219	.	-	.	gene_id XLOC_033901; transcript_id TCONS_00038640; exon_number 3; oId CUFF.74523.1; tss_id TSS79362;
1	Cufflinks	exon	644614	645582	.	+	.	gene_id XLOC_000012; transcript_id TCONS_00000013; exon_number 1; oId CUFF.73.1; tss_id TSS21;
1	Cufflinks	exon	645712	645991	.	+	.	gene_id XLOC_000012; transcript_id TCONS_00000013; exon_number 2; oId CUFF.73.1; tss_id TSS21;
1	Cufflinks	exon	979720	980331	.	+	.	gene_id GRMZM2G172183; transcript_id TCONS_00000014; exon_number 1; oId CUFF.64.1; tss_id TSS13;
1	Cufflinks	exon	980444	980563	.	+	.	gene_id GRMZM2G172183; transcript_id TCONS_00000014; exon_number 2; oId CUFF.64.1; tss_id TSS13;
1	Cufflinks	exon	980683	980799	.	+	.	gene_id GRMZM2G172183; transcript_id TCONS_00000014; exon_number 3; oId CUFF.64.1; tss_id TSS13;
1	Cufflinks	exon	980904	981041	.	+	.	gene_id GRMZM2G172183; transcript_id TCONS_00000014; exon_number 4; oId CUFF.64.1; tss_id TSS13;
1	Cufflinks	exon	981117981251	.	+	.	gene_id GRMZM2G172183; transcript_id TCONS_00000014; exon_number 5; oId CUFF.64.1; tss_id TSS13;	
1	Cufflinks	exon	981360	981539	.	+	.	gene_id GRMZM2G172183; transcript_id TCONS_00000014; exon_number 6; oId CUFF.64.1; tss_id TSS13;
1	Cufflinks	exon	981706	982118.	+	.	gene_id GRMZM2G172183; transcript_id TCONS_00000014; exon_number 7; oId CUFF.64.1; tss_id TSS13;	
1	Cufflinks	exon	982582	982701	.	+	.	gene_id GRMZM2G172183; transcript_id TCONS_00000014; exon_number 8; oId CUFF.64.1; tss_id TSS13;
1	Cufflinks	exon	983327	983449	.	+	.	gene_id GRMZM2G172183; transcript_id TCONS_00000014; exon_number 9; oId CUFF.64.1; tss_id TSS13;
1	Cufflinks	exon	984071	984175	.	+	.	gene_id GRMZM2G172183; transcript_id TCONS_00000014; exon_number 10; oId CUFF.64.1; tss_id TSS13;
1	Cufflinks	exon	984252	984396	.	+	.	gene_id GRMZM2G172183; transcript_id TCONS_00000014; exon_number 11; oId CUFF.64.1; tss_id TSS13;
1	Cufflinks	exon	988535	988696	.	+	.	gene_id GRMZM2G172183; transcript_id TCONS_00000014; exon_number 12; oId CUFF.64.1; tss_id TSS13;
1	Cufflinks	exon	988759	988997	.	+	.	gene_id GRMZM2G172183; transcript_id TCONS_00000014; exon_number 13; oId CUFF.64.1; tss_id TSS13;
1	Cufflinks	exon	989617	989649	.	+	.	gene_id GRMZM2G172183; transcript_id TCONS_00000014; exon_number 14; oId CUFF.64.1; tss_id TSS13;
1	Cufflinks	exon	989812	989893	.	+	.	gene_id GRMZM2G172183; transcript_id TCONS_00000014; exon_number 15; oId CUFF.64.1; tss_id TSS13;
1	Cufflinks	exon	989964	990124	.	+	.	gene_id GRMZM2G172183; transcript_id TCONS_00000014; exon_number 16; oId CUFF.64.1; tss_id TSS13;
1	Cufflinks	exon	990199	990414	.	+	.	gene_id GRMZM2G172183; transcript_id TCONS_00000014; exon_number 17; oId CUFF.64.1; tss_id TSS13;
1	Cufflinks	exon	990501	991166.	+	.	gene_id GRMZM2G172183; transcript_id TCONS_00000014; exon_number 18; oId CUFF.64.1; tss_id TSS13;	

1	Cufflinks	exon	999363	999549	.	+	.	gene_id GRMZM2G374779; transcript_id TCONS_00000018; exon_number 1; oId CUFF.93.1; tss_id TSS26;
1	Cufflinks	exon	999659	999743	.	+	.	gene_id GRMZM2G374779; transcript_id TCONS_00000018; exon_number 2; oId CUFF.93.1; tss_id TSS26;
1	Cufflinks	exon	999845	999896	.	+	.	gene_id GRMZM2G374779; transcript_id TCONS_00000018; exon_number 3; oId CUFF.93.1; tss_id TSS26;
1	Cufflinks	exon	999989	1000009	.	+	.	gene_id GRMZM2G374779; transcript_id TCONS_00000018; exon_number 4; oId CUFF.93.1; tss_id TSS26;
1	Cufflinks	exon	1000087	1000140	.	+	.	gene_id GRMZM2G374779; transcript_id TCONS_00000018; exon_number 5; oId CUFF.93.1; tss_id TSS26;
1	Cufflinks	exon	1000494	1000544	.	+	.	gene_id GRMZM2G374779; transcript_id TCONS_00000018; exon_number 6; oId CUFF.93.1; tss_id TSS26;
1	Cufflinks	exon	1000704	1000766	.	+	.	gene_id GRMZM2G374779; transcript_id TCONS_00000018; exon_number 7; oId CUFF.93.1; tss_id TSS26;
1	Cufflinks	exon	1001050	1001115	.	+	.	gene_id GRMZM2G374779; transcript_id TCONS_00000018; exon_number 8; oId CUFF.93.1; tss_id TSS26;
1	Cufflinks	exon	1001226	1001264	.	+	.	gene_id GRMZM2G374779; transcript_id TCONS_00000018; exon_number 9; oId CUFF.93.1; tss_id TSS26;
1	Cufflinks	exon	1001656	1001778	.	+	.	gene_id GRMZM2G374779; transcript_id TCONS_00000018; exon_number 10; oId CUFF.93.1; tss_id TSS26;
1	Cufflinks	exon	1001867	1001959	.	+	.	gene_id GRMZM2G374779; transcript_id TCONS_00000018; exon_number 11; oId CUFF.93.1; tss_id TSS26;
1	Cufflinks	exon	1002038	1002956	.	+	.	gene_id GRMZM2G374779; transcript_id TCONS_00000018; exon_number 12; oId CUFF.93.1; tss_id TSS26;
1	Cufflinks	exon	1003035	1005012	.	+	.	gene_id GRMZM2G374779; transcript_id TCONS_00000018; exon_number 13; oId CUFF.93.1; tss_id TSS26;
1	Cufflinks	exon	3125558	3125733	.	+	.	gene_id XLOC_000050; transcript_id TCONS_00000056; exon_number 1; oId CUFF.239.1; tss_id TSS112;
1	Cufflinks	exon	3126100	3126370	.	+	.	gene_id XLOC_000050; transcript_id TCONS_00000056; exon_number 2; oId CUFF.239.1; tss_id TSS112;
1	Cufflinks	exon	3126456	3126617	.	+	.	gene_id XLOC_000050; transcript_id TCONS_00000056; exon_number 3; oId CUFF.239.1; tss_id TSS112;
1	Cufflinks	exon	3126718	3126933	.	+	.	gene_id XLOC_000050; transcript_id TCONS_00000056; exon_number 4; oId CUFF.239.1; tss_id TSS112;
1	Cufflinks	exon	3127017	3127054	.	+	.	gene_id XLOC_000050; transcript_id TCONS_00000056; exon_number 5; oId CUFF.239.1; tss_id TSS112;
1	Cufflinks	exon	3127165	3127328	.	+	.	gene_id XLOC_000050; transcript_id TCONS_00000056; exon_number 6; oId CUFF.239.1; tss_id TSS112;
1	Cufflinks	exon	3127457	3127582	.	+	.	gene_id XLOC_000050; transcript_id TCONS_00000056; exon_number 7; oId CUFF.239.1; tss_id TSS112;
1	Cufflinks	exon	3127680	3127805	.	+	.	gene_id XLOC_000050; transcript_id TCONS_00000056; exon_number 8; oId CUFF.239.1; tss_id TSS112;
1	Cufflinks	exon	3127924	3127995	.	+	.	gene_id XLOC_000050; transcript_id TCONS_00000056; exon_number 9; oId CUFF.239.1; tss_id TSS112;
1	Cufflinks	exon	3128079	3128141	.	+	.	gene_id XLOC_000050; transcript_id TCONS_00000056; exon_number 10; oId CUFF.239.1; tss_id TSS112;
1	Cufflinks	exon	3128218	3128280	.	+	.	gene_id XLOC_000050; transcript_id TCONS_00000056; exon_number 11; oId CUFF.239.1; tss_id TSS112;
1	Cufflinks	exon	3128397	3128459	.	+	.	gene_id XLOC_000050; transcript_id TCONS_00000056; exon_number 12; oId CUFF.239.1; tss_id TSS112;
1	Cufflinks	exon	3128528	3128590	.	+	.	gene_id XLOC_000050; transcript_id TCONS_00000056; exon_number 13; oId CUFF.239.1; tss_id TSS112;
1	Cufflinks	exon	3128682	3129266	.	+	.	gene_id XLOC_000050; transcript_id TCONS_00000056; exon_number 14; oId CUFF.239.1; tss_id TSS112;
1	Cufflinks	exon	3128229	3128280	.	+	.	gene_id XLOC_000052; transcript_id TCONS_00000058; exon_number 1; oId CUFF.105.1; tss_id TSS53;
1	Cufflinks	exon	3128397	3128590	.	+	.	gene_id XLOC_000052; transcript_id TCONS_00000058; exon_number 2; oId CUFF.105.1; tss_id TSS53;
1	Cufflinks	exon	3128682	3130830	.	+	.	gene_id XLOC_000052; transcript_id TCONS_00000058; exon_number 3; oId CUFF.105.1; tss_id TSS53;
1	Cufflinks	exon	4765059	4766393	.	+	.	gene_id XLOC_000098; transcript_id TCONS_00000107; exon_number 1; oId CUFF.207.1; tss_id TSS101;
1	Cufflinks	exon	4855614	4855925	.	+	.	gene_id XLOC_000099; transcript_id TCONS_00000108; exon_number 1; oId CUFF.474.1; tss_id TSS221;
1	Cufflinks	exon	4856078	4856351	.	+	.	gene_id XLOC_000099; transcript_id TCONS_00000108; exon_number 2; oId CUFF.474.1; tss_id TSS221;
1	Cufflinks	exon	4856477	4856626	.	+	.	gene_id XLOC_000099; transcript_id TCONS_00000108; exon_number 3; oId CUFF.474.1; tss_id TSS221;
1	Cufflinks	exon	4856757	4856994	.	+	.	gene_id XLOC_000099; transcript_id TCONS_00000108; exon_number 4; oId CUFF.474.1; tss_id TSS221;
1	Cufflinks	exon	4857071	4857299	.	+	.	gene_id XLOC_000099; transcript_id TCONS_00000108; exon_number 5; oId CUFF.474.1; tss_id TSS221;

1	Cufflinks	exon	5080788	5081210	.	+	.	gene_id GRMZM2G327059; transcript_id TCONS_00000117; exon_number 1; oId CUFF.240.2; tss_id TSS109;
1	Cufflinks	exon	5081612	5081720	.	+	.	gene_id GRMZM2G327059; transcript_id TCONS_00000117; exon_number 2; oId CUFF.240.2; tss_id TSS109;
1	Cufflinks	exon	5081860	5082635	.	+	.	gene_id GRMZM2G327059; transcript_id TCONS_00000117; exon_number 3; oId CUFF.240.2; tss_id TSS109;
1	Cufflinks	exon	5083676	5084061	.	+	.	gene_id GRMZM2G327059; transcript_id TCONS_00000117; exon_number 4; oId CUFF.240.2; tss_id TSS109;
1	Cufflinks	exon	5084895	5084955	.	+	.	gene_id GRMZM2G327059; transcript_id TCONS_00000117; exon_number 5; oId CUFF.240.2; tss_id TSS109;
1	Cufflinks	exon	5085393	5086610	.	+	.	gene_id GRMZM2G327059; transcript_id TCONS_00000117; exon_number 6; oId CUFF.240.2; tss_id TSS109;
1	Cufflinks	exon	5238161	5241414	.	+	.	gene_id XLOC_000106; transcript_id TCONS_00000121; exon_number 1; oId CUFF.216.1; tss_id TSS110;
1	Cufflinks	exon	5242183	5242193	.	+	.	gene_id XLOC_000106; transcript_id TCONS_00000121; exon_number 2; oId CUFF.216.1; tss_id TSS110;
1	Cufflinks	exon	5435030	5435795	.	+	.	gene_id GRMZM2G176548; transcript_id TCONS_00000128; exon_number 1; oId CUFF.200.1; tss_id TSS117;
1	Cufflinks	exon	5436239	5436495	.	+	.	gene_id GRMZM2G176548; transcript_id TCONS_00000128; exon_number 2; oId CUFF.200.1; tss_id TSS117;
1	Cufflinks	exon	7078142	7078326	.	+	.	gene_id GRMZM2G409976; transcript_id TCONS_00000156; exon_number 1; oId CUFF.593.2; tss_id TSS298;
1	Cufflinks	exon	7078393	7078709	.	+	.	gene_id GRMZM2G409976; transcript_id TCONS_00000156; exon_number 2; oId CUFF.593.2; tss_id TSS298;
1	Cufflinks	exon	7078940	7079085	.	+	.	gene_id GRMZM2G409976; transcript_id TCONS_00000156; exon_number 3; oId CUFF.593.2; tss_id TSS298;
1	Cufflinks	exon	7079208	7079350	.	+	.	gene_id GRMZM2G409976; transcript_id TCONS_00000156; exon_number 4; oId CUFF.593.2; tss_id TSS298;
1	Cufflinks	exon	7079430	7079553	.	+	.	gene_id GRMZM2G409976; transcript_id TCONS_00000156; exon_number 5; oId CUFF.593.2; tss_id TSS298;
1	Cufflinks	exon	7079659	7082865	.	+	.	gene_id GRMZM2G409976; transcript_id TCONS_00000156; exon_number 6; oId CUFF.593.2; tss_id TSS298;
1	Cufflinks	exon	7555836	7556194	.	+	.	gene_id GRMZM2G110345; transcript_id TCONS_00000171; exon_number 1; oId CUFF.617.1; tss_id TSS323;
1	Cufflinks	exon	7556396	7556487	.	+	.	gene_id GRMZM2G110345; transcript_id TCONS_00000171; exon_number 2; oId CUFF.617.1; tss_id TSS323;
1	Cufflinks	exon	7556639	7556832	.	+	.	gene_id GRMZM2G110345; transcript_id TCONS_00000171; exon_number 3; oId CUFF.617.1; tss_id TSS323;
1	Cufflinks	exon	7557813	7558154	.	+	.	gene_id GRMZM2G110345; transcript_id TCONS_00000171; exon_number 4; oId CUFF.617.1; tss_id TSS323;
1	Cufflinks	exon	7558697	7558774	.	+	.	gene_id GRMZM2G110345; transcript_id TCONS_00000171; exon_number 5; oId CUFF.617.1; tss_id TSS323;
1	Cufflinks	exon	7558859	7558984	.	+	.	gene_id GRMZM2G110345; transcript_id TCONS_00000171; exon_number 6; oId CUFF.617.1; tss_id TSS323;
1	Cufflinks	exon	7559384	7559505	.	+	.	gene_id GRMZM2G110345; transcript_id TCONS_00000171; exon_number 7; oId CUFF.617.1; tss_id TSS323;
1	Cufflinks	exon	7559965	7560004	.	+	.	gene_id GRMZM2G110345; transcript_id TCONS_00000171; exon_number 8; oId CUFF.617.1; tss_id TSS323;
1	Cufflinks	exon	7560448	7560522	.	+	.	gene_id GRMZM2G110345; transcript_id TCONS_00000171; exon_number 9; oId CUFF.617.1; tss_id TSS323;
1	Cufflinks	exon	7560624	7560949	.	+	.	gene_id GRMZM2G110345; transcript_id TCONS_00000171; exon_number 10; oId CUFF.617.1; tss_id TSS323;
1	Cufflinks	exon	7561410	7562014	.	+	.	gene_id GRMZM2G410991; transcript_id TCONS_00000172; exon_number 1; oId CUFF.623.1; tss_id TSS325;
1	Cufflinks	exon	7562108	7562168	.	+	.	gene_id GRMZM2G410991; transcript_id TCONS_00000172; exon_number 2; oId CUFF.623.1; tss_id TSS325;
1	Cufflinks	exon	7563011	7563131	.	+	.	gene_id GRMZM2G410991; transcript_id TCONS_00000172; exon_number 3; oId CUFF.623.1; tss_id TSS325;
1	Cufflinks	exon	7563225	7563412	.	+	.	gene_id GRMZM2G410991; transcript_id TCONS_00000172; exon_number 4; oId CUFF.623.1; tss_id TSS325;
1	Cufflinks	exon	7563498	7564192	.	+	.	gene_id GRMZM2G410991; transcript_id TCONS_00000172; exon_number 5; oId CUFF.623.1; tss_id TSS325;
1	Cufflinks	exon	11473930	11474618	.	+	.	gene_id XLOC_000210; transcript_id TCONS_00000247; exon_number 1; oId CUFF.864.1; tss_id TSS479;
1	Cufflinks	exon	11474766	11474847	.	+	.	gene_id XLOC_000210; transcript_id TCONS_00000247; exon_number 2; oId CUFF.864.1; tss_id TSS479;
1	Cufflinks	exon	11474953	11474971	.	+	.	gene_id XLOC_000210; transcript_id TCONS_00000247; exon_number 3; oId CUFF.864.1; tss_id TSS479;
1	Cufflinks	exon	12173174	12174065	.	+	.	gene_id GRMZM2G377787; transcript_id TCONS_00000264; exon_number 1; oId CUFF.440.1; tss_id TSS232;
1	Cufflinks	exon	12252948	12254065	.	+	.	gene_id XLOC_000228; transcript_id TCONS_00000269; exon_number 1; oId CUFF.435.1; tss_id TSS237;

1	Cufflinks	exon	14960091	14960882	.	+	.	gene_id XLOC_000274; transcript_id TCONS_00000320; exon_number 1; oId CUFF.526.1; tss_id TSS284;
1	Cufflinks	exon	17462417	17462811	.	+	.	gene_id XLOC_000309; transcript_id TCONS_00000361; exon_number 1; oId CUFF.597.1; tss_id TSS321;
1	Cufflinks	exon	22383382	22384394	.	+	.	gene_id XLOC_000380; transcript_id TCONS_00000447; exon_number 1; oId CUFF.1576.1; tss_id TSS888;
1	Cufflinks	exon	22437196	22438908	.	+	.	gene_id XLOC_000381; transcript_id TCONS_00000448; exon_number 1; oId CUFF.719.1; tss_id TSS396;
1	Cufflinks	exon	22645597	22645656	.	+	.	gene_id GRMZM2G055217; transcript_id TCONS_00000454; exon_number 1; oId CUFF.1592.1; tss_id TSS899;
1	Cufflinks	exon	22646337	22646429	.	+	.	gene_id GRMZM2G055217; transcript_id TCONS_00000454; exon_number 2; oId CUFF.1592.1; tss_id TSS899;
1	Cufflinks	exon	22646524	22647044	.	+	.	gene_id GRMZM2G055217; transcript_id TCONS_00000454; exon_number 3; oId CUFF.1592.1; tss_id TSS899;
1	Cufflinks	exon	22648825	22649046	.	+	.	gene_id GRMZM2G055217; transcript_id TCONS_00000454; exon_number 4; oId CUFF.1592.1; tss_id TSS899;
1	Cufflinks	exon	22649350	22649525	.	+	.	gene_id GRMZM2G055217; transcript_id TCONS_00000454; exon_number 5; oId CUFF.1592.1; tss_id TSS899;
1	Cufflinks	exon	22649619	22649910	.	+	.	gene_id GRMZM2G055217; transcript_id TCONS_00000454; exon_number 6; oId CUFF.1592.1; tss_id TSS899;
1	Cufflinks	exon	22650054	22650341	.	+	.	gene_id GRMZM2G055217; transcript_id TCONS_00000454; exon_number 7; oId CUFF.1592.1; tss_id TSS899;
1	Cufflinks	exon	27125995	27126318	.	+	.	gene_id XLOC_000429; transcript_id TCONS_00000507; exon_number 1; oId CUFF.805.1; tss_id TSS447;
1	Cufflinks	exon	29513532	29513670	.	+	.	gene_id XLOC_000463; transcript_id TCONS_00000546; exon_number 1; oId CUFF.1963.1; tss_id TSS1099;
1	Cufflinks	exon	29513756	29514081	.	+	.	gene_id XLOC_000463; transcript_id TCONS_00000546; exon_number 2; oId CUFF.1963.1; tss_id TSS1099;
1	Cufflinks	exon	29514167	29514265	.	+	.	gene_id XLOC_000463; transcript_id TCONS_00000546; exon_number 3; oId CUFF.1963.1; tss_id TSS1099;
1	Cufflinks	exon	29514346	29514477	.	+	.	gene_id XLOC_000463; transcript_id TCONS_00000546; exon_number 4; oId CUFF.1963.1; tss_id TSS1099;
1	Cufflinks	exon	29514572	29515125	.	+	.	gene_id XLOC_000463; transcript_id TCONS_00000546; exon_number 5; oId CUFF.1963.1; tss_id TSS1099;
1	Cufflinks	exon	29515268	29516007	.	+	.	gene_id XLOC_000463; transcript_id TCONS_00000546; exon_number 6; oId CUFF.1963.1; tss_id TSS1099;
1	Cufflinks	exon	29543125	29543575	.	+	.	gene_id XLOC_000465; transcript_id TCONS_00000548; exon_number 1; oId CUFF.897.1; tss_id TSS483;
1	Cufflinks	exon	31254138	31254520	.	+	.	gene_id XLOC_000483; transcript_id TCONS_00000568; exon_number 1; oId CUFF.910.1; tss_id TSS501;
1	Cufflinks	exon	32326220	32326240	.	+	.	gene_id GRMZM5G884325; transcript_id TCONS_00000588; exon_number 1; oId CUFF.2116.1; tss_id TSS1175;
1	Cufflinks	exon	32326338	32327144	.	+	.	gene_id GRMZM5G884325; transcript_id TCONS_00000588; exon_number 2; oId CUFF.2116.1; tss_id TSS1175;
1	Cufflinks	exon	32327244	32327296	.	+	.	gene_id GRMZM5G884325; transcript_id TCONS_00000588; exon_number 3; oId CUFF.2116.1; tss_id TSS1175;
1	Cufflinks	exon	32327397	32327463	.	+	.	gene_id GRMZM5G884325; transcript_id TCONS_00000588; exon_number 4; oId CUFF.2116.1; tss_id TSS1175;
1	Cufflinks	exon	32327559	32327615	.	+	.	gene_id GRMZM5G884325; transcript_id TCONS_00000588; exon_number 5; oId CUFF.2116.1; tss_id TSS1175;
1	Cufflinks	exon	32333012	32333213	.	+	.	gene_id GRMZM5G884325; transcript_id TCONS_00000588; exon_number 6; oId CUFF.2116.1; tss_id TSS1175;
1	Cufflinks	exon	32875353	32875768	.	+	.	gene_id XLOC_000510; transcript_id TCONS_00000599; exon_number 1; oId CUFF.958.1; tss_id TSS530;
1	Cufflinks	exon	36225101	36227564	.	+	.	gene_id XLOC_000550; transcript_id TCONS_00000640; exon_number 1; oId CUFF.1058.1; tss_id TSS570;
1	Cufflinks	exon	38621544	38622277	.	+	.	gene_id GRMZM2G128206; transcript_id TCONS_00000671; exon_number 1; oId CUFF.2425.3; tss_id TSS1341;
1	Cufflinks	exon	38622368	38622405	.	+	.	gene_id GRMZM2G128206; transcript_id TCONS_00000671; exon_number 2; oId CUFF.2425.3; tss_id TSS1341;
1	Cufflinks	exon	38622495	38622566	.	+	.	gene_id GRMZM2G128206; transcript_id TCONS_00000671; exon_number 3; oId CUFF.2425.3; tss_id TSS1341;
1	Cufflinks	exon	38622948	38623079	.	+	.	gene_id GRMZM2G128206; transcript_id TCONS_00000671; exon_number 4; oId CUFF.2425.3; tss_id TSS1341;
1	Cufflinks	exon	39117344	39118213	.	+	.	gene_id XLOC_000584; transcript_id TCONS_00000679; exon_number 1; oId CUFF.1145.1; tss_id TSS608;
1	Cufflinks	exon	41279818	41280468	.	+	.	gene_id GRMZM2G048482; transcript_id TCONS_00000717; exon_number 1; oId CUFF.1184.1; tss_id TSS645;
1	Cufflinks	exon	41280550	41282069	.	+	.	gene_id GRMZM2G048482; transcript_id TCONS_00000717; exon_number 2; oId CUFF.1184.1; tss_id TSS645;
1	Cufflinks	exon	41282174	41282398	.	+	.	gene_id GRMZM2G048482; transcript_id TCONS_00000717; exon_number 3; oId CUFF.1184.1; tss_id TSS645;

1	Cufflinks	exon	41282486	41282687	.	+	.	gene_id GRMZM2G048482; transcript_id TCONS_00000717; exon_number 4; oId CUFF.1184.1; tss_id TSS645;
1	Cufflinks	exon	41282884	41283349	.	+	.	gene_id GRMZM2G048482; transcript_id TCONS_00000717; exon_number 5; oId CUFF.1184.1; tss_id TSS645;
1	Cufflinks	exon	41441234	41441685	.	+	.	gene_id XLOC_000621; transcript_id TCONS_00000720; exon_number 1; oId CUFF.1182.1; tss_id TSS647;
1	Cufflinks	exon	43692630	43692886	.	+	.	gene_id XLOC_000648; transcript_id TCONS_00000751; exon_number 1; oId CUFF.1239.1; tss_id TSS674;
1	Cufflinks	exon	44959729	44960104	.	+	.	gene_id XLOC_000662; transcript_id TCONS_00000770; exon_number 1; oId CUFF.2768.1; tss_id TSS1535;
1	Cufflinks	exon	44964047	44965076	.	+	.	gene_id XLOC_000662; transcript_id TCONS_00000770; exon_number 2; oId CUFF.2768.1; tss_id TSS1535;
1	Cufflinks	exon	45119161	45119779	.	+	.	gene_id XLOC_000663; transcript_id TCONS_00000771; exon_number 1; oId CUFF.1285.1; tss_id TSS691;
1	Cufflinks	exon	45119892	45119972	.	+	.	gene_id XLOC_000663; transcript_id TCONS_00000771; exon_number 2; oId CUFF.1285.1; tss_id TSS691;
1	Cufflinks	exon	45120091	45120699	.	+	.	gene_id XLOC_000663; transcript_id TCONS_00000771; exon_number 3; oId CUFF.1285.1; tss_id TSS691;
1	Cufflinks	exon	45519821	45520211	.	+	.	gene_id XLOC_000672; transcript_id TCONS_00000781; exon_number 1; oId CUFF.1282.1; tss_id TSS700;
1	Cufflinks	exon	45637463	45638222	.	+	.	gene_id GRMZM5G850640; transcript_id TCONS_00000782; exon_number 1; oId CUFF.2827.1; tss_id TSS1566;
1	Cufflinks	exon	45639591	45639907	.	+	.	gene_id GRMZM5G850640; transcript_id TCONS_00000782; exon_number 2; oId CUFF.2827.1; tss_id TSS1566;
1	Cufflinks	exon	45640036	45640147	.	+	.	gene_id GRMZM5G850640; transcript_id TCONS_00000782; exon_number 3; oId CUFF.2827.1; tss_id TSS1566;
1	Cufflinks	exon	45640282	45640413	.	+	.	gene_id GRMZM5G850640; transcript_id TCONS_00000782; exon_number 4; oId CUFF.2827.1; tss_id TSS1566;
1	Cufflinks	exon	45640500	45640713	.	+	.	gene_id GRMZM5G850640; transcript_id TCONS_00000782; exon_number 5; oId CUFF.2827.1; tss_id TSS1566;
1	Cufflinks	exon	45954234	45954542	.	+	.	gene_id XLOC_000682; transcript_id TCONS_00000791; exon_number 1; oId CUFF.1313.1; tss_id TSS710;
1	Cufflinks	exon	47330719	47330940	.	+	.	gene_id XLOC_000708; transcript_id TCONS_00000817; exon_number 1; oId CUFF.1347.1; tss_id TSS736;
1	Cufflinks	exon	52390956	52391285	.	+	.	gene_id XLOC_000765; transcript_id TCONS_00000885; exon_number 1; oId CUFF.3212.1; tss_id TSS1806;
1	Cufflinks	exon	52391417	52393884	.	+	.	gene_id XLOC_000765; transcript_id TCONS_00000885; exon_number 2; oId CUFF.3212.1; tss_id TSS1806;
1	Cufflinks	exon	54604498	54604949	.	+	.	gene_id XLOC_000790; transcript_id TCONS_00000912; exon_number 1; oId CUFF.1513.1; tss_id TSS821;
1	Cufflinks	exon	57893672	57894233	.	+	.	gene_id GRMZM2G064814; transcript_id TCONS_00000959; exon_number 1; oId CUFF.3515.1; tss_id TSS1984;
1	Cufflinks	exon	57894365	57896714	.	+	.	gene_id GRMZM2G064814; transcript_id TCONS_00000959; exon_number 2; oId CUFF.3515.1; tss_id TSS1984;
1	Cufflinks	exon	57896778	57899432	.	+	.	gene_id GRMZM2G064814; transcript_id TCONS_00000959; exon_number 3; oId CUFF.3515.1; tss_id TSS1984;
1	Cufflinks	exon	57899512	57900344	.	+	.	gene_id GRMZM2G064814; transcript_id TCONS_00000959; exon_number 4; oId CUFF.3515.1; tss_id TSS1984;
1	Cufflinks	exon	61934146	61934382	.	+	.	gene_id GRMZM2G081322; transcript_id TCONS_00000997; exon_number 1; oId CUFF.3695.1; tss_id TSS2090;
1	Cufflinks	exon	61934551	61934669	.	+	.	gene_id GRMZM2G081322; transcript_id TCONS_00000997; exon_number 2; oId CUFF.3695.1; tss_id TSS2090;
1	Cufflinks	exon	61934821	61934920	.	+	.	gene_id GRMZM2G081322; transcript_id TCONS_00000997; exon_number 3; oId CUFF.3695.1; tss_id TSS2090;
1	Cufflinks	exon	61935034	61935099	.	+	.	gene_id GRMZM2G081322; transcript_id TCONS_00000997; exon_number 4; oId CUFF.3695.1; tss_id TSS2090;
1	Cufflinks	exon	61935193	61935429	.	+	.	gene_id GRMZM2G081322; transcript_id TCONS_00000997; exon_number 5; oId CUFF.3695.1; tss_id TSS2090;
1	Cufflinks	exon	61935514	61935691	.	+	.	gene_id GRMZM2G081322; transcript_id TCONS_00000997; exon_number 6; oId CUFF.3695.1; tss_id TSS2090;
1	Cufflinks	exon	61935780	61935912	.	+	.	gene_id GRMZM2G081322; transcript_id TCONS_00000997; exon_number 7; oId CUFF.3695.1; tss_id TSS2090;
1	Cufflinks	exon	61936868	61937027	.	+	.	gene_id GRMZM2G081322; transcript_id TCONS_00000997; exon_number 8; oId CUFF.3695.1; tss_id TSS2090;
1	Cufflinks	exon	61937103	61937259	.	+	.	gene_id GRMZM2G081322; transcript_id TCONS_00000997; exon_number 9; oId CUFF.3695.1; tss_id TSS2090;
1	Cufflinks	exon	61937334	61937437	.	+	.	gene_id GRMZM2G081322; transcript_id TCONS_00000997; exon_number 10; oId CUFF.3695.1; tss_id TSS2090;
1	Cufflinks	exon	61937528	61937686	.	+	.	gene_id GRMZM2G081322; transcript_id TCONS_00000997; exon_number 11; oId CUFF.3695.1; tss_id TSS2090;
1	Cufflinks	exon	61938365	61938433	.	+	.	gene_id GRMZM2G081322; transcript_id TCONS_00000997; exon_number 12; oId CUFF.3695.1; tss_id TSS2090;

1	Cufflinks	exon	61938518	61938586	.	+	.	gene_id GRMZM2G081322; transcript_id TCONS_00000997; exon_number 13; oId CUFF.3695.1; tss_id TSS2090;
1	Cufflinks	exon	61938744	61938881	.	+	.	gene_id GRMZM2G081322; transcript_id TCONS_00000997; exon_number 14; oId CUFF.3695.1; tss_id TSS2090;
1	Cufflinks	exon	61939354	61939482	.	+	.	gene_id GRMZM2G081322; transcript_id TCONS_00000997; exon_number 15; oId CUFF.3695.1; tss_id TSS2090;
1	Cufflinks	exon	61940132	61940282	.	+	.	gene_id GRMZM2G081322; transcript_id TCONS_00000997; exon_number 16; oId CUFF.3695.1; tss_id TSS2090;
1	Cufflinks	exon	61940418	61940428	.	+	.	gene_id GRMZM2G081322; transcript_id TCONS_00000997; exon_number 17; oId CUFF.3695.1; tss_id TSS2090;
1	Cufflinks	exon	61940518	61940658	.	+	.	gene_id GRMZM2G081322; transcript_id TCONS_00000997; exon_number 18; oId CUFF.3695.1; tss_id TSS2090;
1	Cufflinks	exon	61940748	61940873	.	+	.	gene_id GRMZM2G081322; transcript_id TCONS_00000997; exon_number 19; oId CUFF.3695.1; tss_id TSS2090;
1	Cufflinks	exon	61941454	61941510	.	+	.	gene_id GRMZM2G081322; transcript_id TCONS_00000997; exon_number 20; oId CUFF.3695.1; tss_id TSS2090;
1	Cufflinks	exon	61941590	61941688	.	+	.	gene_id GRMZM2G081322; transcript_id TCONS_00000997; exon_number 21; oId CUFF.3695.1; tss_id TSS2090;
1	Cufflinks	exon	61941790	61942018	.	+	.	gene_id GRMZM2G081322; transcript_id TCONS_00000997; exon_number 22; oId CUFF.3695.1; tss_id TSS2090;
1	Cufflinks	exon	61942665	61942861	.	+	.	gene_id GRMZM2G081322; transcript_id TCONS_00000997; exon_number 23; oId CUFF.3695.1; tss_id TSS2090;
1	Cufflinks	exon	61943932	61944291	.	+	.	gene_id GRMZM2G081322; transcript_id TCONS_00000997; exon_number 24; oId CUFF.3695.1; tss_id TSS2090;
1	Cufflinks	exon	67777631	67778053	.	+	.	gene_id GRMZM2G179792; transcript_id TCONS_00001097; exon_number 1; oId CUFF.3974.3; tss_id TSS2239;
1	Cufflinks	exon	67778134	67778156	.	+	.	gene_id GRMZM2G179792; transcript_id TCONS_00001097; exon_number 2; oId CUFF.3974.3; tss_id TSS2239;
1	Cufflinks	exon	67778178	67778411	.	+	.	gene_id GRMZM2G179792; transcript_id TCONS_00001097; exon_number 3; oId CUFF.3974.3; tss_id TSS2239;
1	Cufflinks	exon	67778887	67778946	.	+	.	gene_id GRMZM2G179792; transcript_id TCONS_00001097; exon_number 4; oId CUFF.3974.3; tss_id TSS2239;
1	Cufflinks	exon	67779047	67780949	.	+	.	gene_id GRMZM2G179792; transcript_id TCONS_00001097; exon_number 5; oId CUFF.3974.3; tss_id TSS2239;
1	Cufflinks	exon	67781024	67781637	.	+	.	gene_id GRMZM2G179792; transcript_id TCONS_00001097; exon_number 6; oId CUFF.3974.3; tss_id TSS2239;
1	Cufflinks	exon	68883902	68884199	.	+	.	gene_id GRMZM2G022061; transcript_id TCONS_00001112; exon_number 1; oId CUFF.1842.1; tss_id TSS995;
1	Cufflinks	exon	68884482	68884520	.	+	.	gene_id GRMZM2G022061; transcript_id TCONS_00001112; exon_number 2; oId CUFF.1842.1; tss_id TSS995;
1	Cufflinks	exon	68884600	68884748	.	+	.	gene_id GRMZM2G022061; transcript_id TCONS_00001112; exon_number 3; oId CUFF.1842.1; tss_id TSS995;
1	Cufflinks	exon	68884940	68885015	.	+	.	gene_id GRMZM2G022061; transcript_id TCONS_00001112; exon_number 4; oId CUFF.1842.1; tss_id TSS995;
1	Cufflinks	exon	68887040	68887334	.	+	.	gene_id GRMZM2G022061; transcript_id TCONS_00001112; exon_number 5; oId CUFF.1842.1; tss_id TSS995;
1	Cufflinks	exon	68887418	68887524	.	+	.	gene_id GRMZM2G022061; transcript_id TCONS_00001112; exon_number 6; oId CUFF.1842.1; tss_id TSS995;
1	Cufflinks	exon	68887602	68887649	.	+	.	gene_id GRMZM2G022061; transcript_id TCONS_00001112; exon_number 7; oId CUFF.1842.1; tss_id TSS995;
1	Cufflinks	exon	68887729	68887935	.	+	.	gene_id GRMZM2G022061; transcript_id TCONS_00001112; exon_number 8; oId CUFF.1842.1; tss_id TSS995;
1	Cufflinks	exon	68888250	68888351	.	+	.	gene_id GRMZM2G022061; transcript_id TCONS_00001112; exon_number 9; oId CUFF.1842.1; tss_id TSS995;
1	Cufflinks	exon	68888528	68888658	.	+	.	gene_id GRMZM2G022061; transcript_id TCONS_00001112; exon_number 10; oId CUFF.1842.1; tss_id TSS995;
1	Cufflinks	exon	68888757	68888886	.	+	.	gene_id GRMZM2G022061; transcript_id TCONS_00001112; exon_number 11; oId CUFF.1842.1; tss_id TSS995;
1	Cufflinks	exon	68889462	68889560	.	+	.	gene_id GRMZM2G022061; transcript_id TCONS_00001112; exon_number 12; oId CUFF.1842.1; tss_id TSS995;
1	Cufflinks	exon	68889651	68889980	.	+	.	gene_id GRMZM2G022061; transcript_id TCONS_00001112; exon_number 13; oId CUFF.1842.1; tss_id TSS995;
1	Cufflinks	exon	70582852	70582987	.	+	.	gene_id GRMZM2G071688; transcript_id TCONS_00001125; exon_number 1; oId CUFF.1826.1; tss_id TSS1007;
1	Cufflinks	exon	70583095	70583151	.	+	.	gene_id GRMZM2G071688; transcript_id TCONS_00001125; exon_number 2; oId CUFF.1826.1; tss_id TSS1007;
1	Cufflinks	exon	70583237	70583345	.	+	.	gene_id GRMZM2G071688; transcript_id TCONS_00001125; exon_number 3; oId CUFF.1826.1; tss_id TSS1007;
1	Cufflinks	exon	70583552	70583727	.	+	.	gene_id GRMZM2G071688; transcript_id TCONS_00001125; exon_number 4; oId CUFF.1826.1; tss_id TSS1007;
1	Cufflinks	exon	70583823	70583911	.	+	.	gene_id GRMZM2G071688; transcript_id TCONS_00001125; exon_number 5; oId CUFF.1826.1; tss_id TSS1007;

1	Cufflinks	exon	70584009	70584258	.	+	.	gene_id GRMZM2G071688; transcript_id TCONS_00001125; exon_number 6; oId CUFF.1826.1; tss_id TSS1007;
1	Cufflinks	exon	70584356	70584604	.	+	.	gene_id GRMZM2G071688; transcript_id TCONS_00001125; exon_number 7; oId CUFF.1826.1; tss_id TSS1007;
1	Cufflinks	exon	70584679	70585097	.	+	.	gene_id GRMZM2G071688; transcript_id TCONS_00001125; exon_number 8; oId CUFF.1826.1; tss_id TSS1007;
1	Cufflinks	exon	71222469	71223408	.	+	.	gene_id XLOC_000973; transcript_id TCONS_00001133; exon_number 1; oId CUFF.4090.1; tss_id TSS2310;
1	Cufflinks	exon	77153673	77153979	.	+	.	gene_id XLOC_001033; transcript_id TCONS_00001203; exon_number 1; oId CUFF.1974.1; tss_id TSS1076;
1	Cufflinks	exon	86001434	86001765	.	+	.	gene_id GRMZM2G154549; transcript_id TCONS_00001286; exon_number 1; oId CUFF.2140.2; tss_id TSS1150;
1	Cufflinks	exon	86001863	86001939	.	+	.	gene_id GRMZM2G154549; transcript_id TCONS_00001286; exon_number 2; oId CUFF.2140.2; tss_id TSS1150;
1	Cufflinks	exon	86002050	86002091	.	+	.	gene_id GRMZM2G154549; transcript_id TCONS_00001286; exon_number 3; oId CUFF.2140.2; tss_id TSS1150;
1	Cufflinks	exon	86002799	86002840	.	+	.	gene_id GRMZM2G154549; transcript_id TCONS_00001286; exon_number 4; oId CUFF.2140.2; tss_id TSS1150;
1	Cufflinks	exon	86003405	86003488	.	+	.	gene_id GRMZM2G154549; transcript_id TCONS_00001286; exon_number 5; oId CUFF.2140.2; tss_id TSS1150;
1	Cufflinks	exon	86003649	86003736	.	+	.	gene_id GRMZM2G154549; transcript_id TCONS_00001286; exon_number 6; oId CUFF.2140.2; tss_id TSS1150;
1	Cufflinks	exon	86004149	86004246	.	+	.	gene_id GRMZM2G154549; transcript_id TCONS_00001286; exon_number 7; oId CUFF.2140.2; tss_id TSS1150;
1	Cufflinks	exon	86004335	86004907	.	+	.	gene_id GRMZM2G154549; transcript_id TCONS_00001286; exon_number 8; oId CUFF.2140.2; tss_id TSS1150;
1	Cufflinks	exon	92794480	92794761	.	+	.	gene_id XLOC_001161; transcript_id TCONS_00001353; exon_number 1; oId CUFF.2227.1; tss_id TSS1212;
1	Cufflinks	exon	94692189	94692906	.	+	.	gene_id XLOC_001172; transcript_id TCONS_00001364; exon_number 1; oId CUFF.2248.1; tss_id TSS1223;
1	Cufflinks	exon	95199285	95199496	.	+	.	gene_id XLOC_001182; transcript_id TCONS_00001374; exon_number 1; oId CUFF.2269.1; tss_id TSS1233;
1	Cufflinks	exon	95200813	95201045	.	+	.	gene_id XLOC_001182; transcript_id TCONS_00001374; exon_number 2; oId CUFF.2269.1; tss_id TSS1233;
1	Cufflinks	exon	103831944	103832255	.	+	.	gene_id GRMZM2G089147; transcript_id TCONS_00001426; exon_number 1; oId CUFF.5143.1; tss_id TSS2862;
1	Cufflinks	exon	103832965	103833155	.	+	.	gene_id GRMZM2G089147; transcript_id TCONS_00001426; exon_number 2; oId CUFF.5143.1; tss_id TSS2862;
1	Cufflinks	exon	103833228	103833422	.	+	.	gene_id GRMZM2G089147; transcript_id TCONS_00001426; exon_number 3; oId CUFF.5143.1; tss_id TSS2862;
1	Cufflinks	exon	103833597	103833658	.	+	.	gene_id GRMZM2G089147; transcript_id TCONS_00001426; exon_number 4; oId CUFF.5143.1; tss_id TSS2862;
1	Cufflinks	exon	103833785	103833851	.	+	.	gene_id GRMZM2G089147; transcript_id TCONS_00001426; exon_number 5; oId CUFF.5143.1; tss_id TSS2862;
1	Cufflinks	exon	103833943	103834061	.	+	.	gene_id GRMZM2G089147; transcript_id TCONS_00001426; exon_number 6; oId CUFF.5143.1; tss_id TSS2862;
1	Cufflinks	exon	103834156	103834332	.	+	.	gene_id GRMZM2G089147; transcript_id TCONS_00001426; exon_number 7; oId CUFF.5143.1; tss_id TSS2862;
1	Cufflinks	exon	103834446	103835137	.	+	.	gene_id GRMZM2G089147; transcript_id TCONS_00001426; exon_number 8; oId CUFF.5143.1; tss_id TSS2862;
1	Cufflinks	exon	103835402	103835466	.	+	.	gene_id GRMZM2G089147; transcript_id TCONS_00001426; exon_number 9; oId CUFF.5143.1; tss_id TSS2862;
1	Cufflinks	exon	111555834	111558264	.	+	.	gene_id XLOC_001267; transcript_id TCONS_00001466; exon_number 1; oId CUFF.2407.1; tss_id TSS1320;
1	Cufflinks	exon	139799347	139801075	.	+	.	gene_id GRMZM2G167584; transcript_id TCONS_00001557; exon_number 1; oId CUFF.2600.1; tss_id TSS1401;
1	Cufflinks	exon	140729075	140729880	.	+	.	gene_id XLOC_001351; transcript_id TCONS_00001562; exon_number 1; oId CUFF.2619.1; tss_id TSS1406;
1	Cufflinks	exon	162480410	162480689	.	+	.	gene_id XLOC_001451; transcript_id TCONS_00001676; exon_number 1; oId CUFF.6434.1; tss_id TSS3505;
1	Cufflinks	exon	166943082	166945351	.	+	.	gene_id GRMZM2G017486; transcript_id TCONS_00001727; exon_number 1; oId CUFF.2883.1; tss_id TSS1555;
1	Cufflinks	exon	170986484	170986768	.	+	.	gene_id XLOC_001519; transcript_id TCONS_00001762; exon_number 1; oId CUFF.2952.1; tss_id TSS1585;
1	Cufflinks	exon	172138864	172139836	.	+	.	gene_id GRMZM2G332821; transcript_id TCONS_00001773; exon_number 1; oId CUFF.2968.1; tss_id TSS1593;
1	Cufflinks	exon	172139929	172141926	.	+	.	gene_id GRMZM2G332821; transcript_id TCONS_00001773; exon_number 2; oId CUFF.2968.1; tss_id TSS1593;
1	Cufflinks	exon	180715451	180716127	.	+	.	gene_id GRMZM2G423472; transcript_id TCONS_00001845; exon_number 1; oId CUFF.3157.1; tss_id TSS1660;
1	Cufflinks	exon	180716208	180716283	.	+	.	gene_id GRMZM2G423472; transcript_id TCONS_00001845; exon_number 2; oId CUFF.3157.1; tss_id TSS1660;

1	Cufflinks	exon	180716397	180716512	.	+	.	gene_id GRMZM2G423472; transcript_id TCONS_00001845; exon_number 3; oId CUFF.3157.1; tss_id TSS1660;
1	Cufflinks	exon	180717811	180718850	.	+	.	gene_id GRMZM2G423472; transcript_id TCONS_00001845; exon_number 4; oId CUFF.3157.1; tss_id TSS1660;
1	Cufflinks	exon	180718962	180719208	.	+	.	gene_id GRMZM2G423472; transcript_id TCONS_00001845; exon_number 5; oId CUFF.3157.1; tss_id TSS1660;
1	Cufflinks	exon	180719360	180719501	.	+	.	gene_id GRMZM2G423472; transcript_id TCONS_00001845; exon_number 6; oId CUFF.3157.1; tss_id TSS1660;
1	Cufflinks	exon	180719642	180719762	.	+	.	gene_id GRMZM2G423472; transcript_id TCONS_00001845; exon_number 7; oId CUFF.3157.1; tss_id TSS1660;
1	Cufflinks	exon	180719860	180719922	.	+	.	gene_id GRMZM2G423472; transcript_id TCONS_00001845; exon_number 8; oId CUFF.3157.1; tss_id TSS1660;
1	Cufflinks	exon	180720076	180720707	.	+	.	gene_id GRMZM2G423472; transcript_id TCONS_00001845; exon_number 9; oId CUFF.3157.1; tss_id TSS1660;
1	Cufflinks	exon	187375529	187375931	.	+	.	gene_id GRMZM2G083755; transcript_id TCONS_00001933; exon_number 1; oId CUFF.7370.1; tss_id TSS3971;
1	Cufflinks	exon	187376033	187376429	.	+	.	gene_id GRMZM2G083755; transcript_id TCONS_00001933; exon_number 2; oId CUFF.7370.1; tss_id TSS3971;
1	Cufflinks	exon	187377130	187377202	.	+	.	gene_id GRMZM2G083755; transcript_id TCONS_00001933; exon_number 3; oId CUFF.7370.1; tss_id TSS3971;
1	Cufflinks	exon	187377299	187377346	.	+	.	gene_id GRMZM2G083755; transcript_id TCONS_00001933; exon_number 4; oId CUFF.7370.1; tss_id TSS3971;
1	Cufflinks	exon	187377455	187377549	.	+	.	gene_id GRMZM2G083755; transcript_id TCONS_00001933; exon_number 5; oId CUFF.7370.1; tss_id TSS3971;
1	Cufflinks	exon	187377978	187378458	.	+	.	gene_id GRMZM2G083755; transcript_id TCONS_00001933; exon_number 6; oId CUFF.7370.1; tss_id TSS3971;
1	Cufflinks	exon	187378525	187379214	.	+	.	gene_id GRMZM2G083755; transcript_id TCONS_00001933; exon_number 7; oId CUFF.7370.1; tss_id TSS3971;
1	Cufflinks	exon	187432586	187435072	.	+	.	gene_id GRMZM2G441903; transcript_id TCONS_00001941; exon_number 1; oId CUFF.3260.1; tss_id TSS1744;
1	Cufflinks	exon	191614180	191614649	.	+	.	gene_id XLOC_001725; transcript_id TCONS_00002005; exon_number 1; oId CUFF.3349.1; tss_id TSS1802;
1	Cufflinks	exon	196084489	196086422	.	+	.	gene_id GRMZM2G048129; transcript_id TCONS_00002060; exon_number 1; oId CUFF.3441.1; tss_id TSS1853;
1	Cufflinks	exon	196086549	196086931	.	+	.	gene_id GRMZM2G048129; transcript_id TCONS_00002060; exon_number 2; oId CUFF.3441.1; tss_id TSS1853;
1	Cufflinks	exon	196458881	196460106	.	+	.	gene_id XLOC_001785; transcript_id TCONS_00002071; exon_number 1; oId CUFF.3463.1; tss_id TSS1863;
1	Cufflinks	exon	201502172	201502999	.	+	.	gene_id GRMZM5G899800; transcript_id TCONS_00002140; exon_number 1; oId CUFF.8020.1; tss_id TSS4358;
1	Cufflinks	exon	201503101	201503337	.	+	.	gene_id GRMZM5G899800; transcript_id TCONS_00002140; exon_number 2; oId CUFF.8020.1; tss_id TSS4358;
1	Cufflinks	exon	201503422	201503655	.	+	.	gene_id GRMZM5G899800; transcript_id TCONS_00002140; exon_number 3; oId CUFF.8020.1; tss_id TSS4358;
1	Cufflinks	exon	201504738	201504976	.	+	.	gene_id GRMZM5G899800; transcript_id TCONS_00002140; exon_number 4; oId CUFF.8020.1; tss_id TSS4358;
1	Cufflinks	exon	201506275	201506541	.	+	.	gene_id GRMZM5G899800; transcript_id TCONS_00002140; exon_number 5; oId CUFF.8020.1; tss_id TSS4358;
1	Cufflinks	exon	201506661	201506931	.	+	.	gene_id GRMZM5G899800; transcript_id TCONS_00002140; exon_number 6; oId CUFF.8020.1; tss_id TSS4358;
1	Cufflinks	exon	201507269	201509140	.	+	.	gene_id GRMZM5G899800; transcript_id TCONS_00002140; exon_number 7; oId CUFF.8020.1; tss_id TSS4358;
1	Cufflinks	exon	204903793	204903981	.	+	.	gene_id GRMZM2G320689; transcript_id TCONS_00002202; exon_number 1; oId CUFF.8214.1; tss_id TSS4448;
1	Cufflinks	exon	204904092	204904221	.	+	.	gene_id GRMZM2G320689; transcript_id TCONS_00002202; exon_number 2; oId CUFF.8214.1; tss_id TSS4448;
1	Cufflinks	exon	204904737	204904895	.	+	.	gene_id GRMZM2G320689; transcript_id TCONS_00002202; exon_number 3; oId CUFF.8214.1; tss_id TSS4448;
1	Cufflinks	exon	204904981	204905330	.	+	.	gene_id GRMZM2G320689; transcript_id TCONS_00002202; exon_number 4; oId CUFF.8214.1; tss_id TSS4448;
1	Cufflinks	exon	204905408	204905546	.	+	.	gene_id GRMZM2G320689; transcript_id TCONS_00002202; exon_number 5; oId CUFF.8214.1; tss_id TSS4448;
1	Cufflinks	exon	204905657	204905754	.	+	.	gene_id GRMZM2G320689; transcript_id TCONS_00002202; exon_number 6; oId CUFF.8214.1; tss_id TSS4448;
1	Cufflinks	exon	204905834	204905990	.	+	.	gene_id GRMZM2G320689; transcript_id TCONS_00002202; exon_number 7; oId CUFF.8214.1; tss_id TSS4448;
1	Cufflinks	exon	204906541	204906610	.	+	.	gene_id GRMZM2G320689; transcript_id TCONS_00002202; exon_number 8; oId CUFF.8214.1; tss_id TSS4448;
1	Cufflinks	exon	204906714	204906790	.	+	.	gene_id GRMZM2G320689; transcript_id TCONS_00002202; exon_number 9; oId CUFF.8214.1; tss_id TSS4448;
1	Cufflinks	exon	204906895	204907029	.	+	.	gene_id GRMZM2G320689; transcript_id TCONS_00002202; exon_number 10; oId CUFF.8214.1; tss_id TSS4448;

1	Cufflinks	exon	204907108	204907219	.	+	.	gene_id GRMZM2G320689; transcript_id TCONS_00002202; exon_number 11; oId CUFF.8214.1; tss_id TSS4448;
1	Cufflinks	exon	204907301	204907387	.	+	.	gene_id GRMZM2G320689; transcript_id TCONS_00002202; exon_number 12; oId CUFF.8214.1; tss_id TSS4448;
1	Cufflinks	exon	204907470	204907645	.	+	.	gene_id GRMZM2G320689; transcript_id TCONS_00002202; exon_number 13; oId CUFF.8214.1; tss_id TSS4448;
1	Cufflinks	exon	204907747	204907908	.	+	.	gene_id GRMZM2G320689; transcript_id TCONS_00002202; exon_number 14; oId CUFF.8214.1; tss_id TSS4448;
1	Cufflinks	exon	204908001	204908108	.	+	.	gene_id GRMZM2G320689; transcript_id TCONS_00002202; exon_number 15; oId CUFF.8214.1; tss_id TSS4448;
1	Cufflinks	exon	204908204	204908362	.	+	.	gene_id GRMZM2G320689; transcript_id TCONS_00002202; exon_number 16; oId CUFF.8214.1; tss_id TSS4448;
1	Cufflinks	exon	204908438	204908521	.	+	.	gene_id GRMZM2G320689; transcript_id TCONS_00002202; exon_number 17; oId CUFF.8214.1; tss_id TSS4448;
1	Cufflinks	exon	204908639	204908844	.	+	.	gene_id GRMZM2G320689; transcript_id TCONS_00002202; exon_number 18; oId CUFF.8214.1; tss_id TSS4448;
1	Cufflinks	exon	204908940	204909041	.	+	.	gene_id GRMZM2G320689; transcript_id TCONS_00002202; exon_number 19; oId CUFF.8214.1; tss_id TSS4448;
1	Cufflinks	exon	204909283	204909392	.	+	.	gene_id GRMZM2G320689; transcript_id TCONS_00002202; exon_number 20; oId CUFF.8214.1; tss_id TSS4448;
1	Cufflinks	exon	204909483	204909634	.	+	.	gene_id GRMZM2G320689; transcript_id TCONS_00002202; exon_number 21; oId CUFF.8214.1; tss_id TSS4448;
1	Cufflinks	exon	204909747	204910161	.	+	.	gene_id GRMZM2G320689; transcript_id TCONS_00002202; exon_number 22; oId CUFF.8214.1; tss_id TSS4448;
1	Cufflinks	exon	205285975	205287839	.	+	.	gene_id AC212244.4_FG004; transcript_id TCONS_00002212; exon_number 1; oId CUFF.3694.1; tss_id TSS1990;
1	Cufflinks	exon	210641726	210642216	.	+	.	gene_id XLOC_001964; transcript_id TCONS_00002282; exon_number 1; oId CUFF.8466.1; tss_id TSS4595;
1	Cufflinks	exon	210642798	210642938	.	+	.	gene_id XLOC_001964; transcript_id TCONS_00002282; exon_number 2; oId CUFF.8466.1; tss_id TSS4595;
1	Cufflinks	exon	210643059	210643665	.	+	.	gene_id XLOC_001964; transcript_id TCONS_00002282; exon_number 3; oId CUFF.8466.1; tss_id TSS4595;
1	Cufflinks	exon	224091063	224091982	.	+	.	gene_id XLOC_002116; transcript_id TCONS_00002453; exon_number 1; oId CUFF.4087.1; tss_id TSS2207;
1	Cufflinks	exon	224482341	224485355	.	+	.	gene_id GRMZM2G172451; transcript_id TCONS_00002461; exon_number 1; oId CUFF.4113.1; tss_id TSS2215;
1	Cufflinks	exon	226823143	226823557	.	+	.	gene_id XLOC_002148; transcript_id TCONS_00002489; exon_number 1; oId CUFF.9331.1; tss_id TSS5066;
1	Cufflinks	exon	230075799	230076490	.	+	.	gene_id XLOC_002182; transcript_id TCONS_00002528; exon_number 1; oId CUFF.9525.1; tss_id TSS5131;
1	Cufflinks	exon	230076639	230077837	.	+	.	gene_id XLOC_002182; transcript_id TCONS_00002528; exon_number 2; oId CUFF.9525.1; tss_id TSS5131;
1	Cufflinks	exon	231574753	231575534	.	+	.	gene_id XLOC_002196; transcript_id TCONS_00002549; exon_number 1; oId CUFF.4348.1; tss_id TSS2289;
1	Cufflinks	exon	234403006	234403427	.	+	.	gene_id XLOC_002220; transcript_id TCONS_00002576; exon_number 1; oId CUFF.4366.1; tss_id TSS2315;
1	Cufflinks	exon	234403916	234404583	.	+	.	gene_id XLOC_002220; transcript_id TCONS_00002576; exon_number 2; oId CUFF.4366.1; tss_id TSS2315;
1	Cufflinks	exon	234657581	234657761	.	+	.	gene_id XLOC_002224; transcript_id TCONS_00002581; exon_number 1; oId CUFF.9828.1; tss_id TSS5268;
1	Cufflinks	exon	234658999	234660961	.	+	.	gene_id XLOC_002224; transcript_id TCONS_00002581; exon_number 2; oId CUFF.9828.1; tss_id TSS5268;
1	Cufflinks	exon	237715070	237715921	.	+	.	gene_id XLOC_002254; transcript_id TCONS_00002616; exon_number 1; oId CUFF.4455.1; tss_id TSS2352;
1	Cufflinks	exon	237716057	237716135	.	+	.	gene_id XLOC_002254; transcript_id TCONS_00002616; exon_number 2; oId CUFF.4455.1; tss_id TSS2352;
1	Cufflinks	exon	237716236	237716604	.	+	.	gene_id XLOC_002254; transcript_id TCONS_00002616; exon_number 3; oId CUFF.4455.1; tss_id TSS2352;
1	Cufflinks	exon	238877518	238878461	.	+	.	gene_id XLOC_002264; transcript_id TCONS_00002630; exon_number 1; oId CUFF.4482.1; tss_id TSS2362;
1	Cufflinks	exon	241430167	241431867	.	+	.	gene_id XLOC_002293; transcript_id TCONS_00002659; exon_number 1; oId CUFF.10207.1; tss_id TSS5475;
1	Cufflinks	exon	250153600	250154150	.	+	.	gene_id XLOC_002389; transcript_id TCONS_00002767; exon_number 1; oId CUFF.10687.1; tss_id TSS5775;
1	Cufflinks	exon	250154233	250154431	.	+	.	gene_id XLOC_002389; transcript_id TCONS_00002767; exon_number 2; oId CUFF.10687.1; tss_id TSS5775;
1	Cufflinks	exon	250154521	250154797	.	+	.	gene_id XLOC_002389; transcript_id TCONS_00002767; exon_number 3; oId CUFF.10687.1; tss_id TSS5775;
1	Cufflinks	exon	250154883	250155027	.	+	.	gene_id XLOC_002389; transcript_id TCONS_00002767; exon_number 4; oId CUFF.10687.1; tss_id TSS5775;
1	Cufflinks	exon	250155119	250155281	.	+	.	gene_id XLOC_002389; transcript_id TCONS_00002767; exon_number 5; oId CUFF.10687.1; tss_id TSS5775;

1	Cufflinks	exon	250156439	250156741	.	+	.	gene_id XLOC_002389; transcript_id TCONS_00002767; exon_number 6; oId CUFF.10687.1; tss_id TSS5775;
1	Cufflinks	exon	251500979	251501632	.	+	.	gene_id GRMZM5G805732; transcript_id TCONS_00002781; exon_number 1; oId CUFF.4713.1; tss_id TSS2503;
1	Cufflinks	exon	251503401	251504771	.	+	.	gene_id GRMZM5G805732; transcript_id TCONS_00002781; exon_number 2; oId CUFF.4713.1; tss_id TSS2503;
1	Cufflinks	exon	251505760	251505855	.	+	.	gene_id GRMZM5G805732; transcript_id TCONS_00002781; exon_number 3; oId CUFF.4713.1; tss_id TSS2503;
1	Cufflinks	exon	253373863	253375695	.	+	.	gene_id XLOC_002420; transcript_id TCONS_00002808; exon_number 1; oId CUFF.4748.1; tss_id TSS2523;
1	Cufflinks	exon	257374641	257374770	.	+	.	gene_id GRMZM2G409093; transcript_id TCONS_00002852; exon_number 1; oId CUFF.11083.1; tss_id TSS5972;
1	Cufflinks	exon	257375821	257376177	.	+	.	gene_id GRMZM2G409093; transcript_id TCONS_00002852; exon_number 2; oId CUFF.11083.1; tss_id TSS5972;
1	Cufflinks	exon	257379085	257379231	.	+	.	gene_id GRMZM2G409093; transcript_id TCONS_00002852; exon_number 3; oId CUFF.11083.1; tss_id TSS5972;
1	Cufflinks	exon	257379459	257379522	.	+	.	gene_id GRMZM2G409093; transcript_id TCONS_00002852; exon_number 4; oId CUFF.11083.1; tss_id TSS5972;
1	Cufflinks	exon	257379617	257379826	.	+	.	gene_id GRMZM2G409093; transcript_id TCONS_00002852; exon_number 5; oId CUFF.11083.1; tss_id TSS5972;
1	Cufflinks	exon	257381719	257381761	.	+	.	gene_id GRMZM2G409093; transcript_id TCONS_00002852; exon_number 6; oId CUFF.11083.1; tss_id TSS5972;
1	Cufflinks	exon	257381850	257381913	.	+	.	gene_id GRMZM2G409093; transcript_id TCONS_00002852; exon_number 7; oId CUFF.11083.1; tss_id TSS5972;
1	Cufflinks	exon	257382001	257382035	.	+	.	gene_id GRMZM2G409093; transcript_id TCONS_00002852; exon_number 8; oId CUFF.11083.1; tss_id TSS5972;
1	Cufflinks	exon	257382154	257382299	.	+	.	gene_id GRMZM2G409093; transcript_id TCONS_00002852; exon_number 9; oId CUFF.11083.1; tss_id TSS5972;
1	Cufflinks	exon	257382528	257382804	.	+	.	gene_id GRMZM2G409093; transcript_id TCONS_00002852; exon_number 10; oId CUFF.11083.1; tss_id TSS5972;
1	Cufflinks	exon	257382931	257383206	.	+	.	gene_id GRMZM2G409093; transcript_id TCONS_00002852; exon_number 11; oId CUFF.11083.1; tss_id TSS5972;
1	Cufflinks	exon	257383697	257383930	.	+	.	gene_id GRMZM2G409093; transcript_id TCONS_00002852; exon_number 12; oId CUFF.11083.1; tss_id TSS5972;
1	Cufflinks	exon	257384300	257384724	.	+	.	gene_id GRMZM2G409093; transcript_id TCONS_00002852; exon_number 13; oId CUFF.11083.1; tss_id TSS5972;
1	Cufflinks	exon	260804712	260806407	.	+	.	gene_id XLOC_002505; transcript_id TCONS_00002904; exon_number 1; oId CUFF.11264.1; tss_id TSS6094;
1	Cufflinks	exon	261191923	261192453	.	+	.	gene_id XLOC_002507; transcript_id TCONS_00002906; exon_number 1; oId CUFF.4952.1; tss_id TSS2612;
1	Cufflinks	exon	261720652	261721074	.	+	.	gene_id GRMZM2G133633; transcript_id TCONS_00002909; exon_number 1; oId CUFF.11336.1; tss_id TSS6110;
1	Cufflinks	exon	261721156	261721678	.	+	.	gene_id GRMZM2G133633; transcript_id TCONS_00002909; exon_number 2; oId CUFF.11336.1; tss_id TSS6110;
1	Cufflinks	exon	261721798	261723630	.	+	.	gene_id GRMZM2G133633; transcript_id TCONS_00002909; exon_number 3; oId CUFF.11336.1; tss_id TSS6110;
[truncated: 696,667 more chars]
